# Supplementary material for: Development of the Demographic Dividend Effort Index, a novel tool to measure existing efforts to create a favourable environment to harness a demographic dividend: results from an experts’ survey from six sub-Saharan African countries
Source: BMJ Open. 2023 Mar 21;13(3):e059937. doi: 10.1136/bmjopen-2021-059937 (PMC10040031; doi:10.1136/bmjopen-2021-059937)
Supplement: Supplementary data [file bmjopen-2021-059937supp001.pdf]

JOHNS HOPKINS
BLOOMBERG SCHOOL
*of* PUBLIC HEALTH

Bill & Melinda Gates Institute for Population and Reproductive Health

# Demographic Dividend Effort Index Survey

## Introduction

**There is a programmatic need for a standard measure that quantifies the extent of national efforts in policies and programs implemented to cultivate, realize and harness the benefits of a demographic dividend (DD). Despite the remarkable progress achieved by various countries through the integration of demographic dividend principles into their policies and development plans, performance assessment tools and platforms to document best practices and lessons learned to harness the DD are still missing.**

**Thus, the purpose of the DD Effort Index (DDEI) is to provide a standard measure that will quantify the nature and strength of in-country DD efforts by experts and civil society members across the public and private spheres that represent different dimensions of the society. By measuring efforts, we are capturing inputs independent of outputs, such as income per capita. This will allow us to assess the relationship between inputs that go into the development of DD policies and DD outputs.**

**This DDEI is guided by one central question: To what extent have the existing national policy and program efforts enabled a favorable policy environment to cultivate, realize and harness the benefits of a demographic dividend? To answer this question, the DDEI will summarize key informant perceptions across six sectors identified in the literature to produce a favorable environment to harness a DD. These DD framework sectors are: 1) Family Planning, 2) Maternal and Child Health, 3) Education, 4) Women's Empowerment, 5) Labor Market, and 6) Good Governance, Strong Institutions and Stable Economy. Each sector is measured through its own questionnaire and will be assessed in five dimensions identified in the literature; namely, policy, services or programs, advocacy, research, and civil society. Each of the 6 questionnaires, completed by 10 expert observers per country, score items on a scale from 1 to 10, unless otherwise noted.**

**Considering the health and socio-economic impact of COVID-19, and its close relationship with policies that enable a DD favorable policy environment, this DD Effort Index is integrating a module to assess the resilience and sustainability of systems in each sector. The scoring follows the key principles of sustainable and resilient health systems that can effectively respond to emerging infectious disease threats and other public health emergencies.**

**The DDEI provides an opportunity to visualize the national efforts while assessing the extent to which these efforts support a country's goals to cultivate, realize and harness the demographic dividend. The DDEI will be a tool for performance measurement and progress monitoring, which will provide a timely assessment of successes and gaps and reveal best practices and areas of improvement.**



Rusatira JC, *et al. BMJ Open* 2023; 13:e059937. doi: 10.1136/bmjopen-2021-059937

JOHNS HOPKINS
BLOOMBERG SCHOOL
of PUBLIC HEALTH

Bill & Melinda Gates Institute for Population and Reproductive Health

# Demographic Dividend Effort Index Survey

## Consent form

Thank you for taking the time to complete this questionnaire, especially in this unprecedented time.

The Gates Institute is developing a Demographic Dividend Effort Index (DDEI) to establish a standard measure that will allow the assessment of the existing status of national policies, services, and programs in family planning, maternal and child health, other social sectors (e.g., employment and education), and governance to enable a favorable policy environment to cultivate a demographic dividend (DD). By measuring efforts, this tool will capture inputs independent of outputs, such as income per capita. This will allow the assessment of the relationship between inputs that go into the development of DD policies and DD outputs that are observed and commonly used to assess progress.

We would like to ask you some questions because of your expertise in the area. The information collected will be kept confidential, and we will not be collecting your names or any other identification details. Completing this questionnaire will take around 45-60 min of your time.

We will appreciate your participation in this survey. Information from this interview will be used by the Gates Institute and the community of DD practice in your country to assess the national efforts and identify areas to continue supporting the successes and those that need to be strengthened. Researchers may also use the data collected for cross-country analyses.

You are invited to participate in this survey. Please know that this survey is completely voluntary. By participating, there is no direct benefit to you but there is a societal benefit in enabling the government and development partners to strengthen their efforts to cultivate a DD-favorable environment.

You may refuse to answer any question. Should you have any concerns or issues about the study, you may contact the study coordinator at **jcrusatira@jhu.edu** or **+14436510824** by a phone call or WhatsApp.

* Please check “Yes” if you would like to proceed with the survey.

- ◯ Yes
- ◯ No

3

JOHNS HOPKINS
BLOOMBERG SCHOOL
*of* PUBLIC HEALTH

Bill & Melinda Gates Institute for Population and Reproductive Health

## Demographic Dividend Effort Index Survey

### Important notice

Please do not select 'Back' while in the middle of answering a page of questions as the page you are working on will not be saved until you finalize the said page and select 'Next'. If you select 'Back' without finalizing, the progress you have made on the unfinished page will be lost.

Please be aware that your survey is completed when you read "Thank you for completing the demographic dividend effort index. Stay well and safe!"

JOHNS HOPKINS
BLOOMBERG SCHOOL
of PUBLIC HEALTH

Bill & Melinda Gates Institute for Population and Reproductive Health

# Demographic Dividend Effort Index Survey

## Respondent country and expertise

* Please select the country where you are responding from.

- ◯ Kenya
- ◯ Senegal
- ◯ Nigeria
- ◯ Rwanda
- ◯ Tanzania
- ◯ Ethiopia

BMJ Publishing Group Limited (BMJ) disclaims all liability and responsibility arising from any reliance placed on this supplemental material which has been supplied by the author(s)


JOHNS HOPKINS
BLOOMBERG SCHOOL
of PUBLIC HEALTH

Bill & Melinda Gates Institute for Population and Reproductive Health

## Demographic Dividend Effort Index Survey

### Level of expertise

* At which level of Government is your expertise (If your expertise is at the national level please select "National" otherwise select a county?

JOHNS HOPKINS
BLOOMBERG SCHOOL
of PUBLIC HEALTH

Bill & Melinda Gates Institute for Population and Reproductive Health

## Demographic Dividend Effort Index Survey

### Expertise

* Please select the organization you have worked in the most during the last five years. If your organization is not listed, please write it down.

- ◯ Public sector
- ◯ Private sector
- ◯ Non-governmental organization
- ◯ University/research institute
- ◯ Other (please specify)

* Which dimension have you worked in the most during the last five years?

- ◯ Policy/policymaking
- ◯ Services or programs
- ◯ Advocacy
- ◯ Research
- ◯ Civil Society

* In which sector have you worked the most during the last five years?

- ◯ Family Planning
- ◯ Maternal and Child Health
- ◯ Education
- ◯ Women's Empowerment
- ◯ Labor Market
- ◯ Governance and Economic Institutions

Bill & Melinda Gates Institute for Population and Reproductive Health

# Demographic Dividend Effort Index Survey

## Family Planning Questionnaire

**Please respond to the following questions based on your experience/expertise within this specific sector.**

**To give a summary picture of the national demographic dividend effort, please rate the following items. Score each item from 1 to 10, where 1 represents the lowest score (very weak or no effort) and 10 represents the highest score (strong effort). Where applicable, if a policy or activity is non-existent then mark your response as 0. Please note that a few questions have a different scoring scale.**

Please answer each item. All responses will be recorded in the format exemplified below:

| Lowest effort | 1 | 2 | 3 | 4 | 5 | 6 | 7 | 8 | 9 | 10 | Highest effort | I do not know |
|---|---|---|---|---|---|---|---|---|---|---|---|---|
| | | | | | | | | | | | | |

JOHNS HOPKINS BLOOMBERG SCHOOL of PUBLIC HEALTH

Bill & Melinda Gates Institute for Population and Reproductive Health

## Demographic Dividend Effort Index Survey

### Dimension 1. Policy/Policy making

* FP 1.1 Prominence of Family Planning (FP): Extent to which there is a permanent office or post within the appropriate ministry that develops and monitors sectoral policies and strategies, that is well placed within the government, and staffed by adequately skilled personnel.

| | 1 | 2 | 3 | 4 | 5 | 6 | 7 | 8 | 9 | 10 | Don't know |
|---|---|---|---|---|---|---|---|---|---|---|---|
| 1-Lowest -> Highest-10 | ○ | ○ | ○ | ○ | ○ | ○ | ○ | ○ | ○ | ○ | ○ |

* FP 1.2 Policy on Fertility Reduction and FP: Extent to which government policy emphasizes FP for family size as it relates to available resources or demographic reasons over health reasons.

| | 1 | 2 | 3 | 4 | 5 | 6 | 7 | 8 | 9 | 10 | Don't know |
|---|---|---|---|---|---|---|---|---|---|---|---|
| 1-Lowest -> Highest-10 | ○ | ○ | ○ | ○ | ○ | ○ | ○ | ○ | ○ | ○ | ○ |

* FP 1.3 National FP Strategic Plan: Extent to which there is a current, comprehensive, strategic national plan for FP that outlines policies, laws, and regulations for the provisioning of FP. (In making this assessment, consider whether the plan defines a vision; includes multiyear strategies, annual timelines, and implementation plans; is evidence-based; has indicators to measure progress; and considers the FP market.)

| | 1 | 2 | 3 | 4 | 5 | 6 | 7 | 8 | 9 | 10 | Don't know |
|---|---|---|---|---|---|---|---|---|---|---|---|
| 1-Lowest -> Highest-10 | ○ | ○ | ○ | ○ | ○ | ○ | ○ | ○ | ○ | ○ | ○ |

* FP 1.4 Policy on Age at Marriage: Extent to which legal age at marriage for females is set at 18 years or higher and is enforced.

| | 1 | 2 | 3 | 4 | 5 | 6 | 7 | 8 | 9 | 10 | Don't know |
|---|---|---|---|---|---|---|---|---|---|---|---|
| 1-Lowest -> Highest-10 | ○ | ○ | ○ | ○ | ○ | ○ | ○ | ○ | ○ | ○ | ○ |

9

* FP 1.5 Evidence-Based Policymaking: Extent to which the appropriate ministry actively and regularly supports and uses key FP data and reports to engage in policy development and planning.

| | 1 | 2 | 3 | 4 | 5 | 6 | 7 | 8 | 9 | 10 | Don't know |
|---|---|---|---|---|---|---|---|---|---|---|---|
| 1-Lowest -> Highest-10 | ◯ | ◯ | ◯ | ◯ | ◯ | ◯ | ◯ | ◯ | ◯ | ◯ | ◯ |

* FP 1.6 Monitoring and Evaluation of National Policies/Plans/Strategies: Extent to which the national FP plan outlines a comprehensive monitoring and evaluation strategy to track and measure accomplishments in the plan.

(In making this assessment, consider whether the monitoring and evaluation strategy includes goals and objectives, a results framework, measurable indicators [e.g., “SMART”: specific, measurable, attainable, relevant and time-bound]; and an actionable monitoring and evaluation implementation plan/timeline.)

| | 1 | 2 | 3 | 4 | 5 | 6 | 7 | 8 | 9 | 10 | Don't know |
|---|---|---|---|---|---|---|---|---|---|---|---|
| 1-Lowest -> Highest-10 | ◯ | ◯ | ◯ | ◯ | ◯ | ◯ | ◯ | ◯ | ◯ | ◯ | ◯ |

* FP 1.7 Inclusion of Nongovernmental and Cross-sectoral Actors in Policy/Strategy Development: Extent to which nongovernmental agencies, groups (e.g., from civil society), health workers, stakeholders (e.g., from faith-based, nonprofit, and private-for-profit organizations) and actors across non-FP-specific sectors are included in the national FP plan and/or other national documents outlining the country’s strategies and systems related to planning, managing, and developing FP.

| | 1 | 2 | 3 | 4 | 5 | 6 | 7 | 8 | 9 | 10 | Don't know |
|---|---|---|---|---|---|---|---|---|---|---|---|
| 1-Lowest -> Highest-10 | ◯ | ◯ | ◯ | ◯ | ◯ | ◯ | ◯ | ◯ | ◯ | ◯ | ◯ |

* FP 1.8 Financing for FP: Extent to which the government has established and applied guidelines for the costing and payments for FP across supply and demand considerations (i.e. domestic versus international funding – and its expectations for continuity/sustainability, cost of contraceptive supplies, cost of service delivery and transportation, etc.).

(In making this assessment, consider whether the guidelines and budget address the following: the lifespan of costs for FP care and services; variable structures of costs per FP method; costs of delivery and service provision by specialization, location and risk; costs and profits of healthcare/insurance schemes, the financial costs of providing FP weighed against the financing costs of not doing so.)

| | 1 | 2 | 3 | 4 | 5 | 6 | 7 | 8 | 9 | 10 | Don't know |
|---|---|---|---|---|---|---|---|---|---|---|---|
| 1-Lowest -> Highest-10 | ◯ | ◯ | ◯ | ◯ | ◯ | ◯ | ◯ | ◯ | ◯ | ◯ | ◯ |

* FP 1.9 Percent of In-country Funding of FP Budget: Extent to which the total family planning/population budget is derived from in-country sources (e.g., 1 for 10 percent, 5 for 50 percent, 10 for 100 percent).

| | 1 | 2 | 3 | 4 | 5 | 6 | 7 | 8 | 9 | 10 | Don't know |
|---|---|---|---|---|---|---|---|---|---|---|---|
| 1-Lowest -> Highest-10 | ○ | ○ | ○ | ○ | ○ | ○ | ○ | ○ | ○ | ○ | ○ |

* FP 1.10 Information/Communications Technology Infrastructure/Capacity: Extent to which there are adequate infrastructure components (e.g., computers, servers, printers, a local area network, Internet connectivity, and reliable electric power) as well as human resources (e.g., for maintenance and support) at the national and subnational levels to support the functioning of FP-related information systems.
(In making this assessment, consider whether the country conducts at least annual assessments of its information systems; has training curricula that cover data entry, use, software administration, and development; and continuously trains staff from key stakeholder institutions [e.g., Ministry of Health, Ministry of Education, regulatory agencies, training institutions].)

| | 1 | 2 | 3 | 4 | 5 | 6 | 7 | 8 | 9 | 10 | Don't know |
|---|---|---|---|---|---|---|---|---|---|---|---|
| 1-Lowest -> Highest-10 | ○ | ○ | ○ | ○ | ○ | ○ | ○ | ○ | ○ | ○ | ○ |

* FP 1.11 Import Laws and Legal Regulations:

| | 1 | 2 | 3 | 4 | 5 | 6 | 7 | 8 | 9 | 10 | Don't know |
|---|---|---|---|---|---|---|---|---|---|---|---|
| FP 1.11.1 Extent to which import laws and legal regulations facilitate the importation of FP commodities and supplies | ○ | ○ | ○ | ○ | ○ | ○ | ○ | ○ | ○ | ○ | ○ |
| FP 1.11.2 Extent to which contraceptives are manufactured locally. | ○ | ○ | ○ | ○ | ○ | ○ | ○ | ○ | ○ | ○ | ○ |

* FP 1.12 Advertising of Contraceptives Allowed: Extent of freedom from restrictions on advertising of contraceptives in the mass media.

| | 1 | 2 | 3 | 4 | 5 | 6 | 7 | 8 | 9 | 10 | Don't know |
|---|---|---|---|---|---|---|---|---|---|---|---|
| 1-Lowest -> Highest-10 | ○ | ○ | ○ | ○ | ○ | ○ | ○ | ○ | ○ | ○ | ○ |

* FP 1.13 Presence of Pro-Poor FP Policies: Extent to which policies specifically target the poor in rural or urban places.

| | 1 | 2 | 3 | 4 | 5 | 6 | 7 | 8 | 9 | 10 | Don't know |
|---|---|---|---|---|---|---|---|---|---|---|---|
| 1-Lowest -> Highest-10 | ○ | ○ | ○ | ○ | ○ | ○ | ○ | ○ | ○ | ○ | ○ |

* FP 1.14 Political Support for FP: Extent to which elected officials in the country prioritize meeting FP needs to strengthen the FP contributions to the DD by passing laws and regulations and sponsoring actions and policies aimed at improving FP uptake and adherence.

| | 1 | 2 | 3 | 4 | 5 | 6 | 7 | 8 | 9 | 10 | Don't know |
|---|---|---|---|---|---|---|---|---|---|---|---|
| 1-Lowest -> Highest-10 | ○ | ○ | ○ | ○ | ○ | ○ | ○ | ○ | ○ | ○ | ○ |

FP 1.15 Comments: Please use this section to provide any additional comments about the level of effort, challenges, or successes in the country around policies/policymaking for FP. If you marked “I don’t know” for any of the questions, also use this section to explain why you gave that rating

Johns Hopkins Bloomberg School of Public Health

Bill & Melinda Gates Institute for Population and Reproductive Health

## Demographic Dividend Effort Index Survey

### Dimension 2. Services or Programs

* FP 2.1 Administrative Structure: Extent to which the administrative structure and staff at national, provincial and county levels are sufficient to implement the family planning program.

| | 1 | 2 | 3 | 4 | 5 | 6 | 7 | 8 | 9 | 10 | Don't know |
|---|---|---|---|---|---|---|---|---|---|---|---|
| 1-Lowest -> Highest-10 | ○ | ○ | ○ | ○ | ○ | ○ | ○ | ○ | ○ | ○ | ○ |

* FP 2.2 Level of Program Leaders: Relative level of seniority of the director of the national family planning program and whether said director reports to a high level of government.

| | 1 | 2 | 3 | 4 | 5 | 6 | 7 | 8 | 9 | 10 | Don't know |
|---|---|---|---|---|---|---|---|---|---|---|---|
| 1-Lowest -> Highest-10 | ○ | ○ | ○ | ○ | ○ | ○ | ○ | ○ | ○ | ○ | ○ |

* FP. 2.3 Community-Based Distribution: Extent to which areas of the country not easily serviced by clinics or other service points are covered by community-based distribution programs for distribution of contraceptive supplies (especially rural areas).

| | 1 | 2 | 3 | 4 | 5 | 6 | 7 | 8 | 9 | 10 | Don't know |
|---|---|---|---|---|---|---|---|---|---|---|---|
| 1-Lowest -> Highest-10 | ○ | ○ | ○ | ○ | ○ | ○ | ○ | ○ | ○ | ○ | ○ |

* FP 2.4 Postpartum Program: Extent to which all postpartum women receive postpartum family planning services.

| | 1 | 2 | 3 | 4 | 5 | 6 | 7 | 8 | 9 | 10 | Don't know |
|---|---|---|---|---|---|---|---|---|---|---|---|
| 1-Lowest -> Highest-10 | ○ | ○ | ○ | ○ | ○ | ○ | ○ | ○ | ○ | ○ | ○ |

* FP 2.5 Home-Visiting Workers: Extent of coverage of population by workers whose primary task is to visit (rural) women in their homes to talk about family planning and maternal and child health.

| | 1 | 2 | 3 | 4 | 5 | 6 | 7 | 8 | 9 | 10 | Don't know |
|---|---|---|---|---|---|---|---|---|---|---|---|
| 1-Lowest -> Highest-10 | ○ | ○ | ○ | ○ | ○ | ○ | ○ | ○ | ○ | ○ | ○ |

* FP 2.6 Availability and Accessibility of FP Commodities/Supplies and Services Part 1: Extent to which entire population has ready and easy access to (1-Lowest -> Highest-10):

| | 1 | 2 | 3 | 4 | 5 | 6 | 7 | 8 | 9 | 10 | Don't know |
|---|---|---|---|---|---|---|---|---|---|---|---|
| FP 2.6.1 IUD | ○ | ○ | ○ | ○ | ○ | ○ | ○ | ○ | ○ | ○ | ○ |
| FP 2.6.2 Pills | ○ | ○ | ○ | ○ | ○ | ○ | ○ | ○ | ○ | ○ | ○ |
| FP 2.6.3 Injectables | ○ | ○ | ○ | ○ | ○ | ○ | ○ | ○ | ○ | ○ | ○ |
| FP 2.6.4 Implants | ○ | ○ | ○ | ○ | ○ | ○ | ○ | ○ | ○ | ○ | ○ |
| FP 2.6.5 Female Sterilization | ○ | ○ | ○ | ○ | ○ | ○ | ○ | ○ | ○ | ○ | ○ |
| FP 2.6.6 Male Sterilization | ○ | ○ | ○ | ○ | ○ | ○ | ○ | ○ | ○ | ○ | ○ |
| FP 2.6.7 Male Condoms | ○ | ○ | ○ | ○ | ○ | ○ | ○ | ○ | ○ | ○ | ○ |
| FP 2.6.8 Emergency Contraception | ○ | ○ | ○ | ○ | ○ | ○ | ○ | ○ | ○ | ○ | ○ |
| FP 2.6.9 Abortion/menstrual regulation (whether legal or not) | ○ | ○ | ○ | ○ | ○ | ○ | ○ | ○ | ○ | ○ | ○ |
| FP 2.6.10 Others (e.g. female condoms, foam, beads, etc.) | ○ | ○ | ○ | ○ | ○ | ○ | ○ | ○ | ○ | ○ | ○ |

* FP 2.7 Availability and Accessibility of FP Commodities/Supplies and Services Part 2: How well does the supply system operate (it avoids stockouts or interrupted supplies and guarantees a reliable flow at local levels) for the following (1-Lowest -> Highest-10):

| | 1 | 2 | 3 | 4 | 5 | 6 | 7 | 8 | 9 | 10 | Don't know |
|---|---|---|---|---|---|---|---|---|---|---|---|
| FP 2.7.1 IUD | | | | | | | | | | | |
| FP 2.7.2 Pills | | | | | | | | | | | |
| FP 2.7.3 Injectables | | | | | | | | | | | |
| FP 2.7.4 Implants | | | | | | | | | | | |
| FP 2.7.5 Male Condoms | | | | | | | | | | | |
| FP 2.7.6 Emergency Contraception | | | | | | | | | | | |
| FP 2.7.7 Others (e.g. female condoms, foam, beads, etc.) | | | | | | | | | | | |

* FP 2.8 Availability and Accessibility of FP Commodities/Supplies and Services Part 3: How well does the supply system provide necessary equipment and medical supplies at clinical facilities for the following (1-Lowest -> Highest-10):

| | 1 | 2 | 3 | 4 | 5 | 6 | 7 | 8 | 9 | 10 | Don't know |
|---|---|---|---|---|---|---|---|---|---|---|---|
| FP 2.8.1 Female Sterilization | | | | | | | | | | | |
| FP 2.8.2 Male Sterilization | | | | | | | | | | | |
| FP 2.8.3. IUDs | | | | | | | | | | | |
| FP 2.8.4 Implants | | | | | | | | | | | |

* FP 2.9 FP Service Quality: Please rate the general quality of family planning services. (Good quality includes a focus on client needs, with counseling, full information, wide method choice, and safe clinical procedures.)

| | 1 | 2 | 3 | 4 | 5 | 6 | 7 | 8 | 9 | 10 | Don't know |
|---|---|---|---|---|---|---|---|---|---|---|---|
| 1-Lowest -> Highest-10 | | | | | | | | | | | |

* FP 2.10 Incentives and Disincentives: Extent to which monetary or other incentives are used to encourage the adoption of family planning.

| | 1 | 2 | 3 | 4 | 5 | 6 | 7 | 8 | 9 | 10 | Don't know |
|---|---|---|---|---|---|---|---|---|---|---|---|
| 1-Lowest -> Highest-10 | | | | | | | | | | | |

15

* FP 2.11 Adolescent Programs: Extent to which information, services and activities are focused towards adolescents and encourage adolescents to access contraceptive services.

| | 1 | 2 | 3 | 4 | 5 | 6 | 7 | 8 | 9 | 10 | Don't know |
|---|---|---|---|---|---|---|---|---|---|---|---|
| 1-Lowest -> Highest-10 | ◯ | ◯ | ◯ | ◯ | ◯ | ◯ | ◯ | ◯ | ◯ | ◯ | ◯ |

* FP 2.12 Training Program: Extent to which training programs, for each category of staff in the family planning program, are adequate to provide personnel with information and skills necessary to carry out their jobs effectively.

| | 1 | 2 | 3 | 4 | 5 | 6 | 7 | 8 | 9 | 10 | Don't know |
|---|---|---|---|---|---|---|---|---|---|---|---|
| 1-Lowest -> Highest-10 | ◯ | ◯ | ◯ | ◯ | ◯ | ◯ | ◯ | ◯ | ◯ | ◯ | ◯ |

* FP 2.13 Personnel Carry Out Assigned Tasks: Extent to which all categories of family planning program staff (administrative, medical, paramedical, field) carry out assigned tasks effectively

| | 1 | 2 | 3 | 4 | 5 | 6 | 7 | 8 | 9 | 10 | Don't know |
|---|---|---|---|---|---|---|---|---|---|---|---|
| 1-Lowest -> Highest-10 | ◯ | ◯ | ◯ | ◯ | ◯ | ◯ | ◯ | ◯ | ◯ | ◯ | ◯ |

* FP 2.14 Logistics and Transport: Extent to which the logistics and transport systems are sufficient to keep stocks of contraceptive supplies and related equipment available at all service points, at all times and at all levels.

| | 1 | 2 | 3 | 4 | 5 | 6 | 7 | 8 | 9 | 10 | Don't know |
|---|---|---|---|---|---|---|---|---|---|---|---|
| 1-Lowest -> Highest-10 | ◯ | ◯ | ◯ | ◯ | ◯ | ◯ | ◯ | ◯ | ◯ | ◯ | ◯ |

* FP 2.15 Supervision System: Extent to which the system of supervision at all levels is adequate (regular monitoring visits with corrective or supportive action).

| | 1 | 2 | 3 | 4 | 5 | 6 | 7 | 8 | 9 | 10 | Don't know |
|---|---|---|---|---|---|---|---|---|---|---|---|
| 1-Lowest -> Highest-10 | ◯ | ◯ | ◯ | ◯ | ◯ | ◯ | ◯ | ◯ | ◯ | ◯ | ◯ |

* FP 2.16 Involvement of Other Ministries and Public Agencies: Extent to which other ministries, government agencies and non-governmental stakeholders engage with FP activities (e.g., delivery of supplies, services, information, education) or other population activities.

| | 1 | 2 | 3 | 4 | 5 | 6 | 7 | 8 | 9 | 10 | Don't know |
|---|---|---|---|---|---|---|---|---|---|---|---|
| 1-Lowest -> Highest-10 | ◯ | ◯ | ◯ | ◯ | ◯ | ◯ | ◯ | ◯ | ◯ | ◯ | ◯ |

* FP 2.17 Involvement of Private-Sector Agencies and Groups: Extent to which private-sector agencies and groups assist with FP or other population activities.

| | 1 | 2 | 3 | 4 | 5 | 6 | 7 | 8 | 9 | 10 | Don't know |
|---|---|---|---|---|---|---|---|---|---|---|---|
| 1-Lowest -> Highest-10 | ◯ | ◯ | ◯ | ◯ | ◯ | ◯ | ◯ | ◯ | ◯ | ◯ | ◯ |

* FP 2.18 Mass Media for Information Education Communication: Frequency and extent of coverage of mass media messages that provide the population with information on family planning and service sites.

| | 1 | 2 | 3 | 4 | 5 | 6 | 7 | 8 | 9 | 10 | Don't know |
|---|---|---|---|---|---|---|---|---|---|---|---|
| 1-Lowest -> Highest-10 | ◯ | ◯ | ◯ | ◯ | ◯ | ◯ | ◯ | ◯ | ◯ | ◯ | ◯ |

* FP 2.19 Cultural and Social Norms Barriers: Extent to which cultural and social barriers are addressed in family planning programs.

| | 1 | 2 | 3 | 4 | 5 | 6 | 7 | 8 | 9 | 10 | Don't know |
|---|---|---|---|---|---|---|---|---|---|---|---|
| 1-Lowest -> Highest-10 | ◯ | ◯ | ◯ | ◯ | ◯ | ◯ | ◯ | ◯ | ◯ | ◯ | ◯ |

* FP 2.20 Youth: Extent to which the government supports youth-led FP initiatives or involve youth in planning, implementation and evaluation of FP programs.

| | 1 | 2 | 3 | 4 | 5 | 6 | 7 | 8 | 9 | 10 | Don't know |
|---|---|---|---|---|---|---|---|---|---|---|---|
| 1-Lowest -> Highest-10 | ◯ | ◯ | ◯ | ◯ | ◯ | ◯ | ◯ | ◯ | ◯ | ◯ | ◯ |

* FP 2.21 FP Effort Influences – Forces Affecting the FP Program: Forces affecting the FP program can either strengthen or detract from its effectiveness. Check zero (0) if there is no difference either way; check a negative number from -1 to -5 if the influence is negative; or check a positive number from 1 to 5 if the influence is positive. (1 in either direction means little influence and 5 in either direction means strong influence.)

| | -5 | -4 | -3 | -2 | -1 | 0 | 1 | 2 | 3 | 4 | 5 | Don't know |
|---|---|---|---|---|---|---|---|---|---|---|---|---|
| FP 2.21.1 Decentralization | ○ | ○ | ○ | ○ | ○ | ○ | ○ | ○ | ○ | ○ | ○ | ○ |
| FP 2.21.2 HIV/AIDS Programs | ○ | ○ | ○ | ○ | ○ | ○ | ○ | ○ | ○ | ○ | ○ | ○ |
| FP 2.21.3 Incorporation of FP into a broader context of reproductive health | ○ | ○ | ○ | ○ | ○ | ○ | ○ | ○ | ○ | ○ | ○ | ○ |
| FP 2.21.4 Integration of FP with other health services | ○ | ○ | ○ | ○ | ○ | ○ | ○ | ○ | ○ | ○ | ○ | ○ |
| FP 2.21.5 Changes in donor funding | ○ | ○ | ○ | ○ | ○ | ○ | ○ | ○ | ○ | ○ | ○ | ○ |
| FP 2.21.6 Changes in domestic government funding | ○ | ○ | ○ | ○ | ○ | ○ | ○ | ○ | ○ | ○ | ○ | ○ |

FP 2.22 Comments: Please use this section to provide any additional comments about the level of effort, challenges, or successes in the country around services or programs for FP. If you marked “I don’t know” for any of the questions, also use this section to explain why you gave that rating

JOHNS HOPKINS
BLOOMBERG SCHOOL of PUBLIC HEALTH

Bill & Melinda Gates Institute for Population and Reproductive Health

## Demographic Dividend Effort Index Survey

### Dimension 3. Advocacy

* FP 3.1 Influence of FP Leaders/Champions: Extent to which the country has one or more clearly influential leaders or champions who successfully advocate for FP (sectoral) needs at high levels, promote FP in the country by making positive public statements about it, and/or support actions and policies aimed at improving the FP environment and its contributions towards the DD.

| | 1 | 2 | 3 | 4 | 5 | 6 | 7 | 8 | 9 | 10 | Don't know |
|---|---|---|---|---|---|---|---|---|---|---|---|
| 1-Lowest -> Highest-10 | ○ | ○ | ○ | ○ | ○ | ○ | ○ | ○ | ○ | ○ | ○ |

* FP 3.2 Statement by Leaders: Extent to which the head of government, as well as other officials, speak publicly and favorably about family planning at least once or twice a year.

| | 1 | 2 | 3 | 4 | 5 | 6 | 7 | 8 | 9 | 10 | Don't know |
|---|---|---|---|---|---|---|---|---|---|---|---|
| 1-Lowest -> Highest-10 | ○ | ○ | ○ | ○ | ○ | ○ | ○ | ○ | ○ | ○ | ○ |

* FP 3.3 Media Coverage for FP: Extent to and frequency with which national media cover/broadcast FP topics that provides the population with information on family planning and service sites.

| | 1 | 2 | 3 | 4 | 5 | 6 | 7 | 8 | 9 | 10 | Don't know |
|---|---|---|---|---|---|---|---|---|---|---|---|
| 1-Lowest -> Highest-10 | ○ | ○ | ○ | ○ | ○ | ○ | ○ | ○ | ○ | ○ | ○ |

* FP 3.4 FP Effort Influences - Justification: How important is each of the following as a current government justification for the national FP program? (1 means negligible importance; 10 means great importance.)

| | 1 | 2 | 3 | 4 | 5 | 6 | 7 | 8 | 9 | 10 | Don't know |
|---|---|---|---|---|---|---|---|---|---|---|---|
| FP 3.4.1 Reduce the rate of population growth | | | | | | | | | | | |
| FP 3.4.2 Enhance economic development | | | | | | | | | | | |
| FP 3.4.3 Help women and men avoid unplanned and unwanted births | | | | | | | | | | | |
| FP 3.4.4 Improve women’s health | | | | | | | | | | | |
| FP 3.4.5 Improve child health | | | | | | | | | | | |
| FP 3.4.6 Reduce unmarried adolescent childbearing | | | | | | | | | | | |
| FP 3.4.7 Reduce unmet need for contraceptive services | | | | | | | | | | | |

* FP 3.5 FP Effort Influences – Special Populations: To what extent does the family planning program give particular emphasis to special populations? (1 means negligible emphasis; 10 means great emphasis)

| | 1 | 2 | 3 | 4 | 5 | 6 | 7 | 8 | 9 | 10 | Don't know |
|---|---|---|---|---|---|---|---|---|---|---|---|
| FP 3.5.1 Unmarried youth | | | | | | | | | | | |
| FP 3.5.2 The poor | | | | | | | | | | | |
| FP 3.5.3 The disabled | | | | | | | | | | | |
| FP 3.5.4 Rural population | | | | | | | | | | | |
| FP 3.5.5 Other vulnerable groups (e.g. minority and/or indigenous groups) | | | | | | | | | | | |
| FP 3.5.6 Postpartum women for counseling and contraceptive services | | | | | | | | | | | |
| FP 3.5.7 Post-abortion women for counseling and contraceptive services | | | | | | | | | | | |

* FP 3.6 Social Marketing: Extent of coverage of the country by a social marketing program (subsidized contraceptive sales at low cost in commercial sector, especially in urban areas).

| | 1 | 2 | 3 | 4 | 5 | 6 | 7 | 8 | 9 | 10 | Don't know |
|---|---|---|---|---|---|---|---|---|---|---|---|
| 1-Lowest -> Highest-10 | ○ | ○ | ○ | ○ | ○ | ○ | ○ | ○ | ○ | ○ | ○ |

FP 3.7 Comments: Please use this section to provide any additional comments about the level of effort, challenges, or successes in the country around advocacy for FP. If you marked “I don’t know” for any of the questions, also use this section to explain why you gave that rating.

JOHNS HOPKINS
BLOOMBERG SCHOOL
of PUBLIC HEALTH

Bill & Melinda Gates Institute for Population and Reproductive Health

## Demographic Dividend Effort Index Survey

### Dimension 4. Research

* FP 4.1 Strength of Observatory/Stakeholder/Technical Working Group for FP: Extent to which country-level FP stakeholders (e.g., stakeholder leadership group, technical working group, FP observatory, country coordination and facilitation group):

| | 1 | 2 | 3 | 4 | 5 | 6 | 7 | 8 | 9 | 10 | Don't know |
|---|---|---|---|---|---|---|---|---|---|---|---|
| FP 4.1.1 Have representation in government, training institutions, civil society, nongovernmental and faith-based organizations, professional associations, the private sector, etc. | ○ | ○ | ○ | ○ | ○ | ○ | ○ | ○ | ○ | ○ | ○ |
| FP 4.1.2 Meet regularly, report and recommend policy for senior management within the appropriate ministries | ○ | ○ | ○ | ○ | ○ | ○ | ○ | ○ | ○ | ○ | ○ |
| FP 4.1.3 Make an impact on FP within the country | ○ | ○ | ○ | ○ | ○ | ○ | ○ | ○ | ○ | ○ | ○ |

* FP 4.2 Education/Research Strategy: Extent to which the national FP plan and/or other national documents include a comprehensive strategy/approach on FP-related research.

| | 1 | 2 | 3 | 4 | 5 | 6 | 7 | 8 | 9 | 10 | Don't know |
|---|---|---|---|---|---|---|---|---|---|---|---|
| 1-Lowest -> Highest-10 | ○ | ○ | ○ | ○ | ○ | ○ | ○ | ○ | ○ | ○ | ○ |

* FP 4.3 Data Gathering Partners:

| | 1 | 2 | 3 | 4 | 5 | 6 | 7 | 8 | 9 | 10 | Don't know |
|---|---|---|---|---|---|---|---|---|---|---|---|
| FP 4.3.1 Extent to which research is undertaken by government agencies. (This may include statistical agencies/bureaus and/or ministries of economics/finances, education, health, gender, etc.) | ○ | ○ | ○ | ○ | ○ | ○ | ○ | ○ | ○ | ○ | ○ |
| FP 4.3.2 Extent to which research is undertaken by research institutions. | ○ | ○ | ○ | ○ | ○ | ○ | ○ | ○ | ○ | ○ | ○ |
| FP 4.3.3 Extent to which research is undertaken by independent researchers. | ○ | ○ | ○ | ○ | ○ | ○ | ○ | ○ | ○ | ○ | ○ |

* FP 4.4 Topical Data Gathering:

| | 1 | 2 | 3 | 4 | 5 | 6 | 7 | 8 | 9 | 10 | Don't know |
|---|---|---|---|---|---|---|---|---|---|---|---|
| FP 4.4.1 Extent to which family planning uptake (including method mix), demand, and unmet need is researched. | ○ | ○ | ○ | ○ | ○ | ○ | ○ | ○ | ○ | ○ | ○ |
| FP 4.4.2 Extent to which barriers to FP uptake is researched. | ○ | ○ | ○ | ○ | ○ | ○ | ○ | ○ | ○ | ○ | ○ |
| FP 4.4.3 Extent to which the impacts of FP programs are researched. | ○ | ○ | ○ | ○ | ○ | ○ | ○ | ○ | ○ | ○ | ○ |
| FP 4.4.4 Extent to which the quality of service provision for FP is researched. | ○ | ○ | ○ | ○ | ○ | ○ | ○ | ○ | ○ | ○ | ○ |

* FP 4.5 Quality/Coverage of Data: Extent to which current research/data is sex and age disaggregated. Extent to which the country participates in standardized tests and its ranking.

| | 1 | 2 | 3 | 4 | 5 | 6 | 7 | 8 | 9 | 10 | Don't know |
|---|---|---|---|---|---|---|---|---|---|---|---|
| FP 4.5.1 Extent to which a routine statistical system provides reliable periodic information on FP supplies and facilities. | ◯ | ◯ | ◯ | ◯ | ◯ | ◯ | ◯ | ◯ | ◯ | ◯ | ◯ |
| FP 4.5.2 Extent to which a routine statistical system provides good periodic information on FP services and personnel. | ◯ | ◯ | ◯ | ◯ | ◯ | ◯ | ◯ | ◯ | ◯ | ◯ | ◯ |
| FP 4.5.3 Extent to which the routine statistical system provides good periodic information on FP needs among populations in different communities. | ◯ | ◯ | ◯ | ◯ | ◯ | ◯ | ◯ | ◯ | ◯ | ◯ | ◯ |
| FP 4.5.4 Extent to which evaluations of the health monitoring and information system are conducted and applied to ensure the reliability of data. | ◯ | ◯ | ◯ | ◯ | ◯ | ◯ | ◯ | ◯ | ◯ | ◯ | ◯ |

* FP 4.6 Record-Keeping: Extent to which systems for client recordkeeping, clinic reporting and feedback of results are adequate, including being connected at all levels (federal, state/province, county, etc.).

| | 1 | 2 | 3 | 4 | 5 | 6 | 7 | 8 | 9 | 10 | Don't know |
|---|---|---|---|---|---|---|---|---|---|---|---|
| 1-Lowest -> Highest-10 | ◯ | ◯ | ◯ | ◯ | ◯ | ◯ | ◯ | ◯ | ◯ | ◯ | ◯ |

* FP 4.7 Quality Research Institutions: Extent to which the country supports and sustains research institutions that develop research/collect data that support the breadth and quality of information-gathering to inform national FP efforts.

| | 1 | 2 | 3 | 4 | 5 | 6 | 7 | 8 | 9 | 10 | Don't know |
|---|---|---|---|---|---|---|---|---|---|---|---|
| 1-Lowest -> Highest-10 | ◯ | ◯ | ◯ | ◯ | ◯ | ◯ | ◯ | ◯ | ◯ | ◯ | ◯ |

* FP 4.8 Evaluation: Extent to which program statistics, surveys, and small studies are used by specialized staff to report on program operations and measure progress.

| | 1 | 2 | 3 | 4 | 5 | 6 | 7 | 8 | 9 | 10 | Don't know |
|---|---|---|---|---|---|---|---|---|---|---|---|
| 1-Lowest -> Highest-10 | ○ | ○ | ○ | ○ | ○ | ○ | ○ | ○ | ○ | ○ | ○ |

* FP 4.9 Management's Use of Evaluation Findings: Extent to which local-level program managers use research and evaluation findings to improve programming in ways suggested by findings.

| | 1 | 2 | 3 | 4 | 5 | 6 | 7 | 8 | 9 | 10 | Don't know |
|---|---|---|---|---|---|---|---|---|---|---|---|
| 1-Lowest -> Highest-10 | ○ | ○ | ○ | ○ | ○ | ○ | ○ | ○ | ○ | ○ | ○ |

* FP 4.10 Ministerial Use of Evaluation Findings: Extent to which the Ministry administrators/policymakers systematically utilize data to inform policies and interventions to address FP-related issues.

| | 1 | 2 | 3 | 4 | 5 | 6 | 7 | 8 | 9 | 10 | Don't know |
|---|---|---|---|---|---|---|---|---|---|---|---|
| 1-Lowest -> Highest-10 | ○ | ○ | ○ | ○ | ○ | ○ | ○ | ○ | ○ | ○ | ○ |

* FP 4.11 Dissemination of Information to Other Implementing Bodies: Extent to which information is shared or spread across geographical areas and throughout various levels (national, state/province/county, localities/sub-counties).

| | 1 | 2 | 3 | 4 | 5 | 6 | 7 | 8 | 9 | 10 | Don't know |
|---|---|---|---|---|---|---|---|---|---|---|---|
| 1-Lowest -> Highest-10 | ○ | ○ | ○ | ○ | ○ | ○ | ○ | ○ | ○ | ○ | ○ |

FP 4.12 Comments: Please use this section to provide any additional comments about the level of effort, challenges, or successes in the country around FP research. If you marked "I don't know" for any of the questions, also use this section to explain why you gave that rating.

25

JOHNS HOPKINS
BLOOMBERG SCHOOL
*of* PUBLIC HEALTH

Bill & Melinda Gates Institute for Population and Reproductive Health

## Demographic Dividend Effort Index Survey

### Dimension 5. Civil Society Organizations (CSOs)

* FP 5.1 CSO Actor Power: Extent to which CSO actors are in strong placements within institutions or technical working groups or networks concerned with FP.

| | 1 | 2 | 3 | 4 | 5 | 6 | 7 | 8 | 9 | 10 | Don't know |
|---|---|---|---|---|---|---|---|---|---|---|---|
| 1-Lowest -> Highest-10 | ○ | ○ | ○ | ○ | ○ | ○ | ○ | ○ | ○ | ○ | ○ |

* FP 5.2 Budget Analysis as a CSO Tool: Extent to which CSOs utilize budget analysis as a tool to build FP advocacy cases.

| | 1 | 2 | 3 | 4 | 5 | 6 | 7 | 8 | 9 | 10 | Don't know |
|---|---|---|---|---|---|---|---|---|---|---|---|
| 1-Lowest -> Highest-10 | ○ | ○ | ○ | ○ | ○ | ○ | ○ | ○ | ○ | ○ | ○ |

* FP 5.3 Facility-Based Service Delivery Support: Extent to which CSOs are involved in efforts to increase the use and/or quality of services, particularly through increasing the technical and/or operational capacity of CSO-partnered clinics to provide FP services.

| | 1 | 2 | 3 | 4 | 5 | 6 | 7 | 8 | 9 | 10 | Don't know |
|---|---|---|---|---|---|---|---|---|---|---|---|
| 1-Lowest -> Highest-10 | ○ | ○ | ○ | ○ | ○ | ○ | ○ | ○ | ○ | ○ | ○ |

* FP 5.4 Community-Based Service Delivery Support: Extent to which CSOs participate in bringing FP information and services to the communities where people live through activities such as supporting the capacity-building of community-level health workers, expanding the range of contraceptive methods provided by community health workers, educating lower cadre health workers, and/or undertaking information and education campaigns to increase the acceptability of contraception.

| | 1 | 2 | 3 | 4 | 5 | 6 | 7 | 8 | 9 | 10 | Don't know |
|---|---|---|---|---|---|---|---|---|---|---|---|
| 1-Lowest -> Highest-10 | ○ | ○ | ○ | ○ | ○ | ○ | ○ | ○ | ○ | ○ | ○ |



Rusatira JC, *et al. BMJ Open* 2023; 13:e059937. doi: 10.1136/bmjopen-2021-059937

* FP 5.5 Mobile Outreach for Community-Based Service Delivery Support: Extent to which CSOs support outreach models to equip health care providers with FP commodities, supplies, equipment and vehicles, which are then sent as specialist team to provide FP information and methods directly to communities.

| | 1 | 2 | 3 | 4 | 5 | 6 | 7 | 8 | 9 | 10 | Don't know |
|---|---|---|---|---|---|---|---|---|---|---|---|
| 1-Lowest -> Highest-10 | ○ | ○ | ○ | ○ | ○ | ○ | ○ | ○ | ○ | ○ | ○ |

* FP 5.6 Social Franchising: Extent to which CSOs are involved in social franchising opportunities/services to bring private sector services to clients at reduced prices.

| | 1 | 2 | 3 | 4 | 5 | 6 | 7 | 8 | 9 | 10 | Don't know |
|---|---|---|---|---|---|---|---|---|---|---|---|
| 1-Lowest -> Highest-10 | ○ | ○ | ○ | ○ | ○ | ○ | ○ | ○ | ○ | ○ | ○ |

* FP 5.7 mHealth: Extent to which CSOs are leveraging mHealth to utilize text messages and other mobile technologies to improve access to FP information and services.

| | 1 | 2 | 3 | 4 | 5 | 6 | 7 | 8 | 9 | 10 | Don't know |
|---|---|---|---|---|---|---|---|---|---|---|---|
| 1-Lowest -> Highest-10 | ○ | ○ | ○ | ○ | ○ | ○ | ○ | ○ | ○ | ○ | ○ |

* FP 5.8 Human Rights and Quality of Care: Extent to which CSOs advocate for and support rights-based approaches to FP to ensure FP service recipients experience good treatment, confidence in asking questions, and quality counselling, amongst other concerns.

| | 1 | 2 | 3 | 4 | 5 | 6 | 7 | 8 | 9 | 10 | Don't know |
|---|---|---|---|---|---|---|---|---|---|---|---|
| 1-Lowest -> Highest-10 | ○ | ○ | ○ | ○ | ○ | ○ | ○ | ○ | ○ | ○ | ○ |

* FP 5.9 Social and Behavior Change (SBC): Extent to which CSOs support SBC interventions relating, but not limited, to life skills and peer education, adult health education, school-based education, couple's communication, community mobilization, working through gatekeepers, and/or mass media interventions for a general public audience. These may be supported through implementation at the community or individual levels and may relate to outcomes such as increase in FP uptake and changes in FP knowledge and attitudes.

| | 1 | 2 | 3 | 4 | 5 | 6 | 7 | 8 | 9 | 10 | Don't know |
|---|---|---|---|---|---|---|---|---|---|---|---|
| 1-Lowest -> Highest-10 | ○ | ○ | ○ | ○ | ○ | ○ | ○ | ○ | ○ | ○ | ○ |

* FP 5.10 Youth: Extent to which CSOs support FP policies, interventions and programming targeting youth specifically.

| | 1 | 2 | 3 | 4 | 5 | 6 | 7 | 8 | 9 | 10 | Don't know |
|---|---|---|---|---|---|---|---|---|---|---|---|
| 1-Lowest -> Highest-10 | ○ | ○ | ○ | ○ | ○ | ○ | ○ | ○ | ○ | ○ | ○ |

* FP 5.11 Programming for Men: Extent to which CSOs support FP policies, interventions and programming for men.

| | 1 | 2 | 3 | 4 | 5 | 6 | 7 | 8 | 9 | 10 | Don't know |
|---|---|---|---|---|---|---|---|---|---|---|---|
| 1-Lowest -> Highest-10 | ○ | ○ | ○ | ○ | ○ | ○ | ○ | ○ | ○ | ○ | ○ |

* FP 5.12 Advocacy/Accountability: Extent to which CSOs support the training and capacity building of advocates and community members to understand policies, processes, and activities to increase community-led issue identification, collaborative problem solving and more targeted advocacy actions.

| | 1 | 2 | 3 | 4 | 5 | 6 | 7 | 8 | 9 | 10 | Don't know |
|---|---|---|---|---|---|---|---|---|---|---|---|
| 1-Lowest -> Highest-10 | ○ | ○ | ○ | ○ | ○ | ○ | ○ | ○ | ○ | ○ | ○ |

* FP 5.13 CSO-Led Assessment and Monitoring: Extent to which CSOs assess, monitor and report on the effectiveness of policies and programming to improve accountability at the policy and provider levels.

| | 1 | 2 | 3 | 4 | 5 | 6 | 7 | 8 | 9 | 10 | Don't know |
|---|---|---|---|---|---|---|---|---|---|---|---|
| 1-Lowest -> Highest-10 | ○ | ○ | ○ | ○ | ○ | ○ | ○ | ○ | ○ | ○ | ○ |

* FP 5.14 Forming Partnerships between CSOs: Extent to which CSOs have formed national alliances and regional partnerships to strengthen their positioning and potentially improve leadership strength and funding mechanisms.

| | 1 | 2 | 3 | 4 | 5 | 6 | 7 | 8 | 9 | 10 | Don't know |
|---|---|---|---|---|---|---|---|---|---|---|---|
| 1-Lowest -> Highest-10 | ○ | ○ | ○ | ○ | ○ | ○ | ○ | ○ | ○ | ○ | ○ |

FP 5.15 Comments: Please use this section to provide any additional comments about the level of effort, challenges, or successes in the country around the role of the civil society in FP. If you marked “I don’t know” for any of the questions, also use this section to explain why you gave that rating.

JOHNS HOPKINS
BLOOMBERG SCHOOL of PUBLIC HEALTH

Bill & Melinda Gates Institute for Population and Reproductive Health

## Demographic Dividend Effort Index Survey

### Module: Sectoral Resilience and Sustainability

**Considering the health and socio-economic impact of the COVID-19 and the likely impact on the DD progress, this DD effort index has integrated questions to assess the resilience and sustainability of systems in key sectors of the DD framework. The items included in the COVID-19 related questionnaire below follow the key dimensions of resilient systems and provide critical information on the potential of an effective response to emerging infectious disease threats or other public health emergencies.**

**Please score each item from 1 to 10, where 1 represents the lowest score (poor state/capability) and 10 represents the highest score (great state/capability).**

* Physical: facilities, equipment, system states, and capabilities.

| | 1 | 2 | 3 | 4 | 5 | 6 | 7 | 8 | 9 | 10 | Don't know |
|---|---|---|---|---|---|---|---|---|---|---|---|
| FP-M1 - Plan/Prepare - State and capability of equipment, personnel and FP program structure prior to the crisis event. (1 = poor state/capability; 10 = great state/capability) | ○ | ○ | ○ | ○ | ○ | ○ | ○ | ○ | ○ | ○ | ○ |
| FP-M2 - Absorb - Integration: The level of integration of FP services and programs to mitigate the effect of COVID-19 on FP | ○ | ○ | ○ | ○ | ○ | ○ | ○ | ○ | ○ | ○ | ○ |
| FP-M3 - Absorb - Integration: Extent to which effective supply chain management was able to mitigate the effect of COVID-19 on the supply/provision of FP commodities. | ○ | ○ | ○ | ○ | ○ | ○ | ○ | ○ | ○ | ○ | ○ |
| FP-M4 - Recover - Extent to which the FP sector has been able to induce change to recover the pre-COVID-19 level of functionality. | ○ | ○ | ○ | ○ | ○ | ○ | ○ | ○ | ○ | ○ | ○ |
| FP-M5 - Recover - Financing and Donor Management: Level of resource mobilization and allocation to mitigate the effect of COVID-19 on FP. | ○ | ○ | ○ | ○ | ○ | ○ | ○ | ○ | ○ | ○ | ○ |
| FP-M6 - Adapt - Extent to which changes have been made to improve the resilience of the FP sector. | ○ | ○ | ○ | ○ | ○ | ○ | ○ | ○ | ○ | ○ | ○ |

30

* Information: creation, manipulation, storage, and utilization of data.

| | 1 | 2 | 3 | 4 | 5 | 6 | 7 | 8 | 9 | 10 | Don't know |
|---|---|---|---|---|---|---|---|---|---|---|---|
| FP-M7 - Plan/Prepare - Extent to which FP-related data was sufficiently prepared, presented, analyzed and stored prior to the crisis event. | ○ | ○ | ○ | ○ | ○ | ○ | ○ | ○ | ○ | ○ | ○ |
| FP-M8 - Absorb - Extent to which the FP program has been able to conduct a real-time assessment of functionality, in anticipation of cascading losses in FP service provision. | ○ | ○ | ○ | ○ | ○ | ○ | ○ | ○ | ○ | ○ | ○ |
| FP-M9 - Recover - Extent to which FP data has been used to track the progress of recovery and to anticipate recovery scenarios. | ○ | ○ | ○ | ○ | ○ | ○ | ○ | ○ | ○ | ○ | ○ |
| FP-M10 - Adapt - Extent to which the FP sector is creating and improving FP data storage and real-time use protocols. | ○ | ○ | ○ | ○ | ○ | ○ | ○ | ○ | ○ | ○ | ○ |

* Cognitive: understanding, mental models, preconceptions, biases, and values.

| | 1 | 2 | 3 | 4 | 5 | 6 | 7 | 8 | 9 | 10 | Don't know |
|---|---|---|---|---|---|---|---|---|---|---|---|
| FP-M11 - Plan/Prepare - Extent to which the FP program design and operation decisions were prepared in anticipation of crisis events. | ○ | ○ | ○ | ○ | ○ | ○ | ○ | ○ | ○ | ○ | ○ |
| FP-M12 - Absorb - Extent to which the FP sector is responding with sufficient contingency protocols and proactive crisis management. | ○ | ○ | ○ | ○ | ○ | ○ | ○ | ○ | ○ | ○ | ○ |
| FP-M13 - Absorb - Accountability: Level of effort to ensure optimal accountability for the resources allocated to support FP as part of the response to the COVID-19. | ○ | ○ | ○ | ○ | ○ | ○ | ○ | ○ | ○ | ○ | ○ |
| FP-M14 - Recover - Extent to which decisions are oriented towards recovery, which employ evidence-based communication to community members to promote safe behavior. | ○ | ○ | ○ | ○ | ○ | ○ | ○ | ○ | ○ | ○ | ○ |
| FP-M15 - Recover - Awareness: Extent to which the FP sector is building confidence through communication regarding barriers posed by COVID-19 to support access to and uptake of FP. | ○ | ○ | ○ | ○ | ○ | ○ | ○ | ○ | ○ | ○ | ○ |
| FP-M16 - Adapt - Adaptive: Extent to which the national leadership has made changes to adapt and respond to the COVID-19 threats to FP. | ○ | ○ | ○ | ○ | ○ | ○ | ○ | ○ | ○ | ○ | ○ |

* Social: interaction, collaboration, and self-synchronization between individuals, entities and institutions.

32

| | 1 | 2 | 3 | 4 | 5 | 6 | 7 | 8 | 9 | 10 | Don't know |
|---|---|---|---|---|---|---|---|---|---|---|---|
| FP-M17 - Plan/Prepare - Extent to which training on outbreak/crisis management was conducted through, and leveraged, social networks, social capital and institutional and cultural norms prior to the crisis event. | ○ | ○ | ○ | ○ | ○ | ○ | ○ | ○ | ○ | ○ | ○ |
| FP-M18 - Absorb - Extent to which personnel and social institutions have been resourceful and accessible for the outbreak/crisis response. | ○ | ○ | ○ | ○ | ○ | ○ | ○ | ○ | ○ | ○ | ○ |
| FP-M19 - Absorb - Self-Regulating: Extent to which the national leadership had the authority to make sectoral changes in a timely manner through flexible infrastructure. | ○ | ○ | ○ | ○ | ○ | ○ | ○ | ○ | ○ | ○ | ○ |
| FP-M20 - Recover - Extent to which the FP sector is engaging with team and knowledge sharing to enhance systematic recovery. | ○ | ○ | ○ | ○ | ○ | ○ | ○ | ○ | ○ | ○ | ○ |
| FP-M21 - Recover - Diversity: The level of engagement of a multidisciplinary team to mitigate the effects of COVID-19 on FP. | ○ | ○ | ○ | ○ | ○ | ○ | ○ | ○ | ○ | ○ | ○ |
| FP-M22 - Adapt - Extent to which there are additions or changes being made to FP institutions, policies, trainings, programs and culture. | ○ | ○ | ○ | ○ | ○ | ○ | ○ | ○ | ○ | ○ | ○ |
| FP-M23 - Adapt - Leadership and Management: Level of leadership demonstrated by the FP leaders to mitigate the effect of COVID-19 on FP. | ○ | ○ | ○ | ○ | ○ | ○ | ○ | ○ | ○ | ○ | ○ |

* FP M-24 Timeline: Extent of timeliness of the Government reaction - communication and implementation of measures - to mitigate the immediate and longterm impact of COVID-19.
*Note: This refers to the entirety of the national reaction - not the reaction that is specific to your sector.*

| | 1 | 2 | 3 | 4 | 5 | 6 | 7 | 8 | 9 | 10 | Don't know |
|---|---|---|---|---|---|---|---|---|---|---|---|
| 1-Lowest -> Highest-10 | ○ | ○ | ○ | ○ | ○ | ○ | ○ | ○ | ○ | ○ | ○ |

FP M-25 Comments: Please use this section to provide any additional comments about the resilience and sustainability of the national FP systems based on your experience during the COVID-19 pandemics. If you marked “I don’t know” for any of the questions, also use this section to explain why you gave that rating.

JOHNS HOPKINS
BLOOMBERG SCHOOL
of PUBLIC HEALTH

Bill & Melinda Gates Institute for Population and Reproductive Health

## Demographic Dividend Effort Index Survey

### Maternal & Child Health (MCH) Questionnaire

**Please respond to the following questions based on your experience/expertise within this specific sector.**

**To give a summary picture of the national demographic dividend effort, please rate the following items. Score each item from 1 to 10, where 1 represents the lowest score (very weak or no effort) and 10 represents the highest score (strong effort). Where applicable, if a policy or activity is non-existent then mark your response as 0.**

Answer each item. All responses will be recorded in the format exemplified below:

| Lowest effort | 1 | 2 | 3 | 4 | 5 | 6 | 7 | 8 | 9 | 10 | Highest effort | I do not know |
|---|---|---|---|---|---|---|---|---|---|---|---|---|
| | | | | | | | | | | | | |

35

JOHNS HOPKINS
BLOOMBERG SCHOOL
of PUBLIC HEALTH

Bill & Melinda Gates Institute for Population and Reproductive Health

## Demographic Dividend Effort Index Survey

### Dimension 1. Policy/Policy making

* MCH 1.1 Adequate Policy: Ministry of Health policies toward pregnancy and delivery services that account for the breadth of requirements to ensure that every mother and newborn can survive.

| | 1 | 2 | 3 | 4 | 5 | 6 | 7 | 8 | 9 | 10 | Don't know |
|---|---|---|---|---|---|---|---|---|---|---|---|
| MCH 1.1.1 Extent to which reproductive, maternal and child health has been integrated into the national health policy/plan/strategy. | ○ | ○ | ○ | ○ | ○ | ○ | ○ | ○ | ○ | ○ | ○ |
| MCH 1.1.2 Extent to which national reproductive, maternal and child health strategy(ies) and implementation plan(s) have been made available. | ○ | ○ | ○ | ○ | ○ | ○ | ○ | ○ | ○ | ○ | ○ |

* MCH 1.2 Universal Access: Extent to which there are constitutional and legal entitlements that facilitate universal access to reproductive, maternal and child health services.

| | 1 | 2 | 3 | 4 | 5 | 6 | 7 | 8 | 9 | 10 | Don't know |
|---|---|---|---|---|---|---|---|---|---|---|---|
| 1-Lowest -> Highest-10 | ○ | ○ | ○ | ○ | ○ | ○ | ○ | ○ | ○ | ○ | ○ |

* MCH 1.3 Multisector Involvement: Extent to which policies are developed through adequate consultation with interested parties such as other ministries, NGOs, private practitioners, and women’s groups.

| | 1 | 2 | 3 | 4 | 5 | 6 | 7 | 8 | 9 | 10 | Don't know |
|---|---|---|---|---|---|---|---|---|---|---|---|
| 1-Lowest -> Highest-10 | ○ | ○ | ○ | ○ | ○ | ○ | ○ | ○ | ○ | ○ | ○ |

36

* MCH 1.4 Competent Service Providers: Extent to which policies are reasonable and evidence-based concerning which personnel can provide maternal health services (e.g. trained midwives can perform a wide range of medical procedures).

| | 1 | 2 | 3 | 4 | 5 | 6 | 7 | 8 | 9 | 10 | Don't know |
|---|---|---|---|---|---|---|---|---|---|---|---|
| 1-Lowest -> Highest-10 | ◯ | ◯ | ◯ | ◯ | ◯ | ◯ | ◯ | ◯ | ◯ | ◯ | ◯ |

* MCH 1.5 Abortion Legislation: Extent to which a favorable policy exists towards the legal provisioning of abortion services.

| | 1 | 2 | 3 | 4 | 5 | 6 | 7 | 8 | 9 | 10 | Don't know |
|---|---|---|---|---|---|---|---|---|---|---|---|
| 1-Lowest -> Highest-10 | ◯ | ◯ | ◯ | ◯ | ◯ | ◯ | ◯ | ◯ | ◯ | ◯ | ◯ |

* MCH 1.6. Post-Abortion Care Legislation: Extent to which a favorable policy exists toward the treatment of complications of abortions, including complications seen from illegal abortions.

| | 1 | 2 | 3 | 4 | 5 | 6 | 7 | 8 | 9 | 10 | Don't know |
|---|---|---|---|---|---|---|---|---|---|---|---|
| 1-Lowest -> Highest-10 | ◯ | ◯ | ◯ | ◯ | ◯ | ◯ | ◯ | ◯ | ◯ | ◯ | ◯ |

* MCH 1.7 Policy Reviews and Updates: Extent to which policies are regularly reviewed by high-level policymakers and reviews are used to update action plans.

| | 1 | 2 | 3 | 4 | 5 | 6 | 7 | 8 | 9 | 10 | Don't know |
|---|---|---|---|---|---|---|---|---|---|---|---|
| 1-Lowest -> Highest-10 | ◯ | ◯ | ◯ | ◯ | ◯ | ◯ | ◯ | ◯ | ◯ | ◯ | ◯ |

* MCH 1.8 High-Level Actor Placement: Extent and level to which the director of maternal and child health services is involved in high-level decision making.

| | 1 | 2 | 3 | 4 | 5 | 6 | 7 | 8 | 9 | 10 | Don't know |
|---|---|---|---|---|---|---|---|---|---|---|---|
| 1-Lowest -> Highest-10 | ◯ | ◯ | ◯ | ◯ | ◯ | ◯ | ◯ | ◯ | ◯ | ◯ | ◯ |

* MCH 1.9 Financing for MCH: Extent to which the government budgets for safe pregnancy, delivery, perinatal care (for facilities, personnel, supplies, etc.) and children’s health  is adequate for the needs, whether from the Ministry of Health, provincial/ county government or donor support.

| | 1 | 2 | 3 | 4 | 5 | 6 | 7 | 8 | 9 | 10 | Don't know |
|---|---|---|---|---|---|---|---|---|---|---|---|
| 1-Lowest -> Highest-10 | ◯ | ◯ | ◯ | ◯ | ◯ | ◯ | ◯ | ◯ | ◯ | ◯ | ◯ |

* MCH 1.10 Affordability: Extent to which all MCH-related services and drugs are affordable to all clients.

| | 1 | 2 | 3 | 4 | 5 | 6 | 7 | 8 | 9 | 10 | Don't know |
|---|---|---|---|---|---|---|---|---|---|---|---|
| 1-Lowest -> Highest-10 | ◯ | ◯ | ◯ | ◯ | ◯ | ◯ | ◯ | ◯ | ◯ | ◯ | ◯ |

* MCH 1.11 Private Sector: Extent to which the private sector (doctors, midwives, clinics) is active and covers a substantial share of pregnancy and delivery cases.

| | 1 | 2 | 3 | 4 | 5 | 6 | 7 | 8 | 9 | 10 | Don't know |
|---|---|---|---|---|---|---|---|---|---|---|---|
| 1-Lowest -> Highest-10 | ○ | ○ | ○ | ○ | ○ | ○ | ○ | ○ | ○ | ○ | ○ |

* MCH 1.12 MCH Department: Extent to which the Ministry of Health has a department that is specifically devoted to MCH.

| | 1 | 2 | 3 | 4 | 5 | 6 | 7 | 8 | 9 | 10 | Don't know |
|---|---|---|---|---|---|---|---|---|---|---|---|
| 1-Lowest -> Highest-10 | ○ | ○ | ○ | ○ | ○ | ○ | ○ | ○ | ○ | ○ | ○ |

* MCH 1.13 Potential Solutions: Extent to which the government recognizes potential solutions to MCH-related issues through the means of:

| | 1 | 2 | 3 | 4 | 5 | 6 | 7 | 8 | 9 | 10 | Don't know |
|---|---|---|---|---|---|---|---|---|---|---|---|
| MCH 1.13.1 Regulatory efficiency | ○ | ○ | ○ | ○ | ○ | ○ | ○ | ○ | ○ | ○ | ○ |
| MCH 1.13.2 Performance and accountability | ○ | ○ | ○ | ○ | ○ | ○ | ○ | ○ | ○ | ○ | ○ |
| MCH 1.13.3 A human rights-based approach to maternal, newborn and child health | ○ | ○ | ○ | ○ | ○ | ○ | ○ | ○ | ○ | ○ | ○ |
| MCH 1.13.4 Essential health infrastructure | ○ | ○ | ○ | ○ | ○ | ○ | ○ | ○ | ○ | ○ | ○ |
| MCH 1.13.5 Community engagement | ○ | ○ | ○ | ○ | ○ | ○ | ○ | ○ | ○ | ○ | ○ |

MCH 1.14 Comments: Please use this section to provide any additional comments about the level of effort, challenges, or successes in the country around policies/policymaking for MCH. If you marked “I don’t know” for any of the questions, also use this section to explain why you gave that rating

JOHNS HOPKINS
BLOOMBERG SCHOOL
of PUBLIC HEALTH

Bill & Melinda Gates Institute for Population and Reproductive Health

## Demographic Dividend Effort Index Survey

### Dimension 2. Services or Programs

* MCH 2.1 Obstetric Care Availability – All Centers: Extent to which all primary health facilities have trained staff, in place, who can provide obstetric care to:

| | 1 | 2 | 3 | 4 | 5 | 6 | 7 | 8 | 9 | 10 | Don't know |
|---|---|---|---|---|---|---|---|---|---|---|---|
| MCH 2.1.1 Properly manage postpartum hemorrhage cases. | ○ | ○ | ○ | ○ | ○ | ○ | ○ | ○ | ○ | ○ | ○ |
| MCH 2.1.2 Timely and properly administer antibiotics intravenously when needed. | ○ | ○ | ○ | ○ | ○ | ○ | ○ | ○ | ○ | ○ | ○ |
| MCH 2.1.3 Perform manual removal of retained placenta. | ○ | ○ | ○ | ○ | ○ | ○ | ○ | ○ | ○ | ○ | ○ |
| MCH 2.1.4 Perform vacuum aspiration of the uterus, using MVA (manual vacuum aspiration) or an electric suction device. | ○ | ○ | ○ | ○ | ○ | ○ | ○ | ○ | ○ | ○ | ○ |
| MCH 2.1.5 Use a partograph to determine when to refer. | ○ | ○ | ○ | ○ | ○ | ○ | ○ | ○ | ○ | ○ | ○ |
| MCH 2.1.6 Have transportation arrangements to quickly move a woman with obstructed labor to a district/sub-county hospital. | ○ | ○ | ○ | ○ | ○ | ○ | ○ | ○ | ○ | ○ | ○ |
| MCH 2.1.7 Have adequate antibiotic supplies on hand (sufficient supplies of the correct types). | ○ | ○ | ○ | ○ | ○ | ○ | ○ | ○ | ○ | ○ | ○ |

* MCH 2.2 Obstetric Care Availability – Secondary Healthcare Facilities: Extent to which all district/sub-county hospitals have trained staff, in place, who can:

| | 1 | 2 | 3 | 4 | 5 | 6 | 7 | 8 | 9 | 10 | Don't know |
|---|---|---|---|---|---|---|---|---|---|---|---|
| MCH 2.2.1 Provide all functions listed above for primary health facilities. | ○ | ○ | ○ | ○ | ○ | ○ | ○ | ○ | ○ | ○ | ○ |
| MCH 2.2.2 Perform blood transfusions (and have adequate supplies of safe blood on hand). | ○ | ○ | ○ | ○ | ○ | ○ | ○ | ○ | ○ | ○ | ○ |
| MCH 2.2.3 Perform Cesarean section. | ○ | ○ | ○ | ○ | ○ | ○ | ○ | ○ | ○ | ○ | ○ |

* MCH 2.3 Pregnancy-Related Services: Extent to which all pregnant women have adequate access to:

| | 1 | 2 | 3 | 4 | 5 | 6 | 7 | 8 | 9 | 10 | Don't know |
|---|---|---|---|---|---|---|---|---|---|---|---|
| MCH 2.3.1 Treatment for postpartum hemorrhage during or soon after delivery. | ○ | ○ | ○ | ○ | ○ | ○ | ○ | ○ | ○ | ○ | ○ |
| MCH 2.3.2 Management of obstructed labor. | ○ | ○ | ○ | ○ | ○ | ○ | ○ | ○ | ○ | ○ | ○ |
| MCH 2.3.3 Management of pre-eclampsia, eclampsia and its complications. | ○ | ○ | ○ | ○ | ○ | ○ | ○ | ○ | ○ | ○ | ○ |
| MCH 2.3.4 Treatment of post abortion care or for abortion complications. | ○ | ○ | ○ | ○ | ○ | ○ | ○ | ○ | ○ | ○ | ○ |
| MCH 2.3.5 Provision of safe abortion services. | ○ | ○ | ○ | ○ | ○ | ○ | ○ | ○ | ○ | ○ | ○ |
| MCH 2.3.6 Antenatal care during pregnancy. | ○ | ○ | ○ | ○ | ○ | ○ | ○ | ○ | ○ | ○ | ○ |
| MCH 2.3.7 Delivery care by a trained professional attendant. | ○ | ○ | ○ | ○ | ○ | ○ | ○ | ○ | ○ | ○ | ○ |
| MCH 2.3.8 Postpartum family planning services. | ○ | ○ | ○ | ○ | ○ | ○ | ○ | ○ | ○ | ○ | ○ |
| MCH 2.3.9 District/sub-county hospitals that are open 24 hours/day. | ○ | ○ | ○ | ○ | ○ | ○ | ○ | ○ | ○ | ○ | ○ |

* MCH 2.4 Ante-natal Care for Unmarried and HIV Positive Women: Extent to which, at all antenatal visits, all pregnant women:

| | 1 | 2 | 3 | 4 | 5 | 6 | 7 | 8 | 9 | 10 | Don't know |
|---|---|---|---|---|---|---|---|---|---|---|---|
| MCH 2.4.1 Receive WHO-recommended complete antenatal care protocol for HIV negative women | ◯ | ◯ | ◯ | ◯ | ◯ | ◯ | ◯ | ◯ | ◯ | ◯ | ◯ |
| MCH 2.4.2 Receive WHO-recommended complete antenatal care protocol for HIV positive women | ◯ | ◯ | ◯ | ◯ | ◯ | ◯ | ◯ | ◯ | ◯ | ◯ | ◯ |
| MCH 2.4.3 Receive WHO-recommended complete antenatal care protocol for young unmarried women. | ◯ | ◯ | ◯ | ◯ | ◯ | ◯ | ◯ | ◯ | ◯ | ◯ | ◯ |
| MCH 2.4.4 Are informed about danger signs of obstetric and newborn complications and are assisted in planning for any emergency. | ◯ | ◯ | ◯ | ◯ | ◯ | ◯ | ◯ | ◯ | ◯ | ◯ | ◯ |

* MCH 2.5 Newborn Care: Extent to which, for newborn care, all infants whether delivered at home or in a facility:

| | 1 | 2 | 3 | 4 | 5 | 6 | 7 | 8 | 9 | 10 | Don't know |
|---|---|---|---|---|---|---|---|---|---|---|---|
| MCH 2.5.1 Have their mouth and nasal passageways cleared | ○ | ○ | ○ | ○ | ○ | ○ | ○ | ○ | ○ | ○ | ○ |
| MCH 2.5.2 Are dried and kept warm immediately after birth | ○ | ○ | ○ | ○ | ○ | ○ | ○ | ○ | ○ | ○ | ○ |
| MCH 2.5.3 Receive vitamin A | ○ | ○ | ○ | ○ | ○ | ○ | ○ | ○ | ○ | ○ | ○ |
| MCH 2.5.4 Have their umbilical cord cut with a clean blade | ○ | ○ | ○ | ○ | ○ | ○ | ○ | ○ | ○ | ○ | ○ |
| MCH 2.5.5 Complete WHO recommended vaccination calendar | ○ | ○ | ○ | ○ | ○ | ○ | ○ | ○ | ○ | ○ | ○ |
| MCH 2.5.6 Benefit from exclusive breastfeeding promotion and education activities | ○ | ○ | ○ | ○ | ○ | ○ | ○ | ○ | ○ | ○ | ○ |
| MCH 2.5.7 Benefit from nutritional support programs when in need. | ○ | ○ | ○ | ○ | ○ | ○ | ○ | ○ | ○ | ○ | ○ |

* MCH 2.6 Family Planning – All Centers: Extent to which there exists proper provision of family planning at all health centers that:

| | 1 | 2 | 3 | 4 | 5 | 6 | 7 | 8 | 9 | 10 | Don't know |
|---|---|---|---|---|---|---|---|---|---|---|---|
| MCH 2.6.1 Routinely offer family planning services for post-abortion cases | ◯ | ◯ | ◯ | ◯ | ◯ | ◯ | ◯ | ◯ | ◯ | ◯ | ◯ |
| MCH 2.6.2 Routinely offer family planning at postpartum visits | ◯ | ◯ | ◯ | ◯ | ◯ | ◯ | ◯ | ◯ | ◯ | ◯ | ◯ |
| MCH 2.6.3 Have short-term and LARC contraceptive supplies regularly in stock | ◯ | ◯ | ◯ | ◯ | ◯ | ◯ | ◯ | ◯ | ◯ | ◯ | ◯ |
| MCH 2.6.4 Have trained staff, in place, who can provide services for LARCs. | ◯ | ◯ | ◯ | ◯ | ◯ | ◯ | ◯ | ◯ | ◯ | ◯ | ◯ |
| MCH 2.6.5 Have guidelines and protocols necessary for the provision of family planning services. | ◯ | ◯ | ◯ | ◯ | ◯ | ◯ | ◯ | ◯ | ◯ | ◯ | ◯ |



Rusatira JC, *et al. BMJ Open* 2023; 13:e059937. doi: 10.1136/bmjopen-2021-059937

* MCH 2.7 Family Planning – District/Sub-county Hospitals: Extent to which there exists proper provision of family planning at district/sub-county hospitals that:

| | 1 | 2 | 3 | 4 | 5 | 6 | 7 | 8 | 9 | 10 | Don't know |
|---|---|---|---|---|---|---|---|---|---|---|---|
| MCH 2.7.1 Routinely offer family planning services for post-abortion cases | ○ | ○ | ○ | ○ | ○ | ○ | ○ | ○ | ○ | ○ | ○ |
| MCH 2.7.2 Routinely offer family planning at postpartum visits | ○ | ○ | ○ | ○ | ○ | ○ | ○ | ○ | ○ | ○ | ○ |
| MCH 2.7.3 Have short term and LARC contraceptive supplies regularly in stock | ○ | ○ | ○ | ○ | ○ | ○ | ○ | ○ | ○ | ○ | ○ |
| MCH 2.7.4 Have trained staff, in place, who can provide services for LARCs | ○ | ○ | ○ | ○ | ○ | ○ | ○ | ○ | ○ | ○ | ○ |
| MCH 2.7.5 Can offer sterilization to female clients | ○ | ○ | ○ | ○ | ○ | ○ | ○ | ○ | ○ | ○ | ○ |
| MCH 2.7.6 Can offer sterilization to male clients | ○ | ○ | ○ | ○ | ○ | ○ | ○ | ○ | ○ | ○ | ○ |
| MCH 2.7.7 Have guidelines and protocols necessary for the provision of family planning services. | ○ | ○ | ○ | ○ | ○ | ○ | ○ | ○ | ○ | ○ | ○ |

* MCH 2.8 Childhood Illness: Extent to which the WHO-recommended case management of childhood illness is implemented regarding:

| | 1 | 2 | 3 | 4 | 5 | 6 | 7 | 8 | 9 | 10 | Don't know |
|---|---|---|---|---|---|---|---|---|---|---|---|
| MCH 2.8.1 Community case management of childhood illnesses | ○ | ○ | ○ | ○ | ○ | ○ | ○ | ○ | ○ | ○ | ○ |
| MCH 2.8.2 Effectiveness of the referral services from community level to the health facilities | ○ | ○ | ○ | ○ | ○ | ○ | ○ | ○ | ○ | ○ | ○ |

MCH 2.9 Comments: Please use this section to provide any additional comments about the level of effort, challenges, or successes in the country around services or programs for MCH. If you marked “I don’t know” for any of the questions, also use this section to explain why you gave that rating.

JOHNS HOPKINS
BLOOMBERG SCHOOL
of PUBLIC HEALTH

Bill & Melinda Gates Institute for Population and Reproductive Health

## Demographic Dividend Effort Index Survey

### Dimension 3. Advocacy

* MCH 3.1 Government Messages: Extent to which high officials in the government, including the Ministry of Health, issue frequent statements to all stakeholders including the press to support improvements for safe pregnancy, safe delivery and healthy childhood.

| | 1 | 2 | 3 | 4 | 5 | 6 | 7 | 8 | 9 | 10 | Don't know |
|---|---|---|---|---|---|---|---|---|---|---|---|
| 1-Lowest -> Highest-10 | ◯ | ◯ | ◯ | ◯ | ◯ | ◯ | ◯ | ◯ | ◯ | ◯ | ◯ |

* MCH 3.2 Mass Media: Extent to which the national program uses the mass media to educate the public about symptoms of pregnancy complications, safe childbirth and healthy childhood.

| | 1 | 2 | 3 | 4 | 5 | 6 | 7 | 8 | 9 | 10 | Don't know |
|---|---|---|---|---|---|---|---|---|---|---|---|
| 1-Lowest -> Highest-10 | ◯ | ◯ | ◯ | ◯ | ◯ | ◯ | ◯ | ◯ | ◯ | ◯ | ◯ |

* MCH 3.3 Community-Level Media: Extent to which community groups take part in systematic programs to educate the public about safe pregnancy, safe delivery and healthy childhood.

| | 1 | 2 | 3 | 4 | 5 | 6 | 7 | 8 | 9 | 10 | Don't know |
|---|---|---|---|---|---|---|---|---|---|---|---|
| 1-Lowest -> Highest-10 | ◯ | ◯ | ◯ | ◯ | ◯ | ◯ | ◯ | ◯ | ◯ | ◯ | ◯ |

* MCH 3.4 Educational Materials: Extent to which the appropriate ministry (MOH) supplies adequate educational materials (posters, pamphlets, etc.) to delivery facilities to instruct clients about safe practices.

| | 1 | 2 | 3 | 4 | 5 | 6 | 7 | 8 | 9 | 10 | Don't know |
|---|---|---|---|---|---|---|---|---|---|---|---|
| 1-Lowest -> Highest-10 | ◯ | ◯ | ◯ | ◯ | ◯ | ◯ | ◯ | ◯ | ◯ | ◯ | ◯ |

* MCH 3.5 Self-Review of Maternal Mortality Cases: Extent to which each health facility follows a regular procedure to review and learn from every case of a maternal death in the facility.

| | 1 | 2 | 3 | 4 | 5 | 6 | 7 | 8 | 9 | 10 | Don't know |
|---|---|---|---|---|---|---|---|---|---|---|---|
| 1-Lowest -> Highest-10 | ○ | ○ | ○ | ○ | ○ | ○ | ○ | ○ | ○ | ○ | ○ |

* MCH 3.6 Informing Policies with New Evidence: Extent to which each health facility shares lessons from maternal death cases to the ministries to inform new policies or interventions.

| | 1 | 2 | 3 | 4 | 5 | 6 | 7 | 8 | 9 | 10 | Don't know |
|---|---|---|---|---|---|---|---|---|---|---|---|
| 1-Lowest -> Highest-10 | ○ | ○ | ○ | ○ | ○ | ○ | ○ | ○ | ○ | ○ | ○ |

MCH 3.7 Comments: Please use this section to provide any additional comments about the level of effort, challenges, or successes in the country around advocacy for MCH. If you marked “I don’t know” for any of the questions, also use this section to explain why you gave that rating

JOHNS HOPKINS
BLOOMBERG SCHOOL
of PUBLIC HEALTH

Bill & Melinda Gates Institute for Population and Reproductive Health

## Demographic Dividend Effort Index Survey

### Dimension 4. Research

* MCH 4.1 Strength of Observatory/Stakeholder/Technical Working Group for MCH: Extent to which country-level MCH stakeholders (e.g., stakeholder leadership group, technical working group, MCH observatory, country coordination and facilitation group):

| | 1 | 2 | 3 | 4 | 5 | 6 | 7 | 8 | 9 | 10 | Don't know |
|---|---|---|---|---|---|---|---|---|---|---|---|
| MCH 4.1.1 Have representation in government, training institutions, civil society, nongovernmental and faith-based organizations, professional associations, the private sector, etc. | ○ | ○ | ○ | ○ | ○ | ○ | ○ | ○ | ○ | ○ | ○ |
| MCH 4.1.2 Meet regularly, report and recommend policy for senior management within the appropriate ministries | ○ | ○ | ○ | ○ | ○ | ○ | ○ | ○ | ○ | ○ | ○ |
| MCH 4.1.3 Make an impact on MCH within the country | ○ | ○ | ○ | ○ | ○ | ○ | ○ | ○ | ○ | ○ | ○ |

* MCH 4.2 MCH Research Strategy: Extent to which national documents include a comprehensive strategy/approach on MCH-related research and is shared among partners.

| | 1 | 2 | 3 | 4 | 5 | 6 | 7 | 8 | 9 | 10 | Don't know |
|---|---|---|---|---|---|---|---|---|---|---|---|
| 1-Lowest -> Highest-10 | ○ | ○ | ○ | ○ | ○ | ○ | ○ | ○ | ○ | ○ | ○ |

* MCH 4.3 Data-Gathering Partners:

| | 1 | 2 | 3 | 4 | 5 | 6 | 7 | 8 | 9 | 10 | Don't know |
|---|---|---|---|---|---|---|---|---|---|---|---|
| MCH 4.3.1 Extent to which research is undertaken by government agencies. (This may include statistical agencies/bureaus and/or ministries of economics/finances, education, health, gender, etc.) | ○ | ○ | ○ | ○ | ○ | ○ | ○ | ○ | ○ | ○ | ○ |
| MCH 4.3.2 Extent to which data-gathering is undertaken by research institutions. | ○ | ○ | ○ | ○ | ○ | ○ | ○ | ○ | ○ | ○ | ○ |
| MCH 4.3.3 Extent to which data-gathering is undertaken by independent researchers. | ○ | ○ | ○ | ○ | ○ | ○ | ○ | ○ | ○ | ○ | ○ |

* MCH 4.4 Topical Research:

| | 1 | 2 | 3 | 4 | 5 | 6 | 7 | 8 | 9 | 10 | Don't know |
|---|---|---|---|---|---|---|---|---|---|---|---|
| MCH 4.4.1 Extent to which maternal mortality is researched. | ○ | ○ | ○ | ○ | ○ | ○ | ○ | ○ | ○ | ○ | ○ |
| MCH 4.4.2 Extent to which infant and child mortality is researched. | ○ | ○ | ○ | ○ | ○ | ○ | ○ | ○ | ○ | ○ | ○ |
| MCH 4.4.3 Extent to which family planning and post-partum family planning is researched. | ○ | ○ | ○ | ○ | ○ | ○ | ○ | ○ | ○ | ○ | ○ |
| MCH 4.4.4 Extent to which MCH service provision/access and provider quality is researched. | ○ | ○ | ○ | ○ | ○ | ○ | ○ | ○ | ○ | ○ | ○ |

* MCH 4.5 Quality/Coverage of Data: Extent to which current research/data is disaggregated, where relevant, on the basis of sex, age, geographic location, ethnicity, education, income quintile and disability status. Extent to which the country participates in standardized tests and its ranking.

| | 1 | 2 | 3 | 4 | 5 | 6 | 7 | 8 | 9 | 10 | Don't know |
|---|---|---|---|---|---|---|---|---|---|---|---|
| MCH 4.5.1 Extent to which a routine statistical system provides good periodic information on supplies and facilities. | ○ | ○ | ○ | ○ | ○ | ○ | ○ | ○ | ○ | ○ | ○ |
| MCH 4.5.2 Extent to which a routine statistical system provides good periodic information on MCH services and personnel. | ○ | ○ | ○ | ○ | ○ | ○ | ○ | ○ | ○ | ○ | ○ |
| MCH 4.5.3 Extent to which the routine statistical system provides good periodic information on MCH needs among populations in different communities. | ○ | ○ | ○ | ○ | ○ | ○ | ○ | ○ | ○ | ○ | ○ |
| MCH 4.5.4 Extent to which evaluations of the health monitoring and information system are conducted and applied to ensure the reliability of data. | ○ | ○ | ○ | ○ | ○ | ○ | ○ | ○ | ○ | ○ | ○ |
| MCH 4.5.5 Extent to which evaluations of the health monitoring and information system are conducted and applied to explore inequalities in MNCH services and burdens. | ○ | ○ | ○ | ○ | ○ | ○ | ○ | ○ | ○ | ○ | ○ |

* MCH 4.6 Record-Keeping: Extent to which systems for client recordkeeping, clinic reporting and feedback of results are adequate, including being connected at all levels (federal, state/province, county, etc.).

| | 1 | 2 | 3 | 4 | 5 | 6 | 7 | 8 | 9 | 10 | Don't know |
|---|---|---|---|---|---|---|---|---|---|---|---|
| 1-Lowest -> Highest-10 | ○ | ○ | ○ | ○ | ○ | ○ | ○ | ○ | ○ | ○ | ○ |

* MCH 4.7 Quality Research Institutions: Extent to which the country has the capacity to support and sustain research institutions that develop research/collect data to ensure high quality MCH services.

| | 1 | 2 | 3 | 4 | 5 | 6 | 7 | 8 | 9 | 10 | Don't know |
|---|---|---|---|---|---|---|---|---|---|---|---|
| 1-Lowest -> Highest-10 | ◯ | ◯ | ◯ | ◯ | ◯ | ◯ | ◯ | ◯ | ◯ | ◯ | ◯ |

* MCH 4.8 Evaluation: Extent to which program statistics, surveys, and small studies are used by specialized staff to report on program operations and measure progress.

| | 1 | 2 | 3 | 4 | 5 | 6 | 7 | 8 | 9 | 10 | Don't know |
|---|---|---|---|---|---|---|---|---|---|---|---|
| 1-Lowest -> Highest-10 | ◯ | ◯ | ◯ | ◯ | ◯ | ◯ | ◯ | ◯ | ◯ | ◯ | ◯ |

* MCH 4.9 Management's Use of Evaluation Findings: Extent to which local-level program managers use research and evaluation findings to improve programming in ways suggested by findings.

| | 1 | 2 | 3 | 4 | 5 | 6 | 7 | 8 | 9 | 10 | Don't know |
|---|---|---|---|---|---|---|---|---|---|---|---|
| 1-Lowest -> Highest-10 | ◯ | ◯ | ◯ | ◯ | ◯ | ◯ | ◯ | ◯ | ◯ | ◯ | ◯ |

* MCH 4.10 Ministerial Use of Evaluation Findings: Extent to which the Ministry administrators and policymakers systematically utilize data to inform policies and interventions to address MCH-related issues.

| | 1 | 2 | 3 | 4 | 5 | 6 | 7 | 8 | 9 | 10 | Don't know |
|---|---|---|---|---|---|---|---|---|---|---|---|
| 1-Lowest -> Highest-10 | ◯ | ◯ | ◯ | ◯ | ◯ | ◯ | ◯ | ◯ | ◯ | ◯ | ◯ |

* MCH 4.11 Dissemination of Information to Other Implementing Bodies: Extent to which information is shared or spread across geographical areas and throughout various levels (national, state/province, localities).

| | 1 | 2 | 3 | 4 | 5 | 6 | 7 | 8 | 9 | 10 | Don't know |
|---|---|---|---|---|---|---|---|---|---|---|---|
| 1-Lowest -> Highest-10 | ◯ | ◯ | ◯ | ◯ | ◯ | ◯ | ◯ | ◯ | ◯ | ◯ | ◯ |

MCH 4.12 Comments: Please use this section to provide any additional comments about the level of effort, challenges, or successes in the country around MCH research. If you marked "I don't know" for any of the questions, also use this section to explain why you gave that rating.

JOHNS HOPKINS
BLOOMBERG SCHOOL of PUBLIC HEALTH

Bill & Melinda Gates Institute for Population and Reproductive Health

Demographic Dividend Effort Index Survey

## Dimension 5. Civil Society Organizations (CSOs)

* MCH 5.1 CSO Actor Power: Extent to which CSO actors are in strong placements within institutions or technical working groups or networks concerned with MCH.

| | 1 | 2 | 3 | 4 | 5 | 6 | 7 | 8 | 9 | 10 | Don't know |
|---|---|---|---|---|---|---|---|---|---|---|---|
| 1-Lowest -> Highest-10 | ◯ | ◯ | ◯ | ◯ | ◯ | ◯ | ◯ | ◯ | ◯ | ◯ | ◯ |

* MCH 5.2 Budget Analysis as a CSO Tool: Extent to which CSOs utilize budget analysis as a tool to build MCH advocacy cases.

| | 1 | 2 | 3 | 4 | 5 | 6 | 7 | 8 | 9 | 10 | Don't know |
|---|---|---|---|---|---|---|---|---|---|---|---|
| 1-Lowest -> Highest-10 | ◯ | ◯ | ◯ | ◯ | ◯ | ◯ | ◯ | ◯ | ◯ | ◯ | ◯ |

* MCH 5.3 Facility-Based Service Delivery Support: Extent to which CSOs are involved in efforts to increase the use and/or quality of services, particularly through increasing the technical and/or operational capacity of CSO-partnered clinics to provide MCH services.

| | 1 | 2 | 3 | 4 | 5 | 6 | 7 | 8 | 9 | 10 | Don't know |
|---|---|---|---|---|---|---|---|---|---|---|---|
| 1-Lowest -> Highest-10 | ◯ | ◯ | ◯ | ◯ | ◯ | ◯ | ◯ | ◯ | ◯ | ◯ | ◯ |

* MCH 5.4 Community-Based Service Delivery Support: Extent to which CSOs participate in bringing MCH information and services to the communities where people live through activities such as supporting the capacity-building of community-level health workers, expanding the range of MCH services provided by community health workers, educating lower cadre health workers, and/or undertaking information and education campaigns.

| | 1 | 2 | 3 | 4 | 5 | 6 | 7 | 8 | 9 | 10 | Don't know |
|---|---|---|---|---|---|---|---|---|---|---|---|
| 1-Lowest -> Highest-10 | ◯ | ◯ | ◯ | ◯ | ◯ | ◯ | ◯ | ◯ | ◯ | ◯ | ◯ |

* MCH 5.5 Mobile Outreach for Community-Based Service Delivery Support: Extent to which CSOs support outreach models to equip health care providers with MCH-related commodities, supplies, equipment and vehicles, which are then sent as specialist team to provide MCH services directly to communities.

| | 1 | 2 | 3 | 4 | 5 | 6 | 7 | 8 | 9 | 10 | Don't know |
|---|---|---|---|---|---|---|---|---|---|---|---|
| 1-Lowest -> Highest-10 | ○ | ○ | ○ | ○ | ○ | ○ | ○ | ○ | ○ | ○ | ○ |

* MCH 5.6 Social Franchising: Extent to which CSOs are involved with social franchising opportunities/services to bring private sector services to clients at reduced prices of MCH services.

| | 1 | 2 | 3 | 4 | 5 | 6 | 7 | 8 | 9 | 10 | Don't know |
|---|---|---|---|---|---|---|---|---|---|---|---|
| 1-Lowest -> Highest-10 | ○ | ○ | ○ | ○ | ○ | ○ | ○ | ○ | ○ | ○ | ○ |

* MCH 5.7 mHealth: Extent to which CSOs are leveraging mHealth to utilize text messages and other mobile technologies to improve access to MCH information and services.

| | 1 | 2 | 3 | 4 | 5 | 6 | 7 | 8 | 9 | 10 | Don't know |
|---|---|---|---|---|---|---|---|---|---|---|---|
| 1-Lowest -> Highest-10 | ○ | ○ | ○ | ○ | ○ | ○ | ○ | ○ | ○ | ○ | ○ |

* MCH 5.8 Human Rights and Quality of Care: Extent to which CSOs advocate for and support rights-based approaches to MCH to ensure MCH service recipients experience good treatment, confidence in asking questions, and quality counselling, amongst other concerns.

| | 1 | 2 | 3 | 4 | 5 | 6 | 7 | 8 | 9 | 10 | Don't know |
|---|---|---|---|---|---|---|---|---|---|---|---|
| 1-Lowest -> Highest-10 | ○ | ○ | ○ | ○ | ○ | ○ | ○ | ○ | ○ | ○ | ○ |

* MCH 5.9 Social and Behavior Change (SBC): Extent to which CSOs support SBC interventions relating, but not limited, to life skills and peer education, adult health education, school-based education, couple's communication, community mobilization, working through gatekeepers, and/or mass media interventions for a general public audience.

| | 1 | 2 | 3 | 4 | 5 | 6 | 7 | 8 | 9 | 10 | Don't know |
|---|---|---|---|---|---|---|---|---|---|---|---|
| 1-Lowest -> Highest-10 | ○ | ○ | ○ | ○ | ○ | ○ | ○ | ○ | ○ | ○ | ○ |

* MCH 5.10 Advocacy/Accountability: Extent to which CSOs support the training and capacity building of advocates and community members to understand policies, processes, and activities to increase community-led issue identification, collaborative problem solving and more targeted advocacy actions.

| | 1 | 2 | 3 | 4 | 5 | 6 | 7 | 8 | 9 | 10 | Don't know |
|---|---|---|---|---|---|---|---|---|---|---|---|
| 1-Lowest -> Highest-10 | ○ | ○ | ○ | ○ | ○ | ○ | ○ | ○ | ○ | ○ | ○ |

* MCH 5.11 CSO-Led Assessment and Monitoring: Extent to which CSOs assess, monitor and report on the effectiveness of policies and programming to improve accountability at the policy and provider levels.

| | 1 | 2 | 3 | 4 | 5 | 6 | 7 | 8 | 9 | 10 | Don't know |
|---|---|---|---|---|---|---|---|---|---|---|---|
| 1-Lowest -> Highest-10 | ○ | ○ | ○ | ○ | ○ | ○ | ○ | ○ | ○ | ○ | ○ |

* MCH 5.12 Forming Partnerships between CSOs: Extent to which CSOs have formed national alliances and regional partnerships to strengthen their positioning and potentially improve leadership strength and funding mechanisms for MCH.

| | 1 | 2 | 3 | 4 | 5 | 6 | 7 | 8 | 9 | 10 | Don't know |
|---|---|---|---|---|---|---|---|---|---|---|---|
| 1-Lowest -> Highest-10 | ○ | ○ | ○ | ○ | ○ | ○ | ○ | ○ | ○ | ○ | ○ |

MCH 5.13 Comments: Please use this section to provide any additional comments about the level of effort, challenges, or successes in the country around the involvement of the civil society in MCH. If you marked “I don’t know” for any of the questions, also use this section to explain why you gave that rating

JOHNS HOPKINS
BLOOMBERG SCHOOL
of PUBLIC HEALTH

Bill & Melinda Gates Institute for Population and Reproductive Health

## Demographic Dividend Effort Index Survey

### Module: Sectoral Resilience and Sustainability

**Considering the health and socio-economic impact of the COVID-19 and the likely impact on the DD progress, this DD effort index has integrated questions to assess the resilience and sustainability of systems in key sectors of the DD framework. The items included in the COVID-19 related questionnaire below follow the key dimensions of resilient systems and provide critical information on the potential of an effective response to emerging infectious disease threats or other public health emergencies.**

**Please score each item from 1 to 10, where 1 represents the lowest score (poor state/capability) and 10 represents the highest score (great state/capability).**

* Physical: facilities, equipment, system states, and capabilities.

| | 1 | 2 | 3 | 4 | 5 | 6 | 7 | 8 | 9 | 10 | Don't know |
|---|---|---|---|---|---|---|---|---|---|---|---|
| MCH-M1 - Plan/Prepare - State and capability of equipment, personnel and MCH program structure prior to the crisis event. (1 = poor state/capability; 10 = great state/capability) | | | | | | | | | | | |
| MCH-M2 - Absorb - Integration: The level of integration of MCH services and programs to mitigate the effect of COVID-19 on MCH. | | | | | | | | | | | |
| MCH-M3 - Absorb - Integration: Extent to which effective supply chain management was able to mitigate the effect of COVID-19 on the supply/provision of MCH-related commodities. | | | | | | | | | | | |
| MCH-M4 - Recover - Extent to which the MCH program has been able to induce change to recover the pre-COVID-19 level of functionality. | | | | | | | | | | | |
| MCH-M5 - Recover - Financing and Donor Management: Level of resources mobilization and allocation to mitigate the effect of COVID-19 on MCH. | | | | | | | | | | | |
| MCH-M6 - Adapt - Extent to which changes have been made to improve the resilience of the MCH sector. | | | | | | | | | | | |

* Information: creation, manipulation, storage, and utilization of data.

| | 1 | 2 | 3 | 4 | 5 | 6 | 7 | 8 | 9 | 10 | Don't know |
|---|---|---|---|---|---|---|---|---|---|---|---|
| MCH-M7 - Plan/Prepare - Extent to which MCH-related data was sufficiently prepared, presented, analyzed and stored prior to the crisis event. | ○ | ○ | ○ | ○ | ○ | ○ | ○ | ○ | ○ | ○ | ○ |
| MCH-M8 - Absorb - Awareness: Extent of the surveillance capacity to detect the MCH limitations/threats posed by COVID-19. | ○ | ○ | ○ | ○ | ○ | ○ | ○ | ○ | ○ | ○ | ○ |
| MCH-M9 - Recover - Extent to which MCH data has been used to track the progress of recovery and to anticipate recovery scenarios. | ○ | ○ | ○ | ○ | ○ | ○ | ○ | ○ | ○ | ○ | ○ |
| MCH-M10 - Adapt - Extent to which the MCH sector is creating and improving MCH data storage and real-time use protocols. | ○ | ○ | ○ | ○ | ○ | ○ | ○ | ○ | ○ | ○ | ○ |

* Cognitive: understanding, mental models, preconceptions, biases, and values.

| | 1 | 2 | 3 | 4 | 5 | 6 | 7 | 8 | 9 | 10 | Don't know |
|---|---|---|---|---|---|---|---|---|---|---|---|
| MCH-M11 - Plan/Prepare - Extent to which the MCH sector design and operation decisions were prepared in anticipation of crisis events. | ○ | ○ | ○ | ○ | ○ | ○ | ○ | ○ | ○ | ○ | ○ |
| MCH-M12 - Absorb - Extent to which the MCH sector is responding with sufficient contingency protocols and proactive crisis management. | ○ | ○ | ○ | ○ | ○ | ○ | ○ | ○ | ○ | ○ | ○ |
| MCH-M13 - Absorb - Accountability: Level of effort to ensure optimal accountability for the resources allocated to support MCH as part of the response to the COVID-19. | ○ | ○ | ○ | ○ | ○ | ○ | ○ | ○ | ○ | ○ | ○ |
| MCH-M14 - Recover - Extent to which decisions are oriented towards recovery, which employ evidence-based communication to community members to promote safe behavior. | ○ | ○ | ○ | ○ | ○ | ○ | ○ | ○ | ○ | ○ | ○ |
| MCH-M15 - Recover - Awareness: Extent to which the MCH sector is building confidence through communication regarding barriers posed by COVID-19 to support access to and uptake of MCH services and programs. | ○ | ○ | ○ | ○ | ○ | ○ | ○ | ○ | ○ | ○ | ○ |
| MCH-M16 - Adapt – Adaptive: Extent to which the national leadership had the authority to make changes to adapt and respond to the COVID-19 threats to MCH services and programs. | ○ | ○ | ○ | ○ | ○ | ○ | ○ | ○ | ○ | ○ | ○ |

* Social: interaction, collaboration, and self-synchronization between individuals, entities and institutions.

| | 1 | 2 | 3 | 4 | 5 | 6 | 7 | 8 | 9 | 10 | Don't know |
|---|---|---|---|---|---|---|---|---|---|---|---|
| MCH-M17 - Plan/Prepare - Extent to which training on outbreak/crisis management was conducted through, and which leveraged, social networks, social capital and institutional and cultural norms prior to the crisis event. | ○ | ○ | ○ | ○ | ○ | ○ | ○ | ○ | ○ | ○ | ○ |
| MCH-M18 - Absorb - Extent to which personnel and social institutions have been resourceful and accessible for the outbreak/crisis response. | ○ | ○ | ○ | ○ | ○ | ○ | ○ | ○ | ○ | ○ | ○ |
| MCH-M19 - Absorb - Self-Regulating: Extent to which the national leadership had the authority to make sectoral changes in a timely manner through flexible infrastructure. | ○ | ○ | ○ | ○ | ○ | ○ | ○ | ○ | ○ | ○ | ○ |
| MCH-M20 - Recover - Extent to which the MCH sector is engaging with team and knowledge sharing to enhance systematic recovery. | ○ | ○ | ○ | ○ | ○ | ○ | ○ | ○ | ○ | ○ | ○ |
| MCH-M21 - Recover - Diversity: The level of engagement of a multidisciplinary team to mitigate the effect of COVID19 on MCH. | ○ | ○ | ○ | ○ | ○ | ○ | ○ | ○ | ○ | ○ | ○ |
| MCH-M22 - Adapt - Extent to which there are additions or changes being made to MCH institutions, policies, trainings, programs and culture. | ○ | ○ | ○ | ○ | ○ | ○ | ○ | ○ | ○ | ○ | ○ |

| | 1 | 2 | 3 | 4 | 5 | 6 | 7 | 8 | 9 | 10 | Don't know |
|---|---|---|---|---|---|---|---|---|---|---|---|
| MCH-M23 - Adapt - Leadership and Management: Level of leadership demonstrated by the MCH leaders to mitigate the effect of COVID-19 on MCH. | ◯ | ◯ | ◯ | ◯ | ◯ | ◯ | ◯ | ◯ | ◯ | ◯ | ◯ |

* MCH M-24 Timeline: Extent of timeliness of the Government reaction - communication and implementation of measures - to mitigate the immediate and longterm impact of COVID-19.
*Note: This refers to the entirety of the national reaction - not the reaction that is specific to your sector.*

| | 1 | 2 | 3 | 4 | 5 | 6 | 7 | 8 | 9 | 10 | Don't know |
|---|---|---|---|---|---|---|---|---|---|---|---|
| 1-Lowest -> Highest-10 | ◯ | ◯ | ◯ | ◯ | ◯ | ◯ | ◯ | ◯ | ◯ | ◯ | ◯ |

MCH M-25 Comments: Please use this section to provide any additional comments about the resilience and sustainability of the national MCH systems based on your experience during the COVID-19 pandemics. If you marked “I don’t know” for any of the questions, also use this section to explain why you gave that rating.

JOHNS HOPKINS
BLOOMBERG SCHOOL
of PUBLIC HEALTH

Bill & Melinda Gates Institute for Population and Reproductive Health

# Demographic Dividend Effort Index Survey

## Education (ED) Questionnaire

**Please respond to the following questions based on your experience/expertise within this specific sector.**

**To give a summary picture of the national demographic dividend effort, please rate the following items. Score each item from 1 to 10, where 1 represents the lowest score (very weak or no effort) and 10 represents the highest score (strong effort). Where applicable, if a policy or activity is non-existent then mark your response as 0.**

Answer each item. All responses will be recorded in the format exemplified below:

| Lowest effort | 1 | 2 | 3 | 4 | 5 | 6 | 7 | 8 | 9 | 10 | Highest effort | I do not know |
|---|---|---|---|---|---|---|---|---|---|---|---|---|
| | | | | | | | | | | | | |

JOHNS HOPKINS
BLOOMBERG SCHOOL
of PUBLIC HEALTH

Bill & Melinda Gates Institute for Population and Reproductive Health

## Demographic Dividend Effort Index Survey

### Dimension 1. Policy / Policy making

* ED 1.1 Quality: Extent to which there are national plans/strategies/programs to improve the quality of learning in schools.

| | 1 | 2 | 3 | 4 | 5 | 6 | 7 | 8 | 9 | 10 | Don't know |
|---|---|---|---|---|---|---|---|---|---|---|---|
| 1-Lowest -> Highest-10 | ○ | ○ | ○ | ○ | ○ | ○ | ○ | ○ | ○ | ○ | ○ |

* ED 1.2 Disadvantaged Groups:

| | 1 | 2 | 3 | 4 | 5 | 6 | 7 | 8 | 9 | 10 | Don't know |
|---|---|---|---|---|---|---|---|---|---|---|---|
| ED 1.2.1. Extent to which strategies have been developed and implemented/deployed to overcome learning obstacles for pregnant girls and adolescent mothers. | ○ | ○ | ○ | ○ | ○ | ○ | ○ | ○ | ○ | ○ | ○ |
| ED 1.2.2 Extent to which strategies have been developed and implemented/deployed to overcome learning obstacles for disabled populations. | ○ | ○ | ○ | ○ | ○ | ○ | ○ | ○ | ○ | ○ | ○ |

* ED 1.3 Teachers: Extent to which teacher recruitment, development and management issues are included in policy aimed at improving learning outcomes.

| | 1 | 2 | 3 | 4 | 5 | 6 | 7 | 8 | 9 | 10 | Don't know |
|---|---|---|---|---|---|---|---|---|---|---|---|
| 1-Lowest -> Highest-10 | ○ | ○ | ○ | ○ | ○ | ○ | ○ | ○ | ○ | ○ | ○ |

* ED 1.4 Teachers' Accountability: Extent to which national plans include specific measures, including monitoring and evaluation, that aim to hold teachers' accountable for improving learning.

| | 1 | 2 | 3 | 4 | 5 | 6 | 7 | 8 | 9 | 10 | Don't know |
|---|---|---|---|---|---|---|---|---|---|---|---|
| 1-Lowest -> Highest-10 | ○ | ○ | ○ | ○ | ○ | ○ | ○ | ○ | ○ | ○ | ○ |

* ED 1.5 Teacher Quality: Extent to which education policies/strategies/programs include verifiable indicators for teacher quality.

| | 1 | 2 | 3 | 4 | 5 | 6 | 7 | 8 | 9 | 10 | Don't know |
|---|---|---|---|---|---|---|---|---|---|---|---|
| 1-Lowest -> Highest-10 | ○ | ○ | ○ | ○ | ○ | ○ | ○ | ○ | ○ | ○ | ○ |

* ED 1.6 Collaboration in Education: Extent to which the policy makes provisions to engage multiple stakeholders in education (e.g. schools, school-communities, parents, teachers, local government, parent-teacher-student committees, etc.).

| | 1 | 2 | 3 | 4 | 5 | 6 | 7 | 8 | 9 | 10 | Don't know |
|---|---|---|---|---|---|---|---|---|---|---|---|
| 1-Lowest -> Highest-10 | ○ | ○ | ○ | ○ | ○ | ○ | ○ | ○ | ○ | ○ | ○ |

* ED 1.7 Gender Parity SDG: Extent to which national policies/plans/strategies consider the SDG goal for complete gender parity at all levels of education.

| | 1 | 2 | 3 | 4 | 5 | 6 | 7 | 8 | 9 | 10 | Don't know |
|---|---|---|---|---|---|---|---|---|---|---|---|
| 1-Lowest -> Highest-10 | ○ | ○ | ○ | ○ | ○ | ○ | ○ | ○ | ○ | ○ | ○ |

* ED 1.8 Education Financing: Extent to which new education plans include financing proposals with budgets allocated for learning and teaching reforms.

| | 1 | 2 | 3 | 4 | 5 | 6 | 7 | 8 | 9 | 10 | Don't know |
|---|---|---|---|---|---|---|---|---|---|---|---|
| 1-Lowest -> Highest-10 | ○ | ○ | ○ | ○ | ○ | ○ | ○ | ○ | ○ | ○ | ○ |

* ED 1.9 Gender Parity Planning: Extent to which there are national plans/strategies to reach 100% enrollment with gender parity in:

| | 1 | 2 | 3 | 4 | 5 | 6 | 7 | 8 | 9 | 10 | Don't know |
|---|---|---|---|---|---|---|---|---|---|---|---|
| ED 1.9.1 Primary Education | ○ | ○ | ○ | ○ | ○ | ○ | ○ | ○ | ○ | ○ | ○ |
| ED 1.9.2 Secondary Education | ○ | ○ | ○ | ○ | ○ | ○ | ○ | ○ | ○ | ○ | ○ |
| ED 1.9.3 Tertiary Education | ○ | ○ | ○ | ○ | ○ | ○ | ○ | ○ | ○ | ○ | ○ |

* ED 1.10 Adult Illiteracy: Extent to which national policies/plans/strategies aim to address illiteracy.

| | 1 | 2 | 3 | 4 | 5 | 6 | 7 | 8 | 9 | 10 | Don't know |
|---|---|---|---|---|---|---|---|---|---|---|---|
| ED 1.10.1 Extent to which national policies/plans/strategies aim to address adult illiteracy. | ○ | ○ | ○ | ○ | ○ | ○ | ○ | ○ | ○ | ○ | ○ |
| ED 1.10.2 Extent to which national policies/plans/strategies that aim to address adult illiteracy address gendered disparities of this issue. | ○ | ○ | ○ | ○ | ○ | ○ | ○ | ○ | ○ | ○ | ○ |

* ED 1.11 Out-of-School Children: Extent to which policies address the plight of out-of-school children to reintegrate them into the school system or provide alternative opportunities for their educational development.

| | 1 | 2 | 3 | 4 | 5 | 6 | 7 | 8 | 9 | 10 | Don't know |
|---|---|---|---|---|---|---|---|---|---|---|---|
| 1-Lowest -> Highest-10 | ○ | ○ | ○ | ○ | ○ | ○ | ○ | ○ | ○ | ○ | ○ |

* ED 1.12 Technology Integration: Extent to which strategies have been developed and implemented/deployed to integrate information communication and technology in education.

| | 1 | 2 | 3 | 4 | 5 | 6 | 7 | 8 | 9 | 10 | Don't know |
|---|---|---|---|---|---|---|---|---|---|---|---|
| 1-Lowest -> Highest-10 | ○ | ○ | ○ | ○ | ○ | ○ | ○ | ○ | ○ | ○ | ○ |

* ED 1.13. Technology Literacy: Extent to which resources have been allocated to improve computer literacy across education levels.

| | 1 | 2 | 3 | 4 | 5 | 6 | 7 | 8 | 9 | 10 | Don't know |
|---|---|---|---|---|---|---|---|---|---|---|---|
| 1-Lowest -> Highest-10 | ○ | ○ | ○ | ○ | ○ | ○ | ○ | ○ | ○ | ○ | ○ |

ED 1.14. Comments: Please use this section to provide any additional comments about the level of effort, challenges, or successes in the country around policies/policymaking for ED. If you marked “I don’t know” for any of the questions, also use this section to explain why you gave that rating.

JOHNS HOPKINS
BLOOMBERG SCHOOL
of PUBLIC HEALTH

Bill & Melinda Gates Institute for Population and Reproductive Health

## Demographic Dividend Effort Index Survey

### Dimension 2. Services or Programs

* ED 2.1 Teacher Deployment: Extent to which strategies/plans are used to ensure teachers are deployed to areas of the country where they are most needed.

| | 1 | 2 | 3 | 4 | 5 | 6 | 7 | 8 | 9 | 10 | Don't know |
|---|---|---|---|---|---|---|---|---|---|---|---|
| 1-Lowest -> Highest-10 | ◯ | ◯ | ◯ | ◯ | ◯ | ◯ | ◯ | ◯ | ◯ | ◯ | ◯ |

* ED 2.2 Teacher Quality: Extent to which teacher quality is recognized as a key factor in improving learning outcomes.

| | 1 | 2 | 3 | 4 | 5 | 6 | 7 | 8 | 9 | 10 | Don't know |
|---|---|---|---|---|---|---|---|---|---|---|---|
| ED 2.2.1 Extent to which the quality of teacher education is emphasized in wider education policies/plans/strategies. | ◯ | ◯ | ◯ | ◯ | ◯ | ◯ | ◯ | ◯ | ◯ | ◯ | ◯ |
| ED 2.2.2 Extent to which the quality of training and continuous professional development is emphasized in wider education policies/plans/strategies. | ◯ | ◯ | ◯ | ◯ | ◯ | ◯ | ◯ | ◯ | ◯ | ◯ | ◯ |

* ED 2.3 Gender Parity: Extent to which resources have been utilized to improve equality in education between boys and girls.

| | 1 | 2 | 3 | 4 | 5 | 6 | 7 | 8 | 9 | 10 | Don't know |
|---|---|---|---|---|---|---|---|---|---|---|---|
| 1-Lowest -> Highest-10 | ◯ | ◯ | ◯ | ◯ | ◯ | ◯ | ◯ | ◯ | ◯ | ◯ | ◯ |

65

* ED 2.4 Parental Involvement in Education: Extent to which parents are involved in the development and execution of the education strategies (i.e. through the involvement of parent-teacher committees).

| | 1 | 2 | 3 | 4 | 5 | 6 | 7 | 8 | 9 | 10 | Don't know |
|---|---|---|---|---|---|---|---|---|---|---|---|
| 1-Lowest -> Highest-10 | ○ | ○ | ○ | ○ | ○ | ○ | ○ | ○ | ○ | ○ | ○ |

* ED 2.5 Standardized Assessments: Extent to which all schools utilize standardized school and student assessments in line with specifications made by the Ministry of Education.

| | 1 | 2 | 3 | 4 | 5 | 6 | 7 | 8 | 9 | 10 | Don't know |
|---|---|---|---|---|---|---|---|---|---|---|---|
| 1-Lowest -> Highest-10 | ○ | ○ | ○ | ○ | ○ | ○ | ○ | ○ | ○ | ○ | ○ |

* ED 2.6. Use of Assessment to Inform Progress: Extent to which adjustments to schools are made based on results from national standardized school assessments.

| | 1 | 2 | 3 | 4 | 5 | 6 | 7 | 8 | 9 | 10 | Don't know |
|---|---|---|---|---|---|---|---|---|---|---|---|
| 1-Lowest -> Highest-10 | ○ | ○ | ○ | ○ | ○ | ○ | ○ | ○ | ○ | ○ | ○ |

ED 2.7 Comments: Please use this section to provide any additional comments about the level of effort, challenges, or successes in the country around services or programs for ED. If you marked “I don’t know” for any of the questions, also use this section to explain why you gave that rating.

JOHNS HOPKINS
BLOOMBERG SCHOOL
*of* PUBLIC HEALTH

Bill & Melinda Gates Institute for Population and Reproductive Health

## Demographic Dividend Effort Index Survey

### Dimension 3. Advocacy

* ED 3.1 Equality: Extent to which equity and equality in education have been highlighted by advocates.

| | 1 | 2 | 3 | 4 | 5 | 6 | 7 | 8 | 9 | 10 | Don't know |
|---|---|---|---|---|---|---|---|---|---|---|---|
| 1-Lowest -> Highest-10 | ○ | ○ | ○ | ○ | ○ | ○ | ○ | ○ | ○ | ○ | ○ |

* ED 3.2 Quality: Extent to which quality of education takes part in advocacy-related evaluations of the education system.

| | 1 | 2 | 3 | 4 | 5 | 6 | 7 | 8 | 9 | 10 | Don't know |
|---|---|---|---|---|---|---|---|---|---|---|---|
| 1-Lowest -> Highest-10 | ○ | ○ | ○ | ○ | ○ | ○ | ○ | ○ | ○ | ○ | ○ |

* ED 3.3 Teachers' Obstacles: Extent to which the needs of teachers have been highlighted by advocates.

| | 1 | 2 | 3 | 4 | 5 | 6 | 7 | 8 | 9 | 10 | Don't know |
|---|---|---|---|---|---|---|---|---|---|---|---|
| 1-Lowest -> Highest-10 | ○ | ○ | ○ | ○ | ○ | ○ | ○ | ○ | ○ | ○ | ○ |

* ED 3.4 Institutions' Obstacles: Extent to which the challenges of educational institutions have been highlighted in different advocacy efforts.

| | 1 | 2 | 3 | 4 | 5 | 6 | 7 | 8 | 9 | 10 | Don't know |
|---|---|---|---|---|---|---|---|---|---|---|---|
| 1-Lowest -> Highest-10 | ○ | ○ | ○ | ○ | ○ | ○ | ○ | ○ | ○ | ○ | ○ |

ED 3.5 Comments: Please use this section to provide any additional comments about the level of effort, challenges, or successes in the country around advocacy for ED. If you marked "I don't know" for any of the questions, also use this section to explain why you gave that rating

67

JOHNS HOPKINS
BLOOMBERG SCHOOL of PUBLIC HEALTH

Bill & Melinda Gates Institute for Population and Reproductive Health

## Demographic Dividend Effort Index Survey

### Dimension 4. Research

* ED 4.1 Strength of Observatory/Stakeholder/Technical Working Group for ED: Extent to which country-level ED stakeholders (e.g., stakeholder leadership group, technical working group, ED observatory, country coordination and facilitation group):

| | 1 | 2 | 3 | 4 | 5 | 6 | 7 | 8 | 9 | 10 | Don't know |
|---|---|---|---|---|---|---|---|---|---|---|---|
| ED 4.1.1 Have representation in government, training institutions, civil society, nongovernmental and faith-based organizations, professional associations, the private sector, etc. | ○ | ○ | ○ | ○ | ○ | ○ | ○ | ○ | ○ | ○ | ○ |
| ED 4.1.2 Meet regularly, report and recommend policy for senior management within the appropriate ministries | ○ | ○ | ○ | ○ | ○ | ○ | ○ | ○ | ○ | ○ | ○ |
| ED 4.1.3 Make an impact on education within the country | ○ | ○ | ○ | ○ | ○ | ○ | ○ | ○ | ○ | ○ | ○ |

* ED 4.2 Education/Research Strategy: Extent to which the national education plan and/or other national documents include a comprehensive strategy/approach on education-related research, shared among partners.

| | 1 | 2 | 3 | 4 | 5 | 6 | 7 | 8 | 9 | 10 | Don't know |
|---|---|---|---|---|---|---|---|---|---|---|---|
| 1-Lowest -> Highest-10 | ○ | ○ | ○ | ○ | ○ | ○ | ○ | ○ | ○ | ○ | ○ |

* ED 4.3 Data Gathering Partners:

| | 1 | 2 | 3 | 4 | 5 | 6 | 7 | 8 | 9 | 10 | Don't know |
|---|---|---|---|---|---|---|---|---|---|---|---|
| ED 4.3.1 Extent to which research is undertaken by government agencies. (This may include statistical agencies/bureaus and/or ministries of economics/finances, education, health, gender, etc.) | ○ | ○ | ○ | ○ | ○ | ○ | ○ | ○ | ○ | ○ | ○ |
| ED 4.3.2 Extent to which data-gathering is undertaken by research institutions. | ○ | ○ | ○ | ○ | ○ | ○ | ○ | ○ | ○ | ○ | ○ |
| ED 4.3.3 Extent to which data-gathering is undertaken by independent researchers. | ○ | ○ | ○ | ○ | ○ | ○ | ○ | ○ | ○ | ○ | ○ |

* ED 4.4 Topical Research:

| | 1 | 2 | 3 | 4 | 5 | 6 | 7 | 8 | 9 | 10 | Don't know |
|---|---|---|---|---|---|---|---|---|---|---|---|
| ED 4.4.1 Extent to which gender disparities in education are researched. | ○ | ○ | ○ | ○ | ○ | ○ | ○ | ○ | ○ | ○ | ○ |
| ED 4.4.2 Extent to which quality of education is researched. | ○ | ○ | ○ | ○ | ○ | ○ | ○ | ○ | ○ | ○ | ○ |
| ED 4.4.3 Extent to which the obstacles faced by teachers are researched. | ○ | ○ | ○ | ○ | ○ | ○ | ○ | ○ | ○ | ○ | ○ |
| ED 4.4.4 Extent to which the obstacles faced by institutions are researched. | ○ | ○ | ○ | ○ | ○ | ○ | ○ | ○ | ○ | ○ | ○ |

* ED 4.5 Quality/Coverage of Data: Extent to which current research/data is sex and age disaggregated. Extent to which the country participates in standardized tests and its ranking.

| | 1 | 2 | 3 | 4 | 5 | 6 | 7 | 8 | 9 | 10 | Don't know |
|---|---|---|---|---|---|---|---|---|---|---|---|
| ED 4.5.1 Extent to which a routine statistical system (using institution/school-based information) provides good periodic information on supplies and facilities. | ◯ | ◯ | ◯ | ◯ | ◯ | ◯ | ◯ | ◯ | ◯ | ◯ | ◯ |
| ED 4.5.2 Extent to which a routine statistical system (using institution/school-based information) provides good periodic information on education services and personnel. | ◯ | ◯ | ◯ | ◯ | ◯ | ◯ | ◯ | ◯ | ◯ | ◯ | ◯ |
| ED 4.5.3 Extent to which the routine statistical system provides good periodic information on education needs among populations in different communities. | ◯ | ◯ | ◯ | ◯ | ◯ | ◯ | ◯ | ◯ | ◯ | ◯ | ◯ |
| ED 4.5.4 Extent to which evaluations of the education monitoring and information system are conducted and applied to ensure the reliability of data. | ◯ | ◯ | ◯ | ◯ | ◯ | ◯ | ◯ | ◯ | ◯ | ◯ | ◯ |

* ED 4.6 Record-Keeping: Extent to which systems for education-related recordkeeping, reporting and feedback of results are adequate.

| | 1 | 2 | 3 | 4 | 5 | 6 | 7 | 8 | 9 | 10 | Don't know |
|---|---|---|---|---|---|---|---|---|---|---|---|
| 1-Lowest -> Highest-10 | ◯ | ◯ | ◯ | ◯ | ◯ | ◯ | ◯ | ◯ | ◯ | ◯ | ◯ |

* ED 4.7 Quality Research Institutions: Extent to which the country has the capacity to support and sustain research institutions that develop research/collect data that inform efforts to improve education.

| | 1 | 2 | 3 | 4 | 5 | 6 | 7 | 8 | 9 | 10 | Don't know |
|---|---|---|---|---|---|---|---|---|---|---|---|
| 1-Lowest -> Highest-10 | ◯ | ◯ | ◯ | ◯ | ◯ | ◯ | ◯ | ◯ | ◯ | ◯ | ◯ |

* ED 4.8 Evaluation: Extent to which education program statistics, surveys, and small studies are used by specialized staff to report on program operations and measure progress.

| | 1 | 2 | 3 | 4 | 5 | 6 | 7 | 8 | 9 | 10 | Don't know |
|---|---|---|---|---|---|---|---|---|---|---|---|
| 1-Lowest -> Highest-10 | ◯ | ◯ | ◯ | ◯ | ◯ | ◯ | ◯ | ◯ | ◯ | ◯ | ◯ |

* ED 4.9 Management's Use of Evaluation Findings: Extent to which local-level education program managers use research and evaluation findings to improve these programs in ways suggested by findings.

| | 1 | 2 | 3 | 4 | 5 | 6 | 7 | 8 | 9 | 10 | Don't know |
|---|---|---|---|---|---|---|---|---|---|---|---|
| 1-Lowest -> Highest-10 | ◯ | ◯ | ◯ | ◯ | ◯ | ◯ | ◯ | ◯ | ◯ | ◯ | ◯ |

* ED 4.10 Ministerial Use of Evaluation Findings: Extent to which the relevant ministry administrators (i.e. ministries of education) systematically utilize data to inform policies and interventions to address education-related issues.

| | 1 | 2 | 3 | 4 | 5 | 6 | 7 | 8 | 9 | 10 | Don't know |
|---|---|---|---|---|---|---|---|---|---|---|---|
| 1-Lowest -> Highest-10 | ◯ | ◯ | ◯ | ◯ | ◯ | ◯ | ◯ | ◯ | ◯ | ◯ | ◯ |

* ED 4.11 Dissemination of Information to Other Implementing Bodies:

| | 1 | 2 | 3 | 4 | 5 | 6 | 7 | 8 | 9 | 10 | Don't know |
|---|---|---|---|---|---|---|---|---|---|---|---|
| ED 4.11.1 Extent to which information is shared or spread across geographical areas | ◯ | ◯ | ◯ | ◯ | ◯ | ◯ | ◯ | ◯ | ◯ | ◯ | ◯ |
| ED. 4.11.2 Extent to which information is shared or spread throughout various levels (national, state/province, localities). | ◯ | ◯ | ◯ | ◯ | ◯ | ◯ | ◯ | ◯ | ◯ | ◯ | ◯ |

ED 4.12 Comments: Please use this section to provide any additional comments about the level of effort, challenges, or successes in the country around research in education. If you marked "I don't know" for any of the questions, also use this section to explain why you gave that rating

JOHNS HOPKINS
BLOOMBERG SCHOOL of PUBLIC HEALTH

Bill & Melinda Gates Institute for Population and Reproductive Health

## Demographic Dividend Effort Index Survey

### Dimension 5. Civil Society Organizations (CSOs)

* ED 5.1 CSO Actor Power: Extent to which CSO actors are in strong placements within institutions or technical working groups or networks concerned with education.

| | 1 | 2 | 3 | 4 | 5 | 6 | 7 | 8 | 9 | 10 | Don't know |
|---|---|---|---|---|---|---|---|---|---|---|---|
| 1-Lowest -> Highest-10 | ◯ | ◯ | ◯ | ◯ | ◯ | ◯ | ◯ | ◯ | ◯ | ◯ | ◯ |

* ED 5.2 Budget Analysis as a CSO Tool: Extent to which CSOs utilize budget analysis as a tool to build education advocacy cases.

| | 1 | 2 | 3 | 4 | 5 | 6 | 7 | 8 | 9 | 10 | Don't know |
|---|---|---|---|---|---|---|---|---|---|---|---|
| 1-Lowest -> Highest-10 | ◯ | ◯ | ◯ | ◯ | ◯ | ◯ | ◯ | ◯ | ◯ | ◯ | ◯ |

* ED 5.3 Community inclusion: Extent to which CSOs are involved in efforts to explore the opportunities to improve the quality of education.

| | 1 | 2 | 3 | 4 | 5 | 6 | 7 | 8 | 9 | 10 | Don't know |
|---|---|---|---|---|---|---|---|---|---|---|---|
| 1-Lowest -> Highest-10 | ◯ | ◯ | ◯ | ◯ | ◯ | ◯ | ◯ | ◯ | ◯ | ◯ | ◯ |

* ED 5.4 Community Involvement in Education: Extent to which CSOs participate in bringing education information and services to the communities where people live through activities such as supporting the capacity-building of teachers and parents and/or undertaking information and education campaigns to increase the commitment to education in families and communities.

| | 1 | 2 | 3 | 4 | 5 | 6 | 7 | 8 | 9 | 10 | Don't know |
|---|---|---|---|---|---|---|---|---|---|---|---|
| 1-Lowest -> Highest-10 | ◯ | ◯ | ◯ | ◯ | ◯ | ◯ | ◯ | ◯ | ◯ | ◯ | ◯ |

* ED 5.5 Social Franchising: Extent to which CSOs are involved with social franchising opportunities/services to bring quality education services to children and families at reduced costs.

| | 1 | 2 | 3 | 4 | 5 | 6 | 7 | 8 | 9 | 10 | Don't know |
|---|---|---|---|---|---|---|---|---|---|---|---|
| 1-Lowest -> Highest-10 | ○ | ○ | ○ | ○ | ○ | ○ | ○ | ○ | ○ | ○ | ○ |

* ED 5.6 Technology: Extent to which CSOs are leveraging information communication technology (ICT) to improve access to quality education for various communities.

| | 1 | 2 | 3 | 4 | 5 | 6 | 7 | 8 | 9 | 10 | Don't know |
|---|---|---|---|---|---|---|---|---|---|---|---|
| 1-Lowest -> Highest-10 | ○ | ○ | ○ | ○ | ○ | ○ | ○ | ○ | ○ | ○ | ○ |

* ED 5.7 Human Rights and Access to/Quality of Education: Extent to which CSOs advocate for and support rights-based approaches to education to ensure students experience good treatment, confidence in asking questions, and quality education, amongst other concerns.

| | 1 | 2 | 3 | 4 | 5 | 6 | 7 | 8 | 9 | 10 | Don't know |
|---|---|---|---|---|---|---|---|---|---|---|---|
| 1-Lowest -> Highest-10 | ○ | ○ | ○ | ○ | ○ | ○ | ○ | ○ | ○ | ○ | ○ |

* ED 5.8 Social and Behavior Change (SBC): Extent to which CSOs support SBC interventions relating, but not limited, to life skills and peer education, adult health education, school-based education, community mobilization, and/or mass media interventions for a general public audience.

| | 1 | 2 | 3 | 4 | 5 | 6 | 7 | 8 | 9 | 10 | Don't know |
|---|---|---|---|---|---|---|---|---|---|---|---|
| 1-Lowest -> Highest-10 | ○ | ○ | ○ | ○ | ○ | ○ | ○ | ○ | ○ | ○ | ○ |

* ED 5.9 Advocacy/Accountability: Extent to which CSOs support the training and capacity building of advocates and community members to understand policies, processes, and activities to increase community-led issue identification, collaborative problem solving and more targeted advocacy actions to improve education.

| | 1 | 2 | 3 | 4 | 5 | 6 | 7 | 8 | 9 | 10 | Don't know |
|---|---|---|---|---|---|---|---|---|---|---|---|
| 1-Lowest -> Highest-10 | ○ | ○ | ○ | ○ | ○ | ○ | ○ | ○ | ○ | ○ | ○ |

* ED 5.10 CSO-Led Assessment and Monitoring: Extent to which CSOs assess, monitor and report on the effectiveness of policies and programming to improve accountability in education at the policy and teachers' levels.

| | 1 | 2 | 3 | 4 | 5 | 6 | 7 | 8 | 9 | 10 | Don't know |
|---|---|---|---|---|---|---|---|---|---|---|---|
| 1-Lowest -> Highest-10 | ○ | ○ | ○ | ○ | ○ | ○ | ○ | ○ | ○ | ○ | ○ |

* ED 5.11 Forming Partnerships between CSOs: Extent to which CSOs have formed national alliances and regional partnerships to strengthen their positioning and potentially improve leadership strength and funding mechanisms to improve the quality of education.

| | 1 | 2 | 3 | 4 | 5 | 6 | 7 | 8 | 9 | 10 | Don't know |
|---|---|---|---|---|---|---|---|---|---|---|---|
| 1-Lowest -> Highest-10 | ◯ | ◯ | ◯ | ◯ | ◯ | ◯ | ◯ | ◯ | ◯ | ◯ | ◯ |

ED 5.12 Comments: Please use this section to provide any additional comments about the level of effort, challenges, or successes in the country around the role of the civil society in ED. If you marked “I don’t know” for any of the questions, also use this section to explain why you gave that rating

JOHNS HOPKINS
BLOOMBERG SCHOOL
*of* PUBLIC HEALTH

Bill & Melinda Gates Institute for Population and Reproductive Health

## Demographic Dividend Effort Index Survey

### Module: Sectoral Resilience and Sustainability

**Considering the health and socio-economic impact of the COVID-19 and the likely impact on the DD progress, this DD effort index has integrated questions to assess the resilience and sustainability of systems in key sectors of the DD framework. The items included in the COVID-19 related questionnaire below follow the key dimensions of resilient systems and provide critical information on the potential of an effective response to emerging infectious disease threats or other public health emergencies.**

**Please score each item from 1 to 10, where 1 represents the lowest score (poor state/capability) and 10 represents the highest score (great state/capability).**

* Physical: facilities, equipment, system states, and capabilities.

| | 1 | 2 | 3 | 4 | 5 | 6 | 7 | 8 | 9 | 10 | Don't know |
|---|---|---|---|---|---|---|---|---|---|---|---|
| ED-M1 - Plan/Prepare - State and capability of equipment, personnel and ED sector structure prior to the crisis event. (1 = poor state/capability; 10 = great state/capability) | ◯ | ◯ | ◯ | ◯ | ◯ | ◯ | ◯ | ◯ | ◯ | ◯ | ◯ |
| ED-M2 - Absorb - Extent to which COVID-19 was recognized within the ED sector and the performance of the system was able to maintain functionality. | ◯ | ◯ | ◯ | ◯ | ◯ | ◯ | ◯ | ◯ | ◯ | ◯ | ◯ |
| ED-M3 - Absorb - Integration: The level of integration of ED services and programs to mitigate the effect of COVID19 on ED. | ◯ | ◯ | ◯ | ◯ | ◯ | ◯ | ◯ | ◯ | ◯ | ◯ | ◯ |
| ED-M4 - Recover - Extent to which the ED sector has been able to induce change to recover the pre-COVID-19 level of functionality. | ◯ | ◯ | ◯ | ◯ | ◯ | ◯ | ◯ | ◯ | ◯ | ◯ | ◯ |
| ED-M5 - Recover - Financing and Donor Management: Level of resources mobilization and allocation to mitigate the effect of COVID-19 on ED. | ◯ | ◯ | ◯ | ◯ | ◯ | ◯ | ◯ | ◯ | ◯ | ◯ | ◯ |
| ED-M6 - Adapt - Extent to which changes have been made to improve the resilience of the ED sector. | ◯ | ◯ | ◯ | ◯ | ◯ | ◯ | ◯ | ◯ | ◯ | ◯ | ◯ |

* Information: creation, manipulation, storage, and utilization of data.

| | 1 | 2 | 3 | 4 | 5 | 6 | 7 | 8 | 9 | 10 | Don't know |
|---|---|---|---|---|---|---|---|---|---|---|---|
| ED-M7 - Plan/Prepare - Extent to which ED-related data was sufficiently prepared, presented, analyzed and stored prior to the crisis event. | ○ | ○ | ○ | ○ | ○ | ○ | ○ | ○ | ○ | ○ | ○ |
| ED-M8 - Absorb - Awareness: Extent of the surveillance capacity to detect the ED limitations/threats posed by COVID-19. (This includes data that is focused on the variety of education-related concerns that the education sector is facing at each level [primary, secondary, tertiary] of schooling). | ○ | ○ | ○ | ○ | ○ | ○ | ○ | ○ | ○ | ○ | ○ |
| ED-M9 - Recover - Extent to which ED data has been used to track the progress of recovery and to anticipate recovery scenarios. | ○ | ○ | ○ | ○ | ○ | ○ | ○ | ○ | ○ | ○ | ○ |
| ED-M10 - Adapt - Extent to which the ED system is creating and improving ED data storage and real-time use protocols. | ○ | ○ | ○ | ○ | ○ | ○ | ○ | ○ | ○ | ○ | ○ |

* Cognitive: understanding, mental models, preconceptions, biases, and values.

| | 1 | 2 | 3 | 4 | 5 | 6 | 7 | 8 | 9 | 10 | Don't know |
|---|---|---|---|---|---|---|---|---|---|---|---|
| ED-M11 - Plan/Prepare - Extent to which the ED system design and operation decisions were prepared in anticipation of crisis events. | ○ | ○ | ○ | ○ | ○ | ○ | ○ | ○ | ○ | ○ | ○ |
| ED-M12 - Absorb - Extent to which the ED system is responding with sufficient contingency protocols and proactive crisis management. | ○ | ○ | ○ | ○ | ○ | ○ | ○ | ○ | ○ | ○ | ○ |

| | 1 | 2 | 3 | 4 | 5 | 6 | 7 | 8 | 9 | 10 | Don't know |
|---|---|---|---|---|---|---|---|---|---|---|---|
| ED-M13 - Absorb - Accountability: Level of effort to ensure optimal accountability for the resources allocated to support ED as part of the response to the COVID-19. | ○ | ○ | ○ | ○ | ○ | ○ | ○ | ○ | ○ | ○ | ○ |
| ED-M14 - Recover - Extent to which decisions are oriented towards recovery, which employ evidence-based communication to community members to promote safe behavior. | ○ | ○ | ○ | ○ | ○ | ○ | ○ | ○ | ○ | ○ | ○ |
| ED-M15 - Recover - Awareness: Extent to which the ED sector is building confidence through communication regarding barriers posed by COVID-19 to support access to and uptake of ED services and programs. | ○ | ○ | ○ | ○ | ○ | ○ | ○ | ○ | ○ | ○ | ○ |
| ED-M16 - Adapt - Extent to which the ED sector is designing new system configurations, objectives and decision criteria. | ○ | ○ | ○ | ○ | ○ | ○ | ○ | ○ | ○ | ○ | ○ |
| ED-M17 - Adapt - Adaptive: Level of adaptation to respond to the COVID-19 threats to ED. | ○ | ○ | ○ | ○ | ○ | ○ | ○ | ○ | ○ | ○ | ○ |

* Social: interaction, collaboration, and self-synchronization between individuals, entities and institutions.

| | 1 | 2 | 3 | 4 | 5 | 6 | 7 | 8 | 9 | 10 | Don't know |
|---|---|---|---|---|---|---|---|---|---|---|---|
| ED-M18 - Plan/Prepare - Extent to which training on outbreak/crisis management was conducted through, and which leveraged, social networks, social capital and institutional and cultural norms prior to the crisis event. | ○ | ○ | ○ | ○ | ○ | ○ | ○ | ○ | ○ | ○ | ○ |

| | 1 | 2 | 3 | 4 | 5 | 6 | 7 | 8 | 9 | 10 | Don't know |
|---|---|---|---|---|---|---|---|---|---|---|---|
| ED-M19 - Absorb - Extent to which personnel and social institutions have been resourceful and accessible for the outbreak/crisis response. | ○ | ○ | ○ | ○ | ○ | ○ | ○ | ○ | ○ | ○ | ○ |
| ED-M20 - Absorb - Self-Regulating: Extent to which the national leadership had the authority to make sectoral changes in a timely manner through flexible infrastructure. | ○ | ○ | ○ | ○ | ○ | ○ | ○ | ○ | ○ | ○ | ○ |
| ED-M21 - Recover - Extent to which the ED system is engaging with team and knowledge sharing to enhance systematic recovery. | ○ | ○ | ○ | ○ | ○ | ○ | ○ | ○ | ○ | ○ | ○ |
| ED-M22 - Recover - Diversity: The level of engagement of a multidisciplinary team to mitigate the effect of COVID-19 on ED. | ○ | ○ | ○ | ○ | ○ | ○ | ○ | ○ | ○ | ○ | ○ |
| ED-M23 - Adapt - Extent to which there are additions or changes being made to ED institutions, policies, trainings, programs, and culture. | ○ | ○ | ○ | ○ | ○ | ○ | ○ | ○ | ○ | ○ | ○ |
| ED-M24 - Adapt - Leadership and Management: Level of leadership demonstrated by the ED leaders to mitigate the effect of COVID-19 on ED. | ○ | ○ | ○ | ○ | ○ | ○ | ○ | ○ | ○ | ○ | ○ |

* ED M-25 Timeline: Extent of timeliness of the Government reaction - communication and implementation of measures - to mitigate the immediate and longterm impact of COVID-19.
*Note: This refers to the entirety of the national reaction - not the reaction that is specific to your sector.*

| | 1 | 2 | 3 | 4 | 5 | 6 | 7 | 8 | 9 | 10 | Don't know |
|---|---|---|---|---|---|---|---|---|---|---|---|
| 1-Lowest -> Highest-10 | ○ | ○ | ○ | ○ | ○ | ○ | ○ | ○ | ○ | ○ | ○ |

79

ED M-26 Comments: Please use this section to provide any additional comments about the resilience and sustainability of the national education systems based on your experience during the COVID-19 pandemics. If you marked “I don’t know” for any of the questions, also use this section to explain why you gave that rating.

JOHNS HOPKINS
BLOOMBERG SCHOOL
of PUBLIC HEALTH

Bill & Melinda Gates Institute for Population and Reproductive Health

## Demographic Dividend Effort Index Survey

### Women's Empowerment (WE) Questionnaire

**Please respond to the following questions based on your experience/expertise within this specific sector.**

**To give a summary picture of the national demographic dividend effort, please rate the following items. Score each item from 1 to 10, where 1 represents the lowest score (very weak or no effort) and 10 represents the highest score (strong effort). Where applicable, if a policy or activity is non-existent then mark your response as 0.**

Answer each item. All responses will be recorded in the format exemplified below:

| Lowest Effort | 1 | 2 | 3 | 4 | 5 | 6 | 7 | 8 | 9 | 10 | Highest Effort | I do not know |
|---|---|---|---|---|---|---|---|---|---|---|---|---|
| | | | | | | | | | | | | |

JOHNS HOPKINS
BLOOMBERG SCHOOL of PUBLIC HEALTH

Bill & Melinda Gates Institute for Population and Reproductive Health

## Demographic Dividend Effort Index Survey

### Dimension 1. Policy/Policy making

* WE 1.1 Gender Equality Policy: Extent to which gender equality is considered/integrated in country development plans (or within plans in your sector).

| | 1 | 2 | 3 | 4 | 5 | 6 | 7 | 8 | 9 | 10 | Don't know |
|---|---|---|---|---|---|---|---|---|---|---|---|
| 1-Lowest -> Highest-10 | ○ | ○ | ○ | ○ | ○ | ○ | ○ | ○ | ○ | ○ | ○ |

* WE 1.2 Level of commitment to the Convention on the Elimination of all Forms of Discrimination Against Women (CEDAW).

| | 1 | 2 | 3 | 4 | 5 | 6 | 7 | 8 | 9 | 10 | Don't know |
|---|---|---|---|---|---|---|---|---|---|---|---|
| 1-Lowest -> Highest-10 | ○ | ○ | ○ | ○ | ○ | ○ | ○ | ○ | ○ | ○ | ○ |

* WE 1.3 Equity in Marriage and Family Policies: Extent to which the principle of equality between men and women is incorporated in national constitution and other legislation relating to marriage and family relations.

| | 1 | 2 | 3 | 4 | 5 | 6 | 7 | 8 | 9 | 10 | Don't know |
|---|---|---|---|---|---|---|---|---|---|---|---|
| 1-Lowest -> Highest-10 | ○ | ○ | ○ | ○ | ○ | ○ | ○ | ○ | ○ | ○ | ○ |

* WE 1.4 Policy on Age at Marriage: Extent to which legal age at marriage for females is set at 18 years or higher and is enforced.

| | 1 | 2 | 3 | 4 | 5 | 6 | 7 | 8 | 9 | 10 | Don't know |
|---|---|---|---|---|---|---|---|---|---|---|---|
| 1-Lowest -> Highest-10 | ○ | ○ | ○ | ○ | ○ | ○ | ○ | ○ | ○ | ○ | ○ |

* WE 1.5 Violence Against Women Measures: Level of regulations and measures on violence against women, including domestic violence, rape, sexual harassment and trafficking of girls and women.

| | 1 | 2 | 3 | 4 | 5 | 6 | 7 | 8 | 9 | 10 | Don't know |
|---|---|---|---|---|---|---|---|---|---|---|---|
| 1-Lowest -> Highest-10 | ○ | ○ | ○ | ○ | ○ | ○ | ○ | ○ | ○ | ○ | ○ |

* WE 1.6 Female Genital Cutting/Mutilation: Level of regulation and measures on female genital cutting/mutilation.

| | 1 | 2 | 3 | 4 | 5 | 6 | 7 | 8 | 9 | 10 | Don't know |
|---|---|---|---|---|---|---|---|---|---|---|---|
| 1-Lowest -> Highest-10 | ○ | ○ | ○ | ○ | ○ | ○ | ○ | ○ | ○ | ○ | ○ |

* WE 1.7 Women's Health Measures: Level of protocols for the protection of women's health, including sexually transmitted infections including HIV/AIDS, maternal mortality and contraception.

| | 1 | 2 | 3 | 4 | 5 | 6 | 7 | 8 | 9 | 10 | Don't know |
|---|---|---|---|---|---|---|---|---|---|---|---|
| 1-Lowest -> Highest-10 | ○ | ○ | ○ | ○ | ○ | ○ | ○ | ○ | ○ | ○ | ○ |

* WE 1.8 Education Measures: Extent to which there are mechanisms to promote girls' education.

| | 1 | 2 | 3 | 4 | 5 | 6 | 7 | 8 | 9 | 10 | Don't know |
|---|---|---|---|---|---|---|---|---|---|---|---|
| WE 1.8.1 Level of enforcement of policies on girl's education. | ○ | ○ | ○ | ○ | ○ | ○ | ○ | ○ | ○ | ○ | ○ |
| WE 1.8.2 Extent to which there are mechanisms in place to prevent girls' school dropouts. | ○ | ○ | ○ | ○ | ○ | ○ | ○ | ○ | ○ | ○ | ○ |

* WE 1.9 Rights Measures: Level of enforcement of policies on women's rights.

| | 1 | 2 | 3 | 4 | 5 | 6 | 7 | 8 | 9 | 10 | Don't know |
|---|---|---|---|---|---|---|---|---|---|---|---|
| 1-Lowest -> Highest-10 | ○ | ○ | ○ | ○ | ○ | ○ | ○ | ○ | ○ | ○ | ○ |

* WE 1.10 Equitable Work: Extent to which there are policies on equitable working conditions and remunerations between men and women.

| | 1 | 2 | 3 | 4 | 5 | 6 | 7 | 8 | 9 | 10 | Don't know |
|---|---|---|---|---|---|---|---|---|---|---|---|
| 1-Lowest -> Highest-10 | ○ | ○ | ○ | ○ | ○ | ○ | ○ | ○ | ○ | ○ | ○ |

* WE 1.11 Equitable Land/Asset Ownership: Extent to which there are policies to affirm equitable access to and ownership of land.

| | 1 | 2 | 3 | 4 | 5 | 6 | 7 | 8 | 9 | 10 | Don't know |
|---|---|---|---|---|---|---|---|---|---|---|---|
| 1-Lowest -> Highest-10 | ○ | ○ | ○ | ○ | ○ | ○ | ○ | ○ | ○ | ○ | ○ |

* WE 1.12 Conflict, Women & Peacebuilding: Level of implementation of Security UN Resolution 1325 on the impacts of conflict on women and their role in peacebuilding.

| | 1 | 2 | 3 | 4 | 5 | 6 | 7 | 8 | 9 | 10 | Don't know |
|---|---|---|---|---|---|---|---|---|---|---|---|
| 1-Lowest -> Highest-10 | ○ | ○ | ○ | ○ | ○ | ○ | ○ | ○ | ○ | ○ | ○ |

* WE 1.13 Gender Quotas in Politics: Existence, and level of implementation, of quotas for gender-balanced political systems.

| | 1 | 2 | 3 | 4 | 5 | 6 | 7 | 8 | 9 | 10 | Don't know |
|---|---|---|---|---|---|---|---|---|---|---|---|
| WE 1.13.1 Extent to which there is gender equitable decision-making, including support for electoral quotas and gender mainstreaming in all government ministries and departments. | ○ | ○ | ○ | ○ | ○ | ○ | ○ | ○ | ○ | ○ | ○ |
| WE 1.13.2 Extent to which women participate and/or have a significant role in traditional governance systems (e.g. chiefs and village head). | ○ | ○ | ○ | ○ | ○ | ○ | ○ | ○ | ○ | ○ | ○ |

* WE 1.14 Sustainable Development Goal 5: Level of commitment to fulfilling Sustainable Development Goal 5, to achieve gender equality and empower all women and girls.

| | 1 | 2 | 3 | 4 | 5 | 6 | 7 | 8 | 9 | 10 | Don't know |
|---|---|---|---|---|---|---|---|---|---|---|---|
| 1-Lowest -> Highest-10 | ○ | ○ | ○ | ○ | ○ | ○ | ○ | ○ | ○ | ○ | ○ |

* WE 1.15 Customary Law Revision: Extent to which customary laws have been reviewed and modified to align with national policies/frameworks. Or to which existing customary laws reflect efforts towards women’s empowerment.

| | 1 | 2 | 3 | 4 | 5 | 6 | 7 | 8 | 9 | 10 | Don't know |
|---|---|---|---|---|---|---|---|---|---|---|---|
| 1-Lowest -> Highest-10 | ○ | ○ | ○ | ○ | ○ | ○ | ○ | ○ | ○ | ○ | ○ |

* WE 1.16 Costing for WE: Extent to which there is sufficient planning and allocating to cover the costs needed to meet targets set in a national gender plan or equivalent national plan.

| | 1 | 2 | 3 | 4 | 5 | 6 | 7 | 8 | 9 | 10 | Don't know |
|---|---|---|---|---|---|---|---|---|---|---|---|
| 1-Lowest -> Highest-10 | ○ | ○ | ○ | ○ | ○ | ○ | ○ | ○ | ○ | ○ | ○ |

* WE 1.17 Staffing for WE: Extent to which:

| | 1 | 2 | 3 | 4 | 5 | 6 | 7 | 8 | 9 | 10 | Don't know |
|---|---|---|---|---|---|---|---|---|---|---|---|
| WE 1.17.1 Sufficient and qualified staff are employed to implement gender-related activities, including efforts towards training said staff | ◯ | ◯ | ◯ | ◯ | ◯ | ◯ | ◯ | ◯ | ◯ | ◯ | ◯ |
| WE 1.17.2 Efforts are made to appropriately and comprehensively train staff | ◯ | ◯ | ◯ | ◯ | ◯ | ◯ | ◯ | ◯ | ◯ | ◯ | ◯ |
| WE 1.17.3 Staff have sufficient support to carry out their tasks | ◯ | ◯ | ◯ | ◯ | ◯ | ◯ | ◯ | ◯ | ◯ | ◯ | ◯ |
| WE 1.17.4 Assigned staff are located at a sufficiently high level in the administration to work effectively | ◯ | ◯ | ◯ | ◯ | ◯ | ◯ | ◯ | ◯ | ◯ | ◯ | ◯ |

* WE 1.18 Transparency and Accountability: Extent to which the government provides stakeholders with full information on the intentions, plans, programs and budgets available to implement programs in order to ensure accountability and transparency regarding women's empowerment programs. (*Consider measure such as the passage and implementation of a right to information law, regular information sessions for instance in town halls or the provision of such information in leaflets or other material in the local language*)

| | 1 | 2 | 3 | 4 | 5 | 6 | 7 | 8 | 9 | 10 | Don't know |
|---|---|---|---|---|---|---|---|---|---|---|---|
| 1-Lowest -> Highest-10 | ◯ | ◯ | ◯ | ◯ | ◯ | ◯ | ◯ | ◯ | ◯ | ◯ | ◯ |

WE 1.19 Comments: Please use this section to provide any additional comments about the level of effort, challenges, or successes in the country around policymaking for WE. If you marked “I don’t know” for any of the questions, also use this section to explain why you gave that rating.

85

JOHNS HOPKINS
BLOOMBERG SCHOOL of PUBLIC HEALTH

Bill & Melinda Gates Institute for Population and Reproductive Health

## Demographic Dividend Effort Index Survey

### Dimension 2. Services or Programs

* WE 2.1 Gender Mainstreaming: Extent to which services or programs within and across sectors consider implications for women and men of any planned action.

| | 1 | 2 | 3 | 4 | 5 | 6 | 7 | 8 | 9 | 10 | Don't know |
|---|---|---|---|---|---|---|---|---|---|---|---|
| 1-Lowest -> Highest-10 | ◯ | ◯ | ◯ | ◯ | ◯ | ◯ | ◯ | ◯ | ◯ | ◯ | ◯ |

* WE 2.2 Gender-Transformative Programming: Extent to which services or programs within and across sectors integrate gender-transformative principles/approaches.

| | 1 | 2 | 3 | 4 | 5 | 6 | 7 | 8 | 9 | 10 | Don't know |
|---|---|---|---|---|---|---|---|---|---|---|---|
| 1-Lowest -> Highest-10 | ◯ | ◯ | ◯ | ◯ | ◯ | ◯ | ◯ | ◯ | ◯ | ◯ | ◯ |

* WE 2.3 Education: Extent to which education services or programs focus on girl's and women's empowerment within the classroom.

| | 1 | 2 | 3 | 4 | 5 | 6 | 7 | 8 | 9 | 10 | Don't know |
|---|---|---|---|---|---|---|---|---|---|---|---|
| 1-Lowest -> Highest-10 | ◯ | ◯ | ◯ | ◯ | ◯ | ◯ | ◯ | ◯ | ◯ | ◯ | ◯ |

* WE 2.4. Dropouts: Extent to which there are programs to prevent female dropouts.

| | 1 | 2 | 3 | 4 | 5 | 6 | 7 | 8 | 9 | 10 | Don't know |
|---|---|---|---|---|---|---|---|---|---|---|---|
| 1-Lowest -> Highest-10 | ◯ | ◯ | ◯ | ◯ | ◯ | ◯ | ◯ | ◯ | ◯ | ◯ | ◯ |

* WE 2.5 Technology Literacy: Extent to which technology literacy is promoted amongst girls in particular.

| | 1 | 2 | 3 | 4 | 5 | 6 | 7 | 8 | 9 | 10 | Don't know |
|---|---|---|---|---|---|---|---|---|---|---|---|
| 1-Lowest -> Highest-10 | ◯ | ◯ | ◯ | ◯ | ◯ | ◯ | ◯ | ◯ | ◯ | ◯ | ◯ |



Rusatira JC, *et al. BMJ Open* 2023; 13:e059937. doi: 10.1136/bmjopen-2021-059937

* WE 2.6 Employment: Extent to which job-creation programs and labor market polices aim to empower women economically and is designed/implemented accordingly.

| | 1 | 2 | 3 | 4 | 5 | 6 | 7 | 8 | 9 | 10 | Don't know |
|---|---|---|---|---|---|---|---|---|---|---|---|
| 1-Lowest -> Highest-10 | ○ | ○ | ○ | ○ | ○ | ○ | ○ | ○ | ○ | ○ | ○ |

* WE 2.7 Women as Mothers and Workers: Extent to which the government makes possible, through laws, regulations and programs, for women to combine the roles of childbearing, breast-feeding and child-rearing with participation in the workforce.

| | 1 | 2 | 3 | 4 | 5 | 6 | 7 | 8 | 9 | 10 | Don't know |
|---|---|---|---|---|---|---|---|---|---|---|---|
| 1-Lowest -> Highest-10 | ○ | ○ | ○ | ○ | ○ | ○ | ○ | ○ | ○ | ○ | ○ |

* WE 2.8 Family Planning for Empowerment: Extent to which FP services or programs are implemented with the fundamental approach towards empowering women.

| | 1 | 2 | 3 | 4 | 5 | 6 | 7 | 8 | 9 | 10 | Don't know |
|---|---|---|---|---|---|---|---|---|---|---|---|
| 1-Lowest -> Highest-10 | ○ | ○ | ○ | ○ | ○ | ○ | ○ | ○ | ○ | ○ | ○ |

* WE 2.9 Political and Public Representation: Extent of implementation of programs aiming at improving women's equal participation and equitable representation at all levels of the political process and public life.

| | 1 | 2 | 3 | 4 | 5 | 6 | 7 | 8 | 9 | 10 | Don't know |
|---|---|---|---|---|---|---|---|---|---|---|---|
| 1-Lowest -> Highest-10 | ○ | ○ | ○ | ○ | ○ | ○ | ○ | ○ | ○ | ○ | ○ |

* WE 2.10 Violence: Extent of fight against violence against women and children and its elimination nationwide (domestic violence, rape, sexual harassment, trafficking women).

| | 1 | 2 | 3 | 4 | 5 | 6 | 7 | 8 | 9 | 10 | Don't know |
|---|---|---|---|---|---|---|---|---|---|---|---|
| 1-Lowest -> Highest-10 | ○ | ○ | ○ | ○ | ○ | ○ | ○ | ○ | ○ | ○ | ○ |

* WE 2.11 Child Marriage: Extent of law enforcement to eliminate child marriage.

| | 1 | 2 | 3 | 4 | 5 | 6 | 7 | 8 | 9 | 10 | Don't know |
|---|---|---|---|---|---|---|---|---|---|---|---|
| 1-Lowest -> Highest-10 | ○ | ○ | ○ | ○ | ○ | ○ | ○ | ○ | ○ | ○ | ○ |

* WE 2.12 Female Genital Mutilation/Cutting: Extent of law enforcement to eliminate female genital mutilation/cutting.

| | 1 | 2 | 3 | 4 | 5 | 6 | 7 | 8 | 9 | 10 | Don't know |
|---|---|---|---|---|---|---|---|---|---|---|---|
| 1-Lowest -> Highest-10 | ○ | ○ | ○ | ○ | ○ | ○ | ○ | ○ | ○ | ○ | ○ |

WE 2.13 Comments: Please use this section to provide any additional comments about the level of effort, challenges, or successes in the country around services or programs for WE. If you marked “I don’t know” for any of the questions, also use this section to explain why you gave that rating

JOHNS HOPKINS
BLOOMBERG SCHOOL
of PUBLIC HEALTH

Bill & Melinda Gates Institute for Population and Reproductive Health

## Demographic Dividend Effort Index Survey

### Dimension 3. Advocacy

* WE 3.1 Advocates: Extent to which advocates for girls' and women's empowerment exists at the national and sub-national levels.

| | 1 | 2 | 3 | 4 | 5 | 6 | 7 | 8 | 9 | 10 | Don't know |
|---|---|---|---|---|---|---|---|---|---|---|---|
| 1-Lowest -> Highest-10 | ○ | ○ | ○ | ○ | ○ | ○ | ○ | ○ | ○ | ○ | ○ |

* WE 3.2. Girls and Women: Extent to which the advocacy efforts in this area encompass the breadth of concerns in girls' and women's empowerment.

| | 1 | 2 | 3 | 4 | 5 | 6 | 7 | 8 | 9 | 10 | Don't know |
|---|---|---|---|---|---|---|---|---|---|---|---|
| 1-Lowest -> Highest-10 | ○ | ○ | ○ | ○ | ○ | ○ | ○ | ○ | ○ | ○ | ○ |

* WE 3.3 Information Dissemination: Extent to which the government undertakes sufficient efforts to ensure that the population is aware of issues related to WE, and whether the information that is spread is correct and will reach people in a language they understand.

| | 1 | 2 | 3 | 4 | 5 | 6 | 7 | 8 | 9 | 10 | Don't know |
|---|---|---|---|---|---|---|---|---|---|---|---|
| 1-Lowest -> Highest-10 | ○ | ○ | ○ | ○ | ○ | ○ | ○ | ○ | ○ | ○ | ○ |

* WE 3.4 Leveraging Partnerships: Extent to which the government leverages partnerships with CSOs to disseminate WE information through campaigns.

| | 1 | 2 | 3 | 4 | 5 | 6 | 7 | 8 | 9 | 10 | Don't know |
|---|---|---|---|---|---|---|---|---|---|---|---|
| 1-Lowest -> Highest-10 | ○ | ○ | ○ | ○ | ○ | ○ | ○ | ○ | ○ | ○ | ○ |

* WE 3.5 Employment: Extent to which stakeholders call for laws, policies and programs for women to combine the roles of childbearing, breast-feeding and child-rearing with participation in the workforce.

| | 1 | 2 | 3 | 4 | 5 | 6 | 7 | 8 | 9 | 10 | Don't know |
|---|---|---|---|---|---|---|---|---|---|---|---|
| 1-Lowest -> Highest-10 | ◯ | ◯ | ◯ | ◯ | ◯ | ◯ | ◯ | ◯ | ◯ | ◯ | ◯ |

* WE 3.6 Education: Extent to which a priority of advocates is to promote the fulfillment of girls’ and women’s potential through education and skills development.

| | 1 | 2 | 3 | 4 | 5 | 6 | 7 | 8 | 9 | 10 | Don't know |
|---|---|---|---|---|---|---|---|---|---|---|---|
| 1-Lowest -> Highest-10 | ◯ | ◯ | ◯ | ◯ | ◯ | ◯ | ◯ | ◯ | ◯ | ◯ | ◯ |

* WE 3.7 Violence: Extent to which advocates successfully engage with the government to react to and address cases of violence or discrimination against women.

| | 1 | 2 | 3 | 4 | 5 | 6 | 7 | 8 | 9 | 10 | Don't know |
|---|---|---|---|---|---|---|---|---|---|---|---|
| 1-Lowest -> Highest-10 | ◯ | ◯ | ◯ | ◯ | ◯ | ◯ | ◯ | ◯ | ◯ | ◯ | ◯ |

WE 3.8 Comments: Please use this section to provide any additional comments about the level of effort, challenges, or successes in the country around advocacy for WE. If you marked “I don’t know” for any of the questions, also use this section to explain why you gave that rating

JOHNS HOPKINS BLOOMBERG SCHOOL of PUBLIC HEALTH

Bill & Melinda Gates Institute for Population and Reproductive Health

## Demographic Dividend Effort Index Survey

### Dimension 4. Research

* WE 4.1 Strength of Observatory/Stakeholder/Technical Working Group for WE: Extent to which country-level WE stakeholders (e.g., stakeholder leadership group, technical working group, WE observatory, country coordination and facilitation group):

| | 1 | 2 | 3 | 4 | 5 | 6 | 7 | 8 | 9 | 10 | Don't know |
|---|---|---|---|---|---|---|---|---|---|---|---|
| WE 4.1.1 Have representation in government, training institutions, civil society, nongovernmental and faith-based organizations, professional associations, the private sector, etc. | ○ | ○ | ○ | ○ | ○ | ○ | ○ | ○ | ○ | ○ | ○ |
| WE 4.1.2 Meet regularly, report and recommend policy for senior management within the appropriate ministries | ○ | ○ | ○ | ○ | ○ | ○ | ○ | ○ | ○ | ○ | ○ |
| WE 4.1.3 Make an impact on WE within the country | ○ | ○ | ○ | ○ | ○ | ○ | ○ | ○ | ○ | ○ | ○ |

* WE 4.2 Women’s Rights Research Strategy: Extent to which the national WE plan and/or other national documents include a comprehensive strategy/approach on women’s empowerment research, shared among partners.

| | 1 | 2 | 3 | 4 | 5 | 6 | 7 | 8 | 9 | 10 | Don't know |
|---|---|---|---|---|---|---|---|---|---|---|---|
| 1-Lowest -> Highest-10 | ○ | ○ | ○ | ○ | ○ | ○ | ○ | ○ | ○ | ○ | ○ |

* WE 4.3 Data Gathering Partners:

| | 1 | 2 | 3 | 4 | 5 | 6 | 7 | 8 | 9 | 10 | Don't know |
|---|---|---|---|---|---|---|---|---|---|---|---|
| WE 4.3.1 Extent to which research is undertaken by government agencies. (This may include statistical agencies/bureaus and/or ministries of economics/finances, education, health, gender, etc.) | ○ | ○ | ○ | ○ | ○ | ○ | ○ | ○ | ○ | ○ | ○ |
| WE 4.3.2 Extent to which women empowerment research is undertaken by research institutions. | ○ | ○ | ○ | ○ | ○ | ○ | ○ | ○ | ○ | ○ | ○ |
| WE 4.3.3 Extent to which women empowerment research is undertaken by independent researchers. | ○ | ○ | ○ | ○ | ○ | ○ | ○ | ○ | ○ | ○ | ○ |

* WE 4.4 Topical Data Gathering:

| | 1 | 2 | 3 | 4 | 5 | 6 | 7 | 8 | 9 | 10 | Don't know |
|---|---|---|---|---|---|---|---|---|---|---|---|
| WE 4.4.1 Extent to which social and cultural norms affecting women empowerment and gender equality are researched. | ○ | ○ | ○ | ○ | ○ | ○ | ○ | ○ | ○ | ○ | ○ |
| WE 4.4.2 Extent to which health and economic effects of women empowerment are researched. | ○ | ○ | ○ | ○ | ○ | ○ | ○ | ○ | ○ | ○ | ○ |
| WE 4.4.3 Extent to which child marriage is researched to inform policy. | ○ | ○ | ○ | ○ | ○ | ○ | ○ | ○ | ○ | ○ | ○ |
| WE 4.4.4 Extent to which girls' education and skills acquisition outcomes is researched. | ○ | ○ | ○ | ○ | ○ | ○ | ○ | ○ | ○ | ○ | ○ |



Rusatira JC, *et al. BMJ Open* 2023; 13:e059937. doi: 10.1136/bmjopen-2021-059937

* WE 4.5 Quality/Coverage of Data:

| | 1 | 2 | 3 | 4 | 5 | 6 | 7 | 8 | 9 | 10 | Don't know |
|---|---|---|---|---|---|---|---|---|---|---|---|
| WE 4.5.1 Extent to which a routine statistical system provides reliable periodic age-disaggregated data. | ○ | ○ | ○ | ○ | ○ | ○ | ○ | ○ | ○ | ○ | ○ |
| WE 4.5.2 Extent to which the routine statistical system provides good reliable periodic age-disaggregated data on women's empowerment needs among populations in different communities. | ○ | ○ | ○ | ○ | ○ | ○ | ○ | ○ | ○ | ○ | ○ |
| WE 4.5.3 Extent to which evaluations of the monitoring and surveillance systems are conducted and applied to ensure the reliability of age-disaggregated data. | ○ | ○ | ○ | ○ | ○ | ○ | ○ | ○ | ○ | ○ | ○ |

* WE 4.6 Quality Research Institutions: Extent to which the country supports and sustains research institutions that develop research/collect data that support the breadth and quality of information-gathering to inform national WE efforts.

| | 1 | 2 | 3 | 4 | 5 | 6 | 7 | 8 | 9 | 10 | Don't know |
|---|---|---|---|---|---|---|---|---|---|---|---|
| 1-Lowest -> Highest-10 | ○ | ○ | ○ | ○ | ○ | ○ | ○ | ○ | ○ | ○ | ○ |

* WE 4.7 Evaluation: Extent to which program statistics, surveys, and small studies are used by specialized staff to report on program operations and measure progress on WE and gender equality.

| | 1 | 2 | 3 | 4 | 5 | 6 | 7 | 8 | 9 | 10 | Don't know |
|---|---|---|---|---|---|---|---|---|---|---|---|
| 1-Lowest -> Highest-10 | ○ | ○ | ○ | ○ | ○ | ○ | ○ | ○ | ○ | ○ | ○ |

* WE 4.8 Management's Use of Evaluation Findings: Extent to which local-level program managers use research and evaluation findings to improve WE programs in ways suggested by findings.

| | 1 | 2 | 3 | 4 | 5 | 6 | 7 | 8 | 9 | 10 | Don't know |
|---|---|---|---|---|---|---|---|---|---|---|---|
| 1-Lowest -> Highest-10 | ○ | ○ | ○ | ○ | ○ | ○ | ○ | ○ | ○ | ○ | ○ |

* WE 4.9 Ministerial Use of Evaluation Findings: Extent to which the Ministry administrators systematically utilize data to inform policies and interventions to address women's empowerment-related issues.

| | 1 | 2 | 3 | 4 | 5 | 6 | 7 | 8 | 9 | 10 | Don't know |
|---|---|---|---|---|---|---|---|---|---|---|---|
| 1-Lowest -> Highest-10 | ○ | ○ | ○ | ○ | ○ | ○ | ○ | ○ | ○ | ○ | ○ |

* WE 4.10 Dissemination of Information to other Implementing Bodies: Extent to which information is shared or spread across geographical areas and throughout various levels (national, state/province, localities).

| | 1 | 2 | 3 | 4 | 5 | 6 | 7 | 8 | 9 | 10 | Don't know |
|---|---|---|---|---|---|---|---|---|---|---|---|
| 1-Lowest -> Highest-10 | ○ | ○ | ○ | ○ | ○ | ○ | ○ | ○ | ○ | ○ | ○ |

WE 4.11 Comments: Please use this section to provide any additional comments about the level of effort, challenges, or successes in the country around WE research. If you marked "I don't know" for any of the questions, also use this section to explain why you gave that rating

JOHNS HOPKINS
BLOOMBERG SCHOOL of PUBLIC HEALTH

Bill & Melinda Gates Institute for Population and Reproductive Health

## Demographic Dividend Effort Index Survey

### Dimension 5. Civil Society Organizations (CSOs)

* WE 5.1 CSO Actor Power: Extent to which WE civil society actors are in strong position to influence national policy.

| | 1 | 2 | 3 | 4 | 5 | 6 | 7 | 8 | 9 | 10 | Don't know |
|---|---|---|---|---|---|---|---|---|---|---|---|
| 1-Lowest -> Highest-10 | ◯ | ◯ | ◯ | ◯ | ◯ | ◯ | ◯ | ◯ | ◯ | ◯ | ◯ |

* WE 5.2 Budget Analysis as a CSO Tool: Extent to which CSOs utilize budget analysis as a tool to build WE advocacy cases.

| | 1 | 2 | 3 | 4 | 5 | 6 | 7 | 8 | 9 | 10 | Don't know |
|---|---|---|---|---|---|---|---|---|---|---|---|
| 1-Lowest -> Highest-10 | ◯ | ◯ | ◯ | ◯ | ◯ | ◯ | ◯ | ◯ | ◯ | ◯ | ◯ |

* WE 5.3 Community-Based Service Delivery Support: Extent to which CSOs participate in bringing women’s empowerment information and services to the communities where people live through activities such as supporting the capacity-building of girls, women and women’s empowerment champions and/or undertaking information and education campaigns to increase the commitment to women’s empowerment in families and communities.

| | 1 | 2 | 3 | 4 | 5 | 6 | 7 | 8 | 9 | 10 | Don't know |
|---|---|---|---|---|---|---|---|---|---|---|---|
| 1-Lowest -> Highest-10 | ◯ | ◯ | ◯ | ◯ | ◯ | ◯ | ◯ | ◯ | ◯ | ◯ | ◯ |

* WE 5.4 Human Rights and Quality of Implementation: Extent to which CSOs advocate for and support rights-based approaches to women’s empowerment to ensure policy and program beneficiaries experience progress in women’s empowerment and autonomy on their terms.

| | 1 | 2 | 3 | 4 | 5 | 6 | 7 | 8 | 9 | 10 | Don't know |
|---|---|---|---|---|---|---|---|---|---|---|---|
| 1-Lowest -> Highest-10 | ◯ | ◯ | ◯ | ◯ | ◯ | ◯ | ◯ | ◯ | ◯ | ◯ | ◯ |

* WE 5.5 Social and Behavior Change (SBC): Extent to which CSOs support SBC interventions relating, but not limited, to gender-transformative programming, life skills and peer education, adult health education, school-based education, couple's communication, community mobilization, working through gatekeepers, and/or mass media interventions for a general public audience. These may be supported through implementation at the community or individual levels.

| | 1 | 2 | 3 | 4 | 5 | 6 | 7 | 8 | 9 | 10 | Don't know |
|---|---|---|---|---|---|---|---|---|---|---|---|
| 1-Lowest -> Highest-10 | ○ | ○ | ○ | ○ | ○ | ○ | ○ | ○ | ○ | ○ | ○ |

* WE 5.6 Youth: Extent to which CSOs promote gender empowerment through youth programs.

| | 1 | 2 | 3 | 4 | 5 | 6 | 7 | 8 | 9 | 10 | Don't know |
|---|---|---|---|---|---|---|---|---|---|---|---|
| 1-Lowest -> Highest-10 | ○ | ○ | ○ | ○ | ○ | ○ | ○ | ○ | ○ | ○ | ○ |

* WE 5.7 Programming for Men: Extent to which CSOs support WE policies, interventions and programs targeting men.

| | 1 | 2 | 3 | 4 | 5 | 6 | 7 | 8 | 9 | 10 | Don't know |
|---|---|---|---|---|---|---|---|---|---|---|---|
| 1-Lowest -> Highest-10 | ○ | ○ | ○ | ○ | ○ | ○ | ○ | ○ | ○ | ○ | ○ |

* WE 5.8 Advocacy/Accountability: Extent to which CSOs support the training and capacity building of advocates and community members to understand policies, processes, and activities to increase community-led WE issues identification, collaborative problem solving and more targeted advocacy actions.

| | 1 | 2 | 3 | 4 | 5 | 6 | 7 | 8 | 9 | 10 | Don't know |
|---|---|---|---|---|---|---|---|---|---|---|---|
| 1-Lowest -> Highest-10 | ○ | ○ | ○ | ○ | ○ | ○ | ○ | ○ | ○ | ○ | ○ |

* WE 5.9 CSO-Led Assessment and Monitoring: Extent to which CSOs assess, monitor and report on the effectiveness of policies and programming to improve accountability at the policy and provider levels.

| | 1 | 2 | 3 | 4 | 5 | 6 | 7 | 8 | 9 | 10 | Don't know |
|---|---|---|---|---|---|---|---|---|---|---|---|
| 1-Lowest -> Highest-10 | ○ | ○ | ○ | ○ | ○ | ○ | ○ | ○ | ○ | ○ | ○ |

* WE 5.10 Forming Partnerships between CSOs: Extent to which CSOs have formed national alliances and regional partnerships to strengthen their positioning and potentially improve leadership strength and funding mechanisms to advance the women empowerment agenda.

| | 1 | 2 | 3 | 4 | 5 | 6 | 7 | 8 | 9 | 10 | Don't know |
|---|---|---|---|---|---|---|---|---|---|---|---|
| 1-Lowest -> Highest-10 | ○ | ○ | ○ | ○ | ○ | ○ | ○ | ○ | ○ | ○ | ○ |

WE 5.11 Comments: Please use this section to provide any additional comments about the level of effort, challenges, or successes around the role of CSOs in WE. If you marked “I don’t know” for any of the questions, also use this section to explain why you gave that rating

JOHNS HOPKINS
BLOOMBERG SCHOOL
of PUBLIC HEALTH

Bill & Melinda Gates Institute for Population and Reproductive Health

# Demographic Dividend Effort Index Survey

## Module: Sectoral Resilience and Sustainability

**Considering the health and socio-economic impact of the COVID-19 and the likely impact on the DD progress, this DD effort index has integrated questions to assess the resilience and sustainability of systems in key sectors of the DD framework. The items included in the COVID-19 related questionnaire below follow the key dimensions of resilient systems and provide critical information on the potential of an effective response to emerging infectious disease threats or other public health emergencies.**

**Please score each item from 1 to 10, where 1 represents the lowest score (poor state/capability) and 10 represents the highest score (great state/capability).**

* Physical: facilities, equipment, system states, and capabilities.

| | 1 | 2 | 3 | 4 | 5 | 6 | 7 | 8 | 9 | 10 | Don't know |
|---|---|---|---|---|---|---|---|---|---|---|---|
| WE-M1 - Plan/Prepare - State and capability of equipment, personnel and WE sector structure prior to the crisis event. (1 = poor state/capability; 10 = great state/capability) | ○ | ○ | ○ | ○ | ○ | ○ | ○ | ○ | ○ | ○ | ○ |
| WE-M2 - Absorb - Extent to which COVID-19 was recognized within the WE sector and the performance of the system was able to maintain functionality. | ○ | ○ | ○ | ○ | ○ | ○ | ○ | ○ | ○ | ○ | ○ |
| WE-M3 - Absorb - Integration: The level of integration of WE services and programs to mitigate the effect of COVID-19 on WE. | ○ | ○ | ○ | ○ | ○ | ○ | ○ | ○ | ○ | ○ | ○ |
| WE-M4 - Recover - Extent to which the WE programs have been able to induce change to recover the pre-COVID-19 level of functionality. | ○ | ○ | ○ | ○ | ○ | ○ | ○ | ○ | ○ | ○ | ○ |
| WE-M5 - Recover - Financing and Donor Management: Level of resources mobilization and allocation to mitigate the effect of COVID-19 on WE | ○ | ○ | ○ | ○ | ○ | ○ | ○ | ○ | ○ | ○ | ○ |
| WE-M6 - Adapt - Extent to which changes have been made to improve resilience in the WE sector. | ○ | ○ | ○ | ○ | ○ | ○ | ○ | ○ | ○ | ○ | ○ |

* Information: creation, manipulation, storage, and utilization of data.

| | 1 | 2 | 3 | 4 | 5 | 6 | 7 | 8 | 9 | 10 | Don't know |
|---|---|---|---|---|---|---|---|---|---|---|---|
| WE-M7 - Plan/Prepare - Extent to which WE-related data was sufficiently prepared, presented, analyzed and stored prior to the crisis event. | ○ | ○ | ○ | ○ | ○ | ○ | ○ | ○ | ○ | ○ | ○ |
| WE-M8 - Absorb - Extent to which the WE sector has been able to conduct a real-time assessment of functionality, in anticipation of cascading losses in WE programs and services. | ○ | ○ | ○ | ○ | ○ | ○ | ○ | ○ | ○ | ○ | ○ |
| WE-M9 - Absorb - Awareness: Extent of the surveillance capacity to detect the WE limitations/threats posed by COVID-19. | ○ | ○ | ○ | ○ | ○ | ○ | ○ | ○ | ○ | ○ | ○ |
| WE-M10 - Recover - Extent to which WE data have been used to track the progress of recovery and to anticipate recovery scenarios. | ○ | ○ | ○ | ○ | ○ | ○ | ○ | ○ | ○ | ○ | ○ |
| WE-M11 - Adapt - Extent to which the WE sector is creating and improving WE data storage and real-time use protocols. | ○ | ○ | ○ | ○ | ○ | ○ | ○ | ○ | ○ | ○ | ○ |

* Cognitive: understanding, mental models, preconceptions, biases, and values.

| | 1 | 2 | 3 | 4 | 5 | 6 | 7 | 8 | 9 | 10 | Don't know |
|---|---|---|---|---|---|---|---|---|---|---|---|
| WE-M12 - Plan/Prepare - Extent to which the WE programs design and operation decisions were prepared in anticipation of crisis events. | ○ | ○ | ○ | ○ | ○ | ○ | ○ | ○ | ○ | ○ | ○ |
| WE-M13 - Absorb - Extent to which the WE sector is responding with sufficient contingency protocols and proactive crisis management. | ○ | ○ | ○ | ○ | ○ | ○ | ○ | ○ | ○ | ○ | ○ |

| | 1 | 2 | 3 | 4 | 5 | 6 | 7 | 8 | 9 | 10 | Don't know |
|---|---|---|---|---|---|---|---|---|---|---|---|
| WE-M14 - Absorb - Accountability: Level of effort to ensure optimal accountability for the resources allocated to support WE as part of the response to the COVID-19. (These may take the form of support for services attenuated to the needs of women and girls for protection). | ○ | ○ | ○ | ○ | ○ | ○ | ○ | ○ | ○ | ○ | ○ |
| WE-M15 - Recover - Extent to which decisions are oriented towards recovery, which employ evidence-based communication to community members to promote safe behavior. | ○ | ○ | ○ | ○ | ○ | ○ | ○ | ○ | ○ | ○ | ○ |
| WE-M16 - Recover - Awareness: Extent to which the WE sector is building confidence through communication regarding barriers posed by COVID-19 to support access to and uptake of WE services and programs. (This includes surveillance that is attenuated to the gendered disparities in COVID-19 responses and/or cases as well as increased surveillance of WE-related issues such as gender-based violence) | ○ | ○ | ○ | ○ | ○ | ○ | ○ | ○ | ○ | ○ | ○ |
| WE-M17 - Adapt - Extent to which the WE sector is designing new system configurations, objectives and decision criteria. | ○ | ○ | ○ | ○ | ○ | ○ | ○ | ○ | ○ | ○ | ○ |
| WE-M18 - Adapt - Adaptive: Level of adaptation to respond to the COVID-19 threats to WE. | ○ | ○ | ○ | ○ | ○ | ○ | ○ | ○ | ○ | ○ | ○ |

* Social: interaction, collaboration, and self-synchronization between individuals, entities and institutions.

| | 1 | 2 | 3 | 4 | 5 | 6 | 7 | 8 | 9 | 10 | Don't know |
|---|---|---|---|---|---|---|---|---|---|---|---|
| WE-M19 - Plan/Prepare - Extent to which training on outbreak/crisis management was conducted through, and which leveraged, social networks, social capital and institutional and cultural norms prior to the crisis. | | | | | | | | | | | |
| WE-M20 - Absorb - Extent to which personnel and social institutions have been resourceful and accessible for the outbreak/crisis response. | | | | | | | | | | | |
| WE-M21 - Absorb - Self-Regulating: Extent to which the national leadership had the authority to make sectoral changes in a timely manner through flexible infrastructure. | | | | | | | | | | | |
| WE-M22 - Recover - Extent to which the WE sector is engaging with team and knowledge sharing to enhance systematic recovery. | | | | | | | | | | | |
| WE-M23 - Recover - Diversity: The level of engagement of a multidisciplinary team to mitigate the effect of COVID-19 on WE. | | | | | | | | | | | |
| WE-M24 - Adapt - Leadership and Management: Level of leadership demonstrated by the leaders in the WE sector and programs to mitigate the effect of COVID-19 on WE. | | | | | | | | | | | |

* WE M-25 Timeline: Extent of timeliness of the Government reaction - communication and implementation of measures - to mitigate the immediate and longterm impact of COVID-19.
*Note: This refers to the entirety of the national reaction - not the reaction that is specific to your sector.*

| | 1 | 2 | 3 | 4 | 5 | 6 | 7 | 8 | 9 | 10 | Don't know |
|---|---|---|---|---|---|---|---|---|---|---|---|
| 1-Lowest -> Highest-10 | ○ | ○ | ○ | ○ | ○ | ○ | ○ | ○ | ○ | ○ | ○ |

WE M-26 Comments: Please use this section to provide any additional comments about the resilience of the national women empowerment opportunities and systems based on your experience during the COVID-19 pandemics. If you marked “I don’t know” for any of the questions, also use this section to explain why you gave that rating

JOHNS HOPKINS BLOOMBERG SCHOOL of PUBLIC HEALTH

Bill & Melinda Gates Institute for Population and Reproductive Health

## Demographic Dividend Effort Index Survey

### Labor Market (LM) Questionnaire

**Please respond to the following questions based on your experience/expertise within this specific sector.**

**To give a summary picture of the national demographic dividend effort, please rate the following items. Score each item from 1 to 10, where 1 represents the lowest score (very weak or no effort) and 10 represents the highest score (strong effort). Where applicable, if a policy or activity is non-existent then mark your response as 0.**

Answer each item. All responses will be recorded in the format exemplified below:

| Lowest effort | 1 | 2 | 3 | 4 | 5 | 6 | 7 | 8 | 9 | 10 | Highest effort | I do not know |
|---|---|---|---|---|---|---|---|---|---|---|---|---|
| | | | | | | | | | | | | |

JOHNS HOPKINS
BLOOMBERG SCHOOL
of PUBLIC HEALTH

Bill & Melinda Gates Institute for Population and Reproductive Health

## Demographic Dividend Effort Index Survey

### Dimension 1. Policy/Policy making

* LM 1.1 Global Economic Integration: Extent to which the country aims to be integrated in the global economy.

| | 1 | 2 | 3 | 4 | 5 | 6 | 7 | 8 | 9 | 10 | Don't know |
|---|---|---|---|---|---|---|---|---|---|---|---|
| LM 1.1.1 Extent to which the country legislates for trade openness. | ◯ | ◯ | ◯ | ◯ | ◯ | ◯ | ◯ | ◯ | ◯ | ◯ | ◯ |
| LM 1.1.2 Level of effort towards growing national exports. | ◯ | ◯ | ◯ | ◯ | ◯ | ◯ | ◯ | ◯ | ◯ | ◯ | ◯ |

* LM 1.2 Regional Economic Integration: Extent to which the country aims to be integrated in the regional economy.

| | 1 | 2 | 3 | 4 | 5 | 6 | 7 | 8 | 9 | 10 | Don't know |
|---|---|---|---|---|---|---|---|---|---|---|---|
| 1-Lowest -> Highest-10 | ◯ | ◯ | ◯ | ◯ | ◯ | ◯ | ◯ | ◯ | ◯ | ◯ | ◯ |

* LM 1.3 Technology: Extent to which the government has invested in infrastructure, both traditional and technological, to provide the basis for the country to leverage opportunities of the future and/or to integrate technology as a complement to labor.

| | 1 | 2 | 3 | 4 | 5 | 6 | 7 | 8 | 9 | 10 | Don't know |
|---|---|---|---|---|---|---|---|---|---|---|---|
| 1-Lowest -> Highest-10 | ◯ | ◯ | ◯ | ◯ | ◯ | ◯ | ◯ | ◯ | ◯ | ◯ | ◯ |

* LM 1.4 Government Support: Extent to which policies and plans are in place that provide for social safety nets to smooth transitions between jobs or 'gigs' for individuals and buffer income volatility.

| | 1 | 2 | 3 | 4 | 5 | 6 | 7 | 8 | 9 | 10 | Don't know |
|---|---|---|---|---|---|---|---|---|---|---|---|
| LM 1.4.1 Extent to which policies are in place that set a national minimum wage. | | | | | | | | | | | |
| LM 1.4.2 Extent to which policies exist that outline an unemployment insurance system, and the level to which they are effectively implemented. | | | | | | | | | | | |

* LM 1.5 Unemployment: Extent to which policies account for and address unemployment in various population groups and geographic areas.

| | 1 | 2 | 3 | 4 | 5 | 6 | 7 | 8 | 9 | 10 | Don't know |
|---|---|---|---|---|---|---|---|---|---|---|---|
| 1-Lowest -> Highest-10 | | | | | | | | | | | |

* LM 1.6 Disadvantaged/Vulnerable Groups: Extent to which policies exist, and are effectively implemented, to support the employment opportunities of disadvantage/vulnerable groups (i.e. girls and women, youth, persons with disabilities, etc.).

| | 1 | 2 | 3 | 4 | 5 | 6 | 7 | 8 | 9 | 10 | Don't know |
|---|---|---|---|---|---|---|---|---|---|---|---|
| 1-Lowest -> Highest-10 | | | | | | | | | | | |

* LM 1.7 Climate Change and Environmental Sustainability: Extent to which the agricultural and manufacturing sectors are prepared to labor market impacts from climate change effects through planning and strategizing for the future labor market needs.

| | 1 | 2 | 3 | 4 | 5 | 6 | 7 | 8 | 9 | 10 | Don't know |
|---|---|---|---|---|---|---|---|---|---|---|---|
| 1-Lowest -> Highest-10 | | | | | | | | | | | |

* LM 1.8 Urbanization:  Extent to which policies and planning anticipate and are prepared for increasing urbanization as a result of, and contributing to, climate change, and the particular impacts on labor markets.

| | 1 | 2 | 3 | 4 | 5 | 6 | 7 | 8 | 9 | 10 | Don't know |
|---|---|---|---|---|---|---|---|---|---|---|---|
| 1-Lowest -> Highest-10 | | | | | | | | | | | |

* LM 1.9 Service-Led Economy: Extent to which policies and framework are in place to move economies from informal labor to formalized labor responding to local, regional and global demands for goods and services.

| | 1 | 2 | 3 | 4 | 5 | 6 | 7 | 8 | 9 | 10 | Don't know |
|---|---|---|---|---|---|---|---|---|---|---|---|
| 1-Lowest -> Highest-10 | ◯ | ◯ | ◯ | ◯ | ◯ | ◯ | ◯ | ◯ | ◯ | ◯ | ◯ |

* LM 1.10 Corporate Social Responsibility: Extent to which national legislation in the labor market address corporate social responsibilities with the aim of supporting youth entrepreneurship.

| | 1 | 2 | 3 | 4 | 5 | 6 | 7 | 8 | 9 | 10 | Don't know |
|---|---|---|---|---|---|---|---|---|---|---|---|
| 1-Lowest -> Highest-10 | ◯ | ◯ | ◯ | ◯ | ◯ | ◯ | ◯ | ◯ | ◯ | ◯ | ◯ |

* LM 1.11 Targeted Labor Market Investments: Extent to which the government has targeted investments towards sectors with high job-multiplier effects, including Information and Communications Technology (ICT), manufacturing, agriculture and agro-industries in order to generate employment and spur inclusive growth.

| | 1 | 2 | 3 | 4 | 5 | 6 | 7 | 8 | 9 | 10 | Don't know |
|---|---|---|---|---|---|---|---|---|---|---|---|
| 1-Lowest -> Highest-10 | ◯ | ◯ | ◯ | ◯ | ◯ | ◯ | ◯ | ◯ | ◯ | ◯ | ◯ |

* LM 1.12 Self-Employment: Extent to which policies support raising the returns to self-employment and creating more opportunities to move from self-employment into higher paying wage employment. (This includes rural work).

| | 1 | 2 | 3 | 4 | 5 | 6 | 7 | 8 | 9 | 10 | Don't know |
|---|---|---|---|---|---|---|---|---|---|---|---|
| 1-Lowest -> Highest-10 | ◯ | ◯ | ◯ | ◯ | ◯ | ◯ | ◯ | ◯ | ◯ | ◯ | ◯ |

* LM 1.13 Microcredit: Extent to which the government enables access to microcredits for profitable activities, securing affordable credit for small and medium-sized enterprises and self-workers.

| | 1 | 2 | 3 | 4 | 5 | 6 | 7 | 8 | 9 | 10 | Don't know |
|---|---|---|---|---|---|---|---|---|---|---|---|
| 1-Lowest -> Highest-10 | ◯ | ◯ | ◯ | ◯ | ◯ | ◯ | ◯ | ◯ | ◯ | ◯ | ◯ |

* LM 1.14 Savings: Extent to which the government supports the creation of accessible financial products and policies to promote the culture of saving.

| | 1 | 2 | 3 | 4 | 5 | 6 | 7 | 8 | 9 | 10 | Don't know |
|---|---|---|---|---|---|---|---|---|---|---|---|
| 1-Lowest -> Highest-10 | ◯ | ◯ | ◯ | ◯ | ◯ | ◯ | ◯ | ◯ | ◯ | ◯ | ◯ |

* LM 1.15 Skill Acquisition: Extent to which policies exist, and are effectively implemented, to support the acquisition of currently and future-valued skills for the labor market.

| | 1 | 2 | 3 | 4 | 5 | 6 | 7 | 8 | 9 | 10 | Don't know |
|---|---|---|---|---|---|---|---|---|---|---|---|
| 1-Lowest -> Highest-10 | ○ | ○ | ○ | ○ | ○ | ○ | ○ | ○ | ○ | ○ | ○ |

LM 1.16 Comments: Please use this section to provide any additional comments about the level of effort, challenges, or successes in the country around policies/policymaking for LM. If you marked “I don’t know” for any of the questions, also use this section to explain why you gave that rating

JOHNS HOPKINS
BLOOMBERG SCHOOL
of PUBLIC HEALTH

Bill & Melinda Gates Institute for Population and Reproductive Health

## Demographic Dividend Effort Index Survey

### Dimension 2. Services or Programs

* LM 2.1 Unemployment Reduction: Extent to which there are efforts toward developing and implementing strategies aimed at the reduction of the proportion of unemployed by target years (in accordance with Agenda 2063 First Ten Year Implementation Plan).

| | 1 | 2 | 3 | 4 | 5 | 6 | 7 | 8 | 9 | 10 | Don't know |
|---|---|---|---|---|---|---|---|---|---|---|---|
| 1-Lowest -> Highest-10 | ○ | ○ | ○ | ○ | ○ | ○ | ○ | ○ | ○ | ○ | ○ |

* LM 2.2. Youth Unemployment: Extent to which labor market programs have effectively lowered the rate of unemployment, especially among youth.

| | 1 | 2 | 3 | 4 | 5 | 6 | 7 | 8 | 9 | 10 | Don't know |
|---|---|---|---|---|---|---|---|---|---|---|---|
| 1-Lowest -> Highest-10 | ○ | ○ | ○ | ○ | ○ | ○ | ○ | ○ | ○ | ○ | ○ |

* LM 2.3 Access to Microcredit: Extent to which national and regional funds have been established and operationalized in order to increase people's access to affordable business capital.

| | 1 | 2 | 3 | 4 | 5 | 6 | 7 | 8 | 9 | 10 | Don't know |
|---|---|---|---|---|---|---|---|---|---|---|---|
| 1-Lowest -> Highest-10 | ○ | ○ | ○ | ○ | ○ | ○ | ○ | ○ | ○ | ○ | ○ |

* LM 2.4 Harnessing Youth Special Capacities: Extent to which programs and services support the specific talents of youth to aid them in flourishing and achieving their maximum potential. (This includes specific programmatic aims around talent acquisition).

| | 1 | 2 | 3 | 4 | 5 | 6 | 7 | 8 | 9 | 10 | Don't know |
|---|---|---|---|---|---|---|---|---|---|---|---|
| 1-Lowest -> Highest-10 | ○ | ○ | ○ | ○ | ○ | ○ | ○ | ○ | ○ | ○ | ○ |

* LM 2.5 Early Labor Opportunities: Extent to which programs and government legislation engages with private sector partners to expand internships, apprenticeships and on-the-job training opportunities for women and youth.

| | 1 | 2 | 3 | 4 | 5 | 6 | 7 | 8 | 9 | 10 | Don't know |
|---|---|---|---|---|---|---|---|---|---|---|---|
| 1-Lowest -> Highest-10 | | | | | | | | | | | |

* LM 2.6 Youth Entrance into Labor: Extent of the promotion of youth volunteer and junior professional programs and other internship opportunities to enhance capacity and grant exposure to youth in regional and international organizations.

| | 1 | 2 | 3 | 4 | 5 | 6 | 7 | 8 | 9 | 10 | Don't know |
|---|---|---|---|---|---|---|---|---|---|---|---|
| 1-Lowest -> Highest-10 | | | | | | | | | | | |

* LM 2.7 Entrepreneurial Ventures: Extent to which people have enhanced access to government procurement and financial services to reduce the challenge of starting and/or doing business within and across countries.

| | 1 | 2 | 3 | 4 | 5 | 6 | 7 | 8 | 9 | 10 | Don't know |
|---|---|---|---|---|---|---|---|---|---|---|---|
| 1-Lowest -> Highest-10 | | | | | | | | | | | |

* LM 2.8 Resources for Youth Entrepreneurship: Extent to which entrepreneurial ventures of youth receive direct and targeted resource mobilization, supported by a regulatory framework, to reduce the challenge of starting and/or doing business within and across countries.

| | 1 | 2 | 3 | 4 | 5 | 6 | 7 | 8 | 9 | 10 | Don't know |
|---|---|---|---|---|---|---|---|---|---|---|---|
| 1-Lowest -> Highest-10 | | | | | | | | | | | |

* LM 2.9 Vocational Opportunities: Extent to which vocational training opportunities are expanded for skills acquisition for people to enhance their employability, productivity and competitiveness.

| | 1 | 2 | 3 | 4 | 5 | 6 | 7 | 8 | 9 | 10 | Don't know |
|---|---|---|---|---|---|---|---|---|---|---|---|
| 1-Lowest -> Highest-10 | | | | | | | | | | | |

* LM 2.10 Education and Labor: Extent to which improvements have been made to improve inclusive access to education at all levels and to provide viable alternatives for the many young people, particularly adolescent girls, who drop out of the formal education system, by facilitating re-entry, revamping informal education and training through standardized certification within and between countries.

| | 1 | 2 | 3 | 4 | 5 | 6 | 7 | 8 | 9 | 10 | Don't know |
|---|---|---|---|---|---|---|---|---|---|---|---|
| 1-Lowest -> Highest-10 | | | | | | | | | | | |

* LM 2.11 Women-Friendly Labor: Extent to which workplaces in the private and public sectors have adopted women-friendly policies and practices to eliminate any form of gender-based discrimination while allowing women to comfortably be part of the labor force.

| | 1 | 2 | 3 | 4 | 5 | 6 | 7 | 8 | 9 | 10 | Don't know |
|---|---|---|---|---|---|---|---|---|---|---|---|
| 1-Lowest -> Highest-10 | ◯ | ◯ | ◯ | ◯ | ◯ | ◯ | ◯ | ◯ | ◯ | ◯ | ◯ |

* LM 2.12 Job Security:

| | 1 | 2 | 3 | 4 | 5 | 6 | 7 | 8 | 9 | 10 | Don't know |
|---|---|---|---|---|---|---|---|---|---|---|---|
| LM 2.12.1 Extent to which notice periods for redundancy or dismissal are implemented. | ◯ | ◯ | ◯ | ◯ | ◯ | ◯ | ◯ | ◯ | ◯ | ◯ | ◯ |
| LM 2.12.2 Extent to which severance pay for redundancy or dismissal is implemented. | ◯ | ◯ | ◯ | ◯ | ◯ | ◯ | ◯ | ◯ | ◯ | ◯ | ◯ |

* LM 2.13 Minimum Wage: Extent to which a minimum wage is applied to the 'typical' worker (defined as an average wage-earner, in majority race and religious groups, in largest city, and non-union).

| | 1 | 2 | 3 | 4 | 5 | 6 | 7 | 8 | 9 | 10 | Don't know |
|---|---|---|---|---|---|---|---|---|---|---|---|
| 1-Lowest -> Highest-10 | ◯ | ◯ | ◯ | ◯ | ◯ | ◯ | ◯ | ◯ | ◯ | ◯ | ◯ |

* LM 2.14 Labor Market Programs: Extent to which labor market programs have effectively diversified the labor force and the needs of the labor market while simultaneously diversifying the skills of the labor force.

| | 1 | 2 | 3 | 4 | 5 | 6 | 7 | 8 | 9 | 10 | Don't know |
|---|---|---|---|---|---|---|---|---|---|---|---|
| 1-Lowest -> Highest-10 | ◯ | ◯ | ◯ | ◯ | ◯ | ◯ | ◯ | ◯ | ◯ | ◯ | ◯ |

* LM 2.15 Informality in the Labor Market: Extent to which the programs are implemented to transform informality in the labor market into formal labor.

| | 1 | 2 | 3 | 4 | 5 | 6 | 7 | 8 | 9 | 10 | Don't know |
|---|---|---|---|---|---|---|---|---|---|---|---|
| 1-Lowest -> Highest-10 | ◯ | ◯ | ◯ | ◯ | ◯ | ◯ | ◯ | ◯ | ◯ | ◯ | ◯ |

LM 2.16 Comments: Please use this section to provide any additional comments about the level of effort, challenges, or successes in the country around services or programs for LM. If you marked "I don't know" for any of the questions, also use this section to explain why you gave that rating.

JOHNS HOPKINS
BLOOMBERG SCHOOL
of PUBLIC HEALTH

Bill & Melinda Gates Institute for Population and Reproductive Health

## Demographic Dividend Effort Index Survey

### Dimension 3. Advocacy

* LM 3.1 Gender Equality in the Labor Market: Extent to which groups and individuals call for, and see improvements in, gender equality in the workforce.

| | 1 | 2 | 3 | 4 | 5 | 6 | 7 | 8 | 9 | 10 | Don't know |
|---|---|---|---|---|---|---|---|---|---|---|---|
| 1-Lowest -> Highest-10 | ◯ | ◯ | ◯ | ◯ | ◯ | ◯ | ◯ | ◯ | ◯ | ◯ | ◯ |

* LM 3.2 Youth Equality in the Labor Market: Extent to which individuals and groups call for shifts in the labor market and labor market regulations to benefit all rural and urban youth populations.

| | 1 | 2 | 3 | 4 | 5 | 6 | 7 | 8 | 9 | 10 | Don't know |
|---|---|---|---|---|---|---|---|---|---|---|---|
| 1-Lowest -> Highest-10 | ◯ | ◯ | ◯ | ◯ | ◯ | ◯ | ◯ | ◯ | ◯ | ◯ | ◯ |

* LM 3.3 Minimum Wage Advocacy: Extent to which advocacy groups are successful in inciting legislation for a minimum wage, or a rise in the minimum wage if one already exists.

| | 1 | 2 | 3 | 4 | 5 | 6 | 7 | 8 | 9 | 10 | Don't know |
|---|---|---|---|---|---|---|---|---|---|---|---|
| 1-Lowest -> Highest-10 | ◯ | ◯ | ◯ | ◯ | ◯ | ◯ | ◯ | ◯ | ◯ | ◯ | ◯ |

* LM 3.4 Government Advocacy: Extent to which members of the government advocates for improvements within its own structures and priorities. (These improvements may include labor market-focused progress for youth inclusion, opportunities, programming and rights among similar concepts across the population).

| | 1 | 2 | 3 | 4 | 5 | 6 | 7 | 8 | 9 | 10 | Don't know |
|---|---|---|---|---|---|---|---|---|---|---|---|
| 1-Lowest -> Highest-10 | ◯ | ◯ | ◯ | ◯ | ◯ | ◯ | ◯ | ◯ | ◯ | ◯ | ◯ |

* LM 3.5 Protections: Extent to which advocacy exists for job protection, and social protection in the absence of jobs.

| | 1 | 2 | 3 | 4 | 5 | 6 | 7 | 8 | 9 | 10 | Don't know |
|---|---|---|---|---|---|---|---|---|---|---|---|
| 1-Lowest -> Highest-10 | ◯ | ◯ | ◯ | ◯ | ◯ | ◯ | ◯ | ◯ | ◯ | ◯ | ◯ |

LM 3.6 Comments: Please use this section to provide any additional comments about the level of effort, challenges, or successes in advocacy work related to LM. If you marked “I don’t know” for any of the questions, also use this section to explain why you gave that rating.

JOHNS HOPKINS
BLOOMBERG SCHOOL
of PUBLIC HEALTH

Bill & Melinda Gates Institute for Population and Reproductive Health

## Demographic Dividend Effort Index Survey

### Dimension 4. Research

* LM 4.1 Strength of Observatory/Stakeholder/Technical Working Group for the LM: Extent to which country-level LM stakeholders (e.g., stakeholder leadership group, technical working group, LM observatory, country coordination and facilitation group):

| | 1 | 2 | 3 | 4 | 5 | 6 | 7 | 8 | 9 | 10 | Don't know |
|---|---|---|---|---|---|---|---|---|---|---|---|
| LM 4.1.1 Have representation in government, training institutions, civil society, nongovernmental and faith-based organizations, professional associations, the private sector, etc. | ○ | ○ | ○ | ○ | ○ | ○ | ○ | ○ | ○ | ○ | ○ |
| LM 4.1.2 Meet regularly, report and recommend policy for senior management within the appropriate ministries | ○ | ○ | ○ | ○ | ○ | ○ | ○ | ○ | ○ | ○ | ○ |
| LM 4.1.3 Make an impact on LM within the country | ○ | ○ | ○ | ○ | ○ | ○ | ○ | ○ | ○ | ○ | ○ |

* LM 4.2 Labor Market Research Strategy: Extent to which the national documents include a comprehensive strategy/approach on labor market-related research and is shared among partners.

| | 1 | 2 | 3 | 4 | 5 | 6 | 7 | 8 | 9 | 10 | Don't know |
|---|---|---|---|---|---|---|---|---|---|---|---|
| 1-Lowest -> Highest-10 | ○ | ○ | ○ | ○ | ○ | ○ | ○ | ○ | ○ | ○ | ○ |

* LM 4.3 Labor Market Analysis Partners:

| | 1 | 2 | 3 | 4 | 5 | 6 | 7 | 8 | 9 | 10 | Don't know |
|---|---|---|---|---|---|---|---|---|---|---|---|
| LM 4.3.1 Extent to which labor market research is undertaken by government agencies. | ○ | ○ | ○ | ○ | ○ | ○ | ○ | ○ | ○ | ○ | ○ |
| LM 4.3.2 Extent to which labor market research is undertaken by research institutions. | ○ | ○ | ○ | ○ | ○ | ○ | ○ | ○ | ○ | ○ | ○ |
| LM 4.3.3 Extent to which labor market research is undertaken by independent researchers. | ○ | ○ | ○ | ○ | ○ | ○ | ○ | ○ | ○ | ○ | ○ |

* LM 4.4 Areas of Research in the Labor Market:

| | 1 | 2 | 3 | 4 | 5 | 6 | 7 | 8 | 9 | 10 | Don't know |
|---|---|---|---|---|---|---|---|---|---|---|---|
| LM 4.4.1 Extent to which unemployment trends and patterns are researched. | ○ | ○ | ○ | ○ | ○ | ○ | ○ | ○ | ○ | ○ | ○ |
| LM 4.4.2 Extent of research on youth in the labor market. | ○ | ○ | ○ | ○ | ○ | ○ | ○ | ○ | ○ | ○ | ○ |
| LM 4.4.3 Extent of research on women in the labor market. | ○ | ○ | ○ | ○ | ○ | ○ | ○ | ○ | ○ | ○ | ○ |
| LM 4.4.4 Extent of research on the trends and trajectories of the labor market needs. | ○ | ○ | ○ | ○ | ○ | ○ | ○ | ○ | ○ | ○ | ○ |

* LM 4.5 Quality/Coverage of Data:

| | 1 | 2 | 3 | 4 | 5 | 6 | 7 | 8 | 9 | 10 | Don't know |
|---|---|---|---|---|---|---|---|---|---|---|---|
| LM 4.5.1 Extent to which a routine statistical system provides reliable periodic labor market data. | ○ | ○ | ○ | ○ | ○ | ○ | ○ | ○ | ○ | ○ | ○ |
| LM 4.5.2 Extent to which the routine statistical system provides reliable periodic information on LM needs among populations in different communities. | ○ | ○ | ○ | ○ | ○ | ○ | ○ | ○ | ○ | ○ | ○ |
| LM 4.5.3 Extent to which evaluations of the monitoring and surveillance systems are conducted and applied to ensure the reliability of data. | ○ | ○ | ○ | ○ | ○ | ○ | ○ | ○ | ○ | ○ | ○ |

* LM 4.6 Record-Keeping: Extent to which systems for labor market recordkeeping, reporting and feedback of results are adequate.

| | 1 | 2 | 3 | 4 | 5 | 6 | 7 | 8 | 9 | 10 | Don't know |
|---|---|---|---|---|---|---|---|---|---|---|---|
| 1-Lowest -> Highest-10 | ○ | ○ | ○ | ○ | ○ | ○ | ○ | ○ | ○ | ○ | ○ |

* LM 4.7 Quality Research Institutions: Extent to which the country has the capacity to sustain and maintain research institutions that develop research/collect labor market-related data.

| | 1 | 2 | 3 | 4 | 5 | 6 | 7 | 8 | 9 | 10 | Don't know |
|---|---|---|---|---|---|---|---|---|---|---|---|
| 1-Lowest -> Highest-10 | ○ | ○ | ○ | ○ | ○ | ○ | ○ | ○ | ○ | ○ | ○ |

* LM 4.8 Evaluation: Extent to which program statistics, surveys, and small studies are used by specialized staff to report on program operations and measure progress.

| | 1 | 2 | 3 | 4 | 5 | 6 | 7 | 8 | 9 | 10 | Don't know |
|---|---|---|---|---|---|---|---|---|---|---|---|
| 1-Lowest -> Highest-10 | ○ | ○ | ○ | ○ | ○ | ○ | ○ | ○ | ○ | ○ | ○ |

* LM 4.9 Management’s Use of Evaluation Findings: Extent to which local-level program managers use research and evaluation findings to improve LM related services and programs in ways suggested by findings.

| | 1 | 2 | 3 | 4 | 5 | 6 | 7 | 8 | 9 | 10 | Don't know |
|---|---|---|---|---|---|---|---|---|---|---|---|
| 1-Lowest -> Highest-10 | ○ | ○ | ○ | ○ | ○ | ○ | ○ | ○ | ○ | ○ | ○ |

* LM 4.10 Ministerial Use of Evaluation Findings: Extent to which the Ministry administrators systematically utilize data to inform policies and interventions to address LM-related issues.

| | 1 | 2 | 3 | 4 | 5 | 6 | 7 | 8 | 9 | 10 | Don't know |
|---|---|---|---|---|---|---|---|---|---|---|---|
| 1-Lowest -> Highest-10 | ○ | ○ | ○ | ○ | ○ | ○ | ○ | ○ | ○ | ○ | ○ |

* LM 4.11 Dissemination of Information to Other Implementing Bodies: Extent to which information is shared or spread across geographical areas and throughout various levels (national, state/province, localities).

| | 1 | 2 | 3 | 4 | 5 | 6 | 7 | 8 | 9 | 10 | Don't know |
|---|---|---|---|---|---|---|---|---|---|---|---|
| 1-Lowest -> Highest-10 | ○ | ○ | ○ | ○ | ○ | ○ | ○ | ○ | ○ | ○ | ○ |

LM 4.12 Comments: Please use this section to provide any additional comments about the level of effort, challenges, or successes in the country around LM research. If you marked “I don’t know” for any of the questions, also use this section to explain why you gave that rating.

JOHNS HOPKINS
BLOOMBERG SCHOOL of PUBLIC HEALTH

Bill & Melinda Gates Institute for Population and Reproductive Health

## Demographic Dividend Effort Index Survey

### Dimension 5. Civil Society Organizations (CSOs)

* LM 5.1 CSO Actor Power: Extent to which CSO actors are in strong positions to influence policies and action on the LM.

| | 1 | 2 | 3 | 4 | 5 | 6 | 7 | 8 | 9 | 10 | Don't know |
|---|---|---|---|---|---|---|---|---|---|---|---|
| 1-Lowest -> Highest-10 | ○ | ○ | ○ | ○ | ○ | ○ | ○ | ○ | ○ | ○ | ○ |

* LM 5.2 Budget Analysis as a CSO Tool: Extent to which CSOs utilize budget analysis as a tool to build an advocacy case for labor market.

| | 1 | 2 | 3 | 4 | 5 | 6 | 7 | 8 | 9 | 10 | Don't know |
|---|---|---|---|---|---|---|---|---|---|---|---|
| 1-Lowest -> Highest-10 | ○ | ○ | ○ | ○ | ○ | ○ | ○ | ○ | ○ | ○ | ○ |

* LM 5.3 Community-Based Service Delivery Support: Extent to which CSOs participate in bringing LM needs, information and services to the policy makers and vice-versa.

| | 1 | 2 | 3 | 4 | 5 | 6 | 7 | 8 | 9 | 10 | Don't know |
|---|---|---|---|---|---|---|---|---|---|---|---|
| 1-Lowest -> Highest-10 | ○ | ○ | ○ | ○ | ○ | ○ | ○ | ○ | ○ | ○ | ○ |

* LM 5.4 Diversification of Job Opportunities: Extent to which CSOs and the private sector coordinate with the government to ensure optimal job creation and equal participation of women and men.

| | 1 | 2 | 3 | 4 | 5 | 6 | 7 | 8 | 9 | 10 | Don't know |
|---|---|---|---|---|---|---|---|---|---|---|---|
| 1-Lowest -> Highest-10 | ○ | ○ | ○ | ○ | ○ | ○ | ○ | ○ | ○ | ○ | ○ |

* LM 5.5 Human Rights and Quality of Implementation: Extent to which CSOs support rights-based approaches at workplaces.

| | 1 | 2 | 3 | 4 | 5 | 6 | 7 | 8 | 9 | 10 | Don't know |
|---|---|---|---|---|---|---|---|---|---|---|---|
| 1-Lowest -> Highest-10 | ○ | ○ | ○ | ○ | ○ | ○ | ○ | ○ | ○ | ○ | ○ |

* LM 5.6 Youth: Extent to which CSOs support labor market policies, interventions and programming specifically targeting youth.

| | 1 | 2 | 3 | 4 | 5 | 6 | 7 | 8 | 9 | 10 | Don't know |
|---|---|---|---|---|---|---|---|---|---|---|---|
| 1-Lowest -> Highest-10 | ◯ | ◯ | ◯ | ◯ | ◯ | ◯ | ◯ | ◯ | ◯ | ◯ | ◯ |

* LM 5.7 Programming for Women: Extent to which CSOs support labor market policies, services and programs for women.

| | 1 | 2 | 3 | 4 | 5 | 6 | 7 | 8 | 9 | 10 | Don't know |
|---|---|---|---|---|---|---|---|---|---|---|---|
| 1-Lowest -> Highest-10 | ◯ | ◯ | ◯ | ◯ | ◯ | ◯ | ◯ | ◯ | ◯ | ◯ | ◯ |

* LM 5.8 Advocacy/Accountability: Extent to which CSOs support the training and capacity building of advocates and community members to understand policies, processes, and activities to increase community-led issue identification, collaborative problem solving and more targeted advocacy actions to address unemployment.

| | 1 | 2 | 3 | 4 | 5 | 6 | 7 | 8 | 9 | 10 | Don't know |
|---|---|---|---|---|---|---|---|---|---|---|---|
| 1-Lowest -> Highest-10 | ◯ | ◯ | ◯ | ◯ | ◯ | ◯ | ◯ | ◯ | ◯ | ◯ | ◯ |

* LM 5.9 CSO-Led Assessment and Monitoring: Extent to which CSOs assess, monitor and report on the effectiveness of policies and programming to improve accountability at the policy levels.

| | 1 | 2 | 3 | 4 | 5 | 6 | 7 | 8 | 9 | 10 | Don't know |
|---|---|---|---|---|---|---|---|---|---|---|---|
| 1-Lowest -> Highest-10 | ◯ | ◯ | ◯ | ◯ | ◯ | ◯ | ◯ | ◯ | ◯ | ◯ | ◯ |

* LM 5.10 Forming Partnerships between CSOs: Extent to which the private sector has formed national alliances and regional partnerships to strengthen the labor market.

| | 1 | 2 | 3 | 4 | 5 | 6 | 7 | 8 | 9 | 10 | Don't know |
|---|---|---|---|---|---|---|---|---|---|---|---|
| 1-Lowest -> Highest-10 | ◯ | ◯ | ◯ | ◯ | ◯ | ◯ | ◯ | ◯ | ◯ | ◯ | ◯ |

LM 5.11 Comments: Please use this section to provide any additional comments about the level of effort, challenges, or successes on the role of the CSO in the LM. If you marked “I don’t know” for any of the questions, also use this section to explain why you gave that rating

Johns Hopkins Bloomberg School of Public Health

Bill & Melinda Gates Institute for Population and Reproductive Health

## Demographic Dividend Effort Index Survey

### Module: Sectoral Resilience and Sustainability

**Considering the health and socio-economic impact of the COVID-19 and the likely impact on the DD progress, this DD effort index has integrated questions to assess the resilience and sustainability of systems in key sectors of the DD framework. The items included in the COVID-19 related questionnaire below follow the key dimensions of resilient systems and provide critical information on the potential of an effective response to emerging infectious disease threats or other public health emergencies.**

**Please score each item from 1 to 10, where 1 represents the lowest score (poor state/capability) and 10 represents the highest score (great state/capability).**

* Physical: facilities, equipment, system states, and capabilities.

| | 1 | 2 | 3 | 4 | 5 | 6 | 7 | 8 | 9 | 10 | Don't know |
|---|---|---|---|---|---|---|---|---|---|---|---|
| LM-M1 - Plan/Prepare - State and capability of equipment, personnel and LM sector structure prior to the crisis event. (1 = poor state/capability; 10 = great state/capability) | ○ | ○ | ○ | ○ | ○ | ○ | ○ | ○ | ○ | ○ | ○ |
| LM-M2 - Absorb - Extent to which COVID-19 was recognized within the LM sector and the performance of the system was able to maintain optimal employment and economic productivity. | ○ | ○ | ○ | ○ | ○ | ○ | ○ | ○ | ○ | ○ | ○ |
| LM-M3 - Recover - Extent to which the LM sector has been able to induce change to recover the pre-COVID-19 level of functionality. | ○ | ○ | ○ | ○ | ○ | ○ | ○ | ○ | ○ | ○ | ○ |

120

| | 1 | 2 | 3 | 4 | 5 | 6 | 7 | 8 | 9 | 10 | Don't know |
|---|---|---|---|---|---|---|---|---|---|---|---|
| LM-M4 - Recover - Financing and Donor Management: Extent to which there are established social protection mechanisms (and other forms of income smoothing) for unprotected workers (e.g. self-employed workers, that have no access to paid or sick leave). | ○ | ○ | ○ | ○ | ○ | ○ | ○ | ○ | ○ | ○ | ○ |
| LM-M5 - Adapt - Financing and Donor Management: Extent to which there has been institutional and policy reforms to build resilience by a demand-led approach through large-scale social protection systems that can act as economic stabilizers | ○ | ○ | ○ | ○ | ○ | ○ | ○ | ○ | ○ | ○ | ○ |

* Information: creation, manipulation, storage, and utilization of data.

| | 1 | 2 | 3 | 4 | 5 | 6 | 7 | 8 | 9 | 10 | Don't know |
|---|---|---|---|---|---|---|---|---|---|---|---|
| LM-M6 - Plan/Prepare - Extent to which LM-related data was sufficiently prepared, presented, analyzed and stored prior to the crisis event. | ○ | ○ | ○ | ○ | ○ | ○ | ○ | ○ | ○ | ○ | ○ |
| LM-M7 - Absorb - Extent to which the LM sector has been able to conduct a real-time assessment of functionality, in anticipation of cascading losses in LM and productivity. | ○ | ○ | ○ | ○ | ○ | ○ | ○ | ○ | ○ | ○ | ○ |
| LM-M8 - Recover - Extent to which LM data has been used to track the progress of recovery and to anticipate recovery scenarios. | ○ | ○ | ○ | ○ | ○ | ○ | ○ | ○ | ○ | ○ | ○ |
| LM-M9 - Adapt - Extent to which the LM sector is creating and improving LM data storage and real-time use protocols. | ○ | ○ | ○ | ○ | ○ | ○ | ○ | ○ | ○ | ○ | ○ |

* Cognitive: understanding, mental models, preconceptions, biases, and values.

| | 1 | 2 | 3 | 4 | 5 | 6 | 7 | 8 | 9 | 10 | Don't know |
|---|---|---|---|---|---|---|---|---|---|---|---|
| LM-M10 - Plan/Prepare - Extent to which the LM sector design and operation decisions were prepared in anticipation of crisis events. | ○ | ○ | ○ | ○ | ○ | ○ | ○ | ○ | ○ | ○ | ○ |
| LM-M11 - Absorb - Extent to which the LM sector is responding with sufficient contingency protocols and proactive crisis management. | ○ | ○ | ○ | ○ | ○ | ○ | ○ | ○ | ○ | ○ | ○ |

| | 1 | 2 | 3 | 4 | 5 | 6 | 7 | 8 | 9 | 10 | Don't know |
|---|---|---|---|---|---|---|---|---|---|---|---|
| LM-M12 - Absorb - Leadership and Management: Extent to which the government is ensuring workers and employers and their families are protected from the health risks of COVID-19. | ○ | ○ | ○ | ○ | ○ | ○ | ○ | ○ | ○ | ○ | ○ |
| LM-M13 - Absorb - Accountability: Level of effort to ensure optimal accountability for the resources allocated to ensure protection from the health risks of COVID-19 and the recovery of the labor market as part of the response to mitigate COVID-19 impacts. | ○ | ○ | ○ | ○ | ○ | ○ | ○ | ○ | ○ | ○ | ○ |
| LM-M14 - Recover - Extent to which decisions are oriented towards recovery, which employ evidence-based communication to community members to promote safe behavior. | ○ | ○ | ○ | ○ | ○ | ○ | ○ | ○ | ○ | ○ | ○ |
| LM-M15 - Recover - Awareness: Extent to which the government is building confidence through trust and dialogue for building the commitment of employers and workers to the joint action of the government and promoting enterprise-level social dialogue. | ○ | ○ | ○ | ○ | ○ | ○ | ○ | ○ | ○ | ○ | ○ |
| LM-M16 - Adapt - Extent to which the LM sector is designing new system configurations, objectives and decision criteria. | ○ | ○ | ○ | ○ | ○ | ○ | ○ | ○ | ○ | ○ | ○ |

123

| | 1 | 2 | 3 | 4 | 5 | 6 | 7 | 8 | 9 | 10 | Don't know |
|---|---|---|---|---|---|---|---|---|---|---|---|
| LM-M17 - Adapt - Adaptive: Extent to which the government is promoting change in the workspace to protect workers (e.g. occupational and safety health measures, adapted work arrangements, prevention of discrimination and exclusion, expanded access to paid leave, etc.). | ◯ | ◯ | ◯ | ◯ | ◯ | ◯ | ◯ | ◯ | ◯ | ◯ | ◯ |

* Social: interaction, collaboration, and self-synchronization between individuals, entities and institutions.

| | 1 | 2 | 3 | 4 | 5 | 6 | 7 | 8 | 9 | 10 | Don't know |
|---|---|---|---|---|---|---|---|---|---|---|---|
| LM-M18 - Plan/Prepare - Extent to which training on outbreak/crisis management was conducted through, and which leveraged, social networks, social capital and institutional and cultural norms prior to the crisis event. | ◯ | ◯ | ◯ | ◯ | ◯ | ◯ | ◯ | ◯ | ◯ | ◯ | ◯ |
| LM-M19 - Absorb - Extent to which personnel and social institutions have been resourceful and accessible for the outbreak/crisis response. | ◯ | ◯ | ◯ | ◯ | ◯ | ◯ | ◯ | ◯ | ◯ | ◯ | ◯ |
| LM-M20 - Absorb - Self-Regulating: Extent to which the national leadership had the authority to make sectoral changes in a timely manner through flexible labor market infrastructure. | ◯ | ◯ | ◯ | ◯ | ◯ | ◯ | ◯ | ◯ | ◯ | ◯ | ◯ |
| LM-M21 - Recover - Extent to which the LM sector is engaging with team and knowledge sharing to enhance systematic recovery. | ◯ | ◯ | ◯ | ◯ | ◯ | ◯ | ◯ | ◯ | ◯ | ◯ | ◯ |

| | 1 | 2 | 3 | 4 | 5 | 6 | 7 | 8 | 9 | 10 | Don't know |
|---|---|---|---|---|---|---|---|---|---|---|---|
| LM-M22 - Recover - Diversity: Extent to which the government is facilitating a strong and fast recovery of the labor market by diversifying policies that favor both the supply (production of good and services) and demand (consumption and investment). | ○ | ○ | ○ | ○ | ○ | ○ | ○ | ○ | ○ | ○ | ○ |
| LM-M23 - Adapt - Extent to which there are additions or changes being made to LM institutions, policies, trainings, programs and culture. | ○ | ○ | ○ | ○ | ○ | ○ | ○ | ○ | ○ | ○ | ○ |
| LM-M24 - Adapt - Leadership and Management: Extent to which there has been established a tripartite fluent dialogue between government, workers, and employers' organizations. | ○ | ○ | ○ | ○ | ○ | ○ | ○ | ○ | ○ | ○ | ○ |

* LM M-25 Timeline: Extent of timeliness of the Government reaction - communication and implementation of measures - to mitigate the immediate and longterm impact of COVID-19.
*Note: This refers to the entirety of the national reaction - not the reaction that is specific to your sector.*

| | 1 | 2 | 3 | 4 | 5 | 6 | 7 | 8 | 9 | 10 | Don't know |
|---|---|---|---|---|---|---|---|---|---|---|---|
| 1-Lowest -> Highest-10 | ○ | ○ | ○ | ○ | ○ | ○ | ○ | ○ | ○ | ○ | ○ |

LM M-26 Comments: Please use this section to provide any additional comments about the resilience and sustainability of the LM systems based on your experience during the COVID-19 pandemics. If you marked “I don’t know” for any of the questions, also use this section to explain why you gave that rating.

125

JOHNS HOPKINS
BLOOMBERG SCHOOL
of PUBLIC HEALTH

Bill & Melinda Gates Institute for Population and Reproductive Health

## Demographic Dividend Effort Index Survey

### Governance and Economic Institutions (GEI) Questionnaire

**Please respond to the following questions based on your experience/expertise within this specific sector.**

**To give a summary picture of the national demographic dividend effort, please rate the following items. Score each item from 1 to 10, where 1 represents the lowest score (very weak or no effort) and 10 represents the highest score (strong effort). Where applicable, if a policy or activity is non-existent then mark your response as 0.**

Answer each item. All responses will be recorded in the format exemplified below:

| Lowest effort | 1 | 2 | 3 | 4 | 5 | 6 | 7 | 8 | 9 | 10 | Highest effort | I do not know |
|---|---|---|---|---|---|---|---|---|---|---|---|---|
| | | | | | | | | | | | | |

126

JOHNS HOPKINS
BLOOMBERG SCHOOL
of PUBLIC HEALTH

Bill & Melinda Gates Institute for Population and Reproductive Health

## Demographic Dividend Effort Index Survey

### Dimension 1. Policies

* GEI 1.1 Democratic Values: Extent to which the government has ratified, domesticated and implemented regional instruments for shared values, including those relating to democracy, elections and governance and the right to health.

| | 1 | 2 | 3 | 4 | 5 | 6 | 7 | 8 | 9 | 10 | Don't know |
|---|---|---|---|---|---|---|---|---|---|---|---|
| 1-Lowest -> Highest-10 | ○ | ○ | ○ | ○ | ○ | ○ | ○ | ○ | ○ | ○ | ○ |

* GEI 1.2 Monitoring Democratic Values: Extent to which the government has created mechanisms for regular and periodic reporting on the implementation of the regional instruments on shared values, through existing mechanisms such as universal periodic review on the status of international or regional instruments or using independent bodies. (This may take the form of regular reports on the status of implementation).

| | 1 | 2 | 3 | 4 | 5 | 6 | 7 | 8 | 9 | 10 | Don't know |
|---|---|---|---|---|---|---|---|---|---|---|---|
| 1-Lowest -> Highest-10 | ○ | ○ | ○ | ○ | ○ | ○ | ○ | ○ | ○ | ○ | ○ |

* GEI 1.3 Affirmation of Youth Democratic Participation: Extent to which the government has explicitly removed all discriminatory laws and limitations to full participation of young people in electoral processes through inclusive electoral laws and constitutions. (This may include developing and/or strengthening youth leadership programs and/or providing capacity building on management and leadership skills).

| | 1 | 2 | 3 | 4 | 5 | 6 | 7 | 8 | 9 | 10 | Don't know |
|---|---|---|---|---|---|---|---|---|---|---|---|
| 1-Lowest -> Highest-10 | ○ | ○ | ○ | ○ | ○ | ○ | ○ | ○ | ○ | ○ | ○ |

* GEI 1.4. Removing Legal Youth Barriers: Extent to which the government has proscribed all laws posing barriers to the full exercise and enjoyment of the fundamental rights of young people to fully participate in the democratic governance processes at the continental, regional, national and grassroots levels. (This may also include sustaining the mainstreaming of adolescents and youth health and rights issues into the country's growth and transformation agenda).

| | 1 | 2 | 3 | 4 | 5 | 6 | 7 | 8 | 9 | 10 | Don't know |
|---|---|---|---|---|---|---|---|---|---|---|---|
| 1-Lowest -> Highest-10 | ◯ | ◯ | ◯ | ◯ | ◯ | ◯ | ◯ | ◯ | ◯ | ◯ | ◯ |

* GEI 1.5 Youth Policy: Extent to which the youth policy takes into account the latest thinking in youth education, training and employment.

| | 1 | 2 | 3 | 4 | 5 | 6 | 7 | 8 | 9 | 10 | Don't know |
|---|---|---|---|---|---|---|---|---|---|---|---|
| 1-Lowest -> Highest-10 | ◯ | ◯ | ◯ | ◯ | ◯ | ◯ | ◯ | ◯ | ◯ | ◯ | ◯ |

* GEI 1.6 Affirmation of Democratic Participation/Revision of Customary Law: Extent to which the government has reviewed, revised, amended or abolished all laws, regulations, policies, practices and customs that have a discriminatory impact on youth, especially girls and young women, without distinction of any kind, and ensure that the provisions of multiple legal systems comply with international human rights regulations and laws. (These must include protection from harmful practices like early, forced or child marriages, sexual and gender-based violence, female genital mutilation (FGM).

| | 1 | 2 | 3 | 4 | 5 | 6 | 7 | 8 | 9 | 10 | Don't know |
|---|---|---|---|---|---|---|---|---|---|---|---|
| 1-Lowest -> Highest-10 | ◯ | ◯ | ◯ | ◯ | ◯ | ◯ | ◯ | ◯ | ◯ | ◯ | ◯ |

* GEI 1.7 Youth Policymaking Participation: Extent to which the government ensures the involvement of youth leadership and participation in decision-making processes in efforts relating to policymaking including youth and education policy making. Potentially includes efforts to establish a national youth parliament and assembly. (This may include supporting adolescent and youth participation and leadership in health planning and programming at all levels).

| | 1 | 2 | 3 | 4 | 5 | 6 | 7 | 8 | 9 | 10 | Don't know |
|---|---|---|---|---|---|---|---|---|---|---|---|
| 1-Lowest -> Highest-10 | ◯ | ◯ | ◯ | ◯ | ◯ | ◯ | ◯ | ◯ | ◯ | ◯ | ◯ |

* GEI 1.8 Rule of Law: Extent to which the rule of law is formally, legally established to be equitable and implemented independently by the judiciary.

| | 1 | 2 | 3 | 4 | 5 | 6 | 7 | 8 | 9 | 10 | Don't know |
|---|---|---|---|---|---|---|---|---|---|---|---|
| 1-Lowest -> Highest-10 | ◯ | ◯ | ◯ | ◯ | ◯ | ◯ | ◯ | ◯ | ◯ | ◯ | ◯ |

* GEI 1.9 Transparency and Accountability: Extent to which national legislation mandates transparency and accountability across all branches and throughout all sectors.

| | 1 | 2 | 3 | 4 | 5 | 6 | 7 | 8 | 9 | 10 | Don't know |
|---|---|---|---|---|---|---|---|---|---|---|---|
| GEI 1.9.1 Extent to which the public can access public and legislative information as well as the records of state-owned organizations. | ◯ | ◯ | ◯ | ◯ | ◯ | ◯ | ◯ | ◯ | ◯ | ◯ | ◯ |
| GEI 1.9.2 Extent to which there are mandated actions and mechanisms for accountability and sanctioning. | ◯ | ◯ | ◯ | ◯ | ◯ | ◯ | ◯ | ◯ | ◯ | ◯ | ◯ |

* GEI 1.10 National Security: Extent to which the country/government is involved in armed conflict, either internationally or domestically.

| | 1 | 2 | 3 | 4 | 5 | 6 | 7 | 8 | 9 | 10 | Don't know |
|---|---|---|---|---|---|---|---|---|---|---|---|
| GEI 1.10.1 Extent to which the country has cross-border tensions. | ◯ | ◯ | ◯ | ◯ | ◯ | ◯ | ◯ | ◯ | ◯ | ◯ | ◯ |
| GEI 1.10.2 Extent to which the country contains internally displaced persons and/or refugees. | ◯ | ◯ | ◯ | ◯ | ◯ | ◯ | ◯ | ◯ | ◯ | ◯ | ◯ |
| GEI 1.10.3 Extent to which education facilities are protected and alternative learning opportunities are created for children and youth in time of conflict or displacement. | ◯ | ◯ | ◯ | ◯ | ◯ | ◯ | ◯ | ◯ | ◯ | ◯ | ◯ |

* GEI 1.11 Rights: Extent to which the constitution affirms certain rights: freedom of expression, freedom of association and assembly, civil rights and liberties, and protection against ethnic and religious discrimination, health as well as an un-likelihood of human rights abuses by the government.

| | 1 | 2 | 3 | 4 | 5 | 6 | 7 | 8 | 9 | 10 | Don't know |
|---|---|---|---|---|---|---|---|---|---|---|---|
| 1-Lowest -> Highest-10 | ◯ | ◯ | ◯ | ◯ | ◯ | ◯ | ◯ | ◯ | ◯ | ◯ | ◯ |

* GEI 1.12 Education and Democratic Values: Extent to which education policies, strategies and programs promote academic freedom, democratic values and education as human right.

| | 1 | 2 | 3 | 4 | 5 | 6 | 7 | 8 | 9 | 10 | Don't know |
|---|---|---|---|---|---|---|---|---|---|---|---|
| 1-Lowest -> Highest-10 | ○ | ○ | ○ | ○ | ○ | ○ | ○ | ○ | ○ | ○ | ○ |

* GEI 1.13 Affirmation of Safe Learning Environments: Extent to which education policies provide for safe learning environments through protections for children. (This may come in the form of ensuring the safety of girls, banning corporal punishment, etc.).

| | 1 | 2 | 3 | 4 | 5 | 6 | 7 | 8 | 9 | 10 | Don't know |
|---|---|---|---|---|---|---|---|---|---|---|---|
| 1-Lowest -> Highest-10 | ○ | ○ | ○ | ○ | ○ | ○ | ○ | ○ | ○ | ○ | ○ |

GEI 1.14 Comments: Please use this section to provide any additional comments about the level of effort, challenges, or successes in the country around policies/policymaking for GEI. If you marked “I don’t know” for any of the questions, also use this section to explain why you gave that rating

JOHNS HOPKINS BLOOMBERG SCHOOL of PUBLIC HEALTH

Bill & Melinda Gates Institute for Population and Reproductive Health

## Demographic Dividend Effort Index Survey

### Dimension 2. Services or Programs

* GEI 2.1 Strengthening Youth Activities/Institutions: Extent to which efforts are being made to strengthen independent youth formations, networks and organizations including the establishment of independent youth commissions, student unions at the national and sub-regional levels to champion youth rights.

| | 1 | 2 | 3 | 4 | 5 | 6 | 7 | 8 | 9 | 10 | Don't know |
|---|---|---|---|---|---|---|---|---|---|---|---|
| 1-Lowest -> Highest-10 | ◯ | ◯ | ◯ | ◯ | ◯ | ◯ | ◯ | ◯ | ◯ | ◯ | ◯ |

* GEI 2.2 Appointment of Youth Leader: Whether and to what extent there has been a youth envoy appointed to lead advocacy and champion the prioritization of youth issues (e.g. education, training and employment) within decision-making spaces at a variety of levels including the parliament.

| | 1 | 2 | 3 | 4 | 5 | 6 | 7 | 8 | 9 | 10 | Don't know |
|---|---|---|---|---|---|---|---|---|---|---|---|
| 1-Lowest -> Highest-10 | ◯ | ◯ | ◯ | ◯ | ◯ | ◯ | ◯ | ◯ | ◯ | ◯ | ◯ |

* GEI 2.3 Youth Ideals and Aspirations: Extent to which programming is designed to institute youth leadership and empowerment trainings aimed at deepening cultural ideals and aspirations in the youth.

| | 1 | 2 | 3 | 4 | 5 | 6 | 7 | 8 | 9 | 10 | Don't know |
|---|---|---|---|---|---|---|---|---|---|---|---|
| 1-Lowest -> Highest-10 | ◯ | ◯ | ◯ | ◯ | ◯ | ◯ | ◯ | ◯ | ◯ | ◯ | ◯ |

* GEI 2.4 Youth Services: Extent to which programming is designed to address youth-specific needs. (This should consider the development and implementation of adolescent and youth-friendly health services, among other similar programmatic endeavors).

| | 1 | 2 | 3 | 4 | 5 | 6 | 7 | 8 | 9 | 10 | Don't know |
|---|---|---|---|---|---|---|---|---|---|---|---|
| 1-Lowest -> Highest-10 | ◯ | ◯ | ◯ | ◯ | ◯ | ◯ | ◯ | ◯ | ◯ | ◯ | ◯ |

* GEI 2.5 Youth Involvement in National Initiatives: Extent to which there are efforts to strengthen existing youth engagement and participation initiatives of the country *(e.g. at the level of the African Union these may include the African Youth Volunteers Corps, African Union Youth Clubs, AGA-YES activities including the Youth Advisory Panel, the Moot Court and Annual Youth Dialogue on Democracy, Human Rights and Governance*). (This may include engaging adolescents and youth in the provision of technical support to sector ministries and relevant federal and regional government bodies for developing policies, plans, and programs that integrate responses to priority health issues affecting this group).

| | 1 | 2 | 3 | 4 | 5 | 6 | 7 | 8 | 9 | 10 | Don't know |
|---|---|---|---|---|---|---|---|---|---|---|---|
| 1-Lowest -> Highest-10 | ◯ | ◯ | ◯ | ◯ | ◯ | ◯ | ◯ | ◯ | ◯ | ◯ | ◯ |

* GEI 2.6 Personal Safety: Extent to which individual safety is provided for through equitable, reliable police services/activities, contributing to an overall positive perception of personal safety at the household and community levels as well as the school environment. (This may include protecting youth form any form of abuse, neglect or disrespect in the use of health services).

| | 1 | 2 | 3 | 4 | 5 | 6 | 7 | 8 | 9 | 10 | Don't know |
|---|---|---|---|---|---|---|---|---|---|---|---|
| 1-Lowest -> Highest-10 | ◯ | ◯ | ◯ | ◯ | ◯ | ◯ | ◯ | ◯ | ◯ | ◯ | ◯ |

* GEI 2.7 Participation: Extent to which government functions involve the participation of citizens, civil society organizations, and other intersectoral actors to promote democratic elections and the effective power to govern.

| | 1 | 2 | 3 | 4 | 5 | 6 | 7 | 8 | 9 | 10 | Don't know |
|---|---|---|---|---|---|---|---|---|---|---|---|
| 1-Lowest -> Highest-10 | ◯ | ◯ | ◯ | ◯ | ◯ | ◯ | ◯ | ◯ | ◯ | ◯ | ◯ |

* GEI 2.8 Gender: Extent to which women participate in:

| | 1 | 2 | 3 | 4 | 5 | 6 | 7 | 8 | 9 | 10 | Don't know |
|---|---|---|---|---|---|---|---|---|---|---|---|
| GEI 2.8.1 Governance at the national level | ◯ | ◯ | ◯ | ◯ | ◯ | ◯ | ◯ | ◯ | ◯ | ◯ | ◯ |
| GEI 2.8.2 Governance at the sub-national level | ◯ | ◯ | ◯ | ◯ | ◯ | ◯ | ◯ | ◯ | ◯ | ◯ | ◯ |
| GEI 2.8.3 Governance in rural areas | ◯ | ◯ | ◯ | ◯ | ◯ | ◯ | ◯ | ◯ | ◯ | ◯ | ◯ |
| GEI 2.8.4 Governance in urban areas | ◯ | ◯ | ◯ | ◯ | ◯ | ◯ | ◯ | ◯ | ◯ | ◯ | ◯ |
| GEI 2.8.5 Judiciary | ◯ | ◯ | ◯ | ◯ | ◯ | ◯ | ◯ | ◯ | ◯ | ◯ | ◯ |

* GEI 2.9 Promotion of Equality: Extent to which gender equality in all aspects of society is promoted by the government. (This may include equality in education, equality before law, equality in healthcare, equality in employment and renumeration, among many other facets of society).

| | 1 | 2 | 3 | 4 | 5 | 6 | 7 | 8 | 9 | 10 | Don't know |
|---|---|---|---|---|---|---|---|---|---|---|---|
| 1-Lowest -> Highest-10 | ○ | ○ | ○ | ○ | ○ | ○ | ○ | ○ | ○ | ○ | ○ |

* GEI 2.10 Sustainable Economic Opportunity:

| | 1 | 2 | 3 | 4 | 5 | 6 | 7 | 8 | 9 | 10 | Don't know |
|---|---|---|---|---|---|---|---|---|---|---|---|
| GEI 2.10.1 Extent to which the government provides for and ensures that a strong public management system is realized. | ○ | ○ | ○ | ○ | ○ | ○ | ○ | ○ | ○ | ○ | ○ |
| GEI 2.10.2 Extent to which the government provides for and ensures that a strong business environment is realized. | ○ | ○ | ○ | ○ | ○ | ○ | ○ | ○ | ○ | ○ | ○ |
| GEI 2.10.3 Extent to which the government provides for and ensures that a strong public development infrastructure is realized. | ○ | ○ | ○ | ○ | ○ | ○ | ○ | ○ | ○ | ○ | ○ |
| GEI 2.10.4 Extent to which the government provides for and ensures that a strong rural sector for development is realized. | ○ | ○ | ○ | ○ | ○ | ○ | ○ | ○ | ○ | ○ | ○ |

* GEI 2.11 Human Development:

| | 1 | 2 | 3 | 4 | 5 | 6 | 7 | 8 | 9 | 10 | Don't know |
|---|---|---|---|---|---|---|---|---|---|---|---|
| GEI 2.11.1 Extent to which the welfare of the general public is well-handled by the government (consider aspects related, but not limited, to welfare policies and services, socioeconomic integration of youth and social protections and safety nets, among many other areas). This may also that the government, with the obligations to protect, fulfil and respect basic health rights, has given the responsibility of its implementation to its ministerial cabinet. | ○ | ○ | ○ | ○ | ○ | ○ | ○ | ○ | ○ | ○ | ○ |
| GEI 2.11.2 Extent to which the government is promoting the skills development of the population at workplaces. | ○ | ○ | ○ | ○ | ○ | ○ | ○ | ○ | ○ | ○ | ○ |
| GEI 2.11.3 Extent to which the government is promoting the education, knowledge and capacities of the population through schools and continuing education opportunities. | ○ | ○ | ○ | ○ | ○ | ○ | ○ | ○ | ○ | ○ | ○ |
| GEI 2.11.4 Extent to which the government is promoting the health and wellness of the population. | ○ | ○ | ○ | ○ | ○ | ○ | ○ | ○ | ○ | ○ | ○ |

GEI 2.12 Comments: Please use this section to provide any additional comments about the level of effort, challenges, or successes in the country around services or programs for GEI. If you marked “I don’t know” for any of the questions, also use this section to explain why you gave that rating.

JOHNS HOPKINS
BLOOMBERG SCHOOL
of PUBLIC HEALTH

Bill & Melinda Gates Institute for Population and Reproductive Health

## Demographic Dividend Effort Index Survey

### Dimension 3. Advocacy

* GEI 3.1 Youth Empowerment: Extent to which youth empowerment has been enhanced, potentially through the integration of civic education in national educational curricula, media platforms and other channels to instill principles of the rule of law, human rights and individual duties and responsibilities. (This may include the mainstreaming of adolescents and youth health and rights issues into the country’s growth and transformation agenda and educational curricula).

| | 1 | 2 | 3 | 4 | 5 | 6 | 7 | 8 | 9 | 10 | Don't know |
|---|---|---|---|---|---|---|---|---|---|---|---|
| 1-Lowest -> Highest-10 | ○ | ○ | ○ | ○ | ○ | ○ | ○ | ○ | ○ | ○ | ○ |

* GEI 3.2 Government Advocacy: Extent to which members of the government advocates for improvements within its own structures and priorities. (These improvements may include progress for youth inclusion, opportunities, programming and rights among similar concepts across the population).

| | 1 | 2 | 3 | 4 | 5 | 6 | 7 | 8 | 9 | 10 | Don't know |
|---|---|---|---|---|---|---|---|---|---|---|---|
| 1-Lowest -> Highest-10 | ○ | ○ | ○ | ○ | ○ | ○ | ○ | ○ | ○ | ○ | ○ |

* GEI 3.3 Non-governmental Advocacy: Extent to which non-governmental actors advocates for improvements in government structures and priorities. (These improvements may include progress for youth inclusion, opportunities, programming and rights among similar concepts across the population).

| | 1 | 2 | 3 | 4 | 5 | 6 | 7 | 8 | 9 | 10 | Don't know |
|---|---|---|---|---|---|---|---|---|---|---|---|
| 1-Lowest -> Highest-10 | ○ | ○ | ○ | ○ | ○ | ○ | ○ | ○ | ○ | ○ | ○ |

* GEI 3.4 Civil Society Organizations (CSOs): Extent to which CSOs specifically operate as instruments for advocacy in the GEI sector.

| | 1 | 2 | 3 | 4 | 5 | 6 | 7 | 8 | 9 | 10 | Don't know |
|---|---|---|---|---|---|---|---|---|---|---|---|
| 1-Lowest -> Highest-10 | ○ | ○ | ○ | ○ | ○ | ○ | ○ | ○ | ○ | ○ | ○ |

* GEI 3.5 Advocacy Voice: Extent to which advocates have a voice in government discussions.

| | 1 | 2 | 3 | 4 | 5 | 6 | 7 | 8 | 9 | 10 | Don't know |
|---|---|---|---|---|---|---|---|---|---|---|---|
| 1-Lowest -> Highest-10 | ○ | ○ | ○ | ○ | ○ | ○ | ○ | ○ | ○ | ○ | ○ |

* GEI 3.6 Voice of Instrumental Sectors of the DD: Extent to which the government and stakeholders are committed to establish sustainable institutions to ensure:

| | 1 | 2 | 3 | 4 | 5 | 6 | 7 | 8 | 9 | 10 | Don't know |
|---|---|---|---|---|---|---|---|---|---|---|---|
| GEI 3.6.1 Good governance | ○ | ○ | ○ | ○ | ○ | ○ | ○ | ○ | ○ | ○ | ○ |
| GEI 3.6.2 Financial stability | ○ | ○ | ○ | ○ | ○ | ○ | ○ | ○ | ○ | ○ | ○ |
| GEI 3.6.3 Optimal family planning programs | ○ | ○ | ○ | ○ | ○ | ○ | ○ | ○ | ○ | ○ | ○ |
| GEI 3.6.4 Optimal maternal and child health | ○ | ○ | ○ | ○ | ○ | ○ | ○ | ○ | ○ | ○ | ○ |
| GEI 3.6.5 Good quality education | ○ | ○ | ○ | ○ | ○ | ○ | ○ | ○ | ○ | ○ | ○ |
| GEI 3.6.6 Diversified labor market. | ○ | ○ | ○ | ○ | ○ | ○ | ○ | ○ | ○ | ○ | ○ |

GEI 3.7 Comments: Please use this section to provide any additional comments about the level of effort, challenges, or successes in the country around advocacy for GEI. If you marked “I don’t know” for any of the questions, also use this section to explain why you gave that rating

Johns Hopkins Bloomberg School of Public Health

Bill & Melinda Gates Institute for Population and Reproductive Health

## Demographic Dividend Effort Index Survey

### Dimension 4. Research

* GEI 4.1 Strength of Observatory/Stakeholder/Technical Working Group for GEI: Extent to which country-level GEI stakeholders (e.g., stakeholder leadership group, technical working group, GEI observatory, country coordination and facilitation group):

| | 1 | 2 | 3 | 4 | 5 | 6 | 7 | 8 | 9 | 10 | Don't know |
|---|---|---|---|---|---|---|---|---|---|---|---|
| GEI 4.1.1 Have representation in government, training institutions, civil society, nongovernmental and faith-based organizations, professional associations, the private sector, etc. | ○ | ○ | ○ | ○ | ○ | ○ | ○ | ○ | ○ | ○ | ○ |
| GEI 4.1.2 Meet regularly, report and recommend policy for senior management within the appropriate ministries | ○ | ○ | ○ | ○ | ○ | ○ | ○ | ○ | ○ | ○ | ○ |
| GEI 4.1.3 Make an impact on GEI within the country | ○ | ○ | ○ | ○ | ○ | ○ | ○ | ○ | ○ | ○ | ○ |

* GEI 4.2 Education/Research Strategy: Extent to which national planning documents include a comprehensive strategy/approach on improving GEI, shared among partners.

| | 1 | 2 | 3 | 4 | 5 | 6 | 7 | 8 | 9 | 10 | Don't know |
|---|---|---|---|---|---|---|---|---|---|---|---|
| 1-Lowest -> Highest-10 | ○ | ○ | ○ | ○ | ○ | ○ | ○ | ○ | ○ | ○ | ○ |

137

* GEI 4.3 Governance, Financial and Development Data Gathering Partners:

| | 1 | 2 | 3 | 4 | 5 | 6 | 7 | 8 | 9 | 10 | Don't know |
|---|---|---|---|---|---|---|---|---|---|---|---|
| GEI 4.3.1 Extent to which research is undertaken by government agencies. (This may include statistical agencies/bureaus and/or ministries of economics/finances, education, health, gender, etc.) | ○ | ○ | ○ | ○ | ○ | ○ | ○ | ○ | ○ | ○ | ○ |
| GEI 4.3.2 Extent to which research is undertaken by public research institutions. | ○ | ○ | ○ | ○ | ○ | ○ | ○ | ○ | ○ | ○ | ○ |
| GEI 4.3.3 Extent to which research is undertaken by independent researchers. | ○ | ○ | ○ | ○ | ○ | ○ | ○ | ○ | ○ | ○ | ○ |

* GEI 4.4 Governance, Financial and Development Topical Data Gathering:

| | 1 | 2 | 3 | 4 | 5 | 6 | 7 | 8 | 9 | 10 | Don't know |
|---|---|---|---|---|---|---|---|---|---|---|---|
| GEI 4.4.1 Extent to which safety and the rule of law is researched. | ○ | ○ | ○ | ○ | ○ | ○ | ○ | ○ | ○ | ○ | ○ |
| GEI 4.4.2 Extent to which political participation and human rights is measured. | ○ | ○ | ○ | ○ | ○ | ○ | ○ | ○ | ○ | ○ | ○ |
| GEI 4.4.3 Extent to which indicators of a sustainable economy are researched and disseminated to the community. | ○ | ○ | ○ | ○ | ○ | ○ | ○ | ○ | ○ | ○ | ○ |
| GEI 4.4.4 Extent to which human capital development (education, training, and employment, etc.) is researched. | ○ | ○ | ○ | ○ | ○ | ○ | ○ | ○ | ○ | ○ | ○ |

* GEI 4.5 Quality/Coverage of Data:

| | 1 | 2 | 3 | 4 | 5 | 6 | 7 | 8 | 9 | 10 | Don't know |
|---|---|---|---|---|---|---|---|---|---|---|---|
| GEI 4.5.1 Extent to which a routine statistical system provides reliable periodic information on governance indicators. | ○ | ○ | ○ | ○ | ○ | ○ | ○ | ○ | ○ | ○ | ○ |
| GEI 4.5.2 Extent to which the routine statistical system provides reliable periodic information on governance-related needs among populations in different communities. | ○ | ○ | ○ | ○ | ○ | ○ | ○ | ○ | ○ | ○ | ○ |
| GEI 4.5.3 Extent to which a routine statistical system provides reliable periodic information on economic indicators. | ○ | ○ | ○ | ○ | ○ | ○ | ○ | ○ | ○ | ○ | ○ |
| GEI 4.5.4 Extent to which evaluations of the monitoring and surveillance systems are conducted and applied to ensure the reliability of data. | ○ | ○ | ○ | ○ | ○ | ○ | ○ | ○ | ○ | ○ | ○ |

* GEI 4.6 Quality Research Institutions: Extent to which the country has the capacity to support and sustain research institutions that develop research/collect data to inform efforts to ensure good governance, stable economy and strong public institutions.

| | 1 | 2 | 3 | 4 | 5 | 6 | 7 | 8 | 9 | 10 | Don't know |
|---|---|---|---|---|---|---|---|---|---|---|---|
| 1-Lowest -> Highest-10 | ○ | ○ | ○ | ○ | ○ | ○ | ○ | ○ | ○ | ○ | ○ |

* GEI 4.7 Evaluation: Extent to which program statistics, surveys, and small studies are used by specialized staff to report on good governance and financial institutions.

| | 1 | 2 | 3 | 4 | 5 | 6 | 7 | 8 | 9 | 10 | Don't know |
|---|---|---|---|---|---|---|---|---|---|---|---|
| 1-Lowest -> Highest-10 | ○ | ○ | ○ | ○ | ○ | ○ | ○ | ○ | ○ | ○ | ○ |

* GEI 4.8 Management's Use of Evaluation Findings: Extent to which local-level program managers use research and evaluation findings to improve programming around the rule of law, stabilizing the economy and strengthening institutions in ways suggested by findings.

| | 1 | 2 | 3 | 4 | 5 | 6 | 7 | 8 | 9 | 10 | Don't know |
|---|---|---|---|---|---|---|---|---|---|---|---|
| 1-Lowest -> Highest-10 | ◯ | ◯ | ◯ | ◯ | ◯ | ◯ | ◯ | ◯ | ◯ | ◯ | ◯ |

* GEI 4.10 Dissemination of Information to Other Implementing Bodies: Extent to which information is shared or spread across geographical areas and throughout various levels (national, regional, localities).

| | 1 | 2 | 3 | 4 | 5 | 6 | 7 | 8 | 9 | 10 | Don't know |
|---|---|---|---|---|---|---|---|---|---|---|---|
| 1-Lowest -> Highest-10 | ◯ | ◯ | ◯ | ◯ | ◯ | ◯ | ◯ | ◯ | ◯ | ◯ | ◯ |

GEI 4.11 Comments: Please use this section to provide any additional comments about the level of effort, challenges, or successes in research around GEI. If you marked “I don’t know” for any of the questions, also use this section to explain why you gave that rating.

JOHNS HOPKINS
BLOOMBERG SCHOOL
of PUBLIC HEALTH

Bill & Melinda Gates Institute for Population and Reproductive Health

## Demographic Dividend Effort Index Survey

### Dimension 5. Civil Society Organizations (CSOs)

* GEI 5.1 CSO Actor Power: Extent to which CSO actors are in strong positions to positively contribute to the rule of law.

| | 1 | 2 | 3 | 4 | 5 | 6 | 7 | 8 | 9 | 10 | Don't know |
|---|---|---|---|---|---|---|---|---|---|---|---|
| 1-Lowest -> Highest-10 | ○ | ○ | ○ | ○ | ○ | ○ | ○ | ○ | ○ | ○ | ○ |

* GEI 5.2 Budget Analysis as a CSO Tool: Extent to which CSOs utilize budget analysis as a tool to build an advocacy case for good governance, economy stability and stronger public institutions

| | 1 | 2 | 3 | 4 | 5 | 6 | 7 | 8 | 9 | 10 | Don't know |
|---|---|---|---|---|---|---|---|---|---|---|---|
| 1-Lowest -> Highest-10 | ○ | ○ | ○ | ○ | ○ | ○ | ○ | ○ | ○ | ○ | ○ |

* GEI 5.3 Community-Based Service Delivery Support: Extent to which CSOs participate in bringing governance-related information and services to the communities where people live.

| | 1 | 2 | 3 | 4 | 5 | 6 | 7 | 8 | 9 | 10 | Don't know |
|---|---|---|---|---|---|---|---|---|---|---|---|
| 1-Lowest -> Highest-10 | ○ | ○ | ○ | ○ | ○ | ○ | ○ | ○ | ○ | ○ | ○ |

* GEI 5.4 Human Rights and Quality of Implementation: Extent to which CSOs advocate for and support rights-based approaches to governance to ensure policy and program beneficiaries experience progress on their terms.

| | 1 | 2 | 3 | 4 | 5 | 6 | 7 | 8 | 9 | 10 | Don't know |
|---|---|---|---|---|---|---|---|---|---|---|---|
| 1-Lowest -> Highest-10 | ○ | ○ | ○ | ○ | ○ | ○ | ○ | ○ | ○ | ○ | ○ |

* GEI 5.5 Youth: Extent to which CSOs support youth to participate in education, training, employment and leadership.

| | 1 | 2 | 3 | 4 | 5 | 6 | 7 | 8 | 9 | 10 | Don't know |
|---|---|---|---|---|---|---|---|---|---|---|---|
| 1-Lowest -> Highest-10 | ○ | ○ | ○ | ○ | ○ | ○ | ○ | ○ | ○ | ○ | ○ |

* GEI 5.6 Programs for Women: Extent to which CSOs ensure GEI policies support women and girls to participate in leadership.

| | 1 | 2 | 3 | 4 | 5 | 6 | 7 | 8 | 9 | 10 | Don't know |
|---|---|---|---|---|---|---|---|---|---|---|---|
| 1-Lowest -> Highest-10 | ○ | ○ | ○ | ○ | ○ | ○ | ○ | ○ | ○ | ○ | ○ |

* GEI 5.7 Advocacy/Accountability

| | 1 | 2 | 3 | 4 | 5 | 6 | 7 | 8 | 9 | 10 | Don't know |
|---|---|---|---|---|---|---|---|---|---|---|---|
| GEI 5.7.1 Extent to which CSOs support the participation of the community in governance or enforcement of the rule of law. | ○ | ○ | ○ | ○ | ○ | ○ | ○ | ○ | ○ | ○ | ○ |
| GEI 5.7.2 Extent to which the media support the participation of the community in governance or enforcement of the rule of law. | ○ | ○ | ○ | ○ | ○ | ○ | ○ | ○ | ○ | ○ | ○ |

* GEI 5.8 CSO-Led Assessment and Monitoring: Extent to which CSOs assess, monitor and report on the effectiveness of policies and government accountability mechanisms and structures.

| | 1 | 2 | 3 | 4 | 5 | 6 | 7 | 8 | 9 | 10 | Don't know |
|---|---|---|---|---|---|---|---|---|---|---|---|
| 1-Lowest -> Highest-10 | ○ | ○ | ○ | ○ | ○ | ○ | ○ | ○ | ○ | ○ | ○ |

* GEI 5.9 Forming Partnerships between CSOs: Extent to which CSOs have formed national alliances and regional partnerships to strengthen their positioning and potentially improve leadership strength and funding mechanisms for good governance and institutional strengthening related work.

| | 1 | 2 | 3 | 4 | 5 | 6 | 7 | 8 | 9 | 10 | Don't know |
|---|---|---|---|---|---|---|---|---|---|---|---|
| 1-Lowest -> Highest-10 | ○ | ○ | ○ | ○ | ○ | ○ | ○ | ○ | ○ | ○ | ○ |

GEI 5.10 Comments: Please use this section to provide any additional comments about the level of effort, challenges, or successes around the role of the CSOs and media in GEI. If you marked “I don’t know” for any of the questions, also use this section to explain why you gave that rating

JOHNS HOPKINS
BLOOMBERG SCHOOL
of PUBLIC HEALTH

Bill & Melinda Gates Institute for Population and Reproductive Health

## Demographic Dividend Effort Index Survey

### Module: Sectoral Resilience and Sustainability

**Considering the health and socio-economic impact of the COVID-19 and the likely impact on the DD progress, this DD effort index has integrated questions to assess the resilience and sustainability of systems in key sectors of the DD framework. The items included in the COVID-19 related questionnaire below follow the key dimensions of resilient systems and provide critical information on the potential of an effective response to emerging infectious disease threats or other public health emergencies.**

**Please score each item from 1 to 10, where 1 represents the lowest score (poor state/capability) and 10 represents the highest score (great state/capability).**

* Physical: facilities, equipment, system states, and capabilities.

| | 1 | 2 | 3 | 4 | 5 | 6 | 7 | 8 | 9 | 10 | Don't know |
|---|---|---|---|---|---|---|---|---|---|---|---|
| GEI-M1 - Plan/Prepare - State and capability of equipment, personnel and GEI sector system structure prior to the crisis event. (1 = poor state/capability; 10 = great state/capability) | ○ | ○ | ○ | ○ | ○ | ○ | ○ | ○ | ○ | ○ | ○ |
| GEI-M2 - Absorb - Extent to which COVID-19 was recognized within the GEI sector system and the performance of the system was able to maintain functionality. | ○ | ○ | ○ | ○ | ○ | ○ | ○ | ○ | ○ | ○ | ○ |
| GEI-M3 - Absorb - Integration: The level of integration of services and institutional collaborations to ensure continuity of access to essential services during the COVID-19 crisis. | ○ | ○ | ○ | ○ | ○ | ○ | ○ | ○ | ○ | ○ | ○ |
| GEI-M4 - Recover - Extent to which the Government and Economic Institutions have been able to induce change to recover the pre-COVID-19 level of functionality. | ○ | ○ | ○ | ○ | ○ | ○ | ○ | ○ | ○ | ○ | ○ |
| GEI-M5 - Recover - Financing and Donor Management: National bank and donor support to ensure the availability of cash during the COVID-19 crisis | ○ | ○ | ○ | ○ | ○ | ○ | ○ | ○ | ○ | ○ | ○ |
| GEI-M6 - Adapt - Extent to which changes have been made to improve the resilience of the GEI sector system and infrastructure. | ○ | ○ | ○ | ○ | ○ | ○ | ○ | ○ | ○ | ○ | ○ |

* Information: creation, manipulation, storage, and utilization of data.

| | 1 | 2 | 3 | 4 | 5 | 6 | 7 | 8 | 9 | 10 | Don't know |
|---|---|---|---|---|---|---|---|---|---|---|---|
| GEI-M7 - Plan/Prepare - Extent to which GEI-related data was sufficiently prepared, presented, analyzed and stored prior to the crisis event. | ○ | ○ | ○ | ○ | ○ | ○ | ○ | ○ | ○ | ○ | ○ |
| GEI-M8 - Absorb - Awareness: Level of awareness about the threats caused by COVID-19 in the central government. | ○ | ○ | ○ | ○ | ○ | ○ | ○ | ○ | ○ | ○ | ○ |
| GEI-M9 - Recover - Extent to which GEI data has been used to track the progress of recovery and to anticipate recovery scenarios. | ○ | ○ | ○ | ○ | ○ | ○ | ○ | ○ | ○ | ○ | ○ |
| GEI-M10 - Adapt - Extent to which the GEI sector system is creating and improving GEI data storage and real-time use protocols. | ○ | ○ | ○ | ○ | ○ | ○ | ○ | ○ | ○ | ○ | ○ |

* Cognitive: understanding, mental models, preconceptions, biases, and values.

| | 1 | 2 | 3 | 4 | 5 | 6 | 7 | 8 | 9 | 10 | Don't know |
|---|---|---|---|---|---|---|---|---|---|---|---|
| GEI-M11 - Plan/Prepare - Extent to which the GEI sector system design and operation decisions were prepared in anticipation of crisis events. | ○ | ○ | ○ | ○ | ○ | ○ | ○ | ○ | ○ | ○ | ○ |
| GEI-M12 - Absorb - Extent to which the Government and Economic Institutions are responding with sufficient contingency protocols and proactive crisis management. | ○ | ○ | ○ | ○ | ○ | ○ | ○ | ○ | ○ | ○ | ○ |

| | 1 | 2 | 3 | 4 | 5 | 6 | 7 | 8 | 9 | 10 | Don't know |
|---|---|---|---|---|---|---|---|---|---|---|---|
| GEI-M13 - Absorb - Accountability: Level of effort to ensure optimal accountability for the resources allocated to support governance as part of the response to the COVID-19. | ○ | ○ | ○ | ○ | ○ | ○ | ○ | ○ | ○ | ○ | ○ |
| GEI-M14 - Recover - Extent to which decisions are oriented towards recovery, which employ evidence-based communication to community members to promote safe behavior. | ○ | ○ | ○ | ○ | ○ | ○ | ○ | ○ | ○ | ○ | ○ |
| GEI-M15 - Recover - Awareness: Extent to which the government is building confidence among the public though communication and dialogue for evidence-based policymaking and actions. | ○ | ○ | ○ | ○ | ○ | ○ | ○ | ○ | ○ | ○ | ○ |
| GEI-M16 - Adapt - Extent to which the GEI sector system is designing new system configurations, objectives and decision criteria. | ○ | ○ | ○ | ○ | ○ | ○ | ○ | ○ | ○ | ○ | ○ |
| GEI-M17 - Adapt - Adaptive: Level of adaptation of financial and social service systems to ensure easy access via technology-based platforms during the COVID-19. | ○ | ○ | ○ | ○ | ○ | ○ | ○ | ○ | ○ | ○ | ○ |

* Social: interaction, collaboration, and self-synchronization between individuals, entities and institutions.

| | 1 | 2 | 3 | 4 | 5 | 6 | 7 | 8 | 9 | 10 | Don't know |
|---|---|---|---|---|---|---|---|---|---|---|---|
| GEI-M18 - Plan/Prepare - Extent to which training on outbreak/crisis management was conducted through, and which leveraged, social networks, social capital and institutional and cultural norms prior to the crisis event. | ○ | ○ | ○ | ○ | ○ | ○ | ○ | ○ | ○ | ○ | ○ |
| GEI-M19 - Absorb - Extent to which personnel and social institutions have been resourceful and accessible for the outbreak/crisis response. | ○ | ○ | ○ | ○ | ○ | ○ | ○ | ○ | ○ | ○ | ○ |
| GEI-M20 - Absorb - Self-Regulating: Extent to which the national leadership had the authority to make sectoral changes in a timely manner through flexible infrastructure. | ○ | ○ | ○ | ○ | ○ | ○ | ○ | ○ | ○ | ○ | ○ |
| GEI-M21 - Recover - Extent to which the GEI sector system is engaging with team and knowledge sharing to enhance systematic recovery. | ○ | ○ | ○ | ○ | ○ | ○ | ○ | ○ | ○ | ○ | ○ |
| GEI-M22 - Recover - Diversity: The level of engagement of multidisciplinary taskforces to mitigate the effect of COVID-19 at the national and subnational levels. | ○ | ○ | ○ | ○ | ○ | ○ | ○ | ○ | ○ | ○ | ○ |
| GEI-M23 - Adapt - Leadership and Management: Stewardship and management demonstrated by Government in response to the COVID-19 at the national level. | ○ | ○ | ○ | ○ | ○ | ○ | ○ | ○ | ○ | ○ | ○ |

* GEI M-24 Timeline: Extent of timeliness of the Government reaction - communication and implementation of measures - to mitigate the immediate and longterm impact of COVID-19.
*Note: This refers to the entirety of the national reaction - not the reaction that is specific to your sector.*

| | 1 | 2 | 3 | 4 | 5 | 6 | 7 | 8 | 9 | 10 | Don't know |
|---|---|---|---|---|---|---|---|---|---|---|---|
| 1-Lowest -> Highest-10 | ○ | ○ | ○ | ○ | ○ | ○ | ○ | ○ | ○ | ○ | ○ |

GEI M-25 Comments: Please use this section to provide any additional comments about the resilience of the national GEI systems based on your experience during the COVID-19 pandemics.If you marked “I don’t know” for any of the questions, also use this section to explain why you gave that rating.
