# Supplementary material for: Development of the Demographic Dividend Effort Index, a novel tool to measure existing efforts to create a favourable environment to harness a demographic dividend: results from an experts’ survey from six sub-Saharan African countries
Source: BMJ Open. 2023 Mar 21;13(3):e059937. doi: 10.1136/bmjopen-2021-059937 (PMC10040031; doi:10.1136/bmjopen-2021-059937)
Supplement: Supplementary data [file bmjopen-2021-059937supp002.pdf]

JOHNS HOPKINS
BLOOMBERG SCHOOL of PUBLIC HEALTH

Bill & Melinda Gates Institute for Population and Reproductive Health

Enquête de l'Indice des efforts pour le dividende démographique

Introduction

Les programmes de développement ont besoin d'une mesure standard qui quantifie la portée des efforts nationaux en termes de politiques et programmes mis en œuvre pour cultiver, réaliser et exploiter le dividende démographique (DD). En dépit du progrès remarquable accompli par plusieurs pays à travers l'intégration des principes du DD dans leurs politiques et plans de développement, des outils d'évaluation de la performance et des plateformes documentant les leçons et meilleures pratiques pour bénéficier du DD font toujours défaut.

Ainsi, l'objectif de l'Indice des efforts pour le dividende démographique (IEDD) est de fournir une mesure standard qui permettra aux experts et membres de la société civile de quantifier la nature et la puissance des efforts pour le DD dans les pays, autant dans les sphères publiques que privées, à travers différentes dimensions de la société. En mesurant ces efforts, nous documenterons les intrants indépendamment des extrants, dont les revenus par habitant. Cela nous permettra d'apprécier la relation entre les intrants pris en compte dans l'élaboration des politiques de DD et les extrants du DD.

Cet IEDD est guidé par une question centrale : Dans quelle mesure les efforts politiques et programmatiques existants ont-ils permis un environnement favorable à la culture, la réalisation et l'exploitation des bénéfices d'un dividende démographique ? Pour répondre à cette question, l'IEDD résumera les perceptions d'informateurs clés dans six secteurs identifiés dans la littérature scientifique comme produisant un environnement favorable à l'exploitation du DD. Ces secteurs cadres du DD sont : 1) Planification familiale, 2) Santé maternelle et infantile, 3) Education, 4) Autonomisation des femmes, 5) Marché du travail, et 6) Bonne gouvernance, solidité institutionnelle et stabilité économique. Chaque secteur est mesuré via un questionnaire qui lui est propre et est évalué à travers cinq domaines identifiés dans la littérature scientifique, à savoir : la politique/ prise de décision, les services ou programmes, le plaidoyer, la recherche, et la société civile. Chacun des 6 questionnaires, remplis par 10 observateurs experts par pays, notera chaque élément sur une échelle de 1 à 10, sauf indication contraire.

Étant donné l'impact sanitaire et socioéconomique de la pandémie de COVID-19, et sa relation étroite avec les politiques qui permettent un environnement politique favorable au DD, cet Indice des efforts de DD intègre un module pour évaluer la résilience et la durabilité des systèmes dans chaque secteur. La notation suit les principes clés des systèmes de santé résilients et durables pouvant répondre efficacement aux menaces de maladies infectieuses émergentes et autres crises de santé publiques.

Rusatira JC, *et al. BMJ Open* 2023; 13:e059937. doi: 10.1136/bmjopen-2021-059937

L'IEDD permet de visualiser les efforts nationaux, tout en évaluant la mesure dans laquelle ces efforts soutiennent les objectifs du pays pour cultiver, réaliser et exploiter le dividende démographique. L'IEDD servira d'outil pour mesurer la performance et suivre le progrès, ce qui offrira une évaluation opportune des succès et des marges de progression, des meilleures pratiques et des améliorations possibles.

JOHNS HOPKINS
BLOOMBERG SCHOOL
of PUBLIC HEALTH

Bill & Melinda Gates Institute for Population and Reproductive Health

Enquête de l'Indice des efforts pour le dividende démographique

Formulaire de consentement

Merci d'avoir pris le temps de répondre à ce questionnaire, particulièrement en cette période sans précédent.

L'Institut Gates développe actuellement un Indice des efforts pour le dividende démographique (IEDD) afin d'établir une mesure standard qui permettra d'évaluer le statut existant des politiques, services et programmes nationaux de planification familiale, santé maternelle et infantile, et dans d'autres secteurs sociaux (ex : emploi et éducation), ainsi qu'en matière de gouvernance pour permettre un environnement politique favorable au dividende démographique (DD). En mesurant ces efforts, cet outil documentera les intrants indépendamment des extrants, dont les revenus par habitant. Ceci permettra d'évaluer la relation entre les intrants pris en compte dans l'élaboration des politiques de DD et les extrants du DD observés et communément utilisés pour évaluer le progrès.

Nous aimerions vous poser quelques questions en raison de votre expertise dans ce domaine. Les informations collectées seront confidentielles, et nous ne collecterons ni votre nom, ni aucun autre détail permettant de vous identifier. Ce questionnaire devrait vous prendre environ 45-60 min.

Nous vous serions très reconnaissants si vous pouviez participer à cette enquête. Les informations générées par cette interview seront utilisées par l'Institut Gates et la communauté de pratique sur le DD dans votre pays pour évaluer les efforts nationaux, identifier les domaines de réussites qui doivent continuer d'être soutenus et les domaines nécessitant d'être renforcés. Les chercheurs pourront aussi utiliser les données collectées à des fins d'analyse comparative entre les pays.

Vous êtes invité(e) à participer à cette enquête. Sachez que la participation à cette enquête est entièrement volontaire. En participant, vous ne percevrez aucun bénéfice direct, mais il y a des bénéfices sociétaux à permettre aux gouvernements et aux partenaires du développement de renforcer leurs efforts pour cultiver un environnement favorable au DD.

Vous pouvez refuser de répondre à toute question. Si vous avez des inquiétudes ou problèmes concernant cette étude, vous pouvez contacter son coordinateur à **jcrusatira@jhu.edu** ou au **+14436510824** par téléphone ou WhatsApp.

* Veuillez cocher "Oui" si vous souhaitez procéder et répondre à l'enquête.

- ◯ Oui
- ◯ Non

3

JOHNS HOPKINS
BLOOMBERG SCHOOL
of PUBLIC HEALTH

Bill & Melinda Gates Institute for Population and Reproductive Health

Enquête de l’Indice des efforts pour le dividende démographique

Avis important

Veuillez ne pas sélectionner "Page précédente" au milieu d'une page de questions car la page sur laquelle vous travaillez ne sera pas sauvegardée tant que vous n'aurez pas finalisé ladite page et sélectionner "Page suivante". Si vous sélectionnez "Page précédente" sans avoir terminé, vos réponses sur la page inachevée seront perdus.

Sachez que votre enquête est terminée lorsque vous lisez "Merci d'avoir rempli l'indice d'effort du dividende démographique". Merci !

Rusatira JC, *et al. BMJ Open* 2023; 13:e059937. doi: 10.1136/bmjopen-2021-059937

JOHNS HOPKINS
BLOOMBERG SCHOOL
of PUBLIC HEALTH

Bill & Melinda Gates Institute for Population and Reproductive Health

Enquête de l’Indice des efforts pour le dividende démographique

Pays et expertise de l’enquêté(e)

* Veuillez sélectionner le pays duquel vous répondez.

- Kenya
- Senegal
- Nigeria
- Rwanda
- Tanzania
- Ethiopia

JOHNS HOPKINS
BLOOMBERG SCHOOL
of PUBLIC HEALTH

Bill & Melinda Gates Institute for Population and Reproductive Health

Enquête de l’Indice des efforts pour le dividende démographique

Level of expertise

* At which level of Government is your expertise (If your expertise is at the national level please select "National" otherwise select a county?

JOHNS HOPKINS
BLOOMBERG SCHOOL
of PUBLIC HEALTH

Bill & Melinda Gates Institute for Population and Reproductive Health

Enquête de l’Indice des efforts pour le dividende démographique

Niveau d'expertise

* A quel niveau de gouvernement votre expertise se situe-t-elle (Si votre expertise se situe au niveau national, veuillez sélectionner "National", sinon sélectionnez une région ?

JOHNS HOPKINS
BLOOMBERG SCHOOL
of PUBLIC HEALTH

Bill & Melinda Gates Institute for Population and Reproductive Health

Enquête de l'Indice des efforts pour le dividende démographique

Expertise

* Veuillez sélectionner le type d'organisation dans laquelle vous avez le plus travaillé au cours des cinq dernières années. Si votre type d'organisation n'est pas référencé, veuillez préciser de quel type d'organisation il s'agit.

- Secteur public
- Secteur privé
- Organisation non gouvernementale
- Université/ Institut de recherche
- Autre (veuillez préciser)
- Other (please specify)

* Dans quel domaine avez-vous le plus travaillé au cours des cinq dernières années ?

- Politique/ Prise de décision
- Services ou programmes
- Plaidoyer
- Recherche
- Société Civile

* Dans quel secteur avez-vous le plus travaillé dans les cinq dernières années ?

- Planification familiale
- Santé maternelle et infantile
- Education
- Autonomisation des femmes
- Marché du travail
- Gouvernance et institutions économiques

JOHNS HOPKINS
BLOOMBERG SCHOOL
of PUBLIC HEALTH

Bill & Melinda Gates Institute for Population and Reproductive Health

Enquête de l'Indice des efforts pour le dividende démographique

Questionnaire sur la planification familiale (PF)

Répondez aux questions suivantes en fonction de votre expérience/ expertise dans ce secteur spécifique.

Afin d'obtenir une représentation synthétique des efforts nationaux pour le dividende démographique, notez les éléments suivants sur une échelle de 1 à 10, 1 étant le score le plus faible (efforts très faibles ou quasi-inexistants) et 10 le plus élevé (efforts robustes). Le cas échéant, si une politique ou activité n'existe pas, répondez 0.

Donnez un score à chaque élément. Toutes les réponses seront enregistrées au format illustré ci-dessous :

Effort le plus faible	1	2	3	4	5	6	7	8	9	10	Effort le plus élevé	Je ne sais pas

JOHNS HOPKINS
BLOOMBERG SCHOOL of PUBLIC HEALTH

Bill & Melinda Gates Institute for Population and Reproductive Health

Enquête de l'Indice des efforts pour le dividende démographique

Domaine 1. Politique/ Prise de décision

* FP 1.1 La proéminence de la planification familiale (PF) : Mesure dans laquelle il existe un bureau ou poste permanent et bien placé au ministère compétent qui développe et surveille les politiques et stratégies du secteur, et dispose d'effectifs adéquats et de personnel qualifié.

	1	2	3	4	5	6	7	8	9	10	Je ne sais pas
1-Plus faible -> Plus élevé-10	○	○	○	○	○	○	○	○	○	○	○

* FP 1.2 La politique de PF et réduction de la fécondité : Mesure dans laquelle la politique gouvernementale met l'accent sur la PF pour promouvoir la taille des familles en lien avec les ressources disponibles ou des raisons démographiques plutôt que des raisons sanitaires.

	1	2	3	4	5	6	7	8	9	10	Je ne sais pas
1-Plus faible -> Plus élevé-10	○	○	○	○	○	○	○	○	○	○	○

* FP 1.3 Le plan national stratégique de PF : Mesure dans laquelle il existe un plan national actualisé, exhaustif et stratégique pour la PF, définissant les politiques, lois et régulations pour l'offre de PF. (Évaluez la mesure dans laquelle le plan a une vision, comprend des stratégies sur plusieurs années, des calendriers annuels, et des plans de mise en œuvre, tout en étant fondés sur des données probantes, avec des indicateurs de progrès, et tenant compte du marché de la PF).

	1	2	3	4	5	6	7	8	9	10	Je ne sais pas
1-Plus faible -> Plus élevé-10	○	○	○	○	○	○	○	○	○	○	○

* FP 1.4 La politique sur l'âge légal du mariage : Mesure dans laquelle l'âge légal du mariage pour les filles, établi à 18 ans ou plus, est appliqué.

	1	2	3	4	5	6	7	8	9	10	Je ne sais pas
1-Plus faible -> Plus élevé-10	○	○	○	○	○	○	○	○	○	○	○

* FP 1.5 La prise de décision fondée sur des données probantes : Mesure dans laquelle le ministère compétent soutient et utilise activement et régulièrement les données clés et rapports de PF pour élaborer ses politiques et ses plans.

	1	2	3	4	5	6	7	8	9	10	Je ne sais pas
1-Plus faible -> Plus élevé-10	○	○	○	○	○	○	○	○	○	○	○

* FP 1.6 Le suivi et évaluation des politiques/plans/stratégies nationaux : Mesure dans laquelle le plan national de PF établit une stratégie de suivi et évaluation complète pour suivre et mesurer les accomplissements prévus dans le plan. (Évaluez si la stratégie de suivi et évaluation inclut des buts et objectifs, un cadre de résultats, des indicateurs mesurables [ex : "SMART": spécifiques, mesurables, atteignables, réalistes et temporellement définis], et un plan/calendrier exploitable de mise en œuvre.)

	1	2	3	4	5	6	7	8	9	10	Je ne sais pas
1-Plus faible -> Plus élevé-10	○	○	○	○	○	○	○	○	○	○	○

* FP 1.7 L'inclusion des acteurs non gouvernementaux et intersectoriels au développement de politique/stratégie : Mesure dans laquelle les agents non-gouvernementaux, les associations (ex : de la société civile), les travailleurs de la santé, les parties prenantes (ex : organisations confessionnelles, à but non lucratif et à but lucratif) et les acteurs d'autres secteurs que celui de la PF sont inclus dans le plan national de PF et/ou autres documents nationaux établissant les stratégies et systèmes du pays pour planifier, gérer et développer la PF.

	1	2	3	4	5	6	7	8	9	10	Je ne sais pas
1-Plus faible -> Plus élevé-10	○	○	○	○	○	○	○	○	○	○	○

* FP 1.8 Le financement de la PF : Mesure dans laquelle le gouvernement a établi et appliqué des directives pour la budgétisation et le paiement de la PF en termes d'offre et de demande (soit les financements intérieurs vs. internationaux, et leur continuité/durabilité, coût des produits contraceptifs, coût de la prestation de services et du transport, etc.).

(Évaluez si les directives et le budget tiennent compte de la durée de vie des coûts des soins et services de PF ; des tarifications variables par méthode de PF ; des coûts de livraison et prestation de services par spécialité, lieu et risque ; des coûts et profits des plans/politiques d'assurance ; du coût financier de fournir de la PF en comparaison au coût financier de ne pas le faire, etc.).

	1	2	3	4	5	6	7	8	9	10	Je ne sais pas
1-Plus faible -> Plus élevé-10	○	○	○	○	○	○	○	○	○	○	○

* FP 1.9 Le pourcentage du financement intérieur (domestique) pour le budget de la PF : Mesure dans laquelle le budget total de la planification familiale/population est dérivé de sources domestiques (ex : 1 pour 10%, 5 pour 50%, 10 pour 100%).

	1	2	3	4	5	6	7	8	9	10	Je ne sais pas
1-Plus faible -> Plus élevé-10	○	○	○	○	○	○	○	○	○	○	○

* FP 1.10 L'infrastructure/ capacité des Technologies de l'Information et la Communication (TIC) : Mesure dans laquelle il existe des éléments d'infrastructure (ex : ordinateurs, serveurs, imprimantes, réseau local, connexion internet, alimentation électrique fiable) ainsi que des ressources humaines (ex : pour la maintenance et le soutien) au niveau national et infranational permettant de soutenir des systèmes d'informations fonctionnels sur la PF.

(Pour cet élément, évaluez si le pays conduit au moins des évaluations annuelles de ses systèmes informatiques ; a des programmes de formation couvrant la saisie et l'utilisation des données, l'administration et le développement de logiciels ; et forme continuellement le personnel des institutions clés [ex : Ministère de la Santé, Ministère de l'Éducation, les agences régulatrices et les institutions de formation]).

	1	2	3	4	5	6	7	8	9	10	Je ne sais pas
1-Plus faible -> Plus élevé-10	○	○	○	○	○	○	○	○	○	○	○

* FP 1.11 Les lois et régulations sur l'importation :

	1	2	3	4	5	6	7	8	9	10	Je ne sais pas
FP 1.11.1 Mesure dans laquelle les lois et régulations sur l'importation facilitent l'importation des commodités et produits de PF.	○	○	○	○	○	○	○	○	○	○	○
FP 1.11.2 Mesure dans laquelle les contraceptifs sont fabriqués localement.	○	○	○	○	○	○	○	○	○	○	○

* FP 1.12 La publicité des contraceptifs autorisés : Mesure dans laquelle la publicité des contraceptifs dans les médias de masse n'est soumise à aucune restriction.

	1	2	3	4	5	6	7	8	9	10	Je ne sais pas
1-Plus faible -> Plus élevé-10	○	○	○	○	○	○	○	○	○	○	○

* FP 1.13 La présence de politiques de PF favorisant les populations pauvres : Mesure dans laquelle les politiques ciblent spécifiquement les populations pauvres en milieu rural et urbain.

	1	2	3	4	5	6	7	8	9	10	Je ne sais pas
1-Plus faible -> Plus élevé-10	○	○	○	○	○	○	○	○	○	○	○

* FP 1.14 Le soutien politique à la PF : Mesure dans laquelle les élus dans le pays priorisent les besoins de PF pour renforcer les contributions de la PF au DD en adoptant des lois et régulations, et en appuyant des actions et politiques visant à améliorer l'utilisation et l'adhérence à la PF.

	1	2	3	4	5	6	7	8	9	10	Je ne sais pas
1-Plus faible -> Plus élevé-10	○	○	○	○	○	○	○	○	○	○	○

FP 1.15 Commentaires : Veuillez utiliser cette section pour tout commentaire additionnel sur le niveau d'effort, les difficultés et les succès rencontrés dans le pays autour des politiques/ prise de décision pour la PF. Si vous avez répondu « Je ne sais pas » à l'une des questions, utilisez aussi cette section pour expliquer pourquoi.

JOHNS HOPKINS
BLOOMBERG SCHOOL
of PUBLIC HEALTH

Bill & Melinda Gates Institute for Population and Reproductive Health

Enquête de l'Indice des efforts pour le dividende démographique

Domaine 2. Services ou programmes

* FP 2.1 La structure administrative : Mesure dans laquelle la structure administrative et le personnel au niveau national, provincial et des comtés est suffisant pour mettre en œuvre le programme de planification familiale (PF).

	1	2	3	4	5	6	7	8	9	10	Je ne sais pas
1-Plus faible -> Plus élevé-10	○	○	○	○	○	○	○	○	○	○	○

* FP 2.2 Le niveau des responsables du programme : Niveau d'ancienneté relatif du directeur du programme national de planification familiale et mesure dans laquelle le supérieur hiérarchique direct de ce directeur est haut placé au gouvernement.

	1	2	3	4	5	6	7	8	9	10	Je ne sais pas
1-Plus faible -> Plus élevé-10	○	○	○	○	○	○	○	○	○	○	○

* FP. 2.3 La distribution à base communautaire : Mesure dans laquelle les zones du pays non desservies par les cliniques et autres sites de prestation de santé sont couvertes par des programmes de distribution à base communautaire de produits contraceptifs (particulièrement les zones rurales).

	1	2	3	4	5	6	7	8	9	10	Je ne sais pas
1-Plus faible -> Plus élevé-10	○	○	○	○	○	○	○	○	○	○	○

* FP 2.4 Le programme de PF postpartum (PFPP) : Mesure dans laquelle toutes les femmes en postpartum reçoivent des services PFPP.

	1	2	3	4	5	6	7	8	9	10	Je ne sais pas
1-Plus faible -> Plus élevé-10	○	○	○	○	○	○	○	○	○	○	○

* FP 2.5 Les agents travaillant à domicile : Mesure de la couverture de la population par les agents dont la tâche principale est de rendre visite aux femmes (en milieu rural) chez elles pour discuter de planification familiale et de santé maternelle et infantile.

	1	2	3	4	5	6	7	8	9	10	Je ne sais pas
1-Plus faible -> Plus élevé-10	○	○	○	○	○	○	○	○	○	○	○

* FP 2.6 La disponibilité et l'accessibilité des commodités/produits et services de PF (première partie) : Mesure dans laquelle l'ensemble de la population a facilement et directement accès à (1-le plus faible -> le plus élevé-10):

	1	2	3	4	5	6	7	8	9	10	Je ne sais pas
FP 2.6.1 DIU	○	○	○	○	○	○	○	○	○	○	○
FP 2.6.2 Pilule	○	○	○	○	○	○	○	○	○	○	○
FP 2.6.3 Injectable	○	○	○	○	○	○	○	○	○	○	○
FP 2.6.4 Implant	○	○	○	○	○	○	○	○	○	○	○
FP 2.6.5 Stérilisation féminine	○	○	○	○	○	○	○	○	○	○	○
FP 2.6.6 Stérilisation masculine	○	○	○	○	○	○	○	○	○	○	○
FP 2.6.7 Préservatif masculin	○	○	○	○	○	○	○	○	○	○	○
FP 2.6.8 Contraception d'urgence	○	○	○	○	○	○	○	○	○	○	○
FP 2.6.9 Avortement/ régulation de la menstruation (légal ou non)	○	○	○	○	○	○	○	○	○	○	○
FP 2.6.10 Autres (ex : préservatif féminin, mousse, collier du cycle, etc.)	○	○	○	○	○	○	○	○	○	○	○

* FP 2.7 La disponibilité et l'accessibilité des commodités/produits et services de PF, (deuxième partie) : Quel est le niveau de fonctionnalité du système d'approvisionnement (évite t-il les ruptures de stock ou l'interruption de l'approvisionnement, ou permet-il un flux fiable à tous les niveaux ?) pour les méthodes suivantes (1-le plus faible -> le plus élevé-10) :

	1	2	3	4	5	6	7	8	9	10	Don't know
FP 2.7.1 DIU	○	○	○	○	○	○	○	○	○	○	○
FP 2.7.2 Pilule	○	○	○	○	○	○	○	○	○	○	○
FP 2.7.3 Injectable	○	○	○	○	○	○	○	○	○	○	○
FP 2.7.4 Implant	○	○	○	○	○	○	○	○	○	○	○
FP 2.7.5 Préservatif masculin	○	○	○	○	○	○	○	○	○	○	○
FP 2.7.6 Contraception d'urgence	○	○	○	○	○	○	○	○	○	○	○
FP 2.7.7 Autres (ex : préservatif féminin, mousse, collier du cycle, etc.)	○	○	○	○	○	○	○	○	○	○	○

* FP 2.8 La disponibilité et l'accessibilité des commodités/produits et services de PF (troisième partie) : Dans quelle mesure le système d'approvisionnement fournit-il l'équipement et les produits médicaux nécessaires aux structures cliniques pour les méthodes suivantes (1-le plus faible -> le plus élevé-10) :

	1	2	3	4	5	6	7	8	9	10	Je ne sais pas
FP 2.8.1 Stérilisation féminine	○	○	○	○	○	○	○	○	○	○	○
FP 2.8.2 Stérilisation masculine	○	○	○	○	○	○	○	○	○	○	○
FP 2.8.3. DIU	○	○	○	○	○	○	○	○	○	○	○
FP 2.8.4 Implant	○	○	○	○	○	○	○	○	○	○	○

Other (please specify)

* FP 2.9 La qualité des services de PF : Veuillez noter la qualité générale des services de planification familiale. (Des services de haute qualité impliquent d'être orientés sur les besoins des clients, et de fournir du conseil, des informations complètes, un large choix de méthodes, et des procédures cliniques sûres).

	1	2	3	4	5	6	7	8	9	10	Je ne sais pas
1-Plus faible -> Plus élevé-10	○	○	○	○	○	○	○	○	○	○	○

* FP 2.10 Les avantages et les effets dissuasifs : Mesure dans laquelle des avantages monétaires ou d'autre type sont utilisés pour encourager l'adoption de la planification familiale.

	1	2	3	4	5	6	7	8	9	10	Je ne sais pas
1-Plus faible -> Plus élevé-10	○	○	○	○	○	○	○	○	○	○	○

* FP 2.11 Les programmes de PF pour les adolescents : Mesure dans laquelle les informations, services et activités sont focalisés sur les adolescents et encouragent ces derniers à accéder aux services contraceptifs.

	1	2	3	4	5	6	7	8	9	10	Je ne sais pas
1-Plus faible -> Plus élevé-10	○	○	○	○	○	○	○	○	○	○	○

* FP 2.12 Les programmes de formation : Mesure dans laquelle les programmes de formation, pour chaque catégorie de personnel dans le programme de planification familiale, sont adéquats pour transmettre au personnel les informations et compétences nécessaires à la conduite efficace de leur travail.

	1	2	3	4	5	6	7	8	9	10	Je ne sais pas
1-Plus faible -> Plus élevé-10	○	○	○	○	○	○	○	○	○	○	○

* FP 2.13 Les tâches assignées remplies par le personnel : Mesure dans laquelle toutes les catégories du personnel du programme de planification familiale (administratif, médical, paramédical, de terrain) remplissent leurs tâches assignées efficacement.

	1	2	3	4	5	6	7	8	9	10	Je ne sais pas
1-Plus faible -> Plus élevé-10	○	○	○	○	○	○	○	○	○	○	○

* FP 2.14 La logistique et le transport : Mesure dans laquelle les systèmes logistiques et de transport sont suffisants pour maintenir les stocks de produits contraceptifs et l'équipement disponible sur tous les sites de prestation de santé, à tout moment et à tous les niveaux.

	1	2	3	4	5	6	7	8	9	10	Je ne sais pas
1-Plus faible -> Plus élevé-10	○	○	○	○	○	○	○	○	○	○	○

* FP 2.15 Le système de supervision : Mesure dans laquelle le système de supervision à tous les niveaux est adéquat (visite de supervision régulières, suivies par des actions correctives ou d'appui/accompagnantes).

	1	2	3	4	5	6	7	8	9	10	Je ne sais pas
1-Plus faible -> Plus élevé-10	○	○	○	○	○	○	○	○	○	○	○

* FP 2.16 L'implication d'autres ministères et agences publiques : Mesure dans laquelle d'autres ministères, agences gouvernementales et parties prenantes non gouvernementales s'impliquent dans les activités de PF (ex : livraison de produits, prestation de services, information, éducation) ou autres activités pour la population.

	1	2	3	4	5	6	7	8	9	10	Je ne sais pas
1-Plus faible -> Plus élevé-10	○	○	○	○	○	○	○	○	○	○	○

* FP 2.17 L'implication d'agences et groupes du secteur privé : Mesure dans laquelle des agences ou groupes du secteur privé contribuent aux activités de PF ou autres activités pour la population.

	1	2	3	4	5	6	7	8	9	10	Je ne sais pas
1-Plus faible -> Plus élevé-10	○	○	○	○	○	○	○	○	○	○	○

* FP 2.18 Les médias de masse pour la communication, l'éducation et l'information : Fréquence et couverture des messages des médias de masse fournissant à la population des informations sur a planification familiale et les sites de prestation de services.

	1	2	3	4	5	6	7	8	9	10	Je ne sais pas
1-Plus faible -> Plus élevé-10	○	○	○	○	○	○	○	○	○	○	○

* FP 2.19 Les barrières normatives, culturelles et sociales : Mesure dans laquelle les programmes de planification familiale répondent aux barrières culturelles et sociales.

	1	2	3	4	5	6	7	8	9	10	Je ne sais pas
1-Plus faible -> Plus élevé-10	○	○	○	○	○	○	○	○	○	○	○

* FP 2.20 Les jeunes : Mesure dans laquelle le gouvernement soutient des initiatives de PF dirigées par les jeunes en matière de planification, mise en œuvre et évaluation des programmes de PF.

	1	2	3	4	5	6	7	8	9	10	Je ne sais pas
1-Plus faible -> Plus élevé-10	○	○	○	○	○	○	○	○	○	○	○

* FP 2.21 Les influences des efforts de PF (les forces influençant le programme de PF) : Les forces influençant le programme de PF peuvent soit renforcer, soit diminuer son efficacité, Cochez zéro (0) s'il n'y a aucune différence ; cochez un nombre négatif de -1 à -5 si l'influence est négative ; ou cochez un nombre positif de 1 à 5 si l'influence est positive. (1 dans chaque direction signifie peu d'influence et 5 dans chaque direction signifie beaucoup d'influence).

	-5	-4	-3	-2	-1	0	1	2	3	4	5	Je ne sais pas
FP 2.21.1 Décentralisation	○	○	○	○	○	○	○	○	○	○	○	○
FP 2.21.2 Programmes VIH/SIDA	○	○	○	○	○	○	○	○	○	○	○	○
FP 2.21.3 Incorporation de la PF au contexte plus large de la santé reproductive (SR)	○	○	○	○	○	○	○	○	○	○	○	○
FP 2.21.4 Intégration de la PF à d'autres services de santé	○	○	○	○	○	○	○	○	○	○	○	○
FP 2.21.5 Changements dans les financements des bailleurs de fonds	○	○	○	○	○	○	○	○	○	○	○	○
FP 2.21.6 Changements dans les financements du gouvernement	○	○	○	○	○	○	○	○	○	○	○	○

FP 2.22 Commentaires : Veuillez utiliser cette section pour tout commentaire additionnel sur le niveau d'effort, les difficultés et les succès rencontrés dans le pays autour des services ou programmes de PF. Si vous avez répondu « Je ne sais pas » à l'une des questions, utilisez aussi cette section pour expliquer pourquoi.

JOHNS HOPKINS
BLOOMBERG SCHOOL of PUBLIC HEALTH

Bill & Melinda Gates Institute for Population and Reproductive Health

Enquête de l'Indice des efforts pour le dividende démographique

Domaine 3. Plaidoyer

* FP 3.1 L'influence des dirigeants/ champions de PF : Mesure dans laquelle le pays compte sur un ou plusieurs dirigeants ou champions clairement influents plaidant avec succès au profit des besoins (sectoriels) de la PF auprès des décideurs, promouvant la PF dans le pays à travers des déclarations publiques positives, et/ou soutenant des actions et politiques visant à améliorer l'environnement de la PF et ses contributions au DD.

	1	2	3	4	5	6	7	8	9	10	Je ne sais pas
1-Plus faible -> Plus élevé-10	○	○	○	○	○	○	○	○	○	○	○

* FP 3.2 Les déclarations des dirigeants : Mesure dans laquelle le chef du gouvernement, ainsi que d'autres hauts fonctionnaires, parlent publiquement et favorablement de la planification familiale au moins une fois par an.

	1	2	3	4	5	6	7	8	9	10	Je ne sais pas
1-Plus faible -> Plus élevé-10	○	○	○	○	○	○	○	○	○	○	○

* FP 3.3 La couverture médiatique de la PF : Mesure dans laquelle et fréquence à laquelle la couverture médiatique nationale des sujets de PF apporte à la population des informations sur la planification familiale et les sites de prestation de services.

	1	2	3	4	5	6	7	8	9	10	Je ne sais pas
1-Plus faible -> Plus élevé-10	○	○	○	○	○	○	○	○	○	○	○

Rusatira JC, *et al. BMJ Open* 2023; 13:e059937. doi: 10.1136/bmjopen-2021-059937

* FP 3.4 Les influences des efforts de PF (justification) : Quelle est l'importance de chacun des éléments suivants en tant que justification actuelle par le gouvernement de son programme national de PF ? (1 signifie une importance négligeable ; 10 signifie une grande importance.)

	1	2	3	4	5	6	7	8	9	10	Je ne sais pas
3.4.1 Réduire le taux de croissance démographique	○	○	○	○	○	○	○	○	○	○	○
3.4.2 Stimuler le développement économique	○	○	○	○	○	○	○	○	○	○	○
3.4.3 Aider les hommes et les femmes à éviter des naissances non planifiées et non désirées	○	○	○	○	○	○	○	○	○	○	○
3.4.4 Améliorer la santé de la femme	○	○	○	○	○	○	○	○	○	○	○
3.4.5 Améliorer la santé de l'enfant	○	○	○	○	○	○	○	○	○	○	○
3.4.6 Réduire les grossesses chez les adolescentes non mariées	○	○	○	○	○	○	○	○	○	○	○
3.4.7 Réduire les besoins contraceptifs non satisfaits	○	○	○	○	○	○	○	○	○	○	○

* FP 3.5 Les influences des efforts de PF (les populations vulnérables) : Dans quelle mesure le programme de planification familiale met-il l’accent sur les populations vulnérables ou spécifiques ? (1 signifie un accent négligeable ; 10 signifie un accent important).

	1	2	3	4	5	6	7	8	9	10	Je ne sais pas
FP 3.5.1 Les jeunes non mariés	○	○	○	○	○	○	○	○	○	○	○
FP 3.5.2 Les populations vivant dans la pauvreté	○	○	○	○	○	○	○	○	○	○	○
FP 3.5.3 Les personnes en situation de handicap	○	○	○	○	○	○	○	○	○	○	○
FP 3.5.4 Les populations rurales	○	○	○	○	○	○	○	○	○	○	○
FP 3.5.5 Autres groupes vulnérables (ex : les minorités et/ou les groupes indigènes)	○	○	○	○	○	○	○	○	○	○	○
FP 3.5.6 Les femmes en postpartum pour le conseil et les services de contraception	○	○	○	○	○	○	○	○	○	○	○
FP 3.5.7 Les femmes en post-avortement pour le conseil et les services de contraception	○	○	○	○	○	○	○	○	○	○	○

* FP 3.6 Le marketing social : Mesure de la couverture du programme de marketing social du pays (ventes des contraceptifs subventionnés à faible coût dans le secteur commercial, particulièrement en milieu urbain).

	1	2	3	4	5	6	7	8	9	10	Je ne sais pas
1-Plus faible -> Plus élevé-10	○	○	○	○	○	○	○	○	○	○	○

FP 3.7 Commentaires : Veuillez utiliser cette section pour tout commentaire additionnel sur le niveau d’effort, les difficultés et les succès rencontrés dans le pays autour du plaidoyer pour la PF. Si vous avez répondu « Je ne sais pas » à l’une des questions, utilisez aussi cette section pour expliquer pourquoi.

JOHNS HOPKINS
BLOOMBERG SCHOOL of PUBLIC HEALTH

Bill & Melinda Gates Institute for Population and Reproductive Health

Enquête de l’Indice des efforts pour le dividende démographique

Domaine 4. Recherche

* FP 4.1 La force des observatoires/parties prenantes/groupes de travail pour la PF : Mesure dans laquelle les parties prenantes du secteur de la planification familiale du pays (ex : groupe directif de parties prenantes, groupes de travail techniques, observatoire de la PF, groupe de coordination ou facilitation) :

	1	2	3	4	5	6	7	8	9	10	Je ne sais pas
FP 4.1.1 Ont un représentant du gouvernement, des institutions de formation, de la société civile, des organisations non gouvernementales et confessionnelles, des associations professionnelles, du secteur privé, etc.	○	○	○	○	○	○	○	○	○	○	○
FP 4.1.2 Se réunissent régulièrement, émettent des rapports et recommandent des politiques aux directions des ministères compétents.	○	○	○	○	○	○	○	○	○	○	○
FP 4.1.3 Ont un impact sur la PF dans le pays.	○	○	○	○	○	○	○	○	○	○	○

* FP 4.2 La stratégie de recherche en PF : Mesure dans laquelle le plan national de PF et/ou d’autres documents nationaux comprennent une stratégie/approche complète de recherche sur la planification familiale.

	1	2	3	4	5	6	7	8	9	10	Je ne sais pas
1-Plus faible -> Plus élevé-10	○	○	○	○	○	○	○	○	○	○	○

* FP 4.3 Les partenaires collectant les données :

	1	2	3	4	5	6	7	8	9	10	Je ne sais pas
FP 4.3.1 Mesure dans laquelle la collecte des données est entreprise par des agences gouvernementales. (Cela peut comprendre les agences/bureaux de la statistique et/ou les ministères de l'économie/finances, éducation, santé, genre, etc.)	○	○	○	○	○	○	○	○	○	○	○
FP 4.3.2 Mesure dans laquelle la collecte de données est entreprise par des institutions de recherche.	○	○	○	○	○	○	○	○	○	○	○
FP 4.3.3 Mesure dans laquelle la collecte des données est entreprise par des chercheurs indépendants.	○	○	○	○	○	○	○	○	○	○	○

* FP 4.4 La recherche thématique :

	1	2	3	4	5	6	7	8	9	10	Je ne sais pas
FP 4.4.1 Mesure dans laquelle la recherche est menée sur l'adoption de la planification familiale (dont la distribution des méthodes), la demande et les besoins non satisfaits.	○	○	○	○	○	○	○	○	○	○	○
FP 4.4.2 Mesure dans laquelle de la recherche est menée sur les barrières à l'adoption de la PF	○	○	○	○	○	○	○	○	○	○	○
FP 4.4.3 Mesure dans laquelle de la recherche est menée sur les programmes de PF	○	○	○	○	○	○	○	○	○	○	○
FP 4.4.4 Mesure dans laquelle de la recherche est menée sur la qualité de la prestation des services de PF	○	○	○	○	○	○	○	○	○	○	○

* FP 4.5 La qualité/couverture des données : Mesure dans laquelle la recherche/les données actuelles sont désagrégées par sexe et par âge. Mesure dans laquelle le pays participe aux tests standardisés et la place qu'il occupe au classement.

	1	2	3	4	5	6	7	8	9	10	Je ne sais pas
FP 4.5.1 Mesure dans laquelle un système statistique de routine offre de bonnes informations périodiques sur les produits et les établissements de PF	○	○	○	○	○	○	○	○	○	○	○
FP 4.5.2 Mesure dans laquelle un système statistique de routine offre de bonnes informations régulières sur les services et le personnel du secteur de la PF	○	○	○	○	○	○	○	○	○	○	○
FP 4.5.3 Mesure dans laquelle un système statistique de routine offre de bonnes informations périodiques sur les besoins de PF des populations de différentes communautés	○	○	○	○	○	○	○	○	○	○	○
FP 4.5.4 Mesure dans laquelle des évaluations du système de suivi des informations sanitaires sont menées et appliquées pour assurer la fiabilité des données	○	○	○	○	○	○	○	○	○	○	○

* FP 4.6 Les registres : Mesure dans laquelle les systèmes de registres des clients, rapports des cliniques et retours/commentaires sur les résultats sont adéquats et connectés à tous les niveaux (fédéral, état/province, comté, etc.).

	1	2	3	4	5	6	7	8	9	10	Je ne sais pas
1-Plus faible -> Plus élevé-10	○	○	○	○	○	○	○	○	○	○	○

* FP 4.7 La qualité des institutions de recherche : Mesure dans laquelle le pays a la capacité de soutenir et maintenir des institutions de recherche qui développent des études permettant de soutenir la portée et la qualité de la collecte de données pour informer les efforts nationaux de PF.

	1	2	3	4	5	6	7	8	9	10	Je ne sais pas
1-Plus faible -> Plus élevé-10											

* FP 4.8 L'évaluation : Mesure dans laquelle les statistiques, enquêtes et études sur le programme de PF sont utilisées par du personnel spécialisé pour rapporter les opérations du programme et mesurer le progrès.

	1	2	3	4	5	6	7	8	9	10	Je ne sais pas
1-Plus faible -> Plus élevé-10											

* FP 4.9 L'utilisation des résultats d'évaluation par les gestionnaires de programmes : Mesure dans laquelle les gestionnaires locaux du programme de PF utilisent les conclusions de recherche et d'évaluations pour améliorer ces programmes en suivant les recommandations émises.

	1	2	3	4	5	6	7	8	9	10	Je ne sais pas
1-Plus faible -> Plus élevé-10											

* FP 4.10 L'utilisation des résultats d'évaluation par les ministères : Mesure dans laquelle les administrateurs ministériels compétents utilisent systématiquement les données pour informer les politiques et interventions visant à résoudre les problèmes liés à la PF.

	1	2	3	4	5	6	7	8	9	10	Je ne sais pas
1-Plus faible -> Plus élevé-10											

* FP 4.11 La dissémination d'informations à d'autres entités chargées de la mise en œuvre des programmes : Mesure dans laquelle des informations sont partagées ou disséminées entre géographies et à différents niveaux (national, état/province/comté, localités/sous-comtés).

	1	2	3	4	5	6	7	8	9	10	Je ne sais pas
1-Plus faible -> Plus élevé-10											

FP 4.12 Commentaires : Veuillez utiliser cette section pour tout commentaire additionnel sur le niveau d'effort, les difficultés et les succès rencontrés dans le pays autour de la recherche sur la PF. Si vous avez répondu « Je ne sais pas » à l'une des questions, utilisez aussi cette section pour expliquer pourquoi.

Rusatira JC, *et al. BMJ Open* 2023; 13:e059937. doi: 10.1136/bmjopen-2021-059937

JOHNS HOPKINS
BLOOMBERG SCHOOL of PUBLIC HEALTH

Bill & Melinda Gates Institute for Population and Reproductive Health

Enquête de l'Indice des efforts pour le dividende démographique

Domaine 5. Organisations de la société civile (OSC)

* FP 5.1 Le pouvoir des acteurs des OSC : Mesure dans laquelle les acteurs des OSC occupent des postes d'influence dans les institutions, les groupes de travail techniques ou les réseaux concernés par la PF.

	1	2	3	4	5	6	7	8	9	10	Je ne sais pas
1-Plus faible -> Plus élevé-10	○	○	○	○	○	○	○	○	○	○	○

* FP 5.2 L'analyse budgétaire comme outil des OSC : Mesure dans laquelle les OSC utilisent l'analyse budgétaire comme un outil pour développer le plaidoyer en matière de PF.

	1	2	3	4	5	6	7	8	9	10	Je ne sais pas
1-Plus faible -> Plus élevé-10	○	○	○	○	○	○	○	○	○	○	○

* FP 5.3 Le soutien aux services offerts en structure sanitaire : Mesure dans laquelle les OSC sont impliquées dans les efforts visant à augmenter l'utilisation et/ou la qualité des services, particulièrement à travers une plus grande capacité technique et/ou opérationnelle des cliniques travaillant en partenariat avec des CSO pour fournir des services de PF.

	1	2	3	4	5	6	7	8	9	10	Je ne sais pas
1-Plus faible -> Plus élevé-10	○	○	○	○	○	○	○	○	○	○	○

* FP 5.4 Le soutien aux services à base communautaire : Mesure dans laquelle les OSC participent au rapprochement des informations et services de PF avec les communautés à travers des activités telles que le renforcement de compétences des agents de santé communautaire (ASC), l'expansion de la gamme de méthodes contraceptives fournies par les ASC, la formation des cadres inférieurs de professionnels de la santé et/ou les campagnes d'information et d'éducation pour augmenter l'acceptabilité de la contraception.

	1	2	3	4	5	6	7	8	9	10	Je ne sais pas
1-Plus faible -> Plus élevé-10	○	○	○	○	○	○	○	○	○	○	○

* FP 5.5 Le soutien aux services à base communautaire à travers des campagnes mobiles de sensibilisation : Mesure dans laquelle les OSC soutiennent les modèles de services en dehors de cliniques pour que les prestataires de santé aient les commodités et produits de PF, l'équipement et les véhicules nécessaires pour être envoyés ensuite comme équipe spécialiste et fournir des informations et méthodes de PF directement aux communautés.

	1	2	3	4	5	6	7	8	9	10	Je ne sais pas
1-Plus faible -> Plus élevé-10	○	○	○	○	○	○	○	○	○	○	○

* FP 5.6 Mesure dans laquelle les OSC sont impliquées dans la franchise sociale des services de PF de qualité aux client(e)s à moindre coût.

	1	2	3	4	5	6	7	8	9	10	Je ne sais pas
1-Plus faible -> Plus élevé-10	○	○	○	○	○	○	○	○	○	○	○

* FP 5.7 La santé mobile : Mesure dans laquelle les OSC mettent à profit la technologie mobile, comme l'utilisation de la messagerie téléphonique (SMS), pour améliorer l'accès aux informations et services de PF.

	1	2	3	4	5	6	7	8	9	10	Je ne sais pas
1-Plus faible -> Plus élevé-10	○	○	○	○	○	○	○	○	○	○	○

* FP 5.8 Les droits humains, l'accès et la qualité de l'éducation : Mesure dans laquelle les OSC plaident pour des approches basées sur les droits et veillent à ce que les bénéficiaires des services de PF soit traités correctement, n'aient pas peur de poser des questions, et reçoivent des conseils de qualité, entre autres sujets d'importance.

	1	2	3	4	5	6	7	8	9	10	Je ne sais pas
1-Plus faible -> Plus élevé-10	○	○	○	○	○	○	○	○	○	○	○

* FP 5.9 Le changement social et des comportements (CSC) : Mesure dans laquelle les OSC soutiennent les interventions pour le CSC comprenant, mais sans s'y limiter, la promotion des compétences de vie et de l'éducation entre pairs, l'éducation sur la santé de l'adulte, l'éducation en milieu scolaire, la communication au sein du couple, la mobilisation communautaire, les interventions à travers les gardiens de la communauté et/ou les interventions médiatiques destinées au grand public. Ces interventions peuvent être mises en œuvre au niveau communautaire ou individuel, et avoir pour résultat une plus grande utilisation de la PF et des changements dans les connaissances et attitudes liées à la PF.

	1	2	3	4	5	6	7	8	9	10	Je ne sais pas
1-Plus faible -> Plus élevé-10	○	○	○	○	○	○	○	○	○	○	○

* FP 5.10 Les jeunes : Mesure dans laquelle les OSC soutiennent les politiques, interventions et programmes de PF ciblant les jeunes spécifiquement.

	1	2	3	4	5	6	7	8	9	10	Je ne sais pas
1-Plus faible -> Plus élevé-10	○	○	○	○	○	○	○	○	○	○	○

* FP 5.11 Les programmes pour les hommes : Mesure dans laquelle les OSC soutiennent des politiques, interventions et programmes de PF pour les hommes.

	1	2	3	4	5	6	7	8	9	10	Je ne sais pas
1-Plus faible -> Plus élevé-10	○	○	○	○	○	○	○	○	○	○	○

* FP 5.12 Le plaidoyer et la redevabilité : Mesure dans laquelle les OSC soutiennent la formation et le renforcement de compétences des plaideurs et des membres de la communauté pour améliorer leur compréhension des politiques et des processus d'identification de problèmes, de résolution collaborative de ces derniers, et des actions de plaidoyer plus ciblées pour améliorer les programmes de planification familiale.

	1	2	3	4	5	6	7	8	9	10	Je ne sais pas
1-Plus faible -> Plus élevé-10	○	○	○	○	○	○	○	○	○	○	○

* FP 5.13 L'évaluation et le suivi dirigés par les OSC : Mesure dans laquelle les OSC évaluent, suivent et émettent des rapports sur l'efficacité des politiques et programmes pour améliorer la redevabilité des prestataires de santé et des politiques de PF.

	1	2	3	4	5	6	7	8	9	10	Je ne sais pas
1-Plus faible -> Plus élevé-10	○	○	○	○	○	○	○	○	○	○	○

* FP 5.14 Les partenariats entre OSC : Mesure dans laquelle les OSC ont formé des alliances nationales et des partenariats régionaux pour renforcer leur position et potentiellement améliorer le leadership et les mécanismes de financement.

	1	2	3	4	5	6	7	8	9	10	Je ne sais pas
1-Plus faible -> Plus élevé-10	○	○	○	○	○	○	○	○	○	○	○

FP 5.15 Commentaires : Veuillez utiliser cette section pour tout commentaire additionnel sur le niveau d'effort, les difficultés et les succès rencontrés dans le pays autour des organisations de la société civile (OSC) pour la PF. Si vous avez répondu « Je ne sais pas » à l'une des questions, utilisez aussi cette section pour expliquer pourquoi.

JOHNS HOPKINS
BLOOMBERG SCHOOL
of PUBLIC HEALTH

Bill & Melinda Gates Institute for Population and Reproductive Health

Enquête de l'Indice des efforts pour le dividende démographique

Module : Résilience et durabilité du secteur

Étant donné l'impact sanitaire et socioéconomique de la pandémie de COVID-19, et son impact probable sur la progression vers le DD, cet indice des efforts pour le DD intègre des questions pour évaluer la résilience et la durabilité des systèmes dans les secteurs clés du DD. Les éléments couverts par les questions liées à la pandémie de COVID-19 ci-dessous relèvent des dimensions clés des systèmes résilients et visent à apporter des informations déterminantes sur le potentiel d'une réponse efficace aux menaces de maladies infectieuses émergentes et autres crises de santé publique.

Veuillez noter chaque élément sur une échelle de 1 à 10, 1 étant le score le plus faible (faible état/capacité) et 10 le plus élevé (très bon état/capacité).

* Dimension physique : structures, équipements, états du système et capacités

	1	2	3	4	5	6	7	8	9	10	Je ne sais pas
FP M1 - Plan/Préparation - État et capacité de l'équipement, du personnel et de la structure du programme de PF avant la crise. (1 = faible état/capacité ; 10 = très bon état/capacité)	◯	◯	◯	◯	◯	◯	◯	◯	◯	◯	◯
FP M2 - Absorption – Intégration : Niveau d'intégration des services et programmes de PF pour mitiger les effets de la pandémie de COVID-19 sur la PF.	◯	◯	◯	◯	◯	◯	◯	◯	◯	◯	◯
FP M3 - Absorption – Intégration : Mesure dans laquelle la gestion efficace de la chaîne d'approvisionnement a pu mitiger les effets de la pandémie de COVID-19 sur l'approvisionnement/distribution des commodités de PF.	◯	◯	◯	◯	◯	◯	◯	◯	◯	◯	◯
FP M4 - Récupération - Mesure dans laquelle le secteur de la PF a été capable d'induire des changements pour récupérer un niveau de fonctionnalité pré-COVID-19.	◯	◯	◯	◯	◯	◯	◯	◯	◯	◯	◯
FP M5 - Récupération – Gestion du financement et des bailleurs de fonds : Niveau de mobilisation des ressources et allocations pour mitiger les effets de la pandémie de COVID-19 sur la PF.	◯	◯	◯	◯	◯	◯	◯	◯	◯	◯	◯
FP M6 - Adaptation – Mesure dans laquelle des changements ont été effectués pour améliorer la résilience du secteur PF.	◯	◯	◯	◯	◯	◯	◯	◯	◯	◯	◯

* Dimension informatique : création, gestion, stockage et utilisation des données.

	1	2	3	4	5	6	7	8	9	10	Je ne sais pas
FP M7 - Plan/Préparation – Mesure dans laquelle les données liées à la PF étaient suffisamment préparées, présentées, analysées et stockées avant la crise.	○	○	○	○	○	○	○	○	○	○	○
FP M8 - Absorption – Connaissance : Mesure dans laquelle le programme de PF a pu conduire des évaluations en temps réel de son état de fonctionnement, en anticipation des pertes en cascade des services de PF.	○	○	○	○	○	○	○	○	○	○	○
FP M9 - Récupération – Mesure dans laquelle les données de la PF ont été utilisées pour suivre le progrès vers la récupération et anticiper des scenari de récupération.	○	○	○	○	○	○	○	○	○	○	○
FP M10 - Adaptation – Mesure dans laquelle le système de la PF crée et améliore actuellement ses protocoles de stockage et d'utilisation des données de PF en temps réel.	○	○	○	○	○	○	○	○	○	○	○

* Dimension cognitive : compréhension, modèles cognitifs, idées reçues, préjugés et valeurs.

	1	2	3	4	5	6	7	8	9	10	Je ne sais pas
FP M11 - Planification/Préparation – Mesure dans laquelle le système de la PF et les décisions opérationnelles étaient préparés à anticiper la crise.	○	○	○	○	○	○	○	○	○	○	○

	1	2	3	4	5	6	7	8	9	10	Je ne sais pas
FP M12 - Absorption – Mesure dans laquelle la réponse du secteur de la PF a des protocoles de contingence suffisants et une gestion de crise proactive.	○	○	○	○	○	○	○	○	○	○	○
FP M13 - Absorption – Redevabilité : Niveau d'effort pour assurer une redevabilité optimale vis-à-vis des ressources allouées à la PF dans le cadre de la réponse à la pandémie de COVID-19.	○	○	○	○	○	○	○	○	○	○	○
FP M14 - Récupération – Mesure dans laquelle les décisions sont orientées vers la récupération et communiquées aux communautés sur la base de données probantes pour promouvoir des comportements sûrs.	○	○	○	○	○	○	○	○	○	○	○
FP M15 - Récupération – Connaissance/Conscience : Mesure dans laquelle le secteur de la PF établit une certaine confiance à travers sa communication sur les barrières posées par la pandémie de COVID-19 pour soutenir l'accès et l'utilisation des services et programmes de PF.	○	○	○	○	○	○	○	○	○	○	○
FP M16 - Adaptation : Niveau d'adaptation pour répondre aux menaces posées par la pandémie de COVID-19 à la PF.	○	○	○	○	○	○	○	○	○	○	○

* Social dimension: interaction, collaboration and self-synchronization between people, entities and institutions.

36

	1	2	3	4	5	6	7	8	9	10	Je ne sais pas
FP M17 - Planification /Préparation – Mesure dans laquelle une formation sur la gestion d'épidémies/crises a été menée et a permis de mettre à profit les réseaux sociaux, le capital social et les normes institutionnelles et culturelles avant la crise.	○	○	○	○	○	○	○	○	○	○	○
FP M18 - Absorption – Mesure dans laquelle le personnel et les institutions sociales ont été accessibles et ont fait preuve d'ingéniosité dans leur réponse à l'épidémie/crise.	○	○	○	○	○	○	○	○	○	○	○
PF M19 - Absorption - Self-regulation: Extent to which national leaders had the authority to effect timely sectoral changes through an infrastructureflexible.	○	○	○	○	○	○	○	○	○	○	○
FP M20 - Récupération – Mesure dans laquelle le secteur de la PF est impliqué dans le partage de connaissances et d'équipes pour améliorer la récupération du système.	○	○	○	○	○	○	○	○	○	○	○
FP M21 - Récupération – Diversité : Niveau d'engagement d'une équipe multidisciplinaire pour mitiger les effets de la pandémie de COVID-19 sur la PF.	○	○	○	○	○	○	○	○	○	○	○
FP M22 - Adaptation – Mesure dans laquelle des ajouts ou changements sont apportés aux institutions, politiques, formations, programmes et à la culture de la PF.	○	○	○	○	○	○	○	○	○	○	○

	1	2	3	4	5	6	7	8	9	10	Je ne sais pas
FP M23 - Adaptation - Leadership et management : Niveau de leadership démontré par les dirigeants du secteur de la PF pour mitiger les effets de la pandémie de COVID-19 sur la PF.	○	○	○	○	○	○	○	○	○	○	○

* FP M-24 Temporalité : Mesure de la rapidité de la réaction du gouvernement –sa communication et mise en œuvre des mesures- pour mitiger l'impact immédiat et sur le long terme de la pandémie de COVID-19. Cette question porte sur la riposte nationale dans son ensemble, et non la réponse spécifique de votre secteur.

	1	2	3	4	5	6	7	8	9	10	Je ne sais pas
1-Plus faible -> Plus élevé-10	○	○	○	○	○	○	○	○	○	○	○

FP M-25 Commentaires : Veuillez utiliser cette section pour tout commentaire additionnel sur la résilience et la durabilité des systèmes de PF nationaux en fonction de votre expérience de la pandémie de COVID-19. Si vous avez répondu « Je ne sais pas » à l'une des questions, utilisez aussi cette section pour expliquer pourquoi.

JOHNS HOPKINS
BLOOMBERG SCHOOL
of PUBLIC HEALTH

Bill & Melinda Gates Institute for Population and Reproductive Health

Enquête de l’Indice des efforts pour le dividende démographique

Questionnaire sur la santé maternelle et infantile (SMI)

Répondez aux questions suivantes en fonction de votre expérience/ expertise dans ce secteur spécifique.

Afin d’obtenir une représentation synthétique des efforts nationaux pour le dividende démographique, notez les éléments suivants sur une échelle de 1 à 10, 1 étant le score le plus faible (efforts très faibles ou quasi-inexistants) et 10 le plus élevé (efforts robustes). Le cas échéant, si une politique ou activité n’existe pas, répondez 0.

Donnez un score à chaque élément. Toutes les réponses seront enregistrées au format illustré ci-dessous :

Effort le plus faible	1	2	3	4	5	6	7	8	9	10	Effort le plus élevé	Je ne sais pas

JOHNS HOPKINS
BLOOMBERG SCHOOL
of PUBLIC HEALTH

Bill & Melinda Gates Institute for Population and Reproductive Health

Enquête de l'Indice des efforts pour le dividende démographique

Domaine 1. Politique/ Prise de décision

* MCH 1.1 L'adéquation de la politique : Politiques du Ministère de la Santé en faveur des services pendant la grossesse et l'accouchement qui tiennent compte de toutes les conditions requises pour s'assurer que la mère et son nouveau-né survivent.

	1	2	3	4	5	6	7	8	9	10	Je ne sais pas
MCH 1.1.1 Mesure dans laquelle la santé reproductive, maternelle et infantile a été intégrée à la politique/plan/stratégie de santé nationale.	○	○	○	○	○	○	○	○	○	○	○
MCH 1.1.2 Mesure dans laquelle le/les plan(s) de mise en œuvre de la/les stratégie(s) nationale(s) de santé reproductive, maternelle et infantile sont disponibles.	○	○	○	○	○	○	○	○	○	○	○

* MCH 1.2 L'accès universel : Mesure dans laquelle des droits légaux et constitutionnels existent pour faciliter l'accès universel aux services de santé reproductive, maternelle et infantile.

	1	2	3	4	5	6	7	8	9	10	Je ne sais pas
1-Plus faible -> Plus élevé-10	○	○	○	○	○	○	○	○	○	○	○

* MCH 1.3 L'implication de divers secteurs : Mesure dans laquelle des politiques sont élaborées à travers une consultation adéquate des parties intéressées, dont les ministères, les ONG, les praticiens privés et les associations de femmes.

	1	2	3	4	5	6	7	8	9	10	Je ne sais pas
1-Plus faible -> Plus élevé-10	○	○	○	○	○	○	○	○	○	○	○

40

* MCH 1.4 La compétence des prestataires de santé : Mesure dans laquelle les politiques sont rationnelles et fondées sur des données probantes établissant quel personnel peut fournir des services de santé maternelle (ex : les sages-femmes formées peuvent effectuer une vaste gamme de procédures).

	1	2	3	4	5	6	7	8	9	10	Je ne sais pas
1-Plus faible -> Plus élevé-10	○	○	○	○	○	○	○	○	○	○	○

* MCH 1.5 La légalisation de l'avortement : Mesure dans laquelle une politique favorable existe pour assurer l'offre légale de services d'avortement.

	1	2	3	4	5	6	7	8	9	10	Je ne sais pas
1-Plus faible -> Plus élevé-10	○	○	○	○	○	○	○	○	○	○	○

* MCH 1.6. La légalisation des soins post-avortement : Mesure dans laquelle une politique favorable existe pour le traitement des complications dues aux avortements, y compris les complications résultant d'avortements clandestins.

	1	2	3	4	5	6	7	8	9	10	Je ne sais pas
1-Plus faible -> Plus élevé-10	○	○	○	○	○	○	○	○	○	○	○

* MCH 1.7 La révision et mise à jour des politiques : Mesure dans laquelle les politiques sont régulièrement revues par des législateurs de haut niveau et utilisées dans les plans d'action.

	1	2	3	4	5	6	7	8	9	10	Je ne sais pas
1-Plus faible -> Plus élevé-10	○	○	○	○	○	○	○	○	○	○	○

* MCH 1.8 Le haut placement des acteurs : Mesure dans laquelle le/la directeur/trice des services de santé maternelle et infantile est haut placé(e) au gouvernement et impliqué(e) dans la prise de décision.

	1	2	3	4	5	6	7	8	9	10	Je ne sais pas
1-Plus faible -> Plus élevé-10	○	○	○	○	○	○	○	○	○	○	○

* MCH 1.9 Le financement de la SMI : Mesure dans laquelle les budgets du gouvernement pour la grossesse et l'accouchement sans risque, ainsi que pour les soins périnataux (pour les structures de santé, le personnel, les produits, etc.) et la santé infantile sont adaptés aux besoins, et si ces financements proviennent du Ministère de la Santé, gouvernement provincial/ des comtés ou des bailleurs de fonds.

	1	2	3	4	5	6	7	8	9	10	Je ne sais pas
1-Plus faible -> Plus élevé-10	○	○	○	○	○	○	○	○	○	○	○

* MCH 1.10 L'accessibilité financière : Mesure dans laquelle les services et médicaments liés à la SMI sont à un prix abordable pour toutes les clientes.

	1	2	3	4	5	6	7	8	9	10	Je ne sais pas
1-Plus faible -> Plus élevé-10	○	○	○	○	○	○	○	○	○	○	○

* MCH 1.11 Le secteur privé : Mesure dans laquelle le secteur privé (médecins, sages-femmes, cliniques) est actif et couvre une part importance des soins de grossesse et d'accouchement.

	1	2	3	4	5	6	7	8	9	10	Je ne sais pas
1-Plus faible -> Plus élevé-10	○	○	○	○	○	○	○	○	○	○	○

* MCH 1.12 Le directorat de SMI : Mesure dans laquelle le Ministère de la Santé a un directorat spécifiquement dédié à la SMI.

	1	2	3	4	5	6	7	8	9	10	Je ne sais pas
1-Plus faible -> Plus élevé-10	○	○	○	○	○	○	○	○	○	○	○

* MCH 1.13 Les solutions potentielles : Mesure dans laquelle le gouvernement reconnait des solutions potentielles aux problèmes de SMI par les moyens suivants :

	1	2	3	4	5	6	7	8	9	10	Je ne sais pas
MCH 1.13.1 Efficience régulatrice	○	○	○	○	○	○	○	○	○	○	○
MCH 1.13.2 Performance et redevabilité	○	○	○	○	○	○	○	○	○	○	○
MCH 1.13.3 Approche fondée sur le droit de la SMNI	○	○	○	○	○	○	○	○	○	○	○
MCH 1.13.4 Infrastructure de santé essentielle	○	○	○	○	○	○	○	○	○	○	○
MCH 1.13.5 Engagement de la communauté	○	○	○	○	○	○	○	○	○	○	○

MCH 1.14 Commentaires : Utilisez cette section pour apporter des commentaires additionnels sur le niveau d’effort, les défis et les succès du pays autour de la politique/ prise de décision pour la SMI. Si vous avez répondu « Je ne sais pas » à l’une des questions précédentes, utilisez aussi cette section pour expliquer pourquoi.

JOHNS HOPKINS
BLOOMBERG SCHOOL of PUBLIC HEALTH

Bill & Melinda Gates Institute for Population and Reproductive Health

Enquête de l'Indice des efforts pour le dividende démographique

Dimension 2. Services or Programs

* MCH 2.1 La disponibilité des soins obstétriques – Dans tous les centres : Mesure dans laquelle les structures de santé primaire ont du personnel qualifié, en place, et peuvent fournir des soins obstétriques :

	1	2	3	4	5	6	7	8	9	10	Je ne sais pas
MCH 2.1.1 Cas d'hémorragie postpartum adéquatement gérés	○	○	○	○	○	○	○	○	○	○	○
MCH 2.1.2 Administration adéquate et opportune d'antibiotiques en intraveineuse lorsque nécessaire.	○	○	○	○	○	○	○	○	○	○	○
MCH 2.1.3 Expulsion manuelle dans les cas de rétention placentaire.	○	○	○	○	○	○	○	○	○	○	○
MCH 2.1.4 Aspiration de l'utérus manuelle (Méthode de Karman) ou à l'aide d'un appareil à succion électrique.	○	○	○	○	○	○	○	○	○	○	○
MCH 2.1.5 Utilisation d'un partogramme pour savoir quand référer.	○	○	○	○	○	○	○	○	○	○	○
MCH 2.1.6 Transport organized to quickly transfer a woman with obstructed labor to the district / sub-county hospital.	○	○	○	○	○	○	○	○	○	○	○
MCH 2.1.7 Antibiotiques adéquats disponibles (suffisamment de produits du bon type).	○	○	○	○	○	○	○	○	○	○	○

* MCH 2.2 La disponibilité des soins obstétriques – Les structures de santé secondaires : Mesure dans laquelle tous les hôpitaux de districts/sous-comtés ont du personnel formé, en place, capable de :

	1	2	3	4	5	6	7	8	9	10	Je ne sais pas
MCH 2.2.1 Remplir toutes les fonctions citées ci-dessus pour les structures de santé primaires	○	○	○	○	○	○	○	○	○	○	○
MCH 2.2.2 Effectuer des transfusions sanguines (et avoir un approvisionnement en sang sûr, adéquat et disponible).	○	○	○	○	○	○	○	○	○	○	○
MCH 2.2.3 Effectuer des césariennes.	○	○	○	○	○	○	○	○	○	○	○

* MCH 2.3 Les services liés à la grossesse : Mesure dans laquelle toutes les femmes enceintes ont accès à :

	1	2	3	4	5	6	7	8	9	10	Je ne sais pas
MCH 2.3.1 Traitement des hémorragies postpartum pendant ou peu après l'accouchement.	◯	◯	◯	◯	◯	◯	◯	◯	◯	◯	◯
MCH 2.3.2 Gestion de la dystocie.	◯	◯	◯	◯	◯	◯	◯	◯	◯	◯	◯
MCH 2.3.3 Management de la pré-éclampsie, éclampsie et ses complications.	◯	◯	◯	◯	◯	◯	◯	◯	◯	◯	◯
MCH 2.3.4 Soins post-avortement ou pour les complications liées aux avortements.	◯	◯	◯	◯	◯	◯	◯	◯	◯	◯	◯
MCH 2.3.5 Offre de services d'avortement sans risque.	◯	◯	◯	◯	◯	◯	◯	◯	◯	◯	◯
MCH 2.3.6 Soins prénatals pendant la grossesse.	◯	◯	◯	◯	◯	◯	◯	◯	◯	◯	◯
MCH 2.3.7 Soins d'accouchement par un professionnel formé.	◯	◯	◯	◯	◯	◯	◯	◯	◯	◯	◯
MCH 2.3.8 Services de planification familiale postpartum.	◯	◯	◯	◯	◯	◯	◯	◯	◯	◯	◯
MCH 2.3.9 Hôpitaux des districts/ sous-comtés ouverts 24h/24 et 7j/7.	◯	◯	◯	◯	◯	◯	◯	◯	◯	◯	◯

* MCH 2.4 Les soins prénatals pour les femmes non mariées et séropositives : Mesure dans laquelle, à toutes les consultations prénatales, toutes les femmes enceintes :

	1	2	3	4	5	6	7	8	9	10	Je ne sais pas
MCH 2.4.1 Reçoivent des soins prénatals complets conformément aux protocoles recommandés par l'OMS pour les femmes séronégatives.	○	○	○	○	○	○	○	○	○	○	○
MCH 2.4.2 Reçoivent des soins prénatals complets conformément aux protocoles recommandés par l'OMS pour les femmes séropositives.	○	○	○	○	○	○	○	○	○	○	○
MCH 2.4.3 Reçoivent des soins prénatals complets conformément aux protocoles recommandés par l'OMS pour les jeunes femmes non mariées.	○	○	○	○	○	○	○	○	○	○	○
MCH 2.4.4 Sont informées des signes de danger de complications obstétriques et néonatales, et sont assistées pour planifier toute urgence éventuelle.	○	○	○	○	○	○	○	○	○	○	○

* MCH 2.5 Les soins néonatals : Mesure dans laquelle, pour les soins néonatals, tous les nouveau-nés, qu'ils soient nés à la maison ou en structure médicale :

	1	2	3	4	5	6	7	8	9	10	Je ne sais pas
MCH 2.5.1 Ont leur bouche et voie nasale dégagées.	○	○	○	○	○	○	○	○	○	○	○
MCH 2.5.2 Sont séchés et gardés au chaud immédiatement après la naissance.	○	○	○	○	○	○	○	○	○	○	○
MCH 2.5.3 Reçoivent de la vitamine A.	○	○	○	○	○	○	○	○	○	○	○
MCH 2.5.4 Ont leur cordon ombilical coupé avec une lame propre.	○	○	○	○	○	○	○	○	○	○	○
MCH 2.5.5 Suivent le calendrier de vaccination recommandé par l'OMS.	○	○	○	○	○	○	○	○	○	○	○
MCH 2.5.6 Bénéficient des activités de promotion de l'allaitement exclusif.	○	○	○	○	○	○	○	○	○	○	○
MCH 2.5.7 Bénéficient des programmes de soutien alimentaire lorsqu'ils en ont besoin.	○	○	○	○	○	○	○	○	○	○	○

* MCH 2.6 La planification familiale – Dans tous les centres : Mesure dans laquelle la planification familiale est dûment fournie dans tous les centres de santé en :

	1	2	3	4	5	6	7	8	9	10	Je ne sais pas
MCH 2.6.1 Offrant régulièrement des services de planification familiale aux cas post-avortement.	○	○	○	○	○	○	○	○	○	○	○
MCH 2.6.2 Offrant régulièrement des services de planification familiale lors des consultations postpartum.	○	○	○	○	○	○	○	○	○	○	○
MCH 2.6.3 Ayant des méthodes contraceptives réversibles de courte et longue durée régulièrement en stock.	○	○	○	○	○	○	○	○	○	○	○
MCH 2.6.4 Ayant du personnel formé, sur place, pouvant fournir les méthodes LARC.	○	○	○	○	○	○	○	○	○	○	○
MCH 2.6.5 Suivant les directives et protocoles nécessaires à la prestation de services de planification familiale.	○	○	○	○	○	○	○	○	○	○	○

* MCH 2.7 La planification familiale – Mesure dans laquelle la planification familiale est dûment fournie dans tous hôpitaux de districts/sous-comtés en :

	1	2	3	4	5	6	7	8	9	10	Je ne sais pas
MCH 2.7.1 Offrant régulièrement des services de planification familiale aux cas post-avortement.	○	○	○	○	○	○	○	○	○	○	○
MCH 2.7.2 Offrant régulièrement des services de planification familiale lors des consultations postpartum.	○	○	○	○	○	○	○	○	○	○	○
MCH 2.7.3 Ayant des méthodes contraceptives réversibles de courte et longue durée régulièrement en stock.	○	○	○	○	○	○	○	○	○	○	○
MCH 2.7.4 Ayant du personnel formé, sur place, pouvant fournir les méthodes LARC.	○	○	○	○	○	○	○	○	○	○	○
MCH 2.7.5 Pouvant offrir la stérilisation féminine.	○	○	○	○	○	○	○	○	○	○	○
MCH 2.7.6 Pouvant offrir la stérilisation masculine.	○	○	○	○	○	○	○	○	○	○	○
MCH 2.7.7 Suivant les directives et protocoles nécessaires à la prestation de services de planification familiale.	○	○	○	○	○	○	○	○	○	○	○

* MCH 2.8 Les maladies infantiles : Mesure dans laquelle la gestion de maladies infantiles recommandée par l'OMS est mise en œuvre en termes de :

	1	2	3	4	5	6	7	8	9	10	Je ne sais pas
MCH 2.8.1 Gestion à base communautaire des maladies infantiles.	○	○	○	○	○	○	○	○	○	○	○
MCH 2.8.2 Efficacité des services de référence du niveau communautaire vers les structures de santé.	○	○	○	○	○	○	○	○	○	○	○

MCH 2.9 Commentaires : Utilisez cette section pour apporter des commentaires additionnels sur le niveau d'effort, les défis et les succès du pays autour des programmes ou services de SMI. Si vous avez répondu « Je ne sais pas » à l'une des questions précédentes, utilisez aussi cette section pour expliquer pourquoi.

JOHNS HOPKINS
BLOOMBERG SCHOOL
of PUBLIC HEALTH

Bill & Melinda Gates Institute for Population and Reproductive Health

Enquête de l'Indice des efforts pour le dividende démographique

Domaine 3. Le plaidoyer

* MCH 3.1 Les messages du gouvernement : Mesure dans laquelle les hauts fonctionnaires au gouvernement, dont au Ministère de la Santé, s'adressent fréquemment à toutes les parties prenantes, y compris la presse, pour soutenir des améliorations et promouvoir les grossesses et les accouchements sans risque, et les enfants en bonne santé.

	1	2	3	4	5	6	7	8	9	10	Je ne sais pas
1-Plus faible -> Plus élevé-10	○	○	○	○	○	○	○	○	○	○	○

* MCH 3.2 Les médias de masse : Mesure dans laquelle le programme national utilise les médias de masse pour éduquer le public sur les symptômes des complications liées aux grossesses, sur les accouchements sans risque et les enfants en bonne santé.

	1	2	3	4	5	6	7	8	9	10	Je ne sais pas
1-Plus faible -> Plus élevé-10	○	○	○	○	○	○	○	○	○	○	○

* MCH 3.3 Les médias au niveau communautaire : Mesure dans laquelle les groupes communautaires prennent part aux programmes systématiques pour éduquer le public sur les grossesses et accouchements sans risque et les enfants en bonne santé.

	1	2	3	4	5	6	7	8	9	10	Je ne sais pas
1-Plus faible -> Plus élevé-10	○	○	○	○	○	○	○	○	○	○	○

52

* MCH 3.4 Les supports éducatifs : Mesure dans laquelle le ministère compétent (Ministère de la Santé) fournit des supports éducatifs adéquats (posters, brochures, etc.) aux structures de santé pour qu'elles instruisent les client(e)s sur les pratiques sûres/sans risque.

	1	2	3	4	5	6	7	8	9	10	Je ne sais pas
1-Plus faible -> Plus élevé-10	◯	◯	◯	◯	◯	◯	◯	◯	◯	◯	◯

* MCH 3.5 Auto-révision des cas de mortalité maternelle : Mesure dans laquelle chaque structure de santé suit une procédure régulière de révision de tous ses cas de décès maternels ayant eu lieu dans la structure pour en tirer des leçons.

	1	2	3	4	5	6	7	8	9	10	Je ne sais pas
1-Plus faible -> Plus élevé-10	◯	◯	◯	◯	◯	◯	◯	◯	◯	◯	◯

* MCH 3.6 Informer les politiques avec de nouvelles données probantes : Mesure dans laquelle chaque structure de santé transmet les leçons tirées des cas de décès/ mortalité maternelle aux ministères pour informer les nouvelles politiques et interventions.

	1	2	3	4	5	6	7	8	9	10	Je ne sais pas
1-Plus faible -> Plus élevé-10	◯	◯	◯	◯	◯	◯	◯	◯	◯	◯	◯

MCH 3.7 Commentaires : Utilisez cette section pour apporter des commentaires additionnels sur le niveau d'effort, les défis et les succès du pays autour du plaidoyer pour la SMI. Si vous avez répondu « Je ne sais pas » à l'une des questions précédentes, utilisez aussi cette section pour expliquer pourquoi.

Rusatira JC, *et al. BMJ Open* 2023; 13:e059937. doi: 10.1136/bmjopen-2021-059937

JOHNS HOPKINS BLOOMBERG SCHOOL of PUBLIC HEALTH

Bill & Melinda Gates Institute for Population and Reproductive Health

Enquête de l'Indice des efforts pour le dividende démographique

Domaine 4. Recherche

* MCH 4.1 La force des observatoires/parties prenantes/groupes de travail pour la SMI : Mesure dans laquelle les parties prenantes de la SMI du pays (ex : groupe directif de parties prenantes, groupes de travail techniques, observatoire de SMI, groupe de coordination ou facilitation) :

	1	2	3	4	5	6	7	8	9	10	Je ne sais pas
MCH 4.1.1 Ont un représentant du gouvernement, des institutions de formation, de la société civile, des organisations non gouvernementales et confessionnelles, des associations professionnelles, du secteur privé, etc.	○	○	○	○	○	○	○	○	○	○	○
MCH 4.1.2 Se réunissent régulièrement, émettent des rapports et recommandent des politiques aux directions des ministères compétents.	○	○	○	○	○	○	○	○	○	○	○
MCH 4.1.3 Ont un impact sur l'éducation dans le pays.	○	○	○	○	○	○	○	○	○	○	○

* MCH 4.2 MCH La stratégie d'éducation/recherche : Mesure dans laquelle le plan national de SMI et/ou d'autres documents nationaux comprennent une stratégie/approche complète de recherche liée sur la SMI et est partagée entre partenaires.

	1	2	3	4	5	6	7	8	9	10	Don't know
1-Plus faible -> Plus élevé-10	○	○	○	○	○	○	○	○	○	○	○

* MCH 4.3 Les partenaires collectant les données:

	1	2	3	4	5	6	7	8	9	10	Je ne sais pas
MCH 4.3.1 Mesure dans laquelle la collecte des données est entreprise par des agences gouvernementales. Cela peut comprendre les agences/bureaux de la statistique et/ou les ministères de l'économie/finances, éducation, santé, genre, etc.).	○	○	○	○	○	○	○	○	○	○	○
MCH 4.3.2 Mesure dans laquelle la collecte de données est entreprise par des institutions de recherche.	○	○	○	○	○	○	○	○	○	○	○
MCH 4.3.3 Mesure dans laquelle la collecte des données est entreprise par des chercheurs indépendants.	○	○	○	○	○	○	○	○	○	○	○

* MCH 4.4 La recherche thématique :

	1	2	3	4	5	6	7	8	9	10	Je ne sais pas
MCH 4.4.1 Mesure dans laquelle de la recherche est menée sur la mortalité maternelle.	○	○	○	○	○	○	○	○	○	○	○
MCH 4.4.2 Mesure dans laquelle de la recherche est menée sur la mortalité néonatale et infantile.	○	○	○	○	○	○	○	○	○	○	○
MCH 4.4.3 Mesure dans laquelle de la recherche est menée sur la planification familiale et la planification familiale postpartum.	○	○	○	○	○	○	○	○	○	○	○
MCH 4.4.4 Mesure dans laquelle de la recherche est menée sur la prestation/accès aux services et la qualité des prestataires de SMI.	○	○	○	○	○	○	○	○	○	○	○

* MCH 4.5 La qualité/couverture des données : Mesure dans laquelle la recherche/les données actuelles sont désagrégées par sexe, âge, lieu, ethnicité, niveau d'études, quintile de revenus et statut d'handicap. Mesure dans laquelle le pays participe à des tests standardisés et son classement.

	1	2	3	4	5	6	7	8	9	10	Je ne sais pas
MCH 4.5.1 Mesure dans laquelle un système statistique de routine offre de bonnes données périodiques sur les produits/l'approvisionnement et les structures de santé.	◯	◯	◯	◯	◯	◯	◯	◯	◯	◯	◯
MCH 4.5.2 Mesure dans laquelle un système statistique de routine offre de bonnes données périodiques sur les services et le personnel de SMI.	◯	◯	◯	◯	◯	◯	◯	◯	◯	◯	◯
MCH 4.5.3 Mesure dans laquelle un système statistique de routine offre de bonnes données périodiques sur les besoins en SMI de différentes populations dans les communautés.	◯	◯	◯	◯	◯	◯	◯	◯	◯	◯	◯
MCH 4.5.4 4 Mesure dans laquelle des évaluations du système de suivi de l'éducation sont menées et appliquées pour assurer la fiabilité des données.	◯	◯	◯	◯	◯	◯	◯	◯	◯	◯	◯
MCH 4.5.5 Mesure dans laquelle des évaluations du système de suivi de l'éducation sont menées et appliquées pour explorer les inégalités dans les services et fardeaux de la SMNI.	◯	◯	◯	◯	◯	◯	◯	◯	◯	◯	◯

* MCH 4.6 Les registres : Mesure dans laquelle les systèmes de registres, rapports et feedback sur les résultats de SMI sont adéquats, y compris en étant connectés à tous les niveaux (fédéral, état/province, comté, etc.).

	1	2	3	4	5	6	7	8	9	10	Je ne sais pas
1-Plus faible -> Plus élevé-10	◯	◯	◯	◯	◯	◯	◯	◯	◯	◯	◯

56

* MCH 4.7 La qualité des institutions de recherche : Mesure dans laquelle le pays a la capacité de soutenir et maintenir des institutions de recherche qui développent des études et/ou collectent des données permettant des services SMI de haute qualité.

	1	2	3	4	5	6	7	8	9	10	Je ne sais pas
1-Plus faible -> Plus élevé-10	○	○	○	○	○	○	○	○	○	○	○

* MCH 4.8 L'évaluation : Mesure dans laquelle les statistiques, enquêtes et études sur le programme de SMI sont utilisées par du personnel spécialisé pour rapporter les opérations du programme et mesurer le progrès.

	1	2	3	4	5	6	7	8	9	10	Je ne sais pas
1-Plus faible -> Plus élevé-10	○	○	○	○	○	○	○	○	○	○	○

* MCH 4.9 L'utilisation des résultats d'évaluations par les gestionnaires de programmes : Mesure dans laquelle les gestionnaires des programme locaux utilisent les conclusions de recherche et d'évaluations pour améliorer ces programmes en suivant les recommandations émises.

	1	2	3	4	5	6	7	8	9	10	Je ne sais pas
1-Plus faible -> Plus élevé-10	○	○	○	○	○	○	○	○	○	○	○

* MCH 4.10 L'utilisation des résultats d'évaluation par les ministères : Mesure dans laquelle les administrateurs ministériels compétents et responsables d'élaborer les politiques utilisent systématiquement les données pour informer les politiques et interventions visant à résoudre les problèmes liés à la SMI.

	1	2	3	4	5	6	7	8	9	10	Je ne sais pas
1-Plus faible -> Plus élevé-10	○	○	○	○	○	○	○	○	○	○	○

* MCH 4.11 La dissémination d'informations à d'autres entités chargées de la mise en œuvre des programmes : Mesure dans laquelle les informations sont communiquées ou disséminées entre différentes aires géographiques et à différents niveaux (national, état/province, collectivités territoriales).

	1	2	3	4	5	6	7	8	9	10	Je ne sais pas
1-Plus faible -> Plus élevé-10	○	○	○	○	○	○	○	○	○	○	○

MCH 4.12 Commentaires : Utilisez cette section pour apporter des commentaires additionnels sur le niveau d’effort, les défis et les succès du pays autour de la recherche sur la SMI. Si vous avez répondu « Je ne sais pas » à l’une des questions précédentes, utilisez aussi cette section pour expliquer pourquoi.

JOHNS HOPKINS
BLOOMBERG SCHOOL of PUBLIC HEALTH

Bill & Melinda Gates Institute for Population and Reproductive Health

Enquête de l'Indice des efforts pour le dividende démographique

Domaine 5. Organisations de la société civile (OSC)

* MCH 5.1 Le pouvoir des acteurs des OSC : Mesure dans laquelle les acteurs des OSC occupent des postes d'influence dans les institutions, les groupes de travail techniques ou les réseaux concernés par la SMI.

	1	2	3	4	5	6	7	8	9	10	Je ne sais pas
1-Plus faible -> Plus élevé-10	○	○	○	○	○	○	○	○	○	○	○

* MCH 5.2 L'analyse budgétaire comme outil des OSC : Mesure dans laquelle les OSC utilisent l'analyse budgétaire comme un outil pour développer le plaidoyer en matière de SMI.

	1	2	3	4	5	6	7	8	9	10	Je ne sais pas
1-Plus faible -> Plus élevé-10	○	○	○	○	○	○	○	○	○	○	○

* MCH 5.3 Le soutien à la prestation de services en structure sanitaire : Mesure dans laquelle les OSC sont impliquées dans des efforts visant à augmenter l'utilisation et/ou la qualité des services, particulièrement à travers l'augmentation des capacités techniques et/ou opérationnelles des cliniques partenaires des OSC afin de fournir des services de SMI.

	1	2	3	4	5	6	7	8	9	10	Je ne sais pas
1-Plus faible -> Plus élevé-10	○	○	○	○	○	○	○	○	○	○	○

* MCH 5.4 Le soutien à la prestation des services à base communautaire : Mesure dans laquelle les OSC participent au rapprochement des informations et services de SMI avec les communautés à travers des activités comprenant le renforcement de compétences des agents de santé communautaire (ASC), l'expansion de la gamme de services SMI offerts par les ASC, l'orientation/éducation des travailleurs de la santé de cadres inférieurs et/ou des campagnes d'information ou d'éducation.

	1	2	3	4	5	6	7	8	9	10	Je ne sais pas
1-Plus faible -> Plus élevé-10	○	○	○	○	○	○	○	○	○	○	○

* MCH 5.5 Le soutien à la sensibilisation mobile pour l'offre de services à base communautaire : Mesure dans laquelle les OSC soutiennent les modèles de sensibilisation mobile pour équiper les prestataires de santé en leur fournissant des commodités et produits liés à la SMI, de l'équipement et des véhicules afin de fournir en tant qu'équipe spécialiste des services SMI directement aux communautés.

	1	2	3	4	5	6	7	8	9	10	Je ne sais pas
1-Plus faible -> Plus élevé-10	○	○	○	○	○	○	○	○	○	○	○

* MCH 5.6 La franchise sociale : Mesure dans laquelle les OSC sont impliquées dans des opportunités/ services de franchise sociale pour offrir des services de SMI dans le secteur privé à un prix réduit.

	1	2	3	4	5	6	7	8	9	10	Je ne sais pas
1-Plus faible -> Plus élevé-10	○	○	○	○	○	○	○	○	○	○	○

* MCH 5.7 La santé mobile : Mesure dans laquelle les OSC mettent à profit la technologie mobile en utilisant la messagerie (textos) et autres technologies mobiles pour améliorer l'accès aux informations et services de SMI.

	1	2	3	4	5	6	7	8	9	10	Je ne sais pas
1-Plus faible -> Plus élevé-10	○	○	○	○	○	○	○	○	○	○	○

* MCH 5.8 Les droits humains et la qualité des soins : Mesure dans laquelle les OSC soutiennent et plaident pour des approches fondées sur le droit pour s'assurer que les clientes de la SMI soient bien traitées, n'aient pas peur de poser des questions et reçoivent du conseil de qualité, entre autres sujets.

	1	2	3	4	5	6	7	8	9	10	Je ne sais pas
1-Plus faible -> Plus élevé-10	○	○	○	○	○	○	○	○	○	○	○

* MCH 5.9 Le changement social et des comportements (CSC) : Mesure dans laquelle les OSC soutiennent les interventions pour le CSC comprenant, mais sans s'y limiter, les compétences de vie et l'éducation entre pairs, l'éducation sur la santé de l'adulte, l'éducation en milieu scolaire, la communication au sein du couple, la mobilisation communautaire, les interventions à travers les gardiens de la communauté et/ou dans les médias de masse pour le grand public.

	1	2	3	4	5	6	7	8	9	10	Je ne sais pas
1-Plus faible -> Plus élevé-10	○	○	○	○	○	○	○	○	○	○	○

* MCH 5.10 Le plaidoyer/ la redevabilité : Mesure dans laquelle les OSC soutiennent la formation et le renforcement de compétences des plaideurs et des membres de la communauté pour améliorer leur compréhension des politiques et processus, leurs activités d'identification de problèmes, la résolution collaborative de ces problèmes, et des actions de plaidoyer plus ciblées pour améliorer la SMI.

	1	2	3	4	5	6	7	8	9	10	Je ne sais pas
1-Plus faible -> Plus élevé-10	○	○	○	○	○	○	○	○	○	○	○

* MCH 5.11 L'évaluation et le suivi dirigés par les OSC : Mesure dans laquelle les OSC évaluent, suivent et émettent des rapports sur l'efficacité des politiques et programmes pour améliorer la redevabilité des prestataires et des politiques de SMI.

	1	2	3	4	5	6	7	8	9	10	Je ne sais pas
1-Plus faible -> Plus élevé-10	○	○	○	○	○	○	○	○	○	○	○

* MCH 5.12 Les partenariats entre OSC : Mesure dans laquelle les OSC ont formé des alliances nationales et des partenariats régionaux pour renforcer leur position et potentiellement améliorer la force du leadership et les mécanismes de financement pour la SMI.

	1	2	3	4	5	6	7	8	9	10	Je ne sais pas
1-Plus faible -> Plus élevé-10	○	○	○	○	○	○	○	○	○	○	○

MCH 5.13 Commentaires : Utilisez cette section pour apporter des commentaires additionnels sur le niveau d'effort, les défis et les succès des OSC travaillant pour la SMI. Si vous avez répondu « Je ne sais pas » à l'une des questions précédentes, utilisez aussi cette section pour expliquer pourquoi.

JOHNS HOPKINS
BLOOMBERG SCHOOL
of PUBLIC HEALTH

Bill & Melinda Gates Institute for Population and Reproductive Health

Enquête de l'Indice des efforts pour le dividende démographique

Module : Résilience et durabilité du secteur

Étant donné l'impact sanitaire et socioéconomique de la pandémie de COVID-19, et son impact probable sur la progression vers le DD, cet indice des efforts pour le DD intègre des questions pour évaluer la résilience et la durabilité des systèmes dans les secteurs clés du DD. Les éléments couverts par les questions liées à la pandémie de COVID-19 ci-dessous relèvent des dimensions clés des systèmes résilients et visent à apporter des informations déterminantes sur le potentiel d'une réponse efficace aux menaces de maladies infectieuses émergeantes et autres crises de santé publique.

Veuillez noter chaque élément sur une échelle de 1 à 10, 1 étant le score le plus faible (faible état/capacité) et 10 le plus élevé (très bon état/capacité).

* Dimension physique : structures, équipements, états du système et capacités.

	1	2	3	4	5	6	7	8	9	10	Je ne sais pas
MCH-M1 - Planification/Préparation – État et capacité de l'équipement, du personnel et de la structure du programme de SMI avant la crise.	○	○	○	○	○	○	○	○	○	○	○
MCH-M2 – Absorption – Intégration : Niveau d'intégration des services et programmes de SMI pour mitiger les effets de la pandémie de COVID-19 sur la SMI.	○	○	○	○	○	○	○	○	○	○	○

	1	2	3	4	5	6	7	8	9	10	Je ne sais pas
MCH-M3 - Absorption – Intégration : Mesure dans laquelle la gestion efficace de la chaîne d'approvisionnement a permis de mitiger les effets de la pandémie de COVID-19 sur l'approvisionnement/livraison des commodités liées à la SMI.	◯	◯	◯	◯	◯	◯	◯	◯	◯	◯	◯
MCH-M4 - Récupération – Mesure dans laquelle le programme de SMI a été capable d'induire des changements pour récupérer un niveau de fonctionnalité pre-COVID-19.	◯	◯	◯	◯	◯	◯	◯	◯	◯	◯	◯
MCH-M5 - Récupération – Gestion du financement et des bailleurs de fonds : Niveau de mobilisation de ressources et d'allocation à la mitigation des effets de la pandémie de COVID-19 sur la SMI.	◯	◯	◯	◯	◯	◯	◯	◯	◯	◯	◯
MCH-M6 - Adaptation – Mesure dans laquelle des changements ont été effectués pour améliorer la résilience du secteur SMI.	◯	◯	◯	◯	◯	◯	◯	◯	◯	◯	◯

* Dimension informatique : création, gestion, stockage et utilisation des données.

	1	2	3	4	5	6	7	8	9	10	Don't know
MCH-M7 - Planification/Préparation - Mesure dans laquelle les données liées à la SMI étaient suffisamment préparées, présentées, analysées et stockées avant la crise.	○	○	○	○	○	○	○	○	○	○	○
MCH-M8 - Absorption – Connaissance/Conscience : Mesure dans laquelle la capacité de surveillance a pu détecter les limites/menaces posées par la pandémie de COVID-19 à la SMI.	○	○	○	○	○	○	○	○	○	○	○
MCH-M9 - Récupération – Mesure dans laquelle les données de SMI ont été utilisées pour suivre le progrès vers la récupération et anticiper des scenarii de récupération.	○	○	○	○	○	○	○	○	○	○	○
MCH-M10 - Adaptation – Mesure dans laquelle le système de SMI crée et améliore actuellement ses protocoles de stockage et d'utilisation de ses données en temps réel.	○	○	○	○	○	○	○	○	○	○	○

* Dimension cognitive: compréhension, modèles cognitifs, préconception, biais et valeurs.

	1	2	3	4	5	6	7	8	9	10	Je ne sais pas
MCH-M11 - Planification/Préparation - Mesure dans laquelle les décisions de conceptualisation et opérationnelle du secteur SMI étaient préparées à anticiper la crise.	○	○	○	○	○	○	○	○	○	○	○
MCH-M12 - Absorption - Mesure dans laquelle le secteur de la SMI a des protocoles de contingence suffisants et une gestion de crise proactive.	○	○	○	○	○	○	○	○	○	○	○

	1	2	3	4	5	6	7	8	9	10	Je ne sais pas
MCH-M13 - Absorption - Redevabilité: Niveau d'effort pour assurer une redevabiilté optimale vis-à-vis des ressources allouées en soutien à la SMI dans le cadre de la réponse à la pandémie de COVID-19.	○	○	○	○	○	○	○	○	○	○	○
MCH-M14 - Récupération - Mesure dans laquelle les décisions sont orientées vers la récupération, et communiquées sur la base de données probantes pour promouvoir des comportements sûrs auprès des membres de la communauté.	○	○	○	○	○	○	○	○	○	○	○
MCH-M15 - Récupération - Connaissance/Conscience: Mesure dans laquelle le secteur SMI établit une certaine confiance à travers ses communications concernant les barrières posées par la pandémie de COVID-19 pour soutenir l'accès et l'utilisation des services et programmes de SMI.	○	○	○	○	○	○	○	○	○	○	○
MCH-M16 - Adaptation – Adaptif : Mesure dans laquelle les dirigeants nationaux avaient l'autorité d'effectuer des changements pour s'adapter et répondre aux menaces posées par la pandémie de COVID-19 aux services et programmes de SMI.	○	○	○	○	○	○	○	○	○	○	○

* Dimension sociale : interaction, collaboration et auto-synchronisation entre les personnes, les entités et les institutions.

	1	2	3	4	5	6	7	8	9	10	Je ne sais pas
MCH-M17 - Planification/Préparation – Mesure dans laquelle une formation sur la gestion de crise/épidémie a été menée et a permis de mettre à profit les réseaux sociaux, le capital social et institutionnel, et les normes culturelles avant la crise.	○	○	○	○	○	○	○	○	○	○	○
MCH- M18 - Absorption – Mesure dans laquelle le personnel et les institutions sociales ont fait preuve d'ingéniosité et ont été accessibles pendant la réponse à l'épidémie/crise.	○	○	○	○	○	○	○	○	○	○	○
MCH-M19 - Absorption – Autorégulation : Mesure dans laquelle les dirigeants nationaux avaient l'autorité d'effectuer des changements sectoriels en temps opportun à travers une infrastructure flexible.	○	○	○	○	○	○	○	○	○	○	○
MCH-M20 - Récupération – Mesure dans laquelle le secteur SMI est engagé dans le partage de connaissances et d'équipes pour améliorer la récupération systémique.	○	○	○	○	○	○	○	○	○	○	○
MCH-M21 - Récupération – Diversité : Niveau d'engagement d'une équipe multidisciplinaire pour mitiger les effets de la pandémie de COVID-19 sur la SMI.	○	○	○	○	○	○	○	○	○	○	○

66

	1	2	3	4	5	6	7	8	9	10	Je ne sais pas
MCH-M22 - Adaptation – Mesure dans laquelle des ajouts ou modifications ont été apportés aux institutions, politiques, programmes, formations et cultures de SMI.	◯	◯	◯	◯	◯	◯	◯	◯	◯	◯	◯
MCH-M23 - Adaptation - Leadership et management : Niveau de leadership démontré par les dirigeants de la SMI pour mitiger les effets de la pandémie de COVID-19 sur la SMI.	◯	◯	◯	◯	◯	◯	◯	◯	◯	◯	◯

* MCH M-24 Temporalité : Mesure de la rapidité de la réaction du gouvernement –sa communication et mise en œuvre des mesures- pour mitiger l'impact immédiat et sur le long terme de la pandémie de COVID-19. Cette question porte sur la riposte nationale dans son ensemble, et non la réponse spécifique de votre secteur.

	1	2	3	4	5	6	7	8	9	10	Je ne sais pas
1-Plus faible -> Plus élevé-10	◯	◯	◯	◯	◯	◯	◯	◯	◯	◯	◯

MCH M-25 Commentaires : Utilisez cette section pour apporter tout commentaire additionnel sur la résilience et la durabilité du système nationaux de SMI en vous référant à votre expérience de la pandémie de COVID-19. Si vous avez répondu « Je ne sais pas » à l'une des questions, utilisez cette section pour expliquer pourquoi

JOHNS HOPKINS
BLOOMBERG SCHOOL
of PUBLIC HEALTH

Bill & Melinda Gates Institute for Population and Reproductive Health

Enquête de l'Indice des efforts pour le dividende démographique

Questionnaire sur l'éducation (ED)

Répondez aux questions suivantes en fonction de votre expérience/ expertise dans ce secteur spécifique.

Afin d'obtenir une représentation synthétique des efforts nationaux pour le dividende démographique, notez les éléments suivants sur une échelle de 1 à 10, 1 étant le score le plus faible (efforts très faibles ou quasi-inexistants) et 10 le plus élevé (efforts robustes). Le cas échéant, si une politique ou activité n'existe pas, répondez 0.

Donnez un score à chaque élément. Toutes les réponses seront enregistrées au format illustré ci-dessous :

Effort le plus faible	1	2	3	4	5	6	7	8	9	10	Effort le plus élevé	Je ne sais pas

JOHNS HOPKINS
BLOOMBERG SCHOOL of PUBLIC HEALTH

Bill & Melinda Gates Institute for Population and Reproductive Health

Enquête de l’Indice des efforts pour le dividende démographique

Domaine 1. Politique/ Prise de décision

* ED 1.1 La qualité : Mesure dans laquelle il existe un plan/stratégie/programme pour améliorer la qualité de l’enseignement en milieu scolaire.

	1	2	3	4	5	6	7	8	9	10	Je ne sais pas
1-Plus faible -> Plus élevé - 10	◯	◯	◯	◯	◯	◯	◯	◯	◯	◯	◯

* ED 1.2 Les groupes défavorisés :

	1	2	3	4	5	6	7	8	9	10	Je ne sais pas
ED 1.2.1. Mesure dans laquelle les stratégies ont été développées et mises en œuvre/déployées pour surmonter les obstacles d’apprentissage chez les filles enceintes et les mères adolescentes	◯	◯	◯	◯	◯	◯	◯	◯	◯	◯	◯
ED 1.2.2 Mesure dans laquelle des stratégies ont été développées et mises en œuvre/déployées pour surmonter les obstacles d’apprentissage chez les personnes souffrant d’un handicap	◯	◯	◯	◯	◯	◯	◯	◯	◯	◯	◯

* ED 1.3 Les enseignants : Mesure dans laquelle les problèmes liés au recrutement, développement et management du personnel enseignant sont inclus à la politique visant à améliorer les résultats d'apprentissage.

	1	2	3	4	5	6	7	8	9	10	Je ne sais pas
1-Plus faible->Plus élevé-10	○	○	○	○	○	○	○	○	○	○	○

* ED 1.4 La redevabilité des enseignants : Mesure dans laquelle les plans nationaux incluent des mesures spécifiques, dont le suivi et évaluation, visant à rendre les enseignants redevables de l'amélioration de l'apprentissage.

	1	2	3	4	5	6	7	8	9	10	Je ne sais pas
1-Plus faible -> Plus élevé - 10	○	○	○	○	○	○	○	○	○	○	○

* ED 1.5 La qualité des enseignants : Mesure dans laquelle les politiques/stratégies/programmes d'éducation incluent des indicateurs vérifiables de la qualité des enseignants.

	1	2	3	4	5	6	7	8	9	10	Je ne sais pas
1-Plus faible -> Plus élevé - 10	○	○	○	○	○	○	○	○	○	○	○

* ED 1.6 La collaboration dans l'éducation : Mesure dans laquelle la politique prévoit des dispositions pour engager plusieurs parties prenantes dans l'éducation (ex : écoles, communautés du milieu scolaire, parents, enseignants, gouvernement local, associations de parents d'élèves-élèves-enseignants, etc.).

	1	2	3	4	5	6	7	8	9	10	Je ne sais pas
1-Plus faible -> Plus élevé - 10	○	○	○	○	○	○	○	○	○	○	○

* ED 1.7 L'ODD sur la parité : Mesure dans laquelle les politiques/plans/stratégies nationales tiennent compte de l'Objectif de Développement Durable (ODD) sur la parité totale à tous les niveaux d'enseignement.

	1	2	3	4	5	6	7	8	9	10	Je ne sais pas
1-Plus faible -> Plus élevé - 10	○	○	○	○	○	○	○	○	○	○	○

* ED 1.8 Le financement de l'éducation : Mesure dans laquelle les nouveaux plans d'éducation incluent des propositions financières avec des budgets alloués aux réformes de l'enseignement et de l'apprentissage.

	1	2	3	4	5	6	7	8	9	10	Je ne sais pas
1-Plus faible -> Plus élevé - 10	○	○	○	○	○	○	○	○	○	○	○

Rusatira JC, *et al. BMJ Open* 2023; 13:e059937. doi: 10.1136/bmjopen-2021-059937

* ED 1.9 La planification de la parité : Mesure dans laquelle il existe des plans/stratégies nationales pour atteindre 100% d'inscriptions avec parité en :

	1	2	3	4	5	6	7	8	9	10	Je ne sais pas
ED 1.9.1 Ecole primaire	○	○	○	○	○	○	○	○	○	○	○
ED 1.9.2 Ecole secondaire	○	○	○	○	○	○	○	○	○	○	○
ED 1.9.3 Ecole tertiaire/supérieure	○	○	○	○	○	○	○	○	○	○	○

* ED 1.10 L'analphabétisme chez l'adulte : Mesure dans laquelle des politiques/plans/stratégies nationales visent à répondre à l'analphabétisme.

	1	2	3	4	5	6	7	8	9	10	Je ne sais pas
ED 1.10.1 Mesure dans laquelle les politiques/plans/stratégies nationales visent à répondre à l'analphabétisme chez l'adulte.	○	○	○	○	○	○	○	○	○	○	○
ED 1.10.2 Mesure dans laquelle les politiques/plans/stratégies nationales visant à répondre à l'analphabétisme chez l'adulte visent aussi à résorber les inégalités entre les sexes.	○	○	○	○	○	○	○	○	○	○	○

* ED 1.11 Les enfants déscolarisés : Mesure dans laquelle des politiques répondent à la détresse des enfants déscolarisés pour les réintégrer au système scolaire ou leur offrir des opportunités alternatives de développement éducatif.

	1	2	3	4	5	6	7	8	9	10	Je ne sais pas
1-Plus faible -> Plus élevé - 10	○	○	○	○	○	○	○	○	○	○	○

* ED 1.12 L'intégration de la technologie : Mesure dans laquelle des stratégies ont été développées et mises en œuvre/déployées pour intégrer les technologies de l'information et de la communication (TIC) à l'éducation.

	1	2	3	4	5	6	7	8	9	10	Je ne sais pas
1-Plus faible -> Plus élevé - 10	○	○	○	○	○	○	○	○	○	○	○

* ED 1.13. La maîtrise des technologies : Mesure dans laquelle des ressources ont été allouées à l’amélioration de la maîtrise de l’informatique à tous les niveaux d’enseignement.

	1	2	3	4	5	6	7	8	9	10	Je ne sais pas
1-Plus faible -> Plus élevé - 10	◯	◯	◯	◯	◯	◯	◯	◯	◯	◯	◯

ED 1.14. Commentaires : Utilisez cette section pour nous faire part de tout commentaire additionnel sur le niveau d’effort, les défis ou les succès du pays autour des politiques/ de la prise de décision dans le secteur de l’éducation. Si vous avez répondu « Je ne sais pas » à l’une des questions précédentes, veuillez utiliser cette section pour expliquer pourquoi.

JOHNS HOPKINS
BLOOMBERG SCHOOL of PUBLIC HEALTH

Bill & Melinda Gates Institute for Population and Reproductive Health

Enquête de l’Indice des efforts pour le dividende démographique

Domaine 2. Services ou programmes

* ED 2.1 Le déploiement des enseignants : Mesure dans laquelle des stratégies/plans sont utilisés pour veiller à ce que les enseignants soient déployés dans les endroits du pays qui en ont le plus besoin.

	1	2	3	4	5	6	7	8	9	10	Je ne sais pas
1-Plus faible -> Plus élevé-10	○	○	○	○	○	○	○	○	○	○	○

* ED 2.2 La qualité des enseignants : Mesure dans laquelle la qualité des enseignants est reconnue comme un facteur clé pour améliorer les résultats d’apprentissage.

	1	2	3	4	5	6	7	8	9	10	Je ne sais pas
ED 2.2.1 Mesure dans laquelle la qualité de l’enseignement est soulignée dans les politiques/ plans/ stratégies éducatives plus générales.	○	○	○	○	○	○	○	○	○	○	○
ED 2.2.2 Mesure dans laquelle la qualité de la formation et du développement professionnel continu des enseignants est soulignée dans les politiques/ plans/ stratégies éducatives plus générales.	○	○	○	○	○	○	○	○	○	○	○

* ED 2.3 La parité : Mesure dans laquelle les ressources ont été utilisées pour améliorer la parité dans l’éducation entre les garçons et les filles.

	1	2	3	4	5	6	7	8	9	10	Je ne sais pas
1-Plus faible -> Plus élevé-10	○	○	○	○	○	○	○	○	○	○	○

* ED 2.4 L’implication des parents dans l’éducation : Mesure dans laquelle les parents sont impliqués dans le développement et l’exécution des stratégies éducatives (c’est-à-dire à travers l’implication des comités de parents d’élèves et d’enseignants).

	1	2	3	4	5	6	7	8	9	10	Je ne sais pas
1-Plus faible -> Plus élevé-10	○	○	○	○	○	○	○	○	○	○	○

* ED 2.5 Les évaluations standardisées : Mesure dans laquelle toutes les écoles utilisent des évaluations standardisées des écoles et des élèves/étudiants en accord avec les instructions émises par le Ministère de l’Éducation.

	1	2	3	4	5	6	7	8	9	10	Je ne sais pas
1-Plus faible -> Plus élevé-10	○	○	○	○	○	○	○	○	○	○	○

* ED 2.6. L’utilisation des résultats d’évaluation pour informer le progrès : Mesure dans laquelle des ajustements sont apportés aux écoles en fonction des résultats des évaluations scolaires nationales standardisées.

	1	2	3	4	5	6	7	8	9	10	Je ne sais pas
1-Plus faible -> Plus élevé-10	○	○	○	○	○	○	○	○	○	○	○

ED 2.7 Commentaires : Veuillez utiliser cette section pour nous faire part de tout commentaire additionnel sur le niveau d’effort, les défis ou les succès du pays autour des services/ programmes dans le secteur de l’éducation. Si vous avez répondu « Je ne sais pas » à l’une des questions précédentes, veuillez utiliser cette section pour expliquer pourquoi.

JOHNS HOPKINS
BLOOMBERG SCHOOL
of PUBLIC HEALTH

Bill & Melinda Gates Institute for Population and Reproductive Health

Enquête de l'Indice des efforts pour le dividende démographique

Domaine 3. Plaidoyer

* ED 3.1 L'égalité : Mesure dans laquelle l'égalité et l'éducation équitable ont été soulignés par les plaideurs.

	1	2	3	4	5	6	7	8	9	10	Je ne sais pas
1-Plus faible -> Plus élevé-10	○	○	○	○	○	○	○	○	○	○	○

* ED 3.2 La qualité : Mesure dans laquelle la qualité de l'éducation est intégrée aux évaluations liées au plaidoyer pour le système éducatif.

	1	2	3	4	5	6	7	8	9	10	Je ne sais pas
1-Plus faible -> Plus élevé-10	○	○	○	○	○	○	○	○	○	○	○

* ED 3.3 Les obstacles des enseignants : Mesure dans laquelle les besoins des enseignants ont été mis en évidence par les plaideurs.

	1	2	3	4	5	6	7	8	9	10	Je ne sais pas
1-Plus faible -> Plus élevé-10	○	○	○	○	○	○	○	○	○	○	○

* ED 3.4 Les obstacles des institutions : Mesure dans laquelle les difficultés des institutions éducatives ont été mises en évidence dans les différents efforts de plaidoyer.

	1	2	3	4	5	6	7	8	9	10	Je ne sais pas
1-Plus faible -> Plus élevé-10	○	○	○	○	○	○	○	○	○	○	○

ED 3.5 Commentaires : Veuillez utiliser cette section pour nous faire part de tout commentaire additionnel sur le niveau d'effort, les défis ou les succès du pays autour du plaidoyer dans le secteur de l'éducation. Si vous avez répondu « Je ne sais pas » à l'une des questions précédentes, veuillez utiliser cette section pour expliquer pourquoi.

JOHNS HOPKINS
BLOOMBERG SCHOOL
of PUBLIC HEALTH

Bill & Melinda Gates Institute for Population and Reproductive Health

Enquête de l'Indice des efforts pour le dividende démographique

Domaine 4. Recherche

* ED 4.1 La force des observatoires/parties prenantes/groupes de travail pour l'ED : Mesure dans laquelle les parties prenantes du secteur de l'éducation du pays (ex : groupe directif de parties prenantes, groupes de travail techniques, observatoire de l'ED, groupe de coordination ou facilitation) :

	1	2	3	4	5	6	7	8	9	10	Je ne sais pas
ED 4.1.1 Ont un représentant du gouvernement, des institutions de formation, de la société civile, des organisations non gouvernementales et confessionnelles, des associations professionnelles, du secteur privé, etc.	○	○	○	○	○	○	○	○	○	○	○
ED 4.1.2 Se réunissent régulièrement, émettent des rapports et recommandent des politiques aux directions des ministères compétents.	○	○	○	○	○	○	○	○	○	○	○
ED 4.1.3 Ont un impact sur l'éducation dans le pays.	○	○	○	○	○	○	○	○	○	○	○

* ED 4.2 La stratégie d'éducation/recherche : Mesure dans laquelle le plan d'éducation nationale et/ou d'autres documents nationaux comprennent une stratégie/approche complète de recherche liée à l'éducation, partagée entre partenaires.

	1	2	3	4	5	6	7	8	9	10	Je ne sais pas
1-Plus faible -> Plus élevé-10	○	○	○	○	○	○	○	○	○	○	○

* ED 4.3 Les partenaires collectant les données :

	1	2	3	4	5	6	7	8	9	10	Je ne sais pas
ED 4.3.1 Mesure dans laquelle la collecte des données est entreprise par des agences gouvernementales. (Cela peut comprendre les agences/bureaux de la statistique et/ou les ministères de l'économie/finances, éducation, santé, genre, etc.)	○	○	○	○	○	○	○	○	○	○	○
ED 4.3.2 Mesure dans laquelle la collecte de données est entreprise par des institutions de recherche.	○	○	○	○	○	○	○	○	○	○	○
ED 4.3.3 Mesure dans laquelle la collecte des données est entreprise par des chercheurs indépendants.	○	○	○	○	○	○	○	○	○	○	○

* ED 4.4 La recherche thématique :

	1	2	3	4	5	6	7	8	9	10	Je ne sais pas
ED 4.4.1 Mesure dans laquelle de la recherche est menée sur les inégalités de genre dans l'éducation.	○	○	○	○	○	○	○	○	○	○	○
ED 4.4.2 Mesure dans laquelle de la recherche est menée sur la qualité de l'enseignement.	○	○	○	○	○	○	○	○	○	○	○
ED 4.4.3 Mesure dans laquelle de la recherche est menée sur les obstacles auxquels font face les enseignants.	○	○	○	○	○	○	○	○	○	○	○
ED 4.4.4 Mesure dans laquelle de la recherche est menée sur les obstacles auxquels font face les institutions.	○	○	○	○	○	○	○	○	○	○	○

* ED 4.5 La qualité/couverture des données : Mesure dans laquelle la recherche/les données actuelles sont désagrégées par sexe et par âge. Mesure dans laquelle le pays participe aux tests standardisés et son classement.

	1	2	3	4	5	6	7	8	9	10	Je ne sais pas
ED 4.5.1 Mesure dans laquelle un système statistique de routine (utilisant les informations des écoles/institutions) offre de bonnes informations régulières sur les produits et les établissements	○	○	○	○	○	○	○	○	○	○	○
ED 4.5.2 Mesure dans laquelle un système statistique de routine (utilisant les informations des écoles/institutions) offre de bonnes informations régulières sur les services et le personnel du secteur éducatif	○	○	○	○	○	○	○	○	○	○	○
ED 4.5.3 Mesure dans laquelle un système statistique de routine offre de bonnes informations régulières sur les besoins éducatifs des populations de différentes communautés	○	○	○	○	○	○	○	○	○	○	○
ED 4.5.4 Mesure dans laquelle des évaluations du système de suivi de l'éducation sont menées et appliquées pour assurer la fiabilité des données	○	○	○	○	○	○	○	○	○	○	○

* ED 4.6 Les registres : Mesure dans laquelle les systèmes de registres, rapports et feedback sur les résultats de l'éducation sont adéquats.

	1	2	3	4	5	6	7	8	9	10	Je ne sais pas
1-Plus faible -> Plus élevé-10	○	○	○	○	○	○	○	○	○	○	○

* ED 4.7 La qualité des institutions de recherche : Mesure dans laquelle le pays a la capacité de soutenir et maintenir des institutions de recherche qui développent des études et/ou collectent des données permettant d'informer les efforts pour l'amélioration de l'éducation/ l'enseignement.

	1	2	3	4	5	6	7	8	9	10	Je ne sais pas
1-Plus faible -> Plus élevé-10	○	○	○	○	○	○	○	○	○	○	○

* ED 4.8 L'évaluation : Mesure dans laquelle les statistiques, enquêtes et études sur le programme d'éducation sont utilisées par du personnel spécialisé pour rapporter les opérations du programme et mesurer le progrès.

	1	2	3	4	5	6	7	8	9	10	Je ne sais pas
1-Plus faible -> Plus élevé-10	○	○	○	○	○	○	○	○	○	○	○

* ED 4.9 L'utilisation des résultats d'évaluation par les gestionnaires de programmes : Mesure dans laquelle les gestionnaires locaux du programme d'éducation utilisent les conclusions de recherche et d'évaluations pour améliorer ces programmes en suivant les recommandations émises.

	1	2	3	4	5	6	7	8	9	10	Je ne sais pas
1-Plus faible -> Plus élevé - 10	○	○	○	○	○	○	○	○	○	○	○

* ED 4.10 L'utilisation des résultats d'évaluation par les ministères : Mesure dans laquelle les administrateurs ministériels compétents (soit le Ministère de l'Éducation) utilisent systématiquement les données pour informer les politiques et interventions visant à résoudre les problèmes éducatifs.

	1	2	3	4	5	6	7	8	9	10	Je ne sais pas
1-Plus faible -> Plus élevé-10	○	○	○	○	○	○	○	○	○	○	○

* ED 4.11 La dissémination d'informations à d'autres entités chargées de la mise en œuvre des programmes :

	1	2	3	4	5	6	7	8	9	10	Je ne sais pas
ED 4.11.1 Mesure dans laquelle des informations sont partagées ou disséminées entres géographies/ régions.	○	○	○	○	○	○	○	○	○	○	○
ED. 4.11.2 Mesure dans laquelle des informations sont partagées ou disséminées à différents niveaux (national, états/provinces, localités).	○	○	○	○	○	○	○	○	○	○	○

Rusatira JC, *et al. BMJ Open* 2023; 13:e059937. doi: 10.1136/bmjopen-2021-059937

ED 4.12 Commentaires : Veuillez utiliser cette section pour nous faire part de tout commentaire additionnel sur le niveau d'effort, les défis ou les succès du pays autour de la recherche dans le secteur de l'éducation. Si vous avez répondu « Je ne sais pas » à l'une des questions précédentes, veuillez utiliser cette section pour expliquer pourquoi.

JOHNS HOPKINS
BLOOMBERG SCHOOL
of PUBLIC HEALTH

Bill & Melinda Gates Institute for Population and Reproductive Health

Enquête de l'Indice des efforts pour le dividende démographique

Domaine 5. Organisations de la société civile (OSC)

* ED 5.1 Le pouvoir des acteurs des OSC : Mesure dans laquelle les acteurs des OSC occupent des postes d'influence dans les institutions, les groupes de travail techniques ou les réseaux concernés par l'éducation.

	1	2	3	4	5	6	7	8	9	10	Je ne sais pas
1-Plus faible -> Plus élevé-10	○	○	○	○	○	○	○	○	○	○	○

* ED 5.2 L'analyse budgétaire comme outil des OSC : Mesure dans laquelle les OSC utilisent l'analyse budgétaire comme un outil pour développer le plaidoyer en matière d'éducation.

	1	2	3	4	5	6	7	8	9	10	Je ne sais pas
1-Plus faible -> Plus élevé-10	○	○	○	○	○	○	○	○	○	○	○

* ED 5.3 L'inclusion communautaire : Mesure dans laquelle les OSC sont impliquées dans les efforts d'exploration d'opportunités pour l'amélioration de la qualité de l'éducation.

	1	2	3	4	5	6	7	8	9	10	Je ne sais pas
1-Plus faible -> Plus élevé-10	○	○	○	○	○	○	○	○	○	○	○

* ED 5.4 L'implication de la communauté dans l'éducation : Mesure dans laquelle les OSC participent au rapprochement des informations et services éducatifs avec les communautés à travers des activités telles que le renforcement des compétences des enseignants et des parents et/ou les campagnes d'information ou d'éducation pour augmenter l'engagement des familles et des communautés en faveur de l'éducation.

	1	2	3	4	5	6	7	8	9	10	Je ne sais pas
1-Plus faible -> Plus élevé-10	○	○	○	○	○	○	○	○	○	○	○

* ED 5.5 La franchise sociale : Mesure dans laquelle les OSC sont impliquées dans la franchise sociale des services/opportunités éducatives de qualité aux familles et enfants à moindre coût.

	1	2	3	4	5	6	7	8	9	10	Je ne sais pas
1-Plus faible -> Plus élevé-10	◯	◯	◯	◯	◯	◯	◯	◯	◯	◯	◯

* ED 5.6 La technologie : Mesure dans laquelle les OSC mettent à profit les technologies de l'information et de la communication (TIC) pour améliorer l'accès à l'éducation de qualité de diverses communautés.

	1	2	3	4	5	6	7	8	9	10	Je ne sais pas
1-Plus faible -> Plus élevé-10	◯	◯	◯	◯	◯	◯	◯	◯	◯	◯	◯

* ED 5.7 Les droits humains, l'accès et la qualité de l'éducation : Mesure dans laquelle les OSC plaident pour le soutien d'approches éducatives basées sur les droits et veillent à ce que les élèves/étudiants soit traités correctement, n'aient pas peur de poser des questions, et reçoivent un enseignement de qualité, entre autres préoccupations.

	1	2	3	4	5	6	7	8	9	10	Je ne sais pas
1-Plus faible -> Plus élevé-10	◯	◯	◯	◯	◯	◯	◯	◯	◯	◯	◯

* ED 5.8 Le changement social et des comportements (CSC) : Mesure dans laquelle les OSC soutiennent les interventions pour le CSC comprenant, mais sans s'y limiter, aux compétences de vie et à l'éducation entre pairs, l'éducation sur le santé de l'adulte, l'éducation en milieu scolaire, la mobilisation communautaire et/ou les interventions médiatiques destinées au grand public.

	1	2	3	4	5	6	7	8	9	10	Je ne sais pas
1-Plus faible -> Plus élevé-10	◯	◯	◯	◯	◯	◯	◯	◯	◯	◯	◯

* ED 5.9 Le plaidoyer/ la redevabilité : Mesure dans laquelle les OSC soutiennent la formation et le renforcement de compétences des plaideurs et des membres de la communauté pour améliorer leur compréhension des politiques et processus, leurs activités d'identification de problèmes, la résolution collaborative de ces problèmes, et des actions de plaidoyer plus ciblées pour améliorer l'éducation.

	1	2	3	4	5	6	7	8	9	10	Je ne sais pas
1-Plus faible -> Plus élevé-10	◯	◯	◯	◯	◯	◯	◯	◯	◯	◯	◯

* ED 5.10 L'évaluation et le suivi dirigés par les OSC : Mesure dans laquelle les OSC évaluent, suivent et émettent des rapports sur l'efficacité des politiques et programmes pour améliorer la redevabilité des enseignants et des politiques d'éducation.

	1	2	3	4	5	6	7	8	9	10	Don't know
1-Plus faible -> Plus élevé-10	◯	◯	◯	◯	◯	◯	◯	◯	◯	◯	◯

* ED 5.11 Les partenariats entre OSC : Mesure dans laquelle les OSC ont formé des alliances nationales et des partenariats régionaux pour renforcer leur position et potentiellement améliorer la force du leadership et les mécanismes de financement en vue d'améliorer la qualité de l'éducation.

	1	2	3	4	5	6	7	8	9	10	Je ne sais pas
1-Plus faible -> Plus élevé-10	◯	◯	◯	◯	◯	◯	◯	◯	◯	◯	◯

ED 5.12 Commentaires : Veuillez utiliser cette section pour nous faire part de tout commentaire additionnel sur le niveau d'effort, les défis ou les succès du pays autour des organisations de la société civile impliquées dans le secteur de l'éducation. Si vous avez répondu « Je ne sais pas » à l'une des questions précédentes, veuillez utiliser cette section pour expliquer pourquoi.

JOHNS HOPKINS
BLOOMBERG SCHOOL
of PUBLIC HEALTH

Bill & Melinda Gates Institute for Population and Reproductive Health

Enquête de l'Indice des efforts pour le dividende démographique

Module : Résilience et durabilité du secteur

Étant donné l'impact sanitaire et socioéconomique de la pandémie de COVID-19, et son impact probable sur la progression vers le DD, cet indice des efforts pour le DD intègre des questions pour évaluer la résilience et la durabilité des systèmes dans les secteurs clés du DD. Les éléments couverts par les questions liées à la pandémie de COVID-19 ci-dessous relèvent des dimensions clés des systèmes résilients et visent à apporter des informations déterminantes sur le potentiel d'une réponse efficace aux menaces de maladies infectieuses émergeantes et autres crises de santé publique.

Veuillez noter chaque élément sur une échelle de 1 à 10, 1 étant le score le plus faible (faible état/capacité) et 10 le plus élevé (très bon état/capacité).

* Dimension physique : structures, équipements, états du système et capacités

	1	2	3	4	5	6	7	8	9	10	Je ne sais pas
ED-M1 - Planification/Préparation – État et capacité de l'équipement, du personnel et de la structure du secteur de l'ED avant la crise. (1 = faible état/capacité; 10 = très bon état/capacité)	◯	◯	◯	◯	◯	◯	◯	◯	◯	◯	◯
ED-M2 - Absorption – Mesure dans laquelle la pandémie de COVID-19 a été reconnue par le secteur de l'ED, et mesure dans laquelle le système a pu continuer d'être fonctionnel	◯	◯	◯	◯	◯	◯	◯	◯	◯	◯	◯

85

	1	2	3	4	5	6	7	8	9	10	Je ne sais pas
ED-M3 - Absorption – Intégration : Niveau d’intégration des services et programmes d’ED pour mitiger les effets de la pandémie de COVID-19 sur l’ED.	○	○	○	○	○	○	○	○	○	○	○
ED-M4 - Récupération – Mesure dans laquelle le secteur de l’ED a été capable d’induire des changements pour récupérer un niveau de fonctionnalité pré-COVID-19.	○	○	○	○	○	○	○	○	○	○	○
ED-M5 - Récupération – Gestion du financement et des bailleurs de fonds : Niveau de mobilisation des ressources et allocations pour mitiger les effets de la pandémie de COVID-19 sur l’ED.	○	○	○	○	○	○	○	○	○	○	○
ED-M6 - Adaptation – Mesure dans laquelle des changements ont été effectués pour améliorer la résilience du secteur de l’ED.	○	○	○	○	○	○	○	○	○	○	○

* Information : création, gestion, stockage et utilisation des données.

	1	2	3	4	5	6	7	8	9	10	Je ne sais pas
ED-M7 - Planification/ Préparation – Mesure dans laquelle les données liées à l'ED étaient suffisamment préparées, présentées, analysées et stockées avant la crise.	○	○	○	○	○	○	○	○	○	○	○
ED-M8 - Absorption - Knowledge: Extent to which the monitoring capacity was able to detect the limits / threats posed to the ED sector by the COVID-19 pandemic. (This includes data on the various key subjects of the education sector at each level of education [primary, secondary, tertiary / higher]).	○	○	○	○	○	○	○	○	○	○	○
ED-M9 - Récupération – Mesure dans laquelle les données de l'ED ont été utilisées pour suivre le progrès vers la récupération et anticiper des scenarii de récupération.	○	○	○	○	○	○	○	○	○	○	○
ED-M10 - Adaptation – Mesure dans laquelle le système d'ED crée et améliore actuellement ses protocoles de stockage et d'utilisation de ses données en temps réel.	○	○	○	○	○	○	○	○	○	○	○

* Dimension cognitive : compréhension, modèles cognitifs, idées reçues, préjugés et valeurs.

	1	2	3	4	5	6	7	8	9	10	Je ne sais pas
ED-M11 – Planification/ Préparation – Mesure dans laquelle le système d'ED et les décisions opérationnelles étaient préparés pour anticiper la crise.	○	○	○	○	○	○	○	○	○	○	○

	1	2	3	4	5	6	7	8	9	10	Je ne sais pas
ED-M12 - Absorption – Mesure dans laquelle la réponse du système d'ED a des protocoles de contingence suffisants et une gestion de crise proactive.	○	○	○	○	○	○	○	○	○	○	○
ED-M13 - Absorption – Redevabilité : Niveau d'effort pour assurer une redevabilité optimale vis-à-vis des ressources allouées à l'ED dans le cadre de la réponse à la pandémie de COVID-19.	○	○	○	○	○	○	○	○	○	○	○
ED-M14 - Récupération – Mesure dans laquelle les décisions sont orientées sur la récupération et communiquées aux communautés sur la base de données probantes pour promouvoir des comportements sûrs.	○	○	○	○	○	○	○	○	○	○	○
ED-M15 - Récupération – Connaissance : Mesure dans laquelle le secteur de l'ED établit une certaine confiance à travers sa communication concernant les barrières posées par la pandémie de COVID-19 pour soutenir l'accès et l'utilisation des services et programmes d'ED.	○	○	○	○	○	○	○	○	○	○	○
ED-M16 - Adaptation – Mesure dans laquelle le secteur de l'ED conçoit de nouvelles configurations systémiques, de nouveaux objectifs et de nouveaux critères de décision.	○	○	○	○	○	○	○	○	○	○	○

	1	2	3	4	5	6	7	8	9	10	Je ne sais pas
ED-M17 – Adaptation : Niveau d'adaptation pour répondre aux menaces posées par la pandémie de COVID-19 à l'ED.	◯	◯	◯	◯	◯	◯	◯	◯	◯	◯	◯

* Social dimension: interaction, collaboration and self-synchronization between people, entities and institutions.

	1	2	3	4	5	6	7	8	9	10	Je ne sais pas
ED-M18 – Planification /Préparation – Mesure dans laquelle une formation sur la gestion d'épidémies/crises a été menée et a permis de mettre à profit les réseaux sociaux, le capital social et les normes institutionnelles et culturelles avant la crise.	◯	◯	◯	◯	◯	◯	◯	◯	◯	◯	◯
ED-M19 - Absorption – Mesure dans laquelle le personnel et les institutions sociales ont été accessibles et ont fait preuve d'ingéniosité dans leur réponse à l'épidémie/crise.	◯	◯	◯	◯	◯	◯	◯	◯	◯	◯	◯
ED-M20 - Absorption - Self-regulation: Extent to which national leaders had the authority to effect timely sectoral changes through an infrastructure flexible.	◯	◯	◯	◯	◯	◯	◯	◯	◯	◯	◯
ED-M21 – Récupération – Mesure dans laquelle le système d'ED est impliqué dans le partage de connaissances et d'équipes pour améliorer la récupération du système.	◯	◯	◯	◯	◯	◯	◯	◯	◯	◯	◯

	1	2	3	4	5	6	7	8	9	10	Je ne sais pas
ED-M22 - Récupération – Diversité : Niveau d’engagement d’une équipe multidisciplinaire pour mitiger les effets de la pandémie de COVID-19 sur l’ED.	○	○	○	○	○	○	○	○	○	○	○
ED-M23 - Adaptation – Mesure dans laquelle des ajouts ou changements sont apportés aux institutions, politiques, formations, programmes et à la culture de l’ED.	○	○	○	○	○	○	○	○	○	○	○
ED-M24 - Adaptation - Leadership et management : Niveau de leadership démontré par les dirigeants du secteur de l’ED pour mitiger les effets de la pandémie de COVID-19 sur l’ED.	○	○	○	○	○	○	○	○	○	○	○

* ED M-25 Temporalité : Mesure de la rapidité de la réaction du gouvernement –sa communication et mise en œuvre des mesures- pour mitiger l’impact immédiat et sur le long terme de la pandémie de COVID-19. Cette question porte sur la riposte nationale dans son ensemble, et non la réponse spécifique de votre secteur.

	1	2	3	4	5	6	7	8	9	10	Je ne sais pas
1-Plus faible -> Plus élevé-10	○	○	○	○	○	○	○	○	○	○	○

ED M-26 Commentaires : Utilisez cette section pour apporter tout commentaire additionnel sur la résilience et la durabilité du système d’éducation nationale en vous référant à votre expérience de la pandémie de COVID-19. Si vous avez répondu « Je ne sais pas » à l’une des questions, utilisez cette section pour expliquer pourquoi.

JOHNS HOPKINS
BLOOMBERG SCHOOL
of PUBLIC HEALTH

Bill & Melinda Gates Institute for Population and Reproductive Health

Enquête de l'Indice des efforts pour le dividende démographique

Questionnaire sur l'autonomisation des femmes (AF)

Répondez aux questions suivantes en fonction de votre expérience/ expertise dans ce secteur spécifique.

Afin d'obtenir une représentation synthétique des efforts nationaux pour le dividende démographique, notez les éléments suivants sur une échelle de 1 à 10, 1 étant le score le plus faible (efforts très faibles ou quasi-inexistants) et 10 le plus élevé (efforts robustes). Le cas échéant, si une politique ou activité n'existe pas, répondez 0.

Donnez un score à chaque élément. Toutes les réponses seront enregistrées au format illustré ci-dessous :

Effort le plus faible	1	2	3	4	5	6	7	8	9	10	Effort le plus élevé	Je ne sais pas

JOHNS HOPKINS
BLOOMBERG SCHOOL
of PUBLIC HEALTH

Bill & Melinda Gates Institute for Population and Reproductive Health

Enquête de l'Indice des efforts pour le dividende démographique

Domaine 1. Politique/ Prise de décision

* WE 1.1 La politique de parité : Mesure dans laquelle la parité est prise en compte/intégrée aux plans de développement du pays (ou aux plans de votre secteur).

	1	2	3	4	5	6	7	8	9	10	Je ne sais pas
1-Plus faible -> Plus élevé-10	○	○	○	○	○	○	○	○	○	○	○

* WE 1.2 Niveau d'engagement envers la Convention sur l'élimination de toutes les formes de discrimination à l'égard des femmes (CEDAW) :

	1	2	3	4	5	6	7	8	9	10	Je ne sais pas
1-Plus faible -> Plus élevé-10	○	○	○	○	○	○	○	○	○	○	○

* WE 1.3 La parité dans les politiques familiales et de mariage : Mesure dans laquelle le principe d'égalité hommes-femmes est incorporé à la constitution nationale et autres législations concernant le mariage et les relations familiales.

	1	2	3	4	5	6	7	8	9	10	Je ne sais pas
1-Plus faible -> Plus élevé-10	○	○	○	○	○	○	○	○	○	○	○

* WE 1.4 L'âge légal du mariage : Mesure dans laquelle l'âge légal du mariage pour les filles est établi à 18 ans ou plus et est appliqué.

	1	2	3	4	5	6	7	8	9	10	Je ne sais pas
1-Plus faible -> Plus élevé-10	○	○	○	○	○	○	○	○	○	○	○

* WE 1.5 Les mesures contre les violences à l'égard des femmes : Niveau des régulations et mesures contre les violences faites aux femmes, dont la violence intraconjugale, le viol, le harcèlement sexuel et la traite des filles et des femmes.

	1	2	3	4	5	6	7	8	9	10	Je ne sais pas
1-Plus faible -> Plus élevé-10	◯	◯	◯	◯	◯	◯	◯	◯	◯	◯	◯

* WE 1.6 La mutilation génitale féminine : Niveau de régulation et mesures contre la mutilation génitale féminine.

	1	2	3	4	5	6	7	8	9	10	Je ne sais pas
1-Plus faible -> Plus élevé-10	◯	◯	◯	◯	◯	◯	◯	◯	◯	◯	◯

* WE 1.7 Les mesures de santé des femmes : Niveau des protocoles de protection de la santé des femmes, dont les infections sexuellement transmissibles, y compris le VIH/SIDA, la mortalité maternelle et la contraception.

	1	2	3	4	5	6	7	8	9	10	Je ne sais pas
1-Plus faible -> Plus élevé-10	◯	◯	◯	◯	◯	◯	◯	◯	◯	◯	◯

* WE 1.8 Les mesures éducatives : Mesure dans laquelle il existe des mécanismes pour promouvoir l'éducation des filles.

	1	2	3	4	5	6	7	8	9	10	Je ne sais pas
WE 1.8.1 Niveau d'application des politiques sur l'éducation des filles.	◯	◯	◯	◯	◯	◯	◯	◯	◯	◯	◯
WE 1.8.2 Mesure dans laquelle des mécanismes sont en place pour prévenir l'échec/l'abandon scolaire des filles.	◯	◯	◯	◯	◯	◯	◯	◯	◯	◯	◯

* WE 1.9 Les mesures relatives à l'exercice des droits : Niveau d'application des politiques sur les droits des femmes.

	1	2	3	4	5	6	7	8	9	10	Je ne sais pas
1-Plus faible -> Plus élevé-10	◯	◯	◯	◯	◯	◯	◯	◯	◯	◯	◯

* WE 1.10 Le travail équitable : Mesure dans laquelle il existe des politiques sur les conditions de travail équitables et l'égalité des salaires entre hommes et femmes.

	1	2	3	4	5	6	7	8	9	10	Je ne sais pas
1-Plus faible -> Plus élevé-10	○	○	○	○	○	○	○	○	○	○	○

* WE 1.11 L'égalité de la propriété de biens/biens fonciers : Mesure dans laquelle il existe des politiques pour affirmer l'accès équitable à la propriété de biens fonciers.

	1	2	3	4	5	6	7	8	9	10	Je ne sais pas
1-Plus faible -> Plus élevé-10	○	○	○	○	○	○	○	○	○	○	○

* WE 1.12 Le conflit, les femmes et le rétablissement de la paix : Niveau de mise en œuvre de la Résolution 1325 du Conseil de sécurité des Nations Unies sur les impacts des conflits sur les femmes et leur rôle dans le rétablissement de la paix.

	1	2	3	4	5	6	7	8	9	10	Je ne sais pas
1-Plus faible -> Plus élevé-10	○	○	○	○	○	○	○	○	○	○	○

* WE 1.13 Les quotas de femmes dans les politiques : Existence et niveau de mise en œuvre des quotas dans les systèmes politiques observant la parité.

	1	2	3	4	5	6	7	8	9	10	Je ne sais pas
WE 1.13.1 Mesure dans laquelle il existe une prise de décision équitable du point de vue du genre, y compris en soutien au quotas électoraux et à l'intégration des femmes dans tous les ministères et départements du gouvernement.	○	○	○	○	○	○	○	○	○	○	○
WE 1.13.2 Mesure dans laquelle les femmes participant et/ou jouent un rôle significatif dans les systèmes traditionnels de gouvernance (ex : chefs de village, etc.).	○	○	○	○	○	○	○	○	○	○	○

94

* WE 1.14 L'Objectif de Développement Durable (ODD) 5 : Niveau d'engagement pour remplir l'Objectif 5 des ODD, pour réaliser la parité et assurer l'autonomisation de toutes les filles et femmes et les filles.

	1	2	3	4	5	6	7	8	9	10	Je ne sais pas
1-Plus faible -> Plus élevé-10	○	○	○	○	○	○	○	○	○	○	○

* WE 1.15 La révision de la loi coutumière : Mesure dans laquelle les lois coutumières ont été revues et modifiées par des politiques/cadres nationaux ; ou mesure dans laquelle les lois coutumières existantes reflètent les efforts pour l'autonomisation des femmes.

	1	2	3	4	5	6	7	8	9	10	Je ne sais pas
1-Plus faible -> Plus élevé-10	○	○	○	○	○	○	○	○	○	○	○

* WE 1.16 La budgétisation pour l'autonomisation des femmes : Mesure dans laquelle la budgétisation et l'allocation budgétaire sont suffisantes pour couvrir les coûts nécessaires à l'atteinte des cibles établies dans le plan national de la femme ou son équivalent.

	1	2	3	4	5	6	7	8	9	10	Je ne sais pas
1-Plus faible -> Plus élevé-10	○	○	○	○	○	○	○	○	○	○	○

* WE 1.17 Les ressources humaines pour l'autonomisation des femmes : Mesure dans laquelle :

	1	2	3	4	5	6	7	8	9	10	Je ne sais pas
WE 1.17.1 Du personnel en quantité et qualité suffisante est employé pour mettre en œuvre les activités liées au genre, y compris les efforts pour former ce personnel	○	○	○	○	○	○	○	○	○	○	○
WE 1.17.2 Des efforts sont menés pour former le personnel de manière appropriée et intégrale.	○	○	○	○	○	○	○	○	○	○	○
WE 1.17.3 Le personnel est suffisamment soutenu pour mener à bien ses tâches.	○	○	○	○	○	○	○	○	○	○	○
WE 1.17.4 Le personnel assigné est situé à un niveau administratif suffisamment élevé pour travailler efficacement.	○	○	○	○	○	○	○	○	○	○	○

* WE 1.18 La transparence et la redevabilité : Mesure dans laquelle le gouvernement fournit aux parties prenantes des informations complètes sur les intentions, plans, programmes et budgets disponibles pour mettre en œuvre les programmes d'autonomisation des femmes et en assurer la redevabilité et la transparence. (*Peuvent être incluses l'adoption et l'application d'une loi sur le droit à l'information, des sessions informatives régulières, par exemple dans les mairies, ou la transmission de ces informations via des brochures ou autres supports de communication en langue locale*).

	1	2	3	4	5	6	7	8	9	10	Je ne sais pas
1-Plus faible -> Plus élevé-10	○	○	○	○	○	○	○	○	○	○	○

WE 1.19 Commentaire : Utilisez cette section pour apporter des commentaires additionnels sur le niveau d'efforts, les défis et les succès du pays autour de la politique/ prise de décision pour l'autonomisation des femmes. Si vous avez répondu « Je ne sais pas » à l'une des questions ci-dessus, utilisez aussi cette section pour expliquer pourquoi.

JOHNS HOPKINS
BLOOMBERG SCHOOL
of PUBLIC HEALTH

Bill & Melinda Gates Institute for Population and Reproductive Health

Enquête de l'Indice des efforts pour le dividende démographique

Domaine 2. Services ou programmes

* WE 2.1 L'intégration des femmes : Mesure dans laquelle les services ou programmes sectoriels et intersectoriels tiennent compte des implications des actions planifiées pour les hommes et pour les femmes.

	1	2	3	4	5	6	7	8	9	10	Je ne sais pas
1-Plus faible -> Plus élevé-10	○	○	○	○	○	○	○	○	○	○	○

* WE 2.2 Les programmes transformateurs de genre : Mesure dans laquelle les services ou programmes sectoriels ou intersectoriels intègrent des principes/ approches transformateurs de genre.

	1	2	3	4	5	6	7	8	9	10	Je ne sais pas
1-Plus faible -> Plus élevé-10	○	○	○	○	○	○	○	○	○	○	○

* WE 2.3 L'éducation : Mesure dans laquelle les services ou programmes éducatifs se focalisent sur l'autonomisation des filles et des femmes dans les salles de classe.

	1	2	3	4	5	6	7	8	9	10	Je ne sais pas
1-Plus faible -> Plus élevé-10	○	○	○	○	○	○	○	○	○	○	○

* WE 2.4. L'abandon scolaire : Mesure dans laquelle il existe des programmes pour prévenir l'abandon scolaire des filles.

	1	2	3	4	5	6	7	8	9	10	Je ne sais pas
1-Plus faible -> Plus élevé-10	○	○	○	○	○	○	○	○	○	○	○

* WE 2.5 La maîtrise des technologies : Mesure dans laquelle la maîtrise des technologies est promue auprès des filles en particulier.

	1	2	3	4	5	6	7	8	9	10	Je ne sais pas
1-Plus faible -> Plus élevé-10	○	○	○	○	○	○	○	○	○	○	○

* WE 2.6 L'emploi : Mesure dans laquelle les programmes de création d'emploi et les politiques du marché du travail visent à garantir l'indépendance économique des femmes et sont conçus/mis en œuvre en conséquence.

	1	2	3	4	5	6	7	8	9	10	Je ne sais pas
1-Plus faible -> Plus élevé-10	○	○	○	○	○	○	○	○	○	○	○

* WE 2.7 Les femmes-mères-travailleuses : Mesure dans laquelle le gouvernement permet aux femmes, à travers la loi, les régulations et les programmes, de combiner leur rôle de mère pendant la grossesse, l'allaitement et l'éducation de leurs enfants à leur participation à la vie active.

	1	2	3	4	5	6	7	8	9	10	Je ne sais pas
1-Plus faible -> Plus élevé-10	○	○	○	○	○	○	○	○	○	○	○

* WE 2.8 La planification familiale pour l'autonomisation des femmes : Mesure dans laquelle les services ou programmes de PF sont mis en œuvre selon une approche fondamentale d'autonomisation des femmes.

	1	2	3	4	5	6	7	8	9	10	Je ne sais pas
1-Plus faible -> Plus élevé-10	○	○	○	○	○	○	○	○	○	○	○

* WE 2.9 La représentation politique et publique : Mesure de la mise en œuvre des programmes visant à améliorer la participation et la représentation équitables des femmes à tous les niveaux du processus politique et de la vie publique.

	1	2	3	4	5	6	7	8	9	10	Je ne sais pas
1-Plus faible -> Plus élevé-10	○	○	○	○	○	○	○	○	○	○	○

* WE 2.10 La violence : Portée de la lutte contre les violences faites à l'égard des femmes et des enfants, et leur élimination à l'échelle du pays (violences intraconjugales, viol, harcèlement sexuel, traite des femmes).

	1	2	3	4	5	6	7	8	9	10	Je ne sais pas
1-Plus faible -> Plus élevé-10	○	○	○	○	○	○	○	○	○	○	○

* WE 2.11 Le mariage d'enfants : Mesure de l'application des lois pour éliminer le mariage d'enfants.

	1	2	3	4	5	6	7	8	9	10	Je ne sais pas
1-Plus faible -> Plus élevé-10	○	○	○	○	○	○	○	○	○	○	○

* WE 2.12 La mutilation génitale féminine : Mesure de l'application des lois pour éliminer la mutilation génitale féminine.

	1	2	3	4	5	6	7	8	9	10	Je ne sais pas
1-Plus faible -> Plus élevé-10	○	○	○	○	○	○	○	○	○	○	○

WE 2.13 Commentaire : Utilisez cette section pour apporter des commentaires additionnels sur le niveau d'efforts, les défis et les succès du pays autour des services et programmes pour l'autonomisation des femmes. Si vous avez répondu « Je ne sais pas » à l'une des questions ci-dessus, utilisez aussi cette section pour expliquer pourquoi.

Rusatira JC, et al. BMJ Open 2023; 13:e059937. doi: 10.1136/bmjopen-2021-059937

JOHNS HOPKINS
BLOOMBERG SCHOOL
of PUBLIC HEALTH

Bill & Melinda Gates Institute for Population and Reproductive Health

Enquête de l'Indice des efforts pour le dividende démographique

Domaine 3. Plaidoyer

* WE 3.1 Les plaideurs : Mesure dans laquelle des plaideurs pour l'autonomisation des filles et des femmes existent au niveau national et infranational.

	1	2	3	4	5	6	7	8	9	10	Je ne sais pas
1-Plus faible -> Plus élevé-10	○	○	○	○	○	○	○	○	○	○	○

* WE 3.2. Les filles et les femmes : Mesure dans laquelle les efforts de plaidoyer dans ce domaine recouvrent l'ensemble des aspects de l'autonomisation des filles et des femmes.

	1	2	3	4	5	6	7	8	9	10	Je ne sais pas
1-Plus faible -> Plus élevé-10	○	○	○	○	○	○	○	○	○	○	○

* WE 3.3 La dissémination de l'information : Mesure dans laquelle le gouvernement entreprend suffisamment d'efforts pour veiller à ce que la population soit consciente des problèmes liés à l'autonomisation des femmes, et si les informations disséminées sont exactes et communiquées dans la langue parlée par les destinataires/publics cibles.

	1	2	3	4	5	6	7	8	9	10	Je ne sais pas
1-Plus faible -> Plus élevé-10	○	○	○	○	○	○	○	○	○	○	○

* WE 3.4 La mise à profit des partenariats : Mesure dans laquelle le gouvernement met à profit ses partenariats avec des OSC pour disséminer des informations sur l'autonomisation des femmes à travers des campagnes de sensibilisation/information.

	1	2	3	4	5	6	7	8	9	10	Je ne sais pas
1-Plus faible -> Plus élevé-10	○	○	○	○	○	○	○	○	○	○	○

* WE 3.5 L'emploi : Mesure dans laquelle les parties prenantes appellent à l'élaboration de lois, politiques et programmes pour que les femmes puissent combiner leur rôle de mère pendant la grossesse, l'allaitement et l'éducation de leurs enfants avec leur participation à la vie active.

	1	2	3	4	5	6	7	8	9	10	Je ne sais pas
1-Plus faible -> Plus élevé-10	○	○	○	○	○	○	○	○	○	○	○

* WE 3.6 L'éducation : Mesure dans laquelle l'une des priorités des plaideurs est de promouvoir la réalisation du plein potentiel des filles et des femmes à travers l'école et le développement de compétences.

	1	2	3	4	5	6	7	8	9	10	Je ne sais pas
1-Plus faible -> Plus élevé-10	○	○	○	○	○	○	○	○	○	○	○

* WE 3.7 La violence : Mesure dans laquelle les plaideurs réussissent à interpeller le gouvernement pour le faire réagir et répondre aux cas de violence et de discrimination contre les femmes.

	1	2	3	4	5	6	7	8	9	10	Je ne sais pas
1-Plus faible -> Plus élevé-10	○	○	○	○	○	○	○	○	○	○	○

WE 3.8 Commentaire : Utilisez cette section pour apporter des commentaires additionnels sur le niveau d'efforts, les défis et les succès du pays autour du plaidoyer pour l'autonomisation des femmes. Si vous avez répondu « Je ne sais pas » à l'une des questions ci-dessus, utilisez aussi cette section pour expliquer pourquoi.

JOHNS HOPKINS
BLOOMBERG SCHOOL
of PUBLIC HEALTH

Bill & Melinda Gates Institute for Population and Reproductive Health

Enquête de l'Indice des efforts pour le dividende démographique

Domaine 4. Recherche

* WE 4.1 La force des observatoires/parties prenantes/groupes de travail pour l'autonomisation des femmes : Mesure dans laquelle les parties prenantes du secteur (ex : groupe directif de parties prenantes, groupes de travail techniques, observatoire de l'autonomisation des femmes, groupe de coordination ou facilitation) :

	1	2	3	4	5	6	7	8	9	10	Je ne sais pas
WE 4.1.1 Ont un représentant du gouvernement, des institutions de formation, de la société civile, des organisations non gouvernementales et confessionnelles, des associations professionnelles, du secteur privé, etc.	○	○	○	○	○	○	○	○	○	○	○
WE 4.1.2 Se réunissent régulièrement, émettent des rapports et recommandent des politiques aux directions des ministères compétents.	○	○	○	○	○	○	○	○	○	○	○
WE 4.1.3 Ont un impact sur l'autonomisation des femmes dans le pays.	○	○	○	○	○	○	○	○	○	○	○

* WE 4.2 La stratégie de recherche sur les droits des femmes : Mesure dans laquelle le plan national pour l'autonomisation des femmes et/ou autres documents nationaux comprennent une approche/stratégie de recherche sur l'autonomisation des femmes, partagée entre les partenaires.

	1	2	3	4	5	6	7	8	9	10	Je ne sais pas
1-Plus faible -> Plus élevé-10	○	○	○	○	○	○	○	○	○	○	○

102

* WE 4.3 Partenaires collectant des données :

	1	2	3	4	5	6	7	8	9	10	Je ne sais pas
WE 4.3.1 Mesure dans laquelle la collecte des données est entreprise par des agences gouvernementales. (Cela peut comprendre les agences/bureaux de la statistique et/ou les ministères de l'économie/finances, éducation, santé, genre, etc.)	○	○	○	○	○	○	○	○	○	○	○
WE 4.3.2 Mesure dans laquelle la collecte de données est entreprise par des institutions de recherche.	○	○	○	○	○	○	○	○	○	○	○
WE 4.3.3 Mesure dans laquelle la collecte des données est entreprise par des chercheurs indépendants.	○	○	○	○	○	○	○	○	○	○	○

* WE 4.4 La recherche thématique :

	1	2	3	4	5	6	7	8	9	10	Je ne sais pas
WE 4.4.1 Mesure dans laquelle de la recherche est menée sur les normes sociales et culturelles affectant l'autonomisation des femmes et la parité.	○	○	○	○	○	○	○	○	○	○	○
WE 4.4.2 Mesure dans laquelle de la recherche est menée sur les effets économiques et sanitaires de l'autonomisation des femmes.	○	○	○	○	○	○	○	○	○	○	○
WE 4.4.3 Mesure dans laquelle de la recherche est menée sur le mariage d'enfants pour informer la politique.	○	○	○	○	○	○	○	○	○	○	○
WE 4.4.4 Mesure dans laquelle de la recherche est menée sur les résultats de l'éducation et de l'acquisition de compétences chez les filles.	○	○	○	○	○	○	○	○	○	○	○

* WE 4.5 La qualité/ couverture des données :

	1	2	3	4	5	6	7	8	9	10	Je ne sais pas
WE 4.5.1 Mesure dans laquelle un système statistique de routine fiable fournit des données périodiques désagrégées par âge.	○	○	○	○	○	○	○	○	○	○	○
WE 4.5.2 Mesure dans laquelle un système statistique de routine fiable fournit de bonnes données périodiques désagrégées par âge sur les besoins relatifs à l'autonomie des femmes parmi les populations de différentes communautés.	○	○	○	○	○	○	○	○	○	○	○
WE 4.5.3 Mesure dans laquelle les systèmes de suivi et de surveillance sont évalués, et ces évaluations appliquées pour assurer la fiabilité des données désagrégées par âge.	○	○	○	○	○	○	○	○	○	○	○

* WE 4.6 La qualité des institutions de recherche : Mesure dans laquelle le pays soutient et maintient des institutions de recherche qui développent des études permettant de soutenir la portée et la qualité de la collecte de données pour informer les efforts nationaux d'autonomisation des femmes.

	1	2	3	4	5	6	7	8	9	10	Je ne sais pas
1-Plus faible -> Plus élevé-10	○	○	○	○	○	○	○	○	○	○	○

* WE 4.7 L'évaluation : Mesure dans laquelle les statistiques, enquêtes et études sur les programmes sont utilisées par du personnel spécialisé pour rapporter les opérations du programme et mesurer les progrès en matière d'autonomisation des femmes et de parité.

	1	2	3	4	5	6	7	8	9	10	Je ne sais pas
1-Plus faible -> Plus élevé-10	○	○	○	○	○	○	○	○	○	○	○

* WE 4.8 L'utilisation des résultats d'évaluation par les gestionnaires de programme : Mesure dans laquelle les gestionnaires locaux des programmes d'autonomisation des femmes utilisent les conclusions de recherche et d'évaluations pour améliorer ces programmes en suivant les recommandations émises.

	1	2	3	4	5	6	7	8	9	10	Je ne sais pas
1-Plus faible -> Plus élevé-10	◯	◯	◯	◯	◯	◯	◯	◯	◯	◯	◯

* WE 4.9 L'utilisation des résultats d'évaluation par les ministères : Mesure dans laquelle les administrateurs ministériels compétents utilisent systématiquement les données pour informer les politiques et interventions visant à résoudre les problèmes liés à l'autonomisation des femmes.

	1	2	3	4	5	6	7	8	9	10	Je ne sais pas
1-Plus faible -> Plus élevé-10	◯	◯	◯	◯	◯	◯	◯	◯	◯	◯	◯

* WE 4.10 La dissémination d'informations à d'autres entités chargées de la mise en œuvre des programmes : Mesure dans laquelle des informations sont partagées ou disséminées entre géographies et à différents niveaux (national, état/province/comté, localités/sous-comtés).

	1	2	3	4	5	6	7	8	9	10	Je ne sais pas
1-Plus faible -> Plus élevé-10	◯	◯	◯	◯	◯	◯	◯	◯	◯	◯	◯

WE 4.11 Commentaire : Utilisez cette section pour apporter des commentaires additionnels sur le niveau d'efforts, les défis et les succès du pays autour de la recherche sur l'autonomisation des femmes. Si vous avez répondu « Je ne sais pas » à l'une des questions ci-dessus, utilisez aussi cette section pour expliquer pourquoi.

JOHNS HOPKINS
BLOOMBERG SCHOOL
of PUBLIC HEALTH

Bill & Melinda Gates Institute for Population and Reproductive Health

Enquête de l'Indice des efforts pour le dividende démographique

Domaine 5. Organisations de la Société Civile (OSC)

* WE 5.1 Le pouvoir des acteurs des OSC : Mesure dans laquelle les acteurs des OSC sont bien placés pour influencer la politique nationale concernant l'autonomisation des femmes.

	1	2	3	4	5	6	7	8	9	10	Je ne sais pas
1-Plus faible -> Plus élevé-10	○	○	○	○	○	○	○	○	○	○	○

* WE 5.2 L'analyse budgétaire comme outil des OSC : Mesure dans laquelle les OSC utilisent l'analyse budgétaire comme un outil pour développer le plaidoyer en matière d'autonomisation des femmes

	1	2	3	4	5	6	7	8	9	10	Je ne sais pas
1-Plus faible -> Plus élevé-10	○	○	○	○	○	○	○	○	○	○	○

* WE 5.3 Le soutien des services à base communautaire : Mesure dans laquelle les OSC participent au rapprochement des informations et services en matière d'autonomisation des femmes avec les communautés à travers des activités comprenant, mais sans s'y limiter, le renforcement de compétences des filles, les défenseurs/champions de l'autonomisation des femmes, et des campagnes d'éducation ou d'information pour augmenter l'engagement des familles et des communautés pour l'autonomie des femmes.

	1	2	3	4	5	6	7	8	9	10	Je ne sais pas
1-Plus faible -> Plus élevé-10	○	○	○	○	○	○	○	○	○	○	○

* WE 5.4 Les droits humains et la qualité de la mise en œuvre : Mesure dans laquelle les OSC plaident et soutiennent des approches de l'autonomisation des femmes fondées sur le droit pour veiller à ce que les bénéficiaires des programmes et politiques d'autonomisation des femmes puissent voir des avancées selon leurs propres termes.

	1	2	3	4	5	6	7	8	9	10	Je ne sais pas
1-Plus faible -> Plus élevé-10	◯	◯	◯	◯	◯	◯	◯	◯	◯	◯	◯

* WE 5.5 Le changement social et des comportements (CSC) : Mesure dans laquelle les OSC soutiennent des interventions pour le CSC comprenant, mais sans s'y limiter, les programmes transformateurs de genre, les compétences de vie et l'éducation entre pairs, l'éducation à la santé de l'adulte, l'éducation en milieu scolaire, la communication au sein du couple, la mobilisation communautaire, les interventions des gardiens de la communauté et/ou des interventions dans les médias de masse pour le grand public. Ces interventions peuvent être mises en œuvre au niveau communautaire ou individuel.

	1	2	3	4	5	6	7	8	9	10	Je ne sais pas
1-Plus faible -> Plus élevé-10	◯	◯	◯	◯	◯	◯	◯	◯	◯	◯	◯

* WE 5.6 Les jeunes : Mesure dans laquelle les OSC promeuvent l'autonomisation des femmes à travers les programmes pour jeunes.

	1	2	3	4	5	6	7	8	9	10	Je ne sais pas
1-Plus faible -> Plus élevé-10	◯	◯	◯	◯	◯	◯	◯	◯	◯	◯	◯

* WE 5.7 Les programmes pour les hommes : Mesure dans laquelle les OSC soutiennent les politiques, interventions et programmes d'autonomisation des femmes ciblant les hommes.

	1	2	3	4	5	6	7	8	9	10	Je ne sais pas
1-Plus faible -> Plus élevé-10	◯	◯	◯	◯	◯	◯	◯	◯	◯	◯	◯

* WE 5.8 Le plaidoyer/ la redevabilité : Mesure dans laquelle les OSC soutiennent la formation et le renforcement de compétences des membres de la communauté pour augmenter leur compréhension des politiques, processus et activités, et pour renforcer leurs capacités à identifier les problèmes liés à l'autonomisation des femmes, résoudre collectivement ces problèmes et mener des actions de plaidoyer plus ciblées.

	1	2	3	4	5	6	7	8	9	10	Je ne sais pas
1-Plus faible -> Plus élevé-10	◯	◯	◯	◯	◯	◯	◯	◯	◯	◯	◯

* WE 5.9 L'évaluation et le suivi dirigés par les OSC : Mesure dans laquelle les OSC évaluent, suivent et émettent des rapports sur l'efficacité des politiques et programmes pour améliorer la redevabilité des prestataires et des politiques d'autonomisation des femmes.

	1	2	3	4	5	6	7	8	9	10	Je ne sais pas
1-Plus faible -> Plus élevé-10	○	○	○	○	○	○	○	○	○	○	○

* WE 5.10 Les partenariats entre OSC : Mesure dans laquelle les OSC ont formé des alliances nationales et des partenariats régionaux pour renforcer leur position et potentiellement améliorer le leadership et les mécanismes de financement afin de faire progresser l'agenda de l'autonomisation des femmes.

	1	2	3	4	5	6	7	8	9	10	Je ne sais pas
1-Plus faible -> Plus élevé-10	○	○	○	○	○	○	○	○	○	○	○

WE 5.11 Commentaire : Utilisez cette section pour apporter des commentaires additionnels sur le niveau d'efforts, les défis et les succès du pays concernant le rôle des OSC pour l'autonomisation des femmes. Si vous avez répondu « Je ne sais pas » à l'une des questions ci-dessus, utilisez aussi cette section pour expliquer pourquoi.

JOHNS HOPKINS
BLOOMBERG SCHOOL
of PUBLIC HEALTH

Bill & Melinda Gates Institute for Population and Reproductive Health

Enquête de l’Indice des efforts pour le dividende démographique

Module : Résilience et durabilité du secteur

Étant donné l’impact sanitaire et socioéconomique de la pandémie de COVID-19, et son impact probable sur la progression vers le DD, cet indice des efforts pour le DD intègre des questions pour évaluer la résilience et la durabilité des systèmes dans les secteurs clés du DD. Les éléments couverts par les questions liées à la pandémie de COVID-19 ci-dessous relèvent des dimensions clés des systèmes résilients et visent à apporter des informations déterminantes sur le potentiel d’une réponse efficace aux menaces de maladies infectieuses émergentes et autres crises de santé publique.

Veuillez noter chaque élément sur une échelle de 1 à 10, 1 étant le score le plus faible (faible état/capacité) et 10 le plus élevé (très bon état/capacité).

* Dimension physique : structures, équipements, états du système et capacités.

	1	2	3	4	5	6	7	8	9	10	Je ne sais pas
WE-M1 - Plan/Préparation - État et capacité de l’équipement, du personnel et de la structure du secteur de l’autonomisation des femmes avant la crise. (1 = faible état/capacité ; 10 = très bon état/capacité)	○	○	○	○	○	○	○	○	○	○	○
WE-M2 - Absorption – Mesure dans laquelle le secteur de l’autonomisation des femmes a reconnu la pandémie de COVID-19 et pu continuer à être fonctionnel.	○	○	○	○	○	○	○	○	○	○	○

110

	1	2	3	4	5	6	7	8	9	10	Je ne sais pas
WE-M3 - Absorption – Intégration : Niveau d'intégration des services et programmes d'autonomisation des femmes pour mitiger les effets de la pandémie de COVID-19 sur l'autonomie des femmes.	○	○	○	○	○	○	○	○	○	○	○
WE-M4 - Récupération – Mesure dans laquelle les programmes d'autonomisation des femmes ont été capables d'induire des changements pour récupérer un niveau de fonctionnalité pré-COVID-19.	○	○	○	○	○	○	○	○	○	○	○
WE-M5 - Récupération – Gestion du financement et des bailleurs de fonds : Niveau de mobilisation des ressources et de l'allocation budgétaire pour mitiger les effets de la pandémie de COVID-19 sur l'autonomie des femmes.	○	○	○	○	○	○	○	○	○	○	○
WE-M6 - Adaptation – Mesure dans laquelle des changements ont été effectués pour améliorer la résilience du secteur de l'autonomisation des femmes.	○	○	○	○	○	○	○	○	○	○	○

* Dimension informatique : création, gestion, stockage et utilisation des données.

	1	2	3	4	5	6	7	8	9	10	Je ne sais pas
WE-M7 - Plan/Préparation – Mesure dans laquelle les données liées à l'autonomisation des femmes étaient suffisamment préparées, présentées, analysées et stockées avant la crise.	○	○	○	○	○	○	○	○	○	○	○

	1	2	3	4	5	6	7	8	9	10	Je ne sais pas
WE-M8 - Absorption – Connaissance : Mesure dans laquelle le secteur de l'autonomisation des femmes a pu conduire des évaluations en temps réel de son état de fonctionnement, en anticipation des pertes en cascade des services liés à l'autonomie des femmes.	○	○	○	○	○	○	○	○	○	○	○
WE-M9 - Absorption – Connaissance/ Conscience : Capacité du système de surveillance à détecter les limites/ menaces posées à l'autonomie des femmes par la pandémie de COVID-19.	○	○	○	○	○	○	○	○	○	○	○
WE-M10 - Mesure dans laquelle les données sur l'autonomisation des femmes ont été utilisées pour suivre les progrès vers la récupération et anticiper des scenarii de récupération.	○	○	○	○	○	○	○	○	○	○	○
WE-M11 - Adaptation – Mesure dans laquelle le secteur de l'autonomisation des femmes crée et améliore ses protocoles de stockage et utilisation en temps réel de ses données sur l'autonomisation des femmes.	○	○	○	○	○	○	○	○	○	○	○

* Dimension cognitive : compréhension, modèles cognitifs, idées reçues, préjugés et valeurs.

	1	2	3	4	5	6	7	8	9	10	Je ne sais pas
WE-M12 - Planification/Préparation – Mesure dans laquelle le système de conception et les décisions opérationnelles étaient préparés à anticiper la crise.	○	○	○	○	○	○	○	○	○	○	○

	1	2	3	4	5	6	7	8	9	10	Je ne sais pas
WE-M13 - Absorption – Mesure dans laquelle le secteur de l'autonomisation des femmes a des protocoles de contingence suffisants et une gestion de crise proactive.	○	○	○	○	○	○	○	○	○	○	○
WE-M14 - Absorption – Redevabilité : Niveau d'effort pour assurer une redevabilité optimale vis-à-vis des ressources allouées à l'autonomisation des femmes dans le cadre de la réponse à la pandémie de COVID-19. (Cela peut prendre la forme de soutien aux services atténués pour répondre aux besoins de protection des femmes et des filles).	○	○	○	○	○	○	○	○	○	○	○
WE-M15 - Récupération – Mesure dans laquelle les décisions sont orientées sur la récupération et communiquées aux communautés sur la base de données probantes pour promouvoir des comportements sûrs	○	○	○	○	○	○	○	○	○	○	○

	1	2	3	4	5	6	7	8	9	10	Je ne sais pas
WE-M16 - Récupération – Connaissance/Conscience : Mesure dans laquelle le secteur établit une certaine confiance à travers sa communication sur les barrières posées par la pandémie de COVID-19 pour soutenir l'accès et l'utilisation des services et programmes pour l'autonomisation des femmes. (Cela comprend la surveillance des disparités de genre dans les réponses à la pandémie et/ou la surveillance augmentée des questions liées à l'autonomie des femmes, comme la violence basée sur le genre).	○	○	○	○	○	○	○	○	○	○	○
WE-M17 - Adaptation – Mesure dans laquelle le secteur de l'autonomisation des femmes conçoit de nouvelles configurations systémiques, objectifs et critères de décision.	○	○	○	○	○	○	○	○	○	○	○
WE-M18 Adaptation - Niveau d'adaptation pour répondre aux menaces COVID-19 qui pèsent sur l'autonomisation des femmes	○	○	○	○	○	○	○	○	○	○	○

* Social dimension: interaction, collaboration and self-synchronization between people, entities and institutions.

	1	2	3	4	5	6	7	8	9	10	Je ne sais pas
WE-M19 - Planification /Préparation – Mesure dans laquelle une formation sur la gestion d'épidémies/crises a été menée et a permis de mettre à profit les réseaux sociaux, le capital social et les normes institutionnelles et culturelles avant la crise.	○	○	○	○	○	○	○	○	○	○	○
WE-M20 - Absorption – Mesure dans laquelle le personnel et les institutions sociales ont été accessibles et ont fait preuve d'ingéniosité dans leur réponse à l'épidémie/crise.	○	○	○	○	○	○	○	○	○	○	○
WE-M21 - Absorption - Self-regulation: Extent to which national leaders had the authority to effect timely sectoral changes through an infrastructure flexible.	○	○	○	○	○	○	○	○	○	○	○
WE-M22 - Récupération – Mesure dans laquelle le secteur de l'autonomisation des femmes est impliqué dans le partage de connaissances et d'équipes pour améliorer la récupération du système.	○	○	○	○	○	○	○	○	○	○	○
WE-M23 – Niveau d'engagement d'une équipe multidisciplinaire pour mitiger les effets de la pandémie de COVID-19 sur l'autonomisation des femmes.	○	○	○	○	○	○	○	○	○	○	○

	1	2	3	4	5	6	7	8	9	10	Je ne sais pas
WE-M24 - Adaptation - Leadership et management : Niveau de leadership démontré par les dirigeants du secteur de l'autonomisation des femmes pour mitiger les effets de la pandémie de COVID-19 sur l'autonomie des femmes.	○	○	○	○	○	○	○	○	○	○	○

* WE M-25 Temporalité : Mesure de la rapidité de la réaction du gouvernement –sa communication et mise en œuvre des mesures- pour mitiger l'impact immédiat et sur le long terme de la pandémie de COVID-19. Cette question porte sur la riposte nationale dans son ensemble, et non la réponse spécifique de votre secteur.

	1	2	3	4	5	6	7	8	9	10	Je ne sais pas
1-Plus faible -> Plus élevé-10	○	○	○	○	○	○	○	○	○	○	○

WE M-26 Commentaires : Veuillez utiliser cette section pour tout commentaire additionnel sur la résilience et la durabilité des opportunités et systèmes nationaux d'autonomisation des femmes et des filles en fonction de votre expérience de la pandémie de COVID-19. Si vous avez répondu « Je ne sais pas » à l'une des questions, utilisez aussi cette section pour expliquer pourquoi.

JOHNS HOPKINS
BLOOMBERG SCHOOL
of PUBLIC HEALTH

Bill & Melinda Gates Institute for Population and Reproductive Health

Enquête de l’Indice des efforts pour le dividende démographique

Questionnaire sur le marché du travail (MT)

Répondez aux questions suivantes en fonction de votre expérience/ expertise dans ce secteur spécifique.

Afin d’obtenir une représentation synthétique des efforts nationaux pour le dividende démographique, notez les éléments suivants sur une échelle de 1 à 10, 1 étant le score le plus faible (efforts très faibles ou quasi-inexistants) et 10 le plus élevé (efforts robustes). Le cas échéant, si une politique ou activité n’existe pas, répondez 0.

Donnez un score à chaque élément. Toutes les réponses seront enregistrées au format illustré ci-dessous :

Effort le plus faible	1	2	3	4	5	6	7	8	9	10	Effort le plus élevé	Je ne sais pas

JOHNS HOPKINS
BLOOMBERG SCHOOL of PUBLIC HEALTH

Bill & Melinda Gates Institute for Population and Reproductive Health

Enquête de l'Indice des efforts pour le dividende démographique

Domaine 1. Politique/ Prise de décision

* LM 1.1 L'intégration de l'économie mondiale : Mesure dans laquelle le pays vise à s'intégrer à l'économie mondiale.

	1	2	3	4	5	6	7	8	9	10	Je ne sais pas
LM 1.1.1 Mesure dans laquelle le pays légifère pour l'ouverture des échanges commerciaux.	◯	◯	◯	◯	◯	◯	◯	◯	◯	◯	◯
LM 1.1.2 Niveau d'effort en faveur de la croissance des exportations nationales.	◯	◯	◯	◯	◯	◯	◯	◯	◯	◯	◯

* LM 1.2 L'intégration économique régionale : Mesure dans laquelle le pays vise à s'intégrer à l'économie régionale.

	1	2	3	4	5	6	7	8	9	10	Je ne sais pas
1-Plus faible -> Plus élevé-10	◯	◯	◯	◯	◯	◯	◯	◯	◯	◯	◯

* LM 1.3 La technologie : Mesure dans laquelle le gouvernement a investi dans l'infrastructure, à la fois traditionnelle et technologique, pour permettre au pays de saisir les opportunités de demain/ d'intégration technologique en complément de la main d'œuvre.

	1	2	3	4	5	6	7	8	9	10	Je ne sais pas
1-Plus faible -> Plus élevé-10	◯	◯	◯	◯	◯	◯	◯	◯	◯	◯	◯

* LM 1.4 Le soutien du gouvernement : Mesure dans laquelle des politiques et plans sont en place et prévoient des dispositifs de protection sociale pour une transition harmonieuse entre les différents emplois, ou « petits boulots », des personnes, et pour les protéger de la précarité/ volatilité des revenus.

	1	2	3	4	5	6	7	8	9	10	Je ne sais pas
LM 1.4.1 Mesure dans laquelle une politique nationale établissant un salaire minimum est en place.	○	○	○	○	○	○	○	○	○	○	○
LM 1.4.2 Mesure dans laquelle une politique nationale définissant un système d'assurance chômage est en place, et niveau d'efficacité de sa mise en œuvre.	○	○	○	○	○	○	○	○	○	○	○

* LM 1.5 Le chômage : Mesure dans laquelle les politiques rendent compte et répondent au chômage chez différents groupes de la population et dans différentes aires géographiques.

	1	2	3	4	5	6	7	8	9	10	Je ne sais pas
1-Plus faible -> Plus élevé-10	○	○	○	○	○	○	○	○	○	○	○

* LM 1.6 Les groupes défavorisés : Mesure dans laquelle des politiques existent, et sont effectivement mises en œuvre, pour soutenir les opportunités d'embauche des groupes défavorisés (c'est-à-dire les filles et les femmes, les jeunes, les personnes en situation de mobilité réduite ou de handicap, etc.).

	1	2	3	4	5	6	7	8	9	10	Je ne sais pas
1-Plus faible -> Plus élevé-10	○	○	○	○	○	○	○	○	○	○	○

* LM 1.7 Le changement climatique et le développement durable : Mesure dans laquelle les secteurs de l'agriculture et de la production industrielle sont préparés aux impacts des effets du changement climatique sur le marché du travail en élaborant des stratégies de planification familiale en vue des besoins du marché de travail de demain.

	1	2	3	4	5	6	7	8	9	10	Je ne sais pas
1-Plus faible -> Plus élevé-10	○	○	○	○	○	○	○	○	○	○	○

* LM 1.8 L'urbanisation : Mesure dans laquelle les politiques et la planification anticipent et sont préparées à la croissance urbaine résultant et contribuant au changement climatique, et ses impacts particuliers sur les marchés du travail.

	1	2	3	4	5	6	7	8	9	10	Je ne sais pas
1-Plus faible -> Plus élevé-10	○	○	○	○	○	○	○	○	○	○	○

* LM 1.9 L'économie de services : Mesure dans laquelle des politiques et des cadres de travail sont en place pour faire passer les économies basées sur du travail informel au travail formalisé répondant aux demandes locales, régionales et mondiales de biens et de services.

	1	2	3	4	5	6	7	8	9	10	Je ne sais pas
1-Plus faible -> Plus élevé-10	○	○	○	○	○	○	○	○	○	○	○

* LM 1.10 La responsabilité sociale d'entreprise : Mesure dans laquelle la législation nationale du marché du travail utilise la responsabilité sociale des entreprises (RSE) pour soutenir l'entreprenariat chez les jeunes.

	1	2	3	4	5	6	7	8	9	10	Je ne sais pas
1-Plus faible -> Plus élevé-10	○	○	○	○	○	○	○	○	○	○	○

* LM 1.11 Les investissements ciblés dans le marché du travail : Mesure dans laquelle le gouvernement a ciblé ses investissements sur les secteurs générateurs d'emplois, y compris les Technologies de l'Information et la Communication (TIC), le secteur de production industrielle, l'agriculture et les entreprises industrielles agricoles, pour générer de l'emploi et encourager la croissance inclusive.

	1	2	3	4	5	6	7	8	9	10	Je ne sais pas
1-Plus faible -> Plus élevé-10	○	○	○	○	○	○	○	○	○	○	○

* LM 1.12 Les travailleurs indépendants : Mesure dans laquelle les politiques soutiennent l'élévation des retours sur investissement des travailleurs indépendants et la création de plus d'opportunités pour passer du travail indépendant à des emplois mieux rémunérés. (Cela comprend le travail en milieu rural).

	1	2	3	4	5	6	7	8	9	10	Je ne sais pas
1-Plus faible -> Plus élevé-10	○	○	○	○	○	○	○	○	○	○	○

* LM 1.13 Le microcrédit : Mesure dans laquelle le gouvernement permet l'accès à des microcrédits pour exercer des activités à but lucratif, sécurisant des crédits abordables pour les petites et moyennes entreprises et les travailleurs indépendants.

	1	2	3	4	5	6	7	8	9	10	Je ne sais pas
1-Plus faible -> Plus élevé-10	○	○	○	○	○	○	○	○	○	○	○

* LM 1.14 L'épargne : Mesure dans laquelle le gouvernement soutient la création de produits financiers accessibles et des politiques promouvant la culture de l'épargne.

	1	2	3	4	5	6	7	8	9	10	Je ne sais pas
1-Plus faible -> Plus élevé-10	○	○	○	○	○	○	○	○	○	○	○

* LM 1.15 L'acquisition de compétences : Mesure dans laquelle des politiques existent, et sont mises en œuvre efficacement, pour soutenir l'acquisition de compétences valorisées sur le marché du travail d'aujourd'hui et de demain.

	1	2	3	4	5	6	7	8	9	10	Je ne sais pas
1-Plus faible -> Plus élevé-10	○	○	○	○	○	○	○	○	○	○	○

LM 1.16 Commentaires : Veuillez utiliser cette section pour nous faire part de tout commentaire additionnel sur le niveau d'effort, les défis ou les succès du pays autour de la politique/prise de décision dans le secteur du marché du travail. Si vous avez répondu « Je ne sais pas » à l'une des questions précédentes, veuillez utiliser cette section pour expliquer pourquoi.

JOHNS HOPKINS
BLOOMBERG SCHOOL
of PUBLIC HEALTH

Bill & Melinda Gates Institute for Population and Reproductive Health

Enquête de l'Indice des efforts pour le dividende démographique

Domaine 2. Services ou programmes

* LM 2.1 La réduction du chômage : Mesure dans laquelle des efforts sont menés pour développer et mettre en œuvre des stratégies visant à réduire la proportion des personnes au chômage par année cible (en accord avec le Plan de mise en œuvre des dix premières années de l'Agenda 2063).

	1	2	3	4	5	6	7	8	9	10	Je ne sais pas
1-Plus faible -> Plus élevé-10	○	○	○	○	○	○	○	○	○	○	○

* LM 2.2. Le chômage chez les jeunes : Mesure dans laquelle des programmes liés au marché du travail ont effectivement réduit le taux de chômage chez les jeunes en particulier.

	1	2	3	4	5	6	7	8	9	10	Je ne sais pas
1-Plus faible -> Plus élevé-10	○	○	○	○	○	○	○	○	○	○	○

* LM 2.3 L'accès au microcrédit : Mesure dans laquelle les fonds nationaux et régionaux ont été établis et opérationnalisés pour augmenter l'accès des personnes au capital commercial abordable.

	1	2	3	4	5	6	7	8	9	10	Je ne sais pas
1-Plus faible -> Plus élevé-10	○	○	○	○	○	○	○	○	○	○	○

* LM 2.4 L'exploitation des capacités uniques des jeunes : Mesure dans laquelle des programmes et services soutiennent les talents spécifiques des jeunes pour les aider à s'épanouir et réaliser leur plein potentiel. (Cela couvre les objectifs programmatiques spécifiques liés à l'acquisition de talents).

	1	2	3	4	5	6	7	8	9	10	Je ne sais pas
1-Plus faible -> Plus élevé-10	○	○	○	○	○	○	○	○	○	○	○

* LM 2.5 Les opportunités de première embauche : Mesure dans laquelle les programmes et la législation du gouvernement engagent les partenaires du secteur privé à élargir les opportunités de stages, d'apprentissage professionnel et de formation continue au travail pour les femmes et les jeunes.

	1	2	3	4	5	6	7	8	9	10	Je ne sais pas
1-Plus faible -> Plus élevé-10	○	○	○	○	○	○	○	○	○	○	○

* LM 2.6 L'entrée des jeunes dans la population active : Mesure de la promotion du volontariat des jeunes, des programmes pour jeunes professionnelles et autres opportunités similaires pour améliorer leurs capacités et les exposer aux organisations régionales et internationales.

	1	2	3	4	5	6	7	8	9	10	Je ne sais pas
1-Plus faible -> Plus élevé-10	○	○	○	○	○	○	○	○	○	○	○

* LM 2.7 Les risques de l'entreprenariat : Mesure dans laquelle les personnes ont un meilleur accès aux services d'achat et de finance du gouvernement pour réduire les difficultés liées au lancement d'un projet professionnel et/ou à la conduite d'activités commerciales dans et entre les pays.

	1	2	3	4	5	6	7	8	9	10	Je ne sais pas
1-Plus faible -> Plus élevé-10	○	○	○	○	○	○	○	○	○	○	○

* LM 2.8 Les ressources pour l'entreprenariat des jeunes : Mesure dans laquelle les projets d'entreprenariat des jeunes bénéficient d'une mobilisation de ressources directe et ciblée, soutenue par un cadre régulateur, pour réduire les difficultés liées au lancement d'un projet professionnel et/ou à la conduite d'activités commerciales dans et entre les pays.

	1	2	3	4	5	6	7	8	9	10	Je ne sais pas
1-Plus faible -> Plus élevé-10	○	○	○	○	○	○	○	○	○	○	○

* LM 2.9 Les opportunités professionnelles : Mesure dans laquelle les opportunités de formation professionnelles sont amplifiées pour permettre l'acquisition de compétences et améliorer les capacités d'insertion professionnelle, la productivité et la compétitivité.

	1	2	3	4	5	6	7	8	9	10	Je ne sais pas
1-Plus faible -> Plus élevé-10	○	○	○	○	○	○	○	○	○	○	○

* LM 2.10 L'éducation et l'insertion professionnelle : Mesure dans laquelle des améliorations ont été apportées pour favoriser l'accès inclusif à l'éducation à tous les niveaux et offrir des alternatives viables à de nombreux jeunes, particulièrement les filles adolescentes, qui abandonnent le système d'éducation scolaire formel, en facilitant leur réinsertion, en restructurant l'éducation informelle et la formation via une certification standardisée dans et entre les pays.

	1	2	3	4	5	6	7	8	9	10	Je ne sais pas
1-Plus faible -> Plus élevé-10	○	○	○	○	○	○	○	○	○	○	○

* LM 2.11 Le travail « ami des femmes » : Mesure dans laquelle les lieux de travail dans les secteurs privé et public ont adopté des politiques et pratiques « amies des femmes » pour éliminer toute forme de discrimination basée sur le genre, tout en leur permettant de prendre pleinement part à la population active.

	1	2	3	4	5	6	7	8	9	10	Je ne sais pas
1-Plus faible -> Plus élevé-10	○	○	○	○	○	○	○	○	○	○	○

* LM 2.12 La sécurité de l'emploi :

	1	2	3	4	5	6	7	8	9	10	Je ne sais pas
LM 2.12.1 Mesure dans laquelle les périodes de préavis relatifs aux plans sociaux et au licenciement sont respectées.	○	○	○	○	○	○	○	○	○	○	○
LM 2.12.2 Mesure dans laquelle les indemnités de départ dans le cadre de plans sociaux ou de licenciements sont appliquées.	○	○	○	○	○	○	○	○	○	○	○

* LM 2.13 Le salaire minimum : Mesure dans laquelle un salaire minimum est appliqué au travailleur « type » (défini comme un salarié moyen, issu de groupes ethniques et religieux majoritaires, vivant dans les grandes villes, et non syndicalisé).

	1	2	3	4	5	6	7	8	9	10	Je ne sais pas
1-Plus faible -> Plus élevé-10	○	○	○	○	○	○	○	○	○	○	○

* LM 2.14 Les programmes du marché du travail : Mesure dans laquelle les programmes du marché du travail ont effectivement diversifié la main d'œuvre et les besoins du marché du travail, tout en diversifiant simultanément les compétences de cette main d'œuvre.

	1	2	3	4	5	6	7	8	9	10	Je ne sais pas
1-Plus faible -> Plus élevé-10	○	○	○	○	○	○	○	○	○	○	○

* LM 2.15 L'informalité sur le marché du travail : Mesure dans laquelle des programmes sont mis en œuvre pour transformer le travail informel en travail légal.

	1	2	3	4	5	6	7	8	9	10	Je ne sais pas
1-Plus faible -> Plus élevé-10	○	○	○	○	○	○	○	○	○	○	○

LM 2.16 Commentaires : Veuillez utiliser cette section pour nous faire part de tout commentaire additionnel sur le niveau d'effort, les défis ou les succès du pays autour des services et programmes dans le secteur du marché du travail. Si vous avez répondu « Je ne sais pas » à l'une des questions précédentes, veuillez utiliser cette section pour expliquer pourquoi.

Johns Hopkins Bloomberg School of Public Health

Bill & Melinda Gates Institute for Population and Reproductive Health

Enquête de l'Indice des efforts pour le dividende démographique

Domaine 3. Plaidoyer

* LM 3.1 La parité sur le marché du travail : Mesure dans laquelle les groupes ou individus appellent, et voient des améliorations, en matière de parité et d'égalité hommes-femmes sur les lieux de travail.

	1	2	3	4	5	6	7	8	9	10	Je ne sais pas
1-Plus faible -> Plus élevé-10	◯	◯	◯	◯	◯	◯	◯	◯	◯	◯	◯

* LM 3.2 L'égalité des jeunes sur le marché du travail : Mesure dans laquelle les groupes et les individus appellent à des changements du marché du travail et des régulations au bénéfice des populations jeunes rurales et urbaines.

	1	2	3	4	5	6	7	8	9	10	Je ne sais pas
1-Plus faible -> Plus élevé-10	◯	◯	◯	◯	◯	◯	◯	◯	◯	◯	◯

* LM 3.3 Le plaidoyer pour le salaire minimum : Mesure dans laquelle les groupes de plaidoyer réussissent à inciter des lois pour le salaire minimum, ou une augmentation du salaire minimum si une telle loi existe déjà.

	1	2	3	4	5	6	7	8	9	10	Je ne sais pas
1-Plus faible -> Plus élevé-10	◯	◯	◯	◯	◯	◯	◯	◯	◯	◯	◯

* LM 3.4 Le plaidoyer du gouvernement : Mesure dans laquelle les membres du gouvernement plaident pour des améliorations de ses propres structures et priorités. (Cela peut comprendre des progrès sur le marché du travail en matière d'inclusion, opportunités, programmes et droits des jeunes, et autres concepts similaires dans différentes populations).

	1	2	3	4	5	6	7	8	9	10	Je ne sais pas
1-Plus faible -> Plus élevé-10	◯	◯	◯	◯	◯	◯	◯	◯	◯	◯	◯

* LM 3.5 Les protections sociales : Mesure dans laquelle il existe un plaidoyer pour la protection de l'emploi et l'assurance chômage.

	1	2	3	4	5	6	7	8	9	10	Je ne sais pas
1-Plus faible -> Plus élevé-10	◯	◯	◯	◯	◯	◯	◯	◯	◯	◯	◯

LM 3.6 Commentaires : Veuillez utiliser cette section pour nous faire part de tout commentaire additionnel sur le niveau d'effort, les défis ou les succès du pays autour du plaidoyer dans le secteur du marché du travail. Si vous avez répondu « Je ne sais pas » à l'une des questions précédentes, veuillez utiliser cette section pour expliquer pourquoi.

JOHNS HOPKINS
BLOOMBERG SCHOOL
of PUBLIC HEALTH

Bill & Melinda Gates Institute for Population and Reproductive Health

Enquête de l'Indice des efforts pour le dividende démographique

Domaine 4. Recherche

* LM 4.1 La force des observatoires/parties prenantes/groupes de travail dans le secteur du marché du travail : Mesure dans laquelle les parties prenantes du secteur du marché du travail (ex : groupe directif de parties prenantes, groupes de travail techniques, observatoire du marché du travail, groupe de coordination ou facilitation) :

	1	2	3	4	5	6	7	8	9	10	Je ne sais pas
LM 4.1.1 Ont un représentant du gouvernement, des institutions de formation, de la société civile, des organisations non gouvernementales et confessionnelles, des associations professionnelles, du secteur privé, etc.	○	○	○	○	○	○	○	○	○	○	○
LM 4.1.2 Se réunissent régulièrement, émettent des rapports et recommandent des politiques aux directions des ministères compétents.	○	○	○	○	○	○	○	○	○	○	○
LM 4.1.3 Ont un impact sur le marché du travail dans le pays.	○	○	○	○	○	○	○	○	○	○	○

* LM 4.2 La stratégie de recherche sur le marché du travail : Mesure dans laquelle les documents nationaux prévoient une stratégie/approche complète pour la recherche sur le marché du travail qui soit partagée entre partenaires.

	1	2	3	4	5	6	7	8	9	10	Je ne sais pas
1-Plus faible -> Plus élevé-10	○	○	○	○	○	○	○	○	○	○	○

Rusatira JC, *et al. BMJ Open* 2023; 13:e059937. doi: 10.1136/bmjopen-2021-059937

* LM 4.3 Les partenaires de l’analyse du marché du travail :

	1	2	3	4	5	6	7	8	9	10	Je ne sais pas
LM 4.3.1 Mesure dans laquelle la collecte des données est entreprise par des agences gouvernementales.	○	○	○	○	○	○	○	○	○	○	○
LM 4.3.2 Mesure dans laquelle la collecte de données est entreprise par des institutions de recherche.	○	○	○	○	○	○	○	○	○	○	○
LM 4.3.3 Mesure dans laquelle la collecte des données est entreprise par des chercheurs indépendants.	○	○	○	○	○	○	○	○	○	○	○

* LM 4.4 Les domaines de la recherche sur le marché du travail :

	1	2	3	4	5	6	7	8	9	10	Je ne sais pas
LM 4.4.1 Mesure dans laquelle de la recherche est menée sur les tendances du chômage.	○	○	○	○	○	○	○	○	○	○	○
LM 4.4.2 Mesure dans laquelle de la recherche est menée sur les jeunes et le marché du travail.	○	○	○	○	○	○	○	○	○	○	○
LM 4.4.3 Mesure dans laquelle de la recherche est menée sur les femmes et le marché du travail.	○	○	○	○	○	○	○	○	○	○	○
LM 4.4.4 Mesure dans laquelle de la recherche est menée sur les tendances et les trajectoires des besoins du marché du travail.	○	○	○	○	○	○	○	○	○	○	○

* LM 4.5 La qualité/ couverture des données :

	1	2	3	4	5	6	7	8	9	10	Je ne sais pas
LM 4.5.1 Mesure dans laquelle un système statistique de routine fiable fournit des données périodiques sur le marché du travail.	○	○	○	○	○	○	○	○	○	○	○
LM 4.5.2 Mesure dans laquelle un système statistique de routine fiable fournit de bonnes données périodiques sur les besoins du marché du travail parmi les populations de différentes communautés.	○	○	○	○	○	○	○	○	○	○	○
LM 4.5.3 Mesure dans laquelle les systèmes de suivi et de surveillance sont évalués, et ces évaluations appliquées pour assurer la fiabilité des données.	○	○	○	○	○	○	○	○	○	○	○

* LM 4.6 Les registres : Mesure dans laquelle les systèmes de registre, rapports et retours/commentaires sur les résultats du marché du travail sont adéquats.

	1	2	3	4	5	6	7	8	9	10	Je ne sais pas
1-Plus faible -> Plus élevé-10	○	○	○	○	○	○	○	○	○	○	○

* LM 4.7 La qualité des institutions de recherche : Mesure dans laquelle le pays a la capacité de soutenir et maintenir les institutions de recherche qui développent des études/ collectent des données liées au marché du travail.

	1	2	3	4	5	6	7	8	9	10	Je ne sais pas
1-Plus faible -> Plus élevé-10	○	○	○	○	○	○	○	○	○	○	○

* LM 4.8 L'évaluation : Mesure dans laquelle les statistiques de programmes, enquêtes et études sont utilisées par du personnel spécialisé pour rapporter sur les opérations des programmes et mesurer le progrès.

	1	2	3	4	5	6	7	8	9	10	Je ne sais pas
1-Plus faible -> Plus élevé-10	○	○	○	○	○	○	○	○	○	○	○

* LM 4.9 L'utilisation des résultats d'évaluation par les décideurs : Mesure dans laquelle les gestionnaires locaux des programmes du marché du travail utilisent les conclusions de recherche et d'évaluations pour améliorer ces programmes en suivant les recommandations émises.

	1	2	3	4	5	6	7	8	9	10	Je ne sais pas
1-Plus faible -> Plus élevé-10	◯	◯	◯	◯	◯	◯	◯	◯	◯	◯	◯

* LM 4.10 L'utilisation des résultats d'évaluation par les ministères : Mesure dans laquelle les administrateurs ministériels compétents utilisent systématiquement les données pour informer les politiques et interventions visant à résoudre les problèmes liés au marché du travail.

	1	2	3	4	5	6	7	8	9	10	Je ne sais pas
1-Plus faible -> Plus élevé-10	◯	◯	◯	◯	◯	◯	◯	◯	◯	◯	◯

* LM 4.11 La dissémination d'informations à d'autres entités chargées de la mise en œuvre des programmes : Mesure dans laquelle des informations sont partagées ou disséminées entre géographies et à différents niveaux (national, état/province/comté, localités/sous-comtés).

	1	2	3	4	5	6	7	8	9	10	Je ne sais pas
1-Plus faible -> Plus élevé-10	◯	◯	◯	◯	◯	◯	◯	◯	◯	◯	◯

LM 4.12 Commentaires : Veuillez utiliser cette section pour nous faire part de tout commentaire additionnel sur le niveau d'effort, les défis ou les succès du pays autour de la recherche dans le secteur du marché du travail. Si vous avez répondu « Je ne sais pas » à l'une des questions précédentes, veuillez utiliser cette section pour expliquer pourquoi.

JOHNS HOPKINS
BLOOMBERG SCHOOL
of PUBLIC HEALTH

Bill & Melinda Gates Institute for Population and Reproductive Health

Enquête de l’Indice des efforts pour le dividende démographique

Dimension 5. Organisations de la société civile (OSC)

* LM 5.1 Le pouvoir des acteurs des OSC : Mesure dans laquelle les acteurs des OSC sont bien placés pour influencer la politique et l’action nationale concernant le marché du travail.

	1	2	3	4	5	6	7	8	9	10	Je ne sais pas
1-Plus faible -> Plus élevé-10	○	○	○	○	○	○	○	○	○	○	○

* LM 5.2 L’analyse budgétaire comme outil des OSC : Mesure dans laquelle les OSC utilisent l’analyse budgétaire comme un outil pour développer le plaidoyer en matière de marché du travail.

	1	2	3	4	5	6	7	8	9	10	Je ne sais pas
1-Plus faible -> Plus élevé-10	○	○	○	○	○	○	○	○	○	○	○

* LM 5.3 Le soutien des services à base communautaire : Mesure dans laquelle les OSC participent à présenter les besoins, informations et services du marché du travail aux décideurs, et vice-versa.

	1	2	3	4	5	6	7	8	9	10	Je ne sais pas
1-Plus faible -> Plus élevé-10	○	○	○	○	○	○	○	○	○	○	○

* LM 5.4 La diversification des opportunités d’embauche : Mesure dans laquelle les OSC et le secteur privé travaillent en coordination avec le gouvernement pour assurer une création d’emplois optimale et une participation hommes-femmes équitable sur le marché du travail.

	1	2	3	4	5	6	7	8	9	10	Je ne sais pas
1-Plus faible -> Plus élevé-10	○	○	○	○	○	○	○	○	○	○	○

Rusatira JC, *et al. BMJ Open* 2023; 13:e059937. doi: 10.1136/bmjopen-2021-059937

* LM 5.5 Les droits humains et la qualité de la mise en œuvre : Mesure dans laquelle les OSC soutiennent des approches fondées sur le droit sur les lieux de travail.

	1	2	3	4	5	6	7	8	9	10	Je ne sais pas
1-Plus faible -> Plus élevé-10	○	○	○	○	○	○	○	○	○	○	○

* LM 5.6 Les jeunes : Mesure dans laquelle les OSC soutiennent des politiques, interventions et programmes pour l'emploi des jeunes spécifiquement.

	1	2	3	4	5	6	7	8	9	10	Je ne sais pas
1-Plus faible -> Plus élevé-10	○	○	○	○	○	○	○	○	○	○	○

* LM 5.7 Les programmes pour les femmes : Mesure dans laquelle les OSC soutiennent des politiques, services et programmes pour l'emploi des femmes spécifiquement.

	1	2	3	4	5	6	7	8	9	10	Je ne sais pas
1-Plus faible -> Plus élevé-10	○	○	○	○	○	○	○	○	○	○	○

* LM 5.8 Le plaidoyer/ la redevabilité : Mesure dans laquelle les OSC soutiennent la formation et le renforcement de compétences des membres de la communauté pour augmenter leur compréhension des politiques, processus et activités, et pour renforcer leurs capacités à identifier les problèmes liés au marché du travail, résoudre collectivement ces problèmes et mener des actions de plaidoyer plus ciblées pour combattre le chômage.

	1	2	3	4	5	6	7	8	9	10	Je ne sais pas
1-Plus faible -> Plus élevé-10	○	○	○	○	○	○	○	○	○	○	○

* LM 5.9 L'évaluation et le suivi dirigés par les OSC : Mesure dans laquelle les OSC évaluent, suivent et émettent des rapports sur l'efficacité des politiques et programmes pour améliorer la redevabilité au niveau des politiques.

	1	2	3	4	5	6	7	8	9	10	Je ne sais pas
1-Plus faible -> Plus élevé-10	○	○	○	○	○	○	○	○	○	○	○

* LM 5.10 Les partenariats entre OSC : Mesure dans laquelle les OSC ont formé des alliances nationales et des partenariats régionaux pour renforcer le marché du travail.

	1	2	3	4	5	6	7	8	9	10	Je ne sais pas
1-Plus faible -> Plus élevé-10	○	○	○	○	○	○	○	○	○	○	○

LM 5.11. Commentaires : Veuillez utiliser cette section pour nous faire part de tout commentaire additionnel sur le niveau d'effort, les défis ou les succès du pays autour des organisations de la société civile dans le secteur du marché du travail. Si vous avez répondu « Je ne sais pas » à l'une des questions précédentes, veuillez utiliser cette section pour expliquer pourquoi.

JOHNS HOPKINS
BLOOMBERG SCHOOL of PUBLIC HEALTH

Bill & Melinda Gates Institute for Population and Reproductive Health

Enquête de l'Indice des efforts pour le dividende démographique

Module : Résilience et durabilité du secteur

Étant donné l'impact sanitaire et socioéconomique de la pandémie de COVID-19, et son impact probable sur la progression vers le DD, cet indice des efforts pour le DD intègre des questions pour évaluer la résilience et la durabilité des systèmes dans les secteurs clés du DD. Les éléments couverts par les questions liées à la pandémie de COVID-19 ci-dessous relèvent des dimensions clés des systèmes résilients et visent à apporter des informations déterminantes sur le potentiel d'une réponse efficace aux menaces de maladies infectieuses émergentes et autres crises de santé publique.
Veuillez noter chaque élément sur une échelle de 1 à 10, 1 étant le score le plus faible (faible état/capacité) et 10 le plus élevé (très bon état/capacité).

* Dimension physique : structures, équipements, états du système et capacités.

	1	2	3	4	5	6	7	8	9	10	Je ne sais pas
LM-M1 - Plan/Préparation - État et capacité de l'équipement, du personnel et de la structure du secteur du marché du travail avant la crise. (1 = faible état/capacité ; 10 = très bon état/capacité)	◯	◯	◯	◯	◯	◯	◯	◯	◯	◯	◯
LM-M2 - Absorption – Mesure dans laquelle le secteur du marché du travail a reconnu la pandémie de COVID-19 et pu continuer à maintenir un niveau d'emploi et une productivité économique optimale.	◯	◯	◯	◯	◯	◯	◯	◯	◯	◯	◯

135

	1	2	3	4	5	6	7	8	9	10	Je ne sais pas
LM-M3 - Récupération – Mesure dans laquelle le secteur du marché du travail été capable d’induire des changements pour récupérer un niveau de fonctionnalité pré-COVID-19.	○	○	○	○	○	○	○	○	○	○	○
LM-M4 - Récupération – Gestion du financement et des bailleurs de fonds : Mesure dans laquelle il existe des mécanismes établis de protection sociale (et autres formes de stabilisation des revenus) pour les travailleurs sans protection (ex : les travailleurs indépendants, qui non pas accès au congé payé.)	○	○	○	○	○	○	○	○	○	○	○
LM-M5 - Adaptation – Gestion du financement et des bailleurs de fonds : Mesure dans laquelle des réformes institutionnelles et politiques ont été initiées pour renforcer la résilience par une approche déterminée par la demande à travers des systèmes de protection sociale à grande échelle pouvant agir comme stabilisateurs économiques.	○	○	○	○	○	○	○	○	○	○	○

136

* Dimension informatique : création, gestion, stockage et utilisation des données.

	1	2	3	4	5	6	7	8	9	10	Je ne sais pas
LM-M6 - Plan/Préparation – Mesure dans laquelle les données liées au marché du travail étaient suffisamment préparées, présentées, analysées et stockées avant la crise.	○	○	○	○	○	○	○	○	○	○	○
LM-M7 - Absorption – Connaissance : Mesure dans laquelle le secteur du marché du travail a pu conduire des évaluations en temps réel de son état de fonctionnement, en anticipation des pertes en cascade de la productivité du marché du travail.	○	○	○	○	○	○	○	○	○	○	○
LM-M8 - Mesure dans laquelle les données du marché du travail ont été utilisées pour suivre les progrès vers la récupération et anticiper des scenarii de récupération.	○	○	○	○	○	○	○	○	○	○	○
LM-M9 - Adaptation – Mesure dans laquelle le secteur du marché du travail crée et améliore ses protocoles de stockage et utilisation des données en temps réel.	○	○	○	○	○	○	○	○	○	○	○

* Dimension cognitive : compréhension, modèles cognitifs, idées reçues, préjugés et valeurs.

	1	2	3	4	5	6	7	8	9	10	Je ne sais pas
LM-M10 - Planification/Préparation – Mesure dans laquelle le système de conception et les décisions opérationnelles du secteur du marché du travail étaient préparés à anticiper la crise.	○	○	○	○	○	○	○	○	○	○	○

	1	2	3	4	5	6	7	8	9	10	Je ne sais pas
LM-M11 - Absorption – Mesure dans laquelle le secteur du marché du travail a des protocoles de contingence suffisants et une gestion de crise proactive.	○	○	○	○	○	○	○	○	○	○	○
LM-M12 - Absorption - Leadership et management : Mesure dans laquelle le gouvernement garantit aux salariés/ travailleurs et leurs famille une protection contre les risques sanitaires de la pandémie de COVID-19.	○	○	○	○	○	○	○	○	○	○	○
LM-M13 - Absorption – Redevabilité : Niveau d'effort pour assurer une redevabilité optimale vis-à-vis des ressources allouées à la protection contre les risques sanitaires de la pandémie de COVID-19 et la récupération du marché du travail dans le cadre de la réponse pour mitiger les impacts du COVID-19.	○	○	○	○	○	○	○	○	○	○	○
LM-M14 - Récupération – Mesure dans laquelle les décisions sont orientées vers la récupération et communiquées aux membres de la communauté sur la base de données probantes pour promouvoir des comportements sûrs.	○	○	○	○	○	○	○	○	○	○	○

	1	2	3	4	5	6	7	8	9	10	Je ne sais pas
LM-M15 - Récupération – Connaissance/Conscience : Mesure dans laquelle le secteur établit une certaine confiance et un dialogue entre les employeurs et les salariés pour renforcer l'engagement d'action commune du gouvernement et promouvoir le dialogue social au niveau des entreprises.	○	○	○	○	○	○	○	○	○	○	○
LM-M16 - Adaptation – Mesure dans laquelle le secteur du marché du travail conçoit de nouvelles configurations systémiques, objectifs et critères de décision.	○	○	○	○	○	○	○	○	○	○	○
LM-M17 - Adaptation – Adaptatif : Mesure dans laquelle le gouvernement promeut des changements sur les lieux de travail pour protéger les travailleurs (ex : mesures de sécurité et gestes barrières, prévention de la discrimination et de l'exclusion, accès élargi au congé payé, etc.).	○	○	○	○	○	○	○	○	○	○	○

* Dimension sociale : interaction, collaboration et auto-synchronisation entre les personnes, les entités et les institutions.

	1	2	3	4	5	6	7	8	9	10	Je ne sais pas
LM-M18 - Planification /Préparation – Mesure dans laquelle une formation sur la gestion d'épidémies/crises a été menée et a permis de mettre à profit les réseaux sociaux, le capital social et les normes institutionnelles et culturelles avant la crise.	○	○	○	○	○	○	○	○	○	○	○

	1	2	3	4	5	6	7	8	9	10	Je ne sais pas
LM-M19 - Absorption – Mesure dans laquelle le personnel et les institutions sociales ont été accessibles et ont fait preuve d'ingéniosité dans leur réponse à l'épidémie/crise.	◯	◯	◯	◯	◯	◯	◯	◯	◯	◯	◯
LM-M20 - Absorption – Autorégulation : Mesure dans laquelle les dirigeants nationaux avaient l'autorité d'effectuer des changements sectoriels en temps opportun à travers une infrastructure du marché du travail flexible.	◯	◯	◯	◯	◯	◯	◯	◯	◯	◯	◯
LM-M21 - Récupération – Mesure dans laquelle le secteur du marché du travail est impliqué dans le partage de connaissances et d'équipes pour améliorer la récupération du système.	◯	◯	◯	◯	◯	◯	◯	◯	◯	◯	◯
LM-M22 - Récupération – Diversité : Mesure dans laquelle le gouvernement facilite une récupération solide et rapide du marché du travail en diversifiant les politiques favorisant à la fois l'offre (production de biens et services) et la demande (consommation et investissement).	◯	◯	◯	◯	◯	◯	◯	◯	◯	◯	◯
LM-M23 - Adaptation – Mesure dans laquelle des ajouts ou modifications sont apportés aux institutions, politiques, formations, programmes et à la culture du secteur du marché du travail.	◯	◯	◯	◯	◯	◯	◯	◯	◯	◯	◯

	1	2	3	4	5	6	7	8	9	10	Je ne sais pas
LM-M24 - Adaptation - Leadership et management : Mesure dans laquelle il y a eu un dialogue tripartite fluide entre le gouvernement, les organisations des travailleurs/salariés et celles des employeurs.	○	○	○	○	○	○	○	○	○	○	○

* LM M-25 Temporalité : Mesure de la rapidité de la réaction du gouvernement –sa communication et mise en œuvre des mesures- pour mitiger l'impact immédiat et sur le long terme de la pandémie de COVID-19. Cette question porte sur la riposte nationale dans son ensemble, et non la réponse spécifique de votre secteur.

	1	2	3	4	5	6	7	8	9	10	Je ne sais pas
1-Plus faible -> Plus élevé-10	○	○	○	○	○	○	○	○	○	○	○

LM M-26 Commentaires : Utilisez cette section pour apporter tout commentaire additionnel sur la résilience et la durabilité des systèmes du marché du travail en vous référant à votre expérience de la pandémie de COVID-19. Si vous avez répondu « Je ne sais pas » à l'une des questions, utilisez cette section pour expliquer pourquoi.

JOHNS HOPKINS
BLOOMBERG SCHOOL
of PUBLIC HEALTH

Bill & Melinda Gates Institute for Population and Reproductive Health

Enquête de l'Indice des efforts pour le dividende démographique

Questionnaire sur la gouvernance et les institutions économiques (GIE)

Répondez aux questions suivantes en fonction de votre expérience/ expertise dans ce secteur spécifique.

Afin d'obtenir une représentation synthétique des efforts nationaux pour le dividende démographique, notez les éléments suivants sur une échelle de 1 à 10, 1 étant le score le plus faible (efforts très faibles ou quasi-inexistants) et 10 le plus élevé (efforts robustes). Le cas échéant, si une politique ou activité n'existe pas, répondez 0.

Donnez un score à chaque élément. Toutes les réponses seront enregistrées au format illustré ci-dessous :

Effort le plus faible	1	2	3	4	5	6	7	8	9	10	Effort le plus élevé	Je ne sais pas

JOHNS HOPKINS
BLOOMBERG SCHOOL
of PUBLIC HEALTH

Bill & Melinda Gates Institute for Population and Reproductive Health

Enquête de l'Indice des efforts pour le dividende démographique

Domaine 1. Politique/ Prise de décision

* GEI 1.1 Les valeurs démocratiques : Mesure dans laquelle le gouvernement a ratifié, appliqué à son contexte national et mis en œuvre les instruments régionaux sur les valeurs communes, y compris ceux liés à la démocratie, les élections, la gouvernance et le droit à la santé.

	1	2	3	4	5	6	7	8	9	10	Je ne sais pas
1-Plus faible -> Plus élevé-10	○	○	○	○	○	○	○	○	○	○	○

* GEI 1.2 Le suivi des valeurs démocratiques : Mesure dans laquelle le gouvernement a créé des mécanismes de rapport régulier et périodique sur la mise en œuvre des instruments régionaux de valeurs communes à travers les mécanismes existants tels que les revues périodiques universelles du statut des instruments internationaux ou régionaux, ou à travers des entités indépendantes. (Cela peut prendre la forme de rapports réguliers sur le statut de la mise en œuvre).

	1	2	3	4	5	6	7	8	9	10	Je ne sais pas
1-Plus faible -> Plus élevé-10	○	○	○	○	○	○	○	○	○	○	○

* GEI 1.3 L'affirmation de la participation démocratique des jeunes : Mesure dans laquelle le gouvernement a explicitement abrogé toutes les lois et contraintes discriminatoires empêchant la pleine participation des jeunes au processus électoral à travers des lois électorales et constitutions inclusives. (Cela peut inclure le développement et/ou renforcement des programmes de leadership des jeunes et/ou le renforcement de compétences en management et leadership).

	1	2	3	4	5	6	7	8	9	10	Je ne sais pas
1-Plus faible -> Plus élevé-10	○	○	○	○	○	○	○	○	○	○	○

* GEI 1.4. La suppression des barrières légales auxquelles se heurtent les jeunes : Mesure dans laquelle le gouvernement a proscrit toutes les lois posant des barrières au plein exercice et à la pleine jouissance des droits fondamentaux des jeunes à participer pleinement aux processus de gouvernance démocratique au niveau continental, régional, national et communautaire/à la base. (Cela pourrait aussi inclure le soutien de l'intégration des questions de droits et de santé des jeunes et adolescents à l'agenda du gouvernement sur la croissance et la transformation du pays).

	1	2	3	4	5	6	7	8	9	10	Je ne sais pas
1-Plus faible -> Plus élevé-10	○	○	○	○	○	○	○	○	○	○	○

* GEI 1.5 La politique de la jeunesse : Mesure dans laquelle la politique de la jeunesse du pays reflète les paradigmes éducatifs, de formation et d'emploi les plus récents.

	1	2	3	4	5	6	7	8	9	10	Je ne sais pas
1-Plus faible -> Plus élevé-10	○	○	○	○	○	○	○	○	○	○	○

* GEI 1.6 L'affirmation de la participation à la vie démocratique/ révision de la loi coutumière : Mesure dans laquelle le gouvernement a examiné, révisé, amendé ou aboli toutes les lois, régulations, politiques, pratiques et coutumes ayant un effet discriminatoire sur les jeunes, particulièrement les filles et les jeunes filles, sans distinction aucune, s'est assuré que les dispositions des différents systèmes légaux soient conformes aux droits humains internationaux. (Cela doit impérativement inclure la protection contre les pratiques malfaisantes, telles que le mariage précoce ou forcé, la violence sexuelle et basée sur le genre [VBG], et la mutilation génitale féminine [MGF]).

	1	2	3	4	5	6	7	8	9	10	Je ne sais pas
1-Plus faible -> Plus élevé-10	○	○	○	○	○	○	○	○	○	○	○

* GEI 1.7 La participation des jeunes dans la prise de décision politique : Mesure dans laquelle le gouvernement veille à l'implication des jeunes leaders et à leur participation dans les processus de prise de décision politique, y compris la politique sur la jeunesse et l'éducation. (Cela peut inclure les efforts visant à établir un parlement ou une assemblée nationale de jeunes. Cela peut aussi inclure le soutien à la participation et au leadership des jeunes et adolescents dans la planification et les programmes de santé à tous les niveaux de gouvernance).

	1	2	3	4	5	6	7	8	9	10	Je ne sais pas
1-Plus faible -> Plus élevé-10	○	○	○	○	○	○	○	○	○	○	○

Rusatira JC, *et al. BMJ Open* 2023; 13:e059937. doi: 10.1136/bmjopen-2021-059937

* GEI 1.8 L'État de droit : Mesure dans laquelle l'État de droit est formellement et légalement établi de manière équitable et mis en œuvre indépendamment par le pouvoir judiciaire.

	1	2	3	4	5	6	7	8	9	10	Je ne sais pas
1-Plus faible -> Plus élevé-10	◯	◯	◯	◯	◯	◯	◯	◯	◯	◯	◯

* GEI 1.9 La transparence et la redevabilité : Mesure dans laquelle la législation nationale promeut la transparence et la redevabilité de toutes les branches et secteurs.

	1	2	3	4	5	6	7	8	9	10	Je ne sais pas
GEI 1.9.1 Mesure dans laquelle le public peut accéder aux informations législatives et aux registres sur les organisations publiques.	◯	◯	◯	◯	◯	◯	◯	◯	◯	◯	◯
GEI 1.9.2 Mesure dans laquelle il existe des mécanismes de redevabilité rendant cette dernière obligatoire et des sanctions associées.	◯	◯	◯	◯	◯	◯	◯	◯	◯	◯	◯

* GEI 1.10 La sécurité nationale : Mesure dans laquelle le pays/gouvernement est impliqué dans un conflit armé, qu'il soit international ou intérieur.

	1	2	3	4	5	6	7	8	9	10	Je ne sais pas
GEI 1.10.1 Mesure dans laquelle le pays souffre de tensions transfrontalières.	◯	◯	◯	◯	◯	◯	◯	◯	◯	◯	◯
GEI 1.10.2 Mesure dans laquelle le pays contient des personnes internement déplacées et/ou des réfugiés.	◯	◯	◯	◯	◯	◯	◯	◯	◯	◯	◯
GEI 1.10.3 Mesure dans laquelle les établissements éducatifs sont protégés et des opportunités d'apprentissage alternatives créées pour les enfants et les jeunes déplacées ou vivant en situation de conflit.	◯	◯	◯	◯	◯	◯	◯	◯	◯	◯	◯

* GEI 1.11 Les droits : Mesure dans laquelle la constitution affirme certains droits : liberté d'expression, liberté d'association et d'assemblée, droits et libertés civiques, et protection contre la discrimination ethnique et religieuse ; et mesure dans laquelle la violation des droits humains par le gouvernement est improbable.

	1	2	3	4	5	6	7	8	9	10	Je ne sais pas
1-Plus faible -> Plus élevé-10	◯	◯	◯	◯	◯	◯	◯	◯	◯	◯	◯

* GEI 1.12 L'éducation et les valeurs démocratiques : Mesure dans laquelle les politiques, stratégies et programmes d'éducation promeuvent la liberté de penser, les valeurs démocratiques et l'éducation comme un droit fondamental/humain.

	1	2	3	4	5	6	7	8	9	10	Je ne sais pas
1-Plus faible -> Plus élevé-10	◯	◯	◯	◯	◯	◯	◯	◯	◯	◯	◯

* GEI 1.13 L'affirmation des environnements d'apprentissage sûrs : Mesure dans laquelle les politiques d'éducation garantissent des environnements d'apprentissage sûrs à travers la protection de l'enfance. (Cela peut se manifester par la protection de la sécurité des filles, l'interdiction des punitions corporelles, etc.).

	1	2	3	4	5	6	7	8	9	10	Je ne sais pas
1-Plus faible -> Plus élevé-10	◯	◯	◯	◯	◯	◯	◯	◯	◯	◯	◯

GEI 1.14 Commentaires : Utilisez cette section pour nous faire part de tout commentaire additionnel sur le niveau d'effort, les défis ou les succès du pays autour des politiques/ de la prise de décision dans le secteur de la gouvernance et des institutions économiques. Si vous avez répondu « Je ne sais pas » à l'une des questions précédentes, veuillez utiliser cette section pour expliquer pourquoi.

JOHNS HOPKINS
BLOOMBERG SCHOOL
of PUBLIC HEALTH

Bill & Melinda Gates Institute for Population and Reproductive Health

Enquête de l'Indice des efforts pour le dividende démographique

Domaine 2. Services ou programmes

* GEI 2.1 Renforcer les activités/ institutions pour la jeunesse : Mesure dans laquelle des efforts sont menés pour renforcer les formations, réseaux et organisations indépendantes de jeunes, y compris l'établissement de commissions indépendantes de jeunes et de syndicats d'étudiants au niveau national et infranational pour promouvoir les droits des jeunes.

	1	2	3	4	5	6	7	8	9	10	Je ne sais pas
1-Plus faible -> Plus élevé-10	○	○	○	○	○	○	○	○	○	○	○

* GEI 2.2 La nomination de jeunes leaders : Si et mesure dans laquelle un jeune représentant a été nommé pour diriger le plaidoyer et promouvoir la priorisation des questions concernant les jeunes (ex : éducation, formation et emploi) dans les espaces de prise de décision à différents niveaux, y compris au parlement.

	1	2	3	4	5	6	7	8	9	10	Je ne sais pas
1-Plus faible -> Plus élevé-10	○	○	○	○	○	○	○	○	○	○	○

* GEI 2.3 Les idéaux et aspirations des jeunes : Mesure dans laquelle les programmes sont conçus pour instituer le leadership des jeunes, et mesure dans laquelle les formations pour l'autonomisation des jeunes visent à approfondir les idéaux et aspirations culturelles des jeunes.

	1	2	3	4	5	6	7	8	9	10	Je ne sais pas
1-Plus faible -> Plus élevé-10	○	○	○	○	○	○	○	○	○	○	○

* GEI 2.4 Les services des jeunes : Mesure dans laquelle les programmes sont conçus pour répondre aux besoins spécifiques des jeunes. (Cela devrait prendre en compte le développement et la mise en œuvre de services de santé amis des jeunes et adolescents, entre autres programmes similaires).

	1	2	3	4	5	6	7	8	9	10	Je ne sais pas
1-Plus faible -> Plus élevé-10	◯	◯	◯	◯	◯	◯	◯	◯	◯	◯	◯

* GEI 2.5 L'implication des jeunes dans les initiatives nationales : Mesure dans laquelle des efforts sont menés pour renforcer les initiatives existantes du pays en matière d'engagement et de participation des jeunes (e*x : au niveau de l'Union africaine, cela peut inclure le Corps des Jeunes Volontaires de l'Union Africaine, les Clubs de Jeunes de l'Union Africaine, et les activités de AGA-YES, dont le comité consultatif de jeunes, les procès fictifs et le dialogue annuel des jeunes sur la démocratie, les droits de l'Homme et la gouvernance).* (Cela peut inclure l'implication des jeunes et des adolescents dans l'assistance technique apportée aux ministères et entités gouvernementales fédérales ou régionales compétentes pour l'élaboration de politiques, plans et programmes qui intègrent les questions de santé prioritaires affectant ce groupe).

	1	2	3	4	5	6	7	8	9	10	Je ne sais pas
1-Plus faible -> Plus élevé-10	◯	◯	◯	◯	◯	◯	◯	◯	◯	◯	◯

* GEI 2.6 La sécurité : Mesure dans laquelle la sécurité personnelle est garantie à travers des services/ activités de police fiables, contribuant à une perception générale positive de la sécurité des personnes dans les ménages, les communautés et en milieu scolaire. (Cela pourrait inclure la protection des jeunes contre tout type d'abus, négligence ou manque de respect dans l'utilisation des services de santé).

	1	2	3	4	5	6	7	8	9	10	Je ne sais pas
1-Plus faible -> Plus élevé-10	◯	◯	◯	◯	◯	◯	◯	◯	◯	◯	◯

* GEI 2.7 La participation : Mesure dans laquelle les fonctions du gouvernement impliquent la participation des citoyens, des organisations de la société civile et autres acteurs intersectoriels pour promouvoir des élections démocratiques et le pouvoir effectif de gouverner.

	1	2	3	4	5	6	7	8	9	10	Je ne sais pas
1-Plus faible -> Plus élevé-10	◯	◯	◯	◯	◯	◯	◯	◯	◯	◯	◯

* GEI 2.8 Le genre : Mesure dans laquelle les femmes participent à :

	1	2	3	4	5	6	7	8	9	10	Je ne sais pas
GEI 2.8.1 La gouvernance au niveau national											
GEI 2.8.2 La gouvernance au niveau infranational											
GEI 2.8.3 La gouvernance en milieu rural											
GEI 2.8.4 La gouvernance en milieu urbain											
GEI 2.8.5 Judiciaire											

* GEI 2.9 La promotion de l'égalité : Mesure dans laquelle la parité est promue par le gouvernement dans tous les aspects de la société. (Ceci peut inclure la parité et l'égalité dans l'éducation, devant la loi, dans le système de santé, à l'embauche et en termes de rémunération, entre autres facettes de la société).

	1	2	3	4	5	6	7	8	9	10	Je ne sais pas
1-Plus faible -> Plus élevé-10											

* GEI 2.10 La durabilité des opportunités économiques :

	1	2	3	4	5	6	7	8	9	10	Je ne sais pas
GEI 2.10.1 Mesure dans laquelle le gouvernement pourvoit et s'assure qu'un système de gestion publique solide soit réalisé.	◯	◯	◯	◯	◯	◯	◯	◯	◯	◯	◯
GEI 2.10.2 Mesure dans laquelle le gouvernement pourvoit et s'assure qu'un environnement commercial solide soit réalisé.	◯	◯	◯	◯	◯	◯	◯	◯	◯	◯	◯
GEI 2.10.3 Mesure dans laquelle le gouvernement pourvoit et s'assure qu'une infrastructure publique solide soit réalisée.	◯	◯	◯	◯	◯	◯	◯	◯	◯	◯	◯
GEI 2.10.4 Mesure dans laquelle le gouvernement pourvoit et s'assure qu'un secteur rural fort pour le développement soit réalisé.	◯	◯	◯	◯	◯	◯	◯	◯	◯	◯	◯

* GEI 2.11 Le développement humain :

	1	2	3	4	5	6	7	8	9	10	Je ne sais pas
GEI 2.11.1 Mesure dans laquelle le bien-être du public en général est adéquatement géré par le gouvernement (y compris, mais sans s'y limiter, les politiques et services de sécurité sociale, l'intégration socioéconomique et la protection sociale des jeunes, entre autres sujets). Cela peut aussi impliquer la mesure dans laquelle le gouvernement, ayant l'obligation de protéger, remplir et faire respecter les droits fondamentaux de santé, a donné la responsabilité de leur mise en œuvre à son cabinet ministériel.	○	○	○	○	○	○	○	○	○	○	○
GEI 2.11.2 Mesure dans laquelle le gouvernement promeut le développement de compétences de la population sur les lieux de travail.	○	○	○	○	○	○	○	○	○	○	○
GEI 2.11.3 Mesure dans laquelle le gouvernement promeut l'éducation, le développement de connaissances et des capacités de la population à travers l'école et des opportunités de formation professionnelle continue.	○	○	○	○	○	○	○	○	○	○	○
GEI 2.11.4 Mesure dans laquelle le gouvernement promeut la santé et le bien-être de la population.	○	○	○	○	○	○	○	○	○	○	○

GEI 2.12 Commentaires : Utilisez cette section pour nous faire part de tout commentaire additionnel sur le niveau d'effort, les défis ou les succès du pays autour des services ou programmes dans le secteur de la gouvernance et des institutions économiques. Si vous avez répondu « Je ne sais pas » à l'une des questions précédentes, veuillez utiliser cette section pour expliquer pourquoi.

Bill & Melinda Gates Institute for Population and Reproductive Health

Enquête de l'Indice des efforts pour le dividende démographique

Domaine 3. Plaidoyer

* GEI 3.1 L'autonomisation des jeunes : Mesure dans laquelle l'autonomisation des jeunes a été améliorée, potentiellement à travers l'intégration de l'éducation civique au programme d'éducation scolaire nationale, aux plateformes médiatiques et autres canaux pour inculquer les principes de l'État de droit, les droits humains, et les devoirs et responsabilités individuelles. (Cela peut comprendre l'inclusion de la santé des jeunes et des adolescents et des questions relatives au droit dans l'agenda de croissance et transformation du pays et dans les programmes scolaires).

	1	2	3	4	5	6	7	8	9	10	Je ne sais pas
1-Plus faible -> Plus élevé-10	○	○	○	○	○	○	○	○	○	○	○

* GEI 3.2 Le plaidoyer du gouvernement : Mesure dans laque le gouvernement plaide pour des améliorations au sein de ses propres structures et priorités. (Ces améliorations peuvent comprendre des avancées vers l'inclusion, les opportunités, les programmes et les droits des jeunes, ou autres concepts similaires dans différentes populations.).

	1	2	3	4	5	6	7	8	9	10	Je ne sais pas
1-Plus faible -> Plus élevé-10	○	○	○	○	○	○	○	○	○	○	○

* GEI 3.3 Le plaidoyer non-gouvernemental : Mesure dans laquelle les acteurs du gouvernement plaident pour des améliorations des structures et priorités du gouvernement. (Ces améliorations peuvent comprendre des avancées vers l'inclusion les opportunités, les programmes et les droits des jeunes, ou autres concepts similaires dans différentes populations.).

	1	2	3	4	5	6	7	8	9	10	Je ne sais pas
1-Plus faible -> Plus élevé-10	○	○	○	○	○	○	○	○	○	○	○

* GEI 3.4 La voix du plaidoyer : Mesure dans laquelle les plaideurs se font entendre dans les discussions gouvernementales.

	1	2	3	4	5	6	7	8	9	10	Je ne sais pas
1-Plus faible -> Plus élevé-10	○	○	○	○	○	○	○	○	○	○	○

* GEI 3.5 La voix des secteurs clés du DD : Mesure dans laquelle le gouvernement et les parties prenantes sont engagés à établir des institutions durables pour assurer :

	1	2	3	4	5	6	7	8	9	10	Je ne sais pas
GEI 3.5.1 Bonne gouvernance	○	○	○	○	○	○	○	○	○	○	○
GEI 3.5.2 Stabilité financière	○	○	○	○	○	○	○	○	○	○	○
GEI 3.5.3 Programmes optimaux de planification familiale	○	○	○	○	○	○	○	○	○	○	○
GEI 3.5.4 Programmes optimaux de santé maternelle et infantile	○	○	○	○	○	○	○	○	○	○	○
GEI 3.5.5 Éducation de bonne qualité	○	○	○	○	○	○	○	○	○	○	○
GEI 3.5.6 Marché du travail diversifié	○	○	○	○	○	○	○	○	○	○	○

GEI 3.6 Commentaires : Utilisez cette section pour nous faire part de tout commentaire additionnel sur le niveau d'effort, les défis ou les succès du pays autour du plaidoyer dans le secteur de la gouvernance et des institutions économiques. Si vous avez répondu « Je ne sais pas » à l'une des questions précédentes, veuillez utiliser cette section pour expliquer pourquoi.

JOHNS HOPKINS
BLOOMBERG SCHOOL
of PUBLIC HEALTH

Bill & Melinda Gates Institute for Population and Reproductive Health

Enquête de l'Indice des efforts pour le dividende démographique

Domaine 4. Recherche

* GEI 4.1 La force des observatoires/parties prenantes/groupes de travail sur la gouvernance et les institutions économiques (GIE) : Mesure dans laquelle les parties prenantes de la gouvernance et des institutions économiques du pays (ex : groupe directif de parties prenantes, groupes de travail techniques, observatoire de la gouvernance, groupe de coordination ou facilitation) :

	1	2	3	4	5	6	7	8	9	10	Je ne sais pas
GEI 4.1.1 Ont un représentant du gouvernement, des institutions de formation, de la société civile, des organisations non gouvernementales et confessionnelles, des associations professionnelles, du secteur privé, etc.	○	○	○	○	○	○	○	○	○	○	○
GEI 4.1.2 Se réunissent régulièrement, émettent des rapports et recommandent des politiques aux directions des ministères compétents.	○	○	○	○	○	○	○	○	○	○	○
GEI 4.1.3 Ont un impact sur la PF dans le pays.	○	○	○	○	○	○	○	○	○	○	○

* GEI 4.2 La stratégie de recherche sur la gouvernance : Mesure dans laquelle les documents de planification nationale comprennent une stratégie/approche complète de recherche liée à la GIE.

	1	2	3	4	5	6	7	8	9	10	Je ne sais pas
1-Plus faible -> Plus élevé-10	○	○	○	○	○	○	○	○	○	○	○

* GEI 4.3 Les partenaires collectant des données sur la gouvernance, la finance et le développement :

	1	2	3	4	5	6	7	8	9	10	Je ne sais pas
GEI 4.3.1 Mesure dans laquelle la collecte des données est entreprise par des agences gouvernementales. (Cela peut comprendre les agences/bureaux de la statistique et/ou les ministères de l'économie/finances, éducation, santé, genre, etc.).	◯	◯	◯	◯	◯	◯	◯	◯	◯	◯	◯
GEI 4.3.2 Mesure dans laquelle la collecte de données est entreprise par des institutions de recherche.	◯	◯	◯	◯	◯	◯	◯	◯	◯	◯	◯
GEI 4.3.3 Mesure dans laquelle la collecte des données est entreprise par des chercheurs indépendants.	◯	◯	◯	◯	◯	◯	◯	◯	◯	◯	◯

* GEI 4.4 La recherche thématique :

	1	2	3	4	5	6	7	8	9	10	Je ne sais pas
GEI 4.4.1 Mesure dans laquelle de la recherche est menée sur la sécurité et l'État de droit.	◯	◯	◯	◯	◯	◯	◯	◯	◯	◯	◯
GEI 4.4.2 Mesure dans laquelle la participation politique et les droits humains sont mesurés.	◯	◯	◯	◯	◯	◯	◯	◯	◯	◯	◯
GEI 4.4.3 Mesure dans laquelle de la recherche est menée sur les indicateurs de l'économie durable et est disséminée à la communauté.	◯	◯	◯	◯	◯	◯	◯	◯	◯	◯	◯
GEI 4.4.4 Mesure dans laquelle de la recherche est menée sur le développement du capital humain (éducation, formation et emploi, etc.).	◯	◯	◯	◯	◯	◯	◯	◯	◯	◯	◯

* GEI 4.5 La qualité/couverture des données :

	1	2	3	4	5	6	7	8	9	10	Je ne sais pas
GEI 4.5.1 Mesure dans laquelle le système statistique de routine fournit des informations périodiques sur la GIE.	○	○	○	○	○	○	○	○	○	○	○
GEI 4.5.2 Mesure dans laquelle le système statistique de routine fournit des informations périodiques fiables sur les besoins des populations de différentes communautés en matière de gouvernance.	○	○	○	○	○	○	○	○	○	○	○
GEI 4.5.3 Mesure dans laquelle des évaluations des systèmes de suivi et surveillance sont conduites et appliquées pour assurer la fiabilité des données.	○	○	○	○	○	○	○	○	○	○	○

* GEI 4.6 La qualité des institutions de recherche : Mesure dans laquelle le pays a la capacité de soutenir et maintenir des institutions de recherche qui développent des études et/ou collectent des données permettant de soutenir la bonne gouvernance, la stabilité de l'économie et la solidité des institutions publiques.

	1	2	3	4	5	6	7	8	9	10	Je ne sais pas
1-Plus faible -> Plus élevé-10	○	○	○	○	○	○	○	○	○	○	○

* GEI 4.7 L'évaluation : Mesure dans laquelle les statistiques, enquêtes et études sont utilisées par du personnel spécialisé pour générer des rapports sur la bonne gouvernance et les institutions financières

	1	2	3	4	5	6	7	8	9	10	Je ne sais pas
1-Plus faible -> Plus élevé-10	○	○	○	○	○	○	○	○	○	○	○

* GEI 4.8 L'utilisation des résultats d'évaluation par les gestionnaires de programmes : Mesure dans laquelle les gestionnaires locaux de programmes utilisent les conclusions de recherche et d'évaluations pour améliorer l'État de droit, stabiliser l'économie et renforcer les institutions en suivant les recommandations émises.

	1	2	3	4	5	6	7	8	9	10	Je ne sais pas
1-Plus faible -> Plus élevé-10	○	○	○	○	○	○	○	○	○	○	○

* GEI 4.10 La dissémination d'informations à d'autres entités chargées de la mise en œuvre des programmes : Mesure dans laquelle des informations sont partagées ou disséminées entre géographies et à différents niveaux (national, état/province/comté, localités/sous-comtés).

	1	2	3	4	5	6	7	8	9	10	Je ne sais pas
1-Plus faible -> Plus élevé-10	○	○	○	○	○	○	○	○	○	○	○

GEI 4.11 Commentaires : Utilisez cette section pour nous faire part de tout commentaire additionnel sur le niveau d'effort, les défis ou les succès du pays autour de la recherche sur la gouvernance et les institutions économiques. Si vous avez répondu « Je ne sais pas » à l'une des questions précédentes, veuillez utiliser cette section pour expliquer pourquoi.

JOHNS HOPKINS
BLOOMBERG SCHOOL
of PUBLIC HEALTH

Bill & Melinda Gates Institute for Population and Reproductive Health

Enquête de l'Indice des efforts pour le dividende démographique

Domaine 5. Organisations de la société civile (OSC)

* GEI 5.1 Le pouvoir des acteurs des OSC : Mesure dans laquelle les acteurs des OSC occupent des postes d'influence pour contribuer positivement à l'État de droit.

	1	2	3	4	5	6	7	8	9	10	Je ne sais pas
1-Plus faible -> Plus élevé-10	◯	◯	◯	◯	◯	◯	◯	◯	◯	◯	◯

* GEI 5.2 L'analyse budgétaire comme outil des OSC : Mesure dans laquelle les OSC utilisent l'analyse budgétaire comme un outil pour développer le plaidoyer pour la bonne gouvernance, la stabilité de l'économie et la consolidation des institutions publiques.

	1	2	3	4	5	6	7	8	9	10	Je ne sais pas
1-Plus faible -> Plus élevé-10	◯	◯	◯	◯	◯	◯	◯	◯	◯	◯	◯

* GEI 5.3 Le soutien aux services à base communautaire : Mesure dans laquelle les OSC participent au rapprochement des informations et services liés à la gouvernance avec les communautés.

	1	2	3	4	5	6	7	8	9	10	Je ne sais pas
1-Plus faible -> Plus élevé-10	◯	◯	◯	◯	◯	◯	◯	◯	◯	◯	◯

* GEI 5.4 Les droits humains et la qualité de la mise en œuvre : Mesure dans laquelle les OSC plaident et soutiennent des approches de gouvernance fondées sur le droit pour veiller à ce que les bénéficiaires de projets et programmes puissent voir des avancées selon leurs propres termes.

	1	2	3	4	5	6	7	8	9	10	Je ne sais pas
1-Plus faible -> Plus élevé-10	◯	◯	◯	◯	◯	◯	◯	◯	◯	◯	◯

* GEI 5.5 Les jeunes : Mesure dans laquelle les OSC aident les jeunes à participer à l'éducation, la formation, l'emploi et le leadership.

	1	2	3	4	5	6	7	8	9	10	Je ne sais pas
1-Plus faible -> Plus élevé-10	○	○	○	○	○	○	○	○	○	○	○

* GEI 5.6 Les programmes pour les femmes : Mesure dans laquelle les OSC veillent à ce que les politiques de GIE soutiennent la participation des femmes et des filles au leadership.

	1	2	3	4	5	6	7	8	9	10	Je ne sais pas
1-Plus faible -> Plus élevé-10	○	○	○	○	○	○	○	○	○	○	○

* GEI 5.7 Le plaidoyer/ redevabilité :

	1	2	3	4	5	6	7	8	9	10	Je ne sais pas
GEI 5.7.1 Mesure dans laquelle les OSC et les médias soutiennent la participation de la communauté dans la gouvernance et l'application de l'État de droit.	○	○	○	○	○	○	○	○	○	○	○
GEI 5.7.2 Mesure dans laquelle les médias soutiennent la participation de la communauté à la gouvernance ou au respect de l'État de droit.	○	○	○	○	○	○	○	○	○	○	○

* GEI 5.8 Les évaluations et le suivi menés par les OSC : Mesure dans laquelle les OSC évaluent, suivent et émettent des rapports sur l'efficacité des politiques et des mécanismes et structures de redevabilité du gouvernement.

	1	2	3	4	5	6	7	8	9	10	Je ne sais pas
1-Plus faible -> Plus élevé-10	○	○	○	○	○	○	○	○	○	○	○

* GEI 5.9 Les partenariats entre OSC : Mesure dans laquelle les OSC ont formé des alliances nationales et des partenariats régionaux pour renforcer leur position et potentiellement améliorer le leadership et les mécanismes de financement pour une bonne gouvernance et le renforcement institutionnel.

	1	2	3	4	5	6	7	8	9	10	Je ne sais pas
1-Plus faible -> Plus élevé-10	◯	◯	◯	◯	◯	◯	◯	◯	◯	◯	◯

GEI 5.10 Commentaires : Utilisez cette section pour nous faire part de tout commentaire additionnel sur le niveau d'effort, les défis ou les succès du pays autour des OSC dans le secteur de la gouvernance et des institutions économiques. Si vous avez répondu « Je ne sais pas » à l'une des questions précédentes, veuillez utiliser cette section pour expliquer pourquoi.

JOHNS HOPKINS
BLOOMBERG SCHOOL
of PUBLIC HEALTH

Bill & Melinda Gates Institute for Population and Reproductive Health

Enquête de l'Indice des efforts pour le dividende démographique

Module : Résilience et durabilité du secteur

Étant donné l'impact sanitaire et socioéconomique de la pandémie de COVID-19, et son impact probable sur la progression vers le DD, cet indice des efforts pour le DD intègre des questions pour évaluer la résilience et la durabilité des systèmes dans les secteurs clés du DD. Les éléments couverts par les questions liées à la pandémie de COVID-19 ci-dessous relèvent des dimensions clés des systèmes résilients et visent à apporter des informations déterminantes sur le potentiel d'une réponse efficace aux menaces de maladies infectieuses émergeantes et autres crises de santé publique.

Veuillez noter chaque élément sur une échelle de 1 à 10, 1 étant le score le plus faible (faible état/capacité) et 10 le plus élevé (très bon état/capacité).

* Dimension physique : structures, équipements, états du système et capacités.

	1	2	3	4	5	6	7	8	9	10	Je ne sais pas
GEI-M1 – Planification/Préparation : État et capacité de l'équipement, du personnel et de la structure du secteur GIE avant la crise. (1 = faible état/capacité ; 10 = très bon état/capacité)	○	○	○	○	○	○	○	○	○	○	○
GEI-M2 - Absorption - Mesure dans laquelle le système du secteur GIE a reconnu la pandémie de COVID-19 et pu maintenir sa fonctionnalité.	○	○	○	○	○	○	○	○	○	○	○

	1	2	3	4	5	6	7	8	9	10	Je ne sais pas
GEI-M3 - Absorption – Intégration : Niveau d'intégration des services et collaborations institutionnelles pour assurer la continuité de l'accès aux services essentiels pendant la crise COVID-19.	◯	◯	◯	◯	◯	◯	◯	◯	◯	◯	◯
GEI-M4 - Récupération – Mesure dans laquelle les institutions économiques et gouvernementales ont été capables de générer des changements pour récupérer un niveau de fonctionnalité pré-COVID-19.	◯	◯	◯	◯	◯	◯	◯	◯	◯	◯	◯
GEI-M5 - Récupération – Gestion du financement et des bailleurs de fonds : Banque nationale et soutien des bailleurs de fonds pour assurer la disponibilité de liquidités pendant la crise COVID-19.	◯	◯	◯	◯	◯	◯	◯	◯	◯	◯	◯
GEI-M6 - Adaptation – Mesure dans laquelle des changements ont été apportés pour améliorer la résilience du système et de l'infrastructure du secteur GIE.	◯	◯	◯	◯	◯	◯	◯	◯	◯	◯	◯

* Dimension informatique : création, gestion, stockage et utilisation des données.

	1	2	3	4	5	6	7	8	9	10	Je ne sais pas
GEI-M7 - Plan/Préparation – Mesure dans laquelle les données du secteur GIE étaient suffisamment préparées, présentées, analysées et stockées avant la crise.	○	○	○	○	○	○	○	○	○	○	○
GEI-M8 - Absorption – Connaissance : Niveau de connaissance/conscience des menaces causées par la pandémie de COVID-19 dans le gouvernement central.	○	○	○	○	○	○	○	○	○	○	○
GEI-M9 - Récupération – Mesure dans laquelle les données de GIE ont été utilisées pour suivre le progrès vers la récupération et anticiper des scenarii de récupération.	○	○	○	○	○	○	○	○	○	○	○
GEI-M10 - Adaptation – Mesure dans laquelle le système du secteur GIE crée et améliore actuellement ses protocoles de stockage et d'utilisation des données en temps réel.	○	○	○	○	○	○	○	○	○	○	○

* Dimension cognitive : compréhension, modèles cognitifs, idées reçues, préjugés et valeurs.

	1	2	3	4	5	6	7	8	9	10	Je ne sais pas
GEI-M11 - Planification/Préparation – Mesure dans laquelle le système du secteur GIE et les décisions opérationnelles étaient préparés à anticiper la crise	○	○	○	○	○	○	○	○	○	○	○

	1	2	3	4	5	6	7	8	9	10	Je ne sais pas
GEI-M12 - Absorption – Mesure dans laquelle la réponse du secteur GIE a des protocoles de contingence suffisants et une gestion de crise proactive.											
GEI-M13 - Absorption – Redevabilité : Niveau d'effort pour assurer une redevabilité optimale vis-à-vis des ressources allouées pour soutenir la gouvernance dans le cadre de la réponse à la pandémie de COVID-19.											
GEI-M14 - Récupération – Mesure dans laquelle les décisions sont orientées vers la récupération et communiquées aux communautés sur la base de données probantes pour promouvoir des comportements sûrs.											
GEI-M15 - Récupération – Connaissance/Conscience : Mesure dans laquelle le secteur de la PF établit une certaine confiance avec le public à travers sa communication et le dialogue pour la prise de décision et l'action politique fondées sur des données probantes.											
GEI-M16 - Adaptation – Mesure dans laquelle le système du secteur GIE conçoit de nouvelles configurations systémiques, objectifs et critères de décision.											
GEI-M17 - Adaptation – Adaptatif : Niveau d'adaptation des systèmes financiers et des services sociaux pour assurer et faciliter l'accès aux plateformes technologiques pendant la pandémie de COVID-19.											

* Social dimension: interaction, collaboration and self-synchronization between people, entities and institutions.

	1	2	3	4	5	6	7	8	9	10	Je ne sais pas
GEI-M18 - Planification /Préparation – Mesure dans laquelle une formation sur la gestion d'épidémies/crises a été menée et a permis de mettre à profit les réseaux sociaux, le capital social et les normes institutionnelles et culturelles avant la crise.	○	○	○	○	○	○	○	○	○	○	○
GEI-M19 - Absorption – Mesure dans laquelle le personnel et les institutions sociales ont été accessibles et ont fait preuve d'ingéniosité dans leur réponse à l'épidémie/crise.	○	○	○	○	○	○	○	○	○	○	○
GEI-M20 - Absorption - Self-regulation: Extent to which national leaders had the authority to effect timely sectoral changes through an infrastructure flexible.	○	○	○	○	○	○	○	○	○	○	○
GEI-M21 - Récupération – Mesure dans laquelle le secteur GIE est impliqué dans le partage de connaissances et d'équipes pour améliorer la récupération du système.	○	○	○	○	○	○	○	○	○	○	○
GEI-M22 - Récupération – Diversité : Niveau d'engagement d'une équipe multidisciplinaire pour mitiger les effets de la pandémie de COVID-19 au niveau national et infranational.	○	○	○	○	○	○	○	○	○	○	○
GEI-M23 - Adaptation - Leadership et management : Gérance et gestion démontrées par le gouvernement en réponse à la pandémie de COVID-19 au niveau national.	○	○	○	○	○	○	○	○	○	○	○

* GEI M-24 Temporalité : Mesure de la rapidité de la réaction du gouvernement –sa communication et mise en œuvre des mesures- pour mitiger l’impact immédiat et sur le long terme de la pandémie de COVID-19. *Cette question porte sur la riposte nationale dans son ensemble, et non la réponse spécifique de votre secteur.*

	1	2	3	4	5	6	7	8	9	10	Je ne sais pas
1-Plus faible -> Plus élevé-10	○	○	○	○	○	○	○	○	○	○	○

GEI M-25 Commentaires : Utilisez cette section pour apporter tout commentaire additionnel sur la résilience et la durabilité des systèmes nationaux de gouvernance et des institutions économiques en vous référant à votre expérience de la pandémie de COVID-19. Si vous avez répondu « Je ne sais pas » à l’une des questions, utilisez cette section pour expliquer pourquoi.
