# Supplementary material for: Development of the Demographic Dividend Effort Index, a novel tool to measure existing efforts to create a favourable environment to harness a demographic dividend: results from an experts’ survey from six sub-Saharan African countries
Source: BMJ Open. 2023 Mar 21;13(3):e059937. doi: 10.1136/bmjopen-2021-059937 (PMC10040031; doi:10.1136/bmjopen-2021-059937)
Supplement: Supplementary data [file bmjopen-2021-059937supp003.pdf]

Appendix 6.

Demographic Dividend Effort Index (DDEI) References to Source Indices

Family Planning (FP) DDEI Questionnaire

DDEI Questionnaire Item	Reference/ Source Index	Reference/ Source Index Item
Dimension 1. Policy/Policymaking		
*FP 1.2 **Policy on Fertility Reduction and FP**: Extent to which government policy emphasizes FP for family size as it relates to available resources or demographic reasons over health reasons or is simply neutral or opposed.	Family Planning Effort Index (FPEI)	Extent to which government policy stresses family planning for demographic reasons over health reasons or is simply neutral or opposed
*FP 1.4 **Policy on Age at Marriage**: Extent to which legal age at marriage for females is set at 18 years or higher and is enforced.	FPEI	Extent to which legal age at marriage for females is set at18 years or higher and is enforced
*FP 1.9 **Percent of in-country funding of FP budget**: Extent to which total family planning/population budget is derived from in-country sources (e.g., 1 for 10 percent, 5 for 50 percent, 10 for 100 percent).	FPEI	Extent to which total familyplanning/population budget isderived from in-countrysources (e.g., 1 for 10percent, 5 for 50 percent, 10for 100 percent)
FP 1.11.1 **Import Laws and Legal Regulations**: Extent to which import laws and legal regulations facilitate the importation of FP commodities and supplies	FPEI	Extent to which import laws and legal regulations facilitatethe importation ofcontraceptive supplies orextent to whichcontraceptives aremanufactured locally
*FP 1.11.2 **Import Laws and Legal Regulations**: Extent to which contraceptives are manufactured locally.	FPEI	Extent to which import laws and legal regulations facilitatethe importation ofcontraceptive supplies

Note

- *DDEI items are not always sourced from the reference index verbatim.*
- **Items that were dropped because they loaded poorly on the domain factors.*

**DDEI Questionnaire Item**	**Reference/ Source Index**	**Reference/ Source Index Item**
		orextent to whichcontraceptives aremanufactured locally
*FP 1.12 **Advertising of Contraceptives Allowed**: Extent of freedom from restrictions on advertising of contraceptives in the mass media.	FPEI	Extent of freedom from restrictions on advertising of contraceptives in the mass media
FP 1.14 **Political support for FP**: Extent to which elected officials in the country prioritize meeting FP needs to strengthen the FP contributions to the DD by passing laws and regulations and sponsoring actions and policies aimed at improving FP uptake and adherence.	FPEI	Extent to which the head of government, as well as other officials, speak publicly and favorably about family planning at least once or twice a year
Dimension 2. Services or Programs		
*FP 2.1 **Administrative Structure**: Extent to which administrative structure and staff at national, provincial and county levels are sufficient to implement the family planning program.	FPEI	Extent to which administrative structure and staff at national, provincial and county levels are adequate to implement the family planning program
*FP 2.2 **Level of Program Leaders**: High level of seniority of the director of the national family planning program and whether director reports to a high level of government.	FPEI	High level of seniority of the director of the national family planning program and whether director reports to ahigh level of government
*FP. 2.3 **Community-Based Distribution**: Extent to which areas of country not easily serviced by clinics or other service points are covered by community-based distribution programs for distribution of contraceptive supplies (especially rural areas)	FPEI	Extent to which areas of country not easily serviced by clinics or other service points are covered by community-based distribution programs for distribution of contraceptives (especially rural areas)

Note
- *DDEI items are not always sourced from the reference index verbatim.*
- **Items that were dropped because they loaded poorly on the domain factors.*

DDEI Questionnaire Item	Reference/ Source Index	Reference/ Source Index Item
*FP 2.4 **Postpartum Program**: Extent to which all postpartum women receive postpartum family planning services.	FPEI	Extent to which all new mothers in the country receive postpartum family planning assistance.
*FP 2.5 **Home-Visiting Workers**: Extent of coverage of population by workers whose primary task is to visit (rural) women in their homes to talk about family planning and maternal and child health.	FPEI	Extent of coverage of population by workers whose primary task is to visit (rural)women in their homes to talk about family planning and maternal and child health
FP 2.6.1 **Availability and Accessibility of FP Commodities/Supplies and Services Part 1**: Extent to which entire population has ready and easy access to: *IUD*	FPEI	Extent to which entire population has ready and easy access to IUDs *(Or: Pills, Injectables, voluntary Female Sterilization, voluntary male Sterilization, Condoms, safe Abortion or menstrual regulation (whether legal or not)*
FP 2.6.2 **Availability and Accessibility of FP Commodities/Supplies and Services Part 1**: Extent to which entire population has ready and easy access to: *Pills*	FPEI	Extent to which entire population has ready and easy access to IUDs *(Or: Pills, Injectables, voluntary Female Sterilization, voluntary male Sterilization, Condoms, safe Abortion or menstrual regulation (whether legal or not)*
FP 2.6.3 **Availability and Accessibility of FP Commodities/Supplies and Services Part 1**: Extent to which entire population has ready and easy access to: *Pills*	FPEI	Extent to which entire population has ready and easy access to IUDs *(Or: Pills, Injectables, voluntary Female Sterilization, voluntary male Sterilization, Condoms, safe Abortion or menstrual regulation (whether legal or not)*
FP 2.6.4 **Availability and Accessibility of FP Commodities/Supplies and Services Part 1**: Extent to which entire population has ready and easy access to: *Injectables*	FPEI	Extent to which entire population has ready and easy access to IUDs *(Or: Pills, Injectables, voluntary Female Sterilization, voluntary male Sterilization, Condoms,*

Note

- *DDEI items are not always sourced from the reference index verbatim.*
- **Items that were dropped because they loaded poorly on the domain factors.*

DDEI Questionnaire Item	Reference/ Source Index	Reference/ Source Index Item
		safe Abortion or menstrual regulation (whether legal or not)
*FP 2.6.5 **Availability and Accessibility of FP Commodities/Supplies and Services Part 1**: Extent to which entire population has ready and easy access to: *Female Sterilization*	FPEI	Extent to which entire population has ready and easy access to IUDs *(Or: Pills, Injectables, voluntary Female Sterilization, voluntary male Sterilization, Condoms, safe Abortion or menstrual regulation (whether legal or not)*
*FP 2.6.6 **Availability and Accessibility of FP Commodities/Supplies and Services Part 1**: Extent to which entire population has ready and easy access to: *Male Sterilization*	FPEI	Extent to which entire population has ready and easy access to IUDs *(Or: Pills, Injectables, voluntary Female Sterilization, voluntary male Sterilization, Condoms, safe Abortion or menstrual regulation (whether legal or not)*
*FP 2.6.7 **Availability and Accessibility of FP Commodities/Supplies and Services Part 1**: Extent to which entire population has ready and easy access to: Male *Condoms*	FPEI	Extent to which entire population has ready and easy access to IUDs *(Or: Pills, Injectables, voluntary Female Sterilization, voluntary male Sterilization, Condoms, safe Abortion or menstrual regulation (whether legal or not)*
FP 2.6.8 **Availability and Accessibility of FP Commodities/Supplies and Services Part 1**: Extent to which entire population has ready and easy access to: *Emergency Contraception*	FPEI	Extent to which entire population has ready and easy access to IUDs *(Or: Pills, Injectables, voluntary Female Sterilization, voluntary male Sterilization, Condoms, safe Abortion or menstrual regulation (whether legal or not)*
FP 2.6.9 **Availability and Accessibility of FP Commodities/Supplies and Services Part 1**: Extent to which entire population has ready and easy access to *Abortion/menstrual regulation (whether legal or not)*	FPEI	Extent to which entire population has ready and easy access to IUDs *(Or: Pills, Injectables, voluntary Female Sterilization, voluntary male Sterilization, Condoms, safe Abortion or menstrual regulation (whether legal or not)*

Note

- *DDEI items are not always sourced from the reference index verbatim.*
- **Items that were dropped because they loaded poorly on the domain factors.*

**DDEI Questionnaire Item**	**Reference/ Source Index**	**Reference/ Source Index Item**
FP 2.6.10 **Availability and Accessibility of FP Commodities/Supplies and Services Part 1**: Extent to which entire population has ready and easy access to: *Others*	FPEI	Extent to which entire population has ready and easy access to IUDs *(Or: Pills, Injectables, voluntary Female Sterilization, voluntary male Sterilization, Condoms, safe Abortion or menstrual regulation (whether legal or not)*
FP 2.7 **Availability and Accessibility of FP Commodities/Supplies and Services Part 2:** How well does the supply system operate (it avoids stockouts or interrupted supplies and guarantees a reliable flow at local levels) for the following: *IUD, Pills, Injectables, Condoms, Emergency Contraception*	FPEI	How well does the IUD *(Or: Pill, Injectable, Condom)* supply system operate (it avoids stockouts orinterrupted supplies and guarantees a reliable flow at local levels)
FP 2.8.1 **Availability and Accessibility of FP Commodities/Supplies and Services Part 3:** How well does the supply system provide necessary equipment and medical supplies at clinical facilities for the following: *Female Sterilization*	FPEI	How well does the supply system provide necessary equipment and medical supplies for female *(Or male)* sterilization services at clinical facilities?
FP 2.8.2 **Availability and Accessibility of FP Commodities/Supplies and Services Part 3:** How well does the supply system provide necessary equipment and medical supplies at clinical facilities for the following: *Male Sterilization*	FPEI	How well does the supply system provide necessary equipment and medical supplies for female *(Or male)* sterilization services at clinical facilities?
FP 2.8.3 **Availability and Accessibility of FP Commodities/Supplies and Services Part 3:** How well does the supply system provide necessary equipment and medical supplies at clinical facilities for the following: *IUDs*	FPEI	How well does the supply system provide necessary equipment and medical supplies for female *(Or male)* sterilization services at clinical facilities?
FP 2.8.4 **Availability and Accessibility of FP Commodities/Supplies and Services Part 3:** How well does the supply system provide necessary equipment and	FPEI	How well does the supply system provide necessary equipment and medical supplies for female *(Or male)* sterilization services at clinical facilities?

Note

- *DDEI items are not always sourced from the reference index verbatim.*
- **Items that were dropped because they loaded poorly on the domain factors.*

DDEI Questionnaire Item	Reference/ Source Index	Reference/ Source Index Item
medical supplies at clinical facilities for the following: *Implants*		
*FP 2.10 **Incentives and Disincentives**: Extent to which monetary or other incentives are used to encourage the adoption of family planning.	FPEI	Extent to which monetary or other incentives are used to encourage the adoption of family planning
FP 2.12 **Training Program**: Extent to which training programs, for each category of staff in the family planning program, are adequate to provide personnel with information and skills necessary to carry out their jobs effectively.	FPEI	Extent to which training programs, for each category of staff in the family planning program, are adequate to provide personnel with information and skills necessary to carry out their jobs effectively
FP 2.13 **Personnel Carry Out Assigned Tasks**: Extent to which all categories of family planning program staff (administrative, medical, paramedical, field) carry out assigned tasks effectively	FPEI	Extent to which all categories of family planning program staff (administrative, medical, paramedical, field) carry out assigned tasks effectively
FP 2.14 **Logistics and Transport**: Extent to which the logistics and transport systems are sufficient to keep stocks of contraceptive supplies and related equipment available at all service points, at all times and at all levels.	FPEI	Extent to which the logistics and transport systems are sufficient to keep stocks of contraceptive supplies and related equipment available at all service points, at all times and at all levels (central, provincial, local)
FP 2.15 **Supervision System**: Extent to which the system of supervision at all levels is adequate (regular monitoring visits with corrective or supportive action).	FPEI	Extent to which the system of supervision at all levels is adequate (regular monitoring visits with corrective or supportive action)
*FP 2.16 **Involvement of Other Ministries and Public Agencies**: Extent to which other ministries, government	FPEI	Extent to which other ministries and government agencies assist with family planning activities (e.g.,

Note
- *DDEI items are not always sourced from the reference index verbatim.*
- **Items that were dropped because they loaded poorly on the domain factors.*

DDEI Questionnaire Item	Reference/ Source Index	Reference/ Source Index Item
agencies and non-governmental stakeholders engage with FP activities (e.g., delivery of supplies, services, information, education) or other population activities.		delivery of supplies, services, information, education) or other population activities
FP 2.17 **Involvement of private-sector agencies and groups**: Extent to which private-sector agencies and groups assist with FP or other population activities.	FPEI	Extent to which private-sector agencies and groups assist with family planning or other population activities
*FP 2.18 **Mass Media for Information Education Communication**: Frequency and extent of coverage of mass media messages that provide population with information on family planning and service sites.	FPEI	Frequency and extent of coverage of mass media messages that provide population with information on family planning and service sites
*FP 2.21 **FP Effort Influences – Forces Affecting the FP Program**: Forces affecting the FP program can either strengthen or detract from its effectiveness. Check zero (0) if there is no difference either way; check a negative number from -1 to -5 if the influence is negative; or check a positive number from 1 to 5 if the influence is positive. (1 in either direction means little influence and 5 in either direction means strong influence.): a)*Decentralization, b)HIV/AIDS Programs, c)Incorporation of FP into a broader context of reproductive health,d) Integration of FP with other health services, e)Changes in donor funding, f)Changes in domestic government funding*	FPEI	Forces affecting the family planning program can either strengthen or detract from its effectiveness. Check zero (0) if there is no difference either way; check a negative number from-1 to -5 if the influence is negative; or check a positive number from 1 to 5 is the influence is positive. (1 in either direction means little influence and 5 in either direction means strong influence.): *Decentralization (the shift of decision making and resources from the central government to lower administrative levels)), HIV/AIDS Programs, Incorporation of FP into a broader context of reproductive health, Integration of FP with other health services, Changes in donor funding, Changes in domestic government funding*
Dimension 3. Advocacy		

Note

- *DDEI items are not always sourced from the reference index verbatim.*
- **Items that were dropped because they loaded poorly on the domain factors.*

**DDEI Questionnaire Item**	**Reference/ Source Index**	**Reference/ Source Index Item**
FP 3.4 **FP Effort Influences - Justification**: How important is each of the following as a current government justification for the national FP program? (1 means negligible importance; 5 means great importance.): *Reduce rate of population growth, Enhance economic development, Help women and men avoid unplanned and unwanted births, Improve women's health, Improve child health, Reduce unmarried adolescent childbearing, Reduce unmet need for contraceptive services*	FPEI	How important is each of the following as a current justification for the national family planning program? (1 means negligible importance; 10 means great importance.): *Reduce rate of population growth, Enhance economic development, Help women and men avoid unplanned and unwanted births, Improve women's health, Improve child health, Reduce unmarried adolescent childbearing, Reduce unmet need for contraceptive services*
FP 3.5 **FP Effort Influences – Special Populations**: To what extent does the family planning program give particular emphasis to special populations? (1 means negligible emphasis; 5 means great emphasis): *Unmarried youth, The poor, The disabled, Rural population, Other vulnerable groups (e.g. minotirty and/or indigenous groups), Postpartum women for counseling and contraceptive services, Post-abortion women for counseling and contraceptive services*	FPEI	To what extent does the family planning program give particular emphasis to special populations? (1means negligible emphasis; 10 means great emphasis: *Unmarried youth, The poor, Rural population, Postpartum women for counseling and contraceptive services, Post-abortion women for counseling and contraceptive services*
FP 3.6 **Social Marketing:** Extent of coverage of the country by a social marketing program (subsidized contraceptive sales at low cost in commercial sector, especially in urban areas).	FPEI	Extent of coverage of the country by a social marketing program (subsidized contraceptive sales at low cost in commercial sector, especially in urban areas)
Dimension 4. Research		
FP 4.6 **Record-Keeping**: Extent to which systems for client recordkeeping, clinic reporting and feedback of results are adequate, including being connected at all levels (federal, state/province, county, etc.).	FPEI	Extent to which systems forclient recordkeeping, clinicreporting and feedback ofresults are adequate

Note

- *DDEI items are not always sourced from the reference index verbatim.*
- **Items that were dropped because they loaded poorly on the domain factors.*

DDEI Questionnaire Item	Reference/ Source Index	Reference/ Source Index Item
FP 4.8 **Evaluation**: Extent to which program statistics, surveys, and small studies are used by specialized staff to report on program operations and measure progress.	FPEI	Extent to which programstatistics, national surveys, andsmall studies are used byspecialized staff to report onprogram operations andmeasure progress
FP 4.9 **Management's Use of Evaluation Findings**: Extent to which local-level program managers use research and evaluation findings to improve programming in ways suggested by findings.	FPEI	Extent to which program managers use research and evaluation findings to improve the program in ways suggested by findings

Source:
The Family Planning Effort Index: 1999, 2004, and 2009. (USAID 2010).
http://www.healthpolicyplus.com/archive/ns/pubs/hpi/Documents/1110_1_FP_Effort_Index_1999_2004_2009__FINAL_05_08_10_acc.pdf

Note
- *DDEI items are not always sourced from the reference index verbatim.*
- **Items that were dropped because they loaded poorly on the domain factors.*

Maternal and Child Health (MCH) DDEI Questionnaire

DDEI Questionnaire Item	Reference/ Source Index	Reference/ Source Index Item
Dimension 1. Policy/Policymaking		
MCH 1.1 **Adequate Policy**: Extent to which the Ministry of Health's policies toward pregnancy and delivery services account for the breadth of requirements to ensure that every mother and newborn can survive.	Maternal neonatal program index (MNPI)	Ministry of Health policies toward pregnancy and delivery services are adequate. *(Present effort and Effort 3 years ago)*
MCH 1.3 **Multisector Involvement**: Extent to which policies are developed through adequate consultation with interested parties such as other ministries, NGOs, private practitioners, and women's groups.	MNPI	Policies are developed through adequate consultation with interested parties such as otherministries, NGOs, private practitioners, women's groups. *(Present effort and Effort 3 years ago)*
*MCH 1.4 **Competent Service Providers**: Extent to which policies are reasonable and evidence-based concerning which personnel can provide maternal health services (e.g. trained midwives can perform a wide range of medical procedures).	MNPI	Policies are reasonable and fair concerning which personnel can provide maternal health services (e.g. trained midwives can perform a wide range of medical procedures)
MCH 1.6. **Post-Abortion Care Legislation**: Extent to which a favorable policy exists toward the treatment of complications of abortions, including complications seen from illegal abortions.	MNPI	A favorable policy exists toward the treatment of complications of abortions, including complications seen from illegal abortions
MCH 1.7 **Policy Reviews and Updates**: Extent to which policies are regularly reviewed by high-level policymakers and reviews are used to update action plans.	MNPI	Policies are vigorously implemented through regular high-level reviews and updated action plans

Note

- *DDEI items are not always sourced from the reference index verbatim.*
- **Items that were dropped because they loaded poorly on the domain factors.*

DDEI Questionnaire Item	Reference/ Source Index	Reference/ Source Index Item
MCH 1.8 **High-Level Actor Placement**: Extent and level to which the director of maternal and child health services is involved in high-level decision making.	MNPI	The director of services for maternal health is placed at a high administrative level
MCH 1.9 **Financing for MCH**: Extent to which the government budget for safe pregnancy, delivery, perinatal care (for facilities, personnel, supplies, etc.) and children's health is adequate for the needs, whether from the Ministry of Health, provincial/ county government or donor support.	MNPI	The government budget for safe pregnancy, delivery, and postpartum care (for facilities, personnel, supplies, etc.) is adequate for the needs, whether from the Ministry of Health, provincial government or donor support
MCH 1.10 **Affordability**: Extent to which all MCH-related services and drugs are affordable to all clients.	MNPI	All services and drugs are provided free to all clients
*MCH 1.11 **Private Sector**: Extent to which the private sector (doctors, midwives, clinics) is active and covers a substantial share of pregnancy and delivery cases.	MNPI	he private sector (doctors, midwives, clinics, maternity homes) is active and covers a substantial share of pregnancy and delivery cases.
Dimension 2. Services or Programs		
MCH 2.1 **Obstetric Care Availability – All Centers**: Extent to which all primary health facilities have trained staff, in place, who can provide obstetric care to:<br>MCH 2.1.1 Properly manage postpartum hemorrhage cases.<br>MCH 2.1.2 Timely and properly administer antibiotics intravenously when needed.<br>MCH 2.1.3 Perform manual removal of retained placenta.	MNPI	All health centers have trained staff, in place, who can provide obstetric care:<br>Properly manage postpartum hemorrhage cases.<br>Timely and properly administer antibiotics intravenously when needed.<br>Perform manual removal of retained placenta.<br>Perform vacuum aspiration of the uterus, using MVA (manual vacuum aspiration) or an electric suction device.

Note

- *DDEI items are not always sourced from the reference index verbatim.*
- **Items that were dropped because they loaded poorly on the domain factors.*

**DDEI Questionnaire Item**	**Reference/ Source Index**	**Reference/ Source Index Item**
MCH 2.1.4 Perform vacuum aspiration of the uterus, using MVA (manual vacuum aspiration) or an electric suction device.<br>MCH 2.1.5 Use a partograph to determine when to refer.<br>MCH 2.1.6 Have transportation arrangements to quickly move a woman with obstructed labor to a district/sub-county hospital.<br>MCH 2.1.7 Have adequate antibiotic supplies on hand (sufficient supplies of the correct types).		Use a partograph to determine when to refer.<br>Have transportation arrangements to quickly move a woman with obstructed labor to a district/sub-county hospital.<br>Have adequate antibiotic supplies on hand (sufficient supplies of the correct types).
MCH 2.2 **Obstetric Care Availability – Secondary healthcare facilities:** Extent to which all district/sub-county hospitals have trained staff, in place, who can:<br>MCH 2.2.1 Provide all functions listed above for primary health facilities.<br>MCH 2.2.2 Perform blood transfusions (and have adequate supplies of safe blood on hand).<br>MCH 2.2.3 Perform Cesarean section.	MNPI	All First Referral Facilities-District Hospitals-have trained staff, in place, who can:<br>Provide all functions listed above for primary health facilities.<br>Perform blood transfusions (and have adequate supplies of safe blood on hand).<br>Perform Cesarean section.
MCH 2.3 **Pregnancy-Related Services**: Extent to which all pregnant women have adequate access to:<br>MCH 2.3.1 Treatment for postpartum hemorrhage during or soon after delivery.<br>MCH 2.3.2 Management of obstructed labor.<br>MCH 2.3.3 Management of pre-eclampsia, eclampsia and its complications.<br>MCH 2.3.4 Treatment of post abortion care or for abortion complications.<br>MCH 2.3.5 Provision of safe abortion services.<br>MCH 2.3.6 Antenatal care during pregnancy.	MNPI	Access to Services by Pregnant Women – Many women do not have access to a trained professional attendant, a health center with beds, or a district health center—and even if they do, the nearest attendant or facility may not have staff or equipment, or the service may be too expensive. So this section pertains to the percentage of pregnant women with adequate access to each service. (Enter percentage in each cell.)<br>What percentage of pregnant women have adequate access to: *(Separated by Rural and Urban women):*

Note

- *DDEI items are not always sourced from the reference index verbatim.*
- **Items that were dropped because they loaded poorly on the domain factors.*

DDEI Questionnaire Item	Reference/ Source Index	Reference/ Source Index Item
MCH 2.3.7 Delivery care by a trained professional attendant.<br>MCH 2.3.8 Postpartum family planning services.<br>MCH 2.3.9 District/sub-county hospitals that are open 24 hours/day.		Treatment for postpartum hemorrhage during or soon after delivery.<br>Management of obstructed labor.<br>Management of pre-eclampsia, eclampsia and its complications.<br>Treatment of post abortion care or for abortion complications.<br>Provision of safe abortion services.<br>Antenatal care during pregnancy.<br>Delivery care by a trained professional attendant.<br>Postpartum family planning services.<br>District/sub-county hospitals that are open 24 hours/day.
MCH 2.5 **Newborn Care**: Extent to which, for newborn care, all infants whether delivered at home or in a facility:<br>MCH 2.5.1 Have their mouth and nasal passageways cleared<br>MCH 2.5.2 Are dried and kept warm immediately after birth<br>*MCH 2.5.3 Receive vitamin A<br>MCH 2.5.4 Have their umbilical cord cut with a clean blade<br>MCH 2.5.5 Complete WHO recommended vaccination calendar<br>MCH 2.5.6 Benefit from exclusive breastfeeding promotion and education activities<br>MCH 2.5.7 Benefit from nutritional support programs when in need.	MNPI	Delivery and antenatal care – For newborn care, all infants whether delivered at home or in a facility:<br>Have their mouth and nasal passageways cleared:<br>Have their mouth and nasal passageways cleared<br>Are dried and kept warm immediately after birth<br>Receive prophylactic treatment for their eyes<br>Have their umbilical cord cut with a clean blade<br>Receive a DPT injection at 3 months<br>Are scheduled for subsequent immunizations

Note

- *DDEI items are not always sourced from the reference index verbatim.*
- **Items that were dropped because they loaded poorly on the domain factors.*

DDEI Questionnaire Item	Reference/ Source Index	Reference/ Source Index Item
MCH 2.6 **Family Planning – All Centers**: Extent to which there exists proper provision of family planning at all health centers that:<br>MCH 2.6.1 Routinely offer family planning services for post-abortion cases<br>MCH 2.6.2 Routinely offer family planning at postpartum visits<br>MCH 2.6.3 Have short-term and LARC contraceptive supplies regularly in stock<br>MCH 2.6.4 Have trained staff, in place, who can provide services for LARCs.<br>MCH 2.6.5 Have guidelines and protocols necessary for the provision of family planning services.	MNPI	Provision of Family Planning – All health centers:<br>Routinely offer family planning services for post-abortion cases<br>Routinely offer family planning at postpartum visits<br>Have short-term and LARC contraceptive supplies regularly in stock<br>Have trained staff, in place, who can provide services for LARCs.<br>Have guidelines and protocols necessary for the provision of family planning services.
MCH 2.7 **Family Planning – District/sub-county Hospitals:** Extent to which there exists proper provision of family planning at district/sub-county hospitals that:<br>MCH 2.7.1 Routinely offer family planning services for post-abortion cases<br>MCH 2.7.2 Routinely offer family planning at postpartum visits<br>MCH 2.7.3 Have short term and LARC contraceptive supplies regularly in stock<br>MCH 2.7.4 Have trained staff, in place, who can provide services for LARCs<br>*MCH 2.7.5 Can offer sterilization to female clients<br>*MCH 2.7.6 Can offer sterilization to male clients<br>MCH 2.7.7 Have guidelines and protocols necessary for the provision of family planning services.	MNPI	Provision of Family Planning – All first referral facilities-district hospitals:<br>Routinely offer family planning services for post-abortion cases<br>Routinely offer family planning at postpartum visits<br>Have short term and LARC contraceptive supplies regularly in stock<br>Have trained staff, in place, who can provide services for LARCs<br>Can offer sterilization to female clients<br>Can offer sterilization to male clients

Note

- *DDEI items are not always sourced from the reference index verbatim.*
- **Items that were dropped because they loaded poorly on the domain factors.*

DDEI Questionnaire Item	Reference/ Source Index	Reference/ Source Index Item
Dimension 3. Advocacy		
MCH 3.1 **Government Messages**: Extent to which high officials in the government, including the Ministry of Health, issue frequent statements to all stakeholders including the press to support improvements for safe pregnancy, safe delivery and healthy childhood.	MNPI	High officials in the government, including the Ministry of Health, issue frequent statements to the press and public to support improvements for safe pregnancy and delivery
MCH 3.2 **Mass Media**: Extent to which the national program uses the mass media to educate the public about symptoms of pregnancy complications, safe childbirth and healthy childhood.	MNPI	The national program uses the mass media to educate the public about symptoms of pregnancy complications and safe places for childbirth
MCH 3.3.1 **Community-Level Media**: Extent to which community groups take part in systematic programs to educate the public about safe pregnancy	MNPI	Community-level organizations take part in systematic programs to educate the public about safe pregnancy and delivery
MCH 3.3.2 **Community-Level Media**: Extent to which community groups take part in systematic programs to educate the public about safe delivery	MNPI	Community-level organizations take part in systematic programs to educate the public about safe pregnancy and delivery
MCH 3.3.3 **Community-Level Media**: Extent to which community groups take part in systematic programs to educate the public about healthy childhood.	MNPI	Community-level organizations take part in systematic programs to educate the public about safe pregnancy and delivery
MCH 3.4 **Educational Materials**: Extent to which the appropriate ministry (MOH) supplies adequate educational materials (posters, pamphlets, etc.) to delivery facilities to instruct clients about safe practices.	MNPI	he Ministry of Health supplies adequate educational materials (posters, pamphlets, etc.) to delivery facilities to instruct clients about safe practices

Source

Rating Maternal and Neonatal Health Programs in Developing Countries. (Bulatao & Ross). (Measure Evaluation – USAID). 2000. Appendix A Questionnaire Items and Variable Codes for the Maternal and Neonatal Program Effort Index (MNPI)
https://pdf.usaid.gov/pdf_docs/pnacl165.pdf

Note

- *DDEI items are not always sourced from the reference index verbatim.*
- **Items that were dropped because they loaded poorly on the domain factors.*

Education (ED) DEI Questionnaire

DDEI Questionnaire Item	Reference/ Source Index	Reference/ Source Index Item
Dimension 1. Policy/Policymaking		
ED 1.1 **Quality**: Extent to which there are national plans/strategies/programs to improve the quality of learning in schools.	UNESCO. EFA Global Monitoring Report. 2013-14. *The Education for All Development Index.*	Extent of existing strategies to improve quality learning in schools?
ED 1.2 **Disadvantaged Groups**:<br>ED 1.2.1 Extent to which strategies have been developed and implemented/deployed to overcome learning obstacles for pregnant girls and adolescent mothers.<br>ED 1.2.2 Extent to which strategies have been developed and implemented/deployed to overcome learning obstacles for disabled populations.	UNESCO. EFA Global Monitoring Report. 2013-14.	Strategies to overcome learning obstacles for disadvantaged groups.
ED 1.3 **Teachers**: Extent to which teacher recruitment, development and management issues are included in policy aimed at improving learning outcomes.	UNESCO. EFA Global Monitoring Report. 2013-14.	Inclusion of teacher recruitment, development and management issues in policy aimed at improving learning outcomes? If so, what linkages are identified?
ED 1.4 **Teachers' Accountability**: Extent to which national plans include specific measures, including monitoring and evaluation, that aim to hold teachers' accountable for improving learning.	UNESCO. EFA Global Monitoring Report. 2013-14.	Inclusion of specific measures that aim to hold teachers' accountable for improving learning.
ED 1.5 **Teacher Quality:** Extent to which education policies/strategies/programs include verifiable indicators for teacher quality.	UNESCO. EFA Global Monitoring Report. 2013-14.	Inclusion of verifiable indicators for improving learning and for teacher qualityin education policies?

Note

- *DDEI items are not always sourced from the reference index verbatim.*
- **Items that were dropped because they loaded poorly on the domain factors.*

DDEI Questionnaire Item	Reference/ Source Index	Reference/ Source Index Item
ED 1.7 **Gender Parity SDG**: Extent to which national policies/plans/strategies consider the (SDG) goal for complete gender parity at all levels of education.	UNESCO. EFA Global Monitoring Report. 2005. *The Education for All Development Index.*	The EDI constituents and related indicators includes:<br>a. gender: gender-specific EFA index (GEI, thearithmetical mean of the GPIs for the primaryand secondary gross enrolment ratios and theadult literacy rate)
ED 1.8 **Education Financing**: Extent to which new education plans include financing proposals with budgets allocated for learning and teaching reforms.	UNESCO. EFA Global Monitoring Report. 2013-14.	Extent to which new education plans include financing proposals with budgetsallocated for learning and teaching reforms
ED 1.10 **Adult Illiteracy**: Extent to which national policies/plans/strategies aim to address illiteracy.	UNESCO. EFA Global Monitoring Report. 2005. *The Education for All Development Index.*	The EDI constituents and related indicators includes:<br>b. adult literacy: literacy rate of the group aged 15and over;
Dimension 2. Services or Programs		
ED 2.1 **Teacher Deployment**: Extent to which strategies/plans are used to ensure teachers are deployed to areas of the country where they are most needed.	UNESCO. EFA Global Monitoring Report. 2013-14.	Strategies to ensure teachers are deployed to areas of countries where they aremost needed?
ED 2.2 **Teacher Quality**: Extent to which teacher quality is recognized as a key factor in improving learning outcomes.<br>ED 2.2.1 Extent to which the quality of teacher education is emphasized in wider education policies/plans/strategies.<br>ED 2.2.2 Extent to which the quality of training and continuous professional development is emphasized in wider education policies/plans/strategies.	UNESCO. EFA Global Monitoring Report. 2013-14.	How is teacher quality recognized as a key factor in improving learning outcomes?<br><br>Extent of specific strategies to recognize teacher quality

Note

- *DDEI items are not always sourced from the reference index verbatim.*
- **Items that were dropped because they loaded poorly on the domain factors.*

**DDEI Questionnaire Item**	**Reference/ Source Index**	**Reference/ Source Index Item**
ED 2.3 **Gender Parity**: Extent to which resources have been utilized to improve equality in education between boys and girls.	UNESCO. EFA Global Monitoring Report. 2005. *The Education for All Development Index.*	The EDI constituents and related indicators includes: gender: gender-specific EFA index (GEI, thearithmetical mean of the GPIs for the primaryand secondary gross enrolment ratios and theadult literacy rate)

Sources

1. UNESCO. Teaching and learning: achieving quality for all; EFA Global Monitoring Report. 2013-2014. *Part 1: Monitoring Progress Toward the EFA Goals.* https://unesdoc.unesco.org/ark:/48223/pf0000225660

2. UNESCO. Education for all: the quality imperative; EFA Global Monitoring Report. *Chapter 3: Assessing Progress Toward the EFA Goals – The Education for All Development Index.* 2005. https://unesdoc.unesco.org/ark:/48223/pf0000137333

3. SYSTEM APPROACH FOR BETTER EDUCATION RESULTS (SABER) Data Collection Instrument for SABER - School Autonomy and Accountability 2.0: http://wbgfiles.worldbank.org/documents/hdn/ed/saber/supporting_doc/Background/SAA/SABER_SAA_%20Data_Collection_Instrument.pdf

4. What Matters Most for School Autonomy and Accountability: A Framework Paper18: https://documents1.worldbank.org/curated/en/385451468172788612/pdf/What-matters-most-for-school-autonomy-and-accountability-a-framework-paper.pdf

Note

- *DDEI items are not always sourced from the reference index verbatim.*
- **Items that were dropped because they loaded poorly on the domain factors.*

Women Empowerement (WE) DDEI Questionnaire

There was not a specific effort index that was integrated with the Women's Empowerment DDEI Questionnaire. Rather, an existing index was relied on heavily to help the authors draft the DDEI items specifically in reference to levels of effort. This was the African Gender Development Index (AGDI) of 2011: https://archive.uneca.org/sites/default/files/PublicationFiles/agdi_2011_eng_fin.pdf

Labor Market (LM) DDEI Questionnaire

There was not a specific effort index that was integrated with the Labor Market DDEI Questionnaire. Rather, a handful of existing frameworks were relied on heavily to help the authors draft the DDEI items specifically in reference to levels of effort. These were:

1. IMF. The Future of Work in Sub-Saharan Africa. 2018. https://www.imf.org/en/Publications/Departmental-Papers-Policy-Papers/Issues/2018/12/14/The-Future-of-Work-in-Sub-Saharan-Africa-46333
2. IMF. Designing Labor Market Institutions in Emerging Market and Developing Economies: Evidence and Policy Options. 2019. https://www.imf.org/en/Publications/Staff-Discussion-Notes/Issues/2019/05/15/Designing-Labor-Market-Institutions-in-Emerging-and-Developing-Economies-Evidence-and-Policy-46855
3. The African Union Commission & UNFPA. AU Roadmap on Harnessing the Demographic Dividend Through Investments In Youth. 2017. https://wcaro.unfpa.org/sites/default/files/pub-pdf/AU 2017 DD ROADMAP Final - EN.pdf

Good governance and economic institutions (GGEI) DDEI Questionnaire

There was not a specific effort index that was integrated with the Women's Empowerment DDEI Questionnaire. Rather, an existing index was relied on heavily to help the authors draft the DDEI items specifically in reference to levels of effort. This was the AU Roadmap on Harnessing the Demographic Dividend Through Investments In Youth (2017): https://wcaro.unfpa.org/sites/default/files/pub-pdf/AU%202017%20DD%20ROADMAP%20Final%20-%20EN.pdf. We also examined the World Bank Worldwide Governance Indicators: https://info.worldbank.org/governance/wgi/Home/Documents.

General note:

Across all DDEI questionnaires the domain on Civil Society pulls largely from:

1. USAID. Civil Society Involvement in Family Planning A Review of Global Programming and Evidence. 2017. https://evidenceproject.popcouncil.org/wp-content/uploads/2017/12/India-CSO_Research-Report.pdf; and
2. WHO. Strategies, tactics and approaches: Conducting and evaluating national civil society advocacy for reproductive, maternal and child health. 2014. https://apps.who.int/iris/bitstream/handle/10665/100626/9789241506687_eng.pdf;jsessionid=514C2178A6164B5E84306E330B300E64?sequence=1

Note

- *DDEI items are not always sourced from the reference index verbatim.*
- **Items that were dropped because they loaded poorly on the domain factors.*
