# Supplementary material for: Development of the Demographic Dividend Effort Index, a novel tool to measure existing efforts to create a favourable environment to harness a demographic dividend: results from an experts’ survey from six sub-Saharan African countries
Source: BMJ Open. 2023 Mar 21;13(3):e059937. doi: 10.1136/bmjopen-2021-059937 (PMC10040031; doi:10.1136/bmjopen-2021-059937)
Supplement: Supplementary data [file bmjopen-2021-059937supp004.pdf]

Appendix 3. Results from factor analysis

Sector - Family planning

**Policy**		**Service and Programs**		**Advocacy**		**Research**		**Civil society**	
**Obs.**	**75**	**Obs.**	**53**	**Obs.**	**73**	**Obs.**	**75**	**Obs.**	**80**
FP1_1	0.54	FP2_1	**0.47**	FP3_1	**0.45**	FP4_1a	0.66	FP5_1	0.73
FP1_2	**0.41**	FP2_2	**0.39**	FP3_2	**0.31**	FP4_1b	0.70	FP5_2	0.70
FP1_3	0.62	FP2_3	**0.46**	FP3_3	**0.38**	FP4_1c	0.70	FP5_3	0.88
FP1_4	**0.49**	FP2_4	**0.50**	FP3_4a	0.50	FP4_2	0.71	FP5_4	0.88
FP1_5	0.74	FP2_5	**0.25**	FP3_4b	0.59	FP4_3a	0.64	FP5_5	0.87
FP1_6	0.72	FP2_6a	0.74	FP3_4c	0.78	FP4_3b	0.73	FP5_6	0.75
FP1_7	0.61	FP2_6b	0.65	FP3_4d	0.78	FP4_3c	0.60	FP5_7	0.80
FP1_8	0.54	FP2_6c	0.62	FP3_4e	0.81	FP4_4a	0.77	FP5_8	0.88
FP1_9	**0.45**	FP2_6d	0.75	FP3_4f	0.75	FP4_4b	0.74	FP5_9	0.88
FP1_10	0.67	FP2_6e	**0.44**	FP3_4g	0.81	FP4_4c	0.77	FP5_10	0.84
FP1_11a	0.59	FP2_6f	**0.45**	FP3_5a	0.73	FP4_4d	0.67	FP5_11	0.78
FP1_11b	**0.48**	FP2_6g	0.50	FP3_5b	0.77	FP4_5a	0.69	FP5_12	0.87
FP1_12	**0.39**	FP2_6h	0.57	FP3_5c	0.79	FP4_5b	0.78	FP5_13	0.89
FP1_13	0.52	FP2_6i	**0.38**	FP3_5d	0.72	FP4_5c	0.76	FP5_14	0.87
FP1_14	0.66	FP2_6j	0.63	FP3_5e	0.77	FP4_5d	0.79		
		FP2_7a	0.75	FP3_5f	0.70	FP4_6	0.66		
		FP2_7b	0.61	FP3_5g	0.71	FP4_7	0.69		
		FP2_7c	0.66	FP3_6	0.54	FP4_8	0.74		
		FP2_7d	0.75			FP4_9	0.68		
		FP2_7e	0.58			FP4_10	0.72		
		FP2_7f	0.53			FP4_11	0.68		
		FP2_7g	0.57						
		FP2_8a	0.52						
		FP2_8b	**0.38**						
		FP2_8c	0.67						
		FP2_8d	0.68						
		FP2_9	0.62						
		FP2_10	**0.16**						
		FP2_11	0.51						
		FP2_12	0.63						
		FP2_13	0.78						
		FP2_14	0.72						
		FP2_15	0.57						
		FP2_16	**0.43**						
		FP2_17	0.54						
		FP2_18	**0.36**						
		FP2_19	**0.45**						
		FP2_20	0.54						
		FP2_21a	**0.20**						
		FP2_21b	**0.25**						
		FP2_21c	**0.11**						
		FP2_21d	**0.06**						
		FP2_21e	**0.29**						
		FP2_21f	**0.09**						

Note

Bolded and shaded are the loadings of items that were dropped because they loaded poorly (<0.5) on domain factors.

Sector - Maternal and Child Health

Policy		Service and Programs		Advocacy		Research		Civil society	
**Obs.**	**60**	**Obs.**	**40**	**Obs.**	**67**	**Obs.**	**49**	**Obs.**	**52**
MCH1_1a	0.58	MCH2_1a	0.80	MCH3_1	0.78	MCH4_1a	0.80	MCH5_1	0.87
MCH1_1b	0.69	MCH2_1b	0.80	MCH3_2	0.89	MCH4_1b	0.84	MCH5_2	0.87
MCH1_2	0.67	MCH2_1c	0.73	MCH3_3	0.86	MCH4_1c	0.82	MCH5_3	0.91
MCH1_3	0.65	MCH2_1d	0.67	MCH3_4	0.85	MCH4_2	0.87	MCH5_4	0.91
MCH1_4	**0.40**	MCH2_1e	0.83	MCH3_5	0.75	MCH4_3a	0.85	MCH5_5	0.86
MCH1_5	**0.49**	MCH2_1f	0.78	MCH3_6	0.80	MCH4_3b	0.89	MCH5_6	0.88
MCH1_6	0.66	MCH2_1g	0.71			MCH4_3c	0.74	MCH5_7	0.84
MCH1_7	0.81	MCH2_2a	0.86			MCH4_4a	0.88	MCH5_8	0.91
MCH1_8	0.65	MCH2_2b	0.74			MCH4_4b	0.74	MCH5_9	0.88
MCH1_9	0.82	MCH2_2c	0.73			MCH4_4c	0.84	MCH5_10	0.91
MCH1_10	0.68	MCH2_3a	0.87			MCH4_4d	0.86	MCH5_11	0.91
MCH1_11	**0.24**	MCH2_3b	0.82			MCH4_5a	0.88	MCH5_12	0.90
MCH1_12	0.58	MCH2_3c	0.84			MCH4_5b	0.91		
MCH1_13a	0.79	MCH2_3d	0.91			MCH4_5c	0.94		
MCH1_13b	0.81	MCH2_3e	**0.44**			MCH4_5d	0.91		
MCH1_13c	0.82	MCH2_3f	0.62			MCH4_5e	0.92		
MCH1_13d	0.82	MCH2_3g	0.84			MCH4_6	0.77		
MCH1_13e	0.73	MCH2_3h	0.78			MCH4_7	0.74		
		MCH2_3i	0.79			MCH4_8	0.93		
		MCH2_4a	0.84			MCH4_9	0.87		
		MCH2_4b	0.85			MCH4_10	0.89		
		MCH2_4c	0.88			MCH4_11	0.87		
		MCH2_4d	0.87						
		MCH2_5a	0.84						
		MCH2_5b	0.80						
		MCH2_5c	**0.22**						
		MCH2_5d	0.81						
		MCH2_5e	0.79						
		MCH2_5f	0.87						
		MCH2_5g	0.72						
		MCH2_6a	0.81						
		MCH2_6b	0.87						
		MCH2_6c	0.80						
		MCH2_6d	0.77						
		MCH2_6e	0.84						
		MCH2_7a	0.80						
		MCH2_7b	0.87						
		MCH2_7c	0.67						
		MCH2_7d	0.65						
		MCH2_7e	**0.35**						
		MCH2_7f	**0.26**						
		MCH2_7g	0.66						
		MCH2_8a	0.78						
		MCH2_8b	0.86						

Note

Bolded and shaded are the loadings of items that were dropped because they loaded poorly (<0.5) on domain factors.

Sector – Education

**Policy**		**Service and Programs**		**Advocacy**		**Research**		**Civil society**	
**Obs.**	**41**	**Obs.**	**52**	**Obs.**	**53**	**Obs.**	**35**	**Obs.**	**44**
ED1_1	0.74	ED2_1	0.78	ED3_1	0.76	ED4_1a	0.77	ED5_1	0.92
ED1_2a	0.79	ED2_2a	0.79	ED3_2	0.80	ED4_1b	0.88	ED5_2	0.92
ED1_2b	0.76	ED2_2b	0.85	ED3_3	0.87	ED4_1c	0.85	ED5_3	0.94
ED1_3	0.77	ED2_3	0.84	ED3_4	0.88	ED4_2	0.86	ED5_4	0.93
ED1_4	0.77	ED2_4	0.88			ED4_3a	0.76	ED5_5	0.94
ED1_5	0.87	ED2_5	0.70			ED4_3b	0.74	ED5_6	0.85
ED1_6	0.77	ED2_6	0.74			ED4_3c	0.79	ED5_7	0.91
ED1_7	0.78					ED4_4a	0.82	ED5_8	0.92
ED1_8	0.73					ED4_4b	0.80	ED5_9	0.93
ED1_9a	0.72					ED4_4c	0.77	ED5_10	0.94
ED1_9b	0.82					ED4_4d	0.74	ED5_11	0.94
ED1_9c	0.85					ED4_5a	0.84		
ED1_10a	0.78					ED4_5b	0.88		
ED1_10b	0.76					ED4_5c	0.92		
ED1_11	0.81					ED4_5d	0.92		
ED1_12	0.71					ED4_6	0.87		
ED1_13	0.74					ED4_7	0.85		
						ED4_8	0.89		
						ED4_9	0.87		
						ED4_10	0.90		
						ED4_11a	0.93		
						ED4_11b	0.91		

Note
Bolded and shaded are the loadings of items that were dropped because they loaded poorly (<0.5) on domain factors.

Sector - Women Empowerment

**Policy**		**Service and Programs**		**Advocacy**		**Research**		**Civil society**	
Obs.	**55**	Obs.	**62**	Obs.	**66**	Obs.	**42**	Obs.	**60**
WE1_1	0.69	WE2_1	0.90	WE3_1	0.88	WE4_1a	0.81	WE5_1	0.84
WE1_2	0.79	WE2_2	0.90	WE3_2	0.87	WE4_1b	0.86	WE5_2	0.90
WE1_3	0.77	WE2_3	0.73	WE3_3	0.85	WE4_1c	0.84	WE5_3	0.89
WE1_4	0.84	WE2_4	0.90	WE3_4	0.86	WE4_2	0.91	WE5_4	0.85
WE1_5	0.88	WE2_5	0.86	WE3_5	0.76	WE4_3a	0.85	WE5_5	0.91
WE1_6	0.75	WE2_6	0.80	WE3_6	0.86	WE4_3b	0.82	WE5_6	0.89
WE1_7	0.74	WE2_7	0.76	WE3_7	0.85	WE4_3c	0.73	WE5_7	0.83
WE1_8a	0.72	WE2_8	0.77			WE4_4a	0.86	WE5_8	0.92
WE1_8b	0.87	WE2_9	0.89			WE4_4b	0.95	WE5_9	0.87
WE1_9	0.91	WE2_10	0.74			WE4_4c	0.95	WE5_10	0.90
WE1_10	0.81	WE2_11	0.87			WE4_4d	0.93		
WE1_11	0.76	WE2_12	0.68			WE4_5a	0.88		
WE1_12	0.83					WE4_5b	0.93		
WE1_13a	0.75					WE4_5c	0.92		
WE1_13b	0.91					WE4_6	0.95		
WE1_14	0.88					WE4_7	0.94		
WE1_15	0.92					WE4_8	0.96		
WE1_16	0.84					WE4_9	0.90		
WE1_17a	0.87					WE4_10	0.83		
WE1_17b	0.91								
WE1_17c	0.86								
WE1_17d	0.93								
WE1_18	0.89								

Note

Bolded and shaded are the loadings of items that were dropped because they loaded poorly (<0.5) on domain factors.

Sector - Labor market

Policy		Service and Programs		Advocacy		Research		Civil society	
**Obs.**	**51**	**Obs.**	**51**	**Obs.**	**54**	**Obs.**	**46**	**Obs.**	**47**
LM1_1a	**0.47**	LM2_1	0.75	LM3_1	0.56	LM4_1a	0.79	LM5_1	0.84
LM1_1b	**0.36**	LM2_2	0.87	LM3_2	0.81	LM4_1b	0.78	LM5_2	0.92
LM1_2	**0.33**	LM2_3	0.73	LM3_3	0.61	LM4_1c	0.88	LM5_3	0.91
LM1_3	0.71	LM2_4	0.88	LM3_4	0.84	LM4_2	0.80	LM5_4	0.92
LM1_4a	0.58	LM2_5	0.88	LM3_5	0.88	LM4_3a	0.75	LM5_5	0.87
LM1_4b	0.62	LM2_6	0.88			LM4_3b	0.76	LM5_6	0.92
LM1_5	0.82	LM2_7	0.86			LM4_3c	0.79	LM5_7	0.87
LM1_6	0.82	LM2_8	0.78			LM4_4a	0.74	LM5_8	0.89
LM1_7	0.88	LM2_9	0.64			LM4_4b	0.79	LM5_9	0.87
LM1_8	0.86	LM2_10	0.73			LM4_4c	0.71	LM5_10	0.84
LM1_9	0.84	LM2_11	0.63			LM4_4d	0.77		
LM1_10	0.81	LM2_12a	0.58			LM4_5a	0.84		
LM1_11	0.82	LM2_12b	0.67			LM4_5b	0.84		
LM1_12	0.87	LM2_13	0.66			LM4_5c	0.78		
LM1_13	0.70	LM2_14	0.80			LM4_6	0.88		
LM1_14	0.76	LM2_15	0.82			LM4_7	0.78		
LM1_15	0.76					LM4_8	0.79		
						LM4_9	0.73		
						LM4_10	0.77		
						LM4_11	0.82		

Governance and Economic Institution

Policy		Service and Programs		Advocacy		Research		Civil society	
**Obs.**	**37**	**Obs.**	**40**	**Obs.**	**30**	**Obs.**	**26**	**Obs.**	**43**
GEI1_1	0.85	GEI2_1	0.91	GEI3_1	0.65	GEI4_1a	0.85	GEI5_1	0.72
GEI1_2	0.78	GEI2_2	0.91	GEI3_2	0.81	GEI4_1b	0.89	GEI5_2	0.89
GEI1_3	0.84	GEI2_3	0.88	GEI3_3	0.62	GEI4_1c	0.70	GEI5_3	0.84
GEI1_4	0.86	GEI2_4	0.91	GEI3_4	0.64	GEI4_2	0.72	GEI5_4	0.83
GEI1_5	0.89	GEI2_5	0.82	GEI3_5	0.81	GEI4_3a	0.87	GEI5_5	0.78
GEI1_6	0.82	GEI2_6	0.91	GEI3_6a	0.90	GEI4_3b	0.88	GEI5_6	0.73
GEI1_7	0.90	GEI2_7	0.79	GEI3_6b	0.80	GEI4_3c	0.84	GEI5_7a	0.77
GEI1_8	0.89	GEI2_8a	0.88	GEI3_6c	0.67	GEI4_4a	0.85	GEI5_7b	0.71
GEI1_9a	0.90	GEI2_8b	0.88	GEI3_6d	0.59	GEI4_4b	0.92	GEI5_8	0.84
GEI1_9b	0.91	GEI2_8c	0.80	GEI3_6e	0.87	GEI4_4c	0.87	GEI5_9	0.72
GEI1_10a	0.57	GEI2_8d	0.80	GEI3_6f	0.80	GEI4_4d	0.91		
GEI1_10b	**0.42**	GEI2_8e	0.79			GEI4_5a	0.92		
GEI1_10c	0.76	GEI2_9	0.77			GEI4_5b	0.77		
GEI1_11	0.73	GEI2_10a	0.89			GEI4_5c	0.89		
GEI1_12	0.70	GEI2_10b	0.87			GEI4_5d	0.93		
GEI1_13	0.79	GEI2_10c	0.83			GEI4_6	0.88		
		GEI2_10d	0.79			GEI4_7	0.84		
		GEI2_11a	0.85			GEI4_8	0.94		
		GEI2_11c	0.82			GEI4_10	0.81		
		GEI2_11d	0.82						

Note

Bolded and shaded are the loadings of items that were dropped because they loaded poorly (<0.5) on domain factors.
