# Supplementary material for: Development of the Demographic Dividend Effort Index, a novel tool to measure existing efforts to create a favourable environment to harness a demographic dividend: results from an experts’ survey from six sub-Saharan African countries
Source: BMJ Open. 2023 Mar 21;13(3):e059937. doi: 10.1136/bmjopen-2021-059937 (PMC10040031; doi:10.1136/bmjopen-2021-059937)
Supplement: Supplementary data [file bmjopen-2021-059937supp005.pdf]

Appendix 5. Heterogeneity testing

1. Heterogeneity testing by domains and countries (Fixed effect)

	Policy	Programs	Advocacy	Research	Civil society	Overall	Between sub-groups test
**Ethiopia**							
Statistic	0.37	0.58	1.44	0.96	0.46	5.74	1.94
P-Value	1.00	0.99	0.92	0.97	0.99	1.00	0.75
I-squared	0.00%	0.00%	0.00%	0.00%	0.00%	0.00%	
**Kenya**							
Statistic	0.52	1.47	0.95	0.80	0.20	4.53	0.59
P-Value	0.99	0.92	0.97	0.98	1.00	1.00	0.96
I-squared	0.00%	0.00%	0.00%	0.00%	0.00%	0.00%	
**Nigeria**							
Statistic	1.54	1.34	1.20	0.91	0.99	6.58	0.60
P-Value	0.91	0.93	0.95	0.97	0.96	1.00	0.96
I-squared	0.00%	0.00%	0.00%	0.00%	0.00%	0.00%	
**Rwanda**							
Statistic	3.33	11.24	3.78	4.68	3.24	29.93	3.66
P-Value	0.65	0.05	0.58	0.46	0.66	0.42	0.45
I-squared	0.00%	**55.50%**	0.00%	0.00%	0.00%	3.10%	
**Senegal**							
Statistic	0.33	2.00	1.29	0.92	1.57	8.23	2.12
P-Value	1.00	0.85	0.94	0.97	0.90	1.00	0.71
I-squared	0.00%	0.00%	0.00%	0.00%	0.00%	0.00%	
**Tanzania**							
Statistic	1.17	1.16	1.22	1.28	3.15	10.13	2.14
P-Value	0.95	0.95	0.94	0.94	0.68	1.00	0.71
I-squared	0.00%	0.00%	0.00%	0.00%	0.00%	0.00%	

2. Heterogeneity testing by domains and countries (Random effect)

		Policy	Programs	Advocacy	Research	CSO	Overall
**Ethiopia**							
	Statistic	0.37	0.58	1.44	0.96	0.46	5.74
	P	1.00	0.99	0.92	0.97	0.99	1.00
	I-squared	0.00%	0.00%	0.00%	0.00%	0.00%	0.00%
	Tau-squared	0.00	0.00	0.00	0.00	0.00	0.00
**Kenya**							
	Statistic	0.52	1.47	0.95	0.80	0.20	4.53
	P	0.99	0.92	0.97	0.98	1.00	1.00
	I-squared	0.00%	0.00%	0.00%	0.00%	0.00%	0.00%
	Tau-squared	0.00	0.00	0.00	0.00	0.00	0.00
**Nigeria**							
	Statistic	1.54	1.34	1.20	0.91	0.99	6.58
	P	0.91	0.93	0.95	0.97	0.96	1.00
	I-squared	0.00%	0.00%	0.00%	0.00%	0.00%	0.00%
	Tau-squared	0.00	0.00	0.00	0.00	0.00	0.00
**Rwanda**							
	Statistic	3.33	11.24	3.78	4.68	3.24	29.93
	P	0.65	0.05	0.58	0.46	0.66	0.42
	I-squared	0.00%	**55.50%**	0.00%	0.00%	0.00%	3.10%
	Tau-squared	0.00	**0.46**	0.00	0.00	0.00	0.01
**Senegal**							
	Statistic	0.33	2.00	1.29	0.92	1.57	8.23
	P	1.00	0.85	0.94	0.97	0.90	1.00
	I-squared	0.00%	0.00%	0.00%	0.00%	0.00%	0.00%
	Tau-squared	0.00	0.00	0.00	0.00	0.00	0.00
**Tanzania**							
	Statistic	1.17	1.16	1.22	1.28	3.15	10.13
	P	0.95	0.95	0.94	0.94	0.68	1.00
	I-squared	0.00%	0.00%	0.00%	0.00%	0.00%	0.00%
	Tau-squared	0.00	0.00	0.00	0.00	0.00	0.00
