# Supplementary material for: Development of the Demographic Dividend Effort Index, a novel tool to measure existing efforts to create a favourable environment to harness a demographic dividend: results from an experts’ survey from six sub-Saharan African countries
Source: BMJ Open. 2023 Mar 21;13(3):e059937. doi: 10.1136/bmjopen-2021-059937 (PMC10040031; doi:10.1136/bmjopen-2021-059937)
Supplement: Supplementary data [file bmjopen-2021-059937supp006.pdf]

Appendix 6. Percentages of "don't know" by question/item

Variable	Ethiopia		Kenya		Nigeria		Rwanda		Senegal		Tanzania	
	n	%	n	%	n	%	n	%	n	%	n	%
FP 1.1 Prominence of Family Planning (FP): Extent to which there is a permanent												
Don't know	0	0	0	0	0	0	0	0	0	0	1	2.6
FP 1.1 Prominence of Family Planning (FP): Extent to which there is a permanent												
Don't know	0	0	0	0	0	0	0	0	0	0	1	2.6
FP 1.2 Policy on Fertility Reduction and FP: Extent to which government policy e												
FP 1.3 National FP Strategic Plan: Extent to which there is a current, comprehen												
FP 1.4 Policy on Age at Marriage: Extent to which legal age at marriage for fema												
Don't know	0	0	0	0	0	0	0	0	0	0	1	2.6
FP 1.5 Evidence-Based Policymaking: Extent to which the appropriate ministry act												
FP 1.6 Monitoring and Evaluation of National Policies/Plans/Strategies: Extent t												
Don't know	0	0	0	0	0	0	0	0	0	0	3	7.9
FP 1.7 Inclusion of Nongovernmental and Cross-sectoral Actors in Policy/Strategy												
Don't know	0	0	0	0	0	0	0	0	0	0	1	2.6

Note:

- For readability purpose, complete wording for questions/variables has been provided as Appendix 1.
- Only the percentages of respondents who responded "don't know" to the respective question or sub-question was included in this table.
- Highlighted are values > 10%

FP 1.8 Financing for FP: Extent to which the government has established and appl												
Don't know	1	5.9	1	5.9	0	0	0	0	0	0	2	5.3
FP 1.9 Percent of In-country Funding of FP Budget: Extent to which the total fam												
Don't know	4	**23.5**	1	5.9	0	0	0	0	0	0	3	7.9
FP 1.10 Information/Communications Technology Infrastructure/Capacity:¬† Extent t												
Don't know	0	0	1	5.9	1	5.6	0	0	0	0	0	0
FP 1.11 Import Laws and Legal Regulations:¬†												
Don't know	0	0	5	**29.4**	1	5.6	0	0	0	0	2	5.3
Don't know	1	5.9	3	**17.6**	1	5.6	0	0	0	0	4	**12.1**
FP 1.12 Advertising of Contraceptives Allowed: Extent of freedom from restrictio												
Don't know	0	0	1	5.9	0	0	0	0	0	0	0	0
FP 1.13 Presence of Pro-Poor FP Policies: Extent to which policies specifically												
Don't know	0	0	0	0	0	0	0	0	0	0	1	2.6
FP 1.14 Political Support for FP: Extent to which elected officials in the count												
FP 2.1 Administrative Structure: Extent to which the administrative structure an												
Don't know	0	0	0	0	0	0	0	0	0	0	1	2.9
FP 2.2 Level of Program Leaders: Relative level of seniority of the director of												
Don't know	2	**13.3**	0	0	4	**25**	0	0	0	0	1	2.9

Note:

- For readability purpose, complete wording for questions/variables has been provided as Appendix 1.
- Only the percentages of respondents who responded “don’t know” to the respective question or sub-question was included in this table.
- Highlighted are values > 10%

FP. 2.3 Community-Based Distribution: Extent to which areas of the country not e												
Don't know	0	0	1	6.2	0	0	0	0	0	0	1	2.9
FP 2.4 Postpartum Program: Extent to which all postpartum women receive postpart												
Don't know	0	0	0	0	1	6.2	0	0	0	0	0	0
FP 2.5 Home-Visiting Workers: Extent of coverage of population by workers whose												
Don't know	0	0	1	6.2	1	6.2	0	0	0	0	1	2.9
FP 2.6 Availability and Accessibility of FP Commodities/Supplies and Services Pa												
FP 2.6.1	1	6.7	2	**12.5**	0	0	0	0	0	0	3	8.6
FP 2.6.2	0	0	1	6.2	0	0	0	0	0	0	1	3
FP 2.6.3	0	0	1	6.2	2	**13.3**	0	0	0	0	2	5.7
FP 2.6.4	0	0	1	6.2	0	0	0	0	0	0	1	2.9
FP 2.7 Availability and Accessibility of FP Commodities/Supplies and Services Pa												
FP 2.7.1	0	0	0	0	0	0	0	0	0	0	3	8.6
FP 2.7.2	0	0	0	0	0	0	0	0	0	0	3	8.6
FP 2.7.3	0	0	0	0	0	0	0	0	1	**14.3**	3	8.6
FP 2.7.4	0	0	0	0	1	6.2	0	0	0	0	3	8.6
FP 2.7.5	0	0	0	0	0	0	0	0	1	**14.3**	3	8.8
FP 2.7.6	0	0	0	0	3	**18.8**	0	0	0	0	4	**11.4**
FP 2.7.7	0	0	1	6.2	1	6.2	0	0	0	0	4	**11.4**

Note:

- For readability purpose, complete wording for questions/variables has been provided as Appendix 1.
- Only the percentages of respondents who responded “don’t know” to the respective question or sub-question was included in this table.
- Highlighted are values > 10%

FP 2.8 Availability and Accessibility of FP Commodities/Supplies and Services Pa												
FP 2.8.1	0	0	2	**12.5**	1	6.2	0	0	0	0	4	**11.4**
FP 2.8.2	0	0	2	**12.5**	1	6.2	0	0	0	0	4	**11.4**
FP 2.8.3	0	0	0	0	0	0	0	0	0	0	3	8.6
FP 2.8.4	0	0	0	0	0	0	0	0	0	0	3	8.6
FP 2.9 FP Service Quality: Please rate the general quality of family planning se												
FP 2.10 Incentives and Disincentives: Extent to which monetary or other incentiv												
Don't know	0	0	1	6.2	1	6.2	0	0	0	0	3	8.6
FP 2.11 Adolescent Programs: Extent to which information, services and activitie												
Don't know	0	0	1	6.2	0	0	0	0	0	0	0	0
FP 2.12 Training Program: Extent to which training programs, for each category o												
Don't know	0	0	1	6.2	0	0	0	0	0	0	1	2.9
FP 2.13 Personnel Carry Out Assigned Tasks: Extent to which all categories of fa												
Don't know	0	0	1	6.2	0	0	0	0	0	0	2	5.7
FP 2.14 Logistics and Transport: Extent to which the logistics and transport sys												
Don't know	0	0	0	0	0	0	0	0	0	0	3	8.6
FP 2.15 Supervision System: Extent to which the system of supervision at all lev												
Don't know	0	0	0	0	0	0	0	0	0	0	1	2.9

Note:

- For readability purpose, complete wording for questions/variables has been provided as Appendix 1.
- Only the percentages of respondents who responded "don't know" to the respective question or sub-question was included in this table.
- Highlighted are values > 10%

FP 2.16 Involvement of Other Ministries and Public Agencies: Extent to which oth												
Don't know	0	0	0	0	0	0	0	0	0	0	1	2.9
FP 2.17 Involvement of Private-Sector Agencies and Groups: Extent to which priva												
FP 2.18 Mass Media for Information Education Communication: Frequency and extent												
FP 2.19 Cultural and Social Norms Barriers: Extent to which cultural and social												
FP 2.20 Youth: Extent to which the government supports youth-led FP initiatives												
FP 2.21 FP Effort Influences ,Äì Forces Affecting the FP Program: Forces affecting												
FP 2.21.1	0	0	0	0	0	0	0	0	0	0	2	5.9
FP 2.21.2	0	0	0	0	0	0	0	0	0	0	1	3
FP 2.21.3	0	0	0	0	0	0	0	0	0	0	2	5.9
FP 2.21.4	0	0	1	6.2	0	0	0	0	0	0	1	2.9
FP 2.21.5	0	0	0	0	0	0	0	0	0	0	2	5.9
FP 3.1 Influence of FP Leaders/Champions: Extent to which the country has one or												
Don't know	0	0	0	0	0	0	0	0	0	0	1	2.9
FP 3.2 Statement by Leaders: Extent to which the head of government, as well as												
FP 3.3 Media Coverage for FP: Extent to and frequency with which national media												

Note:

- For readability purpose, complete wording for questions/variables has been provided as Appendix 1.
- Only the percentages of respondents who responded “don’t know” to the respective question or sub-question was included in this table.
- Highlighted are values > 10%

FP 3.4 FP Effort Influences - Justification: How important is each of the follow												
FP 3.4.1	0	0	0	0	0	0	0	0	0	0	1	2.9
FP 3.4.2	0	0	1	6.2	0	0	0	0	0	0	0	0
FP 3.4.3	0	0	0	0	0	0	0	0	0	0	1	2.9
FP 3.4.4	0	0	1	6.2	0	0	0	0	0	0	3	8.8
FP 3.4.5	0	0	1	6.7	0	0	0	0	0	0	3	9.4
FP 3.4.6	0	0	1	6.2	0	0	0	0	0	0	1	2.9
FP 3.4.7	0	0	0	0	0	0	0	0	0	0	1	2.9
FP 3.5 FP Effort Influences ‚Äì Special Populations: To what extent does the famil												
Don't know	0	0	2	**12.5**	0	0	0	0	0	0	1	2.9
FP 3.6 Social Marketing: Extent of coverage of the country by a social marketing												
Don't know	0	0	2	**12.5**	2	**13.3**	0	0	0	0	0	0
FP 4.1 Strength of Observatory/Stakeholder/Technical Working Group for FP: Exten												
Don't know	0	0	1	6.2	0	0	0	0	0	0	1	3.1
FP 4.2 Education/Research Strategy: Extent to which the national FP plan and/or												
Don't know	0	0	0	0	1	6.7	0	0	0	0	1	3.1
FP 4.3 Data Gathering Partners:												
FP 4.3.1	0	0	0	0	1	6.7	0	0	0	0	0	0
FP 4.3.2	0	0	0	0	3	**20**	0	0	0	0	0	0

Note:

- For readability purpose, complete wording for questions/variables has been provided as Appendix 1.
- Only the percentages of respondents who responded “don’t know” to the respective question or sub-question was included in this table.
- Highlighted are values > 10%

FP 4.3.3	0	0	0	0	3	**20**	0	0	0	0	1	3.1
FP 4.4 Topical Data Gathering:												
FP 4.4.1	0	0	1	6.2	0	0	0	0	0	0	0	0
FP 4.4.2	0	0	0	0	1	6.7	0	0	0	0	0	0
FP 4.4.3	0	0	0	0	1	6.7	0	0	0	0	0	0
FP 4.4.4	0	0	0	0	2	**13.3**	0	0	0	0	2	6.2
FP 4.5 Quality/Coverage of Data: Extent to which current research/data is sex an												
FP 4.5.1	0	0	0	0	1	6.7	0	0	0	0	1	3.1
FP 4.5.2	0	0	0	0	0	0	0	0	0	0	2	6.2
FP 4.5.3	0	0	0	0	1	6.7	0	0	0	0	3	9.4
FP 4.5.4	0	0	0	0	1	6.7	0	0	0	0	2	6.2
FP 4.6 Record-Keeping: Extent to which systems for client recordkeeping, clinic												
Don't know	0	0	0	0	1	6.7	0	0	0	0	1	3.1
FP 4.7 Quality Research Institutions: Extent to which the country supports and s												
Don't know	0	0	1	6.2	1	6.7	0	0	0	0	1	3.1
FP 4.8 Evaluation: Extent to which program statistics, surveys, and small studie												
Don't know	0	0	1	6.2	2	**13.3**	0	0	0	0	4	**12.5**
FP 4.9 Management‚Äôs Use of Evaluation Findings: Extent to which local-level prog												
Don't know	0	0	1	6.2	1	6.7	0	0	0	0	1	3.1

Note:

- For readability purpose, complete wording for questions/variables has been provided as Appendix 1.
- Only the percentages of respondents who responded “don’t know” to the respective question or sub-question was included in this table.
- Highlighted are values > 10%

FP 4.10 Ministerial Use of Evaluation Findings: Extent to which the Ministry adm												
Don't know	0	0	1	6.2	1	6.7	0	0	0	0	1	3.1
FP 4.11 Dissemination of Information to Other Implementing Bodies: Extent to whi												
Don't know	0	0	1	6.2	0	0	0	0	0	0	1	3.1
FP 5.1 CSO Actor Power: Extent to which CSO actors are in strong placements with												
Don't know	0	0	0	0	0	0	0	0	0	0	1	3.2
FP 5.2 Budget Analysis as a CSO Tool: Extent to which CSOs utilize budget analys												
Don't know	1	6.7	1	6.2	0	0	0	0	0	0	1	3.2
FP 5.3 Facility-Based Service Delivery Support: Extent to which CSOs are involve												
FP 5.4 Community-Based Service Delivery Support: Extent to which CSOs participat												
FP 5.5 Mobile Outreach for Community-Based Service Delivery Support: Extent to w												
Don't know	0	0	0	0	0	0	0	0	0	0	1	3.2
FP 5.6 Social Franchising: Extent to which CSOs are involved in social franchisi												
Don't know	0	0	1	6.2	0	0	0	0	0	0	3	9.7
FP 5.7 mHealth: Extent to which CSOs are leveraging mHealth to utilize text mess												
Don't know	0	0	0	0	2	**13.3**	0	0	0	0	2	6.5

Note:

- For readability purpose, complete wording for questions/variables has been provided as Appendix 1.
- Only the percentages of respondents who responded "don't know" to the respective question or sub-question was included in this table.
- Highlighted are values > 10%

FP 5.8 Human Rights and Quality of Care: Extent to which CSOs advocate for and s														
FP 5.9 Social and Behavior Change (SBC): Extent to which CSOs support SBC interv														
Don't know	0	0	0	0	0	0	0	0	0	0	2	6.5		
FP 5.10 Youth: Extent to which CSOs support FP policies, interventions and progr														
Don't know	0	0	0	0	0	0	0	0	0	0	1	3.2		
FP 5.11 Programming for Men: Extent to which CSOs support FP policies, intervent														
Don't know	0	0	0	0	0	0	0	0	0	0	2	6.5		
FP 5.12 Advocacy/Accountability: Extent to which CSOs support the training and c														
Don't know	0	0	0	0	0	0	0	0	0	0	1	3.2		
FP 5.13 CSO-Led Assessment and Monitoring: Extent to which CSOs assess, monitor														
Don't know	0	0	0	0	0	0	0	0	0	0	3	9.7		
FP 5.14 Forming Partnerships between CSOs: Extent to which CSOs have formed nati														
Don't know	0	0	0	0	0	0	0	0	0	0	2	6.5		
MCH 1.1 Adequate Policy: Ministry of Health policies toward pregnancy and delive														
Don't know	0	0	0	0	0	0	0	0	1	5.9	0	0		
Don't know	0	0	0	0	0	0	0	0	1	5.9	0	0		
MCH 1.2 Universal Access: Extent to which there are constitutional and legal ent														

Note:

- For readability purpose, complete wording for questions/variables has been provided as Appendix 1.
- Only the percentages of respondents who responded “don’t know” to the respective question or sub-question was included in this table.
- Highlighted are values > 10%

Don't know	0	0	0	0	0	0	0	0	1	5.9	0	0
MCH 1.3 Multisector Involvement: Extent to which policies are developed through												
Don't know	0	0	0	0	0	0	0	0	1	5.9	0	0
MCH 1.4 Competent Service Providers: Extent to which policies are reasonable and												
Don't know	0	0	0	0	0	0	0	0	1	5.9	0	0
MCH 1.5 Abortion Legislation: Extent to which a favorable policy exists towards												
Don't know	0	0	1	5.6	0	0	0	0	1	5.9	2	**10.5**
MCH 1.6. Post-Abortion Care Legislation: Extent to which a favorable policy exis												
Don't know	0	0	4	**22.2**	0	0	0	0	1	5.9	2	**10.5**
MCH 1.7 Policy Reviews and Updates: Extent to which policies are regularly revie												
Don't know	0	0	2	**11.1**	0	0	0	0	1	5.9	0	0
MCH 1.8 High-Level Actor Placement: Extent and level to which the director of ma												
Don't know	0	0	4	**22.2**	0	0	0	0	1	5.9	1	5.3
MCH 1.9 Financing for MCH: Extent to which the government budgets for safe pregn												
Don't know	1	6.7	0	0	0	0	0	0	1	5.9	2	**10.5**
MCH 1.10 Affordability: Extent to which all MCH-related services and drugs are a												
Don't know	0	0	0	0	0	0	0	0	1	5.9	0	0

Note:

- For readability purpose, complete wording for questions/variables has been provided as Appendix 1.
- Only the percentages of respondents who responded “don’t know” to the respective question or sub-question was included in this table.
- Highlighted are values > 10%

MCH 1.11 Private Sector: Extent to which the private sector (doctors, midwives,												
Don't know	0	0	0	0	0	0	0	0	1	5.9	1	5.3
MCH 1.12 MCH Department: Extent to which the Ministry of Health has a department												
Don't know	0	0	1	5.6	0	0	0	0	1	5.9	0	0
MCH 1.13 Potential Solutions: Extent to which the government recognizes potentia												
MCH 1.13.1	1	6.7	1	5.6	0	0	0	0	1	5.9	0	0
MCH 1.13.2	0	0	1	5.9	0	0	0	0	1	5.9	0	0
MCH 1.13.3	0	0	2	**11.1**	0	0	0	0	1	5.9	0	0
MCH 1.13.4	0	0	1	5.6	0	0	0	0	1	5.9	0	0
MCH 1.13.5	0	0	1	5.6	0	0	0	0	1	5.9	0	0
MCH 2.1 Obstetric Care Availability ‚Äì All Centers: Extent to which all primary h												
MCH 2.1.2	0	0	1	6.2	0	0	0	0	1	6.2	1	5.9
MCH 2.1.3	1	7.7	2	**12.5**	0	0	0	0	1	6.2	1	5.9
MCH 2.1.4	1	7.7	2	**12.5**	0	0	0	0	1	6.2	1	5.9
MCH 2.1.5	1	8.3	2	**12.5**	0	0	0	0	1	6.2	2	**11.8**
MCH 2.1.6	1	7.7	0	0	0	0	0	0	1	6.2	1	5.9
MCH 2.1.7	0	0	1	6.2	0	0	0	0	1	6.2	0	0
MCH 2.1.8	0	0	0	0	0	0	0	0	1	6.2	0	0
MCH 2.2 Obstetric Care Availability ‚Äì Secondary Healthcare Facilities: Extent to												
MCH 2.2.1	0	0	0	0	0	0	0	0	1	6.2	1	5.9

Note:

- For readability purpose, complete wording for questions/variables has been provided as Appendix 1.
- Only the percentages of respondents who responded “don’t know” to the respective question or sub-question was included in this table.
- Highlighted are values > 10%

MCH 2.2.2	1	8.3	0	0	0	0	0	0	1	6.2	3	**17.6**
MCH 2.2.3	0	0	1	6.2	0	0	0	0	1	6.2	1	6.7
MCH 2.3 Pregnancy-Related Services: Extent to which all pregnant women have adeq												
MCH 2.3.1	1	7.7	1	6.2	0	0	0	0	1	6.2	2	**11.8**
MCH 2.3.2	1	7.7	0	0	0	0	0	0	1	6.2	2	**11.8**
MCH 2.3.3	1	7.7	1	6.2	0	0	0	0	1	6.2	2	**11.8**
MCH 2.3.4	1	7.7	1	6.2	0	0	0	0	1	6.2	1	6.2
MCH 2.3.5	1	7.7	1	6.2	0	0	0	0	1	6.2	1	5.9
MCH 2.3.6	0	0	1	6.2	0	0	0	0	1	6.2	1	6.7
MCH 2.3.7	0	0	1	6.2	0	0	0	0	1	6.2	0	0
MCH 2.3.8	0	0	0	0	0	0	0	0	1	6.2	0	0
MCH 2.3.9	1	7.7	1	6.7	0	0	0	0	1	6.2	2	**12.5**
MCH 2.4 Ante-natal Care for Unmarried and HIV Positive Women: Extent to which, a												
MCH 2.4.1	0	0	1	6.2	1	**12.5**	0	0	1	6.2	3	**17.6**
MCH 2.4.2	1	7.7	1	6.2	1	**12.5**	0	0	1	6.2	3	**17.6**
MCH 2.4.3	0	0	2	**12.5**	0	0	0	0	1	6.2	3	**17.6**
MCH 2.4.4	0	0	1	6.7	0	0	0	0	1	6.2	1	5.9
MCH 2.5 Newborn Care: Extent to which, for newborn care, all infants whether del												
MCH 2.5.1	1	7.7	2	**12.5**	0	0	0	0	1	6.2	3	**17.6**
MCH 2.5.2	1	7.7	2	**12.5**	0	0	0	0	1	6.2	3	**17.6**
MCH 2.5.3	0	0	1	6.2	0	0	0	0	1	6.2	0	0
MCH 2.5.4	0	0	2	**12.5**	0	0	0	0	1	6.2	1	5.9

Note:

- For readability purpose, complete wording for questions/variables has been provided as Appendix 1.
- Only the percentages of respondents who responded “don’t know” to the respective question or sub-question was included in this table.
- Highlighted are values > 10%

MCH 2.5.5	1	7.7	3	**18.8**	0	0	0	0	1	6.2	3	**17.6**
MCH 2.5.6	0	0	1	6.2	0	0	0	0	1	6.2	0	0
MCH 2.5.7	0	0	1	6.2	0	0	0	0	1	6.2	0	0
MCH 2.6 Family Planning ,Äì All Centers: Extent to which there exists proper provi												
MCH 2.6.1	0	0	0	0	1	**12.5**	0	0	1	6.2	1	5.9
MCH 2.6.2	0	0	1	6.2	0	0	0	0	1	6.2	0	0
MCH 2.6.3	0	0	2	**12.5**	0	0	0	0	1	6.2	0	0
MCH 2.6.4	0	0	1	6.2	0	0	0	0	1	6.2	0	0
MCH 2.6.5	0	0	1	6.7	0	0	0	0	1	6.2	0	0
MCH 2.7 Family Planning ,Äì District/Sub-county Hospitals: Extent to which there e												
MCH 2.7.1	1	7.7	1	6.2	1	**12.5**	0	0	1	6.2	1	5.9
MCH 2.7.2	1	7.7	1	6.7	0	0	0	0	1	6.2	0	0
MCH 2.7.3	0	0	1	6.2	0	0	0	0	1	6.2	0	0
MCH 2.7.4	0	0	2	**12.5**	0	0	0	0	1	6.2	0	0
MCH 2.7.5	0	0	1	6.2	1	**12.5**	0	0	1	6.2	2	**12.5**
MCH 2.7.6	0	0	2	**12.5**	1	**12.5**	0	0	1	6.2	2	**11.8**
MCH 2.7.7	0	0	1	6.2	0	0	0	0	1	6.2	0	0
MCH 2.8 Childhood Illness: Extent to which the WHO-recommended case management o												
MCH 2.8.1	0	0	1	6.7	1	**12.5**	0	0	1	6.2	1	5.9
MCH 2.8.2	0	0	1	6.2	0	0	0	0	1	6.7	1	5.9
MCH 3.1 Government Messages: Extent to which high officials in the government, i												

Note:

- For readability purpose, complete wording for questions/variables has been provided as Appendix 1.
- Only the percentages of respondents who responded “don’t know” to the respective question or sub-question was included in this table.
- Highlighted are values > 10%

Don't know	0	0	0	0	0	0	0	0	1	6.2	0	0
MCH 3.2 Mass Media: Extent to which the national program uses the mass media to												
Don't know	0	0	0	0	0	0	0	0	1	6.2	0	0
MCH 3.3 Community-Level Media: Extent to which community groups take part in sys												
Don't know	0	0	0	0	0	0	0	0	1	6.2	0	0
MCH 3.4 Educational Materials: Extent to which the appropriate ministry (MOH) su												
Don't know	1	7.7	0	0	0	0	0	0	1	6.2	0	0
MCH 3.5 Self-Review of Maternal Mortality Cases: Extent to which each health fac												
Don't know	0	0	1	8.3	0	0	0	0	1	6.2	1	6.7
MCH 3.6 Informing Policies with New Evidence: Extent to which each health facili												
Don't know	0	0	1	8.3	0	0	0	0	1	6.2	0	0
MCH 4.1 Strength of Observatory/Stakeholder/Technical Working Group for MCH: Ext												
MCH 4.1.1	0	0	0	0	1	**12.5**	0	0	1	6.2	0	0
MCH 4.1.2	0	0	1	8.3	0	0	0	0	1	6.2	1	7.7
MCH 4.1.3	0	0	0	0	0	0	0	0	1	6.2	1	7.1
MCH 4.2 MCH Research Strategy: Extent to which national documents include a comp												
Don't know	0	0	0	0	0	0	0	0	3	**18.8**	1	7.1
MCH 4.3 Data-Gathering Partners:												

Note:

- For readability purpose, complete wording for questions/variables has been provided as Appendix 1.
- Only the percentages of respondents who responded “don’t know” to the respective question or sub-question was included in this table.
- Highlighted are values > 10%

MCH 4.3.1	0	0	1	8.3	0	0	0	0	1	6.2	1	7.1
MCH 4.3.2	0	0	2	**16.7**	0	0	0	0	1	6.2	1	7.1
MCH 4.3.3	0	0	1	9.1	1	**12.5**	0	0	1	6.2	1	7.7
MCH 4.4 Topical Research:												
MCH 4.4.1	0	0	2	**16.7**	1	**12.5**	0	0	1	6.2	1	7.1
MCH 4.4.2	0	0	1	9.1	1	**12.5**	0	0	1	6.2	1	7.1
MCH 4.4.3	0	0	1	8.3	1	**12.5**	0	0	1	6.2	1	7.1
MCH 4.4.4	0	0	4	**33.3**	1	**12.5**	0	0	1	6.2	1	7.1
MCH 4.5 Quality/Coverage of Data: Extent to which current research/data is disag												
MCH 4.5.1	0	0	2	**16.7**	0	0	0	0	1	6.2	1	7.1
MCH 4.5.2	0	0	1	8.3	0	0	0	0	1	6.2	1	7.1
MCH 4.5.3	0	0	2	**16.7**	0	0	0	0	1	6.2	1	7.1
MCH 4.5.4	0	0	2	**16.7**	0	0	0	0	1	6.2	2	**14.3**
MCH 4.5.5	0	0	3	**25**	0	0	0	0	1	6.2	2	**14.3**
MCH 4.6 Record-Keeping: Extent to which systems for client recordkeeping, clinic												
Don't know	0	0	1	8.3	0	0	0	0	1	6.2	1	7.1
MCH 4.7 Quality Research Institutions: Extent to which the country has the capac												
Don't know	0	0	1	8.3	0	0	0	0	1	6.2	1	7.1
MCH 4.8 Evaluation: Extent to which program statistics, surveys, and small studi												
Don't know	0	0	1	8.3	0	0	0	0	1	6.2	1	7.1

Note:

- For readability purpose, complete wording for questions/variables has been provided as Appendix 1.
- Only the percentages of respondents who responded “don’t know” to the respective question or sub-question was included in this table.
- Highlighted are values > 10%

MCH 4.9 Management‚Äôs Use of Evaluation Findings: Extent to which local-level pro												
Don't know	0	0	1	8.3	0	0	0	0	1	6.2	2	**14.3**
MCH 4.10 Ministerial Use of Evaluation Findings: Extent to which the Ministry ad												
Don't know	0	0	1	8.3	0	0	0	0	1	6.2	1	7.1
MCH 4.11 Dissemination of Information to Other Implementing Bodies: Extent to wh												
Don't know	0	0	2	**16.7**	0	0	0	0	1	6.2	1	7.1
MCH 5.1 CSO Actor Power: Extent to which CSO actors are in strong placements wit												
Don't know	0	0	1	10	0	0	0	0	1	6.7	2	**14.3**
MCH 5.2 Budget Analysis as a CSO Tool: Extent to which CSOs utilize budget analy												
Don't know	2	**20**	1	10	0	0	0	0	1	6.7	3	**21.4**
MCH 5.3 Facility-Based Service Delivery Support: Extent to which CSOs are involv												
Don't know	0	0	0	0	0	0	0	0	1	6.7	1	7.1
MCH 5.4 Community-Based Service Delivery Support: Extent to which CSOs participa												
Don't know	0	0	0	0	0	0	0	0	1	6.7	1	7.1
MCH 5.5 Mobile Outreach for Community-Based Service Delivery Support: Extent to												
Don't know	0	0	0	0	0	0	0	0	1	6.7	1	7.1
MCH 5.6 Social Franchising: Extent to which CSOs are involved with social franch												

Note:

- For readability purpose, complete wording for questions/variables has been provided as Appendix 1.
- Only the percentages of respondents who responded “don’t know” to the respective question or sub-question was included in this table.
- Highlighted are values > 10%

Don't know	0	0	0	0	0	0	0	0	1	6.7	2	**14.3**
MCH 5.7 mHealth: Extent to which CSOs are leveraging mHealth to utilize text mes												
Don't know	0	0	1	10	0	0	0	0	1	6.7	2	**14.3**
MCH 5.8 Human Rights and Quality of Care: Extent to which CSOs advocate for and												
Don't know	0	0	1	10	0	0	0	0	1	6.7	2	**14.3**
MCH 5.9 Social and Behavior Change (SBC): Extent to which CSOs support SBC inter												
Don't know	0	0	1	10	0	0	0	0	1	6.7	1	7.1
MCH 5.10 Advocacy/Accountability: Extent to which CSOs support the training and												
Don't know	0	0	0	0	0	0	0	0	1	6.7	1	7.1
MCH 5.11 CSO-Led Assessment and Monitoring: Extent to which CSOs assess, monitor												
Don't know	0	0	0	0	0	0	0	0	1	6.7	1	7.1
MCH 5.12 Forming Partnerships between CSOs: Extent to which CSOs have formed nat												
Don't know	1	10	1	10	0	0	0	0	1	6.7	2	**14.3**
ED 1.1 Quality: Extent to which there are national plans/strategies/programs to												
ED 1.2 Disadvantaged Groups:												
Don't know	3	**20**	0	0	2	**18.2**	0	0	0	0	0	0
Don't know	1	6.7	1	**20**	0	0	0	0	0	0	0	0
ED 1.3 Teachers: Extent to which teacher recruitment, development and management												

Note:

- For readability purpose, complete wording for questions/variables has been provided as Appendix 1.
- Only the percentages of respondents who responded “don’t know” to the respective question or sub-question was included in this table.
- Highlighted are values > 10%

ED 1.4 Teachers,Äô Accountability: Extent to which national plans include specific												
Don't know	1	6.7	0	0	0	0	0	0	0	0	0	0
ED 1.5 Teacher Quality: Extent to which education policies/strategies/programs i												
Don't know	1	6.7	0	0	0	0	0	0	0	0	0	0
ED 1.6 Collaboration in Education: Extent to which the policy makes provisions t												
ED 1.7 Gender Parity SDG: Extent to which national policies/plans/strategies con												
Don't know	1	6.7	0	0	0	0	0	0	0	0	0	0
ED 1.8 Education Financing: Extent to which new education plans include financin												
Don't know	2	**13.3**	0	0	0	0	0	0	0	0	0	0
ED 1.9 Gender Parity Planning: Extent to which there are national plans/strategi												
ED 1.9.1	0	0	0	0	0	0	1	**14.3**	0	0	0	0
ED 1.9.2	0	0	0	0	0	0	1	**14.3**	0	0	0	0
ED 1.9.3	0	0	0	0	3	**27.3**	0	0	0	0	0	0
ED 1.10 Adult Illiteracy: Extent to which national policies/plans/strategies aim												
ED 1.10.1	2	**13.3**	0	0	1	8.3	0	0	0	0	0	0
ED 1.10.2	2	**13.3**	0	0	1	8.3	0	0	0	0	0	0
ED 1.11 Out-of-School Children: Extent to which policies address the plight of o												
Don't know	1	6.7	0	0	0	0	0	0	0	0	0	0

Note:

- For readability purpose, complete wording for questions/variables has been provided as Appendix 1.
- Only the percentages of respondents who responded "don't know" to the respective question or sub-question was included in this table.
- Highlighted are values > 10%

ED 1.12 Technology Integration: Extent to which strategies have been developed a												
ED 1.13. Technology Literacy: Extent to which resources have been allocated to i												
ED 2.1 Teacher Deployment: Extent to which strategies/plans are used to ensure t												
Don't know	2	**13.3**	0	0	0	0	0	0	0	0	0	0
ED 2.2 Teacher Quality: Extent to which teacher quality is recognized as a key f												
Don't know	0	0	1	**20**	0	0	0	0	0	0	0	0
ED 2.3 Gender Parity: ¬†Extent to which resources have been utilized to improve e												
Don't know	1	6.7	0	0	0	0	0	0	0	0	0	0
ED 2.4 Parental Involvement in Education: Extent to which parents are involved i												
ED 2.5 Standardized Assessments: Extent to which all schools utilize standardize												
Don't know	2	**13.3**	0	0	0	0	0	0	0	0	0	0
ED 2.6. Use of Assessment to Inform Progress: Extent to which adjustments to sch												
Don't know	2	**13.3**	0	0	1	8.3	0	0	0	0	0	0
ED 3.1 Equality: Extent to which equity and equality in education have been high												
ED 3.2 Quality: Extent to which quality of education takes part in advocacy-rela												
Don't know	1	7.1	0	0	0	0	0	0	0	0	0	0

Note:

- For readability purpose, complete wording for questions/variables has been provided as Appendix 1.
- Only the percentages of respondents who responded “don’t know” to the respective question or sub-question was included in this table.
- Highlighted are values > 10%

ED 3.3 Teachers‚Äô Obstacles: Extent to which the needs of teachers have been high												
ED 3.4 Institutions‚Äô Obstacles: Extent to which the challenges of educational in												
ED 4.1 Strength of Observatory/Stakeholder/Technical Working Group for ED: Exten												
ED 4.1.1	2	**14.3**	1	**20**	0	0	0	0	0	0	0	0
ED 4.1.2	2	**16.7**	0	0	1	8.3	0	0	0	0	0	0
ED 4.1.3	2	**15.4**	0	0	0	0	0	0	0	0	0	0
ED 4.2 Education/Research Strategy: Extent to which the national education plan												
Don't know	1	7.1	0	0	1	8.3	0	0	0	0	0	0
ED 4.3 Data Gathering Partners:												
ED 4.3.1	1	7.1	0	0	0	0	0	0	0	0	0	0
ED 4.3.2	1	7.1	0	0	1	9.1	0	0	0	0	0	0
ED 4.3.3	0	0	0	0	1	8.3	0	0	0	0	0	0
ED 4.4 Topical Research:												
ED 4.4.1	0	0	0	0	1	8.3	0	0	0	0	0	0
ED 4.4.2	0	0	0	0	1	8.3	0	0	0	0	0	0
ED 4.4.3	0	0	0	0	1	9.1	0	0	0	0	0	0
ED 4.4.4	0	0	1	**20**	1	8.3	0	0	0	0	0	0
ED 4.5 Quality/Coverage of Data: Extent to which current research/data is sex an												
ED 4.5.1	2	**14.3**	0	0	0	0	0	0	0	0	0	0

Note:

- For readability purpose, complete wording for questions/variables has been provided as Appendix 1.
- Only the percentages of respondents who responded “don’t know” to the respective question or sub-question was included in this table.
- Highlighted are values > 10%

ED 4.5.2	1	7.1	0	0	0	0	0	0	0	0	0	0
ED 4.5.3	1	7.1	0	0	0	0	0	0	0	0	0	0
ED 4.5.4	1	7.1	0	0	0	0	0	0	0	0	0	0
ED 4.6 Record-Keeping: Extent to which systems for education-related recordkeepi												
Don't know	1	7.1	0	0	1	8.3	0	0	0	0	0	0
ED 4.7 Quality Research Institutions: Extent to which the country has the capaci												
Don't know	1	7.1	1	**20**	1	8.3	0	0	0	0	0	0
ED 4.8 Evaluation: Extent to which education program statistics, surveys, and sm												
Don't know	0	0	0	0	1	8.3	0	0	0	0	0	0
ED 4.9 Management‚Äôs Use of Evaluation Findings: Extent to which local-level educ												
Don't know	1	7.1	0	0	1	8.3	0	0	0	0	0	0
ED 4.10 Ministerial Use of Evaluation Findings: Extent to which the relevant min												
Don't know	4	**28.6**	0	0	1	8.3	0	0	0	0	0	0
ED 4.11 Dissemination of Information to Other Implementing Bodies:												
ED 5.1 CSO Actor Power: Extent to which CSO actors are in strong placements with												
Don't know	1	7.1	1	**20**	1	8.3	0	0	0	0	0	0
ED 5.2 Budget Analysis as a CSO Tool: Extent to which CSOs utilize budget analys												
Don't know	5	**35.7**	2	**40**	2	**16.7**	0	0	0	0	0	0

Note:

- For readability purpose, complete wording for questions/variables has been provided as Appendix 1.
- Only the percentages of respondents who responded “don’t know” to the respective question or sub-question was included in this table.
- Highlighted are values > 10%

ED 5.3 Community inclusion: Extent to which CSOs are involved in efforts to expl												
Don't know	0	0	0	0	2	**16.7**	0	0	0	0	0	0
ED 5.4 Community Involvement in Education: Extent to which CSOs participate in b												
Don't know	0	0	0	0	1	8.3	0	0	0	0	0	0
ED 5.5 Social Franchising: Extent to which CSOs are involved with social franchi												
Don't know	0	0	1	**20**	1	8.3	0	0	0	0	0	0
ED 5.6 Technology: Extent to which CSOs are leveraging information communication												
Don't know	0	0	1	**20**	1	8.3	0	0	0	0	0	0
ED 5.7 Human Rights and Access to/Quality of Education: Extent to which CSOs adv												
Don't know	0	0	0	0	2	**16.7**	0	0	0	0	0	0
ED 5.8 Social and Behavior Change (SBC): Extent to which CSOs support SBC interv												
Don't know	0	0	0	0	2	**16.7**	0	0	0	0	0	0
ED 5.9 Advocacy/Accountability: Extent to which CSOs support the training and ca												
Don't know	0	0	1	**20**	2	**16.7**	0	0	0	0	0	0
ED 5.10 CSO-Led Assessment and Monitoring: Extent to which CSOs assess, monitor												
Don't know	1	7.1	1	**20**	2	**16.7**	0	0	1	**11.1**	0	0
ED 5.11 Forming Partnerships between CSOs: Extent to which CSOs have formed nati												

Note:

- For readability purpose, complete wording for questions/variables has been provided as Appendix 1.
- Only the percentages of respondents who responded “don’t know” to the respective question or sub-question was included in this table.
- Highlighted are values > 10%

Don't know	1	7.1	1	**20**	2	**16.7**	0	0	0	0	0	0
WE 1.1 Gender Equality Policy: Extent to which gender equality is considered/int												
WE 1.2 Level of commitment to the Convention on the Elimination of all Forms of												
WE 1.3 Equity in Marriage and Family Policies: Extent to which the principle of												
WE 1.4 Policy on Age at Marriage: Extent to which legal age at marriage for fema												
WE 1.5 Violence Against Women Measures: Level of regulations and measures on vio												
Don't know	0	0	0	0	0	0	1	6.2	0	0	0	0
WE 1.6 Female Genital Cutting/Mutilation: Level of regulation and measures on fe												
Don't know	0	0	0	0	0	0	1	6.2	0	0	0	0
WE 1.7 Women‚Äôs Health Measures: Level of protocols for the protection of women‚Äôs												
WE 1.8 Education Measures: Extent to which there are mechanisms to promote girls												
WE 1.9 Rights Measures: Level of enforcement of policies on women‚Äôs rights.												
WE 1.10 Equitable Work: Extent to which there are policies on equitable working												
WE 1.11 Equitable Land/Asset Ownership: Extent to which there are policies to af												

Note:

- For readability purpose, complete wording for questions/variables has been provided as Appendix 1.
- Only the percentages of respondents who responded "don't know" to the respective question or sub-question was included in this table.
- Highlighted are values > 10%

WE 1.12 Conflict, Women & Peacebuilding: Level of implementation of Security UN												
Don't know	3	**37.5**	1	10	0	0	0	0	0	0	1	7.7
WE 1.13 Gender Quotas in Politics: Existence, and level of implementation, of qu												
WE 1.14 Sustainable Development Goal 5: Level of commitment to fulfilling Sustai												
Don't know	1	**12.5**	0	0	0	0	0	0	0	0	0	0
WE 1.15 Customary Law Revision: Extent to which customary laws have been reviewe												
Don't know	0	0	0	0	0	0	4	**25**	0	0	0	0
WE 1.16 Costing for WE: Extent to which there is sufficient planning and allocat												
Don't know	2	**25**	2	**20**	0	0	0	0	0	0	0	0
WE 1.17 Staffing for WE: Extent to which:												
WE 1.18 Transparency and Accountability: Extent to which the government provides												
Don't know	1	**12.5**	0	0	1	**11.1**	0	0	0	0	0	0
WE 2.1 Gender Mainstreaming: Extent to which services or programs within and acr												
WE 2.2 Gender-Transformative Programming: Extent to which services or programs w												
Don't know	0	0	1	10	0	0	0	0	0	0	0	0
WE 2.3 Education: Extent to which education services or programs focus on girl,Äôs												

Note:

- For readability purpose, complete wording for questions/variables has been provided as Appendix 1.
- Only the percentages of respondents who responded “don’t know” to the respective question or sub-question was included in this table.
- Highlighted are values > 10%

WE 2.4. Dropouts: Extent to which there are programs to prevent female dropouts.												
WE 2.5 Technology Literacy: Extent to which technology literacy is promoted amon												
WE 2.6 Employment: Extent to which job-creation programs and labor market police												
WE 2.7 Women as Mothers and Workers: Extent to which the government makes possib												
WE 2.8 Family Planning for Empowerment: Extent to which FP services or programs												
Don't know	0	0	0	0	0	0	0	0	0	0	1	8.3
WE 2.9 Political and Public Representation: Extent of implementation of programs												
WE 2.10 Violence: Extent of fight against violence against women and children an												
WE 2.11 Child Marriage: Extent of law enforcement to eliminate child marriage.												
WE 2.12 Female Genital Mutilation/Cutting: Extent of law enforcement to eliminat												
Don't know	1	**12.5**	0	0	0	0	3	**18.8**	0	0	0	0
WE 3.1 Advocates: Extent to which advocates for girls,Äô and women,Äôs empowerment e												
Don't know	1	**12.5**	0	0	0	0	0	0	0	0	0	0
WE 3.2. Girls and Women: Extent to which the advocacy efforts in this area encom												
Don't know	1	**12.5**	0	0	0	0	0	0	0	0	0	0

Note:

- For readability purpose, complete wording for questions/variables has been provided as Appendix 1.
- Only the percentages of respondents who responded “don’t know” to the respective question or sub-question was included in this table.
- Highlighted are values > 10%

WE 3.3 Information Dissemination: Extent to which the government undertakes suff												
Don't know	1	**12.5**	0	0	0	0	0	0	0	0	0	0
WE 3.4 Leveraging Partnerships: Extent to which the government leverages partner												
Don't know	1	**12.5**	0	0	0	0	0	0	0	0	0	0
WE 3.5 Employment: Extent to which stakeholders call for laws, policies and prog												
Don't know	1	**12.5**	0	0	0	0	0	0	0	0	0	0
WE 3.6 Education: Extent to which a priority of advocates is to promote the fulf												
Don't know	1	**12.5**	0	0	0	0	0	0	0	0	0	0
WE 3.7 Violence: Extent to which advocates successfully engage with the governme												
Don't know	1	**12.5**	0	0	0	0	1	6.2	0	0	0	0
WE 4.1 Strength of Observatory/Stakeholder/Technical Working Group for WE: Exten												
WE 4.1.1	1	**12.5**	0	0	0	0	0	0	0	0	0	0
WE 4.1.2	2	**28.6**	0	0	0	0	0	0	0	0	0	0
WE 4.1.3	1	**14.3**	0	0	0	0	0	0	0	0	0	0
WE 4.2 Women,Äôs Rights Research Strategy: Extent to which the national¬†WE plan an												
Don't know	1	**12.5**	0	0	1	**11.1**	0	0	0	0	1	8.3
WE 4.3 Data Gathering Partners:												
WE 4.3.1	1	**12.5**	0	0	0	0	0	0	0	0	1	8.3

Note:

- For readability purpose, complete wording for questions/variables has been provided as Appendix 1.
- Only the percentages of respondents who responded “don’t know” to the respective question or sub-question was included in this table.
- Highlighted are values > 10%

WE 4.3.2	1	**14.3**	0	0	0	0	0	0	0	0	1	8.3
WE 4.3.3	2	**25**	0	0	0	0	0	0	0	0	1	8.3
WE 4.4 Topical Data Gathering:												
WE 4.4.1	1	**12.5**	0	0	0	0	0	0	0	0	1	8.3
WE 4.4.2	1	**12.5**	0	0	0	0	0	0	0	0	1	8.3
WE 4.4.3	1	**12.5**	0	0	1	**11.1**	0	0	0	0	1	9.1
WE 4.4.4	1	**12.5**	0	0	0	0	0	0	0	0	1	10
WE 4.5 Quality/Coverage of Data:												
WE 4.5.1	1	**12.5**	1	10	0	0	0	0	0	0	1	9.1
WE 4.5.2	1	**12.5**	1	10	0	0	0	0	0	0	1	8.3
WE 4.5.3	2	**25**	1	10	0	0	0	0	0	0	1	8.3
WE 4.6 Quality Research Institutions: Extent to which the country supports and s												
Don't know	2	**25**	1	10	0	0	0	0	0	0	2	**16.7**
WE 4.7 Evaluation: Extent to which program statistics, surveys, and small studie												
Don't know	3	**37.5**	1	10	0	0	0	0	0	0	1	8.3
WE 4.8 Management,Äôs Use of Evaluation Findings: Extent to which local-level prog												
Don't know	3	**37.5**	0	0	0	0	0	0	0	0	1	8.3
WE 4.9 Ministerial Use of Evaluation Findings: Extent to which the Ministry admi												
Don't know	3	**37.5**	1	10	1	**11.1**	0	0	0	0	1	8.3
WE 4.10 Dissemination of Information to other Implementing Bodies: Extent to whi												

Note:

- For readability purpose, complete wording for questions/variables has been provided as Appendix 1.
- Only the percentages of respondents who responded “don’t know” to the respective question or sub-question was included in this table.
- Highlighted are values > 10%

Don't know	2	**25**	0	0	0	0	0	0	0	0	1	8.3
WE 5.1 CSO Actor Power: Extent to which WE civil society actors are in strong po												
Don't know	1	**14.3**	0	0	0	0	0	0	0	0	1	8.3
WE 5.2 Budget Analysis as a CSO Tool: Extent to which CSOs utilize budget analys												
Don't know	1	**14.3**	1	**11.1**	0	0	0	0	0	0	1	8.3
WE 5.3 Community-Based Service Delivery Support: Extent to which CSOs participat												
Don't know	1	**14.3**	0	0	0	0	0	0	0	0	1	8.3
WE 5.4 Human Rights and Quality of Implementation: Extent to which CSOs advocate												
Don't know	1	**14.3**	0	0	0	0	0	0	0	0	1	8.3
WE 5.5 Social and Behavior Change (SBC): Extent to which CSOs support SBC interv												
Don't know	1	**14.3**	0	0	0	0	0	0	0	0	1	8.3
WE 5.6 Youth: Extent to which CSOs promote gender empowerment through youth prog												
Don't know	1	**14.3**	0	0	1	**11.1**	0	0	0	0	1	8.3
WE 5.7 Programming for Men: Extent to which CSOs support WE policies, interventi												
Don't know	1	**14.3**	0	0	0	0	0	0	0	0	1	8.3
WE 5.8 Advocacy/Accountability: Extent to which CSOs support the training and ca												
Don't know	1	**14.3**	0	0	0	0	0	0	0	0	1	8.3

Note:

- For readability purpose, complete wording for questions/variables has been provided as Appendix 1.
- Only the percentages of respondents who responded “don’t know” to the respective question or sub-question was included in this table.
- Highlighted are values > 10%

WE 5.9 CSO-Led Assessment and Monitoring: Extent to which CSOs assess, monitor a												
Don't know	1	**14.3**	0	0	0	0	0	0	0	0	1	8.3
WE 5.10 Forming Partnerships between CSOs: Extent to which CSOs have formed nati												
Don't know	1	**14.3**	0	0	0	0	0	0	0	0	1	8.3
LM 1.1 Global Economic Integration: Extent to which the country aims to be integ												
LM 1.2 Regional Economic Integration: Extent to which the country aims to be int												
LM 1.3 Technology: Extent to which the government has invested in infrastructure												
LM 1.4 Government Support: Extent to which policies and plans are in place that												
Don't know	0	0	1	7.7	0	0	0	0	0	0	0	0
LM 1.5 Unemployment: Extent to which policies account for and address unemployme												
LM 1.6 Disadvantaged/Vulnerable Groups: Extent to which policies exist, and are												
LM 1.7 Climate Change and Environmental Sustainability: Extent to which the agri												
LM 1.8 Urbanization: ¬†Extent to which policies and planning anticipate and are p												
Don't know	1	**11.1**	0	0	0	0	0	0	0	0	0	0
LM 1.9 Service-Led Economy: Extent to which policies and framework are in place												

Note:

- For readability purpose, complete wording for questions/variables has been provided as Appendix 1.
- Only the percentages of respondents who responded "don't know" to the respective question or sub-question was included in this table.
- Highlighted are values > 10%

LM 1.10 Corporate Social Responsibility: Extent to which national legislation in												
Don't know	2	**22.2**	0	0	0	0	1	**11.1**	0	0	0	0
LM 1.11 Targeted Labor Market Investments: Extent to which the government has ta												
LM 1.12 Self-Employment: Extent to which policies support raising the returns to												
LM 1.13 Microcredit: Extent to which the government enables access to microcredi												
Don't know	0	0	0	0	0	0	1	**11.1**	0	0	0	0
LM 1.14 Savings: Extent to which the government supports the creation of accessi												
LM 1.15 Skill Acquisition: Extent to which policies exist, and are effectively i												
LM 2.1 Unemployment Reduction: Extent to which there are efforts toward developi												
Don't know	0	0	1	7.7	0	0	0	0	0	0	0	0
LM 2.2. Youth Unemployment: Extent to which labor market programs have effective												
Don't know	0	0	1	7.7	0	0	0	0	0	0	0	0
LM 2.3 Access to Microcredit: Extent to which national and regional funds have b												
Don't know	0	0	1	7.7	0	0	0	0	0	0	0	0
LM 2.4 Harnessing Youth Special Capacities: Extent to which programs and service												
Don't know	0	0	0	0	0	0	1	**11.1**	0	0	0	0

Note:

- For readability purpose, complete wording for questions/variables has been provided as Appendix 1.
- Only the percentages of respondents who responded "don't know" to the respective question or sub-question was included in this table.
- Highlighted are values > 10%

LM 2.5 Early Labor Opportunities: Extent to which programs and government legisl												
LM 2.6 Youth Entrance into Labor: Extent of the promotion of youth volunteer and												
LM 2.7 Entrepreneurial Ventures: Extent to which people have enhanced access to												
LM 2.8 Resources for Youth Entrepreneurship: Extent to which entrepreneurial ven												
Don't know	0	0	0	0	0	0	1	**11.1**	0	0	0	0
LM 2.9 Vocational Opportunities: Extent to which vocational training opportuniti												
LM 2.10 Education and Labor: Extent to which improvements have been made to impr												
LM 2.11 Women-Friendly Labor: Extent to which workplaces in the private and publ												
LM 2.12 Job Security:												
Don't know	0	0	0	0	1	**12.5**	0	0	0	0	0	0
LM 2.13 Minimum Wage: Extent to which a minimum wage is applied to the ‚Äòtypical‚Äô												
LM 2.14 Labor Market Programs: Extent to which labor market programs have effect												
Don't know	1	**11.1**	0	0	0	0	0	0	0	0	0	0
LM 2.15 Informality in the Labor Market: Extent to which the programs are implem												
LM 3.1 Gender Equality in the Labor Market: Extent to which groups and individua												

Note:

- For readability purpose, complete wording for questions/variables has been provided as Appendix 1.
- Only the percentages of respondents who responded “don’t know” to the respective question or sub-question was included in this table.
- Highlighted are values > 10%

LM 3.2 Youth Equality in the Labor Market: Extent to which individuals and group												
Don't know	1	**11.1**	0	0	0	0	0	0	0	0	0	0
LM 3.3 Minimum Wage Advocacy: Extent to which advocacy groups are successful in												
Don't know	0	0	1	7.7	0	0	0	0	0	0	0	0
LM 3.4 Government Advocacy: Extent to which members of the government advocates												
Don't know	0	0	1	7.7	0	0	0	0	0	0	0	0
LM 3.5 Protections: Extent to which advocacy exists for job protection, and soci												
Don't know	0	0	1	7.7	0	0	0	0	0	0	0	0
LM 4.1 Strength of Observatory/Stakeholder/Technical Working Group for the LM: E												
LM 4.1.1	1	**11.1**	2	**15.4**	0	0	0	0	0	0	0	0
LM 4.1.2	1	**11.1**	2	**15.4**	0	0	0	0	0	0	0	0
LM 4.1.3	1	**11.1**	3	**23.1**	0	0	0	0	0	0	0	0
LM 4.2 Labor Market Research Strategy: Extent to which the national documents in												
Don't know	0	0	3	**23.1**	0	0	0	0	0	0	0	0
LM 4.3 Labor Market Analysis Partners:												
LM 4.3.1	0	0	2	**15.4**	0	0	0	0	0	0	0	0
LM 4.3.2	0	0	2	**15.4**	0	0	0	0	0	0	0	0
LM 4.3.3	0	0	2	**15.4**	1	**12.5**	0	0	0	0	0	0
LM 4.4 Areas of Research in the Labor Market:												

Note:

- For readability purpose, complete wording for questions/variables has been provided as Appendix 1.
- Only the percentages of respondents who responded "don't know" to the respective question or sub-question was included in this table.
- Highlighted are values > 10%

LM 4.4.1	0	0	1	7.7	1	**12.5**	0	0	0	0	0	0
LM 4.4.2	0	0	1	7.7	1	**12.5**	0	0	0	0	0	0
LM 4.4.3	0	0	1	7.7	1	**12.5**	0	0	0	0	0	0
LM 4.4.4	0	0	2	**15.4**	1	**12.5**	0	0	0	0	0	0
LM 4.5 Quality/Coverage of Data:												
LM 4.5.1	0	0	2	**15.4**	1	**12.5**	0	0	0	0	0	0
LM 4.5.2	0	0	2	**15.4**	1	**12.5**	0	0	0	0	0	0
LM 4.5.3	0	0	2	**15.4**	1	**12.5**	0	0	0	0	0	0
LM 4.6 Record-Keeping: Extent to which systems for labor market recordkeeping, r												
Don't know	0	0	2	**15.4**	1	**12.5**	0	0	0	0	0	0
LM 4.7 Quality Research Institutions: Extent to which the country has the capaci												
Don't know	0	0	2	**15.4**	1	**12.5**	0	0	0	0	0	0
LM 4.8 Evaluation: Extent to which program statistics, surveys, and small studie												
Don't know	0	0	1	7.7	1	**12.5**	0	0	0	0	0	0
LM 4.9 Management,Äôs Use of Evaluation Findings: Extent to which local-level prog												
Don't know	0	0	1	7.7	1	**12.5**	0	0	0	0	0	0
LM 4.10 Ministerial Use of Evaluation Findings: Extent to which the Ministry adm												
Don't know	0	0	2	**15.4**	1	**12.5**	0	0	0	0	0	0
LM 4.11 Dissemination of Information to Other Implementing Bodies: Extent to whi												

Note:

- For readability purpose, complete wording for questions/variables has been provided as Appendix 1.
- Only the percentages of respondents who responded “don’t know” to the respective question or sub-question was included in this table.
- Highlighted are values > 10%

Don't know	0	0	1	7.7	1	**12.5**	0	0	0	0	0	0
LM 5.1 CSO Actor Power: Extent to which CSO actors are in strong positions to in												
Don't know	1	**11.1**	1	7.7	0	0	0	0	0	0	0	0
LM 5.2 Budget Analysis as a CSO Tool: Extent to which CSOs utilize budget analys												
Don't know	2	**22.2**	2	**15.4**	0	0	1	**11.1**	0	0	0	0
LM 5.3 Community-Based Service Delivery Support: Extent to which CSOs participat												
Don't know	2	**22.2**	1	7.7	0	0	1	**11.1**	0	0	0	0
LM 5.4 Diversification of Job Opportunities: Extent to which CSOs and the privat												
Don't know	2	**22.2**	1	7.7	0	0	1	**11.1**	0	0	0	0
LM 5.5 Human Rights and Quality of Implementation: Extent to which CSOs support												
Don't know	3	**33.3**	1	7.7	0	0	1	**11.1**	0	0	0	0
LM 5.6 Youth: Extent to which CSOs support labor market policies, interventions												
Don't know	2	**22.2**	1	7.7	0	0	0	0	0	0	0	0
LM 5.7 Programming for Women: Extent to which CSOs support labor market policies												
Don't know	2	**22.2**	1	7.7	0	0	0	0	0	0	0	0
LM 5.8 Advocacy/Accountability: Extent to which CSOs support the training and ca												
Don't know	2	**22.2**	1	7.7	0	0	1	**11.1**	0	0	0	0

Note:

- For readability purpose, complete wording for questions/variables has been provided as Appendix 1.
- Only the percentages of respondents who responded "don't know" to the respective question or sub-question was included in this table.
- Highlighted are values > 10%

LM 5.9 CSO-Led Assessment and Monitoring: Extent to which CSOs assess, monitor a												
Don't know	2	**22.2**	1	7.7	0	0	1	**11.1**	0	0	0	0
LM 5.10 Forming Partnerships between CSOs: Extent to which the private sector ha												
Don't know	2	**22.2**	1	7.7	0	0	1	**11.1**	0	0	0	0
GEI 1.1 Democratic Values: Extent to which the government has ratified, domestic												
Don't know	1	**11.1**	0	0	0	0	1	**16.7**	0	0	0	0
GEI 1.2 Monitoring Democratic Values: Extent to which the government has created												
Don't know	1	**11.1**	0	0	1	8.3	1	**16.7**	0	0	1	**12.5**
GEI 1.3 Affirmation of Youth Democratic Participation: Extent to which the gover												
Don't know	0	0	0	0	0	0	1	**16.7**	0	0	0	0
GEI 1.4. Removing Legal Youth Barriers: Extent to which the government has prosc												
Don't know	0	0	0	0	0	0	1	**16.7**	0	0	0	0
GEI 1.5 Youth Policy: Extent to which the youth policy takes into account the la												
Don't know	0	0	0	0	0	0	1	**16.7**	0	0	0	0
GEI 1.6 Affirmation of Democratic Participation/Revision of Customary Law: Exten												
Don't know	0	0	0	0	1	8.3	1	**16.7**	0	0	0	0
GEI 1.7 Youth Policymaking Participation: Extent to which the government ensures												

Note:

- For readability purpose, complete wording for questions/variables has been provided as Appendix 1.
- Only the percentages of respondents who responded “don’t know” to the respective question or sub-question was included in this table.
- Highlighted are values > 10%

Don't know	0	0	1	**16.7**	1	8.3	1	**16.7**	0	0	0	0
GEI 1.8 Rule of Law: Extent to which the rule of law is formally, legally establ												
Don't know	1	**11.1**	0	0	1	8.3	1	**16.7**	0	0	0	0
GEI 1.9 Transparency and Accountability: Extent to which national legislation ma												
Don't know	0	0	0	0	0	0	1	**16.7**	0	0	0	0
Don't know	0	0	0	0	1	8.3	1	**16.7**	0	0	0	0
GEI 1.10 National Security: Extent to which the country/government is involved i												
Don't know	1	**11.1**	1	**16.7**	0	0	1	**16.7**	0	0	2	**25**
Don't know	0	0	0	0	0	0	1	**16.7**	0	0	2	**25**
Don't know	1	**11.1**	0	0	0	0	1	**16.7**	0	0	3	**37.5**
GEI 1.11 Rights: Extent to which the constitution affirms certain rights: freedo												
Don't know	0	0	0	0	0	0	1	**16.7**	0	0	0	0
GEI 1.12 Education and Democratic Values: Extent to which education policies, st												
Don't know	0	0	0	0	0	0	1	**16.7**	0	0	0	0
GEI 1.13 Affirmation of Safe Learning Environments: Extent to which education po												
Don't know	0	0	0	0	0	0	1	**16.7**	0	0	0	0
GEI 2.1 Strengthening Youth Activities/Institutions: Extent to which efforts are												
Don't know	1	**12.5**	0	0	0	0	0	0	0	0	0	0

Note:

- For readability purpose, complete wording for questions/variables has been provided as Appendix 1.
- Only the percentages of respondents who responded “don’t know” to the respective question or sub-question was included in this table.
- Highlighted are values > 10%

GEI 2.2 Appointment of Youth Leader: Whether and to what extent there has been a												
Don't know	1	**12.5**	0	0	0	0	0	0	0	0	1	**12.5**
GEI 2.3 Youth Ideals and Aspirations: Extent to which programming is designed to												
Don't know	1	**12.5**	0	0	0	0	0	0	0	0	0	0
GEI 2.4 Youth Services: Extent to which programming is designed to address youth												
Don't know	1	**12.5**	0	0	1	8.3	0	0	0	0	0	0
GEI 2.5 Youth Involvement in National Initiatives: Extent to which there are eff												
Don't know	1	**12.5**	0	0	1	8.3	0	0	0	0	0	0
GEI 2.6 Personal Safety: Extent to which individual safety is provided for throu												
GEI 2.7 Participation: Extent to which government functions involve the particip												
GEI 2.8 Gender: Extent to which women participate in:												
GEI 2.8.1	2	**25**	0	0	0	0	0	0	0	0	0	0
GEI 2.8.2	0	0	1	**20**	0	0	0	0	0	0	0	0
GEI 2.8.3	0	0	0	0	0	0	0	0	0	0	2	**25**
GEI 2.9 Promotion of Equality: Extent to which gender equality in all aspects of												
Don't know	0	0	0	0	1	8.3	0	0	0	0	0	0
GEI 2.10 Sustainable Economic Opportunity:												
GEI 2.11 Human Development:												

Note:

- For readability purpose, complete wording for questions/variables has been provided as Appendix 1.
- Only the percentages of respondents who responded "don't know" to the respective question or sub-question was included in this table.
- Highlighted are values > 10%

GEI 2.11.1	0	0	0	0	1	8.3	0	0	0	0	0	0
GEI 2.11.2	0	0	0	0	1	8.3	0	0	0	0	0	0
GEI 2.11.3	0	0	0	0	1	8.3	0	0	0	0	0	0
GEI 2.11.4	0	0	0	0	1	8.3	0	0	0	0	0	0
GEI 3.1 Youth Empowerment: Extent to which youth empowerment has been enhanced,												
Don't know	0	0	0	0	1	9.1	0	0	0	0	0	0
GEI 3.2 Government Advocacy: Extent to which members of the government advocates												
Don't know	0	0	1	**20**	1	9.1	0	0	0	0	0	0
GEI 3.3 Non-governmental Advocacy: Extent to which non-governmental actors advoc												
Don't know	0	0	1	**20**	2	**18.2**	0	0	0	0	0	0
GEI 3.4 Civil Society Organizations (CSOs): Extent to which CSOs specifically op												
Don't know	0	0	0	0	1	9.1	0	0	0	0	0	0
GEI 3.5 Advocacy Voice: Extent to which advocates have a voice in government dis									**Tanzania**	**Tanzania**	**Total**	**Total**
Don't know	0	0	0	0	1	9.1	0	0	0	0	1	2.7
GEI 3.6 Voice of Instrumental Sectors of the DD: Extent to which the government												
GEI 3.6.1	0	0	0	0	1	9.1	0	0	0	0	0	0
GEI 3.6.2	0	0	0	0	1	9.1	0	0	0	0	0	0
GEI 3.6.3	0	0	0	0	1	9.1	0	0	0	0	0	0
GEI 3.6.4	0	0	0	0	1	9.1	0	0	0	0	0	0

Note:

- For readability purpose, complete wording for questions/variables has been provided as Appendix 1.
- Only the percentages of respondents who responded “don’t know” to the respective question or sub-question was included in this table.
- Highlighted are values > 10%

GEI 3.6.5	0	0	0	0	2	**18.2**	0	0	0	0	0	0
GEI 3.6.6	0	0	0	0	1	9.1	0	0	0	0	0	0
GEI 4.1 Strength of Observatory/Stakeholder/Technical Working Group for GEI: Ext												
GEI 4.1.1	1	**12.5**	0	0	1	9.1	0	0	0	0	0	0
GEI 4.1.2	2	**25**	0	0	1	9.1	0	0	0	0	0	0
GEI 4.1.3	2	**25**	0	0	1	9.1	0	0	0	0	0	0
GEI 4.2¬†Education/Research Strategy: Extent to which national planning documents												
Don't know	0	0	0	0	2	**18.2**	0	0	0	0	1	**14.3**
GEI 4.3 Governance, Financial and Development Data Gathering Partners:												
GEI 4.3.1	0	0	0	0	1	9.1	0	0	0	0	0	0
GEI 4.3.2	0	0	0	0	2	**18.2**	0	0	0	0	0	0
GEI 4.3.3	0	0	1	**20**	1	9.1	0	0	0	0	0	0
GEI 4.4 Governance, Financial and Development Topical Data Gathering:												
GEI 4.4.1	1	**12.5**	0	0	1	9.1	0	0	0	0	0	0
GEI 4.4.2	0	0	0	0	1	9.1	0	0	0	0	2	**28.6**
GEI 4.4.3	0	0	0	0	2	**18.2**	0	0	0	0	0	0
GEI 4.4.4	0	0	0	0	1	9.1	0	0	0	0	0	0
GEI 4.5 Quality/Coverage of Data:												
GEI 4.5.1	0	0	0	0	2	**18.2**	0	0	0	0	1	**14.3**
GEI 4.5.2	0	0	0	0	2	**18.2**	0	0	0	0	1	**14.3**

Note:

- For readability purpose, complete wording for questions/variables has been provided as Appendix 1.
- Only the percentages of respondents who responded “don’t know” to the respective question or sub-question was included in this table.
- Highlighted are values > 10%

GEI 4.5.3	0	0	0	0	2	**18.2**	0	0	0	0	0	0
GEI 4.5.4	0	0	0	0	2	**18.2**	0	0	2	**28.6**	4	**10.8**
GEI 4.6 Quality Research Institutions: Extent to which the country has the capac												
Don't know	0	0	0	0	1	9.1	0	0	0	0	0	0
GEI 4.7 Evaluation: Extent to which program statistics, surveys, and small studi												
Don't know	0	0	0	0	1	9.1	0	0	0	0	0	0
GEI 4.8 Management‚Äôs Use of Evaluation Findings: Extent to which local-level pro												
Don't know	0	0	0	0	1	9.1	0	0	0	0	0	0
GEI 4.10 Dissemination of Information to Other Implementing Bodies: Extent to wh												
Don't know	0	0	0	0	1	9.1	0	0	0	0	0	0
GEI 5.1 CSO Actor Power: Extent to which CSO actors are in strong positions to p												
Don't know	0	0	0	0	1	9.1	1	**16.7**	0	0	0	0
GEI 5.2 Budget Analysis as a CSO Tool: Extent to which CSOs utilize budget analy												
Don't know	3	**42.9**	0	0	1	9.1	1	**16.7**	0	0	0	0
GEI 5.3 Community-Based Service Delivery Support: Extent to which CSOs participa												
Don't know	0	0	0	0	1	9.1	1	**16.7**	0	0	0	0
GEI 5.4 Human Rights and Quality of Implementation: Extent to which CSOs advocat												
Don't know	1	**14.3**	0	0	1	9.1	1	**16.7**	0	0	0	0

Note:

- For readability purpose, complete wording for questions/variables has been provided as Appendix 1.
- Only the percentages of respondents who responded “don’t know” to the respective question or sub-question was included in this table.
- Highlighted are values > 10%

GEI 5.5 Youth: Extent to which CSOs support youth to participate in education, t												
Don't know	1	**14.3**	0	0	1	9.1	1	**16.7**	0	0	0	0
GEI 5.6 Programs for Women: Extent to which CSOs ensure GEI policies support wom												
Don't know	0	0	0	0	1	9.1	1	**16.7**	0	0	0	0
GEI 5.7 Advocacy/Accountability												
Don't know	0	0	0	0	2	**18.2**	1	**16.7**	0	0	0	0
Don't know	0	0	0	0	2	**18.2**	1	**16.7**	0	0	0	0
GEI 5.8 CSO-Led Assessment and Monitoring: Extent to which CSOs assess, monitor												
Don't know	0	0	0	0	1	9.1	1	**16.7**	0	0	0	0
GEI 5.9 Forming Partnerships between CSOs: Extent to which CSOs have formed nati												
Don't know	0	0	0	0	1	9.1	1	**16.7**	0	0	0	0

Note:

- For readability purpose, complete wording for questions/variables has been provided as Appendix 1.
- Only the percentages of respondents who responded “don’t know” to the respective question or sub-question was included in this table.
- Highlighted are values > 10%
