# Supplementary material for: Development of the Demographic Dividend Effort Index, a novel tool to measure existing efforts to create a favourable environment to harness a demographic dividend: results from an experts’ survey from six sub-Saharan African countries
Source: BMJ Open. 2023 Mar 21;13(3):e059937. doi: 10.1136/bmjopen-2021-059937 (PMC10040031; doi:10.1136/bmjopen-2021-059937)
Supplement: Author's manuscript [file bmjopen-2021-059937.draft_revisions.pdf]

BMJ Open is committed to open peer review. As part of this commitment we make the peer review history of every article we publish publicly available.

When an article is published we post the peer reviewers' comments and the authors' responses online. We also post the versions of the paper that were used during peer review. These are the versions that the peer review comments apply to.

The versions of the paper that follow are the versions that were submitted during the peer review process. They are not the versions of record or the final published versions. They should not be cited or distributed as the published version of this manuscript.

BMJ Open is an open access journal and the full, final, typeset and author-corrected version of record of the manuscript is available on our site with no access controls, subscription charges or pay-per-view fees (http://bmjopen.bmj.com).

If you have any questions on BMJ Open's open peer review process please email info.bmjopen@bmj.com

BMJ Open

The Demographic Dividend Effort index: A novel measure to inform health and development policy and programs in Sub-Saharan Africa.

Journal:	*BMJ Open*
Manuscript ID	bmjopen-2021-059937
Article Type:	Original research
Date Submitted by the Author:	17-Dec-2021

Complete List of Authors:	Rusatira, Jean Christophe; Johns Hopkins University Bloomberg School of Public Health, PHFR<br>Cardona, Carolina; Johns Hopkins University Bloomberg School of Public Health, Population Family and Reproductive Health<br>Martinez, Michelle; Johns Hopkins University Bloomberg School of Public Health, Population, Family and Reproductive Health<br>Ahmed, Saifuddin; Johns Hopkins University Bloomberg School of Public Health, Population, Family And Reproductive Health<br>Rimon, Jose; Johns Hopkins University Bloomberg School of Public Health, Population, Family and Reproductive Health

Keywords:	PUBLIC HEALTH, QUALITATIVE RESEARCH, HEALTH ECONOMICS

SCHOLARONE™ Manuscripts

I, the Submitting Author has the right to grant and does grant on behalf of all authors of the Work (as defined in the below author licence), an exclusive licence and/or a non-exclusive licence for contributions from authors who are: i) UK Crown employees; ii) where BMJ has agreed a CC-BY licence shall apply, and/or iii) in accordance with the terms applicable for US Federal Government officers or employees acting as part of their official duties; on a worldwide, perpetual, irrevocable, royalty-free basis to BMJ Publishing Group Ltd ("BMJ") its licensees and where the relevant Journal is co-owned by BMJ to the co-owners of the Journal, to publish the Work in this journal and any other BMJ products and to exploit all rights, as set out in our licence.

The Submitting Author accepts and understands that any supply made under these terms is made by BMJ to the Submitting Author unless you are acting as an employee on behalf of your employer or a postgraduate student of an affiliated institution which is paying any applicable article publishing charge ("APC") for Open Access articles. Where the Submitting Author wishes to make the Work available on an Open Access basis (and intends to pay the relevant APC), the terms of reuse of such Open Access shall be governed by a Creative Commons licence – details of these licences and which Creative Commons licence will apply to this Work are set out in our licence referred to above.

Other than as permitted in any relevant BMJ Author's Self Archiving Policies, I confirm this Work has not been accepted for publication elsewhere, is not being considered for publication elsewhere and does not duplicate material already published. I confirm all authors consent to publication of this Work and authorise the granting of this licence.

For peer review only

The Demographic Dividend Effort index: A novel measure to inform health and development policy and programs in Sub-Saharan Africa.

Authors
Jean Christophe Rusatira*, Carolina Cardona*, Michelle Martinez*, Saifuddin Ahmed*, Jose G. Rimon*.

* Department of Population, Family and Reproductive Health, Johns Hopkins Bloomberg School of Public Health

Corresponding Author
E-mail address: jcrusatira@jhu.edu
Department of Population, Family and Reproductive Health
Johns Hopkins Bloomberg School of Public Health
615 N. Wolfe St., Baltimore, MD 21030, USA

Abstract
Introduction: Over the last decade, the African Union member states have embarked on the demographic dividend (DD) development agenda and have developed their roadmaps and strategic plans. However, there is no standard tool to assess progress, document successes and gaps. This study aimed at developing a tool to measure the extent of national efforts in policies, services, research, and programs implemented to cultivate, realize, and harness the benefits of a potential DD in six Sub-Saharan African countries.

Methods: Online survey was administered using Survey Monkey platform. The survey covered six key sectors Governance and economic institutions, Family planning, Maternal and child health, Education, Women empowerment, and Labor market. Each sector-specific questionnaire was structured around five practice domains: services and programs, policymaking, advocacy, research, and civil society. Knowledgeable informants were identified by the study local partners and a total of 440 experts participated: Ethiopia (73), Kenya (69), Nigeria (67), Rwanda (54), Senegal (81), and Tanzania (96). Each item was scored from 1 to 10 and overall mean scores were generated with equal weights for each item across domains. Their corresponding 95% confidence intervals (CI) were calculated with a t-distribution.

Results: The results revealed overall scores that ranged between 5.4 (95% CI: 5.1-5.8) in Ethiopia and 7.7 (95% CI: 7.5-7.9) in Rwanda. The disaggregated scores by sector revealed low scores in most countries in labor market and women empowerment. Across sectors, domain and item-specific scores revealed several heterogeneities despite average scores around the mid-point.

Conclusion: The Demographic Dividend Effort Index scores highlight important gaps in key health and development sectors and identifies opportunities for improving health and development in different Sub-Saharan Africa countries. In addition to serving as a self-evaluation tool for local actors, the tool can complement the existing quantitative tools such as the global gender gap and the human capital index.

For peer review only - http://bmjopen.bmj.com/site/about/guidelines.xhtml

Strengths and limitations of the study

- The study was designed to develop the first-ever index to measure the extent of national efforts in key health and development sectors to cultivate, realize, and harness the benefits of a potential demographic dividend in different Sub-Saharan African countries.
- The index scores reveal similarities and differences across health and development sectors, which present opportunities for mutual learning between sectors within and between countries.
- Measuring current efforts in health and development domains and sectors provide unique measures of national progress in policies, services, advocacy, research, and programs that can be used to monitor, evaluate, and strengthen strategic health and development interventions.
- The Demographic Dividend Effort Index provides an opportunity for self-evaluation for local policymakers and stakeholders and complements the existing quantitative measures such as the Global Gender Gap and the Human Capital Index.
- The study relied on expert opinions working in different sectors in respective countries which may be prone to bias and may not be comparable across countries.

For peer review only

For peer review only - http://bmjopen.bmj.com/site/about/guidelines.xhtml

Background

The reduction of births and infant deaths during the third stage of the demographic transition produces changes in the age structure of the population. These changes present countries an opportunity to experience a demographic dividend (DD) characterized by economic growth, sustainable development, and social change.[1-3] These demographic changes can spur economic growth by increasing the number of working-age individuals compared to the number of young dependents. However, favorable policies and investments are required for countries to harness the DD opportunity presented by such population age structure changes.

Across the Sub-Saharan African (SSA) region, more than three in four countries are still in the early stages of their demographic transition. They are projected not to reach the peak of the transition, where the DD occurs, until 2050.[4] Although this transition started more than 50 years ago with declines in fertility levels from an average of 6.8 children per woman to an average of 4.8 children by the mid-2010s, it has been slower compared to other continents.[1] Currently, the working-age individuals represent about 60% of the population, and this proportion is expected to keep increasing in the coming few decades.[5] These shifting population dynamics present a unique opportunity for SSA countries to grow their economies and break the cycle of poverty and poor health that has become chronic in the majority of these countries.

Previous literature has identified that, in Asian countries, the DD was responsible for around one-third of their economic growth between the 1960s and 1990s.[6 7] These experiences have sparked an interest among African countries. Over the last decade, countries in the region have embarked on the DD development agenda. The majority of the African Union (AU) member states have developed their DD roadmaps and strategic plans.[8] However, there is no standard tool to measure the extent of national efforts in policies, services, research, advocacy, and programs implemented to cultivate, realize, and harness the benefits of a potential DD in different SSA countries.

The AU and member states recognize that the DD is a key component to achieving sustainable socio-economic growth. In 2017, the AU established the theme of the year as "Harnessing the Demographic Dividend Through Investments in Youth". This theme generated momentum for the DD across the continent, particularly after the development of the AU DD roadmap. The roadmap identifies labor, education, health, governance, and youth empowerment as five fundamental pillars of the DD.[8] Cardona and colleagues further expanded these pillars based on their findings from a systematic literature review that highlighted six key sectors for cultivating and capitalizing on the DD potential in SSA.[9] These sectors are good governance and economic institutions (GGEI), family planning (FP), maternal and child health (MCH), education (ED), women empowerment (WE), and labor market (LM).

A central feature to enable a favorable policy environment, and attain a DD, is good governance and strong economic institutions. Governments have the power to attract foreign direct investments to create jobs and contribute to the economy. Strong financial institutions can promote a culture of saving, supported by favorable interest rates to fuel development. Bloom and colleagues have previously documented the correlation between economic stability, institutional quality and economic growth and have evidenced that unstable countries miss out on their DD opportunities.[1 10 11]

Political and economic stability create an opportunity for a more prosperous community through successful health and development programs. Successful FP programs have been proven to improve

For peer review only

women's participation in the workforce and have been identified as key interventions to reduce maternal and child mortality due to their impact on birth spacing and fertility levels.[12-15] The reduction of infant mortality is also intertwined with the reduction in fertility and directly associated with the reduction in maternal mortality. Conley and colleagues evidenced that a reduction of 0.5-0.9 points in the total fertility rate (TFR) was associated with a fall of 50 deaths per 1,000 live births in child mortality rate.[16] This association is concordant with Becker's theory about the quantity-quality trade-off of children, which predicts that parental investments per child increase as fertility levels decline.[17] As families become smaller, more resources are freed to support children's development and the household as a whole.[14 18]

Related to the quantity-quality trade-off, investments in human capital per child increase as fertility lowers, which can accelerate the economy. Ahmed and colleagues found a potential additional 22.4 percentage points increase in GDP per capita in SSA from an increase of just 13% in education attainment between 2011-2030 which eventually increases the availability of skilled workers[19]. Equitable access to quality education for boys and girls is also necessary for countries to generate skilled workers that can later support their economies. Moreover, the education of girls plays an important role in empowering women, who can later enter the labor force and spark socio-economic growth in SSA countries[20].

Africa's young population presents an opportunity for SSA countries to transform their economies. However, to seize this opportunity, countries may require different strategies depending on their demographic transition stage. On the one hand, countries that are still in the initial stage of their demographic transition also referred to as "pre-dividend" still need to accelerate their demographic transition and develop the human capital of the growing youth population.[18 21] These countries are characterized by a total fertility rate (TFR) of 4 or higher children per woman and a projected growing working-age population by 2030.[18 21] On the other hand, countries that are in the middle of their demographic transition, also referred to as "early-dividend", require smart investments in human capital development so that they can fill various job opportunities in the labor market.

These countries are characterized for having a TFR of less than 4 children per woman and a projected growing working-age population by 2030.[18 21] For the socio-economic growth to happen, multisectoral efforts are required and countries need to generate jobs faster than their population growth, explore innovative strategies to transition young graduates to formal jobs, and facilitate job creation in the private sector.[22]

This study aimed at developing an effort assessment index as a standard measure to quantify the nature and strength of in-country DD efforts in the key sectors identified as necessary to harness a DD. This index is meant to be used by national and global actors in the development sector working in five development domains namely policy, services and programs, advocacy, research, and civil society.

Methodology

Study material

Data were collected through an online survey that measured the efforts that countries are devoting to maximize their potential to reap the benefits of a DD. The survey was administered through an internet-based modality using the SurveyMonkey platform in six countries: Kenya, Senegal, Nigeria, Rwanda, Tanzania, and Ethiopia. These countries were selected because of where they stand in the demographic transition and convenience to successfully implement the study. The survey(s) was administered in two

For peer review only - http://bmjopen.bmj.com/site/about/guidelines.xhtml

waves to accommodate Coronavirus Disease 2019 (COVID-19) lockdowns in different countries. The first wave occurred from August to November 2020, and the second wave that covered Senegal alone took place from March to May 2021. The main language of the questionnaire was English (Appendix 1), and a French version (Appendix 2) was created for Senegal by a certified translator.

The questionnaires were developed for each of the six sectors recommended by Cardona and colleagues: FP, MCH, ED, WE, LM, GGEI.[9] All questionnaires were structured around five domains: 1) policy; 2) services and programs; 3) advocacy; 4) research; and 5) civil society. These domains were selected based on prior similar effort indices implemented in various health and development areas[23-26]. The Demographic Dividend Effort Index (DDEI) questionnaire was composed of 490 items across the six sectors (FP=112, MCH= 101, ED=61, WE=71, LM=68, GGEI=77). Where available, the development of each sector-specific questionnaire integrated validated questions from existing effort index. For instance, the Family Planning Effort Index (FPEI) questionnaire[25] was integrated in the FP questionnaire. Similarly, specific questions from the Maternal and Neonatal Program Index (MNPI)[24], the Rwanda Governance Scorecard[27] and the AIDS Program Effort Index (API)[23] were integrated in different sector-specific questionnaires. Additional questions were drafted based on DD-favorable interventions for respective sectors as summarized in the wheel of prosperity by Cardona et al.[9] During the data collection, participants only ever received single surveys that pertained to their sector of expertise and never the entirety of the aggregate survey. Each item was scored on a scale of 1 to 10, from weaker (1) to stronger (10) perceived level of effort or strength.

The DDEI questionnaire was pilot-tested among 27 experts from Nigeria, Ethiopia, Tanzania, Senegal, Kenya and Rwanda in June-August 2020. This pilot application allowed local experts to provide their feedback and inputs to the questionnaire which were integrated in the final revision of the questionnaire. The final questionnaire was shared with local project partners for review and approval before administering to the national level across all six countries. Each sector-specific questionnaire included items that assessed the presence and strength of policies, services and programs, advocacy efforts, research, and the involvement of civil society organizations in that sector.

Study participants

The in-country partners identified the participants who were knowledgeable in respective sectors and countries. In-country partners included the Centre d'Excellence Régional en Economie Générationnelle in Senegal, the Center for Communication Programs in Nigeria, the National Council for Population and Development in Kenya, the Tanzania Communication and Development Center, the Ethiopian Academy of Sciences, and the Centre for Integrated Development Research and Action in Rwanda. Participants were considered for the survey if they were regarded to have demonstrated expertise in at least one of the six sectors by the in-country partner. Survey participants were accessed through ongoing partnerships with in-country technical working groups via the Communities of Practice or Technical Working Groups that operate within each country.

As with other similar indices, sampling was necessarily purposive given the need to identify knowledgeable respondents. The design entailed the selection of 10 respondents for each sector. Whenever possible, diversity across the five domains of expertise was prioritized. As such, to reach 10 respondents per sector, each of the six sectors were designed to have two respondents with sector expertise in one of the five domains. These were exclusive classifications such that respondents could only be counted towards fulfillment of one sector-specific questionnaire. Regardless of their domain of expertise,

For peer review only - http://bmjopen.bmj.com/site/about/guidelines.xhtml

participants were expected to respond to the entirety of the sector-specific questionnaire. Overall, the sample size goal was 60 respondents per country.

This expected sample size was on the higher end of sample sizes for similar indices such as the Human Resources Effort Index (N = 16-28 per country), the FPEI (N = 1-12 per country), the API (N = 20-25 per country), and the MNPI (N=10-25).[23-26] These indices similarly surveyed across various sectors and, sometimes, domains of expertise. However, each were focused on one specific topic, while the DD required a multisectoral sample frame. As this study aimed for 60 respondents per country, considering an expected response rate similar to that of the FPEI of 49%, which was the highest among the other comparable indices, this study ultimately aimed for at least 30 respondents per country.

Patient and Public involvement

No patients or members of the public were involved in the research design, data analysis, or dissemination of the findings. Local researchers and policymakers contributed to the design of the questionnaire, the implementation of the survey and were involved in the interpretation of the results.

Analysis

The characteristics of the participants are presented by sector and domain of expertise at the country level (Table 1). Then, mean scores are computed for ease of interpretation and comparison across sectors per country (Figure 1). Their corresponding 95% confidence intervals (CI) were calculated with a *t*-distribution because the sample size was relatively small. The overall country scores were computed (Table 2) and mean scores per sector were disaggregated by domain of expertise for ease of interpretability and policy relevance (Table 3). Finally, the results from this analysis were shared with in-country partners, for review, policy translation and dissemination. The partners from respective countries conducted a series of multisectoral meetings and workshops, physical and virtual, to generate policy recommendations based on the results from the analysis.

Results

Respondents

The total sample consisted of 440 respondents distributed as follows: Ethiopia (73), Kenya (69), Nigeria (67), Rwanda (54), Senegal (81), and Tanzania (96). The number of respondents, domain of expertise, and sectors differ across countries. In all countries, except Tanzania, the majority of respondents were from the public sector and in services or programs domain. The average completion rate was 82% and ranged from 78% in Tanzania to 92% in Rwanda. The intended number of participants per sector was achieved in all six countries.

Overall scores

Overall, the average scores were centered around the mid-point of 5 across sectors and domains. The scores were 5.4 (95% CI: 5.1-5.8) in Ethiopia, 5.8 (95% CI: 5.5-6.1) in Kenya, 5.5 (95% CI: 5.1-5.9) in Nigeria, 7.7 (95% CI: 7.5-7.9) in Rwanda, 6.3 (95% CI: 5.9-6.7) in Senegal, and 6.1 (95% CI: 5.8-6.4) in Tanzania.

For peer review only - http://bmjopen.bmj.com/site/about/guidelines.xhtml

Table 1: Sample Characteristics

	Ethiopia	Kenya	Nigeria	Rwanda	Senegal	Tanzania
	% (Obs.)	% (Obs.)	% (Obs.)	% (Obs.)	% (Obs.)	% (Obs.)
**Sector**						
Family Planning	23.3 (17)	24.6 (17)	26.9 (18)	13.0 (7)	8.6 (7)	39.6 (38)
Maternal and Child Health	20.5 (15)	24.6 (17)	11.9 (8)	16.7 (9)	19.8 (16)	19.8 (19)
Education	20.5 (15)	7.2 (5)	17.9 (12)	13.0 (7)	13.6 (11)	10.4 (10)
Women's Empowerment	11.0 (8)	14.5 (10)	13.4 (9)	29.6 (16)	21.0 (17)	13.5 (13)
Labor Market	12.3 (9)	18.8 (13)	11.9 (8)	16.7 (9)	14.8 (12)	8.3 (8)
Governance and Economic Institutions	12.3 (9)	10.1 (7)	17.9 (12)	11.1 (6)	22.2 (18)	8.3 (8)
**Organization**						
Public Sector	21.9 (16)	68.1 (47)	56.7 (38)	83.3 (45)	63.6 (49)	13.5 (13)
Private Sector	5.5 (4)	0.0 (0)	3.0 (2)	13.0 (7)	10.4 (8)	9.4 (9)
Non-governmental organization	27.4 (20)	27.5 (19)	28.4 (19)	1.9 (1)	20.8 (16)	66.7 (64)
University/research institute	41.1 (30)	1.4 (1)	3.0 (2)	0.0 (0)	3.9 (3)	2.1 (2)
Other (please specify)	4.1 (3)	2.9 (2)	9.0 (6)	1.9 (1)	1.3 (1)	8.3 (8)
**Area of Expertise**						
Policy/policymaking	13.7 (10)	20.3 (14)	26.9 (18)	14.8 (8)	17.3 (14)	7.3 (7)
Services or programs	21.9 (16)	58.0 (40)	37.3 (25)	18.5 (10)	66.7 (54)	52.1 (50)
Advocacy	8.2 (6)	11.6 (8)	13.4 (9)	14.8 (8)	8.6 (7)	25.0 (24)
Research	53.4 (39)	5.8 (4)	17.9 (12)	46.3 (25)	2.5 (2)	10.4 (10)
Civil Society	2.7 (2)	4.3 (3)	4.5 (3)	5.6 (3)	4.9 (4)	5.2 (5)
Obs.	73	69	67	54	81	96

Table 2. National overall and sector scores

Sector	Ethiopia	Kenya	Nigeria	Rwanda	Senegal	Tanzania
	Mean (95% CI) (Obs.)					
**Family Planning**	5.7 (5.3-6.1) (17)	5.9 (5.4-6.5) (17)	5.9 (5.1-6.7) (18)	6.4 (6.1-6.6) (7)	6.5 (5.1-7.8) (7)	6.0 (5.6-6.3) (38)
**Maternal and Child Health**	5.9 (4.7-7.2) (15)	6.2 (5.5-6.9) (18)	5.1 (3.8-6.4) (8)	7.4 (7.1-7.6) (9)	7.1 (6.5-7.7) (16)	6.8 (6.1-7.4) (19)
**Education**	5.3 (4.6-6.1) (15)	5.6 (3.8-7.4) (5)	5.9 (5.1-6.8) (12)	8.2 (7.3-9.1) (7)	5.9 (4.5-7.4) (11)	5.2 (4.1-6.4) (10)
**Women's Empowerment**	4.8 (3.4-6.1) (8)	5.1 (4.2-5.9) (10)	5.1 (4.1-6.1) (9)	8.3 (8.1-8.6) (16)	6.4 (5.6-7.3) (17)	6.9 (6.1-7.8) (13)
**Labor Market**	5.1 (4.4-5.9) (9)	5.4 (4.8-6.0) (13)	4.3 (3.1-5.5) (8)	7.6 (7.3-7.8) (9)	5.2 (3.8-6.6) (12)	5.8 (4.3-7.2) (8)
**Gov. and Economic Institutions**	4.9 (4.1-5.7) (9)	6.5 (4.5-8.5) (6)	5.7 (4.7-6.7) (12)	7.8 (7.2-8.4) (6)	6.3 (5.7-6.9) (18)	5.4 (4.3-6.6) (8)
**Overall**	**5.4 (5.1-5.8) (73)**	**5.8 (5.5-6.1) (69)**	**5.5 (5.1-5.9) (67)**	**7.7 (7.5-7.9) (54)**	**6.3 (5.9-6.7) (81)**	**6.1 (5.8-6.4) (96)**

The total scores were disaggregated by sector in Figure 1. The radar plots show that the sectors in most need across countries were LM and WE. Both in Nigeria and Ethiopia, the average score for LM and WE struggled to reach the mid-point. However, this was not the case for Rwanda and Tanzania that scored highest in WE, 8.3 (95% CI: 8.1-8.6) and 6.9 (95% CI: 6.1-7.8), respectively. Ethiopia was the only country that struggled to reach the mid-point in the GGEI sector, 4.9 (95% CI: 4.1-5.7). By contrast, the MCH, ED, and FP sectors received scores above the mid-point across all countries. For example, MCH ranged from a low of 5.1 (95% CI: 3.8-6.4) in Nigeria to a high of 7.4 (95% CI: 7.1-7.6) in Rwanda.

For peer review only - http://bmjopen.bmj.com/site/about/guidelines.xhtml

Table 3: National scores, by sector and domain

Country	Policy/Policy making	Services or programs	Advocacy	Research	Civil Society Organizations (CSOs)
**Family Planning**					
Ethiopia	5.7 (5.2-6.2)	5.5 (5.0-6.0)	6.5 (5.7-7.3)	5.7 (5.0-6.3)	5.1 (4.0-6.1)
Kenya	5.9 (5.2-6.7)	5.8 (5.4-6.3)	6.0 (5.1-6.9)	5.6 (4.7-6.4)	5.9 (4.8-7.0)
Nigeria	5.9 (5.0-6.9)	5.8 (5.0-6.6)	6.3 (5.3-7.4)	6.3 (5.1-7.4)	6.7 (5.6-7.9)
Rwanda	7.6 (7.1-8.1)	5.6 (5.2-6.1)	6.3 (5.6-7.1)	6.8 (6.3-7.2)	7.0 (6.6-7.4)
Senegal	6.2 (5.0-7.4)	6.7 (5.2-8.3)	6.8 (5.0-8.5)	5.8 (3.4-8.2)	5.0 (3.9-6.0)
Tanzania	5.8 (5.5-6.2)	5.9 (5.5-6.3)	5.6 (5.0-6.2)	6.4 (6.0-6.9)	6.2 (5.5-6.9)
**Maternal and Child Health**					
Ethiopia	6.4 (5.1-7.7)	6.1 (4.7-7.6)	5.1 (3.5-6.7)	5.3 (3.7-6.8)	4.9 (3.4-6.4)
Kenya	6.4 (5.6-7.1)	6.4 (5.5-7.3)	5.7 (4.4-6.9)	6.0 (4.7-7.3)	4.9 (3.2-6.7)
Nigeria	5.4 (4.0-6.9)	5.2 (3.7-6.6)	5.2 (3.4-7.0)	4.8 (3.2-6.5)	4.6 (2.8-6.5)
Rwanda	7.5 (7.2-7.7)	7.3 (6.9-7.8)	7.3 (6.9-7.7)	7.5 (7.0-7.9)	7.5 (7.1-7.9)
Senegal	6.5 (5.9-7.2)	7.7 (7.1-8.4)	6.8 (5.7-7.8)	7.0 (6.0-7.9)	6.2 (5.0-7.4)
Tanzania	6.6 (5.9-7.2)	6.8 (6.0-7.6)	6.8 (5.9-7.7)	6.7 (5.9-7.6)	6.5 (5.6-7.4)
**Women's Empowerment**					
Ethiopia	5.3 (3.9-6.7)	4.7 (3.3-6.1)	5.2 (3.0-7.3)	3.8 (2.0-5.6)	5.0 (3.3-6.7)
Kenya	5.2 (4.1-6.3)	5.0 (4.1-5.9)	5.8 (4.8-6.8)	4.6 (3.7-5.4)	5.5 (4.2-6.7)
Nigeria	5.1 (4.0-6.1)	4.9 (3.6-6.2)	5.5 (4.2-6.8)	5.0 (3.7-6.3)	5.4 (4.2-6.5)
Rwanda	8.7 (8.4-9.0)	8.2 (7.8-8.6)	8.2 (7.8-8.7)	8.2 (7.8-8.5)	8.2 (7.8-8.6)
Senegal	6.6 (5.8-7.4)	6.7 (5.7-7.7)	6.5 (5.5-7.5)	5.7 (4.5-6.9)	6.0 (4.7-7.3)
Tanzania	6.8 (5.8-7.9)	6.9 (5.9-7.9)	7.2 (6.3-8.0)	6.7 (5.8-7.6)	8.1 (7.5-8.6)
**Education**					
Ethiopia	6.0 (5.5-6.5)	5.6 (4.6-6.6)	5.8 (4.6-7.0)	5.0 (4.3-5.8)	4.0 (3.1-4.9)
Kenya	5.5 (2.4-8.5)	5.2 (2.6-7.9)	6.0 (4.0-8.0)	5.8 (3.7-7.8)	5.3 (1.8-8.8)
Nigeria	6.1 (5.2-7.0)	5.9 (4.9-6.8)	6.6 (5.5-7.6)	5.8 (4.8-6.8)	5.9 (5.1-6.7)
Rwanda	8.2 (7.1-9.2)	8.1 (6.9-9.4)	8.0 (6.9-9.0)	8.4 (7.5-9.3)	8.1 (7.1-9.1)
Senegal	6.1 (4.7-7.4)	6.4 (4.9-8.0)	5.4 (4.1-6.6)	5.5 (3.7-7.4)	5.6 (3.6-7.6)
Tanzania	5.4 (4.2-6.6)	5.3 (3.6-6.9)	5.1 (3.7-6.5)	5.1 (3.6-6.6)	5.0 (3.3-6.6)
**Labor Market**					
Ethiopia	5.5 (4.5-6.5)	4.8 (4.1-5.4)	4.7 (4.1-5.4)	5.4 (4.4-6.4)	3.8 (2.3-5.4)
Kenya	5.9 (5.2-6.5)	5.5 (4.9-6.1)	5.0 (4.3-5.7)	5.1 (4.1-6.0)	5.4 (4.4-6.4)
Nigeria	4.3 (3.2-5.4)	3.7 (2.4-5.1)	4.3 (3.0-5.7)	4.4 (3.0-5.8)	5.0 (3.2-6.8)
Rwanda	7.8 (7.3-8.3)	7.5 (7.2-7.8)	7.2 (6.5-7.9)	7.7 (7.2-8.1)	7.2 (7.0-7.5)
Senegal	5.4 (4.1-6.7)	4.8 (3.4-6.3)	4.5 (2.8-6.2)	4.7 (3.2-6.3)	4.3 (2.7-5.8)
Tanzania	6.1 (4.4-7.8)	5.2 (3.5-7.0)	5.5 (3.8-7.2)	5.6 (3.9-7.4)	6.2 (4.0-8.4)
**Gov. and Economic Institutions**					
Ethiopia	5.6 (4.5-6.7)	5.2 (4.4-6.0)	5.1 (4.1-6.1)	4.6 (3.6-5.6)	4.7 (3.5-5.8)
Kenya	6.6 (4.3-8.8)	7.0 (4.5-9.4)	7.0 (4.5-9.5)	6.3 (3.5-9.0)	6.2 (3.5-9.0)
Nigeria	5.0 (3.8-6.3)	5.2 (4.0-6.3)	6.1 (4.8-7.4)	5.9 (5.0-6.9)	6.6 (5.5-7.7)
Rwanda	7.8 (6.6-9.0)	8.1 (7.4-8.7)	7.8 (7.0-8.6)	7.7 (7.0-8.3)	7.1 (6.8-7.5)
Senegal	6.2 (5.6-6.8)	6.2 (5.5-6.9)	6.6 (5.9-7.4)	6.6 (5.7-7.5)	6.4 (5.8-7.0)
Tanzania	5.2 (4.0-6.4)	5.3 (4.1-6.4)	5.9 (4.2-7.7)	5.1 (3.8-6.4)	6.4 (4.3-8.4)

For peer review only

Even though the total and sectoral scores were around the midpoint across countries, there were several interesting item-specific variations across items within each sector. These are further explored in the next sections.

Family Planning

The policy domain was structured around 14 items. These items scored the political support for FP, pro-poor FP policies, financing for FP, presence of national FP policies and strategic plans, fertility attainment policies, and age for marriage policies. In all the six countries, the average scores for the policy domain were above the mid-point, and the highest average score was registered in Rwanda, 7.6 (95% CI: 7.1-8.1). Across countries, respondents shared positive views about pro-FP policies but had different views about the support received from policymakers. Three respondents in Ethiopia noted that FP policies and programs are supported for their benefits on maternal and child health and FP tends to be politicized. In Kenya, one respondent commented that "Most politicians still encourage big families to get more voters hence undermining efforts made [towards] achieving [a] demographic dividend."

The services and programs domain included 22 items. These items assessed the strength of FP services and programs, community-based provision and equitable access to effective modern contraceptive methods, quality of services, targeted interventions including post-partum and for youth, demand creation, and public-private partnerships. The average scores for all countries were slightly above the mid-point, and the highest score was recorded in Senegal, 6.7 (95% CI:5.2-8.3). In all countries except Rwanda, respondents recorded low scores corresponding to less than 40% FP budget derived from in-country sources. The need for local funding of FP budgets to foster demand-creation was further emphasized by respondents from Nigeria.

The advocacy domain was composed of six items that assessed media coverage of FP programs, public support by policy makers, justification for FP method, accessibility to social marketing programs and special focus to special populations including adolescents, indigenous people and those with disabilities. Scores were above average in all countries and the highest was recorded in Senegal (6.8, 95% CI: 5.0-8.5). Mixed findings about FP political support were recorded particularly in Kenya and Nigeria. In Kenya three respondents noted that politicians are still encouraging large families to secure more voters while in Nigeria politicians seem to be concerned with the growing populations although and offer their support to strengthen social marketing programs.

The research domain had 11 items that assessed reporting mechanisms, data availability, capacity building, quality of data, capacity of research institutions, data utilization and dissemination. The highest scores were recorded in Rwanda (6.8, 95% CI: 6.3-7.2) and the scores in all the other countries were above the average. There were also shared views across countries about data quality, reliance on Demographic and Health Surveys and low use of smaller studies conducted by locals. One responded in Ethiopia noted a frustration: "I myself [have] conducted research on Factors Encouraging Women to Pursue Family Planning Method. Even though I have done the research by self-sponsoring the Oromia Regional Health Bureau cannot use it".

The civil society domain included 14 items that covered CSO power, budget analysis skills to inform advocacy, facility-based services involvement, social franchising, community-based services involvement, and their active role in targeted programs, monitoring and accountability. Ethiopia and Senegal were scored around the mid-point, and Rwanda scored highest (7.0, 95% CI: 6.6-7.4). Respondents from all countries shared positive views on the crucial role played by the CSOs however the

For peer review only - http://bmjopen.bmj.com/site/about/guidelines.xhtml

views on partnerships between the government and CSOs varied from one country to another. One respondent in Ethiopia noted that "As [the] government has [implemented a] health extension program at community level, the CSOs ha[ve] closed [down] their community service. But [they are] supporting the gov[ernment] by building the capacity of the health extension workers". Another respondent in Tanzania commented "there are [a] number of CSOs active in Tanzania, especially in advocacy and accountability for FP. However, the government ha[s] suppressed their operationalization in doing advocacy to hold the government accountable for FP policies and commitments in place".

Maternal and Child Health

The policy domain comprised of 13 items that assessed the presence and strength of policies around maternal and neonatal survival, universal access to antenatal and perinatal care, abortion, post-abortion care, financing, affordability, multisectoral approaches and private sector involvement. All countries' average scores were at the mid-point or above with Nigeria (5.4, 95% CI:4.0-6.9) and Rwanda (7.5, 95% CI:7.2-7.7) scoring lowest and highest respectively.

The services and programs domain had eight items that focused on obstetric care availability at the primary and secondary healthcare levels, universal access to pregnancy-related services including antenatal care and management of major complications, newborn care, and FP integration. The highest score was recorded in Senegal (6.7, 95% CI:5.2-8.3) and the lowest in Nigeria (5.2, 95% CI:3.7-6.6). Despite high scores of a range of contraceptive methods for women, lower scores were recorded for the provision of the male sterilization method at the district hospitals in Kenya, Senegal, Nigeria, and Ethiopia. Respondents across countries shared the view of the essential role of health extension workers and common experience of stock outs of vaccines and contraceptives, limited skills of providers, resource allocation gaps, and challenges related to MCH policy effectiveness assessment.

The advocacy domain comprised of six items that covered government messages, mass media, community-level media, educational material, self-review of maternal mortality cases and informing policies with new evidence. Rwanda (7.3, CI 6.9 -7.7) scored highest while Ethiopia (5.1, 95% CI: 3.5-6.7) and Nigeria (5.2, 95% CI: 3.4-7.0) scored lowest. Lower scores were consistent in Kenya, Ethiopia, and Kenya for the utilization of evidence to inform policies at health facility levels and self-review of maternal mortality cases.

The research domain included 11 items that focused on data mining infrastructure, research strategy, quality of data, strength of research institutions and data utilization. The highest score was recorded in Rwanda (7.5. 95% CI: 7.0-7.9) and lowest in Nigeria (4.8, 95% CI: 3.2-6.5). Respondents across countries consistently reported data quality challenges, insufficient skills and funding, and underutilization of available platforms. One respondent in Ethiopia noted an issue that "the available technology is not utilized to its maximum benefits e.g., DHIS does not link and analyze vital registration, even though, there is a module for it to do so; data generated using different systems like Maternal Perinatal Death Surveillance Response (MPDSR) is not linked in a central place to facilitate access to existing different maternal data sets."

The civil society domain composed of 12 items that assessed the power and contribution of CSOs in advocacy, programs, services, research, monitoring and evaluation. Overall, the highest average score was recorded in Rwanda (7.5, 95% CI: 7.1-7.9) and the lowest score was recorded in Nigeria (4.6, 95% CI: 2.8-6.5). A notable finding on item-specific scores was that in Kenya, Nigeria and Ethiopia; the CSO

For peer review only - http://bmjopen.bmj.com/site/about/guidelines.xhtml

involvement in social franchising and their contribution in leveraging mHealth opportunities received similar low marks.

Women's Empowerment

The policy domain contained 18 items that addressed gender equality policies, demonstrated level of commitment to the Convention on the Elimination of all Forms of Discrimination Against Women (CEDAW), equity in marriage and family, violence against women measures, favorable policies for land ownership, equitable work, women's participation in policymaking, and transparency and accountability. Of all six countries, Rwanda was scored highest (8.7, 95% CI: 8.4-9.0) and Nigeria scored lowest (5.1, 95% CI: 4.0-6.1). In Nigeria, Ethiopia and Kenya, very low scores were recorded for women's participation in policy-making and traditional governance systems (e.g., chiefs and village head). In the same countries and Senegal lower than average scores were recorded for provision of reliable periodic age disaggregated data on women's empowerment needs among populations in different communities. In Ethiopia, a respondent indicated that "the policy formulation does not consult the gender specialists and most of the policies and laws lack the concrete idea on the relevance of integrating the gender issue and that's why most of the policies and laws are not gender responsive as needed".

The services and programs domain had 12 items that assessed gender mainstreaming, gender transformative programming, school dropouts, technology literacy, employment, violence victimization, FP for women's empowerment, child marriage and female genital mutilation. Overall, the scores across were around the midpoint except for Rwanda which was scored (8.2, 95% CI: 7.8-8.6). Kenya, Ethiopia, and Nigeria scored lower than average on two critical items. First, law enforcement efforts to eliminate child marriage. Second, levels of efforts to which the government makes possible, through laws, regulations, and programs, for women to combine the roles of childbearing, breast-feeding and child-rearing with participation in the workforce. In addition, respondents across countries shared the view on challenges related to WE policy enforcement and translation into programs. A respondent in Tanzania reflected that, "Efforts might be there, but they are one sided/ single handed and do not receive a complimentary support from other areas of enforcement. Laws and legislation in place are weak and/or not enforced as needed".

The advocacy domain comprised of seven items that assessed the existence of advocacy platforms for women's and girls' empowerment, information dissemination, employment, education and elimination of all forms of violence. The highest score was recorded in Rwanda (8.2, 95% CI: 7.8-8.7) and the lowest in Ethiopia (5.2, 95% CI:3.0-7.3). On item specific-scores, Nigeria, Kenya, Ethiopia scored below average on the government undertaking sufficient efforts to ensure that the population is aware of issues related to WE. Similarly, the three countries scored low marks on whether the information that spread is correct and reaches people in a language they understand.

The research domain had ten items that assessed the capacity of research institutions, data quality and utilization at the ministerial level, existence of a women's empowerment research strategy, topical data gathering and dissemination of research findings. The lowest national score was recorded in Ethiopia (3.8 95% CI: 2.0-5.6). In Ethiopia, Kenya and Nigeria lower than average scores were recorded for the extent to which government and private institutions undertake WE and child marriage research. Across countries, respondents shared the view on irregularities of research initiatives and limited utilization of research and evaluation data to inform WE policies and programs. A respondent in Ethiopia noted that "The research institutions do not conduct research periodically and I only see research come out when something

For peer review only - http://bmjopen.bmj.com/site/about/guidelines.xhtml

happens. There is no periodic research to observe a progress on a specific issue especially on gender and women's empowerment".

The civil society domain included ten items that covered the integration of civil society in policy, programs, evaluation, and research. They also addressed the resourcefulness of civil society organizations in generating evidence and utilizing data to inform advocacy efforts. Overall, the national scores were around the mid-point except for Rwanda that scored 8.2 (95% CI:7.8-8.6). On item-specific scores, lower than average marks were recorded in Senegal, Ethiopia and Kenya for the routine statistical system provision of good, reliable, and periodic age-disaggregated data on women's empowerment needs among populations in different communities. Respondents also highlighted the need for coordination and more results-oriented programming. One respondent in Kenya noted that "there are many CSOs [working on] WE in Kajiado [County] but [they] work in [an] uncoordinated manner leading to duplication [and] non-performance as they trade in ' wait and see' [for] under and overutilization of resources".

Education

The policy domain included 13 items that assessed the quality of national plans, strategies, and programs to improve the quality of learning in schools, gender parity, technology integration and learning for out-of-school children. The highest scores were recorded in Rwanda (8.7, 95% CI: 8.4-9.0) and the lowest scores were recorded in Tanzania (5.4, 95% CI: 4.2-6.6). A notable finding was on the item-specific marks in all countries except Rwanda, lower than average scores were recorded for technology literacy. Similarly, the strategies to overcome learning obstacles for pregnant girls and adolescent mothers also scored low in all countries except Rwanda and Kenya.

The services and programs domain had five items that covered teacher quality, teachers' deployment nationwide, parental involvement, standardized assessment and use of assessments to inform progress. Across countries, lower scores were recorded for the use of assessments to inform progress and several respondents emphasized that the ED systems lacked feedback systems, access and utilization of standards results to improve performance. One respondent in Ethiopia said, "Assessment is one of the activities that lagged behind for many years. Though national level large scale assessments are conducted (such as NLA, EGRA, EGMA) none of the study results or reports can be found at schools, woreda and zonal levels. Thus, beneficiaries do not utilize results of the studies to improve practice".

The advocacy domain contained four items that assessed the quality of education, education system, teachers' obstacles, and institutional obstacles. Highest scores were recorded in Rwanda (8.0, 95% CI: 6.9-9.0) and lowest scores were recorded in Tanzania (5.1, 95% CI:3.7-6.5). Respondents across countries highlighted the need to ensure human resource development and promotion of debate about the educational institutions. One respondent in Tanzania emphasized that the "challenges associated with educational institutions should be made as country debate to inform the policy institutions".

The research domain contained 11 items that assessed data gathering, utilization, research capacity and ministerial-level use of evaluation data. The highest scores were recorded in Rwanda (8.4, 95% CI: 7.5-9.3) and the lowest scores were recorded in Ethiopia (5.0, 95% CI: 4.3-5.8). An important finding was that in Ethiopia, Kenya, Tanzania and Senegal, lower than national average scores were recorded for the management's use of evaluation findings, ministerial use of evaluation findings, and the extent to which information is shared or spread throughout various levels (national, state/province, localities). Further,

For peer review only

several respondents from all countries highlighted the need to streamline research interventions to avoid the duplication of efforts and data fatigue.

The civil society domain contained 11 items that assessed the involvement of civil society in the ED sector, the role of the community in ED, partnerships, social franchising, and technology. The lowest scores were recorded in Ethiopia, (4.0 95% CI: 3.1-4.9) and the highest scores were recorded in Rwanda (8.1, 95% CI: 7.1-9.1). Notably, in Ethiopia, the average scores for all items fell below the midpoint. Similarly, in Senegal, scores for community inclusion and the role of civil society in monitoring and evaluation were below the midpoint.

Labor Market

The policy domain comprised of 15 items that covered key aspects of global and regional economic integration, technology, unemployment policies, urbanization, equitable access to employment, access to credit, targeted labor investment, saving, and self-employment policies. The lowest scores were recorded in Nigeria (4.3, 95% CI:3.2-5.4) and the highest scores were recorded in Rwanda (7.8, 95% CI:7.3-8.3). In all countries except Rwanda, very low scores were recorded for existence of policies that outline an unemployment insurance system and the extent to which policies account for and address unemployment in various population groups and geographic areas.

The service or programs domain included 15 items that addressed unemployment reduction, access to microcredit, youth entrance to the labor market, youth entrepreneurship, women-friendly labor, minimum-wage and addressing informality in the labor market. The highest scores were recorded in Rwanda (7.5, 95% CI: 7.2-7.8) and the lowest scores were recorded in Nigeria (3.7, 95% CI: 2.4-5.1). Remarkably, in Nigeria, Ethiopia, Tanzania and Senegal, most questions in this domain scored below the midpoint and respondents across countries emphasized the need to foster services and programs that promote employment with special focus on youth and women. One respondent in Tanzania iterated that “Apprenticeship programs have begun since the past three years but still some of our young graduates do not understand the essence of internship programs. Moreover, women are highly struggling for senior positions both in the Government and private sectors.”

The advocacy domain questionnaire was composed of five items that addressed gender equality in the labor market, youth employment, minimum wage, and advocacy from government members. In Ethiopia, Nigeria and Senegal, the recorded average scores for the advocacy domain were below the midpoint. Notably, item-specific average scores for Ethiopia and Senegal were low for the minimum wage advocacy. Another interesting finding was that gender equality in the labor market scored high in Rwanda, Ethiopia and Tanzania although this score was below the midpoint in Nigeria and Senegal.

The research domain questionnaire was composed of 11 items that addressed the presence of LM observation, research strategy, market analysis, quality of data, data dissemination, management, and managerial use of the evaluation data. In Nigeria and Kenya, the average individual question scores were low for the presence of monitoring and surveillance systems for employment. One respondent in Kenya suggested that “[the] government needs to develop a database for all students coming out of institutions and monitor their progress in the job market. Those not in jobs within six months after joining the job market, should be assisted by governments to start their own businesses through advancement of soft loans.”

For peer review only - http://bmjopen.bmj.com/site/about/guidelines.xhtml

The civil society domain was assessed using 10 items that addressed the power of civil society in the LM, use of budget analysis for advocacy, community-based programs, diversification of job opportunities, worker's rights, women's employment, and civil society partnerships. Below average scores for the research domain were recorded in Ethiopia and Senegal and most items fell under the midpoint in these countries with the diversification of jobs scoring lowest in Senegal and Nigeria.

Governance and Economic Institutions

The policy domain was assessed using 13 items that addressed democratic values, youth participation in democratic processes and policymaking, transparency and accountability, national security, and affirmation of safe learning environment. Across countries, the average scores were around the midpoint except Rwanda (7.8, 95% CI: 6.6-9.0). In Nigeria, Ethiopia, and Tanzania the lowest item-specific average scores were recorded for the extent to which education facilities are protected and alternative learning opportunities are created for children and youth in time of conflict or displacement.

The services and programs domain questionnaire contained 11 items that addressed interventions that strengthen youth organizations and networks, youth leadership, youth involvement in national activities, safety and reliable police services, participation in democratic elections, women's participation, sustainable economic opportunity, and human capital development. Across countries, the average scores were around the midpoint or higher with the highest in Rwanda (8.1, 95% CI:7.4-8.7) and Kenya (7.0 95% CI:4.5-9.4). An interesting finding was that in all countries, on item-specific scores, the highest scores for women's participation in governance were recorded on women's participation in governance at national levels and urban places but the lowest scores were recorded for their participation at sub-national levels and rural places.

The advocacy domain questionnaire contained six items that addressed youth empowerment, government advocacy, non-governmental advocacy, voices of instrumental sectors of the DD that included financial stability, optimal MCH, FP and a diversified labor market. Across countries, the average national scores were around the midpoint with the highest in Rwanda (7.8, 95% CI:7.0-8.6). Notably, on item-specific scores the lowest score was recorded in Ethiopia on diversification of labor market.

The research domain questionnaire was composed of ten items that assessed the presence of governance data, capacity of research instructions working in the areas of governance, finance and development, quality of data and data utilization by individuals and ministries. The scores varied across countries and the highest scores were recorded for Rwanda (7.1, 95% CI:6.8-7.5). The average scores recorded for the majority of individual questions but were below the midpoint in Ethiopia and Tanzania. The lowest score in Ethiopia was for the extent to which safety and the rule of law is researched whereas in Senegal the lowest recorded score was for the extent of measurement of political participation.

The civil society domain was composed of nine items that assessed the power of civil society, use of policy and budget analysis in advocacy efforts, women's sectoral participation, advocacy and accountability, CSO-led assessment, and monitoring. The average scores were around the midpoint across all countries with the lowest in Ethiopia (4.7, 95% CI:3.5-5.8). A prominent finding to highlight was that across all countries, the average scores were high for the extent to which CSOs support youth participating in education, training, employment, and leadership.

For peer review only - http://bmjopen.bmj.com/site/about/guidelines.xhtml

Discussion

This study aimed to develop a tool to assess government efforts in creating a favorable policy environment to seize the benefits of a demographic dividend. The study collected data across six key sectors in Nigeria, Rwanda, Ethiopia, Senegal, Tanzania, and Kenya from key informants identified by the in-country study collaborators.

The DDEI scores revealed patterns that were helpful to guide discussions regarding needed improvements in respective countries across five domains namely policymaking, services or programs, advocacy, research, and civil society. The patterns of efforts scores for the LM, WE, FP, GGEI and ED sectors suggested great challenges for women's self-determination and empowerment, employment, and leadership. These findings agreed with previous predictions by the World Economic Forum that women may have to wait for 100 years to pair males in leadership roles in bigger institutions and take up ministerial roles. This is currently seen in the Global Gender Gap (GGG) Report, which ranks Rwanda in the top ten countries with the smallest gender gap among countries worldwide.[28 29]

Relatedly, Rwanda scored remarkably high on WE, although the scores were also moderately high for Tanzania and Senegal. These findings aligned with how these countries performed on the 2020 GGG report. Of the 153 countries ranked globally and on a maximum score possible of 1, Rwanda was ranked 9th with a score of 0.791 and Tanzania 68th with a score of 0.713. In contrast, Ethiopia ranked 82nd (0.705), Senegal 99th (0.684), Kenya 109th (0.671), and Nigeria 128th (0.635).[28] Although the intent of the DDEI was not to rank countries, these findings emphasized the potential for mutual learning from countries and the utility of the DDEI to dive deeper into specific items that can reveal special lessons or areas that require improvements. This is even more crucial, especially for countries that had a much larger change in ranking during the two years that preceded the report, such as Ethiopia that experienced an improvement of 35 ranks, and Kenya with a decrease of 33 ranks[28].

Furthermore, human capital development is essential for the country to cultivate and harness its DD potential. The 2020 Human Capital Index report revealed that the scores for all the six countries of this study ranged from 0.36 in Nigeria to 0.55 in Kenya. These findings indicate that children born today in the six countries could miss out on 45% to 64% of their future earnings potential because of incomplete education and health that is not full.[30] The DDEI provided an opportunity to qualitatively compare different ongoing efforts initiated to develop human capital and the findings in MCH, ED, and WE were in agreement with the 2020 HCI findings. For instance, the lowest score for MCH was recorded in Nigeria 5.1 (95% CI: 3.8-6.4), and item-specific scores revealed wide variations that allowed for the identification of gaps.

This study offers several strengths. First, the questionnaires and the implementation of the DDEI were conducted in collaboration with in-country partners. This collaboration fosters local ownership of the effort measurement tool. In the future, local institutions can regularly replicate these measurements at national and sub-national levels. Second, unlike prior effort indices, such as the FPEI, (created in 1972), the API (created in 1999), and MNPI (created in 2000), [23 31 32] the DDEI integrated critical sectors into one tool, and sectoral effort measures were not done in silos due to the interconnectedness and potential synergy between sectors (e.g. family planning, education, maternal and child health and women's empowerment). Finally, this tool provides the information to constituents to hold their governments accountable and provide unconventional evidence for planning and evaluation purposes.

For peer review only - http://bmjopen.bmj.com/site/about/guidelines.xhtml

The DDEI was tested at the national level which opened the opportunity to be applied at the sub-national level. In countries where health and development programs are decentralized, sub-national DDEI can be transformational in guiding local budget allocation and self-evaluation. Given the paucity of quantitative data and poor utilization of the existing information systems, the DDEI has the potential to become a unique data source that informs local discussions and conversation at all administrative and planning levels.

Limitations

This study presented a number of limitations. The DDEI relied on experts' opinions, which may be affected by different factors. First, people's judgment may be heavily influenced by their recent experience, which may not reflect the efforts placed over the years. Second, respondent's judgment may be influenced by the level of their expectations, which may also be correlated with the level of satisfaction. For instance, where people have low expectations, the scores may be high despite the weak levels of efforts and vice-versa.

Further, despite detailed instruction on the main purpose of the study, another limitation worth mentioning is that the aggregate average scores for the sectors modulated centering to the mid-point of 5 over the maximum possible 10. This tendency may indicate that respondents may have compensated less generous scores that they gave to some questions with more generous scoring for the others. This also demonstrated the need to analyze item-specific scores for comparisons within domains which revealed wide variations across scores and interesting findings.

The DDEI questionnaire was administered in the midst of the COVID-19 pandemic, which led to abrupt changes in health and development systems in all countries. Although respondents provided great insights about their respective sectors, it is not unrealistic to assume the response rates and responses received may have been affected by the status of pandemics in their countries. Lastly, additional research will be needed to nurture the index, improve the reliability of the scores, assess the need for weighing of items and domains based on the stage of demographic transition while improving the usability of the DDEI data by local and global health and development stakeholders.

Conclusion

This study developed the DDEI for Nigeria, Ethiopia, Kenya, Tanzania, Rwanda, and Senegal and demonstrated the utility of this index. The DDEI yielded several recommendations on opportunities to accelerate socio-economic development and take advantage of the DD potential in these countries. The scores for each sector and domain provided variations that allowed for comparison within sector in each country. By engaging with local experts and partnering with local institutions in a participatory process, the data from the DDEI were used by local policymakers, service provides, program implementers, advocates, and researchers for self-evaluation and to develop recommendations to strengthen ongoing efforts and programs in respective sectors.

The DDEI allowed for multisectoral analysis and provided an opportunity to identify sectors in most need and those that can serve as models in each country. For instance, based on sector-specific scores, LM and WE stood out as the sectors that required particular attention. However, despite higher sector-specific

For peer review only - http://bmjopen.bmj.com/site/about/guidelines.xhtml

scores, a closer look at item-specific scores across domains revealed wide variations that were the basis of generating policy recommendations. The DDEI can be applied to all SSA countries regularly to monitor progress and spark local conversation around human rights and sustainable socio-economic development.

The DDEI can serve as complementary tool to existing global development monitoring tools. The findings from the DDEI aligned with other existing measures such as the GGG and HCI in various countries. Given the quantitative nature of these tools, the DDEI provide an opportunity to assess the inputs and guide policies, investments and programs in specific sectors and items that can positively affect the gender gap and human capital. In addition to driving conversation amongst stakeholders in FP, MCH, ED, WE, GGEI and LM, the DDEI focuses on assessing the inputs that are critical for the outcomes measured by GGG and HCI. The current DDEI has been translated in English and French. In the future, it should be translated in other languages such as Portuguese and Spanish to make it applicable to other countries in the early stages of their demographic transition.

Ethical considerations

The study was conducted in compliance with the Helsinki declaration and was approved by the Johns Hopkins Bloomberg School of Public Health ethical review board (IRB Number: IRB00011522). All participants provided informed consent prior to taking part in this research.

Contributorship statement

Jean Christophe Rusatira, Carolina Cardona, Michelle Martinez, and Jose G. Rimon conceived the study. Jean Christophe Rusatira, Carolina Cardona, Michelle Martinez, Saifuddin Ahmed, Jose G. Rimon developed the questionnaire, led data collection, data analysis, and interpretation. Jean Christophe prepared the first draft of the manuscript. All authors revised and approved the final version of the paper and agree to be accountable for all aspects of the work.

Acknowledgments

We express our deepest thanks to all participants for their contributions to this study. We would also like to thank our local partners and staff who facilitated this work at the Centre d'Excellence Régional en Economie Générationnelle in Senegal, the Center for Communication Programs in Nigeria, the National Council for Population and Development in Kenya, the Tanzania Communication and Development Center, the Ethiopian Academy of Sciences, and the Centre for Integrated Development Research and Action in Rwanda.

Competing interests

The authors declare that they have no competing interests.

Funding

This work was supported by the Bill and Melinda Gates Foundation under the grant OPP1181398.

Data sharing statement

The data used to produce the manuscript are available from the corresponding author on request.

For peer review only

References

1. Bloom D, Canning D, Sevilla J. The demographic dividend: A new perspective on the economic consequences of population change: Rand Corporation 2003.
2. Reher DS. Economic and Social Implications of the Demographic Transition. *Population and Development Review* 2011;37:11-33.
3. Herzer D, Strulik H, Vollmer S. The long-run determinants of fertility: one century of demographic change 1900–1999. *Journal of Economic Growth* 2012;17(4):357-85. doi: 10.1007/s10887-012-9085-6
4. Bloom DEaH, Salal and Rosenberg, Larry and Sevilla, J.P. and Trussell, T. James. A Demographic Dividend for Sub-Saharan Africa: Source, Magnitude, and Realization. *IZA Discussion Paper No 7855* 2014:26.
5. Demographic Dividend Atlas for Africa: Tracking the Potential for a Demographic Dividend: UNFPA, 2017.
6. Bloom DE, Williamson JG. Demographic Transitions and Economic Miracles in Emerging Asia. *The World Bank Economic Review* 1998;12(3):419-55. doi: 10.1093/wber/12.3.419
7. Bloom D, Canning D, Malaney PN. Demographic Change and Economic Growth in Asia: Center for International Development at Harvard University, 1999.
8. AU Roadmap on Harnessing the Demographic Dividend through Investments in Youth. Assembly of the Union Twenty- Sixth Ordinary Session 30-31: African Union Commission, 2016:44.
9. Cardona C, Rusatira JC, Cheng X, et al. Generating and capitalizing on the demographic dividend potential in sub-Saharan Africa: a conceptual framework from a systematic literature review. *Gates Open Research* 2020;4(145):145.
10. Bloom D, Canning D, Malaney PN. Demographic change and economic growth in Asia. *CID Working Paper Series* 1999
11. Bloom DE, Canning D. Global demographic change: Dimensions and economic significance: National Bureau of Economic Research, 2004.
12. Fabic MS, Choi Y, Bongaarts J, et al. Meeting demand for family planning within a generation: the post-2015 agenda. *The Lancet* 2015;385(9981):1928-31. doi: 10.1016/S0140-6736(14)61055-2
13. Bloom DE, Canning D, Hu L, et al. The Contribution of Population Health and Demographic Change to Economic Growth in China and India. *J Comp Econ* 2010;38(1):17-33. doi: 10.1016/j.jce.2009.11.002 [published Online First: 2010/04/27]
14. Li Q, Tsui AO, Liu L, et al. Mortality, fertility, and economic development: An analysis of 201 countries from 1960 to 2015. *Gates Open Res* 2018;2:14. doi: 10.12688/gatesopenres.12804.1 [published Online First: 2018/04/24]
15. Ahmed S, Li Q, Liu L, et al. Maternal deaths averted by contraceptive use: an analysis of 172 countries. *Lancet* 2012;380(9837):111-25. doi: 10.1016/s0140-6736(12)60478-4 [published Online First: 2012/07/13]
16. Conley D, McCord GC, Sachs JD. Africa's lagging demographic transition: Evidence from exogenous impacts of malaria ecology and agricultural technology: National Bureau of Economic Research Cambridge, Mass., USA, 2007.
17. Becker GS, Becker GS. A Treatise on the Family: Harvard university press 2009.
18. Ahmed SA, Cruz M, Quillin B, et al. Demographic Change and Development: Looking at Challenges and Opportunities through a New Typology: The World Bank 2016.
19. Ahmed SA, Cruz M, Go DS, et al. How Significant is Africa's Demographic Dividend for its Future Growth and Poverty Reduction?: The World Bank 2014.

For peer review only

20. Shekar M, Yazbeck A, Hasan R, et al. Population and Development in the Sahel: Policy Choices to Catalyze a Demographic Dividend. *Health, Nutrition and Population (HNP) Discussion Paper, World Bank* 2016 doi: 10.1596/25293
21. WBG. Global Monitoring Report 2015/2016: Development Goals in an Era of Demographic Change. Washington, DC: The World Bank 2016.
22. Ahmed SA, Cruz M. Making the Most of Demographic Change in Southern Africa: The World Bank 2016.
23. Stover J. The AIDS Program Effort Index (API): Results from the Field Test. *Washington, DC: Futures Group* 1999
24. Ross JA, Campbell O, Bulatao R. The maternal and neonatal programme effort index (MNPI). *Tropical medicine & international health* 2001;6(10):787-98.
25. Ross J, Stover J. The family planning program effort index: 1999 cycle. *International Family Planning Perspectives* 2001:119-29.
26. Fort AL, Deussom R, Burlew R, et al. The Human Resources for Health Effort Index: A tool to assess and inform strategic health workforce investments. *Human resources for health* 2017;15(1):1-11.
27. Usengumukiza F, Munyandamutsa JP, Nshutiraguma E. Strengthening democratic governance and accountability in Rwanda: The role of the Rwanda Governance Board (RGB). Democratizing Public Governance in Developing Nations: Routledge 2017:109-33.
28. Forum WE. Global Gender Gap Report 2020: Insight Report. 2019:1-371.
29. Schwab K. The Global Competitiveness Report 2019. Geneva. World Economic Forum, 2019.
30. The Human Capital Index 2020 Update. The Human Capital Index 2020 Update; 2020.
31. Lapham RJ, Mauldin WP. Contraceptive prevalence: the influence of organized family planning programs. *Studies in Family Planning* 1985;16(3):117-37.
32. Ross JA, Campbell OM, Bulatao R. The Maternal and Neonatal Programme Effort Index (MNPI). *Trop Med Int Health* 2001;6(10):787-98. doi: 10.1046/j.1365-3156.2001.00787.x [published Online First: 2001/10/27]

For peer review only

Figure 1: Average scores per country and sector

For peer review only

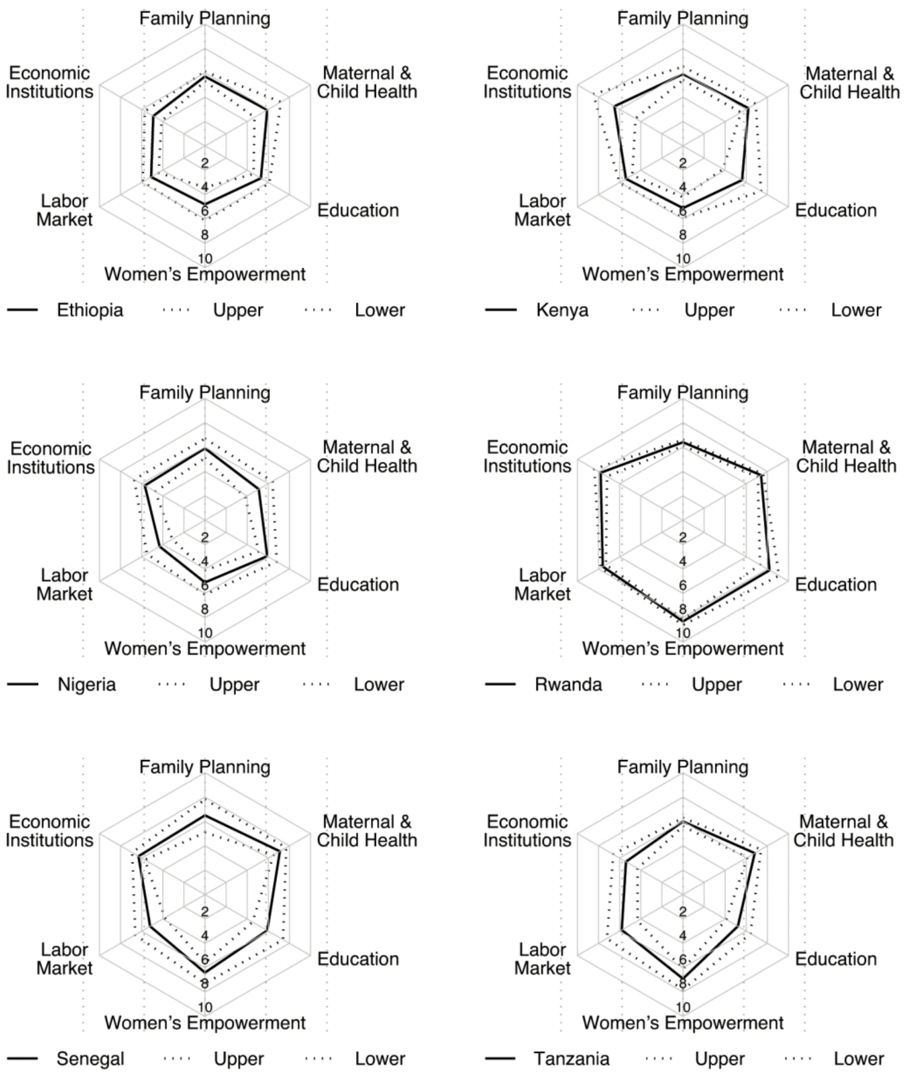

Figure 1: Average scores per country and sector

635x740mm (72 x 72 DPI)

JOHNS HOPKINS
BLOOMBERG SCHOOL
of PUBLIC HEALTH

Bill & Melinda Gates Institute for Population and Reproductive Health

Demographic Dividend Effort Index Survey

Introduction

There is a programmatic need for a standard measure that quantifies the extent of national efforts in policies and programs implemented to cultivate, realize and harness the benefits of a demographic dividend (DD). Despite the remarkable progress achieved by various countries through the integration of demographic dividend principles into their policies and development plans, performance assessment tools and platforms to document best practices and lessons learned to harness the DD are still missing.

Thus, the purpose of the DD Effort Index (DDEI) is to provide a standard measure that will quantify the nature and strength of in-country DD efforts by experts and civil society members across the public and private spheres that represent different dimensions of the society. By measuring efforts, we are capturing inputs independent of outputs, such as income per capita. This will allow us to assess the relationship between inputs that go into the development of DD policies and DD outputs.

This DDEI is guided by one central question: To what extent have the existing national policy and program efforts enabled a favorable policy environment to cultivate, realize and harness the benefits of a demographic dividend? To answer this question, the DDEI will summarize key informant perceptions across six sectors identified in the literature to produce a favorable environment to harness a DD. These DD framework sectors are: 1) Family Planning, 2) Maternal and Child Health, 3) Education, 4) Women's Empowerment, 5) Labor Market, and 6) Good Governance, Strong Institutions and Stable Economy. Each sector is measured through its own questionnaire and will be assessed in five dimensions identified in the literature; namely, policy, services or programs, advocacy, research, and civil society. Each of the 6 questionnaires, completed by 10 expert observers per country, score items on a scale from 1 to 10, unless otherwise noted.

Considering the health and socio-economic impact of COVID-19, and its close relationship with policies that enable a DD favorable policy environment, this DD Effort Index is integrating a module to assess the resilience and sustainability of systems in each sector. The scoring follows the key principles of sustainable and resilient health systems that can effectively respond to emerging infectious disease threats and other public health emergencies.

The DDEI provides an opportunity to visualize the national efforts while assessing the extent to which these efforts support a country’s goals to cultivate, realize and harness the demographic dividend. The DDEI will be a tool for performance measurement and progress monitoring, which will provide a timely assessment of successes and gaps and reveal best practices and areas of improvement.

JOHNS HOPKINS
BLOOMBERG SCHOOL
of PUBLIC HEALTH

Bill & Melinda Gates Institute for Population and Reproductive Health

Demographic Dividend Effort Index Survey

Consent form

Thank you for taking the time to complete this questionnaire, especially in this unprecedented time.

The Gates Institute is developing a Demographic Dividend Effort Index (DDEI) to establish a standard measure that will allow the assessment of the existing status of national policies, services, and programs in family planning, maternal and child health, other social sectors (e.g., employment and education), and governance to enable a favorable policy environment to cultivate a demographic dividend (DD). By measuring efforts, this tool will capture inputs independent of outputs, such as income per capita. This will allow the assessment of the relationship between inputs that go into the development of DD policies and DD outputs that are observed and commonly used to assess progress.

We would like to ask you some questions because of your expertise in the area. The information collected will be kept confidential, and we will not be collecting your names or any other identification details. Completing this questionnaire will take around 45-60 min of your time.

We will appreciate your participation in this survey. Information from this interview will be used by the Gates Institute and the community of DD practice in your country to assess the national efforts and identify areas to continue supporting the successes and those that need to be strengthened. Researchers may also use the data collected for cross-country analyses.

You are invited to participate in this survey. Please know that this survey is completely voluntary. By participating, there is no direct benefit to you but there is a societal benefit in enabling the government and development partners to strengthen their efforts to cultivate a DD-favorable environment.

You may refuse to answer any question. Should you have any concerns or issues about the study, you may contact the study coordinator at **jcrusatira@jhu.edu** or **+14436510824** by a phone call or WhatsApp.

* Please check "Yes" if you would like to proceed with the survey.

- ◯ Yes
- ◯ No

JOHNS HOPKINS
BLOOMBERG SCHOOL
of PUBLIC HEALTH

Bill & Melinda Gates Institute for Population and Reproductive Health

Demographic Dividend Effort Index Survey

Important notice

Please do not select 'Back' while in the middle of answering a page of questions as the page you are working on will not be saved until you finalize the said page and select 'Next'. If you select 'Back' without finalizing, the progress you have made on the unfinished page will be lost.

Please be aware that your survey is completed when you read "Thank you for completing the demographic dividend effort index. Stay well and safe!"

JOHNS HOPKINS
BLOOMBERG SCHOOL
of PUBLIC HEALTH

Bill & Melinda Gates Institute for Population and Reproductive Health

Demographic Dividend Effort Index Survey

Respondent country and expertise

* Please select the country where you are responding from.

- ◯ Kenya
- ◯ Senegal
- ◯ Nigeria
- ◯ Rwanda
- ◯ Tanzania
- ◯ Ethiopia

JOHNS HOPKINS
BLOOMBERG SCHOOL
of PUBLIC HEALTH

Bill & Melinda Gates Institute for Population and Reproductive Health

Demographic Dividend Effort Index Survey

Level of expertise

* At which level of Government is your expertise (If your expertise is at the national level please select "National" otherwise select a county?

JOHNS HOPKINS
BLOOMBERG SCHOOL
of PUBLIC HEALTH

Bill & Melinda Gates Institute for Population and Reproductive Health

Demographic Dividend Effort Index Survey

Expertise

* Please select the organization you have worked in the most during the last five years. If your organization is not listed, please write it down.

- Public sector
- Private sector
- Non-governmental organization
- University/research institute
- Other (please specify)

* Which dimension have you worked in the most during the last five years?

- Policy/policymaking
- Services or programs
- Advocacy
- Research
- Civil Society

* In which sector have you worked the most during the last five years?

- Family Planning
- Maternal and Child Health
- Education
- Women's Empowerment
- Labor Market
- Governance and Economic Institutions

JOHNS HOPKINS
BLOOMBERG SCHOOL
of PUBLIC HEALTH

Bill & Melinda Gates Institute for Population and Reproductive Health

Demographic Dividend Effort Index Survey

Family Planning Questionnaire

Please respond to the following questions based on your experience/expertise within this specific sector.

To give a summary picture of the national demographic dividend effort, please rate the following items. Score each item from 1 to 10, where 1 represents the lowest score (very weak or no effort) and 10 represents the highest score (strong effort). Where applicable, if a policy or activity is non-existent then mark your response as 0. Please note that a few questions have a different scoring scale.

Please answer each item. All responses will be recorded in the format exemplified below:

Lowest effort	1	2	3	4	5	6	7	8	9	10	Highest effort	I do not know

JOHNS HOPKINS
BLOOMBERG SCHOOL
of PUBLIC HEALTH

Bill & Melinda Gates Institute for Population and Reproductive Health

Demographic Dividend Effort Index Survey

Dimension 1. Policy/Policy making

* FP 1.1 Prominence of Family Planning (FP): Extent to which there is a permanent office or post within the appropriate ministry that develops and monitors sectoral policies and strategies, that is well placed within the government, and staffed by adequately skilled personnel.

	1	2	3	4	5	6	7	8	9	10	Don't know
1-Lowest -> Highest-10	○	○	○	○	○	○	○	○	○	○	○

* FP 1.2 Policy on Fertility Reduction and FP: Extent to which government policy emphasizes FP for family size as it relates to available resources or demographic reasons over health reasons.

	1	2	3	4	5	6	7	8	9	10	Don't know
1-Lowest -> Highest-10	○	○	○	○	○	○	○	○	○	○	○

* FP 1.3 National FP Strategic Plan: Extent to which there is a current, comprehensive, strategic national plan for FP that outlines policies, laws, and regulations for the provisioning of FP. (In making this assessment, consider whether the plan defines a vision; includes multiyear strategies, annual timelines, and implementation plans; is evidence-based; has indicators to measure progress; and considers the FP market.)

	1	2	3	4	5	6	7	8	9	10	Don't know
1-Lowest -> Highest-10	○	○	○	○	○	○	○	○	○	○	○

* FP 1.4 Policy on Age at Marriage: Extent to which legal age at marriage for females is set at 18 years or higher and is enforced.

	1	2	3	4	5	6	7	8	9	10	Don't know
1-Lowest -> Highest-10	○	○	○	○	○	○	○	○	○	○	○

* FP 1.5 Evidence-Based Policymaking: Extent to which the appropriate ministry actively and regularly supports and uses key FP data and reports to engage in policy development and planning.

	1	2	3	4	5	6	7	8	9	10	Don't know
1-Lowest -> Highest-10	○	○	○	○	○	○	○	○	○	○	○

* FP 1.6 Monitoring and Evaluation of National Policies/Plans/Strategies: Extent to which the national FP plan outlines a comprehensive monitoring and evaluation strategy to track and measure accomplishments in the plan.

(In making this assessment, consider whether the monitoring and evaluation strategy includes goals and objectives, a results framework, measurable indicators [e.g., "SMART": specific, measurable, attainable, relevant and time-bound]; and an actionable monitoring and evaluation implementation plan/timeline.)

	1	2	3	4	5	6	7	8	9	10	Don't know
1-Lowest -> Highest-10	○	○	○	○	○	○	○	○	○	○	○

* FP 1.7 Inclusion of Nongovernmental and Cross-sectoral Actors in Policy/Strategy Development: Extent to which nongovernmental agencies, groups (e.g., from civil society), health workers, stakeholders (e.g., from faith-based, nonprofit, and private-for-profit organizations) and actors across non-FP-specific sectors are included in the national FP plan and/or other national documents outlining the country's strategies and systems related to planning, managing, and developing FP.

	1	2	3	4	5	6	7	8	9	10	Don't know
1-Lowest -> Highest-10	○	○	○	○	○	○	○	○	○	○	○

* FP 1.8 Financing for FP: Extent to which the government has established and applied guidelines for the costing and payments for FP across supply and demand considerations (i.e. domestic versus international funding – and its expectations for continuity/sustainability, cost of contraceptive supplies, cost of service delivery and transportation, etc.).

(In making this assessment, consider whether the guidelines and budget address the following: the lifespan of costs for FP care and services; variable structures of costs per FP method; costs of delivery and service provision by specialization, location and risk; costs and profits of healthcare/insurance schemes, the financial costs of providing FP weighed against the financing costs of not doing so.)

	1	2	3	4	5	6	7	8	9	10	Don't know
1-Lowest -> Highest-10	○	○	○	○	○	○	○	○	○	○	○

* FP 1.9 Percent of In-country Funding of FP Budget: Extent to which the total family planning/population budget is derived from in-country sources (e.g., 1 for 10 percent, 5 for 50 percent, 10 for 100 percent).

	1	2	3	4	5	6	7	8	9	10	Don't know
1-Lowest -> Highest-10											

* FP 1.10 Information/Communications Technology Infrastructure/Capacity: Extent to which there are adequate infrastructure components (e.g., computers, servers, printers, a local area network, Internet connectivity, and reliable electric power) as well as human resources (e.g., for maintenance and support) at the national and subnational levels to support the functioning of FP-related information systems.
(In making this assessment, consider whether the country conducts at least annual assessments of its information systems; has training curricula that cover data entry, use, software administration, and development; and continuously trains staff from key stakeholder institutions [e.g., Ministry of Health, Ministry of Education, regulatory agencies, training institutions].)

	1	2	3	4	5	6	7	8	9	10	Don't know
1-Lowest -> Highest-10											

* FP 1.11 Import Laws and Legal Regulations:

	1	2	3	4	5	6	7	8	9	10	Don't know
FP 1.11.1 Extent to which import laws and legal regulations facilitate the importation of FP commodities and supplies											
FP 1.11.2 Extent to which contraceptives are manufactured locally.											

* FP 1.12 Advertising of Contraceptives Allowed: Extent of freedom from restrictions on advertising of contraceptives in the mass media.

	1	2	3	4	5	6	7	8	9	10	Don't know
1-Lowest -> Highest-10											

* FP 1.13 Presence of Pro-Poor FP Policies: Extent to which policies specifically target the poor in rural or urban places.

	1	2	3	4	5	6	7	8	9	10	Don't know
1-Lowest -> Highest-10											

* FP 1.14 Political Support for FP: Extent to which elected officials in the country prioritize meeting FP needs to strengthen the FP contributions to the DD by passing laws and regulations and sponsoring actions and policies aimed at improving FP uptake and adherence.

	1	2	3	4	5	6	7	8	9	10	Don't know
1-Lowest -> Highest-10	◯	◯	◯	◯	◯	◯	◯	◯	◯	◯	◯

FP 1.15 Comments: Please use this section to provide any additional comments about the level of effort, challenges, or successes in the country around policies/policymaking for FP. If you marked “I don’t know” for any of the questions, also use this section to explain why you gave that rating

JOHNS HOPKINS
BLOOMBERG SCHOOL
of PUBLIC HEALTH

Bill & Melinda Gates Institute for Population and Reproductive Health

Demographic Dividend Effort Index Survey

Dimension 2. Services or Programs

* FP 2.1 Administrative Structure: Extent to which the administrative structure and staff at national, provincial and county levels are sufficient to implement the family planning program.

	1	2	3	4	5	6	7	8	9	10	Don't know
1-Lowest -> Highest-10	○	○	○	○	○	○	○	○	○	○	○

* FP 2.2 Level of Program Leaders: Relative level of seniority of the director of the national family planning program and whether said director reports to a high level of government.

	1	2	3	4	5	6	7	8	9	10	Don't know
1-Lowest -> Highest-10	○	○	○	○	○	○	○	○	○	○	○

* FP. 2.3 Community-Based Distribution: Extent to which areas of the country not easily serviced by clinics or other service points are covered by community-based distribution programs for distribution of contraceptive supplies (especially rural areas).

	1	2	3	4	5	6	7	8	9	10	Don't know
1-Lowest -> Highest-10	○	○	○	○	○	○	○	○	○	○	○

* FP 2.4 Postpartum Program: Extent to which all postpartum women receive postpartum family planning services.

	1	2	3	4	5	6	7	8	9	10	Don't know
1-Lowest -> Highest-10	○	○	○	○	○	○	○	○	○	○	○

* FP 2.5 Home-Visiting Workers: Extent of coverage of population by workers whose primary task is to visit (rural) women in their homes to talk about family planning and maternal and child health.

	1	2	3	4	5	6	7	8	9	10	Don't know
1-Lowest -> Highest-10	○	○	○	○	○	○	○	○	○	○	○

* FP 2.6 Availability and Accessibility of FP Commodities/Supplies and Services Part 1: Extent to which entire population has ready and easy access to (1-Lowest -> Highest-10):

	1	2	3	4	5	6	7	8	9	10	Don't know
FP 2.6.1 IUD	○	○	○	○	○	○	○	○	○	○	○
FP 2.6.2 Pills	○	○	○	○	○	○	○	○	○	○	○
FP 2.6.3 Injectables	○	○	○	○	○	○	○	○	○	○	○
FP 2.6.4 Implants	○	○	○	○	○	○	○	○	○	○	○
FP 2.6.5 Female Sterilization	○	○	○	○	○	○	○	○	○	○	○
FP 2.6.6 Male Sterilization	○	○	○	○	○	○	○	○	○	○	○
FP 2.6.7 Male Condoms	○	○	○	○	○	○	○	○	○	○	○
FP 2.6.8 Emergency Contraception	○	○	○	○	○	○	○	○	○	○	○
FP 2.6.9 Abortion/menstrual regulation (whether legal or not)	○	○	○	○	○	○	○	○	○	○	○
FP 2.6.10 Others (e.g. female condoms, foam, beads, etc.)	○	○	○	○	○	○	○	○	○	○	○

* FP 2.7 Availability and Accessibility of FP Commodities/Supplies and Services Part 2: How well does the supply system operate (it avoids stockouts or interrupted supplies and guarantees a reliable flow at local levels) for the following (1-Lowest -> Highest-10):

	1	2	3	4	5	6	7	8	9	10	Don't know
FP 2.7.1 IUD											
FP 2.7.2 Pills											
FP 2.7.3 Injectables											
FP 2.7.4 Implants											
FP 2.7.5 Male Condoms											
FP 2.7.6 Emergency Contraception											
FP 2.7.7 Others (e.g. female condoms, foam, beads, etc.)											

* FP 2.8 Availability and Accessibility of FP Commodities/Supplies and Services Part 3: How well does the supply system provide necessary equipment and medical supplies at clinical facilities for the following (1-Lowest -> Highest-10):

	1	2	3	4	5	6	7	8	9	10	Don't know
FP 2.8.1 Female Sterilization											
FP 2.8.2 Male Sterilization											
FP 2.8.3. IUDs											
FP 2.8.4 Implants											

* FP 2.9 FP Service Quality: Please rate the general quality of family planning services. (Good quality includes a focus on client needs, with counseling, full information, wide method choice, and safe clinical procedures.)

	1	2	3	4	5	6	7	8	9	10	Don't know
1-Lowest -> Highest-10											

* FP 2.10 Incentives and Disincentives: Extent to which monetary or other incentives are used to encourage the adoption of family planning.

	1	2	3	4	5	6	7	8	9	10	Don't know
1-Lowest -> Highest-10											

* FP 2.11 Adolescent Programs: Extent to which information, services and activities are focused towards adolescents and encourage adolescents to access contraceptive services.

	1	2	3	4	5	6	7	8	9	10	Don't know
1-Lowest -> Highest-10	○	○	○	○	○	○	○	○	○	○	○

* FP 2.12 Training Program: Extent to which training programs, for each category of staff in the family planning program, are adequate to provide personnel with information and skills necessary to carry out their jobs effectively.

	1	2	3	4	5	6	7	8	9	10	Don't know
1-Lowest -> Highest-10	○	○	○	○	○	○	○	○	○	○	○

* FP 2.13 Personnel Carry Out Assigned Tasks: Extent to which all categories of family planning program staff (administrative, medical, paramedical, field) carry out assigned tasks effectively

	1	2	3	4	5	6	7	8	9	10	Don't know
1-Lowest -> Highest-10	○	○	○	○	○	○	○	○	○	○	○

* FP 2.14 Logistics and Transport: Extent to which the logistics and transport systems are sufficient to keep stocks of contraceptive supplies and related equipment available at all service points, at all times and at all levels.

	1	2	3	4	5	6	7	8	9	10	Don't know
1-Lowest -> Highest-10	○	○	○	○	○	○	○	○	○	○	○

* FP 2.15 Supervision System: Extent to which the system of supervision at all levels is adequate (regular monitoring visits with corrective or supportive action).

	1	2	3	4	5	6	7	8	9	10	Don't know
1-Lowest -> Highest-10	○	○	○	○	○	○	○	○	○	○	○

* FP 2.16 Involvement of Other Ministries and Public Agencies: Extent to which other ministries, government agencies and non-governmental stakeholders engage with FP activities (e.g., delivery of supplies, services, information, education) or other population activities.

	1	2	3	4	5	6	7	8	9	10	Don't know
1-Lowest -> Highest-10	○	○	○	○	○	○	○	○	○	○	○

* FP 2.17 Involvement of Private-Sector Agencies and Groups: Extent to which private-sector agencies and groups assist with FP or other population activities.

	1	2	3	4	5	6	7	8	9	10	Don't know
1-Lowest -> Highest-10	○	○	○	○	○	○	○	○	○	○	○

* FP 2.18 Mass Media for Information Education Communication: Frequency and extent of coverage of mass media messages that provide the population with information on family planning and service sites.

	1	2	3	4	5	6	7	8	9	10	Don't know
1-Lowest -> Highest-10	○	○	○	○	○	○	○	○	○	○	○

* FP 2.19 Cultural and Social Norms Barriers: Extent to which cultural and social barriers are addressed in family planning programs.

	1	2	3	4	5	6	7	8	9	10	Don't know
1-Lowest -> Highest-10	○	○	○	○	○	○	○	○	○	○	○

* FP 2.20 Youth: Extent to which the government supports youth-led FP initiatives or involve youth in planning, implementation and evaluation of FP programs.

	1	2	3	4	5	6	7	8	9	10	Don't know
1-Lowest -> Highest-10	○	○	○	○	○	○	○	○	○	○	○

* FP 2.21 FP Effort Influences – Forces Affecting the FP Program: Forces affecting the FP program can either strengthen or detract from its effectiveness. Check zero (0) if there is no difference either way; check a negative number from -1 to -5 if the influence is negative; or check a positive number from 1 to 5 if the influence is positive. (1 in either direction means little influence and 5 in either direction means strong influence.)

	-5	-4	-3	-2	-1	0	1	2	3	4	5	Don't know
FP 2.21.1 Decentralization	○	○	○	○	○	○	○	○	○	○	○	○
FP 2.21.2 HIV/AIDS Programs	○	○	○	○	○	○	○	○	○	○	○	○
FP 2.21.3 Incorporation of FP into a broader context of reproductive health	○	○	○	○	○	○	○	○	○	○	○	○
FP 2.21.4 Integration of FP with other health services	○	○	○	○	○	○	○	○	○	○	○	○
FP 2.21.5 Changes in donor funding	○	○	○	○	○	○	○	○	○	○	○	○
FP 2.21.6 Changes in domestic government funding	○	○	○	○	○	○	○	○	○	○	○	○

FP 2.22 Comments: Please use this section to provide any additional comments about the level of effort, challenges, or successes in the country around services or programs for FP. If you marked “I don’t know” for any of the questions, also use this section to explain why you gave that rating

JOHNS HOPKINS
BLOOMBERG SCHOOL
of PUBLIC HEALTH

Bill & Melinda Gates Institute for Population and Reproductive Health

Demographic Dividend Effort Index Survey

Dimension 3. Advocacy

* FP 3.1 Influence of FP Leaders/Champions: Extent to which the country has one or more clearly influential leaders or champions who successfully advocate for FP (sectoral) needs at high levels, promote FP in the country by making positive public statements about it, and/or support actions and policies aimed at improving the FP environment and its contributions towards the DD.

	1	2	3	4	5	6	7	8	9	10	Don't know
1-Lowest -> Highest-10	◯	◯	◯	◯	◯	◯	◯	◯	◯	◯	◯

* FP 3.2 Statement by Leaders: Extent to which the head of government, as well as other officials, speak publicly and favorably about family planning at least once or twice a year.

	1	2	3	4	5	6	7	8	9	10	Don't know
1-Lowest -> Highest-10	◯	◯	◯	◯	◯	◯	◯	◯	◯	◯	◯

* FP 3.3 Media Coverage for FP: Extent to and frequency with which national media cover/broadcast FP topics that provides the population with information on family planning and service sites.

	1	2	3	4	5	6	7	8	9	10	Don't know
1-Lowest -> Highest-10	◯	◯	◯	◯	◯	◯	◯	◯	◯	◯	◯

* FP 3.4 FP Effort Influences - Justification: How important is each of the following as a current government justification for the national FP program? (1 means negligible importance; 10 means great importance.)

	1	2	3	4	5	6	7	8	9	10	Don't know
FP 3.4.1 Reduce the rate of population growth	○	○	○	○	○	○	○	○	○	○	○
FP 3.4.2 Enhance economic development	○	○	○	○	○	○	○	○	○	○	○
FP 3.4.3 Help women and men avoid unplanned and unwanted births	○	○	○	○	○	○	○	○	○	○	○
FP 3.4.4 Improve women's health	○	○	○	○	○	○	○	○	○	○	○
FP 3.4.5 Improve child health	○	○	○	○	○	○	○	○	○	○	○
FP 3.4.6 Reduce unmarried adolescent childbearing	○	○	○	○	○	○	○	○	○	○	○
FP 3.4.7 Reduce unmet need for contraceptive services	○	○	○	○	○	○	○	○	○	○	○

* FP 3.5 FP Effort Influences – Special Populations: To what extent does the family planning program give particular emphasis to special populations? (1 means negligible emphasis; 10 means great emphasis)

	1	2	3	4	5	6	7	8	9	10	Don't know
FP 3.5.1 Unmarried youth	○	○	○	○	○	○	○	○	○	○	○
FP 3.5.2 The poor	○	○	○	○	○	○	○	○	○	○	○
FP 3.5.3 The disabled	○	○	○	○	○	○	○	○	○	○	○
FP 3.5.4 Rural population	○	○	○	○	○	○	○	○	○	○	○
FP 3.5.5 Other vulnerable groups (e.g. minority and/or indigenous groups)	○	○	○	○	○	○	○	○	○	○	○
FP 3.5.6 Postpartum women for counseling and contraceptive services	○	○	○	○	○	○	○	○	○	○	○
FP 3.5.7 Post-abortion women for counseling and contraceptive services	○	○	○	○	○	○	○	○	○	○	○

* FP 3.6 Social Marketing: Extent of coverage of the country by a social marketing program (subsidized contraceptive sales at low cost in commercial sector, especially in urban areas).

	1	2	3	4	5	6	7	8	9	10	Don't know
1-Lowest -> Highest-10	○	○	○	○	○	○	○	○	○	○	○

FP 3.7 Comments: Please use this section to provide any additional comments about the level of effort, challenges, or successes in the country around advocacy for FP. If you marked “I don’t know” for any of the questions, also use this section to explain why you gave that rating.

JOHNS HOPKINS
BLOOMBERG SCHOOL of PUBLIC HEALTH

Bill & Melinda Gates Institute for Population and Reproductive Health

Demographic Dividend Effort Index Survey

Dimension 4. Research

* FP 4.1 Strength of Observatory/Stakeholder/Technical Working Group for FP: Extent to which country-level FP stakeholders (e.g., stakeholder leadership group, technical working group, FP observatory, country coordination and facilitation group):

	1	2	3	4	5	6	7	8	9	10	Don't know
FP 4.1.1 Have representation in government, training institutions, civil society, nongovernmental and faith-based organizations, professional associations, the private sector, etc.	○	○	○	○	○	○	○	○	○	○	○
FP 4.1.2 Meet regularly, report and recommend policy for senior management within the appropriate ministries	○	○	○	○	○	○	○	○	○	○	○
FP 4.1.3 Make an impact on FP within the country	○	○	○	○	○	○	○	○	○	○	○

* FP 4.2 Education/Research Strategy: Extent to which the national FP plan and/or other national documents include a comprehensive strategy/approach on FP-related research.

	1	2	3	4	5	6	7	8	9	10	Don't know
1-Lowest -> Highest-10	○	○	○	○	○	○	○	○	○	○	○

* FP 4.3 Data Gathering Partners:

	1	2	3	4	5	6	7	8	9	10	Don't know
FP 4.3.1 Extent to which research is undertaken by government agencies. (This may include statistical agencies/bureaus and/or ministries of economics/finances, education, health, gender, etc.)	○	○	○	○	○	○	○	○	○	○	○
FP 4.3.2 Extent to which research is undertaken by research institutions.	○	○	○	○	○	○	○	○	○	○	○
FP 4.3.3 Extent to which research is undertaken by independent researchers.	○	○	○	○	○	○	○	○	○	○	○

* FP 4.4 Topical Data Gathering:

	1	2	3	4	5	6	7	8	9	10	Don't know
FP 4.4.1 Extent to which family planning uptake (including method mix), demand, and unmet need is researched.	○	○	○	○	○	○	○	○	○	○	○
FP 4.4.2 Extent to which barriers to FP uptake is researched.	○	○	○	○	○	○	○	○	○	○	○
FP 4.4.3 Extent to which the impacts of FP programs are researched.	○	○	○	○	○	○	○	○	○	○	○
FP 4.4.4 Extent to which the quality of service provision for FP is researched.	○	○	○	○	○	○	○	○	○	○	○

* FP 4.5 Quality/Coverage of Data: Extent to which current research/data is sex and age disaggregated. Extent to which the country participates in standardized tests and its ranking.

	1	2	3	4	5	6	7	8	9	10	Don't know
FP 4.5.1 Extent to which a routine statistical system provides reliable periodic information on FP supplies and facilities.	◯	◯	◯	◯	◯	◯	◯	◯	◯	◯	◯
FP 4.5.2 Extent to which a routine statistical system provides good periodic information on FP services and personnel.	◯	◯	◯	◯	◯	◯	◯	◯	◯	◯	◯
FP 4.5.3 Extent to which the routine statistical system provides good periodic information on FP needs among populations in different communities.	◯	◯	◯	◯	◯	◯	◯	◯	◯	◯	◯
FP 4.5.4 Extent to which evaluations of the health monitoring and information system are conducted and applied to ensure the reliability of data.	◯	◯	◯	◯	◯	◯	◯	◯	◯	◯	◯

* FP 4.6 Record-Keeping: Extent to which systems for client recordkeeping, clinic reporting and feedback of results are adequate, including being connected at all levels (federal, state/province, county, etc.).

	1	2	3	4	5	6	7	8	9	10	Don't know
1-Lowest -> Highest-10	◯	◯	◯	◯	◯	◯	◯	◯	◯	◯	◯

* FP 4.7 Quality Research Institutions: Extent to which the country supports and sustains research institutions that develop research/collect data that support the breadth and quality of information-gathering to inform national FP efforts.

	1	2	3	4	5	6	7	8	9	10	Don't know
1-Lowest -> Highest-10	◯	◯	◯	◯	◯	◯	◯	◯	◯	◯	◯

* FP 4.8 Evaluation: Extent to which program statistics, surveys, and small studies are used by specialized staff to report on program operations and measure progress.

	1	2	3	4	5	6	7	8	9	10	Don't know
1-Lowest -> Highest-10	○	○	○	○	○	○	○	○	○	○	○

* FP 4.9 Management's Use of Evaluation Findings: Extent to which local-level program managers use research and evaluation findings to improve programming in ways suggested by findings.

	1	2	3	4	5	6	7	8	9	10	Don't know
1-Lowest -> Highest-10	○	○	○	○	○	○	○	○	○	○	○

* FP 4.10 Ministerial Use of Evaluation Findings: Extent to which the Ministry administrators/policymakers systematically utilize data to inform policies and interventions to address FP-related issues.

	1	2	3	4	5	6	7	8	9	10	Don't know
1-Lowest -> Highest-10	○	○	○	○	○	○	○	○	○	○	○

* FP 4.11 Dissemination of Information to Other Implementing Bodies: Extent to which information is shared or spread across geographical areas and throughout various levels (national, state/province/county, localities/sub-counties).

	1	2	3	4	5	6	7	8	9	10	Don't know
1-Lowest -> Highest-10	○	○	○	○	○	○	○	○	○	○	○

FP 4.12 Comments: Please use this section to provide any additional comments about the level of effort, challenges, or successes in the country around FP research. If you marked “I don't know” for any of the questions, also use this section to explain why you gave that rating.

JOHNS HOPKINS
BLOOMBERG SCHOOL
of PUBLIC HEALTH

Bill & Melinda Gates Institute for Population and Reproductive Health

Demographic Dividend Effort Index Survey

Dimension 5. Civil Society Organizations (CSOs)

* FP 5.1 CSO Actor Power: Extent to which CSO actors are in strong placements within institutions or technical working groups or networks concerned with FP.

	1	2	3	4	5	6	7	8	9	10	Don't know
1-Lowest -> Highest-10	○	○	○	○	○	○	○	○	○	○	○

* FP 5.2 Budget Analysis as a CSO Tool: Extent to which CSOs utilize budget analysis as a tool to build FP advocacy cases.

	1	2	3	4	5	6	7	8	9	10	Don't know
1-Lowest -> Highest-10	○	○	○	○	○	○	○	○	○	○	○

* FP 5.3 Facility-Based Service Delivery Support: Extent to which CSOs are involved in efforts to increase the use and/or quality of services, particularly through increasing the technical and/or operational capacity of CSO-partnered clinics to provide FP services.

	1	2	3	4	5	6	7	8	9	10	Don't know
1-Lowest -> Highest-10	○	○	○	○	○	○	○	○	○	○	○

* FP 5.4 Community-Based Service Delivery Support: Extent to which CSOs participate in bringing FP information and services to the communities where people live through activities such as supporting the capacity-building of community-level health workers, expanding the range of contraceptive methods provided by community health workers, educating lower cadre health workers, and/or undertaking information and education campaigns to increase the acceptability of contraception.

	1	2	3	4	5	6	7	8	9	10	Don't know
1-Lowest -> Highest-10	○	○	○	○	○	○	○	○	○	○	○

* FP 5.5 Mobile Outreach for Community-Based Service Delivery Support: Extent to which CSOs support outreach models to equip health care providers with FP commodities, supplies, equipment and vehicles, which are then sent as specialist team to provide FP information and methods directly to communities.

	1	2	3	4	5	6	7	8	9	10	Don't know
1-Lowest -> Highest-10	○	○	○	○	○	○	○	○	○	○	○

* FP 5.6 Social Franchising: Extent to which CSOs are involved in social franchising opportunities/services to bring private sector services to clients at reduced prices.

	1	2	3	4	5	6	7	8	9	10	Don't know
1-Lowest -> Highest-10	○	○	○	○	○	○	○	○	○	○	○

* FP 5.7 mHealth: Extent to which CSOs are leveraging mHealth to utilize text messages and other mobile technologies to improve access to FP information and services.

	1	2	3	4	5	6	7	8	9	10	Don't know
1-Lowest -> Highest-10	○	○	○	○	○	○	○	○	○	○	○

* FP 5.8 Human Rights and Quality of Care: Extent to which CSOs advocate for and support rights-based approaches to FP to ensure FP service recipients experience good treatment, confidence in asking questions, and quality counselling, amongst other concerns.

	1	2	3	4	5	6	7	8	9	10	Don't know
1-Lowest -> Highest-10	○	○	○	○	○	○	○	○	○	○	○

* FP 5.9 Social and Behavior Change (SBC): Extent to which CSOs support SBC interventions relating, but not limited, to life skills and peer education, adult health education, school-based education, couple's communication, community mobilization, working through gatekeepers, and/or mass media interventions for a general public audience. These may be supported through implementation at the community or individual levels and may relate to outcomes such as increase in FP uptake and changes in FP knowledge and attitudes.

	1	2	3	4	5	6	7	8	9	10	Don't know
1-Lowest -> Highest-10	○	○	○	○	○	○	○	○	○	○	○

* FP 5.10 Youth: Extent to which CSOs support FP policies, interventions and programming targeting youth specifically.

	1	2	3	4	5	6	7	8	9	10	Don't know
1-Lowest -> Highest-10	○	○	○	○	○	○	○	○	○	○	○

* FP 5.11 Programming for Men: Extent to which CSOs support FP policies, interventions and programming for men.

	1	2	3	4	5	6	7	8	9	10	Don't know
1-Lowest -> Highest-10	◯	◯	◯	◯	◯	◯	◯	◯	◯	◯	◯

* FP 5.12 Advocacy/Accountability: Extent to which CSOs support the training and capacity building of advocates and community members to understand policies, processes, and activities to increase community-led issue identification, collaborative problem solving and more targeted advocacy actions.

	1	2	3	4	5	6	7	8	9	10	Don't know
1-Lowest -> Highest-10	◯	◯	◯	◯	◯	◯	◯	◯	◯	◯	◯

* FP 5.13 CSO-Led Assessment and Monitoring: Extent to which CSOs assess, monitor and report on the effectiveness of policies and programming to improve accountability at the policy and provider levels.

	1	2	3	4	5	6	7	8	9	10	Don't know
1-Lowest -> Highest-10	◯	◯	◯	◯	◯	◯	◯	◯	◯	◯	◯

* FP 5.14 Forming Partnerships between CSOs: Extent to which CSOs have formed national alliances and regional partnerships to strengthen their positioning and potentially improve leadership strength and funding mechanisms.

	1	2	3	4	5	6	7	8	9	10	Don't know
1-Lowest -> Highest-10	◯	◯	◯	◯	◯	◯	◯	◯	◯	◯	◯

FP 5.15 Comments: Please use this section to provide any additional comments about the level of effort, challenges, or successes in the country around the role of the civil society in FP. If you marked “I don’t know” for any of the questions, also use this section to explain why you gave that rating.

JOHNS HOPKINS
BLOOMBERG SCHOOL
of PUBLIC HEALTH

Bill & Melinda Gates Institute for Population and Reproductive Health

Demographic Dividend Effort Index Survey

Module: Sectoral Resilience and Sustainability

Considering the health and socio-economic impact of the COVID-19 and the likely impact on the DD progress, this DD effort index has integrated questions to assess the resilience and sustainability of systems in key sectors of the DD framework. The items included in the COVID-19 related questionnaire below follow the key dimensions of resilient systems and provide critical information on the potential of an effective response to emerging infectious disease threats or other public health emergencies.

Please score each item from 1 to 10, where 1 represents the lowest score (poor state/capability) and 10 represents the highest score (great state/capability).

* Physical: facilities, equipment, system states, and capabilities.

	1	2	3	4	5	6	7	8	9	10	Don't know
FP-M1 - Plan/Prepare - State and capability of equipment, personnel and FP program structure prior to the crisis event. (1 = poor state/capability; 10 = great state/capability)	○	○	○	○	○	○	○	○	○	○	○
FP-M2 - Absorb - Integration: The level of integration of FP services and programs to mitigate the effect of COVID-19 on FP	○	○	○	○	○	○	○	○	○	○	○
FP-M3 - Absorb - Integration: Extent to which effective supply chain management was able to mitigate the effect of COVID-19 on the supply/provision of FP commodities.	○	○	○	○	○	○	○	○	○	○	○
FP-M4 - Recover - Extent to which the FP sector has been able to induce change to recover the pre-COVID-19 level of functionality.	○	○	○	○	○	○	○	○	○	○	○
FP-M5 - Recover - Financing and Donor Management: Level of resource mobilization and allocation to mitigate the effect of COVID-19 on FP.	○	○	○	○	○	○	○	○	○	○	○
FP-M6 - Adapt - Extent to which changes have been made to improve the resilience of the FP sector.	○	○	○	○	○	○	○	○	○	○	○

* Information: creation, manipulation, storage, and utilization of data.

	1	2	3	4	5	6	7	8	9	10	Don't know
FP-M7 - Plan/Prepare - Extent to which FP-related data was sufficiently prepared, presented, analyzed and stored prior to the crisis event.	○	○	○	○	○	○	○	○	○	○	○
FP-M8 - Absorb - Extent to which the FP program has been able to conduct a real-time assessment of functionality, in anticipation of cascading losses in FP service provision.	○	○	○	○	○	○	○	○	○	○	○
FP-M9 - Recover - Extent to which FP data has been used to track the progress of recovery and to anticipate recovery scenarios.	○	○	○	○	○	○	○	○	○	○	○
FP-M10 - Adapt - Extent to which the FP sector is creating and improving FP data storage and real-time use protocols.	○	○	○	○	○	○	○	○	○	○	○

* Cognitive: understanding, mental models, preconceptions, biases, and values.

	1	2	3	4	5	6	7	8	9	10	Don't know
FP-M11 - Plan/Prepare - Extent to which the FP program design and operation decisions were prepared in anticipation of crisis events.	○	○	○	○	○	○	○	○	○	○	○
FP-M12 - Absorb - Extent to which the FP sector is responding with sufficient contingency protocols and proactive crisis management.	○	○	○	○	○	○	○	○	○	○	○
FP-M13 - Absorb - Accountability: Level of effort to ensure optimal accountability for the resources allocated to support FP as part of the response to the COVID-19.	○	○	○	○	○	○	○	○	○	○	○
FP-M14 - Recover - Extent to which decisions are oriented towards recovery, which employ evidence-based communication to community members to promote safe behavior.	○	○	○	○	○	○	○	○	○	○	○
FP-M15 - Recover - Awareness: Extent to which the FP sector is building confidence through communication regarding barriers posed by COVID-19 to support access to and uptake of FP.	○	○	○	○	○	○	○	○	○	○	○
FP-M16 - Adapt - Adaptive: Extent to which the national leadership has made changes to adapt and respond to the COVID-19 threats to FP.	○	○	○	○	○	○	○	○	○	○	○

* Social: interaction, collaboration, and self-synchronization between individuals, entities and institutions.

	1	2	3	4	5	6	7	8	9	10	Don't know
FP-M17 - Plan/Prepare - Extent to which training on outbreak/crisis management was conducted through, and leveraged, social networks, social capital and institutional and cultural norms prior to the crisis event.	○	○	○	○	○	○	○	○	○	○	○
FP-M18 - Absorb - Extent to which personnel and social institutions have been resourceful and accessible for the outbreak/crisis response.	○	○	○	○	○	○	○	○	○	○	○
FP-M19 - Absorb - Self-Regulating: Extent to which the national leadership had the authority to make sectoral changes in a timely manner through flexible infrastructure.	○	○	○	○	○	○	○	○	○	○	○
FP-M20 - Recover - Extent to which the FP sector is engaging with team and knowledge sharing to enhance systematic recovery.	○	○	○	○	○	○	○	○	○	○	○
FP-M21 - Recover - Diversity: The level of engagement of a multidisciplinary team to mitigate the effects of COVID-19 on FP.	○	○	○	○	○	○	○	○	○	○	○
FP-M22 - Adapt - Extent to which there are additions or changes being made to FP institutions, policies, trainings, programs and culture.	○	○	○	○	○	○	○	○	○	○	○
FP-M23 - Adapt - Leadership and Management: Level of leadership demonstrated by the FP leaders to mitigate the effect of COVID-19 on FP.	○	○	○	○	○	○	○	○	○	○	○

* FP M-24 Timeline: Extent of timeliness of the Government reaction - communication and implementation of measures - to mitigate the immediate and longterm impact of COVID-19.

Note: This refers to the entirety of the national reaction - not the reaction that is specific to your sector.

	1	2	3	4	5	6	7	8	9	10	Don't know
1-Lowest -> Highest-10	◯	◯	◯	◯	◯	◯	◯	◯	◯	◯	◯

FP M-25 Comments: Please use this section to provide any additional comments about the resilience and sustainability of the national FP systems based on your experience during the COVID-19 pandemics. If you marked “I don’t know” for any of the questions, also use this section to explain why you gave that rating.

JOHNS HOPKINS
BLOOMBERG SCHOOL of PUBLIC HEALTH

Bill & Melinda Gates Institute for Population and Reproductive Health

Demographic Dividend Effort Index Survey

Maternal & Child Health (MCH) Questionnaire

Please respond to the following questions based on your experience/expertise within this specific sector.

To give a summary picture of the national demographic dividend effort, please rate the following items. Score each item from 1 to 10, where 1 represents the lowest score (very weak or no effort) and 10 represents the highest score (strong effort). Where applicable, if a policy or activity is non-existent then mark your response as 0.

Answer each item. All responses will be recorded in the format exemplified below:

Lowest effort	1	2	3	4	5	6	7	8	9	10	Highest effort	I do not know

JOHNS HOPKINS
BLOOMBERG SCHOOL
of PUBLIC HEALTH

Bill & Melinda Gates Institute for Population and Reproductive Health

Demographic Dividend Effort Index Survey

Dimension 1. Policy/Policy making

* MCH 1.1 Adequate Policy: Ministry of Health policies toward pregnancy and delivery services that account for the breadth of requirements to ensure that every mother and newborn can survive.

	1	2	3	4	5	6	7	8	9	10	Don't know
MCH 1.1.1 Extent to which reproductive, maternal and child health has been integrated into the national health policy/plan/strategy.	○	○	○	○	○	○	○	○	○	○	○
MCH 1.1.2 Extent to which national reproductive, maternal and child health strategy(ies) and implementation plan(s) have been made available.	○	○	○	○	○	○	○	○	○	○	○

* MCH 1.2 Universal Access: Extent to which there are constitutional and legal entitlements that facilitate universal access to reproductive, maternal and child health services.

	1	2	3	4	5	6	7	8	9	10	Don't know
1-Lowest -> Highest-10	○	○	○	○	○	○	○	○	○	○	○

* MCH 1.3 Multisector Involvement: Extent to which policies are developed through adequate consultation with interested parties such as other ministries, NGOs, private practitioners, and women’s groups.

	1	2	3	4	5	6	7	8	9	10	Don't know
1-Lowest -> Highest-10	○	○	○	○	○	○	○	○	○	○	○

* MCH 1.4 Competent Service Providers: Extent to which policies are reasonable and evidence-based concerning which personnel can provide maternal health services (e.g. trained midwives can perform a wide range of medical procedures).

	1	2	3	4	5	6	7	8	9	10	Don't know
1-Lowest -> Highest-10	○	○	○	○	○	○	○	○	○	○	○

* MCH 1.5 Abortion Legislation: Extent to which a favorable policy exists towards the legal provisioning of abortion services.

	1	2	3	4	5	6	7	8	9	10	Don't know
1-Lowest -> Highest-10	○	○	○	○	○	○	○	○	○	○	○

* MCH 1.6. Post-Abortion Care Legislation: Extent to which a favorable policy exists toward the treatment of complications of abortions, including complications seen from illegal abortions.

	1	2	3	4	5	6	7	8	9	10	Don't know
1-Lowest -> Highest-10	○	○	○	○	○	○	○	○	○	○	○

* MCH 1.7 Policy Reviews and Updates: Extent to which policies are regularly reviewed by high-level policymakers and reviews are used to update action plans.

	1	2	3	4	5	6	7	8	9	10	Don't know
1-Lowest -> Highest-10	○	○	○	○	○	○	○	○	○	○	○

* MCH 1.8 High-Level Actor Placement: Extent and level to which the director of maternal and child health services is involved in high-level decision making.

	1	2	3	4	5	6	7	8	9	10	Don't know
1-Lowest -> Highest-10	○	○	○	○	○	○	○	○	○	○	○

* MCH 1.9 Financing for MCH: Extent to which the government budgets for safe pregnancy, delivery, perinatal care (for facilities, personnel, supplies, etc.) and children's health is adequate for the needs, whether from the Ministry of Health, provincial/ county government or donor support.

	1	2	3	4	5	6	7	8	9	10	Don't know
1-Lowest -> Highest-10	○	○	○	○	○	○	○	○	○	○	○

* MCH 1.10 Affordability: Extent to which all MCH-related services and drugs are affordable to all clients.

	1	2	3	4	5	6	7	8	9	10	Don't know
1-Lowest -> Highest-10	○	○	○	○	○	○	○	○	○	○	○

* MCH 1.11 Private Sector: Extent to which the private sector (doctors, midwives, clinics) is active and covers a substantial share of pregnancy and delivery cases.

	1	2	3	4	5	6	7	8	9	10	Don't know
1-Lowest -> Highest-10	◯	◯	◯	◯	◯	◯	◯	◯	◯	◯	◯

* MCH 1.12 MCH Department: Extent to which the Ministry of Health has a department that is specifically devoted to MCH.

	1	2	3	4	5	6	7	8	9	10	Don't know
1-Lowest -> Highest-10	◯	◯	◯	◯	◯	◯	◯	◯	◯	◯	◯

* MCH 1.13 Potential Solutions: Extent to which the government recognizes potential solutions to MCH-related issues through the means of:

	1	2	3	4	5	6	7	8	9	10	Don't know
MCH 1.13.1 Regulatory efficiency	◯	◯	◯	◯	◯	◯	◯	◯	◯	◯	◯
MCH 1.13.2 Performance and accountability	◯	◯	◯	◯	◯	◯	◯	◯	◯	◯	◯
MCH 1.13.3 A human rights-based approach to maternal, newborn and child health	◯	◯	◯	◯	◯	◯	◯	◯	◯	◯	◯
MCH 1.13.4 Essential health infrastructure	◯	◯	◯	◯	◯	◯	◯	◯	◯	◯	◯
MCH 1.13.5 Community engagement	◯	◯	◯	◯	◯	◯	◯	◯	◯	◯	◯

MCH 1.14 Comments: Please use this section to provide any additional comments about the level of effort, challenges, or successes in the country around policies/policymaking for MCH. If you marked “I don’t know” for any of the questions, also use this section to explain why you gave that rating

JOHNS HOPKINS
BLOOMBERG SCHOOL
of PUBLIC HEALTH

Bill & Melinda Gates Institute for Population and Reproductive Health

Demographic Dividend Effort Index Survey

Dimension 2. Services or Programs

* MCH 2.1 Obstetric Care Availability – All Centers: Extent to which all primary health facilities have trained staff, in place, who can provide obstetric care to:

	1	2	3	4	5	6	7	8	9	10	Don't know
MCH 2.1.1 Properly manage postpartum hemorrhage cases.											
MCH 2.1.2 Timely and properly administer antibiotics intravenously when needed.											
MCH 2.1.3 Perform manual removal of retained placenta.											
MCH 2.1.4 Perform vacuum aspiration of the uterus, using MVA (manual vacuum aspiration) or an electric suction device.											
MCH 2.1.5 Use a partograph to determine when to refer.											
MCH 2.1.6 Have transportation arrangements to quickly move a woman with obstructed labor to a district/sub-county hospital.											
MCH 2.1.7 Have adequate antibiotic supplies on hand (sufficient supplies of the correct types).											

* MCH 2.2 Obstetric Care Availability – Secondary Healthcare Facilities: Extent to which all district/sub-county hospitals have trained staff, in place, who can:

	1	2	3	4	5	6	7	8	9	10	Don't know
MCH 2.2.1 Provide all functions listed above for primary health facilities.											
MCH 2.2.2 Perform blood transfusions (and have adequate supplies of safe blood on hand).											
MCH 2.2.3 Perform Cesarean section.											

* MCH 2.3 Pregnancy-Related Services: Extent to which all pregnant women have adequate access to:

	1	2	3	4	5	6	7	8	9	10	Don't know
MCH 2.3.1 Treatment for postpartum hemorrhage during or soon after delivery.											
MCH 2.3.2 Management of obstructed labor.											
MCH 2.3.3 Management of pre-eclampsia, eclampsia and its complications.											
MCH 2.3.4 Treatment of post abortion care or for abortion complications.											
MCH 2.3.5 Provision of safe abortion services.											
MCH 2.3.6 Antenatal care during pregnancy.											
MCH 2.3.7 Delivery care by a trained professional attendant.											
MCH 2.3.8 Postpartum family planning services.											
MCH 2.3.9 District/sub-county hospitals that are open 24 hours/day.											

* MCH 2.4 Ante-natal Care for Unmarried and HIV Positive Women: Extent to which, at all antenatal visits, all pregnant women:

	1	2	3	4	5	6	7	8	9	10	Don't know
MCH 2.4.1 Receive WHO-recommended complete antenatal care protocol for HIV negative women	○	○	○	○	○	○	○	○	○	○	○
MCH 2.4.2 Receive WHO-recommended complete antenatal care protocol for HIV positive women	○	○	○	○	○	○	○	○	○	○	○
MCH 2.4.3 Receive WHO-recommended complete antenatal care protocol for young unmarried women.	○	○	○	○	○	○	○	○	○	○	○
MCH 2.4.4 Are informed about danger signs of obstetric and newborn complications and are assisted in planning for any emergency.	○	○	○	○	○	○	○	○	○	○	○

* MCH 2.5 Newborn Care: Extent to which, for newborn care, all infants whether delivered at home or in a facility:

	1	2	3	4	5	6	7	8	9	10	Don't know
MCH 2.5.1 Have their mouth and nasal passageways cleared	○	○	○	○	○	○	○	○	○	○	○
MCH 2.5.2 Are dried and kept warm immediately after birth	○	○	○	○	○	○	○	○	○	○	○
MCH 2.5.3 Receive vitamin A	○	○	○	○	○	○	○	○	○	○	○
MCH 2.5.4 Have their umbilical cord cut with a clean blade	○	○	○	○	○	○	○	○	○	○	○
MCH 2.5.5 Complete WHO recommended vaccination calendar	○	○	○	○	○	○	○	○	○	○	○
MCH 2.5.6 Benefit from exclusive breastfeeding promotion and education activities	○	○	○	○	○	○	○	○	○	○	○
MCH 2.5.7 Benefit from nutritional support programs when in need.	○	○	○	○	○	○	○	○	○	○	○

* MCH 2.6 Family Planning – All Centers: Extent to which there exists proper provision of family planning at all health centers that:

	1	2	3	4	5	6	7	8	9	10	Don't know
MCH 2.6.1 Routinely offer family planning services for post-abortion cases	◯	◯	◯	◯	◯	◯	◯	◯	◯	◯	◯
MCH 2.6.2 Routinely offer family planning at postpartum visits	◯	◯	◯	◯	◯	◯	◯	◯	◯	◯	◯
MCH 2.6.3 Have short-term and LARC contraceptive supplies regularly in stock	◯	◯	◯	◯	◯	◯	◯	◯	◯	◯	◯
MCH 2.6.4 Have trained staff, in place, who can provide services for LARCs.	◯	◯	◯	◯	◯	◯	◯	◯	◯	◯	◯
MCH 2.6.5 Have guidelines and protocols necessary for the provision of family planning services.	◯	◯	◯	◯	◯	◯	◯	◯	◯	◯	◯

* MCH 2.7 Family Planning – District/Sub-county Hospitals: Extent to which there exists proper provision of family planning at district/sub-county hospitals that:

	1	2	3	4	5	6	7	8	9	10	Don't know
MCH 2.7.1 Routinely offer family planning services for post-abortion cases	○	○	○	○	○	○	○	○	○	○	○
MCH 2.7.2 Routinely offer family planning at postpartum visits	○	○	○	○	○	○	○	○	○	○	○
MCH 2.7.3 Have short term and LARC contraceptive supplies regularly in stock	○	○	○	○	○	○	○	○	○	○	○
MCH 2.7.4 Have trained staff, in place, who can provide services for LARCs	○	○	○	○	○	○	○	○	○	○	○
MCH 2.7.5 Can offer sterilization to female clients	○	○	○	○	○	○	○	○	○	○	○
MCH 2.7.6 Can offer sterilization to male clients	○	○	○	○	○	○	○	○	○	○	○
MCH 2.7.7 Have guidelines and protocols necessary for the provision of family planning services.	○	○	○	○	○	○	○	○	○	○	○

* MCH 2.8 Childhood Illness: Extent to which the WHO-recommended case management of childhood illness is implemented regarding:

	1	2	3	4	5	6	7	8	9	10	Don't know
MCH 2.8.1 Community case management of childhood illnesses	○	○	○	○	○	○	○	○	○	○	○
MCH 2.8.2 Effectiveness of the referral services from community level to the health facilities	○	○	○	○	○	○	○	○	○	○	○

MCH 2.9 Comments: Please use this section to provide any additional comments about the level of effort, challenges, or successes in the country around services or programs for MCH. If you marked “I don’t know” for any of the questions, also use this section to explain why you gave that rating.

JOHNS HOPKINS
BLOOMBERG SCHOOL
of PUBLIC HEALTH

Bill & Melinda Gates Institute for Population and Reproductive Health

Demographic Dividend Effort Index Survey

Dimension 3. Advocacy

* MCH 3.1 Government Messages: Extent to which high officials in the government, including the Ministry of Health, issue frequent statements to all stakeholders including the press to support improvements for safe pregnancy, safe delivery and healthy childhood.

	1	2	3	4	5	6	7	8	9	10	Don't know
1-Lowest -> Highest-10											

* MCH 3.2 Mass Media: Extent to which the national program uses the mass media to educate the public about symptoms of pregnancy complications, safe childbirth and healthy childhood.

	1	2	3	4	5	6	7	8	9	10	Don't know
1-Lowest -> Highest-10											

* MCH 3.3 Community-Level Media: Extent to which community groups take part in systematic programs to educate the public about safe pregnancy, safe delivery and healthy childhood.

	1	2	3	4	5	6	7	8	9	10	Don't know
1-Lowest -> Highest-10											

* MCH 3.4 Educational Materials: Extent to which the appropriate ministry (MOH) supplies adequate educational materials (posters, pamphlets, etc.) to delivery facilities to instruct clients about safe practices.

	1	2	3	4	5	6	7	8	9	10	Don't know
1-Lowest -> Highest-10											

* MCH 3.5 Self-Review of Maternal Mortality Cases: Extent to which each health facility follows a regular procedure to review and learn from every case of a maternal death in the facility.

	1	2	3	4	5	6	7	8	9	10	Don't know
1-Lowest -> Highest-10	◯	◯	◯	◯	◯	◯	◯	◯	◯	◯	◯

* MCH 3.6 Informing Policies with New Evidence: Extent to which each health facility shares lessons from maternal death cases to the ministries to inform new policies or interventions.

	1	2	3	4	5	6	7	8	9	10	Don't know
1-Lowest -> Highest-10	◯	◯	◯	◯	◯	◯	◯	◯	◯	◯	◯

MCH 3.7 Comments: Please use this section to provide any additional comments about the level of effort, challenges, or successes in the country around advocacy for MCH. If you marked “I don’t know” for any of the questions, also use this section to explain why you gave that rating

JOHNS HOPKINS
BLOOMBERG SCHOOL
of PUBLIC HEALTH

Bill & Melinda Gates Institute for Population and Reproductive Health

Demographic Dividend Effort Index Survey

Dimension 4. Research

* MCH 4.1 Strength of Observatory/Stakeholder/Technical Working Group for MCH: Extent to which country-level MCH stakeholders (e.g., stakeholder leadership group, technical working group, MCH observatory, country coordination and facilitation group):

	1	2	3	4	5	6	7	8	9	10	Don't know
MCH 4.1.1 Have representation in government, training institutions, civil society, nongovernmental and faith-based organizations, professional associations, the private sector, etc.	○	○	○	○	○	○	○	○	○	○	○
MCH 4.1.2 Meet regularly, report and recommend policy for senior management within the appropriate ministries	○	○	○	○	○	○	○	○	○	○	○
MCH 4.1.3 Make an impact on MCH within the country	○	○	○	○	○	○	○	○	○	○	○

* MCH 4.2 MCH Research Strategy: Extent to which national documents include a comprehensive strategy/approach on MCH-related research and is shared among partners.

	1	2	3	4	5	6	7	8	9	10	Don't know
1-Lowest -> Highest-10	○	○	○	○	○	○	○	○	○	○	○

* MCH 4.3 Data-Gathering Partners:

	1	2	3	4	5	6	7	8	9	10	Don't know
MCH 4.3.1 Extent to which research is undertaken by government agencies. (This may include statistical agencies/bureaus and/or ministries of economics/finances, education, health, gender, etc.)	○	○	○	○	○	○	○	○	○	○	○
MCH 4.3.2 Extent to which data-gathering is undertaken by research institutions.	○	○	○	○	○	○	○	○	○	○	○
MCH 4.3.3 Extent to which data-gathering is undertaken by independent researchers.	○	○	○	○	○	○	○	○	○	○	○

* MCH 4.4 Topical Research:

	1	2	3	4	5	6	7	8	9	10	Don't know
MCH 4.4.1 Extent to which maternal mortality is researched.	○	○	○	○	○	○	○	○	○	○	○
MCH 4.4.2 Extent to which infant and child mortality is researched.	○	○	○	○	○	○	○	○	○	○	○
MCH 4.4.3 Extent to which family planning and post-partum family planning is researched.	○	○	○	○	○	○	○	○	○	○	○
MCH 4.4.4 Extent to which MCH service provision/access and provider quality is researched.	○	○	○	○	○	○	○	○	○	○	○

* MCH 4.5 Quality/Coverage of Data: Extent to which current research/data is disaggregated, where relevant, on the basis of sex, age, geographic location, ethnicity, education, income quintile and disability status. Extent to which the country participates in standardized tests and its ranking.

	1	2	3	4	5	6	7	8	9	10	Don't know
MCH 4.5.1 Extent to which a routine statistical system provides good periodic information on supplies and facilities.	○	○	○	○	○	○	○	○	○	○	○
MCH 4.5.2 Extent to which a routine statistical system provides good periodic information on MCH services and personnel.	○	○	○	○	○	○	○	○	○	○	○
MCH 4.5.3 Extent to which the routine statistical system provides good periodic information on MCH needs among populations in different communities.	○	○	○	○	○	○	○	○	○	○	○
MCH 4.5.4 Extent to which evaluations of the health monitoring and information system are conducted and applied to ensure the reliability of data.	○	○	○	○	○	○	○	○	○	○	○
MCH 4.5.5 Extent to which evaluations of the health monitoring and information system are conducted and applied to explore inequalities in MNCH services and burdens.	○	○	○	○	○	○	○	○	○	○	○

* MCH 4.6 Record-Keeping: Extent to which systems for client recordkeeping, clinic reporting and feedback of results are adequate, including being connected at all levels (federal, state/province, county, etc.).

	1	2	3	4	5	6	7	8	9	10	Don't know
1-Lowest -> Highest-10	○	○	○	○	○	○	○	○	○	○	○

* MCH 4.7 Quality Research Institutions: Extent to which the country has the capacity to support and sustain research institutions that develop research/collect data to ensure high quality MCH services.

	1	2	3	4	5	6	7	8	9	10	Don't know
1-Lowest -> Highest-10	◯	◯	◯	◯	◯	◯	◯	◯	◯	◯	◯

* MCH 4.8 Evaluation: Extent to which program statistics, surveys, and small studies are used by specialized staff to report on program operations and measure progress.

	1	2	3	4	5	6	7	8	9	10	Don't know
1-Lowest -> Highest-10	◯	◯	◯	◯	◯	◯	◯	◯	◯	◯	◯

* MCH 4.9 Management's Use of Evaluation Findings: Extent to which local-level program managers use research and evaluation findings to improve programming in ways suggested by findings.

	1	2	3	4	5	6	7	8	9	10	Don't know
1-Lowest -> Highest-10	◯	◯	◯	◯	◯	◯	◯	◯	◯	◯	◯

* MCH 4.10 Ministerial Use of Evaluation Findings: Extent to which the Ministry administrators and policymakers systematically utilize data to inform policies and interventions to address MCH-related issues.

	1	2	3	4	5	6	7	8	9	10	Don't know
1-Lowest -> Highest-10	◯	◯	◯	◯	◯	◯	◯	◯	◯	◯	◯

* MCH 4.11 Dissemination of Information to Other Implementing Bodies: Extent to which information is shared or spread across geographical areas and throughout various levels (national, state/province, localities).

	1	2	3	4	5	6	7	8	9	10	Don't know
1-Lowest -> Highest-10	◯	◯	◯	◯	◯	◯	◯	◯	◯	◯	◯

MCH 4.12 Comments: Please use this section to provide any additional comments about the level of effort, challenges, or successes in the country around MCH research. If you marked “I don’t know” for any of the questions, also use this section to explain why you gave that rating.

JOHNS HOPKINS
BLOOMBERG SCHOOL
of PUBLIC HEALTH

Bill & Melinda Gates Institute for Population and Reproductive Health

Demographic Dividend Effort Index Survey

Dimension 5. Civil Society Organizations (CSOs)

* MCH 5.1 CSO Actor Power: Extent to which CSO actors are in strong placements within institutions or technical working groups or networks concerned with MCH.

	1	2	3	4	5	6	7	8	9	10	Don't know
1-Lowest -> Highest-10	○	○	○	○	○	○	○	○	○	○	○

* MCH 5.2 Budget Analysis as a CSO Tool: Extent to which CSOs utilize budget analysis as a tool to build MCH advocacy cases.

	1	2	3	4	5	6	7	8	9	10	Don't know
1-Lowest -> Highest-10	○	○	○	○	○	○	○	○	○	○	○

* MCH 5.3 Facility-Based Service Delivery Support: Extent to which CSOs are involved in efforts to increase the use and/or quality of services, particularly through increasing the technical and/or operational capacity of CSO-partnered clinics to provide MCH services.

	1	2	3	4	5	6	7	8	9	10	Don't know
1-Lowest -> Highest-10	○	○	○	○	○	○	○	○	○	○	○

* MCH 5.4 Community-Based Service Delivery Support: Extent to which CSOs participate in bringing MCH information and services to the communities where people live through activities such as supporting the capacity-building of community-level health workers, expanding the range of MCH services provided by community health workers, educating lower cadre health workers, and/or undertaking information and education campaigns.

	1	2	3	4	5	6	7	8	9	10	Don't know
1-Lowest -> Highest-10	○	○	○	○	○	○	○	○	○	○	○

* MCH 5.5 Mobile Outreach for Community-Based Service Delivery Support: Extent to which CSOs support outreach models to equip health care providers with MCH-related commodities, supplies, equipment and vehicles, which are then sent as specialist team to provide MCH services directly to communities.

	1	2	3	4	5	6	7	8	9	10	Don't know
1-Lowest -> Highest-10	○	○	○	○	○	○	○	○	○	○	○

* MCH 5.6 Social Franchising: Extent to which CSOs are involved with social franchising opportunities/services to bring private sector services to clients at reduced prices of MCH services.

	1	2	3	4	5	6	7	8	9	10	Don't know
1-Lowest -> Highest-10	○	○	○	○	○	○	○	○	○	○	○

* MCH 5.7 mHealth: Extent to which CSOs are leveraging mHealth to utilize text messages and other mobile technologies to improve access to MCH information and services.

	1	2	3	4	5	6	7	8	9	10	Don't know
1-Lowest -> Highest-10	○	○	○	○	○	○	○	○	○	○	○

* MCH 5.8 Human Rights and Quality of Care: Extent to which CSOs advocate for and support rights-based approaches to MCH to ensure MCH service recipients experience good treatment, confidence in asking questions, and quality counselling, amongst other concerns.

	1	2	3	4	5	6	7	8	9	10	Don't know
1-Lowest -> Highest-10	○	○	○	○	○	○	○	○	○	○	○

* MCH 5.9 Social and Behavior Change (SBC): Extent to which CSOs support SBC interventions relating, but not limited, to life skills and peer education, adult health education, school-based education, couple's communication, community mobilization, working through gatekeepers, and/or mass media interventions for a general public audience.

	1	2	3	4	5	6	7	8	9	10	Don't know
1-Lowest -> Highest-10	○	○	○	○	○	○	○	○	○	○	○

* MCH 5.10 Advocacy/Accountability: Extent to which CSOs support the training and capacity building of advocates and community members to understand policies, processes, and activities to increase community-led issue identification, collaborative problem solving and more targeted advocacy actions.

	1	2	3	4	5	6	7	8	9	10	Don't know
1-Lowest -> Highest-10	○	○	○	○	○	○	○	○	○	○	○

* MCH 5.11 CSO-Led Assessment and Monitoring: Extent to which CSOs assess, monitor and report on the effectiveness of policies and programming to improve accountability at the policy and provider levels.

	1	2	3	4	5	6	7	8	9	10	Don't know
1-Lowest -> Highest-10	○	○	○	○	○	○	○	○	○	○	○

* MCH 5.12 Forming Partnerships between CSOs: Extent to which CSOs have formed national alliances and regional partnerships to strengthen their positioning and potentially improve leadership strength and funding mechanisms for MCH.

	1	2	3	4	5	6	7	8	9	10	Don't know
1-Lowest -> Highest-10	○	○	○	○	○	○	○	○	○	○	○

MCH 5.13 Comments: Please use this section to provide any additional comments about the level of effort, challenges, or successes in the country around the involvement of the civil society in MCH. If you marked “I don’t know” for any of the questions, also use this section to explain why you gave that rating

JOHNS HOPKINS
BLOOMBERG SCHOOL
of PUBLIC HEALTH

Bill & Melinda Gates Institute for Population and Reproductive Health

Demographic Dividend Effort Index Survey

Module: Sectoral Resilience and Sustainability

Considering the health and socio-economic impact of the COVID-19 and the likely impact on the DD progress, this DD effort index has integrated questions to assess the resilience and sustainability of systems in key sectors of the DD framework. The items included in the COVID-19 related questionnaire below follow the key dimensions of resilient systems and provide critical information on the potential of an effective response to emerging infectious disease threats or other public health emergencies.

Please score each item from 1 to 10, where 1 represents the lowest score (poor state/capability) and 10 represents the highest score (great state/capability).

* Physical: facilities, equipment, system states, and capabilities.

	1	2	3	4	5	6	7	8	9	10	Don't know
MCH-M1 - Plan/Prepare - State and capability of equipment, personnel and MCH program structure prior to the crisis event. (1 = poor state/capability; 10 = great state/capability)	○	○	○	○	○	○	○	○	○	○	○
MCH-M2 - Absorb - Integration: The level of integration of MCH services and programs to mitigate the effect of COVID-19 on MCH.	○	○	○	○	○	○	○	○	○	○	○
MCH-M3 - Absorb - Integration: Extent to which effective supply chain management was able to mitigate the effect of COVID-19 on the supply/provision of MCH-related commodities.	○	○	○	○	○	○	○	○	○	○	○
MCH-M4 - Recover - Extent to which the MCH program has been able to induce change to recover the pre-COVID-19 level of functionality.	○	○	○	○	○	○	○	○	○	○	○
MCH-M5 - Recover - Financing and Donor Management: Level of resources mobilization and allocation to mitigate the effect of COVID-19 on MCH.	○	○	○	○	○	○	○	○	○	○	○
MCH-M6 - Adapt - Extent to which changes have been made to improve the resilience of the MCH sector.	○	○	○	○	○	○	○	○	○	○	○

* Information: creation, manipulation, storage, and utilization of data.

	1	2	3	4	5	6	7	8	9	10	Don't know
MCH-M7 - Plan/Prepare - Extent to which MCH-related data was sufficiently prepared, presented, analyzed and stored prior to the crisis event.	○	○	○	○	○	○	○	○	○	○	○
MCH-M8 - Absorb - Awareness: Extent of the surveillance capacity to detect the MCH limitations/threats posed by COVID-19.	○	○	○	○	○	○	○	○	○	○	○
MCH-M9 - Recover - Extent to which MCH data has been used to track the progress of recovery and to anticipate recovery scenarios.	○	○	○	○	○	○	○	○	○	○	○
MCH-M10 - Adapt - Extent to which the MCH sector is creating and improving MCH data storage and real-time use protocols.	○	○	○	○	○	○	○	○	○	○	○

* Cognitive: understanding, mental models, preconceptions, biases, and values.

	1	2	3	4	5	6	7	8	9	10	Don't know
MCH-M11 - Plan/Prepare - Extent to which the MCH sector design and operation decisions were prepared in anticipation of crisis events.	◯	◯	◯	◯	◯	◯	◯	◯	◯	◯	◯
MCH-M12 - Absorb - Extent to which the MCH sector is responding with sufficient contingency protocols and proactive crisis management.	◯	◯	◯	◯	◯	◯	◯	◯	◯	◯	◯
MCH-M13 - Absorb - Accountability: Level of effort to ensure optimal accountability for the resources allocated to support MCH as part of the response to the COVID-19.	◯	◯	◯	◯	◯	◯	◯	◯	◯	◯	◯
MCH-M14 - Recover - Extent to which decisions are oriented towards recovery, which employ evidence-based communication to community members to promote safe behavior.	◯	◯	◯	◯	◯	◯	◯	◯	◯	◯	◯
MCH-M15 - Recover - Awareness: Extent to which the MCH sector is building confidence through communication regarding barriers posed by COVID-19 to support access to and uptake of MCH services and programs.	◯	◯	◯	◯	◯	◯	◯	◯	◯	◯	◯
MCH-M16 - Adapt – Adaptive: Extent to which the national leadership had the authority to make changes to adapt and respond to the COVID-19 threats to MCH services and programs.	◯	◯	◯	◯	◯	◯	◯	◯	◯	◯	◯

* Social: interaction, collaboration, and self-synchronization between individuals, entities and institutions.

	1	2	3	4	5	6	7	8	9	10	Don't know
MCH-M17 - Plan/Prepare - Extent to which training on outbreak/crisis management was conducted through, and which leveraged, social networks, social capital and institutional and cultural norms prior to the crisis event.	○	○	○	○	○	○	○	○	○	○	○
MCH-M18 - Absorb - Extent to which personnel and social institutions have been resourceful and accessible for the outbreak/crisis response.	○	○	○	○	○	○	○	○	○	○	○
MCH-M19 - Absorb - Self-Regulating: Extent to which the national leadership had the authority to make sectoral changes in a timely manner through flexible infrastructure.	○	○	○	○	○	○	○	○	○	○	○
MCH-M20 - Recover - Extent to which the MCH sector is engaging with team and knowledge sharing to enhance systematic recovery.	○	○	○	○	○	○	○	○	○	○	○
MCH-M21 - Recover - Diversity: The level of engagement of a multidisciplinary team to mitigate the effect of COVID19 on MCH.	○	○	○	○	○	○	○	○	○	○	○
MCH-M22 - Adapt - Extent to which there are additions or changes being made to MCH institutions, policies, trainings, programs and culture.	○	○	○	○	○	○	○	○	○	○	○

	1	2	3	4	5	6	7	8	9	10	Don't know
MCH-M23 - Adapt - Leadership and Management: Level of leadership demonstrated by the MCH leaders to mitigate the effect of COVID-19 on MCH.	○	○	○	○	○	○	○	○	○	○	○

* MCH M-24 Timeline: Extent of timeliness of the Government reaction - communication and implementation of measures - to mitigate the immediate and longterm impact of COVID-19.
Note: This refers to the entirety of the national reaction - not the reaction that is specific to your sector.

	1	2	3	4	5	6	7	8	9	10	Don't know
1-Lowest -> Highest-10	○	○	○	○	○	○	○	○	○	○	○

MCH M-25 Comments: Please use this section to provide any additional comments about the resilience and sustainability of the national MCH systems based on your experience during the COVID-19 pandemics. If you marked “I don’t know” for any of the questions, also use this section to explain why you gave that rating.

JOHNS HOPKINS
BLOOMBERG SCHOOL
of PUBLIC HEALTH

Bill & Melinda Gates Institute for Population and Reproductive Health

Demographic Dividend Effort Index Survey

Education (ED) Questionnaire

Please respond to the following questions based on your experience/expertise within this specific sector.

To give a summary picture of the national demographic dividend effort, please rate the following items. Score each item from 1 to 10, where 1 represents the lowest score (very weak or no effort) and 10 represents the highest score (strong effort). Where applicable, if a policy or activity is non-existent then mark your response as 0.

Answer each item. All responses will be recorded in the format exemplified below:

Lowest effort	1	2	3	4	5	6	7	8	9	10	Highest effort	I do not know

JOHNS HOPKINS
BLOOMBERG SCHOOL
of PUBLIC HEALTH

Bill & Melinda Gates Institute for Population and Reproductive Health

Demographic Dividend Effort Index Survey

Dimension 1. Policy / Policy making

* ED 1.1 Quality: Extent to which there are national plans/strategies/programs to improve the quality of learning in schools.

	1	2	3	4	5	6	7	8	9	10	Don't know
1-Lowest -> Highest-10											

* ED 1.2 Disadvantaged Groups:

	1	2	3	4	5	6	7	8	9	10	Don't know
ED 1.2.1. Extent to which strategies have been developed and implemented/deployed to overcome learning obstacles for pregnant girls and adolescent mothers.											
ED 1.2.2 Extent to which strategies have been developed and implemented/deployed to overcome learning obstacles for disabled populations.											

* ED 1.3 Teachers: Extent to which teacher recruitment, development and management issues are included in policy aimed at improving learning outcomes.

	1	2	3	4	5	6	7	8	9	10	Don't know
1-Lowest -> Highest-10											

* ED 1.4 Teachers' Accountability: Extent to which national plans include specific measures, including monitoring and evaluation, that aim to hold teachers' accountable for improving learning.

	1	2	3	4	5	6	7	8	9	10	Don't know
1-Lowest -> Highest-10	○	○	○	○	○	○	○	○	○	○	○

* ED 1.5 Teacher Quality: Extent to which education policies/strategies/programs include verifiable indicators for teacher quality.

	1	2	3	4	5	6	7	8	9	10	Don't know
1-Lowest -> Highest-10	○	○	○	○	○	○	○	○	○	○	○

* ED 1.6 Collaboration in Education: Extent to which the policy makes provisions to engage multiple stakeholders in education (e.g. schools, school-communities, parents, teachers, local government, parent-teacher-student committees, etc.).

	1	2	3	4	5	6	7	8	9	10	Don't know
1-Lowest -> Highest-10	○	○	○	○	○	○	○	○	○	○	○

* ED 1.7 Gender Parity SDG: Extent to which national policies/plans/strategies consider the SDG goal for complete gender parity at all levels of education.

	1	2	3	4	5	6	7	8	9	10	Don't know
1-Lowest -> Highest-10	○	○	○	○	○	○	○	○	○	○	○

* ED 1.8 Education Financing: Extent to which new education plans include financing proposals with budgets allocated for learning and teaching reforms.

	1	2	3	4	5	6	7	8	9	10	Don't know
1-Lowest -> Highest-10	○	○	○	○	○	○	○	○	○	○	○

* ED 1.9 Gender Parity Planning: Extent to which there are national plans/strategies to reach 100% enrollment with gender parity in:

	1	2	3	4	5	6	7	8	9	10	Don't know
ED 1.9.1 Primary Education	○	○	○	○	○	○	○	○	○	○	○
ED 1.9.2 Secondary Education	○	○	○	○	○	○	○	○	○	○	○
ED 1.9.3 Tertiary Education	○	○	○	○	○	○	○	○	○	○	○

* ED 1.10 Adult Illiteracy: Extent to which national policies/plans/strategies aim to address illiteracy.

	1	2	3	4	5	6	7	8	9	10	Don't know
ED 1.10.1 Extent to which national policies/plans/strategies aim to address adult illiteracy.	○	○	○	○	○	○	○	○	○	○	○
ED 1.10.2 Extent to which national policies/plans/strategies that aim to address adult illiteracy address gendered disparities of this issue.	○	○	○	○	○	○	○	○	○	○	○

* ED 1.11 Out-of-School Children: Extent to which policies address the plight of out-of-school children to reintegrate them into the school system or provide alternative opportunities for their educational development.

	1	2	3	4	5	6	7	8	9	10	Don't know
1-Lowest -> Highest-10	○	○	○	○	○	○	○	○	○	○	○

* ED 1.12 Technology Integration: Extent to which strategies have been developed and implemented/deployed to integrate information communication and technology in education.

	1	2	3	4	5	6	7	8	9	10	Don't know
1-Lowest -> Highest-10	○	○	○	○	○	○	○	○	○	○	○

* ED 1.13. Technology Literacy: Extent to which resources have been allocated to improve computer literacy across education levels.

	1	2	3	4	5	6	7	8	9	10	Don't know
1-Lowest -> Highest-10	○	○	○	○	○	○	○	○	○	○	○

ED 1.14. Comments: Please use this section to provide any additional comments about the level of effort, challenges, or successes in the country around policies/policymaking for ED. If you marked “I don’t know” for any of the questions, also use this section to explain why you gave that rating.

JOHNS HOPKINS
BLOOMBERG SCHOOL
of PUBLIC HEALTH

Bill & Melinda Gates Institute for Population and Reproductive Health

Demographic Dividend Effort Index Survey

Dimension 2. Services or Programs

* ED 2.1 Teacher Deployment: Extent to which strategies/plans are used to ensure teachers are deployed to areas of the country where they are most needed.

	1	2	3	4	5	6	7	8	9	10	Don't know
1-Lowest -> Highest-10	◯	◯	◯	◯	◯	◯	◯	◯	◯	◯	◯

* ED 2.2 Teacher Quality: Extent to which teacher quality is recognized as a key factor in improving learning outcomes.

	1	2	3	4	5	6	7	8	9	10	Don't know
ED 2.2.1 Extent to which the quality of teacher education is emphasized in wider education policies/plans/strategies.	◯	◯	◯	◯	◯	◯	◯	◯	◯	◯	◯
ED 2.2.2 Extent to which the quality of training and continuous professional development is emphasized in wider education policies/plans/strategies.	◯	◯	◯	◯	◯	◯	◯	◯	◯	◯	◯

* ED 2.3 Gender Parity: Extent to which resources have been utilized to improve equality in education between boys and girls.

	1	2	3	4	5	6	7	8	9	10	Don't know
1-Lowest -> Highest-10	◯	◯	◯	◯	◯	◯	◯	◯	◯	◯	◯

* ED 2.4 Parental Involvement in Education: Extent to which parents are involved in the development and execution of the education strategies (i.e. through the involvement of parent-teacher committees).

	1	2	3	4	5	6	7	8	9	10	Don't know
1-Lowest -> Highest-10	○	○	○	○	○	○	○	○	○	○	○

* ED 2.5 Standardized Assessments: Extent to which all schools utilize standardized school and student assessments in line with specifications made by the Ministry of Education.

	1	2	3	4	5	6	7	8	9	10	Don't know
1-Lowest -> Highest-10	○	○	○	○	○	○	○	○	○	○	○

* ED 2.6. Use of Assessment to Inform Progress: Extent to which adjustments to schools are made based on results from national standardized school assessments.

	1	2	3	4	5	6	7	8	9	10	Don't know
1-Lowest -> Highest-10	○	○	○	○	○	○	○	○	○	○	○

ED 2.7 Comments: Please use this section to provide any additional comments about the level of effort, challenges, or successes in the country around services or programs for ED. If you marked “I don’t know” for any of the questions, also use this section to explain why you gave that rating.

JOHNS HOPKINS
BLOOMBERG SCHOOL
of PUBLIC HEALTH

Bill & Melinda Gates Institute for Population and Reproductive Health

Demographic Dividend Effort Index Survey

Dimension 3. Advocacy

* ED 3.1 Equality: Extent to which equity and equality in education have been highlighted by advocates.

	1	2	3	4	5	6	7	8	9	10	Don't know
1-Lowest -> Highest-10	○	○	○	○	○	○	○	○	○	○	○

* ED 3.2 Quality: Extent to which quality of education takes part in advocacy-related evaluations of the education system.

	1	2	3	4	5	6	7	8	9	10	Don't know
1-Lowest -> Highest-10	○	○	○	○	○	○	○	○	○	○	○

* ED 3.3 Teachers' Obstacles: Extent to which the needs of teachers have been highlighted by advocates.

	1	2	3	4	5	6	7	8	9	10	Don't know
1-Lowest -> Highest-10	○	○	○	○	○	○	○	○	○	○	○

* ED 3.4 Institutions' Obstacles: Extent to which the challenges of educational institutions have been highlighted in different advocacy efforts.

	1	2	3	4	5	6	7	8	9	10	Don't know
1-Lowest -> Highest-10	○	○	○	○	○	○	○	○	○	○	○

ED 3.5 Comments: Please use this section to provide any additional comments about the level of effort, challenges, or successes in the country around advocacy for ED. If you marked "I don't know" for any of the questions, also use this section to explain why you gave that rating

JOHNS HOPKINS
BLOOMBERG SCHOOL
of PUBLIC HEALTH

Bill & Melinda Gates Institute for Population and Reproductive Health

Demographic Dividend Effort Index Survey

Dimension 4. Research

* ED 4.1 Strength of Observatory/Stakeholder/Technical Working Group for ED: Extent to which country-level ED stakeholders (e.g., stakeholder leadership group, technical working group, ED observatory, country coordination and facilitation group):

	1	2	3	4	5	6	7	8	9	10	Don't know
ED 4.1.1 Have representation in government, training institutions, civil society, nongovernmental and faith-based organizations, professional associations, the private sector, etc.	○	○	○	○	○	○	○	○	○	○	○
ED 4.1.2 Meet regularly, report and recommend policy for senior management within the appropriate ministries	○	○	○	○	○	○	○	○	○	○	○
ED 4.1.3 Make an impact on education within the country	○	○	○	○	○	○	○	○	○	○	○

* ED 4.2 Education/Research Strategy: Extent to which the national education plan and/or other national documents include a comprehensive strategy/approach on education-related research, shared among partners.

	1	2	3	4	5	6	7	8	9	10	Don't know
1-Lowest -> Highest-10	○	○	○	○	○	○	○	○	○	○	○

* ED 4.3 Data Gathering Partners:

	1	2	3	4	5	6	7	8	9	10	Don't know
ED 4.3.1 Extent to which research is undertaken by government agencies. (This may include statistical agencies/bureaus and/or ministries of economics/finances, education, health, gender, etc.)	○	○	○	○	○	○	○	○	○	○	○
ED 4.3.2 Extent to which data-gathering is undertaken by research institutions.	○	○	○	○	○	○	○	○	○	○	○
ED 4.3.3 Extent to which data-gathering is undertaken by independent researchers.	○	○	○	○	○	○	○	○	○	○	○

* ED 4.4 Topical Research:

	1	2	3	4	5	6	7	8	9	10	Don't know
ED 4.4.1 Extent to which gender disparities in education are researched.	○	○	○	○	○	○	○	○	○	○	○
ED 4.4.2 Extent to which quality of education is researched.	○	○	○	○	○	○	○	○	○	○	○
ED 4.4.3 Extent to which the obstacles faced by teachers are researched.	○	○	○	○	○	○	○	○	○	○	○
ED 4.4.4 Extent to which the obstacles faced by institutions are researched.	○	○	○	○	○	○	○	○	○	○	○

* ED 4.5 Quality/Coverage of Data: Extent to which current research/data is sex and age disaggregated. Extent to which the country participates in standardized tests and its ranking.

	1	2	3	4	5	6	7	8	9	10	Don't know
ED 4.5.1 Extent to which a routine statistical system (using institution/school-based information) provides good periodic information on supplies and facilities.	○	○	○	○	○	○	○	○	○	○	○
ED 4.5.2 Extent to which a routine statistical system (using institution/school-based information) provides good periodic information on education services and personnel.	○	○	○	○	○	○	○	○	○	○	○
ED 4.5.3 Extent to which the routine statistical system provides good periodic information on education needs among populations in different communities.	○	○	○	○	○	○	○	○	○	○	○
ED 4.5.4 Extent to which evaluations of the education monitoring and information system are conducted and applied to ensure the reliability of data.	○	○	○	○	○	○	○	○	○	○	○

* ED 4.6 Record-Keeping: Extent to which systems for education-related recordkeeping, reporting and feedback of results are adequate.

	1	2	3	4	5	6	7	8	9	10	Don't know
1-Lowest -> Highest-10	○	○	○	○	○	○	○	○	○	○	○

* ED 4.7 Quality Research Institutions: Extent to which the country has the capacity to support and sustain research institutions that develop research/collect data that inform efforts to improve education.

	1	2	3	4	5	6	7	8	9	10	Don't know
1-Lowest -> Highest-10	○	○	○	○	○	○	○	○	○	○	○

* ED 4.8 Evaluation: Extent to which education program statistics, surveys, and small studies are used by specialized staff to report on program operations and measure progress.

	1	2	3	4	5	6	7	8	9	10	Don't know
1-Lowest -> Highest-10	◯	◯	◯	◯	◯	◯	◯	◯	◯	◯	◯

* ED 4.9 Management's Use of Evaluation Findings: Extent to which local-level education program managers use research and evaluation findings to improve these programs in ways suggested by findings.

	1	2	3	4	5	6	7	8	9	10	Don't know
1-Lowest -> Highest-10	◯	◯	◯	◯	◯	◯	◯	◯	◯	◯	◯

* ED 4.10 Ministerial Use of Evaluation Findings: Extent to which the relevant ministry administrators (i.e. ministries of education) systematically utilize data to inform policies and interventions to address education-related issues.

	1	2	3	4	5	6	7	8	9	10	Don't know
1-Lowest -> Highest-10	◯	◯	◯	◯	◯	◯	◯	◯	◯	◯	◯

* ED 4.11 Dissemination of Information to Other Implementing Bodies:

	1	2	3	4	5	6	7	8	9	10	Don't know
ED 4.11.1 Extent to which information is shared or spread across geographical areas	◯	◯	◯	◯	◯	◯	◯	◯	◯	◯	◯
ED. 4.11.2 Extent to which information is shared or spread throughout various levels (national, state/province, localities).	◯	◯	◯	◯	◯	◯	◯	◯	◯	◯	◯

ED 4.12 Comments: Please use this section to provide any additional comments about the level of effort, challenges, or successes in the country around research in education. If you marked "I don't know" for any of the questions, also use this section to explain why you gave that rating

JOHNS HOPKINS
BLOOMBERG SCHOOL of PUBLIC HEALTH

Bill & Melinda Gates Institute for Population and Reproductive Health

Demographic Dividend Effort Index Survey

Dimension 5. Civil Society Organizations (CSOs)

* ED 5.1 CSO Actor Power: Extent to which CSO actors are in strong placements within institutions or technical working groups or networks concerned with education.

	1	2	3	4	5	6	7	8	9	10	Don't know
1-Lowest -> Highest-10	○	○	○	○	○	○	○	○	○	○	○

* ED 5.2 Budget Analysis as a CSO Tool: Extent to which CSOs utilize budget analysis as a tool to build education advocacy cases.

	1	2	3	4	5	6	7	8	9	10	Don't know
1-Lowest -> Highest-10	○	○	○	○	○	○	○	○	○	○	○

* ED 5.3 Community inclusion: Extent to which CSOs are involved in efforts to explore the opportunities to improve the quality of education.

	1	2	3	4	5	6	7	8	9	10	Don't know
1-Lowest -> Highest-10	○	○	○	○	○	○	○	○	○	○	○

* ED 5.4 Community Involvement in Education: Extent to which CSOs participate in bringing education information and services to the communities where people live through activities such as supporting the capacity-building of teachers and parents and/or undertaking information and education campaigns to increase the commitment to education in families and communities.

	1	2	3	4	5	6	7	8	9	10	Don't know
1-Lowest -> Highest-10	○	○	○	○	○	○	○	○	○	○	○

* ED 5.5 Social Franchising: Extent to which CSOs are involved with social franchising opportunities/services to bring quality education services to children and families at reduced costs.

	1	2	3	4	5	6	7	8	9	10	Don't know
1-Lowest -> Highest-10											

* ED 5.6 Technology: Extent to which CSOs are leveraging information communication technology (ICT) to improve access to quality education for various communities.

	1	2	3	4	5	6	7	8	9	10	Don't know
1-Lowest -> Highest-10											

* ED 5.7 Human Rights and Access to/Quality of Education: Extent to which CSOs advocate for and support rights-based approaches to education to ensure students experience good treatment, confidence in asking questions, and quality education, amongst other concerns.

	1	2	3	4	5	6	7	8	9	10	Don't know
1-Lowest -> Highest-10											

* ED 5.8 Social and Behavior Change (SBC): Extent to which CSOs support SBC interventions relating, but not limited, to life skills and peer education, adult health education, school-based education, community mobilization, and/or mass media interventions for a general public audience.

	1	2	3	4	5	6	7	8	9	10	Don't know
1-Lowest -> Highest-10											

* ED 5.9 Advocacy/Accountability: Extent to which CSOs support the training and capacity building of advocates and community members to understand policies, processes, and activities to increase community-led issue identification, collaborative problem solving and more targeted advocacy actions to improve education.

	1	2	3	4	5	6	7	8	9	10	Don't know
1-Lowest -> Highest-10											

* ED 5.10 CSO-Led Assessment and Monitoring: Extent to which CSOs assess, monitor and report on the effectiveness of policies and programming to improve accountability in education at the policy and teachers' levels.

	1	2	3	4	5	6	7	8	9	10	Don't know
1-Lowest -> Highest-10											

* ED 5.11 Forming Partnerships between CSOs: Extent to which CSOs have formed national alliances and regional partnerships to strengthen their positioning and potentially improve leadership strength and funding mechanisms to improve the quality of education.

	1	2	3	4	5	6	7	8	9	10	Don't know
1-Lowest -> Highest-10	◯	◯	◯	◯	◯	◯	◯	◯	◯	◯	◯

ED 5.12 Comments: Please use this section to provide any additional comments about the level of effort, challenges, or successes in the country around the role of the civil society in ED. If you marked “I don’t know” for any of the questions, also use this section to explain why you gave that rating

JOHNS HOPKINS
BLOOMBERG SCHOOL
of PUBLIC HEALTH

Bill & Melinda Gates Institute for Population and Reproductive Health

Demographic Dividend Effort Index Survey

Module: Sectoral Resilience and Sustainability

Considering the health and socio-economic impact of the COVID-19 and the likely impact on the DD progress, this DD effort index has integrated questions to assess the resilience and sustainability of systems in key sectors of the DD framework. The items included in the COVID-19 related questionnaire below follow the key dimensions of resilient systems and provide critical information on the potential of an effective response to emerging infectious disease threats or other public health emergencies.

Please score each item from 1 to 10, where 1 represents the lowest score (poor state/capability) and 10 represents the highest score (great state/capability).

* Physical: facilities, equipment, system states, and capabilities.

	1	2	3	4	5	6	7	8	9	10	Don't know
ED-M1 - Plan/Prepare - State and capability of equipment, personnel and ED sector structure prior to the crisis event. (1 = poor state/capability; 10 = great state/capability)	○	○	○	○	○	○	○	○	○	○	○
ED-M2 - Absorb - Extent to which COVID-19 was recognized within the ED sector and the performance of the system was able to maintain functionality.	○	○	○	○	○	○	○	○	○	○	○
ED-M3 - Absorb - Integration: The level of integration of ED services and programs to mitigate the effect of COVID19 on ED.	○	○	○	○	○	○	○	○	○	○	○
ED-M4 - Recover - Extent to which the ED sector has been able to induce change to recover the pre-COVID-19 level of functionality.	○	○	○	○	○	○	○	○	○	○	○
ED-M5 - Recover - Financing and Donor Management: Level of resources mobilization and allocation to mitigate the effect of COVID-19 on ED.	○	○	○	○	○	○	○	○	○	○	○
ED-M6 - Adapt - Extent to which changes have been made to improve the resilience of the ED sector.	○	○	○	○	○	○	○	○	○	○	○

* Information: creation, manipulation, storage, and utilization of data.

	1	2	3	4	5	6	7	8	9	10	Don't know
ED-M7 - Plan/Prepare - Extent to which ED-related data was sufficiently prepared, presented, analyzed and stored prior to the crisis event.	○	○	○	○	○	○	○	○	○	○	○
ED-M8 - Absorb - Awareness: Extent of the surveillance capacity to detect the ED limitations/threats posed by COVID-19. (This includes data that is focused on the variety of education-related concerns that the education sector is facing at each level [primary, secondary, tertiary] of schooling).	○	○	○	○	○	○	○	○	○	○	○
ED-M9 - Recover - Extent to which ED data has been used to track the progress of recovery and to anticipate recovery scenarios.	○	○	○	○	○	○	○	○	○	○	○
ED-M10 - Adapt - Extent to which the ED system is creating and improving ED data storage and real-time use protocols.	○	○	○	○	○	○	○	○	○	○	○

* Cognitive: understanding, mental models, preconceptions, biases, and values.

	1	2	3	4	5	6	7	8	9	10	Don't know
ED-M11 - Plan/Prepare - Extent to which the ED system design and operation decisions were prepared in anticipation of crisis events.	○	○	○	○	○	○	○	○	○	○	○
ED-M12 - Absorb - Extent to which the ED system is responding with sufficient contingency protocols and proactive crisis management.	○	○	○	○	○	○	○	○	○	○	○

	1	2	3	4	5	6	7	8	9	10	Don't know
ED-M13 - Absorb - Accountability: Level of effort to ensure optimal accountability for the resources allocated to support ED as part of the response to the COVID-19.											
ED-M14 - Recover - Extent to which decisions are oriented towards recovery, which employ evidence-based communication to community members to promote safe behavior.											
ED-M15 - Recover - Awareness: Extent to which the ED sector is building confidence through communication regarding barriers posed by COVID-19 to support access to and uptake of ED services and programs.											
ED-M16 - Adapt - Extent to which the ED sector is designing new system configurations, objectives and decision criteria.											
ED-M17 - Adapt - Adaptive: Level of adaptation to respond to the COVID-19 threats to ED.											

* Social: interaction, collaboration, and self-synchronization between individuals, entities and institutions.

	1	2	3	4	5	6	7	8	9	10	Don't know
ED-M18 - Plan/Prepare - Extent to which training on outbreak/crisis management was conducted through, and which leveraged, social networks, social capital and institutional and cultural norms prior to the crisis event.											

	1	2	3	4	5	6	7	8	9	10	Don't know
ED-M19 - Absorb - Extent to which personnel and social institutions have been resourceful and accessible for the outbreak/crisis response.	○	○	○	○	○	○	○	○	○	○	○
ED-M20 - Absorb - Self-Regulating: Extent to which the national leadership had the authority to make sectoral changes in a timely manner through flexible infrastructure.	○	○	○	○	○	○	○	○	○	○	○
ED-M21 - Recover - Extent to which the ED system is engaging with team and knowledge sharing to enhance systematic recovery.	○	○	○	○	○	○	○	○	○	○	○
ED-M22 - Recover - Diversity: The level of engagement of a multidisciplinary team to mitigate the effect of COVID-19 on ED.	○	○	○	○	○	○	○	○	○	○	○
ED-M23 - Adapt - Extent to which there are additions or changes being made to ED institutions, policies, trainings, programs, and culture.	○	○	○	○	○	○	○	○	○	○	○
ED-M24 - Adapt - Leadership and Management: Level of leadership demonstrated by the ED leaders to mitigate the effect of COVID-19 on ED.	○	○	○	○	○	○	○	○	○	○	○

* ED M-25 Timeline: Extent of timeliness of the Government reaction - communication and implementation of measures - to mitigate the immediate and longterm impact of COVID-19.
Note: This refers to the entirety of the national reaction - not the reaction that is specific to your sector.

	1	2	3	4	5	6	7	8	9	10	Don't know
1-Lowest -> Highest-10	○	○	○	○	○	○	○	○	○	○	○

ED M-26 Comments: Please use this section to provide any additional comments about the resilience and sustainability of the national education systems based on your experience during the COVID-19 pandemics. If you marked “I don’t know” for any of the questions, also use this section to explain why you gave that rating.

JOHNS HOPKINS
BLOOMBERG SCHOOL of PUBLIC HEALTH

Bill & Melinda Gates Institute for Population and Reproductive Health

Demographic Dividend Effort Index Survey

Women's Empowerment (WE) Questionnaire

Please respond to the following questions based on your experience/expertise within this specific sector.

To give a summary picture of the national demographic dividend effort, please rate the following items. Score each item from 1 to 10, where 1 represents the lowest score (very weak or no effort) and 10 represents the highest score (strong effort). Where applicable, if a policy or activity is non-existent then mark your response as 0.

Answer each item. All responses will be recorded in the format exemplified below:

Lowest Effort	1	2	3	4	5	6	7	8	9	10	Highest Effort	I do not know

JOHNS HOPKINS
BLOOMBERG SCHOOL
of PUBLIC HEALTH

Bill & Melinda Gates Institute for Population and Reproductive Health

Demographic Dividend Effort Index Survey

Dimension 1. Policy/Policy making

* WE 1.1 Gender Equality Policy: Extent to which gender equality is considered/integrated in country development plans (or within plans in your sector).

	1	2	3	4	5	6	7	8	9	10	Don't know
1-Lowest -> Highest-10	◯	◯	◯	◯	◯	◯	◯	◯	◯	◯	◯

* WE 1.2 Level of commitment to the Convention on the Elimination of all Forms of Discrimination Against Women (CEDAW).

	1	2	3	4	5	6	7	8	9	10	Don't know
1-Lowest -> Highest-10	◯	◯	◯	◯	◯	◯	◯	◯	◯	◯	◯

* WE 1.3 Equity in Marriage and Family Policies: Extent to which the principle of equality between men and women is incorporated in national constitution and other legislation relating to marriage and family relations.

	1	2	3	4	5	6	7	8	9	10	Don't know
1-Lowest -> Highest-10	◯	◯	◯	◯	◯	◯	◯	◯	◯	◯	◯

* WE 1.4 Policy on Age at Marriage: Extent to which legal age at marriage for females is set at 18 years or higher and is enforced.

	1	2	3	4	5	6	7	8	9	10	Don't know
1-Lowest -> Highest-10	◯	◯	◯	◯	◯	◯	◯	◯	◯	◯	◯

* WE 1.5 Violence Against Women Measures: Level of regulations and measures on violence against women, including domestic violence, rape, sexual harassment and trafficking of girls and women.

	1	2	3	4	5	6	7	8	9	10	Don't know
1-Lowest -> Highest-10	◯	◯	◯	◯	◯	◯	◯	◯	◯	◯	◯

* WE 1.6 Female Genital Cutting/Mutilation: Level of regulation and measures on female genital cutting/mutilation.

	1	2	3	4	5	6	7	8	9	10	Don't know
1-Lowest -> Highest-10	◯	◯	◯	◯	◯	◯	◯	◯	◯	◯	◯

* WE 1.7 Women's Health Measures: Level of protocols for the protection of women's health, including sexually transmitted infections including HIV/AIDS, maternal mortality and contraception.

	1	2	3	4	5	6	7	8	9	10	Don't know
1-Lowest -> Highest-10	◯	◯	◯	◯	◯	◯	◯	◯	◯	◯	◯

* WE 1.8 Education Measures: Extent to which there are mechanisms to promote girls' education.

	1	2	3	4	5	6	7	8	9	10	Don't know
WE 1.8.1 Level of enforcement of policies on girl's education.	◯	◯	◯	◯	◯	◯	◯	◯	◯	◯	◯
WE 1.8.2 Extent to which there are mechanisms in place to prevent girls' school dropouts.	◯	◯	◯	◯	◯	◯	◯	◯	◯	◯	◯

* WE 1.9 Rights Measures: Level of enforcement of policies on women's rights.

	1	2	3	4	5	6	7	8	9	10	Don't know
1-Lowest -> Highest-10	◯	◯	◯	◯	◯	◯	◯	◯	◯	◯	◯

* WE 1.10 Equitable Work: Extent to which there are policies on equitable working conditions and remunerations between men and women.

	1	2	3	4	5	6	7	8	9	10	Don't know
1-Lowest -> Highest-10	◯	◯	◯	◯	◯	◯	◯	◯	◯	◯	◯

* WE 1.11 Equitable Land/Asset Ownership: Extent to which there are policies to affirm equitable access to and ownership of land.

	1	2	3	4	5	6	7	8	9	10	Don't know
1-Lowest -> Highest-10	◯	◯	◯	◯	◯	◯	◯	◯	◯	◯	◯

* WE 1.12 Conflict, Women & Peacebuilding: Level of implementation of Security UN Resolution 1325 on the impacts of conflict on women and their role in peacebuilding.

	1	2	3	4	5	6	7	8	9	10	Don't know
1-Lowest -> Highest-10	◯	◯	◯	◯	◯	◯	◯	◯	◯	◯	◯

* WE 1.13 Gender Quotas in Politics: Existence, and level of implementation, of quotas for gender-balanced political systems.

	1	2	3	4	5	6	7	8	9	10	Don't know
WE 1.13.1 Extent to which there is gender equitable decision-making, including support for electoral quotas and gender mainstreaming in all government ministries and departments.	◯	◯	◯	◯	◯	◯	◯	◯	◯	◯	◯
WE 1.13.2 Extent to which women participate and/or have a significant role in traditional governance systems (e.g. chiefs and village head).	◯	◯	◯	◯	◯	◯	◯	◯	◯	◯	◯

* WE 1.14 Sustainable Development Goal 5: Level of commitment to fulfilling Sustainable Development Goal 5, to achieve gender equality and empower all women and girls.

	1	2	3	4	5	6	7	8	9	10	Don't know
1-Lowest -> Highest-10	◯	◯	◯	◯	◯	◯	◯	◯	◯	◯	◯

* WE 1.15 Customary Law Revision: Extent to which customary laws have been reviewed and modified to align with national policies/frameworks. Or to which existing customary laws reflect efforts towards women's empowerment.

	1	2	3	4	5	6	7	8	9	10	Don't know
1-Lowest -> Highest-10	◯	◯	◯	◯	◯	◯	◯	◯	◯	◯	◯

* WE 1.16 Costing for WE: Extent to which there is sufficient planning and allocating to cover the costs needed to meet targets set in a national gender plan or equivalent national plan.

	1	2	3	4	5	6	7	8	9	10	Don't know
1-Lowest -> Highest-10	◯	◯	◯	◯	◯	◯	◯	◯	◯	◯	◯

* WE 1.17 Staffing for WE: Extent to which:

	1	2	3	4	5	6	7	8	9	10	Don't know
WE 1.17.1 Sufficient and qualified staff are employed to implement gender-related activities, including efforts towards training said staff	○	○	○	○	○	○	○	○	○	○	○
WE 1.17.2 Efforts are made to appropriately and comprehensively train staff	○	○	○	○	○	○	○	○	○	○	○
WE 1.17.3 Staff have sufficient support to carry out their tasks	○	○	○	○	○	○	○	○	○	○	○
WE 1.17.4 Assigned staff are located at a sufficiently high level in the administration to work effectively	○	○	○	○	○	○	○	○	○	○	○

* WE 1.18 Transparency and Accountability: Extent to which the government provides stakeholders with full information on the intentions, plans, programs and budgets available to implement programs in order to ensure accountability and transparency regarding women's empowerment programs. (*Consider measure such as the passage and implementation of a right to information law, regular information sessions for instance in town halls or the provision of such information in leaflets or other material in the local language*)

	1	2	3	4	5	6	7	8	9	10	Don't know
1-Lowest -> Highest-10	○	○	○	○	○	○	○	○	○	○	○

WE 1.19 Comments: Please use this section to provide any additional comments about the level of effort, challenges, or successes in the country around policymaking for WE. If you marked “I don’t know” for any of the questions, also use this section to explain why you gave that rating.

JOHNS HOPKINS
BLOOMBERG SCHOOL of PUBLIC HEALTH

Bill & Melinda Gates Institute for Population and Reproductive Health

Demographic Dividend Effort Index Survey

Dimension 2. Services or Programs

* WE 2.1 Gender Mainstreaming: Extent to which services or programs within and across sectors consider implications for women and men of any planned action.

	1	2	3	4	5	6	7	8	9	10	Don't know
1-Lowest -> Highest-10	◯	◯	◯	◯	◯	◯	◯	◯	◯	◯	◯

* WE 2.2 Gender-Transformative Programming: Extent to which services or programs within and across sectors integrate gender-transformative principles/approaches.

	1	2	3	4	5	6	7	8	9	10	Don't know
1-Lowest -> Highest-10	◯	◯	◯	◯	◯	◯	◯	◯	◯	◯	◯

* WE 2.3 Education: Extent to which education services or programs focus on girl’s and women’s empowerment within the classroom.

	1	2	3	4	5	6	7	8	9	10	Don't know
1-Lowest -> Highest-10	◯	◯	◯	◯	◯	◯	◯	◯	◯	◯	◯

* WE 2.4. Dropouts: Extent to which there are programs to prevent female dropouts.

	1	2	3	4	5	6	7	8	9	10	Don't know
1-Lowest -> Highest-10	◯	◯	◯	◯	◯	◯	◯	◯	◯	◯	◯

* WE 2.5 Technology Literacy: Extent to which technology literacy is promoted amongst girls in particular.

	1	2	3	4	5	6	7	8	9	10	Don't know
1-Lowest -> Highest-10	◯	◯	◯	◯	◯	◯	◯	◯	◯	◯	◯

* WE 2.6 Employment: Extent to which job-creation programs and labor market polices aim to empower women economically and is designed/implemented accordingly.

	1	2	3	4	5	6	7	8	9	10	Don't know
1-Lowest -> Highest-10	◯	◯	◯	◯	◯	◯	◯	◯	◯	◯	◯

* WE 2.7 Women as Mothers and Workers: Extent to which the government makes possible, through laws, regulations and programs, for women to combine the roles of childbearing, breast-feeding and child-rearing with participation in the workforce.

	1	2	3	4	5	6	7	8	9	10	Don't know
1-Lowest -> Highest-10	◯	◯	◯	◯	◯	◯	◯	◯	◯	◯	◯

* WE 2.8 Family Planning for Empowerment: Extent to which FP services or programs are implemented with the fundamental approach towards empowering women.

	1	2	3	4	5	6	7	8	9	10	Don't know
1-Lowest -> Highest-10	◯	◯	◯	◯	◯	◯	◯	◯	◯	◯	◯

* WE 2.9 Political and Public Representation: Extent of implementation of programs aiming at improving women's equal participation and equitable representation at all levels of the political process and public life.

	1	2	3	4	5	6	7	8	9	10	Don't know
1-Lowest -> Highest-10	◯	◯	◯	◯	◯	◯	◯	◯	◯	◯	◯

* WE 2.10 Violence: Extent of fight against violence against women and children and its elimination nationwide (domestic violence, rape, sexual harassment, trafficking women).

	1	2	3	4	5	6	7	8	9	10	Don't know
1-Lowest -> Highest-10	◯	◯	◯	◯	◯	◯	◯	◯	◯	◯	◯

* WE 2.11 Child Marriage: Extent of law enforcement to eliminate child marriage.

	1	2	3	4	5	6	7	8	9	10	Don't know
1-Lowest -> Highest-10	◯	◯	◯	◯	◯	◯	◯	◯	◯	◯	◯

* WE 2.12 Female Genital Mutilation/Cutting: Extent of law enforcement to eliminate female genital mutilation/cutting.

	1	2	3	4	5	6	7	8	9	10	Don't know
1-Lowest -> Highest-10	◯	◯	◯	◯	◯	◯	◯	◯	◯	◯	◯

WE 2.13 Comments: Please use this section to provide any additional comments about the level of effort, challenges, or successes in the country around services or programs for WE. If you marked “I don’t know” for any of the questions, also use this section to explain why you gave that rating

JOHNS HOPKINS
BLOOMBERG SCHOOL
of PUBLIC HEALTH

Bill & Melinda Gates Institute for Population and Reproductive Health

Demographic Dividend Effort Index Survey

Dimension 3. Advocacy

* WE 3.1 Advocates: Extent to which advocates for girls' and women's empowerment exists at the national and sub-national levels.

	1	2	3	4	5	6	7	8	9	10	Don't know
1-Lowest -> Highest-10	◯	◯	◯	◯	◯	◯	◯	◯	◯	◯	◯

* WE 3.2. Girls and Women: Extent to which the advocacy efforts in this area encompass the breadth of concerns in girls' and women's empowerment.

	1	2	3	4	5	6	7	8	9	10	Don't know
1-Lowest -> Highest-10	◯	◯	◯	◯	◯	◯	◯	◯	◯	◯	◯

* WE 3.3 Information Dissemination: Extent to which the government undertakes sufficient efforts to ensure that the population is aware of issues related to WE, and whether the information that is spread is correct and will reach people in a language they understand.

	1	2	3	4	5	6	7	8	9	10	Don't know
1-Lowest -> Highest-10	◯	◯	◯	◯	◯	◯	◯	◯	◯	◯	◯

* WE 3.4 Leveraging Partnerships: Extent to which the government leverages partnerships with CSOs to disseminate WE information through campaigns.

	1	2	3	4	5	6	7	8	9	10	Don't know
1-Lowest -> Highest-10	◯	◯	◯	◯	◯	◯	◯	◯	◯	◯	◯

* WE 3.5 Employment: Extent to which stakeholders call for laws, policies and programs for women to combine the roles of childbearing, breast-feeding and child-rearing with participation in the workforce.

	1	2	3	4	5	6	7	8	9	10	Don't know
1-Lowest -> Highest-10	◯	◯	◯	◯	◯	◯	◯	◯	◯	◯	◯

* WE 3.6 Education: Extent to which a priority of advocates is to promote the fulfillment of girls' and women's potential through education and skills development.

	1	2	3	4	5	6	7	8	9	10	Don't know
1-Lowest -> Highest-10	◯	◯	◯	◯	◯	◯	◯	◯	◯	◯	◯

* WE 3.7 Violence: Extent to which advocates successfully engage with the government to react to and address cases of violence or discrimination against women.

	1	2	3	4	5	6	7	8	9	10	Don't know
1-Lowest -> Highest-10	◯	◯	◯	◯	◯	◯	◯	◯	◯	◯	◯

WE 3.8 Comments: Please use this section to provide any additional comments about the level of effort, challenges, or successes in the country around advocacy for WE. If you marked "I don't know" for any of the questions, also use this section to explain why you gave that rating

JOHNS HOPKINS
BLOOMBERG SCHOOL
of PUBLIC HEALTH

Bill & Melinda Gates Institute for Population and Reproductive Health

Demographic Dividend Effort Index Survey

Dimension 4. Research

* WE 4.1 Strength of Observatory/Stakeholder/Technical Working Group for WE: Extent to which country-level WE stakeholders (e.g., stakeholder leadership group, technical working group, WE observatory, country coordination and facilitation group):

	1	2	3	4	5	6	7	8	9	10	Don't know
WE 4.1.1 Have representation in government, training institutions, civil society, nongovernmental and faith-based organizations, professional associations, the private sector, etc.	◯	◯	◯	◯	◯	◯	◯	◯	◯	◯	◯
WE 4.1.2 Meet regularly, report and recommend policy for senior management within the appropriate ministries	◯	◯	◯	◯	◯	◯	◯	◯	◯	◯	◯
WE 4.1.3 Make an impact on WE within the country	◯	◯	◯	◯	◯	◯	◯	◯	◯	◯	◯

* WE 4.2 Women's Rights Research Strategy: Extent to which the national WE plan and/or other national documents include a comprehensive strategy/approach on women's empowerment research, shared among partners.

	1	2	3	4	5	6	7	8	9	10	Don't know
1-Lowest -> Highest-10	◯	◯	◯	◯	◯	◯	◯	◯	◯	◯	◯

* WE 4.3 Data Gathering Partners:

	1	2	3	4	5	6	7	8	9	10	Don't know
WE 4.3.1 Extent to which research is undertaken by government agencies. (This may include statistical agencies/bureaus and/or ministries of economics/finances, education, health, gender, etc.)	○	○	○	○	○	○	○	○	○	○	○
WE 4.3.2 Extent to which women empowerment research is undertaken by research institutions.	○	○	○	○	○	○	○	○	○	○	○
WE 4.3.3 Extent to which women empowerment research is undertaken by independent researchers.	○	○	○	○	○	○	○	○	○	○	○

* WE 4.4 Topical Data Gathering:

	1	2	3	4	5	6	7	8	9	10	Don't know
WE 4.4.1 Extent to which social and cultural norms affecting women empowerment and gender equality are researched.	○	○	○	○	○	○	○	○	○	○	○
WE 4.4.2 Extent to which health and economic effects of women empowerment are researched.	○	○	○	○	○	○	○	○	○	○	○
WE 4.4.3 Extent to which child marriage is researched to inform policy.	○	○	○	○	○	○	○	○	○	○	○
WE 4.4.4 Extent to which girls' education and skills acquisition outcomes is researched.	○	○	○	○	○	○	○	○	○	○	○

* WE 4.5 Quality/Coverage of Data:

	1	2	3	4	5	6	7	8	9	10	Don't know
WE 4.5.1 Extent to which a routine statistical system provides reliable periodic age-disaggregated data.	○	○	○	○	○	○	○	○	○	○	○
WE 4.5.2 Extent to which the routine statistical system provides good reliable periodic age-disaggregated data on women's empowerment needs among populations in different communities.	○	○	○	○	○	○	○	○	○	○	○
WE 4.5.3 Extent to which evaluations of the monitoring and surveillance systems are conducted and applied to ensure the reliability of age-disaggregated data.	○	○	○	○	○	○	○	○	○	○	○

* WE 4.6 Quality Research Institutions: Extent to which the country supports and sustains research institutions that develop research/collect data that support the breadth and quality of information-gathering to inform national WE efforts.

	1	2	3	4	5	6	7	8	9	10	Don't know
1-Lowest -> Highest-10	○	○	○	○	○	○	○	○	○	○	○

* WE 4.7 Evaluation: Extent to which program statistics, surveys, and small studies are used by specialized staff to report on program operations and measure progress on WE and gender equality.

	1	2	3	4	5	6	7	8	9	10	Don't know
1-Lowest -> Highest-10	○	○	○	○	○	○	○	○	○	○	○

* WE 4.8 Management's Use of Evaluation Findings: Extent to which local-level program managers use research and evaluation findings to improve WE programs in ways suggested by findings.

	1	2	3	4	5	6	7	8	9	10	Don't know
1-Lowest -> Highest-10	○	○	○	○	○	○	○	○	○	○	○

* WE 4.9 Ministerial Use of Evaluation Findings: Extent to which the Ministry administrators systematically utilize data to inform policies and interventions to address women’s empowerment-related issues.

	1	2	3	4	5	6	7	8	9	10	Don't know
1-Lowest -> Highest-10	◯	◯	◯	◯	◯	◯	◯	◯	◯	◯	◯

* WE 4.10 Dissemination of Information to other Implementing Bodies: Extent to which information is shared or spread across geographical areas and throughout various levels (national, state/province, localities).

	1	2	3	4	5	6	7	8	9	10	Don't know
1-Lowest -> Highest-10	◯	◯	◯	◯	◯	◯	◯	◯	◯	◯	◯

WE 4.11 Comments: Please use this section to provide any additional comments about the level of effort, challenges, or successes in the country around WE research. If you marked “I don’t know” for any of the questions, also use this section to explain why you gave that rating

JOHNS HOPKINS
BLOOMBERG SCHOOL
of PUBLIC HEALTH

Bill & Melinda Gates Institute for Population and Reproductive Health

Demographic Dividend Effort Index Survey

Dimension 5. Civil Society Organizations (CSOs)

* WE 5.1 CSO Actor Power: Extent to which WE civil society actors are in strong position to influence national policy.

	1	2	3	4	5	6	7	8	9	10	Don't know
1-Lowest -> Highest-10	○	○	○	○	○	○	○	○	○	○	○

* WE 5.2 Budget Analysis as a CSO Tool: Extent to which CSOs utilize budget analysis as a tool to build WE advocacy cases.

	1	2	3	4	5	6	7	8	9	10	Don't know
1-Lowest -> Highest-10	○	○	○	○	○	○	○	○	○	○	○

* WE 5.3 Community-Based Service Delivery Support: Extent to which CSOs participate in bringing women's empowerment information and services to the communities where people live through activities such as supporting the capacity-building of girls, women and women's empowerment champions and/or undertaking information and education campaigns to increase the commitment to women's empowerment in families and communities.

	1	2	3	4	5	6	7	8	9	10	Don't know
1-Lowest -> Highest-10	○	○	○	○	○	○	○	○	○	○	○

* WE 5.4 Human Rights and Quality of Implementation: Extent to which CSOs advocate for and support rights-based approaches to women's empowerment to ensure policy and program beneficiaries experience progress in women's empowerment and autonomy on their terms.

	1	2	3	4	5	6	7	8	9	10	Don't know
1-Lowest -> Highest-10	○	○	○	○	○	○	○	○	○	○	○

* WE 5.5 Social and Behavior Change (SBC): Extent to which CSOs support SBC interventions relating, but not limited, to gender-transformative programming, life skills and peer education, adult health education, school-based education, couple's communication, community mobilization, working through gatekeepers, and/or mass media interventions for a general public audience. These may be supported through implementation at the community or individual levels.

	1	2	3	4	5	6	7	8	9	10	Don't know
1-Lowest -> Highest-10	○	○	○	○	○	○	○	○	○	○	○

* WE 5.6 Youth: Extent to which CSOs promote gender empowerment through youth programs.

	1	2	3	4	5	6	7	8	9	10	Don't know
1-Lowest -> Highest-10	○	○	○	○	○	○	○	○	○	○	○

* WE 5.7 Programming for Men: Extent to which CSOs support WE policies, interventions and programs targeting men.

	1	2	3	4	5	6	7	8	9	10	Don't know
1-Lowest -> Highest-10	○	○	○	○	○	○	○	○	○	○	○

* WE 5.8 Advocacy/Accountability: Extent to which CSOs support the training and capacity building of advocates and community members to understand policies, processes, and activities to increase community-led WE issues identification, collaborative problem solving and more targeted advocacy actions.

	1	2	3	4	5	6	7	8	9	10	Don't know
1-Lowest -> Highest-10	○	○	○	○	○	○	○	○	○	○	○

* WE 5.9 CSO-Led Assessment and Monitoring: Extent to which CSOs assess, monitor and report on the effectiveness of policies and programming to improve accountability at the policy and provider levels.

	1	2	3	4	5	6	7	8	9	10	Don't know
1-Lowest -> Highest-10	○	○	○	○	○	○	○	○	○	○	○

* WE 5.10 Forming Partnerships between CSOs: Extent to which CSOs have formed national alliances and regional partnerships to strengthen their positioning and potentially improve leadership strength and funding mechanisms to advance the women empowerment agenda.

	1	2	3	4	5	6	7	8	9	10	Don't know
1-Lowest -> Highest-10	○	○	○	○	○	○	○	○	○	○	○

WE 5.11 Comments: Please use this section to provide any additional comments about the level of effort, challenges, or successes around the role of CSOs in WE. If you marked “I don’t know” for any of the questions, also use this section to explain why you gave that rating

JOHNS HOPKINS
BLOOMBERG SCHOOL of PUBLIC HEALTH

Bill & Melinda Gates Institute for Population and Reproductive Health

Demographic Dividend Effort Index Survey

Module: Sectoral Resilience and Sustainability

Considering the health and socio-economic impact of the COVID-19 and the likely impact on the DD progress, this DD effort index has integrated questions to assess the resilience and sustainability of systems in key sectors of the DD framework. The items included in the COVID-19 related questionnaire below follow the key dimensions of resilient systems and provide critical information on the potential of an effective response to emerging infectious disease threats or other public health emergencies.

Please score each item from 1 to 10, where 1 represents the lowest score (poor state/capability) and 10 represents the highest score (great state/capability).

* Physical: facilities, equipment, system states, and capabilities.

	1	2	3	4	5	6	7	8	9	10	Don't know
WE-M1 - Plan/Prepare - State and capability of equipment, personnel and WE sector structure prior to the crisis event. (1 = poor state/capability; 10 = great state/capability)	○	○	○	○	○	○	○	○	○	○	○
WE-M2 - Absorb - Extent to which COVID-19 was recognized within the WE sector and the performance of the system was able to maintain functionality.	○	○	○	○	○	○	○	○	○	○	○
WE-M3 - Absorb - Integration: The level of integration of WE services and programs to mitigate the effect of COVID-19 on WE.	○	○	○	○	○	○	○	○	○	○	○
WE-M4 - Recover - Extent to which the WE programs have been able to induce change to recover the pre-COVID-19 level of functionality.	○	○	○	○	○	○	○	○	○	○	○
WE-M5 - Recover - Financing and Donor Management: Level of resources mobilization and allocation to mitigate the effect of COVID-19 on WE	○	○	○	○	○	○	○	○	○	○	○
WE-M6 - Adapt - Extent to which changes have been made to improve resilience in the WE sector.	○	○	○	○	○	○	○	○	○	○	○

* Information: creation, manipulation, storage, and utilization of data.

	1	2	3	4	5	6	7	8	9	10	Don't know
WE-M7 - Plan/Prepare - Extent to which WE-related data was sufficiently prepared, presented, analyzed and stored prior to the crisis event.	○	○	○	○	○	○	○	○	○	○	○
WE-M8 - Absorb - Extent to which the WE sector has been able to conduct a real-time assessment of functionality, in anticipation of cascading losses in WE programs and services.	○	○	○	○	○	○	○	○	○	○	○
WE-M9 - Absorb - Awareness: Extent of the surveillance capacity to detect the WE limitations/threats posed by COVID-19.	○	○	○	○	○	○	○	○	○	○	○
WE-M10 - Recover - Extent to which WE data have been used to track the progress of recovery and to anticipate recovery scenarios.	○	○	○	○	○	○	○	○	○	○	○
WE-M11 - Adapt - Extent to which the WE sector is creating and improving WE data storage and real-time use protocols.	○	○	○	○	○	○	○	○	○	○	○

* Cognitive: understanding, mental models, preconceptions, biases, and values.

	1	2	3	4	5	6	7	8	9	10	Don't know
WE-M12 - Plan/Prepare - Extent to which the WE programs design and operation decisions were prepared in anticipation of crisis events.	○	○	○	○	○	○	○	○	○	○	○
WE-M13 - Absorb - Extent to which the WE sector is responding with sufficient contingency protocols and proactive crisis management.	○	○	○	○	○	○	○	○	○	○	○

	1	2	3	4	5	6	7	8	9	10	Don't know
WE-M14 - Absorb - Accountability: Level of effort to ensure optimal accountability for the resources allocated to support WE as part of the response to the COVID-19. (These may take the form of support for services attenuated to the needs of women and girls for protection).											
WE-M15 - Recover - Extent to which decisions are oriented towards recovery, which employ evidence-based communication to community members to promote safe behavior.											
WE-M16 - Recover - Awareness: Extent to which the WE sector is building confidence through communication regarding barriers posed by COVID-19 to support access to and uptake of WE services and programs. (This includes surveillance that is attenuated to the gendered disparities in COVID-19 responses and/or cases as well as increased surveillance of WE-related issues such as gender-based violence)											
WE-M17 - Adapt - Extent to which the WE sector is designing new system configurations, objectives and decision criteria.											
WE-M18 - Adapt - Adaptive: Level of adaptation to respond to the COVID-19 threats to WE.											

* Social: interaction, collaboration, and self-synchronization between individuals, entities and institutions.

	1	2	3	4	5	6	7	8	9	10	Don't know
WE-M19 - Plan/Prepare - Extent to which training on outbreak/crisis management was conducted through, and which leveraged, social networks, social capital and institutional and cultural norms prior to the crisis.	◯	◯	◯	◯	◯	◯	◯	◯	◯	◯	◯
WE-M20 - Absorb - Extent to which personnel and social institutions have been resourceful and accessible for the outbreak/crisis response.	◯	◯	◯	◯	◯	◯	◯	◯	◯	◯	◯
WE-M21 - Absorb - Self-Regulating: Extent to which the national leadership had the authority to make sectoral changes in a timely manner through flexible infrastructure.	◯	◯	◯	◯	◯	◯	◯	◯	◯	◯	◯
WE-M22 - Recover - Extent to which the WE sector is engaging with team and knowledge sharing to enhance systematic recovery.	◯	◯	◯	◯	◯	◯	◯	◯	◯	◯	◯
WE-M23 - Recover - Diversity: The level of engagement of a multidisciplinary team to mitigate the effect of COVID-19 on WE.	◯	◯	◯	◯	◯	◯	◯	◯	◯	◯	◯
WE-M24 - Adapt - Leadership and Management: Level of leadership demonstrated by the leaders in the WE sector and programs to mitigate the effect of COVID-19 on WE.	◯	◯	◯	◯	◯	◯	◯	◯	◯	◯	◯

* WE M-25 Timeline: Extent of timeliness of the Government reaction - communication and implementation of measures - to mitigate the immediate and longterm impact of COVID-19.
Note: This refers to the entirety of the national reaction - not the reaction that is specific to your sector.

	1	2	3	4	5	6	7	8	9	10	Don't know
1-Lowest -> Highest-10	◯	◯	◯	◯	◯	◯	◯	◯	◯	◯	◯

WE M-26 Comments: Please use this section to provide any additional comments about the resilience of the national women empowerment opportunities and systems based on your experience during the COVID-19 pandemics. If you marked “I don’t know” for any of the questions, also use this section to explain why you gave that rating

JOHNS HOPKINS
BLOOMBERG SCHOOL
of PUBLIC HEALTH

Bill & Melinda Gates Institute for Population and Reproductive Health

Demographic Dividend Effort Index Survey

Labor Market (LM) Questionnaire

Please respond to the following questions based on your experience/expertise within this specific sector.

To give a summary picture of the national demographic dividend effort, please rate the following items. Score each item from 1 to 10, where 1 represents the lowest score (very weak or no effort) and 10 represents the highest score (strong effort). Where applicable, if a policy or activity is non-existent then mark your response as 0.

Answer each item. All responses will be recorded in the format exemplified below:

Lowest effort	1	2	3	4	5	6	7	8	9	10	Highest effort	I do not know

JOHNS HOPKINS
BLOOMBERG SCHOOL
of PUBLIC HEALTH

Bill & Melinda Gates Institute for Population and Reproductive Health

Demographic Dividend Effort Index Survey

Dimension 1. Policy/Policy making

* LM 1.1 Global Economic Integration: Extent to which the country aims to be integrated in the global economy.

	1	2	3	4	5	6	7	8	9	10	Don't know
LM 1.1.1 Extent to which the country legislates for trade openness.	○	○	○	○	○	○	○	○	○	○	○
LM 1.1.2 Level of effort towards growing national exports.	○	○	○	○	○	○	○	○	○	○	○

* LM 1.2 Regional Economic Integration: Extent to which the country aims to be integrated in the regional economy.

	1	2	3	4	5	6	7	8	9	10	Don't know
1-Lowest -> Highest-10	○	○	○	○	○	○	○	○	○	○	○

* LM 1.3 Technology: Extent to which the government has invested in infrastructure, both traditional and technological, to provide the basis for the country to leverage opportunities of the future and/or to integrate technology as a complement to labor.

	1	2	3	4	5	6	7	8	9	10	Don't know
1-Lowest -> Highest-10	○	○	○	○	○	○	○	○	○	○	○

* LM 1.4 Government Support: Extent to which policies and plans are in place that provide for social safety nets to smooth transitions between jobs or 'gigs' for individuals and buffer income volatility.

	1	2	3	4	5	6	7	8	9	10	Don't know
LM 1.4.1 Extent to which policies are in place that set a national minimum wage.	◯	◯	◯	◯	◯	◯	◯	◯	◯	◯	◯
LM 1.4.2 Extent to which policies exist that outline an unemployment insurance system, and the level to which they are effectively implemented.	◯	◯	◯	◯	◯	◯	◯	◯	◯	◯	◯

* LM 1.5 Unemployment: Extent to which policies account for and address unemployment in various population groups and geographic areas.

	1	2	3	4	5	6	7	8	9	10	Don't know
1-Lowest -> Highest-10	◯	◯	◯	◯	◯	◯	◯	◯	◯	◯	◯

* LM 1.6 Disadvantaged/Vulnerable Groups: Extent to which policies exist, and are effectively implemented, to support the employment opportunities of disadvantage/vulnerable groups (i.e. girls and women, youth, persons with disabilities, etc.).

	1	2	3	4	5	6	7	8	9	10	Don't know
1-Lowest -> Highest-10	◯	◯	◯	◯	◯	◯	◯	◯	◯	◯	◯

* LM 1.7 Climate Change and Environmental Sustainability: Extent to which the agricultural and manufacturing sectors are prepared to labor market impacts from climate change effects through planning and strategizing for the future labor market needs.

	1	2	3	4	5	6	7	8	9	10	Don't know
1-Lowest -> Highest-10	◯	◯	◯	◯	◯	◯	◯	◯	◯	◯	◯

* LM 1.8 Urbanization: Extent to which policies and planning anticipate and are prepared for increasing urbanization as a result of, and contributing to, climate change, and the particular impacts on labor markets.

	1	2	3	4	5	6	7	8	9	10	Don't know
1-Lowest -> Highest-10	◯	◯	◯	◯	◯	◯	◯	◯	◯	◯	◯

* LM 1.9 Service-Led Economy: Extent to which policies and framework are in place to move economies from informal labor to formalized labor responding to local, regional and global demands for goods and services.

	1	2	3	4	5	6	7	8	9	10	Don't know
1-Lowest -> Highest-10	◯	◯	◯	◯	◯	◯	◯	◯	◯	◯	◯

* LM 1.10 Corporate Social Responsibility: Extent to which national legislation in the labor market address corporate social responsibilities with the aim of supporting youth entrepreneurship.

	1	2	3	4	5	6	7	8	9	10	Don't know
1-Lowest -> Highest-10	◯	◯	◯	◯	◯	◯	◯	◯	◯	◯	◯

* LM 1.11 Targeted Labor Market Investments: Extent to which the government has targeted investments towards sectors with high job-multiplier effects, including Information and Communications Technology (ICT), manufacturing, agriculture and agro-industries in order to generate employment and spur inclusive growth.

	1	2	3	4	5	6	7	8	9	10	Don't know
1-Lowest -> Highest-10	◯	◯	◯	◯	◯	◯	◯	◯	◯	◯	◯

* LM 1.12 Self-Employment: Extent to which policies support raising the returns to self-employment and creating more opportunities to move from self-employment into higher paying wage employment. (This includes rural work).

	1	2	3	4	5	6	7	8	9	10	Don't know
1-Lowest -> Highest-10	◯	◯	◯	◯	◯	◯	◯	◯	◯	◯	◯

* LM 1.13 Microcredit: Extent to which the government enables access to microcredits for profitable activities, securing affordable credit for small and medium-sized enterprises and self-workers.

	1	2	3	4	5	6	7	8	9	10	Don't know
1-Lowest -> Highest-10	◯	◯	◯	◯	◯	◯	◯	◯	◯	◯	◯

* LM 1.14 Savings: Extent to which the government supports the creation of accessible financial products and policies to promote the culture of saving.

	1	2	3	4	5	6	7	8	9	10	Don't know
1-Lowest -> Highest-10	◯	◯	◯	◯	◯	◯	◯	◯	◯	◯	◯

* LM 1.15 Skill Acquisition: Extent to which policies exist, and are effectively implemented, to support the acquisition of currently and future-valued skills for the labor market.

	1	2	3	4	5	6	7	8	9	10	Don't know
1-Lowest -> Highest-10	○	○	○	○	○	○	○	○	○	○	○

LM 1.16 Comments: Please use this section to provide any additional comments about the level of effort, challenges, or successes in the country around policies/policymaking for LM. If you marked “I don’t know” for any of the questions, also use this section to explain why you gave that rating

JOHNS HOPKINS
BLOOMBERG SCHOOL
of PUBLIC HEALTH

Bill & Melinda Gates Institute for Population and Reproductive Health

Demographic Dividend Effort Index Survey

Dimension 2. Services or Programs

* LM 2.1 Unemployment Reduction: Extent to which there are efforts toward developing and implementing strategies aimed at the reduction of the proportion of unemployed by target years (in accordance with Agenda 2063 First Ten Year Implementation Plan).

	1	2	3	4	5	6	7	8	9	10	Don't know
1-Lowest -> Highest-10											

* LM 2.2. Youth Unemployment: Extent to which labor market programs have effectively lowered the rate of unemployment, especially among youth.

	1	2	3	4	5	6	7	8	9	10	Don't know
1-Lowest -> Highest-10											

* LM 2.3 Access to Microcredit: Extent to which national and regional funds have been established and operationalized in order to increase people’s access to affordable business capital.

	1	2	3	4	5	6	7	8	9	10	Don't know
1-Lowest -> Highest-10											

* LM 2.4 Harnessing Youth Special Capacities: Extent to which programs and services support the specific talents of youth to aid them in flourishing and achieving their maximum potential. (This includes specific programmatic aims around talent acquisition).

	1	2	3	4	5	6	7	8	9	10	Don't know
1-Lowest -> Highest-10											

* LM 2.5 Early Labor Opportunities: Extent to which programs and government legislation engages with private sector partners to expand internships, apprenticeships and on-the-job training opportunities for women and youth.

	1	2	3	4	5	6	7	8	9	10	Don't know
1-Lowest -> Highest-10	◯	◯	◯	◯	◯	◯	◯	◯	◯	◯	◯

* LM 2.6 Youth Entrance into Labor: Extent of the promotion of youth volunteer and junior professional programs and other internship opportunities to enhance capacity and grant exposure to youth in regional and international organizations.

	1	2	3	4	5	6	7	8	9	10	Don't know
1-Lowest -> Highest-10	◯	◯	◯	◯	◯	◯	◯	◯	◯	◯	◯

* LM 2.7 Entrepreneurial Ventures: Extent to which people have enhanced access to government procurement and financial services to reduce the challenge of starting and/or doing business within and across countries.

	1	2	3	4	5	6	7	8	9	10	Don't know
1-Lowest -> Highest-10	◯	◯	◯	◯	◯	◯	◯	◯	◯	◯	◯

* LM 2.8 Resources for Youth Entrepreneurship: Extent to which entrepreneurial ventures of youth receive direct and targeted resource mobilization, supported by a regulatory framework, to reduce the challenge of starting and/or doing business within and across countries.

	1	2	3	4	5	6	7	8	9	10	Don't know
1-Lowest -> Highest-10	◯	◯	◯	◯	◯	◯	◯	◯	◯	◯	◯

* LM 2.9 Vocational Opportunities: Extent to which vocational training opportunities are expanded for skills acquisition for people to enhance their employability, productivity and competitiveness.

	1	2	3	4	5	6	7	8	9	10	Don't know
1-Lowest -> Highest-10	◯	◯	◯	◯	◯	◯	◯	◯	◯	◯	◯

* LM 2.10 Education and Labor: Extent to which improvements have been made to improve inclusive access to education at all levels and to provide viable alternatives for the many young people, particularly adolescent girls, who drop out of the formal education system, by facilitating re-entry, revamping informal education and training through standardized certification within and between countries.

	1	2	3	4	5	6	7	8	9	10	Don't know
1-Lowest -> Highest-10	◯	◯	◯	◯	◯	◯	◯	◯	◯	◯	◯

* LM 2.11 Women-Friendly Labor: Extent to which workplaces in the private and public sectors have adopted women-friendly policies and practices to eliminate any form of gender-based discrimination while allowing women to comfortably be part of the labor force.

	1	2	3	4	5	6	7	8	9	10	Don't know
1-Lowest -> Highest-10	○	○	○	○	○	○	○	○	○	○	○

* LM 2.12 Job Security:

	1	2	3	4	5	6	7	8	9	10	Don't know
LM 2.12.1 Extent to which notice periods for redundancy or dismissal are implemented.	○	○	○	○	○	○	○	○	○	○	○
LM 2.12.2 Extent to which severance pay for redundancy or dismissal is implemented.	○	○	○	○	○	○	○	○	○	○	○

* LM 2.13 Minimum Wage: Extent to which a minimum wage is applied to the 'typical' worker (defined as an average wage-earner, in majority race and religious groups, in largest city, and non-union).

	1	2	3	4	5	6	7	8	9	10	Don't know
1-Lowest -> Highest-10	○	○	○	○	○	○	○	○	○	○	○

* LM 2.14 Labor Market Programs: Extent to which labor market programs have effectively diversified the labor force and the needs of the labor market while simultaneously diversifying the skills of the labor force.

	1	2	3	4	5	6	7	8	9	10	Don't know
1-Lowest -> Highest-10	○	○	○	○	○	○	○	○	○	○	○

* LM 2.15 Informality in the Labor Market: Extent to which the programs are implemented to transform informality in the labor market into formal labor.

	1	2	3	4	5	6	7	8	9	10	Don't know
1-Lowest -> Highest-10	○	○	○	○	○	○	○	○	○	○	○

LM 2.16 Comments: Please use this section to provide any additional comments about the level of effort, challenges, or successes in the country around services or programs for LM. If you marked "I don't know" for any of the questions, also use this section to explain why you gave that rating.

JOHNS HOPKINS
BLOOMBERG SCHOOL
of PUBLIC HEALTH

Bill & Melinda Gates Institute for Population and Reproductive Health

Demographic Dividend Effort Index Survey

Dimension 3. Advocacy

* LM 3.1 Gender Equality in the Labor Market: Extent to which groups and individuals call for, and see improvements in, gender equality in the workforce.

	1	2	3	4	5	6	7	8	9	10	Don't know
1-Lowest -> Highest-10	◯	◯	◯	◯	◯	◯	◯	◯	◯	◯	◯

* LM 3.2 Youth Equality in the Labor Market: Extent to which individuals and groups call for shifts in the labor market and labor market regulations to benefit all rural and urban youth populations.

	1	2	3	4	5	6	7	8	9	10	Don't know
1-Lowest -> Highest-10	◯	◯	◯	◯	◯	◯	◯	◯	◯	◯	◯

* LM 3.3 Minimum Wage Advocacy: Extent to which advocacy groups are successful in inciting legislation for a minimum wage, or a rise in the minimum wage if one already exists.

	1	2	3	4	5	6	7	8	9	10	Don't know
1-Lowest -> Highest-10	◯	◯	◯	◯	◯	◯	◯	◯	◯	◯	◯

* LM 3.4 Government Advocacy: Extent to which members of the government advocates for improvements within its own structures and priorities. (These improvements may include labor market-focused progress for youth inclusion, opportunities, programming and rights among similar concepts across the population).

	1	2	3	4	5	6	7	8	9	10	Don't know
1-Lowest -> Highest-10	◯	◯	◯	◯	◯	◯	◯	◯	◯	◯	◯

* LM 3.5 Protections: Extent to which advocacy exists for job protection, and social protection in the absence of jobs.

	1	2	3	4	5	6	7	8	9	10	Don't know
1-Lowest -> Highest-10	◯	◯	◯	◯	◯	◯	◯	◯	◯	◯	◯

LM 3.6 Comments: Please use this section to provide any additional comments about the level of effort, challenges, or successes in advocacy work related to LM. If you marked “I don’t know” for any of the questions, also use this section to explain why you gave that rating.

JOHNS HOPKINS
BLOOMBERG SCHOOL
of PUBLIC HEALTH

Bill & Melinda Gates Institute for Population and Reproductive Health

Demographic Dividend Effort Index Survey

Dimension 4. Research

* LM 4.1 Strength of Observatory/Stakeholder/Technical Working Group for the LM: Extent to which country-level LM stakeholders (e.g., stakeholder leadership group, technical working group, LM observatory, country coordination and facilitation group):

	1	2	3	4	5	6	7	8	9	10	Don't know
LM 4.1.1 Have representation in government, training institutions, civil society, nongovernmental and faith-based organizations, professional associations, the private sector, etc.	○	○	○	○	○	○	○	○	○	○	○
LM 4.1.2 Meet regularly, report and recommend policy for senior management within the appropriate ministries	○	○	○	○	○	○	○	○	○	○	○
LM 4.1.3 Make an impact on LM within the country	○	○	○	○	○	○	○	○	○	○	○

* LM 4.2 Labor Market Research Strategy: Extent to which the national documents include a comprehensive strategy/approach on labor market-related research and is shared among partners.

	1	2	3	4	5	6	7	8	9	10	Don't know
1-Lowest -> Highest-10	○	○	○	○	○	○	○	○	○	○	○

* LM 4.3 Labor Market Analysis Partners:

	1	2	3	4	5	6	7	8	9	10	Don't know
LM 4.3.1 Extent to which labor market research is undertaken by government agencies.											
LM 4.3.2 Extent to which labor market research is undertaken by research institutions.											
LM 4.3.3 Extent to which labor market research is undertaken by independent researchers.											

* LM 4.4 Areas of Research in the Labor Market:

	1	2	3	4	5	6	7	8	9	10	Don't know
LM 4.4.1 Extent to which unemployment trends and patterns are researched.											
LM 4.4.2 Extent of research on youth in the labor market.											
LM 4.4.3 Extent of research on women in the labor market.											
LM 4.4.4 Extent of research on the trends and trajectories of the labor market needs.											

* LM 4.5 Quality/Coverage of Data:

	1	2	3	4	5	6	7	8	9	10	Don't know
LM 4.5.1 Extent to which a routine statistical system provides reliable periodic labor market data.	○	○	○	○	○	○	○	○	○	○	○
LM 4.5.2 Extent to which the routine statistical system provides reliable periodic information on LM needs among populations in different communities.	○	○	○	○	○	○	○	○	○	○	○
LM 4.5.3 Extent to which evaluations of the monitoring and surveillance systems are conducted and applied to ensure the reliability of data.	○	○	○	○	○	○	○	○	○	○	○

* LM 4.6 Record-Keeping: Extent to which systems for labor market recordkeeping, reporting and feedback of results are adequate.

	1	2	3	4	5	6	7	8	9	10	Don't know
1-Lowest -> Highest-10	○	○	○	○	○	○	○	○	○	○	○

* LM 4.7 Quality Research Institutions: Extent to which the country has the capacity to sustain and maintain research institutions that develop research/collect labor market-related data.

	1	2	3	4	5	6	7	8	9	10	Don't know
1-Lowest -> Highest-10	○	○	○	○	○	○	○	○	○	○	○

* LM 4.8 Evaluation: Extent to which program statistics, surveys, and small studies are used by specialized staff to report on program operations and measure progress.

	1	2	3	4	5	6	7	8	9	10	Don't know
1-Lowest -> Highest-10	○	○	○	○	○	○	○	○	○	○	○

* LM 4.9 Management's Use of Evaluation Findings: Extent to which local-level program managers use research and evaluation findings to improve LM related services and programs in ways suggested by findings.

	1	2	3	4	5	6	7	8	9	10	Don't know
1-Lowest -> Highest-10	○	○	○	○	○	○	○	○	○	○	○

* LM 4.10 Ministerial Use of Evaluation Findings: Extent to which the Ministry administrators systematically utilize data to inform policies and interventions to address LM-related issues.

	1	2	3	4	5	6	7	8	9	10	Don't know
1-Lowest -> Highest-10	◯	◯	◯	◯	◯	◯	◯	◯	◯	◯	◯

* LM 4.11 Dissemination of Information to Other Implementing Bodies: Extent to which information is shared or spread across geographical areas and throughout various levels (national, state/province, localities).

	1	2	3	4	5	6	7	8	9	10	Don't know
1-Lowest -> Highest-10	◯	◯	◯	◯	◯	◯	◯	◯	◯	◯	◯

LM 4.12 Comments: Please use this section to provide any additional comments about the level of effort, challenges, or successes in the country around LM research. If you marked “I don’t know” for any of the questions, also use this section to explain why you gave that rating.

JOHNS HOPKINS BLOOMBERG SCHOOL of PUBLIC HEALTH

Bill & Melinda Gates Institute for Population and Reproductive Health

Demographic Dividend Effort Index Survey

Dimension 5. Civil Society Organizations (CSOs)

* LM 5.1 CSO Actor Power: Extent to which CSO actors are in strong positions to influence policies and action on the LM.

	1	2	3	4	5	6	7	8	9	10	Don't know
1-Lowest -> Highest-10	◯	◯	◯	◯	◯	◯	◯	◯	◯	◯	◯

* LM 5.2 Budget Analysis as a CSO Tool: Extent to which CSOs utilize budget analysis as a tool to build an advocacy case for labor market.

	1	2	3	4	5	6	7	8	9	10	Don't know
1-Lowest -> Highest-10	◯	◯	◯	◯	◯	◯	◯	◯	◯	◯	◯

* LM 5.3 Community-Based Service Delivery Support: Extent to which CSOs participate in bringing LM needs, information and services to the policy makers and vice-versa.

	1	2	3	4	5	6	7	8	9	10	Don't know
1-Lowest -> Highest-10	◯	◯	◯	◯	◯	◯	◯	◯	◯	◯	◯

* LM 5.4 Diversification of Job Opportunities: Extent to which CSOs and the private sector coordinate with the government to ensure optimal job creation and equal participation of women and men.

	1	2	3	4	5	6	7	8	9	10	Don't know
1-Lowest -> Highest-10	◯	◯	◯	◯	◯	◯	◯	◯	◯	◯	◯

* LM 5.5 Human Rights and Quality of Implementation: Extent to which CSOs support rights-based approaches at workplaces.

	1	2	3	4	5	6	7	8	9	10	Don't know
1-Lowest -> Highest-10	◯	◯	◯	◯	◯	◯	◯	◯	◯	◯	◯

* LM 5.6 Youth: Extent to which CSOs support labor market policies, interventions and programming specifically targeting youth.

	1	2	3	4	5	6	7	8	9	10	Don't know
1-Lowest -> Highest-10	○	○	○	○	○	○	○	○	○	○	○

* LM 5.7 Programming for Women: Extent to which CSOs support labor market policies, services and programs for women.

	1	2	3	4	5	6	7	8	9	10	Don't know
1-Lowest -> Highest-10	○	○	○	○	○	○	○	○	○	○	○

* LM 5.8 Advocacy/Accountability: Extent to which CSOs support the training and capacity building of advocates and community members to understand policies, processes, and activities to increase community-led issue identification, collaborative problem solving and more targeted advocacy actions to address unemployment.

	1	2	3	4	5	6	7	8	9	10	Don't know
1-Lowest -> Highest-10	○	○	○	○	○	○	○	○	○	○	○

* LM 5.9 CSO-Led Assessment and Monitoring: Extent to which CSOs assess, monitor and report on the effectiveness of policies and programming to improve accountability at the policy levels.

	1	2	3	4	5	6	7	8	9	10	Don't know
1-Lowest -> Highest-10	○	○	○	○	○	○	○	○	○	○	○

* LM 5.10 Forming Partnerships between CSOs: Extent to which the private sector has formed national alliances and regional partnerships to strengthen the labor market.

	1	2	3	4	5	6	7	8	9	10	Don't know
1-Lowest -> Highest-10	○	○	○	○	○	○	○	○	○	○	○

LM 5.11 Comments: Please use this section to provide any additional comments about the level of effort, challenges, or successes on the role of the CSO in the LM. If you marked “I don’t know” for any of the questions, also use this section to explain why you gave that rating

JOHNS HOPKINS
BLOOMBERG SCHOOL
of PUBLIC HEALTH

Bill & Melinda Gates Institute for Population and Reproductive Health

Demographic Dividend Effort Index Survey

Module: Sectoral Resilience and Sustainability

Considering the health and socio-economic impact of the COVID-19 and the likely impact on the DD progress, this DD effort index has integrated questions to assess the resilience and sustainability of systems in key sectors of the DD framework. The items included in the COVID-19 related questionnaire below follow the key dimensions of resilient systems and provide critical information on the potential of an effective response to emerging infectious disease threats or other public health emergencies.

Please score each item from 1 to 10, where 1 represents the lowest score (poor state/capability) and 10 represents the highest score (great state/capability).

* Physical: facilities, equipment, system states, and capabilities.

	1	2	3	4	5	6	7	8	9	10	Don't know
LM-M1 - Plan/Prepare - State and capability of equipment, personnel and LM sector structure prior to the crisis event. (1 = poor state/capability; 10 = great state/capability)	○	○	○	○	○	○	○	○	○	○	○
LM-M2 - Absorb - Extent to which COVID-19 was recognized within the LM sector and the performance of the system was able to maintain optimal employment and economic productivity.	○	○	○	○	○	○	○	○	○	○	○
LM-M3 - Recover - Extent to which the LM sector has been able to induce change to recover the pre-COVID-19 level of functionality.	○	○	○	○	○	○	○	○	○	○	○

	1	2	3	4	5	6	7	8	9	10	Don't know
LM-M4 - Recover - Financing and Donor Management: Extent to which there are established social protection mechanisms (and other forms of income smoothing) for unprotected workers (e.g. self-employed workers, that have no access to paid or sick leave).	○	○	○	○	○	○	○	○	○	○	○
LM-M5 - Adapt - Financing and Donor Management: Extent to which there has been institutional and policy reforms to build resilience by a demand-led approach through large-scale social protection systems that can act as economic stabilizers	○	○	○	○	○	○	○	○	○	○	○

* Information: creation, manipulation, storage, and utilization of data.

	1	2	3	4	5	6	7	8	9	10	Don't know
LM-M6 - Plan/Prepare - Extent to which LM-related data was sufficiently prepared, presented, analyzed and stored prior to the crisis event.	◯	◯	◯	◯	◯	◯	◯	◯	◯	◯	◯
LM-M7 - Absorb - Extent to which the LM sector has been able to conduct a real-time assessment of functionality, in anticipation of cascading losses in LM and productivity.	◯	◯	◯	◯	◯	◯	◯	◯	◯	◯	◯
LM-M8 - Recover - Extent to which LM data has been used to track the progress of recovery and to anticipate recovery scenarios.	◯	◯	◯	◯	◯	◯	◯	◯	◯	◯	◯
LM-M9 - Adapt - Extent to which the LM sector is creating and improving LM data storage and real-time use protocols.	◯	◯	◯	◯	◯	◯	◯	◯	◯	◯	◯

* Cognitive: understanding, mental models, preconceptions, biases, and values.

	1	2	3	4	5	6	7	8	9	10	Don't know
LM-M10 - Plan/Prepare - Extent to which the LM sector design and operation decisions were prepared in anticipation of crisis events.	◯	◯	◯	◯	◯	◯	◯	◯	◯	◯	◯
LM-M11 - Absorb - Extent to which the LM sector is responding with sufficient contingency protocols and proactive crisis management.	◯	◯	◯	◯	◯	◯	◯	◯	◯	◯	◯

	1	2	3	4	5	6	7	8	9	10	Don't know
LM-M12 - Absorb - Leadership and Management: Extent to which the government is ensuring workers and employers and their families are protected from the health risks of COVID-19.	○	○	○	○	○	○	○	○	○	○	○
LM-M13 - Absorb - Accountability: Level of effort to ensure optimal accountability for the resources allocated to ensure protection from the health risks of COVID-19 and the recovery of the labor market as part of the response to mitigate COVID-19 impacts.	○	○	○	○	○	○	○	○	○	○	○
LM-M14 - Recover - Extent to which decisions are oriented towards recovery, which employ evidence-based communication to community members to promote safe behavior.	○	○	○	○	○	○	○	○	○	○	○
LM-M15 - Recover - Awareness: Extent to which the government is building confidence through trust and dialogue for building the commitment of employers and workers to the joint action of the government and promoting enterprise-level social dialogue.	○	○	○	○	○	○	○	○	○	○	○
LM-M16 - Adapt - Extent to which the LM sector is designing new system configurations, objectives and decision criteria.	○	○	○	○	○	○	○	○	○	○	○

	1	2	3	4	5	6	7	8	9	10	Don't know
LM-M17 - Adapt - Adaptive: Extent to which the government is promoting change in the workspace to protect workers (e.g. occupational and safety health measures, adapted work arrangements, prevention of discrimination and exclusion, expanded access to paid leave, etc.).	○	○	○	○	○	○	○	○	○	○	○

* Social: interaction, collaboration, and self-synchronization between individuals, entities and institutions.

	1	2	3	4	5	6	7	8	9	10	Don't know
LM-M18 - Plan/Prepare - Extent to which training on outbreak/crisis management was conducted through, and which leveraged, social networks, social capital and institutional and cultural norms prior to the crisis event.	○	○	○	○	○	○	○	○	○	○	○
LM-M19 - Absorb - Extent to which personnel and social institutions have been resourceful and accessible for the outbreak/crisis response.	○	○	○	○	○	○	○	○	○	○	○
LM-M20 - Absorb - Self-Regulating: Extent to which the national leadership had the authority to make sectoral changes in a timely manner through flexible labor market infrastructure.	○	○	○	○	○	○	○	○	○	○	○
LM-M21 - Recover - Extent to which the LM sector is engaging with team and knowledge sharing to enhance systematic recovery.	○	○	○	○	○	○	○	○	○	○	○

	1	2	3	4	5	6	7	8	9	10	Don't know
LM-M22 - Recover - Diversity: Extent to which the government is facilitating a strong and fast recovery of the labor market by diversifying policies that favor both the supply (production of good and services) and demand (consumption and investment).	◯	◯	◯	◯	◯	◯	◯	◯	◯	◯	◯
LM-M23 - Adapt - Extent to which there are additions or changes being made to LM institutions, policies, trainings, programs and culture.	◯	◯	◯	◯	◯	◯	◯	◯	◯	◯	◯
LM-M24 - Adapt - Leadership and Management: Extent to which there has been established a tripartite fluent dialogue between government, workers, and employers' organizations.	◯	◯	◯	◯	◯	◯	◯	◯	◯	◯	◯

* LM M-25 Timeline: Extent of timeliness of the Government reaction - communication and implementation of measures - to mitigate the immediate and longterm impact of COVID-19.
Note: This refers to the entirety of the national reaction - not the reaction that is specific to your sector.

	1	2	3	4	5	6	7	8	9	10	Don't know
1-Lowest -> Highest-10	◯	◯	◯	◯	◯	◯	◯	◯	◯	◯	◯

LM M-26 Comments: Please use this section to provide any additional comments about the resilience and sustainability of the LM systems based on your experience during the COVID-19 pandemics. If you marked “I don’t know” for any of the questions, also use this section to explain why you gave that rating.

JOHNS HOPKINS
BLOOMBERG SCHOOL
of PUBLIC HEALTH

Bill & Melinda Gates Institute for Population and Reproductive Health

Demographic Dividend Effort Index Survey

Governance and Economic Institutions (GEI) Questionnaire

Please respond to the following questions based on your experience/expertise within this specific sector.

To give a summary picture of the national demographic dividend effort, please rate the following items. Score each item from 1 to 10, where 1 represents the lowest score (very weak or no effort) and 10 represents the highest score (strong effort). Where applicable, if a policy or activity is non-existent then mark your response as 0.

Answer each item. All responses will be recorded in the format exemplified below:

Lowest effort	1	2	3	4	5	6	7	8	9	10	Highest effort	I do not know

JOHNS HOPKINS
BLOOMBERG SCHOOL
of PUBLIC HEALTH

Bill & Melinda Gates Institute for Population and Reproductive Health

Demographic Dividend Effort Index Survey

Dimension 1. Policies

* GEI 1.1 Democratic Values: Extent to which the government has ratified, domesticated and implemented regional instruments for shared values, including those relating to democracy, elections and governance and the right to health.

	1	2	3	4	5	6	7	8	9	10	Don't know
1-Lowest -> Highest-10	◯	◯	◯	◯	◯	◯	◯	◯	◯	◯	◯

* GEI 1.2 Monitoring Democratic Values: Extent to which the government has created mechanisms for regular and periodic reporting on the implementation of the regional instruments on shared values, through existing mechanisms such as universal periodic review on the status of international or regional instruments or using independent bodies. (This may take the form of regular reports on the status of implementation).

	1	2	3	4	5	6	7	8	9	10	Don't know
1-Lowest -> Highest-10	◯	◯	◯	◯	◯	◯	◯	◯	◯	◯	◯

* GEI 1.3 Affirmation of Youth Democratic Participation: Extent to which the government has explicitly removed all discriminatory laws and limitations to full participation of young people in electoral processes through inclusive electoral laws and constitutions. (This may include developing and/or strengthening youth leadership programs and/or providing capacity building on management and leadership skills).

	1	2	3	4	5	6	7	8	9	10	Don't know
1-Lowest -> Highest-10	◯	◯	◯	◯	◯	◯	◯	◯	◯	◯	◯

* GEI 1.4. Removing Legal Youth Barriers: Extent to which the government has proscribed all laws posing barriers to the full exercise and enjoyment of the fundamental rights of young people to fully participate in the democratic governance processes at the continental, regional, national and grassroots levels. (This may also include sustaining the mainstreaming of adolescents and youth health and rights issues into the country's growth and transformation agenda).

	1	2	3	4	5	6	7	8	9	10	Don't know
1-Lowest -> Highest-10	○	○	○	○	○	○	○	○	○	○	○

* GEI 1.5 Youth Policy: Extent to which the youth policy takes into account the latest thinking in youth education, training and employment.

	1	2	3	4	5	6	7	8	9	10	Don't know
1-Lowest -> Highest-10	○	○	○	○	○	○	○	○	○	○	○

* GEI 1.6 Affirmation of Democratic Participation/Revision of Customary Law: Extent to which the government has reviewed, revised, amended or abolished all laws, regulations, policies, practices and customs that have a discriminatory impact on youth, especially girls and young women, without distinction of any kind, and ensure that the provisions of multiple legal systems comply with international human rights regulations and laws. (These must include protection from harmful practices like early, forced or child marriages, sexual and gender-based violence, female genital mutilation (FGM).

	1	2	3	4	5	6	7	8	9	10	Don't know
1-Lowest -> Highest-10	○	○	○	○	○	○	○	○	○	○	○

* GEI 1.7 Youth Policymaking Participation: Extent to which the government ensures the involvement of youth leadership and participation in decision-making processes in efforts relating to policymaking including youth and education policy making. Potentially includes efforts to establish a national youth parliament and assembly. (This may include supporting adolescent and youth participation and leadership in health planning and programming at all levels).

	1	2	3	4	5	6	7	8	9	10	Don't know
1-Lowest -> Highest-10	○	○	○	○	○	○	○	○	○	○	○

* GEI 1.8 Rule of Law: Extent to which the rule of law is formally, legally established to be equitable and implemented independently by the judiciary.

	1	2	3	4	5	6	7	8	9	10	Don't know
1-Lowest -> Highest-10	○	○	○	○	○	○	○	○	○	○	○

* GEI 1.9 Transparency and Accountability: Extent to which national legislation mandates transparency and accountability across all branches and throughout all sectors.

	1	2	3	4	5	6	7	8	9	10	Don't know
GEI 1.9.1 Extent to which the public can access public and legislative information as well as the records of state-owned organizations.	○	○	○	○	○	○	○	○	○	○	○
GEI 1.9.2 Extent to which there are mandated actions and mechanisms for accountability and sanctioning.	○	○	○	○	○	○	○	○	○	○	○

* GEI 1.10 National Security: Extent to which the country/government is involved in armed conflict, either internationally or domestically.

	1	2	3	4	5	6	7	8	9	10	Don't know
GEI 1.10.1 Extent to which the country has cross-border tensions.	○	○	○	○	○	○	○	○	○	○	○
GEI 1.10.2 Extent to which the country contains internally displaced persons and/or refugees.	○	○	○	○	○	○	○	○	○	○	○
GEI 1.10.3 Extent to which education facilities are protected and alternative learning opportunities are created for children and youth in time of conflict or displacement.	○	○	○	○	○	○	○	○	○	○	○

* GEI 1.11 Rights: Extent to which the constitution affirms certain rights: freedom of expression, freedom of association and assembly, civil rights and liberties, and protection against ethnic and religious discrimination, health as well as an un-likelihood of human rights abuses by the government.

	1	2	3	4	5	6	7	8	9	10	Don't know
1-Lowest -> Highest-10	○	○	○	○	○	○	○	○	○	○	○

* GEI 1.12 Education and Democratic Values: Extent to which education policies, strategies and programs promote academic freedom, democratic values and education as human right.

	1	2	3	4	5	6	7	8	9	10	Don't know
1-Lowest -> Highest-10	◯	◯	◯	◯	◯	◯	◯	◯	◯	◯	◯

* GEI 1.13 Affirmation of Safe Learning Environments: Extent to which education policies provide for safe learning environments through protections for children. (This may come in the form of ensuring the safety of girls, banning corporal punishment, etc.).

	1	2	3	4	5	6	7	8	9	10	Don't know
1-Lowest -> Highest-10	◯	◯	◯	◯	◯	◯	◯	◯	◯	◯	◯

GEI 1.14 Comments: Please use this section to provide any additional comments about the level of effort, challenges, or successes in the country around policies/policymaking for GEI. If you marked “I don’t know” for any of the questions, also use this section to explain why you gave that rating

JOHNS HOPKINS
BLOOMBERG SCHOOL
of PUBLIC HEALTH

Bill & Melinda Gates Institute for Population and Reproductive Health

Demographic Dividend Effort Index Survey

Dimension 2. Services or Programs

* GEI 2.1 Strengthening Youth Activities/Institutions: Extent to which efforts are being made to strengthen independent youth formations, networks and organizations including the establishment of independent youth commissions, student unions at the national and sub-regional levels to champion youth rights.

	1	2	3	4	5	6	7	8	9	10	Don't know
1-Lowest -> Highest-10	○	○	○	○	○	○	○	○	○	○	○

* GEI 2.2 Appointment of Youth Leader: Whether and to what extent there has been a youth envoy appointed to lead advocacy and champion the prioritization of youth issues (e.g. education, training and employment) within decision-making spaces at a variety of levels including the parliament.

	1	2	3	4	5	6	7	8	9	10	Don't know
1-Lowest -> Highest-10	○	○	○	○	○	○	○	○	○	○	○

* GEI 2.3 Youth Ideals and Aspirations: Extent to which programming is designed to institute youth leadership and empowerment trainings aimed at deepening cultural ideals and aspirations in the youth.

	1	2	3	4	5	6	7	8	9	10	Don't know
1-Lowest -> Highest-10	○	○	○	○	○	○	○	○	○	○	○

* GEI 2.4 Youth Services: Extent to which programming is designed to address youth-specific needs. (This should consider the development and implementation of adolescent and youth-friendly health services, among other similar programmatic endeavors).

	1	2	3	4	5	6	7	8	9	10	Don't know
1-Lowest -> Highest-10	○	○	○	○	○	○	○	○	○	○	○

For peer review only - http://bmjopen.bmj.com/site/about/guidelines.xhtml

* GEI 2.5 Youth Involvement in National Initiatives: Extent to which there are efforts to strengthen existing youth engagement and participation initiatives of the country *(e.g. at the level of the African Union these may include the African Youth Volunteers Corps, African Union Youth Clubs, AGA-YES activities including the Youth Advisory Panel, the Moot Court and Annual Youth Dialogue on Democracy, Human Rights and Governance*). (This may include engaging adolescents and youth in the provision of technical support to sector ministries and relevant federal and regional government bodies for developing policies, plans, and programs that integrate responses to priority health issues affecting this group).

	1	2	3	4	5	6	7	8	9	10	Don't know
1-Lowest -> Highest-10	○	○	○	○	○	○	○	○	○	○	○

* GEI 2.6 Personal Safety: Extent to which individual safety is provided for through equitable, reliable police services/activities, contributing to an overall positive perception of personal safety at the household and community levels as well as the school environment. (This may include protecting youth form any form of abuse, neglect or disrespect in the use of health services).

	1	2	3	4	5	6	7	8	9	10	Don't know
1-Lowest -> Highest-10	○	○	○	○	○	○	○	○	○	○	○

* GEI 2.7 Participation: Extent to which government functions involve the participation of citizens, civil society organizations, and other intersectoral actors to promote democratic elections and the effective power to govern.

	1	2	3	4	5	6	7	8	9	10	Don't know
1-Lowest -> Highest-10	○	○	○	○	○	○	○	○	○	○	○

* GEI 2.8 Gender: Extent to which women participate in:

	1	2	3	4	5	6	7	8	9	10	Don't know
GEI 2.8.1 Governance at the national level	○	○	○	○	○	○	○	○	○	○	○
GEI 2.8.2 Governance at the sub-national level	○	○	○	○	○	○	○	○	○	○	○
GEI 2.8.3 Governance in rural areas	○	○	○	○	○	○	○	○	○	○	○
GEI 2.8.4 Governance in urban areas	○	○	○	○	○	○	○	○	○	○	○
GEI 2.8.5 Judiciary	○	○	○	○	○	○	○	○	○	○	○

* GEI 2.9 Promotion of Equality: Extent to which gender equality in all aspects of society is promoted by the government. (This may include equality in education, equality before law, equality in healthcare, equality in employment and renumeration, among many other facets of society).

	1	2	3	4	5	6	7	8	9	10	Don't know
1-Lowest -> Highest-10	○	○	○	○	○	○	○	○	○	○	○

* GEI 2.10 Sustainable Economic Opportunity:

	1	2	3	4	5	6	7	8	9	10	Don't know
GEI 2.10.1 Extent to which the government provides for and ensures that a strong public management system is realized.	○	○	○	○	○	○	○	○	○	○	○
GEI 2.10.2 Extent to which the government provides for and ensures that a strong business environment is realized.	○	○	○	○	○	○	○	○	○	○	○
GEI 2.10.3 Extent to which the government provides for and ensures that a strong public development infrastructure is realized.	○	○	○	○	○	○	○	○	○	○	○
GEI 2.10.4 Extent to which the government provides for and ensures that a strong rural sector for development is realized.	○	○	○	○	○	○	○	○	○	○	○

* GEI 2.11 Human Development:

	1	2	3	4	5	6	7	8	9	10	Don't know
GEI 2.11.1 Extent to which the welfare of the general public is well-handled by the government (consider aspects related, but not limited, to welfare policies and services, socioeconomic integration of youth and social protections and safety nets, among many other areas). This may also that the government, with the obligations to protect, fulfil and respect basic health rights, has given the responsibility of its implementation to its ministerial cabinet.	○	○	○	○	○	○	○	○	○	○	○
GEI 2.11.2 Extent to which the government is promoting the skills development of the population at workplaces.	○	○	○	○	○	○	○	○	○	○	○
GEI 2.11.3 Extent to which the government is promoting the education, knowledge and capacities of the population through schools and continuing education opportunities.	○	○	○	○	○	○	○	○	○	○	○
GEI 2.11.4 Extent to which the government is promoting the health and wellness of the population.	○	○	○	○	○	○	○	○	○	○	○

GEI 2.12 Comments: Please use this section to provide any additional comments about the level of effort, challenges, or successes in the country around services or programs for GEI. If you marked “I don’t know” for any of the questions, also use this section to explain why you gave that rating.

JOHNS HOPKINS
BLOOMBERG SCHOOL
of PUBLIC HEALTH

Bill & Melinda Gates Institute for Population and Reproductive Health

Demographic Dividend Effort Index Survey

Dimension 3. Advocacy

* GEI 3.1 Youth Empowerment: Extent to which youth empowerment has been enhanced, potentially through the integration of civic education in national educational curricula, media platforms and other channels to instill principles of the rule of law, human rights and individual duties and responsibilities. (This may include the mainstreaming of adolescents and youth health and rights issues into the country’s growth and transformation agenda and educational curricula).

	1	2	3	4	5	6	7	8	9	10	Don't know
1-Lowest -> Highest-10	○	○	○	○	○	○	○	○	○	○	○

* GEI 3.2 Government Advocacy: Extent to which members of the government advocates for improvements within its own structures and priorities. (These improvements may include progress for youth inclusion, opportunities, programming and rights among similar concepts across the population).

	1	2	3	4	5	6	7	8	9	10	Don't know
1-Lowest -> Highest-10	○	○	○	○	○	○	○	○	○	○	○

* GEI 3.3 Non-governmental Advocacy: Extent to which non-governmental actors advocates for improvements in government structures and priorities. (These improvements may include progress for youth inclusion, opportunities, programming and rights among similar concepts across the population).

	1	2	3	4	5	6	7	8	9	10	Don't know
1-Lowest -> Highest-10	○	○	○	○	○	○	○	○	○	○	○

* GEI 3.4 Civil Society Organizations (CSOs): Extent to which CSOs specifically operate as instruments for advocacy in the GEI sector.

	1	2	3	4	5	6	7	8	9	10	Don't know
1-Lowest -> Highest-10	○	○	○	○	○	○	○	○	○	○	○

* GEI 3.5 Advocacy Voice: Extent to which advocates have a voice in government discussions.

	1	2	3	4	5	6	7	8	9	10	Don't know
1-Lowest -> Highest-10											

* GEI 3.6 Voice of Instrumental Sectors of the DD: Extent to which the government and stakeholders are committed to establish sustainable institutions to ensure:

	1	2	3	4	5	6	7	8	9	10	Don't know
GEI 3.6.1 Good governance											
GEI 3.6.2 Financial stability											
GEI 3.6.3 Optimal family planning programs											
GEI 3.6.4 Optimal maternal and child health											
GEI 3.6.5 Good quality education											
GEI 3.6.6 Diversified labor market.											

GEI 3.7 Comments: Please use this section to provide any additional comments about the level of effort, challenges, or successes in the country around advocacy for GEI. If you marked “I don’t know” for any of the questions, also use this section to explain why you gave that rating

JOHNS HOPKINS
BLOOMBERG SCHOOL
of PUBLIC HEALTH

Bill & Melinda Gates Institute for Population and Reproductive Health

Demographic Dividend Effort Index Survey

Dimension 4. Research

* GEI 4.1 Strength of Observatory/Stakeholder/Technical Working Group for GEI: Extent to which country-level GEI stakeholders (e.g., stakeholder leadership group, technical working group, GEI observatory, country coordination and facilitation group):

	1	2	3	4	5	6	7	8	9	10	Don't know
GEI 4.1.1 Have representation in government, training institutions, civil society, nongovernmental and faith-based organizations, professional associations, the private sector, etc.	○	○	○	○	○	○	○	○	○	○	○
GEI 4.1.2 Meet regularly, report and recommend policy for senior management within the appropriate ministries	○	○	○	○	○	○	○	○	○	○	○
GEI 4.1.3 Make an impact on GEI within the country	○	○	○	○	○	○	○	○	○	○	○

* GEI 4.2 Education/Research Strategy: Extent to which national planning documents include a comprehensive strategy/approach on improving GEI, shared among partners.

	1	2	3	4	5	6	7	8	9	10	Don't know
1-Lowest -> Highest-10	○	○	○	○	○	○	○	○	○	○	○

* GEI 4.3 Governance, Financial and Development Data Gathering Partners:

	1	2	3	4	5	6	7	8	9	10	Don't know
GEI 4.3.1 Extent to which research is undertaken by government agencies. (This may include statistical agencies/bureaus and/or ministries of economics/finances, education, health, gender, etc.)	◯	◯	◯	◯	◯	◯	◯	◯	◯	◯	◯
GEI 4.3.2 Extent to which research is undertaken by public research institutions.	◯	◯	◯	◯	◯	◯	◯	◯	◯	◯	◯
GEI 4.3.3 Extent to which research is undertaken by independent researchers.	◯	◯	◯	◯	◯	◯	◯	◯	◯	◯	◯

* GEI 4.4 Governance, Financial and Development Topical Data Gathering:

	1	2	3	4	5	6	7	8	9	10	Don't know
GEI 4.4.1 Extent to which safety and the rule of law is researched.	◯	◯	◯	◯	◯	◯	◯	◯	◯	◯	◯
GEI 4.4.2 Extent to which political participation and human rights is measured.	◯	◯	◯	◯	◯	◯	◯	◯	◯	◯	◯
GEI 4.4.3 Extent to which indicators of a sustainable economy are researched and disseminated to the community.	◯	◯	◯	◯	◯	◯	◯	◯	◯	◯	◯
GEI 4.4.4 Extent to which human capital development (education, training, and employment, etc.) is researched.	◯	◯	◯	◯	◯	◯	◯	◯	◯	◯	◯

* GEI 4.5 Quality/Coverage of Data:

	1	2	3	4	5	6	7	8	9	10	Don't know
GEI 4.5.1 Extent to which a routine statistical system provides reliable periodic information on governance indicators.	○	○	○	○	○	○	○	○	○	○	○
GEI 4.5.2 Extent to which the routine statistical system provides reliable periodic information on governance-related needs among populations in different communities.	○	○	○	○	○	○	○	○	○	○	○
GEI 4.5.3 Extent to which a routine statistical system provides reliable periodic information on economic indicators.	○	○	○	○	○	○	○	○	○	○	○
GEI 4.5.4 Extent to which evaluations of the monitoring and surveillance systems are conducted and applied to ensure the reliability of data.	○	○	○	○	○	○	○	○	○	○	○

* GEI 4.6 Quality Research Institutions: Extent to which the country has the capacity to support and sustain research institutions that develop research/collect data to inform efforts to ensure good governance, stable economy and strong public institutions.

	1	2	3	4	5	6	7	8	9	10	Don't know
1-Lowest -> Highest-10	○	○	○	○	○	○	○	○	○	○	○

* GEI 4.7 Evaluation: Extent to which program statistics, surveys, and small studies are used by specialized staff to report on good governance and financial institutions.

	1	2	3	4	5	6	7	8	9	10	Don't know
1-Lowest -> Highest-10	○	○	○	○	○	○	○	○	○	○	○

* GEI 4.8 Management's Use of Evaluation Findings: Extent to which local-level program managers use research and evaluation findings to improve programming around the rule of law, stabilizing the economy and strengthening institutions in ways suggested by findings.

	1	2	3	4	5	6	7	8	9	10	Don't know
1-Lowest -> Highest-10	◯	◯	◯	◯	◯	◯	◯	◯	◯	◯	◯

* GEI 4.10 Dissemination of Information to Other Implementing Bodies: Extent to which information is shared or spread across geographical areas and throughout various levels (national, regional, localities).

	1	2	3	4	5	6	7	8	9	10	Don't know
1-Lowest -> Highest-10	◯	◯	◯	◯	◯	◯	◯	◯	◯	◯	◯

GEI 4.11 Comments: Please use this section to provide any additional comments about the level of effort, challenges, or successes in research around GEI. If you marked "I don't know" for any of the questions, also use this section to explain why you gave that rating.

JOHNS HOPKINS
BLOOMBERG SCHOOL
of PUBLIC HEALTH

Bill & Melinda Gates Institute for Population and Reproductive Health

Demographic Dividend Effort Index Survey

Dimension 5. Civil Society Organizations (CSOs)

* GEI 5.1 CSO Actor Power: Extent to which CSO actors are in strong positions to positively contribute to the rule of law.

	1	2	3	4	5	6	7	8	9	10	Don't know
1-Lowest -> Highest-10	◯	◯	◯	◯	◯	◯	◯	◯	◯	◯	◯

* GEI 5.2 Budget Analysis as a CSO Tool: Extent to which CSOs utilize budget analysis as a tool to build an advocacy case for good governance, economy stability and stronger public institutions

	1	2	3	4	5	6	7	8	9	10	Don't know
1-Lowest -> Highest-10	◯	◯	◯	◯	◯	◯	◯	◯	◯	◯	◯

* GEI 5.3 Community-Based Service Delivery Support: Extent to which CSOs participate in bringing governance-related information and services to the communities where people live.

	1	2	3	4	5	6	7	8	9	10	Don't know
1-Lowest -> Highest-10	◯	◯	◯	◯	◯	◯	◯	◯	◯	◯	◯

* GEI 5.4 Human Rights and Quality of Implementation: Extent to which CSOs advocate for and support rights-based approaches to governance to ensure policy and program beneficiaries experience progress on their terms.

	1	2	3	4	5	6	7	8	9	10	Don't know
1-Lowest -> Highest-10	◯	◯	◯	◯	◯	◯	◯	◯	◯	◯	◯

* GEI 5.5 Youth: Extent to which CSOs support youth to participate in education, training, employment and leadership.

	1	2	3	4	5	6	7	8	9	10	Don't know
1-Lowest -> Highest-10	◯	◯	◯	◯	◯	◯	◯	◯	◯	◯	◯

* GEI 5.6 Programs for Women: Extent to which CSOs ensure GEI policies support women and girls to participate in leadership.

	1	2	3	4	5	6	7	8	9	10	Don't know
1-Lowest -> Highest-10	◯	◯	◯	◯	◯	◯	◯	◯	◯	◯	◯

* GEI 5.7 Advocacy/Accountability

	1	2	3	4	5	6	7	8	9	10	Don't know
GEI 5.7.1 Extent to which CSOs support the participation of the community in governance or enforcement of the rule of law.	◯	◯	◯	◯	◯	◯	◯	◯	◯	◯	◯
GEI 5.7.2 Extent to which the media support the participation of the community in governance or enforcement of the rule of law.	◯	◯	◯	◯	◯	◯	◯	◯	◯	◯	◯

* GEI 5.8 CSO-Led Assessment and Monitoring: Extent to which CSOs assess, monitor and report on the effectiveness of policies and government accountability mechanisms and structures.

	1	2	3	4	5	6	7	8	9	10	Don't know
1-Lowest -> Highest-10	◯	◯	◯	◯	◯	◯	◯	◯	◯	◯	◯

* GEI 5.9 Forming Partnerships between CSOs: Extent to which CSOs have formed national alliances and regional partnerships to strengthen their positioning and potentially improve leadership strength and funding mechanisms for good governance and institutional strengthening related work.

	1	2	3	4	5	6	7	8	9	10	Don't know
1-Lowest -> Highest-10	◯	◯	◯	◯	◯	◯	◯	◯	◯	◯	◯

GEI 5.10 Comments: Please use this section to provide any additional comments about the level of effort, challenges, or successes around the role of the CSOs and media in GEI. If you marked “I don’t know” for any of the questions, also use this section to explain why you gave that rating

JOHNS HOPKINS
BLOOMBERG SCHOOL of PUBLIC HEALTH

Bill & Melinda Gates Institute for Population and Reproductive Health

Demographic Dividend Effort Index Survey

Module: Sectoral Resilience and Sustainability

Considering the health and socio-economic impact of the COVID-19 and the likely impact on the DD progress, this DD effort index has integrated questions to assess the resilience and sustainability of systems in key sectors of the DD framework. The items included in the COVID-19 related questionnaire below follow the key dimensions of resilient systems and provide critical information on the potential of an effective response to emerging infectious disease threats or other public health emergencies.

Please score each item from 1 to 10, where 1 represents the lowest score (poor state/capability) and 10 represents the highest score (great state/capability).

* Physical: facilities, equipment, system states, and capabilities.

	1	2	3	4	5	6	7	8	9	10	Don't know
GEI-M1 - Plan/Prepare - State and capability of equipment, personnel and GEI sector system structure prior to the crisis event. (1 = poor state/capability; 10 = great state/capability)	○	○	○	○	○	○	○	○	○	○	○
GEI-M2 - Absorb - Extent to which COVID-19 was recognized within the GEI sector system and the performance of the system was able to maintain functionality.	○	○	○	○	○	○	○	○	○	○	○
GEI-M3 - Absorb - Integration: The level of integration of services and institutional collaborations to ensure continuity of access to essential services during the COVID-19 crisis.	○	○	○	○	○	○	○	○	○	○	○
GEI-M4 - Recover - Extent to which the Government and Economic Institutions have been able to induce change to recover the pre-COVID-19 level of functionality.	○	○	○	○	○	○	○	○	○	○	○
GEI-M5 - Recover - Financing and Donor Management: National bank and donor support to ensure the availability of cash during the COVID-19 crisis	○	○	○	○	○	○	○	○	○	○	○
GEI-M6 - Adapt - Extent to which changes have been made to improve the resilience of the GEI sector system and infrastructure.	○	○	○	○	○	○	○	○	○	○	○

* Information: creation, manipulation, storage, and utilization of data.

	1	2	3	4	5	6	7	8	9	10	Don't know
GEI-M7 - Plan/Prepare - Extent to which GEI-related data was sufficiently prepared, presented, analyzed and stored prior to the crisis event.	○	○	○	○	○	○	○	○	○	○	○
GEI-M8 - Absorb - Awareness: Level of awareness about the threats caused by COVID-19 in the central government.	○	○	○	○	○	○	○	○	○	○	○
GEI-M9 - Recover - Extent to which GEI data has been used to track the progress of recovery and to anticipate recovery scenarios.	○	○	○	○	○	○	○	○	○	○	○
GEI-M10 - Adapt - Extent to which the GEI sector system is creating and improving GEI data storage and real-time use protocols.	○	○	○	○	○	○	○	○	○	○	○

* Cognitive: understanding, mental models, preconceptions, biases, and values.

	1	2	3	4	5	6	7	8	9	10	Don't know
GEI-M11 - Plan/Prepare - Extent to which the GEI sector system design and operation decisions were prepared in anticipation of crisis events.	○	○	○	○	○	○	○	○	○	○	○
GEI-M12 - Absorb - Extent to which the Government and Economic Institutions are responding with sufficient contingency protocols and proactive crisis management.	○	○	○	○	○	○	○	○	○	○	○

	1	2	3	4	5	6	7	8	9	10	Don't know
GEI-M13 - Absorb - Accountability: Level of effort to ensure optimal accountability for the resources allocated to support governance as part of the response to the COVID-19.	○	○	○	○	○	○	○	○	○	○	○
GEI-M14 - Recover - Extent to which decisions are oriented towards recovery, which employ evidence-based communication to community members to promote safe behavior.	○	○	○	○	○	○	○	○	○	○	○
GEI-M15 - Recover - Awareness: Extent to which the government is building confidence among the public though communication and dialogue for evidence-based policymaking and actions.	○	○	○	○	○	○	○	○	○	○	○
GEI-M16 - Adapt - Extent to which the GEI sector system is designing new system configurations, objectives and decision criteria.	○	○	○	○	○	○	○	○	○	○	○
GEI-M17 - Adapt - Adaptive: Level of adaptation of financial and social service systems to ensure easy access via technology-based platforms during the COVID-19.	○	○	○	○	○	○	○	○	○	○	○

* Social: interaction, collaboration, and self-synchronization between individuals, entities and institutions.

	1	2	3	4	5	6	7	8	9	10	Don't know
GEI-M18 - Plan/Prepare - Extent to which training on outbreak/crisis management was conducted through, and which leveraged, social networks, social capital and institutional and cultural norms prior to the crisis event.											
GEI-M19 - Absorb - Extent to which personnel and social institutions have been resourceful and accessible for the outbreak/crisis response.											
GEI-M20 - Absorb - Self-Regulating: Extent to which the national leadership had the authority to make sectoral changes in a timely manner through flexible infrastructure.											
GEI-M21 - Recover - Extent to which the GEI sector system is engaging with team and knowledge sharing to enhance systematic recovery.											
GEI-M22 - Recover - Diversity: The level of engagement of multidisciplinary taskforces to mitigate the effect of COVID-19 at the national and subnational levels.											
GEI-M23 - Adapt - Leadership and Management: Stewardship and management demonstrated by Government in response to the COVID-19 at the national level.											

* GEI M-24 Timeline: Extent of timeliness of the Government reaction - communication and implementation of measures - to mitigate the immediate and longterm impact of COVID-19.
Note: This refers to the entirety of the national reaction - not the reaction that is specific to your sector.

	1	2	3	4	5	6	7	8	9	10	Don't know
1-Lowest -> Highest-10	◯	◯	◯	◯	◯	◯	◯	◯	◯	◯	◯

GEI M-25 Comments: Please use this section to provide any additional comments about the resilience of the national GEI systems based on your experience during the COVID-19 pandemics.If you marked “I don’t know” for any of the questions, also use this section to explain why you gave that rating.

JOHNS HOPKINS
BLOOMBERG SCHOOL
of PUBLIC HEALTH

Bill & Melinda Gates Institute for Population and Reproductive Health

Enquête de l'Indice des efforts pour le dividende démographique

Introduction

Les programmes de développement ont besoin d'une mesure standard qui quantifie la portée des efforts nationaux en termes de politiques et programmes mis en œuvre pour cultiver, réaliser et exploiter le dividende démographique (DD). En dépit du progrès remarquable accompli par plusieurs pays à travers l'intégration des principes du DD dans leurs politiques et plans de développement, des outils d'évaluation de la performance et des plateformes documentant les leçons et meilleures pratiques pour bénéficier du DD font toujours défaut.

Ainsi, l'objectif de l'Indice des efforts pour le dividende démographique (IEDD) est de fournir une mesure standard qui permettra aux experts et membres de la société civile de quantifier la nature et la puissance des efforts pour le DD dans les pays, autant dans les sphères publiques que privées, à travers différentes dimensions de la société. En mesurant ces efforts, nous documenterons les intrants indépendamment des extrants, dont les revenus par habitant. Cela nous permettra d'apprécier la relation entre les intrants pris en compte dans l'élaboration des politiques de DD et les extrants du DD.

Cet IEDD est guidé par une question centrale : Dans quelle mesure les efforts politiques et programmatiques existants ont-ils permis un environnement favorable à la culture, la réalisation et l'exploitation des bénéfices d'un dividende démographique ? Pour répondre à cette question, l'IEDD résumera les perceptions d'informateurs clés dans six secteurs identifiés dans la littérature scientifique comme produisant un environnement favorable à l'exploitation du DD. Ces secteurs cadres du DD sont : 1) Planification familiale, 2) Santé maternelle et infantile, 3) Education, 4) Autonomisation des femmes, 5) Marché du travail, et 6) Bonne gouvernance, solidité institutionnelle et stabilité économique. Chaque secteur est mesuré via un questionnaire qui lui est propre et est évalué à travers cinq domaines identifiés dans la littérature scientifique, à savoir : la politique/ prise de décision, les services ou programmes, le plaidoyer, la recherche, et la société civile. Chacun des 6 questionnaires, remplis par 10 observateurs experts par pays, notera chaque élément sur une échelle de 1 à 10, sauf indication contraire.

Étant donné l'impact sanitaire et socioéconomique de la pandémie de COVID-19, et sa relation étroite avec les politiques qui permettent un environnement politique favorable au DD, cet Indice des efforts de DD intègre un module pour évaluer la résilience et la durabilité des systèmes dans chaque secteur. La notation suit les principes clés des systèmes de santé résilients et durables pouvant répondre efficacement aux menaces de maladies infectieuses émergentes et autres crises de santé publiques.

L'IEDD permet de visualiser les efforts nationaux, tout en évaluant la mesure dans laquelle ces efforts soutiennent les objectifs du pays pour cultiver, réaliser et exploiter le dividende démographique. L'IEDD servira d'outil pour mesurer la performance et suivre le progrès, ce qui offrira une évaluation opportune des succès et des marges de progression, des meilleures pratiques et des améliorations possibles.

JOHNS HOPKINS
BLOOMBERG SCHOOL
of PUBLIC HEALTH

Bill & Melinda Gates Institute for Population and Reproductive Health

Enquête de l’Indice des efforts pour le dividende démographique

Formulaire de consentement

Merci d’avoir pris le temps de répondre à ce questionnaire, particulièrement en cette période sans précédent.

L’Institut Gates développe actuellement un Indice des efforts pour le dividende démographique (IEDD) afin d’établir une mesure standard qui permettra d’évaluer le statut existant des politiques, services et programmes nationaux de planification familiale, santé maternelle et infantile, et dans d’autres secteurs sociaux (ex : emploi et éducation), ainsi qu’en matière de gouvernance pour permettre un environnement politique favorable au dividende démographique (DD). En mesurant ces efforts, cet outil documentera les intrants indépendamment des extrants, dont les revenus par habitant. Ceci permettra d’évaluer la relation entre les intrants pris en compte dans l’élaboration des politiques de DD et les extrants du DD observés et communément utilisés pour évaluer le progrès.

Nous aimerions vous poser quelques questions en raison de votre expertise dans ce domaine. Les informations collectées seront confidentielles, et nous ne collecterons ni votre nom, ni aucun autre détail permettant de vous identifier. Ce questionnaire devrait vous prendre environ 45-60 min.

Nous vous serions très reconnaissants si vous pouviez participer à cette enquête. Les informations générées par cette interview seront utilisées par l’Institut Gates et la communauté de pratique sur le DD dans votre pays pour évaluer les efforts nationaux, identifier les domaines de réussites qui doivent continuer d’être soutenus et les domaines nécessitant d’être renforcés. Les chercheurs pourront aussi utiliser les données collectées à des fins d’analyse comparative entre les pays.

Vous êtes invité(e) à participer à cette enquête. Sachez que la participation à cette enquête est entièrement volontaire. En participant, vous ne percevrez aucun bénéfice direct, mais il y a des bénéfices sociétaux à permettre aux gouvernements et aux partenaires du développement de renforcer leurs efforts pour cultiver un environnement favorable au DD.

Vous pouvez refuser de répondre à toute question. Si vous avez des inquiétudes ou problèmes concernant cette étude, vous pouvez contacter son coordinateur à **jcrusatira@jhu.edu** ou au **+14436510824** par téléphone ou WhatsApp.

* Veuillez cocher “Oui” si vous souhaitez procéder et répondre à l’enquête.

- ○ Oui
- ○ Non

JOHNS HOPKINS
BLOOMBERG SCHOOL
of PUBLIC HEALTH

Bill & Melinda Gates Institute for Population and Reproductive Health

Enquête de l'Indice des efforts pour le dividende démographique

Avis important

Veuillez ne pas sélectionner "Page précédente" au milieu d'une page de questions car la page sur laquelle vous travaillez ne sera pas sauvegardée tant que vous n'aurez pas finalisé ladite page et sélectionner "Page suivante". Si vous sélectionnez "Page précédente" sans avoir terminé, vos réponses sur la page inachevée seront perdus.

Sachez que votre enquête est terminée lorsque vous lisez "Merci d'avoir rempli l'indice d'effort du dividende démographique". Merci !

JOHNS HOPKINS
BLOOMBERG SCHOOL
of PUBLIC HEALTH

Bill & Melinda Gates Institute for Population and Reproductive Health

Enquête de l’Indice des efforts pour le dividende démographique

Pays et expertise de l’enquêté(e)

* Veuillez sélectionner le pays duquel vous répondez.

- ◯ Kenya
- ◯ Senegal
- ◯ Nigeria
- ◯ Rwanda
- ◯ Tanzania
- ◯ Ethiopia

JOHNS HOPKINS
BLOOMBERG SCHOOL
of PUBLIC HEALTH

Bill & Melinda Gates Institute for Population and Reproductive Health

Enquête de l'Indice des efforts pour le dividende démographique

Level of expertise

* At which level of Government is your expertise (If your expertise is at the national level please select "National" otherwise select a county?

JOHNS HOPKINS
BLOOMBERG SCHOOL
of PUBLIC HEALTH

Bill & Melinda Gates Institute for Population and Reproductive Health

Enquête de l'Indice des efforts pour le dividende démographique

Niveau d'expertise

* A quel niveau de gouvernement votre expertise se situe-t-elle (Si votre expertise se situe au niveau national, veuillez sélectionner "National", sinon sélectionnez une région ?

JOHNS HOPKINS
BLOOMBERG SCHOOL
of PUBLIC HEALTH

Bill & Melinda Gates Institute for Population and Reproductive Health

Enquête de l'Indice des efforts pour le dividende démographique

Expertise

* Veuillez sélectionner le type d'organisation dans laquelle vous avez le plus travaillé au cours des cinq dernières années. Si votre type d'organisation n'est pas référencé, veuillez préciser de quel type d'organisation il s'agit.

- Secteur public
- Secteur privé
- Organisation non gouvernementale
- Université/ Institut de recherche
- Autre (veuillez préciser)
- Other (please specify)

* Dans quel domaine avez-vous le plus travaillé au cours des cinq dernières années ?

- Politique/ Prise de décision
- Services ou programmes
- Plaidoyer
- Recherche
- Société Civile

* Dans quel secteur avez-vous le plus travaillé dans les cinq dernières années ?

- Planification familiale
- Santé maternelle et infantile
- Education
- Autonomisation des femmes
- Marché du travail
- Gouvernance et institutions économiques

JOHNS HOPKINS
BLOOMBERG SCHOOL of PUBLIC HEALTH

Bill & Melinda Gates Institute for Population and Reproductive Health

Enquête de l'Indice des efforts pour le dividende démographique

Questionnaire sur la planification familiale (PF)

Répondez aux questions suivantes en fonction de votre expérience/ expertise dans ce secteur spécifique.

Afin d'obtenir une représentation synthétique des efforts nationaux pour le dividende démographique, notez les éléments suivants sur une échelle de 1 à 10, 1 étant le score le plus faible (efforts très faibles ou quasi-inexistants) et 10 le plus élevé (efforts robustes). Le cas échéant, si une politique ou activité n'existe pas, répondez 0.

Donnez un score à chaque élément. Toutes les réponses seront enregistrées au format illustré ci-dessous :

Effort le plus faible	1	2	3	4	5	6	7	8	9	10	Effort le plus élevé	Je ne sais pas

JOHNS HOPKINS
BLOOMBERG SCHOOL
of PUBLIC HEALTH

Bill & Melinda Gates Institute for Population and Reproductive Health

Enquête de l'Indice des efforts pour le dividende démographique

Domaine 1. Politique/ Prise de décision

* FP 1.1 La proéminence de la planification familiale (PF) : Mesure dans laquelle il existe un bureau ou poste permanent et bien placé au ministère compétent qui développe et surveille les politiques et stratégies du secteur, et dispose d'effectifs adéquats et de personnel qualifié.

	1	2	3	4	5	6	7	8	9	10	Je ne sais pas
1-Plus faible -> Plus élevé-10	◯	◯	◯	◯	◯	◯	◯	◯	◯	◯	◯

* FP 1.2 La politique de PF et réduction de la fécondité : Mesure dans laquelle la politique gouvernementale met l'accent sur la PF pour promouvoir la taille des familles en lien avec les ressources disponibles ou des raisons démographiques plutôt que des raisons sanitaires.

	1	2	3	4	5	6	7	8	9	10	Je ne sais pas
1-Plus faible -> Plus élevé-10	◯	◯	◯	◯	◯	◯	◯	◯	◯	◯	◯

* FP 1.3 Le plan national stratégique de PF : Mesure dans laquelle il existe un plan national actualisé, exhaustif et stratégique pour la PF, définissant les politiques, lois et régulations pour l'offre de PF. (Évaluez la mesure dans laquelle le plan a une vision, comprend des stratégies sur plusieurs années, des calendriers annuels, et des plans de mise en œuvre, tout en étant fondés sur des données probantes, avec des indicateurs de progrès, et tenant compte du marché de la PF).

	1	2	3	4	5	6	7	8	9	10	Je ne sais pas
1-Plus faible -> Plus élevé-10	◯	◯	◯	◯	◯	◯	◯	◯	◯	◯	◯

* FP 1.4 La politique sur l’âge légal du mariage : Mesure dans laquelle l’âge légal du mariage pour les filles, établi à 18 ans ou plus, est appliqué.

	1	2	3	4	5	6	7	8	9	10	Je ne sais pas
1-Plus faible -> Plus élevé-10	○	○	○	○	○	○	○	○	○	○	○

* FP 1.5 La prise de décision fondée sur des données probantes : Mesure dans laquelle le ministère compétent soutient et utilise activement et régulièrement les données clés et rapports de PF pour élaborer ses politiques et ses plans.

	1	2	3	4	5	6	7	8	9	10	Je ne sais pas
1-Plus faible -> Plus élevé-10	○	○	○	○	○	○	○	○	○	○	○

* FP 1.6 Le suivi et évaluation des politiques/plans/stratégies nationaux : Mesure dans laquelle le plan national de PF établit une stratégie de suivi et évaluation complète pour suivre et mesurer les accomplissements prévus dans le plan. (Évaluez si la stratégie de suivi et évaluation inclut des buts et objectifs, un cadre de résultats, des indicateurs mesurables [ex : “SMART”: spécifiques, mesurables, atteignables, réalistes et temporellement définis], et un plan/calendrier exploitable de mise en œuvre.)

	1	2	3	4	5	6	7	8	9	10	Je ne sais pas
1-Plus faible -> Plus élevé-10	○	○	○	○	○	○	○	○	○	○	○

* FP 1.7 L’inclusion des acteurs non gouvernementaux et intersectoriels au développement de politique/stratégie : Mesure dans laquelle les agents non-gouvernementaux, les associations (ex : de la société civile), les travailleurs de la santé, les parties prenantes (ex : organisations confessionnelles, à but non lucratif et à but lucratif) et les acteurs d’autres secteurs que celui de la PF sont inclus dans le plan national de PF et/ou autres documents nationaux établissant les stratégies et systèmes du pays pour planifier, gérer et développer la PF.

	1	2	3	4	5	6	7	8	9	10	Je ne sais pas
1-Plus faible -> Plus élevé-10	○	○	○	○	○	○	○	○	○	○	○

* FP 1.8 Le financement de la PF : Mesure dans laquelle le gouvernement a établi et appliqué des directives pour la budgétisation et le paiement de la PF en termes d'offre et de demande (soit les financements intérieurs vs. internationaux, et leur continuité/durabilité, coût des produits contraceptifs, coût de la prestation de services et du transport, etc.).

(Évaluez si les directives et le budget tiennent compte de la durée de vie des coûts des soins et services de PF ; des tarifications variables par méthode de PF ; des coûts de livraison et prestation de services par spécialité, lieu et risque ; des coûts et profits des plans/politiques d'assurance ; du coût financier de fournir de la PF en comparaison au coût financier de ne pas le faire, etc.).

	1	2	3	4	5	6	7	8	9	10	Je ne sais pas
1-Plus faible -> Plus élevé-10	○	○	○	○	○	○	○	○	○	○	○

* FP 1.9 Le pourcentage du financement intérieur (domestique) pour le budget de la PF : Mesure dans laquelle le budget total de la planification familiale/population est dérivé de sources domestiques (ex : 1 pour 10%, 5 pour 50%, 10 pour 100%).

	1	2	3	4	5	6	7	8	9	10	Je ne sais pas
1-Plus faible -> Plus élevé-10	○	○	○	○	○	○	○	○	○	○	○

* FP 1.10 L'infrastructure/ capacité des Technologies de l'Information et la Communication (TIC) : Mesure dans laquelle il existe des éléments d'infrastructure (ex : ordinateurs, serveurs, imprimantes, réseau local, connexion internet, alimentation électrique fiable) ainsi que des ressources humaines (ex : pour la maintenance et le soutien) au niveau national et infranational permettant de soutenir des systèmes d'informations fonctionnels sur la PF.

(Pour cet élément, évaluez si le pays conduit au moins des évaluations annuelles de ses systèmes informatiques ; a des programmes de formation couvrant la saisie et l'utilisation des données, l'administration et le développement de logiciels ; et forme continuellement le personnel des institutions clés [ex : Ministère de la Santé, Ministère de l'Éducation, les agences régulatrices et les institutions de formation]).

	1	2	3	4	5	6	7	8	9	10	Je ne sais pas
1-Plus faible -> Plus élevé-10	○	○	○	○	○	○	○	○	○	○	○

* FP 1.11 Les lois et régulations sur l’importation :

	1	2	3	4	5	6	7	8	9	10	Je ne sais pas
FP 1.11.1 Mesure dans laquelle les lois et régulations sur l’importation facilitent l’importation des commodités et produits de PF.	◯	◯	◯	◯	◯	◯	◯	◯	◯	◯	◯
FP 1.11.2 Mesure dans laquelle les contraceptifs sont fabriqués localement.	◯	◯	◯	◯	◯	◯	◯	◯	◯	◯	◯

* FP 1.12 La publicité des contraceptifs autorisés : Mesure dans laquelle la publicité des contraceptifs dans les médias de masse n’est soumise à aucune restriction.

	1	2	3	4	5	6	7	8	9	10	Je ne sais pas
1-Plus faible -> Plus élevé-10	◯	◯	◯	◯	◯	◯	◯	◯	◯	◯	◯

* FP 1.13 La présence de politiques de PF favorisant les populations pauvres : Mesure dans laquelle les politiques ciblent spécifiquement les populations pauvres en milieu rural et urbain.

	1	2	3	4	5	6	7	8	9	10	Je ne sais pas
1-Plus faible -> Plus élevé-10	◯	◯	◯	◯	◯	◯	◯	◯	◯	◯	◯

* FP 1.14 Le soutien politique à la PF : Mesure dans laquelle les élus dans le pays priorisent les besoins de PF pour renforcer les contributions de la PF au DD en adoptant des lois et régulations, et en appuyant des actions et politiques visant à améliorer l’utilisation et l’adhérence à la PF.

	1	2	3	4	5	6	7	8	9	10	Je ne sais pas
1-Plus faible -> Plus élevé-10	◯	◯	◯	◯	◯	◯	◯	◯	◯	◯	◯

FP 1.15 Commentaires : Veuillez utiliser cette section pour tout commentaire additionnel sur le niveau d’effort, les difficultés et les succès rencontrés dans le pays autour des politiques/ prise de décision pour la PF. Si vous avez répondu « Je ne sais pas » à l’une des questions, utilisez aussi cette section pour expliquer pourquoi.

JOHNS HOPKINS
BLOOMBERG SCHOOL
of PUBLIC HEALTH

Bill & Melinda Gates Institute for Population and Reproductive Health

Enquête de l’Indice des efforts pour le dividende démographique

Domaine 2. Services ou programmes

* FP 2.1 La structure administrative : Mesure dans laquelle la structure administrative et le personnel au niveau national, provincial et des comtés est suffisant pour mettre en œuvre le programme de planification familiale (PF).

	1	2	3	4	5	6	7	8	9	10	Je ne sais pas
1-Plus faible -> Plus élevé-10											

* FP 2.2 Le niveau des responsables du programme : Niveau d’ancienneté relatif du directeur du programme national de planification familiale et mesure dans laquelle le supérieur hiérarchique direct de ce directeur est haut placé au gouvernement.

	1	2	3	4	5	6	7	8	9	10	Je ne sais pas
1-Plus faible -> Plus élevé-10											

* FP. 2.3 La distribution à base communautaire : Mesure dans laquelle les zones du pays non desservies par les cliniques et autres sites de prestation de santé sont couvertes par des programmes de distribution à base communautaire de produits contraceptifs (particulièrement les zones rurales).

	1	2	3	4	5	6	7	8	9	10	Je ne sais pas
1-Plus faible -> Plus élevé-10											

* FP 2.4 Le programme de PF postpartum (PFPP) : Mesure dans laquelle toutes les femmes en postpartum reçoivent des services PFPP.

	1	2	3	4	5	6	7	8	9	10	Je ne sais pas
1-Plus faible -> Plus élevé-10											

* FP 2.5 Les agents travaillant à domicile : Mesure de la couverture de la population par les agents dont la tâche principale est de rendre visite aux femmes (en milieu rural) chez elles pour discuter de planification familiale et de santé maternelle et infantile.

	1	2	3	4	5	6	7	8	9	10	Je ne sais pas
1-Plus faible -> Plus élevé-10	◯	◯	◯	◯	◯	◯	◯	◯	◯	◯	◯

* FP 2.6 La disponibilité et l'accessibilité des commodités/produits et services de PF (première partie) : Mesure dans laquelle l'ensemble de la population a facilement et directement accès à (1-le plus faible -> le plus élevé-10):

	1	2	3	4	5	6	7	8	9	10	Je ne sais pas
FP 2.6.1 DIU	◯	◯	◯	◯	◯	◯	◯	◯	◯	◯	◯
FP 2.6.2 Pilule	◯	◯	◯	◯	◯	◯	◯	◯	◯	◯	◯
FP 2.6.3 Injectable	◯	◯	◯	◯	◯	◯	◯	◯	◯	◯	◯
FP 2.6.4 Implant	◯	◯	◯	◯	◯	◯	◯	◯	◯	◯	◯
FP 2.6.5 Stérilisation féminine	◯	◯	◯	◯	◯	◯	◯	◯	◯	◯	◯
FP 2.6.6 Stérilisation masculine	◯	◯	◯	◯	◯	◯	◯	◯	◯	◯	◯
FP 2.6.7 Préservatif masculin	◯	◯	◯	◯	◯	◯	◯	◯	◯	◯	◯
FP 2.6.8 Contraception d'urgence	◯	◯	◯	◯	◯	◯	◯	◯	◯	◯	◯
FP 2.6.9 Avortement/ régulation de la menstruation (légal ou non)	◯	◯	◯	◯	◯	◯	◯	◯	◯	◯	◯
FP 2.6.10 Autres (ex : préservatif féminin, mousse, collier du cycle, etc.)	◯	◯	◯	◯	◯	◯	◯	◯	◯	◯	◯

* FP 2.7 La disponibilité et l'accessibilité des commodités/produits et services de PF, (deuxième partie) : Quel est le niveau de fonctionnalité du système d'approvisionnement (évite t-il les ruptures de stock ou l'interruption de l'approvisionnement, ou permet-il un flux fiable à tous les niveaux ?) pour les méthodes suivantes (1-le plus faible -> le plus élevé-10) :

	1	2	3	4	5	6	7	8	9	10	Don't know
FP 2.7.1 DIU	○	○	○	○	○	○	○	○	○	○	○
FP 2.7.2 Pilule	○	○	○	○	○	○	○	○	○	○	○
FP 2.7.3 Injectable	○	○	○	○	○	○	○	○	○	○	○
FP 2.7.4 Implant	○	○	○	○	○	○	○	○	○	○	○
FP 2.7.5 Préservatif masculin	○	○	○	○	○	○	○	○	○	○	○
FP 2.7.6 Contraception d'urgence	○	○	○	○	○	○	○	○	○	○	○
FP 2.7.7 Autres (ex : préservatif féminin, mousse, collier du cycle, etc.)	○	○	○	○	○	○	○	○	○	○	○

* FP 2.8 La disponibilité et l'accessibilité des commodités/produits et services de PF (troisième partie) : Dans quelle mesure le système d'approvisionnement fournit-il l'équipement et les produits médicaux nécessaires aux structures cliniques pour les méthodes suivantes (1-le plus faible -> le plus élevé-10) :

	1	2	3	4	5	6	7	8	9	10	Je ne sais pas
FP 2.8.1 Stérilisation féminine	○	○	○	○	○	○	○	○	○	○	○
FP 2.8.2 Stérilisation masculine	○	○	○	○	○	○	○	○	○	○	○
FP 2.8.3. DIU	○	○	○	○	○	○	○	○	○	○	○
FP 2.8.4 Implant	○	○	○	○	○	○	○	○	○	○	○

Other (please specify)

* FP 2.9 La qualité des services de PF : Veuillez noter la qualité générale des services de planification familiale. (Des services de haute qualité impliquent d'être orientés sur les besoins des clients, et de fournir du conseil, des informations complètes, un large choix de méthodes, et des procédures cliniques sûres).

	1	2	3	4	5	6	7	8	9	10	Je ne sais pas
1-Plus faible -> Plus élevé-10	○	○	○	○	○	○	○	○	○	○	○

* FP 2.10 Les avantages et les effets dissuasifs : Mesure dans laquelle des avantages monétaires ou d'autre type sont utilisés pour encourager l'adoption de la planification familiale.

	1	2	3	4	5	6	7	8	9	10	Je ne sais pas
1-Plus faible -> Plus élevé-10	◯	◯	◯	◯	◯	◯	◯	◯	◯	◯	◯

* FP 2.11 Les programmes de PF pour les adolescents : Mesure dans laquelle les informations, services et activités sont focalisés sur les adolescents et encouragent ces derniers à accéder aux services contraceptifs.

	1	2	3	4	5	6	7	8	9	10	Je ne sais pas
1-Plus faible -> Plus élevé-10	◯	◯	◯	◯	◯	◯	◯	◯	◯	◯	◯

* FP 2.12 Les programmes de formation : Mesure dans laquelle les programmes de formation, pour chaque catégorie de personnel dans le programme de planification familiale, sont adéquats pour transmettre au personnel les informations et compétences nécessaires à la conduite efficace de leur travail.

	1	2	3	4	5	6	7	8	9	10	Je ne sais pas
1-Plus faible -> Plus élevé-10	◯	◯	◯	◯	◯	◯	◯	◯	◯	◯	◯

* FP 2.13 Les tâches assignées remplies par le personnel : Mesure dans laquelle toutes les catégories du personnel du programme de planification familiale (administratif, médical, paramédical, de terrain) remplissent leurs tâches assignées efficacement.

	1	2	3	4	5	6	7	8	9	10	Je ne sais pas
1-Plus faible -> Plus élevé-10	◯	◯	◯	◯	◯	◯	◯	◯	◯	◯	◯

* FP 2.14 La logistique et le transport : Mesure dans laquelle les systèmes logistiques et de transport sont suffisants pour maintenir les stocks de produits contraceptifs et l'équipement disponible sur tous les sites de prestation de santé, à tout moment et à tous les niveaux.

	1	2	3	4	5	6	7	8	9	10	Je ne sais pas
1-Plus faible -> Plus élevé-10	◯	◯	◯	◯	◯	◯	◯	◯	◯	◯	◯

* FP 2.15 Le système de supervision : Mesure dans laquelle le système de supervision à tous les niveaux est adéquat (visite de supervision régulières, suivies par des actions correctives ou d'appui/accompagnantes).

	1	2	3	4	5	6	7	8	9	10	Je ne sais pas
1-Plus faible -> Plus élevé-10	◯	◯	◯	◯	◯	◯	◯	◯	◯	◯	◯

* FP 2.16 L'implication d'autres ministères et agences publiques : Mesure dans laquelle d'autres ministères, agences gouvernementales et parties prenantes non gouvernementales s'impliquent dans les activités de PF (ex : livraison de produits, prestation de services, information, éducation) ou autres activités pour la population.

	1	2	3	4	5	6	7	8	9	10	Je ne sais pas
1-Plus faible -> Plus élevé-10	◯	◯	◯	◯	◯	◯	◯	◯	◯	◯	◯

* FP 2.17 L'implication d'agences et groupes du secteur privé : Mesure dans laquelle des agences ou groupes du secteur privé contribuent aux activités de PF ou autres activités pour la population.

	1	2	3	4	5	6	7	8	9	10	Je ne sais pas
1-Plus faible -> Plus élevé-10	◯	◯	◯	◯	◯	◯	◯	◯	◯	◯	◯

* FP 2.18 Les médias de masse pour la communication, l'éducation et l'information : Fréquence et couverture des messages des médias de masse fournissant à la population des informations sur a planification familiale et les sites de prestation de services.

	1	2	3	4	5	6	7	8	9	10	Je ne sais pas
1-Plus faible -> Plus élevé-10	◯	◯	◯	◯	◯	◯	◯	◯	◯	◯	◯

* FP 2.19 Les barrières normatives, culturelles et sociales : Mesure dans laquelle les programmes de planification familiale répondent aux barrières culturelles et sociales.

	1	2	3	4	5	6	7	8	9	10	Je ne sais pas
1-Plus faible -> Plus élevé-10	◯	◯	◯	◯	◯	◯	◯	◯	◯	◯	◯

* FP 2.20 Les jeunes : Mesure dans laquelle le gouvernement soutient des initiatives de PF dirigées par les jeunes en matière de planification, mise en œuvre et évaluation des programmes de PF.

	1	2	3	4	5	6	7	8	9	10	Je ne sais pas
1-Plus faible -> Plus élevé-10	◯	◯	◯	◯	◯	◯	◯	◯	◯	◯	◯

* FP 2.21 Les influences des efforts de PF (les forces influençant le programme de PF) : Les forces influençant le programme de PF peuvent soit renforcer, soit diminuer son efficacité, Cochez zéro (0) s'il n'y a aucune différence ; cochez un nombre négatif de -1 à -5 si l'influence est négative ; ou cochez un nombre positif de 1 à 5 si l'influence est positive. (1 dans chaque direction signifie peu d'influence et 5 dans chaque direction signifie beaucoup d'influence).

	-5	-4	-3	-2	-1	0	1	2	3	4	5	Je ne sais pas
FP 2.21.1 Décentralisation	○	○	○	○	○	○	○	○	○	○	○	○
FP 2.21.2 Programmes VIH/SIDA	○	○	○	○	○	○	○	○	○	○	○	○
FP 2.21.3 Incorporation de la PF au contexte plus large de la santé reproductive (SR)	○	○	○	○	○	○	○	○	○	○	○	○
FP 2.21.4 Intégration de la PF à d'autres services de santé	○	○	○	○	○	○	○	○	○	○	○	○
FP 2.21.5 Changements dans les financements des bailleurs de fonds	○	○	○	○	○	○	○	○	○	○	○	○
FP 2.21.6 Changements dans les financements du gouvernement	○	○	○	○	○	○	○	○	○	○	○	○

FP 2.22 Commentaires : Veuillez utiliser cette section pour tout commentaire additionnel sur le niveau d'effort, les difficultés et les succès rencontrés dans le pays autour des services ou programmes de PF. Si vous avez répondu « Je ne sais pas » à l'une des questions, utilisez aussi cette section pour expliquer pourquoi.

JOHNS HOPKINS
BLOOMBERG SCHOOL
of PUBLIC HEALTH

Bill & Melinda Gates Institute for Population and Reproductive Health

Enquête de l'Indice des efforts pour le dividende démographique

Domaine 3. Plaidoyer

* FP 3.1 L'influence des dirigeants/ champions de PF : Mesure dans laquelle le pays compte sur un ou plusieurs dirigeants ou champions clairement influents plaidant avec succès au profit des besoins (sectoriels) de la PF auprès des décideurs, promouvant la PF dans le pays à travers des déclarations publiques positives, et/ou soutenant des actions et politiques visant à améliorer l'environnement de la PF et ses contributions au DD.

	1	2	3	4	5	6	7	8	9	10	Je ne sais pas
1-Plus faible -> Plus élevé-10	○	○	○	○	○	○	○	○	○	○	○

* FP 3.2 Les déclarations des dirigeants : Mesure dans laquelle le chef du gouvernement, ainsi que d'autres hauts fonctionnaires, parlent publiquement et favorablement de la planification familiale au moins une fois par an.

	1	2	3	4	5	6	7	8	9	10	Je ne sais pas
1-Plus faible -> Plus élevé-10	○	○	○	○	○	○	○	○	○	○	○

* FP 3.3 La couverture médiatique de la PF : Mesure dans laquelle et fréquence à laquelle la couverture médiatique nationale des sujets de PF apporte à la population des informations sur la planification familiale et les sites de prestation de services.

	1	2	3	4	5	6	7	8	9	10	Je ne sais pas
1-Plus faible -> Plus élevé-10	○	○	○	○	○	○	○	○	○	○	○

* FP 3.4 Les influences des efforts de PF (justification) : Quelle est l'importance de chacun des éléments suivants en tant que justification actuelle par le gouvernement de son programme national de PF ? (1 signifie une importance négligeable ; 10 signifie une grande importance.)

	1	2	3	4	5	6	7	8	9	10	Je ne sais pas
3.4.1 Réduire le taux de croissance démographique	◯	◯	◯	◯	◯	◯	◯	◯	◯	◯	◯
3.4.2 Stimuler le développement économique	◯	◯	◯	◯	◯	◯	◯	◯	◯	◯	◯
3.4.3 Aider les hommes et les femmes à éviter des naissances non planifiées et non désirées	◯	◯	◯	◯	◯	◯	◯	◯	◯	◯	◯
3.4.4 Améliorer la santé de la femme	◯	◯	◯	◯	◯	◯	◯	◯	◯	◯	◯
3.4.5 Améliorer la santé de l'enfant	◯	◯	◯	◯	◯	◯	◯	◯	◯	◯	◯
3.4.6 Réduire les grossesses chez les adolescentes non mariées	◯	◯	◯	◯	◯	◯	◯	◯	◯	◯	◯
3.4.7 Réduire les besoins contraceptifs non satisfaits	◯	◯	◯	◯	◯	◯	◯	◯	◯	◯	◯

* FP 3.5 Les influences des efforts de PF (les populations vulnérables) : Dans quelle mesure le programme de planification familiale met-il l'accent sur les populations vulnérables ou spécifiques ? (1 signifie un accent négligeable ; 10 signifie un accent important).

	1	2	3	4	5	6	7	8	9	10	Je ne sais pas
FP 3.5.1 Les jeunes non mariés	○	○	○	○	○	○	○	○	○	○	○
FP 3.5.2 Les populations vivant dans la pauvreté	○	○	○	○	○	○	○	○	○	○	○
FP 3.5.3 Les personnes en situation de handicap	○	○	○	○	○	○	○	○	○	○	○
FP 3.5.4 Les populations rurales	○	○	○	○	○	○	○	○	○	○	○
FP 3.5.5 Autres groupes vulnérables (ex : les minorités et/ou les groupes indigènes)	○	○	○	○	○	○	○	○	○	○	○
FP 3.5.6 Les femmes en postpartum pour le conseil et les services de contraception	○	○	○	○	○	○	○	○	○	○	○
FP 3.5.7 Les femmes en post-avortement pour le conseil et les services de contraception	○	○	○	○	○	○	○	○	○	○	○

* FP 3.6 Le marketing social : Mesure de la couverture du programme de marketing social du pays (ventes des contraceptifs subventionnés à faible coût dans le secteur commercial, particulièrement en milieu urbain).

	1	2	3	4	5	6	7	8	9	10	Je ne sais pas
1-Plus faible -> Plus élevé-10	○	○	○	○	○	○	○	○	○	○	○

FP 3.7 Commentaires : Veuillez utiliser cette section pour tout commentaire additionnel sur le niveau d'effort, les difficultés et les succès rencontrés dans le pays autour du plaidoyer pour la PF. Si vous avez répondu « Je ne sais pas » à l'une des questions, utilisez aussi cette section pour expliquer pourquoi.

JOHNS HOPKINS
BLOOMBERG SCHOOL
of PUBLIC HEALTH

Bill & Melinda Gates Institute for Population and Reproductive Health

Enquête de l'Indice des efforts pour le dividende démographique

Domaine 4. Recherche

* FP 4.1 La force des observatoires/parties prenantes/groupes de travail pour la PF : Mesure dans laquelle les parties prenantes du secteur de la planification familiale du pays (ex : groupe directif de parties prenantes, groupes de travail techniques, observatoire de la PF, groupe de coordination ou facilitation) :

	1	2	3	4	5	6	7	8	9	10	Je ne sais pas
FP 4.1.1 Ont un représentant du gouvernement, des institutions de formation, de la société civile, des organisations non gouvernementales et confessionnelles, des associations professionnelles, du secteur privé, etc.	○	○	○	○	○	○	○	○	○	○	○
FP 4.1.2 Se réunissent régulièrement, émettent des rapports et recommandent des politiques aux directions des ministères compétents.	○	○	○	○	○	○	○	○	○	○	○
FP 4.1.3 Ont un impact sur la PF dans le pays.	○	○	○	○	○	○	○	○	○	○	○

* FP 4.2 La stratégie de recherche en PF : Mesure dans laquelle le plan national de PF et/ou d'autres documents nationaux comprennent une stratégie/approche complète de recherche sur la planification familiale.

	1	2	3	4	5	6	7	8	9	10	Je ne sais pas
1-Plus faible -> Plus élevé-10	○	○	○	○	○	○	○	○	○	○	○

* FP 4.3 Les partenaires collectant les données :

	1	2	3	4	5	6	7	8	9	10	Je ne sais pas
FP 4.3.1 Mesure dans laquelle la collecte des données est entreprise par des agences gouvernementales. (Cela peut comprendre les agences/bureaux de la statistique et/ou les ministères de l'économie/finances, éducation, santé, genre, etc.)	◯	◯	◯	◯	◯	◯	◯	◯	◯	◯	◯
FP 4.3.2 Mesure dans laquelle la collecte de données est entreprise par des institutions de recherche.	◯	◯	◯	◯	◯	◯	◯	◯	◯	◯	◯
FP 4.3.3 Mesure dans laquelle la collecte des données est entreprise par des chercheurs indépendants.	◯	◯	◯	◯	◯	◯	◯	◯	◯	◯	◯

* FP 4.4 La recherche thématique :

	1	2	3	4	5	6	7	8	9	10	Je ne sais pas
FP 4.4.1 Mesure dans laquelle la recherche est menée sur l'adoption de la planification familiale (dont la distribution des méthodes), la demande et les besoins non satisfaits.	◯	◯	◯	◯	◯	◯	◯	◯	◯	◯	◯
FP 4.4.2 Mesure dans laquelle de la recherche est menée sur les barrières à l'adoption de la PF	◯	◯	◯	◯	◯	◯	◯	◯	◯	◯	◯
FP 4.4.3 Mesure dans laquelle de la recherche est menée sur les programmes de PF	◯	◯	◯	◯	◯	◯	◯	◯	◯	◯	◯
FP 4.4.4 Mesure dans laquelle de la recherche est menée sur la qualité de la prestation des services de PF	◯	◯	◯	◯	◯	◯	◯	◯	◯	◯	◯

* FP 4.5 La qualité/couverture des données : Mesure dans laquelle la recherche/les données actuelles sont désagrégées par sexe et par âge. Mesure dans laquelle le pays participe aux tests standardisés et la place qu'il occupe au classement.

	1	2	3	4	5	6	7	8	9	10	Je ne sais pas
FP 4.5.1 Mesure dans laquelle un système statistique de routine offre de bonnes informations périodiques sur les produits et les établissements de PF	○	○	○	○	○	○	○	○	○	○	○
FP 4.5.2 Mesure dans laquelle un système statistique de routine offre de bonnes informations régulières sur les services et le personnel du secteur de la PF	○	○	○	○	○	○	○	○	○	○	○
FP 4.5.3 Mesure dans laquelle un système statistique de routine offre de bonnes informations périodiques sur les besoins de PF des populations de différentes communautés	○	○	○	○	○	○	○	○	○	○	○
FP 4.5.4 Mesure dans laquelle des évaluations du système de suivi des informations sanitaires sont menées et appliquées pour assurer la fiabilité des données	○	○	○	○	○	○	○	○	○	○	○

* FP 4.6 Les registres : Mesure dans laquelle les systèmes de registres des clients, rapports des cliniques et retours/commentaires sur les résultats sont adéquats et connectés à tous les niveaux (fédéral, état/province, comté, etc.).

	1	2	3	4	5	6	7	8	9	10	Je ne sais pas
1-Plus faible -> Plus élevé-10	○	○	○	○	○	○	○	○	○	○	○

* FP 4.7 La qualité des institutions de recherche : Mesure dans laquelle le pays a la capacité de soutenir et maintenir des institutions de recherche qui développent des études permettant de soutenir la portée et la qualité de la collecte de données pour informer les efforts nationaux de PF.

	1	2	3	4	5	6	7	8	9	10	Je ne sais pas
1-Plus faible -> Plus élevé-10	◯	◯	◯	◯	◯	◯	◯	◯	◯	◯	◯

* FP 4.8 L'évaluation : Mesure dans laquelle les statistiques, enquêtes et études sur le programme de PF sont utilisées par du personnel spécialisé pour rapporter les opérations du programme et mesurer le progrès.

	1	2	3	4	5	6	7	8	9	10	Je ne sais pas
1-Plus faible -> Plus élevé-10	◯	◯	◯	◯	◯	◯	◯	◯	◯	◯	◯

* FP 4.9 L'utilisation des résultats d'évaluation par les gestionnaires de programmes : Mesure dans laquelle les gestionnaires locaux du programme de PF utilisent les conclusions de recherche et d'évaluations pour améliorer ces programmes en suivant les recommandations émises.

	1	2	3	4	5	6	7	8	9	10	Je ne sais pas
1-Plus faible -> Plus élevé-10	◯	◯	◯	◯	◯	◯	◯	◯	◯	◯	◯

* FP 4.10 L'utilisation des résultats d'évaluation par les ministères : Mesure dans laquelle les administrateurs ministériels compétents utilisent systématiquement les données pour informer les politiques et interventions visant à résoudre les problèmes liés à la PF.

	1	2	3	4	5	6	7	8	9	10	Je ne sais pas
1-Plus faible -> Plus élevé-10	◯	◯	◯	◯	◯	◯	◯	◯	◯	◯	◯

* FP 4.11 La dissémination d'informations à d'autres entités chargées de la mise en œuvre des programmes : Mesure dans laquelle des informations sont partagées ou disséminées entre géographies et à différents niveaux (national, état/province/comté, localités/sous-comtés).

	1	2	3	4	5	6	7	8	9	10	Je ne sais pas
1-Plus faible -> Plus élevé-10	◯	◯	◯	◯	◯	◯	◯	◯	◯	◯	◯

FP 4.12 Commentaires : Veuillez utiliser cette section pour tout commentaire additionnel sur le niveau d'effort, les difficultés et les succès rencontrés dans le pays autour de la recherche sur la PF. Si vous avez répondu « Je ne sais pas » à l'une des questions, utilisez aussi cette section pour expliquer pourquoi.

JOHNS HOPKINS
BLOOMBERG SCHOOL
of PUBLIC HEALTH

Bill & Melinda Gates Institute for Population and Reproductive Health

Enquête de l’Indice des efforts pour le dividende démographique

Domaine 5. Organisations de la société civile (OSC)

* FP 5.1 Le pouvoir des acteurs des OSC : Mesure dans laquelle les acteurs des OSC occupent des postes d’influence dans les institutions, les groupes de travail techniques ou les réseaux concernés par la PF.

	1	2	3	4	5	6	7	8	9	10	Je ne sais pas
1-Plus faible -> Plus élevé-10	○	○	○	○	○	○	○	○	○	○	○

* FP 5.2 L’analyse budgétaire comme outil des OSC : Mesure dans laquelle les OSC utilisent l’analyse budgétaire comme un outil pour développer le plaidoyer en matière de PF.

	1	2	3	4	5	6	7	8	9	10	Je ne sais pas
1-Plus faible -> Plus élevé-10	○	○	○	○	○	○	○	○	○	○	○

* FP 5.3 Le soutien aux services offerts en structure sanitaire : Mesure dans laquelle les OSC sont impliquées dans les efforts visant à augmenter l’utilisation et/ou la qualité des services, particulièrement à travers une plus grande capacité technique et/ou opérationnelle des cliniques travaillant en partenariat avec des CSO pour fournir des services de PF.

	1	2	3	4	5	6	7	8	9	10	Je ne sais pas
1-Plus faible -> Plus élevé-10	○	○	○	○	○	○	○	○	○	○	○

* FP 5.4 Le soutien aux services à base communautaire : Mesure dans laquelle les OSC participent au rapprochement des informations et services de PF avec les communautés à travers des activités telles que le renforcement de compétences des agents de santé communautaire (ASC), l'expansion de la gamme de méthodes contraceptives fournies par les ASC, la formation des cadres inférieurs de professionnels de la santé et/ou les campagnes d'information et d'éducation pour augmenter l'acceptabilité de la contraception.

	1	2	3	4	5	6	7	8	9	10	Je ne sais pas
1-Plus faible -> Plus élevé-10	◯	◯	◯	◯	◯	◯	◯	◯	◯	◯	◯

* FP 5.5 Le soutien aux services à base communautaire à travers des campagnes mobiles de sensibilisation : Mesure dans laquelle les OSC soutiennent les modèles de services en dehors de cliniques pour que les prestataires de santé aient les commodités et produits de PF, l'équipement et les véhicules nécessaires pour être envoyés ensuite comme équipe spécialiste et fournir des informations et méthodes de PF directement aux communautés.

	1	2	3	4	5	6	7	8	9	10	Je ne sais pas
1-Plus faible -> Plus élevé-10	◯	◯	◯	◯	◯	◯	◯	◯	◯	◯	◯

* FP 5.6 Mesure dans laquelle les OSC sont impliquées dans la franchise sociale des services de PF de qualité aux client(e)s à moindre coût.

	1	2	3	4	5	6	7	8	9	10	Je ne sais pas
1-Plus faible -> Plus élevé-10	◯	◯	◯	◯	◯	◯	◯	◯	◯	◯	◯

* FP 5.7 La santé mobile : Mesure dans laquelle les OSC mettent à profit la technologie mobile, comme l'utilisation de la messagerie téléphonique (SMS), pour améliorer l'accès aux informations et services de PF.

	1	2	3	4	5	6	7	8	9	10	Je ne sais pas
1-Plus faible -> Plus élevé-10	◯	◯	◯	◯	◯	◯	◯	◯	◯	◯	◯

* FP 5.8 Les droits humains, l'accès et la qualité de l'éducation : Mesure dans laquelle les OSC plaident pour des approches basées sur les droits et veillent à ce que les bénéficiaires des services de PF soit traités correctement, n'aient pas peur de poser des questions, et reçoivent des conseils de qualité, entre autres sujets d'importance.

	1	2	3	4	5	6	7	8	9	10	Je ne sais pas
1-Plus faible -> Plus élevé-10	◯	◯	◯	◯	◯	◯	◯	◯	◯	◯	◯

* FP 5.9 Le changement social et des comportements (CSC) : Mesure dans laquelle les OSC soutiennent les interventions pour le CSC comprenant, mais sans s'y limiter, la promotion des compétences de vie et de l'éducation entre pairs, l'éducation sur la santé de l'adulte, l'éducation en milieu scolaire, la communication au sein du couple, la mobilisation communautaire, les interventions à travers les gardiens de la communauté et/ou les interventions médiatiques destinées au grand public. Ces interventions peuvent être mises en œuvre au niveau communautaire ou individuel, et avoir pour résultat une plus grande utilisation de la PF et des changements dans les connaissances et attitudes liées à la PF.

	1	2	3	4	5	6	7	8	9	10	Je ne sais pas
1-Plus faible -> Plus élevé-10	○	○	○	○	○	○	○	○	○	○	○

* FP 5.10 Les jeunes : Mesure dans laquelle les OSC soutiennent les politiques, interventions et programmes de PF ciblant les jeunes spécifiquement.

	1	2	3	4	5	6	7	8	9	10	Je ne sais pas
1-Plus faible -> Plus élevé-10	○	○	○	○	○	○	○	○	○	○	○

* FP 5.11 Les programmes pour les hommes : Mesure dans laquelle les OSC soutiennent des politiques, interventions et programmes de PF pour les hommes.

	1	2	3	4	5	6	7	8	9	10	Je ne sais pas
1-Plus faible -> Plus élevé-10	○	○	○	○	○	○	○	○	○	○	○

* FP 5.12 Le plaidoyer et la redevabilité : Mesure dans laquelle les OSC soutiennent la formation et le renforcement de compétences des plaideurs et des membres de la communauté pour améliorer leur compréhension des politiques et des processus d'identification de problèmes, de résolution collaborative de ces derniers, et des actions de plaidoyer plus ciblées pour améliorer les programmes de planification familiale.

	1	2	3	4	5	6	7	8	9	10	Je ne sais pas
1-Plus faible -> Plus élevé-10	○	○	○	○	○	○	○	○	○	○	○

* FP 5.13 L'évaluation et le suivi dirigés par les OSC : Mesure dans laquelle les OSC évaluent, suivent et émettent des rapports sur l'efficacité des politiques et programmes pour améliorer la redevabilité des prestataires de santé et des politiques de PF.

	1	2	3	4	5	6	7	8	9	10	Je ne sais pas
1-Plus faible -> Plus élevé-10	○	○	○	○	○	○	○	○	○	○	○

* FP 5.14 Les partenariats entre OSC : Mesure dans laquelle les OSC ont formé des alliances nationales et des partenariats régionaux pour renforcer leur position et potentiellement améliorer le leadership et les mécanismes de financement.

	1	2	3	4	5	6	7	8	9	10	Je ne sais pas
1-Plus faible -> Plus élevé-10	○	○	○	○	○	○	○	○	○	○	○

FP 5.15 Commentaires : Veuillez utiliser cette section pour tout commentaire additionnel sur le niveau d'effort, les difficultés et les succès rencontrés dans le pays autour des organisations de la société civile (OSC) pour la PF. Si vous avez répondu « Je ne sais pas » à l'une des questions, utilisez aussi cette section pour expliquer pourquoi.

JOHNS HOPKINS
BLOOMBERG SCHOOL
of PUBLIC HEALTH

Bill & Melinda Gates Institute for Population and Reproductive Health

Enquête de l'Indice des efforts pour le dividende démographique

Module : Résilience et durabilité du secteur

Étant donné l'impact sanitaire et socioéconomique de la pandémie de COVID-19, et son impact probable sur la progression vers le DD, cet indice des efforts pour le DD intègre des questions pour évaluer la résilience et la durabilité des systèmes dans les secteurs clés du DD. Les éléments couverts par les questions liées à la pandémie de COVID-19 ci-dessous relèvent des dimensions clés des systèmes résilients et visent à apporter des informations déterminantes sur le potentiel d'une réponse efficace aux menaces de maladies infectieuses émergentes et autres crises de santé publique.

Veuillez noter chaque élément sur une échelle de 1 à 10, 1 étant le score le plus faible (faible état/capacité) et 10 le plus élevé (très bon état/capacité).

* Dimension physique : structures, équipements, états du système et capacités

	1	2	3	4	5	6	7	8	9	10	Je ne sais pas
FP M1 - Plan/Préparation - État et capacité de l'équipement, du personnel et de la structure du programme de PF avant la crise. (1 = faible état/capacité ; 10 = très bon état/capacité)	◯	◯	◯	◯	◯	◯	◯	◯	◯	◯	◯
FP M2 - Absorption – Intégration : Niveau d'intégration des services et programmes de PF pour mitiger les effets de la pandémie de COVID-19 sur la PF.	◯	◯	◯	◯	◯	◯	◯	◯	◯	◯	◯
FP M3 - Absorption – Intégration : Mesure dans laquelle la gestion efficace de la chaîne d'approvisionnement a pu mitiger les effets de la pandémie de COVID-19 sur l'approvisionnement/distribution des commodités de PF.	◯	◯	◯	◯	◯	◯	◯	◯	◯	◯	◯
FP M4 - Récupération - Mesure dans laquelle le secteur de la PF a été capable d'induire des changements pour récupérer un niveau de fonctionnalité pré-COVID-19.	◯	◯	◯	◯	◯	◯	◯	◯	◯	◯	◯
FP M5 - Récupération – Gestion du financement et des bailleurs de fonds : Niveau de mobilisation des ressources et allocations pour mitiger les effets de la pandémie de COVID-19 sur la PF.	◯	◯	◯	◯	◯	◯	◯	◯	◯	◯	◯
FP M6 - Adaptation – Mesure dans laquelle des changements ont été effectués pour améliorer la résilience du secteur PF.	◯	◯	◯	◯	◯	◯	◯	◯	◯	◯	◯

* Dimension informatique : création, gestion, stockage et utilisation des données.

	1	2	3	4	5	6	7	8	9	10	Je ne sais pas
FP M7 - Plan/Préparation – Mesure dans laquelle les données liées à la PF étaient suffisamment préparées, présentées, analysées et stockées avant la crise.	◯	◯	◯	◯	◯	◯	◯	◯	◯	◯	◯
FP M8 - Absorption – Connaissance : Mesure dans laquelle le programme de PF a pu conduire des évaluations en temps réel de son état de fonctionnement, en anticipation des pertes en cascade des services de PF.	◯	◯	◯	◯	◯	◯	◯	◯	◯	◯	◯
FP M9 - Récupération – Mesure dans laquelle les données de la PF ont été utilisées pour suivre le progrès vers la récupération et anticiper des scenari de récupération.	◯	◯	◯	◯	◯	◯	◯	◯	◯	◯	◯
FP M10 - Adaptation – Mesure dans laquelle le système de la PF crée et améliore actuellement ses protocoles de stockage et d'utilisation des données de PF en temps réel.	◯	◯	◯	◯	◯	◯	◯	◯	◯	◯	◯

* Dimension cognitive : compréhension, modèles cognitifs, idées reçues, préjugés et valeurs.

	1	2	3	4	5	6	7	8	9	10	Je ne sais pas
FP M11 - Planification/Préparation – Mesure dans laquelle le système de la PF et les décisions opérationnelles étaient préparés à anticiper la crise.	◯	◯	◯	◯	◯	◯	◯	◯	◯	◯	◯

	1	2	3	4	5	6	7	8	9	10	Je ne sais pas
FP M12 - Absorption – Mesure dans laquelle la réponse du secteur de la PF a des protocoles de contingence suffisants et une gestion de crise proactive.	○	○	○	○	○	○	○	○	○	○	○
FP M13 - Absorption – Redevabilité : Niveau d'effort pour assurer une redevabilité optimale vis-à-vis des ressources allouées à la PF dans le cadre de la réponse à la pandémie de COVID-19.	○	○	○	○	○	○	○	○	○	○	○
FP M14 - Récupération – Mesure dans laquelle les décisions sont orientées vers la récupération et communiquées aux communautés sur la base de données probantes pour promouvoir des comportements sûrs.	○	○	○	○	○	○	○	○	○	○	○
FP M15 - Récupération – Connaissance/Conscience : Mesure dans laquelle le secteur de la PF établit une certaine confiance à travers sa communication sur les barrières posées par la pandémie de COVID-19 pour soutenir l'accès et l'utilisation des services et programmes de PF.	○	○	○	○	○	○	○	○	○	○	○
FP M16 - Adaptation : Niveau d'adaptation pour répondre aux menaces posées par la pandémie de COVID-19 à la PF.	○	○	○	○	○	○	○	○	○	○	○

* Social dimension: interaction, collaboration and self-synchronization between people, entities and institutions.

	1	2	3	4	5	6	7	8	9	10	Je ne sais pas
FP M17 - Planification /Préparation – Mesure dans laquelle une formation sur la gestion d'épidémies/crises a été menée et a permis de mettre à profit les réseaux sociaux, le capital social et les normes institutionnelles et culturelles avant la crise.	○	○	○	○	○	○	○	○	○	○	○
FP M18 - Absorption – Mesure dans laquelle le personnel et les institutions sociales ont été accessibles et ont fait preuve d'ingéniosité dans leur réponse à l'épidémie/crise.	○	○	○	○	○	○	○	○	○	○	○
PF M19 - Absorption - Self-regulation: Extent to which national leaders had the authority to effect timely sectoral changes through an infrastructureflexible.	○	○	○	○	○	○	○	○	○	○	○
FP M20 - Récupération – Mesure dans laquelle le secteur de la PF est impliqué dans le partage de connaissances et d'équipes pour améliorer la récupération du système.	○	○	○	○	○	○	○	○	○	○	○
FP M21 - Récupération – Diversité : Niveau d'engagement d'une équipe multidisciplinaire pour mitiger les effets de la pandémie de COVID-19 sur la PF.	○	○	○	○	○	○	○	○	○	○	○
FP M22 - Adaptation – Mesure dans laquelle des ajouts ou changements sont apportés aux institutions, politiques, formations, programmes et à la culture de la PF.	○	○	○	○	○	○	○	○	○	○	○

	1	2	3	4	5	6	7	8	9	10	Je ne sais pas
FP M23 - Adaptation - Leadership et management : Niveau de leadership démontré par les dirigeants du secteur de la PF pour mitiger les effets de la pandémie de COVID-19 sur la PF.	◯	◯	◯	◯	◯	◯	◯	◯	◯	◯	◯

* FP M-24 Temporalité : Mesure de la rapidité de la réaction du gouvernement –sa communication et mise en œuvre des mesures- pour mitiger l'impact immédiat et sur le long terme de la pandémie de COVID-19. Cette question porte sur la riposte nationale dans son ensemble, et non la réponse spécifique de votre secteur.

	1	2	3	4	5	6	7	8	9	10	Je ne sais pas
1-Plus faible -> Plus élevé-10	◯	◯	◯	◯	◯	◯	◯	◯	◯	◯	◯

FP M-25 Commentaires : Veuillez utiliser cette section pour tout commentaire additionnel sur la résilience et la durabilité des systèmes de PF nationaux en fonction de votre expérience de la pandémie de COVID-19. Si vous avez répondu « Je ne sais pas » à l'une des questions, utilisez aussi cette section pour expliquer pourquoi.

JOHNS HOPKINS
BLOOMBERG SCHOOL
of PUBLIC HEALTH

Bill & Melinda Gates Institute for Population and Reproductive Health

Enquête de l'Indice des efforts pour le dividende démographique

Questionnaire sur la santé maternelle et infantile (SMI)

Répondez aux questions suivantes en fonction de votre expérience/ expertise dans ce secteur spécifique.

Afin d'obtenir une représentation synthétique des efforts nationaux pour le dividende démographique, notez les éléments suivants sur une échelle de 1 à 10, 1 étant le score le plus faible (efforts très faibles ou quasi-inexistants) et 10 le plus élevé (efforts robustes). Le cas échéant, si une politique ou activité n'existe pas, répondez 0.

Donnez un score à chaque élément. Toutes les réponses seront enregistrées au format illustré ci-dessous :

Effort le plus faible	1	2	3	4	5	6	7	8	9	10	Effort le plus élevé	Je ne sais pas

JOHNS HOPKINS
BLOOMBERG SCHOOL
of PUBLIC HEALTH

Bill & Melinda Gates Institute for Population and Reproductive Health

Enquête de l'Indice des efforts pour le dividende démographique

Domaine 1. Politique/ Prise de décision

* MCH 1.1 L'adéquation de la politique : Politiques du Ministère de la Santé en faveur des services pendant la grossesse et l'accouchement qui tiennent compte de toutes les conditions requises pour s'assurer que la mère et son nouveau-né survivent.

	1	2	3	4	5	6	7	8	9	10	Je ne sais pas
MCH 1.1.1 Mesure dans laquelle la santé reproductive, maternelle et infantile a été intégrée à la politique/plan/stratégie de santé nationale.	○	○	○	○	○	○	○	○	○	○	○
MCH 1.1.2 Mesure dans laquelle le/les plan(s) de mise en œuvre de la/les stratégie(s) nationale(s) de santé reproductive, maternelle et infantile sont disponibles.	○	○	○	○	○	○	○	○	○	○	○

* MCH 1.2 L'accès universel : Mesure dans laquelle des droits légaux et constitutionnels existent pour faciliter l'accès universel aux services de santé reproductive, maternelle et infantile.

	1	2	3	4	5	6	7	8	9	10	Je ne sais pas
1-Plus faible -> Plus élevé-10	○	○	○	○	○	○	○	○	○	○	○

* MCH 1.3 L'implication de divers secteurs : Mesure dans laquelle des politiques sont élaborées à travers une consultation adéquate des parties intéressées, dont les ministères, les ONG, les praticiens privés et les associations de femmes.

	1	2	3	4	5	6	7	8	9	10	Je ne sais pas
1-Plus faible -> Plus élevé-10	○	○	○	○	○	○	○	○	○	○	○

* MCH 1.4 La compétence des prestataires de santé : Mesure dans laquelle les politiques sont rationnelles et fondées sur des données probantes établissant quel personnel peut fournir des services de santé maternelle (ex : les sages-femmes formées peuvent effectuer une vaste gamme de procédures).

	1	2	3	4	5	6	7	8	9	10	Je ne sais pas
1-Plus faible -> Plus élevé-10	○	○	○	○	○	○	○	○	○	○	○

* MCH 1.5 La légalisation de l'avortement : Mesure dans laquelle une politique favorable existe pour assurer l'offre légale de services d'avortement.

	1	2	3	4	5	6	7	8	9	10	Je ne sais pas
1-Plus faible -> Plus élevé-10	○	○	○	○	○	○	○	○	○	○	○

* MCH 1.6. La légalisation des soins post-avortement : Mesure dans laquelle une politique favorable existe pour le traitement des complications dues aux avortements, y compris les complications résultant d'avortements clandestins.

	1	2	3	4	5	6	7	8	9	10	Je ne sais pas
1-Plus faible -> Plus élevé-10	○	○	○	○	○	○	○	○	○	○	○

* MCH 1.7 La révision et mise à jour des politiques : Mesure dans laquelle les politiques sont régulièrement revues par des législateurs de haut niveau et utilisées dans les plans d'action.

	1	2	3	4	5	6	7	8	9	10	Je ne sais pas
1-Plus faible -> Plus élevé-10	○	○	○	○	○	○	○	○	○	○	○

* MCH 1.8 Le haut placement des acteurs : Mesure dans laquelle le/la directeur/trice des services de santé maternelle et infantile est haut placé(e) au gouvernement et impliqué(e) dans la prise de décision.

	1	2	3	4	5	6	7	8	9	10	Je ne sais pas
1-Plus faible -> Plus élevé-10	○	○	○	○	○	○	○	○	○	○	○

* MCH 1.9 Le financement de la SMI : Mesure dans laquelle les budgets du gouvernement pour la grossesse et l'accouchement sans risque, ainsi que pour les soins périnataux (pour les structures de santé, le personnel, les produits, etc.) et la santé infantile sont adaptés aux besoins, et si ces financements proviennent du Ministère de la Santé, gouvernement provincial/ des comtés ou des bailleurs de fonds.

	1	2	3	4	5	6	7	8	9	10	Je ne sais pas
1-Plus faible -> Plus élevé-10	○	○	○	○	○	○	○	○	○	○	○

* MCH 1.10 L'accessibilité financière : Mesure dans laquelle les services et médicaments liés à la SMI sont à un prix abordable pour toutes les clientes.

	1	2	3	4	5	6	7	8	9	10	Je ne sais pas
1-Plus faible -> Plus élevé-10	○	○	○	○	○	○	○	○	○	○	○

* MCH 1.11 Le secteur privé : Mesure dans laquelle le secteur privé (médecins, sages-femmes, cliniques) est actif et couvre une part importance des soins de grossesse et d'accouchement.

	1	2	3	4	5	6	7	8	9	10	Je ne sais pas
1-Plus faible -> Plus élevé-10	○	○	○	○	○	○	○	○	○	○	○

* MCH 1.12 Le directorat de SMI : Mesure dans laquelle le Ministère de la Santé a un directorat spécifiquement dédié à la SMI.

	1	2	3	4	5	6	7	8	9	10	Je ne sais pas
1-Plus faible -> Plus élevé-10	○	○	○	○	○	○	○	○	○	○	○

* MCH 1.13 Les solutions potentielles : Mesure dans laquelle le gouvernement reconnait des solutions potentielles aux problèmes de SMI par les moyens suivants :

	1	2	3	4	5	6	7	8	9	10	Je ne sais pas
MCH 1.13.1 Efficience régulatrice	○	○	○	○	○	○	○	○	○	○	○
MCH 1.13.2 Performance et redevabilité	○	○	○	○	○	○	○	○	○	○	○
MCH 1.13.3 Approche fondée sur le droit de la SMNI	○	○	○	○	○	○	○	○	○	○	○
MCH 1.13.4 Infrastructure de santé essentielle	○	○	○	○	○	○	○	○	○	○	○
MCH 1.13.5 Engagement de la communauté	○	○	○	○	○	○	○	○	○	○	○

MCH 1.14 Commentaires : Utilisez cette section pour apporter des commentaires additionnels sur le niveau d’effort, les défis et les succès du pays autour de la politique/ prise de décision pour la SMI. Si vous avez répondu « Je ne sais pas » à l’une des questions précédentes, utilisez aussi cette section pour expliquer pourquoi.

JOHNS HOPKINS BLOOMBERG SCHOOL of PUBLIC HEALTH

Bill & Melinda Gates Institute for Population and Reproductive Health

Enquête de l'Indice des efforts pour le dividende démographique

Dimension 2. Services or Programs

* MCH 2.1 La disponibilité des soins obstétriques – Dans tous les centres : Mesure dans laquelle les structures de santé primaire ont du personnel qualifié, en place, et peuvent fournir des soins obstétriques :

	1	2	3	4	5	6	7	8	9	10	Je ne sais pas
MCH 2.1.1 Cas d'hémorragie postpartum adéquatement gérés	○	○	○	○	○	○	○	○	○	○	○
MCH 2.1.2 Administration adéquate et opportune d'antibiotiques en intraveineuse lorsque nécessaire.	○	○	○	○	○	○	○	○	○	○	○
MCH 2.1.3 Expulsion manuelle dans les cas de rétention placentaire.	○	○	○	○	○	○	○	○	○	○	○
MCH 2.1.4 Aspiration de l'utérus manuelle (Méthode de Karman) ou à l'aide d'un appareil à succion électrique.	○	○	○	○	○	○	○	○	○	○	○
MCH 2.1.5 Utilisation d'un partogramme pour savoir quand référer.	○	○	○	○	○	○	○	○	○	○	○
MCH 2.1.6 Transport organized to quickly transfer a woman with obstructed labor to the district / sub-county hospital.	○	○	○	○	○	○	○	○	○	○	○
MCH 2.1.7 Antibiotiques adéquats disponibles (suffisamment de produits du bon type).	○	○	○	○	○	○	○	○	○	○	○

* MCH 2.2 La disponibilité des soins obstétriques – Les structures de santé secondaires : Mesure dans laquelle tous les hôpitaux de districts/sous-comtés ont du personnel formé, en place, capable de :

	1	2	3	4	5	6	7	8	9	10	Je ne sais pas
MCH 2.2.1 Remplir toutes les fonctions citées ci-dessus pour les structures de santé primaires	◯	◯	◯	◯	◯	◯	◯	◯	◯	◯	◯
MCH 2.2.2 Effectuer des transfusions sanguines (et avoir un approvisionnement en sang sûr, adéquat et disponible).	◯	◯	◯	◯	◯	◯	◯	◯	◯	◯	◯
MCH 2.2.3 Effectuer des césariennes.	◯	◯	◯	◯	◯	◯	◯	◯	◯	◯	◯

* MCH 2.3 Les services liés à la grossesse : Mesure dans laquelle toutes les femmes enceintes ont accès à :

	1	2	3	4	5	6	7	8	9	10	Je ne sais pas
MCH 2.3.1 Traitement des hémorragies postpartum pendant ou peu après l'accouchement.	○	○	○	○	○	○	○	○	○	○	○
MCH 2.3.2 Gestion de la dystocie.	○	○	○	○	○	○	○	○	○	○	○
MCH 2.3.3 Management de la pré-éclampsie, éclampsie et ses complications.	○	○	○	○	○	○	○	○	○	○	○
MCH 2.3.4 Soins post-avortement ou pour les complications liées aux avortements.	○	○	○	○	○	○	○	○	○	○	○
MCH 2.3.5 Offre de services d'avortement sans risque.	○	○	○	○	○	○	○	○	○	○	○
MCH 2.3.6 Soins prénatals pendant la grossesse.	○	○	○	○	○	○	○	○	○	○	○
MCH 2.3.7 Soins d'accouchement par un professionnel formé.	○	○	○	○	○	○	○	○	○	○	○
MCH 2.3.8 Services de planification familiale postpartum.	○	○	○	○	○	○	○	○	○	○	○
MCH 2.3.9 Hôpitaux des districts/ sous-comtés ouverts 24h/24 et 7j/7.	○	○	○	○	○	○	○	○	○	○	○

* MCH 2.4 Les soins prénatals pour les femmes non mariées et séropositives : Mesure dans laquelle, à toutes les consultations prénatales, toutes les femmes enceintes :

	1	2	3	4	5	6	7	8	9	10	Je ne sais pas
MCH 2.4.1 Reçoivent des soins prénatals complets conformément aux protocoles recommandés par l'OMS pour les femmes séronégatives.	○	○	○	○	○	○	○	○	○	○	○
MCH 2.4.2 Reçoivent des soins prénatals complets conformément aux protocoles recommandés par l'OMS pour les femmes séropositives.	○	○	○	○	○	○	○	○	○	○	○
MCH 2.4.3 Reçoivent des soins prénatals complets conformément aux protocoles recommandés par l'OMS pour les jeunes femmes non mariées.	○	○	○	○	○	○	○	○	○	○	○
MCH 2.4.4 Sont informées des signes de danger de complications obstétriques et néonatales, et sont assistées pour planifier toute urgence éventuelle.	○	○	○	○	○	○	○	○	○	○	○

* MCH 2.5 Les soins néonatals : Mesure dans laquelle, pour les soins néonatals, tous les nouveau-nés, qu’ils soient nés à la maison ou en structure médicale :

	1	2	3	4	5	6	7	8	9	10	Je ne sais pas
MCH 2.5.1 Ont leur bouche et voie nasale dégagées.	○	○	○	○	○	○	○	○	○	○	○
MCH 2.5.2 Sont séchés et gardés au chaud immédiatement après la naissance.	○	○	○	○	○	○	○	○	○	○	○
MCH 2.5.3 Reçoivent de la vitamine A.	○	○	○	○	○	○	○	○	○	○	○
MCH 2.5.4 Ont leur cordon ombilical coupé avec une lame propre.	○	○	○	○	○	○	○	○	○	○	○
MCH 2.5.5 Suivent le calendrier de vaccination recommandé par l’OMS.	○	○	○	○	○	○	○	○	○	○	○
MCH 2.5.6 Bénéficient des activités de promotion de l’allaitement exclusif.	○	○	○	○	○	○	○	○	○	○	○
MCH 2.5.7 Bénéficient des programmes de soutien alimentaire lorsqu’ils en ont besoin.	○	○	○	○	○	○	○	○	○	○	○

* MCH 2.6 La planification familiale – Dans tous les centres : Mesure dans laquelle la planification familiale est dûment fournie dans tous les centres de santé en :

	1	2	3	4	5	6	7	8	9	10	Je ne sais pas
MCH 2.6.1 Offrant régulièrement des services de planification familiale aux cas post-avortement.	○	○	○	○	○	○	○	○	○	○	○
MCH 2.6.2 Offrant régulièrement des services de planification familiale lors des consultations postpartum.	○	○	○	○	○	○	○	○	○	○	○
MCH 2.6.3 Ayant des méthodes contraceptives réversibles de courte et longue durée régulièrement en stock.	○	○	○	○	○	○	○	○	○	○	○
MCH 2.6.4 Ayant du personnel formé, sur place, pouvant fournir les méthodes LARC.	○	○	○	○	○	○	○	○	○	○	○
MCH 2.6.5 Suivant les directives et protocoles nécessaires à la prestation de services de planification familiale.	○	○	○	○	○	○	○	○	○	○	○

* MCH 2.7 La planification familiale – Mesure dans laquelle la planification familiale est dûment fournie dans tous hôpitaux de districts/sous-comtés en :

	1	2	3	4	5	6	7	8	9	10	Je ne sais pas
MCH 2.7.1 Offrant régulièrement des services de planification familiale aux cas post-avortement.	○	○	○	○	○	○	○	○	○	○	○
MCH 2.7.2 Offrant régulièrement des services de planification familiale lors des consultations postpartum.	○	○	○	○	○	○	○	○	○	○	○
MCH 2.7.3 Ayant des méthodes contraceptives réversibles de courte et longue durée régulièrement en stock.	○	○	○	○	○	○	○	○	○	○	○
MCH 2.7.4 Ayant du personnel formé, sur place, pouvant fournir les méthodes LARC.	○	○	○	○	○	○	○	○	○	○	○
MCH 2.7.5 Pouvant offrir la stérilisation féminine.	○	○	○	○	○	○	○	○	○	○	○
MCH 2.7.6 Pouvant offrir la stérilisation masculine.	○	○	○	○	○	○	○	○	○	○	○
MCH 2.7.7 Suivant les directives et protocoles nécessaires à la prestation de services de planification familiale.	○	○	○	○	○	○	○	○	○	○	○

* MCH 2.8 Les maladies infantiles : Mesure dans laquelle la gestion de maladies infantiles recommandée par l'OMS est mise en œuvre en termes de :

	1	2	3	4	5	6	7	8	9	10	Je ne sais pas
MCH 2.8.1 Gestion à base communautaire des maladies infantiles.	○	○	○	○	○	○	○	○	○	○	○
MCH 2.8.2 Efficacité des services de référence du niveau communautaire vers les structures de santé.	○	○	○	○	○	○	○	○	○	○	○

MCH 2.9 Commentaires : Utilisez cette section pour apporter des commentaires additionnels sur le niveau d’effort, les défis et les succès du pays autour des programmes ou services de SMI. Si vous avez répondu « Je ne sais pas » à l’une des questions précédentes, utilisez aussi cette section pour expliquer pourquoi.

JOHNS HOPKINS
BLOOMBERG SCHOOL of PUBLIC HEALTH

Bill & Melinda Gates Institute for Population and Reproductive Health

Enquête de l'Indice des efforts pour le dividende démographique

Domaine 3. Le plaidoyer

* MCH 3.1 Les messages du gouvernement : Mesure dans laquelle les hauts fonctionnaires au gouvernement, dont au Ministère de la Santé, s'adressent fréquemment à toutes les parties prenantes, y compris la presse, pour soutenir des améliorations et promouvoir les grossesses et les accouchements sans risque, et les enfants en bonne santé.

	1	2	3	4	5	6	7	8	9	10	Je ne sais pas
1-Plus faible -> Plus élevé-10	○	○	○	○	○	○	○	○	○	○	○

* MCH 3.2 Les médias de masse : Mesure dans laquelle le programme national utilise les médias de masse pour éduquer le public sur les symptômes des complications liées aux grossesses, sur les accouchements sans risque et les enfants en bonne santé.

	1	2	3	4	5	6	7	8	9	10	Je ne sais pas
1-Plus faible -> Plus élevé-10	○	○	○	○	○	○	○	○	○	○	○

* MCH 3.3 Les médias au niveau communautaire : Mesure dans laquelle les groupes communautaires prennent part aux programmes systématiques pour éduquer le public sur les grossesses et accouchements sans risque et les enfants en bonne santé.

	1	2	3	4	5	6	7	8	9	10	Je ne sais pas
1-Plus faible -> Plus élevé-10	○	○	○	○	○	○	○	○	○	○	○

* MCH 3.4 Les supports éducatifs : Mesure dans laquelle le ministère compétent (Ministère de la Santé) fournit des supports éducatifs adéquats (posters, brochures, etc.) aux structures de santé pour qu'elles instruisent les client(e)s sur les pratiques sûres/sans risque.

	1	2	3	4	5	6	7	8	9	10	Je ne sais pas
1-Plus faible -> Plus élevé-10	◯	◯	◯	◯	◯	◯	◯	◯	◯	◯	◯

* MCH 3.5 Auto-révision des cas de mortalité maternelle : Mesure dans laquelle chaque structure de santé suit une procédure régulière de révision de tous ses cas de décès maternels ayant eu lieu dans la structure pour en tirer des leçons.

	1	2	3	4	5	6	7	8	9	10	Je ne sais pas
1-Plus faible -> Plus élevé-10	◯	◯	◯	◯	◯	◯	◯	◯	◯	◯	◯

* MCH 3.6 Informer les politiques avec de nouvelles données probantes : Mesure dans laquelle chaque structure de santé transmet les leçons tirées des cas de décès/ mortalité maternelle aux ministères pour informer les nouvelles politiques et interventions.

	1	2	3	4	5	6	7	8	9	10	Je ne sais pas
1-Plus faible -> Plus élevé-10	◯	◯	◯	◯	◯	◯	◯	◯	◯	◯	◯

MCH 3.7 Commentaires : Utilisez cette section pour apporter des commentaires additionnels sur le niveau d'effort, les défis et les succès du pays autour du plaidoyer pour la SMI. Si vous avez répondu « Je ne sais pas » à l'une des questions précédentes, utilisez aussi cette section pour expliquer pourquoi.

JOHNS HOPKINS
BLOOMBERG SCHOOL
of PUBLIC HEALTH

Bill & Melinda Gates Institute for Population and Reproductive Health

Enquête de l'Indice des efforts pour le dividende démographique

Domaine 4. Recherche

* MCH 4.1 La force des observatoires/parties prenantes/groupes de travail pour la SMI : Mesure dans laquelle les parties prenantes de la SMI du pays (ex : groupe directif de parties prenantes, groupes de travail techniques, observatoire de SMI, groupe de coordination ou facilitation) :

	1	2	3	4	5	6	7	8	9	10	Je ne sais pas
MCH 4.1.1 Ont un représentant du gouvernement, des institutions de formation, de la société civile, des organisations non gouvernementales et confessionnelles, des associations professionnelles, du secteur privé, etc.	◯	◯	◯	◯	◯	◯	◯	◯	◯	◯	◯
MCH 4.1.2 Se réunissent régulièrement, émettent des rapports et recommandent des politiques aux directions des ministères compétents.	◯	◯	◯	◯	◯	◯	◯	◯	◯	◯	◯
MCH 4.1.3 Ont un impact sur l'éducation dans le pays.	◯	◯	◯	◯	◯	◯	◯	◯	◯	◯	◯

* MCH 4.2 MCH La stratégie d'éducation/recherche : Mesure dans laquelle le plan national de SMI et/ou d'autres documents nationaux comprennent une stratégie/approche complète de recherche liée sur la SMI et est partagée entre partenaires.

	1	2	3	4	5	6	7	8	9	10	Don't know
1-Plus faible -> Plus élevé-10	◯	◯	◯	◯	◯	◯	◯	◯	◯	◯	◯

* MCH 4.3 Les partenaires collectant les données:

	1	2	3	4	5	6	7	8	9	10	Je ne sais pas
MCH 4.3.1 Mesure dans laquelle la collecte des données est entreprise par des agences gouvernementales. Cela peut comprendre les agences/bureaux de la statistique et/ou les ministères de l'économie/finances, éducation, santé, genre, etc.).	○	○	○	○	○	○	○	○	○	○	○
MCH 4.3.2 Mesure dans laquelle la collecte de données est entreprise par des institutions de recherche.	○	○	○	○	○	○	○	○	○	○	○
MCH 4.3.3 Mesure dans laquelle la collecte des données est entreprise par des chercheurs indépendants.	○	○	○	○	○	○	○	○	○	○	○

* MCH 4.4 La recherche thématique :

	1	2	3	4	5	6	7	8	9	10	Je ne sais pas
MCH 4.4.1 Mesure dans laquelle de la recherche est menée sur la mortalité maternelle.	○	○	○	○	○	○	○	○	○	○	○
MCH 4.4.2 Mesure dans laquelle de la recherche est menée sur la mortalité néonatale et infantile.	○	○	○	○	○	○	○	○	○	○	○
MCH 4.4.3 Mesure dans laquelle de la recherche est menée sur la planification familiale et la planification familiale postpartum.	○	○	○	○	○	○	○	○	○	○	○
MCH 4.4.4 Mesure dans laquelle de la recherche est menée sur la prestation/accès aux services et la qualité des prestataires de SMI.	○	○	○	○	○	○	○	○	○	○	○

* MCH 4.5 La qualité/couverture des données : Mesure dans laquelle la recherche/les données actuelles sont désagrégées par sexe, âge, lieu, ethnicité, niveau d'études, quintile de revenus et statut d'handicap. Mesure dans laquelle le pays participe à des tests standardisés et son classement.

	1	2	3	4	5	6	7	8	9	10	Je ne sais pas
MCH 4.5.1 Mesure dans laquelle un système statistique de routine offre de bonnes données périodiques sur les produits/l'approvisionnement et les structures de santé.											
MCH 4.5.2 Mesure dans laquelle un système statistique de routine offre de bonnes données périodiques sur les services et le personnel de SMI.											
MCH 4.5.3 Mesure dans laquelle un système statistique de routine offre de bonnes données périodiques sur les besoins en SMI de différentes populations dans les communautés.											
MCH 4.5.4 4 Mesure dans laquelle des évaluations du système de suivi de l'éducation sont menées et appliquées pour assurer la fiabilité des données.											
MCH 4.5.5 Mesure dans laquelle des évaluations du système de suivi de l'éducation sont menées et appliquées pour explorer les inégalités dans les services et fardeaux de la SMNI.											

* MCH 4.6 Les registres : Mesure dans laquelle les systèmes de registres, rapports et feedback sur les résultats de SMI sont adéquats, y compris en étant connectés à tous les niveaux (fédéral, état/province, comté, etc.).

	1	2	3	4	5	6	7	8	9	10	Je ne sais pas
1-Plus faible -> Plus élevé-10											

* MCH 4.7 La qualité des institutions de recherche : Mesure dans laquelle le pays a la capacité de soutenir et maintenir des institutions de recherche qui développent des études et/ou collectent des données permettant des services SMI de haute qualité.

	1	2	3	4	5	6	7	8	9	10	Je ne sais pas
1-Plus faible -> Plus élevé-10	◯	◯	◯	◯	◯	◯	◯	◯	◯	◯	◯

* MCH 4.8 L'évaluation : Mesure dans laquelle les statistiques, enquêtes et études sur le programme de SMI sont utilisées par du personnel spécialisé pour rapporter les opérations du programme et mesurer le progrès.

	1	2	3	4	5	6	7	8	9	10	Je ne sais pas
1-Plus faible -> Plus élevé-10	◯	◯	◯	◯	◯	◯	◯	◯	◯	◯	◯

* MCH 4.9 L'utilisation des résultats d'évaluations par les gestionnaires de programmes : Mesure dans laquelle les gestionnaires des programme locaux utilisent les conclusions de recherche et d'évaluations pour améliorer ces programmes en suivant les recommandations émises.

	1	2	3	4	5	6	7	8	9	10	Je ne sais pas
1-Plus faible -> Plus élevé-10	◯	◯	◯	◯	◯	◯	◯	◯	◯	◯	◯

* MCH 4.10 L'utilisation des résultats d'évaluation par les ministères : Mesure dans laquelle les administrateurs ministériels compétents et responsables d'élaborer les politiques utilisent systématiquement les données pour informer les politiques et interventions visant à résoudre les problèmes liés à la SMI.

	1	2	3	4	5	6	7	8	9	10	Je ne sais pas
1-Plus faible -> Plus élevé-10	◯	◯	◯	◯	◯	◯	◯	◯	◯	◯	◯

* MCH 4.11 La dissémination d'informations à d'autres entités chargées de la mise en œuvre des programmes : Mesure dans laquelle les informations sont communiquées ou disséminées entre différentes aires géographiques et à différents niveaux (national, état/province, collectivités territoriales).

	1	2	3	4	5	6	7	8	9	10	Je ne sais pas
1-Plus faible -> Plus élevé-10	◯	◯	◯	◯	◯	◯	◯	◯	◯	◯	◯

MCH 4.12 Commentaires : Utilisez cette section pour apporter des commentaires additionnels sur le niveau d’effort, les défis et les succès du pays autour de la recherche sur la SMI. Si vous avez répondu « Je ne sais pas » à l’une des questions précédentes, utilisez aussi cette section pour expliquer pourquoi.

Bill & Melinda Gates Institute for Population and Reproductive Health

Enquête de l'Indice des efforts pour le dividende démographique

Domaine 5. Organisations de la société civile (OSC)

* MCH 5.1 Le pouvoir des acteurs des OSC : Mesure dans laquelle les acteurs des OSC occupent des postes d'influence dans les institutions, les groupes de travail techniques ou les réseaux concernés par la SMI.

	1	2	3	4	5	6	7	8	9	10	Je ne sais pas
1-Plus faible -> Plus élevé-10	○	○	○	○	○	○	○	○	○	○	○

* MCH 5.2 L'analyse budgétaire comme outil des OSC : Mesure dans laquelle les OSC utilisent l'analyse budgétaire comme un outil pour développer le plaidoyer en matière de SMI.

	1	2	3	4	5	6	7	8	9	10	Je ne sais pas
1-Plus faible -> Plus élevé-10	○	○	○	○	○	○	○	○	○	○	○

* MCH 5.3 Le soutien à la prestation de services en structure sanitaire : Mesure dans laquelle les OSC sont impliquées dans des efforts visant à augmenter l'utilisation et/ou la qualité des services, particulièrement à travers l'augmentation des capacités techniques et/ou opérationnelles des cliniques partenaires des OSC afin de fournir des services de SMI.

	1	2	3	4	5	6	7	8	9	10	Je ne sais pas
1-Plus faible -> Plus élevé-10	○	○	○	○	○	○	○	○	○	○	○

* MCH 5.4 Le soutien à la prestation des services à base communautaire : Mesure dans laquelle les OSC participent au rapprochement des informations et services de SMI avec les communautés à travers des activités comprenant le renforcement de compétences des agents de santé communautaire (ASC), l'expansion de la gamme de services SMI offerts par les ASC, l'orientation/éducation des travailleurs de la santé de cadres inférieurs et/ou des campagnes d'information ou d'éducation.

	1	2	3	4	5	6	7	8	9	10	Je ne sais pas
1-Plus faible -> Plus élevé-10	○	○	○	○	○	○	○	○	○	○	○

* MCH 5.5 Le soutien à la sensibilisation mobile pour l'offre de services à base communautaire : Mesure dans laquelle les OSC soutiennent les modèles de sensibilisation mobile pour équiper les prestataires de santé en leur fournissant des commodités et produits liés à la SMI, de l'équipement et des véhicules afin de fournir en tant qu'équipe spécialiste des services SMI directement aux communautés.

	1	2	3	4	5	6	7	8	9	10	Je ne sais pas
1-Plus faible -> Plus élevé-10	○	○	○	○	○	○	○	○	○	○	○

* MCH 5.6 La franchise sociale : Mesure dans laquelle les OSC sont impliquées dans des opportunités/ services de franchise sociale pour offrir des services de SMI dans le secteur privé à un prix réduit.

	1	2	3	4	5	6	7	8	9	10	Je ne sais pas
1-Plus faible -> Plus élevé-10	○	○	○	○	○	○	○	○	○	○	○

* MCH 5.7 La santé mobile : Mesure dans laquelle les OSC mettent à profit la technologie mobile en utilisant la messagerie (textos) et autres technologies mobiles pour améliorer l'accès aux informations et services de SMI.

	1	2	3	4	5	6	7	8	9	10	Je ne sais pas
1-Plus faible -> Plus élevé-10	○	○	○	○	○	○	○	○	○	○	○

* MCH 5.8 Les droits humains et la qualité des soins : Mesure dans laquelle les OSC soutiennent et plaident pour des approches fondées sur le droit pour s'assurer que les clientes de la SMI soient bien traitées, n'aient pas peur de poser des questions et reçoivent du conseil de qualité, entre autres sujets.

	1	2	3	4	5	6	7	8	9	10	Je ne sais pas
1-Plus faible -> Plus élevé-10	○	○	○	○	○	○	○	○	○	○	○

* MCH 5.9 Le changement social et des comportements (CSC) : Mesure dans laquelle les OSC soutiennent les interventions pour le CSC comprenant, mais sans s'y limiter, les compétences de vie et l'éducation entre pairs, l'éducation sur la santé de l'adulte, l'éducation en milieu scolaire, la communication au sein du couple, la mobilisation communautaire, les interventions à travers les gardiens de la communauté et/ou dans les médias de masse pour le grand public.

	1	2	3	4	5	6	7	8	9	10	Je ne sais pas
1-Plus faible -> Plus élevé-10	○	○	○	○	○	○	○	○	○	○	○

* MCH 5.10 Le plaidoyer/ la redevabilité : Mesure dans laquelle les OSC soutiennent la formation et le renforcement de compétences des plaideurs et des membres de la communauté pour améliorer leur compréhension des politiques et processus, leurs activités d'identification de problèmes, la résolution collaborative de ces problèmes, et des actions de plaidoyer plus ciblées pour améliorer la SMI.

	1	2	3	4	5	6	7	8	9	10	Je ne sais pas
1-Plus faible -> Plus élevé-10	○	○	○	○	○	○	○	○	○	○	○

* MCH 5.11 L'évaluation et le suivi dirigés par les OSC : Mesure dans laquelle les OSC évaluent, suivent et émettent des rapports sur l'efficacité des politiques et programmes pour améliorer la redevabilité des prestataires et des politiques de SMI.

	1	2	3	4	5	6	7	8	9	10	Je ne sais pas
1-Plus faible -> Plus élevé-10	○	○	○	○	○	○	○	○	○	○	○

* MCH 5.12 Les partenariats entre OSC : Mesure dans laquelle les OSC ont formé des alliances nationales et des partenariats régionaux pour renforcer leur position et potentiellement améliorer la force du leadership et les mécanismes de financement pour la SMI.

	1	2	3	4	5	6	7	8	9	10	Je ne sais pas
1-Plus faible -> Plus élevé-10	○	○	○	○	○	○	○	○	○	○	○

MCH 5.13 Commentaires : Utilisez cette section pour apporter des commentaires additionnels sur le niveau d'effort, les défis et les succès des OSC travaillant pour la SMI. Si vous avez répondu « Je ne sais pas » à l'une des questions précédentes, utilisez aussi cette section pour expliquer pourquoi.

JOHNS HOPKINS
BLOOMBERG SCHOOL
of PUBLIC HEALTH

Bill & Melinda Gates Institute for Population and Reproductive Health

Enquête de l'Indice des efforts pour le dividende démographique

Module : Résilience et durabilité du secteur

Étant donné l'impact sanitaire et socioéconomique de la pandémie de COVID-19, et son impact probable sur la progression vers le DD, cet indice des efforts pour le DD intègre des questions pour évaluer la résilience et la durabilité des systèmes dans les secteurs clés du DD. Les éléments couverts par les questions liées à la pandémie de COVID-19 ci-dessous relèvent des dimensions clés des systèmes résilients et visent à apporter des informations déterminantes sur le potentiel d'une réponse efficace aux menaces de maladies infectieuses émergeantes et autres crises de santé publique.

Veuillez noter chaque élément sur une échelle de 1 à 10, 1 étant le score le plus faible (faible état/capacité) et 10 le plus élevé (très bon état/capacité).

* Dimension physique : structures, équipements, états du système et capacités.

	1	2	3	4	5	6	7	8	9	10	Je ne sais pas
MCH-M1 - Planification/Préparation – État et capacité de l'équipement, du personnel et de la structure du programme de SMI avant la crise.	○	○	○	○	○	○	○	○	○	○	○
MCH-M2 – Absorption – Intégration : Niveau d'intégration des services et programmes de SMI pour mitiger les effets de la pandémie de COVID-19 sur la SMI.	○	○	○	○	○	○	○	○	○	○	○

	1	2	3	4	5	6	7	8	9	10	Je ne sais pas
MCH-M3 - Absorption – Intégration : Mesure dans laquelle la gestion efficace de la chaîne d'approvisionnement a permis de mitiger les effets de la pandémie de COVID-19 sur l'approvisionnement/livraison des commodités liées à la SMI.	○	○	○	○	○	○	○	○	○	○	○
MCH-M4 - Récupération – Mesure dans laquelle le programme de SMI a été capable d'induire des changements pour récupérer un niveau de fonctionnalité pre-COVID-19.	○	○	○	○	○	○	○	○	○	○	○
MCH-M5 - Récupération – Gestion du financement et des bailleurs de fonds : Niveau de mobilisation de ressources et d'allocation à la mitigation des effets de la pandémie de COVID-19 sur la SMI.	○	○	○	○	○	○	○	○	○	○	○
MCH-M6 - Adaptation – Mesure dans laquelle des changements ont été effectués pour améliorer la résilience du secteur SMI.	○	○	○	○	○	○	○	○	○	○	○

* Dimension informatique : création, gestion, stockage et utilisation des données.

	1	2	3	4	5	6	7	8	9	10	Don't know
MCH-M7 - Planification/Préparation - Mesure dans laquelle les données liées à la SMI étaient suffisamment préparées, présentées, analysées et stockées avant la crise.	○	○	○	○	○	○	○	○	○	○	○
MCH-M8 - Absorption – Connaissance/Conscience : Mesure dans laquelle la capacité de surveillance a pu détecter les limites/menaces posées par la pandémie de COVID-19 à la SMI.	○	○	○	○	○	○	○	○	○	○	○
MCH-M9 - Récupération – Mesure dans laquelle les données de SMI ont été utilisées pour suivre le progrès vers la récupération et anticiper des scenarii de récupération.	○	○	○	○	○	○	○	○	○	○	○
MCH-M10 - Adaptation – Mesure dans laquelle le système de SMI crée et améliore actuellement ses protocoles de stockage et d'utilisation de ses données en temps réel.	○	○	○	○	○	○	○	○	○	○	○

* Dimension cognitive: compréhension, modèles cognitifs, préconception, biais et valeurs.

	1	2	3	4	5	6	7	8	9	10	Je ne sais pas
MCH-M11 - Planification/Préparation - Mesure dans laquelle les décisions de conceptualisation et opérationnelle du secteur SMI étaient préparées à anticiper la crise.	○	○	○	○	○	○	○	○	○	○	○
MCH-M12 - Absorption - Mesure dans laquelle le secteur de la SMI a des protocoles de contingence suffisants et une gestion de crise proactive.	○	○	○	○	○	○	○	○	○	○	○

	1	2	3	4	5	6	7	8	9	10	Je ne sais pas
MCH-M13 - Absorption - Redevabilité: Niveau d'effort pour assurer une redevabiilté optimale vis-à-vis des ressources allouées en soutien à la SMI dans le cadre de la réponse à la pandémie de COVID-19.	○	○	○	○	○	○	○	○	○	○	○
MCH-M14 - Récupération - Mesure dans laquelle les décisions sont orientées vers la récupération, et communiquées sur la base de données probantes pour promouvoir des comportements sûrs auprès des membres de la communauté.	○	○	○	○	○	○	○	○	○	○	○
MCH-M15 - Récupération - Connaissance/Conscience: Mesure dans laquelle le secteur SMI établit une certaine confiance à travers ses communications concernant les barrières posées par la pandémie de COVID-19 pour soutenir l'accès et l'utilisation des services et programmes de SMI.	○	○	○	○	○	○	○	○	○	○	○
MCH-M16 - Adaptation – Adaptif : Mesure dans laquelle les dirigeants nationaux avaient l'autorité d'effectuer des changements pour s'adapter et répondre aux menaces posées par la pandémie de COVID-19 aux services et programmes de SMI.	○	○	○	○	○	○	○	○	○	○	○

* Dimension sociale : interaction, collaboration et auto-synchronisation entre les personnes, les entités et les institutions.

	1	2	3	4	5	6	7	8	9	10	Je ne sais pas
MCH-M17 - Planification/Préparation – Mesure dans laquelle une formation sur la gestion de crise/épidémie a été menée et a permis de mettre à profit les réseaux sociaux, le capital social et institutionnel, et les normes culturelles avant la crise.	○	○	○	○	○	○	○	○	○	○	○
MCH- M18 - Absorption – Mesure dans laquelle le personnel et les institutions sociales ont fait preuve d'ingéniosité et ont été accessibles pendant la réponse à l'épidémie/crise.	○	○	○	○	○	○	○	○	○	○	○
MCH-M19 - Absorption – Autorégulation : Mesure dans laquelle les dirigeants nationaux avaient l'autorité d'effectuer des changements sectoriels en temps opportun à travers une infrastructure flexible.	○	○	○	○	○	○	○	○	○	○	○
MCH-M20 - Récupération – Mesure dans laquelle le secteur SMI est engagé dans le partage de connaissances et d'équipes pour améliorer la récupération systémique.	○	○	○	○	○	○	○	○	○	○	○
MCH-M21 - Récupération – Diversité : Niveau d'engagement d'une équipe multidisciplinaire pour mitiger les effets de la pandémie de COVID-19 sur la SMI.	○	○	○	○	○	○	○	○	○	○	○

	1	2	3	4	5	6	7	8	9	10	Je ne sais pas
MCH-M22 - Adaptation – Mesure dans laquelle des ajouts ou modifications ont été apportés aux institutions, politiques, programmes, formations et cultures de SMI.	○	○	○	○	○	○	○	○	○	○	○
MCH-M23 - Adaptation - Leadership et management : Niveau de leadership démontré par les dirigeants de la SMI pour mitiger les effets de la pandémie de COVID-19 sur la SMI.	○	○	○	○	○	○	○	○	○	○	○

* MCH M-24 Temporalité : Mesure de la rapidité de la réaction du gouvernement –sa communication et mise en œuvre des mesures- pour mitiger l’impact immédiat et sur le long terme de la pandémie de COVID-19. Cette question porte sur la riposte nationale dans son ensemble, et non la réponse spécifique de votre secteur.

	1	2	3	4	5	6	7	8	9	10	Je ne sais pas
1-Plus faible -> Plus élevé-10	○	○	○	○	○	○	○	○	○	○	○

MCH M-25 Commentaires : Utilisez cette section pour apporter tout commentaire additionnel sur la résilience et la durabilité du système nationaux de SMI en vous référant à votre expérience de la pandémie de COVID-19. Si vous avez répondu « Je ne sais pas » à l’une des questions, utilisez cette section pour expliquer pourquoi

JOHNS HOPKINS
BLOOMBERG SCHOOL
of PUBLIC HEALTH

Bill & Melinda Gates Institute for Population and Reproductive Health

Enquête de l'Indice des efforts pour le dividende démographique

Questionnaire sur l'éducation (ED)

Répondez aux questions suivantes en fonction de votre expérience/ expertise dans ce secteur spécifique.

Afin d'obtenir une représentation synthétique des efforts nationaux pour le dividende démographique, notez les éléments suivants sur une échelle de 1 à 10, 1 étant le score le plus faible (efforts très faibles ou quasi-inexistants) et 10 le plus élevé (efforts robustes). Le cas échéant, si une politique ou activité n'existe pas, répondez 0.

Donnez un score à chaque élément. Toutes les réponses seront enregistrées au format illustré ci-dessous :

Effort le plus faible	1	2	3	4	5	6	7	8	9	10	Effort le plus élevé	Je ne sais pas

Johns Hopkins Bloomberg School of Public Health

Bill & Melinda Gates Institute for Population and Reproductive Health

Enquête de l'Indice des efforts pour le dividende démographique

Domaine 1. Politique/ Prise de décision

* ED 1.1 La qualité : Mesure dans laquelle il existe un plan/stratégie/programme pour améliorer la qualité de l'enseignement en milieu scolaire.

	1	2	3	4	5	6	7	8	9	10	Je ne sais pas
1-Plus faible -> Plus élevé - 10	○	○	○	○	○	○	○	○	○	○	○

* ED 1.2 Les groupes défavorisés :

	1	2	3	4	5	6	7	8	9	10	Je ne sais pas
ED 1.2.1. Mesure dans laquelle les stratégies ont été développées et mises en œuvre/déployées pour surmonter les obstacles d'apprentissage chez les filles enceintes et les mères adolescentes	○	○	○	○	○	○	○	○	○	○	○
ED 1.2.2 Mesure dans laquelle des stratégies ont été développées et mises en œuvre/déployées pour surmonter les obstacles d'apprentissage chez les personnes souffrant d'un handicap	○	○	○	○	○	○	○	○	○	○	○

* ED 1.3 Les enseignants : Mesure dans laquelle les problèmes liés au recrutement, développement et management du personnel enseignant sont inclus à la politique visant à améliorer les résultats d'apprentissage.

	1	2	3	4	5	6	7	8	9	10	Je ne sais pas
1-Plus faible->Plus élevé-10	◯	◯	◯	◯	◯	◯	◯	◯	◯	◯	◯

* ED 1.4 La redevabilité des enseignants : Mesure dans laquelle les plans nationaux incluent des mesures spécifiques, dont le suivi et évaluation, visant à rendre les enseignants redevables de l'amélioration de l'apprentissage.

	1	2	3	4	5	6	7	8	9	10	Je ne sais pas
1-Plus faible -> Plus élevé - 10	◯	◯	◯	◯	◯	◯	◯	◯	◯	◯	◯

* ED 1.5 La qualité des enseignants : Mesure dans laquelle les politiques/stratégies/programmes d'éducation incluent des indicateurs vérifiables de la qualité des enseignants.

	1	2	3	4	5	6	7	8	9	10	Je ne sais pas
1-Plus faible -> Plus élevé - 10	◯	◯	◯	◯	◯	◯	◯	◯	◯	◯	◯

* ED 1.6 La collaboration dans l'éducation : Mesure dans laquelle la politique prévoit des dispositions pour engager plusieurs parties prenantes dans l'éducation (ex : écoles, communautés du milieu scolaire, parents, enseignants, gouvernement local, associations de parents d'élèves-élèves-enseignants, etc.).

	1	2	3	4	5	6	7	8	9	10	Je ne sais pas
1-Plus faible -> Plus élevé - 10	◯	◯	◯	◯	◯	◯	◯	◯	◯	◯	◯

* ED 1.7 L'ODD sur la parité : Mesure dans laquelle les politiques/plans/stratégies nationales tiennent compte de l'Objectif de Développement Durable (ODD) sur la parité totale à tous les niveaux d'enseignement.

	1	2	3	4	5	6	7	8	9	10	Je ne sais pas
1-Plus faible -> Plus élevé - 10	◯	◯	◯	◯	◯	◯	◯	◯	◯	◯	◯

* ED 1.8 Le financement de l'éducation : Mesure dans laquelle les nouveaux plans d'éducation incluent des propositions financières avec des budgets alloués aux réformes de l'enseignement et de l'apprentissage.

	1	2	3	4	5	6	7	8	9	10	Je ne sais pas
1-Plus faible -> Plus élevé - 10	◯	◯	◯	◯	◯	◯	◯	◯	◯	◯	◯

* ED 1.9 La planification de la parité : Mesure dans laquelle il existe des plans/stratégies nationales pour atteindre 100% d'inscriptions avec parité en :

	1	2	3	4	5	6	7	8	9	10	Je ne sais pas
ED 1.9.1 Ecole primaire	◯	◯	◯	◯	◯	◯	◯	◯	◯	◯	◯
ED 1.9.2 Ecole secondaire	◯	◯	◯	◯	◯	◯	◯	◯	◯	◯	◯
ED 1.9.3 Ecole tertiaire/supérieure	◯	◯	◯	◯	◯	◯	◯	◯	◯	◯	◯

* ED 1.10 L'analphabétisme chez l'adulte : Mesure dans laquelle des politiques/plans/stratégies nationales visent à répondre à l'analphabétisme.

	1	2	3	4	5	6	7	8	9	10	Je ne sais pas
ED 1.10.1 Mesure dans laquelle les politiques/plans/stratégies nationales visent à répondre à l'analphabétisme chez l'adulte.	◯	◯	◯	◯	◯	◯	◯	◯	◯	◯	◯
ED 1.10.2 Mesure dans laquelle les politiques/plans/stratégies nationales visant à répondre à l'analphabétisme chez l'adulte visent aussi à résorber les inégalités entre les sexes.	◯	◯	◯	◯	◯	◯	◯	◯	◯	◯	◯

* ED 1.11 Les enfants déscolarisés : Mesure dans laquelle des politiques répondent à la détresse des enfants déscolarisés pour les réintégrer au système scolaire ou leur offrir des opportunités alternatives de développement éducatif.

	1	2	3	4	5	6	7	8	9	10	Je ne sais pas
1-Plus faible -> Plus élevé - 10	◯	◯	◯	◯	◯	◯	◯	◯	◯	◯	◯

* ED 1.12 L'intégration de la technologie : Mesure dans laquelle des stratégies ont été développées et mises en œuvre/déployées pour intégrer les technologies de l'information et de la communication (TIC) à l'éducation.

	1	2	3	4	5	6	7	8	9	10	Je ne sais pas
1-Plus faible -> Plus élevé - 10	◯	◯	◯	◯	◯	◯	◯	◯	◯	◯	◯

* ED 1.13. La maîtrise des technologies : Mesure dans laquelle des ressources ont été allouées à l'amélioration de la maîtrise de l'informatique à tous les niveaux d'enseignement.

	1	2	3	4	5	6	7	8	9	10	Je ne sais pas
1-Plus faible -> Plus élevé - 10	○	○	○	○	○	○	○	○	○	○	○

ED 1.14. Commentaires : Utilisez cette section pour nous faire part de tout commentaire additionnel sur le niveau d'effort, les défis ou les succès du pays autour des politiques/ de la prise de décision dans le secteur de l'éducation. Si vous avez répondu « Je ne sais pas » à l'une des questions précédentes, veuillez utiliser cette section pour expliquer pourquoi.

JOHNS HOPKINS
BLOOMBERG SCHOOL
of PUBLIC HEALTH

Bill & Melinda Gates Institute for Population and Reproductive Health

Enquête de l'Indice des efforts pour le dividende démographique

Domaine 2. Services ou programmes

* ED 2.1 Le déploiement des enseignants : Mesure dans laquelle des stratégies/plans sont utilisés pour veiller à ce que les enseignants soient déployés dans les endroits du pays qui en ont le plus besoin.

	1	2	3	4	5	6	7	8	9	10	Je ne sais pas
1-Plus faible -> Plus élevé-10	◯	◯	◯	◯	◯	◯	◯	◯	◯	◯	◯

* ED 2.2 La qualité des enseignants : Mesure dans laquelle la qualité des enseignants est reconnue comme un facteur clé pour améliorer les résultats d'apprentissage.

	1	2	3	4	5	6	7	8	9	10	Je ne sais pas
ED 2.2.1 Mesure dans laquelle la qualité de l'enseignement est soulignée dans les politiques/ plans/ stratégies éducatives plus générales.	◯	◯	◯	◯	◯	◯	◯	◯	◯	◯	◯
ED 2.2.2 Mesure dans laquelle la qualité de la formation et du développement professionnel continu des enseignants est soulignée dans les politiques/ plans/ stratégies éducatives plus générales.	◯	◯	◯	◯	◯	◯	◯	◯	◯	◯	◯

* ED 2.3 La parité : Mesure dans laquelle les ressources ont été utilisées pour améliorer la parité dans l'éducation entre les garçons et les filles.

	1	2	3	4	5	6	7	8	9	10	Je ne sais pas
1-Plus faible -> Plus élevé-10	○	○	○	○	○	○	○	○	○	○	○

* ED 2.4 L'implication des parents dans l'éducation : Mesure dans laquelle les parents sont impliqués dans le développement et l'exécution des stratégies éducatives (c'est-à-dire à travers l'implication des comités de parents d'élèves et d'enseignants).

	1	2	3	4	5	6	7	8	9	10	Je ne sais pas
1-Plus faible -> Plus élevé-10	○	○	○	○	○	○	○	○	○	○	○

* ED 2.5 Les évaluations standardisées : Mesure dans laquelle toutes les écoles utilisent des évaluations standardisées des écoles et des élèves/étudiants en accord avec les instructions émises par le Ministère de l'Éducation.

	1	2	3	4	5	6	7	8	9	10	Je ne sais pas
1-Plus faible -> Plus élevé-10	○	○	○	○	○	○	○	○	○	○	○

* ED 2.6. L'utilisation des résultats d'évaluation pour informer le progrès : Mesure dans laquelle des ajustements sont apportés aux écoles en fonction des résultats des évaluations scolaires nationales standardisées.

	1	2	3	4	5	6	7	8	9	10	Je ne sais pas
1-Plus faible -> Plus élevé-10	○	○	○	○	○	○	○	○	○	○	○

ED 2.7 Commentaires : Veuillez utiliser cette section pour nous faire part de tout commentaire additionnel sur le niveau d'effort, les défis ou les succès du pays autour des services/ programmes dans le secteur de l'éducation. Si vous avez répondu « Je ne sais pas » à l'une des questions précédentes, veuillez utiliser cette section pour expliquer pourquoi.

JOHNS HOPKINS
BLOOMBERG SCHOOL
of PUBLIC HEALTH

Bill & Melinda Gates Institute for Population and Reproductive Health

Enquête de l'Indice des efforts pour le dividende démographique

Domaine 3. Plaidoyer

* ED 3.1 L'égalité : Mesure dans laquelle l'égalité et l'éducation équitable ont été soulignés par les plaideurs.

	1	2	3	4	5	6	7	8	9	10	Je ne sais pas
1-Plus faible -> Plus élevé-10	◯	◯	◯	◯	◯	◯	◯	◯	◯	◯	◯

* ED 3.2 La qualité : Mesure dans laquelle la qualité de l'éducation est intégrée aux évaluations liées au plaidoyer pour le système éducatif.

	1	2	3	4	5	6	7	8	9	10	Je ne sais pas
1-Plus faible -> Plus élevé-10	◯	◯	◯	◯	◯	◯	◯	◯	◯	◯	◯

* ED 3.3 Les obstacles des enseignants : Mesure dans laquelle les besoins des enseignants ont été mis en évidence par les plaideurs.

	1	2	3	4	5	6	7	8	9	10	Je ne sais pas
1-Plus faible -> Plus élevé-10	◯	◯	◯	◯	◯	◯	◯	◯	◯	◯	◯

* ED 3.4 Les obstacles des institutions : Mesure dans laquelle les difficultés des institutions éducatives ont été mises en évidence dans les différents efforts de plaidoyer.

	1	2	3	4	5	6	7	8	9	10	Je ne sais pas
1-Plus faible -> Plus élevé-10	◯	◯	◯	◯	◯	◯	◯	◯	◯	◯	◯

ED 3.5 Commentaires : Veuillez utiliser cette section pour nous faire part de tout commentaire additionnel sur le niveau d’effort, les défis ou les succès du pays autour du plaidoyer dans le secteur de l’éducation. Si vous avez répondu « Je ne sais pas » à l’une des questions précédentes, veuillez utiliser cette section pour expliquer pourquoi.

JOHNS HOPKINS
BLOOMBERG SCHOOL
of PUBLIC HEALTH

Bill & Melinda Gates Institute for Population and Reproductive Health

Enquête de l'Indice des efforts pour le dividende démographique

Domaine 4. Recherche

* ED 4.1 La force des observatoires/parties prenantes/groupes de travail pour l'ED : Mesure dans laquelle les parties prenantes du secteur de l'éducation du pays (ex : groupe directif de parties prenantes, groupes de travail techniques, observatoire de l'ED, groupe de coordination ou facilitation) :

	1	2	3	4	5	6	7	8	9	10	Je ne sais pas
ED 4.1.1 Ont un représentant du gouvernement, des institutions de formation, de la société civile, des organisations non gouvernementales et confessionnelles, des associations professionnelles, du secteur privé, etc.	○	○	○	○	○	○	○	○	○	○	○
ED 4.1.2 Se réunissent régulièrement, émettent des rapports et recommandent des politiques aux directions des ministères compétents.	○	○	○	○	○	○	○	○	○	○	○
ED 4.1.3 Ont un impact sur l'éducation dans le pays.	○	○	○	○	○	○	○	○	○	○	○

* ED 4.2 La stratégie d'éducation/recherche : Mesure dans laquelle le plan d'éducation nationale et/ou d'autres documents nationaux comprennent une stratégie/approche complète de recherche liée à l'éducation, partagée entre partenaires.

	1	2	3	4	5	6	7	8	9	10	Je ne sais pas
1-Plus faible -> Plus élevé-10	○	○	○	○	○	○	○	○	○	○	○

* ED 4.3 Les partenaires collectant les données :

	1	2	3	4	5	6	7	8	9	10	Je ne sais pas
ED 4.3.1 Mesure dans laquelle la collecte des données est entreprise par des agences gouvernementales. (Cela peut comprendre les agences/bureaux de la statistique et/ou les ministères de l'économie/finances, éducation, santé, genre, etc.)	◯	◯	◯	◯	◯	◯	◯	◯	◯	◯	◯
ED 4.3.2 Mesure dans laquelle la collecte de données est entreprise par des institutions de recherche.	◯	◯	◯	◯	◯	◯	◯	◯	◯	◯	◯
ED 4.3.3 Mesure dans laquelle la collecte des données est entreprise par des chercheurs indépendants.	◯	◯	◯	◯	◯	◯	◯	◯	◯	◯	◯

* ED 4.4 La recherche thématique :

	1	2	3	4	5	6	7	8	9	10	Je ne sais pas
ED 4.4.1 Mesure dans laquelle de la recherche est menée sur les inégalités de genre dans l'éducation.	◯	◯	◯	◯	◯	◯	◯	◯	◯	◯	◯
ED 4.4.2 Mesure dans laquelle de la recherche est menée sur la qualité de l'enseignement.	◯	◯	◯	◯	◯	◯	◯	◯	◯	◯	◯
ED 4.4.3 Mesure dans laquelle de la recherche est menée sur les obstacles auxquels font face les enseignants.	◯	◯	◯	◯	◯	◯	◯	◯	◯	◯	◯
ED 4.4.4 Mesure dans laquelle de la recherche est menée sur les obstacles auxquels font face les institutions.	◯	◯	◯	◯	◯	◯	◯	◯	◯	◯	◯

* ED 4.5 La qualité/couverture des données : Mesure dans laquelle la recherche/les données actuelles sont désagrégées par sexe et par âge. Mesure dans laquelle le pays participe aux tests standardisés et son classement.

	1	2	3	4	5	6	7	8	9	10	Je ne sais pas
ED 4.5.1 Mesure dans laquelle un système statistique de routine (utilisant les informations des écoles/institutions) offre de bonnes informations régulières sur les produits et les établissements	○	○	○	○	○	○	○	○	○	○	○
ED 4.5.2 Mesure dans laquelle un système statistique de routine (utilisant les informations des écoles/institutions) offre de bonnes informations régulières sur les services et le personnel du secteur éducatif	○	○	○	○	○	○	○	○	○	○	○
ED 4.5.3 Mesure dans laquelle un système statistique de routine offre de bonnes informations régulières sur les besoins éducatifs des populations de différentes communautés	○	○	○	○	○	○	○	○	○	○	○
ED 4.5.4 Mesure dans laquelle des évaluations du système de suivi de l’éducation sont menées et appliquées pour assurer la fiabilité des données	○	○	○	○	○	○	○	○	○	○	○

* ED 4.6 Les registres : Mesure dans laquelle les systèmes de registres, rapports et feedback sur les résultats de l’éducation sont adéquats.

	1	2	3	4	5	6	7	8	9	10	Je ne sais pas
1-Plus faible -> Plus élevé-10	○	○	○	○	○	○	○	○	○	○	○

* ED 4.7 La qualité des institutions de recherche : Mesure dans laquelle le pays a la capacité de soutenir et maintenir des institutions de recherche qui développent des études et/ou collectent des données permettant d'informer les efforts pour l'amélioration de l'éducation/ l'enseignement.

	1	2	3	4	5	6	7	8	9	10	Je ne sais pas
1-Plus faible -> Plus élevé-10	○	○	○	○	○	○	○	○	○	○	○

* ED 4.8 L'évaluation : Mesure dans laquelle les statistiques, enquêtes et études sur le programme d'éducation sont utilisées par du personnel spécialisé pour rapporter les opérations du programme et mesurer le progrès.

	1	2	3	4	5	6	7	8	9	10	Je ne sais pas
1-Plus faible -> Plus élevé-10	○	○	○	○	○	○	○	○	○	○	○

* ED 4.9 L'utilisation des résultats d'évaluation par les gestionnaires de programmes : Mesure dans laquelle les gestionnaires locaux du programme d'éducation utilisent les conclusions de recherche et d'évaluations pour améliorer ces programmes en suivant les recommandations émises.

	1	2	3	4	5	6	7	8	9	10	Je ne sais pas
1-Plus faible -> Plus élevé - 10	○	○	○	○	○	○	○	○	○	○	○

* ED 4.10 L'utilisation des résultats d'évaluation par les ministères : Mesure dans laquelle les administrateurs ministériels compétents (soit le Ministère de l'Éducation) utilisent systématiquement les données pour informer les politiques et interventions visant à résoudre les problèmes éducatifs.

	1	2	3	4	5	6	7	8	9	10	Je ne sais pas
1-Plus faible -> Plus élevé-10	○	○	○	○	○	○	○	○	○	○	○

* ED 4.11 La dissémination d'informations à d'autres entités chargées de la mise en œuvre des programmes :

	1	2	3	4	5	6	7	8	9	10	Je ne sais pas
ED 4.11.1 Mesure dans laquelle des informations sont partagées ou disséminées entres géographies/ régions.	○	○	○	○	○	○	○	○	○	○	○
ED. 4.11.2 Mesure dans laquelle des informations sont partagées ou disséminées à différents niveaux (national, états/provinces, localités).	○	○	○	○	○	○	○	○	○	○	○

ED 4.12 Commentaires : Veuillez utiliser cette section pour nous faire part de tout commentaire additionnel sur le niveau d'effort, les défis ou les succès du pays autour de la recherche dans le secteur de l'éducation. Si vous avez répondu « Je ne sais pas » à l'une des questions précédentes, veuillez utiliser cette section pour expliquer pourquoi.

JOHNS HOPKINS BLOOMBERG SCHOOL of PUBLIC HEALTH

Bill & Melinda Gates Institute for Population and Reproductive Health

Enquête de l'Indice des efforts pour le dividende démographique

Domaine 5. Organisations de la société civile (OSC)

* ED 5.1 Le pouvoir des acteurs des OSC : Mesure dans laquelle les acteurs des OSC occupent des postes d'influence dans les institutions, les groupes de travail techniques ou les réseaux concernés par l'éducation.

	1	2	3	4	5	6	7	8	9	10	Je ne sais pas
1-Plus faible -> Plus élevé-10	○	○	○	○	○	○	○	○	○	○	○

* ED 5.2 L'analyse budgétaire comme outil des OSC : Mesure dans laquelle les OSC utilisent l'analyse budgétaire comme un outil pour développer le plaidoyer en matière d'éducation.

	1	2	3	4	5	6	7	8	9	10	Je ne sais pas
1-Plus faible -> Plus élevé-10	○	○	○	○	○	○	○	○	○	○	○

* ED 5.3 L'inclusion communautaire : Mesure dans laquelle les OSC sont impliquées dans les efforts d'exploration d'opportunités pour l'amélioration de la qualité de l'éducation.

	1	2	3	4	5	6	7	8	9	10	Je ne sais pas
1-Plus faible -> Plus élevé-10	○	○	○	○	○	○	○	○	○	○	○

* ED 5.4 L'implication de la communauté dans l'éducation : Mesure dans laquelle les OSC participent au rapprochement des informations et services éducatifs avec les communautés à travers des activités telles que le renforcement des compétences des enseignants et des parents et/ou les campagnes d'information ou d'éducation pour augmenter l'engagement des familles et des communautés en faveur de l'éducation.

	1	2	3	4	5	6	7	8	9	10	Je ne sais pas
1-Plus faible -> Plus élevé-10	○	○	○	○	○	○	○	○	○	○	○

* ED 5.5 La franchise sociale : Mesure dans laquelle les OSC sont impliquées dans la franchise sociale des services/opportunités éducatives de qualité aux familles et enfants à moindre coût.

	1	2	3	4	5	6	7	8	9	10	Je ne sais pas
1-Plus faible -> Plus élevé-10	○	○	○	○	○	○	○	○	○	○	○

* ED 5.6 La technologie : Mesure dans laquelle les OSC mettent à profit les technologies de l'information et de la communication (TIC) pour améliorer l'accès à l'éducation de qualité de diverses communautés.

	1	2	3	4	5	6	7	8	9	10	Je ne sais pas
1-Plus faible -> Plus élevé-10	○	○	○	○	○	○	○	○	○	○	○

* ED 5.7 Les droits humains, l'accès et la qualité de l'éducation : Mesure dans laquelle les OSC plaident pour le soutien d'approches éducatives basées sur les droits et veillent à ce que les élèves/étudiants soit traités correctement, n'aient pas peur de poser des questions, et reçoivent un enseignement de qualité, entre autres préoccupations.

	1	2	3	4	5	6	7	8	9	10	Je ne sais pas
1-Plus faible -> Plus élevé-10	○	○	○	○	○	○	○	○	○	○	○

* ED 5.8 Le changement social et des comportements (CSC) : Mesure dans laquelle les OSC soutiennent les interventions pour le CSC comprenant, mais sans s'y limiter, aux compétences de vie et à l'éducation entre pairs, l'éducation sur le santé de l'adulte, l'éducation en milieu scolaire, la mobilisation communautaire et/ou les interventions médiatiques destinées au grand public.

	1	2	3	4	5	6	7	8	9	10	Je ne sais pas
1-Plus faible -> Plus élevé-10	○	○	○	○	○	○	○	○	○	○	○

* ED 5.9 Le plaidoyer/ la redevabilité : Mesure dans laquelle les OSC soutiennent la formation et le renforcement de compétences des plaideurs et des membres de la communauté pour améliorer leur compréhension des politiques et processus, leurs activités d'identification de problèmes, la résolution collaborative de ces problèmes, et des actions de plaidoyer plus ciblées pour améliorer l'éducation.

	1	2	3	4	5	6	7	8	9	10	Je ne sais pas
1-Plus faible -> Plus élevé-10	○	○	○	○	○	○	○	○	○	○	○

* ED 5.10 L'évaluation et le suivi dirigés par les OSC : Mesure dans laquelle les OSC évaluent, suivent et émettent des rapports sur l'efficacité des politiques et programmes pour améliorer la redevabilité des enseignants et des politiques d'éducation.

	1	2	3	4	5	6	7	8	9	10	Don't know
1-Plus faible -> Plus élevé-10	◯	◯	◯	◯	◯	◯	◯	◯	◯	◯	◯

* ED 5.11 Les partenariats entre OSC : Mesure dans laquelle les OSC ont formé des alliances nationales et des partenariats régionaux pour renforcer leur position et potentiellement améliorer la force du leadership et les mécanismes de financement en vue d'améliorer la qualité de l'éducation.

	1	2	3	4	5	6	7	8	9	10	Je ne sais pas
1-Plus faible -> Plus élevé-10	◯	◯	◯	◯	◯	◯	◯	◯	◯	◯	◯

ED 5.12 Commentaires : Veuillez utiliser cette section pour nous faire part de tout commentaire additionnel sur le niveau d'effort, les défis ou les succès du pays autour des organisations de la société civile impliquées dans le secteur de l'éducation. Si vous avez répondu « Je ne sais pas » à l'une des questions précédentes, veuillez utiliser cette section pour expliquer pourquoi.

JOHNS HOPKINS
BLOOMBERG SCHOOL
of PUBLIC HEALTH

Bill & Melinda Gates Institute for Population and Reproductive Health

Enquête de l'Indice des efforts pour le dividende démographique

Module : Résilience et durabilité du secteur

Étant donné l'impact sanitaire et socioéconomique de la pandémie de COVID-19, et son impact probable sur la progression vers le DD, cet indice des efforts pour le DD intègre des questions pour évaluer la résilience et la durabilité des systèmes dans les secteurs clés du DD. Les éléments couverts par les questions liées à la pandémie de COVID-19 ci-dessous relèvent des dimensions clés des systèmes résilients et visent à apporter des informations déterminantes sur le potentiel d'une réponse efficace aux menaces de maladies infectieuses émergeantes et autres crises de santé publique.

Veuillez noter chaque élément sur une échelle de 1 à 10, 1 étant le score le plus faible (faible état/capacité) et 10 le plus élevé (très bon état/capacité).

* Dimension physique : structures, équipements, états du système et capacités

	1	2	3	4	5	6	7	8	9	10	Je ne sais pas
ED-M1 - Planification/Préparation – État et capacité de l'équipement, du personnel et de la structure du secteur de l'ED avant la crise. (1 = faible état/capacité; 10 = très bon état/capacité)	○	○	○	○	○	○	○	○	○	○	○
ED-M2 - Absorption – Mesure dans laquelle la pandémie de COVID-19 a été reconnue par le secteur de l'ED, et mesure dans laquelle le système a pu continuer d'être fonctionnel	○	○	○	○	○	○	○	○	○	○	○

	1	2	3	4	5	6	7	8	9	10	Je ne sais pas
ED-M3 - Absorption – Intégration : Niveau d'intégration des services et programmes d'ED pour mitiger les effets de la pandémie de COVID-19 sur l'ED.	◯	◯	◯	◯	◯	◯	◯	◯	◯	◯	◯
ED-M4 - Récupération – Mesure dans laquelle le secteur de l'ED a été capable d'induire des changements pour récupérer un niveau de fonctionnalité pré-COVID-19.	◯	◯	◯	◯	◯	◯	◯	◯	◯	◯	◯
ED-M5 - Récupération – Gestion du financement et des bailleurs de fonds : Niveau de mobilisation des ressources et allocations pour mitiger les effets de la pandémie de COVID-19 sur l'ED.	◯	◯	◯	◯	◯	◯	◯	◯	◯	◯	◯
ED-M6 - Adaptation – Mesure dans laquelle des changements ont été effectués pour améliorer la résilience du secteur de l'ED.	◯	◯	◯	◯	◯	◯	◯	◯	◯	◯	◯

* Information : création, gestion, stockage et utilisation des données.

	1	2	3	4	5	6	7	8	9	10	Je ne sais pas
ED-M7 - Planification/ Préparation – Mesure dans laquelle les données liées à l'ED étaient suffisamment préparées, présentées, analysées et stockées avant la crise.	○	○	○	○	○	○	○	○	○	○	○
ED-M8 - Absorption - Knowledge: Extent to which the monitoring capacity was able to detect the limits / threats posed to the ED sector by the COVID-19 pandemic. (This includes data on the various key subjects of the education sector at each level of education [primary, secondary, tertiary / higher]).	○	○	○	○	○	○	○	○	○	○	○
ED-M9 - Récupération – Mesure dans laquelle les données de l'ED ont été utilisées pour suivre le progrès vers la récupération et anticiper des scenarii de récupération.	○	○	○	○	○	○	○	○	○	○	○
ED-M10 - Adaptation – Mesure dans laquelle le système d'ED crée et améliore actuellement ses protocoles de stockage et d'utilisation de ses données en temps réel.	○	○	○	○	○	○	○	○	○	○	○

* Dimension cognitive : compréhension, modèles cognitifs, idées reçues, préjugés et valeurs.

	1	2	3	4	5	6	7	8	9	10	Je ne sais pas
ED-M11 – Planification/ Préparation – Mesure dans laquelle le système d'ED et les décisions opérationnelles étaient préparés pour anticiper la crise.	○	○	○	○	○	○	○	○	○	○	○

	1	2	3	4	5	6	7	8	9	10	Je ne sais pas
ED-M12 - Absorption – Mesure dans laquelle la réponse du système d'ED a des protocoles de contingence suffisants et une gestion de crise proactive.	○	○	○	○	○	○	○	○	○	○	○
ED-M13 - Absorption – Redevabilité : Niveau d'effort pour assurer une redevabilité optimale vis-à-vis des ressources allouées à l'ED dans le cadre de la réponse à la pandémie de COVID-19.	○	○	○	○	○	○	○	○	○	○	○
ED-M14 - Récupération – Mesure dans laquelle les décisions sont orientées sur la récupération et communiquées aux communautés sur la base de données probantes pour promouvoir des comportements sûrs.	○	○	○	○	○	○	○	○	○	○	○
ED-M15 - Récupération – Connaissance : Mesure dans laquelle le secteur de l'ED établit une certaine confiance à travers sa communication concernant les barrières posées par la pandémie de COVID-19 pour soutenir l'accès et l'utilisation des services et programmes d'ED.	○	○	○	○	○	○	○	○	○	○	○
ED-M16 - Adaptation – Mesure dans laquelle le secteur de l'ED conçoit de nouvelles configurations systémiques, de nouveaux objectifs et de nouveaux critères de décision.	○	○	○	○	○	○	○	○	○	○	○

	1	2	3	4	5	6	7	8	9	10	Je ne sais pas
ED-M17 – Adaptation : Niveau d'adaptation pour répondre aux menaces posées par la pandémie de COVID-19 à l'ED.	○	○	○	○	○	○	○	○	○	○	○

* Social dimension: interaction, collaboration and self-synchronization between people, entities and institutions.

	1	2	3	4	5	6	7	8	9	10	Je ne sais pas
ED-M18 – Planification /Préparation – Mesure dans laquelle une formation sur la gestion d'épidémies/crises a été menée et a permis de mettre à profit les réseaux sociaux, le capital social et les normes institutionnelles et culturelles avant la crise.	○	○	○	○	○	○	○	○	○	○	○
ED-M19 - Absorption – Mesure dans laquelle le personnel et les institutions sociales ont été accessibles et ont fait preuve d'ingéniosité dans leur réponse à l'épidémie/crise.	○	○	○	○	○	○	○	○	○	○	○
ED-M20 - Absorption - Self-regulation: Extent to which national leaders had the authority to effect timely sectoral changes through an infrastructure flexible.	○	○	○	○	○	○	○	○	○	○	○
ED-M21 – Récupération – Mesure dans laquelle le système d'ED est impliqué dans le partage de connaissances et d'équipes pour améliorer la récupération du système.	○	○	○	○	○	○	○	○	○	○	○

	1	2	3	4	5	6	7	8	9	10	Je ne sais pas
ED-M22 - Récupération – Diversité : Niveau d'engagement d'une équipe multidisciplinaire pour mitiger les effets de la pandémie de COVID-19 sur l'ED.	○	○	○	○	○	○	○	○	○	○	○
ED-M23 - Adaptation – Mesure dans laquelle des ajouts ou changements sont apportés aux institutions, politiques, formations, programmes et à la culture de l'ED.	○	○	○	○	○	○	○	○	○	○	○
ED-M24 - Adaptation - Leadership et management : Niveau de leadership démontré par les dirigeants du secteur de l'ED pour mitiger les effets de la pandémie de COVID-19 sur l'ED.	○	○	○	○	○	○	○	○	○	○	○

* ED M-25 Temporalité : Mesure de la rapidité de la réaction du gouvernement –sa communication et mise en œuvre des mesures- pour mitiger l'impact immédiat et sur le long terme de la pandémie de COVID-19. Cette question porte sur la riposte nationale dans son ensemble, et non la réponse spécifique de votre secteur.

	1	2	3	4	5	6	7	8	9	10	Je ne sais pas
1-Plus faible -> Plus élevé-10	○	○	○	○	○	○	○	○	○	○	○

ED M-26 Commentaires : Utilisez cette section pour apporter tout commentaire additionnel sur la résilience et la durabilité du système d'éducation nationale en vous référant à votre expérience de la pandémie de COVID-19. Si vous avez répondu « Je ne sais pas » à l'une des questions, utilisez cette section pour expliquer pourquoi.

JOHNS HOPKINS
BLOOMBERG SCHOOL
of PUBLIC HEALTH

Bill & Melinda Gates Institute for Population and Reproductive Health

Enquête de l'Indice des efforts pour le dividende démographique

Questionnaire sur l'autonomisation des femmes (AF)

Répondez aux questions suivantes en fonction de votre expérience/ expertise dans ce secteur spécifique.

Afin d'obtenir une représentation synthétique des efforts nationaux pour le dividende démographique, notez les éléments suivants sur une échelle de 1 à 10, 1 étant le score le plus faible (efforts très faibles ou quasi-inexistants) et 10 le plus élevé (efforts robustes). Le cas échéant, si une politique ou activité n'existe pas, répondez 0.

Donnez un score à chaque élément. Toutes les réponses seront enregistrées au format illustré ci-dessous :

Effort le plus faible	1	2	3	4	5	6	7	8	9	10	Effort le plus élevé	Je ne sais pas

Bill & Melinda Gates Institute for Population and Reproductive Health

Enquête de l'Indice des efforts pour le dividende démographique

Domaine 1. Politique/ Prise de décision

* WE 1.1 La politique de parité : Mesure dans laquelle la parité est prise en compte/intégrée aux plans de développement du pays (ou aux plans de votre secteur).

	1	2	3	4	5	6	7	8	9	10	Je ne sais pas
1-Plus faible -> Plus élevé-10	◯	◯	◯	◯	◯	◯	◯	◯	◯	◯	◯

* WE 1.2 Niveau d'engagement envers la Convention sur l'élimination de toutes les formes de discrimination à l'égard des femmes (CEDAW) :

	1	2	3	4	5	6	7	8	9	10	Je ne sais pas
1-Plus faible -> Plus élevé-10	◯	◯	◯	◯	◯	◯	◯	◯	◯	◯	◯

* WE 1.3 La parité dans les politiques familiales et de mariage : Mesure dans laquelle le principe d'égalité hommes-femmes est incorporé à la constitution nationale et autres législations concernant le mariage et les relations familiales.

	1	2	3	4	5	6	7	8	9	10	Je ne sais pas
1-Plus faible -> Plus élevé-10	◯	◯	◯	◯	◯	◯	◯	◯	◯	◯	◯

* WE 1.4 L'âge légal du mariage : Mesure dans laquelle l'âge légal du mariage pour les filles est établi à 18 ans ou plus et est appliqué.

	1	2	3	4	5	6	7	8	9	10	Je ne sais pas
1-Plus faible -> Plus élevé-10	◯	◯	◯	◯	◯	◯	◯	◯	◯	◯	◯

* WE 1.5 Les mesures contre les violences à l'égard des femmes : Niveau des régulations et mesures contre les violences faites aux femmes, dont la violence intraconjugale, le viol, le harcèlement sexuel et la traite des filles et des femmes.

	1	2	3	4	5	6	7	8	9	10	Je ne sais pas
1-Plus faible -> Plus élevé-10	◯	◯	◯	◯	◯	◯	◯	◯	◯	◯	◯

* WE 1.6 La mutilation génitale féminine : Niveau de régulation et mesures contre la mutilation génitale féminine.

	1	2	3	4	5	6	7	8	9	10	Je ne sais pas
1-Plus faible -> Plus élevé-10	◯	◯	◯	◯	◯	◯	◯	◯	◯	◯	◯

* WE 1.7 Les mesures de santé des femmes : Niveau des protocoles de protection de la santé des femmes, dont les infections sexuellement transmissibles, y compris le VIH/SIDA, la mortalité maternelle et la contraception.

	1	2	3	4	5	6	7	8	9	10	Je ne sais pas
1-Plus faible -> Plus élevé-10	◯	◯	◯	◯	◯	◯	◯	◯	◯	◯	◯

* WE 1.8 Les mesures éducatives : Mesure dans laquelle il existe des mécanismes pour promouvoir l'éducation des filles.

	1	2	3	4	5	6	7	8	9	10	Je ne sais pas
WE 1.8.1 Niveau d'application des politiques sur l'éducation des filles.	◯	◯	◯	◯	◯	◯	◯	◯	◯	◯	◯
WE 1.8.2 Mesure dans laquelle des mécanismes sont en place pour prévenir l'échec/l'abandon scolaire des filles.	◯	◯	◯	◯	◯	◯	◯	◯	◯	◯	◯

* WE 1.9 Les mesures relatives à l'exercice des droits : Niveau d'application des politiques sur les droits des femmes.

	1	2	3	4	5	6	7	8	9	10	Je ne sais pas
1-Plus faible -> Plus élevé-10	◯	◯	◯	◯	◯	◯	◯	◯	◯	◯	◯

* WE 1.10 Le travail équitable : Mesure dans laquelle il existe des politiques sur les conditions de travail équitables et l'égalité des salaires entre hommes et femmes.

	1	2	3	4	5	6	7	8	9	10	Je ne sais pas
1-Plus faible -> Plus élevé-10	○	○	○	○	○	○	○	○	○	○	○

* WE 1.11 L'égalité de la propriété de biens/biens fonciers : Mesure dans laquelle il existe des politiques pour affirmer l'accès équitable à la propriété de biens fonciers.

	1	2	3	4	5	6	7	8	9	10	Je ne sais pas
1-Plus faible -> Plus élevé-10	○	○	○	○	○	○	○	○	○	○	○

* WE 1.12 Le conflit, les femmes et le rétablissement de la paix : Niveau de mise en œuvre de la Résolution 1325 du Conseil de sécurité des Nations Unies sur les impacts des conflits sur les femmes et leur rôle dans le rétablissement de la paix.

	1	2	3	4	5	6	7	8	9	10	Je ne sais pas
1-Plus faible -> Plus élevé-10	○	○	○	○	○	○	○	○	○	○	○

* WE 1.13 Les quotas de femmes dans les politiques : Existence et niveau de mise en œuvre des quotas dans les systèmes politiques observant la parité.

	1	2	3	4	5	6	7	8	9	10	Je ne sais pas
WE 1.13.1 Mesure dans laquelle il existe une prise de décision équitable du point de vue du genre, y compris en soutien au quotas électoraux et à l'intégration des femmes dans tous les ministères et départements du gouvernement.	○	○	○	○	○	○	○	○	○	○	○
WE 1.13.2 Mesure dans laquelle les femmes participant et/ou jouent un rôle significatif dans les systèmes traditionnels de gouvernance (ex : chefs de village, etc.).	○	○	○	○	○	○	○	○	○	○	○

* WE 1.14 L'Objectif de Développement Durable (ODD) 5 : Niveau d'engagement pour remplir l'Objectif 5 des ODD, pour réaliser la parité et assurer l'autonomisation de toutes les filles et femmes et les filles.

	1	2	3	4	5	6	7	8	9	10	Je ne sais pas
1-Plus faible -> Plus élevé-10	◯	◯	◯	◯	◯	◯	◯	◯	◯	◯	◯

* WE 1.15 La révision de la loi coutumière : Mesure dans laquelle les lois coutumières ont été revues et modifiées par des politiques/cadres nationaux ; ou mesure dans laquelle les lois coutumières existantes reflètent les efforts pour l'autonomisation des femmes.

	1	2	3	4	5	6	7	8	9	10	Je ne sais pas
1-Plus faible -> Plus élevé-10	◯	◯	◯	◯	◯	◯	◯	◯	◯	◯	◯

* WE 1.16 La budgétisation pour l'autonomisation des femmes : Mesure dans laquelle la budgétisation et l'allocation budgétaire sont suffisantes pour couvrir les coûts nécessaires à l'atteinte des cibles établies dans le plan national de la femme ou son équivalent.

	1	2	3	4	5	6	7	8	9	10	Je ne sais pas
1-Plus faible -> Plus élevé-10	◯	◯	◯	◯	◯	◯	◯	◯	◯	◯	◯

* WE 1.17 Les ressources humaines pour l'autonomisation des femmes : Mesure dans laquelle :

	1	2	3	4	5	6	7	8	9	10	Je ne sais pas
WE 1.17.1 Du personnel en quantité et qualité suffisante est employé pour mettre en œuvre les activités liées au genre, y compris les efforts pour former ce personnel	◯	◯	◯	◯	◯	◯	◯	◯	◯	◯	◯
WE 1.17.2 Des efforts sont menés pour former le personnel de manière appropriée et intégrale.	◯	◯	◯	◯	◯	◯	◯	◯	◯	◯	◯
WE 1.17.3 Le personnel est suffisamment soutenu pour mener à bien ses tâches.	◯	◯	◯	◯	◯	◯	◯	◯	◯	◯	◯
WE 1.17.4 Le personnel assigné est situé à un niveau administratif suffisamment élevé pour travailler efficacement.	◯	◯	◯	◯	◯	◯	◯	◯	◯	◯	◯

* WE 1.18 La transparence et la redevabilité : Mesure dans laquelle le gouvernement fournit aux parties prenantes des informations complètes sur les intentions, plans, programmes et budgets disponibles pour mettre en œuvre les programmes d'autonomisation des femmes et en assurer la redevabilité et la transparence. (*Peuvent être incluses l'adoption et l'application d'une loi sur le droit à l'information, des sessions informatives régulières, par exemple dans les mairies, ou la transmission de ces informations via des brochures ou autres supports de communication en langue locale*).

	1	2	3	4	5	6	7	8	9	10	Je ne sais pas
1-Plus faible -> Plus élevé-10	◯	◯	◯	◯	◯	◯	◯	◯	◯	◯	◯

WE 1.19 Commentaire : Utilisez cette section pour apporter des commentaires additionnels sur le niveau d'efforts, les défis et les succès du pays autour de la politique/ prise de décision pour l'autonomisation des femmes. Si vous avez répondu « Je ne sais pas » à l'une des questions ci-dessus, utilisez aussi cette section pour expliquer pourquoi.

JOHNS HOPKINS
BLOOMBERG SCHOOL of PUBLIC HEALTH

Bill & Melinda Gates Institute for Population and Reproductive Health

Enquête de l'Indice des efforts pour le dividende démographique

Domaine 2. Services ou programmes

* WE 2.1 L'intégration des femmes : Mesure dans laquelle les services ou programmes sectoriels et intersectoriels tiennent compte des implications des actions planifiées pour les hommes et pour les femmes.

	1	2	3	4	5	6	7	8	9	10	Je ne sais pas
1-Plus faible -> Plus élevé-10	○	○	○	○	○	○	○	○	○	○	○

* WE 2.2 Les programmes transformateurs de genre : Mesure dans laquelle les services ou programmes sectoriels ou intersectoriels intègrent des principes/ approches transformateurs de genre.

	1	2	3	4	5	6	7	8	9	10	Je ne sais pas
1-Plus faible -> Plus élevé-10	○	○	○	○	○	○	○	○	○	○	○

* WE 2.3 L'éducation : Mesure dans laquelle les services ou programmes éducatifs se focalisent sur l'autonomisation des filles et des femmes dans les salles de classe.

	1	2	3	4	5	6	7	8	9	10	Je ne sais pas
1-Plus faible -> Plus élevé-10	○	○	○	○	○	○	○	○	○	○	○

* WE 2.4. L'abandon scolaire : Mesure dans laquelle il existe des programmes pour prévenir l'abandon scolaire des filles.

	1	2	3	4	5	6	7	8	9	10	Je ne sais pas
1-Plus faible -> Plus élevé-10	○	○	○	○	○	○	○	○	○	○	○

* WE 2.5 La maîtrise des technologies : Mesure dans laquelle la maîtrise des technologies est promue auprès des filles en particulier.

	1	2	3	4	5	6	7	8	9	10	Je ne sais pas
1-Plus faible -> Plus élevé-10	○	○	○	○	○	○	○	○	○	○	○

* WE 2.6 L'emploi : Mesure dans laquelle les programmes de création d'emploi et les politiques du marché du travail visent à garantir l'indépendance économique des femmes et sont conçus/mis en œuvre en conséquence.

	1	2	3	4	5	6	7	8	9	10	Je ne sais pas
1-Plus faible -> Plus élevé-10	○	○	○	○	○	○	○	○	○	○	○

* WE 2.7 Les femmes-mères-travailleuses : Mesure dans laquelle le gouvernement permet aux femmes, à travers la loi, les régulations et les programmes, de combiner leur rôle de mère pendant la grossesse, l'allaitement et l'éducation de leurs enfants à leur participation à la vie active.

	1	2	3	4	5	6	7	8	9	10	Je ne sais pas
1-Plus faible -> Plus élevé-10	○	○	○	○	○	○	○	○	○	○	○

* WE 2.8 La planification familiale pour l'autonomisation des femmes : Mesure dans laquelle les services ou programmes de PF sont mis en œuvre selon une approche fondamentale d'autonomisation des femmes.

	1	2	3	4	5	6	7	8	9	10	Je ne sais pas
1-Plus faible -> Plus élevé-10	○	○	○	○	○	○	○	○	○	○	○

* WE 2.9 La représentation politique et publique : Mesure de la mise en œuvre des programmes visant à améliorer la participation et la représentation équitables des femmes à tous les niveaux du processus politique et de la vie publique.

	1	2	3	4	5	6	7	8	9	10	Je ne sais pas
1-Plus faible -> Plus élevé-10	○	○	○	○	○	○	○	○	○	○	○

* WE 2.10 La violence : Portée de la lutte contre les violences faites à l'égard des femmes et des enfants, et leur élimination à l'échelle du pays (violences intraconjugales, viol, harcèlement sexuel, traite des femmes).

	1	2	3	4	5	6	7	8	9	10	Je ne sais pas
1-Plus faible -> Plus élevé-10	○	○	○	○	○	○	○	○	○	○	○

* WE 2.11 Le mariage d’enfants : Mesure de l’application des lois pour éliminer le mariage d’enfants.

	1	2	3	4	5	6	7	8	9	10	Je ne sais pas
1-Plus faible -> Plus élevé-10	◯	◯	◯	◯	◯	◯	◯	◯	◯	◯	◯

* WE 2.12 La mutilation génitale féminine : Mesure de l’application des lois pour éliminer la mutilation génitale féminine.

	1	2	3	4	5	6	7	8	9	10	Je ne sais pas
1-Plus faible -> Plus élevé-10	◯	◯	◯	◯	◯	◯	◯	◯	◯	◯	◯

WE 2.13 Commentaire : Utilisez cette section pour apporter des commentaires additionnels sur le niveau d’efforts, les défis et les succès du pays autour des services et programmes pour l’autonomisation des femmes. Si vous avez répondu « Je ne sais pas » à l’une des questions ci-dessus, utilisez aussi cette section pour expliquer pourquoi.

JOHNS HOPKINS
BLOOMBERG SCHOOL
of PUBLIC HEALTH

Bill & Melinda Gates Institute for Population and Reproductive Health

Enquête de l’Indice des efforts pour le dividende démographique

Domaine 3. Plaidoyer

* WE 3.1 Les plaideurs : Mesure dans laquelle des plaideurs pour l’autonomisation des filles et des femmes existent au niveau national et infranational.

	1	2	3	4	5	6	7	8	9	10	Je ne sais pas
1-Plus faible -> Plus élevé-10	◯	◯	◯	◯	◯	◯	◯	◯	◯	◯	◯

* WE 3.2. Les filles et les femmes : Mesure dans laquelle les efforts de plaidoyer dans ce domaine recouvrent l’ensemble des aspects de l’autonomisation des filles et des femmes.

	1	2	3	4	5	6	7	8	9	10	Je ne sais pas
1-Plus faible -> Plus élevé-10	◯	◯	◯	◯	◯	◯	◯	◯	◯	◯	◯

* WE 3.3 La dissémination de l’information : Mesure dans laquelle le gouvernement entreprend suffisamment d’efforts pour veiller à ce que la population soit consciente des problèmes liés à l’autonomisation des femmes, et si les informations disséminées sont exactes et communiquées dans la langue parlée par les destinataires/publics cibles.

	1	2	3	4	5	6	7	8	9	10	Je ne sais pas
1-Plus faible -> Plus élevé-10	◯	◯	◯	◯	◯	◯	◯	◯	◯	◯	◯

* WE 3.4 La mise à profit des partenariats : Mesure dans laquelle le gouvernement met à profit ses partenariats avec des OSC pour disséminer des informations sur l’autonomisation des femmes à travers des campagnes de sensibilisation/information.

	1	2	3	4	5	6	7	8	9	10	Je ne sais pas
1-Plus faible -> Plus élevé-10	◯	◯	◯	◯	◯	◯	◯	◯	◯	◯	◯

* WE 3.5 L’emploi : Mesure dans laquelle les parties prenantes appellent à l’élaboration de lois, politiques et programmes pour que les femmes puissent combiner leur rôle de mère pendant la grossesse, l’allaitement et l’éducation de leurs enfants avec leur participation à la vie active.

	1	2	3	4	5	6	7	8	9	10	Je ne sais pas
1-Plus faible -> Plus élevé-10	○	○	○	○	○	○	○	○	○	○	○

* WE 3.6 L’éducation : Mesure dans laquelle l’une des priorités des plaideurs est de promouvoir la réalisation du plein potentiel des filles et des femmes à travers l’école et le développement de compétences.

	1	2	3	4	5	6	7	8	9	10	Je ne sais pas
1-Plus faible -> Plus élevé-10	○	○	○	○	○	○	○	○	○	○	○

* WE 3.7 La violence : Mesure dans laquelle les plaideurs réussissent à interpeller le gouvernement pour le faire réagir et répondre aux cas de violence et de discrimination contre les femmes.

	1	2	3	4	5	6	7	8	9	10	Je ne sais pas
1-Plus faible -> Plus élevé-10	○	○	○	○	○	○	○	○	○	○	○

WE 3.8 Commentaire : Utilisez cette section pour apporter des commentaires additionnels sur le niveau d’efforts, les défis et les succès du pays autour du plaidoyer pour l’autonomisation des femmes. Si vous avez répondu « Je ne sais pas » à l’une des questions ci-dessus, utilisez aussi cette section pour expliquer pourquoi.

JOHNS HOPKINS
BLOOMBERG SCHOOL
of PUBLIC HEALTH

Bill & Melinda Gates Institute for Population and Reproductive Health

Enquête de l'Indice des efforts pour le dividende démographique

Domaine 4. Recherche

* WE 4.1 La force des observatoires/parties prenantes/groupes de travail pour l'autonomisation des femmes : Mesure dans laquelle les parties prenantes du secteur (ex : groupe directif de parties prenantes, groupes de travail techniques, observatoire de l'autonomisation des femmes, groupe de coordination ou facilitation) :

	1	2	3	4	5	6	7	8	9	10	Je ne sais pas
WE 4.1.1 Ont un représentant du gouvernement, des institutions de formation, de la société civile, des organisations non gouvernementales et confessionnelles, des associations professionnelles, du secteur privé, etc.	◯	◯	◯	◯	◯	◯	◯	◯	◯	◯	◯
WE 4.1.2 Se réunissent régulièrement, émettent des rapports et recommandent des politiques aux directions des ministères compétents.	◯	◯	◯	◯	◯	◯	◯	◯	◯	◯	◯
WE 4.1.3 Ont un impact sur l'autonomisation des femmes dans le pays.	◯	◯	◯	◯	◯	◯	◯	◯	◯	◯	◯

* WE 4.2 La stratégie de recherche sur les droits des femmes : Mesure dans laquelle le plan national pour l'autonomisation des femmes et/ou autres documents nationaux comprennent une approche/stratégie de recherche sur l'autonomisation des femmes, partagée entre les partenaires.

	1	2	3	4	5	6	7	8	9	10	Je ne sais pas
1-Plus faible -> Plus élevé-10	◯	◯	◯	◯	◯	◯	◯	◯	◯	◯	◯

* WE 4.3 Partenaires collectant des données :

	1	2	3	4	5	6	7	8	9	10	Je ne sais pas
WE 4.3.1 Mesure dans laquelle la collecte des données est entreprise par des agences gouvernementales. (Cela peut comprendre les agences/bureaux de la statistique et/ou les ministères de l'économie/finances, éducation, santé, genre, etc.)	◯	◯	◯	◯	◯	◯	◯	◯	◯	◯	◯
WE 4.3.2 Mesure dans laquelle la collecte de données est entreprise par des institutions de recherche.	◯	◯	◯	◯	◯	◯	◯	◯	◯	◯	◯
WE 4.3.3 Mesure dans laquelle la collecte des données est entreprise par des chercheurs indépendants.	◯	◯	◯	◯	◯	◯	◯	◯	◯	◯	◯

* WE 4.4 La recherche thématique :

	1	2	3	4	5	6	7	8	9	10	Je ne sais pas
WE 4.4.1 Mesure dans laquelle de la recherche est menée sur les normes sociales et culturelles affectant l'autonomisation des femmes et la parité.	◯	◯	◯	◯	◯	◯	◯	◯	◯	◯	◯
WE 4.4.2 Mesure dans laquelle de la recherche est menée sur les effets économiques et sanitaires de l'autonomisation des femmes.	◯	◯	◯	◯	◯	◯	◯	◯	◯	◯	◯
WE 4.4.3 Mesure dans laquelle de la recherche est menée sur le mariage d'enfants pour informer la politique.	◯	◯	◯	◯	◯	◯	◯	◯	◯	◯	◯
WE 4.4.4 Mesure dans laquelle de la recherche est menée sur les résultats de l'éducation et de l'acquisition de compétences chez les filles.	◯	◯	◯	◯	◯	◯	◯	◯	◯	◯	◯

* WE 4.5 La qualité/ couverture des données :

	1	2	3	4	5	6	7	8	9	10	Je ne sais pas
WE 4.5.1 Mesure dans laquelle un système statistique de routine fiable fournit des données périodiques désagrégées par âge.	◯	◯	◯	◯	◯	◯	◯	◯	◯	◯	◯
WE 4.5.2 Mesure dans laquelle un système statistique de routine fiable fournit de bonnes données périodiques désagrégées par âge sur les besoins relatifs à l'autonomie des femmes parmi les populations de différentes communautés.	◯	◯	◯	◯	◯	◯	◯	◯	◯	◯	◯
WE 4.5.3 Mesure dans laquelle les systèmes de suivi et de surveillance sont évalués, et ces évaluations appliquées pour assurer la fiabilité des données désagrégées par âge.	◯	◯	◯	◯	◯	◯	◯	◯	◯	◯	◯

* WE 4.6 La qualité des institutions de recherche : Mesure dans laquelle le pays soutient et maintient des institutions de recherche qui développent des études permettant de soutenir la portée et la qualité de la collecte de données pour informer les efforts nationaux d'autonomisation des femmes.

	1	2	3	4	5	6	7	8	9	10	Je ne sais pas
1-Plus faible -> Plus élevé-10	◯	◯	◯	◯	◯	◯	◯	◯	◯	◯	◯

* WE 4.7 L'évaluation : Mesure dans laquelle les statistiques, enquêtes et études sur les programmes sont utilisées par du personnel spécialisé pour rapporter les opérations du programme et mesurer les progrès en matière d'autonomisation des femmes et de parité.

	1	2	3	4	5	6	7	8	9	10	Je ne sais pas
1-Plus faible -> Plus élevé-10	◯	◯	◯	◯	◯	◯	◯	◯	◯	◯	◯

* WE 4.8 L’utilisation des résultats d’évaluation par les gestionnaires de programme : Mesure dans laquelle les gestionnaires locaux des programmes d’autonomisation des femmes utilisent les conclusions de recherche et d’évaluations pour améliorer ces programmes en suivant les recommandations émises.

	1	2	3	4	5	6	7	8	9	10	Je ne sais pas
1-Plus faible -> Plus élevé-10	○	○	○	○	○	○	○	○	○	○	○

* WE 4.9 L’utilisation des résultats d’évaluation par les ministères : Mesure dans laquelle les administrateurs ministériels compétents utilisent systématiquement les données pour informer les politiques et interventions visant à résoudre les problèmes liés à l’autonomisation des femmes.

	1	2	3	4	5	6	7	8	9	10	Je ne sais pas
1-Plus faible -> Plus élevé-10	○	○	○	○	○	○	○	○	○	○	○

* WE 4.10 La dissémination d’informations à d’autres entités chargées de la mise en œuvre des programmes : Mesure dans laquelle des informations sont partagées ou disséminées entre géographies et à différents niveaux (national, état/province/comté, localités/sous-comtés).

	1	2	3	4	5	6	7	8	9	10	Je ne sais pas
1-Plus faible -> Plus élevé-10	○	○	○	○	○	○	○	○	○	○	○

WE 4.11 Commentaire : Utilisez cette section pour apporter des commentaires additionnels sur le niveau d’efforts, les défis et les succès du pays autour de la recherche sur l’autonomisation des femmes. Si vous avez répondu « Je ne sais pas » à l’une des questions ci-dessus, utilisez aussi cette section pour expliquer pourquoi.

JOHNS HOPKINS
BLOOMBERG SCHOOL of PUBLIC HEALTH

Bill & Melinda Gates Institute for Population and Reproductive Health

Enquête de l'Indice des efforts pour le dividende démographique

Domaine 5. Organisations de la Société Civile (OSC)

* WE 5.1 Le pouvoir des acteurs des OSC : Mesure dans laquelle les acteurs des OSC sont bien placés pour influencer la politique nationale concernant l'autonomisation des femmes.

	1	2	3	4	5	6	7	8	9	10	Je ne sais pas
1-Plus faible -> Plus élevé-10	◯	◯	◯	◯	◯	◯	◯	◯	◯	◯	◯

* WE 5.2 L'analyse budgétaire comme outil des OSC : Mesure dans laquelle les OSC utilisent l'analyse budgétaire comme un outil pour développer le plaidoyer en matière d'autonomisation des femmes

	1	2	3	4	5	6	7	8	9	10	Je ne sais pas
1-Plus faible -> Plus élevé-10	◯	◯	◯	◯	◯	◯	◯	◯	◯	◯	◯

* WE 5.3 Le soutien des services à base communautaire : Mesure dans laquelle les OSC participent au rapprochement des informations et services en matière d'autonomisation des femmes avec les communautés à travers des activités comprenant, mais sans s'y limiter, le renforcement de compétences des filles, les défenseurs/champions de l'autonomisation des femmes, et des campagnes d'éducation ou d'information pour augmenter l'engagement des familles et des communautés pour l'autonomie des femmes.

	1	2	3	4	5	6	7	8	9	10	Je ne sais pas
1-Plus faible -> Plus élevé-10	◯	◯	◯	◯	◯	◯	◯	◯	◯	◯	◯

* WE 5.4 Les droits humains et la qualité de la mise en œuvre : Mesure dans laquelle les OSC plaident et soutiennent des approches de l'autonomisation des femmes fondées sur le droit pour veiller à ce que les bénéficiaires des programmes et politiques d'autonomisation des femmes puissent voir des avancées selon leurs propres termes.

	1	2	3	4	5	6	7	8	9	10	Je ne sais pas
1-Plus faible -> Plus élevé-10	◯	◯	◯	◯	◯	◯	◯	◯	◯	◯	◯

* WE 5.5 Le changement social et des comportements (CSC) : Mesure dans laquelle les OSC soutiennent des interventions pour le CSC comprenant, mais sans s'y limiter, les programmes transformateurs de genre, les compétences de vie et l'éducation entre pairs, l'éducation à la santé de l'adulte, l'éducation en milieu scolaire, la communication au sein du couple, la mobilisation communautaire, les interventions des gardiens de la communauté et/ou des interventions dans les médias de masse pour le grand public. Ces interventions peuvent être mises en œuvre au niveau communautaire ou individuel.

	1	2	3	4	5	6	7	8	9	10	Je ne sais pas
1-Plus faible -> Plus élevé-10	◯	◯	◯	◯	◯	◯	◯	◯	◯	◯	◯

* WE 5.6 Les jeunes : Mesure dans laquelle les OSC promeuvent l'autonomisation des femmes à travers les programmes pour jeunes.

	1	2	3	4	5	6	7	8	9	10	Je ne sais pas
1-Plus faible -> Plus élevé-10	◯	◯	◯	◯	◯	◯	◯	◯	◯	◯	◯

* WE 5.7 Les programmes pour les hommes : Mesure dans laquelle les OSC soutiennent les politiques, interventions et programmes d'autonomisation des femmes ciblant les hommes.

	1	2	3	4	5	6	7	8	9	10	Je ne sais pas
1-Plus faible -> Plus élevé-10	◯	◯	◯	◯	◯	◯	◯	◯	◯	◯	◯

* WE 5.8 Le plaidoyer/ la redevabilité : Mesure dans laquelle les OSC soutiennent la formation et le renforcement de compétences des membres de la communauté pour augmenter leur compréhension des politiques, processus et activités, et pour renforcer leurs capacités à identifier les problèmes liés à l'autonomisation des femmes, résoudre collectivement ces problèmes et mener des actions de plaidoyer plus ciblées.

	1	2	3	4	5	6	7	8	9	10	Je ne sais pas
1-Plus faible -> Plus élevé-10	◯	◯	◯	◯	◯	◯	◯	◯	◯	◯	◯

* WE 5.9 L'évaluation et le suivi dirigés par les OSC : Mesure dans laquelle les OSC évaluent, suivent et émettent des rapports sur l'efficacité des politiques et programmes pour améliorer la redevabilité des prestataires et des politiques d'autonomisation des femmes.

	1	2	3	4	5	6	7	8	9	10	Je ne sais pas
1-Plus faible -> Plus élevé-10	○	○	○	○	○	○	○	○	○	○	○

* WE 5.10 Les partenariats entre OSC : Mesure dans laquelle les OSC ont formé des alliances nationales et des partenariats régionaux pour renforcer leur position et potentiellement améliorer le leadership et les mécanismes de financement afin de faire progresser l'agenda de l'autonomisation des femmes.

	1	2	3	4	5	6	7	8	9	10	Je ne sais pas
1-Plus faible -> Plus élevé-10	○	○	○	○	○	○	○	○	○	○	○

WE 5.11 Commentaire : Utilisez cette section pour apporter des commentaires additionnels sur le niveau d'efforts, les défis et les succès du pays concernant le rôle des OSC pour l'autonomisation des femmes. Si vous avez répondu « Je ne sais pas » à l'une des questions ci-dessus, utilisez aussi cette section pour expliquer pourquoi.

JOHNS HOPKINS
BLOOMBERG SCHOOL
of PUBLIC HEALTH

Bill & Melinda Gates Institute for Population and Reproductive Health

Enquête de l’Indice des efforts pour le dividende démographique

Module : Résilience et durabilité du secteur

Étant donné l’impact sanitaire et socioéconomique de la pandémie de COVID-19, et son impact probable sur la progression vers le DD, cet indice des efforts pour le DD intègre des questions pour évaluer la résilience et la durabilité des systèmes dans les secteurs clés du DD. Les éléments couverts par les questions liées à la pandémie de COVID-19 ci-dessous relèvent des dimensions clés des systèmes résilients et visent à apporter des informations déterminantes sur le potentiel d’une réponse efficace aux menaces de maladies infectieuses émergentes et autres crises de santé publique.

Veuillez noter chaque élément sur une échelle de 1 à 10, 1 étant le score le plus faible (faible état/capacité) et 10 le plus élevé (très bon état/capacité).

* Dimension physique : structures, équipements, états du système et capacités.

	1	2	3	4	5	6	7	8	9	10	Je ne sais pas
WE-M1 - Plan/Préparation - État et capacité de l’équipement, du personnel et de la structure du secteur de l’autonomisation des femmes avant la crise. (1 = faible état/capacité ; 10 = très bon état/capacité)											
WE-M2 - Absorption – Mesure dans laquelle le secteur de l’autonomisation des femmes a reconnu la pandémie de COVID-19 et pu continuer à être fonctionnel.											

	1	2	3	4	5	6	7	8	9	10	Je ne sais pas
WE-M3 - Absorption – Intégration : Niveau d'intégration des services et programmes d'autonomisation des femmes pour mitiger les effets de la pandémie de COVID-19 sur l'autonomie des femmes.	○	○	○	○	○	○	○	○	○	○	○
WE-M4 - Récupération – Mesure dans laquelle les programmes d'autonomisation des femmes ont été capables d'induire des changements pour récupérer un niveau de fonctionnalité pré-COVID-19.	○	○	○	○	○	○	○	○	○	○	○
WE-M5 - Récupération – Gestion du financement et des bailleurs de fonds : Niveau de mobilisation des ressources et de l'allocation budgétaire pour mitiger les effets de la pandémie de COVID-19 sur l'autonomie des femmes.	○	○	○	○	○	○	○	○	○	○	○
WE-M6 - Adaptation – Mesure dans laquelle des changements ont été effectués pour améliorer la résilience du secteur de l'autonomisation des femmes.	○	○	○	○	○	○	○	○	○	○	○

* Dimension informatique : création, gestion, stockage et utilisation des données.

	1	2	3	4	5	6	7	8	9	10	Je ne sais pas
WE-M7 - Plan/Préparation – Mesure dans laquelle les données liées à l'autonomisation des femmes étaient suffisamment préparées, présentées, analysées et stockées avant la crise.	○	○	○	○	○	○	○	○	○	○	○

	1	2	3	4	5	6	7	8	9	10	Je ne sais pas
WE-M8 - Absorption – Connaissance : Mesure dans laquelle le secteur de l'autonomisation des femmes a pu conduire des évaluations en temps réel de son état de fonctionnement, en anticipation des pertes en cascade des services liés à l'autonomie des femmes.	○	○	○	○	○	○	○	○	○	○	○
WE-M9 - Absorption – Connaissance/ Conscience : Capacité du système de surveillance à détecter les limites/ menaces posées à l'autonomie des femmes par la pandémie de COVID-19.	○	○	○	○	○	○	○	○	○	○	○
WE-M10 - Mesure dans laquelle les données sur l'autonomisation des femmes ont été utilisées pour suivre les progrès vers la récupération et anticiper des scenarii de récupération.	○	○	○	○	○	○	○	○	○	○	○
WE-M11 - Adaptation – Mesure dans laquelle le secteur de l'autonomisation des femmes crée et améliore ses protocoles de stockage et utilisation en temps réel de ses données sur l'autonomisation des femmes.	○	○	○	○	○	○	○	○	○	○	○

* Dimension cognitive : compréhension, modèles cognitifs, idées reçues, préjugés et valeurs.

	1	2	3	4	5	6	7	8	9	10	Je ne sais pas
WE-M12 - Planification/Préparation – Mesure dans laquelle le système de conception et les décisions opérationnelles étaient préparés à anticiper la crise.	○	○	○	○	○	○	○	○	○	○	○

	1	2	3	4	5	6	7	8	9	10	Je ne sais pas
WE-M13 - Absorption – Mesure dans laquelle le secteur de l'autonomisation des femmes a des protocoles de contingence suffisants et une gestion de crise proactive.	○	○	○	○	○	○	○	○	○	○	○
WE-M14 - Absorption – Redevabilité : Niveau d'effort pour assurer une redevabilité optimale vis-à-vis des ressources allouées à l'autonomisation des femmes dans le cadre de la réponse à la pandémie de COVID-19. (Cela peut prendre la forme de soutien aux services atténués pour répondre aux besoins de protection des femmes et des filles).	○	○	○	○	○	○	○	○	○	○	○
WE-M15 - Récupération – Mesure dans laquelle les décisions sont orientées sur la récupération et communiquées aux communautés sur la base de données probantes pour promouvoir des comportements sûrs	○	○	○	○	○	○	○	○	○	○	○

	1	2	3	4	5	6	7	8	9	10	Je ne sais pas
WE-M16 - Récupération – Connaissance/Conscience : Mesure dans laquelle le secteur établit une certaine confiance à travers sa communication sur les barrières posées par la pandémie de COVID-19 pour soutenir l'accès et l'utilisation des services et programmes pour l'autonomisation des femmes. (Cela comprend la surveillance des disparités de genre dans les réponses à la pandémie et/ou la surveillance augmentée des questions liées à l'autonomie des femmes, comme la violence basée sur le genre).	○	○	○	○	○	○	○	○	○	○	○
WE-M17 - Adaptation – Mesure dans laquelle le secteur de l'autonomisation des femmes conçoit de nouvelles configurations systémiques, objectifs et critères de décision.	○	○	○	○	○	○	○	○	○	○	○
WE-M18 Adaptation - Niveau d'adaptation pour répondre aux menaces COVID-19 qui pèsent sur l'autonomisation des femmes	○	○	○	○	○	○	○	○	○	○	○

* Social dimension: interaction, collaboration and self-synchronization between people, entities and institutions.

	1	2	3	4	5	6	7	8	9	10	Je ne sais pas
WE-M19 - Planification /Préparation – Mesure dans laquelle une formation sur la gestion d'épidémies/crises a été menée et a permis de mettre à profit les réseaux sociaux, le capital social et les normes institutionnelles et culturelles avant la crise.	○	○	○	○	○	○	○	○	○	○	○
WE-M20 - Absorption – Mesure dans laquelle le personnel et les institutions sociales ont été accessibles et ont fait preuve d'ingéniosité dans leur réponse à l'épidémie/crise.	○	○	○	○	○	○	○	○	○	○	○
WE-M21 - Absorption - Self-regulation: Extent to which national leaders had the authority to effect timely sectoral changes through an infrastructure flexible.	○	○	○	○	○	○	○	○	○	○	○
WE-M22 - Récupération – Mesure dans laquelle le secteur de l'autonomisation des femmes est impliqué dans le partage de connaissances et d'équipes pour améliorer la récupération du système.	○	○	○	○	○	○	○	○	○	○	○
WE-M23 – Niveau d'engagement d'une équipe multidisciplinaire pour mitiger les effets de la pandémie de COVID-19 sur l'autonomisation des femmes.	○	○	○	○	○	○	○	○	○	○	○

	1	2	3	4	5	6	7	8	9	10	Je ne sais pas
WE-M24 - Adaptation - Leadership et management : Niveau de leadership démontré par les dirigeants du secteur de l'autonomisation des femmes pour mitiger les effets de la pandémie de COVID-19 sur l'autonomie des femmes.	○	○	○	○	○	○	○	○	○	○	○

* WE M-25 Temporalité : Mesure de la rapidité de la réaction du gouvernement –sa communication et mise en œuvre des mesures- pour mitiger l'impact immédiat et sur le long terme de la pandémie de COVID-19. Cette question porte sur la riposte nationale dans son ensemble, et non la réponse spécifique de votre secteur.

	1	2	3	4	5	6	7	8	9	10	Je ne sais pas
1-Plus faible -> Plus élevé-10	○	○	○	○	○	○	○	○	○	○	○

WE M-26 Commentaires : Veuillez utiliser cette section pour tout commentaire additionnel sur la résilience et la durabilité des opportunités et systèmes nationaux d'autonomisation des femmes et des filles en fonction de votre expérience de la pandémie de COVID-19. Si vous avez répondu « Je ne sais pas » à l'une des questions, utilisez aussi cette section pour expliquer pourquoi.

JOHNS HOPKINS
BLOOMBERG SCHOOL
of PUBLIC HEALTH

Bill & Melinda Gates Institute for Population and Reproductive Health

Enquête de l’Indice des efforts pour le dividende démographique

Questionnaire sur le marché du travail (MT)

Répondez aux questions suivantes en fonction de votre expérience/ expertise dans ce secteur spécifique.

Afin d’obtenir une représentation synthétique des efforts nationaux pour le dividende démographique, notez les éléments suivants sur une échelle de 1 à 10, 1 étant le score le plus faible (efforts très faibles ou quasi-inexistants) et 10 le plus élevé (efforts robustes). Le cas échéant, si une politique ou activité n’existe pas, répondez 0.

Donnez un score à chaque élément. Toutes les réponses seront enregistrées au format illustré ci-dessous :

Effort le plus faible	1	2	3	4	5	6	7	8	9	10	Effort le plus élevé	Je ne sais pas

JOHNS HOPKINS
BLOOMBERG SCHOOL
of PUBLIC HEALTH

Bill & Melinda Gates Institute for Population and Reproductive Health

Enquête de l'Indice des efforts pour le dividende démographique

Domaine 1. Politique/ Prise de décision

* LM 1.1 L'intégration de l'économie mondiale : Mesure dans laquelle le pays vise à s'intégrer à l'économie mondiale.

	1	2	3	4	5	6	7	8	9	10	Je ne sais pas
LM 1.1.1 Mesure dans laquelle le pays légifère pour l'ouverture des échanges commerciaux.	◯	◯	◯	◯	◯	◯	◯	◯	◯	◯	◯
LM 1.1.2 Niveau d'effort en faveur de la croissance des exportations nationales.	◯	◯	◯	◯	◯	◯	◯	◯	◯	◯	◯

* LM 1.2 L'intégration économique régionale : Mesure dans laquelle le pays vise à s'intégrer à l'économie régionale.

	1	2	3	4	5	6	7	8	9	10	Je ne sais pas
1-Plus faible -> Plus élevé-10	◯	◯	◯	◯	◯	◯	◯	◯	◯	◯	◯

* LM 1.3 La technologie : Mesure dans laquelle le gouvernement a investi dans l'infrastructure, à la fois traditionnelle et technologique, pour permettre au pays de saisir les opportunités de demain/ d'intégration technologique en complément de la main d'œuvre.

	1	2	3	4	5	6	7	8	9	10	Je ne sais pas
1-Plus faible -> Plus élevé-10	◯	◯	◯	◯	◯	◯	◯	◯	◯	◯	◯

* LM 1.4 Le soutien du gouvernement : Mesure dans laquelle des politiques et plans sont en place et prévoient des dispositifs de protection sociale pour une transition harmonieuse entre les différents emplois, ou « petits boulots », des personnes, et pour les protéger de la précarité/ volatilité des revenus.

	1	2	3	4	5	6	7	8	9	10	Je ne sais pas
LM 1.4.1 Mesure dans laquelle une politique nationale établissant un salaire minimum est en place.	○	○	○	○	○	○	○	○	○	○	○
LM 1.4.2 Mesure dans laquelle une politique nationale définissant un système d'assurance chômage est en place, et niveau d'efficacité de sa mise en œuvre.	○	○	○	○	○	○	○	○	○	○	○

* LM 1.5 Le chômage : Mesure dans laquelle les politiques rendent compte et répondent au chômage chez différents groupes de la population et dans différentes aires géographiques.

	1	2	3	4	5	6	7	8	9	10	Je ne sais pas
1-Plus faible -> Plus élevé-10	○	○	○	○	○	○	○	○	○	○	○

* LM 1.6 Les groupes défavorisés : Mesure dans laquelle des politiques existent, et sont effectivement mises en œuvre, pour soutenir les opportunités d'embauche des groupes défavorisés (c'est-à-dire les filles et les femmes, les jeunes, les personnes en situation de mobilité réduite ou de handicap, etc.).

	1	2	3	4	5	6	7	8	9	10	Je ne sais pas
1-Plus faible -> Plus élevé-10	○	○	○	○	○	○	○	○	○	○	○

* LM 1.7 Le changement climatique et le développement durable : Mesure dans laquelle les secteurs de l'agriculture et de la production industrielle sont préparés aux impacts des effets du changement climatique sur le marché du travail en élaborant des stratégies de planification familiale en vue des besoins du marché de travail de demain.

	1	2	3	4	5	6	7	8	9	10	Je ne sais pas
1-Plus faible -> Plus élevé-10	○	○	○	○	○	○	○	○	○	○	○

* LM 1.8 L'urbanisation : Mesure dans laquelle les politiques et la planification anticipent et sont préparées à la croissance urbaine résultant et contribuant au changement climatique, et ses impacts particuliers sur les marchés du travail.

	1	2	3	4	5	6	7	8	9	10	Je ne sais pas
1-Plus faible -> Plus élevé-10	◯	◯	◯	◯	◯	◯	◯	◯	◯	◯	◯

* LM 1.9 L'économie de services : Mesure dans laquelle des politiques et des cadres de travail sont en place pour faire passer les économies basées sur du travail informel au travail formalisé répondant aux demandes locales, régionales et mondiales de biens et de services.

	1	2	3	4	5	6	7	8	9	10	Je ne sais pas
1-Plus faible -> Plus élevé-10	◯	◯	◯	◯	◯	◯	◯	◯	◯	◯	◯

* LM 1.10 La responsabilité sociale d'entreprise : Mesure dans laquelle la législation nationale du marché du travail utilise la responsabilité sociale des entreprises (RSE) pour soutenir l'entreprenariat chez les jeunes.

	1	2	3	4	5	6	7	8	9	10	Je ne sais pas
1-Plus faible -> Plus élevé-10	◯	◯	◯	◯	◯	◯	◯	◯	◯	◯	◯

* LM 1.11 Les investissements ciblés dans le marché du travail : Mesure dans laquelle le gouvernement a ciblé ses investissements sur les secteurs générateurs d'emplois, y compris les Technologies de l'Information et la Communication (TIC), le secteur de production industrielle, l'agriculture et les entreprises industrielles agricoles, pour générer de l'emploi et encourager la croissance inclusive.

	1	2	3	4	5	6	7	8	9	10	Je ne sais pas
1-Plus faible -> Plus élevé-10	◯	◯	◯	◯	◯	◯	◯	◯	◯	◯	◯

* LM 1.12 Les travailleurs indépendants : Mesure dans laquelle les politiques soutiennent l'élévation des retours sur investissement des travailleurs indépendants et la création de plus d'opportunités pour passer du travail indépendant à des emplois mieux rémunérés. (Cela comprend le travail en milieu rural).

	1	2	3	4	5	6	7	8	9	10	Je ne sais pas
1-Plus faible -> Plus élevé-10	◯	◯	◯	◯	◯	◯	◯	◯	◯	◯	◯

* LM 1.13 Le microcrédit : Mesure dans laquelle le gouvernement permet l'accès à des microcrédits pour exercer des activités à but lucratif, sécurisant des crédits abordables pour les petites et moyennes entreprises et les travailleurs indépendants.

	1	2	3	4	5	6	7	8	9	10	Je ne sais pas
1-Plus faible -> Plus élevé-10	○	○	○	○	○	○	○	○	○	○	○

* LM 1.14 L'épargne : Mesure dans laquelle le gouvernement soutient la création de produits financiers accessibles et des politiques promouvant la culture de l'épargne.

	1	2	3	4	5	6	7	8	9	10	Je ne sais pas
1-Plus faible -> Plus élevé-10	○	○	○	○	○	○	○	○	○	○	○

* LM 1.15 L'acquisition de compétences : Mesure dans laquelle des politiques existent, et sont mises en œuvre efficacement, pour soutenir l'acquisition de compétences valorisées sur le marché du travail d'aujourd'hui et de demain.

	1	2	3	4	5	6	7	8	9	10	Je ne sais pas
1-Plus faible -> Plus élevé-10	○	○	○	○	○	○	○	○	○	○	○

LM 1.16 Commentaires : Veuillez utiliser cette section pour nous faire part de tout commentaire additionnel sur le niveau d'effort, les défis ou les succès du pays autour de la politique/prise de décision dans le secteur du marché du travail. Si vous avez répondu « Je ne sais pas » à l'une des questions précédentes, veuillez utiliser cette section pour expliquer pourquoi.

JOHNS HOPKINS
BLOOMBERG SCHOOL
of PUBLIC HEALTH

Bill & Melinda Gates Institute for Population and Reproductive Health

Enquête de l'Indice des efforts pour le dividende démographique

Domaine 2. Services ou programmes

* LM 2.1 La réduction du chômage : Mesure dans laquelle des efforts sont menés pour développer et mettre en œuvre des stratégies visant à réduire la proportion des personnes au chômage par année cible (en accord avec le Plan de mise en œuvre des dix premières années de l'Agenda 2063).

	1	2	3	4	5	6	7	8	9	10	Je ne sais pas
1-Plus faible -> Plus élevé-10	○	○	○	○	○	○	○	○	○	○	○

* LM 2.2. Le chômage chez les jeunes : Mesure dans laquelle des programmes liés au marché du travail ont effectivement réduit le taux de chômage chez les jeunes en particulier.

	1	2	3	4	5	6	7	8	9	10	Je ne sais pas
1-Plus faible -> Plus élevé-10	○	○	○	○	○	○	○	○	○	○	○

* LM 2.3 L'accès au microcrédit : Mesure dans laquelle les fonds nationaux et régionaux ont été établis et opérationnalisés pour augmenter l'accès des personnes au capital commercial abordable.

	1	2	3	4	5	6	7	8	9	10	Je ne sais pas
1-Plus faible -> Plus élevé-10	○	○	○	○	○	○	○	○	○	○	○

* LM 2.4 L'exploitation des capacités uniques des jeunes : Mesure dans laquelle des programmes et services soutiennent les talents spécifiques des jeunes pour les aider à s'épanouir et réaliser leur plein potentiel. (Cela couvre les objectifs programmatiques spécifiques liés à l'acquisition de talents).

	1	2	3	4	5	6	7	8	9	10	Je ne sais pas
1-Plus faible -> Plus élevé-10	○	○	○	○	○	○	○	○	○	○	○

* LM 2.5 Les opportunités de première embauche : Mesure dans laquelle les programmes et la législation du gouvernement engagent les partenaires du secteur privé à élargir les opportunités de stages, d'apprentissage professionnel et de formation continue au travail pour les femmes et les jeunes.

	1	2	3	4	5	6	7	8	9	10	Je ne sais pas
1-Plus faible -> Plus élevé-10	◯	◯	◯	◯	◯	◯	◯	◯	◯	◯	◯

* LM 2.6 L'entrée des jeunes dans la population active : Mesure de la promotion du volontariat des jeunes, des programmes pour jeunes professionnelles et autres opportunités similaires pour améliorer leurs capacités et les exposer aux organisations régionales et internationales.

	1	2	3	4	5	6	7	8	9	10	Je ne sais pas
1-Plus faible -> Plus élevé-10	◯	◯	◯	◯	◯	◯	◯	◯	◯	◯	◯

* LM 2.7 Les risques de l'entreprenariat : Mesure dans laquelle les personnes ont un meilleur accès aux services d'achat et de finance du gouvernement pour réduire les difficultés liées au lancement d'un projet professionnel et/ou à la conduite d'activités commerciales dans et entre les pays.

	1	2	3	4	5	6	7	8	9	10	Je ne sais pas
1-Plus faible -> Plus élevé-10	◯	◯	◯	◯	◯	◯	◯	◯	◯	◯	◯

* LM 2.8 Les ressources pour l'entreprenariat des jeunes : Mesure dans laquelle les projets d'entreprenariat des jeunes bénéficient d'une mobilisation de ressources directe et ciblée, soutenue par un cadre régulateur, pour réduire les difficultés liées au lancement d'un projet professionnel et/ou à la conduite d'activités commerciales dans et entre les pays.

	1	2	3	4	5	6	7	8	9	10	Je ne sais pas
1-Plus faible -> Plus élevé-10	◯	◯	◯	◯	◯	◯	◯	◯	◯	◯	◯

* LM 2.9 Les opportunités professionnelles : Mesure dans laquelle les opportunités de formation professionnelles sont amplifiées pour permettre l'acquisition de compétences et améliorer les capacités d'insertion professionnelle, la productivité et la compétitivité.

	1	2	3	4	5	6	7	8	9	10	Je ne sais pas
1-Plus faible -> Plus élevé-10	◯	◯	◯	◯	◯	◯	◯	◯	◯	◯	◯

* LM 2.10 L'éducation et l'insertion professionnelle : Mesure dans laquelle des améliorations ont été apportées pour favoriser l'accès inclusif à l'éducation à tous les niveaux et offrir des alternatives viables à de nombreux jeunes, particulièrement les filles adolescentes, qui abandonnent le système d'éducation scolaire formel, en facilitant leur réinsertion, en restructurant l'éducation informelle et la formation via une certification standardisée dans et entre les pays.

	1	2	3	4	5	6	7	8	9	10	Je ne sais pas
1-Plus faible -> Plus élevé-10	◯	◯	◯	◯	◯	◯	◯	◯	◯	◯	◯

* LM 2.11 Le travail « ami des femmes » : Mesure dans laquelle les lieux de travail dans les secteurs privé et public ont adopté des politiques et pratiques « amies des femmes » pour éliminer toute forme de discrimination basée sur le genre, tout en leur permettant de prendre pleinement part à la population active.

	1	2	3	4	5	6	7	8	9	10	Je ne sais pas
1-Plus faible -> Plus élevé-10	◯	◯	◯	◯	◯	◯	◯	◯	◯	◯	◯

* LM 2.12 La sécurité de l'emploi :

	1	2	3	4	5	6	7	8	9	10	Je ne sais pas
LM 2.12.1 Mesure dans laquelle les périodes de préavis relatifs aux plans sociaux et au licenciement sont respectées.	◯	◯	◯	◯	◯	◯	◯	◯	◯	◯	◯
LM 2.12.2 Mesure dans laquelle les indemnités de départ dans le cadre de plans sociaux ou de licenciements sont appliquées.	◯	◯	◯	◯	◯	◯	◯	◯	◯	◯	◯

* LM 2.13 Le salaire minimum : Mesure dans laquelle un salaire minimum est appliqué au travailleur « type » (défini comme un salarié moyen, issu de groupes ethniques et religieux majoritaires, vivant dans les grandes villes, et non syndicalisé).

	1	2	3	4	5	6	7	8	9	10	Je ne sais pas
1-Plus faible -> Plus élevé-10	◯	◯	◯	◯	◯	◯	◯	◯	◯	◯	◯

* LM 2.14 Les programmes du marché du travail : Mesure dans laquelle les programmes du marché du travail ont effectivement diversifié la main d’œuvre et les besoins du marché du travail, tout en diversifiant simultanément les compétences de cette main d’œuvre.

	1	2	3	4	5	6	7	8	9	10	Je ne sais pas
1-Plus faible -> Plus élevé-10											

* LM 2.15 L’informalité sur le marché du travail : Mesure dans laquelle des programmes sont mis en œuvre pour transformer le travail informel en travail légal.

	1	2	3	4	5	6	7	8	9	10	Je ne sais pas
1-Plus faible -> Plus élevé-10											

LM 2.16 Commentaires : Veuillez utiliser cette section pour nous faire part de tout commentaire additionnel sur le niveau d’effort, les défis ou les succès du pays autour des services et programmes dans le secteur du marché du travail. Si vous avez répondu « Je ne sais pas » à l’une des questions précédentes, veuillez utiliser cette section pour expliquer pourquoi.

JOHNS HOPKINS
BLOOMBERG SCHOOL
of PUBLIC HEALTH

Bill & Melinda Gates Institute for Population and Reproductive Health

Enquête de l'Indice des efforts pour le dividende démographique

Domaine 3. Plaidoyer

* LM 3.1 La parité sur le marché du travail : Mesure dans laquelle les groupes ou individus appellent, et voient des améliorations, en matière de parité et d'égalité hommes-femmes sur les lieux de travail.

	1	2	3	4	5	6	7	8	9	10	Je ne sais pas
1-Plus faible -> Plus élevé-10	○	○	○	○	○	○	○	○	○	○	○

* LM 3.2 L'égalité des jeunes sur le marché du travail : Mesure dans laquelle les groupes et les individus appellent à des changements du marché du travail et des régulations au bénéfice des populations jeunes rurales et urbaines.

	1	2	3	4	5	6	7	8	9	10	Je ne sais pas
1-Plus faible -> Plus élevé-10	○	○	○	○	○	○	○	○	○	○	○

* LM 3.3 Le plaidoyer pour le salaire minimum : Mesure dans laquelle les groupes de plaidoyer réussissent à inciter des lois pour le salaire minimum, ou une augmentation du salaire minimum si une telle loi existe déjà.

	1	2	3	4	5	6	7	8	9	10	Je ne sais pas
1-Plus faible -> Plus élevé-10	○	○	○	○	○	○	○	○	○	○	○

* LM 3.4 Le plaidoyer du gouvernement : Mesure dans laquelle les membres du gouvernement plaident pour des améliorations de ses propres structures et priorités. (Cela peut comprendre des progrès sur le marché du travail en matière d'inclusion, opportunités, programmes et droits des jeunes, et autres concepts similaires dans différentes populations).

	1	2	3	4	5	6	7	8	9	10	Je ne sais pas
1-Plus faible -> Plus élevé-10	○	○	○	○	○	○	○	○	○	○	○

* LM 3.5 Les protections sociales : Mesure dans laquelle il existe un plaidoyer pour la protection de l’emploi et l’assurance chômage.

	1	2	3	4	5	6	7	8	9	10	Je ne sais pas
1-Plus faible -> Plus élevé-10	○	○	○	○	○	○	○	○	○	○	○

LM 3.6 Commentaires : Veuillez utiliser cette section pour nous faire part de tout commentaire additionnel sur le niveau d’effort, les défis ou les succès du pays autour du plaidoyer dans le secteur du marché du travail. Si vous avez répondu « Je ne sais pas » à l’une des questions précédentes, veuillez utiliser cette section pour expliquer pourquoi.

JOHNS HOPKINS
BLOOMBERG SCHOOL of PUBLIC HEALTH

Bill & Melinda Gates Institute for Population and Reproductive Health

Enquête de l’Indice des efforts pour le dividende démographique

Domaine 4. Recherche

* LM 4.1 La force des observatoires/parties prenantes/groupes de travail dans le secteur du marché du travail : Mesure dans laquelle les parties prenantes du secteur du marché du travail (ex : groupe directif de parties prenantes, groupes de travail techniques, observatoire du marché du travail, groupe de coordination ou facilitation) :

	1	2	3	4	5	6	7	8	9	10	Je ne sais pas
LM 4.1.1 Ont un représentant du gouvernement, des institutions de formation, de la société civile, des organisations non gouvernementales et confessionnelles, des associations professionnelles, du secteur privé, etc.											
LM 4.1.2 Se réunissent régulièrement, émettent des rapports et recommandent des politiques aux directions des ministères compétents.											
LM 4.1.3 Ont un impact sur le marché du travail dans le pays.											

* LM 4.2 La stratégie de recherche sur le marché du travail : Mesure dans laquelle les documents nationaux prévoient une stratégie/approche complète pour la recherche sur le marché du travail qui soit partagée entre partenaires.

	1	2	3	4	5	6	7	8	9	10	Je ne sais pas
1-Plus faible -> Plus élevé-10											

* LM 4.3 Les partenaires de l'analyse du marché du travail :

	1	2	3	4	5	6	7	8	9	10	Je ne sais pas
LM 4.3.1 Mesure dans laquelle la collecte des données est entreprise par des agences gouvernementales.	○	○	○	○	○	○	○	○	○	○	○
LM 4.3.2 Mesure dans laquelle la collecte de données est entreprise par des institutions de recherche.	○	○	○	○	○	○	○	○	○	○	○
LM 4.3.3 Mesure dans laquelle la collecte des données est entreprise par des chercheurs indépendants.	○	○	○	○	○	○	○	○	○	○	○

* LM 4.4 Les domaines de la recherche sur le marché du travail :

	1	2	3	4	5	6	7	8	9	10	Je ne sais pas
LM 4.4.1 Mesure dans laquelle de la recherche est menée sur les tendances du chômage.	○	○	○	○	○	○	○	○	○	○	○
LM 4.4.2 Mesure dans laquelle de la recherche est menée sur les jeunes et le marché du travail.	○	○	○	○	○	○	○	○	○	○	○
LM 4.4.3 Mesure dans laquelle de la recherche est menée sur les femmes et le marché du travail.	○	○	○	○	○	○	○	○	○	○	○
LM 4.4.4 Mesure dans laquelle de la recherche est menée sur les tendances et les trajectoires des besoins du marché du travail.	○	○	○	○	○	○	○	○	○	○	○

* LM 4.5 La qualité/ couverture des données :

	1	2	3	4	5	6	7	8	9	10	Je ne sais pas
LM 4.5.1 Mesure dans laquelle un système statistique de routine fiable fournit des données périodiques sur le marché du travail.	○	○	○	○	○	○	○	○	○	○	○
LM 4.5.2 Mesure dans laquelle un système statistique de routine fiable fournit de bonnes données périodiques sur les besoins du marché du travail parmi les populations de différentes communautés.	○	○	○	○	○	○	○	○	○	○	○
LM 4.5.3 Mesure dans laquelle les systèmes de suivi et de surveillance sont évalués, et ces évaluations appliquées pour assurer la fiabilité des données.	○	○	○	○	○	○	○	○	○	○	○

* LM 4.6 Les registres : Mesure dans laquelle les systèmes de registre, rapports et retours/commentaires sur les résultats du marché du travail sont adéquats.

	1	2	3	4	5	6	7	8	9	10	Je ne sais pas
1-Plus faible -> Plus élevé-10	○	○	○	○	○	○	○	○	○	○	○

* LM 4.7 La qualité des institutions de recherche : Mesure dans laquelle le pays a la capacité de soutenir et maintenir les institutions de recherche qui développent des études/ collectent des données liées au marché du travail.

	1	2	3	4	5	6	7	8	9	10	Je ne sais pas
1-Plus faible -> Plus élevé-10	○	○	○	○	○	○	○	○	○	○	○

* LM 4.8 L’évaluation : Mesure dans laquelle les statistiques de programmes, enquêtes et études sont utilisées par du personnel spécialisé pour rapporter sur les opérations des programmes et mesurer le progrès.

	1	2	3	4	5	6	7	8	9	10	Je ne sais pas
1-Plus faible -> Plus élevé-10	○	○	○	○	○	○	○	○	○	○	○

* LM 4.9 L'utilisation des résultats d'évaluation par les décideurs : Mesure dans laquelle les gestionnaires locaux des programmes du marché du travail utilisent les conclusions de recherche et d'évaluations pour améliorer ces programmes en suivant les recommandations émises.

	1	2	3	4	5	6	7	8	9	10	Je ne sais pas
1-Plus faible -> Plus élevé-10	○	○	○	○	○	○	○	○	○	○	○

* LM 4.10 L'utilisation des résultats d'évaluation par les ministères : Mesure dans laquelle les administrateurs ministériels compétents utilisent systématiquement les données pour informer les politiques et interventions visant à résoudre les problèmes liés au marché du travail.

	1	2	3	4	5	6	7	8	9	10	Je ne sais pas
1-Plus faible -> Plus élevé-10	○	○	○	○	○	○	○	○	○	○	○

* LM 4.11 La dissémination d'informations à d'autres entités chargées de la mise en œuvre des programmes : Mesure dans laquelle des informations sont partagées ou disséminées entre géographies et à différents niveaux (national, état/province/comté, localités/sous-comtés).

	1	2	3	4	5	6	7	8	9	10	Je ne sais pas
1-Plus faible -> Plus élevé-10	○	○	○	○	○	○	○	○	○	○	○

LM 4.12 Commentaires : Veuillez utiliser cette section pour nous faire part de tout commentaire additionnel sur le niveau d'effort, les défis ou les succès du pays autour de la recherche dans le secteur du marché du travail. Si vous avez répondu « Je ne sais pas » à l'une des questions précédentes, veuillez utiliser cette section pour expliquer pourquoi.

JOHNS HOPKINS
BLOOMBERG SCHOOL
of PUBLIC HEALTH

Bill & Melinda Gates Institute for Population and Reproductive Health

Enquête de l'Indice des efforts pour le dividende démographique

Dimension 5. Organisations de la société civile (OSC)

* LM 5.1 Le pouvoir des acteurs des OSC : Mesure dans laquelle les acteurs des OSC sont bien placés pour influencer la politique et l'action nationale concernant le marché du travail.

	1	2	3	4	5	6	7	8	9	10	Je ne sais pas
1-Plus faible -> Plus élevé-10	○	○	○	○	○	○	○	○	○	○	○

* LM 5.2 L'analyse budgétaire comme outil des OSC : Mesure dans laquelle les OSC utilisent l'analyse budgétaire comme un outil pour développer le plaidoyer en matière de marché du travail.

	1	2	3	4	5	6	7	8	9	10	Je ne sais pas
1-Plus faible -> Plus élevé-10	○	○	○	○	○	○	○	○	○	○	○

* LM 5.3 Le soutien des services à base communautaire : Mesure dans laquelle les OSC participent à présenter les besoins, informations et services du marché du travail aux décideurs, et vice-versa.

	1	2	3	4	5	6	7	8	9	10	Je ne sais pas
1-Plus faible -> Plus élevé-10	○	○	○	○	○	○	○	○	○	○	○

* LM 5.4 La diversification des opportunités d'embauche : Mesure dans laquelle les OSC et le secteur privé travaillent en coordination avec le gouvernement pour assurer une création d'emplois optimale et une participation hommes-femmes équitable sur le marché du travail.

	1	2	3	4	5	6	7	8	9	10	Je ne sais pas
1-Plus faible -> Plus élevé-10	○	○	○	○	○	○	○	○	○	○	○

* LM 5.5 Les droits humains et la qualité de la mise en œuvre : Mesure dans laquelle les OSC soutiennent des approches fondées sur le droit sur les lieux de travail.

	1	2	3	4	5	6	7	8	9	10	Je ne sais pas
1-Plus faible -> Plus élevé-10	○	○	○	○	○	○	○	○	○	○	○

* LM 5.6 Les jeunes : Mesure dans laquelle les OSC soutiennent des politiques, interventions et programmes pour l'emploi des jeunes spécifiquement.

	1	2	3	4	5	6	7	8	9	10	Je ne sais pas
1-Plus faible -> Plus élevé-10	○	○	○	○	○	○	○	○	○	○	○

* LM 5.7 Les programmes pour les femmes : Mesure dans laquelle les OSC soutiennent des politiques, services et programmes pour l'emploi des femmes spécifiquement.

	1	2	3	4	5	6	7	8	9	10	Je ne sais pas
1-Plus faible -> Plus élevé-10	○	○	○	○	○	○	○	○	○	○	○

* LM 5.8 Le plaidoyer/ la redevabilité : Mesure dans laquelle les OSC soutiennent la formation et le renforcement de compétences des membres de la communauté pour augmenter leur compréhension des politiques, processus et activités, et pour renforcer leurs capacités à identifier les problèmes liés au marché du travail, résoudre collectivement ces problèmes et mener des actions de plaidoyer plus ciblées pour combattre le chômage.

	1	2	3	4	5	6	7	8	9	10	Je ne sais pas
1-Plus faible -> Plus élevé-10	○	○	○	○	○	○	○	○	○	○	○

* LM 5.9 L'évaluation et le suivi dirigés par les OSC : Mesure dans laquelle les OSC évaluent, suivent et émettent des rapports sur l'efficacité des politiques et programmes pour améliorer la redevabilité au niveau des politiques.

	1	2	3	4	5	6	7	8	9	10	Je ne sais pas
1-Plus faible -> Plus élevé-10	○	○	○	○	○	○	○	○	○	○	○

* LM 5.10 Les partenariats entre OSC : Mesure dans laquelle les OSC ont formé des alliances nationales et des partenariats régionaux pour renforcer le marché du travail.

	1	2	3	4	5	6	7	8	9	10	Je ne sais pas
1-Plus faible -> Plus élevé-10	◯	◯	◯	◯	◯	◯	◯	◯	◯	◯	◯

LM 5.11. Commentaires : Veuillez utiliser cette section pour nous faire part de tout commentaire additionnel sur le niveau d'effort, les défis ou les succès du pays autour des organisations de la société civile dans le secteur du marché du travail. Si vous avez répondu « Je ne sais pas » à l'une des questions précédentes, veuillez utiliser cette section pour expliquer pourquoi.

JOHNS HOPKINS
BLOOMBERG SCHOOL
of PUBLIC HEALTH

Bill & Melinda Gates Institute for Population and Reproductive Health

Enquête de l'Indice des efforts pour le dividende démographique

Module : Résilience et durabilité du secteur

Étant donné l'impact sanitaire et socioéconomique de la pandémie de COVID-19, et son impact probable sur la progression vers le DD, cet indice des efforts pour le DD intègre des questions pour évaluer la résilience et la durabilité des systèmes dans les secteurs clés du DD. Les éléments couverts par les questions liées à la pandémie de COVID-19 ci-dessous relèvent des dimensions clés des systèmes résilients et visent à apporter des informations déterminantes sur le potentiel d'une réponse efficace aux menaces de maladies infectieuses émergentes et autres crises de santé publique.
Veuillez noter chaque élément sur une échelle de 1 à 10, 1 étant le score le plus faible (faible état/capacité) et 10 le plus élevé (très bon état/capacité).

* Dimension physique : structures, équipements, états du système et capacités.

	1	2	3	4	5	6	7	8	9	10	Je ne sais pas
LM-M1 - Plan/Préparation - État et capacité de l'équipement, du personnel et de la structure du secteur du marché du travail avant la crise. (1 = faible état/capacité ; 10 = très bon état/capacité)	◯	◯	◯	◯	◯	◯	◯	◯	◯	◯	◯
LM-M2 - Absorption – Mesure dans laquelle le secteur du marché du travail a reconnu la pandémie de COVID-19 et pu continuer à maintenir un niveau d'emploi et une productivité économique optimale.	◯	◯	◯	◯	◯	◯	◯	◯	◯	◯	◯

	1	2	3	4	5	6	7	8	9	10	Je ne sais pas
LM-M3 - Récupération – Mesure dans laquelle le secteur du marché du travail été capable d'induire des changements pour récupérer un niveau de fonctionnalité pré-COVID-19.	○	○	○	○	○	○	○	○	○	○	○
LM-M4 - Récupération – Gestion du financement et des bailleurs de fonds : Mesure dans laquelle il existe des mécanismes établis de protection sociale (et autres formes de stabilisation des revenus) pour les travailleurs sans protection (ex : les travailleurs indépendants, qui non pas accès au congé payé.)	○	○	○	○	○	○	○	○	○	○	○
LM-M5 - Adaptation – Gestion du financement et des bailleurs de fonds : Mesure dans laquelle des réformes institutionnelles et politiques ont été initiées pour renforcer la résilience par une approche déterminée par la demande à travers des systèmes de protection sociale à grande échelle pouvant agir comme stabilisateurs économiques.	○	○	○	○	○	○	○	○	○	○	○

* Dimension informatique : création, gestion, stockage et utilisation des données.

	1	2	3	4	5	6	7	8	9	10	Je ne sais pas
LM-M6 - Plan/Préparation – Mesure dans laquelle les données liées au marché du travail étaient suffisamment préparées, présentées, analysées et stockées avant la crise.	○	○	○	○	○	○	○	○	○	○	○
LM-M7 - Absorption – Connaissance : Mesure dans laquelle le secteur du marché du travail a pu conduire des évaluations en temps réel de son état de fonctionnement, en anticipation des pertes en cascade de la productivité du marché du travail.	○	○	○	○	○	○	○	○	○	○	○
LM-M8 - Mesure dans laquelle les données du marché du travail ont été utilisées pour suivre les progrès vers la récupération et anticiper des scenarii de récupération.	○	○	○	○	○	○	○	○	○	○	○
LM-M9 - Adaptation – Mesure dans laquelle le secteur du marché du travail crée et améliore ses protocoles de stockage et utilisation des données en temps réel.	○	○	○	○	○	○	○	○	○	○	○

* Dimension cognitive : compréhension, modèles cognitifs, idées reçues, préjugés et valeurs.

	1	2	3	4	5	6	7	8	9	10	Je ne sais pas
LM-M10 - Planification/Préparation – Mesure dans laquelle le système de conception et les décisions opérationnelles du secteur du marché du travail étaient préparés à anticiper la crise.	○	○	○	○	○	○	○	○	○	○	○

	1	2	3	4	5	6	7	8	9	10	Je ne sais pas
LM-M11 - Absorption – Mesure dans laquelle le secteur du marché du travail a des protocoles de contingence suffisants et une gestion de crise proactive.	○	○	○	○	○	○	○	○	○	○	○
LM-M12 - Absorption - Leadership et management : Mesure dans laquelle le gouvernement garantit aux salariés/ travailleurs et leurs famille une protection contre les risques sanitaires de la pandémie de COVID-19.	○	○	○	○	○	○	○	○	○	○	○
LM-M13 - Absorption – Redevabilité : Niveau d'effort pour assurer une redevabilité optimale vis-à-vis des ressources allouées à la protection contre les risques sanitaires de la pandémie de COVID-19 et la récupération du marché du travail dans le cadre de la réponse pour mitiger les impacts du COVID-19.	○	○	○	○	○	○	○	○	○	○	○
LM-M14 - Récupération – Mesure dans laquelle les décisions sont orientées vers la récupération et communiquées aux membres de la communauté sur la base de données probantes pour promouvoir des comportements sûrs.	○	○	○	○	○	○	○	○	○	○	○

	1	2	3	4	5	6	7	8	9	10	Je ne sais pas
LM-M15 - Récupération – Connaissance/Conscience : Mesure dans laquelle le secteur établit une certaine confiance et un dialogue entre les employeurs et les salariés pour renforcer l'engagement d'action commune du gouvernement et promouvoir le dialogue social au niveau des entreprises.	○	○	○	○	○	○	○	○	○	○	○
LM-M16 - Adaptation – Mesure dans laquelle le secteur du marché du travail conçoit de nouvelles configurations systémiques, objectifs et critères de décision.	○	○	○	○	○	○	○	○	○	○	○
LM-M17 - Adaptation – Adaptatif : Mesure dans laquelle le gouvernement promeut des changements sur les lieux de travail pour protéger les travailleurs (ex : mesures de sécurité et gestes barrières, prévention de la discrimination et de l'exclusion, accès élargi au congé payé, etc.).	○	○	○	○	○	○	○	○	○	○	○

* Dimension sociale : interaction, collaboration et auto-synchronisation entre les personnes, les entités et les institutions.

	1	2	3	4	5	6	7	8	9	10	Je ne sais pas
LM-M18 - Planification /Préparation – Mesure dans laquelle une formation sur la gestion d'épidémies/crises a été menée et a permis de mettre à profit les réseaux sociaux, le capital social et les normes institutionnelles et culturelles avant la crise.	○	○	○	○	○	○	○	○	○	○	○

	1	2	3	4	5	6	7	8	9	10	Je ne sais pas
LM-M19 - Absorption – Mesure dans laquelle le personnel et les institutions sociales ont été accessibles et ont fait preuve d'ingéniosité dans leur réponse à l'épidémie/crise.	○	○	○	○	○	○	○	○	○	○	○
LM-M20 - Absorption – Autorégulation : Mesure dans laquelle les dirigeants nationaux avaient l'autorité d'effectuer des changements sectoriels en temps opportun à travers une infrastructure du marché du travail flexible.	○	○	○	○	○	○	○	○	○	○	○
LM-M21 - Récupération – Mesure dans laquelle le secteur du marché du travail est impliqué dans le partage de connaissances et d'équipes pour améliorer la récupération du système.	○	○	○	○	○	○	○	○	○	○	○
LM-M22 - Récupération – Diversité : Mesure dans laquelle le gouvernement facilite une récupération solide et rapide du marché du travail en diversifiant les politiques favorisant à la fois l'offre (production de biens et services) et la demande (consommation et investissement).	○	○	○	○	○	○	○	○	○	○	○
LM-M23 - Adaptation – Mesure dans laquelle des ajouts ou modifications sont apportés aux institutions, politiques, formations, programmes et à la culture du secteur du marché du travail.	○	○	○	○	○	○	○	○	○	○	○

	1	2	3	4	5	6	7	8	9	10	Je ne sais pas
LM-M24 - Adaptation - Leadership et management : Mesure dans laquelle il y a eu un dialogue tripartite fluide entre le gouvernement, les organisations des travailleurs/salariés et celles des employeurs.	○	○	○	○	○	○	○	○	○	○	○

* LM M-25 Temporalité : Mesure de la rapidité de la réaction du gouvernement –sa communication et mise en œuvre des mesures- pour mitiger l’impact immédiat et sur le long terme de la pandémie de COVID-19. Cette question porte sur la riposte nationale dans son ensemble, et non la réponse spécifique de votre secteur.

	1	2	3	4	5	6	7	8	9	10	Je ne sais pas
1-Plus faible -> Plus élevé-10	○	○	○	○	○	○	○	○	○	○	○

LM M-26 Commentaires : Utilisez cette section pour apporter tout commentaire additionnel sur la résilience et la durabilité des systèmes du marché du travail en vous référant à votre expérience de la pandémie de COVID-19. Si vous avez répondu « Je ne sais pas » à l’une des questions, utilisez cette section pour expliquer pourquoi.

JOHNS HOPKINS
BLOOMBERG SCHOOL
of PUBLIC HEALTH

Bill & Melinda Gates Institute for Population and Reproductive Health

Enquête de l'Indice des efforts pour le dividende démographique

Questionnaire sur la gouvernance et les institutions économiques (GIE)

Répondez aux questions suivantes en fonction de votre expérience/ expertise dans ce secteur spécifique.

Afin d'obtenir une représentation synthétique des efforts nationaux pour le dividende démographique, notez les éléments suivants sur une échelle de 1 à 10, 1 étant le score le plus faible (efforts très faibles ou quasi-inexistants) et 10 le plus élevé (efforts robustes). Le cas échéant, si une politique ou activité n'existe pas, répondez 0.

Donnez un score à chaque élément. Toutes les réponses seront enregistrées au format illustré ci-dessous :

Effort le plus faible	1	2	3	4	5	6	7	8	9	10	Effort le plus élevé	Je ne sais pas

JOHNS HOPKINS BLOOMBERG SCHOOL of PUBLIC HEALTH

Bill & Melinda Gates Institute for Population and Reproductive Health

Enquête de l'Indice des efforts pour le dividende démographique

Domaine 1. Politique/ Prise de décision

* GEI 1.1 Les valeurs démocratiques : Mesure dans laquelle le gouvernement a ratifié, appliqué à son contexte national et mis en œuvre les instruments régionaux sur les valeurs communes, y compris ceux liés à la démocratie, les élections, la gouvernance et le droit à la santé.

	1	2	3	4	5	6	7	8	9	10	Je ne sais pas
1-Plus faible -> Plus élevé-10	○	○	○	○	○	○	○	○	○	○	○

* GEI 1.2 Le suivi des valeurs démocratiques : Mesure dans laquelle le gouvernement a créé des mécanismes de rapport régulier et périodique sur la mise en œuvre des instruments régionaux de valeurs communes à travers les mécanismes existants tels que les revues périodiques universelles du statut des instruments internationaux ou régionaux, ou à travers des entités indépendantes. (Cela peut prendre la forme de rapports réguliers sur le statut de la mise en œuvre).

	1	2	3	4	5	6	7	8	9	10	Je ne sais pas
1-Plus faible -> Plus élevé-10	○	○	○	○	○	○	○	○	○	○	○

* GEI 1.3 L'affirmation de la participation démocratique des jeunes : Mesure dans laquelle le gouvernement a explicitement abrogé toutes les lois et contraintes discriminatoires empêchant la pleine participation des jeunes au processus électoral à travers des lois électorales et constitutions inclusives. (Cela peut inclure le développement et/ou renforcement des programmes de leadership des jeunes et/ou le renforcement de compétences en management et leadership).

	1	2	3	4	5	6	7	8	9	10	Je ne sais pas
1-Plus faible -> Plus élevé-10	○	○	○	○	○	○	○	○	○	○	○

* GEI 1.4. La suppression des barrières légales auxquelles se heurtent les jeunes : Mesure dans laquelle le gouvernement a proscrit toutes les lois posant des barrières au plein exercice et à la pleine jouissance des droits fondamentaux des jeunes à participer pleinement aux processus de gouvernance démocratique au niveau continental, régional, national et communautaire/à la base. (Cela pourrait aussi inclure le soutien de l'intégration des questions de droits et de santé des jeunes et adolescents à l'agenda du gouvernement sur la croissance et la transformation du pays).

	1	2	3	4	5	6	7	8	9	10	Je ne sais pas
1-Plus faible -> Plus élevé-10	○	○	○	○	○	○	○	○	○	○	○

* GEI 1.5 La politique de la jeunesse : Mesure dans laquelle la politique de la jeunesse du pays reflète les paradigmes éducatifs, de formation et d'emploi les plus récents.

	1	2	3	4	5	6	7	8	9	10	Je ne sais pas
1-Plus faible -> Plus élevé-10	○	○	○	○	○	○	○	○	○	○	○

* GEI 1.6 L'affirmation de la participation à la vie démocratique/ révision de la loi coutumière : Mesure dans laquelle le gouvernement a examiné, révisé, amendé ou aboli toutes les lois, régulations, politiques, pratiques et coutumes ayant un effet discriminatoire sur les jeunes, particulièrement les filles et les jeunes filles, sans distinction aucune, s'est assuré que les dispositions des différents systèmes légaux soient conformes aux droits humains internationaux. (Cela doit impérativement inclure la protection contre les pratiques malfaisantes, telles que le mariage précoce ou forcé, la violence sexuelle et basée sur le genre [VBG], et la mutilation génitale féminine [MGF]).

	1	2	3	4	5	6	7	8	9	10	Je ne sais pas
1-Plus faible -> Plus élevé-10	○	○	○	○	○	○	○	○	○	○	○

* GEI 1.7 La participation des jeunes dans la prise de décision politique : Mesure dans laquelle le gouvernement veille à l'implication des jeunes leaders et à leur participation dans les processus de prise de décision politique, y compris la politique sur la jeunesse et l'éducation. (Cela peut inclure les efforts visant à établir un parlement ou une assemblée nationale de jeunes. Cela peut aussi inclure le soutien à la participation et au leadership des jeunes et adolescents dans la planification et les programmes de santé à tous les niveaux de gouvernance).

	1	2	3	4	5	6	7	8	9	10	Je ne sais pas
1-Plus faible -> Plus élevé-10	○	○	○	○	○	○	○	○	○	○	○

* GEI 1.8 L'État de droit : Mesure dans laquelle l'État de droit est formellement et légalement établi de manière équitable et mis en œuvre indépendamment par le pouvoir judiciaire.

	1	2	3	4	5	6	7	8	9	10	Je ne sais pas
1-Plus faible -> Plus élevé-10	○	○	○	○	○	○	○	○	○	○	○

* GEI 1.9 La transparence et la redevabilité : Mesure dans laquelle la législation nationale promeut la transparence et la redevabilité de toutes les branches et secteurs.

	1	2	3	4	5	6	7	8	9	10	Je ne sais pas
GEI 1.9.1 Mesure dans laquelle le public peut accéder aux informations législatives et aux registres sur les organisations publiques.	○	○	○	○	○	○	○	○	○	○	○
GEI 1.9.2 Mesure dans laquelle il existe des mécanismes de redevabilité rendant cette dernière obligatoire et des sanctions associées.	○	○	○	○	○	○	○	○	○	○	○

* GEI 1.10 La sécurité nationale : Mesure dans laquelle le pays/gouvernement est impliqué dans un conflit armé, qu'il soit international ou intérieur.

	1	2	3	4	5	6	7	8	9	10	Je ne sais pas
GEI 1.10.1 Mesure dans laquelle le pays souffre de tensions transfrontalières.	○	○	○	○	○	○	○	○	○	○	○
GEI 1.10.2 Mesure dans laquelle le pays contient des personnes internement déplacées et/ou des réfugiés.	○	○	○	○	○	○	○	○	○	○	○
GEI 1.10.3 Mesure dans laquelle les établissements éducatifs sont protégés et des opportunités d'apprentissage alternatives créées pour les enfants et les jeunes déplacées ou vivant en situation de conflit.	○	○	○	○	○	○	○	○	○	○	○

* GEI 1.11 Les droits : Mesure dans laquelle la constitution affirme certains droits : liberté d'expression, liberté d'association et d'assemblée, droits et libertés civiques, et protection contre la discrimination ethnique et religieuse ; et mesure dans laquelle la violation des droits humains par le gouvernement est improbable.

	1	2	3	4	5	6	7	8	9	10	Je ne sais pas
1-Plus faible -> Plus élevé-10	◯	◯	◯	◯	◯	◯	◯	◯	◯	◯	◯

* GEI 1.12 L'éducation et les valeurs démocratiques : Mesure dans laquelle les politiques, stratégies et programmes d'éducation promeuvent la liberté de penser, les valeurs démocratiques et l'éducation comme un droit fondamental/humain.

	1	2	3	4	5	6	7	8	9	10	Je ne sais pas
1-Plus faible -> Plus élevé-10	◯	◯	◯	◯	◯	◯	◯	◯	◯	◯	◯

* GEI 1.13 L'affirmation des environnements d'apprentissage sûrs : Mesure dans laquelle les politiques d'éducation garantissent des environnements d'apprentissage sûrs à travers la protection de l'enfance. (Cela peut se manifester par la protection de la sécurité des filles, l'interdiction des punitions corporelles, etc.).

	1	2	3	4	5	6	7	8	9	10	Je ne sais pas
1-Plus faible -> Plus élevé-10	◯	◯	◯	◯	◯	◯	◯	◯	◯	◯	◯

GEI 1.14 Commentaires : Utilisez cette section pour nous faire part de tout commentaire additionnel sur le niveau d'effort, les défis ou les succès du pays autour des politiques/ de la prise de décision dans le secteur de la gouvernance et des institutions économiques. Si vous avez répondu « Je ne sais pas » à l'une des questions précédentes, veuillez utiliser cette section pour expliquer pourquoi.

JOHNS HOPKINS
BLOOMBERG SCHOOL
of PUBLIC HEALTH

Bill & Melinda Gates Institute for Population and Reproductive Health

Enquête de l'Indice des efforts pour le dividende démographique

Domaine 2. Services ou programmes

* GEI 2.1 Renforcer les activités/ institutions pour la jeunesse : Mesure dans laquelle des efforts sont menés pour renforcer les formations, réseaux et organisations indépendantes de jeunes, y compris l'établissement de commissions indépendantes de jeunes et de syndicats d'étudiants au niveau national et infranational pour promouvoir les droits des jeunes.

	1	2	3	4	5	6	7	8	9	10	Je ne sais pas
1-Plus faible -> Plus élevé-10	○	○	○	○	○	○	○	○	○	○	○

* GEI 2.2 La nomination de jeunes leaders : Si et mesure dans laquelle un jeune représentant a été nommé pour diriger le plaidoyer et promouvoir la priorisation des questions concernant les jeunes (ex : éducation, formation et emploi) dans les espaces de prise de décision à différents niveaux, y compris au parlement.

	1	2	3	4	5	6	7	8	9	10	Je ne sais pas
1-Plus faible -> Plus élevé-10	○	○	○	○	○	○	○	○	○	○	○

* GEI 2.3 Les idéaux et aspirations des jeunes : Mesure dans laquelle les programmes sont conçus pour instituer le leadership des jeunes, et mesure dans laquelle les formations pour l'autonomisation des jeunes visent à approfondir les idéaux et aspirations culturelles des jeunes.

	1	2	3	4	5	6	7	8	9	10	Je ne sais pas
1-Plus faible -> Plus élevé-10	○	○	○	○	○	○	○	○	○	○	○

* GEI 2.4 Les services des jeunes : Mesure dans laquelle les programmes sont conçus pour répondre aux besoins spécifiques des jeunes. (Cela devrait prendre en compte le développement et la mise en œuvre de services de santé amis des jeunes et adolescents, entre autres programmes similaires).

	1	2	3	4	5	6	7	8	9	10	Je ne sais pas
1-Plus faible -> Plus élevé-10	◯	◯	◯	◯	◯	◯	◯	◯	◯	◯	◯

* GEI 2.5 L’implication des jeunes dans les initiatives nationales : Mesure dans laquelle des efforts sont menés pour renforcer les initiatives existantes du pays en matière d’engagement et de participation des jeunes (e*x : au niveau de l’Union africaine, cela peut inclure le Corps des Jeunes Volontaires de l’Union Africaine, les Clubs de Jeunes de l’Union Africaine, et les activités de AGA-YES, dont le comité consultatif de jeunes, les procès fictifs et le dialogue annuel des jeunes sur la démocratie, les droits de l’Homme et la gouvernance).* (Cela peut inclure l’implication des jeunes et des adolescents dans l’assistance technique apportée aux ministères et entités gouvernementales fédérales ou régionales compétentes pour l’élaboration de politiques, plans et programmes qui intègrent les questions de santé prioritaires affectant ce groupe).

	1	2	3	4	5	6	7	8	9	10	Je ne sais pas
1-Plus faible -> Plus élevé-10	◯	◯	◯	◯	◯	◯	◯	◯	◯	◯	◯

* GEI 2.6 La sécurité : Mesure dans laquelle la sécurité personnelle est garantie à travers des services/ activités de police fiables, contribuant à une perception générale positive de la sécurité des personnes dans les ménages, les communautés et en milieu scolaire. (Cela pourrait inclure la protection des jeunes contre tout type d’abus, négligence ou manque de respect dans l’utilisation des services de santé).

	1	2	3	4	5	6	7	8	9	10	Je ne sais pas
1-Plus faible -> Plus élevé-10	◯	◯	◯	◯	◯	◯	◯	◯	◯	◯	◯

* GEI 2.7 La participation : Mesure dans laquelle les fonctions du gouvernement impliquent la participation des citoyens, des organisations de la société civile et autres acteurs intersectoriels pour promouvoir des élections démocratiques et le pouvoir effectif de gouverner.

	1	2	3	4	5	6	7	8	9	10	Je ne sais pas
1-Plus faible -> Plus élevé-10	◯	◯	◯	◯	◯	◯	◯	◯	◯	◯	◯

* GEI 2.8 Le genre : Mesure dans laquelle les femmes participent à :

	1	2	3	4	5	6	7	8	9	10	Je ne sais pas
GEI 2.8.1 La gouvernance au niveau national	◯	◯	◯	◯	◯	◯	◯	◯	◯	◯	◯
GEI 2.8.2 La gouvernance au niveau infranational	◯	◯	◯	◯	◯	◯	◯	◯	◯	◯	◯
GEI 2.8.3 La gouvernance en milieu rural	◯	◯	◯	◯	◯	◯	◯	◯	◯	◯	◯
GEI 2.8.4 La gouvernance en milieu urbain	◯	◯	◯	◯	◯	◯	◯	◯	◯	◯	◯
GEI 2.8.5 Judiciaire	◯	◯	◯	◯	◯	◯	◯	◯	◯	◯	◯

* GEI 2.9 La promotion de l’égalité : Mesure dans laquelle la parité est promue par le gouvernement dans tous les aspects de la société. (Ceci peut inclure la parité et l’égalité dans l’éducation, devant la loi, dans le système de santé, à l’embauche et en termes de rémunération, entre autres facettes de la société).

	1	2	3	4	5	6	7	8	9	10	Je ne sais pas
1-Plus faible -> Plus élevé-10	◯	◯	◯	◯	◯	◯	◯	◯	◯	◯	◯

* GEI 2.10 La durabilité des opportunités économiques :

	1	2	3	4	5	6	7	8	9	10	Je ne sais pas
GEI 2.10.1 Mesure dans laquelle le gouvernement pourvoit et s'assure qu'un système de gestion publique solide soit réalisé.	◯	◯	◯	◯	◯	◯	◯	◯	◯	◯	◯
GEI 2.10.2 Mesure dans laquelle le gouvernement pourvoit et s'assure qu'un environnement commercial solide soit réalisé.	◯	◯	◯	◯	◯	◯	◯	◯	◯	◯	◯
GEI 2.10.3 Mesure dans laquelle le gouvernement pourvoit et s'assure qu'une infrastructure publique solide soit réalisée.	◯	◯	◯	◯	◯	◯	◯	◯	◯	◯	◯
GEI 2.10.4 Mesure dans laquelle le gouvernement pourvoit et s'assure qu'un secteur rural fort pour le développement soit réalisé.	◯	◯	◯	◯	◯	◯	◯	◯	◯	◯	◯

* GEI 2.11 Le développement humain :

	1	2	3	4	5	6	7	8	9	10	Je ne sais pas
GEI 2.11.1 Mesure dans laquelle le bien-être du public en général est adéquatement géré par le gouvernement (y compris, mais sans s'y limiter, les politiques et services de sécurité sociale, l'intégration socioéconomique et la protection sociale des jeunes, entre autres sujets). Cela peut aussi impliquer la mesure dans laquelle le gouvernement, ayant l'obligation de protéger, remplir et faire respecter les droits fondamentaux de santé, a donné la responsabilité de leur mise en œuvre à son cabinet ministériel.	○	○	○	○	○	○	○	○	○	○	○
GEI 2.11.2 Mesure dans laquelle le gouvernement promeut le développement de compétences de la population sur les lieux de travail.	○	○	○	○	○	○	○	○	○	○	○
GEI 2.11.3 Mesure dans laquelle le gouvernement promeut l'éducation, le développement de connaissances et des capacités de la population à travers l'école et des opportunités de formation professionnelle continue.	○	○	○	○	○	○	○	○	○	○	○
GEI 2.11.4 Mesure dans laquelle le gouvernement promeut la santé et le bien-être de la population.	○	○	○	○	○	○	○	○	○	○	○

GEI 2.12 Commentaires : Utilisez cette section pour nous faire part de tout commentaire additionnel sur le niveau d'effort, les défis ou les succès du pays autour des services ou programmes dans le secteur de la gouvernance et des institutions économiques. Si vous avez répondu « Je ne sais pas » à l'une des questions précédentes, veuillez utiliser cette section pour expliquer pourquoi.

JOHNS HOPKINS BLOOMBERG SCHOOL of PUBLIC HEALTH

Bill & Melinda Gates Institute for Population and Reproductive Health

Enquête de l’Indice des efforts pour le dividende démographique

Domaine 3. Plaidoyer

* GEI 3.1 L’autonomisation des jeunes : Mesure dans laquelle l’autonomisation des jeunes a été améliorée, potentiellement à travers l’intégration de l’éducation civique au programme d’éducation scolaire nationale, aux plateformes médiatiques et autres canaux pour inculquer les principes de l’État de droit, les droits humains, et les devoirs et responsabilités individuelles. (Cela peut comprendre l’inclusion de la santé des jeunes et des adolescents et des questions relatives au droit dans l’agenda de croissance et transformation du pays et dans les programmes scolaires).

	1	2	3	4	5	6	7	8	9	10	Je ne sais pas
1-Plus faible -> Plus élevé-10	○	○	○	○	○	○	○	○	○	○	○

* GEI 3.2 Le plaidoyer du gouvernement : Mesure dans laque le gouvernement plaide pour des améliorations au sein de ses propres structures et priorités. (Ces améliorations peuvent comprendre des avancées vers l’inclusion, les opportunités, les programmes et les droits des jeunes, ou autres concepts similaires dans différentes populations.).

	1	2	3	4	5	6	7	8	9	10	Je ne sais pas
1-Plus faible -> Plus élevé-10	○	○	○	○	○	○	○	○	○	○	○

* GEI 3.3 Le plaidoyer non-gouvernemental : Mesure dans laquelle les acteurs du gouvernement plaident pour des améliorations des structures et priorités du gouvernement. (Ces améliorations peuvent comprendre des avancées vers l’inclusion les opportunités, les programmes et les droits des jeunes, ou autres concepts similaires dans différentes populations.).

	1	2	3	4	5	6	7	8	9	10	Je ne sais pas
1-Plus faible -> Plus élevé-10	○	○	○	○	○	○	○	○	○	○	○

* GEI 3.4 La voix du plaidoyer : Mesure dans laquelle les plaideurs se font entendre dans les discussions gouvernementales.

	1	2	3	4	5	6	7	8	9	10	Je ne sais pas
1-Plus faible -> Plus élevé-10	◯	◯	◯	◯	◯	◯	◯	◯	◯	◯	◯

* GEI 3.5 La voix des secteurs clés du DD : Mesure dans laquelle le gouvernement et les parties prenantes sont engagés à établir des institutions durables pour assurer :

	1	2	3	4	5	6	7	8	9	10	Je ne sais pas
GEI 3.5.1 Bonne gouvernance	◯	◯	◯	◯	◯	◯	◯	◯	◯	◯	◯
GEI 3.5.2 Stabilité financière	◯	◯	◯	◯	◯	◯	◯	◯	◯	◯	◯
GEI 3.5.3 Programmes optimaux de planification familiale	◯	◯	◯	◯	◯	◯	◯	◯	◯	◯	◯
GEI 3.5.4 Programmes optimaux de santé maternelle et infantile	◯	◯	◯	◯	◯	◯	◯	◯	◯	◯	◯
GEI 3.5.5 Éducation de bonne qualité	◯	◯	◯	◯	◯	◯	◯	◯	◯	◯	◯
GEI 3.5.6 Marché du travail diversifié	◯	◯	◯	◯	◯	◯	◯	◯	◯	◯	◯

GEI 3.6 Commentaires : Utilisez cette section pour nous faire part de tout commentaire additionnel sur le niveau d’effort, les défis ou les succès du pays autour du plaidoyer dans le secteur de la gouvernance et des institutions économiques. Si vous avez répondu « Je ne sais pas » à l’une des questions précédentes, veuillez utiliser cette section pour expliquer pourquoi.

JOHNS HOPKINS
BLOOMBERG SCHOOL
of PUBLIC HEALTH

Bill & Melinda Gates Institute for Population and Reproductive Health

Enquête de l'Indice des efforts pour le dividende démographique

Domaine 4. Recherche

* GEI 4.1 La force des observatoires/parties prenantes/groupes de travail sur la gouvernance et les institutions économiques (GIE) : Mesure dans laquelle les parties prenantes de la gouvernance et des institutions économiques du pays (ex : groupe directif de parties prenantes, groupes de travail techniques, observatoire de la gouvernance, groupe de coordination ou facilitation) :

	1	2	3	4	5	6	7	8	9	10	Je ne sais pas
GEI 4.1.1 Ont un représentant du gouvernement, des institutions de formation, de la société civile, des organisations non gouvernementales et confessionnelles, des associations professionnelles, du secteur privé, etc.	○	○	○	○	○	○	○	○	○	○	○
GEI 4.1.2 Se réunissent régulièrement, émettent des rapports et recommandent des politiques aux directions des ministères compétents.	○	○	○	○	○	○	○	○	○	○	○
GEI 4.1.3 Ont un impact sur la PF dans le pays.	○	○	○	○	○	○	○	○	○	○	○

* GEI 4.2 La stratégie de recherche sur la gouvernance : Mesure dans laquelle les documents de planification nationale comprennent une stratégie/approche complète de recherche liée à la GIE.

	1	2	3	4	5	6	7	8	9	10	Je ne sais pas
1-Plus faible -> Plus élevé-10	○	○	○	○	○	○	○	○	○	○	○

* GEI 4.3 Les partenaires collectant des données sur la gouvernance, la finance et le développement :

	1	2	3	4	5	6	7	8	9	10	Je ne sais pas
GEI 4.3.1 Mesure dans laquelle la collecte des données est entreprise par des agences gouvernementales. (Cela peut comprendre les agences/bureaux de la statistique et/ou les ministères de l'économie/finances, éducation, santé, genre, etc.).	○	○	○	○	○	○	○	○	○	○	○
GEI 4.3.2 Mesure dans laquelle la collecte de données est entreprise par des institutions de recherche.	○	○	○	○	○	○	○	○	○	○	○
GEI 4.3.3 Mesure dans laquelle la collecte des données est entreprise par des chercheurs indépendants.	○	○	○	○	○	○	○	○	○	○	○

* GEI 4.4 La recherche thématique :

	1	2	3	4	5	6	7	8	9	10	Je ne sais pas
GEI 4.4.1 Mesure dans laquelle de la recherche est menée sur la sécurité et l'État de droit.	○	○	○	○	○	○	○	○	○	○	○
GEI 4.4.2 Mesure dans laquelle la participation politique et les droits humains sont mesurés.	○	○	○	○	○	○	○	○	○	○	○
GEI 4.4.3 Mesure dans laquelle de la recherche est menée sur les indicateurs de l'économie durable et est disséminée à la communauté.	○	○	○	○	○	○	○	○	○	○	○
GEI 4.4.4 Mesure dans laquelle de la recherche est menée sur le développement du capital humain (éducation, formation et emploi, etc.).	○	○	○	○	○	○	○	○	○	○	○

* GEI 4.5 La qualité/couverture des données :

	1	2	3	4	5	6	7	8	9	10	Je ne sais pas
GEI 4.5.1 Mesure dans laquelle le système statistique de routine fournit des informations périodiques sur la GIE.	○	○	○	○	○	○	○	○	○	○	○
GEI 4.5.2 Mesure dans laquelle le système statistique de routine fournit des informations périodiques fiables sur les besoins des populations de différentes communautés en matière de gouvernance.	○	○	○	○	○	○	○	○	○	○	○
GEI 4.5.3 Mesure dans laquelle des évaluations des systèmes de suivi et surveillance sont conduites et appliquées pour assurer la fiabilité des données.	○	○	○	○	○	○	○	○	○	○	○

* GEI 4.6 La qualité des institutions de recherche : Mesure dans laquelle le pays a la capacité de soutenir et maintenir des institutions de recherche qui développent des études et/ou collectent des données permettant de soutenir la bonne gouvernance, la stabilité de l'économie et la solidité des institutions publiques.

	1	2	3	4	5	6	7	8	9	10	Je ne sais pas
1-Plus faible -> Plus élevé-10	○	○	○	○	○	○	○	○	○	○	○

* GEI 4.7 L'évaluation : Mesure dans laquelle les statistiques, enquêtes et études sont utilisées par du personnel spécialisé pour générer des rapports sur la bonne gouvernance et les institutions financières

	1	2	3	4	5	6	7	8	9	10	Je ne sais pas
1-Plus faible -> Plus élevé-10	○	○	○	○	○	○	○	○	○	○	○

* GEI 4.8 L'utilisation des résultats d'évaluation par les gestionnaires de programmes : Mesure dans laquelle les gestionnaires locaux de programmes utilisent les conclusions de recherche et d'évaluations pour améliorer l'État de droit, stabiliser l'économie et renforcer les institutions en suivant les recommandations émises.

	1	2	3	4	5	6	7	8	9	10	Je ne sais pas
1-Plus faible -> Plus élevé-10	○	○	○	○	○	○	○	○	○	○	○

* GEI 4.10 La dissémination d'informations à d'autres entités chargées de la mise en œuvre des programmes : Mesure dans laquelle des informations sont partagées ou disséminées entre géographies et à différents niveaux (national, état/province/comté, localités/sous-comtés).

	1	2	3	4	5	6	7	8	9	10	Je ne sais pas
1-Plus faible -> Plus élevé-10	○	○	○	○	○	○	○	○	○	○	○

GEI 4.11 Commentaires : Utilisez cette section pour nous faire part de tout commentaire additionnel sur le niveau d'effort, les défis ou les succès du pays autour de la recherche sur la gouvernance et les institutions économiques. Si vous avez répondu « Je ne sais pas » à l'une des questions précédentes, veuillez utiliser cette section pour expliquer pourquoi.

JOHNS HOPKINS BLOOMBERG SCHOOL of PUBLIC HEALTH

Bill & Melinda Gates Institute for Population and Reproductive Health

Enquête de l’Indice des efforts pour le dividende démographique

Domaine 5. Organisations de la société civile (OSC)

* GEI 5.1 Le pouvoir des acteurs des OSC : Mesure dans laquelle les acteurs des OSC occupent des postes d’influence pour contribuer positivement à l’État de droit.

	1	2	3	4	5	6	7	8	9	10	Je ne sais pas
1-Plus faible -> Plus élevé-10	◯	◯	◯	◯	◯	◯	◯	◯	◯	◯	◯

* GEI 5.2 L’analyse budgétaire comme outil des OSC : Mesure dans laquelle les OSC utilisent l’analyse budgétaire comme un outil pour développer le plaidoyer pour la bonne gouvernance, la stabilité de l’économie et la consolidation des institutions publiques.

	1	2	3	4	5	6	7	8	9	10	Je ne sais pas
1-Plus faible -> Plus élevé-10	◯	◯	◯	◯	◯	◯	◯	◯	◯	◯	◯

* GEI 5.3 Le soutien aux services à base communautaire : Mesure dans laquelle les OSC participent au rapprochement des informations et services liés à la gouvernance avec les communautés.

	1	2	3	4	5	6	7	8	9	10	Je ne sais pas
1-Plus faible -> Plus élevé-10	◯	◯	◯	◯	◯	◯	◯	◯	◯	◯	◯

* GEI 5.4 Les droits humains et la qualité de la mise en œuvre : Mesure dans laquelle les OSC plaident et soutiennent des approches de gouvernance fondées sur le droit pour veiller à ce que les bénéficiaires de projets et programmes puissent voir des avancées selon leurs propres termes.

	1	2	3	4	5	6	7	8	9	10	Je ne sais pas
1-Plus faible -> Plus élevé-10	◯	◯	◯	◯	◯	◯	◯	◯	◯	◯	◯

* GEI 5.5 Les jeunes : Mesure dans laquelle les OSC aident les jeunes à participer à l'éducation, la formation, l'emploi et le leadership.

	1	2	3	4	5	6	7	8	9	10	Je ne sais pas
1-Plus faible -> Plus élevé-10	○	○	○	○	○	○	○	○	○	○	○

* GEI 5.6 Les programmes pour les femmes : Mesure dans laquelle les OSC veillent à ce que les politiques de GIE soutiennent la participation des femmes et des filles au leadership.

	1	2	3	4	5	6	7	8	9	10	Je ne sais pas
1-Plus faible -> Plus élevé-10	○	○	○	○	○	○	○	○	○	○	○

* GEI 5.7 Le plaidoyer/ redevabilité :

	1	2	3	4	5	6	7	8	9	10	Je ne sais pas
GEI 5.7.1 Mesure dans laquelle les OSC et les médias soutiennent la participation de la communauté dans la gouvernance et l'application de l'État de droit.	○	○	○	○	○	○	○	○	○	○	○
GEI 5.7.2 Mesure dans laquelle les médias soutiennent la participation de la communauté à la gouvernance ou au respect de l'État de droit.	○	○	○	○	○	○	○	○	○	○	○

* GEI 5.8 Les évaluations et le suivi menés par les OSC : Mesure dans laquelle les OSC évaluent, suivent et émettent des rapports sur l'efficacité des politiques et des mécanismes et structures de redevabilité du gouvernement.

	1	2	3	4	5	6	7	8	9	10	Je ne sais pas
1-Plus faible -> Plus élevé-10	○	○	○	○	○	○	○	○	○	○	○

* GEI 5.9 Les partenariats entre OSC : Mesure dans laquelle les OSC ont formé des alliances nationales et des partenariats régionaux pour renforcer leur position et potentiellement améliorer le leadership et les mécanismes de financement pour une bonne gouvernance et le renforcement institutionnel.

	1	2	3	4	5	6	7	8	9	10	Je ne sais pas
1-Plus faible -> Plus élevé-10	◯	◯	◯	◯	◯	◯	◯	◯	◯	◯	◯

GEI 5.10 Commentaires : Utilisez cette section pour nous faire part de tout commentaire additionnel sur le niveau d'effort, les défis ou les succès du pays autour des OSC dans le secteur de la gouvernance et des institutions économiques. Si vous avez répondu « Je ne sais pas » à l'une des questions précédentes, veuillez utiliser cette section pour expliquer pourquoi.

JOHNS HOPKINS
BLOOMBERG SCHOOL
of PUBLIC HEALTH

Bill & Melinda Gates Institute for Population and Reproductive Health

Enquête de l'Indice des efforts pour le dividende démographique

Module : Résilience et durabilité du secteur

Étant donné l'impact sanitaire et socioéconomique de la pandémie de COVID-19, et son impact probable sur la progression vers le DD, cet indice des efforts pour le DD intègre des questions pour évaluer la résilience et la durabilité des systèmes dans les secteurs clés du DD. Les éléments couverts par les questions liées à la pandémie de COVID-19 ci-dessous relèvent des dimensions clés des systèmes résilients et visent à apporter des informations déterminantes sur le potentiel d'une réponse efficace aux menaces de maladies infectieuses émergeantes et autres crises de santé publique.

Veuillez noter chaque élément sur une échelle de 1 à 10, 1 étant le score le plus faible (faible état/capacité) et 10 le plus élevé (très bon état/capacité).

* Dimension physique : structures, équipements, états du système et capacités.

	1	2	3	4	5	6	7	8	9	10	Je ne sais pas
GEI-M1 – Planification/Préparation : État et capacité de l'équipement, du personnel et de la structure du secteur GIE avant la crise. (1 = faible état/capacité ; 10 = très bon état/capacité)	○	○	○	○	○	○	○	○	○	○	○
GEI-M2 - Absorption - Mesure dans laquelle le système du secteur GIE a reconnu la pandémie de COVID-19 et pu maintenir sa fonctionnalité.	○	○	○	○	○	○	○	○	○	○	○

	1	2	3	4	5	6	7	8	9	10	Je ne sais pas
GEI-M3 - Absorption – Intégration : Niveau d'intégration des services et collaborations institutionnelles pour assurer la continuité de l'accès aux services essentiels pendant la crise COVID-19.	○	○	○	○	○	○	○	○	○	○	○
GEI-M4 - Récupération – Mesure dans laquelle les institutions économiques et gouvernementales ont été capables de générer des changements pour récupérer un niveau de fonctionnalité pré-COVID-19.	○	○	○	○	○	○	○	○	○	○	○
GEI-M5 - Récupération – Gestion du financement et des bailleurs de fonds : Banque nationale et soutien des bailleurs de fonds pour assurer la disponibilité de liquidités pendant la crise COVID-19.	○	○	○	○	○	○	○	○	○	○	○
GEI-M6 - Adaptation – Mesure dans laquelle des changements ont été apportés pour améliorer la résilience du système et de l'infrastructure du secteur GIE.	○	○	○	○	○	○	○	○	○	○	○

* Dimension informatique : création, gestion, stockage et utilisation des données.

	1	2	3	4	5	6	7	8	9	10	Je ne sais pas
GEI-M7 - Plan/Préparation – Mesure dans laquelle les données du secteur GIE étaient suffisamment préparées, présentées, analysées et stockées avant la crise.	○	○	○	○	○	○	○	○	○	○	○
GEI-M8 - Absorption – Connaissance : Niveau de connaissance/conscience des menaces causées par la pandémie de COVID-19 dans le gouvernement central.	○	○	○	○	○	○	○	○	○	○	○
GEI-M9 - Récupération – Mesure dans laquelle les données de GIE ont été utilisées pour suivre le progrès vers la récupération et anticiper des scenarii de récupération.	○	○	○	○	○	○	○	○	○	○	○
GEI-M10 - Adaptation – Mesure dans laquelle le système du secteur GIE crée et améliore actuellement ses protocoles de stockage et d'utilisation des données en temps réel.	○	○	○	○	○	○	○	○	○	○	○

* Dimension cognitive : compréhension, modèles cognitifs, idées reçues, préjugés et valeurs.

	1	2	3	4	5	6	7	8	9	10	Je ne sais pas
GEI-M11 - Planification/Préparation – Mesure dans laquelle le système du secteur GIE et les décisions opérationnelles étaient préparés à anticiper la crise	○	○	○	○	○	○	○	○	○	○	○

	1	2	3	4	5	6	7	8	9	10	Je ne sais pas
GEI-M12 - Absorption – Mesure dans laquelle la réponse du secteur GIE a des protocoles de contingence suffisants et une gestion de crise proactive.	○	○	○	○	○	○	○	○	○	○	○
GEI-M13 - Absorption – Redevabilité : Niveau d'effort pour assurer une redevabilité optimale vis-à-vis des ressources allouées pour soutenir la gouvernance dans le cadre de la réponse à la pandémie de COVID-19.	○	○	○	○	○	○	○	○	○	○	○
GEI-M14 - Récupération – Mesure dans laquelle les décisions sont orientées vers la récupération et communiquées aux communautés sur la base de données probantes pour promouvoir des comportements sûrs.	○	○	○	○	○	○	○	○	○	○	○
GEI-M15 - Récupération – Connaissance/Conscience : Mesure dans laquelle le secteur de la PF établit une certaine confiance avec le public à travers sa communication et le dialogue pour la prise de décision et l'action politique fondées sur des données probantes.	○	○	○	○	○	○	○	○	○	○	○
GEI-M16 - Adaptation – Mesure dans laquelle le système du secteur GIE conçoit de nouvelles configurations systémiques, objectifs et critères de décision.	○	○	○	○	○	○	○	○	○	○	○
GEI-M17 - Adaptation – Adaptatif : Niveau d'adaptation des systèmes financiers et des services sociaux pour assurer et faciliter l'accès aux plateformes technologiques pendant la pandémie de COVID-19.	○	○	○	○	○	○	○	○	○	○	○

* Social dimension: interaction, collaboration and self-synchronization between people, entities and institutions.

	1	2	3	4	5	6	7	8	9	10	Je ne sais pas
GEI-M18 - Planification /Préparation – Mesure dans laquelle une formation sur la gestion d'épidémies/crises a été menée et a permis de mettre à profit les réseaux sociaux, le capital social et les normes institutionnelles et culturelles avant la crise.											
GEI-M19 - Absorption – Mesure dans laquelle le personnel et les institutions sociales ont été accessibles et ont fait preuve d'ingéniosité dans leur réponse à l'épidémie/crise.											
GEI-M20 - Absorption - Self-regulation: Extent to which national leaders had the authority to effect timely sectoral changes through an infrastructure flexible.											
GEI-M21 - Récupération – Mesure dans laquelle le secteur GIE est impliqué dans le partage de connaissances et d'équipes pour améliorer la récupération du système.											
GEI-M22 - Récupération – Diversité : Niveau d'engagement d'une équipe multidisciplinaire pour mitiger les effets de la pandémie de COVID-19 au niveau national et infranational.											
GEI-M23 - Adaptation - Leadership et management : Gérance et gestion démontrées par le gouvernement en réponse à la pandémie de COVID-19 au niveau national.											

* GEI M-24 Temporalité : Mesure de la rapidité de la réaction du gouvernement –sa communication et mise en œuvre des mesures- pour mitiger l'impact immédiat et sur le long terme de la pandémie de COVID-19. *Cette question porte sur la riposte nationale dans son ensemble, et non la réponse spécifique de votre secteur.*

	1	2	3	4	5	6	7	8	9	10	Je ne sais pas
1-Plus faible -> Plus élevé-10	◯	◯	◯	◯	◯	◯	◯	◯	◯	◯	◯

GEI M-25 Commentaires : Utilisez cette section pour apporter tout commentaire additionnel sur la résilience et la durabilité des systèmes nationaux de gouvernance et des institutions économiques en vous référant à votre expérience de la pandémie de COVID-19. Si vous avez répondu « Je ne sais pas » à l'une des questions, utilisez cette section pour expliquer pourquoi.

BMJ Open

A new demographic dividend effort index (DDEI): results from an experts' survey from six sub-Saharan African countries.

Journal:	*BMJ Open*
Manuscript ID	bmjopen-2021-059937.R1
Article Type:	Original research
Date Submitted by the Author:	19-Jul-2022
Complete List of Authors:	Rusatira, Jean Christophe; Johns Hopkins University Bloomberg School of Public Health, PHFR<br>Cardona, Carolina; Johns Hopkins University Bloomberg School of Public Health, Population Family and Reproductive Health<br>Martinez-Baack, Michelle ; Johns Hopkins University Bloomberg School of Public Health, PHFR<br>Rimon, Jose; Johns Hopkins University Bloomberg School of Public Health, Population, Family and Reproductive Health<br>Ahmed, Saifuddin; Johns Hopkins University Bloomberg School of Public Health, Population, Family And Reproductive Health
**Primary Subject Heading**:	Global health
Secondary Subject Heading:	Global health, Health policy
Keywords:	PUBLIC HEALTH, HEALTH ECONOMICS, Demography < TROPICAL MEDICINE, STATISTICS & RESEARCH METHODS, International health services < HEALTH SERVICES ADMINISTRATION & MANAGEMENT

SCHOLARONE™
Manuscripts

I, the Submitting Author has the right to grant and does grant on behalf of all authors of the Work (as defined in the below author licence), an exclusive licence and/or a non-exclusive licence for contributions from authors who are: i) UK Crown employees; ii) where BMJ has agreed a CC-BY licence shall apply, and/or iii) in accordance with the terms applicable for US Federal Government officers or employees acting as part of their official duties; on a worldwide, perpetual, irrevocable, royalty-free basis to BMJ Publishing Group Ltd ("BMJ") its licensees and where the relevant Journal is co-owned by BMJ to the co-owners of the Journal, to publish the Work in this journal and any other BMJ products and to exploit all rights, as set out in our licence.

The Submitting Author accepts and understands that any supply made under these terms is made by BMJ to the Submitting Author unless you are acting as an employee on behalf of your employer or a postgraduate student of an affiliated institution which is paying any applicable article publishing charge ("APC") for Open Access articles. Where the Submitting Author wishes to make the Work available on an Open Access basis (and intends to pay the relevant APC), the terms of reuse of such Open Access shall be governed by a Creative Commons licence – details of these licences and which Creative Commons licence will apply to this Work are set out in our licence referred to above.

Other than as permitted in any relevant BMJ Author's Self Archiving Policies, I confirm this Work has not been accepted for publication elsewhere, is not being considered for publication elsewhere and does not duplicate material already published. I confirm all authors consent to publication of this Work and authorise the granting of this licence.

For peer review only

A new demographic dividend effort index (DDEI): results from an experts' survey from six sub-Saharan African countries.

Authors
Jean Christophe Rusatira*, Carolina Cardona*, Michelle Martinez-Baack*, Jose G. Rimon*, Saifuddin Ahmed*

* Department of Population, Family and Reproductive Health, Johns Hopkins Bloomberg School of Public Health

Corresponding Author
E-mail address: jcrusatira@jhu.edu
Department of Population, Family and Reproductive Health
Johns Hopkins Bloomberg School of Public Health
615 N. Wolfe St., Baltimore, MD 21030, USA

Objective: This study aimed at developing a tool to measure the extent of national efforts in policies, services, research, and programs implemented to cultivate and harness the benefits of a potential demographic dividend (DD) in six Sub-Saharan African countries.

Design: The survey was self-administered online using the SurveyMonkey platform. The survey questionnaire covered six key sectors: good governance and economic institutions, family planning, maternal and child health, education, women's empowerment, and the labor market. Each sector-specific questionnaire was structured around five practice domains: policymaking, services and programs, advocacy, research, and civil society. Each item was scored from 1 to 10 and overall mean scores were generated with equal weights for each item across domains. Their corresponding 95% confidence intervals (CI) were calculated with a t-distribution. Internal consistency and construct validity were examined using Cronbach's alpha and confirmatory factor analyses, respectively.

Setting: Sub-Saharan Africa

Participants: A total of 440 knowledgeable informants from six countries; namely, Ethiopia (73), Kenya (69), Nigeria (67), Rwanda (54), Senegal (81), and Tanzania (96).

Results: The results revealed overall scores that ranged between 5.4 (95% CI: 5.1-5.8) in Ethiopia and 7.7 (95% CI: 7.5-7.9) in Rwanda. The disaggregated scores by sector revealed low scores in most countries in labor market and women's empowerment. Across domains, sectors and countries, item-specific scores revealed negligible to substantial heterogeneities. Across the countries and sectors, the DDEI has high Cronbach's alpha values in most domains (>0.8).

Conclusion: The Demographic Dividend Effort Index scores highlight important gaps in key health and development sectors. Our validation examinations suggest that the DDEI is reliable and valid for measuring the efforts in key DD sectors. The DDEI can serve as a self-evaluation tool for local actors and may complement existing quantitative tools such as the Global Gender Gap and the Human Capital Index.

For peer review only - http://bmjopen.bmj.com/site/about/guidelines.xhtml

Strengths and limitations of the study

- The study questionnaire was developed using existing effort indices and integrated inputs from local partners working in different health and development sectors.
- The study collected data from knowledgeable informants from six countries that are in the early stages of their demographic transition to explore the applicability and utility of a demographic dividend effort index.
- The Demographic Dividend Effort Index revealed overall high reliability and validity for measuring the progress in six key demographic dividend sectors across the six countries.
- The heterogeneity testing revealed substantial heterogeneity across sectors in Rwanda, Senegal, and Tanzania.
- The study relied on expert opinions working in different sectors in respective countries, which may be prone to bias and may not be comparable across countries.

For peer review only

For peer review only - http://bmjopen.bmj.com/site/about/guidelines.xhtml

Background

The reduction of births and infant deaths during the third stage of the demographic transition produces changes in the age structure of the population.[1] These changes present countries with an opportunity to experience a demographic dividend (DD) characterized by economic growth, sustainable development, and social change.[2-4] These demographic changes can spur economic growth by increasing the number of working-age individuals compared to the number of young dependents. However, favorable policies and investments are required for countries to harness the DD opportunity presented by such population age structure changes.

Across the Sub-Saharan African (SSA) region, more than three in four countries are still in the early stages of their demographic transition. They are projected not to reach the peak of the transition, where the DD occurs, until 2050.[5] Although this transition started more than 50 years ago, with declines in fertility levels from an average of 6.8 children per woman to an average of 4.8 children by the mid-2010s, it has been slower compared to other continents.[2] Currently, working-age individuals represent about 60% of the population, and this proportion is expected to keep increasing in the coming few decades.[6] These shifting population dynamics present a unique opportunity for SSA countries to grow their economies and break the cycle of poverty and poor health that has become chronic in the majority of these countries.

Previous literature has identified that, in Asian countries, the DD was responsible for around one-third of their economic growth between the 1960s and 1990s.[7 8] These experiences have sparked an interest among African countries. Over the last decade, countries in the region have embarked on the DD development agenda. The majority of the African Union (AU) member states have developed their DD roadmaps and strategic plans.[9] However, there is no standard tool to measure the extent of national efforts in policies, services, research, advocacy, and programs implemented to cultivate, realize, and harness the benefits of a potential DD in different SSA countries.

The AU and member states recognize that the DD is a key component to achieving sustainable socio-economic growth. In 2017, the AU established the theme of the year as "Harnessing the Demographic Dividend Through Investments in Youth". This theme generated momentum for the DD across the continent, particularly after the development of the AU DD roadmap. The roadmap identifies labor, education, health, governance, and youth empowerment as five fundamental pillars of the DD.[9] Cardona and colleagues further expanded these pillars based on their findings from a systematic literature review that highlighted six key sectors for cultivating and capitalizing on the DD potential in SSA.[10] These sectors are good governance and economic institutions (GGEI), family planning (FP), maternal and child health (MCH), education (ED), women's empowerment (WE), and the labor market (LM).

A central feature to enable a favorable policy environment, and attain a DD, is good governance and strong economic institutions. Governments have the power to attract foreign direct investments to create jobs and contribute to the economy. Strong financial institutions can promote a culture of saving, supported by favorable interest rates to fuel development. Bloom and colleagues have previously documented the correlation between economic stability, institutional quality and economic growth and have evidenced that unstable countries miss out on their DD opportunities.[2 11 12]

Political and economic stability create an opportunity for a more prosperous community through successful health and development programs. Successful FP programs have been proven to improve

For peer review only - http://bmjopen.bmj.com/site/about/guidelines.xhtml

women's participation in the workforce and have been identified as key interventions to reduce maternal and child mortality due to their impact on birth spacing and fertility levels.[13-16] The reduction of infant mortality is also intertwined with the reduction in fertility and is directly associated with the reduction in maternal mortality. Conley and colleagues evidenced that a reduction of 0.5-0.9 points in the total fertility rate (TFR) was associated with a fall of 50 deaths per 1,000 live births in the child mortality rate.[17] This association is concordant with Becker's theory about the quantity-quality trade-off of children, which predicts that parental investments per child increase as fertility levels decline.[18] As families become smaller, more resources are freed to support children's development and the household as a whole.[15 19]

Related to the quantity-quality trade-off, investments in human capital per child increase as fertility lowers, which can accelerate the economy. Ahmed and colleagues found a potential additional 22.4% increase in GDP per capita in SSA from an increase of just 13% in education attainment between 2011-2030 which eventually increases the availability of skilled workers[20]. Equitable access to quality education for boys and girls is also necessary for countries to generate skilled workers that can later support their economies. Moreover, the education of girls plays an important role in empowering women, who can later enter the labor force and spark socio-economic growth in SSA countries[21].

Africa's young population presents an opportunity for SSA countries to transform their economies. However, to seize this opportunity, countries may require different strategies depending on their demographic transition stage. On the one hand, countries that are still in the initial stage of their demographic transition also referred to as "pre-dividend" still need to accelerate their demographic transition and develop the human capital of the growing youth population.[19 22] These countries are characterized by a total fertility rate (TFR) of 4 or higher children per woman and a projected growing working-age population by 2030.[19 22] On the other hand, countries that are in the middle of their demographic transition, also referred to as "early-dividend", require smart investments in human capital development so that they can fill various job opportunities in the labor market. These countries are characterized by having a TFR of fewer than 4 children per woman and a projected growing working-age population by 2030.[19 22] For the socio-economic growth to happen, multisectoral efforts are required and countries need to generate jobs faster than their population growth, explore innovative strategies to transition young graduates to formal jobs, and facilitate job creation in the private sector.[23]

Over the last four decades, effort measurements have been proven to be reliable approaches to informing international health and development programs. Since the 1970s, the family planning effort index (FPEI) has provided reliable estimates to measure the strength of family planning programs in more than 90 countries and has been proven to be valid relative to family planning outcomes.[24-26] Building on the success of the FPEI, the same approach has been applied in HIV/AIDS programs and Maternal and child health which led to the development of the AIDS Program Effort Index (API) in more than 35 countries [27] and Maternal and Neonatal Program Index (MNPI) in more than 46 countries.[28] However, because health and development systems do not operate in silos, the successful implementation of FPEI, API and MNPI present an opportunity to develop a multidimensional index to measure DD progress in SSA.

This study aimed at developing an effort assessment index as a standard measure to quantify the nature and strength of in-country DD efforts in the key sectors identified as necessary to harness a DD. This index is meant to be used by national and global actors in the development sector working in five development domains; namely, policy, services and programs, advocacy, research, and civil society.

For peer review only - http://bmjopen.bmj.com/site/about/guidelines.xhtml

Methodology

Study design

Data were collected through an online survey that measured the efforts that countries are devoting to maximize their potential to reap the benefits of a DD. The survey was administered through an internet-based modality using the SurveyMonkey platform in six countries: Kenya, Senegal, Nigeria, Rwanda, Tanzania, and Ethiopia. These countries were selected because of where they stand in the demographic transition and convenience to successfully implement the study. The selection of countries sought to include both pre-dividend and early dividend countries[29] in which the study team had ongoing existing partners with the ability to engage a diverse pool of respondents. The survey(s) was administered in two waves to accommodate Coronavirus Disease 2019 (COVID-19) lockdowns in different countries. The first wave occurred from August to November 2020, and the second wave that covered Senegal alone took place from March to May 2021. The main language of the questionnaire was English (Appendix 1), and a French version (Appendix 2) was created for Senegal by a certified translator.

The questionnaires were developed for each of the six sectors recommended by Cardona and colleagues: FP, MCH, ED, WE, LM, and GGEI.[10] All questionnaires were structured around five domains: 1) policy; 2) services and programs; 3) advocacy; 4) research; and 5) civil society. These domains were selected based on prior similar effort indices implemented in various health and development areas[27 28 30 31]. These domains also corresponded to the areas of practice in the field and were confirmed by the local partners during the study design process. The Demographic Dividend Effort Index (DDEI) questionnaire was composed of 490 items divided across the six sectors (112 items for FP, 101 items for MCH, 61 items for ED, 71 items for WE, 68 items for LM, 77 items for GGEI). Where available, the development of each sector-specific questionnaire integrated validated questions from existing effort indices. For instance, the Family Planning Effort Index (FPEI) questionnaire[30] was integrated into the FP questionnaire. Similarly, specific questions from the Maternal and Neonatal Program Index (MNPI)[28], the Rwanda Governance Scorecard[32] and the AIDS Program Effort Index (API)[27] were integrated into different sector-specific questionnaires. Additional questions were drafted based on DD-favorable interventions for respective sectors as summarized in the wheel of prosperity by Cardona et al.[10] During the data collection, participants only ever received single surveys that pertained to their sector of expertise and never the entirety of the aggregate survey. Each item was scored on a scale of 1 to 10, from weaker (1) to stronger (10) perceived level of effort or strength.

The DDEI questionnaire was pilot-tested among 27 experts from Nigeria, Ethiopia, Tanzania, Senegal, Kenya and Rwanda in June-August 2020. This pilot study allowed local experts to provide their feedback and inputs to the questionnaire, which were integrated into the final revision. The local experts were requested to provide qualitative feedback on content, conciseness and completeness by sector and were requested to provide recommendations on changes to be made. The final questionnaire was shared with local project partners for review and approval before administering to the national level across all six countries. Each sector-specific questionnaire included items that assessed the presence and strength of policies, services and programs, advocacy efforts, research, and the involvement of civil society organizations in that sector. No personal identification or socio-demographic characteristics information was collected to limit the concern of being identifiable for the respondents and minimize information bias.

For peer review only - http://bmjopen.bmj.com/site/about/guidelines.xhtml

Study participants

The in-country partners identified the potential participants who were knowledgeable in respective domains and sectors in each country. In-country partners included the Centre d'Excellence Régional en Economie Générationnelle in Senegal, the Center for Communication Programs in Nigeria, the National Council for Population and Development in Kenya, the Tanzania Communication and Development Center, the Ethiopian Academy of Sciences, and the Centre for Integrated Development Research and Action in Rwanda. Participants were considered for the survey if they were regarded to have demonstrated expertise in at least one of the six sectors by the in-country partner. Expertise was defined by having worked in one of the health or development sectors at the national level as a policymaker, advocate, service provider, researcher, or member of a civil society organization. The local study partners had the best knowledge of potential respondents and led the data collection process locally.

As with other similar indices, sampling was necessarily purposive given the need to identify knowledgeable respondents. The design entailed the selection of 10 respondents for each sector. Whenever possible, diversity across the five domains of expertise was prioritized. As such, to reach 10 respondents per sector, each of the six sectors was designed to have two respondents with sector expertise in one of the five domains. These were exclusive classifications such that respondents could only be counted towards the completion of one sector-specific questionnaire. Overall, the sample size goal was 60 respondents per country. The survey was administered through an online modality "SurveyMonkey" in each country. Local partners in respective countries appointed one contact person to respond to any queries regarding the study. Requests to fill the surveys were sent through existing mailing lists of national technical working groups and up to three follow-up calls were made to all individuals for improving survey response rates.

This expected sample size was on the higher end of sample sizes for similar indices such as the Human Resources Effort Index (N = 16-28 per country), the FPEI (N = 1-12 per country), the API (N = 20-25 per country), and the MNPI (N=10-25).[27 28 30 31] These indices similarly surveyed across various sectors and, sometimes, domains of expertise. However, each of the existing indices was focused on one specific topic, while the DD required a multisectoral sample frame. As this study aimed for 60 respondents per country, considering an expected response rate similar to that of the FPEI of 49%, which was the highest among the other comparative indices, this study ultimately aimed for at least 30 respondents per country.

Patient and Public involvement

No patients or members of the public were involved in the research design, data analysis, or dissemination of the findings. Local researchers and policymakers contributed to the design of the questionnaire, the implementation of the survey and were involved in the interpretation of the results.

Analysis

The characteristics of the participants are presented by sector and domain of expertise at the country level (Table 1). Then, mean scores are computed for ease of interpretation and comparison across sectors per country (Figure 1). Their corresponding 95% confidence intervals (CI) were calculated with a *t*-distribution because the sample size was relatively small. The weighted overall country scores were computed (Table 2) to allow for equal contribution of each sector to the overall score irrespective of the number of respondents in the sector and mean scores per sector were disaggregated by domain of expertise for ease of interpretability and policy relevance (Table 3). To test the reliability and validity of our estimates, Cronbach's alpha and confirmatory factor analyses were utilized. Heterogeneity testing was

For peer review only - http://bmjopen.bmj.com/site/about/guidelines.xhtml

also done to assess the consistency of scores by domains in each country. Finally, the results from this analysis were shared with in-country partners, for review, policy translation and dissemination. The partners from respective countries conducted a series of multisectoral meetings and workshops, physical and virtual, to generate policy recommendations based on the results from the analysis. The data have been made available through the Harvard Dataverse open access repository.[33]

Cronbach's Alpha analysis

Because the data were collected separately by sector and country, Cronbach's alpha analysis was conducted by checking for "item-test" and "item-rest" correlations for each domain (five practice areas) for the six different sectors namely FP, MCH, ED, LM, WE and GGEI in each country. The item-rest correlations were used for examining the relevance of an item in the domain. The item-rest correlation indicated the association of an item with the total score on the other items, hence a score with items with higher item-rest correlations will result in a higher coefficient α for the test.[34] To ensure the best performance of the score with the most relevant items, we decided to exclude the items only if they had an "item-rest" correlation of <0.4 across all countries.[35] This is because alpha values may vary when variables are context-sensitive and in the countries where the same variables indicate high reliability, the fraction of a test score that is attributable to error eventually decreases. Hence, despite the low item-rest correlation in some countries, items with high item-rest correlation in at least one country were used to measure the construct in question.[36] The items that showed perfect pairwise correlation were excluded from the analysis.

Confirmatory factor analysis

Confirmatory factor analysis (CFA) was done using pooled data to allow for sufficient sample size using the "sem" Stata command that allows one item to be anchored with a value of 1.0 and the rest to have a loading value. The results were used to assess the fit of the retained data to measure the same underlying latent construct or domain. The expectation was that the loadings would not be too low, suggesting that the items we have included are appropriate and measuring the same constructs. Based on the results from this model, five indices were used to assess the goodness of fit of the data and whether the variables were used to measure the same construct. First, the Standardized Root Mean squared residual (SRMR) by which a good fit is indicated by a small value limited to 0.08. Second, the Coefficient of Determination (CD) by which, a model is said to have a good fit if it has a value close to 1.[37] Third, the Root Mean Square Error of Approximation (RMSEA), using its upper and lower bound; if the lower bound is below 0.05, the hypothesis that the fit is close is not rejected and if the upper bound is above 0.10, the hypothesis that the fit is poor is not rejected.[38] Fourth, Comparative Fit Index (CFI) by which a value close to 1 indicates a good fit.[38] Fifth, Tucker-Lewis index (TLI) by which a value close to 1 indicates a good fit.[37]

Heterogeneity testing

Given that the measures were collected from different groups, we formally checked for heterogeneity statistically. Statistical testing for heterogeneity among respondents was done using meta-analytical techniques with the "metan" Stata command. This analysis used pooled data by country to allow for sufficient sample size and assessment of heterogeneity across domains. To assess the overall extent of heterogeneity and whether it was adequate to compute summary estimates, the fixed-effects were compared to random-effect estimates. By using this method, substantial discrepancies between the estimates from the two models indicate considerable heterogeneity that can make summary estimates misleading.[39] To further assess heterogeneity, the chi-square test of heterogeneity and I-square (I^2) were used. The I^2 estimate of 0% to 40% indicate no/negligible heterogeneity, 30% to 60% indicate moderate heterogeneity, 50% to 90% indicate substantial heterogeneity and 75% to 100% show considerable heterogeneity.[40]

For peer review only - http://bmjopen.bmj.com/site/about/guidelines.xhtml

Results

Respondents

The total sample consisted of 440 respondents distributed as follows: Ethiopia (73), Kenya (69), Nigeria (67), Rwanda (54), Senegal (81), and Tanzania (96). The number of respondents, domain of expertise, and sectors differ across countries. In all countries, except Tanzania, the majority of respondents were from the public sector and the services and programs domains. The average completion rate was 82% and ranged from 78% in Tanzania to 92% in Rwanda. The intended number of participants per sector was achieved in all six countries.

Overall scores

Overall, the average scores were centered around the mid-point of 5 across sectors and domains. The scores were 5.4 (95% CI: 5.1-5.8) in Ethiopia, 5.8 (95% CI: 5.5-6.1) in Kenya, 5.5 (95% CI: 5.1-5.9) in Nigeria, 7.7 (95% CI: 7.5-7.9) in Rwanda, 6.3 (95% CI: 5.9-6.7) in Senegal, and 6.1 (95% CI: 5.8-6.4) in Tanzania.

Table 1: Sample Characteristics

	Ethiopia	Kenya	Nigeria	Rwanda	Senegal	Tanzania
	% (Obs.)	% (Obs.)	% (Obs.)	% (Obs.)	% (Obs.)	% (Obs.)
**Sector**						
Family Planning	23.3 (17)	24.6 (17)	26.9 (18)	13.0 (7)	8.6 (7)	39.6 (38)
Maternal and Child Health	20.5 (15)	24.6 (17)	11.9 (8)	16.7 (9)	19.8 (16)	19.8 (19)
Education	20.5 (15)	7.2 (5)	17.9 (12)	13.0 (7)	13.6 (11)	10.4 (10)
Women's Empowerment	11.0 (8)	14.5 (10)	13.4 (9)	29.6 (16)	21.0 (17)	13.5 (13)
Labor Market	12.3 (9)	18.8 (13)	11.9 (8)	16.7 (9)	14.8 (12)	8.3 (8)
Good Governance and Economic Institutions	12.3 (9)	10.1 (7)	17.9 (12)	11.1 (6)	22.2 (18)	8.3 (8)
**Organization**						
Public Sector	21.9 (16)	68.1 (47)	56.7 (38)	83.3 (45)	63.6 (49)	13.5 (13)
Private Sector	5.5 (4)	0.0 (0)	3.0 (2)	13.0 (7)	10.4 (8)	9.4 (9)
Non-governmental organization	27.4 (20)	27.5 (19)	28.4 (19)	1.9 (1)	20.8 (16)	66.7 (64)
University/research institute	41.1 (30)	1.4 (1)	3.0 (2)	0.0 (0)	3.9 (3)	2.1 (2)
Other (please specify)	4.1 (3)	2.9 (2)	9.0 (6)	1.9 (1)	1.3 (1)	8.3 (8)
**Area of Expertise**						
Policy/policymaking	13.7 (10)	20.3 (14)	26.9 (18)	14.8 (8)	17.3 (14)	7.3 (7)
Services or programs	21.9 (16)	58.0 (40)	37.3 (25)	18.5 (10)	66.7 (54)	52.1 (50)
Advocacy	8.2 (6)	11.6 (8)	13.4 (9)	14.8 (8)	8.6 (7)	25.0 (24)
Research	53.4 (39)	5.8 (4)	17.9 (12)	46.3 (25)	2.5 (2)	10.4 (10)
Civil Society	2.7 (2)	4.3 (3)	4.5 (3)	5.6 (3)	4.9 (4)	5.2 (5)
Obs.	73	69	67	54	81	96

The total scores were disaggregated by sector in Figure 1. The radar plots show that the sectors in most needs across countries were LM and WE. Both in Nigeria and Ethiopia, the average score for LM and WE struggled to reach the mid-point. However, this was not the case for Rwanda and Tanzania which scored highest in WE, 8.3 (95% CI: 8.1-8.6) and 6.9 (95% CI: 6.1-7.8), respectively. Ethiopia was the only country that struggled to reach the mid-point in the GGEI sector, 4.9 (95% CI: 4.1-5.7). By contrast, the MCH, ED, and FP sectors received scores above the mid-point across all countries. For example, MCH ranged from a low of 5.1 (95% CI: 3.8-6.4) in Nigeria to a high of 7.4 (95% CI: 7.1-7.6) in Rwanda.

For peer review only - http://bmjopen.bmj.com/site/about/guidelines.xhtml

Table 2. National overall and sector scores

	Ethiopia	Kenya	Nigeria	Rwanda	Senegal	Tanzania
**Sector**	Mean (95% CI) (Obs.)					
**Family Planning**	5.7 (5.3-6.1) (17)	5.9 (5.4-6.5) (17)	5.9 (5.1-6.7) (18)	6.4 (6.1-6.6) (7)	6.5 (5.1-7.8) (7)	6.0 (5.6-6.3) (38)
**Maternal and Child Health**	5.9 (4.7-7.2) (15)	6.2 (5.5-6.9) (18)	5.1 (3.8-6.4) (8)	7.4 (7.1-7.6) (9)	7.1 (6.5-7.7) (16)	6.8 (6.1-7.4) (19)
**Education**	5.3 (4.6-6.1) (15)	5.6 (3.8-7.4) (5)	5.9 (5.1-6.8) (12)	8.2 (7.3-9.1) (7)	5.9 (4.5-7.4) (11)	5.2 (4.1-6.4) (10)
**Women's Empowerment**	4.8 (3.4-6.1) (8)	5.1 (4.2-5.9) (10)	5.1 (4.1-6.1) (9)	8.3 (8.1-8.6) (16)	6.4 (5.6-7.3) (17)	6.9 (6.1-7.8) (13)
**Labor Market**	5.1 (4.4-5.9) (9)	5.4 (4.8-6.0) (13)	4.3 (3.1-5.5) (8)	7.6 (7.3-7.8) (9)	5.2 (3.8-6.6) (12)	5.8 (4.3-7.2) (8)
**Good Governance and Economic Institutions**	4.9 (4.1-5.7) (9)	6.5 (4.5-8.5) (6)	5.7 (4.7-6.7) (12)	7.8 (7.2-8.4) (6)	6.3 (5.7-6.9) (18)	5.4 (4.3-6.6) (8)
**Weighted Overall Score**	**5.4 (4.5-6.3) (73)**	**5.8 (4.9-6.8) (69)**	**5.5 (4.5-6.5) (67)**	**7.7 (7.3-8.1) (54)**	**6.3 (4.7-7.9) (81)**	**6.1 (5.4-6.8) (96)**

Even though the total and sectoral scores were around the midpoint across countries, there were several interesting item-specific variations across items within each sector. These are further explored in the next sections.

Family Planning

The policy domain was structured around 14 items. These items scored the political support for FP, pro-poor FP policies, financing for FP, presence of national FP policies and strategic plans, fertility attainment policies, and age for marriage policies. In all six countries, the average scores for the policy domain were above the mid-point, and the highest average score was registered in Rwanda, 7.6 (95% CI: 7.1-8.1). Across countries, respondents shared positive views about pro-FP policies but had different views about the support received from policymakers. Three respondents in Ethiopia noted that FP policies and programs are supported for their benefits to maternal and child health and FP tends to be politicized. In Kenya, one respondent commented that "Most politicians still encourage big families to get more voters hence undermining efforts made [towards] achieving [a] demographic dividend."

The services and programs domain included 22 items. These items assessed the strength of FP services and programs, community-based provision and equitable access to effective modern contraceptive methods, quality of services, targeted interventions including post-partum and for youth, demand creation, and public-private partnerships. The average scores for all countries were slightly above the mid-point, and the highest score was recorded in Senegal, 6.7 (95% CI:5.2-8.3). In all countries except Rwanda, respondents recorded low scores corresponding to less than 40% FP budget derived from in-country sources. The need for local funding of FP budgets to foster demand-creation was further emphasized by respondents from Nigeria.

The advocacy domain was composed of six items that assessed media coverage of FP programs, public support by policymakers, justification for the FP method, accessibility to social marketing programs and a special focus on special populations including adolescents, indigenous people and those with disabilities. Scores were above average in all countries and the highest was recorded in Senegal (6.8, 95% CI: 5.0-8.5). Mixed findings about FP political support were recorded, particularly in Kenya and Nigeria. In Kenya, three respondents noted that politicians are still encouraging large families to secure more voters

For peer review only - http://bmjopen.bmj.com/site/about/guidelines.xhtml

while in Nigeria politicians seem to be concerned with the growing population and offer their support to strengthen social marketing programs.

Table 3: National scores, by sector and domain

Country	Policy/Policymaking	Services or programs	Advocacy	Research	Civil Society Organizations (CSOs)
**Family Planning**					
Ethiopia	5.7 (5.2-6.2)	5.5 (5.0-6.0)	6.5 (5.7-7.3)	5.7 (5.0-6.3)	5.1 (4.0-6.1)
Kenya	5.9 (5.2-6.7)	5.8 (5.4-6.3)	6.0 (5.1-6.9)	5.6 (4.7-6.4)	5.9 (4.8-7.0)
Nigeria	5.9 (5.0-6.9)	5.8 (5.0-6.6)	6.3 (5.3-7.4)	6.3 (5.1-7.4)	6.7 (5.6-7.9)
Rwanda	7.6 (7.1-8.1)	5.6 (5.2-6.1)	6.3 (5.6-7.1)	6.8 (6.3-7.2)	7.0 (6.6-7.4)
Senegal	6.2 (5.0-7.4)	6.7 (5.2-8.3)	6.8 (5.0-8.5)	5.8 (3.4-8.2)	5.0 (3.9-6.0)
Tanzania	5.8 (5.5-6.2)	5.9 (5.5-6.3)	5.6 (5.0-6.2)	6.4 (6.0-6.9)	6.2 (5.5-6.9)
**Maternal and Child Health**					
Ethiopia	6.4 (5.1-7.7)	6.1 (4.7-7.6)	5.1 (3.5-6.7)	5.3 (3.7-6.8)	4.9 (3.4-6.4)
Kenya	6.4 (5.6-7.1)	6.4 (5.5-7.3)	5.7 (4.4-6.9)	6.0 (4.7-7.3)	4.9 (3.2-6.7)
Nigeria	5.4 (4.0-6.9)	5.2 (3.7-6.6)	5.2 (3.4-7.0)	4.8 (3.2-6.5)	4.6 (2.8-6.5)
Rwanda	7.5 (7.2-7.7)	7.3 (6.9-7.8)	7.3 (6.9-7.7)	7.5 (7.0-7.9)	7.5 (7.1-7.9)
Senegal	6.5 (5.9-7.2)	7.7 (7.1-8.4)	6.8 (5.7-7.8)	7.0 (6.0-7.9)	6.2 (5.0-7.4)
Tanzania	6.6 (5.9-7.2)	6.8 (6.0-7.6)	6.8 (5.9-7.7)	6.7 (5.9-7.6)	6.5 (5.6-7.4)
**Women's Empowerment**					
Ethiopia	5.3 (3.9-6.7)	4.7 (3.3-6.1)	5.2 (3.0-7.3)	3.8 (2.0-5.6)	5.0 (3.3-6.7)
Kenya	5.2 (4.1-6.3)	5.0 (4.1-5.9)	5.8 (4.8-6.8)	4.6 (3.7-5.4)	5.5 (4.2-6.7)
Nigeria	5.1 (4.0-6.1)	4.9 (3.6-6.2)	5.5 (4.2-6.8)	5.0 (3.7-6.3)	5.4 (4.2-6.5)
Rwanda	8.7 (8.4-9.0)	8.2 (7.8-8.6)	8.2 (7.8-8.7)	8.2 (7.8-8.5)	8.2 (7.8-8.6)
Senegal	6.6 (5.8-7.4)	6.7 (5.7-7.7)	6.5 (5.5-7.5)	5.7 (4.5-6.9)	6.0 (4.7-7.3)
Tanzania	6.8 (5.8-7.9)	6.9 (5.9-7.9)	7.2 (6.3-8.0)	6.7 (5.8-7.6)	8.1 (7.5-8.6)
**Education**					
Ethiopia	6.0 (5.5-6.5)	5.6 (4.6-6.6)	5.8 (4.6-7.0)	5.0 (4.3-5.8)	4.0 (3.1-4.9)
Kenya	5.5 (2.4-8.5)	5.2 (2.6-7.9)	6.0 (4.0-8.0)	5.8 (3.7-7.8)	5.3 (1.8-8.8)
Nigeria	6.1 (5.2-7.0)	5.9 (4.9-6.8)	6.6 (5.5-7.6)	5.8 (4.8-6.8)	5.9 (5.1-6.7)
Rwanda	8.2 (7.1-9.2)	8.1 (6.9-9.4)	8.0 (6.9-9.0)	8.4 (7.5-9.3)	8.1 (7.1-9.1)
Senegal	6.1 (4.7-7.4)	6.4 (4.9-8.0)	5.4 (4.1-6.6)	5.5 (3.7-7.4)	5.6 (3.6-7.6)
Tanzania	5.4 (4.2-6.6)	5.3 (3.6-6.9)	5.1 (3.7-6.5)	5.1 (3.6-6.6)	5.0 (3.3-6.6)
**Labor Market**					
Ethiopia	5.5 (4.5-6.5)	4.8 (4.1-5.4)	4.7 (4.1-5.4)	5.4 (4.4-6.4)	3.8 (2.3-5.4)
Kenya	5.9 (5.2-6.5)	5.5 (4.9-6.1)	5.0 (4.3-5.7)	5.1 (4.1-6.0)	5.4 (4.4-6.4)
Nigeria	4.3 (3.2-5.4)	3.7 (2.4-5.1)	4.3 (3.0-5.7)	4.4 (3.0-5.8)	5.0 (3.2-6.8)
Rwanda	7.8 (7.3-8.3)	7.5 (7.2-7.8)	7.2 (6.5-7.9)	7.7 (7.2-8.1)	7.2 (7.0-7.5)
Senegal	5.4 (4.1-6.7)	4.8 (3.4-6.3)	4.5 (2.8-6.2)	4.7 (3.2-6.3)	4.3 (2.7-5.8)
Tanzania	6.1 (4.4-7.8)	5.2 (3.5-7.0)	5.5 (3.8-7.2)	5.6 (3.9-7.4)	6.2 (4.0-8.4)
**Gov. and Economic Institutions**					
Ethiopia	5.6 (4.5-6.7)	5.2 (4.4-6.0)	5.1 (4.1-6.1)	4.6 (3.6-5.6)	4.7 (3.5-5.8)
Kenya	6.6 (4.3-8.8)	7.0 (4.5-9.4)	7.0 (4.5-9.5)	6.3 (3.5-9.0)	6.2 (3.5-9.0)
Nigeria	5.0 (3.8-6.3)	5.2 (4.0-6.3)	6.1 (4.8-7.4)	5.9 (5.0-6.9)	6.6 (5.5-7.7)
Rwanda	7.8 (6.6-9.0)	8.1 (7.4-8.7)	7.8 (7.0-8.6)	7.7 (7.0-8.3)	7.1 (6.8-7.5)
Senegal	6.2 (5.6-6.8)	6.2 (5.5-6.9)	6.6 (5.9-7.4)	6.6 (5.7-7.5)	6.4 (5.8-7.0)
Tanzania	5.2 (4.0-6.4)	5.3 (4.1-6.4)	5.9 (4.2-7.7)	5.1 (3.8-6.4)	6.4 (4.3-8.4)

For peer review only

The research domain had 11 items that assessed reporting mechanisms, data availability, capacity building, quality of data, the capacity of research institutions, data utilization and dissemination. The highest scores were recorded in Rwanda (6.8, 95% CI: 6.3-7.2) and the scores in all the other countries were above the average. There were also shared views across countries about data quality, reliance on Demographic and Health Surveys and low use of smaller studies conducted by local investigators. One respondent in Ethiopia noted a frustration: "I myself [have] conducted research on Factors Encouraging Women to Pursue Family Planning Method. Even though I have done the research by self-sponsoring the Oromia Regional Health Bureau cannot use it".

The civil society domain included 14 items that covered civil society organization (CSO) power, budget analysis skills to inform advocacy, facility-based services involvement, social franchising, community-based services involvement, and their active role in targeted programs, monitoring and accountability. Ethiopia and Senegal scored around the mid-point, and Rwanda scored the highest (7.0, 95% CI: 6.6-7.4). Respondents from all countries shared positive views on the crucial role played by the CSOs however the views on partnerships between the government and CSOs varied from one country to another. One respondent in Ethiopia noted that "As [the] government has [implemented a] health extension program at the community level, the CSOs ha[ve] closed [down] their community service. But [they are] supporting the gov[ernment] by building the capacity of the health extension workers". Another respondent in Tanzania commented "there are [a] number of CSOs active in Tanzania, especially in advocacy and accountability for FP. However, the government ha[s] suppressed their operationalization in doing advocacy to hold the government accountable for FP policies and commitments in place".

Maternal and Child Health

The policy domain comprised 13 items that assessed the presence and strength of policies around maternal and neonatal survival, universal access to antenatal and perinatal care, abortion, post-abortion care, financing, affordability, multisectoral approaches and private sector involvement. All countries' average scores were at the mid-point or above with Nigeria (5.4, 95% CI:4.0-6.9) and Rwanda (7.5, 95% CI:7.2-7.7) scoring lowest and highest respectively.

The services and programs domain had eight items that focused on obstetric care available at the primary and secondary healthcare levels, universal access to pregnancy-related services including antenatal care and management of major complications, newborn care, and FP integration. The highest score was recorded in Senegal (6.7, 95% CI:5.2-8.3) and the lowest in Nigeria (5.2, 95% CI:3.7-6.6). Despite high scores for a range of contraceptive methods for women, lower scores were recorded for the provision of the male sterilization method at the district hospitals in Kenya, Senegal, Nigeria, and Ethiopia. Respondents across countries shared the view of the essential role of health extension workers and common experience of stockouts of vaccines and contraceptives, limited skills of providers, resource allocation gaps, and challenges related to MCH policy effectiveness assessment.

The advocacy domain comprised six items that covered government messages, mass media, community-level media, educational material, self-review of maternal mortality cases and informing policies with new evidence. Rwanda (7.3, CI 6.9 -7.7) scored highest while Ethiopia (5.1, 95% CI: 3.5-6.7) and Nigeria (5.2, 95% CI: 3.4-7.0) scored lowest. Lower scores were consistent in Kenya, Ethiopia, and Kenya for the utilization of evidence to inform policies at health facility levels and self-review of maternal mortality cases.

For peer review only - http://bmjopen.bmj.com/site/about/guidelines.xhtml

The research domain included 11 items that focused on data mining infrastructure, research strategy, quality of data, the strength of research institutions and data utilization. The highest score was recorded in Rwanda (7.5. 95% CI: 7.0-7.9) and the lowest in Nigeria (4.8, 95% CI: 3.2-6.5). Respondents across countries consistently reported data quality challenges, insufficient skills and funding, and underutilization of available platforms. One respondent in Ethiopia noted an issue that "the available technology is not utilized to its maximum benefits e.g., DHIS [District Health Information Software] does not link and analyze vital registration, even though, there is a module for it to do so; data generated using different systems like Maternal Perinatal Death Surveillance Response is not linked in a central place to facilitate access to existing different maternal data sets."

The civil society domain is composed of 12 items that assessed the power and contribution of CSOs in advocacy, programs, services, research, monitoring, and evaluation. Overall, the highest average score was recorded in Rwanda (7.5, 95% CI: 7.1-7.9) and the lowest score was recorded in Nigeria (4.6, 95% CI: 2.8-6.5). A notable finding on item-specific scores was that in Kenya, Nigeria and Ethiopia; the CSO involvement in social franchising and their contribution to leveraging mHealth opportunities received similar low marks.

Women's Empowerment

The policy domain contained 18 items that addressed gender equality policies, demonstrated a level of commitment to the Convention on the Elimination of all Forms of Discrimination Against Women (CEDAW), equity in marriage and family, violence against women measures, favorable policies for land ownership, equitable work, women's participation in policymaking, and transparency and accountability. Of all six countries, Rwanda scored highest (8.7, 95% CI: 8.4-9.0) and Nigeria scored lowest (5.1, 95% CI: 4.0-6.1). In Nigeria, Ethiopia and Kenya, very low scores were recorded for women's participation in policy-making and traditional governance systems (e.g., chiefs and village heads). In the same countries and Senegal lower than average scores were recorded for the provision of reliable periodic age-disaggregated data on women's empowerment needs among populations in different communities. In Ethiopia, a respondent indicated that "the policy formulation does not consult the gender specialists and most of the policies and laws lack the concrete idea on the relevance of integrating the gender issue and that's why most of the policies and laws are not gender-responsive as needed".

The services and programs domain had 12 items that assessed gender mainstreaming, gender transformative programming, school dropouts, technology literacy, employment, violence victimization, FP for women's empowerment, child marriage and female genital mutilation. Overall, the scores across were around the midpoint except for Rwanda which scored 8.2 (95% CI: 7.8-8.6). Kenya, Ethiopia, and Nigeria scored lower than average on two critical items. First, law enforcement efforts to eliminate child marriage. Second, levels of efforts that the government makes possible, through laws, regulations, and programs, for women to combine the roles of childbearing, breastfeeding and child-rearing with participation in the workforce. In addition, respondents across countries shared their views on challenges related to WE policy enforcement and translation into programs. A respondent in Tanzania reflected that "Efforts might be there, but they are one-sided/single-handed and do not [receive] complementary support from other areas of enforcement. Laws and legislation in place are weak and/or not enforced as needed".

The advocacy domain comprised seven items that assessed the existence of advocacy platforms for women's and girls' empowerment, information dissemination, employment, education and elimination of all forms of violence. The highest score was recorded in Rwanda (8.2, 95% CI: 7.8-8.7) and the lowest in

For peer review only - http://bmjopen.bmj.com/site/about/guidelines.xhtml

Ethiopia (5.2, 95% CI:3.0-7.3). On item specific-scores, Nigeria, Kenya, and Ethiopia scored below average on the government undertaking sufficient efforts to ensure that the population is aware of issues related to WE. Similarly, the three countries scored low marks on whether the information spread is correct and reaches people in a language they understand.

The research domain had 10 items that assessed the capacity of research institutions, data quality and utilization at the ministerial level, the existence of a women's empowerment research strategy, topical data gathering and dissemination of research findings. The lowest national score was recorded in Ethiopia (3.8 95% CI: 2.0-5.6). In Ethiopia, Kenya and Nigeria lower than average scores were recorded for the extent to which government and private institutions undertake WE and child marriage research. Across countries, respondents shared their views on irregularities in research initiatives and limited utilization of research and evaluation data to inform WE policies and programs. A respondent in Ethiopia noted that "The research institutions do not conduct research periodically and I only see research come out when something happens. There is no periodic research to observe progress on a specific issue, especially on gender and women's empowerment".

The civil society domain included 10 items that covered the integration of civil society in policy, programs, evaluation, and research. They also addressed the resourcefulness of civil society organizations in generating evidence and utilizing data to inform advocacy efforts. Overall, the national scores were around the mid-point except for Rwanda which scored 8.2 (95% CI:7.8-8.6). On item-specific scores, lower than average marks were recorded in Senegal, Ethiopia and Kenya for the routine statistical system provision of good, reliable, and periodic age-disaggregated data on women's empowerment needs among populations in different communities. Respondents also highlighted the need for coordination and more results-oriented programming. One respondent in Kenya noted that "there are many CSOs [working on] WE in Kajiado [County] but [they] work in [an] uncoordinated manner leading to duplication [and] non-performance as they trade in ' wait and see' [for] under and overutilization of resources".

Education

The policy domain included 13 items that assessed the quality of national plans, strategies, and programs to improve the quality of learning in schools, gender parity, technology integration and learning for out-of-school children. The highest scores were recorded in Rwanda (8.7, 95% CI: 8.4-9.0) and the lowest scores were recorded in Tanzania (5.4, 95% CI: 4.2-6.6). A notable finding was on the item-specific marks in all countries except Rwanda, lower than average scores were recorded for technology literacy. Similarly, the strategies to overcome learning obstacles for pregnant girls and adolescent mothers also scored low in all countries except Rwanda and Kenya.

The services and programs domain had five items that covered teacher quality, teachers' deployment nationwide, parental involvement, standardized assessment and use of assessments to inform progress. Across countries, lower scores were recorded for the use of assessments to inform progress and several respondents emphasized that the ED sector lacked feedback systems, access to, and utilization of standards results to improve performance. One respondent in Ethiopia said, "Assessment is one of the activities that lagged behind for many years. Though national level large-scale assessments are conducted (such as NLA, EGRA, and EGMA) none of the study results or reports can be found at schools, woreda and zonal levels. Thus, beneficiaries do not utilize results of the studies to improve practice".

For peer review only - http://bmjopen.bmj.com/site/about/guidelines.xhtml

The advocacy domain contained four items that assessed the quality of education, the education system, teachers' obstacles, and institutional obstacles. The highest scores were recorded in Rwanda (8.0, 95% CI: 6.9-9.0) and the lowest scores were recorded in Tanzania (5.1, 95% CI:3.7-6.5). Respondents across countries highlighted the need to ensure human resource development and promotion of debate about educational institutions. One respondent in Tanzania emphasized that the "challenges associated with educational institutions should be made as country debate to inform the policy institutions".

The research domain contained 11 items that assessed data gathering, utilization, research capacity and ministerial-level use of evaluation data. The highest scores were recorded in Rwanda (8.4, 95% CI: 7.5-9.3) and the lowest scores were recorded in Ethiopia (5.0, 95% CI: 4.3-5.8). An important finding was that in Ethiopia, Kenya, Tanzania and Senegal, lower than national average scores were recorded for the management's use of evaluation findings, ministerial use of evaluation findings, and the extent to which information is shared or spread throughout various levels (national, state/province, localities). Further, several respondents from all countries highlighted the need to streamline research interventions to avoid duplication of efforts and data fatigue.

The civil society domain contained 11 items that assessed the involvement of civil society in the ED sector, the role of the community in ED, partnerships, social franchising, and technology. The lowest scores were recorded in Ethiopia, (4.0 95% CI: 3.1-4.9) and the highest scores were recorded in Rwanda (8.1, 95% CI: 7.1-9.1). Notably, in Ethiopia, the average scores for all items fell below the midpoint. Similarly, in Senegal, scores for community inclusion and the role of civil society in monitoring and evaluation were below the midpoint.

Labor Market

The policy domain comprised 15 items that covered key aspects of global and regional economic integration, technology, unemployment policies, urbanization, equitable access to employment, access to credit, targeted labor investment, saving, and self-employment policies. The lowest scores were recorded in Nigeria (4.3, 95% CI:3.2-5.4) and the highest scores were recorded in Rwanda (7.8, 95% CI:7.3-8.3). In all countries except Rwanda, very low scores were recorded for the existence of policies that outline an unemployment insurance system and the extent to which policies account for and address unemployment in various population groups and geographic areas.

The service or programs domain included 15 items that addressed unemployment reduction, access to microcredit, youth entrance to the labor market, youth entrepreneurship, women-friendly labor, minimum-wage and addressing informality in the labor market. The highest scores were recorded in Rwanda (7.5, 95% CI: 7.2-7.8) and the lowest scores were recorded in Nigeria (3.7, 95% CI: 2.4-5.1). Remarkably, in Nigeria, Ethiopia, Tanzania and Senegal, most questions in this domain scored below the midpoint and respondents across countries emphasized the need to foster services and programs that promote employment with a special focus on youth and women. One respondent in Tanzania iterated that "Apprenticeship programs have begun in the past three years but still some of our young graduates do not understand the essence of internship programs. Moreover, women are highly struggling for senior positions both in the Government and private sectors."

The advocacy domain questionnaire was composed of five items that addressed gender equality in the labor market, youth employment, minimum wage, and advocacy from government members. In Ethiopia, Nigeria and Senegal, the recorded average scores for the advocacy domain were below the midpoint.

For peer review only - http://bmjopen.bmj.com/site/about/guidelines.xhtml

Notably, item-specific average scores for Ethiopia and Senegal were low for minimum wage advocacy. Another interesting finding was that gender equality in the labor market scored highly in Rwanda, Ethiopia and Tanzania although this score was below the midpoint in Nigeria and Senegal.

The research domain questionnaire was composed of 11 items that addressed the presence of LM observation, research strategy, market analysis, quality of data, data dissemination, management, and managerial use of the evaluation data. In Nigeria and Kenya, the average individual question scores were low for the presence of monitoring and surveillance systems for employment. One respondent in Kenya suggested that "[the] government needs to develop a database for all students coming out of institutions and monitor their progress in the job market. Those not in jobs within six months after joining the job market should be assisted by governments to start their own businesses through the advancement of soft loans."

The civil society domain was assessed using 10 items that addressed the power of civil society in the LM, use of budget analysis for advocacy, community-based programs, diversification of job opportunities, worker's rights, women's employment, and civil society partnerships. Below average scores for the research domain were recorded in Ethiopia and Senegal and most items fell under the midpoint in these countries with the diversification of jobs scoring lowest in Senegal and Nigeria.

Good Governance and Economic Institutions

The policy domain was assessed using 13 items that addressed democratic values, youth participation in democratic processes and policymaking, transparency and accountability, national security, and affirmation of a safe learning environment. Across countries, the average scores were around the midpoint except in Rwanda (7.8, 95% CI: 6.6-9.0). In Nigeria, Ethiopia, and Tanzania the lowest item-specific average scores were recorded for the extent to which education facilities are protected and alternative learning opportunities are created for children and youth in a time of conflict or displacement.

The services and programs domain questionnaire contained 11 items that addressed interventions that strengthen youth organizations and networks, youth leadership, youth involvement in national activities, safety and reliable police services, participation in democratic elections, women's participation in governance, sustainable economic opportunity, and human capital development. Across countries, the average scores were around the midpoint or higher with the highest in Rwanda (8.1, 95% CI:7.4-8.7) and Kenya (7.0, 95% CI:4.5-9.4). An interesting finding was that in all countries, on item-specific scores, the highest scores for women's participation in governance were recorded for women's participation in governance at national levels and urban places, but the lowest scores were recorded for their participation at sub-national levels and rural places.

The advocacy domain questionnaire contained six items that addressed youth empowerment, government advocacy, non-governmental advocacy, and voices of instrumental sectors of the DD that included financial stability, optimal MCH, FP and a diversified labor market. Across countries, the average national scores were around the midpoint with the highest in Rwanda (7.8, 95% CI:7.0-8.6). Notably, among item-specific scores, the lowest score was recorded in Ethiopia on diversification of the labor market.

The research domain questionnaire was composed of 10 items that assessed the presence of governance data, the capacity of research instructions working in the areas of governance, finance and development, quality of data and data utilization by individuals and ministries. The scores varied across countries and

For peer review only - http://bmjopen.bmj.com/site/about/guidelines.xhtml

the highest scores were recorded for Rwanda (7.1, 95% CI:6.8-7.5). The average scores were recorded for the majority of individual questions but were below the midpoint in Ethiopia and Tanzania. The lowest score in Ethiopia was for the extent to which safety and the rule of law are researched whereas in Senegal the lowest recorded score was for the extent of measurement of political participation.

The civil society domain was composed of nine items that assessed the power of civil society, the use of policy and budget analysis in advocacy efforts, women's sectoral participation, advocacy and accountability, CSO-led assessment, and monitoring. The average scores were around the midpoint across all countries with the lowest in Ethiopia (4.7, 95% CI:3.5-5.8). A prominent finding to highlight was that across all countries, the average scores were high for the extent to which CSOs support youth participating in education, training, employment, and leadership.

Reliability and Validity

The alpha analysis revealed item-correlation estimates that were >0.4 in at least one country for all the items (variable), hence no items were excluded in further analysis. For detailed item-rest correlations for each item by domain, country and sector see Appendix 3. In the FP sector, the coefficients were >0.8 in all domains and countries except for the policy domain in Tanzania (α=0.72). In the MCH sector, the coefficient estimates were 0.8 or higher in all other domains and countries except Senegal (α=0.59). For the WE sector, the coefficient α estimates were >0.8 in all domains and countries. In the ED sector, the coefficient α estimates were >0.8 except for advocacy in Senegal (α=0.70) and research in Nigeria (α=0.71). For the labor market, the coefficient α estimates were >0.7 in nearly all countries and domains except advocacy in Ethiopia (α=0.40). In the GGEI sector, the coefficient α estimates were >0.7 in all countries and domains.

The confirmatory analysis revealed reasonably high loadings and good model fitting: SRMR estimates ranged from 0.04 to 0.15 with the estimates for most domains across sectors estimated at around 0.08. The CD estimates ranged from 0.89 to 0.99 across domains and sectors. The RMSEA estimates range from 0.14-0.46 across domains and sectors. The lower bound ranged between 0.08-0.46 and the upper bound ranged between 0.16-0.59. The CFI estimates were around 0.6 or higher except for services and programs in FP and MCH. For detailed confirmatory analysis results by domains and sector see Appendix 4.

The heterogeneity tests by domains across countries yielded similar results for fixed and random effect models. The I^2 estimates ranged from 0.0% to 62.5% in Ethiopia. In Kenya, the I^2 estimate was highest in services and programs (22.9%) and lowest (0.0%) in Policy, research, and civil society. In Nigeria, I^2 estimates ranged from 22.4% in research to 45.1% in services and programs. In Rwanda, the estimates ranged from 76.6% in research to 94.6% in services and programs. In Senegal, the I^2 estimates ranged from 47.0% in research to 88.6% in advocacy. In Tanzania, the lowest estimate was in research (39.9%) and the highest was in civil society (80.2%). For detailed heterogeneity testing results see Appendix 5.

Discussion

This study aimed to develop a tool to assess government efforts in creating a favorable policy environment to seize the benefits of a demographic dividend. The study collected data across six key sectors in Nigeria, Rwanda, Ethiopia, Senegal, Tanzania, and Kenya from key informants identified by the in-country study collaborators.

For peer review only

The DDEI scores revealed patterns that were helpful to guide discussions regarding needed improvements in respective countries across five domains; namely, policymaking, services and programs, advocacy, research, and civil society. The patterns of efforts scores for the LM, WE, FP, GGEI and ED sectors suggested great challenges for women's self-determination and empowerment, employment, and leadership. These findings agreed with previous predictions by the World Economic Forum that women may have to wait for 100 years to pair males in leadership roles in bigger institutions and take up ministerial roles. This is currently seen in the Global Gender Gap (GGG) Report, which ranks Rwanda in the top ten countries with the smallest gender gap among countries worldwide.[41 42]

Relatedly, Rwanda scored remarkably high on WE, although the scores were also moderately high for Tanzania and Senegal. These findings aligned with how these countries performed on the 2020 GGG report. Of the 153 countries ranked globally and on a maximum score possible of 1, Rwanda was ranked 9th with a score of 0.791 and Tanzania 68th with a score of 0.713. In contrast, Ethiopia ranked 82nd (0.705), Senegal 99th (0.684), Kenya 109th (0.671), and Nigeria 128th (0.635).[41] Although the intent of the DDEI was not to rank countries, these findings emphasized the potential for mutual learning from countries and the utility of the DDEI to dive deeper into specific items that can reveal special lessons or areas that require improvements. This is even more crucial, especially for countries that had a much larger change in ranking during the two years that preceded the report, such as Ethiopia which experienced an improvement of 35 ranks, and Kenya with a decrease of 33 ranks[41].

Furthermore, human capital development is essential for the country to cultivate and harness its DD potential. The 2020 Human Capital Index report revealed that the scores for all six countries of this study ranged from 0.36 in Nigeria to 0.55 in Kenya. These findings indicate that children born today in the six countries could miss out on 45% to 64% of their future earnings potential because of incomplete education and health that is not full.[43] The DDEI provided an opportunity to qualitatively compare different ongoing efforts initiated to develop human capital and the findings in MCH, ED, and WE were in agreement with the 2020 HCI findings. For instance, the lowest score for MCH was recorded in Nigeria 5.1 (95% CI: 3.8-6.4), and item-specific scores revealed wide variations that allowed for the identification of gaps.

This study offers several strengths. First, the questionnaires and the implementation of the DDEI were conducted in collaboration with in-country partners. This collaboration fosters local ownership of the effort measurement tool. Second, high Cronbach's alpha and high loading of the items in the CFA tool suggested high reliability and validity of the tool across countries. These tests also highlighted some items that appeared to be least reliable in selected countries; this information may be used to reduce the items of questionnaires in respective countries. The heterogeneity tests across domains revealed negligible to moderate heterogeneity in Ethiopia, Kenya and Nigeria, which indicates score consistency across domains in these countries and validity of the summary scores generated.[44] In the future, local institutions can regularly replicate these measurements at national and sub-national levels. Third, unlike prior effort indices, such as the FPEI, (created in 1972), the API (created in 1999), and MNPI (created in 2000), [27 45 46] the DDEI integrated critical sectors into one tool, and sectoral effort measures were not done in silos due to the interconnectedness and potential synergy between sectors (e.g. family planning, education, maternal and child health and women's empowerment). Finally, this tool provides information to constituents to hold their governments accountable and provide unconventional evidence for planning and evaluation purposes.

For peer review only - http://bmjopen.bmj.com/site/about/guidelines.xhtml

The DDEI was tested at the national level which opened the opportunity to be applied at the sub-national level. In countries where health and development programs are decentralized, sub-national DDEI can be transformational in guiding local budget allocation and self-evaluation. Given the paucity of quantitative data and poor utilization of the existing information systems, the DDEI has the potential to become a unique data source that informs local discussions and conversations at all administrative and planning levels. However, special emphasis should always be made that beyond strategic allocation of resources, favorable changes in age structure accompanied by a mix of jobs that allows for optimal productivity for all workers are important preconditions to fully realize the country's DD potential.[47 48]

Limitations

This study presented a number of limitations. The DDEI relied on experts' opinions, which may be affected by different factors. The respondent's judgment may be heavily influenced by their recent experience, which may not reflect the efforts placed over the years. Their judgment may be influenced by the level of their expectations, which may also be correlated with their level of satisfaction. For instance, where people have low expectations, the scores may be high despite the weak levels of effort, and vice-versa. The challenges related to the reliability of observers' judgments have been documented in other effort measurements such as the FPEI, API and MNPI.[28] However, despite this limitation, effort measures capture unique data that can guide planning and action, especially in countries where the availability of program data is limited.

Furthermore, some of the items maintained in the score had low item-rest correlation estimates in some of the countries although they had higher estimates in other countries. This may suggest that some items may be context dependent. For multi-country applications of this tool, these items were not dropped because their item-rest correlations were low in one setting, but not in others. In some of the countries, the sample size was small for some of the domains which may have affected the reliability and validity tests. In Rwanda, Senegal and Tanzania, heterogeneity estimates were high. Although heterogeneity may be due to chance alone, these findings warrant a larger sample size in future similar surveys. Nevertheless, the sample size could be limited by available knowledgeable respondents or experts in respective sectors for specific countries.

Further, other limitations worth mentioning are related to the nature of the responses and scores obtained from the respondents. The proportions of respondents who did not know the answer to the questions and consequently could not provide a score varied across sectors and countries although no questions had a high (>10%) proportion of "don't know" across all the six countries. The proportions were notably high in MCH research in Kenya, Nigeria and Tanzania, in ED civil society in Kenya and Nigeria, in WE advocacy in Ethiopia, in LM research and civil society in Ethiopia, Kenya and Nigeria and in GGEI advocacy and civil society in Nigeria and Rwanda. This finding indicates that for future surveys, some of the questions could be amended to make "don't know" answers easier to interpret or removed where found ambiguous. For detailed percentages of "don't know" responses received by item across countries see Appendix 6. Another limitation is that the aggregate average scores for the sectors modulated centering to the mid-point of 5 over the maximum possible 10. This tendency may indicate that respondents may have compensated for less generous scores that they gave to some questions with more generous scoring for others. However, this is less plausible because the item-specific scores within domains revealed wide variations across scores.

For peer review only - http://bmjopen.bmj.com/site/about/guidelines.xhtml

The DDEI questionnaire was administered in the midst of the COVID-19 pandemic, which led to abrupt changes in health and development systems in all countries. Although respondents provided great insights about their respective sectors, it is not unrealistic to assume the response rates and responses received may have been affected by the status of pandemics in their countries. Lastly, additional research will be needed to nurture the index, improve the reliability of the scores, and assess the need for weighing items and domains based on the stage of demographic transition while improving the usability of the DDEI data by local and global health and development stakeholders.

Conclusion

This study developed the DDEI for Nigeria, Ethiopia, Kenya, Tanzania, Rwanda, and Senegal and demonstrated the utility of this index. The DDEI yielded several recommendations on opportunities to accelerate socio-economic development and take advantage of the DD potential in these countries. The scores for each sector and domain provided variations that allowed for comparison within a sector in each country. By engaging with local experts and partnering with local institutions in a participatory process, the data from the DDEI were used by local policymakers, service providers, program implementers, advocates, and researchers for self-evaluation and to develop recommendations to strengthen ongoing efforts and programs in respective sectors.

The DDEI allowed for multisectoral analysis and provided an opportunity to identify sectors in most need and those that can serve as models in each country. For instance, based on sector-specific scores, LM and WE stood out as the sectors that required particular attention. However, despite higher sector-specific scores, a closer look at item-specific scores across domains revealed wide variations that were the basis of generating policy recommendations. The DDEI can be applied to all SSA countries regularly to monitor progress and spark local conversation around human rights and sustainable socio-economic development.

The DDEI can serve as a complementary tool to existing global development monitoring tools. The findings from the DDEI aligned with other existing measures such as the GGG and HCI in various countries. Given the quantitative nature of these tools, the DDEI provides an opportunity to assess the inputs and guide policies, investments and programs in specific sectors and items that can positively affect the gender gap and human capital. In addition to driving conversation amongst stakeholders in FP, MCH, ED, WE, GGEI and LM, the DDEI focuses on assessing the inputs that are critical for the outcomes measured by GGG and HCI. The current DDEI has been translated into English and French. In the future, it should be translated into other languages such as Portuguese and Spanish to make it applicable to other countries in the early stages of their demographic transition.

Ethical considerations

The study was conducted in compliance with the Helsinki declaration and was approved by the Johns Hopkins Bloomberg School of Public Health ethical review board (IRB Number: IRB00011522). All participants provided informed consent prior to taking part in this research.

Contributorship statement

Jean Christophe Rusatira, Carolina Cardona, Michelle Martinez-Baack, and Jose G. Rimon conceived the study. Jean Christophe Rusatira, Carolina Cardona, Michelle Martinez-Baack, Saifuddin Ahmed, and Jose G. Rimon developed the questionnaire, led data collection, data analysis, and interpretation. Jean

For peer review only - http://bmjopen.bmj.com/site/about/guidelines.xhtml

Christophe prepared the first draft of the manuscript. All authors revised and approved the final and revised versions of the paper and agree to be accountable for all aspects of the work.

Acknowledgments
We express our deepest thanks to all participants for their contributions to this study. We would also like to thank our local partners and staff who facilitated this work at the Centre d'Excellence Régional en Economie Générationnelle in Senegal, the Center for Communication Programs in Nigeria, the National Council for Population and Development in Kenya, the Tanzania Communication and Development Center, the Ethiopian Academy of Sciences, and the Centre for Integrated Development Research and Action in Rwanda.

Competing interests
The are no competing interests for any author.

Funding
This work was supported by the Bill and Melinda Gates Foundation under the grant OPP1181398.

Data sharing statement
The data and questionnaires used to generate this article are available on Harvard Dataverse open access repository accessible at https://doi.org/10.7910/DVN/ZSNPMV.

For peer review only

For peer review only - http://bmjopen.bmj.com/site/about/guidelines.xhtml

References

1. González AL, González-González MJ. Third demographic transition and demographic dividend: An application based on panel data analysis. *Bulletin of Geography Socio-economic Series* 2018(42):59-82.
2. Bloom D, Canning D, Sevilla J. The demographic dividend: A new perspective on the economic consequences of population change: Rand Corporation 2003.
3. Reher DS. Economic and Social Implications of the Demographic Transition. *Population and development review* 2011;37:11-33.
4. Herzer D, Strulik H, Vollmer S. The long-run determinants of fertility: one century of demographic change 1900–1999. *Journal of Economic Growth* 2012;17(4):357-85. doi: 10.1007/s10887-012-9085-6
5. Bloom D, Humair S, Rosenberg L, et al. A Demographic Dividend for Sub-Saharan Africa: Source, Magnitude, and Realization. *IZA Discussion Paper* 2014(7855):26.
6. UNFPA. Demographic Dividend Atlas for Africa: Tracking the Potential for a Demographic Dividend: United Nations Population Fund, 2017.
7. Bloom DE, Williamson JG. Demographic Transitions and Economic Miracles in Emerging Asia. *The World Bank Economic Review* 1998;12(3):419-55. doi: 10.1093/wber/12.3.419
8. Bloom D, Canning D, Malaney PN. Demographic Change and Economic Growth in Asia: Center for International Development at Harvard University, 1999.
9. African Union Commission. AU Roadmap on Harnessing the Demographic Dividend through Investments in Youth. Assembly of the Union Twenty- Sixth Ordinary Session 30-31: African Union Commission, 2017:44.
10. Cardona C, Rusatira JC, Cheng X, et al. Generating and capitalizing on the demographic dividend potential in sub-Saharan Africa: a conceptual framework from a systematic literature review. *Gates open research* 2020;4(145):145.
11. Bloom D, Canning D, Malaney PN. Demographic change and economic growth in Asia. *CID Working Paper Series* 1999
12. Bloom DE, Canning D. Global demographic change: Dimensions and economic significance: National Bureau of Economic Research, 2004.
13. Fabic MS, Choi Y, Bongaarts J, et al. Meeting demand for family planning within a generation: the post-2015 agenda. *The Lancet* 2015;385(9981):1928-31. doi: 10.1016/S0140-6736(14)61055-2
14. Bloom DE, Canning D, Hu L, et al. The Contribution of Population Health and Demographic Change to Economic Growth in China and India. *J Comp Econ* 2010;38(1):17-33. doi: 10.1016/j.jce.2009.11.002 [published Online First: 2010/04/27]
15. Li Q, Tsui AO, Liu L, et al. Mortality, fertility, and economic development: An analysis of 201 countries from 1960 to 2015. *Gates Open Res* 2018;2:14. doi: 10.12688/gatesopenres.12804.1 [published Online First: 2018/04/24]
16. Ahmed S, Li Q, Liu L, et al. Maternal deaths averted by contraceptive use: an analysis of 172 countries. *Lancet* 2012;380(9837):111-25. doi: 10.1016/s0140-6736(12)60478-4 [published Online First: 2012/07/13]
17. Conley D, McCord GC, Sachs JD. Africa's lagging demographic transition: Evidence from exogenous impacts of malaria ecology and agricultural technology: National Bureau of Economic Research Cambridge, Mass., USA, 2007.
18. Becker GS, Becker GS. A Treatise on the Family: Harvard university press 2009.
19. Ahmed SA, Cruz M, Quillin B, et al. Demographic Change and Development: Looking at Challenges and Opportunities through a New Typology: The World Bank 2016.

For peer review only

For peer review only - http://bmjopen.bmj.com/site/about/guidelines.xhtml

20. Ahmed SA, Cruz M, Go DS, et al. How Significant is Africa's Demographic Dividend for its Future Growth and Poverty Reduction?: The World Bank 2014.
21. Shekar M, Yazbeck A, Hasan R, et al. Population and Development in the Sahel: Policy Choices to Catalyze a Demographic Dividend. *Health, Nutrition and Population (HNP) Discussion Paper, World Bank* 2016 doi: 10.1596/25293
22. WBG. Global Monitoring Report 2015/2016: Development Goals in an Era of Demographic Change. Washington, DC: The World Bank 2016.
23. Ahmed SA, Cruz M. Making the Most of Demographic Change in Southern Africa: The World Bank 2016.
24. Ross JA, Stover J. Effort Indices for national family planning programs, 1999 cycle: MEASURE Evaluation, Carolina Population Center, University of North Carolina 2000.
25. Ross JA, Mauldin WP. Family planning programs: efforts and results, 1972-94. *Studies in family planning* 1996:137-47.
26. Mauldin WP, Berelson B, Sykes Z. Conditions of fertility decline in developing countries, 1965-75. *Studies in Family Planning* 1978;9(5):89-147.
27. Stover J. The AIDS Program Effort Index (API): Results from the Field Test. *Washington, DC: Futures Group* 1999
28. Ross JA, Campbell O, Bulatao R. The maternal and neonatal programme effort index (MNPI). *Tropical medicine & international health* 2001;6(10):787-98.
29. Bank W. Global monitoring report 2015/2016: Development goals in an era of demographic change: The World Bank, 2015.
30. Ross J, Stover J. The family planning program effort index: 1999 cycle. *International Family Planning Perspectives* 2001:119-29.
31. Fort AL, Deussom R, Burlew R, et al. The Human Resources for Health Effort Index: A tool to assess and inform strategic health workforce investments. *Human resources for health* 2017;15(1):1-11.
32. Usengumukiza F, Munyandamutsa JP, Nshutiraguma E. Strengthening democratic governance and accountability in Rwanda: The role of the Rwanda Governance Board (RGB). Democratizing Public Governance in Developing Nations: Routledge 2017:109-33.
[Dataset] [33.] Rusatira JC, Cardona C, Martinez-Baack M, et al. Demographic Dividend Effort Index (DDEI). VERSION 1 ed: Harvard Dataverse, 2022. https://doi.org/10.7910/DVN/ZSNPMV
34. Lord FM, Novick MR. Statistical theories of mental test scores: IAP 2008.
35. De Groot A, Van Naerssen R. Studietoetsen. Construeren, afnemen, analyseren, deel 1. Studietoetsen Construeren, afnemen, analyseren, deel 1: De Gruyter Mouton 2018.
36. Henson RK. Understanding internal consistency reliability estimates: A conceptual primer on coefficient alpha. *Measurement and evaluation in counseling and development* 2001;34(3):177-89.
37. Browne MW, Cudeck R. Alternative ways of assessing model fit. *Sociological methods & research* 1992;21(2):230-58.
38. Bentler PM. Comparative fit indexes in structural models. *Psychological bulletin* 1990;107(2):238.
39. Poole C, Greenland S. Random-effects meta-analyses are not always conservative. *American Journal of Epidemiology* 1999;150(5):469-75.
40. Higgins JP, Thompson SG, Deeks JJ, et al. Measuring inconsistency in meta-analyses. *Bmj* 2003;327(7414):557-60.
41. Forum WE. Global Gender Gap Report 2020: Insight Report. 2019:1-371.
42. Schwab K. The Global Competitiveness Report 2019. Geneva. World Economic Forum, 2019.

For peer review only

For peer review only - http://bmjopen.bmj.com/site/about/guidelines.xhtml

43. The Human Capital Index 2020 Update. The Human Capital Index 2020 Update; 2020.
44. Fletcher J. What is heterogeneity and is it important? *Bmj* 2007;334(7584):94-96.
45. Lapham RJ, Mauldin WP. Contraceptive prevalence: the influence of organized family planning programs. *Studies in Family Planning* 1985;16(3):117-37.
46. Ross JA, Campbell OM, Bulatao R. The Maternal and Neonatal Programme Effort Index (MNPI). *Trop Med Int Health* 2001;6(10):787-98. doi: 10.1046/j.1365-3156.2001.00787.x [published Online First: 2001/10/27]
47. Bloom DE, Canning D, Fink G, et al. Realizing the demographic dividend: Is Africa any different?2007.
48. Bloom DE. 7 billion and counting. *Science* 2011;333(6042):562-69.

For peer review only

Figure 1: Average scores per country and sector

For peer review only

For peer review only - http://bmjopen.bmj.com/site/about/guidelines.xhtml

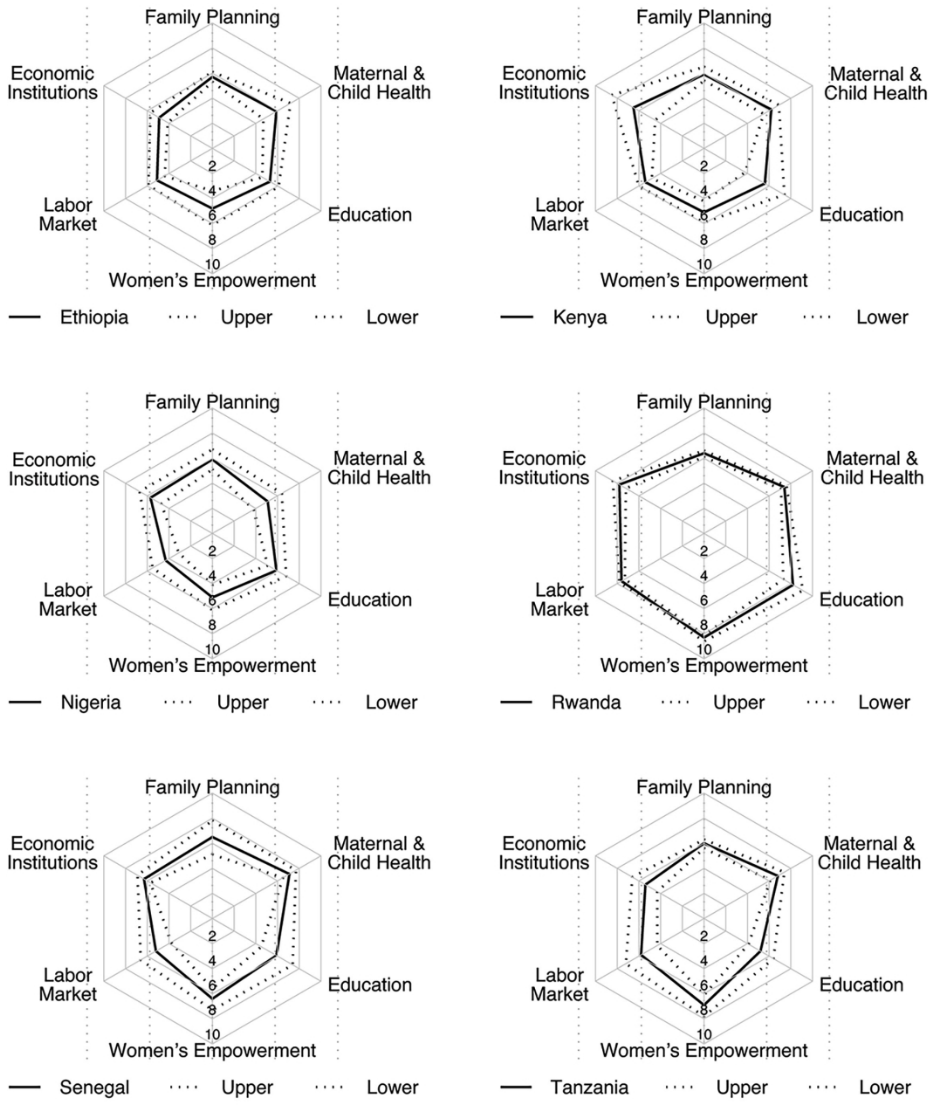

Average scores per country and sector

203x254mm (300 x 300 DPI)

JOHNS HOPKINS
BLOOMBERG SCHOOL of PUBLIC HEALTH

Bill & Melinda Gates Institute for Population and Reproductive Health

Demographic Dividend Effort Index Survey

Introduction

There is a programmatic need for a standard measure that quantifies the extent of national efforts in policies and programs implemented to cultivate, realize and harness the benefits of a demographic dividend (DD). Despite the remarkable progress achieved by various countries through the integration of demographic dividend principles into their policies and development plans, performance assessment tools and platforms to document best practices and lessons learned to harness the DD are still missing.

Thus, the purpose of the DD Effort Index (DDEI) is to provide a standard measure that will quantify the nature and strength of in-country DD efforts by experts and civil society members across the public and private spheres that represent different dimensions of the society. By measuring efforts, we are capturing inputs independent of outputs, such as income per capita. This will allow us to assess the relationship between inputs that go into the development of DD policies and DD outputs.

This DDEI is guided by one central question: To what extent have the existing national policy and program efforts enabled a favorable policy environment to cultivate, realize and harness the benefits of a demographic dividend? To answer this question, the DDEI will summarize key informant perceptions across six sectors identified in the literature to produce a favorable environment to harness a DD. These DD framework sectors are: 1) Family Planning, 2) Maternal and Child Health, 3) Education, 4) Women's Empowerment, 5) Labor Market, and 6) Good Governance, Strong Institutions and Stable Economy. Each sector is measured through its own questionnaire and will be assessed in five dimensions identified in the literature; namely, policy, services or programs, advocacy, research, and civil society. Each of the 6 questionnaires, completed by 10 expert observers per country, score items on a scale from 1 to 10, unless otherwise noted.

Considering the health and socio-economic impact of COVID-19, and its close relationship with policies that enable a DD favorable policy environment, this DD Effort Index is integrating a module to assess the resilience and sustainability of systems in each sector. The scoring follows the key principles of sustainable and resilient health systems that can effectively respond to emerging infectious disease threats and other public health emergencies.

The DDEI provides an opportunity to visualize the national efforts while assessing the extent to which these efforts support a country's goals to cultivate, realize and harness the demographic dividend. The DDEI will be a tool for performance measurement and progress monitoring, which will provide a timely assessment of successes and gaps and reveal best practices and areas of improvement.

JOHNS HOPKINS
BLOOMBERG SCHOOL
of PUBLIC HEALTH

Bill & Melinda Gates Institute for Population and Reproductive Health

Demographic Dividend Effort Index Survey

Consent form

Thank you for taking the time to complete this questionnaire, especially in this unprecedented time.

The Gates Institute is developing a Demographic Dividend Effort Index (DDEI) to establish a standard measure that will allow the assessment of the existing status of national policies, services, and programs in family planning, maternal and child health, other social sectors (e.g., employment and education), and governance to enable a favorable policy environment to cultivate a demographic dividend (DD). By measuring efforts, this tool will capture inputs independent of outputs, such as income per capita. This will allow the assessment of the relationship between inputs that go into the development of DD policies and DD outputs that are observed and commonly used to assess progress.

We would like to ask you some questions because of your expertise in the area. The information collected will be kept confidential, and we will not be collecting your names or any other identification details. Completing this questionnaire will take around 45-60 min of your time.

We will appreciate your participation in this survey. Information from this interview will be used by the Gates Institute and the community of DD practice in your country to assess the national efforts and identify areas to continue supporting the successes and those that need to be strengthened. Researchers may also use the data collected for cross-country analyses.

You are invited to participate in this survey. Please know that this survey is completely voluntary. By participating, there is no direct benefit to you but there is a societal benefit in enabling the government and development partners to strengthen their efforts to cultivate a DD-favorable environment.

You may refuse to answer any question. Should you have any concerns or issues about the study, you may contact the study coordinator at **jcrusatira@jhu.edu** or **+14436510824** by a phone call or WhatsApp.

* Please check “Yes” if you would like to proceed with the survey.

- ◯ Yes
- ◯ No

JOHNS HOPKINS
BLOOMBERG SCHOOL
of PUBLIC HEALTH

Bill & Melinda Gates Institute for Population and Reproductive Health

Demographic Dividend Effort Index Survey

Important notice

Please do not select 'Back' while in the middle of answering a page of questions as the page you are working on will not be saved until you finalize the said page and select 'Next'. If you select 'Back' without finalizing, the progress you have made on the unfinished page will be lost.

Please be aware that your survey is completed when you read "Thank you for completing the demographic dividend effort index. Stay well and safe!"

JOHNS HOPKINS
BLOOMBERG SCHOOL
of PUBLIC HEALTH

Bill & Melinda Gates Institute for Population and Reproductive Health

Demographic Dividend Effort Index Survey

Respondent country and expertise

* Please select the country where you are responding from.

- Kenya
- Senegal
- Nigeria
- Rwanda
- Tanzania
- Ethiopia

JOHNS HOPKINS
BLOOMBERG SCHOOL
of PUBLIC HEALTH

Bill & Melinda Gates Institute for Population and Reproductive Health

Demographic Dividend Effort Index Survey

Level of expertise

* At which level of Government is your expertise (If your expertise is at the national level please select "National" otherwise select a county?

JOHNS HOPKINS
BLOOMBERG SCHOOL
of PUBLIC HEALTH

Bill & Melinda Gates Institute for Population and Reproductive Health

Demographic Dividend Effort Index Survey

Expertise

* Please select the organization you have worked in the most during the last five years. If your organization is not listed, please write it down.

- Public sector
- Private sector
- Non-governmental organization
- University/research institute
- Other (please specify)

* Which dimension have you worked in the most during the last five years?

- Policy/policymaking
- Services or programs
- Advocacy
- Research
- Civil Society

* In which sector have you worked the most during the last five years?

- Family Planning
- Maternal and Child Health
- Education
- Women's Empowerment
- Labor Market
- Governance and Economic Institutions

JOHNS HOPKINS
BLOOMBERG SCHOOL
of PUBLIC HEALTH

Bill & Melinda Gates Institute for Population and Reproductive Health

Demographic Dividend Effort Index Survey

Family Planning Questionnaire

Please respond to the following questions based on your experience/expertise within this specific sector.

To give a summary picture of the national demographic dividend effort, please rate the following items. Score each item from 1 to 10, where 1 represents the lowest score (very weak or no effort) and 10 represents the highest score (strong effort). Where applicable, if a policy or activity is non-existent then mark your response as 0. Please note that a few questions have a different scoring scale.

Please answer each item. All responses will be recorded in the format exemplified below:

Lowest effort	1	2	3	4	5	6	7	8	9	10	Highest effort	I do not know

JOHNS HOPKINS
BLOOMBERG SCHOOL
of PUBLIC HEALTH

Bill & Melinda Gates Institute for Population and Reproductive Health

Demographic Dividend Effort Index Survey

Dimension 1. Policy/Policy making

* FP 1.1 Prominence of Family Planning (FP): Extent to which there is a permanent office or post within the appropriate ministry that develops and monitors sectoral policies and strategies, that is well placed within the government, and staffed by adequately skilled personnel.

	1	2	3	4	5	6	7	8	9	10	Don't know
1-Lowest -> Highest-10											

* FP 1.2 Policy on Fertility Reduction and FP: Extent to which government policy emphasizes FP for family size as it relates to available resources or demographic reasons over health reasons.

	1	2	3	4	5	6	7	8	9	10	Don't know
1-Lowest -> Highest-10											

* FP 1.3 National FP Strategic Plan: Extent to which there is a current, comprehensive, strategic national plan for FP that outlines policies, laws, and regulations for the provisioning of FP. (In making this assessment, consider whether the plan defines a vision; includes multiyear strategies, annual timelines, and implementation plans; is evidence-based; has indicators to measure progress; and considers the FP market.)

	1	2	3	4	5	6	7	8	9	10	Don't know
1-Lowest -> Highest-10											

* FP 1.4 Policy on Age at Marriage: Extent to which legal age at marriage for females is set at 18 years or higher and is enforced.

	1	2	3	4	5	6	7	8	9	10	Don't know
1-Lowest -> Highest-10											

* FP 1.5 Evidence-Based Policymaking: Extent to which the appropriate ministry actively and regularly supports and uses key FP data and reports to engage in policy development and planning.

	1	2	3	4	5	6	7	8	9	10	Don't know
1-Lowest -> Highest-10	◯	◯	◯	◯	◯	◯	◯	◯	◯	◯	◯

* FP 1.6 Monitoring and Evaluation of National Policies/Plans/Strategies: Extent to which the national FP plan outlines a comprehensive monitoring and evaluation strategy to track and measure accomplishments in the plan.

(In making this assessment, consider whether the monitoring and evaluation strategy includes goals and objectives, a results framework, measurable indicators [e.g., “SMART”: specific, measurable, attainable, relevant and time-bound]; and an actionable monitoring and evaluation implementation plan/timeline.)

	1	2	3	4	5	6	7	8	9	10	Don't know
1-Lowest -> Highest-10	◯	◯	◯	◯	◯	◯	◯	◯	◯	◯	◯

* FP 1.7 Inclusion of Nongovernmental and Cross-sectoral Actors in Policy/Strategy Development: Extent to which nongovernmental agencies, groups (e.g., from civil society), health workers, stakeholders (e.g., from faith-based, nonprofit, and private-for-profit organizations) and actors across non-FP-specific sectors are included in the national FP plan and/or other national documents outlining the country’s strategies and systems related to planning, managing, and developing FP.

	1	2	3	4	5	6	7	8	9	10	Don't know
1-Lowest -> Highest-10	◯	◯	◯	◯	◯	◯	◯	◯	◯	◯	◯

* FP 1.8 Financing for FP: Extent to which the government has established and applied guidelines for the costing and payments for FP across supply and demand considerations (i.e. domestic versus international funding – and its expectations for continuity/sustainability, cost of contraceptive supplies, cost of service delivery and transportation, etc.).

(In making this assessment, consider whether the guidelines and budget address the following: the lifespan of costs for FP care and services; variable structures of costs per FP method; costs of delivery and service provision by specialization, location and risk; costs and profits of healthcare/insurance schemes, the financial costs of providing FP weighed against the financing costs of not doing so.)

	1	2	3	4	5	6	7	8	9	10	Don't know
1-Lowest -> Highest-10	◯	◯	◯	◯	◯	◯	◯	◯	◯	◯	◯

* FP 1.9 Percent of In-country Funding of FP Budget: Extent to which the total family planning/population budget is derived from in-country sources (e.g., 1 for 10 percent, 5 for 50 percent, 10 for 100 percent).

	1	2	3	4	5	6	7	8	9	10	Don't know
1-Lowest -> Highest-10	○	○	○	○	○	○	○	○	○	○	○

* FP 1.10 Information/Communications Technology Infrastructure/Capacity: Extent to which there are adequate infrastructure components (e.g., computers, servers, printers, a local area network, Internet connectivity, and reliable electric power) as well as human resources (e.g., for maintenance and support) at the national and subnational levels to support the functioning of FP-related information systems.
(In making this assessment, consider whether the country conducts at least annual assessments of its information systems; has training curricula that cover data entry, use, software administration, and development; and continuously trains staff from key stakeholder institutions [e.g., Ministry of Health, Ministry of Education, regulatory agencies, training institutions].)

	1	2	3	4	5	6	7	8	9	10	Don't know
1-Lowest -> Highest-10	○	○	○	○	○	○	○	○	○	○	○

* FP 1.11 Import Laws and Legal Regulations:

	1	2	3	4	5	6	7	8	9	10	Don't know
FP 1.11.1 Extent to which import laws and legal regulations facilitate the importation of FP commodities and supplies	○	○	○	○	○	○	○	○	○	○	○
FP 1.11.2 Extent to which contraceptives are manufactured locally.	○	○	○	○	○	○	○	○	○	○	○

* FP 1.12 Advertising of Contraceptives Allowed: Extent of freedom from restrictions on advertising of contraceptives in the mass media.

	1	2	3	4	5	6	7	8	9	10	Don't know
1-Lowest -> Highest-10	○	○	○	○	○	○	○	○	○	○	○

* FP 1.13 Presence of Pro-Poor FP Policies: Extent to which policies specifically target the poor in rural or urban places.

	1	2	3	4	5	6	7	8	9	10	Don't know
1-Lowest -> Highest-10	○	○	○	○	○	○	○	○	○	○	○

* FP 1.14 Political Support for FP: Extent to which elected officials in the country prioritize meeting FP needs to strengthen the FP contributions to the DD by passing laws and regulations and sponsoring actions and policies aimed at improving FP uptake and adherence.

	1	2	3	4	5	6	7	8	9	10	Don't know
1-Lowest -> Highest-10	○	○	○	○	○	○	○	○	○	○	○

FP 1.15 Comments: Please use this section to provide any additional comments about the level of effort, challenges, or successes in the country around policies/policymaking for FP. If you marked “I don’t know” for any of the questions, also use this section to explain why you gave that rating

Bill & Melinda Gates Institute for Population and Reproductive Health

Demographic Dividend Effort Index Survey

Dimension 2. Services or Programs

* FP 2.1 Administrative Structure: Extent to which the administrative structure and staff at national, provincial and county levels are sufficient to implement the family planning program.

	1	2	3	4	5	6	7	8	9	10	Don't know
1-Lowest -> Highest-10											

* FP 2.2 Level of Program Leaders: Relative level of seniority of the director of the national family planning program and whether said director reports to a high level of government.

	1	2	3	4	5	6	7	8	9	10	Don't know
1-Lowest -> Highest-10											

* FP. 2.3 Community-Based Distribution: Extent to which areas of the country not easily serviced by clinics or other service points are covered by community-based distribution programs for distribution of contraceptive supplies (especially rural areas).

	1	2	3	4	5	6	7	8	9	10	Don't know
1-Lowest -> Highest-10											

* FP 2.4 Postpartum Program: Extent to which all postpartum women receive postpartum family planning services.

	1	2	3	4	5	6	7	8	9	10	Don't know
1-Lowest -> Highest-10											

* FP 2.5 Home-Visiting Workers: Extent of coverage of population by workers whose primary task is to visit (rural) women in their homes to talk about family planning and maternal and child health.

	1	2	3	4	5	6	7	8	9	10	Don't know
1-Lowest -> Highest-10	○	○	○	○	○	○	○	○	○	○	○

* FP 2.6 Availability and Accessibility of FP Commodities/Supplies and Services Part 1: Extent to which entire population has ready and easy access to (1-Lowest -> Highest-10):

	1	2	3	4	5	6	7	8	9	10	Don't know
FP 2.6.1 IUD	○	○	○	○	○	○	○	○	○	○	○
FP 2.6.2 Pills	○	○	○	○	○	○	○	○	○	○	○
FP 2.6.3 Injectables	○	○	○	○	○	○	○	○	○	○	○
FP 2.6.4 Implants	○	○	○	○	○	○	○	○	○	○	○
FP 2.6.5 Female Sterilization	○	○	○	○	○	○	○	○	○	○	○
FP 2.6.6 Male Sterilization	○	○	○	○	○	○	○	○	○	○	○
FP 2.6.7 Male Condoms	○	○	○	○	○	○	○	○	○	○	○
FP 2.6.8 Emergency Contraception	○	○	○	○	○	○	○	○	○	○	○
FP 2.6.9 Abortion/menstrual regulation (whether legal or not)	○	○	○	○	○	○	○	○	○	○	○
FP 2.6.10 Others (e.g. female condoms, foam, beads, etc.)	○	○	○	○	○	○	○	○	○	○	○

* FP 2.7 Availability and Accessibility of FP Commodities/Supplies and Services Part 2: How well does the supply system operate (it avoids stockouts or interrupted supplies and guarantees a reliable flow at local levels) for the following (1-Lowest -> Highest-10):

	1	2	3	4	5	6	7	8	9	10	Don't know
FP 2.7.1 IUD	○	○	○	○	○	○	○	○	○	○	○
FP 2.7.2 Pills	○	○	○	○	○	○	○	○	○	○	○
FP 2.7.3 Injectables	○	○	○	○	○	○	○	○	○	○	○
FP 2.7.4 Implants	○	○	○	○	○	○	○	○	○	○	○
FP 2.7.5 Male Condoms	○	○	○	○	○	○	○	○	○	○	○
FP 2.7.6 Emergency Contraception	○	○	○	○	○	○	○	○	○	○	○
FP 2.7.7 Others (e.g. female condoms, foam, beads, etc.)	○	○	○	○	○	○	○	○	○	○	○

* FP 2.8 Availability and Accessibility of FP Commodities/Supplies and Services Part 3: How well does the supply system provide necessary equipment and medical supplies at clinical facilities for the following (1-Lowest -> Highest-10):

	1	2	3	4	5	6	7	8	9	10	Don't know
FP 2.8.1 Female Sterilization	○	○	○	○	○	○	○	○	○	○	○
FP 2.8.2 Male Sterilization	○	○	○	○	○	○	○	○	○	○	○
FP 2.8.3. IUDs	○	○	○	○	○	○	○	○	○	○	○
FP 2.8.4 Implants	○	○	○	○	○	○	○	○	○	○	○

* FP 2.9 FP Service Quality: Please rate the general quality of family planning services. (Good quality includes a focus on client needs, with counseling, full information, wide method choice, and safe clinical procedures.)

	1	2	3	4	5	6	7	8	9	10	Don't know
1-Lowest -> Highest-10	○	○	○	○	○	○	○	○	○	○	○

* FP 2.10 Incentives and Disincentives: Extent to which monetary or other incentives are used to encourage the adoption of family planning.

	1	2	3	4	5	6	7	8	9	10	Don't know
1-Lowest -> Highest-10	○	○	○	○	○	○	○	○	○	○	○

* FP 2.11 Adolescent Programs: Extent to which information, services and activities are focused towards adolescents and encourage adolescents to access contraceptive services.

	1	2	3	4	5	6	7	8	9	10	Don't know
1-Lowest -> Highest-10											

* FP 2.12 Training Program: Extent to which training programs, for each category of staff in the family planning program, are adequate to provide personnel with information and skills necessary to carry out their jobs effectively.

	1	2	3	4	5	6	7	8	9	10	Don't know
1-Lowest -> Highest-10											

* FP 2.13 Personnel Carry Out Assigned Tasks: Extent to which all categories of family planning program staff (administrative, medical, paramedical, field) carry out assigned tasks effectively

	1	2	3	4	5	6	7	8	9	10	Don't know
1-Lowest -> Highest-10											

* FP 2.14 Logistics and Transport: Extent to which the logistics and transport systems are sufficient to keep stocks of contraceptive supplies and related equipment available at all service points, at all times and at all levels.

	1	2	3	4	5	6	7	8	9	10	Don't know
1-Lowest -> Highest-10											

* FP 2.15 Supervision System: Extent to which the system of supervision at all levels is adequate (regular monitoring visits with corrective or supportive action).

	1	2	3	4	5	6	7	8	9	10	Don't know
1-Lowest -> Highest-10											

* FP 2.16 Involvement of Other Ministries and Public Agencies: Extent to which other ministries, government agencies and non-governmental stakeholders engage with FP activities (e.g., delivery of supplies, services, information, education) or other population activities.

	1	2	3	4	5	6	7	8	9	10	Don't know
1-Lowest -> Highest-10											

* FP 2.17 Involvement of Private-Sector Agencies and Groups: Extent to which private-sector agencies and groups assist with FP or other population activities.

	1	2	3	4	5	6	7	8	9	10	Don't know
1-Lowest -> Highest-10	○	○	○	○	○	○	○	○	○	○	○

* FP 2.18 Mass Media for Information Education Communication: Frequency and extent of coverage of mass media messages that provide the population with information on family planning and service sites.

	1	2	3	4	5	6	7	8	9	10	Don't know
1-Lowest -> Highest-10	○	○	○	○	○	○	○	○	○	○	○

* FP 2.19 Cultural and Social Norms Barriers: Extent to which cultural and social barriers are addressed in family planning programs.

	1	2	3	4	5	6	7	8	9	10	Don't know
1-Lowest -> Highest-10	○	○	○	○	○	○	○	○	○	○	○

* FP 2.20 Youth: Extent to which the government supports youth-led FP initiatives or involve youth in planning, implementation and evaluation of FP programs.

	1	2	3	4	5	6	7	8	9	10	Don't know
1-Lowest -> Highest-10	○	○	○	○	○	○	○	○	○	○	○

* FP 2.21 FP Effort Influences – Forces Affecting the FP Program: Forces affecting the FP program can either strengthen or detract from its effectiveness. Check zero (0) if there is no difference either way; check a negative number from -1 to -5 if the influence is negative; or check a positive number from 1 to 5 if the influence is positive. (1 in either direction means little influence and 5 in either direction means strong influence.)

	-5	-4	-3	-2	-1	0	1	2	3	4	5	Don't know
FP 2.21.1 Decentralization	○	○	○	○	○	○	○	○	○	○	○	○
FP 2.21.2 HIV/AIDS Programs	○	○	○	○	○	○	○	○	○	○	○	○
FP 2.21.3 Incorporation of FP into a broader context of reproductive health	○	○	○	○	○	○	○	○	○	○	○	○
FP 2.21.4 Integration of FP with other health services	○	○	○	○	○	○	○	○	○	○	○	○
FP 2.21.5 Changes in donor funding	○	○	○	○	○	○	○	○	○	○	○	○
FP 2.21.6 Changes in domestic government funding	○	○	○	○	○	○	○	○	○	○	○	○

FP 2.22 Comments: Please use this section to provide any additional comments about the level of effort, challenges, or successes in the country around services or programs for FP. If you marked “I don’t know” for any of the questions, also use this section to explain why you gave that rating

JOHNS HOPKINS
BLOOMBERG SCHOOL
of PUBLIC HEALTH

Bill & Melinda Gates Institute for Population and Reproductive Health

Demographic Dividend Effort Index Survey

Dimension 3. Advocacy

* FP 3.1 Influence of FP Leaders/Champions: Extent to which the country has one or more clearly influential leaders or champions who successfully advocate for FP (sectoral) needs at high levels, promote FP in the country by making positive public statements about it, and/or support actions and policies aimed at improving the FP environment and its contributions towards the DD.

	1	2	3	4	5	6	7	8	9	10	Don't know
1-Lowest -> Highest-10	○	○	○	○	○	○	○	○	○	○	○

* FP 3.2 Statement by Leaders: Extent to which the head of government, as well as other officials, speak publicly and favorably about family planning at least once or twice a year.

	1	2	3	4	5	6	7	8	9	10	Don't know
1-Lowest -> Highest-10	○	○	○	○	○	○	○	○	○	○	○

* FP 3.3 Media Coverage for FP: Extent to and frequency with which national media cover/broadcast FP topics that provides the population with information on family planning and service sites.

	1	2	3	4	5	6	7	8	9	10	Don't know
1-Lowest -> Highest-10	○	○	○	○	○	○	○	○	○	○	○

* FP 3.4 FP Effort Influences - Justification: How important is each of the following as a current government justification for the national FP program? (1 means negligible importance; 10 means great importance.)

	1	2	3	4	5	6	7	8	9	10	Don't know
FP 3.4.1 Reduce the rate of population growth	◯	◯	◯	◯	◯	◯	◯	◯	◯	◯	◯
FP 3.4.2 Enhance economic development	◯	◯	◯	◯	◯	◯	◯	◯	◯	◯	◯
FP 3.4.3 Help women and men avoid unplanned and unwanted births	◯	◯	◯	◯	◯	◯	◯	◯	◯	◯	◯
FP 3.4.4 Improve women's health	◯	◯	◯	◯	◯	◯	◯	◯	◯	◯	◯
FP 3.4.5 Improve child health	◯	◯	◯	◯	◯	◯	◯	◯	◯	◯	◯
FP 3.4.6 Reduce unmarried adolescent childbearing	◯	◯	◯	◯	◯	◯	◯	◯	◯	◯	◯
FP 3.4.7 Reduce unmet need for contraceptive services	◯	◯	◯	◯	◯	◯	◯	◯	◯	◯	◯

* FP 3.5 FP Effort Influences – Special Populations: To what extent does the family planning program give particular emphasis to special populations? (1 means negligible emphasis; 10 means great emphasis)

	1	2	3	4	5	6	7	8	9	10	Don't know
FP 3.5.1 Unmarried youth	◯	◯	◯	◯	◯	◯	◯	◯	◯	◯	◯
FP 3.5.2 The poor	◯	◯	◯	◯	◯	◯	◯	◯	◯	◯	◯
FP 3.5.3 The disabled	◯	◯	◯	◯	◯	◯	◯	◯	◯	◯	◯
FP 3.5.4 Rural population	◯	◯	◯	◯	◯	◯	◯	◯	◯	◯	◯
FP 3.5.5 Other vulnerable groups (e.g. minority and/or indigenous groups)	◯	◯	◯	◯	◯	◯	◯	◯	◯	◯	◯
FP 3.5.6 Postpartum women for counseling and contraceptive services	◯	◯	◯	◯	◯	◯	◯	◯	◯	◯	◯
FP 3.5.7 Post-abortion women for counseling and contraceptive services	◯	◯	◯	◯	◯	◯	◯	◯	◯	◯	◯

* FP 3.6 Social Marketing: Extent of coverage of the country by a social marketing program (subsidized contraceptive sales at low cost in commercial sector, especially in urban areas).

	1	2	3	4	5	6	7	8	9	10	Don't know
1-Lowest -> Highest-10	○	○	○	○	○	○	○	○	○	○	○

FP 3.7 Comments: Please use this section to provide any additional comments about the level of effort, challenges, or successes in the country around advocacy for FP. If you marked “I don’t know” for any of the questions, also use this section to explain why you gave that rating.

JOHNS HOPKINS
BLOOMBERG SCHOOL
of PUBLIC HEALTH

Bill & Melinda Gates Institute for Population and Reproductive Health

Demographic Dividend Effort Index Survey

Dimension 4. Research

* FP 4.1 Strength of Observatory/Stakeholder/Technical Working Group for FP: Extent to which country-level FP stakeholders (e.g., stakeholder leadership group, technical working group, FP observatory, country coordination and facilitation group):

	1	2	3	4	5	6	7	8	9	10	Don't know
FP 4.1.1 Have representation in government, training institutions, civil society, nongovernmental and faith-based organizations, professional associations, the private sector, etc.											
FP 4.1.2 Meet regularly, report and recommend policy for senior management within the appropriate ministries											
FP 4.1.3 Make an impact on FP within the country											

* FP 4.2 Education/Research Strategy: Extent to which the national FP plan and/or other national documents include a comprehensive strategy/approach on FP-related research.

	1	2	3	4	5	6	7	8	9	10	Don't know
1-Lowest -> Highest-10											

* FP 4.3 Data Gathering Partners:

	1	2	3	4	5	6	7	8	9	10	Don't know
FP 4.3.1 Extent to which research is undertaken by government agencies. (This may include statistical agencies/bureaus and/or ministries of economics/finances, education, health, gender, etc.)	○	○	○	○	○	○	○	○	○	○	○
FP 4.3.2 Extent to which research is undertaken by research institutions.	○	○	○	○	○	○	○	○	○	○	○
FP 4.3.3 Extent to which research is undertaken by independent researchers.	○	○	○	○	○	○	○	○	○	○	○

* FP 4.4 Topical Data Gathering:

	1	2	3	4	5	6	7	8	9	10	Don't know
FP 4.4.1 Extent to which family planning uptake (including method mix), demand, and unmet need is researched.	○	○	○	○	○	○	○	○	○	○	○
FP 4.4.2 Extent to which barriers to FP uptake is researched.	○	○	○	○	○	○	○	○	○	○	○
FP 4.4.3 Extent to which the impacts of FP programs are researched.	○	○	○	○	○	○	○	○	○	○	○
FP 4.4.4 Extent to which the quality of service provision for FP is researched.	○	○	○	○	○	○	○	○	○	○	○

* FP 4.5 Quality/Coverage of Data: Extent to which current research/data is sex and age disaggregated. Extent to which the country participates in standardized tests and its ranking.

	1	2	3	4	5	6	7	8	9	10	Don't know
FP 4.5.1 Extent to which a routine statistical system provides reliable periodic information on FP supplies and facilities.	○	○	○	○	○	○	○	○	○	○	○
FP 4.5.2 Extent to which a routine statistical system provides good periodic information on FP services and personnel.	○	○	○	○	○	○	○	○	○	○	○
FP 4.5.3 Extent to which the routine statistical system provides good periodic information on FP needs among populations in different communities.	○	○	○	○	○	○	○	○	○	○	○
FP 4.5.4 Extent to which evaluations of the health monitoring and information system are conducted and applied to ensure the reliability of data.	○	○	○	○	○	○	○	○	○	○	○

* FP 4.6 Record-Keeping: Extent to which systems for client recordkeeping, clinic reporting and feedback of results are adequate, including being connected at all levels (federal, state/province, county, etc.).

	1	2	3	4	5	6	7	8	9	10	Don't know
1-Lowest -> Highest-10	○	○	○	○	○	○	○	○	○	○	○

* FP 4.7 Quality Research Institutions: Extent to which the country supports and sustains research institutions that develop research/collect data that support the breadth and quality of information-gathering to inform national FP efforts.

	1	2	3	4	5	6	7	8	9	10	Don't know
1-Lowest -> Highest-10	○	○	○	○	○	○	○	○	○	○	○

* FP 4.8 Evaluation: Extent to which program statistics, surveys, and small studies are used by specialized staff to report on program operations and measure progress.

	1	2	3	4	5	6	7	8	9	10	Don't know
1-Lowest -> Highest-10	○	○	○	○	○	○	○	○	○	○	○

* FP 4.9 Management's Use of Evaluation Findings: Extent to which local-level program managers use research and evaluation findings to improve programming in ways suggested by findings.

	1	2	3	4	5	6	7	8	9	10	Don't know
1-Lowest -> Highest-10	○	○	○	○	○	○	○	○	○	○	○

* FP 4.10 Ministerial Use of Evaluation Findings: Extent to which the Ministry administrators/policymakers systematically utilize data to inform policies and interventions to address FP-related issues.

	1	2	3	4	5	6	7	8	9	10	Don't know
1-Lowest -> Highest-10	○	○	○	○	○	○	○	○	○	○	○

* FP 4.11 Dissemination of Information to Other Implementing Bodies: Extent to which information is shared or spread across geographical areas and throughout various levels (national, state/province/county, localities/sub-counties).

	1	2	3	4	5	6	7	8	9	10	Don't know
1-Lowest -> Highest-10	○	○	○	○	○	○	○	○	○	○	○

FP 4.12 Comments: Please use this section to provide any additional comments about the level of effort, challenges, or successes in the country around FP research. If you marked "I don't know" for any of the questions, also use this section to explain why you gave that rating.

JOHNS HOPKINS
BLOOMBERG SCHOOL
of PUBLIC HEALTH

Bill & Melinda Gates Institute for Population and Reproductive Health

Demographic Dividend Effort Index Survey

Dimension 5. Civil Society Organizations (CSOs)

* FP 5.1 CSO Actor Power: Extent to which CSO actors are in strong placements within institutions or technical working groups or networks concerned with FP.

	1	2	3	4	5	6	7	8	9	10	Don't know
1-Lowest -> Highest-10	○	○	○	○	○	○	○	○	○	○	○

* FP 5.2 Budget Analysis as a CSO Tool: Extent to which CSOs utilize budget analysis as a tool to build FP advocacy cases.

	1	2	3	4	5	6	7	8	9	10	Don't know
1-Lowest -> Highest-10	○	○	○	○	○	○	○	○	○	○	○

* FP 5.3 Facility-Based Service Delivery Support: Extent to which CSOs are involved in efforts to increase the use and/or quality of services, particularly through increasing the technical and/or operational capacity of CSO-partnered clinics to provide FP services.

	1	2	3	4	5	6	7	8	9	10	Don't know
1-Lowest -> Highest-10	○	○	○	○	○	○	○	○	○	○	○

* FP 5.4 Community-Based Service Delivery Support: Extent to which CSOs participate in bringing FP information and services to the communities where people live through activities such as supporting the capacity-building of community-level health workers, expanding the range of contraceptive methods provided by community health workers, educating lower cadre health workers, and/or undertaking information and education campaigns to increase the acceptability of contraception.

	1	2	3	4	5	6	7	8	9	10	Don't know
1-Lowest -> Highest-10	○	○	○	○	○	○	○	○	○	○	○

* FP 5.5 Mobile Outreach for Community-Based Service Delivery Support: Extent to which CSOs support outreach models to equip health care providers with FP commodities, supplies, equipment and vehicles, which are then sent as specialist team to provide FP information and methods directly to communities.

	1	2	3	4	5	6	7	8	9	10	Don't know
1-Lowest -> Highest-10	◯	◯	◯	◯	◯	◯	◯	◯	◯	◯	◯

* FP 5.6 Social Franchising: Extent to which CSOs are involved in social franchising opportunities/services to bring private sector services to clients at reduced prices.

	1	2	3	4	5	6	7	8	9	10	Don't know
1-Lowest -> Highest-10	◯	◯	◯	◯	◯	◯	◯	◯	◯	◯	◯

* FP 5.7 mHealth: Extent to which CSOs are leveraging mHealth to utilize text messages and other mobile technologies to improve access to FP information and services.

	1	2	3	4	5	6	7	8	9	10	Don't know
1-Lowest -> Highest-10	◯	◯	◯	◯	◯	◯	◯	◯	◯	◯	◯

* FP 5.8 Human Rights and Quality of Care: Extent to which CSOs advocate for and support rights-based approaches to FP to ensure FP service recipients experience good treatment, confidence in asking questions, and quality counselling, amongst other concerns.

	1	2	3	4	5	6	7	8	9	10	Don't know
1-Lowest -> Highest-10	◯	◯	◯	◯	◯	◯	◯	◯	◯	◯	◯

* FP 5.9 Social and Behavior Change (SBC): Extent to which CSOs support SBC interventions relating, but not limited, to life skills and peer education, adult health education, school-based education, couple's communication, community mobilization, working through gatekeepers, and/or mass media interventions for a general public audience. These may be supported through implementation at the community or individual levels and may relate to outcomes such as increase in FP uptake and changes in FP knowledge and attitudes.

	1	2	3	4	5	6	7	8	9	10	Don't know
1-Lowest -> Highest-10	◯	◯	◯	◯	◯	◯	◯	◯	◯	◯	◯

* FP 5.10 Youth: Extent to which CSOs support FP policies, interventions and programming targeting youth specifically.

	1	2	3	4	5	6	7	8	9	10	Don't know
1-Lowest -> Highest-10	◯	◯	◯	◯	◯	◯	◯	◯	◯	◯	◯

* FP 5.11 Programming for Men: Extent to which CSOs support FP policies, interventions and programming for men.

	1	2	3	4	5	6	7	8	9	10	Don't know
1-Lowest -> Highest-10	◯	◯	◯	◯	◯	◯	◯	◯	◯	◯	◯

* FP 5.12 Advocacy/Accountability: Extent to which CSOs support the training and capacity building of advocates and community members to understand policies, processes, and activities to increase community-led issue identification, collaborative problem solving and more targeted advocacy actions.

	1	2	3	4	5	6	7	8	9	10	Don't know
1-Lowest -> Highest-10	◯	◯	◯	◯	◯	◯	◯	◯	◯	◯	◯

* FP 5.13 CSO-Led Assessment and Monitoring: Extent to which CSOs assess, monitor and report on the effectiveness of policies and programming to improve accountability at the policy and provider levels.

	1	2	3	4	5	6	7	8	9	10	Don't know
1-Lowest -> Highest-10	◯	◯	◯	◯	◯	◯	◯	◯	◯	◯	◯

* FP 5.14 Forming Partnerships between CSOs: Extent to which CSOs have formed national alliances and regional partnerships to strengthen their positioning and potentially improve leadership strength and funding mechanisms.

	1	2	3	4	5	6	7	8	9	10	Don't know
1-Lowest -> Highest-10	◯	◯	◯	◯	◯	◯	◯	◯	◯	◯	◯

FP 5.15 Comments: Please use this section to provide any additional comments about the level of effort, challenges, or successes in the country around the role of the civil society in FP. If you marked “I don’t know” for any of the questions, also use this section to explain why you gave that rating.

JOHNS HOPKINS
BLOOMBERG SCHOOL
of PUBLIC HEALTH

Bill & Melinda Gates Institute for Population and Reproductive Health

Demographic Dividend Effort Index Survey

Module: Sectoral Resilience and Sustainability

Considering the health and socio-economic impact of the COVID-19 and the likely impact on the DD progress, this DD effort index has integrated questions to assess the resilience and sustainability of systems in key sectors of the DD framework. The items included in the COVID-19 related questionnaire below follow the key dimensions of resilient systems and provide critical information on the potential of an effective response to emerging infectious disease threats or other public health emergencies.

Please score each item from 1 to 10, where 1 represents the lowest score (poor state/capability) and 10 represents the highest score (great state/capability).

* Physical: facilities, equipment, system states, and capabilities.

	1	2	3	4	5	6	7	8	9	10	Don't know
FP-M1 - Plan/Prepare - State and capability of equipment, personnel and FP program structure prior to the crisis event. (1 = poor state/capability; 10 = great state/capability)	○	○	○	○	○	○	○	○	○	○	○
FP-M2 - Absorb - Integration: The level of integration of FP services and programs to mitigate the effect of COVID-19 on FP	○	○	○	○	○	○	○	○	○	○	○
FP-M3 - Absorb - Integration: Extent to which effective supply chain management was able to mitigate the effect of COVID-19 on the supply/provision of FP commodities.	○	○	○	○	○	○	○	○	○	○	○
FP-M4 - Recover - Extent to which the FP sector has been able to induce change to recover the pre-COVID-19 level of functionality.	○	○	○	○	○	○	○	○	○	○	○
FP-M5 - Recover - Financing and Donor Management: Level of resource mobilization and allocation to mitigate the effect of COVID-19 on FP.	○	○	○	○	○	○	○	○	○	○	○
FP-M6 - Adapt - Extent to which changes have been made to improve the resilience of the FP sector.	○	○	○	○	○	○	○	○	○	○	○

* Information: creation, manipulation, storage, and utilization of data.

	1	2	3	4	5	6	7	8	9	10	Don't know
FP-M7 - Plan/Prepare - Extent to which FP-related data was sufficiently prepared, presented, analyzed and stored prior to the crisis event.	○	○	○	○	○	○	○	○	○	○	○
FP-M8 - Absorb - Extent to which the FP program has been able to conduct a real-time assessment of functionality, in anticipation of cascading losses in FP service provision.	○	○	○	○	○	○	○	○	○	○	○
FP-M9 - Recover - Extent to which FP data has been used to track the progress of recovery and to anticipate recovery scenarios.	○	○	○	○	○	○	○	○	○	○	○
FP-M10 - Adapt - Extent to which the FP sector is creating and improving FP data storage and real-time use protocols.	○	○	○	○	○	○	○	○	○	○	○

* Cognitive: understanding, mental models, preconceptions, biases, and values.

	1	2	3	4	5	6	7	8	9	10	Don't know
FP-M11 - Plan/Prepare - Extent to which the FP program design and operation decisions were prepared in anticipation of crisis events.	○	○	○	○	○	○	○	○	○	○	○
FP-M12 - Absorb - Extent to which the FP sector is responding with sufficient contingency protocols and proactive crisis management.	○	○	○	○	○	○	○	○	○	○	○
FP-M13 - Absorb - Accountability: Level of effort to ensure optimal accountability for the resources allocated to support FP as part of the response to the COVID-19.	○	○	○	○	○	○	○	○	○	○	○
FP-M14 - Recover - Extent to which decisions are oriented towards recovery, which employ evidence-based communication to community members to promote safe behavior.	○	○	○	○	○	○	○	○	○	○	○
FP-M15 - Recover - Awareness: Extent to which the FP sector is building confidence through communication regarding barriers posed by COVID-19 to support access to and uptake of FP.	○	○	○	○	○	○	○	○	○	○	○
FP-M16 - Adapt - Adaptive: Extent to which the national leadership has made changes to adapt and respond to the COVID-19 threats to FP.	○	○	○	○	○	○	○	○	○	○	○

* Social: interaction, collaboration, and self-synchronization between individuals, entities and institutions.

	1	2	3	4	5	6	7	8	9	10	Don't know
FP-M17 - Plan/Prepare - Extent to which training on outbreak/crisis management was conducted through, and leveraged, social networks, social capital and institutional and cultural norms prior to the crisis event.											
FP-M18 - Absorb - Extent to which personnel and social institutions have been resourceful and accessible for the outbreak/crisis response.											
FP-M19 - Absorb - Self-Regulating: Extent to which the national leadership had the authority to make sectoral changes in a timely manner through flexible infrastructure.											
FP-M20 - Recover - Extent to which the FP sector is engaging with team and knowledge sharing to enhance systematic recovery.											
FP-M21 - Recover - Diversity: The level of engagement of a multidisciplinary team to mitigate the effects of COVID-19 on FP.											
FP-M22 - Adapt - Extent to which there are additions or changes being made to FP institutions, policies, trainings, programs and culture.											
FP-M23 - Adapt - Leadership and Management: Level of leadership demonstrated by the FP leaders to mitigate the effect of COVID-19 on FP.											

* FP M-24 Timeline: Extent of timeliness of the Government reaction - communication and implementation of measures - to mitigate the immediate and longterm impact of COVID-19.

Note: This refers to the entirety of the national reaction - not the reaction that is specific to your sector.

	1	2	3	4	5	6	7	8	9	10	Don't know
1-Lowest -> Highest-10	◯	◯	◯	◯	◯	◯	◯	◯	◯	◯	◯

FP M-25 Comments: Please use this section to provide any additional comments about the resilience and sustainability of the national FP systems based on your experience during the COVID-19 pandemics. If you marked “I don’t know” for any of the questions, also use this section to explain why you gave that rating.

JOHNS HOPKINS
BLOOMBERG SCHOOL
of PUBLIC HEALTH

Bill & Melinda Gates Institute for Population and Reproductive Health

Demographic Dividend Effort Index Survey

Maternal & Child Health (MCH) Questionnaire

Please respond to the following questions based on your experience/expertise within this specific sector.

To give a summary picture of the national demographic dividend effort, please rate the following items. Score each item from 1 to 10, where 1 represents the lowest score (very weak or no effort) and 10 represents the highest score (strong effort). Where applicable, if a policy or activity is non-existent then mark your response as 0.

Answer each item. All responses will be recorded in the format exemplified below:

Lowest effort	1	2	3	4	5	6	7	8	9	10	Highest effort	I do not know

JOHNS HOPKINS
BLOOMBERG SCHOOL
of PUBLIC HEALTH

Bill & Melinda Gates Institute for Population and Reproductive Health

Demographic Dividend Effort Index Survey

Dimension 1. Policy/Policy making

* MCH 1.1 Adequate Policy: Ministry of Health policies toward pregnancy and delivery services that account for the breadth of requirements to ensure that every mother and newborn can survive.

	1	2	3	4	5	6	7	8	9	10	Don't know
MCH 1.1.1 Extent to which reproductive, maternal and child health has been integrated into the national health policy/plan/strategy.	○	○	○	○	○	○	○	○	○	○	○
MCH 1.1.2 Extent to which national reproductive, maternal and child health strategy(ies) and implementation plan(s) have been made available.	○	○	○	○	○	○	○	○	○	○	○

* MCH 1.2 Universal Access: Extent to which there are constitutional and legal entitlements that facilitate universal access to reproductive, maternal and child health services.

	1	2	3	4	5	6	7	8	9	10	Don't know
1-Lowest -> Highest-10	○	○	○	○	○	○	○	○	○	○	○

* MCH 1.3 Multisector Involvement: Extent to which policies are developed through adequate consultation with interested parties such as other ministries, NGOs, private practitioners, and women's groups.

	1	2	3	4	5	6	7	8	9	10	Don't know
1-Lowest -> Highest-10	○	○	○	○	○	○	○	○	○	○	○

* MCH 1.4 Competent Service Providers: Extent to which policies are reasonable and evidence-based concerning which personnel can provide maternal health services (e.g. trained midwives can perform a wide range of medical procedures).

	1	2	3	4	5	6	7	8	9	10	Don't know
1-Lowest -> Highest-10											

* MCH 1.5 Abortion Legislation: Extent to which a favorable policy exists towards the legal provisioning of abortion services.

	1	2	3	4	5	6	7	8	9	10	Don't know
1-Lowest -> Highest-10											

* MCH 1.6. Post-Abortion Care Legislation: Extent to which a favorable policy exists toward the treatment of complications of abortions, including complications seen from illegal abortions.

	1	2	3	4	5	6	7	8	9	10	Don't know
1-Lowest -> Highest-10											

* MCH 1.7 Policy Reviews and Updates: Extent to which policies are regularly reviewed by high-level policymakers and reviews are used to update action plans.

	1	2	3	4	5	6	7	8	9	10	Don't know
1-Lowest -> Highest-10											

* MCH 1.8 High-Level Actor Placement: Extent and level to which the director of maternal and child health services is involved in high-level decision making.

	1	2	3	4	5	6	7	8	9	10	Don't know
1-Lowest -> Highest-10											

* MCH 1.9 Financing for MCH: Extent to which the government budgets for safe pregnancy, delivery, perinatal care (for facilities, personnel, supplies, etc.) and children's health is adequate for the needs, whether from the Ministry of Health, provincial/ county government or donor support.

	1	2	3	4	5	6	7	8	9	10	Don't know
1-Lowest -> Highest-10											

* MCH 1.10 Affordability: Extent to which all MCH-related services and drugs are affordable to all clients.

	1	2	3	4	5	6	7	8	9	10	Don't know
1-Lowest -> Highest-10											

* MCH 1.11 Private Sector: Extent to which the private sector (doctors, midwives, clinics) is active and covers a substantial share of pregnancy and delivery cases.

	1	2	3	4	5	6	7	8	9	10	Don't know
1-Lowest -> Highest-10	○	○	○	○	○	○	○	○	○	○	○

* MCH 1.12 MCH Department: Extent to which the Ministry of Health has a department that is specifically devoted to MCH.

	1	2	3	4	5	6	7	8	9	10	Don't know
1-Lowest -> Highest-10	○	○	○	○	○	○	○	○	○	○	○

* MCH 1.13 Potential Solutions: Extent to which the government recognizes potential solutions to MCH-related issues through the means of:

	1	2	3	4	5	6	7	8	9	10	Don't know
MCH 1.13.1 Regulatory efficiency	○	○	○	○	○	○	○	○	○	○	○
MCH 1.13.2 Performance and accountability	○	○	○	○	○	○	○	○	○	○	○
MCH 1.13.3 A human rights-based approach to maternal, newborn and child health	○	○	○	○	○	○	○	○	○	○	○
MCH 1.13.4 Essential health infrastructure	○	○	○	○	○	○	○	○	○	○	○
MCH 1.13.5 Community engagement	○	○	○	○	○	○	○	○	○	○	○

MCH 1.14 Comments: Please use this section to provide any additional comments about the level of effort, challenges, or successes in the country around policies/policymaking for MCH. If you marked “I don’t know” for any of the questions, also use this section to explain why you gave that rating

JOHNS HOPKINS
BLOOMBERG SCHOOL
of PUBLIC HEALTH

Bill & Melinda Gates Institute for Population and Reproductive Health

Demographic Dividend Effort Index Survey

Dimension 2. Services or Programs

* MCH 2.1 Obstetric Care Availability – All Centers: Extent to which all primary health facilities have trained staff, in place, who can provide obstetric care to:

	1	2	3	4	5	6	7	8	9	10	Don't know
MCH 2.1.1 Properly manage postpartum hemorrhage cases.	○	○	○	○	○	○	○	○	○	○	○
MCH 2.1.2 Timely and properly administer antibiotics intravenously when needed.	○	○	○	○	○	○	○	○	○	○	○
MCH 2.1.3 Perform manual removal of retained placenta.	○	○	○	○	○	○	○	○	○	○	○
MCH 2.1.4 Perform vacuum aspiration of the uterus, using MVA (manual vacuum aspiration) or an electric suction device.	○	○	○	○	○	○	○	○	○	○	○
MCH 2.1.5 Use a partograph to determine when to refer.	○	○	○	○	○	○	○	○	○	○	○
MCH 2.1.6 Have transportation arrangements to quickly move a woman with obstructed labor to a district/sub-county hospital.	○	○	○	○	○	○	○	○	○	○	○
MCH 2.1.7 Have adequate antibiotic supplies on hand (sufficient supplies of the correct types).	○	○	○	○	○	○	○	○	○	○	○

* MCH 2.2 Obstetric Care Availability – Secondary Healthcare Facilities: Extent to which all district/sub-county hospitals have trained staff, in place, who can:

	1	2	3	4	5	6	7	8	9	10	Don't know
MCH 2.2.1 Provide all functions listed above for primary health facilities.											
MCH 2.2.2 Perform blood transfusions (and have adequate supplies of safe blood on hand).											
MCH 2.2.3 Perform Cesarean section.											

* MCH 2.3 Pregnancy-Related Services: Extent to which all pregnant women have adequate access to:

	1	2	3	4	5	6	7	8	9	10	Don't know
MCH 2.3.1 Treatment for postpartum hemorrhage during or soon after delivery.											
MCH 2.3.2 Management of obstructed labor.											
MCH 2.3.3 Management of pre-eclampsia, eclampsia and its complications.											
MCH 2.3.4 Treatment of post abortion care or for abortion complications.											
MCH 2.3.5 Provision of safe abortion services.											
MCH 2.3.6 Antenatal care during pregnancy.											
MCH 2.3.7 Delivery care by a trained professional attendant.											
MCH 2.3.8 Postpartum family planning services.											
MCH 2.3.9 District/sub-county hospitals that are open 24 hours/day.											

* MCH 2.4 Ante-natal Care for Unmarried and HIV Positive Women: Extent to which, at all antenatal visits, all pregnant women:

	1	2	3	4	5	6	7	8	9	10	Don't know
MCH 2.4.1 Receive WHO-recommended complete antenatal care protocol for HIV negative women	○	○	○	○	○	○	○	○	○	○	○
MCH 2.4.2 Receive WHO-recommended complete antenatal care protocol for HIV positive women	○	○	○	○	○	○	○	○	○	○	○
MCH 2.4.3 Receive WHO-recommended complete antenatal care protocol for young unmarried women.	○	○	○	○	○	○	○	○	○	○	○
MCH 2.4.4 Are informed about danger signs of obstetric and newborn complications and are assisted in planning for any emergency.	○	○	○	○	○	○	○	○	○	○	○

* MCH 2.5 Newborn Care: Extent to which, for newborn care, all infants whether delivered at home or in a facility:

	1	2	3	4	5	6	7	8	9	10	Don't know
MCH 2.5.1 Have their mouth and nasal passageways cleared	○	○	○	○	○	○	○	○	○	○	○
MCH 2.5.2 Are dried and kept warm immediately after birth	○	○	○	○	○	○	○	○	○	○	○
MCH 2.5.3 Receive vitamin A	○	○	○	○	○	○	○	○	○	○	○
MCH 2.5.4 Have their umbilical cord cut with a clean blade	○	○	○	○	○	○	○	○	○	○	○
MCH 2.5.5 Complete WHO recommended vaccination calendar	○	○	○	○	○	○	○	○	○	○	○
MCH 2.5.6 Benefit from exclusive breastfeeding promotion and education activities	○	○	○	○	○	○	○	○	○	○	○
MCH 2.5.7 Benefit from nutritional support programs when in need.	○	○	○	○	○	○	○	○	○	○	○

* MCH 2.6 Family Planning – All Centers: Extent to which there exists proper provision of family planning at all health centers that:

	1	2	3	4	5	6	7	8	9	10	Don't know
MCH 2.6.1 Routinely offer family planning services for post-abortion cases	○	○	○	○	○	○	○	○	○	○	○
MCH 2.6.2 Routinely offer family planning at postpartum visits	○	○	○	○	○	○	○	○	○	○	○
MCH 2.6.3 Have short-term and LARC contraceptive supplies regularly in stock	○	○	○	○	○	○	○	○	○	○	○
MCH 2.6.4 Have trained staff, in place, who can provide services for LARCs.	○	○	○	○	○	○	○	○	○	○	○
MCH 2.6.5 Have guidelines and protocols necessary for the provision of family planning services.	○	○	○	○	○	○	○	○	○	○	○

* MCH 2.7 Family Planning – District/Sub-county Hospitals: Extent to which there exists proper provision of family planning at district/sub-county hospitals that:

	1	2	3	4	5	6	7	8	9	10	Don't know
MCH 2.7.1 Routinely offer family planning services for post-abortion cases	○	○	○	○	○	○	○	○	○	○	○
MCH 2.7.2 Routinely offer family planning at postpartum visits	○	○	○	○	○	○	○	○	○	○	○
MCH 2.7.3 Have short term and LARC contraceptive supplies regularly in stock	○	○	○	○	○	○	○	○	○	○	○
MCH 2.7.4 Have trained staff, in place, who can provide services for LARCs	○	○	○	○	○	○	○	○	○	○	○
MCH 2.7.5 Can offer sterilization to female clients	○	○	○	○	○	○	○	○	○	○	○
MCH 2.7.6 Can offer sterilization to male clients	○	○	○	○	○	○	○	○	○	○	○
MCH 2.7.7 Have guidelines and protocols necessary for the provision of family planning services.	○	○	○	○	○	○	○	○	○	○	○

* MCH 2.8 Childhood Illness: Extent to which the WHO-recommended case management of childhood illness is implemented regarding:

	1	2	3	4	5	6	7	8	9	10	Don't know
MCH 2.8.1 Community case management of childhood illnesses	○	○	○	○	○	○	○	○	○	○	○
MCH 2.8.2 Effectiveness of the referral services from community level to the health facilities	○	○	○	○	○	○	○	○	○	○	○

MCH 2.9 Comments: Please use this section to provide any additional comments about the level of effort, challenges, or successes in the country around services or programs for MCH. If you marked “I don’t know” for any of the questions, also use this section to explain why you gave that rating.

JOHNS HOPKINS
BLOOMBERG SCHOOL
of PUBLIC HEALTH

Bill & Melinda Gates Institute for Population and Reproductive Health

Demographic Dividend Effort Index Survey

Dimension 3. Advocacy

* MCH 3.1 Government Messages: Extent to which high officials in the government, including the Ministry of Health, issue frequent statements to all stakeholders including the press to support improvements for safe pregnancy, safe delivery and healthy childhood.

	1	2	3	4	5	6	7	8	9	10	Don't know
1-Lowest -> Highest-10	◯	◯	◯	◯	◯	◯	◯	◯	◯	◯	◯

* MCH 3.2 Mass Media: Extent to which the national program uses the mass media to educate the public about symptoms of pregnancy complications, safe childbirth and healthy childhood.

	1	2	3	4	5	6	7	8	9	10	Don't know
1-Lowest -> Highest-10	◯	◯	◯	◯	◯	◯	◯	◯	◯	◯	◯

* MCH 3.3 Community-Level Media: Extent to which community groups take part in systematic programs to educate the public about safe pregnancy, safe delivery and healthy childhood.

	1	2	3	4	5	6	7	8	9	10	Don't know
1-Lowest -> Highest-10	◯	◯	◯	◯	◯	◯	◯	◯	◯	◯	◯

* MCH 3.4 Educational Materials: Extent to which the appropriate ministry (MOH) supplies adequate educational materials (posters, pamphlets, etc.) to delivery facilities to instruct clients about safe practices.

	1	2	3	4	5	6	7	8	9	10	Don't know
1-Lowest -> Highest-10	◯	◯	◯	◯	◯	◯	◯	◯	◯	◯	◯

* MCH 3.5 Self-Review of Maternal Mortality Cases: Extent to which each health facility follows a regular procedure to review and learn from every case of a maternal death in the facility.

	1	2	3	4	5	6	7	8	9	10	Don't know
1-Lowest -> Highest-10	◯	◯	◯	◯	◯	◯	◯	◯	◯	◯	◯

* MCH 3.6 Informing Policies with New Evidence: Extent to which each health facility shares lessons from maternal death cases to the ministries to inform new policies or interventions.

	1	2	3	4	5	6	7	8	9	10	Don't know
1-Lowest -> Highest-10	◯	◯	◯	◯	◯	◯	◯	◯	◯	◯	◯

MCH 3.7 Comments: Please use this section to provide any additional comments about the level of effort, challenges, or successes in the country around advocacy for MCH. If you marked “I don’t know” for any of the questions, also use this section to explain why you gave that rating

JOHNS HOPKINS
BLOOMBERG SCHOOL of PUBLIC HEALTH

Bill & Melinda Gates Institute for Population and Reproductive Health

Demographic Dividend Effort Index Survey

Dimension 4. Research

* MCH 4.1 Strength of Observatory/Stakeholder/Technical Working Group for MCH: Extent to which country-level MCH stakeholders (e.g., stakeholder leadership group, technical working group, MCH observatory, country coordination and facilitation group):

	1	2	3	4	5	6	7	8	9	10	Don't know
MCH 4.1.1 Have representation in government, training institutions, civil society, nongovernmental and faith-based organizations, professional associations, the private sector, etc.	○	○	○	○	○	○	○	○	○	○	○
MCH 4.1.2 Meet regularly, report and recommend policy for senior management within the appropriate ministries	○	○	○	○	○	○	○	○	○	○	○
MCH 4.1.3 Make an impact on MCH within the country	○	○	○	○	○	○	○	○	○	○	○

* MCH 4.2 MCH Research Strategy: Extent to which national documents include a comprehensive strategy/approach on MCH-related research and is shared among partners.

	1	2	3	4	5	6	7	8	9	10	Don't know
1-Lowest -> Highest-10	○	○	○	○	○	○	○	○	○	○	○

* MCH 4.3 Data-Gathering Partners:

	1	2	3	4	5	6	7	8	9	10	Don't know
MCH 4.3.1 Extent to which research is undertaken by government agencies. (This may include statistical agencies/bureaus and/or ministries of economics/finances, education, health, gender, etc.)	○	○	○	○	○	○	○	○	○	○	○
MCH 4.3.2 Extent to which data-gathering is undertaken by research institutions.	○	○	○	○	○	○	○	○	○	○	○
MCH 4.3.3 Extent to which data-gathering is undertaken by independent researchers.	○	○	○	○	○	○	○	○	○	○	○

* MCH 4.4 Topical Research:

	1	2	3	4	5	6	7	8	9	10	Don't know
MCH 4.4.1 Extent to which maternal mortality is researched.	○	○	○	○	○	○	○	○	○	○	○
MCH 4.4.2 Extent to which infant and child mortality is researched.	○	○	○	○	○	○	○	○	○	○	○
MCH 4.4.3 Extent to which family planning and post-partum family planning is researched.	○	○	○	○	○	○	○	○	○	○	○
MCH 4.4.4 Extent to which MCH service provision/access and provider quality is researched.	○	○	○	○	○	○	○	○	○	○	○

* MCH 4.5 Quality/Coverage of Data: Extent to which current research/data is disaggregated, where relevant, on the basis of sex, age, geographic location, ethnicity, education, income quintile and disability status. Extent to which the country participates in standardized tests and its ranking.

	1	2	3	4	5	6	7	8	9	10	Don't know
MCH 4.5.1 Extent to which a routine statistical system provides good periodic information on supplies and facilities.	◯	◯	◯	◯	◯	◯	◯	◯	◯	◯	◯
MCH 4.5.2 Extent to which a routine statistical system provides good periodic information on MCH services and personnel.	◯	◯	◯	◯	◯	◯	◯	◯	◯	◯	◯
MCH 4.5.3 Extent to which the routine statistical system provides good periodic information on MCH needs among populations in different communities.	◯	◯	◯	◯	◯	◯	◯	◯	◯	◯	◯
MCH 4.5.4 Extent to which evaluations of the health monitoring and information system are conducted and applied to ensure the reliability of data.	◯	◯	◯	◯	◯	◯	◯	◯	◯	◯	◯
MCH 4.5.5 Extent to which evaluations of the health monitoring and information system are conducted and applied to explore inequalities in MNCH services and burdens.	◯	◯	◯	◯	◯	◯	◯	◯	◯	◯	◯

* MCH 4.6 Record-Keeping: Extent to which systems for client recordkeeping, clinic reporting and feedback of results are adequate, including being connected at all levels (federal, state/province, county, etc.).

	1	2	3	4	5	6	7	8	9	10	Don't know
1-Lowest -> Highest-10	◯	◯	◯	◯	◯	◯	◯	◯	◯	◯	◯

* MCH 4.7 Quality Research Institutions: Extent to which the country has the capacity to support and sustain research institutions that develop research/collect data to ensure high quality MCH services.

	1	2	3	4	5	6	7	8	9	10	Don't know
1-Lowest -> Highest-10	◯	◯	◯	◯	◯	◯	◯	◯	◯	◯	◯

* MCH 4.8 Evaluation: Extent to which program statistics, surveys, and small studies are used by specialized staff to report on program operations and measure progress.

	1	2	3	4	5	6	7	8	9	10	Don't know
1-Lowest -> Highest-10	◯	◯	◯	◯	◯	◯	◯	◯	◯	◯	◯

* MCH 4.9 Management's Use of Evaluation Findings: Extent to which local-level program managers use research and evaluation findings to improve programming in ways suggested by findings.

	1	2	3	4	5	6	7	8	9	10	Don't know
1-Lowest -> Highest-10	◯	◯	◯	◯	◯	◯	◯	◯	◯	◯	◯

* MCH 4.10 Ministerial Use of Evaluation Findings: Extent to which the Ministry administrators and policymakers systematically utilize data to inform policies and interventions to address MCH-related issues.

	1	2	3	4	5	6	7	8	9	10	Don't know
1-Lowest -> Highest-10	◯	◯	◯	◯	◯	◯	◯	◯	◯	◯	◯

* MCH 4.11 Dissemination of Information to Other Implementing Bodies: Extent to which information is shared or spread across geographical areas and throughout various levels (national, state/province, localities).

	1	2	3	4	5	6	7	8	9	10	Don't know
1-Lowest -> Highest-10	◯	◯	◯	◯	◯	◯	◯	◯	◯	◯	◯

MCH 4.12 Comments: Please use this section to provide any additional comments about the level of effort, challenges, or successes in the country around MCH research. If you marked "I don't know" for any of the questions, also use this section to explain why you gave that rating.

JOHNS HOPKINS
BLOOMBERG SCHOOL
of PUBLIC HEALTH

Bill & Melinda Gates Institute for Population and Reproductive Health

Demographic Dividend Effort Index Survey

Dimension 5. Civil Society Organizations (CSOs)

* MCH 5.1 CSO Actor Power: Extent to which CSO actors are in strong placements within institutions or technical working groups or networks concerned with MCH.

	1	2	3	4	5	6	7	8	9	10	Don't know
1-Lowest -> Highest-10	○	○	○	○	○	○	○	○	○	○	○

* MCH 5.2 Budget Analysis as a CSO Tool: Extent to which CSOs utilize budget analysis as a tool to build MCH advocacy cases.

	1	2	3	4	5	6	7	8	9	10	Don't know
1-Lowest -> Highest-10	○	○	○	○	○	○	○	○	○	○	○

* MCH 5.3 Facility-Based Service Delivery Support: Extent to which CSOs are involved in efforts to increase the use and/or quality of services, particularly through increasing the technical and/or operational capacity of CSO-partnered clinics to provide MCH services.

	1	2	3	4	5	6	7	8	9	10	Don't know
1-Lowest -> Highest-10	○	○	○	○	○	○	○	○	○	○	○

* MCH 5.4 Community-Based Service Delivery Support: Extent to which CSOs participate in bringing MCH information and services to the communities where people live through activities such as supporting the capacity-building of community-level health workers, expanding the range of MCH services provided by community health workers, educating lower cadre health workers, and/or undertaking information and education campaigns.

	1	2	3	4	5	6	7	8	9	10	Don't know
1-Lowest -> Highest-10	○	○	○	○	○	○	○	○	○	○	○

* MCH 5.5 Mobile Outreach for Community-Based Service Delivery Support: Extent to which CSOs support outreach models to equip health care providers with MCH-related commodities, supplies, equipment and vehicles, which are then sent as specialist team to provide MCH services directly to communities.

	1	2	3	4	5	6	7	8	9	10	Don't know
1-Lowest -> Highest-10	○	○	○	○	○	○	○	○	○	○	○

* MCH 5.6 Social Franchising: Extent to which CSOs are involved with social franchising opportunities/services to bring private sector services to clients at reduced prices of MCH services.

	1	2	3	4	5	6	7	8	9	10	Don't know
1-Lowest -> Highest-10	○	○	○	○	○	○	○	○	○	○	○

* MCH 5.7 mHealth: Extent to which CSOs are leveraging mHealth to utilize text messages and other mobile technologies to improve access to MCH information and services.

	1	2	3	4	5	6	7	8	9	10	Don't know
1-Lowest -> Highest-10	○	○	○	○	○	○	○	○	○	○	○

* MCH 5.8 Human Rights and Quality of Care: Extent to which CSOs advocate for and support rights-based approaches to MCH to ensure MCH service recipients experience good treatment, confidence in asking questions, and quality counselling, amongst other concerns.

	1	2	3	4	5	6	7	8	9	10	Don't know
1-Lowest -> Highest-10	○	○	○	○	○	○	○	○	○	○	○

* MCH 5.9 Social and Behavior Change (SBC): Extent to which CSOs support SBC interventions relating, but not limited, to life skills and peer education, adult health education, school-based education, couple's communication, community mobilization, working through gatekeepers, and/or mass media interventions for a general public audience.

	1	2	3	4	5	6	7	8	9	10	Don't know
1-Lowest -> Highest-10	○	○	○	○	○	○	○	○	○	○	○

* MCH 5.10 Advocacy/Accountability: Extent to which CSOs support the training and capacity building of advocates and community members to understand policies, processes, and activities to increase community-led issue identification, collaborative problem solving and more targeted advocacy actions.

	1	2	3	4	5	6	7	8	9	10	Don't know
1-Lowest -> Highest-10	○	○	○	○	○	○	○	○	○	○	○

* MCH 5.11 CSO-Led Assessment and Monitoring: Extent to which CSOs assess, monitor and report on the effectiveness of policies and programming to improve accountability at the policy and provider levels.

	1	2	3	4	5	6	7	8	9	10	Don't know
1-Lowest -> Highest-10	○	○	○	○	○	○	○	○	○	○	○

* MCH 5.12 Forming Partnerships between CSOs: Extent to which CSOs have formed national alliances and regional partnerships to strengthen their positioning and potentially improve leadership strength and funding mechanisms for MCH.

	1	2	3	4	5	6	7	8	9	10	Don't know
1-Lowest -> Highest-10	○	○	○	○	○	○	○	○	○	○	○

MCH 5.13 Comments: Please use this section to provide any additional comments about the level of effort, challenges, or successes in the country around the involvement of the civil society in MCH. If you marked “I don’t know” for any of the questions, also use this section to explain why you gave that rating

JOHNS HOPKINS
BLOOMBERG SCHOOL
of PUBLIC HEALTH

Bill & Melinda Gates Institute for Population and Reproductive Health

Demographic Dividend Effort Index Survey

Module: Sectoral Resilience and Sustainability

Considering the health and socio-economic impact of the COVID-19 and the likely impact on the DD progress, this DD effort index has integrated questions to assess the resilience and sustainability of systems in key sectors of the DD framework. The items included in the COVID-19 related questionnaire below follow the key dimensions of resilient systems and provide critical information on the potential of an effective response to emerging infectious disease threats or other public health emergencies.

Please score each item from 1 to 10, where 1 represents the lowest score (poor state/capability) and 10 represents the highest score (great state/capability).

* Physical: facilities, equipment, system states, and capabilities.

	1	2	3	4	5	6	7	8	9	10	Don't know
MCH-M1 - Plan/Prepare - State and capability of equipment, personnel and MCH program structure prior to the crisis event. (1 = poor state/capability; 10 = great state/capability)	◯	◯	◯	◯	◯	◯	◯	◯	◯	◯	◯
MCH-M2 - Absorb - Integration: The level of integration of MCH services and programs to mitigate the effect of COVID-19 on MCH.	◯	◯	◯	◯	◯	◯	◯	◯	◯	◯	◯
MCH-M3 - Absorb - Integration: Extent to which effective supply chain management was able to mitigate the effect of COVID-19 on the supply/provision of MCH-related commodities.	◯	◯	◯	◯	◯	◯	◯	◯	◯	◯	◯
MCH-M4 - Recover - Extent to which the MCH program has been able to induce change to recover the pre-COVID-19 level of functionality.	◯	◯	◯	◯	◯	◯	◯	◯	◯	◯	◯
MCH-M5 - Recover - Financing and Donor Management: Level of resources mobilization and allocation to mitigate the effect of COVID-19 on MCH.	◯	◯	◯	◯	◯	◯	◯	◯	◯	◯	◯
MCH-M6 - Adapt - Extent to which changes have been made to improve the resilience of the MCH sector.	◯	◯	◯	◯	◯	◯	◯	◯	◯	◯	◯

* Information: creation, manipulation, storage, and utilization of data.

	1	2	3	4	5	6	7	8	9	10	Don't know
MCH-M7 - Plan/Prepare - Extent to which MCH-related data was sufficiently prepared, presented, analyzed and stored prior to the crisis event.	○	○	○	○	○	○	○	○	○	○	○
MCH-M8 - Absorb - Awareness: Extent of the surveillance capacity to detect the MCH limitations/threats posed by COVID-19.	○	○	○	○	○	○	○	○	○	○	○
MCH-M9 - Recover - Extent to which MCH data has been used to track the progress of recovery and to anticipate recovery scenarios.	○	○	○	○	○	○	○	○	○	○	○
MCH-M10 - Adapt - Extent to which the MCH sector is creating and improving MCH data storage and real-time use protocols.	○	○	○	○	○	○	○	○	○	○	○

* Cognitive: understanding, mental models, preconceptions, biases, and values.

	1	2	3	4	5	6	7	8	9	10	Don't know
MCH-M11 - Plan/Prepare - Extent to which the MCH sector design and operation decisions were prepared in anticipation of crisis events.	○	○	○	○	○	○	○	○	○	○	○
MCH-M12 - Absorb - Extent to which the MCH sector is responding with sufficient contingency protocols and proactive crisis management.	○	○	○	○	○	○	○	○	○	○	○
MCH-M13 - Absorb - Accountability: Level of effort to ensure optimal accountability for the resources allocated to support MCH as part of the response to the COVID-19.	○	○	○	○	○	○	○	○	○	○	○
MCH-M14 - Recover - Extent to which decisions are oriented towards recovery, which employ evidence-based communication to community members to promote safe behavior.	○	○	○	○	○	○	○	○	○	○	○
MCH-M15 - Recover - Awareness: Extent to which the MCH sector is building confidence through communication regarding barriers posed by COVID-19 to support access to and uptake of MCH services and programs.	○	○	○	○	○	○	○	○	○	○	○
MCH-M16 - Adapt – Adaptive: Extent to which the national leadership had the authority to make changes to adapt and respond to the COVID-19 threats to MCH services and programs.	○	○	○	○	○	○	○	○	○	○	○

* Social: interaction, collaboration, and self-synchronization between individuals, entities and institutions.

	1	2	3	4	5	6	7	8	9	10	Don't know
MCH-M17 - Plan/Prepare - Extent to which training on outbreak/crisis management was conducted through, and which leveraged, social networks, social capital and institutional and cultural norms prior to the crisis event.	○	○	○	○	○	○	○	○	○	○	○
MCH-M18 - Absorb - Extent to which personnel and social institutions have been resourceful and accessible for the outbreak/crisis response.	○	○	○	○	○	○	○	○	○	○	○
MCH-M19 - Absorb - Self-Regulating: Extent to which the national leadership had the authority to make sectoral changes in a timely manner through flexible infrastructure.	○	○	○	○	○	○	○	○	○	○	○
MCH-M20 - Recover - Extent to which the MCH sector is engaging with team and knowledge sharing to enhance systematic recovery.	○	○	○	○	○	○	○	○	○	○	○
MCH-M21 - Recover - Diversity: The level of engagement of a multidisciplinary team to mitigate the effect of COVID19 on MCH.	○	○	○	○	○	○	○	○	○	○	○
MCH-M22 - Adapt - Extent to which there are additions or changes being made to MCH institutions, policies, trainings, programs and culture.	○	○	○	○	○	○	○	○	○	○	○

	1	2	3	4	5	6	7	8	9	10	Don't know
MCH-M23 - Adapt - Leadership and Management: Level of leadership demonstrated by the MCH leaders to mitigate the effect of COVID-19 on MCH.	○	○	○	○	○	○	○	○	○	○	○

* MCH M-24 Timeline: Extent of timeliness of the Government reaction - communication and implementation of measures - to mitigate the immediate and longterm impact of COVID-19.
Note: This refers to the entirety of the national reaction - not the reaction that is specific to your sector.

	1	2	3	4	5	6	7	8	9	10	Don't know
1-Lowest -> Highest-10	○	○	○	○	○	○	○	○	○	○	○

MCH M-25 Comments: Please use this section to provide any additional comments about the resilience and sustainability of the national MCH systems based on your experience during the COVID-19 pandemics. If you marked “I don’t know” for any of the questions, also use this section to explain why you gave that rating.

JOHNS HOPKINS
BLOOMBERG SCHOOL
of PUBLIC HEALTH

Bill & Melinda Gates Institute for Population and Reproductive Health

Demographic Dividend Effort Index Survey

Education (ED) Questionnaire

Please respond to the following questions based on your experience/expertise within this specific sector.

To give a summary picture of the national demographic dividend effort, please rate the following items. Score each item from 1 to 10, where 1 represents the lowest score (very weak or no effort) and 10 represents the highest score (strong effort). Where applicable, if a policy or activity is non-existent then mark your response as 0.

Answer each item. All responses will be recorded in the format exemplified below:

Lowest effort	1	2	3	4	5	6	7	8	9	10	Highest effort	I do not know

JOHNS HOPKINS BLOOMBERG SCHOOL of PUBLIC HEALTH

Bill & Melinda Gates Institute for Population and Reproductive Health

Demographic Dividend Effort Index Survey

Dimension 1. Policy / Policy making

* ED 1.1 Quality: Extent to which there are national plans/strategies/programs to improve the quality of learning in schools.

	1	2	3	4	5	6	7	8	9	10	Don't know
1-Lowest -> Highest-10	◯	◯	◯	◯	◯	◯	◯	◯	◯	◯	◯

* ED 1.2 Disadvantaged Groups:

	1	2	3	4	5	6	7	8	9	10	Don't know
ED 1.2.1. Extent to which strategies have been developed and implemented/deployed to overcome learning obstacles for pregnant girls and adolescent mothers.	◯	◯	◯	◯	◯	◯	◯	◯	◯	◯	◯
ED 1.2.2 Extent to which strategies have been developed and implemented/deployed to overcome learning obstacles for disabled populations.	◯	◯	◯	◯	◯	◯	◯	◯	◯	◯	◯

* ED 1.3 Teachers: Extent to which teacher recruitment, development and management issues are included in policy aimed at improving learning outcomes.

	1	2	3	4	5	6	7	8	9	10	Don't know
1-Lowest -> Highest-10	◯	◯	◯	◯	◯	◯	◯	◯	◯	◯	◯

* ED 1.4 Teachers' Accountability: Extent to which national plans include specific measures, including monitoring and evaluation, that aim to hold teachers' accountable for improving learning.

	1	2	3	4	5	6	7	8	9	10	Don't know
1-Lowest -> Highest-10	○	○	○	○	○	○	○	○	○	○	○

* ED 1.5 Teacher Quality: Extent to which education policies/strategies/programs include verifiable indicators for teacher quality.

	1	2	3	4	5	6	7	8	9	10	Don't know
1-Lowest -> Highest-10	○	○	○	○	○	○	○	○	○	○	○

* ED 1.6 Collaboration in Education: Extent to which the policy makes provisions to engage multiple stakeholders in education (e.g. schools, school-communities, parents, teachers, local government, parent-teacher-student committees, etc.).

	1	2	3	4	5	6	7	8	9	10	Don't know
1-Lowest -> Highest-10	○	○	○	○	○	○	○	○	○	○	○

* ED 1.7 Gender Parity SDG: Extent to which national policies/plans/strategies consider the SDG goal for complete gender parity at all levels of education.

	1	2	3	4	5	6	7	8	9	10	Don't know
1-Lowest -> Highest-10	○	○	○	○	○	○	○	○	○	○	○

* ED 1.8 Education Financing: Extent to which new education plans include financing proposals with budgets allocated for learning and teaching reforms.

	1	2	3	4	5	6	7	8	9	10	Don't know
1-Lowest -> Highest-10	○	○	○	○	○	○	○	○	○	○	○

* ED 1.9 Gender Parity Planning: Extent to which there are national plans/strategies to reach 100% enrollment with gender parity in:

	1	2	3	4	5	6	7	8	9	10	Don't know
ED 1.9.1 Primary Education	○	○	○	○	○	○	○	○	○	○	○
ED 1.9.2 Secondary Education	○	○	○	○	○	○	○	○	○	○	○
ED 1.9.3 Tertiary Education	○	○	○	○	○	○	○	○	○	○	○

* ED 1.10 Adult Illiteracy: Extent to which national policies/plans/strategies aim to address illiteracy.

	1	2	3	4	5	6	7	8	9	10	Don't know
ED 1.10.1 Extent to which national policies/plans/strategies aim to address adult illiteracy.	○	○	○	○	○	○	○	○	○	○	○
ED 1.10.2 Extent to which national policies/plans/strategies that aim to address adult illiteracy address gendered disparities of this issue.	○	○	○	○	○	○	○	○	○	○	○

* ED 1.11 Out-of-School Children: Extent to which policies address the plight of out-of-school children to reintegrate them into the school system or provide alternative opportunities for their educational development.

	1	2	3	4	5	6	7	8	9	10	Don't know
1-Lowest -> Highest-10	○	○	○	○	○	○	○	○	○	○	○

* ED 1.12 Technology Integration: Extent to which strategies have been developed and implemented/deployed to integrate information communication and technology in education.

	1	2	3	4	5	6	7	8	9	10	Don't know
1-Lowest -> Highest-10	○	○	○	○	○	○	○	○	○	○	○

* ED 1.13. Technology Literacy: Extent to which resources have been allocated to improve computer literacy across education levels.

	1	2	3	4	5	6	7	8	9	10	Don't know
1-Lowest -> Highest-10	○	○	○	○	○	○	○	○	○	○	○

ED 1.14. Comments: Please use this section to provide any additional comments about the level of effort, challenges, or successes in the country around policies/policymaking for ED. If you marked “I don’t know” for any of the questions, also use this section to explain why you gave that rating.

JOHNS HOPKINS
BLOOMBERG SCHOOL
of PUBLIC HEALTH

Bill & Melinda Gates Institute for Population and Reproductive Health

Demographic Dividend Effort Index Survey

Dimension 2. Services or Programs

* ED 2.1 Teacher Deployment: Extent to which strategies/plans are used to ensure teachers are deployed to areas of the country where they are most needed.

	1	2	3	4	5	6	7	8	9	10	Don't know
1-Lowest -> Highest-10	○	○	○	○	○	○	○	○	○	○	○

* ED 2.2 Teacher Quality: Extent to which teacher quality is recognized as a key factor in improving learning outcomes.

	1	2	3	4	5	6	7	8	9	10	Don't know
ED 2.2.1 Extent to which the quality of teacher education is emphasized in wider education policies/plans/strategies.	○	○	○	○	○	○	○	○	○	○	○
ED 2.2.2 Extent to which the quality of training and continuous professional development is emphasized in wider education policies/plans/strategies.	○	○	○	○	○	○	○	○	○	○	○

* ED 2.3 Gender Parity: Extent to which resources have been utilized to improve equality in education between boys and girls.

	1	2	3	4	5	6	7	8	9	10	Don't know
1-Lowest -> Highest-10	○	○	○	○	○	○	○	○	○	○	○

* ED 2.4 Parental Involvement in Education: Extent to which parents are involved in the development and execution of the education strategies (i.e. through the involvement of parent-teacher committees).

	1	2	3	4	5	6	7	8	9	10	Don't know
1-Lowest -> Highest-10											

* ED 2.5 Standardized Assessments: Extent to which all schools utilize standardized school and student assessments in line with specifications made by the Ministry of Education.

	1	2	3	4	5	6	7	8	9	10	Don't know
1-Lowest -> Highest-10											

* ED 2.6. Use of Assessment to Inform Progress: Extent to which adjustments to schools are made based on results from national standardized school assessments.

	1	2	3	4	5	6	7	8	9	10	Don't know
1-Lowest -> Highest-10											

ED 2.7 Comments: Please use this section to provide any additional comments about the level of effort, challenges, or successes in the country around services or programs for ED. If you marked “I don’t know” for any of the questions, also use this section to explain why you gave that rating.

JOHNS HOPKINS
BLOOMBERG SCHOOL
of PUBLIC HEALTH

Bill & Melinda Gates Institute for Population and Reproductive Health

Demographic Dividend Effort Index Survey

Dimension 3. Advocacy

* ED 3.1 Equality: Extent to which equity and equality in education have been highlighted by advocates.

	1	2	3	4	5	6	7	8	9	10	Don't know
1-Lowest -> Highest-10	◯	◯	◯	◯	◯	◯	◯	◯	◯	◯	◯

* ED 3.2 Quality: Extent to which quality of education takes part in advocacy-related evaluations of the education system.

	1	2	3	4	5	6	7	8	9	10	Don't know
1-Lowest -> Highest-10	◯	◯	◯	◯	◯	◯	◯	◯	◯	◯	◯

* ED 3.3 Teachers' Obstacles: Extent to which the needs of teachers have been highlighted by advocates.

	1	2	3	4	5	6	7	8	9	10	Don't know
1-Lowest -> Highest-10	◯	◯	◯	◯	◯	◯	◯	◯	◯	◯	◯

* ED 3.4 Institutions' Obstacles: Extent to which the challenges of educational institutions have been highlighted in different advocacy efforts.

	1	2	3	4	5	6	7	8	9	10	Don't know
1-Lowest -> Highest-10	◯	◯	◯	◯	◯	◯	◯	◯	◯	◯	◯

ED 3.5 Comments: Please use this section to provide any additional comments about the level of effort, challenges, or successes in the country around advocacy for ED. If you marked "I don't know" for any of the questions, also use this section to explain why you gave that rating

JOHNS HOPKINS
BLOOMBERG SCHOOL
of PUBLIC HEALTH

Bill & Melinda Gates Institute for Population and Reproductive Health

Demographic Dividend Effort Index Survey

Dimension 4. Research

* ED 4.1 Strength of Observatory/Stakeholder/Technical Working Group for ED: Extent to which country-level ED stakeholders (e.g., stakeholder leadership group, technical working group, ED observatory, country coordination and facilitation group):

	1	2	3	4	5	6	7	8	9	10	Don't know
ED 4.1.1 Have representation in government, training institutions, civil society, nongovernmental and faith-based organizations, professional associations, the private sector, etc.	○	○	○	○	○	○	○	○	○	○	○
ED 4.1.2 Meet regularly, report and recommend policy for senior management within the appropriate ministries	○	○	○	○	○	○	○	○	○	○	○
ED 4.1.3 Make an impact on education within the country	○	○	○	○	○	○	○	○	○	○	○

* ED 4.2 Education/Research Strategy: Extent to which the national education plan and/or other national documents include a comprehensive strategy/approach on education-related research, shared among partners.

	1	2	3	4	5	6	7	8	9	10	Don't know
1-Lowest -> Highest-10	○	○	○	○	○	○	○	○	○	○	○

* ED 4.3 Data Gathering Partners:

	1	2	3	4	5	6	7	8	9	10	Don't know
ED 4.3.1 Extent to which research is undertaken by government agencies. (This may include statistical agencies/bureaus and/or ministries of economics/finances, education, health, gender, etc.)											
ED 4.3.2 Extent to which data-gathering is undertaken by research institutions.											
ED 4.3.3 Extent to which data-gathering is undertaken by independent researchers.											

* ED 4.4 Topical Research:

	1	2	3	4	5	6	7	8	9	10	Don't know
ED 4.4.1 Extent to which gender disparities in education are researched.											
ED 4.4.2 Extent to which quality of education is researched.											
ED 4.4.3 Extent to which the obstacles faced by teachers are researched.											
ED 4.4.4 Extent to which the obstacles faced by institutions are researched.											

* ED 4.5 Quality/Coverage of Data: Extent to which current research/data is sex and age disaggregated. Extent to which the country participates in standardized tests and its ranking.

	1	2	3	4	5	6	7	8	9	10	Don't know
ED 4.5.1 Extent to which a routine statistical system (using institution/school-based information) provides good periodic information on supplies and facilities.	◯	◯	◯	◯	◯	◯	◯	◯	◯	◯	◯
ED 4.5.2 Extent to which a routine statistical system (using institution/school-based information) provides good periodic information on education services and personnel.	◯	◯	◯	◯	◯	◯	◯	◯	◯	◯	◯
ED 4.5.3 Extent to which the routine statistical system provides good periodic information on education needs among populations in different communities.	◯	◯	◯	◯	◯	◯	◯	◯	◯	◯	◯
ED 4.5.4 Extent to which evaluations of the education monitoring and information system are conducted and applied to ensure the reliability of data.	◯	◯	◯	◯	◯	◯	◯	◯	◯	◯	◯

* ED 4.6 Record-Keeping: Extent to which systems for education-related recordkeeping, reporting and feedback of results are adequate.

	1	2	3	4	5	6	7	8	9	10	Don't know
1-Lowest -> Highest-10	◯	◯	◯	◯	◯	◯	◯	◯	◯	◯	◯

* ED 4.7 Quality Research Institutions: Extent to which the country has the capacity to support and sustain research institutions that develop research/collect data that inform efforts to improve education.

	1	2	3	4	5	6	7	8	9	10	Don't know
1-Lowest -> Highest-10	◯	◯	◯	◯	◯	◯	◯	◯	◯	◯	◯

* ED 4.8 Evaluation: Extent to which education program statistics, surveys, and small studies are used by specialized staff to report on program operations and measure progress.

	1	2	3	4	5	6	7	8	9	10	Don't know
1-Lowest -> Highest-10	○	○	○	○	○	○	○	○	○	○	○

* ED 4.9 Management's Use of Evaluation Findings: Extent to which local-level education program managers use research and evaluation findings to improve these programs in ways suggested by findings.

	1	2	3	4	5	6	7	8	9	10	Don't know
1-Lowest -> Highest-10	○	○	○	○	○	○	○	○	○	○	○

* ED 4.10 Ministerial Use of Evaluation Findings: Extent to which the relevant ministry administrators (i.e. ministries of education) systematically utilize data to inform policies and interventions to address education-related issues.

	1	2	3	4	5	6	7	8	9	10	Don't know
1-Lowest -> Highest-10	○	○	○	○	○	○	○	○	○	○	○

* ED 4.11 Dissemination of Information to Other Implementing Bodies:

	1	2	3	4	5	6	7	8	9	10	Don't know
ED 4.11.1 Extent to which information is shared or spread across geographical areas	○	○	○	○	○	○	○	○	○	○	○
ED. 4.11.2 Extent to which information is shared or spread throughout various levels (national, state/province, localities).	○	○	○	○	○	○	○	○	○	○	○

ED 4.12 Comments: Please use this section to provide any additional comments about the level of effort, challenges, or successes in the country around research in education. If you marked "I don't know" for any of the questions, also use this section to explain why you gave that rating

JOHNS HOPKINS
BLOOMBERG SCHOOL
of PUBLIC HEALTH

Bill & Melinda Gates Institute for Population and Reproductive Health

Demographic Dividend Effort Index Survey

Dimension 5. Civil Society Organizations (CSOs)

* ED 5.1 CSO Actor Power: Extent to which CSO actors are in strong placements within institutions or technical working groups or networks concerned with education.

	1	2	3	4	5	6	7	8	9	10	Don't know
1-Lowest -> Highest-10	○	○	○	○	○	○	○	○	○	○	○

* ED 5.2 Budget Analysis as a CSO Tool: Extent to which CSOs utilize budget analysis as a tool to build education advocacy cases.

	1	2	3	4	5	6	7	8	9	10	Don't know
1-Lowest -> Highest-10	○	○	○	○	○	○	○	○	○	○	○

* ED 5.3 Community inclusion: Extent to which CSOs are involved in efforts to explore the opportunities to improve the quality of education.

	1	2	3	4	5	6	7	8	9	10	Don't know
1-Lowest -> Highest-10	○	○	○	○	○	○	○	○	○	○	○

* ED 5.4 Community Involvement in Education: Extent to which CSOs participate in bringing education information and services to the communities where people live through activities such as supporting the capacity-building of teachers and parents and/or undertaking information and education campaigns to increase the commitment to education in families and communities.

	1	2	3	4	5	6	7	8	9	10	Don't know
1-Lowest -> Highest-10	○	○	○	○	○	○	○	○	○	○	○

* ED 5.5 Social Franchising: Extent to which CSOs are involved with social franchising opportunities/services to bring quality education services to children and families at reduced costs.

	1	2	3	4	5	6	7	8	9	10	Don't know
1-Lowest -> Highest-10	◯	◯	◯	◯	◯	◯	◯	◯	◯	◯	◯

* ED 5.6 Technology: Extent to which CSOs are leveraging information communication technology (ICT) to improve access to quality education for various communities.

	1	2	3	4	5	6	7	8	9	10	Don't know
1-Lowest -> Highest-10	◯	◯	◯	◯	◯	◯	◯	◯	◯	◯	◯

* ED 5.7 Human Rights and Access to/Quality of Education: Extent to which CSOs advocate for and support rights-based approaches to education to ensure students experience good treatment, confidence in asking questions, and quality education, amongst other concerns.

	1	2	3	4	5	6	7	8	9	10	Don't know
1-Lowest -> Highest-10	◯	◯	◯	◯	◯	◯	◯	◯	◯	◯	◯

* ED 5.8 Social and Behavior Change (SBC): Extent to which CSOs support SBC interventions relating, but not limited, to life skills and peer education, adult health education, school-based education, community mobilization, and/or mass media interventions for a general public audience.

	1	2	3	4	5	6	7	8	9	10	Don't know
1-Lowest -> Highest-10	◯	◯	◯	◯	◯	◯	◯	◯	◯	◯	◯

* ED 5.9 Advocacy/Accountability: Extent to which CSOs support the training and capacity building of advocates and community members to understand policies, processes, and activities to increase community-led issue identification, collaborative problem solving and more targeted advocacy actions to improve education.

	1	2	3	4	5	6	7	8	9	10	Don't know
1-Lowest -> Highest-10	◯	◯	◯	◯	◯	◯	◯	◯	◯	◯	◯

* ED 5.10 CSO-Led Assessment and Monitoring: Extent to which CSOs assess, monitor and report on the effectiveness of policies and programming to improve accountability in education at the policy and teachers' levels.

	1	2	3	4	5	6	7	8	9	10	Don't know
1-Lowest -> Highest-10	◯	◯	◯	◯	◯	◯	◯	◯	◯	◯	◯

* ED 5.11 Forming Partnerships between CSOs: Extent to which CSOs have formed national alliances and regional partnerships to strengthen their positioning and potentially improve leadership strength and funding mechanisms to improve the quality of education.

	1	2	3	4	5	6	7	8	9	10	Don't know
1-Lowest -> Highest-10	◯	◯	◯	◯	◯	◯	◯	◯	◯	◯	◯

ED 5.12 Comments: Please use this section to provide any additional comments about the level of effort, challenges, or successes in the country around the role of the civil society in ED. If you marked “I don’t know” for any of the questions, also use this section to explain why you gave that rating

JOHNS HOPKINS
BLOOMBERG SCHOOL
of PUBLIC HEALTH

Bill & Melinda Gates Institute for Population and Reproductive Health

Demographic Dividend Effort Index Survey

Module: Sectoral Resilience and Sustainability

Considering the health and socio-economic impact of the COVID-19 and the likely impact on the DD progress, this DD effort index has integrated questions to assess the resilience and sustainability of systems in key sectors of the DD framework. The items included in the COVID-19 related questionnaire below follow the key dimensions of resilient systems and provide critical information on the potential of an effective response to emerging infectious disease threats or other public health emergencies.

Please score each item from 1 to 10, where 1 represents the lowest score (poor state/capability) and 10 represents the highest score (great state/capability).

* Physical: facilities, equipment, system states, and capabilities.

	1	2	3	4	5	6	7	8	9	10	Don't know
ED-M1 - Plan/Prepare - State and capability of equipment, personnel and ED sector structure prior to the crisis event. (1 = poor state/capability; 10 = great state/capability)	○	○	○	○	○	○	○	○	○	○	○
ED-M2 - Absorb - Extent to which COVID-19 was recognized within the ED sector and the performance of the system was able to maintain functionality.	○	○	○	○	○	○	○	○	○	○	○
ED-M3 - Absorb - Integration: The level of integration of ED services and programs to mitigate the effect of COVID19 on ED.	○	○	○	○	○	○	○	○	○	○	○
ED-M4 - Recover - Extent to which the ED sector has been able to induce change to recover the pre-COVID-19 level of functionality.	○	○	○	○	○	○	○	○	○	○	○
ED-M5 - Recover - Financing and Donor Management: Level of resources mobilization and allocation to mitigate the effect of COVID-19 on ED.	○	○	○	○	○	○	○	○	○	○	○
ED-M6 - Adapt - Extent to which changes have been made to improve the resilience of the ED sector.	○	○	○	○	○	○	○	○	○	○	○

* Information: creation, manipulation, storage, and utilization of data.

	1	2	3	4	5	6	7	8	9	10	Don't know
ED-M7 - Plan/Prepare - Extent to which ED-related data was sufficiently prepared, presented, analyzed and stored prior to the crisis event.	○	○	○	○	○	○	○	○	○	○	○
ED-M8 - Absorb - Awareness: Extent of the surveillance capacity to detect the ED limitations/threats posed by COVID-19. (This includes data that is focused on the variety of education-related concerns that the education sector is facing at each level [primary, secondary, tertiary] of schooling).	○	○	○	○	○	○	○	○	○	○	○
ED-M9 - Recover - Extent to which ED data has been used to track the progress of recovery and to anticipate recovery scenarios.	○	○	○	○	○	○	○	○	○	○	○
ED-M10 - Adapt - Extent to which the ED system is creating and improving ED data storage and real-time use protocols.	○	○	○	○	○	○	○	○	○	○	○

* Cognitive: understanding, mental models, preconceptions, biases, and values.

	1	2	3	4	5	6	7	8	9	10	Don't know
ED-M11 - Plan/Prepare - Extent to which the ED system design and operation decisions were prepared in anticipation of crisis events.	○	○	○	○	○	○	○	○	○	○	○
ED-M12 - Absorb - Extent to which the ED system is responding with sufficient contingency protocols and proactive crisis management.	○	○	○	○	○	○	○	○	○	○	○

	1	2	3	4	5	6	7	8	9	10	Don't know
ED-M13 - Absorb - Accountability: Level of effort to ensure optimal accountability for the resources allocated to support ED as part of the response to the COVID-19.											
ED-M14 - Recover - Extent to which decisions are oriented towards recovery, which employ evidence-based communication to community members to promote safe behavior.											
ED-M15 - Recover - Awareness: Extent to which the ED sector is building confidence through communication regarding barriers posed by COVID-19 to support access to and uptake of ED services and programs.											
ED-M16 - Adapt - Extent to which the ED sector is designing new system configurations, objectives and decision criteria.											
ED-M17 - Adapt - Adaptive: Level of adaptation to respond to the COVID-19 threats to ED.											

* Social: interaction, collaboration, and self-synchronization between individuals, entities and institutions.

	1	2	3	4	5	6	7	8	9	10	Don't know
ED-M18 - Plan/Prepare - Extent to which training on outbreak/crisis management was conducted through, and which leveraged, social networks, social capital and institutional and cultural norms prior to the crisis event.											

	1	2	3	4	5	6	7	8	9	10	Don't know
ED-M19 - Absorb - Extent to which personnel and social institutions have been resourceful and accessible for the outbreak/crisis response.	◯	◯	◯	◯	◯	◯	◯	◯	◯	◯	◯
ED-M20 - Absorb - Self-Regulating: Extent to which the national leadership had the authority to make sectoral changes in a timely manner through flexible infrastructure.	◯	◯	◯	◯	◯	◯	◯	◯	◯	◯	◯
ED-M21 - Recover - Extent to which the ED system is engaging with team and knowledge sharing to enhance systematic recovery.	◯	◯	◯	◯	◯	◯	◯	◯	◯	◯	◯
ED-M22 - Recover - Diversity: The level of engagement of a multidisciplinary team to mitigate the effect of COVID-19 on ED.	◯	◯	◯	◯	◯	◯	◯	◯	◯	◯	◯
ED-M23 - Adapt - Extent to which there are additions or changes being made to ED institutions, policies, trainings, programs, and culture.	◯	◯	◯	◯	◯	◯	◯	◯	◯	◯	◯
ED-M24 - Adapt - Leadership and Management: Level of leadership demonstrated by the ED leaders to mitigate the effect of COVID-19 on ED.	◯	◯	◯	◯	◯	◯	◯	◯	◯	◯	◯

* ED M-25 Timeline: Extent of timeliness of the Government reaction - communication and implementation of measures - to mitigate the immediate and longterm impact of COVID-19.
Note: This refers to the entirety of the national reaction - not the reaction that is specific to your sector.

	1	2	3	4	5	6	7	8	9	10	Don't know
1-Lowest -> Highest-10	◯	◯	◯	◯	◯	◯	◯	◯	◯	◯	◯

ED M-26 Comments: Please use this section to provide any additional comments about the resilience and sustainability of the national education systems based on your experience during the COVID-19 pandemics. If you marked “I don’t know” for any of the questions, also use this section to explain why you gave that rating.

JOHNS HOPKINS
BLOOMBERG SCHOOL
of PUBLIC HEALTH

Bill & Melinda Gates Institute for Population and Reproductive Health

Demographic Dividend Effort Index Survey

Women's Empowerment (WE) Questionnaire

Please respond to the following questions based on your experience/expertise within this specific sector.

To give a summary picture of the national demographic dividend effort, please rate the following items. Score each item from 1 to 10, where 1 represents the lowest score (very weak or no effort) and 10 represents the highest score (strong effort). Where applicable, if a policy or activity is non-existent then mark your response as 0.

Answer each item. All responses will be recorded in the format exemplified below:

Lowest Effort	1	2	3	4	5	6	7	8	9	10	Highest Effort	I do not know

JOHNS HOPKINS
BLOOMBERG SCHOOL
of PUBLIC HEALTH

Bill & Melinda Gates Institute for Population and Reproductive Health

Demographic Dividend Effort Index Survey

Dimension 1. Policy/Policy making

* WE 1.1 Gender Equality Policy: Extent to which gender equality is considered/integrated in country development plans (or within plans in your sector).

	1	2	3	4	5	6	7	8	9	10	Don't know
1-Lowest -> Highest-10	○	○	○	○	○	○	○	○	○	○	○

* WE 1.2 Level of commitment to the Convention on the Elimination of all Forms of Discrimination Against Women (CEDAW).

	1	2	3	4	5	6	7	8	9	10	Don't know
1-Lowest -> Highest-10	○	○	○	○	○	○	○	○	○	○	○

* WE 1.3 Equity in Marriage and Family Policies: Extent to which the principle of equality between men and women is incorporated in national constitution and other legislation relating to marriage and family relations.

	1	2	3	4	5	6	7	8	9	10	Don't know
1-Lowest -> Highest-10	○	○	○	○	○	○	○	○	○	○	○

* WE 1.4 Policy on Age at Marriage: Extent to which legal age at marriage for females is set at 18 years or higher and is enforced.

	1	2	3	4	5	6	7	8	9	10	Don't know
1-Lowest -> Highest-10	○	○	○	○	○	○	○	○	○	○	○

* WE 1.5 Violence Against Women Measures: Level of regulations and measures on violence against women, including domestic violence, rape, sexual harassment and trafficking of girls and women.

	1	2	3	4	5	6	7	8	9	10	Don't know
1-Lowest -> Highest-10	○	○	○	○	○	○	○	○	○	○	○

* WE 1.6 Female Genital Cutting/Mutilation: Level of regulation and measures on female genital cutting/mutilation.

	1	2	3	4	5	6	7	8	9	10	Don't know
1-Lowest -> Highest-10	○	○	○	○	○	○	○	○	○	○	○

* WE 1.7 Women's Health Measures: Level of protocols for the protection of women's health, including sexually transmitted infections including HIV/AIDS, maternal mortality and contraception.

	1	2	3	4	5	6	7	8	9	10	Don't know
1-Lowest -> Highest-10	○	○	○	○	○	○	○	○	○	○	○

* WE 1.8 Education Measures: Extent to which there are mechanisms to promote girls' education.

	1	2	3	4	5	6	7	8	9	10	Don't know
WE 1.8.1 Level of enforcement of policies on girl's education.	○	○	○	○	○	○	○	○	○	○	○
WE 1.8.2 Extent to which there are mechanisms in place to prevent girls' school dropouts.	○	○	○	○	○	○	○	○	○	○	○

* WE 1.9 Rights Measures: Level of enforcement of policies on women's rights.

	1	2	3	4	5	6	7	8	9	10	Don't know
1-Lowest -> Highest-10	○	○	○	○	○	○	○	○	○	○	○

* WE 1.10 Equitable Work: Extent to which there are policies on equitable working conditions and remunerations between men and women.

	1	2	3	4	5	6	7	8	9	10	Don't know
1-Lowest -> Highest-10	○	○	○	○	○	○	○	○	○	○	○

* WE 1.11 Equitable Land/Asset Ownership: Extent to which there are policies to affirm equitable access to and ownership of land.

	1	2	3	4	5	6	7	8	9	10	Don't know
1-Lowest -> Highest-10	○	○	○	○	○	○	○	○	○	○	○

* WE 1.12 Conflict, Women & Peacebuilding: Level of implementation of Security UN Resolution 1325 on the impacts of conflict on women and their role in peacebuilding.

	1	2	3	4	5	6	7	8	9	10	Don't know
1-Lowest -> Highest-10	○	○	○	○	○	○	○	○	○	○	○

* WE 1.13 Gender Quotas in Politics: Existence, and level of implementation, of quotas for gender-balanced political systems.

	1	2	3	4	5	6	7	8	9	10	Don't know
WE 1.13.1 Extent to which there is gender equitable decision-making, including support for electoral quotas and gender mainstreaming in all government ministries and departments.	○	○	○	○	○	○	○	○	○	○	○
WE 1.13.2 Extent to which women participate and/or have a significant role in traditional governance systems (e.g. chiefs and village head).	○	○	○	○	○	○	○	○	○	○	○

* WE 1.14 Sustainable Development Goal 5: Level of commitment to fulfilling Sustainable Development Goal 5, to achieve gender equality and empower all women and girls.

	1	2	3	4	5	6	7	8	9	10	Don't know
1-Lowest -> Highest-10	○	○	○	○	○	○	○	○	○	○	○

* WE 1.15 Customary Law Revision: Extent to which customary laws have been reviewed and modified to align with national policies/frameworks. Or to which existing customary laws reflect efforts towards women's empowerment.

	1	2	3	4	5	6	7	8	9	10	Don't know
1-Lowest -> Highest-10	○	○	○	○	○	○	○	○	○	○	○

* WE 1.16 Costing for WE: Extent to which there is sufficient planning and allocating to cover the costs needed to meet targets set in a national gender plan or equivalent national plan.

	1	2	3	4	5	6	7	8	9	10	Don't know
1-Lowest -> Highest-10	○	○	○	○	○	○	○	○	○	○	○

* WE 1.17 Staffing for WE: Extent to which:

	1	2	3	4	5	6	7	8	9	10	Don't know
WE 1.17.1 Sufficient and qualified staff are employed to implement gender-related activities, including efforts towards training said staff	○	○	○	○	○	○	○	○	○	○	○
WE 1.17.2 Efforts are made to appropriately and comprehensively train staff	○	○	○	○	○	○	○	○	○	○	○
WE 1.17.3 Staff have sufficient support to carry out their tasks	○	○	○	○	○	○	○	○	○	○	○
WE 1.17.4 Assigned staff are located at a sufficiently high level in the administration to work effectively	○	○	○	○	○	○	○	○	○	○	○

* WE 1.18 Transparency and Accountability: Extent to which the government provides stakeholders with full information on the intentions, plans, programs and budgets available to implement programs in order to ensure accountability and transparency regarding women's empowerment programs. (*Consider measure such as the passage and implementation of a right to information law, regular information sessions for instance in town halls or the provision of such information in leaflets or other material in the local language*)

	1	2	3	4	5	6	7	8	9	10	Don't know
1-Lowest -> Highest-10	○	○	○	○	○	○	○	○	○	○	○

WE 1.19 Comments: Please use this section to provide any additional comments about the level of effort, challenges, or successes in the country around policymaking for WE. If you marked “I don’t know” for any of the questions, also use this section to explain why you gave that rating.

JOHNS HOPKINS
BLOOMBERG SCHOOL
of PUBLIC HEALTH

Bill & Melinda Gates Institute for Population and Reproductive Health

Demographic Dividend Effort Index Survey

Dimension 2. Services or Programs

* WE 2.1 Gender Mainstreaming: Extent to which services or programs within and across sectors consider implications for women and men of any planned action.

	1	2	3	4	5	6	7	8	9	10	Don't know
1-Lowest -> Highest-10	◯	◯	◯	◯	◯	◯	◯	◯	◯	◯	◯

* WE 2.2 Gender-Transformative Programming: Extent to which services or programs within and across sectors integrate gender-transformative principles/approaches.

	1	2	3	4	5	6	7	8	9	10	Don't know
1-Lowest -> Highest-10	◯	◯	◯	◯	◯	◯	◯	◯	◯	◯	◯

* WE 2.3 Education: Extent to which education services or programs focus on girl’s and women’s empowerment within the classroom.

	1	2	3	4	5	6	7	8	9	10	Don't know
1-Lowest -> Highest-10	◯	◯	◯	◯	◯	◯	◯	◯	◯	◯	◯

* WE 2.4. Dropouts: Extent to which there are programs to prevent female dropouts.

	1	2	3	4	5	6	7	8	9	10	Don't know
1-Lowest -> Highest-10	◯	◯	◯	◯	◯	◯	◯	◯	◯	◯	◯

* WE 2.5 Technology Literacy: Extent to which technology literacy is promoted amongst girls in particular.

	1	2	3	4	5	6	7	8	9	10	Don't know
1-Lowest -> Highest-10	◯	◯	◯	◯	◯	◯	◯	◯	◯	◯	◯

* WE 2.6 Employment: Extent to which job-creation programs and labor market polices aim to empower women economically and is designed/implemented accordingly.

	1	2	3	4	5	6	7	8	9	10	Don't know
1-Lowest -> Highest-10	○	○	○	○	○	○	○	○	○	○	○

* WE 2.7 Women as Mothers and Workers: Extent to which the government makes possible, through laws, regulations and programs, for women to combine the roles of childbearing, breast-feeding and child-rearing with participation in the workforce.

	1	2	3	4	5	6	7	8	9	10	Don't know
1-Lowest -> Highest-10	○	○	○	○	○	○	○	○	○	○	○

* WE 2.8 Family Planning for Empowerment: Extent to which FP services or programs are implemented with the fundamental approach towards empowering women.

	1	2	3	4	5	6	7	8	9	10	Don't know
1-Lowest -> Highest-10	○	○	○	○	○	○	○	○	○	○	○

* WE 2.9 Political and Public Representation: Extent of implementation of programs aiming at improving women's equal participation and equitable representation at all levels of the political process and public life.

	1	2	3	4	5	6	7	8	9	10	Don't know
1-Lowest -> Highest-10	○	○	○	○	○	○	○	○	○	○	○

* WE 2.10 Violence: Extent of fight against violence against women and children and its elimination nationwide (domestic violence, rape, sexual harassment, trafficking women).

	1	2	3	4	5	6	7	8	9	10	Don't know
1-Lowest -> Highest-10	○	○	○	○	○	○	○	○	○	○	○

* WE 2.11 Child Marriage: Extent of law enforcement to eliminate child marriage.

	1	2	3	4	5	6	7	8	9	10	Don't know
1-Lowest -> Highest-10	○	○	○	○	○	○	○	○	○	○	○

* WE 2.12 Female Genital Mutilation/Cutting: Extent of law enforcement to eliminate female genital mutilation/cutting.

	1	2	3	4	5	6	7	8	9	10	Don't know
1-Lowest -> Highest-10	○	○	○	○	○	○	○	○	○	○	○

WE 2.13 Comments: Please use this section to provide any additional comments about the level of effort, challenges, or successes in the country around services or programs for WE. If you marked “I don’t know” for any of the questions, also use this section to explain why you gave that rating

JOHNS HOPKINS
BLOOMBERG SCHOOL
of PUBLIC HEALTH

Bill & Melinda Gates Institute for Population and Reproductive Health

Demographic Dividend Effort Index Survey

Dimension 3. Advocacy

* WE 3.1 Advocates: Extent to which advocates for girls' and women's empowerment exists at the national and sub-national levels.

	1	2	3	4	5	6	7	8	9	10	Don't know
1-Lowest -> Highest-10	◯	◯	◯	◯	◯	◯	◯	◯	◯	◯	◯

* WE 3.2. Girls and Women: Extent to which the advocacy efforts in this area encompass the breadth of concerns in girls' and women's empowerment.

	1	2	3	4	5	6	7	8	9	10	Don't know
1-Lowest -> Highest-10	◯	◯	◯	◯	◯	◯	◯	◯	◯	◯	◯

* WE 3.3 Information Dissemination: Extent to which the government undertakes sufficient efforts to ensure that the population is aware of issues related to WE, and whether the information that is spread is correct and will reach people in a language they understand.

	1	2	3	4	5	6	7	8	9	10	Don't know
1-Lowest -> Highest-10	◯	◯	◯	◯	◯	◯	◯	◯	◯	◯	◯

* WE 3.4 Leveraging Partnerships: Extent to which the government leverages partnerships with CSOs to disseminate WE information through campaigns.

	1	2	3	4	5	6	7	8	9	10	Don't know
1-Lowest -> Highest-10	◯	◯	◯	◯	◯	◯	◯	◯	◯	◯	◯

* WE 3.5 Employment: Extent to which stakeholders call for laws, policies and programs for women to combine the roles of childbearing, breast-feeding and child-rearing with participation in the workforce.

	1	2	3	4	5	6	7	8	9	10	Don't know
1-Lowest -> Highest-10	○	○	○	○	○	○	○	○	○	○	○

* WE 3.6 Education: Extent to which a priority of advocates is to promote the fulfillment of girls' and women's potential through education and skills development.

	1	2	3	4	5	6	7	8	9	10	Don't know
1-Lowest -> Highest-10	○	○	○	○	○	○	○	○	○	○	○

* WE 3.7 Violence: Extent to which advocates successfully engage with the government to react to and address cases of violence or discrimination against women.

	1	2	3	4	5	6	7	8	9	10	Don't know
1-Lowest -> Highest-10	○	○	○	○	○	○	○	○	○	○	○

WE 3.8 Comments: Please use this section to provide any additional comments about the level of effort, challenges, or successes in the country around advocacy for WE. If you marked "I don't know" for any of the questions, also use this section to explain why you gave that rating

JOHNS HOPKINS
BLOOMBERG SCHOOL
of PUBLIC HEALTH

Bill & Melinda Gates Institute for Population and Reproductive Health

Demographic Dividend Effort Index Survey

Dimension 4. Research

* WE 4.1 Strength of Observatory/Stakeholder/Technical Working Group for WE: Extent to which country-level WE stakeholders (e.g., stakeholder leadership group, technical working group, WE observatory, country coordination and facilitation group):

	1	2	3	4	5	6	7	8	9	10	Don't know
WE 4.1.1 Have representation in government, training institutions, civil society, nongovernmental and faith-based organizations, professional associations, the private sector, etc.	○	○	○	○	○	○	○	○	○	○	○
WE 4.1.2 Meet regularly, report and recommend policy for senior management within the appropriate ministries	○	○	○	○	○	○	○	○	○	○	○
WE 4.1.3 Make an impact on WE within the country	○	○	○	○	○	○	○	○	○	○	○

* WE 4.2 Women's Rights Research Strategy: Extent to which the national WE plan and/or other national documents include a comprehensive strategy/approach on women's empowerment research, shared among partners.

	1	2	3	4	5	6	7	8	9	10	Don't know
1-Lowest -> Highest-10	○	○	○	○	○	○	○	○	○	○	○

* WE 4.3 Data Gathering Partners:

	1	2	3	4	5	6	7	8	9	10	Don't know
WE 4.3.1 Extent to which research is undertaken by government agencies. (This may include statistical agencies/bureaus and/or ministries of economics/finances, education, health, gender, etc.)	○	○	○	○	○	○	○	○	○	○	○
WE 4.3.2 Extent to which women empowerment research is undertaken by research institutions.	○	○	○	○	○	○	○	○	○	○	○
WE 4.3.3 Extent to which women empowerment research is undertaken by independent researchers.	○	○	○	○	○	○	○	○	○	○	○

* WE 4.4 Topical Data Gathering:

	1	2	3	4	5	6	7	8	9	10	Don't know
WE 4.4.1 Extent to which social and cultural norms affecting women empowerment and gender equality are researched.	○	○	○	○	○	○	○	○	○	○	○
WE 4.4.2 Extent to which health and economic effects of women empowerment are researched.	○	○	○	○	○	○	○	○	○	○	○
WE 4.4.3 Extent to which child marriage is researched to inform policy.	○	○	○	○	○	○	○	○	○	○	○
WE 4.4.4 Extent to which girls' education and skills acquisition outcomes is researched.	○	○	○	○	○	○	○	○	○	○	○

* WE 4.5 Quality/Coverage of Data:

	1	2	3	4	5	6	7	8	9	10	Don't know
WE 4.5.1 Extent to which a routine statistical system provides reliable periodic age-disaggregated data.	○	○	○	○	○	○	○	○	○	○	○
WE 4.5.2 Extent to which the routine statistical system provides good reliable periodic age-disaggregated data on women's empowerment needs among populations in different communities.	○	○	○	○	○	○	○	○	○	○	○
WE 4.5.3 Extent to which evaluations of the monitoring and surveillance systems are conducted and applied to ensure the reliability of age-disaggregated data.	○	○	○	○	○	○	○	○	○	○	○

* WE 4.6 Quality Research Institutions: Extent to which the country supports and sustains research institutions that develop research/collect data that support the breadth and quality of information-gathering to inform national WE efforts.

	1	2	3	4	5	6	7	8	9	10	Don't know
1-Lowest -> Highest-10	○	○	○	○	○	○	○	○	○	○	○

* WE 4.7 Evaluation: Extent to which program statistics, surveys, and small studies are used by specialized staff to report on program operations and measure progress on WE and gender equality.

	1	2	3	4	5	6	7	8	9	10	Don't know
1-Lowest -> Highest-10	○	○	○	○	○	○	○	○	○	○	○

* WE 4.8 Management's Use of Evaluation Findings: Extent to which local-level program managers use research and evaluation findings to improve WE programs in ways suggested by findings.

	1	2	3	4	5	6	7	8	9	10	Don't know
1-Lowest -> Highest-10	○	○	○	○	○	○	○	○	○	○	○

* WE 4.9 Ministerial Use of Evaluation Findings: Extent to which the Ministry administrators systematically utilize data to inform policies and interventions to address women's empowerment-related issues.

	1	2	3	4	5	6	7	8	9	10	Don't know
1-Lowest -> Highest-10	◯	◯	◯	◯	◯	◯	◯	◯	◯	◯	◯

* WE 4.10 Dissemination of Information to other Implementing Bodies: Extent to which information is shared or spread across geographical areas and throughout various levels (national, state/province, localities).

	1	2	3	4	5	6	7	8	9	10	Don't know
1-Lowest -> Highest-10	◯	◯	◯	◯	◯	◯	◯	◯	◯	◯	◯

WE 4.11 Comments: Please use this section to provide any additional comments about the level of effort, challenges, or successes in the country around WE research. If you marked “I don’t know” for any of the questions, also use this section to explain why you gave that rating

JOHNS HOPKINS
BLOOMBERG SCHOOL
of PUBLIC HEALTH

Bill & Melinda Gates Institute for Population and Reproductive Health

Demographic Dividend Effort Index Survey

Dimension 5. Civil Society Organizations (CSOs)

* WE 5.1 CSO Actor Power: Extent to which WE civil society actors are in strong position to influence national policy.

	1	2	3	4	5	6	7	8	9	10	Don't know
1-Lowest -> Highest-10	◯	◯	◯	◯	◯	◯	◯	◯	◯	◯	◯

* WE 5.2 Budget Analysis as a CSO Tool: Extent to which CSOs utilize budget analysis as a tool to build WE advocacy cases.

	1	2	3	4	5	6	7	8	9	10	Don't know
1-Lowest -> Highest-10	◯	◯	◯	◯	◯	◯	◯	◯	◯	◯	◯

* WE 5.3 Community-Based Service Delivery Support: Extent to which CSOs participate in bringing women's empowerment information and services to the communities where people live through activities such as supporting the capacity-building of girls, women and women's empowerment champions and/or undertaking information and education campaigns to increase the commitment to women's empowerment in families and communities.

	1	2	3	4	5	6	7	8	9	10	Don't know
1-Lowest -> Highest-10	◯	◯	◯	◯	◯	◯	◯	◯	◯	◯	◯

* WE 5.4 Human Rights and Quality of Implementation: Extent to which CSOs advocate for and support rights-based approaches to women's empowerment to ensure policy and program beneficiaries experience progress in women's empowerment and autonomy on their terms.

	1	2	3	4	5	6	7	8	9	10	Don't know
1-Lowest -> Highest-10	◯	◯	◯	◯	◯	◯	◯	◯	◯	◯	◯

* WE 5.5 Social and Behavior Change (SBC): Extent to which CSOs support SBC interventions relating, but not limited, to gender-transformative programming, life skills and peer education, adult health education, school-based education, couple's communication, community mobilization, working through gatekeepers, and/or mass media interventions for a general public audience. These may be supported through implementation at the community or individual levels.

	1	2	3	4	5	6	7	8	9	10	Don't know
1-Lowest -> Highest-10	○	○	○	○	○	○	○	○	○	○	○

* WE 5.6 Youth: Extent to which CSOs promote gender empowerment through youth programs.

	1	2	3	4	5	6	7	8	9	10	Don't know
1-Lowest -> Highest-10	○	○	○	○	○	○	○	○	○	○	○

* WE 5.7 Programming for Men: Extent to which CSOs support WE policies, interventions and programs targeting men.

	1	2	3	4	5	6	7	8	9	10	Don't know
1-Lowest -> Highest-10	○	○	○	○	○	○	○	○	○	○	○

* WE 5.8 Advocacy/Accountability: Extent to which CSOs support the training and capacity building of advocates and community members to understand policies, processes, and activities to increase community-led WE issues identification, collaborative problem solving and more targeted advocacy actions.

	1	2	3	4	5	6	7	8	9	10	Don't know
1-Lowest -> Highest-10	○	○	○	○	○	○	○	○	○	○	○

* WE 5.9 CSO-Led Assessment and Monitoring: Extent to which CSOs assess, monitor and report on the effectiveness of policies and programming to improve accountability at the policy and provider levels.

	1	2	3	4	5	6	7	8	9	10	Don't know
1-Lowest -> Highest-10	○	○	○	○	○	○	○	○	○	○	○

* WE 5.10 Forming Partnerships between CSOs: Extent to which CSOs have formed national alliances and regional partnerships to strengthen their positioning and potentially improve leadership strength and funding mechanisms to advance the women empowerment agenda.

	1	2	3	4	5	6	7	8	9	10	Don't know
1-Lowest -> Highest-10	○	○	○	○	○	○	○	○	○	○	○

WE 5.11 Comments: Please use this section to provide any additional comments about the level of effort, challenges, or successes around the role of CSOs in WE. If you marked “I don’t know” for any of the questions, also use this section to explain why you gave that rating

JOHNS HOPKINS
BLOOMBERG SCHOOL
of PUBLIC HEALTH

Bill & Melinda Gates Institute for Population and Reproductive Health

Demographic Dividend Effort Index Survey

Module: Sectoral Resilience and Sustainability

Considering the health and socio-economic impact of the COVID-19 and the likely impact on the DD progress, this DD effort index has integrated questions to assess the resilience and sustainability of systems in key sectors of the DD framework. The items included in the COVID-19 related questionnaire below follow the key dimensions of resilient systems and provide critical information on the potential of an effective response to emerging infectious disease threats or other public health emergencies.

Please score each item from 1 to 10, where 1 represents the lowest score (poor state/capability) and 10 represents the highest score (great state/capability).

* Physical: facilities, equipment, system states, and capabilities.

	1	2	3	4	5	6	7	8	9	10	Don't know
WE-M1 - Plan/Prepare - State and capability of equipment, personnel and WE sector structure prior to the crisis event. (1 = poor state/capability; 10 = great state/capability)	○	○	○	○	○	○	○	○	○	○	○
WE-M2 - Absorb - Extent to which COVID-19 was recognized within the WE sector and the performance of the system was able to maintain functionality.	○	○	○	○	○	○	○	○	○	○	○
WE-M3 - Absorb - Integration: The level of integration of WE services and programs to mitigate the effect of COVID-19 on WE.	○	○	○	○	○	○	○	○	○	○	○
WE-M4 - Recover - Extent to which the WE programs have been able to induce change to recover the pre-COVID-19 level of functionality.	○	○	○	○	○	○	○	○	○	○	○
WE-M5 - Recover - Financing and Donor Management: Level of resources mobilization and allocation to mitigate the effect of COVID-19 on WE	○	○	○	○	○	○	○	○	○	○	○
WE-M6 - Adapt - Extent to which changes have been made to improve resilience in the WE sector.	○	○	○	○	○	○	○	○	○	○	○

* Information: creation, manipulation, storage, and utilization of data.

	1	2	3	4	5	6	7	8	9	10	Don't know
WE-M7 - Plan/Prepare - Extent to which WE-related data was sufficiently prepared, presented, analyzed and stored prior to the crisis event.	○	○	○	○	○	○	○	○	○	○	○
WE-M8 - Absorb - Extent to which the WE sector has been able to conduct a real-time assessment of functionality, in anticipation of cascading losses in WE programs and services.	○	○	○	○	○	○	○	○	○	○	○
WE-M9 - Absorb - Awareness: Extent of the surveillance capacity to detect the WE limitations/threats posed by COVID-19.	○	○	○	○	○	○	○	○	○	○	○
WE-M10 - Recover - Extent to which WE data have been used to track the progress of recovery and to anticipate recovery scenarios.	○	○	○	○	○	○	○	○	○	○	○
WE-M11 - Adapt - Extent to which the WE sector is creating and improving WE data storage and real-time use protocols.	○	○	○	○	○	○	○	○	○	○	○

* Cognitive: understanding, mental models, preconceptions, biases, and values.

	1	2	3	4	5	6	7	8	9	10	Don't know
WE-M12 - Plan/Prepare - Extent to which the WE programs design and operation decisions were prepared in anticipation of crisis events.	○	○	○	○	○	○	○	○	○	○	○
WE-M13 - Absorb - Extent to which the WE sector is responding with sufficient contingency protocols and proactive crisis management.	○	○	○	○	○	○	○	○	○	○	○

	1	2	3	4	5	6	7	8	9	10	Don't know
WE-M14 - Absorb - Accountability: Level of effort to ensure optimal accountability for the resources allocated to support WE as part of the response to the COVID-19. (These may take the form of support for services attenuated to the needs of women and girls for protection).	◯	◯	◯	◯	◯	◯	◯	◯	◯	◯	◯
WE-M15 - Recover - Extent to which decisions are oriented towards recovery, which employ evidence-based communication to community members to promote safe behavior.	◯	◯	◯	◯	◯	◯	◯	◯	◯	◯	◯
WE-M16 - Recover - Awareness: Extent to which the WE sector is building confidence through communication regarding barriers posed by COVID-19 to support access to and uptake of WE services and programs. (This includes surveillance that is attenuated to the gendered disparities in COVID-19 responses and/or cases as well as increased surveillance of WE-related issues such as gender-based violence)	◯	◯	◯	◯	◯	◯	◯	◯	◯	◯	◯
WE-M17 - Adapt - Extent to which the WE sector is designing new system configurations, objectives and decision criteria.	◯	◯	◯	◯	◯	◯	◯	◯	◯	◯	◯
WE-M18 - Adapt - Adaptive: Level of adaptation to respond to the COVID-19 threats to WE.	◯	◯	◯	◯	◯	◯	◯	◯	◯	◯	◯

* Social: interaction, collaboration, and self-synchronization between individuals, entities and institutions.

	1	2	3	4	5	6	7	8	9	10	Don't know
WE-M19 - Plan/Prepare - Extent to which training on outbreak/crisis management was conducted through, and which leveraged, social networks, social capital and institutional and cultural norms prior to the crisis.											
WE-M20 - Absorb - Extent to which personnel and social institutions have been resourceful and accessible for the outbreak/crisis response.											
WE-M21 - Absorb - Self-Regulating: Extent to which the national leadership had the authority to make sectoral changes in a timely manner through flexible infrastructure.											
WE-M22 - Recover - Extent to which the WE sector is engaging with team and knowledge sharing to enhance systematic recovery.											
WE-M23 - Recover - Diversity: The level of engagement of a multidisciplinary team to mitigate the effect of COVID-19 on WE.											
WE-M24 - Adapt - Leadership and Management: Level of leadership demonstrated by the leaders in the WE sector and programs to mitigate the effect of COVID-19 on WE.											

* WE M-25 Timeline: Extent of timeliness of the Government reaction - communication and implementation of measures - to mitigate the immediate and longterm impact of COVID-19.
Note: This refers to the entirety of the national reaction - not the reaction that is specific to your sector.

	1	2	3	4	5	6	7	8	9	10	Don't know
1-Lowest -> Highest-10	○	○	○	○	○	○	○	○	○	○	○

WE M-26 Comments: Please use this section to provide any additional comments about the resilience of the national women empowerment opportunities and systems based on your experience during the COVID-19 pandemics. If you marked “I don’t know” for any of the questions, also use this section to explain why you gave that rating

JOHNS HOPKINS
BLOOMBERG SCHOOL
of PUBLIC HEALTH

Bill & Melinda Gates Institute for Population and Reproductive Health

Demographic Dividend Effort Index Survey

Labor Market (LM) Questionnaire

Please respond to the following questions based on your experience/expertise within this specific sector.

To give a summary picture of the national demographic dividend effort, please rate the following items. Score each item from 1 to 10, where 1 represents the lowest score (very weak or no effort) and 10 represents the highest score (strong effort). Where applicable, if a policy or activity is non-existent then mark your response as 0.

Answer each item. All responses will be recorded in the format exemplified below:

Lowest effort	1	2	3	4	5	6	7	8	9	10	Highest effort	I do not know

JOHNS HOPKINS
BLOOMBERG SCHOOL
of PUBLIC HEALTH

Bill & Melinda Gates Institute for Population and Reproductive Health

Demographic Dividend Effort Index Survey

Dimension 1. Policy/Policy making

* LM 1.1 Global Economic Integration: Extent to which the country aims to be integrated in the global economy.

	1	2	3	4	5	6	7	8	9	10	Don't know
LM 1.1.1 Extent to which the country legislates for trade openness.	○	○	○	○	○	○	○	○	○	○	○
LM 1.1.2 Level of effort towards growing national exports.	○	○	○	○	○	○	○	○	○	○	○

* LM 1.2 Regional Economic Integration: Extent to which the country aims to be integrated in the regional economy.

	1	2	3	4	5	6	7	8	9	10	Don't know
1-Lowest -> Highest-10	○	○	○	○	○	○	○	○	○	○	○

* LM 1.3 Technology: Extent to which the government has invested in infrastructure, both traditional and technological, to provide the basis for the country to leverage opportunities of the future and/or to integrate technology as a complement to labor.

	1	2	3	4	5	6	7	8	9	10	Don't know
1-Lowest -> Highest-10	○	○	○	○	○	○	○	○	○	○	○

* LM 1.4 Government Support: Extent to which policies and plans are in place that provide for social safety nets to smooth transitions between jobs or ‘gigs’ for individuals and buffer income volatility.

	1	2	3	4	5	6	7	8	9	10	Don't know
LM 1.4.1 Extent to which policies are in place that set a national minimum wage.	◯	◯	◯	◯	◯	◯	◯	◯	◯	◯	◯
LM 1.4.2 Extent to which policies exist that outline an unemployment insurance system, and the level to which they are effectively implemented.	◯	◯	◯	◯	◯	◯	◯	◯	◯	◯	◯

* LM 1.5 Unemployment: Extent to which policies account for and address unemployment in various population groups and geographic areas.

	1	2	3	4	5	6	7	8	9	10	Don't know
1-Lowest -> Highest-10	◯	◯	◯	◯	◯	◯	◯	◯	◯	◯	◯

* LM 1.6 Disadvantaged/Vulnerable Groups: Extent to which policies exist, and are effectively implemented, to support the employment opportunities of disadvantage/vulnerable groups (i.e. girls and women, youth, persons with disabilities, etc.).

	1	2	3	4	5	6	7	8	9	10	Don't know
1-Lowest -> Highest-10	◯	◯	◯	◯	◯	◯	◯	◯	◯	◯	◯

* LM 1.7 Climate Change and Environmental Sustainability: Extent to which the agricultural and manufacturing sectors are prepared to labor market impacts from climate change effects through planning and strategizing for the future labor market needs.

	1	2	3	4	5	6	7	8	9	10	Don't know
1-Lowest -> Highest-10	◯	◯	◯	◯	◯	◯	◯	◯	◯	◯	◯

* LM 1.8 Urbanization: Extent to which policies and planning anticipate and are prepared for increasing urbanization as a result of, and contributing to, climate change, and the particular impacts on labor markets.

	1	2	3	4	5	6	7	8	9	10	Don't know
1-Lowest -> Highest-10	◯	◯	◯	◯	◯	◯	◯	◯	◯	◯	◯

* LM 1.9 Service-Led Economy: Extent to which policies and framework are in place to move economies from informal labor to formalized labor responding to local, regional and global demands for goods and services.

	1	2	3	4	5	6	7	8	9	10	Don't know
1-Lowest -> Highest-10	◯	◯	◯	◯	◯	◯	◯	◯	◯	◯	◯

* LM 1.10 Corporate Social Responsibility: Extent to which national legislation in the labor market address corporate social responsibilities with the aim of supporting youth entrepreneurship.

	1	2	3	4	5	6	7	8	9	10	Don't know
1-Lowest -> Highest-10	◯	◯	◯	◯	◯	◯	◯	◯	◯	◯	◯

* LM 1.11 Targeted Labor Market Investments: Extent to which the government has targeted investments towards sectors with high job-multiplier effects, including Information and Communications Technology (ICT), manufacturing, agriculture and agro-industries in order to generate employment and spur inclusive growth.

	1	2	3	4	5	6	7	8	9	10	Don't know
1-Lowest -> Highest-10	◯	◯	◯	◯	◯	◯	◯	◯	◯	◯	◯

* LM 1.12 Self-Employment: Extent to which policies support raising the returns to self-employment and creating more opportunities to move from self-employment into higher paying wage employment. (This includes rural work).

	1	2	3	4	5	6	7	8	9	10	Don't know
1-Lowest -> Highest-10	◯	◯	◯	◯	◯	◯	◯	◯	◯	◯	◯

* LM 1.13 Microcredit: Extent to which the government enables access to microcredits for profitable activities, securing affordable credit for small and medium-sized enterprises and self-workers.

	1	2	3	4	5	6	7	8	9	10	Don't know
1-Lowest -> Highest-10	◯	◯	◯	◯	◯	◯	◯	◯	◯	◯	◯

* LM 1.14 Savings: Extent to which the government supports the creation of accessible financial products and policies to promote the culture of saving.

	1	2	3	4	5	6	7	8	9	10	Don't know
1-Lowest -> Highest-10	◯	◯	◯	◯	◯	◯	◯	◯	◯	◯	◯

* LM 1.15 Skill Acquisition: Extent to which policies exist, and are effectively implemented, to support the acquisition of currently and future-valued skills for the labor market.

	1	2	3	4	5	6	7	8	9	10	Don't know
1-Lowest -> Highest-10	◯	◯	◯	◯	◯	◯	◯	◯	◯	◯	◯

LM 1.16 Comments: Please use this section to provide any additional comments about the level of effort, challenges, or successes in the country around policies/policymaking for LM. If you marked “I don’t know” for any of the questions, also use this section to explain why you gave that rating

JOHNS HOPKINS
BLOOMBERG SCHOOL
of PUBLIC HEALTH

Bill & Melinda Gates Institute for Population and Reproductive Health

Demographic Dividend Effort Index Survey

Dimension 2. Services or Programs

* LM 2.1 Unemployment Reduction: Extent to which there are efforts toward developing and implementing strategies aimed at the reduction of the proportion of unemployed by target years (in accordance with Agenda 2063 First Ten Year Implementation Plan).

	1	2	3	4	5	6	7	8	9	10	Don't know
1-Lowest -> Highest-10	◯	◯	◯	◯	◯	◯	◯	◯	◯	◯	◯

* LM 2.2. Youth Unemployment: Extent to which labor market programs have effectively lowered the rate of unemployment, especially among youth.

	1	2	3	4	5	6	7	8	9	10	Don't know
1-Lowest -> Highest-10	◯	◯	◯	◯	◯	◯	◯	◯	◯	◯	◯

* LM 2.3 Access to Microcredit: Extent to which national and regional funds have been established and operationalized in order to increase people’s access to affordable business capital.

	1	2	3	4	5	6	7	8	9	10	Don't know
1-Lowest -> Highest-10	◯	◯	◯	◯	◯	◯	◯	◯	◯	◯	◯

* LM 2.4 Harnessing Youth Special Capacities: Extent to which programs and services support the specific talents of youth to aid them in flourishing and achieving their maximum potential. (This includes specific programmatic aims around talent acquisition).

	1	2	3	4	5	6	7	8	9	10	Don't know
1-Lowest -> Highest-10	◯	◯	◯	◯	◯	◯	◯	◯	◯	◯	◯

* LM 2.5 Early Labor Opportunities: Extent to which programs and government legislation engages with private sector partners to expand internships, apprenticeships and on-the-job training opportunities for women and youth.

	1	2	3	4	5	6	7	8	9	10	Don't know
1-Lowest -> Highest-10											

* LM 2.6 Youth Entrance into Labor: Extent of the promotion of youth volunteer and junior professional programs and other internship opportunities to enhance capacity and grant exposure to youth in regional and international organizations.

	1	2	3	4	5	6	7	8	9	10	Don't know
1-Lowest -> Highest-10											

* LM 2.7 Entrepreneurial Ventures: Extent to which people have enhanced access to government procurement and financial services to reduce the challenge of starting and/or doing business within and across countries.

	1	2	3	4	5	6	7	8	9	10	Don't know
1-Lowest -> Highest-10											

* LM 2.8 Resources for Youth Entrepreneurship: Extent to which entrepreneurial ventures of youth receive direct and targeted resource mobilization, supported by a regulatory framework, to reduce the challenge of starting and/or doing business within and across countries.

	1	2	3	4	5	6	7	8	9	10	Don't know
1-Lowest -> Highest-10											

* LM 2.9 Vocational Opportunities: Extent to which vocational training opportunities are expanded for skills acquisition for people to enhance their employability, productivity and competitiveness.

	1	2	3	4	5	6	7	8	9	10	Don't know
1-Lowest -> Highest-10											

* LM 2.10 Education and Labor: Extent to which improvements have been made to improve inclusive access to education at all levels and to provide viable alternatives for the many young people, particularly adolescent girls, who drop out of the formal education system, by facilitating re-entry, revamping informal education and training through standardized certification within and between countries.

	1	2	3	4	5	6	7	8	9	10	Don't know
1-Lowest -> Highest-10											

* LM 2.11 Women-Friendly Labor: Extent to which workplaces in the private and public sectors have adopted women-friendly policies and practices to eliminate any form of gender-based discrimination while allowing women to comfortably be part of the labor force.

	1	2	3	4	5	6	7	8	9	10	Don't know
1-Lowest -> Highest-10	◯	◯	◯	◯	◯	◯	◯	◯	◯	◯	◯

* LM 2.12 Job Security:

	1	2	3	4	5	6	7	8	9	10	Don't know
LM 2.12.1 Extent to which notice periods for redundancy or dismissal are implemented.	◯	◯	◯	◯	◯	◯	◯	◯	◯	◯	◯
LM 2.12.2 Extent to which severance pay for redundancy or dismissal is implemented.	◯	◯	◯	◯	◯	◯	◯	◯	◯	◯	◯

* LM 2.13 Minimum Wage: Extent to which a minimum wage is applied to the ‘typical’ worker (defined as an average wage-earner, in majority race and religious groups, in largest city, and non-union).

	1	2	3	4	5	6	7	8	9	10	Don't know
1-Lowest -> Highest-10	◯	◯	◯	◯	◯	◯	◯	◯	◯	◯	◯

* LM 2.14 Labor Market Programs: Extent to which labor market programs have effectively diversified the labor force and the needs of the labor market while simultaneously diversifying the skills of the labor force.

	1	2	3	4	5	6	7	8	9	10	Don't know
1-Lowest -> Highest-10	◯	◯	◯	◯	◯	◯	◯	◯	◯	◯	◯

* LM 2.15 Informality in the Labor Market: Extent to which the programs are implemented to transform informality in the labor market into formal labor.

	1	2	3	4	5	6	7	8	9	10	Don't know
1-Lowest -> Highest-10	◯	◯	◯	◯	◯	◯	◯	◯	◯	◯	◯

LM 2.16 Comments: Please use this section to provide any additional comments about the level of effort, challenges, or successes in the country around services or programs for LM. If you marked “I don’t know” for any of the questions, also use this section to explain why you gave that rating.

JOHNS HOPKINS
BLOOMBERG SCHOOL
of PUBLIC HEALTH

Bill & Melinda Gates Institute for Population and Reproductive Health

Demographic Dividend Effort Index Survey

Dimension 3. Advocacy

* LM 3.1 Gender Equality in the Labor Market: Extent to which groups and individuals call for, and see improvements in, gender equality in the workforce.

	1	2	3	4	5	6	7	8	9	10	Don't know
1-Lowest -> Highest-10	○	○	○	○	○	○	○	○	○	○	○

* LM 3.2 Youth Equality in the Labor Market: Extent to which individuals and groups call for shifts in the labor market and labor market regulations to benefit all rural and urban youth populations.

	1	2	3	4	5	6	7	8	9	10	Don't know
1-Lowest -> Highest-10	○	○	○	○	○	○	○	○	○	○	○

* LM 3.3 Minimum Wage Advocacy: Extent to which advocacy groups are successful in inciting legislation for a minimum wage, or a rise in the minimum wage if one already exists.

	1	2	3	4	5	6	7	8	9	10	Don't know
1-Lowest -> Highest-10	○	○	○	○	○	○	○	○	○	○	○

* LM 3.4 Government Advocacy: Extent to which members of the government advocates for improvements within its own structures and priorities. (These improvements may include labor market-focused progress for youth inclusion, opportunities, programming and rights among similar concepts across the population).

	1	2	3	4	5	6	7	8	9	10	Don't know
1-Lowest -> Highest-10	○	○	○	○	○	○	○	○	○	○	○

* LM 3.5 Protections: Extent to which advocacy exists for job protection, and social protection in the absence of jobs.

	1	2	3	4	5	6	7	8	9	10	Don't know
1-Lowest -> Highest-10	◯	◯	◯	◯	◯	◯	◯	◯	◯	◯	◯

LM 3.6 Comments: Please use this section to provide any additional comments about the level of effort, challenges, or successes in advocacy work related to LM. If you marked “I don’t know” for any of the questions, also use this section to explain why you gave that rating.

JOHNS HOPKINS
BLOOMBERG SCHOOL of PUBLIC HEALTH

Bill & Melinda Gates Institute for Population and Reproductive Health

Demographic Dividend Effort Index Survey

Dimension 4. Research

* LM 4.1 Strength of Observatory/Stakeholder/Technical Working Group for the LM: Extent to which country-level LM stakeholders (e.g., stakeholder leadership group, technical working group, LM observatory, country coordination and facilitation group):

	1	2	3	4	5	6	7	8	9	10	Don't know
LM 4.1.1 Have representation in government, training institutions, civil society, nongovernmental and faith-based organizations, professional associations, the private sector, etc.	○	○	○	○	○	○	○	○	○	○	○
LM 4.1.2 Meet regularly, report and recommend policy for senior management within the appropriate ministries	○	○	○	○	○	○	○	○	○	○	○
LM 4.1.3 Make an impact on LM within the country	○	○	○	○	○	○	○	○	○	○	○

* LM 4.2 Labor Market Research Strategy: Extent to which the national documents include a comprehensive strategy/approach on labor market-related research and is shared among partners.

	1	2	3	4	5	6	7	8	9	10	Don't know
1-Lowest -> Highest-10	○	○	○	○	○	○	○	○	○	○	○

* LM 4.3 Labor Market Analysis Partners:

	1	2	3	4	5	6	7	8	9	10	Don't know
LM 4.3.1 Extent to which labor market research is undertaken by government agencies.											
LM 4.3.2 Extent to which labor market research is undertaken by research institutions.											
LM 4.3.3 Extent to which labor market research is undertaken by independent researchers.											

* LM 4.4 Areas of Research in the Labor Market:

	1	2	3	4	5	6	7	8	9	10	Don't know
LM 4.4.1 Extent to which unemployment trends and patterns are researched.											
LM 4.4.2 Extent of research on youth in the labor market.											
LM 4.4.3 Extent of research on women in the labor market.											
LM 4.4.4 Extent of research on the trends and trajectories of the labor market needs.											

* LM 4.5 Quality/Coverage of Data:

	1	2	3	4	5	6	7	8	9	10	Don't know
LM 4.5.1 Extent to which a routine statistical system provides reliable periodic labor market data.	○	○	○	○	○	○	○	○	○	○	○
LM 4.5.2 Extent to which the routine statistical system provides reliable periodic information on LM needs among populations in different communities.	○	○	○	○	○	○	○	○	○	○	○
LM 4.5.3 Extent to which evaluations of the monitoring and surveillance systems are conducted and applied to ensure the reliability of data.	○	○	○	○	○	○	○	○	○	○	○

* LM 4.6 Record-Keeping: Extent to which systems for labor market recordkeeping, reporting and feedback of results are adequate.

	1	2	3	4	5	6	7	8	9	10	Don't know
1-Lowest -> Highest-10	○	○	○	○	○	○	○	○	○	○	○

* LM 4.7 Quality Research Institutions: Extent to which the country has the capacity to sustain and maintain research institutions that develop research/collect labor market-related data.

	1	2	3	4	5	6	7	8	9	10	Don't know
1-Lowest -> Highest-10	○	○	○	○	○	○	○	○	○	○	○

* LM 4.8 Evaluation: Extent to which program statistics, surveys, and small studies are used by specialized staff to report on program operations and measure progress.

	1	2	3	4	5	6	7	8	9	10	Don't know
1-Lowest -> Highest-10	○	○	○	○	○	○	○	○	○	○	○

* LM 4.9 Management's Use of Evaluation Findings: Extent to which local-level program managers use research and evaluation findings to improve LM related services and programs in ways suggested by findings.

	1	2	3	4	5	6	7	8	9	10	Don't know
1-Lowest -> Highest-10	○	○	○	○	○	○	○	○	○	○	○

* LM 4.10 Ministerial Use of Evaluation Findings: Extent to which the Ministry administrators systematically utilize data to inform policies and interventions to address LM-related issues.

	1	2	3	4	5	6	7	8	9	10	Don't know
1-Lowest -> Highest-10	◯	◯	◯	◯	◯	◯	◯	◯	◯	◯	◯

* LM 4.11 Dissemination of Information to Other Implementing Bodies: Extent to which information is shared or spread across geographical areas and throughout various levels (national, state/province, localities).

	1	2	3	4	5	6	7	8	9	10	Don't know
1-Lowest -> Highest-10	◯	◯	◯	◯	◯	◯	◯	◯	◯	◯	◯

LM 4.12 Comments: Please use this section to provide any additional comments about the level of effort, challenges, or successes in the country around LM research. If you marked “I don’t know” for any of the questions, also use this section to explain why you gave that rating.

JOHNS HOPKINS
BLOOMBERG SCHOOL
of PUBLIC HEALTH

Bill & Melinda Gates Institute for Population and Reproductive Health

Demographic Dividend Effort Index Survey

Dimension 5. Civil Society Organizations (CSOs)

* LM 5.1 CSO Actor Power: Extent to which CSO actors are in strong positions to influence policies and action on the LM.

	1	2	3	4	5	6	7	8	9	10	Don't know
1-Lowest -> Highest-10	◯	◯	◯	◯	◯	◯	◯	◯	◯	◯	◯

* LM 5.2 Budget Analysis as a CSO Tool: Extent to which CSOs utilize budget analysis as a tool to build an advocacy case for labor market.

	1	2	3	4	5	6	7	8	9	10	Don't know
1-Lowest -> Highest-10	◯	◯	◯	◯	◯	◯	◯	◯	◯	◯	◯

* LM 5.3 Community-Based Service Delivery Support: Extent to which CSOs participate in bringing LM needs, information and services to the policy makers and vice-versa.

	1	2	3	4	5	6	7	8	9	10	Don't know
1-Lowest -> Highest-10	◯	◯	◯	◯	◯	◯	◯	◯	◯	◯	◯

* LM 5.4 Diversification of Job Opportunities: Extent to which CSOs and the private sector coordinate with the government to ensure optimal job creation and equal participation of women and men.

	1	2	3	4	5	6	7	8	9	10	Don't know
1-Lowest -> Highest-10	◯	◯	◯	◯	◯	◯	◯	◯	◯	◯	◯

* LM 5.5 Human Rights and Quality of Implementation: Extent to which CSOs support rights-based approaches at workplaces.

	1	2	3	4	5	6	7	8	9	10	Don't know
1-Lowest -> Highest-10	◯	◯	◯	◯	◯	◯	◯	◯	◯	◯	◯

* LM 5.6 Youth: Extent to which CSOs support labor market policies, interventions and programming specifically targeting youth.

	1	2	3	4	5	6	7	8	9	10	Don't know
1-Lowest -> Highest-10	◯	◯	◯	◯	◯	◯	◯	◯	◯	◯	◯

* LM 5.7 Programming for Women: Extent to which CSOs support labor market policies, services and programs for women.

	1	2	3	4	5	6	7	8	9	10	Don't know
1-Lowest -> Highest-10	◯	◯	◯	◯	◯	◯	◯	◯	◯	◯	◯

* LM 5.8 Advocacy/Accountability: Extent to which CSOs support the training and capacity building of advocates and community members to understand policies, processes, and activities to increase community-led issue identification, collaborative problem solving and more targeted advocacy actions to address unemployment.

	1	2	3	4	5	6	7	8	9	10	Don't know
1-Lowest -> Highest-10	◯	◯	◯	◯	◯	◯	◯	◯	◯	◯	◯

* LM 5.9 CSO-Led Assessment and Monitoring: Extent to which CSOs assess, monitor and report on the effectiveness of policies and programming to improve accountability at the policy levels.

	1	2	3	4	5	6	7	8	9	10	Don't know
1-Lowest -> Highest-10	◯	◯	◯	◯	◯	◯	◯	◯	◯	◯	◯

* LM 5.10 Forming Partnerships between CSOs: Extent to which the private sector has formed national alliances and regional partnerships to strengthen the labor market.

	1	2	3	4	5	6	7	8	9	10	Don't know
1-Lowest -> Highest-10	◯	◯	◯	◯	◯	◯	◯	◯	◯	◯	◯

LM 5.11 Comments: Please use this section to provide any additional comments about the level of effort, challenges, or successes on the role of the CSO in the LM. If you marked “I don’t know” for any of the questions, also use this section to explain why you gave that rating

JOHNS HOPKINS
BLOOMBERG SCHOOL
of PUBLIC HEALTH

Bill & Melinda Gates Institute for Population and Reproductive Health

Demographic Dividend Effort Index Survey

Module: Sectoral Resilience and Sustainability

Considering the health and socio-economic impact of the COVID-19 and the likely impact on the DD progress, this DD effort index has integrated questions to assess the resilience and sustainability of systems in key sectors of the DD framework. The items included in the COVID-19 related questionnaire below follow the key dimensions of resilient systems and provide critical information on the potential of an effective response to emerging infectious disease threats or other public health emergencies.

Please score each item from 1 to 10, where 1 represents the lowest score (poor state/capability) and 10 represents the highest score (great state/capability).

* Physical: facilities, equipment, system states, and capabilities.

	1	2	3	4	5	6	7	8	9	10	Don't know
LM-M1 - Plan/Prepare - State and capability of equipment, personnel and LM sector structure prior to the crisis event. (1 = poor state/capability; 10 = great state/capability)	○	○	○	○	○	○	○	○	○	○	○
LM-M2 - Absorb - Extent to which COVID-19 was recognized within the LM sector and the performance of the system was able to maintain optimal employment and economic productivity.	○	○	○	○	○	○	○	○	○	○	○
LM-M3 - Recover - Extent to which the LM sector has been able to induce change to recover the pre-COVID-19 level of functionality.	○	○	○	○	○	○	○	○	○	○	○

	1	2	3	4	5	6	7	8	9	10	Don't know
LM-M4 - Recover - Financing and Donor Management: Extent to which there are established social protection mechanisms (and other forms of income smoothing) for unprotected workers (e.g. self-employed workers, that have no access to paid or sick leave).	○	○	○	○	○	○	○	○	○	○	○
LM-M5 - Adapt - Financing and Donor Management: Extent to which there has been institutional and policy reforms to build resilience by a demand-led approach through large-scale social protection systems that can act as economic stabilizers	○	○	○	○	○	○	○	○	○	○	○

* Information: creation, manipulation, storage, and utilization of data.

	1	2	3	4	5	6	7	8	9	10	Don't know
LM-M6 - Plan/Prepare - Extent to which LM-related data was sufficiently prepared, presented, analyzed and stored prior to the crisis event.	○	○	○	○	○	○	○	○	○	○	○
LM-M7 - Absorb - Extent to which the LM sector has been able to conduct a real-time assessment of functionality, in anticipation of cascading losses in LM and productivity.	○	○	○	○	○	○	○	○	○	○	○
LM-M8 - Recover - Extent to which LM data has been used to track the progress of recovery and to anticipate recovery scenarios.	○	○	○	○	○	○	○	○	○	○	○
LM-M9 - Adapt - Extent to which the LM sector is creating and improving LM data storage and real-time use protocols.	○	○	○	○	○	○	○	○	○	○	○

* Cognitive: understanding, mental models, preconceptions, biases, and values.

	1	2	3	4	5	6	7	8	9	10	Don't know
LM-M10 - Plan/Prepare - Extent to which the LM sector design and operation decisions were prepared in anticipation of crisis events.	○	○	○	○	○	○	○	○	○	○	○
LM-M11 - Absorb - Extent to which the LM sector is responding with sufficient contingency protocols and proactive crisis management.	○	○	○	○	○	○	○	○	○	○	○

	1	2	3	4	5	6	7	8	9	10	Don't know
LM-M12 - Absorb - Leadership and Management: Extent to which the government is ensuring workers and employers and their families are protected from the health risks of COVID-19.	○	○	○	○	○	○	○	○	○	○	○
LM-M13 - Absorb - Accountability: Level of effort to ensure optimal accountability for the resources allocated to ensure protection from the health risks of COVID-19 and the recovery of the labor market as part of the response to mitigate COVID-19 impacts.	○	○	○	○	○	○	○	○	○	○	○
LM-M14 - Recover - Extent to which decisions are oriented towards recovery, which employ evidence-based communication to community members to promote safe behavior.	○	○	○	○	○	○	○	○	○	○	○
LM-M15 - Recover - Awareness: Extent to which the government is building confidence through trust and dialogue for building the commitment of employers and workers to the joint action of the government and promoting enterprise-level social dialogue.	○	○	○	○	○	○	○	○	○	○	○
LM-M16 - Adapt - Extent to which the LM sector is designing new system configurations, objectives and decision criteria.	○	○	○	○	○	○	○	○	○	○	○

	1	2	3	4	5	6	7	8	9	10	Don't know
LM-M17 - Adapt - Adaptive: Extent to which the government is promoting change in the workspace to protect workers (e.g. occupational and safety health measures, adapted work arrangements, prevention of discrimination and exclusion, expanded access to paid leave, etc.).	○	○	○	○	○	○	○	○	○	○	○

* Social: interaction, collaboration, and self-synchronization between individuals, entities and institutions.

	1	2	3	4	5	6	7	8	9	10	Don't know
LM-M18 - Plan/Prepare - Extent to which training on outbreak/crisis management was conducted through, and which leveraged, social networks, social capital and institutional and cultural norms prior to the crisis event.	○	○	○	○	○	○	○	○	○	○	○
LM-M19 - Absorb - Extent to which personnel and social institutions have been resourceful and accessible for the outbreak/crisis response.	○	○	○	○	○	○	○	○	○	○	○
LM-M20 - Absorb - Self-Regulating: Extent to which the national leadership had the authority to make sectoral changes in a timely manner through flexible labor market infrastructure.	○	○	○	○	○	○	○	○	○	○	○
LM-M21 - Recover - Extent to which the LM sector is engaging with team and knowledge sharing to enhance systematic recovery.	○	○	○	○	○	○	○	○	○	○	○

	1	2	3	4	5	6	7	8	9	10	Don't know
LM-M22 - Recover - Diversity: Extent to which the government is facilitating a strong and fast recovery of the labor market by diversifying policies that favor both the supply (production of good and services) and demand (consumption and investment).	◯	◯	◯	◯	◯	◯	◯	◯	◯	◯	◯
LM-M23 - Adapt - Extent to which there are additions or changes being made to LM institutions, policies, trainings, programs and culture.	◯	◯	◯	◯	◯	◯	◯	◯	◯	◯	◯
LM-M24 - Adapt - Leadership and Management: Extent to which there has been established a tripartite fluent dialogue between government, workers, and employers' organizations.	◯	◯	◯	◯	◯	◯	◯	◯	◯	◯	◯

* LM M-25 Timeline: Extent of timeliness of the Government reaction - communication and implementation of measures - to mitigate the immediate and longterm impact of COVID-19.
Note: This refers to the entirety of the national reaction - not the reaction that is specific to your sector.

	1	2	3	4	5	6	7	8	9	10	Don't know
1-Lowest -> Highest-10	◯	◯	◯	◯	◯	◯	◯	◯	◯	◯	◯

LM M-26 Comments: Please use this section to provide any additional comments about the resilience and sustainability of the LM systems based on your experience during the COVID-19 pandemics. If you marked “I don’t know” for any of the questions, also use this section to explain why you gave that rating.

JOHNS HOPKINS
BLOOMBERG SCHOOL
of PUBLIC HEALTH

Bill & Melinda Gates Institute for Population and Reproductive Health

Demographic Dividend Effort Index Survey

Governance and Economic Institutions (GEI) Questionnaire

Please respond to the following questions based on your experience/expertise within this specific sector.

To give a summary picture of the national demographic dividend effort, please rate the following items. Score each item from 1 to 10, where 1 represents the lowest score (very weak or no effort) and 10 represents the highest score (strong effort). Where applicable, if a policy or activity is non-existent then mark your response as 0.

Answer each item. All responses will be recorded in the format exemplified below:

Lowest effort	1	2	3	4	5	6	7	8	9	10	Highest effort	I do not know

JOHNS HOPKINS
BLOOMBERG SCHOOL
of PUBLIC HEALTH

Bill & Melinda Gates Institute for Population and Reproductive Health

Demographic Dividend Effort Index Survey

Dimension 1. Policies

* GEI 1.1 Democratic Values: Extent to which the government has ratified, domesticated and implemented regional instruments for shared values, including those relating to democracy, elections and governance and the right to health.

	1	2	3	4	5	6	7	8	9	10	Don't know
1-Lowest -> Highest-10											

* GEI 1.2 Monitoring Democratic Values: Extent to which the government has created mechanisms for regular and periodic reporting on the implementation of the regional instruments on shared values, through existing mechanisms such as universal periodic review on the status of international or regional instruments or using independent bodies. (This may take the form of regular reports on the status of implementation).

	1	2	3	4	5	6	7	8	9	10	Don't know
1-Lowest -> Highest-10											

* GEI 1.3 Affirmation of Youth Democratic Participation: Extent to which the government has explicitly removed all discriminatory laws and limitations to full participation of young people in electoral processes through inclusive electoral laws and constitutions. (This may include developing and/or strengthening youth leadership programs and/or providing capacity building on management and leadership skills).

	1	2	3	4	5	6	7	8	9	10	Don't know
1-Lowest -> Highest-10											

* GEI 1.4. Removing Legal Youth Barriers: Extent to which the government has proscribed all laws posing barriers to the full exercise and enjoyment of the fundamental rights of young people to fully participate in the democratic governance processes at the continental, regional, national and grassroots levels. (This may also include sustaining the mainstreaming of adolescents and youth health and rights issues into the country's growth and transformation agenda).

	1	2	3	4	5	6	7	8	9	10	Don't know
1-Lowest -> Highest-10	○	○	○	○	○	○	○	○	○	○	○

* GEI 1.5 Youth Policy: Extent to which the youth policy takes into account the latest thinking in youth education, training and employment.

	1	2	3	4	5	6	7	8	9	10	Don't know
1-Lowest -> Highest-10	○	○	○	○	○	○	○	○	○	○	○

* GEI 1.6 Affirmation of Democratic Participation/Revision of Customary Law: Extent to which the government has reviewed, revised, amended or abolished all laws, regulations, policies, practices and customs that have a discriminatory impact on youth, especially girls and young women, without distinction of any kind, and ensure that the provisions of multiple legal systems comply with international human rights regulations and laws. (These must include protection from harmful practices like early, forced or child marriages, sexual and gender-based violence, female genital mutilation (FGM).

	1	2	3	4	5	6	7	8	9	10	Don't know
1-Lowest -> Highest-10	○	○	○	○	○	○	○	○	○	○	○

* GEI 1.7 Youth Policymaking Participation: Extent to which the government ensures the involvement of youth leadership and participation in decision-making processes in efforts relating to policymaking including youth and education policy making. Potentially includes efforts to establish a national youth parliament and assembly. (This may include supporting adolescent and youth participation and leadership in health planning and programming at all levels).

	1	2	3	4	5	6	7	8	9	10	Don't know
1-Lowest -> Highest-10	○	○	○	○	○	○	○	○	○	○	○

* GEI 1.8 Rule of Law: Extent to which the rule of law is formally, legally established to be equitable and implemented independently by the judiciary.

	1	2	3	4	5	6	7	8	9	10	Don't know
1-Lowest -> Highest-10	○	○	○	○	○	○	○	○	○	○	○

* GEI 1.9 Transparency and Accountability: Extent to which national legislation mandates transparency and accountability across all branches and throughout all sectors.

	1	2	3	4	5	6	7	8	9	10	Don't know
GEI 1.9.1 Extent to which the public can access public and legislative information as well as the records of state-owned organizations.	○	○	○	○	○	○	○	○	○	○	○
GEI 1.9.2 Extent to which there are mandated actions and mechanisms for accountability and sanctioning.	○	○	○	○	○	○	○	○	○	○	○

* GEI 1.10 National Security: Extent to which the country/government is involved in armed conflict, either internationally or domestically.

	1	2	3	4	5	6	7	8	9	10	Don't know
GEI 1.10.1 Extent to which the country has cross-border tensions.	○	○	○	○	○	○	○	○	○	○	○
GEI 1.10.2 Extent to which the country contains internally displaced persons and/or refugees.	○	○	○	○	○	○	○	○	○	○	○
GEI 1.10.3 Extent to which education facilities are protected and alternative learning opportunities are created for children and youth in time of conflict or displacement.	○	○	○	○	○	○	○	○	○	○	○

* GEI 1.11 Rights: Extent to which the constitution affirms certain rights: freedom of expression, freedom of association and assembly, civil rights and liberties, and protection against ethnic and religious discrimination, health as well as an un-likelihood of human rights abuses by the government.

	1	2	3	4	5	6	7	8	9	10	Don't know
1-Lowest -> Highest-10	○	○	○	○	○	○	○	○	○	○	○

* GEI 1.12 Education and Democratic Values: Extent to which education policies, strategies and programs promote academic freedom, democratic values and education as human right.

	1	2	3	4	5	6	7	8	9	10	Don't know
1-Lowest -> Highest-10	◯	◯	◯	◯	◯	◯	◯	◯	◯	◯	◯

* GEI 1.13 Affirmation of Safe Learning Environments: Extent to which education policies provide for safe learning environments through protections for children. (This may come in the form of ensuring the safety of girls, banning corporal punishment, etc.).

	1	2	3	4	5	6	7	8	9	10	Don't know
1-Lowest -> Highest-10	◯	◯	◯	◯	◯	◯	◯	◯	◯	◯	◯

GEI 1.14 Comments: Please use this section to provide any additional comments about the level of effort, challenges, or successes in the country around policies/policymaking for GEI. If you marked “I don’t know” for any of the questions, also use this section to explain why you gave that rating

JOHNS HOPKINS
BLOOMBERG SCHOOL
of PUBLIC HEALTH

Bill & Melinda Gates Institute for Population and Reproductive Health

Demographic Dividend Effort Index Survey

Dimension 2. Services or Programs

* GEI 2.1 Strengthening Youth Activities/Institutions: Extent to which efforts are being made to strengthen independent youth formations, networks and organizations including the establishment of independent youth commissions, student unions at the national and sub-regional levels to champion youth rights.

	1	2	3	4	5	6	7	8	9	10	Don't know
1-Lowest -> Highest-10	○	○	○	○	○	○	○	○	○	○	○

* GEI 2.2 Appointment of Youth Leader: Whether and to what extent there has been a youth envoy appointed to lead advocacy and champion the prioritization of youth issues (e.g. education, training and employment) within decision-making spaces at a variety of levels including the parliament.

	1	2	3	4	5	6	7	8	9	10	Don't know
1-Lowest -> Highest-10	○	○	○	○	○	○	○	○	○	○	○

* GEI 2.3 Youth Ideals and Aspirations: Extent to which programming is designed to institute youth leadership and empowerment trainings aimed at deepening cultural ideals and aspirations in the youth.

	1	2	3	4	5	6	7	8	9	10	Don't know
1-Lowest -> Highest-10	○	○	○	○	○	○	○	○	○	○	○

* GEI 2.4 Youth Services: Extent to which programming is designed to address youth-specific needs. (This should consider the development and implementation of adolescent and youth-friendly health services, among other similar programmatic endeavors).

	1	2	3	4	5	6	7	8	9	10	Don't know
1-Lowest -> Highest-10	○	○	○	○	○	○	○	○	○	○	○

* GEI 2.5 Youth Involvement in National Initiatives: Extent to which there are efforts to strengthen existing youth engagement and participation initiatives of the country *(e.g. at the level of the African Union these may include the African Youth Volunteers Corps, African Union Youth Clubs, AGA-YES activities including the Youth Advisory Panel, the Moot Court and Annual Youth Dialogue on Democracy, Human Rights and Governance*). (This may include engaging adolescents and youth in the provision of technical support to sector ministries and relevant federal and regional government bodies for developing policies, plans, and programs that integrate responses to priority health issues affecting this group).

	1	2	3	4	5	6	7	8	9	10	Don't know
1-Lowest -> Highest-10	◯	◯	◯	◯	◯	◯	◯	◯	◯	◯	◯

* GEI 2.6 Personal Safety: Extent to which individual safety is provided for through equitable, reliable police services/activities, contributing to an overall positive perception of personal safety at the household and community levels as well as the school environment. (This may include protecting youth form any form of abuse, neglect or disrespect in the use of health services).

	1	2	3	4	5	6	7	8	9	10	Don't know
1-Lowest -> Highest-10	◯	◯	◯	◯	◯	◯	◯	◯	◯	◯	◯

* GEI 2.7 Participation: Extent to which government functions involve the participation of citizens, civil society organizations, and other intersectoral actors to promote democratic elections and the effective power to govern.

	1	2	3	4	5	6	7	8	9	10	Don't know
1-Lowest -> Highest-10	◯	◯	◯	◯	◯	◯	◯	◯	◯	◯	◯

* GEI 2.8 Gender: Extent to which women participate in:

	1	2	3	4	5	6	7	8	9	10	Don't know
GEI 2.8.1 Governance at the national level	◯	◯	◯	◯	◯	◯	◯	◯	◯	◯	◯
GEI 2.8.2 Governance at the sub-national level	◯	◯	◯	◯	◯	◯	◯	◯	◯	◯	◯
GEI 2.8.3 Governance in rural areas	◯	◯	◯	◯	◯	◯	◯	◯	◯	◯	◯
GEI 2.8.4 Governance in urban areas	◯	◯	◯	◯	◯	◯	◯	◯	◯	◯	◯
GEI 2.8.5 Judiciary	◯	◯	◯	◯	◯	◯	◯	◯	◯	◯	◯

* GEI 2.9 Promotion of Equality: Extent to which gender equality in all aspects of society is promoted by the government. (This may include equality in education, equality before law, equality in healthcare, equality in employment and renumeration, among many other facets of society).

	1	2	3	4	5	6	7	8	9	10	Don't know
1-Lowest -> Highest-10	○	○	○	○	○	○	○	○	○	○	○

* GEI 2.10 Sustainable Economic Opportunity:

	1	2	3	4	5	6	7	8	9	10	Don't know
GEI 2.10.1 Extent to which the government provides for and ensures that a strong public management system is realized.	○	○	○	○	○	○	○	○	○	○	○
GEI 2.10.2 Extent to which the government provides for and ensures that a strong business environment is realized.	○	○	○	○	○	○	○	○	○	○	○
GEI 2.10.3 Extent to which the government provides for and ensures that a strong public development infrastructure is realized.	○	○	○	○	○	○	○	○	○	○	○
GEI 2.10.4 Extent to which the government provides for and ensures that a strong rural sector for development is realized.	○	○	○	○	○	○	○	○	○	○	○

* GEI 2.11 Human Development:

	1	2	3	4	5	6	7	8	9	10	Don't know
GEI 2.11.1 Extent to which the welfare of the general public is well-handled by the government (consider aspects related, but not limited, to welfare policies and services, socioeconomic integration of youth and social protections and safety nets, among many other areas). This may also that the government, with the obligations to protect, fulfil and respect basic health rights, has given the responsibility of its implementation to its ministerial cabinet.											
GEI 2.11.2 Extent to which the government is promoting the skills development of the population at workplaces.											
GEI 2.11.3 Extent to which the government is promoting the education, knowledge and capacities of the population through schools and continuing education opportunities.											
GEI 2.11.4 Extent to which the government is promoting the health and wellness of the population.											

GEI 2.12 Comments: Please use this section to provide any additional comments about the level of effort, challenges, or successes in the country around services or programs for GEI. If you marked “I don’t know” for any of the questions, also use this section to explain why you gave that rating.

JOHNS HOPKINS
BLOOMBERG SCHOOL
of PUBLIC HEALTH

Bill & Melinda Gates Institute for Population and Reproductive Health

Demographic Dividend Effort Index Survey

Dimension 3. Advocacy

* GEI 3.1 Youth Empowerment: Extent to which youth empowerment has been enhanced, potentially through the integration of civic education in national educational curricula, media platforms and other channels to instill principles of the rule of law, human rights and individual duties and responsibilities. (This may include the mainstreaming of adolescents and youth health and rights issues into the country's growth and transformation agenda and educational curricula).

	1	2	3	4	5	6	7	8	9	10	Don't know
1-Lowest -> Highest-10	○	○	○	○	○	○	○	○	○	○	○

* GEI 3.2 Government Advocacy: Extent to which members of the government advocates for improvements within its own structures and priorities. (These improvements may include progress for youth inclusion, opportunities, programming and rights among similar concepts across the population).

	1	2	3	4	5	6	7	8	9	10	Don't know
1-Lowest -> Highest-10	○	○	○	○	○	○	○	○	○	○	○

* GEI 3.3 Non-governmental Advocacy: Extent to which non-governmental actors advocates for improvements in government structures and priorities. (These improvements may include progress for youth inclusion, opportunities, programming and rights among similar concepts across the population).

	1	2	3	4	5	6	7	8	9	10	Don't know
1-Lowest -> Highest-10	○	○	○	○	○	○	○	○	○	○	○

* GEI 3.4 Civil Society Organizations (CSOs): Extent to which CSOs specifically operate as instruments for advocacy in the GEI sector.

	1	2	3	4	5	6	7	8	9	10	Don't know
1-Lowest -> Highest-10	○	○	○	○	○	○	○	○	○	○	○

* GEI 3.5 Advocacy Voice: Extent to which advocates have a voice in government discussions.

	1	2	3	4	5	6	7	8	9	10	Don't know
1-Lowest -> Highest-10	◯	◯	◯	◯	◯	◯	◯	◯	◯	◯	◯

* GEI 3.6 Voice of Instrumental Sectors of the DD: Extent to which the government and stakeholders are committed to establish sustainable institutions to ensure:

	1	2	3	4	5	6	7	8	9	10	Don't know
GEI 3.6.1 Good governance	◯	◯	◯	◯	◯	◯	◯	◯	◯	◯	◯
GEI 3.6.2 Financial stability	◯	◯	◯	◯	◯	◯	◯	◯	◯	◯	◯
GEI 3.6.3 Optimal family planning programs	◯	◯	◯	◯	◯	◯	◯	◯	◯	◯	◯
GEI 3.6.4 Optimal maternal and child health	◯	◯	◯	◯	◯	◯	◯	◯	◯	◯	◯
GEI 3.6.5 Good quality education	◯	◯	◯	◯	◯	◯	◯	◯	◯	◯	◯
GEI 3.6.6 Diversified labor market.	◯	◯	◯	◯	◯	◯	◯	◯	◯	◯	◯

GEI 3.7 Comments: Please use this section to provide any additional comments about the level of effort, challenges, or successes in the country around advocacy for GEI. If you marked “I don’t know” for any of the questions, also use this section to explain why you gave that rating

JOHNS HOPKINS
BLOOMBERG SCHOOL
of PUBLIC HEALTH

Bill & Melinda Gates Institute for Population and Reproductive Health

Demographic Dividend Effort Index Survey

Dimension 4. Research

* GEI 4.1 Strength of Observatory/Stakeholder/Technical Working Group for GEI: Extent to which country-level GEI stakeholders (e.g., stakeholder leadership group, technical working group, GEI observatory, country coordination and facilitation group):

	1	2	3	4	5	6	7	8	9	10	Don't know
GEI 4.1.1 Have representation in government, training institutions, civil society, nongovernmental and faith-based organizations, professional associations, the private sector, etc.	○	○	○	○	○	○	○	○	○	○	○
GEI 4.1.2 Meet regularly, report and recommend policy for senior management within the appropriate ministries	○	○	○	○	○	○	○	○	○	○	○
GEI 4.1.3 Make an impact on GEI within the country	○	○	○	○	○	○	○	○	○	○	○

* GEI 4.2 Education/Research Strategy: Extent to which national planning documents include a comprehensive strategy/approach on improving GEI, shared among partners.

	1	2	3	4	5	6	7	8	9	10	Don't know
1-Lowest -> Highest-10	○	○	○	○	○	○	○	○	○	○	○

* GEI 4.3 Governance, Financial and Development Data Gathering Partners:

	1	2	3	4	5	6	7	8	9	10	Don't know
GEI 4.3.1 Extent to which research is undertaken by government agencies. (This may include statistical agencies/bureaus and/or ministries of economics/finances, education, health, gender, etc.)	○	○	○	○	○	○	○	○	○	○	○
GEI 4.3.2 Extent to which research is undertaken by public research institutions.	○	○	○	○	○	○	○	○	○	○	○
GEI 4.3.3 Extent to which research is undertaken by independent researchers.	○	○	○	○	○	○	○	○	○	○	○

* GEI 4.4 Governance, Financial and Development Topical Data Gathering:

	1	2	3	4	5	6	7	8	9	10	Don't know
GEI 4.4.1 Extent to which safety and the rule of law is researched.	○	○	○	○	○	○	○	○	○	○	○
GEI 4.4.2 Extent to which political participation and human rights is measured.	○	○	○	○	○	○	○	○	○	○	○
GEI 4.4.3 Extent to which indicators of a sustainable economy are researched and disseminated to the community.	○	○	○	○	○	○	○	○	○	○	○
GEI 4.4.4 Extent to which human capital development (education, training, and employment, etc.) is researched.	○	○	○	○	○	○	○	○	○	○	○

* GEI 4.5 Quality/Coverage of Data:

	1	2	3	4	5	6	7	8	9	10	Don't know
GEI 4.5.1 Extent to which a routine statistical system provides reliable periodic information on governance indicators.	○	○	○	○	○	○	○	○	○	○	○
GEI 4.5.2 Extent to which the routine statistical system provides reliable periodic information on governance-related needs among populations in different communities.	○	○	○	○	○	○	○	○	○	○	○
GEI 4.5.3 Extent to which a routine statistical system provides reliable periodic information on economic indicators.	○	○	○	○	○	○	○	○	○	○	○
GEI 4.5.4 Extent to which evaluations of the monitoring and surveillance systems are conducted and applied to ensure the reliability of data.	○	○	○	○	○	○	○	○	○	○	○

* GEI 4.6 Quality Research Institutions: Extent to which the country has the capacity to support and sustain research institutions that develop research/collect data to inform efforts to ensure good governance, stable economy and strong public institutions.

	1	2	3	4	5	6	7	8	9	10	Don't know
1-Lowest -> Highest-10	○	○	○	○	○	○	○	○	○	○	○

* GEI 4.7 Evaluation: Extent to which program statistics, surveys, and small studies are used by specialized staff to report on good governance and financial institutions.

	1	2	3	4	5	6	7	8	9	10	Don't know
1-Lowest -> Highest-10	○	○	○	○	○	○	○	○	○	○	○

* GEI 4.8 Management's Use of Evaluation Findings: Extent to which local-level program managers use research and evaluation findings to improve programming around the rule of law, stabilizing the economy and strengthening institutions in ways suggested by findings.

	1	2	3	4	5	6	7	8	9	10	Don't know
1-Lowest -> Highest-10	○	○	○	○	○	○	○	○	○	○	○

* GEI 4.10 Dissemination of Information to Other Implementing Bodies: Extent to which information is shared or spread across geographical areas and throughout various levels (national, regional, localities).

	1	2	3	4	5	6	7	8	9	10	Don't know
1-Lowest -> Highest-10	○	○	○	○	○	○	○	○	○	○	○

GEI 4.11 Comments: Please use this section to provide any additional comments about the level of effort, challenges, or successes in research around GEI. If you marked "I don't know" for any of the questions, also use this section to explain why you gave that rating.

JOHNS HOPKINS
BLOOMBERG SCHOOL
of PUBLIC HEALTH

Bill & Melinda Gates Institute for Population and Reproductive Health

Demographic Dividend Effort Index Survey

Dimension 5. Civil Society Organizations (CSOs)

* GEI 5.1 CSO Actor Power: Extent to which CSO actors are in strong positions to positively contribute to the rule of law.

	1	2	3	4	5	6	7	8	9	10	Don't know
1-Lowest -> Highest-10	○	○	○	○	○	○	○	○	○	○	○

* GEI 5.2 Budget Analysis as a CSO Tool: Extent to which CSOs utilize budget analysis as a tool to build an advocacy case for good governance, economy stability and stronger public institutions

	1	2	3	4	5	6	7	8	9	10	Don't know
1-Lowest -> Highest-10	○	○	○	○	○	○	○	○	○	○	○

* GEI 5.3 Community-Based Service Delivery Support: Extent to which CSOs participate in bringing governance-related information and services to the communities where people live.

	1	2	3	4	5	6	7	8	9	10	Don't know
1-Lowest -> Highest-10	○	○	○	○	○	○	○	○	○	○	○

* GEI 5.4 Human Rights and Quality of Implementation: Extent to which CSOs advocate for and support rights-based approaches to governance to ensure policy and program beneficiaries experience progress on their terms.

	1	2	3	4	5	6	7	8	9	10	Don't know
1-Lowest -> Highest-10	○	○	○	○	○	○	○	○	○	○	○

* GEI 5.5 Youth: Extent to which CSOs support youth to participate in education, training, employment and leadership.

	1	2	3	4	5	6	7	8	9	10	Don't know
1-Lowest -> Highest-10	○	○	○	○	○	○	○	○	○	○	○

* GEI 5.6 Programs for Women: Extent to which CSOs ensure GEI policies support women and girls to participate in leadership.

	1	2	3	4	5	6	7	8	9	10	Don't know
1-Lowest -> Highest-10	○	○	○	○	○	○	○	○	○	○	○

* GEI 5.7 Advocacy/Accountability

	1	2	3	4	5	6	7	8	9	10	Don't know
GEI 5.7.1 Extent to which CSOs support the participation of the community in governance or enforcement of the rule of law.	○	○	○	○	○	○	○	○	○	○	○
GEI 5.7.2 Extent to which the media support the participation of the community in governance or enforcement of the rule of law.	○	○	○	○	○	○	○	○	○	○	○

* GEI 5.8 CSO-Led Assessment and Monitoring: Extent to which CSOs assess, monitor and report on the effectiveness of policies and government accountability mechanisms and structures.

	1	2	3	4	5	6	7	8	9	10	Don't know
1-Lowest -> Highest-10	○	○	○	○	○	○	○	○	○	○	○

* GEI 5.9 Forming Partnerships between CSOs: Extent to which CSOs have formed national alliances and regional partnerships to strengthen their positioning and potentially improve leadership strength and funding mechanisms for good governance and institutional strengthening related work.

	1	2	3	4	5	6	7	8	9	10	Don't know
1-Lowest -> Highest-10	○	○	○	○	○	○	○	○	○	○	○

GEI 5.10 Comments: Please use this section to provide any additional comments about the level of effort, challenges, or successes around the role of the CSOs and media in GEI. If you marked “I don’t know” for any of the questions, also use this section to explain why you gave that rating

JOHNS HOPKINS
BLOOMBERG SCHOOL
of PUBLIC HEALTH

Bill & Melinda Gates Institute for Population and Reproductive Health

Demographic Dividend Effort Index Survey

Module: Sectoral Resilience and Sustainability

Considering the health and socio-economic impact of the COVID-19 and the likely impact on the DD progress, this DD effort index has integrated questions to assess the resilience and sustainability of systems in key sectors of the DD framework. The items included in the COVID-19 related questionnaire below follow the key dimensions of resilient systems and provide critical information on the potential of an effective response to emerging infectious disease threats or other public health emergencies.

Please score each item from 1 to 10, where 1 represents the lowest score (poor state/capability) and 10 represents the highest score (great state/capability).

* Physical: facilities, equipment, system states, and capabilities.

	1	2	3	4	5	6	7	8	9	10	Don't know
GEI-M1 - Plan/Prepare - State and capability of equipment, personnel and GEI sector system structure prior to the crisis event. (1 = poor state/capability; 10 = great state/capability)											
GEI-M2 - Absorb - Extent to which COVID-19 was recognized within the GEI sector system and the performance of the system was able to maintain functionality.											
GEI-M3 - Absorb - Integration: The level of integration of services and institutional collaborations to ensure continuity of access to essential services during the COVID-19 crisis.											
GEI-M4 - Recover - Extent to which the Government and Economic Institutions have been able to induce change to recover the pre-COVID-19 level of functionality.											
GEI-M5 - Recover - Financing and Donor Management: National bank and donor support to ensure the availability of cash during the COVID-19 crisis											
GEI-M6 - Adapt - Extent to which changes have been made to improve the resilience of the GEI sector system and infrastructure.											

* Information: creation, manipulation, storage, and utilization of data.

	1	2	3	4	5	6	7	8	9	10	Don't know
GEI-M7 - Plan/Prepare - Extent to which GEI-related data was sufficiently prepared, presented, analyzed and stored prior to the crisis event.	○	○	○	○	○	○	○	○	○	○	○
GEI-M8 - Absorb - Awareness: Level of awareness about the threats caused by COVID-19 in the central government.	○	○	○	○	○	○	○	○	○	○	○
GEI-M9 - Recover - Extent to which GEI data has been used to track the progress of recovery and to anticipate recovery scenarios.	○	○	○	○	○	○	○	○	○	○	○
GEI-M10 - Adapt - Extent to which the GEI sector system is creating and improving GEI data storage and real-time use protocols.	○	○	○	○	○	○	○	○	○	○	○

* Cognitive: understanding, mental models, preconceptions, biases, and values.

	1	2	3	4	5	6	7	8	9	10	Don't know
GEI-M11 - Plan/Prepare - Extent to which the GEI sector system design and operation decisions were prepared in anticipation of crisis events.	○	○	○	○	○	○	○	○	○	○	○
GEI-M12 - Absorb - Extent to which the Government and Economic Institutions are responding with sufficient contingency protocols and proactive crisis management.	○	○	○	○	○	○	○	○	○	○	○

	1	2	3	4	5	6	7	8	9	10	Don't know
GEI-M13 - Absorb - Accountability: Level of effort to ensure optimal accountability for the resources allocated to support governance as part of the response to the COVID-19.	○	○	○	○	○	○	○	○	○	○	○
GEI-M14 - Recover - Extent to which decisions are oriented towards recovery, which employ evidence-based communication to community members to promote safe behavior.	○	○	○	○	○	○	○	○	○	○	○
GEI-M15 - Recover - Awareness: Extent to which the government is building confidence among the public though communication and dialogue for evidence-based policymaking and actions.	○	○	○	○	○	○	○	○	○	○	○
GEI-M16 - Adapt - Extent to which the GEI sector system is designing new system configurations, objectives and decision criteria.	○	○	○	○	○	○	○	○	○	○	○
GEI-M17 - Adapt - Adaptive: Level of adaptation of financial and social service systems to ensure easy access via technology-based platforms during the COVID-19.	○	○	○	○	○	○	○	○	○	○	○

* Social: interaction, collaboration, and self-synchronization between individuals, entities and institutions.

	1	2	3	4	5	6	7	8	9	10	Don't know
GEI-M18 - Plan/Prepare - Extent to which training on outbreak/crisis management was conducted through, and which leveraged, social networks, social capital and institutional and cultural norms prior to the crisis event.	○	○	○	○	○	○	○	○	○	○	○
GEI-M19 - Absorb - Extent to which personnel and social institutions have been resourceful and accessible for the outbreak/crisis response.	○	○	○	○	○	○	○	○	○	○	○
GEI-M20 - Absorb - Self-Regulating: Extent to which the national leadership had the authority to make sectoral changes in a timely manner through flexible infrastructure.	○	○	○	○	○	○	○	○	○	○	○
GEI-M21 - Recover - Extent to which the GEI sector system is engaging with team and knowledge sharing to enhance systematic recovery.	○	○	○	○	○	○	○	○	○	○	○
GEI-M22 - Recover - Diversity: The level of engagement of multidisciplinary taskforces to mitigate the effect of COVID-19 at the national and subnational levels.	○	○	○	○	○	○	○	○	○	○	○
GEI-M23 - Adapt - Leadership and Management: Stewardship and management demonstrated by Government in response to the COVID-19 at the national level.	○	○	○	○	○	○	○	○	○	○	○

* GEI M-24 Timeline: Extent of timeliness of the Government reaction - communication and implementation of measures - to mitigate the immediate and longterm impact of COVID-19.

Note: This refers to the entirety of the national reaction - not the reaction that is specific to your sector.

	1	2	3	4	5	6	7	8	9	10	Don't know
1-Lowest -> Highest-10	◯	◯	◯	◯	◯	◯	◯	◯	◯	◯	◯

GEI M-25 Comments: Please use this section to provide any additional comments about the resilience of the national GEI systems based on your experience during the COVID-19 pandemics.If you marked “I don’t know” for any of the questions, also use this section to explain why you gave that rating.

JOHNS HOPKINS
BLOOMBERG SCHOOL of PUBLIC HEALTH

Bill & Melinda Gates Institute for Population and Reproductive Health

Enquête de l'Indice des efforts pour le dividende démographique

Introduction

Les programmes de développement ont besoin d'une mesure standard qui quantifie la portée des efforts nationaux en termes de politiques et programmes mis en œuvre pour cultiver, réaliser et exploiter le dividende démographique (DD). En dépit du progrès remarquable accompli par plusieurs pays à travers l'intégration des principes du DD dans leurs politiques et plans de développement, des outils d'évaluation de la performance et des plateformes documentant les leçons et meilleures pratiques pour bénéficier du DD font toujours défaut.

Ainsi, l'objectif de l'Indice des efforts pour le dividende démographique (IEDD) est de fournir une mesure standard qui permettra aux experts et membres de la société civile de quantifier la nature et la puissance des efforts pour le DD dans les pays, autant dans les sphères publiques que privées, à travers différentes dimensions de la société. En mesurant ces efforts, nous documenterons les intrants indépendamment des extrants, dont les revenus par habitant. Cela nous permettra d'apprécier la relation entre les intrants pris en compte dans l'élaboration des politiques de DD et les extrants du DD.

Cet IEDD est guidé par une question centrale : Dans quelle mesure les efforts politiques et programmatiques existants ont-ils permis un environnement favorable à la culture, la réalisation et l'exploitation des bénéfices d'un dividende démographique ? Pour répondre à cette question, l'IEDD résumera les perceptions d'informateurs clés dans six secteurs identifiés dans la littérature scientifique comme produisant un environnement favorable à l'exploitation du DD. Ces secteurs cadres du DD sont : 1) Planification familiale, 2) Santé maternelle et infantile, 3) Education, 4) Autonomisation des femmes, 5) Marché du travail, et 6) Bonne gouvernance, solidité institutionnelle et stabilité économique. Chaque secteur est mesuré via un questionnaire qui lui est propre et est évalué à travers cinq domaines identifiés dans la littérature scientifique, à savoir : la politique/ prise de décision, les services ou programmes, le plaidoyer, la recherche, et la société civile. Chacun des 6 questionnaires, remplis par 10 observateurs experts par pays, notera chaque élément sur une échelle de 1 à 10, sauf indication contraire.

Étant donné l'impact sanitaire et socioéconomique de la pandémie de COVID-19, et sa relation étroite avec les politiques qui permettent un environnement politique favorable au DD, cet Indice des efforts de DD intègre un module pour évaluer la résilience et la durabilité des systèmes dans chaque secteur. La notation suit les principes clés des systèmes de santé résilients et durables pouvant répondre efficacement aux menaces de maladies infectieuses émergentes et autres crises de santé publiques.

L'IEDD permet de visualiser les efforts nationaux, tout en évaluant la mesure dans laquelle ces efforts soutiennent les objectifs du pays pour cultiver, réaliser et exploiter le dividende démographique. L'IEDD servira d'outil pour mesurer la performance et suivre le progrès, ce qui offrira une évaluation opportune des succès et des marges de progression, des meilleures pratiques et des améliorations possibles.

JOHNS HOPKINS
BLOOMBERG SCHOOL of PUBLIC HEALTH

Bill & Melinda Gates Institute for Population and Reproductive Health

Enquête de l'Indice des efforts pour le dividende démographique

Formulaire de consentement

Merci d'avoir pris le temps de répondre à ce questionnaire, particulièrement en cette période sans précédent.

L'Institut Gates développe actuellement un Indice des efforts pour le dividende démographique (IEDD) afin d'établir une mesure standard qui permettra d'évaluer le statut existant des politiques, services et programmes nationaux de planification familiale, santé maternelle et infantile, et dans d'autres secteurs sociaux (ex : emploi et éducation), ainsi qu'en matière de gouvernance pour permettre un environnement politique favorable au dividende démographique (DD). En mesurant ces efforts, cet outil documentera les intrants indépendamment des extrants, dont les revenus par habitant. Ceci permettra d'évaluer la relation entre les intrants pris en compte dans l'élaboration des politiques de DD et les extrants du DD observés et communément utilisés pour évaluer le progrès.

Nous aimerions vous poser quelques questions en raison de votre expertise dans ce domaine. Les informations collectées seront confidentielles, et nous ne collecterons ni votre nom, ni aucun autre détail permettant de vous identifier. Ce questionnaire devrait vous prendre environ 45-60 min.

Nous vous serions très reconnaissants si vous pouviez participer à cette enquête. Les informations générées par cette interview seront utilisées par l'Institut Gates et la communauté de pratique sur le DD dans votre pays pour évaluer les efforts nationaux, identifier les domaines de réussites qui doivent continuer d'être soutenus et les domaines nécessitant d'être renforcés. Les chercheurs pourront aussi utiliser les données collectées à des fins d'analyse comparative entre les pays.

Vous êtes invité(e) à participer à cette enquête. Sachez que la participation à cette enquête est entièrement volontaire. En participant, vous ne percevrez aucun bénéfice direct, mais il y a des bénéfices sociétaux à permettre aux gouvernements et aux partenaires du développement de renforcer leurs efforts pour cultiver un environnement favorable au DD.

Vous pouvez refuser de répondre à toute question. Si vous avez des inquiétudes ou problèmes concernant cette étude, vous pouvez contacter son coordinateur à **jcrusatira@jhu.edu** ou au **+14436510824** par téléphone ou WhatsApp.

* Veuillez cocher "Oui" si vous souhaitez procéder et répondre à l'enquête.

- ◯ Oui
- ◯ Non

JOHNS HOPKINS
BLOOMBERG SCHOOL of PUBLIC HEALTH

Bill & Melinda Gates Institute for Population and Reproductive Health

Enquête de l’Indice des efforts pour le dividende démographique

Avis important

Veuillez ne pas sélectionner "Page précédente" au milieu d'une page de questions car la page sur laquelle vous travaillez ne sera pas sauvegardée tant que vous n'aurez pas finalisé ladite page et sélectionner "Page suivante". Si vous sélectionnez "Page précédente" sans avoir terminé, vos réponses sur la page inachevée seront perdus.

Sachez que votre enquête est terminée lorsque vous lisez "Merci d'avoir rempli l'indice d'effort du dividende démographique". Merci !

JOHNS HOPKINS
BLOOMBERG SCHOOL
of PUBLIC HEALTH

Bill & Melinda Gates Institute for Population and Reproductive Health

Enquête de l'Indice des efforts pour le dividende démographique

Pays et expertise de l'enquêté(e)

* Veuillez sélectionner le pays duquel vous répondez.

- Kenya
- Senegal
- Nigeria
- Rwanda
- Tanzania
- Ethiopia

JOHNS HOPKINS
BLOOMBERG SCHOOL
of PUBLIC HEALTH

Bill & Melinda Gates Institute for Population and Reproductive Health

Enquête de l'Indice des efforts pour le dividende démographique

Level of expertise

* At which level of Government is your expertise (If your expertise is at the national level please select "National" otherwise select a county?

JOHNS HOPKINS
BLOOMBERG SCHOOL
of PUBLIC HEALTH

Bill & Melinda Gates Institute for Population and Reproductive Health

Enquête de l'Indice des efforts pour le dividende démographique

Niveau d'expertise

* A quel niveau de gouvernement votre expertise se situe-t-elle (Si votre expertise se situe au niveau national, veuillez sélectionner "National", sinon sélectionnez une région ?

JOHNS HOPKINS
BLOOMBERG SCHOOL
of PUBLIC HEALTH

Bill & Melinda Gates Institute for Population and Reproductive Health

Enquête de l'Indice des efforts pour le dividende démographique

Expertise

* Veuillez sélectionner le type d'organisation dans laquelle vous avez le plus travaillé au cours des cinq dernières années. Si votre type d'organisation n'est pas référencé, veuillez préciser de quel type d'organisation il s'agit.

- Secteur public
- Secteur privé
- Organisation non gouvernementale
- Université/ Institut de recherche
- Autre (veuillez préciser)
- Other (please specify)

* Dans quel domaine avez-vous le plus travaillé au cours des cinq dernières années ?

- Politique/ Prise de décision
- Services ou programmes
- Plaidoyer
- Recherche
- Société Civile

* Dans quel secteur avez-vous le plus travaillé dans les cinq dernières années ?

- Planification familiale
- Santé maternelle et infantile
- Education
- Autonomisation des femmes
- Marché du travail
- Gouvernance et institutions économiques

JOHNS HOPKINS
BLOOMBERG SCHOOL of PUBLIC HEALTH

Bill & Melinda Gates Institute for Population and Reproductive Health

Enquête de l'Indice des efforts pour le dividende démographique

Questionnaire sur la planification familiale (PF)

Répondez aux questions suivantes en fonction de votre expérience/ expertise dans ce secteur spécifique.

Afin d'obtenir une représentation synthétique des efforts nationaux pour le dividende démographique, notez les éléments suivants sur une échelle de 1 à 10, 1 étant le score le plus faible (efforts très faibles ou quasi-inexistants) et 10 le plus élevé (efforts robustes). Le cas échéant, si une politique ou activité n'existe pas, répondez 0.

Donnez un score à chaque élément. Toutes les réponses seront enregistrées au format illustré ci-dessous :

Effort le plus faible	1	2	3	4	5	6	7	8	9	10	Effort le plus élevé	Je ne sais pas

JOHNS HOPKINS
BLOOMBERG SCHOOL
of PUBLIC HEALTH

Bill & Melinda Gates Institute for Population and Reproductive Health

Enquête de l’Indice des efforts pour le dividende démographique

Domaine 1. Politique/ Prise de décision

* FP 1.1 La proéminence de la planification familiale (PF) : Mesure dans laquelle il existe un bureau ou poste permanent et bien placé au ministère compétent qui développe et surveille les politiques et stratégies du secteur, et dispose d’effectifs adéquats et de personnel qualifié.

	1	2	3	4	5	6	7	8	9	10	Je ne sais pas
1-Plus faible -> Plus élevé-10	○	○	○	○	○	○	○	○	○	○	○

* FP 1.2 La politique de PF et réduction de la fécondité : Mesure dans laquelle la politique gouvernementale met l’accent sur la PF pour promouvoir la taille des familles en lien avec les ressources disponibles ou des raisons démographiques plutôt que des raisons sanitaires.

	1	2	3	4	5	6	7	8	9	10	Je ne sais pas
1-Plus faible -> Plus élevé-10	○	○	○	○	○	○	○	○	○	○	○

* FP 1.3 Le plan national stratégique de PF : Mesure dans laquelle il existe un plan national actualisé, exhaustif et stratégique pour la PF, définissant les politiques, lois et régulations pour l’offre de PF. (Évaluez la mesure dans laquelle le plan a une vision, comprend des stratégies sur plusieurs années, des calendriers annuels, et des plans de mise en œuvre, tout en étant fondés sur des données probantes, avec des indicateurs de progrès, et tenant compte du marché de la PF).

	1	2	3	4	5	6	7	8	9	10	Je ne sais pas
1-Plus faible -> Plus élevé-10	○	○	○	○	○	○	○	○	○	○	○

* FP 1.4 La politique sur l'âge légal du mariage : Mesure dans laquelle l'âge légal du mariage pour les filles, établi à 18 ans ou plus, est appliqué.

	1	2	3	4	5	6	7	8	9	10	Je ne sais pas
1-Plus faible -> Plus élevé-10	◯	◯	◯	◯	◯	◯	◯	◯	◯	◯	◯

* FP 1.5 La prise de décision fondée sur des données probantes : Mesure dans laquelle le ministère compétent soutient et utilise activement et régulièrement les données clés et rapports de PF pour élaborer ses politiques et ses plans.

	1	2	3	4	5	6	7	8	9	10	Je ne sais pas
1-Plus faible -> Plus élevé-10	◯	◯	◯	◯	◯	◯	◯	◯	◯	◯	◯

* FP 1.6 Le suivi et évaluation des politiques/plans/stratégies nationaux : Mesure dans laquelle le plan national de PF établit une stratégie de suivi et évaluation complète pour suivre et mesurer les accomplissements prévus dans le plan. (Évaluez si la stratégie de suivi et évaluation inclut des buts et objectifs, un cadre de résultats, des indicateurs mesurables [ex : "SMART": spécifiques, mesurables, atteignables, réalistes et temporellement définis], et un plan/calendrier exploitable de mise en œuvre.)

	1	2	3	4	5	6	7	8	9	10	Je ne sais pas
1-Plus faible -> Plus élevé-10	◯	◯	◯	◯	◯	◯	◯	◯	◯	◯	◯

* FP 1.7 L'inclusion des acteurs non gouvernementaux et intersectoriels au développement de politique/stratégie : Mesure dans laquelle les agents non-gouvernementaux, les associations (ex : de la société civile), les travailleurs de la santé, les parties prenantes (ex : organisations confessionnelles, à but non lucratif et à but lucratif) et les acteurs d'autres secteurs que celui de la PF sont inclus dans le plan national de PF et/ou autres documents nationaux établissant les stratégies et systèmes du pays pour planifier, gérer et développer la PF.

	1	2	3	4	5	6	7	8	9	10	Je ne sais pas
1-Plus faible -> Plus élevé-10	◯	◯	◯	◯	◯	◯	◯	◯	◯	◯	◯

* FP 1.8 Le financement de la PF : Mesure dans laquelle le gouvernement a établi et appliqué des directives pour la budgétisation et le paiement de la PF en termes d'offre et de demande (soit les financements intérieurs vs. internationaux, et leur continuité/durabilité, coût des produits contraceptifs, coût de la prestation de services et du transport, etc.).

(Évaluez si les directives et le budget tiennent compte de la durée de vie des coûts des soins et services de PF ; des tarifications variables par méthode de PF ; des coûts de livraison et prestation de services par spécialité, lieu et risque ; des coûts et profits des plans/politiques d'assurance ; du coût financier de fournir de la PF en comparaison au coût financier de ne pas le faire, etc.).

	1	2	3	4	5	6	7	8	9	10	Je ne sais pas
1-Plus faible -> Plus élevé-10	◯	◯	◯	◯	◯	◯	◯	◯	◯	◯	◯

* FP 1.9 Le pourcentage du financement intérieur (domestique) pour le budget de la PF : Mesure dans laquelle le budget total de la planification familiale/population est dérivé de sources domestiques (ex : 1 pour 10%, 5 pour 50%, 10 pour 100%).

	1	2	3	4	5	6	7	8	9	10	Je ne sais pas
1-Plus faible -> Plus élevé-10	◯	◯	◯	◯	◯	◯	◯	◯	◯	◯	◯

* FP 1.10 L'infrastructure/ capacité des Technologies de l'Information et la Communication (TIC) : Mesure dans laquelle il existe des éléments d'infrastructure (ex : ordinateurs, serveurs, imprimantes, réseau local, connexion internet, alimentation électrique fiable) ainsi que des ressources humaines (ex : pour la maintenance et le soutien) au niveau national et infranational permettant de soutenir des systèmes d'informations fonctionnels sur la PF.

(Pour cet élément, évaluez si le pays conduit au moins des évaluations annuelles de ses systèmes informatiques ; a des programmes de formation couvrant la saisie et l'utilisation des données, l'administration et le développement de logiciels ; et forme continuellement le personnel des institutions clés [ex : Ministère de la Santé, Ministère de l'Éducation, les agences régulatrices et les institutions de formation]).

	1	2	3	4	5	6	7	8	9	10	Je ne sais pas
1-Plus faible -> Plus élevé-10	◯	◯	◯	◯	◯	◯	◯	◯	◯	◯	◯

* FP 1.11 Les lois et régulations sur l'importation :

	1	2	3	4	5	6	7	8	9	10	Je ne sais pas
FP 1.11.1 Mesure dans laquelle les lois et régulations sur l'importation facilitent l'importation des commodités et produits de PF.	○	○	○	○	○	○	○	○	○	○	○
FP 1.11.2 Mesure dans laquelle les contraceptifs sont fabriqués localement.	○	○	○	○	○	○	○	○	○	○	○

* FP 1.12 La publicité des contraceptifs autorisés : Mesure dans laquelle la publicité des contraceptifs dans les médias de masse n'est soumise à aucune restriction.

	1	2	3	4	5	6	7	8	9	10	Je ne sais pas
1-Plus faible -> Plus élevé-10	○	○	○	○	○	○	○	○	○	○	○

* FP 1.13 La présence de politiques de PF favorisant les populations pauvres : Mesure dans laquelle les politiques ciblent spécifiquement les populations pauvres en milieu rural et urbain.

	1	2	3	4	5	6	7	8	9	10	Je ne sais pas
1-Plus faible -> Plus élevé-10	○	○	○	○	○	○	○	○	○	○	○

* FP 1.14 Le soutien politique à la PF : Mesure dans laquelle les élus dans le pays priorisent les besoins de PF pour renforcer les contributions de la PF au DD en adoptant des lois et régulations, et en appuyant des actions et politiques visant à améliorer l'utilisation et l'adhérence à la PF.

	1	2	3	4	5	6	7	8	9	10	Je ne sais pas
1-Plus faible -> Plus élevé-10	○	○	○	○	○	○	○	○	○	○	○

FP 1.15 Commentaires : Veuillez utiliser cette section pour tout commentaire additionnel sur le niveau d'effort, les difficultés et les succès rencontrés dans le pays autour des politiques/ prise de décision pour la PF. Si vous avez répondu « Je ne sais pas » à l'une des questions, utilisez aussi cette section pour expliquer pourquoi.

JOHNS HOPKINS
BLOOMBERG SCHOOL
of PUBLIC HEALTH

Bill & Melinda Gates Institute for Population and Reproductive Health

Enquête de l'Indice des efforts pour le dividende démographique

Domaine 2. Services ou programmes

* FP 2.1 La structure administrative : Mesure dans laquelle la structure administrative et le personnel au niveau national, provincial et des comtés est suffisant pour mettre en œuvre le programme de planification familiale (PF).

	1	2	3	4	5	6	7	8	9	10	Je ne sais pas
1-Plus faible -> Plus élevé-10											

* FP 2.2 Le niveau des responsables du programme : Niveau d'ancienneté relatif du directeur du programme national de planification familiale et mesure dans laquelle le supérieur hiérarchique direct de ce directeur est haut placé au gouvernement.

	1	2	3	4	5	6	7	8	9	10	Je ne sais pas
1-Plus faible -> Plus élevé-10											

* FP. 2.3 La distribution à base communautaire : Mesure dans laquelle les zones du pays non desservies par les cliniques et autres sites de prestation de santé sont couvertes par des programmes de distribution à base communautaire de produits contraceptifs (particulièrement les zones rurales).

	1	2	3	4	5	6	7	8	9	10	Je ne sais pas
1-Plus faible -> Plus élevé-10											

* FP 2.4 Le programme de PF postpartum (PFPP) : Mesure dans laquelle toutes les femmes en postpartum reçoivent des services PFPP.

	1	2	3	4	5	6	7	8	9	10	Je ne sais pas
1-Plus faible -> Plus élevé-10											

* FP 2.5 Les agents travaillant à domicile : Mesure de la couverture de la population par les agents dont la tâche principale est de rendre visite aux femmes (en milieu rural) chez elles pour discuter de planification familiale et de santé maternelle et infantile.

	1	2	3	4	5	6	7	8	9	10	Je ne sais pas
1-Plus faible -> Plus élevé-10	○	○	○	○	○	○	○	○	○	○	○

* FP 2.6 La disponibilité et l'accessibilité des commodités/produits et services de PF (première partie) : Mesure dans laquelle l'ensemble de la population a facilement et directement accès à (1-le plus faible -> le plus élevé-10):

	1	2	3	4	5	6	7	8	9	10	Je ne sais pas
FP 2.6.1 DIU	○	○	○	○	○	○	○	○	○	○	○
FP 2.6.2 Pilule	○	○	○	○	○	○	○	○	○	○	○
FP 2.6.3 Injectable	○	○	○	○	○	○	○	○	○	○	○
FP 2.6.4 Implant	○	○	○	○	○	○	○	○	○	○	○
FP 2.6.5 Stérilisation féminine	○	○	○	○	○	○	○	○	○	○	○
FP 2.6.6 Stérilisation masculine	○	○	○	○	○	○	○	○	○	○	○
FP 2.6.7 Préservatif masculin	○	○	○	○	○	○	○	○	○	○	○
FP 2.6.8 Contraception d'urgence	○	○	○	○	○	○	○	○	○	○	○
FP 2.6.9 Avortement/ régulation de la menstruation (légal ou non)	○	○	○	○	○	○	○	○	○	○	○
FP 2.6.10 Autres (ex : préservatif féminin, mousse, collier du cycle, etc.)	○	○	○	○	○	○	○	○	○	○	○

* FP 2.7 La disponibilité et l'accessibilité des commodités/produits et services de PF, (deuxième partie) : Quel est le niveau de fonctionnalité du système d'approvisionnement (évite t-il les ruptures de stock ou l'interruption de l'approvisionnement, ou permet-il un flux fiable à tous les niveaux ?) pour les méthodes suivantes (1-le plus faible -> le plus élevé-10) :

	1	2	3	4	5	6	7	8	9	10	Don't know
FP 2.7.1 DIU	○	○	○	○	○	○	○	○	○	○	○
FP 2.7.2 Pilule	○	○	○	○	○	○	○	○	○	○	○
FP 2.7.3 Injectable	○	○	○	○	○	○	○	○	○	○	○
FP 2.7.4 Implant	○	○	○	○	○	○	○	○	○	○	○
FP 2.7.5 Préservatif masculin	○	○	○	○	○	○	○	○	○	○	○
FP 2.7.6 Contraception d'urgence	○	○	○	○	○	○	○	○	○	○	○
FP 2.7.7 Autres (ex : préservatif féminin, mousse, collier du cycle, etc.)	○	○	○	○	○	○	○	○	○	○	○

* FP 2.8 La disponibilité et l'accessibilité des commodités/produits et services de PF (troisième partie) : Dans quelle mesure le système d'approvisionnement fournit-il l'équipement et les produits médicaux nécessaires aux structures cliniques pour les méthodes suivantes (1-le plus faible -> le plus élevé-10) :

	1	2	3	4	5	6	7	8	9	10	Je ne sais pas
FP 2.8.1 Stérilisation féminine	○	○	○	○	○	○	○	○	○	○	○
FP 2.8.2 Stérilisation masculine	○	○	○	○	○	○	○	○	○	○	○
FP 2.8.3. DIU	○	○	○	○	○	○	○	○	○	○	○
FP 2.8.4 Implant	○	○	○	○	○	○	○	○	○	○	○

Other (please specify)

* FP 2.9 La qualité des services de PF : Veuillez noter la qualité générale des services de planification familiale. (Des services de haute qualité impliquent d'être orientés sur les besoins des clients, et de fournir du conseil, des informations complètes, un large choix de méthodes, et des procédures cliniques sûres).

	1	2	3	4	5	6	7	8	9	10	Je ne sais pas
1-Plus faible -> Plus élevé-10	○	○	○	○	○	○	○	○	○	○	○

* FP 2.10 Les avantages et les effets dissuasifs : Mesure dans laquelle des avantages monétaires ou d'autre type sont utilisés pour encourager l'adoption de la planification familiale.

	1	2	3	4	5	6	7	8	9	10	Je ne sais pas
1-Plus faible -> Plus élevé-10	○	○	○	○	○	○	○	○	○	○	○

* FP 2.11 Les programmes de PF pour les adolescents : Mesure dans laquelle les informations, services et activités sont focalisés sur les adolescents et encouragent ces derniers à accéder aux services contraceptifs.

	1	2	3	4	5	6	7	8	9	10	Je ne sais pas
1-Plus faible -> Plus élevé-10	○	○	○	○	○	○	○	○	○	○	○

* FP 2.12 Les programmes de formation : Mesure dans laquelle les programmes de formation, pour chaque catégorie de personnel dans le programme de planification familiale, sont adéquats pour transmettre au personnel les informations et compétences nécessaires à la conduite efficace de leur travail.

	1	2	3	4	5	6	7	8	9	10	Je ne sais pas
1-Plus faible -> Plus élevé-10	○	○	○	○	○	○	○	○	○	○	○

* FP 2.13 Les tâches assignées remplies par le personnel : Mesure dans laquelle toutes les catégories du personnel du programme de planification familiale (administratif, médical, paramédical, de terrain) remplissent leurs tâches assignées efficacement.

	1	2	3	4	5	6	7	8	9	10	Je ne sais pas
1-Plus faible -> Plus élevé-10	○	○	○	○	○	○	○	○	○	○	○

* FP 2.14 La logistique et le transport : Mesure dans laquelle les systèmes logistiques et de transport sont suffisants pour maintenir les stocks de produits contraceptifs et l'équipement disponible sur tous les sites de prestation de santé, à tout moment et à tous les niveaux.

	1	2	3	4	5	6	7	8	9	10	Je ne sais pas
1-Plus faible -> Plus élevé-10	○	○	○	○	○	○	○	○	○	○	○

* FP 2.15 Le système de supervision : Mesure dans laquelle le système de supervision à tous les niveaux est adéquat (visite de supervision régulières, suivies par des actions correctives ou d'appui/accompagnantes).

	1	2	3	4	5	6	7	8	9	10	Je ne sais pas
1-Plus faible -> Plus élevé-10	○	○	○	○	○	○	○	○	○	○	○

* FP 2.16 L’implication d’autres ministères et agences publiques : Mesure dans laquelle d’autres ministères, agences gouvernementales et parties prenantes non gouvernementales s’impliquent dans les activités de PF (ex : livraison de produits, prestation de services, information, éducation) ou autres activités pour la population.

	1	2	3	4	5	6	7	8	9	10	Je ne sais pas
1-Plus faible -> Plus élevé-10	○	○	○	○	○	○	○	○	○	○	○

* FP 2.17 L’implication d’agences et groupes du secteur privé : Mesure dans laquelle des agences ou groupes du secteur privé contribuent aux activités de PF ou autres activités pour la population.

	1	2	3	4	5	6	7	8	9	10	Je ne sais pas
1-Plus faible -> Plus élevé-10	○	○	○	○	○	○	○	○	○	○	○

* FP 2.18 Les médias de masse pour la communication, l’éducation et l’information : Fréquence et couverture des messages des médias de masse fournissant à la population des informations sur a planification familiale et les sites de prestation de services.

	1	2	3	4	5	6	7	8	9	10	Je ne sais pas
1-Plus faible -> Plus élevé-10	○	○	○	○	○	○	○	○	○	○	○

* FP 2.19 Les barrières normatives, culturelles et sociales : Mesure dans laquelle les programmes de planification familiale répondent aux barrières culturelles et sociales.

	1	2	3	4	5	6	7	8	9	10	Je ne sais pas
1-Plus faible -> Plus élevé-10	○	○	○	○	○	○	○	○	○	○	○

* FP 2.20 Les jeunes : Mesure dans laquelle le gouvernement soutient des initiatives de PF dirigées par les jeunes en matière de planification, mise en œuvre et évaluation des programmes de PF.

	1	2	3	4	5	6	7	8	9	10	Je ne sais pas
1-Plus faible -> Plus élevé-10	○	○	○	○	○	○	○	○	○	○	○

* FP 2.21 Les influences des efforts de PF (les forces influençant le programme de PF) : Les forces influençant le programme de PF peuvent soit renforcer, soit diminuer son efficacité, Cochez zéro (0) s'il n'y a aucune différence ; cochez un nombre négatif de -1 à -5 si l'influence est négative ; ou cochez un nombre positif de 1 à 5 si l'influence est positive. (1 dans chaque direction signifie peu d'influence et 5 dans chaque direction signifie beaucoup d'influence).

	-5	-4	-3	-2	-1	0	1	2	3	4	5	Je ne sais pas
FP 2.21.1 Décentralisation	○	○	○	○	○	○	○	○	○	○	○	○
FP 2.21.2 Programmes VIH/SIDA	○	○	○	○	○	○	○	○	○	○	○	○
FP 2.21.3 Incorporation de la PF au contexte plus large de la santé reproductive (SR)	○	○	○	○	○	○	○	○	○	○	○	○
FP 2.21.4 Intégration de la PF à d'autres services de santé	○	○	○	○	○	○	○	○	○	○	○	○
FP 2.21.5 Changements dans les financements des bailleurs de fonds	○	○	○	○	○	○	○	○	○	○	○	○
FP 2.21.6 Changements dans les financements du gouvernement	○	○	○	○	○	○	○	○	○	○	○	○

FP 2.22 Commentaires : Veuillez utiliser cette section pour tout commentaire additionnel sur le niveau d'effort, les difficultés et les succès rencontrés dans le pays autour des services ou programmes de PF. Si vous avez répondu « Je ne sais pas » à l'une des questions, utilisez aussi cette section pour expliquer pourquoi.

JOHNS HOPKINS
BLOOMBERG SCHOOL
of PUBLIC HEALTH

Bill & Melinda Gates Institute for Population and Reproductive Health

Enquête de l'Indice des efforts pour le dividende démographique

Domaine 3. Plaidoyer

* FP 3.1 L'influence des dirigeants/ champions de PF : Mesure dans laquelle le pays compte sur un ou plusieurs dirigeants ou champions clairement influents plaidant avec succès au profit des besoins (sectoriels) de la PF auprès des décideurs, promouvant la PF dans le pays à travers des déclarations publiques positives, et/ou soutenant des actions et politiques visant à améliorer l'environnement de la PF et ses contributions au DD.

	1	2	3	4	5	6	7	8	9	10	Je ne sais pas
1-Plus faible -> Plus élevé-10	○	○	○	○	○	○	○	○	○	○	○

* FP 3.2 Les déclarations des dirigeants : Mesure dans laquelle le chef du gouvernement, ainsi que d'autres hauts fonctionnaires, parlent publiquement et favorablement de la planification familiale au moins une fois par an.

	1	2	3	4	5	6	7	8	9	10	Je ne sais pas
1-Plus faible -> Plus élevé-10	○	○	○	○	○	○	○	○	○	○	○

* FP 3.3 La couverture médiatique de la PF : Mesure dans laquelle et fréquence à laquelle la couverture médiatique nationale des sujets de PF apporte à la population des informations sur la planification familiale et les sites de prestation de services.

	1	2	3	4	5	6	7	8	9	10	Je ne sais pas
1-Plus faible -> Plus élevé-10	○	○	○	○	○	○	○	○	○	○	○

* FP 3.4 Les influences des efforts de PF (justification) : Quelle est l'importance de chacun des éléments suivants en tant que justification actuelle par le gouvernement de son programme national de PF ? (1 signifie une importance négligeable ; 10 signifie une grande importance.)

	1	2	3	4	5	6	7	8	9	10	Je ne sais pas
3.4.1 Réduire le taux de croissance démographique	○	○	○	○	○	○	○	○	○	○	○
3.4.2 Stimuler le développement économique	○	○	○	○	○	○	○	○	○	○	○
3.4.3 Aider les hommes et les femmes à éviter des naissances non planifiées et non désirées	○	○	○	○	○	○	○	○	○	○	○
3.4.4 Améliorer la santé de la femme	○	○	○	○	○	○	○	○	○	○	○
3.4.5 Améliorer la santé de l'enfant	○	○	○	○	○	○	○	○	○	○	○
3.4.6 Réduire les grossesses chez les adolescentes non mariées	○	○	○	○	○	○	○	○	○	○	○
3.4.7 Réduire les besoins contraceptifs non satisfaits	○	○	○	○	○	○	○	○	○	○	○

* FP 3.5 Les influences des efforts de PF (les populations vulnérables) : Dans quelle mesure le programme de planification familiale met-il l'accent sur les populations vulnérables ou spécifiques ? (1 signifie un accent négligeable ; 10 signifie un accent important).

	1	2	3	4	5	6	7	8	9	10	Je ne sais pas
FP 3.5.1 Les jeunes non mariés											
FP 3.5.2 Les populations vivant dans la pauvreté											
FP 3.5.3 Les personnes en situation de handicap											
FP 3.5.4 Les populations rurales											
FP 3.5.5 Autres groupes vulnérables (ex : les minorités et/ou les groupes indigènes)											
FP 3.5.6 Les femmes en postpartum pour le conseil et les services de contraception											
FP 3.5.7 Les femmes en post-avortement pour le conseil et les services de contraception											

* FP 3.6 Le marketing social : Mesure de la couverture du programme de marketing social du pays (ventes des contraceptifs subventionnés à faible coût dans le secteur commercial, particulièrement en milieu urbain).

	1	2	3	4	5	6	7	8	9	10	Je ne sais pas
1-Plus faible -> Plus élevé-10											

FP 3.7 Commentaires : Veuillez utiliser cette section pour tout commentaire additionnel sur le niveau d'effort, les difficultés et les succès rencontrés dans le pays autour du plaidoyer pour la PF. Si vous avez répondu « Je ne sais pas » à l'une des questions, utilisez aussi cette section pour expliquer pourquoi.

Johns Hopkins
Bloomberg School of Public Health

Bill & Melinda Gates Institute for Population and Reproductive Health

Enquête de l'Indice des efforts pour le dividende démographique

Domaine 4. Recherche

* FP 4.1 La force des observatoires/parties prenantes/groupes de travail pour la PF : Mesure dans laquelle les parties prenantes du secteur de la planification familiale du pays (ex : groupe directif de parties prenantes, groupes de travail techniques, observatoire de la PF, groupe de coordination ou facilitation) :

	1	2	3	4	5	6	7	8	9	10	Je ne sais pas
FP 4.1.1 Ont un représentant du gouvernement, des institutions de formation, de la société civile, des organisations non gouvernementales et confessionnelles, des associations professionnelles, du secteur privé, etc.	○	○	○	○	○	○	○	○	○	○	○
FP 4.1.2 Se réunissent régulièrement, émettent des rapports et recommandent des politiques aux directions des ministères compétents.	○	○	○	○	○	○	○	○	○	○	○
FP 4.1.3 Ont un impact sur la PF dans le pays.	○	○	○	○	○	○	○	○	○	○	○

* FP 4.2 La stratégie de recherche en PF : Mesure dans laquelle le plan national de PF et/ou d'autres documents nationaux comprennent une stratégie/approche complète de recherche sur la planification familiale.

	1	2	3	4	5	6	7	8	9	10	Je ne sais pas
1-Plus faible -> Plus élevé-10	○	○	○	○	○	○	○	○	○	○	○

* FP 4.3 Les partenaires collectant les données :

	1	2	3	4	5	6	7	8	9	10	Je ne sais pas
FP 4.3.1 Mesure dans laquelle la collecte des données est entreprise par des agences gouvernementales. (Cela peut comprendre les agences/bureaux de la statistique et/ou les ministères de l'économie/finances, éducation, santé, genre, etc.)	○	○	○	○	○	○	○	○	○	○	○
FP 4.3.2 Mesure dans laquelle la collecte de données est entreprise par des institutions de recherche.	○	○	○	○	○	○	○	○	○	○	○
FP 4.3.3 Mesure dans laquelle la collecte des données est entreprise par des chercheurs indépendants.	○	○	○	○	○	○	○	○	○	○	○

* FP 4.4 La recherche thématique :

	1	2	3	4	5	6	7	8	9	10	Je ne sais pas
FP 4.4.1 Mesure dans laquelle la recherche est menée sur l'adoption de la planification familiale (dont la distribution des méthodes), la demande et les besoins non satisfaits.	○	○	○	○	○	○	○	○	○	○	○
FP 4.4.2 Mesure dans laquelle de la recherche est menée sur les barrières à l'adoption de la PF	○	○	○	○	○	○	○	○	○	○	○
FP 4.4.3 Mesure dans laquelle de la recherche est menée sur les programmes de PF	○	○	○	○	○	○	○	○	○	○	○
FP 4.4.4 Mesure dans laquelle de la recherche est menée sur la qualité de la prestation des services de PF	○	○	○	○	○	○	○	○	○	○	○

* FP 4.5 La qualité/couverture des données : Mesure dans laquelle la recherche/les données actuelles sont désagrégées par sexe et par âge. Mesure dans laquelle le pays participe aux tests standardisés et la place qu'il occupe au classement.

	1	2	3	4	5	6	7	8	9	10	Je ne sais pas
FP 4.5.1 Mesure dans laquelle un système statistique de routine offre de bonnes informations périodiques sur les produits et les établissements de PF	○	○	○	○	○	○	○	○	○	○	○
FP 4.5.2 Mesure dans laquelle un système statistique de routine offre de bonnes informations régulières sur les services et le personnel du secteur de la PF	○	○	○	○	○	○	○	○	○	○	○
FP 4.5.3 Mesure dans laquelle un système statistique de routine offre de bonnes informations périodiques sur les besoins de PF des populations de différentes communautés	○	○	○	○	○	○	○	○	○	○	○
FP 4.5.4 Mesure dans laquelle des évaluations du système de suivi des informations sanitaires sont menées et appliquées pour assurer la fiabilité des données	○	○	○	○	○	○	○	○	○	○	○

* FP 4.6 Les registres : Mesure dans laquelle les systèmes de registres des clients, rapports des cliniques et retours/commentaires sur les résultats sont adéquats et connectés à tous les niveaux (fédéral, état/province, comté, etc.).

	1	2	3	4	5	6	7	8	9	10	Je ne sais pas
1-Plus faible -> Plus élevé-10	○	○	○	○	○	○	○	○	○	○	○

* FP 4.7 La qualité des institutions de recherche : Mesure dans laquelle le pays a la capacité de soutenir et maintenir des institutions de recherche qui développent des études permettant de soutenir la portée et la qualité de la collecte de données pour informer les efforts nationaux de PF.

	1	2	3	4	5	6	7	8	9	10	Je ne sais pas
1-Plus faible -> Plus élevé-10	◯	◯	◯	◯	◯	◯	◯	◯	◯	◯	◯

* FP 4.8 L'évaluation : Mesure dans laquelle les statistiques, enquêtes et études sur le programme de PF sont utilisées par du personnel spécialisé pour rapporter les opérations du programme et mesurer le progrès.

	1	2	3	4	5	6	7	8	9	10	Je ne sais pas
1-Plus faible -> Plus élevé-10	◯	◯	◯	◯	◯	◯	◯	◯	◯	◯	◯

* FP 4.9 L'utilisation des résultats d'évaluation par les gestionnaires de programmes : Mesure dans laquelle les gestionnaires locaux du programme de PF utilisent les conclusions de recherche et d'évaluations pour améliorer ces programmes en suivant les recommandations émises.

	1	2	3	4	5	6	7	8	9	10	Je ne sais pas
1-Plus faible -> Plus élevé-10	◯	◯	◯	◯	◯	◯	◯	◯	◯	◯	◯

* FP 4.10 L'utilisation des résultats d'évaluation par les ministères : Mesure dans laquelle les administrateurs ministériels compétents utilisent systématiquement les données pour informer les politiques et interventions visant à résoudre les problèmes liés à la PF.

	1	2	3	4	5	6	7	8	9	10	Je ne sais pas
1-Plus faible -> Plus élevé-10	◯	◯	◯	◯	◯	◯	◯	◯	◯	◯	◯

* FP 4.11 La dissémination d'informations à d'autres entités chargées de la mise en œuvre des programmes : Mesure dans laquelle des informations sont partagées ou disséminées entre géographies et à différents niveaux (national, état/province/comté, localités/sous-comtés).

	1	2	3	4	5	6	7	8	9	10	Je ne sais pas
1-Plus faible -> Plus élevé-10	◯	◯	◯	◯	◯	◯	◯	◯	◯	◯	◯

FP 4.12 Commentaires : Veuillez utiliser cette section pour tout commentaire additionnel sur le niveau d'effort, les difficultés et les succès rencontrés dans le pays autour de la recherche sur la PF. Si vous avez répondu « Je ne sais pas » à l'une des questions, utilisez aussi cette section pour expliquer pourquoi.

JOHNS HOPKINS
BLOOMBERG SCHOOL
of PUBLIC HEALTH

Bill & Melinda Gates Institute for Population and Reproductive Health

Enquête de l'Indice des efforts pour le dividende démographique

Domaine 5. Organisations de la société civile (OSC)

* FP 5.1 Le pouvoir des acteurs des OSC : Mesure dans laquelle les acteurs des OSC occupent des postes d'influence dans les institutions, les groupes de travail techniques ou les réseaux concernés par la PF.

	1	2	3	4	5	6	7	8	9	10	Je ne sais pas
1-Plus faible -> Plus élevé-10	○	○	○	○	○	○	○	○	○	○	○

* FP 5.2 L'analyse budgétaire comme outil des OSC : Mesure dans laquelle les OSC utilisent l'analyse budgétaire comme un outil pour développer le plaidoyer en matière de PF.

	1	2	3	4	5	6	7	8	9	10	Je ne sais pas
1-Plus faible -> Plus élevé-10	○	○	○	○	○	○	○	○	○	○	○

* FP 5.3 Le soutien aux services offerts en structure sanitaire : Mesure dans laquelle les OSC sont impliquées dans les efforts visant à augmenter l'utilisation et/ou la qualité des services, particulièrement à travers une plus grande capacité technique et/ou opérationnelle des cliniques travaillant en partenariat avec des CSO pour fournir des services de PF.

	1	2	3	4	5	6	7	8	9	10	Je ne sais pas
1-Plus faible -> Plus élevé-10	○	○	○	○	○	○	○	○	○	○	○

* FP 5.4 Le soutien aux services à base communautaire : Mesure dans laquelle les OSC participent au rapprochement des informations et services de PF avec les communautés à travers des activités telles que le renforcement de compétences des agents de santé communautaire (ASC), l'expansion de la gamme de méthodes contraceptives fournies par les ASC, la formation des cadres inférieurs de professionnels de la santé et/ou les campagnes d'information et d'éducation pour augmenter l'acceptabilité de la contraception.

	1	2	3	4	5	6	7	8	9	10	Je ne sais pas
1-Plus faible -> Plus élevé-10	○	○	○	○	○	○	○	○	○	○	○

* FP 5.5 Le soutien aux services à base communautaire à travers des campagnes mobiles de sensibilisation : Mesure dans laquelle les OSC soutiennent les modèles de services en dehors de cliniques pour que les prestataires de santé aient les commodités et produits de PF, l'équipement et les véhicules nécessaires pour être envoyés ensuite comme équipe spécialiste et fournir des informations et méthodes de PF directement aux communautés.

	1	2	3	4	5	6	7	8	9	10	Je ne sais pas
1-Plus faible -> Plus élevé-10	○	○	○	○	○	○	○	○	○	○	○

* FP 5.6 Mesure dans laquelle les OSC sont impliquées dans la franchise sociale des services de PF de qualité aux client(e)s à moindre coût.

	1	2	3	4	5	6	7	8	9	10	Je ne sais pas
1-Plus faible -> Plus élevé-10	○	○	○	○	○	○	○	○	○	○	○

* FP 5.7 La santé mobile : Mesure dans laquelle les OSC mettent à profit la technologie mobile, comme l'utilisation de la messagerie téléphonique (SMS), pour améliorer l'accès aux informations et services de PF.

	1	2	3	4	5	6	7	8	9	10	Je ne sais pas
1-Plus faible -> Plus élevé-10	○	○	○	○	○	○	○	○	○	○	○

* FP 5.8 Les droits humains, l'accès et la qualité de l'éducation : Mesure dans laquelle les OSC plaident pour des approches basées sur les droits et veillent à ce que les bénéficiaires des services de PF soit traités correctement, n'aient pas peur de poser des questions, et reçoivent des conseils de qualité, entre autres sujets d'importance.

	1	2	3	4	5	6	7	8	9	10	Je ne sais pas
1-Plus faible -> Plus élevé-10	○	○	○	○	○	○	○	○	○	○	○

* FP 5.9 Le changement social et des comportements (CSC) : Mesure dans laquelle les OSC soutiennent les interventions pour le CSC comprenant, mais sans s'y limiter, la promotion des compétences de vie et de l'éducation entre pairs, l'éducation sur la santé de l'adulte, l'éducation en milieu scolaire, la communication au sein du couple, la mobilisation communautaire, les interventions à travers les gardiens de la communauté et/ou les interventions médiatiques destinées au grand public. Ces interventions peuvent être mises en œuvre au niveau communautaire ou individuel, et avoir pour résultat une plus grande utilisation de la PF et des changements dans les connaissances et attitudes liées à la PF.

	1	2	3	4	5	6	7	8	9	10	Je ne sais pas
1-Plus faible -> Plus élevé-10	○	○	○	○	○	○	○	○	○	○	○

* FP 5.10 Les jeunes : Mesure dans laquelle les OSC soutiennent les politiques, interventions et programmes de PF ciblant les jeunes spécifiquement.

	1	2	3	4	5	6	7	8	9	10	Je ne sais pas
1-Plus faible -> Plus élevé-10	○	○	○	○	○	○	○	○	○	○	○

* FP 5.11 Les programmes pour les hommes : Mesure dans laquelle les OSC soutiennent des politiques, interventions et programmes de PF pour les hommes.

	1	2	3	4	5	6	7	8	9	10	Je ne sais pas
1-Plus faible -> Plus élevé-10	○	○	○	○	○	○	○	○	○	○	○

* FP 5.12 Le plaidoyer et la redevabilité : Mesure dans laquelle les OSC soutiennent la formation et le renforcement de compétences des plaideurs et des membres de la communauté pour améliorer leur compréhension des politiques et des processus d'identification de problèmes, de résolution collaborative de ces derniers, et des actions de plaidoyer plus ciblées pour améliorer les programmes de planification familiale.

	1	2	3	4	5	6	7	8	9	10	Je ne sais pas
1-Plus faible -> Plus élevé-10	○	○	○	○	○	○	○	○	○	○	○

* FP 5.13 L'évaluation et le suivi dirigés par les OSC : Mesure dans laquelle les OSC évaluent, suivent et émettent des rapports sur l'efficacité des politiques et programmes pour améliorer la redevabilité des prestataires de santé et des politiques de PF.

	1	2	3	4	5	6	7	8	9	10	Je ne sais pas
1-Plus faible -> Plus élevé-10	○	○	○	○	○	○	○	○	○	○	○

* FP 5.14 Les partenariats entre OSC : Mesure dans laquelle les OSC ont formé des alliances nationales et des partenariats régionaux pour renforcer leur position et potentiellement améliorer le leadership et les mécanismes de financement.

	1	2	3	4	5	6	7	8	9	10	Je ne sais pas
1-Plus faible -> Plus élevé-10	◯	◯	◯	◯	◯	◯	◯	◯	◯	◯	◯

FP 5.15 Commentaires : Veuillez utiliser cette section pour tout commentaire additionnel sur le niveau d'effort, les difficultés et les succès rencontrés dans le pays autour des organisations de la société civile (OSC) pour la PF. Si vous avez répondu « Je ne sais pas » à l'une des questions, utilisez aussi cette section pour expliquer pourquoi.

JOHNS HOPKINS
BLOOMBERG SCHOOL of PUBLIC HEALTH

Bill & Melinda Gates Institute for Population and Reproductive Health

Enquête de l'Indice des efforts pour le dividende démographique

Module : Résilience et durabilité du secteur

Étant donné l'impact sanitaire et socioéconomique de la pandémie de COVID-19, et son impact probable sur la progression vers le DD, cet indice des efforts pour le DD intègre des questions pour évaluer la résilience et la durabilité des systèmes dans les secteurs clés du DD. Les éléments couverts par les questions liées à la pandémie de COVID-19 ci-dessous relèvent des dimensions clés des systèmes résilients et visent à apporter des informations déterminantes sur le potentiel d'une réponse efficace aux menaces de maladies infectieuses émergentes et autres crises de santé publique.

Veuillez noter chaque élément sur une échelle de 1 à 10, 1 étant le score le plus faible (faible état/capacité) et 10 le plus élevé (très bon état/capacité).

* Dimension physique : structures, équipements, états du système et capacités

	1	2	3	4	5	6	7	8	9	10	Je ne sais pas
FP M1 - Plan/Préparation - État et capacité de l'équipement, du personnel et de la structure du programme de PF avant la crise. (1 = faible état/capacité ; 10 = très bon état/capacité)	○	○	○	○	○	○	○	○	○	○	○
FP M2 - Absorption – Intégration : Niveau d'intégration des services et programmes de PF pour mitiger les effets de la pandémie de COVID-19 sur la PF.	○	○	○	○	○	○	○	○	○	○	○
FP M3 - Absorption – Intégration : Mesure dans laquelle la gestion efficace de la chaîne d'approvisionnement a pu mitiger les effets de la pandémie de COVID-19 sur l'approvisionnement/distribution des commodités de PF.	○	○	○	○	○	○	○	○	○	○	○
FP M4 - Récupération - Mesure dans laquelle le secteur de la PF a été capable d'induire des changements pour récupérer un niveau de fonctionnalité pré-COVID-19.	○	○	○	○	○	○	○	○	○	○	○
FP M5 - Récupération – Gestion du financement et des bailleurs de fonds : Niveau de mobilisation des ressources et allocations pour mitiger les effets de la pandémie de COVID-19 sur la PF.	○	○	○	○	○	○	○	○	○	○	○
FP M6 - Adaptation – Mesure dans laquelle des changements ont été effectués pour améliorer la résilience du secteur PF.	○	○	○	○	○	○	○	○	○	○	○

* Dimension informatique : création, gestion, stockage et utilisation des données.

	1	2	3	4	5	6	7	8	9	10	Je ne sais pas
FP M7 - Plan/Préparation – Mesure dans laquelle les données liées à la PF étaient suffisamment préparées, présentées, analysées et stockées avant la crise.	○	○	○	○	○	○	○	○	○	○	○
FP M8 - Absorption – Connaissance : Mesure dans laquelle le programme de PF a pu conduire des évaluations en temps réel de son état de fonctionnement, en anticipation des pertes en cascade des services de PF.	○	○	○	○	○	○	○	○	○	○	○
FP M9 - Récupération – Mesure dans laquelle les données de la PF ont été utilisées pour suivre le progrès vers la récupération et anticiper des scenari de récupération.	○	○	○	○	○	○	○	○	○	○	○
FP M10 - Adaptation – Mesure dans laquelle le système de la PF crée et améliore actuellement ses protocoles de stockage et d'utilisation des données de PF en temps réel.	○	○	○	○	○	○	○	○	○	○	○

* Dimension cognitive : compréhension, modèles cognitifs, idées reçues, préjugés et valeurs.

	1	2	3	4	5	6	7	8	9	10	Je ne sais pas
FP M11 - Planification/Préparation – Mesure dans laquelle le système de la PF et les décisions opérationnelles étaient préparés à anticiper la crise.	○	○	○	○	○	○	○	○	○	○	○

	1	2	3	4	5	6	7	8	9	10	Je ne sais pas
FP M12 - Absorption – Mesure dans laquelle la réponse du secteur de la PF a des protocoles de contingence suffisants et une gestion de crise proactive.	○	○	○	○	○	○	○	○	○	○	○
FP M13 - Absorption – Redevabilité : Niveau d'effort pour assurer une redevabilité optimale vis-à-vis des ressources allouées à la PF dans le cadre de la réponse à la pandémie de COVID-19.	○	○	○	○	○	○	○	○	○	○	○
FP M14 - Récupération – Mesure dans laquelle les décisions sont orientées vers la récupération et communiquées aux communautés sur la base de données probantes pour promouvoir des comportements sûrs.	○	○	○	○	○	○	○	○	○	○	○
FP M15 - Récupération – Connaissance/Conscience : Mesure dans laquelle le secteur de la PF établit une certaine confiance à travers sa communication sur les barrières posées par la pandémie de COVID-19 pour soutenir l'accès et l'utilisation des services et programmes de PF.	○	○	○	○	○	○	○	○	○	○	○
FP M16 - Adaptation : Niveau d'adaptation pour répondre aux menaces posées par la pandémie de COVID-19 à la PF.	○	○	○	○	○	○	○	○	○	○	○

* Social dimension: interaction, collaboration and self-synchronization between people, entities and institutions.

	1	2	3	4	5	6	7	8	9	10	Je ne sais pas
FP M17 - Planification /Préparation – Mesure dans laquelle une formation sur la gestion d'épidémies/crises a été menée et a permis de mettre à profit les réseaux sociaux, le capital social et les normes institutionnelles et culturelles avant la crise.	◯	◯	◯	◯	◯	◯	◯	◯	◯	◯	◯
FP M18 - Absorption – Mesure dans laquelle le personnel et les institutions sociales ont été accessibles et ont fait preuve d'ingéniosité dans leur réponse à l'épidémie/crise.	◯	◯	◯	◯	◯	◯	◯	◯	◯	◯	◯
PF M19 - Absorption - Self-regulation: Extent to which national leaders had the authority to effect timely sectoral changes through an infrastructureflexible.	◯	◯	◯	◯	◯	◯	◯	◯	◯	◯	◯
FP M20 - Récupération – Mesure dans laquelle le secteur de la PF est impliqué dans le partage de connaissances et d'équipes pour améliorer la récupération du système.	◯	◯	◯	◯	◯	◯	◯	◯	◯	◯	◯
FP M21 - Récupération – Diversité : Niveau d'engagement d'une équipe multidisciplinaire pour mitiger les effets de la pandémie de COVID-19 sur la PF.	◯	◯	◯	◯	◯	◯	◯	◯	◯	◯	◯
FP M22 - Adaptation – Mesure dans laquelle des ajouts ou changements sont apportés aux institutions, politiques, formations, programmes et à la culture de la PF.	◯	◯	◯	◯	◯	◯	◯	◯	◯	◯	◯

	1	2	3	4	5	6	7	8	9	10	Je ne sais pas
FP M23 - Adaptation - Leadership et management : Niveau de leadership démontré par les dirigeants du secteur de la PF pour mitiger les effets de la pandémie de COVID-19 sur la PF.	◯	◯	◯	◯	◯	◯	◯	◯	◯	◯	◯

* FP M-24 Temporalité : Mesure de la rapidité de la réaction du gouvernement –sa communication et mise en œuvre des mesures- pour mitiger l’impact immédiat et sur le long terme de la pandémie de COVID-19. Cette question porte sur la riposte nationale dans son ensemble, et non la réponse spécifique de votre secteur.

	1	2	3	4	5	6	7	8	9	10	Je ne sais pas
1-Plus faible -> Plus élevé-10	◯	◯	◯	◯	◯	◯	◯	◯	◯	◯	◯

FP M-25 Commentaires : Veuillez utiliser cette section pour tout commentaire additionnel sur la résilience et la durabilité des systèmes de PF nationaux en fonction de votre expérience de la pandémie de COVID-19. Si vous avez répondu « Je ne sais pas » à l’une des questions, utilisez aussi cette section pour expliquer pourquoi.

JOHNS HOPKINS
BLOOMBERG SCHOOL
of PUBLIC HEALTH

Bill & Melinda Gates Institute for Population and Reproductive Health

Enquête de l'Indice des efforts pour le dividende démographique

Questionnaire sur la santé maternelle et infantile (SMI)

Répondez aux questions suivantes en fonction de votre expérience/ expertise dans ce secteur spécifique.

Afin d'obtenir une représentation synthétique des efforts nationaux pour le dividende démographique, notez les éléments suivants sur une échelle de 1 à 10, 1 étant le score le plus faible (efforts très faibles ou quasi-inexistants) et 10 le plus élevé (efforts robustes). Le cas échéant, si une politique ou activité n'existe pas, répondez 0.

Donnez un score à chaque élément. Toutes les réponses seront enregistrées au format illustré ci-dessous :

Effort le plus faible	1	2	3	4	5	6	7	8	9	10	Effort le plus élevé	Je ne sais pas

JOHNS HOPKINS
BLOOMBERG SCHOOL
of PUBLIC HEALTH

Bill & Melinda Gates Institute for Population and Reproductive Health

Enquête de l'Indice des efforts pour le dividende démographique

Domaine 1. Politique/ Prise de décision

* MCH 1.1 L'adéquation de la politique : Politiques du Ministère de la Santé en faveur des services pendant la grossesse et l'accouchement qui tiennent compte de toutes les conditions requises pour s'assurer que la mère et son nouveau-né survivent.

	1	2	3	4	5	6	7	8	9	10	Je ne sais pas
MCH 1.1.1 Mesure dans laquelle la santé reproductive, maternelle et infantile a été intégrée à la politique/plan/stratégie de santé nationale.											
MCH 1.1.2 Mesure dans laquelle le/les plan(s) de mise en œuvre de la/les stratégie(s) nationale(s) de santé reproductive, maternelle et infantile sont disponibles.											

* MCH 1.2 L'accès universel : Mesure dans laquelle des droits légaux et constitutionnels existent pour faciliter l'accès universel aux services de santé reproductive, maternelle et infantile.

	1	2	3	4	5	6	7	8	9	10	Je ne sais pas
1-Plus faible -> Plus élevé-10											

* MCH 1.3 L'implication de divers secteurs : Mesure dans laquelle des politiques sont élaborées à travers une consultation adéquate des parties intéressées, dont les ministères, les ONG, les praticiens privés et les associations de femmes.

	1	2	3	4	5	6	7	8	9	10	Je ne sais pas
1-Plus faible -> Plus élevé-10											

* MCH 1.4 La compétence des prestataires de santé : Mesure dans laquelle les politiques sont rationnelles et fondées sur des données probantes établissant quel personnel peut fournir des services de santé maternelle (ex : les sages-femmes formées peuvent effectuer une vaste gamme de procédures).

	1	2	3	4	5	6	7	8	9	10	Je ne sais pas
1-Plus faible -> Plus élevé-10											

* MCH 1.5 La légalisation de l’avortement : Mesure dans laquelle une politique favorable existe pour assurer l’offre légale de services d’avortement.

	1	2	3	4	5	6	7	8	9	10	Je ne sais pas
1-Plus faible -> Plus élevé-10											

* MCH 1.6. La légalisation des soins post-avortement : Mesure dans laquelle une politique favorable existe pour le traitement des complications dues aux avortements, y compris les complications résultant d’avortements clandestins.

	1	2	3	4	5	6	7	8	9	10	Je ne sais pas
1-Plus faible -> Plus élevé-10											

* MCH 1.7 La révision et mise à jour des politiques : Mesure dans laquelle les politiques sont régulièrement revues par des législateurs de haut niveau et utilisées dans les plans d’action.

	1	2	3	4	5	6	7	8	9	10	Je ne sais pas
1-Plus faible -> Plus élevé-10											

* MCH 1.8 Le haut placement des acteurs : Mesure dans laquelle le/la directeur/trice des services de santé maternelle et infantile est haut placé(e) au gouvernement et impliqué(e) dans la prise de décision.

	1	2	3	4	5	6	7	8	9	10	Je ne sais pas
1-Plus faible -> Plus élevé-10											

* MCH 1.9 Le financement de la SMI : Mesure dans laquelle les budgets du gouvernement pour la grossesse et l'accouchement sans risque, ainsi que pour les soins périnataux (pour les structures de santé, le personnel, les produits, etc.) et la santé infantile sont adaptés aux besoins, et si ces financements proviennent du Ministère de la Santé, gouvernement provincial/ des comtés ou des bailleurs de fonds.

	1	2	3	4	5	6	7	8	9	10	Je ne sais pas
1-Plus faible -> Plus élevé-10	○	○	○	○	○	○	○	○	○	○	○

* MCH 1.10 L'accessibilité financière : Mesure dans laquelle les services et médicaments liés à la SMI sont à un prix abordable pour toutes les clientes.

	1	2	3	4	5	6	7	8	9	10	Je ne sais pas
1-Plus faible -> Plus élevé-10	○	○	○	○	○	○	○	○	○	○	○

* MCH 1.11 Le secteur privé : Mesure dans laquelle le secteur privé (médecins, sages-femmes, cliniques) est actif et couvre une part importance des soins de grossesse et d'accouchement.

	1	2	3	4	5	6	7	8	9	10	Je ne sais pas
1-Plus faible -> Plus élevé-10	○	○	○	○	○	○	○	○	○	○	○

* MCH 1.12 Le directorat de SMI : Mesure dans laquelle le Ministère de la Santé a un directorat spécifiquement dédié à la SMI.

	1	2	3	4	5	6	7	8	9	10	Je ne sais pas
1-Plus faible -> Plus élevé-10	○	○	○	○	○	○	○	○	○	○	○

* MCH 1.13 Les solutions potentielles : Mesure dans laquelle le gouvernement reconnait des solutions potentielles aux problèmes de SMI par les moyens suivants :

	1	2	3	4	5	6	7	8	9	10	Je ne sais pas
MCH 1.13.1 Efficience régulatrice	◯	◯	◯	◯	◯	◯	◯	◯	◯	◯	◯
MCH 1.13.2 Performance et redevabilité	◯	◯	◯	◯	◯	◯	◯	◯	◯	◯	◯
MCH 1.13.3 Approche fondée sur le droit de la SMNI	◯	◯	◯	◯	◯	◯	◯	◯	◯	◯	◯
MCH 1.13.4 Infrastructure de santé essentielle	◯	◯	◯	◯	◯	◯	◯	◯	◯	◯	◯
MCH 1.13.5 Engagement de la communauté	◯	◯	◯	◯	◯	◯	◯	◯	◯	◯	◯

MCH 1.14 Commentaires : Utilisez cette section pour apporter des commentaires additionnels sur le niveau d'effort, les défis et les succès du pays autour de la politique/ prise de décision pour la SMI. Si vous avez répondu « Je ne sais pas » à l'une des questions précédentes, utilisez aussi cette section pour expliquer pourquoi.

JOHNS HOPKINS
BLOOMBERG SCHOOL
of PUBLIC HEALTH

Bill & Melinda Gates Institute for Population and Reproductive Health

Enquête de l'Indice des efforts pour le dividende démographique

Dimension 2. Services or Programs

* MCH 2.1 La disponibilité des soins obstétriques – Dans tous les centres : Mesure dans laquelle les structures de santé primaire ont du personnel qualifié, en place, et peuvent fournir des soins obstétriques :

	1	2	3	4	5	6	7	8	9	10	Je ne sais pas
MCH 2.1.1 Cas d'hémorragie postpartum adéquatement gérés	○	○	○	○	○	○	○	○	○	○	○
MCH 2.1.2 Administration adéquate et opportune d'antibiotiques en intraveineuse lorsque nécessaire.	○	○	○	○	○	○	○	○	○	○	○
MCH 2.1.3 Expulsion manuelle dans les cas de rétention placentaire.	○	○	○	○	○	○	○	○	○	○	○
MCH 2.1.4 Aspiration de l'utérus manuelle (Méthode de Karman) ou à l'aide d'un appareil à succion électrique.	○	○	○	○	○	○	○	○	○	○	○
MCH 2.1.5 Utilisation d'un partogramme pour savoir quand référer.	○	○	○	○	○	○	○	○	○	○	○
MCH 2.1.6 Transport organized to quickly transfer a woman with obstructed labor to the district / sub-county hospital.	○	○	○	○	○	○	○	○	○	○	○
MCH 2.1.7 Antibiotiques adéquats disponibles (suffisamment de produits du bon type).	○	○	○	○	○	○	○	○	○	○	○

* MCH 2.2 La disponibilité des soins obstétriques – Les structures de santé secondaires : Mesure dans laquelle tous les hôpitaux de districts/sous-comtés ont du personnel formé, en place, capable de :

	1	2	3	4	5	6	7	8	9	10	Je ne sais pas
MCH 2.2.1 Remplir toutes les fonctions citées ci-dessus pour les structures de santé primaires	◯	◯	◯	◯	◯	◯	◯	◯	◯	◯	◯
MCH 2.2.2 Effectuer des transfusions sanguines (et avoir un approvisionnement en sang sûr, adéquat et disponible).	◯	◯	◯	◯	◯	◯	◯	◯	◯	◯	◯
MCH 2.2.3 Effectuer des césariennes.	◯	◯	◯	◯	◯	◯	◯	◯	◯	◯	◯

* MCH 2.3 Les services liés à la grossesse : Mesure dans laquelle toutes les femmes enceintes ont accès à :

	1	2	3	4	5	6	7	8	9	10	Je ne sais pas
MCH 2.3.1 Traitement des hémorragies postpartum pendant ou peu après l’accouchement.	◯	◯	◯	◯	◯	◯	◯	◯	◯	◯	◯
MCH 2.3.2 Gestion de la dystocie.	◯	◯	◯	◯	◯	◯	◯	◯	◯	◯	◯
MCH 2.3.3 Management de la pré-éclampsie, éclampsie et ses complications.	◯	◯	◯	◯	◯	◯	◯	◯	◯	◯	◯
MCH 2.3.4 Soins post-avortement ou pour les complications liées aux avortements.	◯	◯	◯	◯	◯	◯	◯	◯	◯	◯	◯
MCH 2.3.5 Offre de services d’avortement sans risque.	◯	◯	◯	◯	◯	◯	◯	◯	◯	◯	◯
MCH 2.3.6 Soins prénatals pendant la grossesse.	◯	◯	◯	◯	◯	◯	◯	◯	◯	◯	◯
MCH 2.3.7 Soins d’accouchement par un professionnel formé.	◯	◯	◯	◯	◯	◯	◯	◯	◯	◯	◯
MCH 2.3.8 Services de planification familiale postpartum.	◯	◯	◯	◯	◯	◯	◯	◯	◯	◯	◯
MCH 2.3.9 Hôpitaux des districts/ sous-comtés ouverts 24h/24 et 7j/7.	◯	◯	◯	◯	◯	◯	◯	◯	◯	◯	◯

* MCH 2.4 Les soins prénatals pour les femmes non mariées et séropositives : Mesure dans laquelle, à toutes les consultations prénatales, toutes les femmes enceintes :

	1	2	3	4	5	6	7	8	9	10	Je ne sais pas
MCH 2.4.1 Reçoivent des soins prénatals complets conformément aux protocoles recommandés par l'OMS pour les femmes séronégatives.	◯	◯	◯	◯	◯	◯	◯	◯	◯	◯	◯
MCH 2.4.2 Reçoivent des soins prénatals complets conformément aux protocoles recommandés par l'OMS pour les femmes séropositives.	◯	◯	◯	◯	◯	◯	◯	◯	◯	◯	◯
MCH 2.4.3 Reçoivent des soins prénatals complets conformément aux protocoles recommandés par l'OMS pour les jeunes femmes non mariées.	◯	◯	◯	◯	◯	◯	◯	◯	◯	◯	◯
MCH 2.4.4 Sont informées des signes de danger de complications obstétriques et néonatales, et sont assistées pour planifier toute urgence éventuelle.	◯	◯	◯	◯	◯	◯	◯	◯	◯	◯	◯

* MCH 2.5 Les soins néonatals : Mesure dans laquelle, pour les soins néonatals, tous les nouveau-nés, qu'ils soient nés à la maison ou en structure médicale :

	1	2	3	4	5	6	7	8	9	10	Je ne sais pas
MCH 2.5.1 Ont leur bouche et voie nasale dégagées.	○	○	○	○	○	○	○	○	○	○	○
MCH 2.5.2 Sont séchés et gardés au chaud immédiatement après la naissance.	○	○	○	○	○	○	○	○	○	○	○
MCH 2.5.3 Reçoivent de la vitamine A.	○	○	○	○	○	○	○	○	○	○	○
MCH 2.5.4 Ont leur cordon ombilical coupé avec une lame propre.	○	○	○	○	○	○	○	○	○	○	○
MCH 2.5.5 Suivent le calendrier de vaccination recommandé par l'OMS.	○	○	○	○	○	○	○	○	○	○	○
MCH 2.5.6 Bénéficient des activités de promotion de l'allaitement exclusif.	○	○	○	○	○	○	○	○	○	○	○
MCH 2.5.7 Bénéficient des programmes de soutien alimentaire lorsqu'ils en ont besoin.	○	○	○	○	○	○	○	○	○	○	○

* MCH 2.6 La planification familiale – Dans tous les centres : Mesure dans laquelle la planification familiale est dûment fournie dans tous les centres de santé en :

	1	2	3	4	5	6	7	8	9	10	Je ne sais pas
MCH 2.6.1 Offrant régulièrement des services de planification familiale aux cas post-avortement.	○	○	○	○	○	○	○	○	○	○	○
MCH 2.6.2 Offrant régulièrement des services de planification familiale lors des consultations postpartum.	○	○	○	○	○	○	○	○	○	○	○
MCH 2.6.3 Ayant des méthodes contraceptives réversibles de courte et longue durée régulièrement en stock.	○	○	○	○	○	○	○	○	○	○	○
MCH 2.6.4 Ayant du personnel formé, sur place, pouvant fournir les méthodes LARC.	○	○	○	○	○	○	○	○	○	○	○
MCH 2.6.5 Suivant les directives et protocoles nécessaires à la prestation de services de planification familiale.	○	○	○	○	○	○	○	○	○	○	○

* MCH 2.7 La planification familiale – Mesure dans laquelle la planification familiale est dûment fournie dans tous hôpitaux de districts/sous-comtés en :

	1	2	3	4	5	6	7	8	9	10	Je ne sais pas
MCH 2.7.1 Offrant régulièrement des services de planification familiale aux cas post-avortement.	○	○	○	○	○	○	○	○	○	○	○
MCH 2.7.2 Offrant régulièrement des services de planification familiale lors des consultations postpartum.	○	○	○	○	○	○	○	○	○	○	○
MCH 2.7.3 Ayant des méthodes contraceptives réversibles de courte et longue durée régulièrement en stock.	○	○	○	○	○	○	○	○	○	○	○
MCH 2.7.4 Ayant du personnel formé, sur place, pouvant fournir les méthodes LARC.	○	○	○	○	○	○	○	○	○	○	○
MCH 2.7.5 Pouvant offrir la stérilisation féminine.	○	○	○	○	○	○	○	○	○	○	○
MCH 2.7.6 Pouvant offrir la stérilisation masculine.	○	○	○	○	○	○	○	○	○	○	○
MCH 2.7.7 Suivant les directives et protocoles nécessaires à la prestation de services de planification familiale.	○	○	○	○	○	○	○	○	○	○	○

* MCH 2.8 Les maladies infantiles : Mesure dans laquelle la gestion de maladies infantiles recommandée par l'OMS est mise en œuvre en termes de :

	1	2	3	4	5	6	7	8	9	10	Je ne sais pas
MCH 2.8.1 Gestion à base communautaire des maladies infantiles.	○	○	○	○	○	○	○	○	○	○	○
MCH 2.8.2 Efficacité des services de référence du niveau communautaire vers les structures de santé.	○	○	○	○	○	○	○	○	○	○	○

MCH 2.9 Commentaires : Utilisez cette section pour apporter des commentaires additionnels sur le niveau d’effort, les défis et les succès du pays autour des programmes ou services de SMI. Si vous avez répondu « Je ne sais pas » à l’une des questions précédentes, utilisez aussi cette section pour expliquer pourquoi.

JOHNS HOPKINS
BLOOMBERG SCHOOL
of PUBLIC HEALTH

Bill & Melinda Gates Institute for Population and Reproductive Health

Enquête de l’Indice des efforts pour le dividende démographique

Domaine 3. Le plaidoyer

* MCH 3.1 Les messages du gouvernement : Mesure dans laquelle les hauts fonctionnaires au gouvernement, dont au Ministère de la Santé, s’adressent fréquemment à toutes les parties prenantes, y compris la presse, pour soutenir des améliorations et promouvoir les grossesses et les accouchements sans risque, et les enfants en bonne santé.

	1	2	3	4	5	6	7	8	9	10	Je ne sais pas
1-Plus faible -> Plus élevé-10	○	○	○	○	○	○	○	○	○	○	○

* MCH 3.2 Les médias de masse : Mesure dans laquelle le programme national utilise les médias de masse pour éduquer le public sur les symptômes des complications liées aux grossesses, sur les accouchements sans risque et les enfants en bonne santé.

	1	2	3	4	5	6	7	8	9	10	Je ne sais pas
1-Plus faible -> Plus élevé-10	○	○	○	○	○	○	○	○	○	○	○

* MCH 3.3 Les médias au niveau communautaire : Mesure dans laquelle les groupes communautaires prennent part aux programmes systématiques pour éduquer le public sur les grossesses et accouchements sans risque et les enfants en bonne santé.

	1	2	3	4	5	6	7	8	9	10	Je ne sais pas
1-Plus faible -> Plus élevé-10	○	○	○	○	○	○	○	○	○	○	○

* MCH 3.4 Les supports éducatifs : Mesure dans laquelle le ministère compétent (Ministère de la Santé) fournit des supports éducatifs adéquats (posters, brochures, etc.) aux structures de santé pour qu'elles instruisent les client(e)s sur les pratiques sûres/sans risque.

	1	2	3	4	5	6	7	8	9	10	Je ne sais pas
1-Plus faible -> Plus élevé-10	◯	◯	◯	◯	◯	◯	◯	◯	◯	◯	◯

* MCH 3.5 Auto-révision des cas de mortalité maternelle : Mesure dans laquelle chaque structure de santé suit une procédure régulière de révision de tous ses cas de décès maternels ayant eu lieu dans la structure pour en tirer des leçons.

	1	2	3	4	5	6	7	8	9	10	Je ne sais pas
1-Plus faible -> Plus élevé-10	◯	◯	◯	◯	◯	◯	◯	◯	◯	◯	◯

* MCH 3.6 Informer les politiques avec de nouvelles données probantes : Mesure dans laquelle chaque structure de santé transmet les leçons tirées des cas de décès/ mortalité maternelle aux ministères pour informer les nouvelles politiques et interventions.

	1	2	3	4	5	6	7	8	9	10	Je ne sais pas
1-Plus faible -> Plus élevé-10	◯	◯	◯	◯	◯	◯	◯	◯	◯	◯	◯

MCH 3.7 Commentaires : Utilisez cette section pour apporter des commentaires additionnels sur le niveau d'effort, les défis et les succès du pays autour du plaidoyer pour la SMI. Si vous avez répondu « Je ne sais pas » à l'une des questions précédentes, utilisez aussi cette section pour expliquer pourquoi.

JOHNS HOPKINS
BLOOMBERG SCHOOL
of PUBLIC HEALTH

Bill & Melinda Gates Institute for Population and Reproductive Health

Enquête de l'Indice des efforts pour le dividende démographique

Domaine 4. Recherche

* MCH 4.1 La force des observatoires/parties prenantes/groupes de travail pour la SMI : Mesure dans laquelle les parties prenantes de la SMI du pays (ex : groupe directif de parties prenantes, groupes de travail techniques, observatoire de SMI, groupe de coordination ou facilitation) :

	1	2	3	4	5	6	7	8	9	10	Je ne sais pas
MCH 4.1.1 Ont un représentant du gouvernement, des institutions de formation, de la société civile, des organisations non gouvernementales et confessionnelles, des associations professionnelles, du secteur privé, etc.	○	○	○	○	○	○	○	○	○	○	○
MCH 4.1.2 Se réunissent régulièrement, émettent des rapports et recommandent des politiques aux directions des ministères compétents.	○	○	○	○	○	○	○	○	○	○	○
MCH 4.1.3 Ont un impact sur l'éducation dans le pays.	○	○	○	○	○	○	○	○	○	○	○

* MCH 4.2 MCH La stratégie d'éducation/recherche : Mesure dans laquelle le plan national de SMI et/ou d'autres documents nationaux comprennent une stratégie/approche complète de recherche liée sur la SMI et est partagée entre partenaires.

	1	2	3	4	5	6	7	8	9	10	Don't know
1-Plus faible -> Plus élevé-10	○	○	○	○	○	○	○	○	○	○	○

* MCH 4.3 Les partenaires collectant les données:

	1	2	3	4	5	6	7	8	9	10	Je ne sais pas
MCH 4.3.1 Mesure dans laquelle la collecte des données est entreprise par des agences gouvernementales. Cela peut comprendre les agences/bureaux de la statistique et/ou les ministères de l'économie/finances, éducation, santé, genre, etc.).	○	○	○	○	○	○	○	○	○	○	○
MCH 4.3.2 Mesure dans laquelle la collecte de données est entreprise par des institutions de recherche.	○	○	○	○	○	○	○	○	○	○	○
MCH 4.3.3 Mesure dans laquelle la collecte des données est entreprise par des chercheurs indépendants.	○	○	○	○	○	○	○	○	○	○	○

* MCH 4.4 La recherche thématique :

	1	2	3	4	5	6	7	8	9	10	Je ne sais pas
MCH 4.4.1 Mesure dans laquelle de la recherche est menée sur la mortalité maternelle.	○	○	○	○	○	○	○	○	○	○	○
MCH 4.4.2 Mesure dans laquelle de la recherche est menée sur la mortalité néonatale et infantile.	○	○	○	○	○	○	○	○	○	○	○
MCH 4.4.3 Mesure dans laquelle de la recherche est menée sur la planification familiale et la planification familiale postpartum.	○	○	○	○	○	○	○	○	○	○	○
MCH 4.4.4 Mesure dans laquelle de la recherche est menée sur la prestation/accès aux services et la qualité des prestataires de SMI.	○	○	○	○	○	○	○	○	○	○	○

* MCH 4.5 La qualité/couverture des données : Mesure dans laquelle la recherche/les données actuelles sont désagrégées par sexe, âge, lieu, ethnicité, niveau d'études, quintile de revenus et statut d'handicap. Mesure dans laquelle le pays participe à des tests standardisés et son classement.

	1	2	3	4	5	6	7	8	9	10	Je ne sais pas
MCH 4.5.1 Mesure dans laquelle un système statistique de routine offre de bonnes données périodiques sur les produits/l'approvisionnement et les structures de santé.	○	○	○	○	○	○	○	○	○	○	○
MCH 4.5.2 Mesure dans laquelle un système statistique de routine offre de bonnes données périodiques sur les services et le personnel de SMI.	○	○	○	○	○	○	○	○	○	○	○
MCH 4.5.3 Mesure dans laquelle un système statistique de routine offre de bonnes données périodiques sur les besoins en SMI de différentes populations dans les communautés.	○	○	○	○	○	○	○	○	○	○	○
MCH 4.5.4 4 Mesure dans laquelle des évaluations du système de suivi de l'éducation sont menées et appliquées pour assurer la fiabilité des données.	○	○	○	○	○	○	○	○	○	○	○
MCH 4.5.5 Mesure dans laquelle des évaluations du système de suivi de l'éducation sont menées et appliquées pour explorer les inégalités dans les services et fardeaux de la SMNI.	○	○	○	○	○	○	○	○	○	○	○

* MCH 4.6 Les registres : Mesure dans laquelle les systèmes de registres, rapports et feedback sur les résultats de SMI sont adéquats, y compris en étant connectés à tous les niveaux (fédéral, état/province, comté, etc.).

	1	2	3	4	5	6	7	8	9	10	Je ne sais pas
1-Plus faible -> Plus élevé-10	○	○	○	○	○	○	○	○	○	○	○

* MCH 4.7 La qualité des institutions de recherche : Mesure dans laquelle le pays a la capacité de soutenir et maintenir des institutions de recherche qui développent des études et/ou collectent des données permettant des services SMI de haute qualité.

	1	2	3	4	5	6	7	8	9	10	Je ne sais pas
1-Plus faible -> Plus élevé-10											

* MCH 4.8 L'évaluation : Mesure dans laquelle les statistiques, enquêtes et études sur le programme de SMI sont utilisées par du personnel spécialisé pour rapporter les opérations du programme et mesurer le progrès.

	1	2	3	4	5	6	7	8	9	10	Je ne sais pas
1-Plus faible -> Plus élevé-10											

* MCH 4.9 L'utilisation des résultats d'évaluations par les gestionnaires de programmes : Mesure dans laquelle les gestionnaires des programme locaux utilisent les conclusions de recherche et d'évaluations pour améliorer ces programmes en suivant les recommandations émises.

	1	2	3	4	5	6	7	8	9	10	Je ne sais pas
1-Plus faible -> Plus élevé-10											

* MCH 4.10 L'utilisation des résultats d'évaluation par les ministères : Mesure dans laquelle les administrateurs ministériels compétents et responsables d'élaborer les politiques utilisent systématiquement les données pour informer les politiques et interventions visant à résoudre les problèmes liés à la SMI.

	1	2	3	4	5	6	7	8	9	10	Je ne sais pas
1-Plus faible -> Plus élevé-10											

* MCH 4.11 La dissémination d'informations à d'autres entités chargées de la mise en œuvre des programmes : Mesure dans laquelle les informations sont communiquées ou disséminées entre différentes aires géographiques et à différents niveaux (national, état/province, collectivités territoriales).

	1	2	3	4	5	6	7	8	9	10	Je ne sais pas
1-Plus faible -> Plus élevé-10											

MCH 4.12 Commentaires : Utilisez cette section pour apporter des commentaires additionnels sur le niveau d'effort, les défis et les succès du pays autour de la recherche sur la SMI. Si vous avez répondu « Je ne sais pas » à l'une des questions précédentes, utilisez aussi cette section pour expliquer pourquoi.

JOHNS HOPKINS
BLOOMBERG SCHOOL
of PUBLIC HEALTH

Bill & Melinda Gates Institute for Population and Reproductive Health

Enquête de l’Indice des efforts pour le dividende démographique

Domaine 5. Organisations de la société civile (OSC)

* MCH 5.1 Le pouvoir des acteurs des OSC : Mesure dans laquelle les acteurs des OSC occupent des postes d’influence dans les institutions, les groupes de travail techniques ou les réseaux concernés par la SMI.

	1	2	3	4	5	6	7	8	9	10	Je ne sais pas
1-Plus faible -> Plus élevé-10	○	○	○	○	○	○	○	○	○	○	○

* MCH 5.2 L’analyse budgétaire comme outil des OSC : Mesure dans laquelle les OSC utilisent l’analyse budgétaire comme un outil pour développer le plaidoyer en matière de SMI.

	1	2	3	4	5	6	7	8	9	10	Je ne sais pas
1-Plus faible -> Plus élevé-10	○	○	○	○	○	○	○	○	○	○	○

* MCH 5.3 Le soutien à la prestation de services en structure sanitaire : Mesure dans laquelle les OSC sont impliquées dans des efforts visant à augmenter l’utilisation et/ou la qualité des services, particulièrement à travers l’augmentation des capacités techniques et/ou opérationnelles des cliniques partenaires des OSC afin de fournir des services de SMI.

	1	2	3	4	5	6	7	8	9	10	Je ne sais pas
1-Plus faible -> Plus élevé-10	○	○	○	○	○	○	○	○	○	○	○

* MCH 5.4 Le soutien à la prestation des services à base communautaire : Mesure dans laquelle les OSC participent au rapprochement des informations et services de SMI avec les communautés à travers des activités comprenant le renforcement de compétences des agents de santé communautaire (ASC), l'expansion de la gamme de services SMI offerts par les ASC, l'orientation/éducation des travailleurs de la santé de cadres inférieurs et/ou des campagnes d'information ou d'éducation.

	1	2	3	4	5	6	7	8	9	10	Je ne sais pas
1-Plus faible -> Plus élevé-10	○	○	○	○	○	○	○	○	○	○	○

* MCH 5.5 Le soutien à la sensibilisation mobile pour l'offre de services à base communautaire : Mesure dans laquelle les OSC soutiennent les modèles de sensibilisation mobile pour équiper les prestataires de santé en leur fournissant des commodités et produits liés à la SMI, de l'équipement et des véhicules afin de fournir en tant qu'équipe spécialiste des services SMI directement aux communautés.

	1	2	3	4	5	6	7	8	9	10	Je ne sais pas
1-Plus faible -> Plus élevé-10	○	○	○	○	○	○	○	○	○	○	○

* MCH 5.6 La franchise sociale : Mesure dans laquelle les OSC sont impliquées dans des opportunités/services de franchise sociale pour offrir des services de SMI dans le secteur privé à un prix réduit.

	1	2	3	4	5	6	7	8	9	10	Je ne sais pas
1-Plus faible -> Plus élevé-10	○	○	○	○	○	○	○	○	○	○	○

* MCH 5.7 La santé mobile : Mesure dans laquelle les OSC mettent à profit la technologie mobile en utilisant la messagerie (textos) et autres technologies mobiles pour améliorer l'accès aux informations et services de SMI.

	1	2	3	4	5	6	7	8	9	10	Je ne sais pas
1-Plus faible -> Plus élevé-10	○	○	○	○	○	○	○	○	○	○	○

* MCH 5.8 Les droits humains et la qualité des soins : Mesure dans laquelle les OSC soutiennent et plaident pour des approches fondées sur le droit pour s'assurer que les clientes de la SMI soient bien traitées, n'aient pas peur de poser des questions et reçoivent du conseil de qualité, entre autres sujets.

	1	2	3	4	5	6	7	8	9	10	Je ne sais pas
1-Plus faible -> Plus élevé-10	○	○	○	○	○	○	○	○	○	○	○

* MCH 5.9 Le changement social et des comportements (CSC) : Mesure dans laquelle les OSC soutiennent les interventions pour le CSC comprenant, mais sans s'y limiter, les compétences de vie et l'éducation entre pairs, l'éducation sur la santé de l'adulte, l'éducation en milieu scolaire, la communication au sein du couple, la mobilisation communautaire, les interventions à travers les gardiens de la communauté et/ou dans les médias de masse pour le grand public.

	1	2	3	4	5	6	7	8	9	10	Je ne sais pas
1-Plus faible -> Plus élevé-10	○	○	○	○	○	○	○	○	○	○	○

* MCH 5.10 Le plaidoyer/ la redevabilité : Mesure dans laquelle les OSC soutiennent la formation et le renforcement de compétences des plaideurs et des membres de la communauté pour améliorer leur compréhension des politiques et processus, leurs activités d'identification de problèmes, la résolution collaborative de ces problèmes, et des actions de plaidoyer plus ciblées pour améliorer la SMI.

	1	2	3	4	5	6	7	8	9	10	Je ne sais pas
1-Plus faible -> Plus élevé-10	○	○	○	○	○	○	○	○	○	○	○

* MCH 5.11 L'évaluation et le suivi dirigés par les OSC : Mesure dans laquelle les OSC évaluent, suivent et émettent des rapports sur l'efficacité des politiques et programmes pour améliorer la redevabilité des prestataires et des politiques de SMI.

	1	2	3	4	5	6	7	8	9	10	Je ne sais pas
1-Plus faible -> Plus élevé-10	○	○	○	○	○	○	○	○	○	○	○

* MCH 5.12 Les partenariats entre OSC : Mesure dans laquelle les OSC ont formé des alliances nationales et des partenariats régionaux pour renforcer leur position et potentiellement améliorer la force du leadership et les mécanismes de financement pour la SMI.

	1	2	3	4	5	6	7	8	9	10	Je ne sais pas
1-Plus faible -> Plus élevé-10	○	○	○	○	○	○	○	○	○	○	○

MCH 5.13 Commentaires : Utilisez cette section pour apporter des commentaires additionnels sur le niveau d'effort, les défis et les succès des OSC travaillant pour la SMI. Si vous avez répondu « Je ne sais pas » à l'une des questions précédentes, utilisez aussi cette section pour expliquer pourquoi.

JOHNS HOPKINS BLOOMBERG SCHOOL of PUBLIC HEALTH

Bill & Melinda Gates Institute for Population and Reproductive Health

Enquête de l'Indice des efforts pour le dividende démographique

Module : Résilience et durabilité du secteur

Étant donné l'impact sanitaire et socioéconomique de la pandémie de COVID-19, et son impact probable sur la progression vers le DD, cet indice des efforts pour le DD intègre des questions pour évaluer la résilience et la durabilité des systèmes dans les secteurs clés du DD. Les éléments couverts par les questions liées à la pandémie de COVID-19 ci-dessous relèvent des dimensions clés des systèmes résilients et visent à apporter des informations déterminantes sur le potentiel d'une réponse efficace aux menaces de maladies infectieuses émergeantes et autres crises de santé publique.

Veuillez noter chaque élément sur une échelle de 1 à 10, 1 étant le score le plus faible (faible état/capacité) et 10 le plus élevé (très bon état/capacité).

* Dimension physique : structures, équipements, états du système et capacités.

	1	2	3	4	5	6	7	8	9	10	Je ne sais pas
MCH-M1 - Planification/Préparation – État et capacité de l'équipement, du personnel et de la structure du programme de SMI avant la crise.	○	○	○	○	○	○	○	○	○	○	○
MCH-M2 – Absorption – Intégration : Niveau d'intégration des services et programmes de SMI pour mitiger les effets de la pandémie de COVID-19 sur la SMI.	○	○	○	○	○	○	○	○	○	○	○

	1	2	3	4	5	6	7	8	9	10	Je ne sais pas
MCH-M3 - Absorption – Intégration : Mesure dans laquelle la gestion efficace de la chaîne d’approvisionnement a permis de mitiger les effets de la pandémie de COVID-19 sur l’approvisionnement/livraison des commodités liées à la SMI.											
MCH-M4 - Récupération – Mesure dans laquelle le programme de SMI a été capable d’induire des changements pour récupérer un niveau de fonctionnalité pre-COVID-19.											
MCH-M5 - Récupération – Gestion du financement et des bailleurs de fonds : Niveau de mobilisation de ressources et d’allocation à la mitigation des effets de la pandémie de COVID-19 sur la SMI.											
MCH-M6 - Adaptation – Mesure dans laquelle des changements ont été effectués pour améliorer la résilience du secteur SMI.											

* Dimension informatique : création, gestion, stockage et utilisation des données.

	1	2	3	4	5	6	7	8	9	10	Don't know
MCH-M7 - Planification/Préparation - Mesure dans laquelle les données liées à la SMI étaient suffisamment préparées, présentées, analysées et stockées avant la crise.	○	○	○	○	○	○	○	○	○	○	○
MCH-M8 - Absorption – Connaissance/Conscience : Mesure dans laquelle la capacité de surveillance a pu détecter les limites/menaces posées par la pandémie de COVID-19 à la SMI.	○	○	○	○	○	○	○	○	○	○	○
MCH-M9 - Récupération – Mesure dans laquelle les données de SMI ont été utilisées pour suivre le progrès vers la récupération et anticiper des scenarii de récupération.	○	○	○	○	○	○	○	○	○	○	○
MCH-M10 - Adaptation – Mesure dans laquelle le système de SMI crée et améliore actuellement ses protocoles de stockage et d’utilisation de ses données en temps réel.	○	○	○	○	○	○	○	○	○	○	○

* Dimension cognitive: compréhension, modèles cognitifs, préconception, biais et valeurs.

	1	2	3	4	5	6	7	8	9	10	Je ne sais pas
MCH-M11 - Planification/Préparation - Mesure dans laquelle les décisions de conceptualisation et opérationnelle du secteur SMI étaient préparées à anticiper la crise.	○	○	○	○	○	○	○	○	○	○	○
MCH-M12 - Absorption - Mesure dans laquelle le secteur de la SMI a des protocoles de contingence suffisants et une gestion de crise proactive.	○	○	○	○	○	○	○	○	○	○	○

	1	2	3	4	5	6	7	8	9	10	Je ne sais pas
MCH-M13 - Absorption - Redevabilité: Niveau d'effort pour assurer une redevabiilté optimale vis-à-vis des ressources allouées en soutien à la SMI dans le cadre de la réponse à la pandémie de COVID-19.	○	○	○	○	○	○	○	○	○	○	○
MCH-M14 - Récupération - Mesure dans laquelle les décisions sont orientées vers la récupération, et communiquées sur la base de données probantes pour promouvoir des comportements sûrs auprès des membres de la communauté.	○	○	○	○	○	○	○	○	○	○	○
MCH-M15 - Récupération - Connaissance/Conscience: Mesure dans laquelle le secteur SMI établit une certaine confiance à travers ses communications concernant les barrières posées par la pandémie de COVID-19 pour soutenir l'accès et l'utilisation des services et programmes de SMI.	○	○	○	○	○	○	○	○	○	○	○
MCH-M16 - Adaptation – Adaptif : Mesure dans laquelle les dirigeants nationaux avaient l'autorité d'effectuer des changements pour s'adapter et répondre aux menaces posées par la pandémie de COVID-19 aux services et programmes de SMI.	○	○	○	○	○	○	○	○	○	○	○

* Dimension sociale : interaction, collaboration et auto-synchronisation entre les personnes, les entités et les institutions.

	1	2	3	4	5	6	7	8	9	10	Je ne sais pas
MCH-M17 - Planification/Préparation – Mesure dans laquelle une formation sur la gestion de crise/épidémie a été menée et a permis de mettre à profit les réseaux sociaux, le capital social et institutionnel, et les normes culturelles avant la crise.	○	○	○	○	○	○	○	○	○	○	○
MCH- M18 - Absorption – Mesure dans laquelle le personnel et les institutions sociales ont fait preuve d'ingéniosité et ont été accessibles pendant la réponse à l'épidémie/crise.	○	○	○	○	○	○	○	○	○	○	○
MCH-M19 - Absorption – Autorégulation : Mesure dans laquelle les dirigeants nationaux avaient l'autorité d'effectuer des changements sectoriels en temps opportun à travers une infrastructure flexible.	○	○	○	○	○	○	○	○	○	○	○
MCH-M20 - Récupération – Mesure dans laquelle le secteur SMI est engagé dans le partage de connaissances et d'équipes pour améliorer la récupération systémique.	○	○	○	○	○	○	○	○	○	○	○
MCH-M21 - Récupération – Diversité : Niveau d'engagement d'une équipe multidisciplinaire pour mitiger les effets de la pandémie de COVID-19 sur la SMI.	○	○	○	○	○	○	○	○	○	○	○

	1	2	3	4	5	6	7	8	9	10	Je ne sais pas
MCH-M22 - Adaptation – Mesure dans laquelle des ajouts ou modifications ont été apportés aux institutions, politiques, programmes, formations et cultures de SMI.	○	○	○	○	○	○	○	○	○	○	○
MCH-M23 - Adaptation - Leadership et management : Niveau de leadership démontré par les dirigeants de la SMI pour mitiger les effets de la pandémie de COVID-19 sur la SMI.	○	○	○	○	○	○	○	○	○	○	○

* MCH M-24 Temporalité : Mesure de la rapidité de la réaction du gouvernement –sa communication et mise en œuvre des mesures- pour mitiger l'impact immédiat et sur le long terme de la pandémie de COVID-19. Cette question porte sur la riposte nationale dans son ensemble, et non la réponse spécifique de votre secteur.

	1	2	3	4	5	6	7	8	9	10	Je ne sais pas
1-Plus faible -> Plus élevé-10	○	○	○	○	○	○	○	○	○	○	○

MCH M-25 Commentaires : Utilisez cette section pour apporter tout commentaire additionnel sur la résilience et la durabilité du système nationaux de SMI en vous référant à votre expérience de la pandémie de COVID-19. Si vous avez répondu « Je ne sais pas » à l'une des questions, utilisez cette section pour expliquer pourquoi

JOHNS HOPKINS
BLOOMBERG SCHOOL
of PUBLIC HEALTH

Bill & Melinda Gates Institute for Population and Reproductive Health

Enquête de l’Indice des efforts pour le dividende démographique

Questionnaire sur l’éducation (ED)

Répondez aux questions suivantes en fonction de votre expérience/ expertise dans ce secteur spécifique.

Afin d’obtenir une représentation synthétique des efforts nationaux pour le dividende démographique, notez les éléments suivants sur une échelle de 1 à 10, 1 étant le score le plus faible (efforts très faibles ou quasi-inexistants) et 10 le plus élevé (efforts robustes). Le cas échéant, si une politique ou activité n’existe pas, répondez 0.

Donnez un score à chaque élément. Toutes les réponses seront enregistrées au format illustré ci-dessous :

Effort le plus faible	1	2	3	4	5	6	7	8	9	10	Effort le plus élevé	Je ne sais pas

JOHNS HOPKINS
BLOOMBERG SCHOOL
of PUBLIC HEALTH

Bill & Melinda Gates Institute for Population and Reproductive Health

Enquête de l'Indice des efforts pour le dividende démographique

Domaine 1. Politique/ Prise de décision

* ED 1.1 La qualité : Mesure dans laquelle il existe un plan/stratégie/programme pour améliorer la qualité de l'enseignement en milieu scolaire.

	1	2	3	4	5	6	7	8	9	10	Je ne sais pas
1-Plus faible -> Plus élevé - 10	○	○	○	○	○	○	○	○	○	○	○

* ED 1.2 Les groupes défavorisés :

	1	2	3	4	5	6	7	8	9	10	Je ne sais pas
ED 1.2.1. Mesure dans laquelle les stratégies ont été développées et mises en œuvre/déployées pour surmonter les obstacles d'apprentissage chez les filles enceintes et les mères adolescentes	○	○	○	○	○	○	○	○	○	○	○
ED 1.2.2 Mesure dans laquelle des stratégies ont été développées et mises en œuvre/déployées pour surmonter les obstacles d'apprentissage chez les personnes souffrant d'un handicap	○	○	○	○	○	○	○	○	○	○	○

* ED 1.3 Les enseignants : Mesure dans laquelle les problèmes liés au recrutement, développement et management du personnel enseignant sont inclus à la politique visant à améliorer les résultats d’apprentissage.

	1	2	3	4	5	6	7	8	9	10	Je ne sais pas
1-Plus faible->Plus élevé-10	○	○	○	○	○	○	○	○	○	○	○

* ED 1.4 La redevabilité des enseignants : Mesure dans laquelle les plans nationaux incluent des mesures spécifiques, dont le suivi et évaluation, visant à rendre les enseignants redevables de l’amélioration de l’apprentissage.

	1	2	3	4	5	6	7	8	9	10	Je ne sais pas
1-Plus faible -> Plus élevé - 10	○	○	○	○	○	○	○	○	○	○	○

* ED 1.5 La qualité des enseignants : Mesure dans laquelle les politiques/stratégies/programmes d’éducation incluent des indicateurs vérifiables de la qualité des enseignants.

	1	2	3	4	5	6	7	8	9	10	Je ne sais pas
1-Plus faible -> Plus élevé - 10	○	○	○	○	○	○	○	○	○	○	○

* ED 1.6 La collaboration dans l’éducation : Mesure dans laquelle la politique prévoit des dispositions pour engager plusieurs parties prenantes dans l’éducation (ex : écoles, communautés du milieu scolaire, parents, enseignants, gouvernement local, associations de parents d’élèves-élèves-enseignants, etc.).

	1	2	3	4	5	6	7	8	9	10	Je ne sais pas
1-Plus faible -> Plus élevé - 10	○	○	○	○	○	○	○	○	○	○	○

* ED 1.7 L’ODD sur la parité : Mesure dans laquelle les politiques/plans/stratégies nationales tiennent compte de l’Objectif de Développement Durable (ODD) sur la parité totale à tous les niveaux d’enseignement.

	1	2	3	4	5	6	7	8	9	10	Je ne sais pas
1-Plus faible -> Plus élevé - 10	○	○	○	○	○	○	○	○	○	○	○

* ED 1.8 Le financement de l’éducation : Mesure dans laquelle les nouveaux plans d’éducation incluent des propositions financières avec des budgets alloués aux réformes de l’enseignement et de l’apprentissage.

	1	2	3	4	5	6	7	8	9	10	Je ne sais pas
1-Plus faible -> Plus élevé - 10	○	○	○	○	○	○	○	○	○	○	○

* ED 1.9 La planification de la parité : Mesure dans laquelle il existe des plans/stratégies nationales pour atteindre 100% d'inscriptions avec parité en :

	1	2	3	4	5	6	7	8	9	10	Je ne sais pas
ED 1.9.1 Ecole primaire	○	○	○	○	○	○	○	○	○	○	○
ED 1.9.2 Ecole secondaire	○	○	○	○	○	○	○	○	○	○	○
ED 1.9.3 Ecole tertiaire/supérieure	○	○	○	○	○	○	○	○	○	○	○

* ED 1.10 L'analphabétisme chez l'adulte : Mesure dans laquelle des politiques/plans/stratégies nationales visent à répondre à l'analphabétisme.

	1	2	3	4	5	6	7	8	9	10	Je ne sais pas
ED 1.10.1 Mesure dans laquelle les politiques/plans/stratégies nationales visent à répondre à l'analphabétisme chez l'adulte.	○	○	○	○	○	○	○	○	○	○	○
ED 1.10.2 Mesure dans laquelle les politiques/plans/stratégies nationales visant à répondre à l'analphabétisme chez l'adulte visent aussi à résorber les inégalités entre les sexes.	○	○	○	○	○	○	○	○	○	○	○

* ED 1.11 Les enfants déscolarisés : Mesure dans laquelle des politiques répondent à la détresse des enfants déscolarisés pour les réintégrer au système scolaire ou leur offrir des opportunités alternatives de développement éducatif.

	1	2	3	4	5	6	7	8	9	10	Je ne sais pas
1-Plus faible -> Plus élevé - 10	○	○	○	○	○	○	○	○	○	○	○

* ED 1.12 L'intégration de la technologie : Mesure dans laquelle des stratégies ont été développées et mises en œuvre/déployées pour intégrer les technologies de l'information et de la communication (TIC) à l'éducation.

	1	2	3	4	5	6	7	8	9	10	Je ne sais pas
1-Plus faible -> Plus élevé - 10	○	○	○	○	○	○	○	○	○	○	○

* ED 1.13. La maîtrise des technologies : Mesure dans laquelle des ressources ont été allouées à l’amélioration de la maîtrise de l’informatique à tous les niveaux d’enseignement.

	1	2	3	4	5	6	7	8	9	10	Je ne sais pas
1-Plus faible -> Plus élevé - 10	◯	◯	◯	◯	◯	◯	◯	◯	◯	◯	◯

ED 1.14. Commentaires : Utilisez cette section pour nous faire part de tout commentaire additionnel sur le niveau d’effort, les défis ou les succès du pays autour des politiques/ de la prise de décision dans le secteur de l’éducation. Si vous avez répondu « Je ne sais pas » à l’une des questions précédentes, veuillez utiliser cette section pour expliquer pourquoi.

JOHNS HOPKINS
BLOOMBERG SCHOOL
of PUBLIC HEALTH

Bill & Melinda Gates Institute for Population and Reproductive Health

Enquête de l'Indice des efforts pour le dividende démographique

Domaine 2. Services ou programmes

* ED 2.1 Le déploiement des enseignants : Mesure dans laquelle des stratégies/plans sont utilisés pour veiller à ce que les enseignants soient déployés dans les endroits du pays qui en ont le plus besoin.

	1	2	3	4	5	6	7	8	9	10	Je ne sais pas
1-Plus faible -> Plus élevé-10	○	○	○	○	○	○	○	○	○	○	○

* ED 2.2 La qualité des enseignants : Mesure dans laquelle la qualité des enseignants est reconnue comme un facteur clé pour améliorer les résultats d'apprentissage.

	1	2	3	4	5	6	7	8	9	10	Je ne sais pas
ED 2.2.1 Mesure dans laquelle la qualité de l'enseignement est soulignée dans les politiques/ plans/ stratégies éducatives plus générales.	○	○	○	○	○	○	○	○	○	○	○
ED 2.2.2 Mesure dans laquelle la qualité de la formation et du développement professionnel continu des enseignants est soulignée dans les politiques/ plans/ stratégies éducatives plus générales.	○	○	○	○	○	○	○	○	○	○	○

* ED 2.3 La parité : Mesure dans laquelle les ressources ont été utilisées pour améliorer la parité dans l'éducation entre les garçons et les filles.

	1	2	3	4	5	6	7	8	9	10	Je ne sais pas
1-Plus faible -> Plus élevé-10	○	○	○	○	○	○	○	○	○	○	○

* ED 2.4 L'implication des parents dans l'éducation : Mesure dans laquelle les parents sont impliqués dans le développement et l'exécution des stratégies éducatives (c'est-à-dire à travers l'implication des comités de parents d'élèves et d'enseignants).

	1	2	3	4	5	6	7	8	9	10	Je ne sais pas
1-Plus faible -> Plus élevé-10	○	○	○	○	○	○	○	○	○	○	○

* ED 2.5 Les évaluations standardisées : Mesure dans laquelle toutes les écoles utilisent des évaluations standardisées des écoles et des élèves/étudiants en accord avec les instructions émises par le Ministère de l'Éducation.

	1	2	3	4	5	6	7	8	9	10	Je ne sais pas
1-Plus faible -> Plus élevé-10	○	○	○	○	○	○	○	○	○	○	○

* ED 2.6. L'utilisation des résultats d'évaluation pour informer le progrès : Mesure dans laquelle des ajustements sont apportés aux écoles en fonction des résultats des évaluations scolaires nationales standardisées.

	1	2	3	4	5	6	7	8	9	10	Je ne sais pas
1-Plus faible -> Plus élevé-10	○	○	○	○	○	○	○	○	○	○	○

ED 2.7 Commentaires : Veuillez utiliser cette section pour nous faire part de tout commentaire additionnel sur le niveau d'effort, les défis ou les succès du pays autour des services/ programmes dans le secteur de l'éducation. Si vous avez répondu « Je ne sais pas » à l'une des questions précédentes, veuillez utiliser cette section pour expliquer pourquoi.

JOHNS HOPKINS
BLOOMBERG SCHOOL
of PUBLIC HEALTH

Bill & Melinda Gates Institute for Population and Reproductive Health

Enquête de l'Indice des efforts pour le dividende démographique

Domaine 3. Plaidoyer

* ED 3.1 L'égalité : Mesure dans laquelle l'égalité et l'éducation équitable ont été soulignés par les plaideurs.

	1	2	3	4	5	6	7	8	9	10	Je ne sais pas
1-Plus faible -> Plus élevé-10	○	○	○	○	○	○	○	○	○	○	○

* ED 3.2 La qualité : Mesure dans laquelle la qualité de l'éducation est intégrée aux évaluations liées au plaidoyer pour le système éducatif.

	1	2	3	4	5	6	7	8	9	10	Je ne sais pas
1-Plus faible -> Plus élevé-10	○	○	○	○	○	○	○	○	○	○	○

* ED 3.3 Les obstacles des enseignants : Mesure dans laquelle les besoins des enseignants ont été mis en évidence par les plaideurs.

	1	2	3	4	5	6	7	8	9	10	Je ne sais pas
1-Plus faible -> Plus élevé-10	○	○	○	○	○	○	○	○	○	○	○

* ED 3.4 Les obstacles des institutions : Mesure dans laquelle les difficultés des institutions éducatives ont été mises en évidence dans les différents efforts de plaidoyer.

	1	2	3	4	5	6	7	8	9	10	Je ne sais pas
1-Plus faible -> Plus élevé-10	○	○	○	○	○	○	○	○	○	○	○

ED 3.5 Commentaires : Veuillez utiliser cette section pour nous faire part de tout commentaire additionnel sur le niveau d'effort, les défis ou les succès du pays autour du plaidoyer dans le secteur de l'éducation. Si vous avez répondu « Je ne sais pas » à l'une des questions précédentes, veuillez utiliser cette section pour expliquer pourquoi.

JOHNS HOPKINS
BLOOMBERG SCHOOL
of PUBLIC HEALTH

Bill & Melinda Gates Institute for Population and Reproductive Health

Enquête de l’Indice des efforts pour le dividende démographique

Domaine 4. Recherche

* ED 4.1 La force des observatoires/parties prenantes/groupes de travail pour l’ED : Mesure dans laquelle les parties prenantes du secteur de l’éducation du pays (ex : groupe directif de parties prenantes, groupes de travail techniques, observatoire de l’ED, groupe de coordination ou facilitation) :

	1	2	3	4	5	6	7	8	9	10	Je ne sais pas
ED 4.1.1 Ont un représentant du gouvernement, des institutions de formation, de la société civile, des organisations non gouvernementales et confessionnelles, des associations professionnelles, du secteur privé, etc.	○	○	○	○	○	○	○	○	○	○	○
ED 4.1.2 Se réunissent régulièrement, émettent des rapports et recommandent des politiques aux directions des ministères compétents.	○	○	○	○	○	○	○	○	○	○	○
ED 4.1.3 Ont un impact sur l’éducation dans le pays.	○	○	○	○	○	○	○	○	○	○	○

* ED 4.2 La stratégie d’éducation/recherche : Mesure dans laquelle le plan d’éducation nationale et/ou d’autres documents nationaux comprennent une stratégie/approche complète de recherche liée à l’éducation, partagée entre partenaires.

	1	2	3	4	5	6	7	8	9	10	Je ne sais pas
1-Plus faible -> Plus élevé-10	○	○	○	○	○	○	○	○	○	○	○

* ED 4.3 Les partenaires collectant les données :

	1	2	3	4	5	6	7	8	9	10	Je ne sais pas
ED 4.3.1 Mesure dans laquelle la collecte des données est entreprise par des agences gouvernementales. (Cela peut comprendre les agences/bureaux de la statistique et/ou les ministères de l'économie/finances, éducation, santé, genre, etc.)	○	○	○	○	○	○	○	○	○	○	○
ED 4.3.2 Mesure dans laquelle la collecte de données est entreprise par des institutions de recherche.	○	○	○	○	○	○	○	○	○	○	○
ED 4.3.3 Mesure dans laquelle la collecte des données est entreprise par des chercheurs indépendants.	○	○	○	○	○	○	○	○	○	○	○

* ED 4.4 La recherche thématique :

	1	2	3	4	5	6	7	8	9	10	Je ne sais pas
ED 4.4.1 Mesure dans laquelle de la recherche est menée sur les inégalités de genre dans l'éducation.	○	○	○	○	○	○	○	○	○	○	○
ED 4.4.2 Mesure dans laquelle de la recherche est menée sur la qualité de l'enseignement.	○	○	○	○	○	○	○	○	○	○	○
ED 4.4.3 Mesure dans laquelle de la recherche est menée sur les obstacles auxquels font face les enseignants.	○	○	○	○	○	○	○	○	○	○	○
ED 4.4.4 Mesure dans laquelle de la recherche est menée sur les obstacles auxquels font face les institutions.	○	○	○	○	○	○	○	○	○	○	○

* ED 4.5 La qualité/couverture des données : Mesure dans laquelle la recherche/les données actuelles sont désagrégées par sexe et par âge. Mesure dans laquelle le pays participe aux tests standardisés et son classement.

	1	2	3	4	5	6	7	8	9	10	Je ne sais pas
ED 4.5.1 Mesure dans laquelle un système statistique de routine (utilisant les informations des écoles/institutions) offre de bonnes informations régulières sur les produits et les établissements	○	○	○	○	○	○	○	○	○	○	○
ED 4.5.2 Mesure dans laquelle un système statistique de routine (utilisant les informations des écoles/institutions) offre de bonnes informations régulières sur les services et le personnel du secteur éducatif	○	○	○	○	○	○	○	○	○	○	○
ED 4.5.3 Mesure dans laquelle un système statistique de routine offre de bonnes informations régulières sur les besoins éducatifs des populations de différentes communautés	○	○	○	○	○	○	○	○	○	○	○
ED 4.5.4 Mesure dans laquelle des évaluations du système de suivi de l'éducation sont menées et appliquées pour assurer la fiabilité des données	○	○	○	○	○	○	○	○	○	○	○

* ED 4.6 Les registres : Mesure dans laquelle les systèmes de registres, rapports et feedback sur les résultats de l'éducation sont adéquats.

	1	2	3	4	5	6	7	8	9	10	Je ne sais pas
1-Plus faible -> Plus élevé-10	○	○	○	○	○	○	○	○	○	○	○

* ED 4.7 La qualité des institutions de recherche : Mesure dans laquelle le pays a la capacité de soutenir et maintenir des institutions de recherche qui développent des études et/ou collectent des données permettant d'informer les efforts pour l'amélioration de l'éducation/ l'enseignement.

	1	2	3	4	5	6	7	8	9	10	Je ne sais pas
1-Plus faible -> Plus élevé-10	◯	◯	◯	◯	◯	◯	◯	◯	◯	◯	◯

* ED 4.8 L'évaluation : Mesure dans laquelle les statistiques, enquêtes et études sur le programme d'éducation sont utilisées par du personnel spécialisé pour rapporter les opérations du programme et mesurer le progrès.

	1	2	3	4	5	6	7	8	9	10	Je ne sais pas
1-Plus faible -> Plus élevé-10	◯	◯	◯	◯	◯	◯	◯	◯	◯	◯	◯

* ED 4.9 L'utilisation des résultats d'évaluation par les gestionnaires de programmes : Mesure dans laquelle les gestionnaires locaux du programme d'éducation utilisent les conclusions de recherche et d'évaluations pour améliorer ces programmes en suivant les recommandations émises.

	1	2	3	4	5	6	7	8	9	10	Je ne sais pas
1-Plus faible -> Plus élevé - 10	◯	◯	◯	◯	◯	◯	◯	◯	◯	◯	◯

* ED 4.10 L'utilisation des résultats d'évaluation par les ministères : Mesure dans laquelle les administrateurs ministériels compétents (soit le Ministère de l'Éducation) utilisent systématiquement les données pour informer les politiques et interventions visant à résoudre les problèmes éducatifs.

	1	2	3	4	5	6	7	8	9	10	Je ne sais pas
1-Plus faible -> Plus élevé-10	◯	◯	◯	◯	◯	◯	◯	◯	◯	◯	◯

* ED 4.11 La dissémination d'informations à d'autres entités chargées de la mise en œuvre des programmes :

	1	2	3	4	5	6	7	8	9	10	Je ne sais pas
ED 4.11.1 Mesure dans laquelle des informations sont partagées ou disséminées entres géographies/ régions.	◯	◯	◯	◯	◯	◯	◯	◯	◯	◯	◯
ED. 4.11.2 Mesure dans laquelle des informations sont partagées ou disséminées à différents niveaux (national, états/provinces, localités).	◯	◯	◯	◯	◯	◯	◯	◯	◯	◯	◯

ED 4.12 Commentaires : Veuillez utiliser cette section pour nous faire part de tout commentaire additionnel sur le niveau d’effort, les défis ou les succès du pays autour de la recherche dans le secteur de l’éducation. Si vous avez répondu « Je ne sais pas » à l’une des questions précédentes, veuillez utiliser cette section pour expliquer pourquoi.

JOHNS HOPKINS
BLOOMBERG SCHOOL
of PUBLIC HEALTH

Bill & Melinda Gates Institute for Population and Reproductive Health

Enquête de l'Indice des efforts pour le dividende démographique

Domaine 5. Organisations de la société civile (OSC)

* ED 5.1 Le pouvoir des acteurs des OSC : Mesure dans laquelle les acteurs des OSC occupent des postes d'influence dans les institutions, les groupes de travail techniques ou les réseaux concernés par l'éducation.

	1	2	3	4	5	6	7	8	9	10	Je ne sais pas
1-Plus faible -> Plus élevé-10	○	○	○	○	○	○	○	○	○	○	○

* ED 5.2 L'analyse budgétaire comme outil des OSC : Mesure dans laquelle les OSC utilisent l'analyse budgétaire comme un outil pour développer le plaidoyer en matière d'éducation.

	1	2	3	4	5	6	7	8	9	10	Je ne sais pas
1-Plus faible -> Plus élevé-10	○	○	○	○	○	○	○	○	○	○	○

* ED 5.3 L'inclusion communautaire : Mesure dans laquelle les OSC sont impliquées dans les efforts d'exploration d'opportunités pour l'amélioration de la qualité de l'éducation.

	1	2	3	4	5	6	7	8	9	10	Je ne sais pas
1-Plus faible -> Plus élevé-10	○	○	○	○	○	○	○	○	○	○	○

* ED 5.4 L'implication de la communauté dans l'éducation : Mesure dans laquelle les OSC participent au rapprochement des informations et services éducatifs avec les communautés à travers des activités telles que le renforcement des compétences des enseignants et des parents et/ou les campagnes d'information ou d'éducation pour augmenter l'engagement des familles et des communautés en faveur de l'éducation.

	1	2	3	4	5	6	7	8	9	10	Je ne sais pas
1-Plus faible -> Plus élevé-10	○	○	○	○	○	○	○	○	○	○	○

* ED 5.5 La franchise sociale : Mesure dans laquelle les OSC sont impliquées dans la franchise sociale des services/opportunités éducatives de qualité aux familles et enfants à moindre coût.

	1	2	3	4	5	6	7	8	9	10	Je ne sais pas
1-Plus faible -> Plus élevé-10	○	○	○	○	○	○	○	○	○	○	○

* ED 5.6 La technologie : Mesure dans laquelle les OSC mettent à profit les technologies de l'information et de la communication (TIC) pour améliorer l'accès à l'éducation de qualité de diverses communautés.

	1	2	3	4	5	6	7	8	9	10	Je ne sais pas
1-Plus faible -> Plus élevé-10	○	○	○	○	○	○	○	○	○	○	○

* ED 5.7 Les droits humains, l'accès et la qualité de l'éducation : Mesure dans laquelle les OSC plaident pour le soutien d'approches éducatives basées sur les droits et veillent à ce que les élèves/étudiants soit traités correctement, n'aient pas peur de poser des questions, et reçoivent un enseignement de qualité, entre autres préoccupations.

	1	2	3	4	5	6	7	8	9	10	Je ne sais pas
1-Plus faible -> Plus élevé-10	○	○	○	○	○	○	○	○	○	○	○

* ED 5.8 Le changement social et des comportements (CSC) : Mesure dans laquelle les OSC soutiennent les interventions pour le CSC comprenant, mais sans s'y limiter, aux compétences de vie et à l'éducation entre pairs, l'éducation sur le santé de l'adulte, l'éducation en milieu scolaire, la mobilisation communautaire et/ou les interventions médiatiques destinées au grand public.

	1	2	3	4	5	6	7	8	9	10	Je ne sais pas
1-Plus faible -> Plus élevé-10	○	○	○	○	○	○	○	○	○	○	○

* ED 5.9 Le plaidoyer/ la redevabilité : Mesure dans laquelle les OSC soutiennent la formation et le renforcement de compétences des plaideurs et des membres de la communauté pour améliorer leur compréhension des politiques et processus, leurs activités d'identification de problèmes, la résolution collaborative de ces problèmes, et des actions de plaidoyer plus ciblées pour améliorer l'éducation.

	1	2	3	4	5	6	7	8	9	10	Je ne sais pas
1-Plus faible -> Plus élevé-10	○	○	○	○	○	○	○	○	○	○	○

* ED 5.10 L'évaluation et le suivi dirigés par les OSC : Mesure dans laquelle les OSC évaluent, suivent et émettent des rapports sur l'efficacité des politiques et programmes pour améliorer la redevabilité des enseignants et des politiques d'éducation.

	1	2	3	4	5	6	7	8	9	10	Don't know
1-Plus faible -> Plus élevé-10	◯	◯	◯	◯	◯	◯	◯	◯	◯	◯	◯

* ED 5.11 Les partenariats entre OSC : Mesure dans laquelle les OSC ont formé des alliances nationales et des partenariats régionaux pour renforcer leur position et potentiellement améliorer la force du leadership et les mécanismes de financement en vue d'améliorer la qualité de l'éducation.

	1	2	3	4	5	6	7	8	9	10	Je ne sais pas
1-Plus faible -> Plus élevé-10	◯	◯	◯	◯	◯	◯	◯	◯	◯	◯	◯

ED 5.12 Commentaires : Veuillez utiliser cette section pour nous faire part de tout commentaire additionnel sur le niveau d'effort, les défis ou les succès du pays autour des organisations de la société civile impliquées dans le secteur de l'éducation. Si vous avez répondu « Je ne sais pas » à l'une des questions précédentes, veuillez utiliser cette section pour expliquer pourquoi.

JOHNS HOPKINS
BLOOMBERG SCHOOL
of PUBLIC HEALTH

Bill & Melinda Gates Institute for Population and Reproductive Health

Enquête de l’Indice des efforts pour le dividende démographique

Module : Résilience et durabilité du secteur

Étant donné l’impact sanitaire et socioéconomique de la pandémie de COVID-19, et son impact probable sur la progression vers le DD, cet indice des efforts pour le DD intègre des questions pour évaluer la résilience et la durabilité des systèmes dans les secteurs clés du DD. Les éléments couverts par les questions liées à la pandémie de COVID-19 ci-dessous relèvent des dimensions clés des systèmes résilients et visent à apporter des informations déterminantes sur le potentiel d’une réponse efficace aux menaces de maladies infectieuses émergeantes et autres crises de santé publique.

Veuillez noter chaque élément sur une échelle de 1 à 10, 1 étant le score le plus faible (faible état/capacité) et 10 le plus élevé (très bon état/capacité).

* Dimension physique : structures, équipements, états du système et capacités

	1	2	3	4	5	6	7	8	9	10	Je ne sais pas
ED-M1 - Planification/Préparation – État et capacité de l’équipement, du personnel et de la structure du secteur de l’ED avant la crise. (1 = faible état/capacité; 10 = très bon état/capacité)											
ED-M2 - Absorption – Mesure dans laquelle la pandémie de COVID-19 a été reconnue par le secteur de l’ED, et mesure dans laquelle le système a pu continuer d’être fonctionnel											

	1	2	3	4	5	6	7	8	9	10	Je ne sais pas
ED-M3 - Absorption – Intégration : Niveau d’intégration des services et programmes d’ED pour mitiger les effets de la pandémie de COVID-19 sur l’ED.	◯	◯	◯	◯	◯	◯	◯	◯	◯	◯	◯
ED-M4 - Récupération – Mesure dans laquelle le secteur de l’ED a été capable d’induire des changements pour récupérer un niveau de fonctionnalité pré-COVID-19.	◯	◯	◯	◯	◯	◯	◯	◯	◯	◯	◯
ED-M5 - Récupération – Gestion du financement et des bailleurs de fonds : Niveau de mobilisation des ressources et allocations pour mitiger les effets de la pandémie de COVID-19 sur l’ED.	◯	◯	◯	◯	◯	◯	◯	◯	◯	◯	◯
ED-M6 - Adaptation – Mesure dans laquelle des changements ont été effectués pour améliorer la résilience du secteur de l’ED.	◯	◯	◯	◯	◯	◯	◯	◯	◯	◯	◯

* Information : création, gestion, stockage et utilisation des données.

	1	2	3	4	5	6	7	8	9	10	Je ne sais pas
ED-M7 - Planification/ Préparation – Mesure dans laquelle les données liées à l'ED étaient suffisamment préparées, présentées, analysées et stockées avant la crise.	○	○	○	○	○	○	○	○	○	○	○
ED-M8 - Absorption - Knowledge: Extent to which the monitoring capacity was able to detect the limits / threats posed to the ED sector by the COVID-19 pandemic. (This includes data on the various key subjects of the education sector at each level of education [primary, secondary, tertiary / higher]).	○	○	○	○	○	○	○	○	○	○	○
ED-M9 - Récupération – Mesure dans laquelle les données de l'ED ont été utilisées pour suivre le progrès vers la récupération et anticiper des scenarii de récupération.	○	○	○	○	○	○	○	○	○	○	○
ED-M10 - Adaptation – Mesure dans laquelle le système d'ED crée et améliore actuellement ses protocoles de stockage et d'utilisation de ses données en temps réel.	○	○	○	○	○	○	○	○	○	○	○

* Dimension cognitive : compréhension, modèles cognitifs, idées reçues, préjugés et valeurs.

	1	2	3	4	5	6	7	8	9	10	Je ne sais pas
ED-M11 – Planification/ Préparation – Mesure dans laquelle le système d'ED et les décisions opérationnelles étaient préparés pour anticiper la crise.	○	○	○	○	○	○	○	○	○	○	○

	1	2	3	4	5	6	7	8	9	10	Je ne sais pas
ED-M12 - Absorption – Mesure dans laquelle la réponse du système d'ED a des protocoles de contingence suffisants et une gestion de crise proactive.	○	○	○	○	○	○	○	○	○	○	○
ED-M13 - Absorption – Redevabilité : Niveau d'effort pour assurer une redevabilité optimale vis-à-vis des ressources allouées à l'ED dans le cadre de la réponse à la pandémie de COVID-19.	○	○	○	○	○	○	○	○	○	○	○
ED-M14 - Récupération – Mesure dans laquelle les décisions sont orientées sur la récupération et communiquées aux communautés sur la base de données probantes pour promouvoir des comportements sûrs.	○	○	○	○	○	○	○	○	○	○	○
ED-M15 - Récupération – Connaissance : Mesure dans laquelle le secteur de l'ED établit une certaine confiance à travers sa communication concernant les barrières posées par la pandémie de COVID-19 pour soutenir l'accès et l'utilisation des services et programmes d'ED.	○	○	○	○	○	○	○	○	○	○	○
ED-M16 - Adaptation – Mesure dans laquelle le secteur de l'ED conçoit de nouvelles configurations systémiques, de nouveaux objectifs et de nouveaux critères de décision.	○	○	○	○	○	○	○	○	○	○	○

	1	2	3	4	5	6	7	8	9	10	Je ne sais pas
ED-M17 – Adaptation : Niveau d'adaptation pour répondre aux menaces posées par la pandémie de COVID-19 à l'ED.	○	○	○	○	○	○	○	○	○	○	○

* Social dimension: interaction, collaboration and self-synchronization between people, entities and institutions.

	1	2	3	4	5	6	7	8	9	10	Je ne sais pas
ED-M18 – Planification /Préparation – Mesure dans laquelle une formation sur la gestion d'épidémies/crises a été menée et a permis de mettre à profit les réseaux sociaux, le capital social et les normes institutionnelles et culturelles avant la crise.	○	○	○	○	○	○	○	○	○	○	○
ED-M19 - Absorption – Mesure dans laquelle le personnel et les institutions sociales ont été accessibles et ont fait preuve d'ingéniosité dans leur réponse à l'épidémie/crise.	○	○	○	○	○	○	○	○	○	○	○
ED-M20 - Absorption - Self-regulation: Extent to which national leaders had the authority to effect timely sectoral changes through an infrastructure flexible.	○	○	○	○	○	○	○	○	○	○	○
ED-M21 – Récupération – Mesure dans laquelle le système d'ED est impliqué dans le partage de connaissances et d'équipes pour améliorer la récupération du système.	○	○	○	○	○	○	○	○	○	○	○

	1	2	3	4	5	6	7	8	9	10	Je ne sais pas
ED-M22 - Récupération – Diversité : Niveau d'engagement d'une équipe multidisciplinaire pour mitiger les effets de la pandémie de COVID-19 sur l'ED.	◯	◯	◯	◯	◯	◯	◯	◯	◯	◯	◯
ED-M23 - Adaptation – Mesure dans laquelle des ajouts ou changements sont apportés aux institutions, politiques, formations, programmes et à la culture de l'ED.	◯	◯	◯	◯	◯	◯	◯	◯	◯	◯	◯
ED-M24 - Adaptation - Leadership et management : Niveau de leadership démontré par les dirigeants du secteur de l'ED pour mitiger les effets de la pandémie de COVID-19 sur l'ED.	◯	◯	◯	◯	◯	◯	◯	◯	◯	◯	◯

* ED M-25 Temporalité : Mesure de la rapidité de la réaction du gouvernement –sa communication et mise en œuvre des mesures- pour mitiger l'impact immédiat et sur le long terme de la pandémie de COVID-19. Cette question porte sur la riposte nationale dans son ensemble, et non la réponse spécifique de votre secteur.

	1	2	3	4	5	6	7	8	9	10	Je ne sais pas
1-Plus faible -> Plus élevé-10	◯	◯	◯	◯	◯	◯	◯	◯	◯	◯	◯

ED M-26 Commentaires : Utilisez cette section pour apporter tout commentaire additionnel sur la résilience et la durabilité du système d'éducation nationale en vous référant à votre expérience de la pandémie de COVID-19. Si vous avez répondu « Je ne sais pas » à l'une des questions, utilisez cette section pour expliquer pourquoi.

JOHNS HOPKINS
BLOOMBERG SCHOOL
of PUBLIC HEALTH

Bill & Melinda Gates Institute for Population and Reproductive Health

Enquête de l’Indice des efforts pour le dividende démographique

Questionnaire sur l’autonomisation des femmes (AF)

Répondez aux questions suivantes en fonction de votre expérience/ expertise dans ce secteur spécifique.

Afin d’obtenir une représentation synthétique des efforts nationaux pour le dividende démographique, notez les éléments suivants sur une échelle de 1 à 10, 1 étant le score le plus faible (efforts très faibles ou quasi-inexistants) et 10 le plus élevé (efforts robustes). Le cas échéant, si une politique ou activité n’existe pas, répondez 0.

Donnez un score à chaque élément. Toutes les réponses seront enregistrées au format illustré ci-dessous :

Effort le plus faible	1	2	3	4	5	6	7	8	9	10	Effort le plus élevé	Je ne sais pas

Bill & Melinda Gates Institute for Population and Reproductive Health

Enquête de l'Indice des efforts pour le dividende démographique

Domaine 1. Politique/ Prise de décision

* WE 1.1 La politique de parité : Mesure dans laquelle la parité est prise en compte/intégrée aux plans de développement du pays (ou aux plans de votre secteur).

	1	2	3	4	5	6	7	8	9	10	Je ne sais pas
1-Plus faible -> Plus élevé-10	○	○	○	○	○	○	○	○	○	○	○

* WE 1.2 Niveau d'engagement envers la Convention sur l'élimination de toutes les formes de discrimination à l'égard des femmes (CEDAW) :

	1	2	3	4	5	6	7	8	9	10	Je ne sais pas
1-Plus faible -> Plus élevé-10	○	○	○	○	○	○	○	○	○	○	○

* WE 1.3 La parité dans les politiques familiales et de mariage : Mesure dans laquelle le principe d'égalité hommes-femmes est incorporé à la constitution nationale et autres législations concernant le mariage et les relations familiales.

	1	2	3	4	5	6	7	8	9	10	Je ne sais pas
1-Plus faible -> Plus élevé-10	○	○	○	○	○	○	○	○	○	○	○

* WE 1.4 L'âge légal du mariage : Mesure dans laquelle l'âge légal du mariage pour les filles est établi à 18 ans ou plus et est appliqué.

	1	2	3	4	5	6	7	8	9	10	Je ne sais pas
1-Plus faible -> Plus élevé-10	○	○	○	○	○	○	○	○	○	○	○

* WE 1.5 Les mesures contre les violences à l'égard des femmes : Niveau des régulations et mesures contre les violences faites aux femmes, dont la violence intraconjugale, le viol, le harcèlement sexuel et la traite des filles et des femmes.

	1	2	3	4	5	6	7	8	9	10	Je ne sais pas
1-Plus faible -> Plus élevé-10											

* WE 1.6 La mutilation génitale féminine : Niveau de régulation et mesures contre la mutilation génitale féminine.

	1	2	3	4	5	6	7	8	9	10	Je ne sais pas
1-Plus faible -> Plus élevé-10											

* WE 1.7 Les mesures de santé des femmes : Niveau des protocoles de protection de la santé des femmes, dont les infections sexuellement transmissibles, y compris le VIH/SIDA, la mortalité maternelle et la contraception.

	1	2	3	4	5	6	7	8	9	10	Je ne sais pas
1-Plus faible -> Plus élevé-10											

* WE 1.8 Les mesures éducatives : Mesure dans laquelle il existe des mécanismes pour promouvoir l'éducation des filles.

	1	2	3	4	5	6	7	8	9	10	Je ne sais pas
WE 1.8.1 Niveau d'application des politiques sur l'éducation des filles.											
WE 1.8.2 Mesure dans laquelle des mécanismes sont en place pour prévenir l'échec/l'abandon scolaire des filles.											

* WE 1.9 Les mesures relatives à l'exercice des droits : Niveau d'application des politiques sur les droits des femmes.

	1	2	3	4	5	6	7	8	9	10	Je ne sais pas
1-Plus faible -> Plus élevé-10											

* WE 1.10 Le travail équitable : Mesure dans laquelle il existe des politiques sur les conditions de travail équitables et l'égalité des salaires entre hommes et femmes.

	1	2	3	4	5	6	7	8	9	10	Je ne sais pas
1-Plus faible -> Plus élevé-10	○	○	○	○	○	○	○	○	○	○	○

* WE 1.11 L'égalité de la propriété de biens/biens fonciers : Mesure dans laquelle il existe des politiques pour affirmer l'accès équitable à la propriété de biens fonciers.

	1	2	3	4	5	6	7	8	9	10	Je ne sais pas
1-Plus faible -> Plus élevé-10	○	○	○	○	○	○	○	○	○	○	○

* WE 1.12 Le conflit, les femmes et le rétablissement de la paix : Niveau de mise en œuvre de la Résolution 1325 du Conseil de sécurité des Nations Unies sur les impacts des conflits sur les femmes et leur rôle dans le rétablissement de la paix.

	1	2	3	4	5	6	7	8	9	10	Je ne sais pas
1-Plus faible -> Plus élevé-10	○	○	○	○	○	○	○	○	○	○	○

* WE 1.13 Les quotas de femmes dans les politiques : Existence et niveau de mise en œuvre des quotas dans les systèmes politiques observant la parité.

	1	2	3	4	5	6	7	8	9	10	Je ne sais pas
WE 1.13.1 Mesure dans laquelle il existe une prise de décision équitable du point de vue du genre, y compris en soutien au quotas électoraux et à l'intégration des femmes dans tous les ministères et départements du gouvernement.	○	○	○	○	○	○	○	○	○	○	○
WE 1.13.2 Mesure dans laquelle les femmes participant et/ou jouent un rôle significatif dans les systèmes traditionnels de gouvernance (ex : chefs de village, etc.).	○	○	○	○	○	○	○	○	○	○	○

* WE 1.14 L'Objectif de Développement Durable (ODD) 5 : Niveau d'engagement pour remplir l'Objectif 5 des ODD, pour réaliser la parité et assurer l'autonomisation de toutes les filles et femmes et les filles.

	1	2	3	4	5	6	7	8	9	10	Je ne sais pas
1-Plus faible -> Plus élevé-10											

* WE 1.15 La révision de la loi coutumière : Mesure dans laquelle les lois coutumières ont été revues et modifiées par des politiques/cadres nationaux ; ou mesure dans laquelle les lois coutumières existantes reflètent les efforts pour l'autonomisation des femmes.

	1	2	3	4	5	6	7	8	9	10	Je ne sais pas
1-Plus faible -> Plus élevé-10											

* WE 1.16 La budgétisation pour l'autonomisation des femmes : Mesure dans laquelle la budgétisation et l'allocation budgétaire sont suffisantes pour couvrir les coûts nécessaires à l'atteinte des cibles établies dans le plan national de la femme ou son équivalent.

	1	2	3	4	5	6	7	8	9	10	Je ne sais pas
1-Plus faible -> Plus élevé-10											

* WE 1.17 Les ressources humaines pour l'autonomisation des femmes : Mesure dans laquelle :

	1	2	3	4	5	6	7	8	9	10	Je ne sais pas
WE 1.17.1 Du personnel en quantité et qualité suffisante est employé pour mettre en œuvre les activités liées au genre, y compris les efforts pour former ce personnel											
WE 1.17.2 Des efforts sont menés pour former le personnel de manière appropriée et intégrale.											
WE 1.17.3 Le personnel est suffisamment soutenu pour mener à bien ses tâches.											
WE 1.17.4 Le personnel assigné est situé à un niveau administratif suffisamment élevé pour travailler efficacement.											

* WE 1.18 La transparence et la redevabilité : Mesure dans laquelle le gouvernement fournit aux parties prenantes des informations complètes sur les intentions, plans, programmes et budgets disponibles pour mettre en œuvre les programmes d'autonomisation des femmes et en assurer la redevabilité et la transparence. (*Peuvent être incluses l'adoption et l'application d'une loi sur le droit à l'information, des sessions informatives régulières, par exemple dans les mairies, ou la transmission de ces informations via des brochures ou autres supports de communication en langue locale*).

	1	2	3	4	5	6	7	8	9	10	Je ne sais pas
1-Plus faible -> Plus élevé-10	○	○	○	○	○	○	○	○	○	○	○

WE 1.19 Commentaire : Utilisez cette section pour apporter des commentaires additionnels sur le niveau d'efforts, les défis et les succès du pays autour de la politique/ prise de décision pour l'autonomisation des femmes. Si vous avez répondu « Je ne sais pas » à l'une des questions ci-dessus, utilisez aussi cette section pour expliquer pourquoi.

JOHNS HOPKINS
BLOOMBERG SCHOOL
of PUBLIC HEALTH

Bill & Melinda Gates Institute for Population and Reproductive Health

Enquête de l'Indice des efforts pour le dividende démographique

Domaine 2. Services ou programmes

* WE 2.1 L'intégration des femmes : Mesure dans laquelle les services ou programmes sectoriels et intersectoriels tiennent compte des implications des actions planifiées pour les hommes et pour les femmes.

	1	2	3	4	5	6	7	8	9	10	Je ne sais pas
1-Plus faible -> Plus élevé-10	◯	◯	◯	◯	◯	◯	◯	◯	◯	◯	◯

* WE 2.2 Les programmes transformateurs de genre : Mesure dans laquelle les services ou programmes sectoriels ou intersectoriels intègrent des principes/ approches transformateurs de genre.

	1	2	3	4	5	6	7	8	9	10	Je ne sais pas
1-Plus faible -> Plus élevé-10	◯	◯	◯	◯	◯	◯	◯	◯	◯	◯	◯

* WE 2.3 L'éducation : Mesure dans laquelle les services ou programmes éducatifs se focalisent sur l'autonomisation des filles et des femmes dans les salles de classe.

	1	2	3	4	5	6	7	8	9	10	Je ne sais pas
1-Plus faible -> Plus élevé-10	◯	◯	◯	◯	◯	◯	◯	◯	◯	◯	◯

* WE 2.4. L'abandon scolaire : Mesure dans laquelle il existe des programmes pour prévenir l'abandon scolaire des filles.

	1	2	3	4	5	6	7	8	9	10	Je ne sais pas
1-Plus faible -> Plus élevé-10	◯	◯	◯	◯	◯	◯	◯	◯	◯	◯	◯

* WE 2.5 La maîtrise des technologies : Mesure dans laquelle la maîtrise des technologies est promue auprès des filles en particulier.

	1	2	3	4	5	6	7	8	9	10	Je ne sais pas
1-Plus faible -> Plus élevé-10	◯	◯	◯	◯	◯	◯	◯	◯	◯	◯	◯

* WE 2.6 L'emploi : Mesure dans laquelle les programmes de création d'emploi et les politiques du marché du travail visent à garantir l'indépendance économique des femmes et sont conçus/mis en œuvre en conséquence.

	1	2	3	4	5	6	7	8	9	10	Je ne sais pas
1-Plus faible -> Plus élevé-10	◯	◯	◯	◯	◯	◯	◯	◯	◯	◯	◯

* WE 2.7 Les femmes-mères-travailleuses : Mesure dans laquelle le gouvernement permet aux femmes, à travers la loi, les régulations et les programmes, de combiner leur rôle de mère pendant la grossesse, l'allaitement et l'éducation de leurs enfants à leur participation à la vie active.

	1	2	3	4	5	6	7	8	9	10	Je ne sais pas
1-Plus faible -> Plus élevé-10	◯	◯	◯	◯	◯	◯	◯	◯	◯	◯	◯

* WE 2.8 La planification familiale pour l'autonomisation des femmes : Mesure dans laquelle les services ou programmes de PF sont mis en œuvre selon une approche fondamentale d'autonomisation des femmes.

	1	2	3	4	5	6	7	8	9	10	Je ne sais pas
1-Plus faible -> Plus élevé-10	◯	◯	◯	◯	◯	◯	◯	◯	◯	◯	◯

* WE 2.9 La représentation politique et publique : Mesure de la mise en œuvre des programmes visant à améliorer la participation et la représentation équitables des femmes à tous les niveaux du processus politique et de la vie publique.

	1	2	3	4	5	6	7	8	9	10	Je ne sais pas
1-Plus faible -> Plus élevé-10	◯	◯	◯	◯	◯	◯	◯	◯	◯	◯	◯

* WE 2.10 La violence : Portée de la lutte contre les violences faites à l'égard des femmes et des enfants, et leur élimination à l'échelle du pays (violences intraconjugales, viol, harcèlement sexuel, traite des femmes).

	1	2	3	4	5	6	7	8	9	10	Je ne sais pas
1-Plus faible -> Plus élevé-10	◯	◯	◯	◯	◯	◯	◯	◯	◯	◯	◯

* WE 2.11 Le mariage d’enfants : Mesure de l’application des lois pour éliminer le mariage d’enfants.

	1	2	3	4	5	6	7	8	9	10	Je ne sais pas
1-Plus faible -> Plus élevé-10	◯	◯	◯	◯	◯	◯	◯	◯	◯	◯	◯

* WE 2.12 La mutilation génitale féminine : Mesure de l’application des lois pour éliminer la mutilation génitale féminine.

	1	2	3	4	5	6	7	8	9	10	Je ne sais pas
1-Plus faible -> Plus élevé-10	◯	◯	◯	◯	◯	◯	◯	◯	◯	◯	◯

WE 2.13 Commentaire : Utilisez cette section pour apporter des commentaires additionnels sur le niveau d’efforts, les défis et les succès du pays autour des services et programmes pour l’autonomisation des femmes. Si vous avez répondu « Je ne sais pas » à l’une des questions ci-dessus, utilisez aussi cette section pour expliquer pourquoi.

JOHNS HOPKINS
BLOOMBERG SCHOOL
of PUBLIC HEALTH

Bill & Melinda Gates Institute for Population and Reproductive Health

Enquête de l'Indice des efforts pour le dividende démographique

Domaine 3. Plaidoyer

* WE 3.1 Les plaideurs : Mesure dans laquelle des plaideurs pour l'autonomisation des filles et des femmes existent au niveau national et infranational.

	1	2	3	4	5	6	7	8	9	10	Je ne sais pas
1-Plus faible -> Plus élevé-10	◯	◯	◯	◯	◯	◯	◯	◯	◯	◯	◯

* WE 3.2. Les filles et les femmes : Mesure dans laquelle les efforts de plaidoyer dans ce domaine recouvrent l'ensemble des aspects de l'autonomisation des filles et des femmes.

	1	2	3	4	5	6	7	8	9	10	Je ne sais pas
1-Plus faible -> Plus élevé-10	◯	◯	◯	◯	◯	◯	◯	◯	◯	◯	◯

* WE 3.3 La dissémination de l'information : Mesure dans laquelle le gouvernement entreprend suffisamment d'efforts pour veiller à ce que la population soit consciente des problèmes liés à l'autonomisation des femmes, et si les informations disséminées sont exactes et communiquées dans la langue parlée par les destinataires/publics cibles.

	1	2	3	4	5	6	7	8	9	10	Je ne sais pas
1-Plus faible -> Plus élevé-10	◯	◯	◯	◯	◯	◯	◯	◯	◯	◯	◯

* WE 3.4 La mise à profit des partenariats : Mesure dans laquelle le gouvernement met à profit ses partenariats avec des OSC pour disséminer des informations sur l'autonomisation des femmes à travers des campagnes de sensibilisation/information.

	1	2	3	4	5	6	7	8	9	10	Je ne sais pas
1-Plus faible -> Plus élevé-10	◯	◯	◯	◯	◯	◯	◯	◯	◯	◯	◯

* WE 3.5 L'emploi : Mesure dans laquelle les parties prenantes appellent à l'élaboration de lois, politiques et programmes pour que les femmes puissent combiner leur rôle de mère pendant la grossesse, l'allaitement et l'éducation de leurs enfants avec leur participation à la vie active.

	1	2	3	4	5	6	7	8	9	10	Je ne sais pas
1-Plus faible -> Plus élevé-10	○	○	○	○	○	○	○	○	○	○	○

* WE 3.6 L'éducation : Mesure dans laquelle l'une des priorités des plaideurs est de promouvoir la réalisation du plein potentiel des filles et des femmes à travers l'école et le développement de compétences.

	1	2	3	4	5	6	7	8	9	10	Je ne sais pas
1-Plus faible -> Plus élevé-10	○	○	○	○	○	○	○	○	○	○	○

* WE 3.7 La violence : Mesure dans laquelle les plaideurs réussissent à interpeller le gouvernement pour le faire réagir et répondre aux cas de violence et de discrimination contre les femmes.

	1	2	3	4	5	6	7	8	9	10	Je ne sais pas
1-Plus faible -> Plus élevé-10	○	○	○	○	○	○	○	○	○	○	○

WE 3.8 Commentaire : Utilisez cette section pour apporter des commentaires additionnels sur le niveau d'efforts, les défis et les succès du pays autour du plaidoyer pour l'autonomisation des femmes. Si vous avez répondu « Je ne sais pas » à l'une des questions ci-dessus, utilisez aussi cette section pour expliquer pourquoi.

JOHNS HOPKINS
BLOOMBERG SCHOOL
of PUBLIC HEALTH

Bill & Melinda Gates Institute for Population and Reproductive Health

Enquête de l'Indice des efforts pour le dividende démographique

Domaine 4. Recherche

* WE 4.1 La force des observatoires/parties prenantes/groupes de travail pour l'autonomisation des femmes : Mesure dans laquelle les parties prenantes du secteur (ex : groupe directif de parties prenantes, groupes de travail techniques, observatoire de l'autonomisation des femmes, groupe de coordination ou facilitation) :

	1	2	3	4	5	6	7	8	9	10	Je ne sais pas
WE 4.1.1 Ont un représentant du gouvernement, des institutions de formation, de la société civile, des organisations non gouvernementales et confessionnelles, des associations professionnelles, du secteur privé, etc.	○	○	○	○	○	○	○	○	○	○	○
WE 4.1.2 Se réunissent régulièrement, émettent des rapports et recommandent des politiques aux directions des ministères compétents.	○	○	○	○	○	○	○	○	○	○	○
WE 4.1.3 Ont un impact sur l'autonomisation des femmes dans le pays.	○	○	○	○	○	○	○	○	○	○	○

* WE 4.2 La stratégie de recherche sur les droits des femmes : Mesure dans laquelle le plan national pour l'autonomisation des femmes et/ou autres documents nationaux comprennent une approche/stratégie de recherche sur l'autonomisation des femmes, partagée entre les partenaires.

	1	2	3	4	5	6	7	8	9	10	Je ne sais pas
1-Plus faible -> Plus élevé-10	○	○	○	○	○	○	○	○	○	○	○

* WE 4.3 Partenaires collectant des données :

	1	2	3	4	5	6	7	8	9	10	Je ne sais pas
WE 4.3.1 Mesure dans laquelle la collecte des données est entreprise par des agences gouvernementales. (Cela peut comprendre les agences/bureaux de la statistique et/ou les ministères de l'économie/finances, éducation, santé, genre, etc.)	◯	◯	◯	◯	◯	◯	◯	◯	◯	◯	◯
WE 4.3.2 Mesure dans laquelle la collecte de données est entreprise par des institutions de recherche.	◯	◯	◯	◯	◯	◯	◯	◯	◯	◯	◯
WE 4.3.3 Mesure dans laquelle la collecte des données est entreprise par des chercheurs indépendants.	◯	◯	◯	◯	◯	◯	◯	◯	◯	◯	◯

* WE 4.4 La recherche thématique :

	1	2	3	4	5	6	7	8	9	10	Je ne sais pas
WE 4.4.1 Mesure dans laquelle de la recherche est menée sur les normes sociales et culturelles affectant l'autonomisation des femmes et la parité.	◯	◯	◯	◯	◯	◯	◯	◯	◯	◯	◯
WE 4.4.2 Mesure dans laquelle de la recherche est menée sur les effets économiques et sanitaires de l'autonomisation des femmes.	◯	◯	◯	◯	◯	◯	◯	◯	◯	◯	◯
WE 4.4.3 Mesure dans laquelle de la recherche est menée sur le mariage d'enfants pour informer la politique.	◯	◯	◯	◯	◯	◯	◯	◯	◯	◯	◯
WE 4.4.4 Mesure dans laquelle de la recherche est menée sur les résultats de l'éducation et de l'acquisition de compétences chez les filles.	◯	◯	◯	◯	◯	◯	◯	◯	◯	◯	◯

* WE 4.5 La qualité/ couverture des données :

	1	2	3	4	5	6	7	8	9	10	Je ne sais pas
WE 4.5.1 Mesure dans laquelle un système statistique de routine fiable fournit des données périodiques désagrégées par âge.	◯	◯	◯	◯	◯	◯	◯	◯	◯	◯	◯
WE 4.5.2 Mesure dans laquelle un système statistique de routine fiable fournit de bonnes données périodiques désagrégées par âge sur les besoins relatifs à l'autonomie des femmes parmi les populations de différentes communautés.	◯	◯	◯	◯	◯	◯	◯	◯	◯	◯	◯
WE 4.5.3 Mesure dans laquelle les systèmes de suivi et de surveillance sont évalués, et ces évaluations appliquées pour assurer la fiabilité des données désagrégées par âge.	◯	◯	◯	◯	◯	◯	◯	◯	◯	◯	◯

* WE 4.6 La qualité des institutions de recherche : Mesure dans laquelle le pays soutient et maintient des institutions de recherche qui développent des études permettant de soutenir la portée et la qualité de la collecte de données pour informer les efforts nationaux d'autonomisation des femmes.

	1	2	3	4	5	6	7	8	9	10	Je ne sais pas
1-Plus faible -> Plus élevé-10	◯	◯	◯	◯	◯	◯	◯	◯	◯	◯	◯

* WE 4.7 L'évaluation : Mesure dans laquelle les statistiques, enquêtes et études sur les programmes sont utilisées par du personnel spécialisé pour rapporter les opérations du programme et mesurer les progrès en matière d'autonomisation des femmes et de parité.

	1	2	3	4	5	6	7	8	9	10	Je ne sais pas
1-Plus faible -> Plus élevé-10	◯	◯	◯	◯	◯	◯	◯	◯	◯	◯	◯

* WE 4.8 L'utilisation des résultats d'évaluation par les gestionnaires de programme : Mesure dans laquelle les gestionnaires locaux des programmes d'autonomisation des femmes utilisent les conclusions de recherche et d'évaluations pour améliorer ces programmes en suivant les recommandations émises.

	1	2	3	4	5	6	7	8	9	10	Je ne sais pas
1-Plus faible -> Plus élevé-10	○	○	○	○	○	○	○	○	○	○	○

* WE 4.9 L'utilisation des résultats d'évaluation par les ministères : Mesure dans laquelle les administrateurs ministériels compétents utilisent systématiquement les données pour informer les politiques et interventions visant à résoudre les problèmes liés à l'autonomisation des femmes.

	1	2	3	4	5	6	7	8	9	10	Je ne sais pas
1-Plus faible -> Plus élevé-10	○	○	○	○	○	○	○	○	○	○	○

* WE 4.10 La dissémination d'informations à d'autres entités chargées de la mise en œuvre des programmes : Mesure dans laquelle des informations sont partagées ou disséminées entre géographies et à différents niveaux (national, état/province/comté, localités/sous-comtés).

	1	2	3	4	5	6	7	8	9	10	Je ne sais pas
1-Plus faible -> Plus élevé-10	○	○	○	○	○	○	○	○	○	○	○

WE 4.11 Commentaire : Utilisez cette section pour apporter des commentaires additionnels sur le niveau d'efforts, les défis et les succès du pays autour de la recherche sur l'autonomisation des femmes. Si vous avez répondu « Je ne sais pas » à l'une des questions ci-dessus, utilisez aussi cette section pour expliquer pourquoi.

JOHNS HOPKINS
BLOOMBERG SCHOOL of PUBLIC HEALTH

Bill & Melinda Gates Institute for Population and Reproductive Health

Enquête de l’Indice des efforts pour le dividende démographique

Domaine 5. Organisations de la Société Civile (OSC)

* WE 5.1 Le pouvoir des acteurs des OSC : Mesure dans laquelle les acteurs des OSC sont bien placés pour influencer la politique nationale concernant l’autonomisation des femmes.

	1	2	3	4	5	6	7	8	9	10	Je ne sais pas
1-Plus faible -> Plus élevé-10	○	○	○	○	○	○	○	○	○	○	○

* WE 5.2 L’analyse budgétaire comme outil des OSC : Mesure dans laquelle les OSC utilisent l’analyse budgétaire comme un outil pour développer le plaidoyer en matière d’autonomisation des femmes

	1	2	3	4	5	6	7	8	9	10	Je ne sais pas
1-Plus faible -> Plus élevé-10	○	○	○	○	○	○	○	○	○	○	○

* WE 5.3 Le soutien des services à base communautaire : Mesure dans laquelle les OSC participent au rapprochement des informations et services en matière d’autonomisation des femmes avec les communautés à travers des activités comprenant, mais sans s’y limiter, le renforcement de compétences des filles, les défenseurs/champions de l’autonomisation des femmes, et des campagnes d’éducation ou d’information pour augmenter l’engagement des familles et des communautés pour l’autonomie des femmes.

	1	2	3	4	5	6	7	8	9	10	Je ne sais pas
1-Plus faible -> Plus élevé-10	○	○	○	○	○	○	○	○	○	○	○

* WE 5.4 Les droits humains et la qualité de la mise en œuvre : Mesure dans laquelle les OSC plaident et soutiennent des approches de l'autonomisation des femmes fondées sur le droit pour veiller à ce que les bénéficiaires des programmes et politiques d'autonomisation des femmes puissent voir des avancées selon leurs propres termes.

	1	2	3	4	5	6	7	8	9	10	Je ne sais pas
1-Plus faible -> Plus élevé-10	◯	◯	◯	◯	◯	◯	◯	◯	◯	◯	◯

* WE 5.5 Le changement social et des comportements (CSC) : Mesure dans laquelle les OSC soutiennent des interventions pour le CSC comprenant, mais sans s'y limiter, les programmes transformateurs de genre, les compétences de vie et l'éducation entre pairs, l'éducation à la santé de l'adulte, l'éducation en milieu scolaire, la communication au sein du couple, la mobilisation communautaire, les interventions des gardiens de la communauté et/ou des interventions dans les médias de masse pour le grand public. Ces interventions peuvent être mises en œuvre au niveau communautaire ou individuel.

	1	2	3	4	5	6	7	8	9	10	Je ne sais pas
1-Plus faible -> Plus élevé-10	◯	◯	◯	◯	◯	◯	◯	◯	◯	◯	◯

* WE 5.6 Les jeunes : Mesure dans laquelle les OSC promeuvent l'autonomisation des femmes à travers les programmes pour jeunes.

	1	2	3	4	5	6	7	8	9	10	Je ne sais pas
1-Plus faible -> Plus élevé-10	◯	◯	◯	◯	◯	◯	◯	◯	◯	◯	◯

* WE 5.7 Les programmes pour les hommes : Mesure dans laquelle les OSC soutiennent les politiques, interventions et programmes d'autonomisation des femmes ciblant les hommes.

	1	2	3	4	5	6	7	8	9	10	Je ne sais pas
1-Plus faible -> Plus élevé-10	◯	◯	◯	◯	◯	◯	◯	◯	◯	◯	◯

* WE 5.8 Le plaidoyer/ la redevabilité : Mesure dans laquelle les OSC soutiennent la formation et le renforcement de compétences des membres de la communauté pour augmenter leur compréhension des politiques, processus et activités, et pour renforcer leurs capacités à identifier les problèmes liés à l'autonomisation des femmes, résoudre collectivement ces problèmes et mener des actions de plaidoyer plus ciblées.

	1	2	3	4	5	6	7	8	9	10	Je ne sais pas
1-Plus faible -> Plus élevé-10	◯	◯	◯	◯	◯	◯	◯	◯	◯	◯	◯

* WE 5.9 L’évaluation et le suivi dirigés par les OSC : Mesure dans laquelle les OSC évaluent, suivent et émettent des rapports sur l’efficacité des politiques et programmes pour améliorer la redevabilité des prestataires et des politiques d’autonomisation des femmes.

	1	2	3	4	5	6	7	8	9	10	Je ne sais pas
1-Plus faible -> Plus élevé-10	○	○	○	○	○	○	○	○	○	○	○

* WE 5.10 Les partenariats entre OSC : Mesure dans laquelle les OSC ont formé des alliances nationales et des partenariats régionaux pour renforcer leur position et potentiellement améliorer le leadership et les mécanismes de financement afin de faire progresser l’agenda de l’autonomisation des femmes.

	1	2	3	4	5	6	7	8	9	10	Je ne sais pas
1-Plus faible -> Plus élevé-10	○	○	○	○	○	○	○	○	○	○	○

WE 5.11 Commentaire : Utilisez cette section pour apporter des commentaires additionnels sur le niveau d’efforts, les défis et les succès du pays concernant le rôle des OSC pour l’autonomisation des femmes. Si vous avez répondu « Je ne sais pas » à l’une des questions ci-dessus, utilisez aussi cette section pour expliquer pourquoi.

JOHNS HOPKINS
BLOOMBERG SCHOOL
of PUBLIC HEALTH

Bill & Melinda Gates Institute for Population and Reproductive Health

Enquête de l’Indice des efforts pour le dividende démographique

Module : Résilience et durabilité du secteur

Étant donné l’impact sanitaire et socioéconomique de la pandémie de COVID-19, et son impact probable sur la progression vers le DD, cet indice des efforts pour le DD intègre des questions pour évaluer la résilience et la durabilité des systèmes dans les secteurs clés du DD. Les éléments couverts par les questions liées à la pandémie de COVID-19 ci-dessous relèvent des dimensions clés des systèmes résilients et visent à apporter des informations déterminantes sur le potentiel d’une réponse efficace aux menaces de maladies infectieuses émergentes et autres crises de santé publique.

Veuillez noter chaque élément sur une échelle de 1 à 10, 1 étant le score le plus faible (faible état/capacité) et 10 le plus élevé (très bon état/capacité).

* Dimension physique : structures, équipements, états du système et capacités.

	1	2	3	4	5	6	7	8	9	10	Je ne sais pas
WE-M1 - Plan/Préparation - État et capacité de l’équipement, du personnel et de la structure du secteur de l’autonomisation des femmes avant la crise. (1 = faible état/capacité ; 10 = très bon état/capacité)	○	○	○	○	○	○	○	○	○	○	○
WE-M2 - Absorption – Mesure dans laquelle le secteur de l’autonomisation des femmes a reconnu la pandémie de COVID-19 et pu continuer à être fonctionnel.	○	○	○	○	○	○	○	○	○	○	○

	1	2	3	4	5	6	7	8	9	10	Je ne sais pas
WE-M3 - Absorption – Intégration : Niveau d'intégration des services et programmes d'autonomisation des femmes pour mitiger les effets de la pandémie de COVID-19 sur l'autonomie des femmes.	○	○	○	○	○	○	○	○	○	○	○
WE-M4 - Récupération – Mesure dans laquelle les programmes d'autonomisation des femmes ont été capables d'induire des changements pour récupérer un niveau de fonctionnalité pré-COVID-19.	○	○	○	○	○	○	○	○	○	○	○
WE-M5 - Récupération – Gestion du financement et des bailleurs de fonds : Niveau de mobilisation des ressources et de l'allocation budgétaire pour mitiger les effets de la pandémie de COVID-19 sur l'autonomie des femmes.	○	○	○	○	○	○	○	○	○	○	○
WE-M6 - Adaptation – Mesure dans laquelle des changements ont été effectués pour améliorer la résilience du secteur de l'autonomisation des femmes.	○	○	○	○	○	○	○	○	○	○	○

* Dimension informatique : création, gestion, stockage et utilisation des données.

	1	2	3	4	5	6	7	8	9	10	Je ne sais pas
WE-M7 - Plan/Préparation – Mesure dans laquelle les données liées à l'autonomisation des femmes étaient suffisamment préparées, présentées, analysées et stockées avant la crise.	○	○	○	○	○	○	○	○	○	○	○

	1	2	3	4	5	6	7	8	9	10	Je ne sais pas
WE-M8 - Absorption – Connaissance : Mesure dans laquelle le secteur de l'autonomisation des femmes a pu conduire des évaluations en temps réel de son état de fonctionnement, en anticipation des pertes en cascade des services liés à l'autonomie des femmes.	○	○	○	○	○	○	○	○	○	○	○
WE-M9 - Absorption – Connaissance/ Conscience : Capacité du système de surveillance à détecter les limites/ menaces posées à l'autonomie des femmes par la pandémie de COVID-19.	○	○	○	○	○	○	○	○	○	○	○
WE-M10 - Mesure dans laquelle les données sur l'autonomisation des femmes ont été utilisées pour suivre les progrès vers la récupération et anticiper des scenarii de récupération.	○	○	○	○	○	○	○	○	○	○	○
WE-M11 - Adaptation – Mesure dans laquelle le secteur de l'autonomisation des femmes crée et améliore ses protocoles de stockage et utilisation en temps réel de ses données sur l'autonomisation des femmes.	○	○	○	○	○	○	○	○	○	○	○

* Dimension cognitive : compréhension, modèles cognitifs, idées reçues, préjugés et valeurs.

	1	2	3	4	5	6	7	8	9	10	Je ne sais pas
WE-M12 - Planification/Préparation – Mesure dans laquelle le système de conception et les décisions opérationnelles étaient préparés à anticiper la crise.	○	○	○	○	○	○	○	○	○	○	○

	1	2	3	4	5	6	7	8	9	10	Je ne sais pas
WE-M13 - Absorption – Mesure dans laquelle le secteur de l'autonomisation des femmes a des protocoles de contingence suffisants et une gestion de crise proactive.	◯	◯	◯	◯	◯	◯	◯	◯	◯	◯	◯
WE-M14 - Absorption – Redevabilité : Niveau d'effort pour assurer une redevabilité optimale vis-à-vis des ressources allouées à l'autonomisation des femmes dans le cadre de la réponse à la pandémie de COVID-19. (Cela peut prendre la forme de soutien aux services atténués pour répondre aux besoins de protection des femmes et des filles).	◯	◯	◯	◯	◯	◯	◯	◯	◯	◯	◯
WE-M15 - Récupération – Mesure dans laquelle les décisions sont orientées sur la récupération et communiquées aux communautés sur la base de données probantes pour promouvoir des comportements sûrs	◯	◯	◯	◯	◯	◯	◯	◯	◯	◯	◯

	1	2	3	4	5	6	7	8	9	10	Je ne sais pas
WE-M16 - Récupération – Connaissance/Conscience : Mesure dans laquelle le secteur établit une certaine confiance à travers sa communication sur les barrières posées par la pandémie de COVID-19 pour soutenir l'accès et l'utilisation des services et programmes pour l'autonomisation des femmes. (Cela comprend la surveillance des disparités de genre dans les réponses à la pandémie et/ou la surveillance augmentée des questions liées à l'autonomie des femmes, comme la violence basée sur le genre).	○	○	○	○	○	○	○	○	○	○	○
WE-M17 - Adaptation – Mesure dans laquelle le secteur de l'autonomisation des femmes conçoit de nouvelles configurations systémiques, objectifs et critères de décision.	○	○	○	○	○	○	○	○	○	○	○
WE-M18 Adaptation - Niveau d'adaptation pour répondre aux menaces COVID-19 qui pèsent sur l'autonomisation des femmes	○	○	○	○	○	○	○	○	○	○	○

* Social dimension: interaction, collaboration and self-synchronization between people, entities and institutions.

	1	2	3	4	5	6	7	8	9	10	Je ne sais pas
WE-M19 - Planification /Préparation – Mesure dans laquelle une formation sur la gestion d'épidémies/crises a été menée et a permis de mettre à profit les réseaux sociaux, le capital social et les normes institutionnelles et culturelles avant la crise.	○	○	○	○	○	○	○	○	○	○	○
WE-M20 - Absorption – Mesure dans laquelle le personnel et les institutions sociales ont été accessibles et ont fait preuve d'ingéniosité dans leur réponse à l'épidémie/crise.	○	○	○	○	○	○	○	○	○	○	○
WE-M21 - Absorption - Self-regulation: Extent to which national leaders had the authority to effect timely sectoral changes through an infrastructure flexible.	○	○	○	○	○	○	○	○	○	○	○
WE-M22 - Récupération – Mesure dans laquelle le secteur de l'autonomisation des femmes est impliqué dans le partage de connaissances et d'équipes pour améliorer la récupération du système.	○	○	○	○	○	○	○	○	○	○	○
WE-M23 – Niveau d'engagement d'une équipe multidisciplinaire pour mitiger les effets de la pandémie de COVID-19 sur l'autonomisation des femmes.	○	○	○	○	○	○	○	○	○	○	○

	1	2	3	4	5	6	7	8	9	10	Je ne sais pas
WE-M24 - Adaptation - Leadership et management : Niveau de leadership démontré par les dirigeants du secteur de l'autonomisation des femmes pour mitiger les effets de la pandémie de COVID-19 sur l'autonomie des femmes.	○	○	○	○	○	○	○	○	○	○	○

* WE M-25 Temporalité : Mesure de la rapidité de la réaction du gouvernement –sa communication et mise en œuvre des mesures- pour mitiger l'impact immédiat et sur le long terme de la pandémie de COVID-19. Cette question porte sur la riposte nationale dans son ensemble, et non la réponse spécifique de votre secteur.

	1	2	3	4	5	6	7	8	9	10	Je ne sais pas
1-Plus faible -> Plus élevé-10	○	○	○	○	○	○	○	○	○	○	○

WE M-26 Commentaires : Veuillez utiliser cette section pour tout commentaire additionnel sur la résilience et la durabilité des opportunités et systèmes nationaux d'autonomisation des femmes et des filles en fonction de votre expérience de la pandémie de COVID-19. Si vous avez répondu « Je ne sais pas » à l'une des questions, utilisez aussi cette section pour expliquer pourquoi.

JOHNS HOPKINS
BLOOMBERG SCHOOL of PUBLIC HEALTH

Bill & Melinda Gates Institute for Population and Reproductive Health

Enquête de l'Indice des efforts pour le dividende démographique

Questionnaire sur le marché du travail (MT)

Répondez aux questions suivantes en fonction de votre expérience/ expertise dans ce secteur spécifique.

Afin d'obtenir une représentation synthétique des efforts nationaux pour le dividende démographique, notez les éléments suivants sur une échelle de 1 à 10, 1 étant le score le plus faible (efforts très faibles ou quasi-inexistants) et 10 le plus élevé (efforts robustes). Le cas échéant, si une politique ou activité n'existe pas, répondez 0.

Donnez un score à chaque élément. Toutes les réponses seront enregistrées au format illustré ci-dessous :

Effort le plus faible	1	2	3	4	5	6	7	8	9	10	Effort le plus élevé	Je ne sais pas

JOHNS HOPKINS
BLOOMBERG SCHOOL of PUBLIC HEALTH

Bill & Melinda Gates Institute for Population and Reproductive Health

Enquête de l'Indice des efforts pour le dividende démographique

Domaine 1. Politique/ Prise de décision

* LM 1.1 L'intégration de l'économie mondiale : Mesure dans laquelle le pays vise à s'intégrer à l'économie mondiale.

	1	2	3	4	5	6	7	8	9	10	Je ne sais pas
LM 1.1.1 Mesure dans laquelle le pays légifère pour l'ouverture des échanges commerciaux.	◯	◯	◯	◯	◯	◯	◯	◯	◯	◯	◯
LM 1.1.2 Niveau d'effort en faveur de la croissance des exportations nationales.	◯	◯	◯	◯	◯	◯	◯	◯	◯	◯	◯

* LM 1.2 L'intégration économique régionale : Mesure dans laquelle le pays vise à s'intégrer à l'économie régionale.

	1	2	3	4	5	6	7	8	9	10	Je ne sais pas
1-Plus faible -> Plus élevé-10	◯	◯	◯	◯	◯	◯	◯	◯	◯	◯	◯

* LM 1.3 La technologie : Mesure dans laquelle le gouvernement a investi dans l'infrastructure, à la fois traditionnelle et technologique, pour permettre au pays de saisir les opportunités de demain/ d'intégration technologique en complément de la main d'œuvre.

	1	2	3	4	5	6	7	8	9	10	Je ne sais pas
1-Plus faible -> Plus élevé-10	◯	◯	◯	◯	◯	◯	◯	◯	◯	◯	◯

* LM 1.4 Le soutien du gouvernement : Mesure dans laquelle des politiques et plans sont en place et prévoient des dispositifs de protection sociale pour une transition harmonieuse entre les différents emplois, ou « petits boulots », des personnes, et pour les protéger de la précarité/ volatilité des revenus.

	1	2	3	4	5	6	7	8	9	10	Je ne sais pas
LM 1.4.1 Mesure dans laquelle une politique nationale établissant un salaire minimum est en place.	◯	◯	◯	◯	◯	◯	◯	◯	◯	◯	◯
LM 1.4.2 Mesure dans laquelle une politique nationale définissant un système d'assurance chômage est en place, et niveau d'efficacité de sa mise en œuvre.	◯	◯	◯	◯	◯	◯	◯	◯	◯	◯	◯

* LM 1.5 Le chômage : Mesure dans laquelle les politiques rendent compte et répondent au chômage chez différents groupes de la population et dans différentes aires géographiques.

	1	2	3	4	5	6	7	8	9	10	Je ne sais pas
1-Plus faible -> Plus élevé-10	◯	◯	◯	◯	◯	◯	◯	◯	◯	◯	◯

* LM 1.6 Les groupes défavorisés : Mesure dans laquelle des politiques existent, et sont effectivement mises en œuvre, pour soutenir les opportunités d'embauche des groupes défavorisés (c'est-à-dire les filles et les femmes, les jeunes, les personnes en situation de mobilité réduite ou de handicap, etc.).

	1	2	3	4	5	6	7	8	9	10	Je ne sais pas
1-Plus faible -> Plus élevé-10	◯	◯	◯	◯	◯	◯	◯	◯	◯	◯	◯

* LM 1.7 Le changement climatique et le développement durable : Mesure dans laquelle les secteurs de l'agriculture et de la production industrielle sont préparés aux impacts des effets du changement climatique sur le marché du travail en élaborant des stratégies de planification familiale en vue des besoins du marché de travail de demain.

	1	2	3	4	5	6	7	8	9	10	Je ne sais pas
1-Plus faible -> Plus élevé-10	◯	◯	◯	◯	◯	◯	◯	◯	◯	◯	◯

* LM 1.8 L'urbanisation : Mesure dans laquelle les politiques et la planification anticipent et sont préparées à la croissance urbaine résultant et contribuant au changement climatique, et ses impacts particuliers sur les marchés du travail.

	1	2	3	4	5	6	7	8	9	10	Je ne sais pas
1-Plus faible -> Plus élevé-10	○	○	○	○	○	○	○	○	○	○	○

* LM 1.9 L'économie de services : Mesure dans laquelle des politiques et des cadres de travail sont en place pour faire passer les économies basées sur du travail informel au travail formalisé répondant aux demandes locales, régionales et mondiales de biens et de services.

	1	2	3	4	5	6	7	8	9	10	Je ne sais pas
1-Plus faible -> Plus élevé-10	○	○	○	○	○	○	○	○	○	○	○

* LM 1.10 La responsabilité sociale d'entreprise : Mesure dans laquelle la législation nationale du marché du travail utilise la responsabilité sociale des entreprises (RSE) pour soutenir l'entreprenariat chez les jeunes.

	1	2	3	4	5	6	7	8	9	10	Je ne sais pas
1-Plus faible -> Plus élevé-10	○	○	○	○	○	○	○	○	○	○	○

* LM 1.11 Les investissements ciblés dans le marché du travail : Mesure dans laquelle le gouvernement a ciblé ses investissements sur les secteurs générateurs d'emplois, y compris les Technologies de l'Information et la Communication (TIC), le secteur de production industrielle, l'agriculture et les entreprises industrielles agricoles, pour générer de l'emploi et encourager la croissance inclusive.

	1	2	3	4	5	6	7	8	9	10	Je ne sais pas
1-Plus faible -> Plus élevé-10	○	○	○	○	○	○	○	○	○	○	○

* LM 1.12 Les travailleurs indépendants : Mesure dans laquelle les politiques soutiennent l'élévation des retours sur investissement des travailleurs indépendants et la création de plus d'opportunités pour passer du travail indépendant à des emplois mieux rémunérés. (Cela comprend le travail en milieu rural).

	1	2	3	4	5	6	7	8	9	10	Je ne sais pas
1-Plus faible -> Plus élevé-10	○	○	○	○	○	○	○	○	○	○	○

* LM 1.13 Le microcrédit : Mesure dans laquelle le gouvernement permet l'accès à des microcrédits pour exercer des activités à but lucratif, sécurisant des crédits abordables pour les petites et moyennes entreprises et les travailleurs indépendants.

	1	2	3	4	5	6	7	8	9	10	Je ne sais pas
1-Plus faible -> Plus élevé-10	◯	◯	◯	◯	◯	◯	◯	◯	◯	◯	◯

* LM 1.14 L'épargne : Mesure dans laquelle le gouvernement soutient la création de produits financiers accessibles et des politiques promouvant la culture de l'épargne.

	1	2	3	4	5	6	7	8	9	10	Je ne sais pas
1-Plus faible -> Plus élevé-10	◯	◯	◯	◯	◯	◯	◯	◯	◯	◯	◯

* LM 1.15 L'acquisition de compétences : Mesure dans laquelle des politiques existent, et sont mises en œuvre efficacement, pour soutenir l'acquisition de compétences valorisées sur le marché du travail d'aujourd'hui et de demain.

	1	2	3	4	5	6	7	8	9	10	Je ne sais pas
1-Plus faible -> Plus élevé-10	◯	◯	◯	◯	◯	◯	◯	◯	◯	◯	◯

LM 1.16 Commentaires : Veuillez utiliser cette section pour nous faire part de tout commentaire additionnel sur le niveau d'effort, les défis ou les succès du pays autour de la politique/prise de décision dans le secteur du marché du travail. Si vous avez répondu « Je ne sais pas » à l'une des questions précédentes, veuillez utiliser cette section pour expliquer pourquoi.

JOHNS HOPKINS
BLOOMBERG SCHOOL of PUBLIC HEALTH

Bill & Melinda Gates Institute for Population and Reproductive Health

Enquête de l'Indice des efforts pour le dividende démographique

Domaine 2. Services ou programmes

* LM 2.1 La réduction du chômage : Mesure dans laquelle des efforts sont menés pour développer et mettre en œuvre des stratégies visant à réduire la proportion des personnes au chômage par année cible (en accord avec le Plan de mise en œuvre des dix premières années de l'Agenda 2063).

	1	2	3	4	5	6	7	8	9	10	Je ne sais pas
1-Plus faible -> Plus élevé-10	○	○	○	○	○	○	○	○	○	○	○

* LM 2.2. Le chômage chez les jeunes : Mesure dans laquelle des programmes liés au marché du travail ont effectivement réduit le taux de chômage chez les jeunes en particulier.

	1	2	3	4	5	6	7	8	9	10	Je ne sais pas
1-Plus faible -> Plus élevé-10	○	○	○	○	○	○	○	○	○	○	○

* LM 2.3 L'accès au microcrédit : Mesure dans laquelle les fonds nationaux et régionaux ont été établis et opérationnalisés pour augmenter l'accès des personnes au capital commercial abordable.

	1	2	3	4	5	6	7	8	9	10	Je ne sais pas
1-Plus faible -> Plus élevé-10	○	○	○	○	○	○	○	○	○	○	○

* LM 2.4 L'exploitation des capacités uniques des jeunes : Mesure dans laquelle des programmes et services soutiennent les talents spécifiques des jeunes pour les aider à s'épanouir et réaliser leur plein potentiel. (Cela couvre les objectifs programmatiques spécifiques liés à l'acquisition de talents).

	1	2	3	4	5	6	7	8	9	10	Je ne sais pas
1-Plus faible -> Plus élevé-10	○	○	○	○	○	○	○	○	○	○	○

* LM 2.5 Les opportunités de première embauche : Mesure dans laquelle les programmes et la législation du gouvernement engagent les partenaires du secteur privé à élargir les opportunités de stages, d'apprentissage professionnel et de formation continue au travail pour les femmes et les jeunes.

	1	2	3	4	5	6	7	8	9	10	Je ne sais pas
1-Plus faible -> Plus élevé-10	○	○	○	○	○	○	○	○	○	○	○

* LM 2.6 L'entrée des jeunes dans la population active : Mesure de la promotion du volontariat des jeunes, des programmes pour jeunes professionnelles et autres opportunités similaires pour améliorer leurs capacités et les exposer aux organisations régionales et internationales.

	1	2	3	4	5	6	7	8	9	10	Je ne sais pas
1-Plus faible -> Plus élevé-10	○	○	○	○	○	○	○	○	○	○	○

* LM 2.7 Les risques de l'entreprenariat : Mesure dans laquelle les personnes ont un meilleur accès aux services d'achat et de finance du gouvernement pour réduire les difficultés liées au lancement d'un projet professionnel et/ou à la conduite d'activités commerciales dans et entre les pays.

	1	2	3	4	5	6	7	8	9	10	Je ne sais pas
1-Plus faible -> Plus élevé-10	○	○	○	○	○	○	○	○	○	○	○

* LM 2.8 Les ressources pour l'entreprenariat des jeunes : Mesure dans laquelle les projets d'entreprenariat des jeunes bénéficient d'une mobilisation de ressources directe et ciblée, soutenue par un cadre régulateur, pour réduire les difficultés liées au lancement d'un projet professionnel et/ou à la conduite d'activités commerciales dans et entre les pays.

	1	2	3	4	5	6	7	8	9	10	Je ne sais pas
1-Plus faible -> Plus élevé-10	○	○	○	○	○	○	○	○	○	○	○

* LM 2.9 Les opportunités professionnelles : Mesure dans laquelle les opportunités de formation professionnelles sont amplifiées pour permettre l'acquisition de compétences et améliorer les capacités d'insertion professionnelle, la productivité et la compétitivité.

	1	2	3	4	5	6	7	8	9	10	Je ne sais pas
1-Plus faible -> Plus élevé-10	○	○	○	○	○	○	○	○	○	○	○

* LM 2.10 L’éducation et l’insertion professionnelle : Mesure dans laquelle des améliorations ont été apportées pour favoriser l’accès inclusif à l’éducation à tous les niveaux et offrir des alternatives viables à de nombreux jeunes, particulièrement les filles adolescentes, qui abandonnent le système d’éducation scolaire formel, en facilitant leur réinsertion, en restructurant l’éducation informelle et la formation via une certification standardisée dans et entre les pays.

	1	2	3	4	5	6	7	8	9	10	Je ne sais pas
1-Plus faible -> Plus élevé-10	○	○	○	○	○	○	○	○	○	○	○

* LM 2.11 Le travail « ami des femmes » : Mesure dans laquelle les lieux de travail dans les secteurs privé et public ont adopté des politiques et pratiques « amies des femmes » pour éliminer toute forme de discrimination basée sur le genre, tout en leur permettant de prendre pleinement part à la population active.

	1	2	3	4	5	6	7	8	9	10	Je ne sais pas
1-Plus faible -> Plus élevé-10	○	○	○	○	○	○	○	○	○	○	○

* LM 2.12 La sécurité de l’emploi :

	1	2	3	4	5	6	7	8	9	10	Je ne sais pas
LM 2.12.1 Mesure dans laquelle les périodes de préavis relatifs aux plans sociaux et au licenciement sont respectées.	○	○	○	○	○	○	○	○	○	○	○
LM 2.12.2 Mesure dans laquelle les indemnités de départ dans le cadre de plans sociaux ou de licenciements sont appliquées.	○	○	○	○	○	○	○	○	○	○	○

* LM 2.13 Le salaire minimum : Mesure dans laquelle un salaire minimum est appliqué au travailleur « type » (défini comme un salarié moyen, issu de groupes ethniques et religieux majoritaires, vivant dans les grandes villes, et non syndicalisé).

	1	2	3	4	5	6	7	8	9	10	Je ne sais pas
1-Plus faible -> Plus élevé-10	○	○	○	○	○	○	○	○	○	○	○

* LM 2.14 Les programmes du marché du travail : Mesure dans laquelle les programmes du marché du travail ont effectivement diversifié la main d'œuvre et les besoins du marché du travail, tout en diversifiant simultanément les compétences de cette main d'œuvre.

	1	2	3	4	5	6	7	8	9	10	Je ne sais pas
1-Plus faible -> Plus élevé-10	○	○	○	○	○	○	○	○	○	○	○

* LM 2.15 L'informalité sur le marché du travail : Mesure dans laquelle des programmes sont mis en œuvre pour transformer le travail informel en travail légal.

	1	2	3	4	5	6	7	8	9	10	Je ne sais pas
1-Plus faible -> Plus élevé-10	○	○	○	○	○	○	○	○	○	○	○

LM 2.16 Commentaires : Veuillez utiliser cette section pour nous faire part de tout commentaire additionnel sur le niveau d'effort, les défis ou les succès du pays autour des services et programmes dans le secteur du marché du travail. Si vous avez répondu « Je ne sais pas » à l'une des questions précédentes, veuillez utiliser cette section pour expliquer pourquoi.

JOHNS HOPKINS
BLOOMBERG SCHOOL
of PUBLIC HEALTH

Bill & Melinda Gates Institute for Population and Reproductive Health

Enquête de l’Indice des efforts pour le dividende démographique

Domaine 3. Plaidoyer

* LM 3.1 La parité sur le marché du travail : Mesure dans laquelle les groupes ou individus appellent, et voient des améliorations, en matière de parité et d’égalité hommes-femmes sur les lieux de travail.

	1	2	3	4	5	6	7	8	9	10	Je ne sais pas
1-Plus faible -> Plus élevé-10	○	○	○	○	○	○	○	○	○	○	○

* LM 3.2 L’égalité des jeunes sur le marché du travail : Mesure dans laquelle les groupes et les individus appellent à des changements du marché du travail et des régulations au bénéfice des populations jeunes rurales et urbaines.

	1	2	3	4	5	6	7	8	9	10	Je ne sais pas
1-Plus faible -> Plus élevé-10	○	○	○	○	○	○	○	○	○	○	○

* LM 3.3 Le plaidoyer pour le salaire minimum : Mesure dans laquelle les groupes de plaidoyer réussissent à inciter des lois pour le salaire minimum, ou une augmentation du salaire minimum si une telle loi existe déjà.

	1	2	3	4	5	6	7	8	9	10	Je ne sais pas
1-Plus faible -> Plus élevé-10	○	○	○	○	○	○	○	○	○	○	○

* LM 3.4 Le plaidoyer du gouvernement : Mesure dans laquelle les membres du gouvernement plaident pour des améliorations de ses propres structures et priorités. (Cela peut comprendre des progrès sur le marché du travail en matière d’inclusion, opportunités, programmes et droits des jeunes, et autres concepts similaires dans différentes populations).

	1	2	3	4	5	6	7	8	9	10	Je ne sais pas
1-Plus faible -> Plus élevé-10	○	○	○	○	○	○	○	○	○	○	○

* LM 3.5 Les protections sociales : Mesure dans laquelle il existe un plaidoyer pour la protection de l’emploi et l’assurance chômage.

	1	2	3	4	5	6	7	8	9	10	Je ne sais pas
1-Plus faible -> Plus élevé-10	◯	◯	◯	◯	◯	◯	◯	◯	◯	◯	◯

LM 3.6 Commentaires : Veuillez utiliser cette section pour nous faire part de tout commentaire additionnel sur le niveau d’effort, les défis ou les succès du pays autour du plaidoyer dans le secteur du marché du travail. Si vous avez répondu « Je ne sais pas » à l’une des questions précédentes, veuillez utiliser cette section pour expliquer pourquoi.

JOHNS HOPKINS
BLOOMBERG SCHOOL
of PUBLIC HEALTH

Bill & Melinda Gates Institute for Population and Reproductive Health

Enquête de l’Indice des efforts pour le dividende démographique

Domaine 4. Recherche

* LM 4.1 La force des observatoires/parties prenantes/groupes de travail dans le secteur du marché du travail : Mesure dans laquelle les parties prenantes du secteur du marché du travail (ex : groupe directif de parties prenantes, groupes de travail techniques, observatoire du marché du travail, groupe de coordination ou facilitation) :

	1	2	3	4	5	6	7	8	9	10	Je ne sais pas
LM 4.1.1 Ont un représentant du gouvernement, des institutions de formation, de la société civile, des organisations non gouvernementales et confessionnelles, des associations professionnelles, du secteur privé, etc.	○	○	○	○	○	○	○	○	○	○	○
LM 4.1.2 Se réunissent régulièrement, émettent des rapports et recommandent des politiques aux directions des ministères compétents.	○	○	○	○	○	○	○	○	○	○	○
LM 4.1.3 Ont un impact sur le marché du travail dans le pays.	○	○	○	○	○	○	○	○	○	○	○

* LM 4.2 La stratégie de recherche sur le marché du travail : Mesure dans laquelle les documents nationaux prévoient une stratégie/approche complète pour la recherche sur le marché du travail qui soit partagée entre partenaires.

	1	2	3	4	5	6	7	8	9	10	Je ne sais pas
1-Plus faible -> Plus élevé-10	○	○	○	○	○	○	○	○	○	○	○

* LM 4.3 Les partenaires de l'analyse du marché du travail :

	1	2	3	4	5	6	7	8	9	10	Je ne sais pas
LM 4.3.1 Mesure dans laquelle la collecte des données est entreprise par des agences gouvernementales.	○	○	○	○	○	○	○	○	○	○	○
LM 4.3.2 Mesure dans laquelle la collecte de données est entreprise par des institutions de recherche.	○	○	○	○	○	○	○	○	○	○	○
LM 4.3.3 Mesure dans laquelle la collecte des données est entreprise par des chercheurs indépendants.	○	○	○	○	○	○	○	○	○	○	○

* LM 4.4 Les domaines de la recherche sur le marché du travail :

	1	2	3	4	5	6	7	8	9	10	Je ne sais pas
LM 4.4.1 Mesure dans laquelle de la recherche est menée sur les tendances du chômage.	○	○	○	○	○	○	○	○	○	○	○
LM 4.4.2 Mesure dans laquelle de la recherche est menée sur les jeunes et le marché du travail.	○	○	○	○	○	○	○	○	○	○	○
LM 4.4.3 Mesure dans laquelle de la recherche est menée sur les femmes et le marché du travail.	○	○	○	○	○	○	○	○	○	○	○
LM 4.4.4 Mesure dans laquelle de la recherche est menée sur les tendances et les trajectoires des besoins du marché du travail.	○	○	○	○	○	○	○	○	○	○	○

* LM 4.5 La qualité/ couverture des données :

	1	2	3	4	5	6	7	8	9	10	Je ne sais pas
LM 4.5.1 Mesure dans laquelle un système statistique de routine fiable fournit des données périodiques sur le marché du travail.	○	○	○	○	○	○	○	○	○	○	○
LM 4.5.2 Mesure dans laquelle un système statistique de routine fiable fournit de bonnes données périodiques sur les besoins du marché du travail parmi les populations de différentes communautés.	○	○	○	○	○	○	○	○	○	○	○
LM 4.5.3 Mesure dans laquelle les systèmes de suivi et de surveillance sont évalués, et ces évaluations appliquées pour assurer la fiabilité des données.	○	○	○	○	○	○	○	○	○	○	○

* LM 4.6 Les registres : Mesure dans laquelle les systèmes de registre, rapports et retours/commentaires sur les résultats du marché du travail sont adéquats.

	1	2	3	4	5	6	7	8	9	10	Je ne sais pas
1-Plus faible -> Plus élevé-10	○	○	○	○	○	○	○	○	○	○	○

* LM 4.7 La qualité des institutions de recherche : Mesure dans laquelle le pays a la capacité de soutenir et maintenir les institutions de recherche qui développent des études/ collectent des données liées au marché du travail.

	1	2	3	4	5	6	7	8	9	10	Je ne sais pas
1-Plus faible -> Plus élevé-10	○	○	○	○	○	○	○	○	○	○	○

* LM 4.8 L'évaluation : Mesure dans laquelle les statistiques de programmes, enquêtes et études sont utilisées par du personnel spécialisé pour rapporter sur les opérations des programmes et mesurer le progrès.

	1	2	3	4	5	6	7	8	9	10	Je ne sais pas
1-Plus faible -> Plus élevé-10	○	○	○	○	○	○	○	○	○	○	○

* LM 4.9 L'utilisation des résultats d'évaluation par les décideurs : Mesure dans laquelle les gestionnaires locaux des programmes du marché du travail utilisent les conclusions de recherche et d'évaluations pour améliorer ces programmes en suivant les recommandations émises.

	1	2	3	4	5	6	7	8	9	10	Je ne sais pas
1-Plus faible -> Plus élevé-10	○	○	○	○	○	○	○	○	○	○	○

* LM 4.10 L'utilisation des résultats d'évaluation par les ministères : Mesure dans laquelle les administrateurs ministériels compétents utilisent systématiquement les données pour informer les politiques et interventions visant à résoudre les problèmes liés au marché du travail.

	1	2	3	4	5	6	7	8	9	10	Je ne sais pas
1-Plus faible -> Plus élevé-10	○	○	○	○	○	○	○	○	○	○	○

* LM 4.11 La dissémination d'informations à d'autres entités chargées de la mise en œuvre des programmes : Mesure dans laquelle des informations sont partagées ou disséminées entre géographies et à différents niveaux (national, état/province/comté, localités/sous-comtés).

	1	2	3	4	5	6	7	8	9	10	Je ne sais pas
1-Plus faible -> Plus élevé-10	○	○	○	○	○	○	○	○	○	○	○

LM 4.12 Commentaires : Veuillez utiliser cette section pour nous faire part de tout commentaire additionnel sur le niveau d'effort, les défis ou les succès du pays autour de la recherche dans le secteur du marché du travail. Si vous avez répondu « Je ne sais pas » à l'une des questions précédentes, veuillez utiliser cette section pour expliquer pourquoi.

Bill & Melinda Gates Institute for Population and Reproductive Health

Enquête de l'Indice des efforts pour le dividende démographique

Dimension 5. Organisations de la société civile (OSC)

* LM 5.1 Le pouvoir des acteurs des OSC : Mesure dans laquelle les acteurs des OSC sont bien placés pour influencer la politique et l'action nationale concernant le marché du travail.

	1	2	3	4	5	6	7	8	9	10	Je ne sais pas
1-Plus faible -> Plus élevé-10	○	○	○	○	○	○	○	○	○	○	○

* LM 5.2 L'analyse budgétaire comme outil des OSC : Mesure dans laquelle les OSC utilisent l'analyse budgétaire comme un outil pour développer le plaidoyer en matière de marché du travail.

	1	2	3	4	5	6	7	8	9	10	Je ne sais pas
1-Plus faible -> Plus élevé-10	○	○	○	○	○	○	○	○	○	○	○

* LM 5.3 Le soutien des services à base communautaire : Mesure dans laquelle les OSC participent à présenter les besoins, informations et services du marché du travail aux décideurs, et vice-versa.

	1	2	3	4	5	6	7	8	9	10	Je ne sais pas
1-Plus faible -> Plus élevé-10	○	○	○	○	○	○	○	○	○	○	○

* LM 5.4 La diversification des opportunités d'embauche : Mesure dans laquelle les OSC et le secteur privé travaillent en coordination avec le gouvernement pour assurer une création d'emplois optimale et une participation hommes-femmes équitable sur le marché du travail.

	1	2	3	4	5	6	7	8	9	10	Je ne sais pas
1-Plus faible -> Plus élevé-10	○	○	○	○	○	○	○	○	○	○	○

* LM 5.5 Les droits humains et la qualité de la mise en œuvre : Mesure dans laquelle les OSC soutiennent des approches fondées sur le droit sur les lieux de travail.

	1	2	3	4	5	6	7	8	9	10	Je ne sais pas
1-Plus faible -> Plus élevé-10	○	○	○	○	○	○	○	○	○	○	○

* LM 5.6 Les jeunes : Mesure dans laquelle les OSC soutiennent des politiques, interventions et programmes pour l'emploi des jeunes spécifiquement.

	1	2	3	4	5	6	7	8	9	10	Je ne sais pas
1-Plus faible -> Plus élevé-10	○	○	○	○	○	○	○	○	○	○	○

* LM 5.7 Les programmes pour les femmes : Mesure dans laquelle les OSC soutiennent des politiques, services et programmes pour l'emploi des femmes spécifiquement.

	1	2	3	4	5	6	7	8	9	10	Je ne sais pas
1-Plus faible -> Plus élevé-10	○	○	○	○	○	○	○	○	○	○	○

* LM 5.8 Le plaidoyer/ la redevabilité : Mesure dans laquelle les OSC soutiennent la formation et le renforcement de compétences des membres de la communauté pour augmenter leur compréhension des politiques, processus et activités, et pour renforcer leurs capacités à identifier les problèmes liés au marché du travail, résoudre collectivement ces problèmes et mener des actions de plaidoyer plus ciblées pour combattre le chômage.

	1	2	3	4	5	6	7	8	9	10	Je ne sais pas
1-Plus faible -> Plus élevé-10	○	○	○	○	○	○	○	○	○	○	○

* LM 5.9 L'évaluation et le suivi dirigés par les OSC : Mesure dans laquelle les OSC évaluent, suivent et émettent des rapports sur l'efficacité des politiques et programmes pour améliorer la redevabilité au niveau des politiques.

	1	2	3	4	5	6	7	8	9	10	Je ne sais pas
1-Plus faible -> Plus élevé-10	○	○	○	○	○	○	○	○	○	○	○

* LM 5.10 Les partenariats entre OSC : Mesure dans laquelle les OSC ont formé des alliances nationales et des partenariats régionaux pour renforcer le marché du travail.

	1	2	3	4	5	6	7	8	9	10	Je ne sais pas
1-Plus faible -> Plus élevé-10	◯	◯	◯	◯	◯	◯	◯	◯	◯	◯	◯

LM 5.11. Commentaires : Veuillez utiliser cette section pour nous faire part de tout commentaire additionnel sur le niveau d’effort, les défis ou les succès du pays autour des organisations de la société civile dans le secteur du marché du travail. Si vous avez répondu « Je ne sais pas » à l’une des questions précédentes, veuillez utiliser cette section pour expliquer pourquoi.

JOHNS HOPKINS
BLOOMBERG SCHOOL
of PUBLIC HEALTH

Bill & Melinda Gates Institute for Population and Reproductive Health

Enquête de l’Indice des efforts pour le dividende démographique

Module : Résilience et durabilité du secteur

Étant donné l’impact sanitaire et socioéconomique de la pandémie de COVID-19, et son impact probable sur la progression vers le DD, cet indice des efforts pour le DD intègre des questions pour évaluer la résilience et la durabilité des systèmes dans les secteurs clés du DD. Les éléments couverts par les questions liées à la pandémie de COVID-19 ci-dessous relèvent des dimensions clés des systèmes résilients et visent à apporter des informations déterminantes sur le potentiel d’une réponse efficace aux menaces de maladies infectieuses émergentes et autres crises de santé publique.

Veuillez noter chaque élément sur une échelle de 1 à 10, 1 étant le score le plus faible (faible état/capacité) et 10 le plus élevé (très bon état/capacité).

* Dimension physique : structures, équipements, états du système et capacités.

	1	2	3	4	5	6	7	8	9	10	Je ne sais pas
LM-M1 - Plan/Préparation - État et capacité de l’équipement, du personnel et de la structure du secteur du marché du travail avant la crise. (1 = faible état/capacité ; 10 = très bon état/capacité)	◯	◯	◯	◯	◯	◯	◯	◯	◯	◯	◯
LM-M2 - Absorption – Mesure dans laquelle le secteur du marché du travail a reconnu la pandémie de COVID-19 et pu continuer à maintenir un niveau d’emploi et une productivité économique optimale.	◯	◯	◯	◯	◯	◯	◯	◯	◯	◯	◯

	1	2	3	4	5	6	7	8	9	10	Je ne sais pas
LM-M3 - Récupération – Mesure dans laquelle le secteur du marché du travail été capable d'induire des changements pour récupérer un niveau de fonctionnalité pré-COVID-19.	○	○	○	○	○	○	○	○	○	○	○
LM-M4 - Récupération – Gestion du financement et des bailleurs de fonds : Mesure dans laquelle il existe des mécanismes établis de protection sociale (et autres formes de stabilisation des revenus) pour les travailleurs sans protection (ex : les travailleurs indépendants, qui non pas accès au congé payé.)	○	○	○	○	○	○	○	○	○	○	○
LM-M5 - Adaptation – Gestion du financement et des bailleurs de fonds : Mesure dans laquelle des réformes institutionnelles et politiques ont été initiées pour renforcer la résilience par une approche déterminée par la demande à travers des systèmes de protection sociale à grande échelle pouvant agir comme stabilisateurs économiques.	○	○	○	○	○	○	○	○	○	○	○

* Dimension informatique : création, gestion, stockage et utilisation des données.

	1	2	3	4	5	6	7	8	9	10	Je ne sais pas
LM-M6 - Plan/Préparation – Mesure dans laquelle les données liées au marché du travail étaient suffisamment préparées, présentées, analysées et stockées avant la crise.	◯	◯	◯	◯	◯	◯	◯	◯	◯	◯	◯
LM-M7 - Absorption – Connaissance : Mesure dans laquelle le secteur du marché du travail a pu conduire des évaluations en temps réel de son état de fonctionnement, en anticipation des pertes en cascade de la productivité du marché du travail.	◯	◯	◯	◯	◯	◯	◯	◯	◯	◯	◯
LM-M8 - Mesure dans laquelle les données du marché du travail ont été utilisées pour suivre les progrès vers la récupération et anticiper des scenarii de récupération.	◯	◯	◯	◯	◯	◯	◯	◯	◯	◯	◯
LM-M9 - Adaptation – Mesure dans laquelle le secteur du marché du travail crée et améliore ses protocoles de stockage et utilisation des données en temps réel.	◯	◯	◯	◯	◯	◯	◯	◯	◯	◯	◯

* Dimension cognitive : compréhension, modèles cognitifs, idées reçues, préjugés et valeurs.

	1	2	3	4	5	6	7	8	9	10	Je ne sais pas
LM-M10 - Planification/Préparation – Mesure dans laquelle le système de conception et les décisions opérationnelles du secteur du marché du travail étaient préparés à anticiper la crise.	◯	◯	◯	◯	◯	◯	◯	◯	◯	◯	◯

	1	2	3	4	5	6	7	8	9	10	Je ne sais pas
LM-M11 - Absorption – Mesure dans laquelle le secteur du marché du travail a des protocoles de contingence suffisants et une gestion de crise proactive.	○	○	○	○	○	○	○	○	○	○	○
LM-M12 - Absorption - Leadership et management : Mesure dans laquelle le gouvernement garantit aux salariés/ travailleurs et leurs famille une protection contre les risques sanitaires de la pandémie de COVID-19.	○	○	○	○	○	○	○	○	○	○	○
LM-M13 - Absorption – Redevabilité : Niveau d'effort pour assurer une redevabilité optimale vis-à-vis des ressources allouées à la protection contre les risques sanitaires de la pandémie de COVID-19 et la récupération du marché du travail dans le cadre de la réponse pour mitiger les impacts du COVID-19.	○	○	○	○	○	○	○	○	○	○	○
LM-M14 - Récupération – Mesure dans laquelle les décisions sont orientées vers la récupération et communiquées aux membres de la communauté sur la base de données probantes pour promouvoir des comportements sûrs.	○	○	○	○	○	○	○	○	○	○	○

	1	2	3	4	5	6	7	8	9	10	Je ne sais pas
LM-M15 - Récupération – Connaissance/Conscience : Mesure dans laquelle le secteur établit une certaine confiance et un dialogue entre les employeurs et les salariés pour renforcer l'engagement d'action commune du gouvernement et promouvoir le dialogue social au niveau des entreprises.	○	○	○	○	○	○	○	○	○	○	○
LM-M16 - Adaptation – Mesure dans laquelle le secteur du marché du travail conçoit de nouvelles configurations systémiques, objectifs et critères de décision.	○	○	○	○	○	○	○	○	○	○	○
LM-M17 - Adaptation – Adaptatif : Mesure dans laquelle le gouvernement promeut des changements sur les lieux de travail pour protéger les travailleurs (ex : mesures de sécurité et gestes barrières, prévention de la discrimination et de l'exclusion, accès élargi au congé payé, etc.).	○	○	○	○	○	○	○	○	○	○	○

* Dimension sociale : interaction, collaboration et auto-synchronisation entre les personnes, les entités et les institutions.

	1	2	3	4	5	6	7	8	9	10	Je ne sais pas
LM-M18 - Planification /Préparation – Mesure dans laquelle une formation sur la gestion d'épidémies/crises a été menée et a permis de mettre à profit les réseaux sociaux, le capital social et les normes institutionnelles et culturelles avant la crise.	○	○	○	○	○	○	○	○	○	○	○

	1	2	3	4	5	6	7	8	9	10	Je ne sais pas
LM-M19 - Absorption – Mesure dans laquelle le personnel et les institutions sociales ont été accessibles et ont fait preuve d’ingéniosité dans leur réponse à l’épidémie/crise.	○	○	○	○	○	○	○	○	○	○	○
LM-M20 - Absorption – Autorégulation : Mesure dans laquelle les dirigeants nationaux avaient l’autorité d’effectuer des changements sectoriels en temps opportun à travers une infrastructure du marché du travail flexible.	○	○	○	○	○	○	○	○	○	○	○
LM-M21 - Récupération – Mesure dans laquelle le secteur du marché du travail est impliqué dans le partage de connaissances et d’équipes pour améliorer la récupération du système.	○	○	○	○	○	○	○	○	○	○	○
LM-M22 - Récupération – Diversité : Mesure dans laquelle le gouvernement facilite une récupération solide et rapide du marché du travail en diversifiant les politiques favorisant à la fois l’offre (production de biens et services) et la demande (consommation et investissement).	○	○	○	○	○	○	○	○	○	○	○
LM-M23 - Adaptation – Mesure dans laquelle des ajouts ou modifications sont apportés aux institutions, politiques, formations, programmes et à la culture du secteur du marché du travail.	○	○	○	○	○	○	○	○	○	○	○

	1	2	3	4	5	6	7	8	9	10	Je ne sais pas
LM-M24 - Adaptation - Leadership et management : Mesure dans laquelle il y a eu un dialogue tripartite fluide entre le gouvernement, les organisations des travailleurs/salariés et celles des employeurs.	◯	◯	◯	◯	◯	◯	◯	◯	◯	◯	◯

* LM M-25 Temporalité : Mesure de la rapidité de la réaction du gouvernement –sa communication et mise en œuvre des mesures- pour mitiger l'impact immédiat et sur le long terme de la pandémie de COVID-19. Cette question porte sur la riposte nationale dans son ensemble, et non la réponse spécifique de votre secteur.

	1	2	3	4	5	6	7	8	9	10	Je ne sais pas
1-Plus faible -> Plus élevé-10	◯	◯	◯	◯	◯	◯	◯	◯	◯	◯	◯

LM M-26 Commentaires : Utilisez cette section pour apporter tout commentaire additionnel sur la résilience et la durabilité des systèmes du marché du travail en vous référant à votre expérience de la pandémie de COVID-19. Si vous avez répondu « Je ne sais pas » à l'une des questions, utilisez cette section pour expliquer pourquoi.

JOHNS HOPKINS
BLOOMBERG SCHOOL
of PUBLIC HEALTH

Bill & Melinda Gates Institute for Population and Reproductive Health

Enquête de l'Indice des efforts pour le dividende démographique

Questionnaire sur la gouvernance et les institutions économiques (GIE)

Répondez aux questions suivantes en fonction de votre expérience/ expertise dans ce secteur spécifique.

Afin d'obtenir une représentation synthétique des efforts nationaux pour le dividende démographique, notez les éléments suivants sur une échelle de 1 à 10, 1 étant le score le plus faible (efforts très faibles ou quasi-inexistants) et 10 le plus élevé (efforts robustes). Le cas échéant, si une politique ou activité n'existe pas, répondez 0.

Donnez un score à chaque élément. Toutes les réponses seront enregistrées au format illustré ci-dessous :

Effort le plus faible	1	2	3	4	5	6	7	8	9	10	Effort le plus élevé	Je ne sais pas

JOHNS HOPKINS
BLOOMBERG SCHOOL
of PUBLIC HEALTH

Bill & Melinda Gates Institute for Population and Reproductive Health

Enquête de l’Indice des efforts pour le dividende démographique

Domaine 1. Politique/ Prise de décision

* GEI 1.1 Les valeurs démocratiques : Mesure dans laquelle le gouvernement a ratifié, appliqué à son contexte national et mis en œuvre les instruments régionaux sur les valeurs communes, y compris ceux liés à la démocratie, les élections, la gouvernance et le droit à la santé.

	1	2	3	4	5	6	7	8	9	10	Je ne sais pas
1-Plus faible -> Plus élevé-10	○	○	○	○	○	○	○	○	○	○	○

* GEI 1.2 Le suivi des valeurs démocratiques : Mesure dans laquelle le gouvernement a créé des mécanismes de rapport régulier et périodique sur la mise en œuvre des instruments régionaux de valeurs communes à travers les mécanismes existants tels que les revues périodiques universelles du statut des instruments internationaux ou régionaux, ou à travers des entités indépendantes. (Cela peut prendre la forme de rapports réguliers sur le statut de la mise en œuvre).

	1	2	3	4	5	6	7	8	9	10	Je ne sais pas
1-Plus faible -> Plus élevé-10	○	○	○	○	○	○	○	○	○	○	○

* GEI 1.3 L’affirmation de la participation démocratique des jeunes : Mesure dans laquelle le gouvernement a explicitement abrogé toutes les lois et contraintes discriminatoires empêchant la pleine participation des jeunes au processus électoral à travers des lois électorales et constitutions inclusives. (Cela peut inclure le développement et/ou renforcement des programmes de leadership des jeunes et/ou le renforcement de compétences en management et leadership).

	1	2	3	4	5	6	7	8	9	10	Je ne sais pas
1-Plus faible -> Plus élevé-10	○	○	○	○	○	○	○	○	○	○	○

* GEI 1.4. La suppression des barrières légales auxquelles se heurtent les jeunes : Mesure dans laquelle le gouvernement a proscrit toutes les lois posant des barrières au plein exercice et à la pleine jouissance des droits fondamentaux des jeunes à participer pleinement aux processus de gouvernance démocratique au niveau continental, régional, national et communautaire/à la base. (Cela pourrait aussi inclure le soutien de l'intégration des questions de droits et de santé des jeunes et adolescents à l'agenda du gouvernement sur la croissance et la transformation du pays).

	1	2	3	4	5	6	7	8	9	10	Je ne sais pas
1-Plus faible -> Plus élevé-10	○	○	○	○	○	○	○	○	○	○	○

* GEI 1.5 La politique de la jeunesse : Mesure dans laquelle la politique de la jeunesse du pays reflète les paradigmes éducatifs, de formation et d'emploi les plus récents.

	1	2	3	4	5	6	7	8	9	10	Je ne sais pas
1-Plus faible -> Plus élevé-10	○	○	○	○	○	○	○	○	○	○	○

* GEI 1.6 L'affirmation de la participation à la vie démocratique/ révision de la loi coutumière : Mesure dans laquelle le gouvernement a examiné, révisé, amendé ou aboli toutes les lois, régulations, politiques, pratiques et coutumes ayant un effet discriminatoire sur les jeunes, particulièrement les filles et les jeunes filles, sans distinction aucune, s'est assuré que les dispositions des différents systèmes légaux soient conformes aux droits humains internationaux. (Cela doit impérativement inclure la protection contre les pratiques malfaisantes, telles que le mariage précoce ou forcé, la violence sexuelle et basée sur le genre [VBG], et la mutilation génitale féminine [MGF]).

	1	2	3	4	5	6	7	8	9	10	Je ne sais pas
1-Plus faible -> Plus élevé-10	○	○	○	○	○	○	○	○	○	○	○

* GEI 1.7 La participation des jeunes dans la prise de décision politique : Mesure dans laquelle le gouvernement veille à l'implication des jeunes leaders et à leur participation dans les processus de prise de décision politique, y compris la politique sur la jeunesse et l'éducation. (Cela peut inclure les efforts visant à établir un parlement ou une assemblée nationale de jeunes. Cela peut aussi inclure le soutien à la participation et au leadership des jeunes et adolescents dans la planification et les programmes de santé à tous les niveaux de gouvernance).

	1	2	3	4	5	6	7	8	9	10	Je ne sais pas
1-Plus faible -> Plus élevé-10	○	○	○	○	○	○	○	○	○	○	○

* GEI 1.8 L'État de droit : Mesure dans laquelle l'État de droit est formellement et légalement établi de manière équitable et mis en œuvre indépendamment par le pouvoir judiciaire.

	1	2	3	4	5	6	7	8	9	10	Je ne sais pas
1-Plus faible -> Plus élevé-10	○	○	○	○	○	○	○	○	○	○	○

* GEI 1.9 La transparence et la redevabilité : Mesure dans laquelle la législation nationale promeut la transparence et la redevabilité de toutes les branches et secteurs.

	1	2	3	4	5	6	7	8	9	10	Je ne sais pas
GEI 1.9.1 Mesure dans laquelle le public peut accéder aux informations législatives et aux registres sur les organisations publiques.	○	○	○	○	○	○	○	○	○	○	○
GEI 1.9.2 Mesure dans laquelle il existe des mécanismes de redevabilité rendant cette dernière obligatoire et des sanctions associées.	○	○	○	○	○	○	○	○	○	○	○

* GEI 1.10 La sécurité nationale : Mesure dans laquelle le pays/gouvernement est impliqué dans un conflit armé, qu'il soit international ou intérieur.

	1	2	3	4	5	6	7	8	9	10	Je ne sais pas
GEI 1.10.1 Mesure dans laquelle le pays souffre de tensions transfrontalières.	○	○	○	○	○	○	○	○	○	○	○
GEI 1.10.2 Mesure dans laquelle le pays contient des personnes internement déplacées et/ou des réfugiés.	○	○	○	○	○	○	○	○	○	○	○
GEI 1.10.3 Mesure dans laquelle les établissements éducatifs sont protégés et des opportunités d'apprentissage alternatives créées pour les enfants et les jeunes déplacées ou vivant en situation de conflit.	○	○	○	○	○	○	○	○	○	○	○

* GEI 1.11 Les droits : Mesure dans laquelle la constitution affirme certains droits : liberté d’expression, liberté d’association et d’assemblée, droits et libertés civiques, et protection contre la discrimination ethnique et religieuse ; et mesure dans laquelle la violation des droits humains par le gouvernement est improbable.

	1	2	3	4	5	6	7	8	9	10	Je ne sais pas
1-Plus faible -> Plus élevé-10	◯	◯	◯	◯	◯	◯	◯	◯	◯	◯	◯

* GEI 1.12 L’éducation et les valeurs démocratiques : Mesure dans laquelle les politiques, stratégies et programmes d’éducation promeuvent la liberté de penser, les valeurs démocratiques et l’éducation comme un droit fondamental/humain.

	1	2	3	4	5	6	7	8	9	10	Je ne sais pas
1-Plus faible -> Plus élevé-10	◯	◯	◯	◯	◯	◯	◯	◯	◯	◯	◯

* GEI 1.13 L’affirmation des environnements d’apprentissage sûrs : Mesure dans laquelle les politiques d’éducation garantissent des environnements d’apprentissage sûrs à travers la protection de l’enfance. (Cela peut se manifester par la protection de la sécurité des filles, l’interdiction des punitions corporelles, etc.).

	1	2	3	4	5	6	7	8	9	10	Je ne sais pas
1-Plus faible -> Plus élevé-10	◯	◯	◯	◯	◯	◯	◯	◯	◯	◯	◯

GEI 1.14 Commentaires : Utilisez cette section pour nous faire part de tout commentaire additionnel sur le niveau d’effort, les défis ou les succès du pays autour des politiques/ de la prise de décision dans le secteur de la gouvernance et des institutions économiques. Si vous avez répondu « Je ne sais pas » à l’une des questions précédentes, veuillez utiliser cette section pour expliquer pourquoi.

JOHNS HOPKINS
BLOOMBERG SCHOOL
of PUBLIC HEALTH

Bill & Melinda Gates Institute for Population and Reproductive Health

Enquête de l’Indice des efforts pour le dividende démographique

Domaine 2. Services ou programmes

* GEI 2.1 Renforcer les activités/ institutions pour la jeunesse : Mesure dans laquelle des efforts sont menés pour renforcer les formations, réseaux et organisations indépendantes de jeunes, y compris l’établissement de commissions indépendantes de jeunes et de syndicats d’étudiants au niveau national et infranational pour promouvoir les droits des jeunes.

	1	2	3	4	5	6	7	8	9	10	Je ne sais pas
1-Plus faible -> Plus élevé-10	○	○	○	○	○	○	○	○	○	○	○

* GEI 2.2 La nomination de jeunes leaders : Si et mesure dans laquelle un jeune représentant a été nommé pour diriger le plaidoyer et promouvoir la priorisation des questions concernant les jeunes (ex : éducation, formation et emploi) dans les espaces de prise de décision à différents niveaux, y compris au parlement.

	1	2	3	4	5	6	7	8	9	10	Je ne sais pas
1-Plus faible -> Plus élevé-10	○	○	○	○	○	○	○	○	○	○	○

* GEI 2.3 Les idéaux et aspirations des jeunes : Mesure dans laquelle les programmes sont conçus pour instituer le leadership des jeunes, et mesure dans laquelle les formations pour l’autonomisation des jeunes visent à approfondir les idéaux et aspirations culturelles des jeunes.

	1	2	3	4	5	6	7	8	9	10	Je ne sais pas
1-Plus faible -> Plus élevé-10	○	○	○	○	○	○	○	○	○	○	○

* GEI 2.4 Les services des jeunes : Mesure dans laquelle les programmes sont conçus pour répondre aux besoins spécifiques des jeunes. (Cela devrait prendre en compte le développement et la mise en œuvre de services de santé amis des jeunes et adolescents, entre autres programmes similaires).

	1	2	3	4	5	6	7	8	9	10	Je ne sais pas
1-Plus faible -> Plus élevé-10	○	○	○	○	○	○	○	○	○	○	○

* GEI 2.5 L’implication des jeunes dans les initiatives nationales : Mesure dans laquelle des efforts sont menés pour renforcer les initiatives existantes du pays en matière d’engagement et de participation des jeunes (e*x : au niveau de l’Union africaine, cela peut inclure le Corps des Jeunes Volontaires de l’Union Africaine, les Clubs de Jeunes de l’Union Africaine, et les activités de AGA-YES, dont le comité consultatif de jeunes, les procès fictifs et le dialogue annuel des jeunes sur la démocratie, les droits de l’Homme et la gouvernance).* (Cela peut inclure l’implication des jeunes et des adolescents dans l’assistance technique apportée aux ministères et entités gouvernementales fédérales ou régionales compétentes pour l’élaboration de politiques, plans et programmes qui intègrent les questions de santé prioritaires affectant ce groupe).

	1	2	3	4	5	6	7	8	9	10	Je ne sais pas
1-Plus faible -> Plus élevé-10	○	○	○	○	○	○	○	○	○	○	○

* GEI 2.6 La sécurité : Mesure dans laquelle la sécurité personnelle est garantie à travers des services/ activités de police fiables, contribuant à une perception générale positive de la sécurité des personnes dans les ménages, les communautés et en milieu scolaire. (Cela pourrait inclure la protection des jeunes contre tout type d’abus, négligence ou manque de respect dans l’utilisation des services de santé).

	1	2	3	4	5	6	7	8	9	10	Je ne sais pas
1-Plus faible -> Plus élevé-10	○	○	○	○	○	○	○	○	○	○	○

* GEI 2.7 La participation : Mesure dans laquelle les fonctions du gouvernement impliquent la participation des citoyens, des organisations de la société civile et autres acteurs intersectoriels pour promouvoir des élections démocratiques et le pouvoir effectif de gouverner.

	1	2	3	4	5	6	7	8	9	10	Je ne sais pas
1-Plus faible -> Plus élevé-10	○	○	○	○	○	○	○	○	○	○	○

* GEI 2.8 Le genre : Mesure dans laquelle les femmes participent à :

	1	2	3	4	5	6	7	8	9	10	Je ne sais pas
GEI 2.8.1 La gouvernance au niveau national	◯	◯	◯	◯	◯	◯	◯	◯	◯	◯	◯
GEI 2.8.2 La gouvernance au niveau infranational	◯	◯	◯	◯	◯	◯	◯	◯	◯	◯	◯
GEI 2.8.3 La gouvernance en milieu rural	◯	◯	◯	◯	◯	◯	◯	◯	◯	◯	◯
GEI 2.8.4 La gouvernance en milieu urbain	◯	◯	◯	◯	◯	◯	◯	◯	◯	◯	◯
GEI 2.8.5 Judiciaire	◯	◯	◯	◯	◯	◯	◯	◯	◯	◯	◯

* GEI 2.9 La promotion de l’égalité : Mesure dans laquelle la parité est promue par le gouvernement dans tous les aspects de la société. (Ceci peut inclure la parité et l’égalité dans l’éducation, devant la loi, dans le système de santé, à l’embauche et en termes de rémunération, entre autres facettes de la société).

	1	2	3	4	5	6	7	8	9	10	Je ne sais pas
1-Plus faible -> Plus élevé-10	◯	◯	◯	◯	◯	◯	◯	◯	◯	◯	◯

* GEI 2.10 La durabilité des opportunités économiques :

	1	2	3	4	5	6	7	8	9	10	Je ne sais pas
GEI 2.10.1 Mesure dans laquelle le gouvernement pourvoit et s'assure qu'un système de gestion publique solide soit réalisé.	○	○	○	○	○	○	○	○	○	○	○
GEI 2.10.2 Mesure dans laquelle le gouvernement pourvoit et s'assure qu'un environnement commercial solide soit réalisé.	○	○	○	○	○	○	○	○	○	○	○
GEI 2.10.3 Mesure dans laquelle le gouvernement pourvoit et s'assure qu'une infrastructure publique solide soit réalisée.	○	○	○	○	○	○	○	○	○	○	○
GEI 2.10.4 Mesure dans laquelle le gouvernement pourvoit et s'assure qu'un secteur rural fort pour le développement soit réalisé.	○	○	○	○	○	○	○	○	○	○	○

* GEI 2.11 Le développement humain :

	1	2	3	4	5	6	7	8	9	10	Je ne sais pas
GEI 2.11.1 Mesure dans laquelle le bien-être du public en général est adéquatement géré par le gouvernement (y compris, mais sans s'y limiter, les politiques et services de sécurité sociale, l'intégration socioéconomique et la protection sociale des jeunes, entre autres sujets). Cela peut aussi impliquer la mesure dans laquelle le gouvernement, ayant l'obligation de protéger, remplir et faire respecter les droits fondamentaux de santé, a donné la responsabilité de leur mise en œuvre à son cabinet ministériel.											
GEI 2.11.2 Mesure dans laquelle le gouvernement promeut le développement de compétences de la population sur les lieux de travail.											
GEI 2.11.3 Mesure dans laquelle le gouvernement promeut l'éducation, le développement de connaissances et des capacités de la population à travers l'école et des opportunités de formation professionnelle continue.											
GEI 2.11.4 Mesure dans laquelle le gouvernement promeut la santé et le bien-être de la population.											

GEI 2.12 Commentaires : Utilisez cette section pour nous faire part de tout commentaire additionnel sur le niveau d'effort, les défis ou les succès du pays autour des services ou programmes dans le secteur de la gouvernance et des institutions économiques. Si vous avez répondu « Je ne sais pas » à l'une des questions précédentes, veuillez utiliser cette section pour expliquer pourquoi.

JOHNS HOPKINS
BLOOMBERG SCHOOL of PUBLIC HEALTH

Bill & Melinda Gates Institute for Population and Reproductive Health

Enquête de l'Indice des efforts pour le dividende démographique

Domaine 3. Plaidoyer

* GEI 3.1 L'autonomisation des jeunes : Mesure dans laquelle l'autonomisation des jeunes a été améliorée, potentiellement à travers l'intégration de l'éducation civique au programme d'éducation scolaire nationale, aux plateformes médiatiques et autres canaux pour inculquer les principes de l'État de droit, les droits humains, et les devoirs et responsabilités individuelles. (Cela peut comprendre l'inclusion de la santé des jeunes et des adolescents et des questions relatives au droit dans l'agenda de croissance et transformation du pays et dans les programmes scolaires).

	1	2	3	4	5	6	7	8	9	10	Je ne sais pas
1-Plus faible -> Plus élevé-10	○	○	○	○	○	○	○	○	○	○	○

* GEI 3.2 Le plaidoyer du gouvernement : Mesure dans laque le gouvernement plaide pour des améliorations au sein de ses propres structures et priorités. (Ces améliorations peuvent comprendre des avancées vers l'inclusion, les opportunités, les programmes et les droits des jeunes, ou autres concepts similaires dans différentes populations.).

	1	2	3	4	5	6	7	8	9	10	Je ne sais pas
1-Plus faible -> Plus élevé-10	○	○	○	○	○	○	○	○	○	○	○

* GEI 3.3 Le plaidoyer non-gouvernemental : Mesure dans laquelle les acteurs du gouvernement plaident pour des améliorations des structures et priorités du gouvernement. (Ces améliorations peuvent comprendre des avancées vers l'inclusion les opportunités, les programmes et les droits des jeunes, ou autres concepts similaires dans différentes populations.).

	1	2	3	4	5	6	7	8	9	10	Je ne sais pas
1-Plus faible -> Plus élevé-10	○	○	○	○	○	○	○	○	○	○	○

* GEI 3.4 La voix du plaidoyer : Mesure dans laquelle les plaideurs se font entendre dans les discussions gouvernementales.

	1	2	3	4	5	6	7	8	9	10	Je ne sais pas
1-Plus faible -> Plus élevé-10	○	○	○	○	○	○	○	○	○	○	○

* GEI 3.5 La voix des secteurs clés du DD : Mesure dans laquelle le gouvernement et les parties prenantes sont engagés à établir des institutions durables pour assurer :

	1	2	3	4	5	6	7	8	9	10	Je ne sais pas
GEI 3.5.1 Bonne gouvernance	○	○	○	○	○	○	○	○	○	○	○
GEI 3.5.2 Stabilité financière	○	○	○	○	○	○	○	○	○	○	○
GEI 3.5.3 Programmes optimaux de planification familiale	○	○	○	○	○	○	○	○	○	○	○
GEI 3.5.4 Programmes optimaux de santé maternelle et infantile	○	○	○	○	○	○	○	○	○	○	○
GEI 3.5.5 Éducation de bonne qualité	○	○	○	○	○	○	○	○	○	○	○
GEI 3.5.6 Marché du travail diversifié	○	○	○	○	○	○	○	○	○	○	○

GEI 3.6 Commentaires : Utilisez cette section pour nous faire part de tout commentaire additionnel sur le niveau d’effort, les défis ou les succès du pays autour du plaidoyer dans le secteur de la gouvernance et des institutions économiques. Si vous avez répondu « Je ne sais pas » à l’une des questions précédentes, veuillez utiliser cette section pour expliquer pourquoi.

JOHNS HOPKINS
BLOOMBERG SCHOOL
of PUBLIC HEALTH

Bill & Melinda Gates Institute for Population and Reproductive Health

Enquête de l'Indice des efforts pour le dividende démographique

Domaine 4. Recherche

* GEI 4.1 La force des observatoires/parties prenantes/groupes de travail sur la gouvernance et les institutions économiques (GIE) : Mesure dans laquelle les parties prenantes de la gouvernance et des institutions économiques du pays (ex : groupe directif de parties prenantes, groupes de travail techniques, observatoire de la gouvernance, groupe de coordination ou facilitation) :

	1	2	3	4	5	6	7	8	9	10	Je ne sais pas
GEI 4.1.1 Ont un représentant du gouvernement, des institutions de formation, de la société civile, des organisations non gouvernementales et confessionnelles, des associations professionnelles, du secteur privé, etc.	◯	◯	◯	◯	◯	◯	◯	◯	◯	◯	◯
GEI 4.1.2 Se réunissent régulièrement, émettent des rapports et recommandent des politiques aux directions des ministères compétents.	◯	◯	◯	◯	◯	◯	◯	◯	◯	◯	◯
GEI 4.1.3 Ont un impact sur la PF dans le pays.	◯	◯	◯	◯	◯	◯	◯	◯	◯	◯	◯

* GEI 4.2 La stratégie de recherche sur la gouvernance : Mesure dans laquelle les documents de planification nationale comprennent une stratégie/approche complète de recherche liée à la GIE.

	1	2	3	4	5	6	7	8	9	10	Je ne sais pas
1-Plus faible -> Plus élevé-10	◯	◯	◯	◯	◯	◯	◯	◯	◯	◯	◯

* GEI 4.3 Les partenaires collectant des données sur la gouvernance, la finance et le développement :

	1	2	3	4	5	6	7	8	9	10	Je ne sais pas
GEI 4.3.1 Mesure dans laquelle la collecte des données est entreprise par des agences gouvernementales. (Cela peut comprendre les agences/bureaux de la statistique et/ou les ministères de l'économie/finances, éducation, santé, genre, etc.).											
GEI 4.3.2 Mesure dans laquelle la collecte de données est entreprise par des institutions de recherche.											
GEI 4.3.3 Mesure dans laquelle la collecte des données est entreprise par des chercheurs indépendants.											

* GEI 4.4 La recherche thématique :

	1	2	3	4	5	6	7	8	9	10	Je ne sais pas
GEI 4.4.1 Mesure dans laquelle de la recherche est menée sur la sécurité et l'État de droit.											
GEI 4.4.2 Mesure dans laquelle la participation politique et les droits humains sont mesurés.											
GEI 4.4.3 Mesure dans laquelle de la recherche est menée sur les indicateurs de l'économie durable et est disséminée à la communauté.											
GEI 4.4.4 Mesure dans laquelle de la recherche est menée sur le développement du capital humain (éducation, formation et emploi, etc.).											

* GEI 4.5 La qualité/couverture des données :

	1	2	3	4	5	6	7	8	9	10	Je ne sais pas
GEI 4.5.1 Mesure dans laquelle le système statistique de routine fournit des informations périodiques sur la GIE.	○	○	○	○	○	○	○	○	○	○	○
GEI 4.5.2 Mesure dans laquelle le système statistique de routine fournit des informations périodiques fiables sur les besoins des populations de différentes communautés en matière de gouvernance.	○	○	○	○	○	○	○	○	○	○	○
GEI 4.5.3 Mesure dans laquelle des évaluations des systèmes de suivi et surveillance sont conduites et appliquées pour assurer la fiabilité des données.	○	○	○	○	○	○	○	○	○	○	○

* GEI 4.6 La qualité des institutions de recherche : Mesure dans laquelle le pays a la capacité de soutenir et maintenir des institutions de recherche qui développent des études et/ou collectent des données permettant de soutenir la bonne gouvernance, la stabilité de l'économie et la solidité des institutions publiques.

	1	2	3	4	5	6	7	8	9	10	Je ne sais pas
1-Plus faible -> Plus élevé-10	○	○	○	○	○	○	○	○	○	○	○

* GEI 4.7 L'évaluation : Mesure dans laquelle les statistiques, enquêtes et études sont utilisées par du personnel spécialisé pour générer des rapports sur la bonne gouvernance et les institutions financières

	1	2	3	4	5	6	7	8	9	10	Je ne sais pas
1-Plus faible -> Plus élevé-10	○	○	○	○	○	○	○	○	○	○	○

* GEI 4.8 L'utilisation des résultats d'évaluation par les gestionnaires de programmes : Mesure dans laquelle les gestionnaires locaux de programmes utilisent les conclusions de recherche et d'évaluations pour améliorer l'État de droit, stabiliser l'économie et renforcer les institutions en suivant les recommandations émises.

	1	2	3	4	5	6	7	8	9	10	Je ne sais pas
1-Plus faible -> Plus élevé-10	○	○	○	○	○	○	○	○	○	○	○

For peer review only - http://bmjopen.bmj.com/site/about/guidelines.xhtml

* GEI 4.10 La dissémination d'informations à d'autres entités chargées de la mise en œuvre des programmes : Mesure dans laquelle des informations sont partagées ou disséminées entre géographies et à différents niveaux (national, état/province/comté, localités/sous-comtés).

	1	2	3	4	5	6	7	8	9	10	Je ne sais pas
1-Plus faible -> Plus élevé-10	○	○	○	○	○	○	○	○	○	○	○

GEI 4.11 Commentaires : Utilisez cette section pour nous faire part de tout commentaire additionnel sur le niveau d'effort, les défis ou les succès du pays autour de la recherche sur la gouvernance et les institutions économiques. Si vous avez répondu « Je ne sais pas » à l'une des questions précédentes, veuillez utiliser cette section pour expliquer pourquoi.

JOHNS HOPKINS
BLOOMBERG SCHOOL of PUBLIC HEALTH

Bill & Melinda Gates Institute for Population and Reproductive Health

Enquête de l’Indice des efforts pour le dividende démographique

Domaine 5. Organisations de la société civile (OSC)

* GEI 5.1 Le pouvoir des acteurs des OSC : Mesure dans laquelle les acteurs des OSC occupent des postes d’influence pour contribuer positivement à l’État de droit.

	1	2	3	4	5	6	7	8	9	10	Je ne sais pas
1-Plus faible -> Plus élevé-10											

* GEI 5.2 L’analyse budgétaire comme outil des OSC : Mesure dans laquelle les OSC utilisent l’analyse budgétaire comme un outil pour développer le plaidoyer pour la bonne gouvernance, la stabilité de l’économie et la consolidation des institutions publiques.

	1	2	3	4	5	6	7	8	9	10	Je ne sais pas
1-Plus faible -> Plus élevé-10											

* GEI 5.3 Le soutien aux services à base communautaire : Mesure dans laquelle les OSC participent au rapprochement des informations et services liés à la gouvernance avec les communautés.

	1	2	3	4	5	6	7	8	9	10	Je ne sais pas
1-Plus faible -> Plus élevé-10											

* GEI 5.4 Les droits humains et la qualité de la mise en œuvre : Mesure dans laquelle les OSC plaident et soutiennent des approches de gouvernance fondées sur le droit pour veiller à ce que les bénéficiaires de projets et programmes puissent voir des avancées selon leurs propres termes.

	1	2	3	4	5	6	7	8	9	10	Je ne sais pas
1-Plus faible -> Plus élevé-10											

* GEI 5.5 Les jeunes : Mesure dans laquelle les OSC aident les jeunes à participer à l'éducation, la formation, l'emploi et le leadership.

	1	2	3	4	5	6	7	8	9	10	Je ne sais pas
1-Plus faible -> Plus élevé-10	◯	◯	◯	◯	◯	◯	◯	◯	◯	◯	◯

* GEI 5.6 Les programmes pour les femmes : Mesure dans laquelle les OSC veillent à ce que les politiques de GIE soutiennent la participation des femmes et des filles au leadership.

	1	2	3	4	5	6	7	8	9	10	Je ne sais pas
1-Plus faible -> Plus élevé-10	◯	◯	◯	◯	◯	◯	◯	◯	◯	◯	◯

* GEI 5.7 Le plaidoyer/ redevabilité :

	1	2	3	4	5	6	7	8	9	10	Je ne sais pas
GEI 5.7.1 Mesure dans laquelle les OSC et les médias soutiennent la participation de la communauté dans la gouvernance et l'application de l'État de droit.	◯	◯	◯	◯	◯	◯	◯	◯	◯	◯	◯
GEI 5.7.2 Mesure dans laquelle les médias soutiennent la participation de la communauté à la gouvernance ou au respect de l'État de droit.	◯	◯	◯	◯	◯	◯	◯	◯	◯	◯	◯

* GEI 5.8 Les évaluations et le suivi menés par les OSC : Mesure dans laquelle les OSC évaluent, suivent et émettent des rapports sur l'efficacité des politiques et des mécanismes et structures de redevabilité du gouvernement.

	1	2	3	4	5	6	7	8	9	10	Je ne sais pas
1-Plus faible -> Plus élevé-10	◯	◯	◯	◯	◯	◯	◯	◯	◯	◯	◯

* GEI 5.9 Les partenariats entre OSC : Mesure dans laquelle les OSC ont formé des alliances nationales et des partenariats régionaux pour renforcer leur position et potentiellement améliorer le leadership et les mécanismes de financement pour une bonne gouvernance et le renforcement institutionnel.

	1	2	3	4	5	6	7	8	9	10	Je ne sais pas
1-Plus faible -> Plus élevé-10	◯	◯	◯	◯	◯	◯	◯	◯	◯	◯	◯

GEI 5.10 Commentaires : Utilisez cette section pour nous faire part de tout commentaire additionnel sur le niveau d’effort, les défis ou les succès du pays autour des OSC dans le secteur de la gouvernance et des institutions économiques. Si vous avez répondu « Je ne sais pas » à l’une des questions précédentes, veuillez utiliser cette section pour expliquer pourquoi.

JOHNS HOPKINS
BLOOMBERG SCHOOL
of PUBLIC HEALTH

Bill & Melinda Gates Institute for Population and Reproductive Health

Enquête de l'Indice des efforts pour le dividende démographique

Module : Résilience et durabilité du secteur

Étant donné l'impact sanitaire et socioéconomique de la pandémie de COVID-19, et son impact probable sur la progression vers le DD, cet indice des efforts pour le DD intègre des questions pour évaluer la résilience et la durabilité des systèmes dans les secteurs clés du DD. Les éléments couverts par les questions liées à la pandémie de COVID-19 ci-dessous relèvent des dimensions clés des systèmes résilients et visent à apporter des informations déterminantes sur le potentiel d'une réponse efficace aux menaces de maladies infectieuses émergeantes et autres crises de santé publique.

Veuillez noter chaque élément sur une échelle de 1 à 10, 1 étant le score le plus faible (faible état/capacité) et 10 le plus élevé (très bon état/capacité).

* Dimension physique : structures, équipements, états du système et capacités.

	1	2	3	4	5	6	7	8	9	10	Je ne sais pas
GEI-M1 – Planification/Préparation : État et capacité de l'équipement, du personnel et de la structure du secteur GIE avant la crise. (1 = faible état/capacité ; 10 = très bon état/capacité)	◯	◯	◯	◯	◯	◯	◯	◯	◯	◯	◯
GEI-M2 - Absorption - Mesure dans laquelle le système du secteur GIE a reconnu la pandémie de COVID-19 et pu maintenir sa fonctionnalité.	◯	◯	◯	◯	◯	◯	◯	◯	◯	◯	◯

	1	2	3	4	5	6	7	8	9	10	Je ne sais pas
GEI-M3 - Absorption – Intégration : Niveau d'intégration des services et collaborations institutionnelles pour assurer la continuité de l'accès aux services essentiels pendant la crise COVID-19.	○	○	○	○	○	○	○	○	○	○	○
GEI-M4 - Récupération – Mesure dans laquelle les institutions économiques et gouvernementales ont été capables de générer des changements pour récupérer un niveau de fonctionnalité pré-COVID-19.	○	○	○	○	○	○	○	○	○	○	○
GEI-M5 - Récupération – Gestion du financement et des bailleurs de fonds : Banque nationale et soutien des bailleurs de fonds pour assurer la disponibilité de liquidités pendant la crise COVID-19.	○	○	○	○	○	○	○	○	○	○	○
GEI-M6 - Adaptation – Mesure dans laquelle des changements ont été apportés pour améliorer la résilience du système et de l'infrastructure du secteur GIE.	○	○	○	○	○	○	○	○	○	○	○

* Dimension informatique : création, gestion, stockage et utilisation des données.

	1	2	3	4	5	6	7	8	9	10	Je ne sais pas
GEI-M7 - Plan/Préparation – Mesure dans laquelle les données du secteur GIE étaient suffisamment préparées, présentées, analysées et stockées avant la crise.	○	○	○	○	○	○	○	○	○	○	○
GEI-M8 - Absorption – Connaissance : Niveau de connaissance/conscience des menaces causées par la pandémie de COVID-19 dans le gouvernement central.	○	○	○	○	○	○	○	○	○	○	○
GEI-M9 - Récupération – Mesure dans laquelle les données de GIE ont été utilisées pour suivre le progrès vers la récupération et anticiper des scenarii de récupération.	○	○	○	○	○	○	○	○	○	○	○
GEI-M10 - Adaptation – Mesure dans laquelle le système du secteur GIE crée et améliore actuellement ses protocoles de stockage et d'utilisation des données en temps réel.	○	○	○	○	○	○	○	○	○	○	○

* Dimension cognitive : compréhension, modèles cognitifs, idées reçues, préjugés et valeurs.

	1	2	3	4	5	6	7	8	9	10	Je ne sais pas
GEI-M11 - Planification/Préparation – Mesure dans laquelle le système du secteur GIE et les décisions opérationnelles étaient préparés à anticiper la crise	○	○	○	○	○	○	○	○	○	○	○

	1	2	3	4	5	6	7	8	9	10	Je ne sais pas
GEI-M12 - Absorption – Mesure dans laquelle la réponse du secteur GIE a des protocoles de contingence suffisants et une gestion de crise proactive.	○	○	○	○	○	○	○	○	○	○	○
GEI-M13 - Absorption – Redevabilité : Niveau d'effort pour assurer une redevabilité optimale vis-à-vis des ressources allouées pour soutenir la gouvernance dans le cadre de la réponse à la pandémie de COVID-19.	○	○	○	○	○	○	○	○	○	○	○
GEI-M14 - Récupération – Mesure dans laquelle les décisions sont orientées vers la récupération et communiquées aux communautés sur la base de données probantes pour promouvoir des comportements sûrs.	○	○	○	○	○	○	○	○	○	○	○
GEI-M15 - Récupération – Connaissance/Conscience : Mesure dans laquelle le secteur de la PF établit une certaine confiance avec le public à travers sa communication et le dialogue pour la prise de décision et l'action politique fondées sur des données probantes.	○	○	○	○	○	○	○	○	○	○	○
GEI-M16 - Adaptation – Mesure dans laquelle le système du secteur GIE conçoit de nouvelles configurations systémiques, objectifs et critères de décision.	○	○	○	○	○	○	○	○	○	○	○
GEI-M17 - Adaptation – Adaptatif : Niveau d'adaptation des systèmes financiers et des services sociaux pour assurer et faciliter l'accès aux plateformes technologiques pendant la pandémie de COVID-19.	○	○	○	○	○	○	○	○	○	○	○

* Social dimension: interaction, collaboration and self-synchronization between people, entities and institutions.

	1	2	3	4	5	6	7	8	9	10	Je ne sais pas
GEI-M18 - Planification /Préparation – Mesure dans laquelle une formation sur la gestion d'épidémies/crises a été menée et a permis de mettre à profit les réseaux sociaux, le capital social et les normes institutionnelles et culturelles avant la crise.	○	○	○	○	○	○	○	○	○	○	○
GEI-M19 - Absorption – Mesure dans laquelle le personnel et les institutions sociales ont été accessibles et ont fait preuve d'ingéniosité dans leur réponse à l'épidémie/crise.	○	○	○	○	○	○	○	○	○	○	○
GEI-M20 - Absorption - Self-regulation: Extent to which national leaders had the authority to effect timely sectoral changes through an infrastructure flexible.	○	○	○	○	○	○	○	○	○	○	○
GEI-M21 - Récupération – Mesure dans laquelle le secteur GIE est impliqué dans le partage de connaissances et d'équipes pour améliorer la récupération du système.	○	○	○	○	○	○	○	○	○	○	○
GEI-M22 - Récupération – Diversité : Niveau d'engagement d'une équipe multidisciplinaire pour mitiger les effets de la pandémie de COVID-19 au niveau national et infranational.	○	○	○	○	○	○	○	○	○	○	○
GEI-M23 - Adaptation - Leadership et management : Gérance et gestion démontrées par le gouvernement en réponse à la pandémie de COVID-19 au niveau national.	○	○	○	○	○	○	○	○	○	○	○

* GEI M-24 Temporalité : Mesure de la rapidité de la réaction du gouvernement –sa communication et mise en œuvre des mesures- pour mitiger l'impact immédiat et sur le long terme de la pandémie de COVID-19. *Cette question porte sur la riposte nationale dans son ensemble, et non la réponse spécifique de votre secteur.*

	1	2	3	4	5	6	7	8	9	10	Je ne sais pas
1-Plus faible -> Plus élevé-10	○	○	○	○	○	○	○	○	○	○	○

GEI M-25 Commentaires : Utilisez cette section pour apporter tout commentaire additionnel sur la résilience et la durabilité des systèmes nationaux de gouvernance et des institutions économiques en vous référant à votre expérience de la pandémie de COVID-19. Si vous avez répondu « Je ne sais pas » à l'une des questions, utilisez cette section pour expliquer pourquoi.

Appendix 3. Cronbach's Alpha analysis

a) Sector: Family Planning

	Ethiopia	Kenya	Nigeria	Rwanda	Senegal	Tanzania
			**Item rest correlation**			
**Family Planning**						
**Policy**						
**N**	**13**	**10**	**16**	**7**	**4**	**25**
**Cronbach's Alpha**	**0.79**	**0.90**	**0.95**	**0.94**	**0.84**	**0.72**
FP1_1	**0.32**	0.66	0.80	0.84	0.79	**0.38**
FP1_2	**0.11**	0.63	**0.34**	0.84	0.65	**0.26**
FP1_3	0.45	0.59	0.89	0.71	**0.38**	0.41
FP1_4	0.72	0.48	0.71	0.52	0.87	**0.20**
FP1_5	0.59	0.68	0.78	0.80	0.66	0.64
FP1_6	0.73	0.45	0.90	0.77	0.69	0.62
FP1_7	0.48	0.70	0.72	0.75	0.60	**0.32**
FP1_8	0.46	0.66	0.78	0.53	**0.08**	**0.28**
FP1_9	0.55	0.45	0.57	0.79	**0.23**	**0.03**
FP1_10	0.69	0.53	0.75	0.73	**0.29**	0.41
FP1_11a	**0.05**	0.55	0.67	0.75	**0.00**	0.51
FP1_11b	0.43	**0.19**	0.57	0.75	**0.39**	**0.06**
FP1_12	**0.01**	0.50	0.61	0.77	**0.24**	**0.30**
FP1_13	**0.06**	0.70	0.79	0.62	**0.33**	0.50
FP1_14	0.62	0.79	0.80	0.62	**0.08**	**0.35**
**Services and Programs**						
**N**	**12**	**8**	**6**	**6**	**4**	**17**
**Cronbach's Alpha**	**0.94**	**0.86**	**0.96**	**0.95**	**0.91**	**0.94**
FP2_1	0.49	0.69	0.80	**0.27**	0.62	**0.40**
FP2_2	**0.31**	**0.40**	0.61	0.67	0.73	**0.26**
FP2_3	0.59	0.52	0.64	0.68	**0.29**	**0.29**
FP2_4	0.55	**0.31**	0.60	**0.04**	0.54	0.43
FP2_5	0.41	**0.39**	0.59	**0.28**	**0.07**	0.45
FP2_6a	0.79	**0.32**	0.87	0.45	**0.38**	0.71
FP2_6b	0.71	**0.04**	0.79	0.45	0.96	0.62
FP2_6c	0.84	**0.02**	0.80	0.45	0.92	0.51
FP2_6d	0.72	**0.20**	0.71	0.45	0.46	0.53
FP2_6e	**0.40**	0.63	0.43	0.45	**0.26**	0.60
FP2_6f	0.53	0.67	**0.33**	0.45	**0.03**	0.55
FP2_6g	0.45	**0.25**	0.62	0.83	0.81	**0.37**
FP2_6h	**0.34**	**0.11**	0.67	0.56	**0.05**	0.46
FP2_6i	0.69	**0.33**	0.47	0.62	0.90	0.40
FP2_6j	0.68	0.40	0.61	0.95	0.45	0.51
FP2_7a	0.77	**0.04**	0.81	0.88	0.83	0.75

For peer review only - http://bmjopen.bmj.com/site/about/guidelines.xhtml

FP2_7b	0.60	**0.03**	0.84	0.88	**0.20**	0.69
FP2_7c	0.73	**0.04**	0.80	0.88	**0.31**	0.70
FP2_7d	0.80	**0.13**	0.83	0.88	0.79	0.74
FP2_7e	0.65	**0.25**	0.74	0.88	**0.37**	0.41
FP2_7f	**0.27**	**0.15**	0.63	0.88	**0.25**	0.56
FP2_7g	**0.37**	**0.21**	0.49	0.83	0.81	0.42
FP2_8a	0.66	**0.29**	0.46	0.62	**0.10**	0.70
FP2_8b	0.62	0.55	**0.32**	0.61	0.51	0.61
FP2_8c	0.63	**0.05**	0.79	0.61	**0.29**	0.73
FP2_8d	0.67	**0.06**	0.78	0.61	**0.31**	0.78
FP2_9	0.64	**0.39**	0.75	0.50	0.65	0.68
FP2_10	**0.29**	**0.36**	0.63	0.55	**0.17**	**0.31**
FP2_11	0.59	**0.39**	0.57	0.55	0.56	0.75
FP2_12	0.76	0.44	0.66	0.67	0.64	0.74
FP2_13	0.93	0.43	0.73	0.55	0.72	0.83
FP2_14	0.81	0.52	0.71	**0.32**	0.71	0.62
FP2_15	0.95	0.46	0.48	**0.29**	0.57	0.58
FP2_16	**0.21**	0.45	0.54	**0.33**	**0.22**	0.45
FP2_17	0.43	**0.39**	0.65	0.75	0.56	0.47
FP2_18	**0.01**	0.75	0.78	**0.15**	**0.25**	0.58
FP2_19	**0.20**	**0.34**	0.52	0.91	0.54	0.50
FP2_20	0.42	**0.40**	0.68	0.91	**0.31**	0.59
FP2_21a	**0.04**	**0.24**	**0.12**	0.57	**0.25**	0.45
FP2_21b	**0.08**	**0.30**	**0.36**	0.57	**0.06**	0.40
FP2_21c	**0.35**	**0.34**	0.47	0.56	0.70	0.48
FP2_21d	0.67	**0.34**	**0.30**	0.56	0.67	**0.29**
FP2_21e	**0.38**	**0.05**	0.54	0.56	0.74	**0.39**
FP2_21f	**0.27**	**0.01**	**0.14**	0.57	0.55	**0.08**
**Advocacy**						
**N**	**15**	**10**	**10**	**7**	**5**	**26**
**Cronbach's Alpha**	**0.91**	**0.94**	**0.96**	**0.97**	**0.81**	**0.93**
FP3_1	**0.14**	0.65	0.81	0.55	0.88	0.49
FP3_2	**0.08**	0.61	0.62	0.46	0.93	0.44
FP3_3	**0.06**	0.46	0.80	0.82	**0.28**	0.53
FP3_4a	0.57	0.61	0.60	0.94	0.77	0.47
FP3_4b	0.46	0.71	0.50	0.90	0.81	0.64
FP3_4c	0.85	0.73	0.85	0.81	**0.02**	0.73
FP3_4d	0.81	0.88	0.69	0.82	0.54	0.76
FP3_4e	0.80	0.88	0.66	0.82	**0.13**	0.78
FP3_4f	0.80	0.69	0.65	0.82	**0.16**	0.68
FP3_4g	0.77	0.69	0.88	0.92	**0.08**	0.75
FP3_5a	0.70	0.62	0.74	**0.33**	0.41	0.68
FP3_5b	0.69	0.81	0.73	0.81	**0.04**	0.69
FP3_5c	0.64	0.83	0.72	0.81	**0.38**	0.72
FP3_5d	0.66	0.77	0.76	0.81	**0.14**	0.60
FP3_5e	0.70	0.61	0.83	0.81	0.52	0.65
FP3_5f	0.78	0.62	0.83	0.81	**0.36**	0.44

For peer review only - http://bmjopen.bmj.com/site/about/guidelines.xhtml

FP3_5g	0.65	0.65	0.80	0.81	**0.14**	0.46
FP3_6	**0.32**	**0.39**	0.82	0.73	0.60	0.61
**Research**						
**N**	**15**	**12**	**11**	**7**	**3**	**27**
**Cronbach's Alpha**	**0.94**	**0.94**	**0.98**	**0.95**	**0.86**	**0.93**
FP4_1a	0.44	0.43	0.86	0.63	0.71	0.47
FP4_1b	**0.31**	0.65	0.89	0.82	0.93	0.59
FP4_1c	0.42	0.52	0.96	0.74	0.98	0.57
FP4_2	0.73	0.51	0.93	0.66	**0.25**	0.60
FP4_3a	0.82	0.67	0.87	0.84	0.81	0.54
FP4_3b	0.61	0.85	0.81	0.79	0.67	0.69
FP4_3c	0.64	0.83	0.49	0.79	**0.34**	0.50
FP4_4a	0.70	0.87	0.83	0.67	0.73	0.84
FP4_4b	0.58	0.82	0.84	0.89	**0.40**	0.71
FP4_4c	0.50	0.71	0.86	0.89	0.65	0.70
FP4_4d	0.49	0.58	0.67	0.61	0.42	0.75
FP4_5a	0.73	0.54	0.80	0.47	0.89	0.48
FP4_5b	0.75	0.64	0.91	0.68	0.83	0.44
FP4_5c	0.68	0.75	0.80	0.61	0.67	0.63
FP4_5d	0.88	0.73	0.82	0.72	0.80	0.72
FP4_6	0.73	**0.39**	0.65	0.63	0.84	0.74
FP4_7	0.58	0.65	0.81	0.89	0.49	0.63
FP4_8	0.44	0.42	0.90	**0.37**	0.93	0.66
FP4_9	0.84	0.61	0.69	0.40	0.85	0.40
FP4_10	0.90	0.57	0.79		0.87	0.46
FP4_11	0.87	**0.22**	0.94	0.93	**0.27**	0.64
**Civil society**						
**N**	**14**	**15**	**13**	**7**	**4**	**27**
**Cronbach's Alpha**	**0.96**	**0.97**	**0.98**	**0.93**	**0.92**	**0.97**
FP5_1	0.89	0.75	0.67		0.86	0.64
FP5_2	**0.33**	0.69	0.70	0.55	0.79	0.73
FP5_3	0.88	0.80	0.95	0.42	0.55	0.84
FP5_4	0.91	0.84	0.84	0.81	0.66	0.85
FP5_5	0.85	0.82	0.85	0.60	0.89	0.92
FP5_6	0.73	0.82	0.90	0.82	**0.27**	0.69
FP5_7	0.76	0.84	0.86	0.56	0.61	0.81
FP5_8	0.92	0.78	0.90	0.81	0.77	0.92
FP5_9	0.94	0.90	0.86	0.70	0.97	0.86
FP5_10	0.73	0.95	0.94	0.69	0.66	0.87
FP5_11	0.79	0.61	0.94	0.81	**0.13**	0.62
FP5_12	0.52	0.90	0.96	0.71	0.79	0.80
FP5_13	0.65	0.84	0.91	0.76	0.42	0.94

b) Sector: Maternal and Child Health

Item	Ethiopia	Kenya	Nigeria	Rwanda	Senegal	Tanzania
	Item rest correlation					
**Policy**						
**N**	**12**	**11**	**7**	**9**	**7**	**14**
**Cronbach's Alpha**	**0.98**	**0.94**	**0.81**	**0.93**	**0.59**	**0.93**
MCH1_1a	0.96	0.76	0.91	0.57	**0.28**	0.59
MCH1_1b	0.75	0.81	0.47	0.82	**0.09**	0.53
MCH1_2	0.93	0.68	0.56	0.77	0.62	0.63
MCH1_3	0.86	0.61	0.54	0.87	0.52	0.54
MCH1_4	0.73	0.76	**0.21**	0.72	0.50	0.47
MCH1_5	0.79	0.56	0.58	0.73	**0.04**	**0.32**
MCH1_6	0.90	0.57	0.46	0.50	**0.09**	0.66
MCH1_7	0.81	0.85	**0.09**	0.63	0.69	0.86
MCH1_8	0.74	0.79	**0.34**	**0.38**	0.60	0.83
MCH1_9	0.83	0.61	**0.22**	0.50	0.48	0.82
MCH1_10	0.88	0.70	**0.38**	0.52	**0.08**	0.67
MCH1_11	0.72	**0.01**	0.52	0.84	**0.09**	0.47
MCH1_12	0.91	0.62	0.63	0.65	**0.25**	0.55
MCH1_13a	0.94	0.74	0.46	**0.25**	**0.25**	0.75
MCH1_13b	0.76	0.75	**0.11**	0.42	**0.19**	0.78
MCH1_13c	0.83	0.70	**0.16**	0.87	**0.08**	0.72
MCH1_13d	0.92	0.77	**0.21**	0.87	**0.33**	0.57
MCH1_13e	0.90	0.75	**0.09**	0.63	**0.33**	0.61
**Services**						
**N**	**10**	**5**	**5**	**6**	**8**	**6**
**Cronbach's Alpha**	**0.99**	**0.85**	**0.98**	**0.80**	**0.96**	**0.98**
MCH2_1a	0.76	**0.24**	0.93		**0.30**	0.61
MCH2_1b	0.76	**0.07**	0.91	**0.18**	0.86	0.59
MCH2_1c	0.89	0.81	0.71	0.48	**0.31**	0.69
MCH2_1d	0.89	0.47	0.53	0.48	**0.29**	0.59
MCH2_1e	0.86	0.41	0.81	0.69	0.80	0.74
MCH2_1f	0.83	0.71	0.64	0.53	0.75	0.48
MCH2_1g	0.85	**0.28**	0.52	**0.38**	0.65	**0.40**
MCH2_2a	0.88	**0.24**	0.45	0.47	0.82	0.75
MCH2_2b	0.94	0.53	0.42	0.43	**0.39**	0.65
MCH2_2c	0.85	0.93	0.59	0.70	0.68	0.74
MCH2_3a	0.88	0.65	0.92	0.70	0.52	0.88
MCH2_3b	0.89	**0.38**	0.89	**0.36**	0.80	0.88
MCH2_3c	0.91	0.68	0.78	0.70	**0.36**	0.87
MCH2_3d	0.95	0.62	0.91	0.47	0.82	0.67
MCH2_3e	0.86	0.47	0.70	0.60	**0.02**	0.60
MCH2_3f	0.84	0.51	0.53	0.82	0.77	0.66
MCH2_3g	0.83	0.51	0.83	0.82	0.79	0.72
MCH2_3h	0.89	0.52	0.63	0.66	0.90	0.64

MCH2_3i	0.81	**0.21**	0.86	0.61	0.75	0.67
MCH2_4a	0.93	**0.30**	0.82	0.88	0.83	0.74
MCH2_4b	0.96	**0.37**	0.72	0.97	0.78	0.70
MCH2_4c	0.94	0.52	0.71	0.71	0.55	0.68
MCH2_4d	0.88	0.55	0.96	**0.16**	0.81	0.83
MCH2_5a	0.88	0.56	0.66	0.95	0.92	0.82
MCH2_5b	0.89	0.60	0.68	**0.21**	0.87	0.74
MCH2_5c	0.45	0.47	**0.39**	0.90	**0.22**	0.69
MCH2_5d	0.98	**0.34**	0.67	0.70	0.76	0.63
MCH2_5e	0.79	0.47	0.86	0.90	**0.17**	0.71
MCH2_5f	0.92	0.47	0.93	0.78	0.59	0.75
MCH2_5g	0.95	0.59	0.87	0.80	0.63	0.78
MCH2_6a	0.86	**0.07**	0.66	0.71	0.66	0.83
MCH2_6b	0.91	**0.25**	0.82	0.71	0.69	0.79
MCH2_6c	0.97	**0.37**	0.84	0.72	0.64	0.81
MCH2_6d	0.80	0.77	0.89	0.62	0.80	0.84
MCH2_6e	0.91	0.53	0.82	0.70	0.93	0.67
MCH2_7a	0.93	0.43	0.57	0.96	0.80	0.88
MCH2_7b	0.96	0.48	0.86	0.72	0.77	0.84
MCH2_7c	0.90	**0.32**	0.92	0.50	0.41	0.71
MCH2_7d	0.76	0.41	**0.40**	0.55	0.82	0.83
MCH2_7e	0.71	0.80	**0.12**	0.63	**0.33**	0.76
MCH2_7f	0.56	0.72	**0.34**	0.82	0.52	0.67
MCH2_7g	0.95	**0.38**	0.81	0.82	0.48	0.68
MCH2_8a	0.93	0.53	0.92	0.72	0.75	0.61
MCH2_8b	0.90	0.60	0.78	0.52	0.83	0.87
**Advocacy**						
**N**	**12**	**11**	**8**	**8**	**14**	**14**
**Cronbach's Alpha**	**0.96**	**0.90**	**0.94**	**0.90**	**0.93**	**0.83**
MCH3_1	0.88	0.72	0.95	0.85	0.88	0.45
MCH3_2	0.89	0.79	0.82	0.77	0.83	0.84
MCH3_3	0.87	0.73	0.88	0.80	0.96	0.51
MCH3_4	0.91	0.87	0.78	0.63	0.91	0.62
MCH3_5	0.92	0.61	0.69	0.63	0.66	0.66
MCH3_6	0.86	0.62	0.76	0.77	0.68	0.62
**Research**						
**N**	**11**	**8**	**5**	**8**	**7**	**10**
**Cronbach's Alpha**	**0.99**	**0.98**	**0.96**	**0.96**	**0.95**	**0.98**
MCH4_1a	0.92	0.71	0.57	0.78	0.86	0.74
MCH4_1b	0.95	0.84	**0.35**	**0.24**	0.54	0.64
MCH4_1c	0.98	0.80	0.79	0.77	0.84	**0.35**
MCH4_2	0.90	0.86	0.77	0.77	0.82	0.91
MCH4_3a	0.85	0.79	0.91	**0.24**	0.68	0.90
MCH4_3b	0.94	0.90	0.84	0.87	0.63	0.89
MCH4_3c	0.83	0.91	**0.38**	0.81	0.83	0.80
MCH4_4a	0.85	0.67	0.83	0.64	0.83	0.82
MCH4_4b	0.83	0.80	0.77	0.78	**0.32**	0.81

MCH4_4c	0.92	0.74	0.94	0.92	0.43	0.82
MCH4_4d	0.76	0.98	0.93	0.81	0.57	0.90
MCH4_5a	0.93	0.86	0.74	0.87	0.90	0.88
MCH4_5b	0.96	0.87	0.79	0.84	0.90	0.89
MCH4_5c	0.96	0.94	0.90	0.70	0.85	0.84
MCH4_5d	0.86	0.91	0.71	0.84	0.90	0.90
MCH4_5e	0.94	0.85	0.69	0.81	0.57	0.94
MCH4_6	0.79	0.46	**0.31**	0.85	0.87	0.68
MCH4_7	0.78	0.87	0.47	0.46	0.92	0.79
MCH4_8	0.94	0.91	0.93	0.73	0.89	0.87
MCH4_9	0.82	0.90	0.74	0.79	0.88	0.83
MCH4_10	0.86	0.91	0.83	0.84	0.92	0.90
MCH4_11	0.86	0.94	0.66	0.70	0.85	0.72
**Civil Society**						
**N**	**8**	**9**	**8**	**8**	**8**	**11**
**Cronbach's Alpha**	**0.97**	**0.98**	**0.98**	**0.90**	**0.96**	**0.95**
MCH5_1	0.93	0.93	0.85	0.40	0.90	0.69
MCH5_2	0.86	0.79	0.91	0.80	0.72	0.83
MCH5_3	0.93	0.79	0.92	0.64	0.90	0.86
MCH5_4	0.91	0.94	0.91	0.41	0.86	0.84
MCH5_5	0.74	0.81	0.86	0.84	0.95	0.74
MCH5_6	0.74	0.90	0.81	0.80	0.82	0.85
MCH5_7	0.75	0.79	0.90	0.44	0.65	0.78
MCH5_8	0.89	0.94	0.98	0.91	0.66	0.84
MCH5_9	0.81	0.93	0.82	0.46	0.82	0.86
MCH5_10	0.83	0.97	0.93	0.67	0.93	0.57
MCH5_11	0.92	0.97	0.85	0.74	0.85	0.60
MCH5_12	0.92	0.89	0.94	0.78	0.78	0.66

only

c) Sector: Women Empowerment

	Ethiopia	Kenya	Nigeria	Rwanda	Senegal	Tanzania
			Item rest correlation			
**Policy**						
**N**	4	**8**	**8**	**11**	**13**	**11**
**Cronbach's Alpha**	**0.97**	**0.97**	**0.96**	**0.93**	**0.95**	**0.98**
WE1_1	0.60	0.77	0.71	0.75	**0.28**	0.58
WE1_2	0.91	0.79	0.66	0.68	0.59	0.52
WE1_3	0.48	0.88	0.91	0.49	0.60	0.93
WE1_4	0.73	0.84	0.84	0.47	0.62	0.83
WE1_5	0.96	0.87	0.78	0.62	0.74	0.72
WE1_6	0.89	0.62	0.49	0.70	0.66	0.80
WE1_7	0.85	0.69	0.48	0.70	0.45	0.66
WE1_8a	0.86	0.71	0.40	0.77	0.69	0.74
WE1_8b	0.92	0.70	0.85	0.61	0.74	0.88
WE1_9	0.94	0.72	0.91	0.63	0.92	0.84
WE1_10	0.86	0.85	**0.19**	**0.33**	0.63	0.83
WE1_11	**0.40**	0.78	0.44	0.55	0.68	0.86
WE1_12	0.97	0.52	**0.39**	0.81	0.83	0.86
WE1_13a	0.55	**0.21**	0.90	**0.34**	0.54	0.77
WE1_13b	0.79	0.86	0.83	0.55	0.92	0.90
WE1_14	0.70	0.69	0.80	0.78	0.88	0.83
WE1_15	0.82	0.86	0.71	0.56	0.85	0.97
WE1_16	0.89	0.62	0.58	0.70	0.72	0.95
WE1_17a	0.95	0.74	0.61	0.64	0.86	0.89
WE1_17b	0.83	0.72	0.86	0.70	0.90	0.94
WE1_17c	0.95	0.77	0.85	0.42	0.84	0.61
WE1_17d	0.94	0.81	0.96	**0.33**	0.85	0.94
WE1_18	0.94	0.73	0.63	0.77	0.74	0.92
**Programs and services**						
**N**	7	**9**	**9**	**13**	**13**	**11**
**Cronbach's Alpha**	**0.94**	**0.91**	**0.96**	**0.91**	**0.95**	**0.94**
WE2_1	0.91	0.85	0.95	0.69	0.81	0.81
WE2_2	0.59	0.71	0.93	0.69	0.92	0.77
WE2_3	0.78	0.86	0.90	0.66	0.73	**0.18**
WE2_4	0.87	0.88	0.94	0.68	0.78	0.91
WE2_5	0.85	0.74	0.87	0.80	0.76	0.73
WE2_6	0.64	0.82	0.82	0.60	0.65	0.82
WE2_7	0.60	0.48	0.93	0.61	0.68	0.41
WE2_8	0.70	0.63	0.91	0.66	0.63	0.79
WE2_9	0.85	0.88	0.97	0.50	0.81	0.81
WE2_10	0.69	0.41	0.53	0.83	0.64	0.76
WE2_11	0.89	0.52	0.78	0.47	0.82	0.81
WE2_12	0.77	**0.27**	**0.09**	0.65	0.75	0.76
**Advocacy**						

**N**	7	**10**	**9**	**15**	**13**	**12**
**Cronbach's Alpha**	**0.96**	**0.88**	**0.90**	**0.90**	**0.95**	**0.87**
WE3_1	0.91	0.73	0.86	0.84	0.84	0.71
WE3_2	0.90	0.69	0.77	0.81	0.88	0.80
WE3_3	0.91	**0.34**	0.88	0.73	0.91	**0.37**
WE3_4	0.81	0.85	0.77	0.58	0.88	0.79
WE3_5	0.79	0.52	**0.36**	0.61	0.75	0.57
WE3_6	0.87	0.91	0.82	0.73	0.79	0.67
WE3_7	0.94	0.66	0.70	0.70	0.83	0.68
**Research**						
**N**	4	**7**	**6**	**11**	**6**	**8**
**Cronbach's Alpha**	**0.98**	**0.93**	**0.98**	**0.90**	**0.95**	**0.96**
WE4_1a	0.92	0.77	0.69	0.70	**0.23**	**0.35**
WE4_1b	0.64	**0.37**	0.74	0.43	0.84	0.77
WE4_1c	0.86	0.54	0.69	0.44	0.88	**0.30**
WE4_2	0.98	0.62	0.89	0.62	0.72	0.86
WE4_3a	0.81	0.94	0.95	0.64	0.67	0.75
WE4_3b	0.97	0.73	0.93	0.52	0.88	**0.22**
WE4_3c	0.93	0.74	0.66	0.46	0.78	**0.21**
WE4_4a	0.94	0.61	0.87	**0.09**	0.90	0.87
WE4_4b	0.93	0.74	0.94	0.58	0.84	0.93
WE4_4c	0.82	0.65	0.78	0.52	0.83	0.88
WE4_4d	0.92	0.74	0.89	0.47	0.80	0.88
WE4_5a	0.96	0.64	0.79	0.82	0.76	0.87
WE4_5b	0.94	0.72	0.90	0.67	0.84	0.85
WE4_5c	0.78	0.47	0.83	0.79	0.90	0.88
WE4_6	0.93	0.63	0.85	0.81	0.90	0.90
WE4_7	0.99	0.71	0.90	0.64	0.73	0.89
WE4_8	0.73	0.63	0.92	**0.29**	0.89	0.88
WE4_9	0.73	0.69	0.72	0.62	0.89	0.82
WE4_10	0.89	**0.30**	0.76	0.63	0.88	0.86
**Research**						
**N**	6	**8**	**8**	**16**	**11**	**11**
**Cronbach's Alpha**	**0.95**	**0.94**	**0.96**	**0.92**	**0.97**	**0.84**
WE5_1	0.85	0.78	0.79	0.55	0.97	**0.12**
WE5_2	0.88	0.81	0.82	0.84	0.94	0.61
WE5_3	0.85	0.81	0.91	0.70	0.90	0.53
WE5_4	0.64	0.76	0.91	0.67	0.82	**0.35**
WE5_5	0.84	0.75	0.90	0.72	0.85	0.66
WE5_6	0.83	0.88	0.86	0.70	0.86	0.75
WE5_7	0.90	0.52	0.72	0.68	0.82	0.83
WE5_8	0.89	0.69	0.82	0.75	0.97	0.46
WE5_9	0.70	0.93	0.85	0.77	0.61	0.80
WE5_10	0.68	0.88	0.83	0.64	0.87	**0.37**

d) Sector: Education

	Ethiopia	Kenya	Nigeria	Rwanda	Senegal	Tanzania
			Item rest correlation			
**Policy**						
**N**	**11**	**4**	**7**	**6**	**4**	**9**
**Cronbach's Alpha**	**0.82**	**0.99**	**0.94**	**0.98**	**0.90**	**0.96**
ED1_1	0.70	0.91	0.61	0.87	0.69	0.57
ED1_2a	0.48	0.95	0.53	**0.29**	0.55	0.62
ED1_2b	**0.39**	0.96	0.75	0.53	0.54	0.74
ED1_3	0.54	0.84	0.72	0.86	**0.31**	0.75
ED1_4	**0.11**	0.98	0.80	0.90	0.80	0.75
ED1_5	0.54	0.98	0.89	0.91	0.81	0.92
ED1_6	**0.35**	0.70	0.82	0.90	0.81	0.91
ED1_7	0.71	0.98	0.84	0.96	0.47	0.84
ED1_8	0.44	0.86	0.94	0.91	0.83	0.45
ED1_9a	**0.27**	0.84	**0.28**	0.98	0.70	0.84
ED1_9b	0.46	0.91	**0.39**	0.89	0.83	0.88
ED1_9c	**0.22**	0.85	0.84	0.98	0.95	0.72
ED1_10a	**0.29**	0.95	0.48	0.96	0.78	0.77
ED1_10b	**0.02**	0.94	0.81	0.93	0.71	0.67
ED1_11	0.62	0.95	0.77	0.81	0.65	0.73
ED1_12	0.43	0.99	0.55	0.95	0.54	0.83
ED1_13	**0.36**	0.94	0.75	0.94	**0.27**	0.91
**Services and Programs**						
**N**	**12**	**4**	**11**	**7**	**9**	**9**
**Cronbach's Alpha**	**0.91**	**0.94**	**0.91**	**0.96**	**0.89**	**0.96**
ED2_1	0.70	0.93	**0.35**	0.90	0.75	0.78
ED2_2a	0.83	0.88	0.88	0.85	0.67	0.72
ED2_2b	0.74	0.95	0.81	0.95	0.83	0.92
ED2_3	0.66	0.83	0.87	0.94	0.75	0.83
ED2_4	0.87	0.98	0.84	0.66	0.79	0.96
ED2_5	0.88	**0.10**	0.60	0.76	0.57	0.97
ED2_6	0.61	0.72	0.73	0.93	0.65	0.89
**Advocacy**						
**N**	**13**	**5**	**12**	**7**	**7**	**9**
**Cronbach's Alpha**	**0.91**	**0.82**	**0.87**	**0.95**	**0.71**	**0.92**
ED3_1	0.78	0.88	0.70	0.89	**0.03**	0.76
ED3_2	0.79	**0.31**	0.67	0.95	**0.29**	0.91
ED3_3	0.72	0.75	0.72	0.76	0.96	0.87
ED3_4	0.83	0.77	0.82	0.92	0.92	0.77

For peer review only - http://bmjopen.bmj.com/site/about/guidelines.xhtml

**Research**						
**N**	**6**	**2**	**9**	**6**	**5**	**7**
**Cronbach's Alpha**	**0.94**	**0.70**	**0.94**	**0.98**	**0.97**	**0.98**
ED4_1a	0.62	**0.22**	0.51	0.62	0.95	0.91
ED4_1b	0.58	**0.09**	0.74	0.89	0.93	0.75
ED4_1c	0.55	**0.10**	0.75	0.80	0.80	0.94
ED4_2	0.49	0.42	0.66	0.89	0.85	0.93
ED4_3a	0.62	**0.06**	0.83	0.72	0.84	**0.14**
ED4_3b	0.47	**0.07**	0.83	**0.28**	0.83	0.64
ED4_3c	**0.28**	0.93	0.89	**0.18**	0.89	0.69
ED4_4a	0.50	0.87	0.52	0.62	0.77	0.82
ED4_4b	0.47	0.79	0.62	0.94	0.61	0.92
ED4_4c	0.42	0.88	0.58	0.94	0.70	0.83
ED4_4d	**0.27**	0.62	**0.30**	0.94	0.92	0.91
ED4_5a	0.87	0.88	0.84	0.84	0.55	0.84
ED4_5b	0.85	0.92	0.79	0.87	0.60	0.96
ED4_5c	0.81	0.95	0.82	0.93	0.74	0.93
ED4_5d	0.80	0.94	0.92	0.93	0.79	0.79
ED4_6	0.69	0.96	0.78	0.96	0.86	0.73
ED4_7	0.70	0.59	0.67	0.96	0.88	0.95
ED4_8	0.86	0.88	0.46	0.88	0.93	0.89
ED4_9	0.71	**0.10**	0.66	0.93	0.90	0.89
ED4_10	0.75	**0.01**	0.79	0.80	0.96	0.60
ED4_11a	0.76	0.81	0.77	0.97	0.74	0.83
ED4_11b	0.75	**0.16**	0.72	0.93	0.87	0.89
**Civil society**						
**N**	**6**	**8**	**8**	**16**	**11**	**11**
**Cronbach's Alpha**	**0.95**	**0.94**	**0.96**	**0.92**	**0.97**	**0.84**
ED5_1	0.89	0.99	0.56	0.93	0.89	0.71
ED5_2	0.87	0.98	0.72	0.92	0.97	0.61
ED5_3	0.62	0.91	0.82	0.94	0.93	0.83
ED5_4	0.88	1.00	0.84	0.98	0.80	0.95
ED5_5	0.75	0.97	0.64	0.82	0.93	0.97
ED5_6	0.65	1.00	0.57	0.86	0.71	0.90
ED5_7	0.86	0.93	0.67	0.97	0.93	0.78
ED5_8	0.80	0.96	0.85	0.89	0.86	0.86
ED5_9	0.82	0.89	0.87	0.88	0.91	0.91
ED5_10	0.89	0.90	0.85	0.84	0.87	0.92
ED5_11	0.69	1.00	0.85	0.93	0.90	0.95

e) Sector: Labor Market

	Ethiopia	Kenya	Nigeria	Rwanda	Senegal	Tanzania
			Item rest correlation			
**Policy**						
**N**	**6**	**12**	**8**	**8**	**9**	**8**
**Cronbach's Alpha**	**0.89**	**0.84**	**0.94**	**0.88**	**0.91**	**0.97**
LM1_1a	**0.34**	**0.26**	**0.12**	0.70	**0.06**	0.89
LM1_1b	0.86	0.48	**0.31**	0.71	**0.13**	0.41
LM1_2	0.40	**0.23**	**0.19**	0.93	**0.19**	0.73
LM1_3	0.74	**0.35**	0.87	0.85	0.61	0.81
LM1_4a	**0.10**	**0.23**	**0.11**	0.64	**0.14**	0.96
LM1_4b	**0.08**	**0.15**	0.75	**0.03**	0.48	0.43
LM1_5	**0.11**	0.70	0.83	0.44	0.80	0.91
LM1_6	0.80	0.64	0.63	0.54	0.84	0.92
LM1_7	0.72	**0.26**	0.94	0.92	0.90	0.96
LM1_8	0.96	**0.24**	0.87	0.67	0.86	0.80
LM1_9	0.53	0.80	0.86	0.43	0.89	0.93
LM1_10	0.68	0.69	0.92	0.83	0.91	0.69
LM1_11	0.80	0.71	0.81	0.54	0.93	0.92
LM1_12	0.64	0.64	0.85	**0.02**	0.90	0.92
LM1_13	0.75	0.44	0.64	0.71	0.85	**0.36**
LM1_14	0.80	0.52	0.68	0.70	0.74	0.90
LM1_15	0.65	**0.20**	0.77	0.76	0.45	0.95
**Services and Programs**						
**N**	**8**	**12**	**7**	**8**	**8**	**8**
**Cronbach's Alpha**	**0.83**	**0.86**	**0.96**	**0.78**	**0.94**	**0.96**
LM2_1	**0.38**	0.62	0.96	0.56	0.77	0.90
LM2_2	**0.04**	0.56	0.95	0.57	0.85	0.88
LM2_3	0.43	0.62	**0.27**	**0.19**	0.76	0.88
LM2_4	0.51	0.51	0.96	0.70	0.95	0.85
LM2_5	0.88	0.49	0.92	0.44	0.94	0.76
LM2_6	0.80	0.52	0.94	**0.30**	0.91	0.86
LM2_7	0.57	0.71	0.94	0.58	0.76	0.90
LM2_8	**0.13**	0.67	0.91	0.84	0.72	0.73
LM2_9	0.85	**0.28**	0.95	0.56	0.74	0.50
LM2_10	0.91	**0.37**	0.96	**0.35**	0.60	0.66
LM2_11	0.67	0.69	0.41	**0.32**	0.49	0.67
LM2_12a	**0.08**	**0.22**	**0.37**	**0.05**	0.49	0.72
LM2_12b	**0.36**	**0.26**	0.63	**0.21**	0.48	0.72
LM2_13	**0.17**	0.51	0.56	**0.13**	0.64	0.76
LM2_14	0.43	0.52	0.85	0.40	0.83	0.86
LM2_15	**0.07**	0.42	0.88	0.68	0.80	0.85
**Advocacy**						
**N**	**8**	**12**	**8**	**9**	**9**	**8**
**Cronbach's Alpha**	**0.40**	**0.76**	**0.90**	**0.85**	**0.85**	**0.90**
LM3_1	**0.09**	0.49	0.66	**0.35**	0.49	0.85

LM3_2	**0.09**	**0.18**	0.78	0.86	0.86	0.89
LM3_3	**0.37**	0.62	0.77	0.93	0.83	0.56
LM3_4	0.68	0.63	0.77	0.93	0.63	0.92
LM3_5	**0.28**	0.61	0.84	0.82	0.82	0.67
**Research**						
**N**	**8**	**10**	**7**	**8**	**7**	**6**
**Cronbach's Alpha**	**0.94**	**0.96**	**0.96**	**0.97**	**0.96**	**0.96**
LM4_1a	0.68	0.65	0.91	0.77	0.78	0.76
LM4_1b	**0.04**	0.64	0.90	0.61	0.85	0.89
LM4_1c	**0.40**	0.79	0.95	0.92	0.91	0.93
LM4_2	0.56	0.63	0.80	0.91	0.60	0.80
LM4_3a	0.40	0.88	0.53	0.93	**0.33**	0.83
LM4_3b	0.46	0.71	0.42	0.92	0.91	0.68
LM4_3c	**0.27**	0.67	0.92	0.69	0.81	0.78
LM4_4a	0.95	0.82	0.52	0.95	0.73	0.52
LM4_4b	0.91	0.78	0.85	0.94	0.85	0.79
LM4_4c	0.92	0.82	0.67	0.79	0.84	**0.39**
LM4_4d	0.80	0.72	0.57	**0.08**	0.85	0.51
LM4_5a	0.81	0.83	0.65	0.87	0.94	0.69
LM4_5b	0.75	0.72	0.58	0.95	0.92	0.87
LM4_5c	0.66	0.63	**0.36**	0.85	0.85	0.79
LM4_6	0.86	0.78	0.91	**0.24**	0.66	0.88
LM4_7	0.85	0.47	0.77	0.86	0.64	0.94
LM4_8	0.88	0.79	0.70	0.53	0.65	0.55
LM4_9	**0.26**	0.57	0.75	**0.33**	**0.40**	0.76
LM4_10	**0.38**	0.72	0.77	0.95	0.85	0.62
LM4_11	0.61	0.56	0.84	0.95	0.90	0.97
**Civil society**						
**N**	**6**	**11**	**8**	**8**	**7**	**7**
**Cronbach's Alpha**	**0.95**	**0.95**	**0.97**	**0.71**	**0.96**	**0.98**
LM5_1	0.87	0.80	0.83	0.88	0.94	0.90
LM5_2	0.83	0.91	0.83	0.67	0.92	0.91
LM5_3	0.90	0.96	0.78	0.65	0.86	0.92
LM5_4	0.71	0.92	0.90	**0.14**	0.98	0.96
LM5_5	0.90	0.80	0.84	0.42	0.95	0.90
LM5_6	0.96	0.86	0.89	0.59	0.92	0.93
LM5_7	0.75	0.84	0.91	**0.34**	0.78	0.93
LM5_8	0.72	0.72	0.94	**0.13**	0.92	0.93
LM5_9	0.84	0.63	0.92	0.78	0.73	0.91
LM5_10	0.91	0.53	0.81	0.78	0.75	0.89

For peer review only - http://bmjopen.bmj.com/site/about/guidelines.xhtml

f) Sector: Good Governance and Economic Institutions

	Ethiopia	Kenya	Nigeria	Rwanda	Senegal	Tanzania
**Policy**						
**N**	**5**	**4**	**10**	**5**	**8**	**5**
**Cronbach's Alpha**	**0.90**	**0.96**	**0.97**	**0.98**	**0.87**	**0.92**
GEI1_1	0.88	0.63	0.78	0.98	0.63	0.63
GEI1_2	0.77	**0.33**	0.68	0.98	0.60	0.70
GEI1_3	0.49	0.99	0.91	0.86	0.66	0.63
GEI1_4	0.45	0.89	0.73	0.78	0.66	0.83
GEI1_5	0.82	0.82	0.90	0.90	0.71	0.65
GEI1_6	**0.29**	0.89	0.97	0.89	0.58	0.55
GEI1_7	0.49	0.96	0.85	0.99	0.43	0.88
GEI1_8	0.89	0.71	0.89	0.90	0.61	0.91
GEI1_9a	0.86	0.72	0.93	0.93	0.70	0.66
GEI1_9b	0.89	0.93	0.92	0.96	0.42	0.81
GEI1_10a	0.60	0.78	0.79	0.90	0.43	**0.35**
GEI1_10b	**0.01**	0.68	0.51	0.90	**0.36**	0.53
GEI1_10c	0.82	0.91	0.74	0.63	**0.09**	0.70
GEI1_11	**0.17**	0.60	0.95	0.89	0.68	**0.38**
GEI1_12	0.57	0.85	0.74	0.89	**0.17**	0.46
GEI1_13	0.86	0.69	0.87	0.84	**0.16**	0.52
**Services and Programs**						
**N**	**2**	**4**	**10**	**6**	**12**	**6.00**
**Cronbach's Alpha**	**0.85**	**0.91**	**0.97**	**0.96**	**0.94**	**0.92**
GEI2_1	**0.37**	0.95	0.91	0.77	0.74	0.95
GEI2_2	0.56	0.92	0.82	0.70	0.72	0.83
GEI2_3	**0.30**	0.93	0.80	0.90	0.81	0.93
GEI2_4	0.98	0.85	0.93	0.83	0.81	0.86
GEI2_5	**0.21**	0.58	0.72	0.46	0.74	0.70
GEI2_6	**0.26**	0.85	0.78	0.80	0.67	0.93
GEI2_7	**0.10**	0.93	0.80	0.91	0.73	**0.14**
GEI2_8a	0.75	0.85	0.69	0.77	0.79	0.72
GEI2_8b	0.63	0.87	0.70	0.79	0.69	0.70
GEI2_8c	0.78	0.48	0.72	**0.23**	0.68	0.78
GEI2_8d	0.74	0.96	0.71	**0.18**	0.71	0.59
GEI2_8e	0.76	0.73	0.65	0.59	0.77	0.59
GEI2_9	0.71	0.78	0.85	0.47	0.46	0.73
GEI2_10a	**0.36**	0.95	0.94	0.59	0.83	0.81
GEI2_10b	**0.38**	0.86	0.87	0.95	0.65	0.99
GEI2_10c	**0.15**	0.65	0.83	0.70	0.80	0.86
GEI2_10d	**0.09**	0.72	0.79	**0.39**	0.58	0.88
GEI2_11a	**0.14**	0.94	0.77	0.46	0.87	0.58
GEI2_11b	0.62	0.99	0.81	0.55	0.66	0.51
GEI2_11c	0.73	0.82	0.78	0.47	0.60	0.54
GEI2_11d	0.42	0.90	0.85	**0.08**	0.68	0.64
**Advocacy**						
**N**	**6**	**4**	**8**	**6**	**16**	**6.00**

**Cronbach's Alpha**	**0.85**	**0.91**	**0.97**	**0.96**	**0.94**	**0.92**
GEI3_1	0.64	0.86	0.84	0.50	0.78	0.44
GEI3_2	0.63	0.68	0.81	0.73	0.93	0.71
GEI3_3	**0.39**	0.84	0.83	0.98	0.88	0.86
GEI3_4	0.93	0.80	0.52	0.89	0.81	0.87
GEI3_5	0.50	0.93	0.89	0.77		0.75
GEI3_6a	**0.19**	0.93	0.95	0.77	0.77	0.93
GEI3_6b	**0.35**	0.99	0.94	0.90	0.67	**0.02**
GEI3_6c	0.89	0.96	0.79	0.77	0.66	0.94
GEI3_6d	0.68	0.92	0.93	0.87	0.55	0.91
GEI3_6e	**0.07**	0.82	0.91	0.87	0.90	0.96
GEI3_6f	0.53	0.51	0.81	0.87	0.56	0.97
**Research**						
**N**	**5**	**4**	**7**	**6**	**13**	**6.00**
**Cronbach's Alpha**	**0.94**	**0.98**	**0.97**	**0.93**	**0.97**	**0.96**
GEI4_1a	0.72	0.86	0.78	0.40	0.74	0.50
GEI4_1b	0.89	1.00	0.82	0.55	0.81	0.59
GEI4_1c	0.80	0.90	0.76	0.66	0.87	0.75
GEI4_2	**0.11**	0.86	0.81	0.59	0.88	0.84
GEI4_3a	0.72	0.87	0.62	0.79	0.87	0.83
GEI4_3b	0.76	0.95	**0.35**	0.66	0.91	0.81
GEI4_3c	0.84	0.77	**0.29**	0.68	0.70	0.81
GEI4_4a	0.75	0.94	0.69	0.71	0.90	0.66
GEI4_4b	0.68	0.91	0.79	0.81	0.84	0.83
GEI4_4c	0.77	0.75	0.91	0.75	0.90	0.87
GEI4_4d	0.84	0.96	0.92	0.85	0.77	0.81
GEI4_5a	0.96	0.88	0.72	0.48	0.91	0.54
GEI4_5b	**0.28**	0.98	0.79	0.65	0.71	0.80
GEI4_5c	**0.29**	0.94	0.76	0.49	0.94	0.76
GEI4_5d	0.83	0.98	0.91	0.58		0.69
GEI4_6	0.81	0.93	0.88	0.83	0.59	0.47
GEI4_7	0.48	0.97	0.82	0.56	0.82	0.90
GEI4_8	0.83	0.97	0.91	0.71	0.82	0.83
GEI4_10	0.87	**0.36**	0.86	0.47	0.44	0.77
**Civil Society**						
**N**	**4**	**5**	**9**	**5**	**15**	**7.00**
**Cronbach's Alpha**	**0.75**	**0.96**	**0.97**	**0.92**	**0.91**	**0.96**
GEI5_1	**0.38**	0.97	0.75	**0.33**	0.63	0.59
GEI5_2	0.55	0.83	0.89	0.98	0.84	0.85
GEI5_3	**0.13**	0.83	0.92	0.97	0.57	0.97
GEI5_4	0.70	0.91	0.83	0.98	0.75	0.98
GEI5_5	0.49	0.85	0.93	0.98	0.86	0.86
GEI5_6	0.57	0.95	0.82	0.96	0.73	0.90
GEI5_7a	**0.20**	0.82	0.93	0.98	0.55	0.83
GEI5_7b	0.40	0.66	0.88	**0.35**	0.52	0.68
GEI5_8	0.49	0.86	0.85		0.75	0.87
GEI5_9	**0.04**	0.69	0.96	**0.35**	0.55	0.75

For peer review only - http://bmjopen.bmj.com/site/about/guidelines.xhtml

Notes:

1. The list of items corresponds to the list of variables as detailed in the questionnaire provided as Appendix 1 (English) and Appendix 2 (French). For example, in Appendix 3, item FP1_1 represents question FP 1.1 in Appendix 1 or 2.
2. Bolded are the item correlations <=0.4

For peer review only

Appendix 4. Confirmatory factor analysis

	Policy	Services and Programs	Advocacy	Research	Civil Society
**Family Planning**					
Population error					
RMSEA	0.14	0.19	0.21	0.19	0.15
90% CI - lower bound	0.11	0.18	0.19	0.17	0.12
90% CI - upper bound	0.16	0.20	0.23	0.20	0.17
p-value	<0.001	<0.001	<0.001	<0.001	<0.001
Baseline comparison					
CFI	0.69	0.26	0.58	0.63	0.89
TLI	0.64	0.23	0.53	0.59	0.87
Size of residuals					
SRMR	0.12	0.16	0.13	0.11	0.05
CD	0.89	0.95	0.95	0.96	0.97
**Maternal and Child Health**					
Population error					
RMSEA	0.17	0.46	0.21	0.22	0.25
90% CI - lower bound	0.15	0.45	0.14	0.20	0.22
90% CI - upper bound	0.19	0.47	0.29	0.24	0.28
p-value	<0.001	<0.001	<0.001	<0.001	<0.001
Baseline comparison					
CFI	0.69	0.17	0.91	0.72	0.81
TLI	0.65	0.12	0.86	0.69	0.77
Size of residuals					
SRMR	0.11	0.09	0.05	0.06	0.05
CD	0.95	0.99	0.93	0.99	0.98
**Women Empowerment**					
Population error					
RMSEA	0.16	0.16	0.15	0.18	0.16
90% CI - lower bound	0.14	0.12	0.09	0.15	0.12
90% CI - upper bound	0.18	0.19	0.22	0.20	0.21
p-value	<0.001	<0.001	0.01	<0.001	<0.001
Baseline comparison					
CFI	0.81	0.89	0.95	0.85	0.92
TLI	0.79	0.86	0.92	0.83	0.90
Size of residuals					
SRMR	0.06	0.05	0.04	0.05	0.04
CD	0.99	0.97	0.95	0.99	0.97
**Education**					
Population error					
RMSEA	0.21	0.29	0.42	0.25	0.16
90% CI - lower bound	0.18	0.23	0.26	0.23	0.12
90% CI - upper bound	0.23	0.36	0.59	0.27	0.21
p-value	<0.001	<0.001	<0.001	<0.001	<0.001
Baseline comparison					
CFI	0.70	0.79	0.88	0.66	0.92
TLI	0.66	0.69	0.63	0.62	0.90
Size of residuals					
SRMR	0.09	0.09	0.09	0.08	0.04
CD	0.96	0.93	0.93	0.99	0.97

For peer review only - http://bmjopen.bmj.com/site/about/guidelines.xhtml

**Labor Market**					
Population error					
RMSEA	0.18	0.21	0.19	0.26	0.20
90% CI - lower bound	0.15	0.18	0.08	0.24	0.15
90% CI - upper bound	0.20	0.23	0.30	0.28	0.25
p-value	<0.001	<0.001	0.03	<0.001	<0.001
Baseline comparison					
CFI	0.74	0.72	0.93	0.58	0.89
TLI	0.71	0.67	0.87	0.53	0.86
Size of residuals					
SRMR	0.11	0.10	0.06	0.11	0.04
CD	0.96	0.97	0.92	0.97	0.97
**Governance and economic institutions**					
Population error					
RMSEA	0.24	0.23	0.30	0.32	0.26
90% CI - lower bound	0.21	0.21	0.25	0.29	0.21
90% CI - upper bound	0.27	0.25	0.35	0.35	0.31
p-value	<0.001	<0.001	<0.001	<0.001	<0.001
Baseline comparison					
CFI	0.70	0.70	0.63	0.59	0.74
TLI	0.65	0.66	0.54	0.53	0.66
Size of residuals					
SRMR	0.11	0.07	0.15	0.08	0.10
CD	0.98	0.98	0.96	0.99	0.95

For peer review only - http://bmjopen.bmj.com/site/about/guidelines.xhtml

Appendix 5. Heterogeneity testing

1. Heterogeneity testing by domains and countries (Fixed effect)

	Policy	Programs	Advocacy	Research	Civil society	Overall	Between sub-groups test
**Ethiopia**							
Statistic	2.55	4.94	13.34	6.13	3.87	51.05	20.22
P-Value	0.769	0.423	0.02	0.294	0.568	0.007	<0.001
I-squared	0.0%	0.0%	62.5%	18.4%	0.0%	43.2%	
**Kenya**							
Statistic	3.43	6.48	5.1	4.72	1.28	25.89	4.88
P-Value	0.634	0.262	0.404	0.45	0.937	0.631	0.3
I-squared	0.0%	22.9%	2.0%	0.0%	0.0%	0.0%	
**Nigeria**							
Statistic	8.06	9.1	8.94	6.45	7.18	44.86	5.13
P-Value	0.153	0.105	0.112	0.265	0.208	0.03	0.274
I-squared	38.0%	45.1%	44.0%	22.4%	30.4%	35.3%	
**Rwanda**							
Statistic	34.89	97.23	25.87	21.36	27.62	252.46	45.49
P-Value	<0.001	<0.001	<0.001	0.001	<0.001	<0.001	<0.001
I-squared	85.7%	94.9%	80.7%	76.6%	81.9%	88.5%	
**Senegal**							
Statistic	25.68	15.8	44	9.44	13.79	111.89	3.18
P-Value	<0.001	<0.001	<0.001	0.093	0.017	<0.001	0.529
I-squared	80.5%	68.3%	88.6%	47.0%	63.7%	74.1%	
**Tanzania**							
Statistic	9.36	9.9	13.22	8.32	25.23	87.9	21.87
P-Value	0.096	0.078	0.021	0.14	<0.001	<0.001	<0.001
I-squared	46.6%	49.5%	62.2%	39.9%	80.2%	67.0%	

only

2. Heterogeneity testing by sectors (Random effect)

		Policy	Programs	Advocacy	Research	CSO	Overall
**Ethiopia**							
	Statistic	2.55	4.94	13.34	6.13	3.87	51.05
	P	0.77	0.42	0.02	0.29	0.57	0.01
	I-squared	0.0%	0.0%	62.5%	18.4%	0.0%	43.2%
	Tau-squared	0.00	0.00	0.45	0.06	0.00	0.16
**Kenya**							
	Statistic	3.43	6.48	5.10	4.72	1.28	25.89
	P	0.63	0.26	0.40	0.45	0.94	0.63
	I-squared	0.0%	22.9%	2.0%	0.0%	0.0%	0.0%
	Tau-squared	0.00	0.05	0.01	0.00	0.00	0.00
**Nigeria**							
	Statistic	8.06	9.10	8.94	6.45	7.18	44.86
	P	0.15	0.11	0.11	0.27	0.21	0.03
	I-squared	38.0%	45.1%	44.0%	22.4%	30.4%	35.3%
	Tau-squared	0.18	0.27	0.32	0.11	0.16	0.19
**Rwanda**							
	Statistic	34.89	97.23	25.87	21.36	27.62	252.46
	P	0.00	0.00	0.00	0.00	0.00	0.00
	I-squared	85.7%	94.9%	80.7%	76.6%	81.9%	88.5%
	Tau-squared	0.34	0.96	0.38	0.25	0.15	0.39
**Senegal**							
	Statistic	25.68	15.80	44.00	9.44	13.79	111.89
	P	0.00	0.01	0.00	0.09	0.02	0.00
	I-squared	80.5%	68.3%	88.6%	47.0%	63.7%	74.1%
	Tau-squared	1.28	1.43	4.28	0.76	1.67	1.30
**Tanzania**							
	Statistic	9.36	9.90	13.22	8.32	25.23	87.90
	P	0.10	0.08	0.02	0.14	0.00	0.00
	I-squared	46.6%	49.5%	62.2%	39.9%	80.2%	67.0%
	Tau-squared	0.15	0.21	0.46	0.14	1.09	0.34

Appendix 6. Percentages of "don't know" by question/item

Variable	Ethiopia		Kenya		Nigeria		Rwanda		Senegal		Tanzania	
	n	%	n	%	n	%	n	%	n	%	n	%
FP 1.1 Prominence of Family Planning (FP): Extent to which there is a permanent												
Don't know	0	0	0	0	0	0	0	0	0	0	1	2.6
FP 1.1 Prominence of Family Planning (FP): Extent to which there is a permanent												
Don't know	0	0	0	0	0	0	0	0	0	0	1	2.6
FP 1.2 Policy on Fertility Reduction and FP: Extent to which government policy e												
FP 1.3 National FP Strategic Plan: Extent to which there is a current, comprehen												
FP 1.4 Policy on Age at Marriage: Extent to which legal age at marriage for fema												
Don't know	0	0	0	0	0	0	0	0	0	0	1	2.6
FP 1.5 Evidence-Based Policymaking: Extent to which the appropriate ministry act												
FP 1.6 Monitoring and Evaluation of National Policies/Plans/Strategies: Extent t												
Don't know	0	0	0	0	0	0	0	0	0	0	3	7.9
FP 1.7 Inclusion of Nongovernmental and Cross-sectoral Actors in Policy/Strategy												
Don't know	0	0	0	0	0	0	0	0	0	0	1	2.6

Note:

- For readability purpose, complete wording for questions/variables has been provided as Appendix 1.
- Only the percentages of respondents who responded "don't know" to the respective question or sub-question was included in this table.
- Highlighted are values > 10%

For peer review only

FP 1.8 Financing for FP: Extent to which the government has established and appl												
Don't know	1	5.9	1	5.9	0	0	0	0	0	0	2	5.3
FP 1.9 Percent of In-country Funding of FP Budget: Extent to which the total fam												
Don't know	4	**23.5**	1	5.9	0	0	0	0	0	0	3	7.9
FP 1.10 Information/Communications Technology Infrastructure/Capacity:¬† Extent t												
Don't know	0	0	1	5.9	1	5.6	0	0	0	0	0	0
FP 1.11 Import Laws and Legal Regulations:¬†												
Don't know	0	0	5	**29.4**	1	5.6	0	0	0	0	2	5.3
Don't know	1	5.9	3	**17.6**	1	5.6	0	0	0	0	4	**12.1**
FP 1.12 Advertising of Contraceptives Allowed: Extent of freedom from restrictio												
Don't know	0	0	1	5.9	0	0	0	0	0	0	0	0
FP 1.13 Presence of Pro-Poor FP Policies: Extent to which policies specifically												
Don't know	0	0	0	0	0	0	0	0	0	0	1	2.6
FP 1.14 Political Support for FP: Extent to which elected officials in the count												
FP 2.1 Administrative Structure: Extent to which the administrative structure an												
Don't know	0	0	0	0	0	0	0	0	0	0	1	2.9
FP 2.2 Level of Program Leaders: Relative level of seniority of the director of												
Don't know	2	**13.3**	0	0	4	**25**	0	0	0	0	1	2.9

Note:

- For readability purpose, complete wording for questions/variables has been provided as Appendix 1.
- Only the percentages of respondents who responded “don’t know” to the respective question or sub-question was included in this table.
- Highlighted are values > 10%

For peer review only

FP. 2.3 Community-Based Distribution: Extent to which areas of the country not e												
Don't know	0	0	1	6.2	0	0	0	0	0	0	1	2.9
FP 2.4 Postpartum Program: Extent to which all postpartum women receive postpart												
Don't know	0	0	0	0	1	6.2	0	0	0	0	0	0
FP 2.5 Home-Visiting Workers: Extent of coverage of population by workers whose												
Don't know	0	0	1	6.2	1	6.2	0	0	0	0	1	2.9
FP 2.6 Availability and Accessibility of FP Commodities/Supplies and Services Pa												
FP 2.6.1	1	6.7	2	**12.5**	0	0	0	0	0	0	3	8.6
FP 2.6.2	0	0	1	6.2	0	0	0	0	0	0	1	3
FP 2.6.3	0	0	1	6.2	2	**13.3**	0	0	0	0	2	5.7
FP 2.6.4	0	0	1	6.2	0	0	0	0	0	0	1	2.9
FP 2.7 Availability and Accessibility of FP Commodities/Supplies and Services Pa												
FP 2.7.1	0	0	0	0	0	0	0	0	0	0	3	8.6
FP 2.7.2	0	0	0	0	0	0	0	0	0	0	3	8.6
FP 2.7.3	0	0	0	0	0	0	0	0	1	**14.3**	3	8.6
FP 2.7.4	0	0	0	0	1	6.2	0	0	0	0	3	8.6
FP 2.7.5	0	0	0	0	0	0	0	0	1	**14.3**	3	8.8
FP 2.7.6	0	0	0	0	3	**18.8**	0	0	0	0	4	**11.4**
FP 2.7.7	0	0	1	6.2	1	6.2	0	0	0	0	4	**11.4**

Note:

- For readability purpose, complete wording for questions/variables has been provided as Appendix 1.
- Only the percentages of respondents who responded "don't know" to the respective question or sub-question was included in this table.
- Highlighted are values > 10%

For peer review only

FP 2.8 Availability and Accessibility of FP Commodities/Supplies and Services Pa												
FP 2.8.1	0	0	2	**12.5**	1	6.2	0	0	0	0	4	**11.4**
FP 2.8.2	0	0	2	**12.5**	1	6.2	0	0	0	0	4	**11.4**
FP 2.8.3	0	0	0	0	0	0	0	0	0	0	3	8.6
FP 2.8.4	0	0	0	0	0	0	0	0	0	0	3	8.6
FP 2.9 FP Service Quality: Please rate the general quality of family planning se												
FP 2.10 Incentives and Disincentives: Extent to which monetary or other incentiv												
Don't know	0	0	1	6.2	1	6.2	0	0	0	0	3	8.6
FP 2.11 Adolescent Programs: Extent to which information, services and activitie												
Don't know	0	0	1	6.2	0	0	0	0	0	0	0	0
FP 2.12 Training Program: Extent to which training programs, for each category o												
Don't know	0	0	1	6.2	0	0	0	0	0	0	1	2.9
FP 2.13 Personnel Carry Out Assigned Tasks: Extent to which all categories of fa												
Don't know	0	0	1	6.2	0	0	0	0	0	0	2	5.7
FP 2.14 Logistics and Transport: Extent to which the logistics and transport sys												
Don't know	0	0	0	0	0	0	0	0	0	0	3	8.6
FP 2.15 Supervision System: Extent to which the system of supervision at all lev												
Don't know	0	0	0	0	0	0	0	0	0	0	1	2.9

Note:

- For readability purpose, complete wording for questions/variables has been provided as Appendix 1.
- Only the percentages of respondents who responded “don’t know” to the respective question or sub-question was included in this table.
- Highlighted are values > 10%

For peer review only

FP 2.16 Involvement of Other Ministries and Public Agencies: Extent to which oth												
Don't know	0	0	0	0	0	0	0	0	0	0	1	2.9
FP 2.17 Involvement of Private-Sector Agencies and Groups: Extent to which priva												
FP 2.18 Mass Media for Information Education Communication: Frequency and extent												
FP 2.19 Cultural and Social Norms Barriers: Extent to which cultural and social												
FP 2.20 Youth: Extent to which the government supports youth-led FP initiatives												
FP 2.21 FP Effort Influences ,Äì Forces Affecting the FP Program: Forces affecting												
FP 2.21.1	0	0	0	0	0	0	0	0	0	0	2	5.9
FP 2.21.2	0	0	0	0	0	0	0	0	0	0	1	3
FP 2.21.3	0	0	0	0	0	0	0	0	0	0	2	5.9
FP 2.21.4	0	0	1	6.2	0	0	0	0	0	0	1	2.9
FP 2.21.5	0	0	0	0	0	0	0	0	0	0	2	5.9
FP 3.1 Influence of FP Leaders/Champions: Extent to which the country has one or												
Don't know	0	0	0	0	0	0	0	0	0	0	1	2.9
FP 3.2 Statement by Leaders: Extent to which the head of government, as well as												
FP 3.3 Media Coverage for FP: Extent to and frequency with which national media												

For peer review only

Note:

- For readability purpose, complete wording for questions/variables has been provided as Appendix 1.
- Only the percentages of respondents who responded “don’t know” to the respective question or sub-question was included in this table.
- Highlighted are values > 10%

FP 3.4 FP Effort Influences - Justification: How important is each of the follow												
FP 3.4.1	0	0	0	0	0	0	0	0	0	0	1	2.9
FP 3.4.2	0	0	1	6.2	0	0	0	0	0	0	0	0
FP 3.4.3	0	0	0	0	0	0	0	0	0	0	1	2.9
FP 3.4.4	0	0	1	6.2	0	0	0	0	0	0	3	8.8
FP 3.4.5	0	0	1	6.7	0	0	0	0	0	0	3	9.4
FP 3.4.6	0	0	1	6.2	0	0	0	0	0	0	1	2.9
FP 3.4.7	0	0	0	0	0	0	0	0	0	0	1	2.9
FP 3.5 FP Effort Influences ‚Äì Special Populations: To what extent does the famil												
Don't know	0	0	2	**12.5**	0	0	0	0	0	0	1	2.9
FP 3.6 Social Marketing: Extent of coverage of the country by a social marketing												
Don't know	0	0	2	**12.5**	2	**13.3**	0	0	0	0	0	0
FP 4.1 Strength of Observatory/Stakeholder/Technical Working Group for FP: Exten												
Don't know	0	0	1	6.2	0	0	0	0	0	0	1	3.1
FP 4.2 Education/Research Strategy: Extent to which the national FP plan and/or												
Don't know	0	0	0	0	1	6.7	0	0	0	0	1	3.1
FP 4.3 Data Gathering Partners:												
FP 4.3.1	0	0	0	0	1	6.7	0	0	0	0	0	0
FP 4.3.2	0	0	0	0	3	**20**	0	0	0	0	0	0

Note:

- For readability purpose, complete wording for questions/variables has been provided as Appendix 1.
- Only the percentages of respondents who responded “don’t know” to the respective question or sub-question was included in this table.
- Highlighted are values > 10%

For peer review only

FP 4.3.3	0	0	0	0	3	**20**	0	0	0	0	1	3.1
FP 4.4 Topical Data Gathering:												
FP 4.4.1	0	0	1	6.2	0	0	0	0	0	0	0	0
FP 4.4.2	0	0	0	0	1	6.7	0	0	0	0	0	0
FP 4.4.3	0	0	0	0	1	6.7	0	0	0	0	0	0
FP 4.4.4	0	0	0	0	2	**13.3**	0	0	0	0	2	6.2
FP 4.5 Quality/Coverage of Data: Extent to which current research/data is sex an												
FP 4.5.1	0	0	0	0	1	6.7	0	0	0	0	1	3.1
FP 4.5.2	0	0	0	0	0	0	0	0	0	0	2	6.2
FP 4.5.3	0	0	0	0	1	6.7	0	0	0	0	3	9.4
FP 4.5.4	0	0	0	0	1	6.7	0	0	0	0	2	6.2
FP 4.6 Record-Keeping: Extent to which systems for client recordkeeping, clinic												
Don't know	0	0	0	0	1	6.7	0	0	0	0	1	3.1
FP 4.7 Quality Research Institutions: Extent to which the country supports and s												
Don't know	0	0	1	6.2	1	6.7	0	0	0	0	1	3.1
FP 4.8 Evaluation: Extent to which program statistics, surveys, and small studie												
Don't know	0	0	1	6.2	2	**13.3**	0	0	0	0	4	**12.5**
FP 4.9 Management‚Äôs Use of Evaluation Findings: Extent to which local-level prog												
Don't know	0	0	1	6.2	1	6.7	0	0	0	0	1	3.1

Note:

- For readability purpose, complete wording for questions/variables has been provided as Appendix 1.
- Only the percentages of respondents who responded “don’t know” to the respective question or sub-question was included in this table.
- Highlighted are values > 10%

For peer review only

FP 4.10 Ministerial Use of Evaluation Findings: Extent to which the Ministry adm												
Don't know	0	0	1	6.2	1	6.7	0	0	0	0	1	3.1
FP 4.11 Dissemination of Information to Other Implementing Bodies: Extent to whi												
Don't know	0	0	1	6.2	0	0	0	0	0	0	1	3.1
FP 5.1 CSO Actor Power: Extent to which CSO actors are in strong placements with												
Don't know	0	0	0	0	0	0	0	0	0	0	1	3.2
FP 5.2 Budget Analysis as a CSO Tool: Extent to which CSOs utilize budget analys												
Don't know	1	6.7	1	6.2	0	0	0	0	0	0	1	3.2
FP 5.3 Facility-Based Service Delivery Support: Extent to which CSOs are involve												
FP 5.4 Community-Based Service Delivery Support: Extent to which CSOs participat												
FP 5.5 Mobile Outreach for Community-Based Service Delivery Support: Extent to w												
Don't know	0	0	0	0	0	0	0	0	0	0	1	3.2
FP 5.6 Social Franchising: Extent to which CSOs are involved in social franchisi												
Don't know	0	0	1	6.2	0	0	0	0	0	0	3	9.7
FP 5.7 mHealth: Extent to which CSOs are leveraging mHealth to utilize text mess												
Don't know	0	0	0	0	2	**13.3**	0	0	0	0	2	6.5

Note:

- For readability purpose, complete wording for questions/variables has been provided as Appendix 1.
- Only the percentages of respondents who responded “don’t know” to the respective question or sub-question was included in this table.
- Highlighted are values > 10%

For peer review only

FP 5.8 Human Rights and Quality of Care: Extent to which CSOs advocate for and s												
FP 5.9 Social and Behavior Change (SBC): Extent to which CSOs support SBC interv												
Don't know	0	0	0	0	0	0	0	0	0	0	2	6.5
FP 5.10 Youth: Extent to which CSOs support FP policies, interventions and progr												
Don't know	0	0	0	0	0	0	0	0	0	0	1	3.2
FP 5.11 Programming for Men: Extent to which CSOs support FP policies, intervent												
Don't know	0	0	0	0	0	0	0	0	0	0	2	6.5
FP 5.12 Advocacy/Accountability: Extent to which CSOs support the training and c												
Don't know	0	0	0	0	0	0	0	0	0	0	1	3.2
FP 5.13 CSO-Led Assessment and Monitoring: Extent to which CSOs assess, monitor												
Don't know	0	0	0	0	0	0	0	0	0	0	3	9.7
FP 5.14 Forming Partnerships between CSOs: Extent to which CSOs have formed nati												
Don't know	0	0	0	0	0	0	0	0	0	0	2	6.5
MCH 1.1 Adequate Policy: Ministry of Health policies toward pregnancy and delive												
Don't know	0	0	0	0	0	0	0	0	1	5.9	0	0
Don't know	0	0	0	0	0	0	0	0	1	5.9	0	0
MCH 1.2 Universal Access: Extent to which there are constitutional and legal ent												

Note:

- For readability purpose, complete wording for questions/variables has been provided as Appendix 1.
- Only the percentages of respondents who responded “don’t know” to the respective question or sub-question was included in this table.
- Highlighted are values > 10%

For peer review only

Don't know	0	0	0	0	0	0	0	0	1	5.9	0	0
MCH 1.3 Multisector Involvement: Extent to which policies are developed through												
Don't know	0	0	0	0	0	0	0	0	1	5.9	0	0
MCH 1.4 Competent Service Providers: Extent to which policies are reasonable and												
Don't know	0	0	0	0	0	0	0	0	1	5.9	0	0
MCH 1.5 Abortion Legislation: Extent to which a favorable policy exists towards												
Don't know	0	0	1	5.6	0	0	0	0	1	5.9	2	**10.5**
MCH 1.6. Post-Abortion Care Legislation: Extent to which a favorable policy exis												
Don't know	0	0	4	**22.2**	0	0	0	0	1	5.9	2	**10.5**
MCH 1.7 Policy Reviews and Updates: Extent to which policies are regularly revie												
Don't know	0	0	2	**11.1**	0	0	0	0	1	5.9	0	0
MCH 1.8 High-Level Actor Placement: Extent and level to which the director of ma												
Don't know	0	0	4	**22.2**	0	0	0	0	1	5.9	1	5.3
MCH 1.9 Financing for MCH: Extent to which the government budgets for safe pregn												
Don't know	1	6.7	0	0	0	0	0	0	1	5.9	2	**10.5**
MCH 1.10 Affordability: Extent to which all MCH-related services and drugs are a												
Don't know	0	0	0	0	0	0	0	0	1	5.9	0	0

Note:

- For readability purpose, complete wording for questions/variables has been provided as Appendix 1.
- Only the percentages of respondents who responded “don’t know” to the respective question or sub-question was included in this table.
- Highlighted are values > 10%

For peer review only

MCH 1.11 Private Sector: Extent to which the private sector (doctors, midwives,												
Don't know	0	0	0	0	0	0	0	0	1	5.9	1	5.3
MCH 1.12 MCH Department: Extent to which the Ministry of Health has a department												
Don't know	0	0	1	5.6	0	0	0	0	1	5.9	0	0
MCH 1.13 Potential Solutions: Extent to which the government recognizes potentia												
MCH 1.13.1	1	6.7	1	5.6	0	0	0	0	1	5.9	0	0
MCH 1.13.2	0	0	1	5.9	0	0	0	0	1	5.9	0	0
MCH 1.13.3	0	0	2	**11.1**	0	0	0	0	1	5.9	0	0
MCH 1.13.4	0	0	1	5.6	0	0	0	0	1	5.9	0	0
MCH 1.13.5	0	0	1	5.6	0	0	0	0	1	5.9	0	0
MCH 2.1 Obstetric Care Availability ,Äì All Centers: Extent to which all primary h												
MCH 2.1.2	0	0	1	6.2	0	0	0	0	1	6.2	1	5.9
MCH 2.1.3	1	7.7	2	**12.5**	0	0	0	0	1	6.2	1	5.9
MCH 2.1.4	1	7.7	2	**12.5**	0	0	0	0	1	6.2	1	5.9
MCH 2.1.5	1	8.3	2	**12.5**	0	0	0	0	1	6.2	2	**11.8**
MCH 2.1.6	1	7.7	0	0	0	0	0	0	1	6.2	1	5.9
MCH 2.1.7	0	0	1	6.2	0	0	0	0	1	6.2	0	0
MCH 2.1.8	0	0	0	0	0	0	0	0	1	6.2	0	0
MCH 2.2 Obstetric Care Availability ,Äì Secondary Healthcare Facilities: Extent to												
MCH 2.2.1	0	0	0	0	0	0	0	0	1	6.2	1	5.9

Note:

- For readability purpose, complete wording for questions/variables has been provided as Appendix 1.
- Only the percentages of respondents who responded “don’t know” to the respective question or sub-question was included in this table.
- Highlighted are values > 10%

For peer review only

MCH 2.2.2	1	8.3	0	0	0	0	0	0	1	6.2	3	**17.6**
MCH 2.2.3	0	0	1	6.2	0	0	0	0	1	6.2	1	6.7
MCH 2.3 Pregnancy-Related Services: Extent to which all pregnant women have adeq												
MCH 2.3.1	1	7.7	1	6.2	0	0	0	0	1	6.2	2	**11.8**
MCH 2.3.2	1	7.7	0	0	0	0	0	0	1	6.2	2	**11.8**
MCH 2.3.3	1	7.7	1	6.2	0	0	0	0	1	6.2	2	**11.8**
MCH 2.3.4	1	7.7	1	6.2	0	0	0	0	1	6.2	1	6.2
MCH 2.3.5	1	7.7	1	6.2	0	0	0	0	1	6.2	1	5.9
MCH 2.3.6	0	0	1	6.2	0	0	0	0	1	6.2	1	6.7
MCH 2.3.7	0	0	1	6.2	0	0	0	0	1	6.2	0	0
MCH 2.3.8	0	0	0	0	0	0	0	0	1	6.2	0	0
MCH 2.3.9	1	7.7	1	6.7	0	0	0	0	1	6.2	2	**12.5**
MCH 2.4 Ante-natal Care for Unmarried and HIV Positive Women: Extent to which, a												
MCH 2.4.1	0	0	1	6.2	1	**12.5**	0	0	1	6.2	3	**17.6**
MCH 2.4.2	1	7.7	1	6.2	1	**12.5**	0	0	1	6.2	3	**17.6**
MCH 2.4.3	0	0	2	**12.5**	0	0	0	0	1	6.2	3	**17.6**
MCH 2.4.4	0	0	1	6.7	0	0	0	0	1	6.2	1	5.9
MCH 2.5 Newborn Care: Extent to which, for newborn care, all infants whether del												
MCH 2.5.1	1	7.7	2	**12.5**	0	0	0	0	1	6.2	3	**17.6**
MCH 2.5.2	1	7.7	2	**12.5**	0	0	0	0	1	6.2	3	**17.6**
MCH 2.5.3	0	0	1	6.2	0	0	0	0	1	6.2	0	0
MCH 2.5.4	0	0	2	**12.5**	0	0	0	0	1	6.2	1	5.9

Note:

- For readability purpose, complete wording for questions/variables has been provided as Appendix 1.
- Only the percentages of respondents who responded “don’t know” to the respective question or sub-question was included in this table.
- Highlighted are values > 10%

For peer review only

MCH 2.5.5	1	7.7	3	**18.8**	0	0	0	0	1	6.2	3	**17.6**
MCH 2.5.6	0	0	1	6.2	0	0	0	0	1	6.2	0	0
MCH 2.5.7	0	0	1	6.2	0	0	0	0	1	6.2	0	0
MCH 2.6 Family Planning ‚Äì All Centers: Extent to which there exists proper provi												
MCH 2.6.1	0	0	0	0	1	**12.5**	0	0	1	6.2	1	5.9
MCH 2.6.2	0	0	1	6.2	0	0	0	0	1	6.2	0	0
MCH 2.6.3	0	0	2	**12.5**	0	0	0	0	1	6.2	0	0
MCH 2.6.4	0	0	1	6.2	0	0	0	0	1	6.2	0	0
MCH 2.6.5	0	0	1	6.7	0	0	0	0	1	6.2	0	0
MCH 2.7 Family Planning ‚Äì District/Sub-county Hospitals: Extent to which there e												
MCH 2.7.1	1	7.7	1	6.2	1	**12.5**	0	0	1	6.2	1	5.9
MCH 2.7.2	1	7.7	1	6.7	0	0	0	0	1	6.2	0	0
MCH 2.7.3	0	0	1	6.2	0	0	0	0	1	6.2	0	0
MCH 2.7.4	0	0	2	**12.5**	0	0	0	0	1	6.2	0	0
MCH 2.7.5	0	0	1	6.2	1	**12.5**	0	0	1	6.2	2	**12.5**
MCH 2.7.6	0	0	2	**12.5**	1	**12.5**	0	0	1	6.2	2	**11.8**
MCH 2.7.7	0	0	1	6.2	0	0	0	0	1	6.2	0	0
MCH 2.8 Childhood Illness: Extent to which the WHO-recommended case management o												
MCH 2.8.1	0	0	1	6.7	1	**12.5**	0	0	1	6.2	1	5.9
MCH 2.8.2	0	0	1	6.2	0	0	0	0	1	6.7	1	5.9
MCH 3.1 Government Messages: Extent to which high officials in the government, i												

Note:

- For readability purpose, complete wording for questions/variables has been provided as Appendix 1.
- Only the percentages of respondents who responded “don’t know” to the respective question or sub-question was included in this table.
- Highlighted are values > 10%

For peer review only

Don't know	0	0	0	0	0	0	0	0	1	6.2	0	0
MCH 3.2 Mass Media: Extent to which the national program uses the mass media to												
Don't know	0	0	0	0	0	0	0	0	1	6.2	0	0
MCH 3.3 Community-Level Media: Extent to which community groups take part in sys												
Don't know	0	0	0	0	0	0	0	0	1	6.2	0	0
MCH 3.4 Educational Materials: Extent to which the appropriate ministry (MOH) su												
Don't know	1	7.7	0	0	0	0	0	0	1	6.2	0	0
MCH 3.5 Self-Review of Maternal Mortality Cases: Extent to which each health fac												
Don't know	0	0	1	8.3	0	0	0	0	1	6.2	1	6.7
MCH 3.6 Informing Policies with New Evidence: Extent to which each health facili												
Don't know	0	0	1	8.3	0	0	0	0	1	6.2	0	0
MCH 4.1 Strength of Observatory/Stakeholder/Technical Working Group for MCH: Ext												
MCH 4.1.1	0	0	0	0	1	**12.5**	0	0	1	6.2	0	0
MCH 4.1.2	0	0	1	8.3	0	0	0	0	1	6.2	1	7.7
MCH 4.1.3	0	0	0	0	0	0	0	0	1	6.2	1	7.1
MCH 4.2 MCH Research Strategy: Extent to which national documents include a comp												
Don't know	0	0	0	0	0	0	0	0	3	**18.8**	1	7.1
MCH 4.3 Data-Gathering Partners:												

Note:

- For readability purpose, complete wording for questions/variables has been provided as Appendix 1.
- Only the percentages of respondents who responded "don't know" to the respective question or sub-question was included in this table.
- Highlighted are values > 10%

For peer review only

MCH 4.3.1	0	0	1	8.3	0	0	0	0	1	6.2	1	7.1
MCH 4.3.2	0	0	2	**16.7**	0	0	0	0	1	6.2	1	7.1
MCH 4.3.3	0	0	1	9.1	1	**12.5**	0	0	1	6.2	1	7.7
MCH 4.4 Topical Research:												
MCH 4.4.1	0	0	2	**16.7**	1	**12.5**	0	0	1	6.2	1	7.1
MCH 4.4.2	0	0	1	9.1	1	**12.5**	0	0	1	6.2	1	7.1
MCH 4.4.3	0	0	1	8.3	1	**12.5**	0	0	1	6.2	1	7.1
MCH 4.4.4	0	0	4	**33.3**	1	**12.5**	0	0	1	6.2	1	7.1
MCH 4.5 Quality/Coverage of Data: Extent to which current research/data is disag												
MCH 4.5.1	0	0	2	**16.7**	0	0	0	0	1	6.2	1	7.1
MCH 4.5.2	0	0	1	8.3	0	0	0	0	1	6.2	1	7.1
MCH 4.5.3	0	0	2	**16.7**	0	0	0	0	1	6.2	1	7.1
MCH 4.5.4	0	0	2	**16.7**	0	0	0	0	1	6.2	2	**14.3**
MCH 4.5.5	0	0	3	**25**	0	0	0	0	1	6.2	2	**14.3**
MCH 4.6 Record-Keeping: Extent to which systems for client recordkeeping, clinic												
Don't know	0	0	1	8.3	0	0	0	0	1	6.2	1	7.1
MCH 4.7 Quality Research Institutions: Extent to which the country has the capac												
Don't know	0	0	1	8.3	0	0	0	0	1	6.2	1	7.1
MCH 4.8 Evaluation: Extent to which program statistics, surveys, and small studi												
Don't know	0	0	1	8.3	0	0	0	0	1	6.2	1	7.1

Note:

- For readability purpose, complete wording for questions/variables has been provided as Appendix 1.
- Only the percentages of respondents who responded “don’t know” to the respective question or sub-question was included in this table.
- Highlighted are values > 10%

For peer review only

MCH 4.9 Management‚Äôs Use of Evaluation Findings: Extent to which local-level pro												
Don't know	0	0	1	8.3	0	0	0	0	1	6.2	2	**14.3**
MCH 4.10 Ministerial Use of Evaluation Findings: Extent to which the Ministry ad												
Don't know	0	0	1	8.3	0	0	0	0	1	6.2	1	7.1
MCH 4.11 Dissemination of Information to Other Implementing Bodies: Extent to wh												
Don't know	0	0	2	**16.7**	0	0	0	0	1	6.2	1	7.1
MCH 5.1 CSO Actor Power: Extent to which CSO actors are in strong placements wit												
Don't know	0	0	1	10	0	0	0	0	1	6.7	2	**14.3**
MCH 5.2 Budget Analysis as a CSO Tool: Extent to which CSOs utilize budget analy												
Don't know	2	**20**	1	10	0	0	0	0	1	6.7	3	**21.4**
MCH 5.3 Facility-Based Service Delivery Support: Extent to which CSOs are involv												
Don't know	0	0	0	0	0	0	0	0	1	6.7	1	7.1
MCH 5.4 Community-Based Service Delivery Support: Extent to which CSOs participa												
Don't know	0	0	0	0	0	0	0	0	1	6.7	1	7.1
MCH 5.5 Mobile Outreach for Community-Based Service Delivery Support: Extent to												
Don't know	0	0	0	0	0	0	0	0	1	6.7	1	7.1
MCH 5.6 Social Franchising: Extent to which CSOs are involved with social franch												

Note:

- For readability purpose, complete wording for questions/variables has been provided as Appendix 1.
- Only the percentages of respondents who responded “don’t know” to the respective question or sub-question was included in this table.
- Highlighted are values > 10%

For peer review only

For peer review only - http://bmjopen.bmj.com/site/about/guidelines.xhtml

Don't know	0	0	0	0	0	0	0	0	1	6.7	2	**14.3**
MCH 5.7 mHealth: Extent to which CSOs are leveraging mHealth to utilize text mes												
Don't know	0	0	1	10	0	0	0	0	1	6.7	2	**14.3**
MCH 5.8 Human Rights and Quality of Care: Extent to which CSOs advocate for and												
Don't know	0	0	1	10	0	0	0	0	1	6.7	2	**14.3**
MCH 5.9 Social and Behavior Change (SBC): Extent to which CSOs support SBC inter												
Don't know	0	0	1	10	0	0	0	0	1	6.7	1	7.1
MCH 5.10 Advocacy/Accountability: Extent to which CSOs support the training and												
Don't know	0	0	0	0	0	0	0	0	1	6.7	1	7.1
MCH 5.11 CSO-Led Assessment and Monitoring: Extent to which CSOs assess, monitor												
Don't know	0	0	0	0	0	0	0	0	1	6.7	1	7.1
MCH 5.12 Forming Partnerships between CSOs: Extent to which CSOs have formed nat												
Don't know	1	10	1	10	0	0	0	0	1	6.7	2	**14.3**
ED 1.1 Quality: Extent to which there are national plans/strategies/programs to												
ED 1.2 Disadvantaged Groups:												
Don't know	3	**20**	0	0	2	**18.2**	0	0	0	0	0	0
Don't know	1	6.7	1	**20**	0	0	0	0	0	0	0	0
ED 1.3 Teachers: Extent to which teacher recruitment, development and management												

Note:

- For readability purpose, complete wording for questions/variables has been provided as Appendix 1.
- Only the percentages of respondents who responded “don’t know” to the respective question or sub-question was included in this table.
- Highlighted are values > 10%

For peer review only

ED 1.4 Teachers,Äô Accountability: Extent to which national plans include specific												
Don't know	1	6.7	0	0	0	0	0	0	0	0	0	0
ED 1.5 Teacher Quality: Extent to which education policies/strategies/programs i												
Don't know	1	6.7	0	0	0	0	0	0	0	0	0	0
ED 1.6 Collaboration in Education: Extent to which the policy makes provisions t												
ED 1.7 Gender Parity SDG: Extent to which national policies/plans/strategies con												
Don't know	1	6.7	0	0	0	0	0	0	0	0	0	0
ED 1.8 Education Financing: Extent to which new education plans include financin												
Don't know	2	**13.3**	0	0	0	0	0	0	0	0	0	0
ED 1.9 Gender Parity Planning: Extent to which there are national plans/strategi												
ED 1.9.1	0	0	0	0	0	0	1	**14.3**	0	0	0	0
ED 1.9.2	0	0	0	0	0	0	1	**14.3**	0	0	0	0
ED 1.9.3	0	0	0	0	3	**27.3**	0	0	0	0	0	0
ED 1.10 Adult Illiteracy: Extent to which national policies/plans/strategies aim												
ED 1.10.1	2	**13.3**	0	0	1	8.3	0	0	0	0	0	0
ED 1.10.2	2	**13.3**	0	0	1	8.3	0	0	0	0	0	0
ED 1.11 Out-of-School Children: Extent to which policies address the plight of o												
Don't know	1	6.7	0	0	0	0	0	0	0	0	0	0

Note:

- For readability purpose, complete wording for questions/variables has been provided as Appendix 1.
- Only the percentages of respondents who responded “don’t know” to the respective question or sub-question was included in this table.
- Highlighted are values > 10%

For peer review only

ED 1.12 Technology Integration: Extent to which strategies have been developed a												
ED 1.13. Technology Literacy: Extent to which resources have been allocated to i												
ED 2.1 Teacher Deployment: Extent to which strategies/plans are used to ensure t												
Don't know	2	**13.3**	0	0	0	0	0	0	0	0	0	0
ED 2.2 Teacher Quality: Extent to which teacher quality is recognized as a key f												
Don't know	0	0	1	**20**	0	0	0	0	0	0	0	0
ED 2.3 Gender Parity: ¬†Extent to which resources have been utilized to improve e												
Don't know	1	6.7	0	0	0	0	0	0	0	0	0	0
ED 2.4 Parental Involvement in Education: Extent to which parents are involved i												
ED 2.5 Standardized Assessments: Extent to which all schools utilize standardize												
Don't know	2	**13.3**	0	0	0	0	0	0	0	0	0	0
ED 2.6. Use of Assessment to Inform Progress: Extent to which adjustments to sch												
Don't know	2	**13.3**	0	0	1	8.3	0	0	0	0	0	0
ED 3.1 Equality: Extent to which equity and equality in education have been high												
ED 3.2 Quality: Extent to which quality of education takes part in advocacy-rela												
Don't know	1	7.1	0	0	0	0	0	0	0	0	0	0

Note:

- For readability purpose, complete wording for questions/variables has been provided as Appendix 1.
- Only the percentages of respondents who responded “don’t know” to the respective question or sub-question was included in this table.
- Highlighted are values > 10%

For peer review only

ED 3.3 Teachers,Äô Obstacles: Extent to which the needs of teachers have been high												
ED 3.4 Institutions,Äô Obstacles: Extent to which the challenges of educational in												
ED 4.1 Strength of Observatory/Stakeholder/Technical Working Group for ED: Exten												
ED 4.1.1	2	**14.3**	1	**20**	0	0	0	0	0	0	0	0
ED 4.1.2	2	**16.7**	0	0	1	8.3	0	0	0	0	0	0
ED 4.1.3	2	**15.4**	0	0	0	0	0	0	0	0	0	0
ED 4.2 Education/Research Strategy: Extent to which the national education plan												
Don't know	1	7.1	0	0	1	8.3	0	0	0	0	0	0
ED 4.3 Data Gathering Partners:												
ED 4.3.1	1	7.1	0	0	0	0	0	0	0	0	0	0
ED 4.3.2	1	7.1	0	0	1	9.1	0	0	0	0	0	0
ED 4.3.3	0	0	0	0	1	8.3	0	0	0	0	0	0
ED 4.4 Topical Research:												
ED 4.4.1	0	0	0	0	1	8.3	0	0	0	0	0	0
ED 4.4.2	0	0	0	0	1	8.3	0	0	0	0	0	0
ED 4.4.3	0	0	0	0	1	9.1	0	0	0	0	0	0
ED 4.4.4	0	0	1	**20**	1	8.3	0	0	0	0	0	0
ED 4.5 Quality/Coverage of Data: Extent to which current research/data is sex an												
ED 4.5.1	2	**14.3**	0	0	0	0	0	0	0	0	0	0

Note:

- For readability purpose, complete wording for questions/variables has been provided as Appendix 1.
- Only the percentages of respondents who responded “don’t know” to the respective question or sub-question was included in this table.
- Highlighted are values > 10%

For peer review only

ED 4.5.2	1	7.1	0	0	0	0	0	0	0	0	0	0
ED 4.5.3	1	7.1	0	0	0	0	0	0	0	0	0	0
ED 4.5.4	1	7.1	0	0	0	0	0	0	0	0	0	0
ED 4.6 Record-Keeping: Extent to which systems for education-related recordkeepi												
Don't know	1	7.1	0	0	1	8.3	0	0	0	0	0	0
ED 4.7 Quality Research Institutions: Extent to which the country has the capaci												
Don't know	1	7.1	1	**20**	1	8.3	0	0	0	0	0	0
ED 4.8 Evaluation: Extent to which education program statistics, surveys, and sm												
Don't know	0	0	0	0	1	8.3	0	0	0	0	0	0
ED 4.9 Management‚Äôs Use of Evaluation Findings: Extent to which local-level educ												
Don't know	1	7.1	0	0	1	8.3	0	0	0	0	0	0
ED 4.10 Ministerial Use of Evaluation Findings: Extent to which the relevant min												
Don't know	4	**28.6**	0	0	1	8.3	0	0	0	0	0	0
ED 4.11 Dissemination of Information to Other Implementing Bodies:												
ED 5.1 CSO Actor Power: Extent to which CSO actors are in strong placements with												
Don't know	1	7.1	1	**20**	1	8.3	0	0	0	0	0	0
ED 5.2 Budget Analysis as a CSO Tool: Extent to which CSOs utilize budget analys												
Don't know	5	**35.7**	2	**40**	2	**16.7**	0	0	0	0	0	0

Note:

- For readability purpose, complete wording for questions/variables has been provided as Appendix 1.
- Only the percentages of respondents who responded “don’t know” to the respective question or sub-question was included in this table.
- Highlighted are values > 10%

For peer review only

ED 5.3 Community inclusion: Extent to which CSOs are involved in efforts to expl												
Don't know	0	0	0	0	2	16.7	0	0	0	0	0	0
ED 5.4 Community Involvement in Education: Extent to which CSOs participate in b												
Don't know	0	0	0	0	1	8.3	0	0	0	0	0	0
ED 5.5 Social Franchising: Extent to which CSOs are involved with social franchi												
Don't know	0	0	1	20	1	8.3	0	0	0	0	0	0
ED 5.6 Technology: Extent to which CSOs are leveraging information communication												
Don't know	0	0	1	20	1	8.3	0	0	0	0	0	0
ED 5.7 Human Rights and Access to/Quality of Education: Extent to which CSOs adv												
Don't know	0	0	0	0	2	16.7	0	0	0	0	0	0
ED 5.8 Social and Behavior Change (SBC): Extent to which CSOs support SBC interv												
Don't know	0	0	0	0	2	16.7	0	0	0	0	0	0
ED 5.9 Advocacy/Accountability: Extent to which CSOs support the training and ca												
Don't know	0	0	1	20	2	16.7	0	0	0	0	0	0
ED 5.10 CSO-Led Assessment and Monitoring: Extent to which CSOs assess, monitor												
Don't know	1	7.1	1	20	2	16.7	0	0	1	11.1	0	0
ED 5.11 Forming Partnerships between CSOs: Extent to which CSOs have formed nati												

Note:

- For readability purpose, complete wording for questions/variables has been provided as Appendix 1.
- Only the percentages of respondents who responded “don’t know” to the respective question or sub-question was included in this table.
- Highlighted are values > 10%

For peer review only

Don't know	1	7.1	1	**20**	2	**16.7**	0	0	0	0	0	0
WE 1.1 Gender Equality Policy: Extent to which gender equality is considered/int												
WE 1.2 Level of commitment to the Convention on the Elimination of all Forms of												
WE 1.3 Equity in Marriage and Family Policies: Extent to which the principle of												
WE 1.4 Policy on Age at Marriage: Extent to which legal age at marriage for fema												
WE 1.5 Violence Against Women Measures: Level of regulations and measures on vio												
Don't know	0	0	0	0	0	0	1	6.2	0	0	0	0
WE 1.6 Female Genital Cutting/Mutilation: Level of regulation and measures on fe												
Don't know	0	0	0	0	0	0	1	6.2	0	0	0	0
WE 1.7 Women‚Äôs Health Measures: Level of protocols for the protection of women‚Äôs												
WE 1.8 Education Measures: Extent to which there are mechanisms to promote girls												
WE 1.9 Rights Measures: Level of enforcement of policies on women‚Äôs rights.												
WE 1.10 Equitable Work: Extent to which there are policies on equitable working												
WE 1.11 Equitable Land/Asset Ownership: Extent to which there are policies to af												

Note:

- For readability purpose, complete wording for questions/variables has been provided as Appendix 1.
- Only the percentages of respondents who responded “don’t know” to the respective question or sub-question was included in this table.
- Highlighted are values > 10%

For peer review only

WE 1.12 Conflict, Women & Peacebuilding: Level of implementation of Security UN												
Don't know	3	**37.5**	1	10	0	0	0	0	0	0	1	7.7
WE 1.13 Gender Quotas in Politics: Existence, and level of implementation, of qu												
WE 1.14 Sustainable Development Goal 5: Level of commitment to fulfilling Sustai												
Don't know	1	**12.5**	0	0	0	0	0	0	0	0	0	0
WE 1.15 Customary Law Revision: Extent to which customary laws have been reviewe												
Don't know	0	0	0	0	0	0	4	**25**	0	0	0	0
WE 1.16 Costing for WE: Extent to which there is sufficient planning and allocat												
Don't know	2	**25**	2	**20**	0	0	0	0	0	0	0	0
WE 1.17 Staffing for WE: Extent to which:												
WE 1.18 Transparency and Accountability: Extent to which the government provides												
Don't know	1	**12.5**	0	0	1	**11.1**	0	0	0	0	0	0
WE 2.1 Gender Mainstreaming: Extent to which services or programs within and acr												
WE 2.2 Gender-Transformative Programming: Extent to which services or programs w												
Don't know	0	0	1	10	0	0	0	0	0	0	0	0
WE 2.3 Education: Extent to which education services or programs focus on girl‚Äôs												

Note:

- For readability purpose, complete wording for questions/variables has been provided as Appendix 1.
- Only the percentages of respondents who responded “don’t know” to the respective question or sub-question was included in this table.
- Highlighted are values > 10%

For peer review only

WE 2.4. Dropouts: Extent to which there are programs to prevent female dropouts.												
WE 2.5 Technology Literacy: Extent to which technology literacy is promoted amon												
WE 2.6 Employment: Extent to which job-creation programs and labor market police												
WE 2.7 Women as Mothers and Workers: Extent to which the government makes possib												
WE 2.8 Family Planning for Empowerment: Extent to which FP services or programs												
Don't know	0	0	0	0	0	0	0	0	0	0	1	8.3
WE 2.9 Political and Public Representation: Extent of implementation of programs												
WE 2.10 Violence: Extent of fight against violence against women and children an												
WE 2.11 Child Marriage: Extent of law enforcement to eliminate child marriage.												
WE 2.12 Female Genital Mutilation/Cutting: Extent of law enforcement to eliminat												
Don't know	1	**12.5**	0	0	0	0	3	**18.8**	0	0	0	0
WE 3.1 Advocates: Extent to which advocates for girls‚Äô and women‚Äôs empowerment e												
Don't know	1	**12.5**	0	0	0	0	0	0	0	0	0	0
WE 3.2. Girls and Women: Extent to which the advocacy efforts in this area encom												
Don't know	1	**12.5**	0	0	0	0	0	0	0	0	0	0

Note:

- For readability purpose, complete wording for questions/variables has been provided as Appendix 1.
- Only the percentages of respondents who responded "don't know" to the respective question or sub-question was included in this table.
- Highlighted are values > 10%

For peer review only

WE 3.3 Information Dissemination: Extent to which the government undertakes suff												
Don't know	1	**12.5**	0	0	0	0	0	0	0	0	0	0
WE 3.4 Leveraging Partnerships: Extent to which the government leverages partner												
Don't know	1	**12.5**	0	0	0	0	0	0	0	0	0	0
WE 3.5 Employment: Extent to which stakeholders call for laws, policies and prog												
Don't know	1	**12.5**	0	0	0	0	0	0	0	0	0	0
WE 3.6 Education: Extent to which a priority of advocates is to promote the fulf												
Don't know	1	**12.5**	0	0	0	0	0	0	0	0	0	0
WE 3.7 Violence: Extent to which advocates successfully engage with the governme												
Don't know	1	**12.5**	0	0	0	0	1	6.2	0	0	0	0
WE 4.1 Strength of Observatory/Stakeholder/Technical Working Group for WE: Exten												
WE 4.1.1	1	**12.5**	0	0	0	0	0	0	0	0	0	0
WE 4.1.2	2	**28.6**	0	0	0	0	0	0	0	0	0	0
WE 4.1.3	1	**14.3**	0	0	0	0	0	0	0	0	0	0
WE 4.2 Women‚Äôs Rights Research Strategy: Extent to which the national¬†WE plan an												
Don't know	1	**12.5**	0	0	1	**11.1**	0	0	0	0	1	8.3
WE 4.3 Data Gathering Partners:												
WE 4.3.1	1	**12.5**	0	0	0	0	0	0	0	0	1	8.3

Note:

- For readability purpose, complete wording for questions/variables has been provided as Appendix 1.
- Only the percentages of respondents who responded “don’t know” to the respective question or sub-question was included in this table.
- Highlighted are values > 10%

For peer review only

WE 4.3.2	1	**14.3**	0	0	0	0	0	0	0	0	1	8.3
WE 4.3.3	2	**25**	0	0	0	0	0	0	0	0	1	8.3
WE 4.4 Topical Data Gathering:												
WE 4.4.1	1	**12.5**	0	0	0	0	0	0	0	0	1	8.3
WE 4.4.2	1	**12.5**	0	0	0	0	0	0	0	0	1	8.3
WE 4.4.3	1	**12.5**	0	0	1	**11.1**	0	0	0	0	1	9.1
WE 4.4.4	1	**12.5**	0	0	0	0	0	0	0	0	1	10
WE 4.5 Quality/Coverage of Data:												
WE 4.5.1	1	**12.5**	1	10	0	0	0	0	0	0	1	9.1
WE 4.5.2	1	**12.5**	1	10	0	0	0	0	0	0	1	8.3
WE 4.5.3	2	**25**	1	10	0	0	0	0	0	0	1	8.3
WE 4.6 Quality Research Institutions: Extent to which the country supports and s												
Don't know	2	**25**	1	10	0	0	0	0	0	0	2	**16.7**
WE 4.7 Evaluation: Extent to which program statistics, surveys, and small studie												
Don't know	3	**37.5**	1	10	0	0	0	0	0	0	1	8.3
WE 4.8 Management‚Äôs Use of Evaluation Findings: Extent to which local-level prog												
Don't know	3	**37.5**	0	0	0	0	0	0	0	0	1	8.3
WE 4.9 Ministerial Use of Evaluation Findings: Extent to which the Ministry admi												
Don't know	3	**37.5**	1	10	1	**11.1**	0	0	0	0	1	8.3
WE 4.10 Dissemination of Information to other Implementing Bodies: Extent to whi												

Note:

- For readability purpose, complete wording for questions/variables has been provided as Appendix 1.
- Only the percentages of respondents who responded “don’t know” to the respective question or sub-question was included in this table.
- Highlighted are values > 10%

For peer review only

Don't know	2	**25**	0	0	0	0	0	0	0	0	1	8.3
WE 5.1 CSO Actor Power: Extent to which WE civil society actors are in strong po												
Don't know	1	**14.3**	0	0	0	0	0	0	0	0	1	8.3
WE 5.2 Budget Analysis as a CSO Tool: Extent to which CSOs utilize budget analys												
Don't know	1	**14.3**	1	**11.1**	0	0	0	0	0	0	1	8.3
WE 5.3 Community-Based Service Delivery Support: Extent to which CSOs participat												
Don't know	1	**14.3**	0	0	0	0	0	0	0	0	1	8.3
WE 5.4 Human Rights and Quality of Implementation: Extent to which CSOs advocate												
Don't know	1	**14.3**	0	0	0	0	0	0	0	0	1	8.3
WE 5.5 Social and Behavior Change (SBC): Extent to which CSOs support SBC interv												
Don't know	1	**14.3**	0	0	0	0	0	0	0	0	1	8.3
WE 5.6 Youth: Extent to which CSOs promote gender empowerment through youth prog												
Don't know	1	**14.3**	0	0	1	**11.1**	0	0	0	0	1	8.3
WE 5.7 Programming for Men: Extent to which CSOs support WE policies, interventi												
Don't know	1	**14.3**	0	0	0	0	0	0	0	0	1	8.3
WE 5.8 Advocacy/Accountability: Extent to which CSOs support the training and ca												
Don't know	1	**14.3**	0	0	0	0	0	0	0	0	1	8.3

Note:

- For readability purpose, complete wording for questions/variables has been provided as Appendix 1.
- Only the percentages of respondents who responded “don’t know” to the respective question or sub-question was included in this table.
- Highlighted are values > 10%

For peer review only

WE 5.9 CSO-Led Assessment and Monitoring: Extent to which CSOs assess, monitor a												
Don't know	1	**14.3**	0	0	0	0	0	0	0	0	1	8.3
WE 5.10 Forming Partnerships between CSOs: Extent to which CSOs have formed nati												
Don't know	1	**14.3**	0	0	0	0	0	0	0	0	1	8.3
LM 1.1 Global Economic Integration: Extent to which the country aims to be integ												
LM 1.2 Regional Economic Integration: Extent to which the country aims to be int												
LM 1.3 Technology: Extent to which the government has invested in infrastructure												
LM 1.4 Government Support: Extent to which policies and plans are in place that												
Don't know	0	0	1	7.7	0	0	0	0	0	0	0	0
LM 1.5 Unemployment: Extent to which policies account for and address unemployme												
LM 1.6 Disadvantaged/Vulnerable Groups: Extent to which policies exist, and are												
LM 1.7 Climate Change and Environmental Sustainability: Extent to which the agri												
LM 1.8 Urbanization: ¬†Extent to which policies and planning anticipate and are p												
Don't know	1	**11.1**	0	0	0	0	0	0	0	0	0	0
LM 1.9 Service-Led Economy: Extent to which policies and framework are in place												

Note:

- For readability purpose, complete wording for questions/variables has been provided as Appendix 1.
- Only the percentages of respondents who responded “don’t know” to the respective question or sub-question was included in this table.
- Highlighted are values > 10%

For peer review only

LM 1.10 Corporate Social Responsibility: Extent to which national legislation in												
Don't know	2	**22.2**	0	0	0	0	1	**11.1**	0	0	0	0
LM 1.11 Targeted Labor Market Investments: Extent to which the government has ta												
LM 1.12 Self-Employment: Extent to which policies support raising the returns to												
LM 1.13 Microcredit: Extent to which the government enables access to microcredi												
Don't know	0	0	0	0	0	0	1	**11.1**	0	0	0	0
LM 1.14 Savings: Extent to which the government supports the creation of accessi												
LM 1.15 Skill Acquisition: Extent to which policies exist, and are effectively i												
LM 2.1 Unemployment Reduction: Extent to which there are efforts toward developi												
Don't know	0	0	1	7.7	0	0	0	0	0	0	0	0
LM 2.2. Youth Unemployment: Extent to which labor market programs have effective												
Don't know	0	0	1	7.7	0	0	0	0	0	0	0	0
LM 2.3 Access to Microcredit: Extent to which national and regional funds have b												
Don't know	0	0	1	7.7	0	0	0	0	0	0	0	0
LM 2.4 Harnessing Youth Special Capacities: Extent to which programs and service												
Don't know	0	0	0	0	0	0	1	**11.1**	0	0	0	0

Note:

- For readability purpose, complete wording for questions/variables has been provided as Appendix 1.
- Only the percentages of respondents who responded "don't know" to the respective question or sub-question was included in this table.
- Highlighted are values > 10%

For peer review only

LM 2.5 Early Labor Opportunities: Extent to which programs and government legisl												
LM 2.6 Youth Entrance into Labor: Extent of the promotion of youth volunteer and												
LM 2.7 Entrepreneurial Ventures: Extent to which people have enhanced access to												
LM 2.8 Resources for Youth Entrepreneurship: Extent to which entrepreneurial ven												
Don't know	0	0	0	0	0	0	1	**11.1**	0	0	0	0
LM 2.9 Vocational Opportunities: Extent to which vocational training opportuniti												
LM 2.10 Education and Labor: Extent to which improvements have been made to impr												
LM 2.11 Women-Friendly Labor: Extent to which workplaces in the private and publ												
LM 2.12 Job Security:												
Don't know	0	0	0	0	1	**12.5**	0	0	0	0	0	0
LM 2.13 Minimum Wage: Extent to which a minimum wage is applied to the ‚Äòtypical‚Äô												
LM 2.14 Labor Market Programs: Extent to which labor market programs have effect												
Don't know	1	**11.1**	0	0	0	0	0	0	0	0	0	0
LM 2.15 Informality in the Labor Market: Extent to which the programs are implem												
LM 3.1 Gender Equality in the Labor Market: Extent to which groups and individua												

Note:

- For readability purpose, complete wording for questions/variables has been provided as Appendix 1.
- Only the percentages of respondents who responded “don’t know” to the respective question or sub-question was included in this table.
- Highlighted are values > 10%

For peer review only

LM 3.2 Youth Equality in the Labor Market: Extent to which individuals and group												
Don't know	1	11.1	0	0	0	0	0	0	0	0	0	0
LM 3.3 Minimum Wage Advocacy: Extent to which advocacy groups are successful in												
Don't know	0	0	1	7.7	0	0	0	0	0	0	0	0
LM 3.4 Government Advocacy: Extent to which members of the government advocates												
Don't know	0	0	1	7.7	0	0	0	0	0	0	0	0
LM 3.5 Protections: Extent to which advocacy exists for job protection, and soci												
Don't know	0	0	1	7.7	0	0	0	0	0	0	0	0
LM 4.1 Strength of Observatory/Stakeholder/Technical Working Group for the LM: E												
LM 4.1.1	1	11.1	2	15.4	0	0	0	0	0	0	0	0
LM 4.1.2	1	11.1	2	15.4	0	0	0	0	0	0	0	0
LM 4.1.3	1	11.1	3	23.1	0	0	0	0	0	0	0	0
LM 4.2 Labor Market Research Strategy: Extent to which the national documents in												
Don't know	0	0	3	23.1	0	0	0	0	0	0	0	0
LM 4.3 Labor Market Analysis Partners:												
LM 4.3.1	0	0	2	15.4	0	0	0	0	0	0	0	0
LM 4.3.2	0	0	2	15.4	0	0	0	0	0	0	0	0
LM 4.3.3	0	0	2	15.4	1	12.5	0	0	0	0	0	0
LM 4.4 Areas of Research in the Labor Market:												

Note:

- For readability purpose, complete wording for questions/variables has been provided as Appendix 1.
- Only the percentages of respondents who responded “don’t know” to the respective question or sub-question was included in this table.
- Highlighted are values > 10%

For peer review only

LM 4.4.1	0	0	1	7.7	1	**12.5**	0	0	0	0	0	0
LM 4.4.2	0	0	1	7.7	1	**12.5**	0	0	0	0	0	0
LM 4.4.3	0	0	1	7.7	1	**12.5**	0	0	0	0	0	0
LM 4.4.4	0	0	2	**15.4**	1	**12.5**	0	0	0	0	0	0
LM 4.5 Quality/Coverage of Data:												
LM 4.5.1	0	0	2	**15.4**	1	**12.5**	0	0	0	0	0	0
LM 4.5.2	0	0	2	**15.4**	1	**12.5**	0	0	0	0	0	0
LM 4.5.3	0	0	2	**15.4**	1	**12.5**	0	0	0	0	0	0
LM 4.6 Record-Keeping: Extent to which systems for labor market recordkeeping, r												
Don't know	0	0	2	**15.4**	1	**12.5**	0	0	0	0	0	0
LM 4.7 Quality Research Institutions: Extent to which the country has the capaci												
Don't know	0	0	2	**15.4**	1	**12.5**	0	0	0	0	0	0
LM 4.8 Evaluation: Extent to which program statistics, surveys, and small studie												
Don't know	0	0	1	7.7	1	**12.5**	0	0	0	0	0	0
LM 4.9 Management‚Äôs Use of Evaluation Findings: Extent to which local-level prog												
Don't know	0	0	1	7.7	1	**12.5**	0	0	0	0	0	0
LM 4.10 Ministerial Use of Evaluation Findings: Extent to which the Ministry adm												
Don't know	0	0	2	**15.4**	1	**12.5**	0	0	0	0	0	0
LM 4.11 Dissemination of Information to Other Implementing Bodies: Extent to whi												

Note:

- For readability purpose, complete wording for questions/variables has been provided as Appendix 1.
- Only the percentages of respondents who responded “don’t know” to the respective question or sub-question was included in this table.
- Highlighted are values > 10%

For peer review only

Don't know	0	0	1	7.7	1	**12.5**	0	0	0	0	0	0
LM 5.1 CSO Actor Power: Extent to which CSO actors are in strong positions to in												
Don't know	1	**11.1**	1	7.7	0	0	0	0	0	0	0	0
LM 5.2 Budget Analysis as a CSO Tool: Extent to which CSOs utilize budget analys												
Don't know	2	**22.2**	2	**15.4**	0	0	1	**11.1**	0	0	0	0
LM 5.3 Community-Based Service Delivery Support: Extent to which CSOs participat												
Don't know	2	**22.2**	1	7.7	0	0	1	**11.1**	0	0	0	0
LM 5.4 Diversification of Job Opportunities: Extent to which CSOs and the privat												
Don't know	2	**22.2**	1	7.7	0	0	1	**11.1**	0	0	0	0
LM 5.5 Human Rights and Quality of Implementation: Extent to which CSOs support												
Don't know	3	**33.3**	1	7.7	0	0	1	**11.1**	0	0	0	0
LM 5.6 Youth: Extent to which CSOs support labor market policies, interventions												
Don't know	2	**22.2**	1	7.7	0	0	0	0	0	0	0	0
LM 5.7 Programming for Women: Extent to which CSOs support labor market policies												
Don't know	2	**22.2**	1	7.7	0	0	0	0	0	0	0	0
LM 5.8 Advocacy/Accountability: Extent to which CSOs support the training and ca												
Don't know	2	**22.2**	1	7.7	0	0	1	**11.1**	0	0	0	0

Note:

- For readability purpose, complete wording for questions/variables has been provided as Appendix 1.
- Only the percentages of respondents who responded “don’t know” to the respective question or sub-question was included in this table.
- Highlighted are values > 10%

For peer review only

LM 5.9 CSO-Led Assessment and Monitoring: Extent to which CSOs assess, monitor a												
Don't know	2	**22.2**	1	7.7	0	0	1	**11.1**	0	0	0	0
LM 5.10 Forming Partnerships between CSOs: Extent to which the private sector ha												
Don't know	2	**22.2**	1	7.7	0	0	1	**11.1**	0	0	0	0
GEI 1.1 Democratic Values: Extent to which the government has ratified, domestic												
Don't know	1	**11.1**	0	0	0	0	1	**16.7**	0	0	0	0
GEI 1.2 Monitoring Democratic Values: Extent to which the government has created												
Don't know	1	**11.1**	0	0	1	8.3	1	**16.7**	0	0	1	**12.5**
GEI 1.3 Affirmation of Youth Democratic Participation: Extent to which the gover												
Don't know	0	0	0	0	0	0	1	**16.7**	0	0	0	0
GEI 1.4. Removing Legal Youth Barriers: Extent to which the government has prosc												
Don't know	0	0	0	0	0	0	1	**16.7**	0	0	0	0
GEI 1.5 Youth Policy: Extent to which the youth policy takes into account the la												
Don't know	0	0	0	0	0	0	1	**16.7**	0	0	0	0
GEI 1.6 Affirmation of Democratic Participation/Revision of Customary Law: Exten												
Don't know	0	0	0	0	1	8.3	1	**16.7**	0	0	0	0
GEI 1.7 Youth Policymaking Participation: Extent to which the government ensures												

Note:

- For readability purpose, complete wording for questions/variables has been provided as Appendix 1.
- Only the percentages of respondents who responded “don’t know” to the respective question or sub-question was included in this table.
- Highlighted are values > 10%

For peer review only

Don't know	0	0	1	**16.7**	1	8.3	1	**16.7**	0	0	0	0
GEI 1.8 Rule of Law: Extent to which the rule of law is formally, legally establ												
Don't know	1	**11.1**	0	0	1	8.3	1	**16.7**	0	0	0	0
GEI 1.9 Transparency and Accountability: Extent to which national legislation ma												
Don't know	0	0	0	0	0	0	1	**16.7**	0	0	0	0
Don't know	0	0	0	0	1	8.3	1	**16.7**	0	0	0	0
GEI 1.10 National Security: Extent to which the country/government is involved i												
Don't know	1	**11.1**	1	**16.7**	0	0	1	**16.7**	0	0	2	**25**
Don't know	0	0	0	0	0	0	1	**16.7**	0	0	2	**25**
Don't know	1	**11.1**	0	0	0	0	1	**16.7**	0	0	3	**37.5**
GEI 1.11 Rights: Extent to which the constitution affirms certain rights: freedo												
Don't know	0	0	0	0	0	0	1	**16.7**	0	0	0	0
GEI 1.12 Education and Democratic Values: Extent to which education policies, st												
Don't know	0	0	0	0	0	0	1	**16.7**	0	0	0	0
GEI 1.13 Affirmation of Safe Learning Environments: Extent to which education po												
Don't know	0	0	0	0	0	0	1	**16.7**	0	0	0	0
GEI 2.1 Strengthening Youth Activities/Institutions: Extent to which efforts are												
Don't know	1	**12.5**	0	0	0	0	0	0	0	0	0	0

Note:

- For readability purpose, complete wording for questions/variables has been provided as Appendix 1.
- Only the percentages of respondents who responded “don’t know” to the respective question or sub-question was included in this table.
- Highlighted are values > 10%

For peer review only

GEI 2.2 Appointment of Youth Leader: Whether and to what extent there has been a												
Don't know	1	**12.5**	0	0	0	0	0	0	0	0	1	**12.5**
GEI 2.3 Youth Ideals and Aspirations: Extent to which programming is designed to												
Don't know	1	**12.5**	0	0	0	0	0	0	0	0	0	0
GEI 2.4 Youth Services: Extent to which programming is designed to address youth												
Don't know	1	**12.5**	0	0	1	8.3	0	0	0	0	0	0
GEI 2.5 Youth Involvement in National Initiatives: Extent to which there are eff												
Don't know	1	**12.5**	0	0	1	8.3	0	0	0	0	0	0
GEI 2.6 Personal Safety: Extent to which individual safety is provided for throu												
GEI 2.7 Participation: Extent to which government functions involve the particip												
GEI 2.8 Gender: Extent to which women participate in:												
GEI 2.8.1	2	**25**	0	0	0	0	0	0	0	0	0	0
GEI 2.8.2	0	0	1	**20**	0	0	0	0	0	0	0	0
GEI 2.8.3	0	0	0	0	0	0	0	0	0	0	2	**25**
GEI 2.9 Promotion of Equality: Extent to which gender equality in all aspects of												
Don't know	0	0	0	0	1	8.3	0	0	0	0	0	0
GEI 2.10 Sustainable Economic Opportunity:												
GEI 2.11 Human Development:												

Note:

- For readability purpose, complete wording for questions/variables has been provided as Appendix 1.
- Only the percentages of respondents who responded “don’t know” to the respective question or sub-question was included in this table.
- Highlighted are values > 10%

For peer review only

GEI 2.11.1	0	0	0	0	1	8.3	0	0	0	0	0	0
GEI 2.11.2	0	0	0	0	1	8.3	0	0	0	0	0	0
GEI 2.11.3	0	0	0	0	1	8.3	0	0	0	0	0	0
GEI 2.11.4	0	0	0	0	1	8.3	0	0	0	0	0	0
GEI 3.1 Youth Empowerment: Extent to which youth empowerment has been enhanced,												
Don't know	0	0	0	0	1	9.1	0	0	0	0	0	0
GEI 3.2 Government Advocacy: Extent to which members of the government advocates												
Don't know	0	0	1	**20**	1	9.1	0	0	0	0	0	0
GEI 3.3 Non-governmental Advocacy: Extent to which non-governmental actors advoc												
Don't know	0	0	1	**20**	2	**18.2**	0	0	0	0	0	0
GEI 3.4 Civil Society Organizations (CSOs): Extent to which CSOs specifically op												
Don't know	0	0	0	0	1	9.1	0	0	0	0	0	0
GEI 3.5 Advocacy Voice: Extent to which advocates have a voice in government dis									**Tanzania**	**Tanzania**	**Total**	**Total**
Don't know	0	0	0	0	1	9.1	0	0	0	0	1	2.7
GEI 3.6 Voice of Instrumental Sectors of the DD: Extent to which the government												
GEI 3.6.1	0	0	0	0	1	9.1	0	0	0	0	0	0
GEI 3.6.2	0	0	0	0	1	9.1	0	0	0	0	0	0
GEI 3.6.3	0	0	0	0	1	9.1	0	0	0	0	0	0
GEI 3.6.4	0	0	0	0	1	9.1	0	0	0	0	0	0

Note:

- For readability purpose, complete wording for questions/variables has been provided as Appendix 1.
- Only the percentages of respondents who responded “don’t know” to the respective question or sub-question was included in this table.
- Highlighted are values > 10%

For peer review only

GEI 3.6.5	0	0	0	0	2	**18.2**	0	0	0	0	0	0
GEI 3.6.6	0	0	0	0	1	9.1	0	0	0	0	0	0
GEI 4.1 Strength of Observatory/Stakeholder/Technical Working Group for GEI: Ext												
GEI 4.1.1	1	**12.5**	0	0	1	9.1	0	0	0	0	0	0
GEI 4.1.2	2	**25**	0	0	1	9.1	0	0	0	0	0	0
GEI 4.1.3	2	**25**	0	0	1	9.1	0	0	0	0	0	0
GEI 4.2¬†Education/Research Strategy: Extent to which national planning documents												
Don't know	0	0	0	0	2	**18.2**	0	0	0	0	1	**14.3**
GEI 4.3 Governance, Financial and Development Data Gathering Partners:												
GEI 4.3.1	0	0	0	0	1	9.1	0	0	0	0	0	0
GEI 4.3.2	0	0	0	0	2	**18.2**	0	0	0	0	0	0
GEI 4.3.3	0	0	1	**20**	1	9.1	0	0	0	0	0	0
GEI 4.4 Governance, Financial and Development Topical Data Gathering:												
GEI 4.4.1	1	**12.5**	0	0	1	9.1	0	0	0	0	0	0
GEI 4.4.2	0	0	0	0	1	9.1	0	0	0	0	2	**28.6**
GEI 4.4.3	0	0	0	0	2	**18.2**	0	0	0	0	0	0
GEI 4.4.4	0	0	0	0	1	9.1	0	0	0	0	0	0
GEI 4.5 Quality/Coverage of Data:												
GEI 4.5.1	0	0	0	0	2	**18.2**	0	0	0	0	1	**14.3**
GEI 4.5.2	0	0	0	0	2	**18.2**	0	0	0	0	1	**14.3**

Note:

- For readability purpose, complete wording for questions/variables has been provided as Appendix 1.
- Only the percentages of respondents who responded “don’t know” to the respective question or sub-question was included in this table.
- Highlighted are values > 10%

For peer review only

GEI 4.5.3	0	0	0	0	2	18.2	0	0	0	0	0	0
GEI 4.5.4	0	0	0	0	2	18.2	0	0	2	28.6	4	10.8
GEI 4.6 Quality Research Institutions: Extent to which the country has the capac												
Don't know	0	0	0	0	1	9.1	0	0	0	0	0	0
GEI 4.7 Evaluation: Extent to which program statistics, surveys, and small studi												
Don't know	0	0	0	0	1	9.1	0	0	0	0	0	0
GEI 4.8 Management,Äôs Use of Evaluation Findings: Extent to which local-level pro												
Don't know	0	0	0	0	1	9.1	0	0	0	0	0	0
GEI 4.10 Dissemination of Information to Other Implementing Bodies: Extent to wh												
Don't know	0	0	0	0	1	9.1	0	0	0	0	0	0
GEI 5.1 CSO Actor Power: Extent to which CSO actors are in strong positions to p												
Don't know	0	0	0	0	1	9.1	1	16.7	0	0	0	0
GEI 5.2 Budget Analysis as a CSO Tool: Extent to which CSOs utilize budget analy												
Don't know	3	42.9	0	0	1	9.1	1	16.7	0	0	0	0
GEI 5.3 Community-Based Service Delivery Support: Extent to which CSOs participa												
Don't know	0	0	0	0	1	9.1	1	16.7	0	0	0	0
GEI 5.4 Human Rights and Quality of Implementation: Extent to which CSOs advocat												
Don't know	1	14.3	0	0	1	9.1	1	16.7	0	0	0	0

Note:

- For readability purpose, complete wording for questions/variables has been provided as Appendix 1.
- Only the percentages of respondents who responded “don’t know” to the respective question or sub-question was included in this table.
- Highlighted are values > 10%

For peer review only

GEI 5.5 Youth: Extent to which CSOs support youth to participate in education, t												
Don't know	1	**14.3**	0	0	1	9.1	1	**16.7**	0	0	0	0
GEI 5.6 Programs for Women: Extent to which CSOs ensure GEI policies support wom												
Don't know	0	0	0	0	1	9.1	1	**16.7**	0	0	0	0
GEI 5.7 Advocacy/Accountability												
Don't know	0	0	0	0	2	**18.2**	1	**16.7**	0	0	0	0
Don't know	0	0	0	0	2	**18.2**	1	**16.7**	0	0	0	0
GEI 5.8 CSO-Led Assessment and Monitoring: Extent to which CSOs assess, monitor												
Don't know	0	0	0	0	1	9.1	1	**16.7**	0	0	0	0
GEI 5.9 Forming Partnerships between CSOs: Extent to which CSOs have formed nati												
Don't know	0	0	0	0	1	9.1	1	**16.7**	0	0	0	0

Note:

- For readability purpose, complete wording for questions/variables has been provided as Appendix 1.
- Only the percentages of respondents who responded “don’t know” to the respective question or sub-question was included in this table.
- Highlighted are values > 10%

For peer review only

STROBE checklist — A new demographic dividend effort index (DDEI): results from an experts' survey from six sub-Saharan African countries.

	Page	Recommendation (Strobe)
**Title and abstract**	1	(*a*) Indicate the study's design with a commonly used term in the title or the abstract
		(*b*) Provide in the abstract an informative and balanced summary of what was done and what was found
**Introduction**		
Background/rationale	3-4	Explain the scientific background and rationale for the investigation being reported
**Methods**		
Study design	5	Present key elements of study design early in the paper
Setting	5-6	Describe the setting, locations, and relevant dates, including periods of recruitment, exposure, follow-up, and data collection
Participants	6	*Cross-sectional study*—Give the eligibility criteria, and the sources and methods of selection of participants
Variables	6	Clearly define all outcomes, exposures, predictors, potential confounders, and effect modifiers. Give diagnostic criteria, if applicable
Data sources/ measurement	5-7	For each variable of interest, give sources of data and details of methods of assessment (measurement). Describe comparability of assessment methods if there is more than one group
Bias	6	Describe any efforts to address potential sources of bias
Study size	6	Explain how the study size was arrived at
Quantitative variables	6-7	Explain how quantitative variables were handled in the analyses. If applicable, describe which groupings were chosen and why
Statistical methods	6-7	(*a*) Describe all statistical methods, including those used to control for confounding
		(*b*) Describe any methods used to examine subgroups and interactions
		(*c*) Explain how missing data were addressed
		(*d*) *Cross-sectional study*—If applicable, describe analytical methods taking account of sampling strategy
		(*e*) Describe any sensitivity analyses

Continued on next page

For peer review only

**Results**		
Participants	6	(a) Report numbers of individuals at each stage of study—eg numbers potentially eligible, examined for eligibility, confirmed eligible, included in the study, completing follow-up, and analysed
Descriptive data	8	(a) Give characteristics of study participants (eg demographic, clinical, social) and information on exposures and potential confounders
Outcome data	8-9	*Cross-sectional study*—Report numbers of outcome events or summary measures
Main results	9-16	(*a*) Give unadjusted estimates and, if applicable, confounder-adjusted estimates and their precision (eg, 95% confidence interval). Make clear which confounders were adjusted for and why they were included
		(*b*) Report category boundaries when continuous variables were categorized
		(*c*) If relevant, consider translating estimates of relative risk into absolute risk for a meaningful time period
Other analyses	16	Report other analyses done—eg analyses of subgroups and interactions, and sensitivity analyses
**Discussion**		
Key results	16-18	Summarise key results with reference to study objectives
Limitations	18	Discuss limitations of the study, taking into account sources of potential bias or imprecision. Discuss both direction and magnitude of any potential bias
Interpretation	17-19	Give a cautious overall interpretation of results considering objectives, limitations, multiplicity of analyses, results from similar studies, and other relevant evidence
Generalisability	17-19	Discuss the generalisability (external validity) of the study results
**Other information**		
Funding	20	Give the source of funding and the role of the funders for the present study and, if applicable, for the original study on which the present article is based

For peer review only

For peer review only - http://bmjopen.bmj.com/site/about/guidelines.xhtml

BMJ Open

A new demographic dividend effort index (DDEI): results from an experts' survey from six sub-Saharan African countries.

Journal:	*BMJ Open*
Manuscript ID	bmjopen-2021-059937.R2
Article Type:	Original research
Date Submitted by the Author:	21-Dec-2022
Complete List of Authors:	Rusatira, Jean Christophe; Johns Hopkins University Bloomberg School of Public Health, PHFR<br>Cardona, Carolina; Johns Hopkins University Bloomberg School of Public Health, Population Family and Reproductive Health<br>Martinez-Baack, Michelle ; Johns Hopkins University Bloomberg School of Public Health, PHFR<br>Rimon, Jose; Johns Hopkins University Bloomberg School of Public Health, Population, Family and Reproductive Health<br>Ahmed, Saifuddin; Johns Hopkins University Bloomberg School of Public Health, Population, Family And Reproductive Health
**Primary Subject Heading**:	Global health
Secondary Subject Heading:	Global health, Health policy
Keywords:	PUBLIC HEALTH, HEALTH ECONOMICS, Demography < TROPICAL MEDICINE, STATISTICS & RESEARCH METHODS, International health services < HEALTH SERVICES ADMINISTRATION & MANAGEMENT

SCHOLARONE™
Manuscripts

I, the Submitting Author has the right to grant and does grant on behalf of all authors of the Work (as defined in the below author licence), an exclusive licence and/or a non-exclusive licence for contributions from authors who are: i) UK Crown employees; ii) where BMJ has agreed a CC-BY licence shall apply, and/or iii) in accordance with the terms applicable for US Federal Government officers or employees acting as part of their official duties; on a worldwide, perpetual, irrevocable, royalty-free basis to BMJ Publishing Group Ltd ("BMJ") its licensees and where the relevant Journal is co-owned by BMJ to the co-owners of the Journal, to publish the Work in this journal and any other BMJ products and to exploit all rights, as set out in our licence.

The Submitting Author accepts and understands that any supply made under these terms is made by BMJ to the Submitting Author unless you are acting as an employee on behalf of your employer or a postgraduate student of an affiliated institution which is paying any applicable article publishing charge ("APC") for Open Access articles. Where the Submitting Author wishes to make the Work available on an Open Access basis (and intends to pay the relevant APC), the terms of reuse of such Open Access shall be governed by a Creative Commons licence – details of these licences and which Creative Commons licence will apply to this Work are set out in our licence referred to above.

Other than as permitted in any relevant BMJ Author's Self Archiving Policies, I confirm this Work has not been accepted for publication elsewhere, is not being considered for publication elsewhere and does not duplicate material already published. I confirm all authors consent to publication of this Work and authorise the granting of this licence.

For peer review only

A new demographic dividend effort index (DDEI): results from an experts' survey from six sub-Saharan African countries.

Authors
Jean Christophe Rusatira*, Carolina Cardona*, Michelle Martinez-Baack*, Jose G. Rimon*, Saifuddin Ahmed*

* Department of Population, Family and Reproductive Health, Johns Hopkins Bloomberg School of Public Health

Corresponding Author
E-mail address: jcrusatira@jhu.edu
Department of Population, Family and Reproductive Health
Johns Hopkins Bloomberg School of Public Health
615 N. Wolfe St., Baltimore, MD 21030, USA

Objective: This study aimed at developing a tool to measure the extent of national efforts in policies, services, research, and programs implemented to cultivate and harness the benefits of a potential demographic dividend in six Sub-Saharan African countries.

Design: The survey was self-administered online using the SurveyMonkey platform. The survey questionnaire covered six key sectors: governance and economic institutions, family planning, maternal and child health, education, women's empowerment, and the labor market. Each sector-specific questionnaire was structured around five practice domains: policymaking, services and programs, advocacy, research, and civil society. Each item was scored from 1 to 10. Factor analysis was used to select the items to be retained for final score estimation. Simple averages were computed to estimate sectoral and domain scores and overall country scores were estimated using weighted country mean scores. Internal consistency, construct validity and reliability were examined using factor analysis and Cronbach's alpha.

Setting: Sub-Saharan Africa

Participants: A total of 440 knowledgeable informants from six countries; namely, Ethiopia (73), Kenya (69), Nigeria (67), Rwanda (54), Senegal (81), and Tanzania (96).

Results: Based on the results from factor analysis, 38 items were dropped from the analysis and Cronbach's alpha results ranged from 0.84-0.98 across domains. The overall Demographic Dividend Effort Index (DDEI) scores ranged between 5.4 (95% CI:5.1-5.8) in Ethiopia to 7.7 (95% CI:7.5-8.0) in Rwanda. In most countries, the disaggregated scores by sector revealed low scores in the labor market and women's empowerment.

Conclusion: The DDEI scores highlight important gaps in key health and development sectors. The DDEI proved to be a reliable and internally consistent tool for effort measurement in key demographic dividend sectors. The DDEI can serve as a self-evaluation tool for local actors and may complement existing quantitative tools such as the Global Gender Gap and the Human Capital Index.

Strengths and limitations of the study

- The study questionnaire was developed using existing effort indices and integrated inputs from local partners working in different health and development sectors.
- The study collected data from knowledgeable informants from six countries that are in the early stages of their demographic transition to explore the applicability and utility of a demographic dividend effort index.
- The Demographic Dividend Effort Index revealed overall high reliability and internal consistency for measuring the efforts in six key demographic dividend sectors across the six countries.
- The study relied on expert opinions working in different sectors in respective countries, which may be prone to bias and may not be comparable across countries.
- A structural equation model could not be implemented for validation because of sample size limitations.

peer review only

For peer review only - http://bmjopen.bmj.com/site/about/guidelines.xhtml

Background

The reduction of births and infant deaths during the third stage of the demographic transition produces changes in the age structure of a population.[1] These changes present countries with an opportunity to experience a demographic dividend (DD) characterized by economic growth, sustainable development, and social change.[2-4] These demographic changes can spur economic growth by increasing the number of working-age individuals compared to the number of young dependents. However, favorable policies and investments are required for countries to harness the DD opportunity presented by such population age structure changes.

Across the Sub-Saharan African (SSA) region, more than three in four countries are still in the early stages of their demographic transition. They are projected not to reach the peak of the transition, where the DD occurs, until 2050.[5] Although this transition started more than 50 years ago, with declines in fertility levels from an average of 6.8 children per woman to an average of 4.8 children by the mid-2010s, it has been slower compared to other continents.[2] Currently, working-age individuals represent about 60% of the population, and this proportion is expected to keep increasing in the coming few decades.[6] These shifting population dynamics present a unique opportunity for SSA countries to grow their economies and break the cycle of poverty and poor health that has become chronic in the majority of these countries.

Previous literature has identified that, in Asian countries, the DD was responsible for around one-third of their economic growth between the 1960s and 1990s.[7, 8] These experiences have sparked an interest among African countries. Over the last decade, countries in the region have embarked on the DD development agenda. The majority of the African Union (AU) member states have developed their DD roadmaps and strategic plans.[9] However, there is no standard tool to measure the extent of national efforts in policies, services, research, advocacy, and programs implemented to cultivate, realize, and harness the benefits of a potential DD in different SSA countries.

The AU and member states recognize that the DD is a key component to achieving sustainable socio-economic growth. In 2017, the AU established the theme of the year as "Harnessing the Demographic Dividend Through Investments in Youth". This theme generated momentum for the DD across the continent, particularly after the development of the AU DD roadmap. The roadmap identifies labor, education, health, governance, and youth empowerment as five fundamental pillars of the DD.[9] Cardona and colleagues further expanded these pillars based on their findings from a systematic literature review that highlighted six key sectors for cultivating and capitalizing on the DD potential in SSA.[10] These sectors are governance and economic institutions (GEI), family planning (FP), maternal and child health (MCH), education (ED), women's empowerment (WE), and the labor market (LM).

A central feature to enable a favorable policy environment, and attain a DD, is good governance and strong economic institutions. Governments have the power to attract foreign direct investments to create jobs and contribute to the economy. Strong financial institutions can promote a culture of saving, supported by favorable interest rates to fuel development. Bloom and colleagues have previously documented the correlation between economic stability, institutional quality and economic growth and have evidenced that unstable countries miss out on their DD opportunities.[2, 11, 12]

Political and economic stability creates an opportunity for a more prosperous community through successful health and development programs. Successful FP programs have been proven to improve

women's participation in the workforce and have been identified as key interventions to reduce maternal and child mortality due to their impact on birth spacing and fertility levels.[13-16] The reduction of infant mortality is also intertwined with the reduction in fertility and is directly associated with the reduction in maternal mortality. Conley and colleagues evidenced that a reduction of 0.5-0.9 points in the total fertility rate (TFR) was associated with a fall of 50 deaths per 1,000 live births in the child mortality rate.[17] This association is concordant with Becker's theory about the quantity-quality trade-off of children, which predicts that parental investments per child increase as fertility levels decline.[18] As families become smaller, more resources are freed to support children's development and the household as a whole.[15, 19]

Related to the quantity-quality trade-off, investments in human capital per child increase as fertility lowers, which can accelerate the economy. Ahmed and colleagues found a potential additional 22.4% increase in GDP per capita in SSA from an increase of just 13% in education attainment between 2011-2030, which eventually increases the availability of skilled workers.[20] Equitable access to quality education for boys and girls is also necessary for countries to generate skilled workers that can later support their economies. Moreover, the education of girls plays an important role in empowering women, who can later enter the labor force and foster socio-economic growth in SSA countries.[21]

Africa's young population presents an opportunity for SSA countries to transform their economies. However, to seize this opportunity, countries may require different strategies depending on their demographic transition stage. On the one hand, countries that are still in the initial stage of their demographic transition, also referred to as "pre-dividend", still need to accelerate their demographic transition and develop the human capital of the growing youth population.[19, 22] These countries are characterized by a total fertility rate (TFR) of 4 or higher children per woman and a projected growing working-age population by 2030.[19, 22] On the other hand, countries that are in the middle of their demographic transition, also referred to as "early-dividend", require smart investments in human capital development so that they can fill various job opportunities in the labor market. These countries are characterized by having a TFR of fewer than 4 children per woman and a projected growing working-age population by 2030.[19, 22] For the socio-economic growth to happen, multisectoral efforts are required and countries need to generate jobs faster than their population growth, explore innovative strategies to transition young graduates to formal jobs, and facilitate job creation in the private sector.[23]

Over the last four decades, effort measurements have been proven to be reliable approaches to informing international health and development programs. Since the 1970s, the Family Planning Effort Index (FPEI) has provided reliable estimates to measure the strength of family planning programs in more than 90 countries and has been proven to be valid relative to family planning outcomes.[24-26] Building on the success of the FPEI, the same approach has been applied in HIV/AIDS programs and maternal and child health which led to the development of the AIDS Program Effort Index (API) in more than 35 countries[27] and the Maternal and Neonatal Program Index (MNPI) in more than 46 countries.[28] However, because health and development systems do not operate in silos, the successful implementation of FPEI, API and MNPI presents an opportunity to develop a multidimensional index to measure DD progress in SSA.

This study aimed at developing an effort assessment index as a standard measure to quantify the nature and strength of in-country DD efforts in the key sectors identified as necessary to harness a DD. This

For peer review only - http://bmjopen.bmj.com/site/about/guidelines.xhtml

index is meant to be used by national and global actors in the development sector working in five development domains: policy, services and programs, advocacy, research, and civil society.

Methodology

Study design

Data were collected through an online survey that measured the efforts that countries are devoting to maximize their potential to reap the benefits of a DD. The survey was administered through an internet-based modality using the SurveyMonkey platform in six countries: Kenya, Senegal, Nigeria, Rwanda, Tanzania, and Ethiopia. These countries were selected because of where they stand in the demographic transition and the convenience to successfully implement the study. The selection of countries sought to include both pre-dividend and early-dividend countries[29] in which the study team had ongoing existing partners with the ability to engage a diverse pool of respondents. The survey(s) was administered in two waves to accommodate Coronavirus Disease 2019 (COVID-19) lockdowns in different countries. The first wave occurred from August to November 2020, and the second wave that covered Senegal alone took place from March to May 2021. The main language of the questionnaire was English (Appendix 1), and a French version (Appendix 2) was created for Senegal by a certified translator.

The questionnaires were developed for each of the six sectors recommended by Cardona and colleagues: FP, MCH, ED, WE, LM, and GEI.[10] All questionnaires were structured around five domains: 1) policy; 2) services and programs; 3) advocacy; 4) research; and 5) civil society. These domains were selected based on prior similar effort indices implemented in various health and development areas.[27, 28, 30, 31] These domains also corresponded to the areas of practice in the field and were confirmed by the local partners during the study design process. The Demographic Dividend Effort Index (DDEI) questionnaire was composed of 490 items divided across the six sectors (112 items for FP, 101 items for MCH, 61 items for ED, 71 items for WE, 68 items for LM, and 77 items for GEI). Where available, the development of each sector-specific questionnaire integrated validated questions from existing effort indices. For instance, the Family Planning Effort Index (FPEI) questionnaire[30] was integrated into the FP questionnaire. Similarly, specific questions from the Maternal and Neonatal Program Index (MNPI),[28] the Rwanda Governance Scorecard,[32] and the AIDS Program Effort Index (API)[27] were integrated into different sector-specific questionnaires. Additional questions were drafted based on DD-favorable interventions for respective sectors as summarized in the wheel of prosperity by Cardona et al.[10] More details on the questions drawn from existing indices have been provided as supplemental material (Appendix 3). During data collection, participants only ever received single surveys that pertained to their sector of expertise and never the entirety of the aggregate survey. Each item was scored on a scale of 1 to 10, from weaker (1) to stronger (10) perceived level of effort or strength. The scoring range of 1-10 has been used in the FPEI and API[27, 30] and allowed a wide variability needed to detect differences between domains and sectors.

The DDEI questionnaire was pilot-tested among 27 experts from Nigeria, Ethiopia, Tanzania, Senegal, Kenya and Rwanda from June to August 2020. This pilot study allowed local experts to provide their feedback and inputs to the questionnaire, which were integrated into the final revision. The local experts were requested to provide qualitative feedback on content, conciseness, and completeness by sector, and were requested to provide recommendations on changes to be made. Following the feedback from local experts, the language and content of the questionnaire were amended to improve clarity and conciseness. The final questionnaire was shared with local project partners for final review and approval before

administering at the national level across all six countries. Each sector-specific questionnaire included items that assessed the presence and strength of policies, services and programs, advocacy efforts, research, and the involvement of civil society organizations in that sector. No personal identification or socio-demographic characteristics information was collected to limit the concern of being identifiable for the respondents and minimize information bias.

Study participants

The in-country partners identified the potential participants who were knowledgeable in respective domains and sectors in each country. In-country partners included the Centre d'Excellence Régional en Economie Générationnelle in Senegal, the Center for Communication Programs in Nigeria, the National Council for Population and Development in Kenya, the Tanzania Communication and Development Center, the Ethiopian Academy of Sciences, and the Centre for Integrated Development Research and Action in Rwanda. Participants were considered for the survey if they were regarded to have demonstrated expertise in at least one of the six sectors by the in-country partner. Expertise was defined as having worked in one of the health or development sectors at the national level as a policymaker, advocate, service provider, researcher, or member of a civil society organization. The local study partners had the best knowledge of potential respondents and led the data collection process locally.

As with other similar indices, sampling was necessarily purposive given the need to identify knowledgeable respondents. The design entailed the selection of 10 respondents for each sector. Whenever possible, diversity across the five domains of expertise was prioritized. As such, to reach 10 respondents per sector, each of the six sectors was designed to have two respondents with sector expertise in one of the five domains. These were exclusive classifications such that respondents could only be counted toward the completion of one sector-specific questionnaire. The survey was administered through an online modality, "SurveyMonkey", in each country. Local partners in respective countries appointed one contact person to respond to any queries regarding the study. Requests to complete the surveys were sent through existing mailing lists of national technical working groups and up to three follow-up calls were made to all individuals for improving survey response rates. The sample size goal was 60 respondents per country.

The expected sample size was on the higher end of sample sizes for similar indices such as the Human Resources Effort Index (N = 16-28 per country), the FPEI (N = 1-12 per country), the API (N = 20-25 per country), and the MNPI (N=10-25).[27, 28, 30, 31] These indices similarly surveyed across various sectors and, sometimes, domains of expertise. However, each of the existing indices was focused on one specific topic, while the DD required a multisectoral sample frame. As this study aimed for 60 respondents per country, considering an expected response rate similar to that of the FPEI of 49%, which was the highest among the other comparative indices, this study ultimately aimed for at least 30 respondents per country.

Patient and Public involvement

No patients or members of the public were involved in the research design, data analysis, or dissemination of the findings. Local researchers and policymakers contributed to the design of the questionnaire, the implementation of the survey, and were involved in the interpretation of the results.

Analysis

Exploratory data analysis

Exploratory data analysis was done to summarize sample characteristics and distribution of respondents across sectors and domains of expertise at the country level. The proportions of respondents by sector, area of expertise and country were presented in a table to facilitate an easier visualization.

Factor and Cronbach's alpha analysis

Factor analysis was implemented to assess construct internal validity for each domain in each sector. This process allowed us to identify the latent structure revealed by the data. The analysis was conducted by respective sectors retaining one factor for each domain to identify which items loaded on that domain. This process allowed us to assess how each of the items included in the questionnaire loaded on the domain under which the item was administered. Items were retained if their factor loadings were 0.5 or higher. This threshold has been recommended by Costello and Osborne as a more reliable threshold that allows for the selection of items that strongly influence the domain, which improves the internal validity of the measurement.[33] Cronbach's alpha estimates were generated to assess the reliability of the retained items for each domain. A coefficient above 0.60 signals an adequate reliability coefficient.[34]

Index scores

The items retained from factor analysis were used to compute mean scores for countries, sectors, and domains. Sectoral and domain scores were computed as the simple average across the items retained. The total score for each country was computed as a weighted average that accounts for differences in the number of participants across sectors. Hence, sectoral weights for the total score were constructed as the ratio between the number of participants within a sector and the total number of participants for each country. For all scores, corresponding 95% confidence intervals (CI) were calculated with a *t*-distribution because the sample size was relatively small. These estimates were presented graphically for ease of interpretation and comparison across sectors per country.

Data translation and availability

To establish policy relevance, the results from this analysis were shared with in-country partners for review and policy translation. The partners from respective countries conducted a series of multisectoral meetings and workshops, in-person and virtual, to generate policy recommendations based on the results from the analysis. The data have been made available through the Harvard Dataverse open-access repository.[35]

Heterogeneity testing

Given that the measures were collected from different groups, we formally checked for heterogeneity statistically. Statistical testing for heterogeneity among respondents was done using meta-analytical techniques. This analysis used pooled data by country to allow for sufficient sample size and assessment of heterogeneity across domains. To assess the overall extent of heterogeneity and whether it was adequate to compute summary estimates, the fixed-effects were compared to random-effect estimates. By using this method, substantial discrepancies between the estimates from the two models indicate considerable heterogeneity that can make summary estimates misleading.[36] To further assess heterogeneity across domains in respective countries I-squared (I^2) and Tau-squared were used. The I^2 values of 25%, 50%, and 75% have been interpreted as representing small, moderate, and high levels of heterogeneity.[37] Tau-squared captures the variance between measurements of efforts by domains which can be captured by random-effect meta-analytic models.

For peer review only - http://bmjopen.bmj.com/site/about/guidelines.xhtml

Results

Respondents

The sample characteristics of the participants are presented by sector and domain of expertise at the country level (Table 1). The total sample consisted of 440 respondents distributed as follows: Ethiopia (73), Kenya (69), Nigeria (67), Rwanda (54), Senegal (81), and Tanzania (96). The number of respondents, domain of expertise, and sectors varied across countries. In all countries, except Tanzania, the majority of respondents were from the public sector and the services and programs domains. The average completion rate was 82% and ranged from 78% in Tanzania to 92% in Rwanda. The intended number of participants per sector was achieved in all six countries.

Table 1: Sample Characteristics

	Ethiopia	Kenya	Nigeria	Rwanda	Senegal	Tanzania
	% (Obs.)	% (Obs.)	% (Obs.)	% (Obs.)	% (Obs.)	% (Obs.)
**Sector**						
Family Planning	23.3 (17)	24.6 (17)	26.9 (18)	13.0 (7)	8.6 (7)	39.6 (38)
Maternal and Child Health	20.5 (15)	24.6 (17)	11.9 (8)	16.7 (9)	19.8 (16)	19.8 (19)
Education	20.5 (15)	7.2 (5)	17.9 (12)	13.0 (7)	13.6 (11)	10.4 (10)
Women's Empowerment	11.0 (8)	14.5 (10)	13.4 (9)	29.6 (16)	21.0 (17)	13.5 (13)
Labor Market	12.3 (9)	18.8 (13)	11.9 (8)	16.7 (9)	14.8 (12)	8.3 (8)
Governance and Economic Institutions	12.3 (9)	10.1 (7)	17.9 (12)	11.1 (6)	22.2 (18)	8.3 (8)
**Organization**						
Public Sector	21.9 (16)	68.1 (47)	56.7 (38)	83.3 (45)	63.6 (49)	13.5 (13)
Private Sector	5.5 (4)	0.0 (0)	3.0 (2)	13.0 (7)	10.4 (8)	9.4 (9)
Non-governmental organization	27.4 (20)	27.5 (19)	28.4 (19)	1.9 (1)	20.8 (16)	66.7 (64)
University/research institute	41.1 (30)	1.4 (1)	3.0 (2)	0.0 (0)	3.9 (3)	2.1 (2)
Other (please specify)	4.1 (3)	2.9 (2)	9.0 (6)	1.9 (1)	1.3 (1)	8.3 (8)
**Domain**						
Policymaking	13.7 (10)	20.3 (14)	26.9 (18)	14.8 (8)	17.3 (14)	7.3 (7)
Services or programs	21.9 (16)	58.0 (40)	37.3 (25)	18.5 (10)	66.7 (54)	52.1 (50)
Advocacy	8.2 (6)	11.6 (8)	13.4 (9)	14.8 (8)	8.6 (7)	25.0 (24)
Research	53.4 (39)	5.8 (4)	17.9 (12)	46.3 (25)	2.5 (2)	10.4 (10)
Civil Society	2.7 (2)	4.3 (3)	4.5 (3)	5.6 (3)	4.9 (4)	5.2 (5)
Obs.	73	69	67	54	81	96

Factor and Cronbach's alpha analysis

The items that loaded poorly (<0.5) were dropped from further analysis, which reduced the number of items from 112 to 85 items for FP, 101 to 94 items for MCH, 68 to 65 items for LM and 77 to 76 items for GEI. For detailed factor analysis results by domain and sector, see Appendix 4. Cronbach's alpha analysis results revealed excellent reliability with alpha estimates ranging from 0.84 in LM to 0.98 in ED (Table 2).

Table 2. Results from Cronbach's alpha analysis

Sector	Domain				
	Policy	Services and Programs	Advocacy	Research	Civil Society
**Family Planning**	0.85	0.93	0.93	0.95	0.97
**Maternal and Child Health**	0.94	0.99	0.92	0.98	0.97
**Education**	0.95	0.92	0.89	0.97	0.98

For peer review only - http://bmjopen.bmj.com/site/about/guidelines.xhtml

**Women's Empowerment**	0.98	0.96	0.94	0.98	0.97
**Labor Market**	0.95	0.95	0.84	0.96	0.97
**Governance and Economic Institutions**	0.95	0.97	0.94	0.97	0.94

Index scores

The weighted overall country scores were computed to account for differences in the number of participants per sector. For example, in Ethiopia, there were 17 participants from the family planning sector, while there were only 8 participants in the women's empowerment sector. It would not be a balanced score if it assigned the same weight across sectors. Overall, the weighted average scores ranged from 5.4 (95% CI:5.1-5.8) in Ethiopia to 7.7 (95% CI:7.5-8.0) in Rwanda (Table 3). The second highest scores were for Tanzania (6.3, 95% CI:6.0-6.6) and Senegal (6.3, 95% CI:5.9-6.7). The second and third lowest scores were for Nigeria (5.5, 95% CI: 5.1-5.9) and Kenya (5.9, 95% CI: 5.5-6.2), respectively.

Table 3. National overall and sector scores

	Ethiopia	Kenya	Nigeria	Rwanda	Senegal	Tanzania
**Sector**	Mean (95% CIs) (Obs.)					
**Family Planning**	5.8 (5.3-6.3) (17)	6.2 (5.6-6.8) (17)	6.1 (5.2-7.0) (18)	6.5 (6.3-6.7) (7)	6.5 (5.1-7.8) (7)	6.3 (5.9-6.7) (38)
**Maternal and Child Health**	6.0 (4.7-7.2) (15)	6.2 (5.5-7.0) (18)	5.1 (3.8-6.4) (8)	7.4 (7.1-7.6) (9)	7.2 (6.6-7.8) (16)	6.9 (6.3-7.6) (19)
**Education**	5.3 (4.6-6.1) (15)	5.6 (3.8-7.4) (5)	5.9 (5.1-6.8) (12)	8.2 (7.3-9.1) (7)	5.9 (4.5-7.4) (11)	5.2 (4.1-6.4) (10)
**Women's Empowerment**	4.8 (3.4-6.1) (8)	5.1 (4.2-5.9) (10)	5.1 (4.1-6.1) (9)	8.3 (8.1-8.6) (16)	6.4 (5.6-7.3) (17)	6.9 (6.1-7.8) (13)
**Labor Market**	5.1 (4.3-5.9) (9)	5.3 (4.7-5.9) (13)	4.2 (2.9-5.5) (8)	7.6 (7.3-7.8) (9)	5.2 (3.7-6.7) (12)	5.7 (4.3-7.2) (8)
**Governance and Economic Institutions**	4.9 (4.1-5.7) (9)	6.5 (4.6-8.5) (6)	5.7 (4.7-6.7) (12)	7.8 (7.2-8.4) (6)	6.3 (5.7-6.9) (18)	5.4 (4.3-6.6) (8)
**Weighted Overall Score**	**5.4 (5.1-5.8) (73)**	**5.9 (5.5-6.2) (69)**	**5.5 (5.1-5.9) (67)**	**7.7 (7.5-8.0) (54)**	**6.3 (5.9-6.7) (81)**	**6.3 (6.0-6.6) (96)**

The mean scores per sector were disaggregated by domain of expertise for ease of interpretability and policy relevance. The total scores disaggregated by sector are presented in Figure 1. The radar plots indicate that the sectors with most needs across countries were LM and WE. Both in Nigeria and Ethiopia, the average score for LM and WE struggled to reach the mid-point. However, this was not the case for Rwanda and Tanzania which scored highest in WE, 8.3 (95% CI:8.1-8.6) and 6.9 (95% CI:6.1-7.8), respectively. Ethiopia was the only country that struggled to reach the mid-point in the GEI sector, 4.9 (95% CI:4.1-5.7). By contrast, the MCH, ED, and FP sectors received scores above the mid-point across all countries. For example, MCH ranged from a low of 5.1 (95% CI:3.8-6.4) in Nigeria to a high of 7.4 (95% CI:7.1-7.6) in Rwanda. Furthermore, sectoral scores revealed several interesting variations across domains and countries.

In Ethiopia, across the six sectors, none scored 5 or higher across all five domains. The scores were lowest for civil society engagement relative to other domains in FP, MCH, LM, and GEI. In most cases, policy or policymaking scored highest across sectors. Of all the scores, the highest average score was recorded in FP advocacy (6.9, 95% CI:6.0-7.9), and the lowest in research for WE (3.8, 95% CI:2.0-5.6) as well as civil society engagement in LM (3.8, 95% CI:2.3-5.4). The other scores that were below the mid-point were for services and programs in WE (4.7, 95% CI:3.3-6.1) and LM (4.8, 95% CI:4.1-5.4), advocacy in LM (4.7, 95% CI:4.1-5.4), research in WE (3.8, 95% CI:2.0-5.6) and GEI (4.6, 95% CI:3.6-5.6), and civil society engagement in MCH (4.9, 95% CI:3.4-6.4), ED (4.0, 95% CI:3.1-4.9) and GEI 4.7

For peer review only - http://bmjopen.bmj.com/site/about/guidelines.xhtml

(3.5-5.8). However, in most instances, the differences in scores between domains were not statistically significant due to overlapping confidence intervals.

In Kenya, the average scores were higher than the mid-point across sectors and domains except for research in LM (4.6, 95% CI:3.7-5.4) and civil society in MCH (4.9, 95% CI:3.2-6.7). Scores were around 6 for FP in all the domains. Similarly, except for civil society, the scores were around 6 for MCH. The scores for ED, WE and LM were mostly around 5 and hardly reached 6. GEI scored consistently highest compared to the other sectors across the five domains with the highest score in advocacy (7.0, 95% CI:4.5-9.5).

In Nigeria, most scores reached the mid-point or higher except for LM which hardly reached 5 in any of the five domains. LM scored lowest in services and programs (3.7, 95% CI:2.4-5.1), followed by policymaking (3.9, 95% CI:2.6-5.3), advocacy (4.3, 95% CI:3.0-5.7) and research (4.4, 95% CI:3.0-5.8). The FP scores were highest in nearly all domains compared to the other sectors followed by ED. MCH scores consistently scored around the mid-point or lower in research (4.8, 95% CI:3.2-6.5) and civil society (4.6, 95% CI:2.8-6.5). The scores for WE were consistently second or third lowest across the domains.

In Rwanda, scores were mostly around 7 and none of the domains were scored below the mid-point. The scores were consistently higher for WE and ED across domains and the highest was in policymaking for WE (8.7, 95% CI:8.4-9.0) and research for ED (8.4, 95% CI:7.5-9.3). The lower scores were recorded in FP in nearly all the domains and the lowest score was in services and programs (5.7, 95% CI:5.2-6.2). MCH and LM recorded second or third-lowest scores across the five domains. GEI scored the third highest scores across all five domains.

In Senegal, most scores were above the mid-point across all sectors and domains except in LM. The scores were higher in MCH with the highest in services and programs (8.0, 95% CI:7.3-8.7). The lowest scores recorded were in civil society roles in LM (4.3, 95% CI:2.7-5.8), advocacy (4.5, 95% CI:2.8-6.2), research and in services and programs (4.8, 95% CI:3.4-6.3). The scores for WE were consistently around 6 or higher, except in research (5.7, 95% CI:4.5-6.9).

In Tanzania, all the scores across sectors and domains were around 5 or higher. In nearly all the domains, WE scored higher with the highest score in civil society (8.1, 95% CI:7.5-8.6). The lowest scores were recorded mostly in ED and GEI with the lowest score in civil society participation in ED (5.0, 95% CI:3.3-6.6). MCH and FP scored second and third highest across domains, respectively, with the scores ranging from 6.2 (95% CI:5.5-6.9) to 6.9 (95% CI:6.1-7.6).

Heterogeneity testing

The heterogeneity tests by domains across countries yielded similar results for fixed and random effect models. The I^2 estimates ranged from 0.0% to 55.5% in services and programs for Rwanda. In all the other countries I^2 was 0.0% across all the domains and sectors, suggesting no heterogeneity. Tau-squared was 0.0 across all the domains, sectors, and countries, except in Rwanda where it was 0.46. For detailed heterogeneity testing results, see Appendix 5.

Discussion

This study aimed to develop a tool to assess national efforts in establishing a favorable policy environment to cultivate a demographic dividend. The study collected and analyzed data across six key sectors in Nigeria, Rwanda, Ethiopia, Senegal, Tanzania, and Kenya from key informants identified by the in-country study collaborators.

The DDEI scores revealed patterns that were helpful to guide discussions regarding needed improvements in respective countries across five domains; namely, policymaking, services and programs, advocacy, research, and civil society. The patterns of efforts scores for the LM, WE, FP, GEI and ED sectors suggested great challenges for women's self-determination and empowerment, employment, and leadership. These findings agreed with previous predictions by the World Economic Forum that women may have to wait for 100 years to pair males in leadership roles in bigger institutions and take up ministerial roles. This is currently seen in the Global Gender Gap (GGG) Report, which ranks Rwanda in the top ten countries with the smallest gender gap among countries worldwide.[38, 39]

Relatedly, Rwanda scored remarkably high on WE, although the scores were also moderately high for Tanzania and Senegal. These findings aligned with how these countries performed on the 2022 GGG report, except for Kenya. Of the 146 countries ranked globally and on a maximum score possible of 1, Rwanda was ranked 6th with a score of 0.811 and Tanzania 64th with a score of 0.719. In contrast, Ethiopia ranked 74nd (0.710), Senegal 112th (0.668), and Nigeria 123th (0.639).[38] Kenya's rank has remarkably improved from the 2020 GGG report, having moved from 109th with a score of 0.671 to 57th with a score of 0.729. It is possible that the transformations that occurred in the last two years were not captured in the effort measurements, especially because the experts were asked about their experiences over the last 3-5 years prior to the DDEI survey. However, such rapid change in GGG rank indicates the importance of repeating the effort measurements on an annual basis. Further, although the intent of the DDEI was not to rank countries, the study findings underscored the potential for mutual learning from countries and the utility of the DDEI to dive deeper into specific items that can reveal special lessons or areas that require improvements. This is especially crucial for countries that had a much larger change in ranking during the two years that preceded the report, such as Ethiopia which experienced an improvement of more than 40 ranks, and Kenya with a rise of 80 ranks in the last few years.[38, 40]

Furthermore, human capital development is essential for a country to cultivate and harness its DD potential. The 2020 Human Capital Index report revealed that the scores for all six countries of this study ranged from 0.36 in Nigeria to 0.55 in Kenya. These findings indicate that children born today in the six countries could miss out on 45% to 64% of their future earnings potential because of incomplete education and health that is not full.[41] The DDEI provided an opportunity to qualitatively compare different ongoing efforts initiated to develop human capital and the findings in MCH, ED, and WE were in agreement with the 2020 HCI findings. For instance, the lowest score for MCH was recorded in Nigeria (5.1, 95% CI:3.8-6.4), and domain-specific scores revealed larger gaps in research and civil engagement although the scores in the other domains were also low.

This study offers several strengths. First, the questionnaires and the implementation of the DDEI were conducted in collaboration with in-country partners. This collaboration fosters local ownership of the effort measurement tool. Second, high factor loadings and Cronbach's alpha suggested high internal consistency and reliability of the tool across countries. Our analysis also highlighted 38 items that did not seem to measure the same construct and were dropped from the analysis. Dropping these items improved the quality and length of the questionnaire of the DDEI. Third, the heterogeneity tests across domains

revealed negligible heterogeneity in all countries except in the domain of services for Rwanda, which indicates score consistency across domains in these countries and the validity of the summary scores generated.[42] In the future, local institutions can regularly replicate these measurements at national and sub-national levels. Fourth, unlike prior effort indices, such as the FPEI, (created in 1972), the API (created in 1999), and MNPI (created in 2000),[27, 43, 44] the DDEI integrated critical sectors into one tool, and sectoral effort measures were not done in silos due to the interconnectedness and potential synergy between sectors (e.g. family planning, education, maternal and child health, and women's empowerment). Finally, this tool provides information to constituents that could be used to hold their governments accountable and provide unconventional evidence for planning and evaluation purposes.

The DDEI was tested at the national level which opened the opportunity to be applied at the sub-national level. In countries where health and development programs are decentralized, sub-national DDEI can be transformational in guiding local budget allocation and self-evaluation. Given the paucity of quantitative data and poor utilization of the existing information systems, the DDEI has the potential to become a unique data source that informs local discussions and conversations at all administrative and planning levels. However, special emphasis should always be made that beyond strategic allocation of resources, favorable changes in age structure accompanied by a mix of jobs that allows for optimal productivity for all workers are important preconditions to fully realize the country's DD potential.[45, 46]

This study also presented a few limitations. The DDEI relied on experts' opinions, which may be affected by different factors. The respondent's judgment may be heavily influenced by their recent experience, which may not reflect the efforts placed over the years. Their judgment may be influenced by the level of their expectations, which may also be correlated with their level of satisfaction. For instance, where people have low expectations, the scores may be high despite the weak levels of effort, and vice-versa. The challenges related to the reliability of observers' judgments have been documented in other effort measurements such as the FPEI, API and MNPI.[28] However, despite this limitation, effort measures capture unique data that can guide planning and action, especially in countries where the availability of program data is limited. Although the DDEI could be used as a monitoring tool to assess the progress in the indicators that have shown relevance or relationship to DD achievements in the literature, due to the duration that may be required for a DD to happen, the DDEI estimates should not be interpreted as a prediction of achieving a DD. In addition, in some of the countries, the sample size was small for some of the domains which may have affected the reliability and validity of tests. While the sample size could be limited by available knowledgeable respondents or experts in respective sectors for specific countries, the small sample size made it impossible to conduct reliable confirmatory factor analysis.

Furthermore, other limitations worth mentioning are related to the nature of the responses and scores obtained from the respondents. The proportions of respondents who did not know the answer to the questions and consequently could not provide a score varied across sectors and countries, although no questions had a high (>10%) proportion of "don't know" responses across all six countries. The proportions were notably high in MCH research in Kenya, Nigeria and Tanzania, in ED civil society in Kenya and Nigeria, in WE advocacy in Ethiopia, in LM research and civil society in Ethiopia, Kenya and Nigeria and GEI advocacy and civil society in Nigeria and Rwanda. This finding indicates that for future surveys, some of the questions could be amended to make "don't know" items easier to interpret or removed where found ambiguous. For detailed percentages of "don't know" responses received by item across countries, see Appendix 6. Another limitation is that the aggregate average scores for the sectors modulated centering to the mid-point of 5 over the maximum possible 10. This tendency may indicate that

For peer review only - http://bmjopen.bmj.com/site/about/guidelines.xhtml

respondents may have compensated for less generous scores that they gave to some questions with more generous scoring for others. However, this is less plausible because the item-specific scores within domains revealed wide variations across scores.

Lastly, the DDEI questionnaire was administered in the midst of the COVID-19 pandemic, which led to abrupt changes in health and development systems in all countries. Although respondents provided great insights about their respective sectors, it is not unrealistic to assume the response rates and responses received may have been affected by the status of pandemics in their countries. Additional research will be needed to nurture the index, include questions that may be used for validation and assess the need for weighing items and domains based on the stage of demographic transition, while improving the usability of the DDEI data by local and global health and development stakeholders.

Conclusion

This study developed the DDEI for Nigeria, Ethiopia, Kenya, Tanzania, Rwanda, and Senegal and demonstrated the utility of this index. The DDEI yielded several recommendations on opportunities to accelerate socio-economic development and take advantage of the DD potential in these countries. The scores for each sector and domain provided variations that allowed for comparison within a sector in each country. By engaging with local experts and partnering with local institutions in a participatory process, the data from the DDEI were used by local policymakers, service providers, program implementers, advocates, and researchers for self-evaluation and to develop recommendations to strengthen ongoing efforts and programs in respective sectors.

The DDEI allowed for multisectoral analysis and provided an opportunity to identify sectors in most need and those that can serve as models in each country. For instance, based on sector-specific scores, LM and WE stood out as the sectors that required particular attention. However, despite higher sector-specific scores, a closer look at item-specific scores across domains revealed wide variations that were the basis of generating policy recommendations. The DDEI can be applied to all SSA countries regularly to monitor progress and foster local conversation around cultivating sustainable socio-economic development, which ultimately may contribute to realizing the benefits of a DD.

The DDEI can serve as a complementary tool to existing global development monitoring tools. The findings from the DDEI aligned with other existing measures such as the GGG and HCI in various countries. Given the quantitative nature of these tools, the DDEI provides an opportunity to assess the inputs and guide policies, investments and programs in specific sectors and items that can positively affect the gender gap and human capital. In addition to driving conversation amongst stakeholders in FP, MCH, ED, WE, GEI and LM, the DDEI focuses on assessing the inputs that are critical for the outcomes measured by GGG and HCI. The current DDEI has been translated into English and French. In the future, it should be translated into other languages such as Portuguese and Spanish to make it applicable to other countries in the early stages of their demographic transition.

Ethical considerations

The study was conducted in compliance with the Helsinki Declaration and was approved by the Johns Hopkins Bloomberg School of Public Health ethical review board (IRB Number: IRB00011522). All participants provided informed consent before taking part in this research.

Contributorship statement
Jean Christophe Rusatira, Carolina Cardona, Michelle Martinez-Baack, and Jose G. Rimon conceived the study. Jean Christophe Rusatira, Carolina Cardona, Michelle Martinez-Baack, Saifuddin Ahmed, and Jose G. Rimon developed the questionnaire, led data collection, data analysis, and interpretation. Jean Christophe prepared the first draft of the manuscript. All authors revised and approved the final and revised versions of the paper and agree to be accountable for all aspects of the work.

Acknowledgments
We express our deepest thanks to all participants for their contributions to this study. We would also like to thank our local partners and staff who facilitated this work at the Centre d'Excellence Régional en Economie Générationnelle in Senegal, the Center for Communication Programs in Nigeria, the National Council for Population and Development in Kenya, the Tanzania Communication and Development Center, the Ethiopian Academy of Sciences, and the Centre for Integrated Development Research and Action in Rwanda.

Competing interests
The are no competing interests for any author.

Funding
This work was supported by the Bill and Melinda Gates Foundation under the grant OPP1181398.

Data sharing statement
The data and questionnaires used to generate this article are available on Harvard Dataverse open-access repository accessible at https://doi.org/10.7910/DVN/ZSNPMV.

view only

References

[1] A. L. González and M. J. González-González, "Third demographic transition and demographic dividend: An application based on panel data analysis," *Bulletin of Geography. Socio-economic Series,* no. 42, pp. 59-82, 2018.

[2] D. Bloom, D. Canning, and J. Sevilla, *The demographic dividend: A new perspective on the economic consequences of population change*. Rand Corporation, 2003.

[3] D. S. Reher, "Economic and Social Implications of the Demographic Transition," *Population and Development Review,* vol. 37, pp. 11-33, 2011. [Online]. Available: http://www.jstor.org/stable/41762397.

[4] D. Herzer, H. Strulik, and S. Vollmer, "The long-run determinants of fertility: one century of demographic change 1900–1999," *Journal of Economic Growth,* vol. 17, no. 4, pp. 357-385, 2012/12/01 2012, doi: 10.1007/s10887-012-9085-6.

[5] D. Bloom, S. Humair, L. Rosenberg, J. P. Sevilla, and T. J. Trussell, "A Demographic Dividend for Sub-Saharan Africa: Source, Magnitude, and Realization," *IZA Discussion Paper,* no. 7855, p. 26, 2014. [Online]. Available: SSRN: https://ssrn.com/abstract=2374636.

[6] UNFPA, "Demographic Dividend Atlas for Africa: Tracking the Potential for a Demographic Dividend," United Nations Population Fund, 2017.

[7] D. E. Bloom and J. G. Williamson, "Demographic Transitions and Economic Miracles in Emerging Asia," *The World Bank Economic Review,* vol. 12, no. 3, pp. 419-455, 1998, doi: 10.1093/wber/12.3.419.

[8] D. Bloom, D. Canning, and P. N. Malaney, "Demographic Change and Economic Growth in Asia," Center for International Development at Harvard University, 1999. [Online]. Available: https://EconPapers.repec.org/RePEc:wop:cidhav:15

[9] African Union Commission, "AU Roadmap on Harnessing the Demographic Dividend through Investments in Youth," in "Assembly of the Union Twenty- Sixth Ordinary Session 30-31," African Union Commission, 23 February 2017 2017.

[10] C. Cardona *et al.*, "Generating and capitalizing on the demographic dividend potential in sub-Saharan Africa: a conceptual framework from a systematic literature review," *Gates Open Research,* vol. 4, no. 145, p. 145, 2020.

[11] D. Bloom, D. Canning, and P. N. Malaney, "Demographic change and economic growth in Asia," *CID Working Paper Series,* 1999.

[12] D. E. Bloom and D. Canning, "Global demographic change: Dimensions and economic significance," National Bureau of Economic Research, 2004.

[13] M. S. Fabic *et al.*, "Meeting demand for family planning within a generation: the post-2015 agenda," *The Lancet,* vol. 385, no. 9981, pp. 1928-1931, 2015, doi: 10.1016/S0140-6736(14)61055-2.

[14] D. E. Bloom, D. Canning, L. Hu, Y. Liu, A. Mahal, and W. Yip, "The Contribution of Population Health and Demographic Change to Economic Growth in China and India," *J Comp Econ,* vol. 38, no. 1, pp. 17-33, Mar 1 2010, doi: 10.1016/j.jce.2009.11.002.

[15] Q. Li, A. O. Tsui, L. Liu, and S. Ahmed, "Mortality, fertility, and economic development: An analysis of 201 countries from 1960 to 2015," (in eng), *Gates Open Res,* vol. 2, p. 14, Mar 1 2018, doi: 10.12688/gatesopenres.12804.1.

For peer review only

[16] S. Ahmed, Q. Li, L. Liu, and A. O. Tsui, "Maternal deaths averted by contraceptive use: an analysis of 172 countries," (in eng), *Lancet,* vol. 380, no. 9837, pp. 111-25, Jul 14 2012, doi: 10.1016/s0140-6736(12)60478-4.

[17] D. Conley, G. C. McCord, and J. D. Sachs, "Africa's lagging demographic transition: Evidence from exogenous impacts of malaria ecology and agricultural technology," ed: National Bureau of Economic Research Cambridge, Mass., USA, 2007.

[18] G. S. Becker and G. S. Becker, *A Treatise on the Family*. Harvard university press, 2009.

[19] S. A. Ahmed, M. Cruz, B. Quillin, and P. Schellekens, *Demographic Change and Development: Looking at Challenges and Opportunities through a New Typology* (Policy Research Working Papers). The World Bank, 2016, p. 42.

[20] S. A. Ahmed, M. Cruz, D. S. Go, M. Maliszewska, and I. Osorio-Rodarte, *How Significant is Africa's Demographic Dividend for its Future Growth and Poverty Reduction?* (Policy Research Working Papers). The World Bank, 2014, p. 39.

[21] M. Shekar, A. Yazbeck, R. Hasan, and A. Bakilana, "Population and Development in the Sahel: Policy Choices to Catalyze a Demographic Dividend," *Health, Nutrition and Population (HNP) Discussion Paper, World Bank,* 2016, doi: 10.1596/25293.

[22] WBG, *Global Monitoring Report 2015/2016: Development Goals in an Era of Demographic Change*. Washington, DC: The World Bank, 2016.

[23] S. A. Ahmed and M. Cruz, *Making the Most of Demographic Change in Southern Africa* (Policy Research Working Papers). The World Bank, 2016, p. 30.

[24] J. A. Ross and J. Stover, *Effort Indices for national family planning programs, 1999 cycle*. MEASURE Evaluation, Carolina Population Center, University of North Carolina, 2000.

[25] J. A. Ross and W. P. Mauldin, "Family planning programs: efforts and results, 1972-94," *Studies in family planning,* pp. 137-147, 1996.

[26] W. P. Mauldin, B. Berelson, and Z. Sykes, "Conditions of fertility decline in developing countries, 1965-75," *Studies in Family Planning,* vol. 9, no. 5, pp. 89-147, 1978.

[27] J. Stover, "The AIDS Program Effort Index (API): Results from the Field Test," *Washington, DC: Futures Group,* 1999.

[28] J. A. Ross, O. Campbell, and R. Bulatao, "The maternal and neonatal programme effort index (MNPI)," *Tropical medicine & international health,* vol. 6, no. 10, pp. 787-798, 2001.

[29] W. Bank, "Global monitoring report 2015/2016: Development goals in an era of demographic change," ed: The World Bank, 2015.

[30] J. Ross and J. Stover, "The family planning program effort index: 1999 cycle," *International Family Planning Perspectives,* pp. 119-129, 2001.

[31] A. L. Fort, R. Deussom, R. Burlew, K. Gilroy, and D. Nelson, "The Human Resources for Health Effort Index: A tool to assess and inform strategic health workforce investments," *Human resources for health,* vol. 15, no. 1, pp. 1-11, 2017.

[32] F. Usengumukiza, J. P. Munyandamutsa, and E. Nshutiraguma, "Strengthening democratic governance and accountability in Rwanda: The role of the Rwanda Governance Board (RGB)," in *Democratizing Public Governance in Developing Nations*: Routledge, 2017, pp. 109-133.

[33] A. B. Costello and J. Osborne, "Best practices in exploratory factor analysis: Four recommendations for getting the most from your analysis," *Practical assessment, research, and evaluation,* vol. 10, no. 1, p. 7, 2005.

For peer review only - http://bmjopen.bmj.com/site/about/guidelines.xhtml

[34] R. DeVellis, "Chapter 6: Factor Analysis," *Scale development: Theory and applications,* vol. 26, p. 95, 2012.

[Dataset] [35] J. C. Rusatira, C. Cardona, M. Martinez-Baack, J. G. Rimon, and S. Ahmed. *Demographic Dividend Effort Index (DDEI)*, Harvard Dataverse, doi: doi:10.7910/DVN/ZSNPMV.

[36] C. Poole and S. Greenland, "Random-effects meta-analyses are not always conservative," *American Journal of Epidemiology,* vol. 150, no. 5, pp. 469-475, 1999.

[37] J. P. Higgins, S. G. Thompson, J. J. Deeks, and D. G. Altman, "Measuring inconsistency in meta-analyses," *Bmj,* vol. 327, no. 7414, pp. 557-560, 2003.

[38] W. E. Forum, "Global Gender Gap Report 2020: Insight Report," pp. 1-371, 2019.

[39] K. Schwab, "The Global Competitiveness Report 2019. Geneva. World Economic Forum," ed, 2019.

[40] W. E. Forum, "Global Gender Gap Report 2022," 2022. [Online]. Available: https://www.weforum.org/reports/global-gender-gap-report-2022/

[41] W. B. Group, "The Human Capital Index 2020 Update," in *The Human Capital Index 2020 Update*, 2020, doi: 10.1596/978-1-4648-1552-2.

[42] J. Fletcher, "What is heterogeneity and is it important?," *Bmj,* vol. 334, no. 7584, pp. 94-96, 2007.

[43] R. J. Lapham and W. P. Mauldin, "Contraceptive prevalence: the influence of organized family planning programs," *Studies in Family Planning,* vol. 16, no. 3, pp. 117-137, 1985.

[44] J. A. Ross, O. M. Campbell, and R. Bulatao, "The Maternal and Neonatal Programme Effort Index (MNPI)," (in eng), *Trop Med Int Health,* vol. 6, no. 10, pp. 787-98, Oct 2001, doi: 10.1046/j.1365-3156.2001.00787.x.

[45] D. E. Bloom, D. Canning, G. Fink, and J. Finlay, *Realizing the demographic dividend: Is Africa any different?* 2007.

[46] D. E. Bloom, "7 billion and counting," *Science,* vol. 333, no. 6042, pp. 562-569, 2011.

For peer review only

Figure 1: Average scores per country and sector

For peer review only

For peer review only - http://bmjopen.bmj.com/site/about/guidelines.xhtml

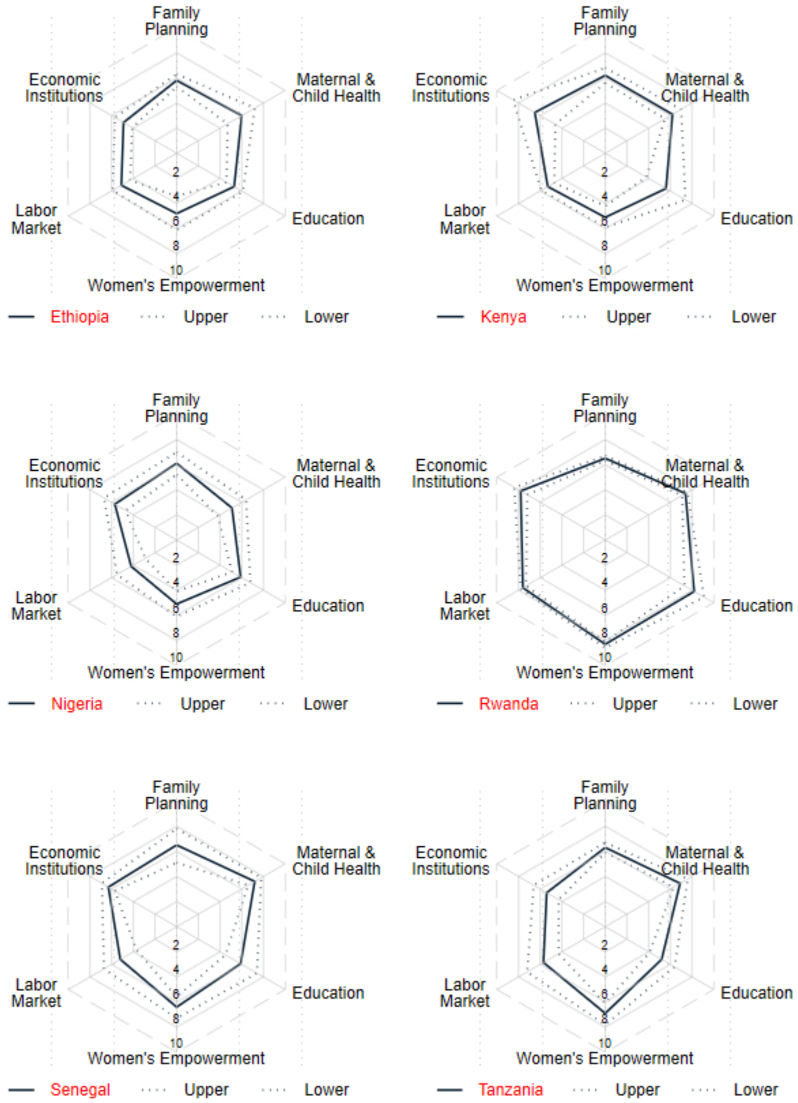

Average scores per country and sector

232x310mm (72 x 72 DPI)

JOHNS HOPKINS
BLOOMBERG SCHOOL
of PUBLIC HEALTH

Bill & Melinda Gates Institute for Population and Reproductive Health

Demographic Dividend Effort Index Survey

Introduction

There is a programmatic need for a standard measure that quantifies the extent of national efforts in policies and programs implemented to cultivate, realize and harness the benefits of a demographic dividend (DD). Despite the remarkable progress achieved by various countries through the integration of demographic dividend principles into their policies and development plans, performance assessment tools and platforms to document best practices and lessons learned to harness the DD are still missing.

Thus, the purpose of the DD Effort Index (DDEI) is to provide a standard measure that will quantify the nature and strength of in-country DD efforts by experts and civil society members across the public and private spheres that represent different dimensions of the society. By measuring efforts, we are capturing inputs independent of outputs, such as income per capita. This will allow us to assess the relationship between inputs that go into the development of DD policies and DD outputs.

This DDEI is guided by one central question: To what extent have the existing national policy and program efforts enabled a favorable policy environment to cultivate, realize and harness the benefits of a demographic dividend? To answer this question, the DDEI will summarize key informant perceptions across six sectors identified in the literature to produce a favorable environment to harness a DD. These DD framework sectors are: 1) Family Planning, 2) Maternal and Child Health, 3) Education, 4) Women's Empowerment, 5) Labor Market, and 6) Good Governance, Strong Institutions and Stable Economy. Each sector is measured through its own questionnaire and will be assessed in five dimensions identified in the literature; namely, policy, services or programs, advocacy, research, and civil society. Each of the 6 questionnaires, completed by 10 expert observers per country, score items on a scale from 1 to 10, unless otherwise noted.

Considering the health and socio-economic impact of COVID-19, and its close relationship with policies that enable a DD favorable policy environment, this DD Effort Index is integrating a module to assess the resilience and sustainability of systems in each sector. The scoring follows the key principles of sustainable and resilient health systems that can effectively respond to emerging infectious disease threats and other public health emergencies.

The DDEI provides an opportunity to visualize the national efforts while assessing the extent to which these efforts support a country's goals to cultivate, realize and harness the demographic dividend. The DDEI will be a tool for performance measurement and progress monitoring, which will provide a timely assessment of successes and gaps and reveal best practices and areas of improvement.

JOHNS HOPKINS
BLOOMBERG SCHOOL of PUBLIC HEALTH

Bill & Melinda Gates Institute for Population and Reproductive Health

Demographic Dividend Effort Index Survey

Consent form

Thank you for taking the time to complete this questionnaire, especially in this unprecedented time.

The Gates Institute is developing a Demographic Dividend Effort Index (DDEI) to establish a standard measure that will allow the assessment of the existing status of national policies, services, and programs in family planning, maternal and child health, other social sectors (e.g., employment and education), and governance to enable a favorable policy environment to cultivate a demographic dividend (DD). By measuring efforts, this tool will capture inputs independent of outputs, such as income per capita. This will allow the assessment of the relationship between inputs that go into the development of DD policies and DD outputs that are observed and commonly used to assess progress.

We would like to ask you some questions because of your expertise in the area. The information collected will be kept confidential, and we will not be collecting your names or any other identification details. Completing this questionnaire will take around 45-60 min of your time.

We will appreciate your participation in this survey. Information from this interview will be used by the Gates Institute and the community of DD practice in your country to assess the national efforts and identify areas to continue supporting the successes and those that need to be strengthened. Researchers may also use the data collected for cross-country analyses.

You are invited to participate in this survey. Please know that this survey is completely voluntary. By participating, there is no direct benefit to you but there is a societal benefit in enabling the government and development partners to strengthen their efforts to cultivate a DD-favorable environment.

You may refuse to answer any question. Should you have any concerns or issues about the study, you may contact the study coordinator at **jcrusatira@jhu.edu** or **+14436510824** by a phone call or WhatsApp.

* Please check “Yes” if you would like to proceed with the survey.

◯ Yes

◯ No

JOHNS HOPKINS
BLOOMBERG SCHOOL of PUBLIC HEALTH

Bill & Melinda Gates Institute for Population and Reproductive Health

Demographic Dividend Effort Index Survey

Important notice

Please do not select 'Back' while in the middle of answering a page of questions as the page you are working on will not be saved until you finalize the said page and select 'Next'. If you select 'Back' without finalizing, the progress you have made on the unfinished page will be lost.

Please be aware that your survey is completed when you read "Thank you for completing the demographic dividend effort index. Stay well and safe!"

JOHNS HOPKINS
BLOOMBERG SCHOOL
of PUBLIC HEALTH

Bill & Melinda Gates Institute for Population and Reproductive Health

Demographic Dividend Effort Index Survey

Respondent country and expertise

* Please select the country where you are responding from.

- Kenya
- Senegal
- Nigeria
- Rwanda
- Tanzania
- Ethiopia

JOHNS HOPKINS
BLOOMBERG SCHOOL
of PUBLIC HEALTH

Bill & Melinda Gates Institute for Population and Reproductive Health

Demographic Dividend Effort Index Survey

Level of expertise

* At which level of Government is your expertise (If your expertise is at the national level please select "National" otherwise select a county?

JOHNS HOPKINS
BLOOMBERG SCHOOL
of PUBLIC HEALTH

Bill & Melinda Gates Institute for Population and Reproductive Health

Demographic Dividend Effort Index Survey

Expertise

* Please select the organization you have worked in the most during the last five years. If your organization is not listed, please write it down.

- Public sector
- Private sector
- Non-governmental organization
- University/research institute
- Other (please specify)

* Which dimension have you worked in the most during the last five years?

- Policy/policymaking
- Services or programs
- Advocacy
- Research
- Civil Society

* In which sector have you worked the most during the last five years?

- Family Planning
- Maternal and Child Health
- Education
- Women's Empowerment
- Labor Market
- Governance and Economic Institutions

JOHNS HOPKINS
BLOOMBERG SCHOOL
of PUBLIC HEALTH

Bill & Melinda Gates Institute for Population and Reproductive Health

Demographic Dividend Effort Index Survey

Family Planning Questionnaire

Please respond to the following questions based on your experience/expertise within this specific sector.

To give a summary picture of the national demographic dividend effort, please rate the following items. Score each item from 1 to 10, where 1 represents the lowest score (very weak or no effort) and 10 represents the highest score (strong effort). Where applicable, if a policy or activity is non-existent then mark your response as 0. Please note that a few questions have a different scoring scale.

Please answer each item. All responses will be recorded in the format exemplified below:

Lowest effort	1	2	3	4	5	6	7	8	9	10	Highest effort	I do not know

Bill & Melinda Gates Institute for Population and Reproductive Health

Demographic Dividend Effort Index Survey

Dimension 1. Policy/Policy making

* FP 1.1 Prominence of Family Planning (FP): Extent to which there is a permanent office or post within the appropriate ministry that develops and monitors sectoral policies and strategies, that is well placed within the government, and staffed by adequately skilled personnel.

	1	2	3	4	5	6	7	8	9	10	Don't know
1-Lowest -> Highest-10	◯	◯	◯	◯	◯	◯	◯	◯	◯	◯	◯

* FP 1.2 Policy on Fertility Reduction and FP: Extent to which government policy emphasizes FP for family size as it relates to available resources or demographic reasons over health reasons.

	1	2	3	4	5	6	7	8	9	10	Don't know
1-Lowest -> Highest-10	◯	◯	◯	◯	◯	◯	◯	◯	◯	◯	◯

* FP 1.3 National FP Strategic Plan: Extent to which there is a current, comprehensive, strategic national plan for FP that outlines policies, laws, and regulations for the provisioning of FP. (In making this assessment, consider whether the plan defines a vision; includes multiyear strategies, annual timelines, and implementation plans; is evidence-based; has indicators to measure progress; and considers the FP market.)

	1	2	3	4	5	6	7	8	9	10	Don't know
1-Lowest -> Highest-10	◯	◯	◯	◯	◯	◯	◯	◯	◯	◯	◯

* FP 1.4 Policy on Age at Marriage: Extent to which legal age at marriage for females is set at 18 years or higher and is enforced.

	1	2	3	4	5	6	7	8	9	10	Don't know
1-Lowest -> Highest-10	◯	◯	◯	◯	◯	◯	◯	◯	◯	◯	◯

* FP 1.5 Evidence-Based Policymaking: Extent to which the appropriate ministry actively and regularly supports and uses key FP data and reports to engage in policy development and planning.

	1	2	3	4	5	6	7	8	9	10	Don't know
1-Lowest -> Highest-10	○	○	○	○	○	○	○	○	○	○	○

* FP 1.6 Monitoring and Evaluation of National Policies/Plans/Strategies: Extent to which the national FP plan outlines a comprehensive monitoring and evaluation strategy to track and measure accomplishments in the plan.

(In making this assessment, consider whether the monitoring and evaluation strategy includes goals and objectives, a results framework, measurable indicators [e.g., "SMART": specific, measurable, attainable, relevant and time-bound]; and an actionable monitoring and evaluation implementation plan/timeline.)

	1	2	3	4	5	6	7	8	9	10	Don't know
1-Lowest -> Highest-10	○	○	○	○	○	○	○	○	○	○	○

* FP 1.7 Inclusion of Nongovernmental and Cross-sectoral Actors in Policy/Strategy Development: Extent to which nongovernmental agencies, groups (e.g., from civil society), health workers, stakeholders (e.g., from faith-based, nonprofit, and private-for-profit organizations) and actors across non-FP-specific sectors are included in the national FP plan and/or other national documents outlining the country's strategies and systems related to planning, managing, and developing FP.

	1	2	3	4	5	6	7	8	9	10	Don't know
1-Lowest -> Highest-10	○	○	○	○	○	○	○	○	○	○	○

* FP 1.8 Financing for FP: Extent to which the government has established and applied guidelines for the costing and payments for FP across supply and demand considerations (i.e. domestic versus international funding – and its expectations for continuity/sustainability, cost of contraceptive supplies, cost of service delivery and transportation, etc.).

(In making this assessment, consider whether the guidelines and budget address the following: the lifespan of costs for FP care and services; variable structures of costs per FP method; costs of delivery and service provision by specialization, location and risk; costs and profits of healthcare/insurance schemes, the financial costs of providing FP weighed against the financing costs of not doing so.)

	1	2	3	4	5	6	7	8	9	10	Don't know
1-Lowest -> Highest-10	○	○	○	○	○	○	○	○	○	○	○

* FP 1.9 Percent of In-country Funding of FP Budget: Extent to which the total family planning/population budget is derived from in-country sources (e.g., 1 for 10 percent, 5 for 50 percent, 10 for 100 percent).

	1	2	3	4	5	6	7	8	9	10	Don't know
1-Lowest -> Highest-10	○	○	○	○	○	○	○	○	○	○	○

* FP 1.10 Information/Communications Technology Infrastructure/Capacity: Extent to which there are adequate infrastructure components (e.g., computers, servers, printers, a local area network, Internet connectivity, and reliable electric power) as well as human resources (e.g., for maintenance and support) at the national and subnational levels to support the functioning of FP-related information systems.
(In making this assessment, consider whether the country conducts at least annual assessments of its information systems; has training curricula that cover data entry, use, software administration, and development; and continuously trains staff from key stakeholder institutions [e.g., Ministry of Health, Ministry of Education, regulatory agencies, training institutions].)

	1	2	3	4	5	6	7	8	9	10	Don't know
1-Lowest -> Highest-10	○	○	○	○	○	○	○	○	○	○	○

* FP 1.11 Import Laws and Legal Regulations:

	1	2	3	4	5	6	7	8	9	10	Don't know
FP 1.11.1 Extent to which import laws and legal regulations facilitate the importation of FP commodities and supplies	○	○	○	○	○	○	○	○	○	○	○
FP 1.11.2 Extent to which contraceptives are manufactured locally.	○	○	○	○	○	○	○	○	○	○	○

* FP 1.12 Advertising of Contraceptives Allowed: Extent of freedom from restrictions on advertising of contraceptives in the mass media.

	1	2	3	4	5	6	7	8	9	10	Don't know
1-Lowest -> Highest-10	○	○	○	○	○	○	○	○	○	○	○

* FP 1.13 Presence of Pro-Poor FP Policies: Extent to which policies specifically target the poor in rural or urban places.

	1	2	3	4	5	6	7	8	9	10	Don't know
1-Lowest -> Highest-10	○	○	○	○	○	○	○	○	○	○	○

* FP 1.14 Political Support for FP: Extent to which elected officials in the country prioritize meeting FP needs to strengthen the FP contributions to the DD by passing laws and regulations and sponsoring actions and policies aimed at improving FP uptake and adherence.

	1	2	3	4	5	6	7	8	9	10	Don't know
1-Lowest -> Highest-10	○	○	○	○	○	○	○	○	○	○	○

FP 1.15 Comments: Please use this section to provide any additional comments about the level of effort, challenges, or successes in the country around policies/policymaking for FP. If you marked “I don’t know” for any of the questions, also use this section to explain why you gave that rating

JOHNS HOPKINS
BLOOMBERG SCHOOL
of PUBLIC HEALTH

Bill & Melinda Gates Institute for Population and Reproductive Health

Demographic Dividend Effort Index Survey

Dimension 2. Services or Programs

* FP 2.1 Administrative Structure: Extent to which the administrative structure and staff at national, provincial and county levels are sufficient to implement the family planning program.

	1	2	3	4	5	6	7	8	9	10	Don't know
1-Lowest -> Highest-10	○	○	○	○	○	○	○	○	○	○	○

* FP 2.2 Level of Program Leaders: Relative level of seniority of the director of the national family planning program and whether said director reports to a high level of government.

	1	2	3	4	5	6	7	8	9	10	Don't know
1-Lowest -> Highest-10	○	○	○	○	○	○	○	○	○	○	○

* FP. 2.3 Community-Based Distribution: Extent to which areas of the country not easily serviced by clinics or other service points are covered by community-based distribution programs for distribution of contraceptive supplies (especially rural areas).

	1	2	3	4	5	6	7	8	9	10	Don't know
1-Lowest -> Highest-10	○	○	○	○	○	○	○	○	○	○	○

* FP 2.4 Postpartum Program: Extent to which all postpartum women receive postpartum family planning services.

	1	2	3	4	5	6	7	8	9	10	Don't know
1-Lowest -> Highest-10	○	○	○	○	○	○	○	○	○	○	○

* FP 2.5 Home-Visiting Workers: Extent of coverage of population by workers whose primary task is to visit (rural) women in their homes to talk about family planning and maternal and child health.

	1	2	3	4	5	6	7	8	9	10	Don't know
1-Lowest -> Highest-10	◯	◯	◯	◯	◯	◯	◯	◯	◯	◯	◯

* FP 2.6 Availability and Accessibility of FP Commodities/Supplies and Services Part 1: Extent to which entire population has ready and easy access to (1-Lowest -> Highest-10):

	1	2	3	4	5	6	7	8	9	10	Don't know
FP 2.6.1 IUD	◯	◯	◯	◯	◯	◯	◯	◯	◯	◯	◯
FP 2.6.2 Pills	◯	◯	◯	◯	◯	◯	◯	◯	◯	◯	◯
FP 2.6.3 Injectables	◯	◯	◯	◯	◯	◯	◯	◯	◯	◯	◯
FP 2.6.4 Implants	◯	◯	◯	◯	◯	◯	◯	◯	◯	◯	◯
FP 2.6.5 Female Sterilization	◯	◯	◯	◯	◯	◯	◯	◯	◯	◯	◯
FP 2.6.6 Male Sterilization	◯	◯	◯	◯	◯	◯	◯	◯	◯	◯	◯
FP 2.6.7 Male Condoms	◯	◯	◯	◯	◯	◯	◯	◯	◯	◯	◯
FP 2.6.8 Emergency Contraception	◯	◯	◯	◯	◯	◯	◯	◯	◯	◯	◯
FP 2.6.9 Abortion/menstrual regulation (whether legal or not)	◯	◯	◯	◯	◯	◯	◯	◯	◯	◯	◯
FP 2.6.10 Others (e.g. female condoms, foam, beads, etc.)	◯	◯	◯	◯	◯	◯	◯	◯	◯	◯	◯

* FP 2.7 Availability and Accessibility of FP Commodities/Supplies and Services Part 2: How well does the supply system operate (it avoids stockouts or interrupted supplies and guarantees a reliable flow at local levels) for the following (1-Lowest -> Highest-10):

	1	2	3	4	5	6	7	8	9	10	Don't know
FP 2.7.1 IUD	○	○	○	○	○	○	○	○	○	○	○
FP 2.7.2 Pills	○	○	○	○	○	○	○	○	○	○	○
FP 2.7.3 Injectables	○	○	○	○	○	○	○	○	○	○	○
FP 2.7.4 Implants	○	○	○	○	○	○	○	○	○	○	○
FP 2.7.5 Male Condoms	○	○	○	○	○	○	○	○	○	○	○
FP 2.7.6 Emergency Contraception	○	○	○	○	○	○	○	○	○	○	○
FP 2.7.7 Others (e.g. female condoms, foam, beads, etc.)	○	○	○	○	○	○	○	○	○	○	○

* FP 2.8 Availability and Accessibility of FP Commodities/Supplies and Services Part 3: How well does the supply system provide necessary equipment and medical supplies at clinical facilities for the following (1-Lowest -> Highest-10):

	1	2	3	4	5	6	7	8	9	10	Don't know
FP 2.8.1 Female Sterilization	○	○	○	○	○	○	○	○	○	○	○
FP 2.8.2 Male Sterilization	○	○	○	○	○	○	○	○	○	○	○
FP 2.8.3. IUDs	○	○	○	○	○	○	○	○	○	○	○
FP 2.8.4 Implants	○	○	○	○	○	○	○	○	○	○	○

* FP 2.9 FP Service Quality: Please rate the general quality of family planning services. (Good quality includes a focus on client needs, with counseling, full information, wide method choice, and safe clinical procedures.)

	1	2	3	4	5	6	7	8	9	10	Don't know
1-Lowest -> Highest-10	○	○	○	○	○	○	○	○	○	○	○

* FP 2.10 Incentives and Disincentives: Extent to which monetary or other incentives are used to encourage the adoption of family planning.

	1	2	3	4	5	6	7	8	9	10	Don't know
1-Lowest -> Highest-10	○	○	○	○	○	○	○	○	○	○	○

* FP 2.11 Adolescent Programs: Extent to which information, services and activities are focused towards adolescents and encourage adolescents to access contraceptive services.

	1	2	3	4	5	6	7	8	9	10	Don't know
1-Lowest -> Highest-10	○	○	○	○	○	○	○	○	○	○	○

* FP 2.12 Training Program: Extent to which training programs, for each category of staff in the family planning program, are adequate to provide personnel with information and skills necessary to carry out their jobs effectively.

	1	2	3	4	5	6	7	8	9	10	Don't know
1-Lowest -> Highest-10	○	○	○	○	○	○	○	○	○	○	○

* FP 2.13 Personnel Carry Out Assigned Tasks: Extent to which all categories of family planning program staff (administrative, medical, paramedical, field) carry out assigned tasks effectively

	1	2	3	4	5	6	7	8	9	10	Don't know
1-Lowest -> Highest-10	○	○	○	○	○	○	○	○	○	○	○

* FP 2.14 Logistics and Transport: Extent to which the logistics and transport systems are sufficient to keep stocks of contraceptive supplies and related equipment available at all service points, at all times and at all levels.

	1	2	3	4	5	6	7	8	9	10	Don't know
1-Lowest -> Highest-10	○	○	○	○	○	○	○	○	○	○	○

* FP 2.15 Supervision System: Extent to which the system of supervision at all levels is adequate (regular monitoring visits with corrective or supportive action).

	1	2	3	4	5	6	7	8	9	10	Don't know
1-Lowest -> Highest-10	○	○	○	○	○	○	○	○	○	○	○

* FP 2.16 Involvement of Other Ministries and Public Agencies: Extent to which other ministries, government agencies and non-governmental stakeholders engage with FP activities (e.g., delivery of supplies, services, information, education) or other population activities.

	1	2	3	4	5	6	7	8	9	10	Don't know
1-Lowest -> Highest-10	○	○	○	○	○	○	○	○	○	○	○

* FP 2.17 Involvement of Private-Sector Agencies and Groups: Extent to which private-sector agencies and groups assist with FP or other population activities.

	1	2	3	4	5	6	7	8	9	10	Don't know
1-Lowest -> Highest-10	○	○	○	○	○	○	○	○	○	○	○

* FP 2.18 Mass Media for Information Education Communication: Frequency and extent of coverage of mass media messages that provide the population with information on family planning and service sites.

	1	2	3	4	5	6	7	8	9	10	Don't know
1-Lowest -> Highest-10	○	○	○	○	○	○	○	○	○	○	○

* FP 2.19 Cultural and Social Norms Barriers: Extent to which cultural and social barriers are addressed in family planning programs.

	1	2	3	4	5	6	7	8	9	10	Don't know
1-Lowest -> Highest-10	○	○	○	○	○	○	○	○	○	○	○

* FP 2.20 Youth: Extent to which the government supports youth-led FP initiatives or involve youth in planning, implementation and evaluation of FP programs.

	1	2	3	4	5	6	7	8	9	10	Don't know
1-Lowest -> Highest-10	○	○	○	○	○	○	○	○	○	○	○

* FP 2.21 FP Effort Influences – Forces Affecting the FP Program: Forces affecting the FP program can either strengthen or detract from its effectiveness. Check zero (0) if there is no difference either way; check a negative number from -1 to -5 if the influence is negative; or check a positive number from 1 to 5 if the influence is positive. (1 in either direction means little influence and 5 in either direction means strong influence.)

	-5	-4	-3	-2	-1	0	1	2	3	4	5	Don't know
FP 2.21.1 Decentralization	○	○	○	○	○	○	○	○	○	○	○	○
FP 2.21.2 HIV/AIDS Programs	○	○	○	○	○	○	○	○	○	○	○	○
FP 2.21.3 Incorporation of FP into a broader context of reproductive health	○	○	○	○	○	○	○	○	○	○	○	○
FP 2.21.4 Integration of FP with other health services	○	○	○	○	○	○	○	○	○	○	○	○
FP 2.21.5 Changes in donor funding	○	○	○	○	○	○	○	○	○	○	○	○
FP 2.21.6 Changes in domestic government funding	○	○	○	○	○	○	○	○	○	○	○	○

FP 2.22 Comments: Please use this section to provide any additional comments about the level of effort, challenges, or successes in the country around services or programs for FP. If you marked “I don’t know” for any of the questions, also use this section to explain why you gave that rating

JOHNS HOPKINS
BLOOMBERG SCHOOL
of PUBLIC HEALTH

Bill & Melinda Gates Institute for Population and Reproductive Health

Demographic Dividend Effort Index Survey

Dimension 3. Advocacy

* FP 3.1 Influence of FP Leaders/Champions: Extent to which the country has one or more clearly influential leaders or champions who successfully advocate for FP (sectoral) needs at high levels, promote FP in the country by making positive public statements about it, and/or support actions and policies aimed at improving the FP environment and its contributions towards the DD.

	1	2	3	4	5	6	7	8	9	10	Don't know
1-Lowest -> Highest-10	○	○	○	○	○	○	○	○	○	○	○

* FP 3.2 Statement by Leaders: Extent to which the head of government, as well as other officials, speak publicly and favorably about family planning at least once or twice a year.

	1	2	3	4	5	6	7	8	9	10	Don't know
1-Lowest -> Highest-10	○	○	○	○	○	○	○	○	○	○	○

* FP 3.3 Media Coverage for FP: Extent to and frequency with which national media cover/broadcast FP topics that provides the population with information on family planning and service sites.

	1	2	3	4	5	6	7	8	9	10	Don't know
1-Lowest -> Highest-10	○	○	○	○	○	○	○	○	○	○	○

* FP 3.4 FP Effort Influences - Justification: How important is each of the following as a current government justification for the national FP program? (1 means negligible importance; 10 means great importance.)

	1	2	3	4	5	6	7	8	9	10	Don't know
FP 3.4.1 Reduce the rate of population growth	○	○	○	○	○	○	○	○	○	○	○
FP 3.4.2 Enhance economic development	○	○	○	○	○	○	○	○	○	○	○
FP 3.4.3 Help women and men avoid unplanned and unwanted births	○	○	○	○	○	○	○	○	○	○	○
FP 3.4.4 Improve women's health	○	○	○	○	○	○	○	○	○	○	○
FP 3.4.5 Improve child health	○	○	○	○	○	○	○	○	○	○	○
FP 3.4.6 Reduce unmarried adolescent childbearing	○	○	○	○	○	○	○	○	○	○	○
FP 3.4.7 Reduce unmet need for contraceptive services	○	○	○	○	○	○	○	○	○	○	○

* FP 3.5 FP Effort Influences – Special Populations: To what extent does the family planning program give particular emphasis to special populations? (1 means negligible emphasis; 10 means great emphasis)

	1	2	3	4	5	6	7	8	9	10	Don't know
FP 3.5.1 Unmarried youth	○	○	○	○	○	○	○	○	○	○	○
FP 3.5.2 The poor	○	○	○	○	○	○	○	○	○	○	○
FP 3.5.3 The disabled	○	○	○	○	○	○	○	○	○	○	○
FP 3.5.4 Rural population	○	○	○	○	○	○	○	○	○	○	○
FP 3.5.5 Other vulnerable groups (e.g. minority and/or indigenous groups)	○	○	○	○	○	○	○	○	○	○	○
FP 3.5.6 Postpartum women for counseling and contraceptive services	○	○	○	○	○	○	○	○	○	○	○
FP 3.5.7 Post-abortion women for counseling and contraceptive services	○	○	○	○	○	○	○	○	○	○	○

* FP 3.6 Social Marketing: Extent of coverage of the country by a social marketing program (subsidized contraceptive sales at low cost in commercial sector, especially in urban areas).

	1	2	3	4	5	6	7	8	9	10	Don't know
1-Lowest -> Highest-10	○	○	○	○	○	○	○	○	○	○	○

FP 3.7 Comments: Please use this section to provide any additional comments about the level of effort, challenges, or successes in the country around advocacy for FP. If you marked “I don’t know” for any of the questions, also use this section to explain why you gave that rating.

JOHNS HOPKINS
BLOOMBERG SCHOOL
of PUBLIC HEALTH

Bill & Melinda Gates Institute for Population and Reproductive Health

Demographic Dividend Effort Index Survey

Dimension 4. Research

* FP 4.1 Strength of Observatory/Stakeholder/Technical Working Group for FP: Extent to which country-level FP stakeholders (e.g., stakeholder leadership group, technical working group, FP observatory, country coordination and facilitation group):

	1	2	3	4	5	6	7	8	9	10	Don't know
FP 4.1.1 Have representation in government, training institutions, civil society, nongovernmental and faith-based organizations, professional associations, the private sector, etc.	○	○	○	○	○	○	○	○	○	○	○
FP 4.1.2 Meet regularly, report and recommend policy for senior management within the appropriate ministries	○	○	○	○	○	○	○	○	○	○	○
FP 4.1.3 Make an impact on FP within the country	○	○	○	○	○	○	○	○	○	○	○

* FP 4.2 Education/Research Strategy: Extent to which the national FP plan and/or other national documents include a comprehensive strategy/approach on FP-related research.

	1	2	3	4	5	6	7	8	9	10	Don't know
1-Lowest -> Highest-10	○	○	○	○	○	○	○	○	○	○	○

* FP 4.3 Data Gathering Partners:

	1	2	3	4	5	6	7	8	9	10	Don't know
FP 4.3.1 Extent to which research is undertaken by government agencies. (This may include statistical agencies/bureaus and/or ministries of economics/finances, education, health, gender, etc.)	○	○	○	○	○	○	○	○	○	○	○
FP 4.3.2 Extent to which research is undertaken by research institutions.	○	○	○	○	○	○	○	○	○	○	○
FP 4.3.3 Extent to which research is undertaken by independent researchers.	○	○	○	○	○	○	○	○	○	○	○

* FP 4.4 Topical Data Gathering:

	1	2	3	4	5	6	7	8	9	10	Don't know
FP 4.4.1 Extent to which family planning uptake (including method mix), demand, and unmet need is researched.	○	○	○	○	○	○	○	○	○	○	○
FP 4.4.2 Extent to which barriers to FP uptake is researched.	○	○	○	○	○	○	○	○	○	○	○
FP 4.4.3 Extent to which the impacts of FP programs are researched.	○	○	○	○	○	○	○	○	○	○	○
FP 4.4.4 Extent to which the quality of service provision for FP is researched.	○	○	○	○	○	○	○	○	○	○	○

* FP 4.5 Quality/Coverage of Data: Extent to which current research/data is sex and age disaggregated. Extent to which the country participates in standardized tests and its ranking.

	1	2	3	4	5	6	7	8	9	10	Don't know
FP 4.5.1 Extent to which a routine statistical system provides reliable periodic information on FP supplies and facilities.	○	○	○	○	○	○	○	○	○	○	○
FP 4.5.2 Extent to which a routine statistical system provides good periodic information on FP services and personnel.	○	○	○	○	○	○	○	○	○	○	○
FP 4.5.3 Extent to which the routine statistical system provides good periodic information on FP needs among populations in different communities.	○	○	○	○	○	○	○	○	○	○	○
FP 4.5.4 Extent to which evaluations of the health monitoring and information system are conducted and applied to ensure the reliability of data.	○	○	○	○	○	○	○	○	○	○	○

* FP 4.6 Record-Keeping: Extent to which systems for client recordkeeping, clinic reporting and feedback of results are adequate, including being connected at all levels (federal, state/province, county, etc.).

	1	2	3	4	5	6	7	8	9	10	Don't know
1-Lowest -> Highest-10	○	○	○	○	○	○	○	○	○	○	○

* FP 4.7 Quality Research Institutions: Extent to which the country supports and sustains research institutions that develop research/collect data that support the breadth and quality of information-gathering to inform national FP efforts.

	1	2	3	4	5	6	7	8	9	10	Don't know
1-Lowest -> Highest-10	○	○	○	○	○	○	○	○	○	○	○

* FP 4.8 Evaluation: Extent to which program statistics, surveys, and small studies are used by specialized staff to report on program operations and measure progress.

	1	2	3	4	5	6	7	8	9	10	Don't know
1-Lowest -> Highest-10	○	○	○	○	○	○	○	○	○	○	○

* FP 4.9 Management's Use of Evaluation Findings: Extent to which local-level program managers use research and evaluation findings to improve programming in ways suggested by findings.

	1	2	3	4	5	6	7	8	9	10	Don't know
1-Lowest -> Highest-10	○	○	○	○	○	○	○	○	○	○	○

* FP 4.10 Ministerial Use of Evaluation Findings: Extent to which the Ministry administrators/policymakers systematically utilize data to inform policies and interventions to address FP-related issues.

	1	2	3	4	5	6	7	8	9	10	Don't know
1-Lowest -> Highest-10	○	○	○	○	○	○	○	○	○	○	○

* FP 4.11 Dissemination of Information to Other Implementing Bodies: Extent to which information is shared or spread across geographical areas and throughout various levels (national, state/province/county, localities/sub-counties).

	1	2	3	4	5	6	7	8	9	10	Don't know
1-Lowest -> Highest-10	○	○	○	○	○	○	○	○	○	○	○

FP 4.12 Comments: Please use this section to provide any additional comments about the level of effort, challenges, or successes in the country around FP research. If you marked "I don't know" for any of the questions, also use this section to explain why you gave that rating.

JOHNS HOPKINS
BLOOMBERG SCHOOL
of PUBLIC HEALTH

Bill & Melinda Gates Institute for Population and Reproductive Health

Demographic Dividend Effort Index Survey

Dimension 5. Civil Society Organizations (CSOs)

* FP 5.1 CSO Actor Power: Extent to which CSO actors are in strong placements within institutions or technical working groups or networks concerned with FP.

	1	2	3	4	5	6	7	8	9	10	Don't know
1-Lowest -> Highest-10	○	○	○	○	○	○	○	○	○	○	○

* FP 5.2 Budget Analysis as a CSO Tool: Extent to which CSOs utilize budget analysis as a tool to build FP advocacy cases.

	1	2	3	4	5	6	7	8	9	10	Don't know
1-Lowest -> Highest-10	○	○	○	○	○	○	○	○	○	○	○

* FP 5.3 Facility-Based Service Delivery Support: Extent to which CSOs are involved in efforts to increase the use and/or quality of services, particularly through increasing the technical and/or operational capacity of CSO-partnered clinics to provide FP services.

	1	2	3	4	5	6	7	8	9	10	Don't know
1-Lowest -> Highest-10	○	○	○	○	○	○	○	○	○	○	○

* FP 5.4 Community-Based Service Delivery Support: Extent to which CSOs participate in bringing FP information and services to the communities where people live through activities such as supporting the capacity-building of community-level health workers, expanding the range of contraceptive methods provided by community health workers, educating lower cadre health workers, and/or undertaking information and education campaigns to increase the acceptability of contraception.

	1	2	3	4	5	6	7	8	9	10	Don't know
1-Lowest -> Highest-10	○	○	○	○	○	○	○	○	○	○	○

* FP 5.5 Mobile Outreach for Community-Based Service Delivery Support: Extent to which CSOs support outreach models to equip health care providers with FP commodities, supplies, equipment and vehicles, which are then sent as specialist team to provide FP information and methods directly to communities.

	1	2	3	4	5	6	7	8	9	10	Don't know
1-Lowest -> Highest-10	○	○	○	○	○	○	○	○	○	○	○

* FP 5.6 Social Franchising: Extent to which CSOs are involved in social franchising opportunities/services to bring private sector services to clients at reduced prices.

	1	2	3	4	5	6	7	8	9	10	Don't know
1-Lowest -> Highest-10	○	○	○	○	○	○	○	○	○	○	○

* FP 5.7 mHealth: Extent to which CSOs are leveraging mHealth to utilize text messages and other mobile technologies to improve access to FP information and services.

	1	2	3	4	5	6	7	8	9	10	Don't know
1-Lowest -> Highest-10	○	○	○	○	○	○	○	○	○	○	○

* FP 5.8 Human Rights and Quality of Care: Extent to which CSOs advocate for and support rights-based approaches to FP to ensure FP service recipients experience good treatment, confidence in asking questions, and quality counselling, amongst other concerns.

	1	2	3	4	5	6	7	8	9	10	Don't know
1-Lowest -> Highest-10	○	○	○	○	○	○	○	○	○	○	○

* FP 5.9 Social and Behavior Change (SBC): Extent to which CSOs support SBC interventions relating, but not limited, to life skills and peer education, adult health education, school-based education, couple's communication, community mobilization, working through gatekeepers, and/or mass media interventions for a general public audience. These may be supported through implementation at the community or individual levels and may relate to outcomes such as increase in FP uptake and changes in FP knowledge and attitudes.

	1	2	3	4	5	6	7	8	9	10	Don't know
1-Lowest -> Highest-10	○	○	○	○	○	○	○	○	○	○	○

* FP 5.10 Youth: Extent to which CSOs support FP policies, interventions and programming targeting youth specifically.

	1	2	3	4	5	6	7	8	9	10	Don't know
1-Lowest -> Highest-10	○	○	○	○	○	○	○	○	○	○	○

* FP 5.11 Programming for Men: Extent to which CSOs support FP policies, interventions and programming for men.

	1	2	3	4	5	6	7	8	9	10	Don't know
1-Lowest -> Highest-10	◯	◯	◯	◯	◯	◯	◯	◯	◯	◯	◯

* FP 5.12 Advocacy/Accountability: Extent to which CSOs support the training and capacity building of advocates and community members to understand policies, processes, and activities to increase community-led issue identification, collaborative problem solving and more targeted advocacy actions.

	1	2	3	4	5	6	7	8	9	10	Don't know
1-Lowest -> Highest-10	◯	◯	◯	◯	◯	◯	◯	◯	◯	◯	◯

* FP 5.13 CSO-Led Assessment and Monitoring: Extent to which CSOs assess, monitor and report on the effectiveness of policies and programming to improve accountability at the policy and provider levels.

	1	2	3	4	5	6	7	8	9	10	Don't know
1-Lowest -> Highest-10	◯	◯	◯	◯	◯	◯	◯	◯	◯	◯	◯

* FP 5.14 Forming Partnerships between CSOs: Extent to which CSOs have formed national alliances and regional partnerships to strengthen their positioning and potentially improve leadership strength and funding mechanisms.

	1	2	3	4	5	6	7	8	9	10	Don't know
1-Lowest -> Highest-10	◯	◯	◯	◯	◯	◯	◯	◯	◯	◯	◯

FP 5.15 Comments: Please use this section to provide any additional comments about the level of effort, challenges, or successes in the country around the role of the civil society in FP. If you marked “I don’t know” for any of the questions, also use this section to explain why you gave that rating.

JOHNS HOPKINS
BLOOMBERG SCHOOL
of PUBLIC HEALTH

Bill & Melinda Gates Institute for Population and Reproductive Health

Demographic Dividend Effort Index Survey

Module: Sectoral Resilience and Sustainability

Considering the health and socio-economic impact of the COVID-19 and the likely impact on the DD progress, this DD effort index has integrated questions to assess the resilience and sustainability of systems in key sectors of the DD framework. The items included in the COVID-19 related questionnaire below follow the key dimensions of resilient systems and provide critical information on the potential of an effective response to emerging infectious disease threats or other public health emergencies.

Please score each item from 1 to 10, where 1 represents the lowest score (poor state/capability) and 10 represents the highest score (great state/capability).

* Physical: facilities, equipment, system states, and capabilities.

	1	2	3	4	5	6	7	8	9	10	Don't know
FP-M1 - Plan/Prepare - State and capability of equipment, personnel and FP program structure prior to the crisis event. (1 = poor state/capability; 10 = great state/capability)	○	○	○	○	○	○	○	○	○	○	○
FP-M2 - Absorb - Integration: The level of integration of FP services and programs to mitigate the effect of COVID-19 on FP	○	○	○	○	○	○	○	○	○	○	○
FP-M3 - Absorb - Integration: Extent to which effective supply chain management was able to mitigate the effect of COVID-19 on the supply/provision of FP commodities.	○	○	○	○	○	○	○	○	○	○	○
FP-M4 - Recover - Extent to which the FP sector has been able to induce change to recover the pre-COVID-19 level of functionality.	○	○	○	○	○	○	○	○	○	○	○
FP-M5 - Recover - Financing and Donor Management: Level of resource mobilization and allocation to mitigate the effect of COVID-19 on FP.	○	○	○	○	○	○	○	○	○	○	○
FP-M6 - Adapt - Extent to which changes have been made to improve the resilience of the FP sector.	○	○	○	○	○	○	○	○	○	○	○

* Information: creation, manipulation, storage, and utilization of data.

	1	2	3	4	5	6	7	8	9	10	Don't know
FP-M7 - Plan/Prepare - Extent to which FP-related data was sufficiently prepared, presented, analyzed and stored prior to the crisis event.											
FP-M8 - Absorb - Extent to which the FP program has been able to conduct a real-time assessment of functionality, in anticipation of cascading losses in FP service provision.											
FP-M9 - Recover - Extent to which FP data has been used to track the progress of recovery and to anticipate recovery scenarios.											
FP-M10 - Adapt - Extent to which the FP sector is creating and improving FP data storage and real-time use protocols.											

* Cognitive: understanding, mental models, preconceptions, biases, and values.

	1	2	3	4	5	6	7	8	9	10	Don't know
FP-M11 - Plan/Prepare - Extent to which the FP program design and operation decisions were prepared in anticipation of crisis events.	○	○	○	○	○	○	○	○	○	○	○
FP-M12 - Absorb - Extent to which the FP sector is responding with sufficient contingency protocols and proactive crisis management.	○	○	○	○	○	○	○	○	○	○	○
FP-M13 - Absorb - Accountability: Level of effort to ensure optimal accountability for the resources allocated to support FP as part of the response to the COVID-19.	○	○	○	○	○	○	○	○	○	○	○
FP-M14 - Recover - Extent to which decisions are oriented towards recovery, which employ evidence-based communication to community members to promote safe behavior.	○	○	○	○	○	○	○	○	○	○	○
FP-M15 - Recover - Awareness: Extent to which the FP sector is building confidence through communication regarding barriers posed by COVID-19 to support access to and uptake of FP.	○	○	○	○	○	○	○	○	○	○	○
FP-M16 - Adapt - Adaptive: Extent to which the national leadership has made changes to adapt and respond to the COVID-19 threats to FP.	○	○	○	○	○	○	○	○	○	○	○

* Social: interaction, collaboration, and self-synchronization between individuals, entities and institutions.

	1	2	3	4	5	6	7	8	9	10	Don't know
FP-M17 - Plan/Prepare - Extent to which training on outbreak/crisis management was conducted through, and leveraged, social networks, social capital and institutional and cultural norms prior to the crisis event.	○	○	○	○	○	○	○	○	○	○	○
FP-M18 - Absorb - Extent to which personnel and social institutions have been resourceful and accessible for the outbreak/crisis response.	○	○	○	○	○	○	○	○	○	○	○
FP-M19 - Absorb - Self-Regulating: Extent to which the national leadership had the authority to make sectoral changes in a timely manner through flexible infrastructure.	○	○	○	○	○	○	○	○	○	○	○
FP-M20 - Recover - Extent to which the FP sector is engaging with team and knowledge sharing to enhance systematic recovery.	○	○	○	○	○	○	○	○	○	○	○
FP-M21 - Recover - Diversity: The level of engagement of a multidisciplinary team to mitigate the effects of COVID-19 on FP.	○	○	○	○	○	○	○	○	○	○	○
FP-M22 - Adapt - Extent to which there are additions or changes being made to FP institutions, policies, trainings, programs and culture.	○	○	○	○	○	○	○	○	○	○	○
FP-M23 - Adapt - Leadership and Management: Level of leadership demonstrated by the FP leaders to mitigate the effect of COVID-19 on FP.	○	○	○	○	○	○	○	○	○	○	○

* FP M-24 Timeline: Extent of timeliness of the Government reaction - communication and implementation of measures - to mitigate the immediate and longterm impact of COVID-19.

Note: This refers to the entirety of the national reaction - not the reaction that is specific to your sector.

	1	2	3	4	5	6	7	8	9	10	Don't know
1-Lowest -> Highest-10	◯	◯	◯	◯	◯	◯	◯	◯	◯	◯	◯

FP M-25 Comments: Please use this section to provide any additional comments about the resilience and sustainability of the national FP systems based on your experience during the COVID-19 pandemics. If you marked “I don’t know” for any of the questions, also use this section to explain why you gave that rating.

JOHNS HOPKINS
BLOOMBERG SCHOOL
of PUBLIC HEALTH

Bill & Melinda Gates Institute for Population and Reproductive Health

Demographic Dividend Effort Index Survey

Maternal & Child Health (MCH) Questionnaire

Please respond to the following questions based on your experience/expertise within this specific sector.

To give a summary picture of the national demographic dividend effort, please rate the following items. Score each item from 1 to 10, where 1 represents the lowest score (very weak or no effort) and 10 represents the highest score (strong effort). Where applicable, if a policy or activity is non-existent then mark your response as 0.

Answer each item. All responses will be recorded in the format exemplified below:

Lowest effort	1	2	3	4	5	6	7	8	9	10	Highest effort	I do not know

JOHNS HOPKINS BLOOMBERG SCHOOL of PUBLIC HEALTH

Bill & Melinda Gates Institute for Population and Reproductive Health

Demographic Dividend Effort Index Survey

Dimension 1. Policy/Policy making

* MCH 1.1 Adequate Policy: Ministry of Health policies toward pregnancy and delivery services that account for the breadth of requirements to ensure that every mother and newborn can survive.

	1	2	3	4	5	6	7	8	9	10	Don't know
MCH 1.1.1 Extent to which reproductive, maternal and child health has been integrated into the national health policy/plan/strategy.	○	○	○	○	○	○	○	○	○	○	○
MCH 1.1.2 Extent to which national reproductive, maternal and child health strategy(ies) and implementation plan(s) have been made available.	○	○	○	○	○	○	○	○	○	○	○

* MCH 1.2 Universal Access: Extent to which there are constitutional and legal entitlements that facilitate universal access to reproductive, maternal and child health services.

	1	2	3	4	5	6	7	8	9	10	Don't know
1-Lowest -> Highest-10	○	○	○	○	○	○	○	○	○	○	○

* MCH 1.3 Multisector Involvement: Extent to which policies are developed through adequate consultation with interested parties such as other ministries, NGOs, private practitioners, and women's groups.

	1	2	3	4	5	6	7	8	9	10	Don't know
1-Lowest -> Highest-10	○	○	○	○	○	○	○	○	○	○	○

* MCH 1.4 Competent Service Providers: Extent to which policies are reasonable and evidence-based concerning which personnel can provide maternal health services (e.g. trained midwives can perform a wide range of medical procedures).

	1	2	3	4	5	6	7	8	9	10	Don't know
1-Lowest -> Highest-10	○	○	○	○	○	○	○	○	○	○	○

* MCH 1.5 Abortion Legislation: Extent to which a favorable policy exists towards the legal provisioning of abortion services.

	1	2	3	4	5	6	7	8	9	10	Don't know
1-Lowest -> Highest-10	○	○	○	○	○	○	○	○	○	○	○

* MCH 1.6. Post-Abortion Care Legislation: Extent to which a favorable policy exists toward the treatment of complications of abortions, including complications seen from illegal abortions.

	1	2	3	4	5	6	7	8	9	10	Don't know
1-Lowest -> Highest-10	○	○	○	○	○	○	○	○	○	○	○

* MCH 1.7 Policy Reviews and Updates: Extent to which policies are regularly reviewed by high-level policymakers and reviews are used to update action plans.

	1	2	3	4	5	6	7	8	9	10	Don't know
1-Lowest -> Highest-10	○	○	○	○	○	○	○	○	○	○	○

* MCH 1.8 High-Level Actor Placement: Extent and level to which the director of maternal and child health services is involved in high-level decision making.

	1	2	3	4	5	6	7	8	9	10	Don't know
1-Lowest -> Highest-10	○	○	○	○	○	○	○	○	○	○	○

* MCH 1.9 Financing for MCH: Extent to which the government budgets for safe pregnancy, delivery, perinatal care (for facilities, personnel, supplies, etc.) and children's health is adequate for the needs, whether from the Ministry of Health, provincial/ county government or donor support.

	1	2	3	4	5	6	7	8	9	10	Don't know
1-Lowest -> Highest-10	○	○	○	○	○	○	○	○	○	○	○

* MCH 1.10 Affordability: Extent to which all MCH-related services and drugs are affordable to all clients.

	1	2	3	4	5	6	7	8	9	10	Don't know
1-Lowest -> Highest-10	○	○	○	○	○	○	○	○	○	○	○

* MCH 1.11 Private Sector: Extent to which the private sector (doctors, midwives, clinics) is active and covers a substantial share of pregnancy and delivery cases.

	1	2	3	4	5	6	7	8	9	10	Don't know
1-Lowest -> Highest-10	○	○	○	○	○	○	○	○	○	○	○

* MCH 1.12 MCH Department: Extent to which the Ministry of Health has a department that is specifically devoted to MCH.

	1	2	3	4	5	6	7	8	9	10	Don't know
1-Lowest -> Highest-10	○	○	○	○	○	○	○	○	○	○	○

* MCH 1.13 Potential Solutions: Extent to which the government recognizes potential solutions to MCH-related issues through the means of:

	1	2	3	4	5	6	7	8	9	10	Don't know
MCH 1.13.1 Regulatory efficiency	○	○	○	○	○	○	○	○	○	○	○
MCH 1.13.2 Performance and accountability	○	○	○	○	○	○	○	○	○	○	○
MCH 1.13.3 A human rights-based approach to maternal, newborn and child health	○	○	○	○	○	○	○	○	○	○	○
MCH 1.13.4 Essential health infrastructure	○	○	○	○	○	○	○	○	○	○	○
MCH 1.13.5 Community engagement	○	○	○	○	○	○	○	○	○	○	○

MCH 1.14 Comments: Please use this section to provide any additional comments about the level of effort, challenges, or successes in the country around policies/policymaking for MCH. If you marked “I don’t know” for any of the questions, also use this section to explain why you gave that rating

JOHNS HOPKINS
BLOOMBERG SCHOOL
of PUBLIC HEALTH

Bill & Melinda Gates Institute for Population and Reproductive Health

Demographic Dividend Effort Index Survey

Dimension 2. Services or Programs

* MCH 2.1 Obstetric Care Availability – All Centers: Extent to which all primary health facilities have trained staff, in place, who can provide obstetric care to:

	1	2	3	4	5	6	7	8	9	10	Don't know
MCH 2.1.1 Properly manage postpartum hemorrhage cases.	○	○	○	○	○	○	○	○	○	○	○
MCH 2.1.2 Timely and properly administer antibiotics intravenously when needed.	○	○	○	○	○	○	○	○	○	○	○
MCH 2.1.3 Perform manual removal of retained placenta.	○	○	○	○	○	○	○	○	○	○	○
MCH 2.1.4 Perform vacuum aspiration of the uterus, using MVA (manual vacuum aspiration) or an electric suction device.	○	○	○	○	○	○	○	○	○	○	○
MCH 2.1.5 Use a partograph to determine when to refer.	○	○	○	○	○	○	○	○	○	○	○
MCH 2.1.6 Have transportation arrangements to quickly move a woman with obstructed labor to a district/sub-county hospital.	○	○	○	○	○	○	○	○	○	○	○
MCH 2.1.7 Have adequate antibiotic supplies on hand (sufficient supplies of the correct types).	○	○	○	○	○	○	○	○	○	○	○

* MCH 2.2 Obstetric Care Availability – Secondary Healthcare Facilities: Extent to which all district/sub-county hospitals have trained staff, in place, who can:

	1	2	3	4	5	6	7	8	9	10	Don't know
MCH 2.2.1 Provide all functions listed above for primary health facilities.	○	○	○	○	○	○	○	○	○	○	○
MCH 2.2.2 Perform blood transfusions (and have adequate supplies of safe blood on hand).	○	○	○	○	○	○	○	○	○	○	○
MCH 2.2.3 Perform Cesarean section.	○	○	○	○	○	○	○	○	○	○	○

* MCH 2.3 Pregnancy-Related Services: Extent to which all pregnant women have adequate access to:

	1	2	3	4	5	6	7	8	9	10	Don't know
MCH 2.3.1 Treatment for postpartum hemorrhage during or soon after delivery.	○	○	○	○	○	○	○	○	○	○	○
MCH 2.3.2 Management of obstructed labor.	○	○	○	○	○	○	○	○	○	○	○
MCH 2.3.3 Management of pre-eclampsia, eclampsia and its complications.	○	○	○	○	○	○	○	○	○	○	○
MCH 2.3.4 Treatment of post abortion care or for abortion complications.	○	○	○	○	○	○	○	○	○	○	○
MCH 2.3.5 Provision of safe abortion services.	○	○	○	○	○	○	○	○	○	○	○
MCH 2.3.6 Antenatal care during pregnancy.	○	○	○	○	○	○	○	○	○	○	○
MCH 2.3.7 Delivery care by a trained professional attendant.	○	○	○	○	○	○	○	○	○	○	○
MCH 2.3.8 Postpartum family planning services.	○	○	○	○	○	○	○	○	○	○	○
MCH 2.3.9 District/sub-county hospitals that are open 24 hours/day.	○	○	○	○	○	○	○	○	○	○	○

* MCH 2.4 Ante-natal Care for Unmarried and HIV Positive Women: Extent to which, at all antenatal visits, all pregnant women:

	1	2	3	4	5	6	7	8	9	10	Don't know
MCH 2.4.1 Receive WHO-recommended complete antenatal care protocol for HIV negative women	○	○	○	○	○	○	○	○	○	○	○
MCH 2.4.2 Receive WHO-recommended complete antenatal care protocol for HIV positive women	○	○	○	○	○	○	○	○	○	○	○
MCH 2.4.3 Receive WHO-recommended complete antenatal care protocol for young unmarried women.	○	○	○	○	○	○	○	○	○	○	○
MCH 2.4.4 Are informed about danger signs of obstetric and newborn complications and are assisted in planning for any emergency.	○	○	○	○	○	○	○	○	○	○	○

* MCH 2.5 Newborn Care: Extent to which, for newborn care, all infants whether delivered at home or in a facility:

	1	2	3	4	5	6	7	8	9	10	Don't know
MCH 2.5.1 Have their mouth and nasal passageways cleared	○	○	○	○	○	○	○	○	○	○	○
MCH 2.5.2 Are dried and kept warm immediately after birth	○	○	○	○	○	○	○	○	○	○	○
MCH 2.5.3 Receive vitamin A	○	○	○	○	○	○	○	○	○	○	○
MCH 2.5.4 Have their umbilical cord cut with a clean blade	○	○	○	○	○	○	○	○	○	○	○
MCH 2.5.5 Complete WHO recommended vaccination calendar	○	○	○	○	○	○	○	○	○	○	○
MCH 2.5.6 Benefit from exclusive breastfeeding promotion and education activities	○	○	○	○	○	○	○	○	○	○	○
MCH 2.5.7 Benefit from nutritional support programs when in need.	○	○	○	○	○	○	○	○	○	○	○

* MCH 2.6 Family Planning – All Centers: Extent to which there exists proper provision of family planning at all health centers that:

	1	2	3	4	5	6	7	8	9	10	Don't know
MCH 2.6.1 Routinely offer family planning services for post-abortion cases	○	○	○	○	○	○	○	○	○	○	○
MCH 2.6.2 Routinely offer family planning at postpartum visits	○	○	○	○	○	○	○	○	○	○	○
MCH 2.6.3 Have short-term and LARC contraceptive supplies regularly in stock	○	○	○	○	○	○	○	○	○	○	○
MCH 2.6.4 Have trained staff, in place, who can provide services for LARCs.	○	○	○	○	○	○	○	○	○	○	○
MCH 2.6.5 Have guidelines and protocols necessary for the provision of family planning services.	○	○	○	○	○	○	○	○	○	○	○

* MCH 2.7 Family Planning – District/Sub-county Hospitals: Extent to which there exists proper provision of family planning at district/sub-county hospitals that:

	1	2	3	4	5	6	7	8	9	10	Don't know
MCH 2.7.1 Routinely offer family planning services for post-abortion cases	○	○	○	○	○	○	○	○	○	○	○
MCH 2.7.2 Routinely offer family planning at postpartum visits	○	○	○	○	○	○	○	○	○	○	○
MCH 2.7.3 Have short term and LARC contraceptive supplies regularly in stock	○	○	○	○	○	○	○	○	○	○	○
MCH 2.7.4 Have trained staff, in place, who can provide services for LARCs	○	○	○	○	○	○	○	○	○	○	○
MCH 2.7.5 Can offer sterilization to female clients	○	○	○	○	○	○	○	○	○	○	○
MCH 2.7.6 Can offer sterilization to male clients	○	○	○	○	○	○	○	○	○	○	○
MCH 2.7.7 Have guidelines and protocols necessary for the provision of family planning services.	○	○	○	○	○	○	○	○	○	○	○

* MCH 2.8 Childhood Illness: Extent to which the WHO-recommended case management of childhood illness is implemented regarding:

	1	2	3	4	5	6	7	8	9	10	Don't know
MCH 2.8.1 Community case management of childhood illnesses	○	○	○	○	○	○	○	○	○	○	○
MCH 2.8.2 Effectiveness of the referral services from community level to the health facilities	○	○	○	○	○	○	○	○	○	○	○

MCH 2.9 Comments: Please use this section to provide any additional comments about the level of effort, challenges, or successes in the country around services or programs for MCH. If you marked “I don’t know” for any of the questions, also use this section to explain why you gave that rating.

JOHNS HOPKINS
BLOOMBERG SCHOOL
of PUBLIC HEALTH

Bill & Melinda Gates Institute for Population and Reproductive Health

Demographic Dividend Effort Index Survey

Dimension 3. Advocacy

* MCH 3.1 Government Messages: Extent to which high officials in the government, including the Ministry of Health, issue frequent statements to all stakeholders including the press to support improvements for safe pregnancy, safe delivery and healthy childhood.

	1	2	3	4	5	6	7	8	9	10	Don't know
1-Lowest -> Highest-10	○	○	○	○	○	○	○	○	○	○	○

* MCH 3.2 Mass Media: Extent to which the national program uses the mass media to educate the public about symptoms of pregnancy complications, safe childbirth and healthy childhood.

	1	2	3	4	5	6	7	8	9	10	Don't know
1-Lowest -> Highest-10	○	○	○	○	○	○	○	○	○	○	○

* MCH 3.3 Community-Level Media: Extent to which community groups take part in systematic programs to educate the public about safe pregnancy, safe delivery and healthy childhood.

	1	2	3	4	5	6	7	8	9	10	Don't know
1-Lowest -> Highest-10	○	○	○	○	○	○	○	○	○	○	○

* MCH 3.4 Educational Materials: Extent to which the appropriate ministry (MOH) supplies adequate educational materials (posters, pamphlets, etc.) to delivery facilities to instruct clients about safe practices.

	1	2	3	4	5	6	7	8	9	10	Don't know
1-Lowest -> Highest-10	○	○	○	○	○	○	○	○	○	○	○

* MCH 3.5 Self-Review of Maternal Mortality Cases: Extent to which each health facility follows a regular procedure to review and learn from every case of a maternal death in the facility.

	1	2	3	4	5	6	7	8	9	10	Don't know
1-Lowest -> Highest-10	◯	◯	◯	◯	◯	◯	◯	◯	◯	◯	◯

* MCH 3.6 Informing Policies with New Evidence: Extent to which each health facility shares lessons from maternal death cases to the ministries to inform new policies or interventions.

	1	2	3	4	5	6	7	8	9	10	Don't know
1-Lowest -> Highest-10	◯	◯	◯	◯	◯	◯	◯	◯	◯	◯	◯

MCH 3.7 Comments: Please use this section to provide any additional comments about the level of effort, challenges, or successes in the country around advocacy for MCH. If you marked “I don’t know” for any of the questions, also use this section to explain why you gave that rating

JOHNS HOPKINS
BLOOMBERG SCHOOL
of PUBLIC HEALTH

Bill & Melinda Gates Institute for Population and Reproductive Health

Demographic Dividend Effort Index Survey

Dimension 4. Research

* MCH 4.1 Strength of Observatory/Stakeholder/Technical Working Group for MCH: Extent to which country-level MCH stakeholders (e.g., stakeholder leadership group, technical working group, MCH observatory, country coordination and facilitation group):

	1	2	3	4	5	6	7	8	9	10	Don't know
MCH 4.1.1 Have representation in government, training institutions, civil society, nongovernmental and faith-based organizations, professional associations, the private sector, etc.	○	○	○	○	○	○	○	○	○	○	○
MCH 4.1.2 Meet regularly, report and recommend policy for senior management within the appropriate ministries	○	○	○	○	○	○	○	○	○	○	○
MCH 4.1.3 Make an impact on MCH within the country	○	○	○	○	○	○	○	○	○	○	○

* MCH 4.2 MCH Research Strategy: Extent to which national documents include a comprehensive strategy/approach on MCH-related research and is shared among partners.

	1	2	3	4	5	6	7	8	9	10	Don't know
1-Lowest -> Highest-10	○	○	○	○	○	○	○	○	○	○	○

* MCH 4.3 Data-Gathering Partners:

	1	2	3	4	5	6	7	8	9	10	Don't know
MCH 4.3.1 Extent to which research is undertaken by government agencies. (This may include statistical agencies/bureaus and/or ministries of economics/finances, education, health, gender, etc.)	○	○	○	○	○	○	○	○	○	○	○
MCH 4.3.2 Extent to which data-gathering is undertaken by research institutions.	○	○	○	○	○	○	○	○	○	○	○
MCH 4.3.3 Extent to which data-gathering is undertaken by independent researchers.	○	○	○	○	○	○	○	○	○	○	○

* MCH 4.4 Topical Research:

	1	2	3	4	5	6	7	8	9	10	Don't know
MCH 4.4.1 Extent to which maternal mortality is researched.	○	○	○	○	○	○	○	○	○	○	○
MCH 4.4.2 Extent to which infant and child mortality is researched.	○	○	○	○	○	○	○	○	○	○	○
MCH 4.4.3 Extent to which family planning and post-partum family planning is researched.	○	○	○	○	○	○	○	○	○	○	○
MCH 4.4.4 Extent to which MCH service provision/access and provider quality is researched.	○	○	○	○	○	○	○	○	○	○	○

* MCH 4.5 Quality/Coverage of Data: Extent to which current research/data is disaggregated, where relevant, on the basis of sex, age, geographic location, ethnicity, education, income quintile and disability status. Extent to which the country participates in standardized tests and its ranking.

	1	2	3	4	5	6	7	8	9	10	Don't know
MCH 4.5.1 Extent to which a routine statistical system provides good periodic information on supplies and facilities.	○	○	○	○	○	○	○	○	○	○	○
MCH 4.5.2 Extent to which a routine statistical system provides good periodic information on MCH services and personnel.	○	○	○	○	○	○	○	○	○	○	○
MCH 4.5.3 Extent to which the routine statistical system provides good periodic information on MCH needs among populations in different communities.	○	○	○	○	○	○	○	○	○	○	○
MCH 4.5.4 Extent to which evaluations of the health monitoring and information system are conducted and applied to ensure the reliability of data.	○	○	○	○	○	○	○	○	○	○	○
MCH 4.5.5 Extent to which evaluations of the health monitoring and information system are conducted and applied to explore inequalities in MNCH services and burdens.	○	○	○	○	○	○	○	○	○	○	○

* MCH 4.6 Record-Keeping: Extent to which systems for client recordkeeping, clinic reporting and feedback of results are adequate, including being connected at all levels (federal, state/province, county, etc.).

	1	2	3	4	5	6	7	8	9	10	Don't know
1-Lowest -> Highest-10	○	○	○	○	○	○	○	○	○	○	○

* MCH 4.7 Quality Research Institutions: Extent to which the country has the capacity to support and sustain research institutions that develop research/collect data to ensure high quality MCH services.

	1	2	3	4	5	6	7	8	9	10	Don't know
1-Lowest -> Highest-10	○	○	○	○	○	○	○	○	○	○	○

* MCH 4.8 Evaluation: Extent to which program statistics, surveys, and small studies are used by specialized staff to report on program operations and measure progress.

	1	2	3	4	5	6	7	8	9	10	Don't know
1-Lowest -> Highest-10	○	○	○	○	○	○	○	○	○	○	○

* MCH 4.9 Management's Use of Evaluation Findings: Extent to which local-level program managers use research and evaluation findings to improve programming in ways suggested by findings.

	1	2	3	4	5	6	7	8	9	10	Don't know
1-Lowest -> Highest-10	○	○	○	○	○	○	○	○	○	○	○

* MCH 4.10 Ministerial Use of Evaluation Findings: Extent to which the Ministry administrators and policymakers systematically utilize data to inform policies and interventions to address MCH-related issues.

	1	2	3	4	5	6	7	8	9	10	Don't know
1-Lowest -> Highest-10	○	○	○	○	○	○	○	○	○	○	○

* MCH 4.11 Dissemination of Information to Other Implementing Bodies: Extent to which information is shared or spread across geographical areas and throughout various levels (national, state/province, localities).

	1	2	3	4	5	6	7	8	9	10	Don't know
1-Lowest -> Highest-10	○	○	○	○	○	○	○	○	○	○	○

MCH 4.12 Comments: Please use this section to provide any additional comments about the level of effort, challenges, or successes in the country around MCH research. If you marked "I don't know" for any of the questions, also use this section to explain why you gave that rating.

JOHNS HOPKINS
BLOOMBERG SCHOOL
of PUBLIC HEALTH

Bill & Melinda Gates Institute for Population and Reproductive Health

Demographic Dividend Effort Index Survey

Dimension 5. Civil Society Organizations (CSOs)

* MCH 5.1 CSO Actor Power: Extent to which CSO actors are in strong placements within institutions or technical working groups or networks concerned with MCH.

	1	2	3	4	5	6	7	8	9	10	Don't know
1-Lowest -> Highest-10	○	○	○	○	○	○	○	○	○	○	○

* MCH 5.2 Budget Analysis as a CSO Tool: Extent to which CSOs utilize budget analysis as a tool to build MCH advocacy cases.

	1	2	3	4	5	6	7	8	9	10	Don't know
1-Lowest -> Highest-10	○	○	○	○	○	○	○	○	○	○	○

* MCH 5.3 Facility-Based Service Delivery Support: Extent to which CSOs are involved in efforts to increase the use and/or quality of services, particularly through increasing the technical and/or operational capacity of CSO-partnered clinics to provide MCH services.

	1	2	3	4	5	6	7	8	9	10	Don't know
1-Lowest -> Highest-10	○	○	○	○	○	○	○	○	○	○	○

* MCH 5.4 Community-Based Service Delivery Support: Extent to which CSOs participate in bringing MCH information and services to the communities where people live through activities such as supporting the capacity-building of community-level health workers, expanding the range of MCH services provided by community health workers, educating lower cadre health workers, and/or undertaking information and education campaigns.

	1	2	3	4	5	6	7	8	9	10	Don't know
1-Lowest -> Highest-10	○	○	○	○	○	○	○	○	○	○	○

* MCH 5.5 Mobile Outreach for Community-Based Service Delivery Support: Extent to which CSOs support outreach models to equip health care providers with MCH-related commodities, supplies, equipment and vehicles, which are then sent as specialist team to provide MCH services directly to communities.

	1	2	3	4	5	6	7	8	9	10	Don't know
1-Lowest -> Highest-10	○	○	○	○	○	○	○	○	○	○	○

* MCH 5.6 Social Franchising: Extent to which CSOs are involved with social franchising opportunities/services to bring private sector services to clients at reduced prices of MCH services.

	1	2	3	4	5	6	7	8	9	10	Don't know
1-Lowest -> Highest-10	○	○	○	○	○	○	○	○	○	○	○

* MCH 5.7 mHealth: Extent to which CSOs are leveraging mHealth to utilize text messages and other mobile technologies to improve access to MCH information and services.

	1	2	3	4	5	6	7	8	9	10	Don't know
1-Lowest -> Highest-10	○	○	○	○	○	○	○	○	○	○	○

* MCH 5.8 Human Rights and Quality of Care: Extent to which CSOs advocate for and support rights-based approaches to MCH to ensure MCH service recipients experience good treatment, confidence in asking questions, and quality counselling, amongst other concerns.

	1	2	3	4	5	6	7	8	9	10	Don't know
1-Lowest -> Highest-10	○	○	○	○	○	○	○	○	○	○	○

* MCH 5.9 Social and Behavior Change (SBC): Extent to which CSOs support SBC interventions relating, but not limited, to life skills and peer education, adult health education, school-based education, couple's communication, community mobilization, working through gatekeepers, and/or mass media interventions for a general public audience.

	1	2	3	4	5	6	7	8	9	10	Don't know
1-Lowest -> Highest-10	○	○	○	○	○	○	○	○	○	○	○

* MCH 5.10 Advocacy/Accountability: Extent to which CSOs support the training and capacity building of advocates and community members to understand policies, processes, and activities to increase community-led issue identification, collaborative problem solving and more targeted advocacy actions.

	1	2	3	4	5	6	7	8	9	10	Don't know
1-Lowest -> Highest-10	○	○	○	○	○	○	○	○	○	○	○

* MCH 5.11 CSO-Led Assessment and Monitoring: Extent to which CSOs assess, monitor and report on the effectiveness of policies and programming to improve accountability at the policy and provider levels.

	1	2	3	4	5	6	7	8	9	10	Don't know
1-Lowest -> Highest-10	◯	◯	◯	◯	◯	◯	◯	◯	◯	◯	◯

* MCH 5.12 Forming Partnerships between CSOs: Extent to which CSOs have formed national alliances and regional partnerships to strengthen their positioning and potentially improve leadership strength and funding mechanisms for MCH.

	1	2	3	4	5	6	7	8	9	10	Don't know
1-Lowest -> Highest-10	◯	◯	◯	◯	◯	◯	◯	◯	◯	◯	◯

MCH 5.13 Comments: Please use this section to provide any additional comments about the level of effort, challenges, or successes in the country around the involvement of the civil society in MCH. If you marked “I don’t know” for any of the questions, also use this section to explain why you gave that rating

JOHNS HOPKINS
BLOOMBERG SCHOOL
of PUBLIC HEALTH

Bill & Melinda Gates Institute for Population and Reproductive Health

Demographic Dividend Effort Index Survey

Module: Sectoral Resilience and Sustainability

Considering the health and socio-economic impact of the COVID-19 and the likely impact on the DD progress, this DD effort index has integrated questions to assess the resilience and sustainability of systems in key sectors of the DD framework. The items included in the COVID-19 related questionnaire below follow the key dimensions of resilient systems and provide critical information on the potential of an effective response to emerging infectious disease threats or other public health emergencies.

Please score each item from 1 to 10, where 1 represents the lowest score (poor state/capability) and 10 represents the highest score (great state/capability).

* Physical: facilities, equipment, system states, and capabilities.

	1	2	3	4	5	6	7	8	9	10	Don't know
MCH-M1 - Plan/Prepare - State and capability of equipment, personnel and MCH program structure prior to the crisis event. (1 = poor state/capability; 10 = great state/capability)	◯	◯	◯	◯	◯	◯	◯	◯	◯	◯	◯
MCH-M2 - Absorb - Integration: The level of integration of MCH services and programs to mitigate the effect of COVID-19 on MCH.	◯	◯	◯	◯	◯	◯	◯	◯	◯	◯	◯
MCH-M3 - Absorb - Integration: Extent to which effective supply chain management was able to mitigate the effect of COVID-19 on the supply/provision of MCH-related commodities.	◯	◯	◯	◯	◯	◯	◯	◯	◯	◯	◯
MCH-M4 - Recover - Extent to which the MCH program has been able to induce change to recover the pre-COVID-19 level of functionality.	◯	◯	◯	◯	◯	◯	◯	◯	◯	◯	◯
MCH-M5 - Recover - Financing and Donor Management: Level of resources mobilization and allocation to mitigate the effect of COVID-19 on MCH.	◯	◯	◯	◯	◯	◯	◯	◯	◯	◯	◯
MCH-M6 - Adapt - Extent to which changes have been made to improve the resilience of the MCH sector.	◯	◯	◯	◯	◯	◯	◯	◯	◯	◯	◯

* Information: creation, manipulation, storage, and utilization of data.

	1	2	3	4	5	6	7	8	9	10	Don't know
MCH-M7 - Plan/Prepare - Extent to which MCH-related data was sufficiently prepared, presented, analyzed and stored prior to the crisis event.	◯	◯	◯	◯	◯	◯	◯	◯	◯	◯	◯
MCH-M8 - Absorb - Awareness: Extent of the surveillance capacity to detect the MCH limitations/threats posed by COVID-19.	◯	◯	◯	◯	◯	◯	◯	◯	◯	◯	◯
MCH-M9 - Recover - Extent to which MCH data has been used to track the progress of recovery and to anticipate recovery scenarios.	◯	◯	◯	◯	◯	◯	◯	◯	◯	◯	◯
MCH-M10 - Adapt - Extent to which the MCH sector is creating and improving MCH data storage and real-time use protocols.	◯	◯	◯	◯	◯	◯	◯	◯	◯	◯	◯

* Cognitive: understanding, mental models, preconceptions, biases, and values.

	1	2	3	4	5	6	7	8	9	10	Don't know
MCH-M11 - Plan/Prepare - Extent to which the MCH sector design and operation decisions were prepared in anticipation of crisis events.	○	○	○	○	○	○	○	○	○	○	○
MCH-M12 - Absorb - Extent to which the MCH sector is responding with sufficient contingency protocols and proactive crisis management.	○	○	○	○	○	○	○	○	○	○	○
MCH-M13 - Absorb - Accountability: Level of effort to ensure optimal accountability for the resources allocated to support MCH as part of the response to the COVID-19.	○	○	○	○	○	○	○	○	○	○	○
MCH-M14 - Recover - Extent to which decisions are oriented towards recovery, which employ evidence-based communication to community members to promote safe behavior.	○	○	○	○	○	○	○	○	○	○	○
MCH-M15 - Recover - Awareness: Extent to which the MCH sector is building confidence through communication regarding barriers posed by COVID-19 to support access to and uptake of MCH services and programs.	○	○	○	○	○	○	○	○	○	○	○
MCH-M16 - Adapt – Adaptive: Extent to which the national leadership had the authority to make changes to adapt and respond to the COVID-19 threats to MCH services and programs.	○	○	○	○	○	○	○	○	○	○	○

* Social: interaction, collaboration, and self-synchronization between individuals, entities and institutions.

	1	2	3	4	5	6	7	8	9	10	Don't know
MCH-M17 - Plan/Prepare - Extent to which training on outbreak/crisis management was conducted through, and which leveraged, social networks, social capital and institutional and cultural norms prior to the crisis event.	○	○	○	○	○	○	○	○	○	○	○
MCH-M18 - Absorb - Extent to which personnel and social institutions have been resourceful and accessible for the outbreak/crisis response.	○	○	○	○	○	○	○	○	○	○	○
MCH-M19 - Absorb - Self-Regulating: Extent to which the national leadership had the authority to make sectoral changes in a timely manner through flexible infrastructure.	○	○	○	○	○	○	○	○	○	○	○
MCH-M20 - Recover - Extent to which the MCH sector is engaging with team and knowledge sharing to enhance systematic recovery.	○	○	○	○	○	○	○	○	○	○	○
MCH-M21 - Recover - Diversity: The level of engagement of a multidisciplinary team to mitigate the effect of COVID19 on MCH.	○	○	○	○	○	○	○	○	○	○	○
MCH-M22 - Adapt - Extent to which there are additions or changes being made to MCH institutions, policies, trainings, programs and culture.	○	○	○	○	○	○	○	○	○	○	○

	1	2	3	4	5	6	7	8	9	10	Don't know
MCH-M23 - Adapt - Leadership and Management: Level of leadership demonstrated by the MCH leaders to mitigate the effect of COVID-19 on MCH.	○	○	○	○	○	○	○	○	○	○	○

* MCH M-24 Timeline: Extent of timeliness of the Government reaction - communication and implementation of measures - to mitigate the immediate and longterm impact of COVID-19.
Note: This refers to the entirety of the national reaction - not the reaction that is specific to your sector.

	1	2	3	4	5	6	7	8	9	10	Don't know
1-Lowest -> Highest-10	○	○	○	○	○	○	○	○	○	○	○

MCH M-25 Comments: Please use this section to provide any additional comments about the resilience and sustainability of the national MCH systems based on your experience during the COVID-19 pandemics. If you marked “I don’t know” for any of the questions, also use this section to explain why you gave that rating.

JOHNS HOPKINS
BLOOMBERG SCHOOL of PUBLIC HEALTH

Bill & Melinda Gates Institute for Population and Reproductive Health

Demographic Dividend Effort Index Survey

Education (ED) Questionnaire

Please respond to the following questions based on your experience/expertise within this specific sector.

To give a summary picture of the national demographic dividend effort, please rate the following items. Score each item from 1 to 10, where 1 represents the lowest score (very weak or no effort) and 10 represents the highest score (strong effort). Where applicable, if a policy or activity is non-existent then mark your response as 0.

Answer each item. All responses will be recorded in the format exemplified below:

Lowest effort	1	2	3	4	5	6	7	8	9	10	Highest effort	I do not know

JOHNS HOPKINS
BLOOMBERG SCHOOL
of PUBLIC HEALTH

Bill & Melinda Gates Institute for Population and Reproductive Health

Demographic Dividend Effort Index Survey

Dimension 1. Policy / Policy making

* ED 1.1 Quality: Extent to which there are national plans/strategies/programs to improve the quality of learning in schools.

	1	2	3	4	5	6	7	8	9	10	Don't know
1-Lowest -> Highest-10	○	○	○	○	○	○	○	○	○	○	○

* ED 1.2 Disadvantaged Groups:

	1	2	3	4	5	6	7	8	9	10	Don't know
ED 1.2.1. Extent to which strategies have been developed and implemented/deployed to overcome learning obstacles for pregnant girls and adolescent mothers.	○	○	○	○	○	○	○	○	○	○	○
ED 1.2.2 Extent to which strategies have been developed and implemented/deployed to overcome learning obstacles for disabled populations.	○	○	○	○	○	○	○	○	○	○	○

* ED 1.3 Teachers: Extent to which teacher recruitment, development and management issues are included in policy aimed at improving learning outcomes.

	1	2	3	4	5	6	7	8	9	10	Don't know
1-Lowest -> Highest-10	○	○	○	○	○	○	○	○	○	○	○

* ED 1.4 Teachers' Accountability: Extent to which national plans include specific measures, including monitoring and evaluation, that aim to hold teachers' accountable for improving learning.

	1	2	3	4	5	6	7	8	9	10	Don't know
1-Lowest -> Highest-10	○	○	○	○	○	○	○	○	○	○	○

* ED 1.5 Teacher Quality: Extent to which education policies/strategies/programs include verifiable indicators for teacher quality.

	1	2	3	4	5	6	7	8	9	10	Don't know
1-Lowest -> Highest-10	○	○	○	○	○	○	○	○	○	○	○

* ED 1.6 Collaboration in Education: Extent to which the policy makes provisions to engage multiple stakeholders in education (e.g. schools, school-communities, parents, teachers, local government, parent-teacher-student committees, etc.).

	1	2	3	4	5	6	7	8	9	10	Don't know
1-Lowest -> Highest-10	○	○	○	○	○	○	○	○	○	○	○

* ED 1.7 Gender Parity SDG: Extent to which national policies/plans/strategies consider the SDG goal for complete gender parity at all levels of education.

	1	2	3	4	5	6	7	8	9	10	Don't know
1-Lowest -> Highest-10	○	○	○	○	○	○	○	○	○	○	○

* ED 1.8 Education Financing: Extent to which new education plans include financing proposals with budgets allocated for learning and teaching reforms.

	1	2	3	4	5	6	7	8	9	10	Don't know
1-Lowest -> Highest-10	○	○	○	○	○	○	○	○	○	○	○

* ED 1.9 Gender Parity Planning: Extent to which there are national plans/strategies to reach 100% enrollment with gender parity in:

	1	2	3	4	5	6	7	8	9	10	Don't know
ED 1.9.1 Primary Education	○	○	○	○	○	○	○	○	○	○	○
ED 1.9.2 Secondary Education	○	○	○	○	○	○	○	○	○	○	○
ED 1.9.3 Tertiary Education	○	○	○	○	○	○	○	○	○	○	○

* ED 1.10 Adult Illiteracy: Extent to which national policies/plans/strategies aim to address illiteracy.

	1	2	3	4	5	6	7	8	9	10	Don't know
ED 1.10.1 Extent to which national policies/plans/strategies aim to address adult illiteracy.	○	○	○	○	○	○	○	○	○	○	○
ED 1.10.2 Extent to which national policies/plans/strategies that aim to address adult illiteracy address gendered disparities of this issue.	○	○	○	○	○	○	○	○	○	○	○

* ED 1.11 Out-of-School Children: Extent to which policies address the plight of out-of-school children to reintegrate them into the school system or provide alternative opportunities for their educational development.

	1	2	3	4	5	6	7	8	9	10	Don't know
1-Lowest -> Highest-10	○	○	○	○	○	○	○	○	○	○	○

* ED 1.12 Technology Integration: Extent to which strategies have been developed and implemented/deployed to integrate information communication and technology in education.

	1	2	3	4	5	6	7	8	9	10	Don't know
1-Lowest -> Highest-10	○	○	○	○	○	○	○	○	○	○	○

* ED 1.13. Technology Literacy: Extent to which resources have been allocated to improve computer literacy across education levels.

	1	2	3	4	5	6	7	8	9	10	Don't know
1-Lowest -> Highest-10	○	○	○	○	○	○	○	○	○	○	○

ED 1.14. Comments: Please use this section to provide any additional comments about the level of effort, challenges, or successes in the country around policies/policymaking for ED. If you marked “I don’t know” for any of the questions, also use this section to explain why you gave that rating.

JOHNS HOPKINS
BLOOMBERG SCHOOL
of PUBLIC HEALTH

Bill & Melinda Gates Institute for Population and Reproductive Health

Demographic Dividend Effort Index Survey

Dimension 2. Services or Programs

* ED 2.1 Teacher Deployment: Extent to which strategies/plans are used to ensure teachers are deployed to areas of the country where they are most needed.

	1	2	3	4	5	6	7	8	9	10	Don't know
1-Lowest -> Highest-10	◯	◯	◯	◯	◯	◯	◯	◯	◯	◯	◯

* ED 2.2 Teacher Quality: Extent to which teacher quality is recognized as a key factor in improving learning outcomes.

	1	2	3	4	5	6	7	8	9	10	Don't know
ED 2.2.1 Extent to which the quality of teacher education is emphasized in wider education policies/plans/strategies.	◯	◯	◯	◯	◯	◯	◯	◯	◯	◯	◯
ED 2.2.2 Extent to which the quality of training and continuous professional development is emphasized in wider education policies/plans/strategies.	◯	◯	◯	◯	◯	◯	◯	◯	◯	◯	◯

* ED 2.3 Gender Parity: Extent to which resources have been utilized to improve equality in education between boys and girls.

	1	2	3	4	5	6	7	8	9	10	Don't know
1-Lowest -> Highest-10	◯	◯	◯	◯	◯	◯	◯	◯	◯	◯	◯

* ED 2.4 Parental Involvement in Education: Extent to which parents are involved in the development and execution of the education strategies (i.e. through the involvement of parent-teacher committees).

	1	2	3	4	5	6	7	8	9	10	Don't know
1-Lowest -> Highest-10	○	○	○	○	○	○	○	○	○	○	○

* ED 2.5 Standardized Assessments: Extent to which all schools utilize standardized school and student assessments in line with specifications made by the Ministry of Education.

	1	2	3	4	5	6	7	8	9	10	Don't know
1-Lowest -> Highest-10	○	○	○	○	○	○	○	○	○	○	○

* ED 2.6. Use of Assessment to Inform Progress: Extent to which adjustments to schools are made based on results from national standardized school assessments.

	1	2	3	4	5	6	7	8	9	10	Don't know
1-Lowest -> Highest-10	○	○	○	○	○	○	○	○	○	○	○

ED 2.7 Comments: Please use this section to provide any additional comments about the level of effort, challenges, or successes in the country around services or programs for ED. If you marked “I don’t know” for any of the questions, also use this section to explain why you gave that rating.

JOHNS HOPKINS
BLOOMBERG SCHOOL
of PUBLIC HEALTH

Bill & Melinda Gates Institute for Population and Reproductive Health

Demographic Dividend Effort Index Survey

Dimension 3. Advocacy

* ED 3.1 Equality: Extent to which equity and equality in education have been highlighted by advocates.

	1	2	3	4	5	6	7	8	9	10	Don't know
1-Lowest -> Highest-10	○	○	○	○	○	○	○	○	○	○	○

* ED 3.2 Quality: Extent to which quality of education takes part in advocacy-related evaluations of the education system.

	1	2	3	4	5	6	7	8	9	10	Don't know
1-Lowest -> Highest-10	○	○	○	○	○	○	○	○	○	○	○

* ED 3.3 Teachers' Obstacles: Extent to which the needs of teachers have been highlighted by advocates.

	1	2	3	4	5	6	7	8	9	10	Don't know
1-Lowest -> Highest-10	○	○	○	○	○	○	○	○	○	○	○

* ED 3.4 Institutions' Obstacles: Extent to which the challenges of educational institutions have been highlighted in different advocacy efforts.

	1	2	3	4	5	6	7	8	9	10	Don't know
1-Lowest -> Highest-10	○	○	○	○	○	○	○	○	○	○	○

ED 3.5 Comments: Please use this section to provide any additional comments about the level of effort, challenges, or successes in the country around advocacy for ED. If you marked "I don't know" for any of the questions, also use this section to explain why you gave that rating

JOHNS HOPKINS
BLOOMBERG SCHOOL
of PUBLIC HEALTH

Bill & Melinda Gates Institute for Population and Reproductive Health

Demographic Dividend Effort Index Survey

Dimension 4. Research

* ED 4.1 Strength of Observatory/Stakeholder/Technical Working Group for ED: Extent to which country-level ED stakeholders (e.g., stakeholder leadership group, technical working group, ED observatory, country coordination and facilitation group):

	1	2	3	4	5	6	7	8	9	10	Don't know
ED 4.1.1 Have representation in government, training institutions, civil society, nongovernmental and faith-based organizations, professional associations, the private sector, etc.	○	○	○	○	○	○	○	○	○	○	○
ED 4.1.2 Meet regularly, report and recommend policy for senior management within the appropriate ministries	○	○	○	○	○	○	○	○	○	○	○
ED 4.1.3 Make an impact on education within the country	○	○	○	○	○	○	○	○	○	○	○

* ED 4.2 Education/Research Strategy: Extent to which the national education plan and/or other national documents include a comprehensive strategy/approach on education-related research, shared among partners.

	1	2	3	4	5	6	7	8	9	10	Don't know
1-Lowest -> Highest-10	○	○	○	○	○	○	○	○	○	○	○

* ED 4.3 Data Gathering Partners:

	1	2	3	4	5	6	7	8	9	10	Don't know
ED 4.3.1 Extent to which research is undertaken by government agencies. (This may include statistical agencies/bureaus and/or ministries of economics/finances, education, health, gender, etc.)	○	○	○	○	○	○	○	○	○	○	○
ED 4.3.2 Extent to which data-gathering is undertaken by research institutions.	○	○	○	○	○	○	○	○	○	○	○
ED 4.3.3 Extent to which data-gathering is undertaken by independent researchers.	○	○	○	○	○	○	○	○	○	○	○

* ED 4.4 Topical Research:

	1	2	3	4	5	6	7	8	9	10	Don't know
ED 4.4.1 Extent to which gender disparities in education are researched.	○	○	○	○	○	○	○	○	○	○	○
ED 4.4.2 Extent to which quality of education is researched.	○	○	○	○	○	○	○	○	○	○	○
ED 4.4.3 Extent to which the obstacles faced by teachers are researched.	○	○	○	○	○	○	○	○	○	○	○
ED 4.4.4 Extent to which the obstacles faced by institutions are researched.	○	○	○	○	○	○	○	○	○	○	○

* ED 4.5 Quality/Coverage of Data: Extent to which current research/data is sex and age disaggregated. Extent to which the country participates in standardized tests and its ranking.

	1	2	3	4	5	6	7	8	9	10	Don't know
ED 4.5.1 Extent to which a routine statistical system (using institution/school-based information) provides good periodic information on supplies and facilities.	○	○	○	○	○	○	○	○	○	○	○
ED 4.5.2 Extent to which a routine statistical system (using institution/school-based information) provides good periodic information on education services and personnel.	○	○	○	○	○	○	○	○	○	○	○
ED 4.5.3 Extent to which the routine statistical system provides good periodic information on education needs among populations in different communities.	○	○	○	○	○	○	○	○	○	○	○
ED 4.5.4 Extent to which evaluations of the education monitoring and information system are conducted and applied to ensure the reliability of data.	○	○	○	○	○	○	○	○	○	○	○

* ED 4.6 Record-Keeping: Extent to which systems for education-related recordkeeping, reporting and feedback of results are adequate.

	1	2	3	4	5	6	7	8	9	10	Don't know
1-Lowest -> Highest-10	○	○	○	○	○	○	○	○	○	○	○

* ED 4.7 Quality Research Institutions: Extent to which the country has the capacity to support and sustain research institutions that develop research/collect data that inform efforts to improve education.

	1	2	3	4	5	6	7	8	9	10	Don't know
1-Lowest -> Highest-10	○	○	○	○	○	○	○	○	○	○	○

* ED 4.8 Evaluation: Extent to which education program statistics, surveys, and small studies are used by specialized staff to report on program operations and measure progress.

	1	2	3	4	5	6	7	8	9	10	Don't know
1-Lowest -> Highest-10	○	○	○	○	○	○	○	○	○	○	○

* ED 4.9 Management's Use of Evaluation Findings: Extent to which local-level education program managers use research and evaluation findings to improve these programs in ways suggested by findings.

	1	2	3	4	5	6	7	8	9	10	Don't know
1-Lowest -> Highest-10	○	○	○	○	○	○	○	○	○	○	○

* ED 4.10 Ministerial Use of Evaluation Findings: Extent to which the relevant ministry administrators (i.e. ministries of education) systematically utilize data to inform policies and interventions to address education-related issues.

	1	2	3	4	5	6	7	8	9	10	Don't know
1-Lowest -> Highest-10	○	○	○	○	○	○	○	○	○	○	○

* ED 4.11 Dissemination of Information to Other Implementing Bodies:

	1	2	3	4	5	6	7	8	9	10	Don't know
ED 4.11.1 Extent to which information is shared or spread across geographical areas	○	○	○	○	○	○	○	○	○	○	○
ED. 4.11.2 Extent to which information is shared or spread throughout various levels (national, state/province, localities).	○	○	○	○	○	○	○	○	○	○	○

ED 4.12 Comments: Please use this section to provide any additional comments about the level of effort, challenges, or successes in the country around research in education. If you marked "I don't know" for any of the questions, also use this section to explain why you gave that rating

JOHNS HOPKINS
BLOOMBERG SCHOOL
of PUBLIC HEALTH

Bill & Melinda Gates Institute for Population and Reproductive Health

Demographic Dividend Effort Index Survey

Dimension 5. Civil Society Organizations (CSOs)

* ED 5.1 CSO Actor Power: Extent to which CSO actors are in strong placements within institutions or technical working groups or networks concerned with education.

	1	2	3	4	5	6	7	8	9	10	Don't know
1-Lowest -> Highest-10	◯	◯	◯	◯	◯	◯	◯	◯	◯	◯	◯

* ED 5.2 Budget Analysis as a CSO Tool: Extent to which CSOs utilize budget analysis as a tool to build education advocacy cases.

	1	2	3	4	5	6	7	8	9	10	Don't know
1-Lowest -> Highest-10	◯	◯	◯	◯	◯	◯	◯	◯	◯	◯	◯

* ED 5.3 Community inclusion: Extent to which CSOs are involved in efforts to explore the opportunities to improve the quality of education.

	1	2	3	4	5	6	7	8	9	10	Don't know
1-Lowest -> Highest-10	◯	◯	◯	◯	◯	◯	◯	◯	◯	◯	◯

* ED 5.4 Community Involvement in Education: Extent to which CSOs participate in bringing education information and services to the communities where people live through activities such as supporting the capacity-building of teachers and parents and/or undertaking information and education campaigns to increase the commitment to education in families and communities.

	1	2	3	4	5	6	7	8	9	10	Don't know
1-Lowest -> Highest-10	◯	◯	◯	◯	◯	◯	◯	◯	◯	◯	◯

* ED 5.5 Social Franchising: Extent to which CSOs are involved with social franchising opportunities/services to bring quality education services to children and families at reduced costs.

	1	2	3	4	5	6	7	8	9	10	Don't know
1-Lowest -> Highest-10	◯	◯	◯	◯	◯	◯	◯	◯	◯	◯	◯

* ED 5.6 Technology: Extent to which CSOs are leveraging information communication technology (ICT) to improve access to quality education for various communities.

	1	2	3	4	5	6	7	8	9	10	Don't know
1-Lowest -> Highest-10	◯	◯	◯	◯	◯	◯	◯	◯	◯	◯	◯

* ED 5.7 Human Rights and Access to/Quality of Education: Extent to which CSOs advocate for and support rights-based approaches to education to ensure students experience good treatment, confidence in asking questions, and quality education, amongst other concerns.

	1	2	3	4	5	6	7	8	9	10	Don't know
1-Lowest -> Highest-10	◯	◯	◯	◯	◯	◯	◯	◯	◯	◯	◯

* ED 5.8 Social and Behavior Change (SBC): Extent to which CSOs support SBC interventions relating, but not limited, to life skills and peer education, adult health education, school-based education, community mobilization, and/or mass media interventions for a general public audience.

	1	2	3	4	5	6	7	8	9	10	Don't know
1-Lowest -> Highest-10	◯	◯	◯	◯	◯	◯	◯	◯	◯	◯	◯

* ED 5.9 Advocacy/Accountability: Extent to which CSOs support the training and capacity building of advocates and community members to understand policies, processes, and activities to increase community-led issue identification, collaborative problem solving and more targeted advocacy actions to improve education.

	1	2	3	4	5	6	7	8	9	10	Don't know
1-Lowest -> Highest-10	◯	◯	◯	◯	◯	◯	◯	◯	◯	◯	◯

* ED 5.10 CSO-Led Assessment and Monitoring: Extent to which CSOs assess, monitor and report on the effectiveness of policies and programming to improve accountability in education at the policy and teachers' levels.

	1	2	3	4	5	6	7	8	9	10	Don't know
1-Lowest -> Highest-10	◯	◯	◯	◯	◯	◯	◯	◯	◯	◯	◯

* ED 5.11 Forming Partnerships between CSOs: Extent to which CSOs have formed national alliances and regional partnerships to strengthen their positioning and potentially improve leadership strength and funding mechanisms to improve the quality of education.

	1	2	3	4	5	6	7	8	9	10	Don't know
1-Lowest -> Highest-10	◯	◯	◯	◯	◯	◯	◯	◯	◯	◯	◯

ED 5.12 Comments: Please use this section to provide any additional comments about the level of effort, challenges, or successes in the country around the role of the civil society in ED. If you marked “I don’t know” for any of the questions, also use this section to explain why you gave that rating

JOHNS HOPKINS
BLOOMBERG SCHOOL
of PUBLIC HEALTH

Bill & Melinda Gates Institute for Population and Reproductive Health

Demographic Dividend Effort Index Survey

Module: Sectoral Resilience and Sustainability

Considering the health and socio-economic impact of the COVID-19 and the likely impact on the DD progress, this DD effort index has integrated questions to assess the resilience and sustainability of systems in key sectors of the DD framework. The items included in the COVID-19 related questionnaire below follow the key dimensions of resilient systems and provide critical information on the potential of an effective response to emerging infectious disease threats or other public health emergencies.

Please score each item from 1 to 10, where 1 represents the lowest score (poor state/capability) and 10 represents the highest score (great state/capability).

* Physical: facilities, equipment, system states, and capabilities.

	1	2	3	4	5	6	7	8	9	10	Don't know
ED-M1 - Plan/Prepare - State and capability of equipment, personnel and ED sector structure prior to the crisis event. (1 = poor state/capability; 10 = great state/capability)	○	○	○	○	○	○	○	○	○	○	○
ED-M2 - Absorb - Extent to which COVID-19 was recognized within the ED sector and the performance of the system was able to maintain functionality.	○	○	○	○	○	○	○	○	○	○	○
ED-M3 - Absorb - Integration: The level of integration of ED services and programs to mitigate the effect of COVID19 on ED.	○	○	○	○	○	○	○	○	○	○	○
ED-M4 - Recover - Extent to which the ED sector has been able to induce change to recover the pre-COVID-19 level of functionality.	○	○	○	○	○	○	○	○	○	○	○
ED-M5 - Recover - Financing and Donor Management: Level of resources mobilization and allocation to mitigate the effect of COVID-19 on ED.	○	○	○	○	○	○	○	○	○	○	○
ED-M6 - Adapt - Extent to which changes have been made to improve the resilience of the ED sector.	○	○	○	○	○	○	○	○	○	○	○

* Information: creation, manipulation, storage, and utilization of data.

	1	2	3	4	5	6	7	8	9	10	Don't know
ED-M7 - Plan/Prepare - Extent to which ED-related data was sufficiently prepared, presented, analyzed and stored prior to the crisis event.	○	○	○	○	○	○	○	○	○	○	○
ED-M8 - Absorb - Awareness: Extent of the surveillance capacity to detect the ED limitations/threats posed by COVID-19. (This includes data that is focused on the variety of education-related concerns that the education sector is facing at each level [primary, secondary, tertiary] of schooling).	○	○	○	○	○	○	○	○	○	○	○
ED-M9 - Recover - Extent to which ED data has been used to track the progress of recovery and to anticipate recovery scenarios.	○	○	○	○	○	○	○	○	○	○	○
ED-M10 - Adapt - Extent to which the ED system is creating and improving ED data storage and real-time use protocols.	○	○	○	○	○	○	○	○	○	○	○

* Cognitive: understanding, mental models, preconceptions, biases, and values.

	1	2	3	4	5	6	7	8	9	10	Don't know
ED-M11 - Plan/Prepare - Extent to which the ED system design and operation decisions were prepared in anticipation of crisis events.	○	○	○	○	○	○	○	○	○	○	○
ED-M12 - Absorb - Extent to which the ED system is responding with sufficient contingency protocols and proactive crisis management.	○	○	○	○	○	○	○	○	○	○	○

	1	2	3	4	5	6	7	8	9	10	Don't know
ED-M13 - Absorb - Accountability: Level of effort to ensure optimal accountability for the resources allocated to support ED as part of the response to the COVID-19.	○	○	○	○	○	○	○	○	○	○	○
ED-M14 - Recover - Extent to which decisions are oriented towards recovery, which employ evidence-based communication to community members to promote safe behavior.	○	○	○	○	○	○	○	○	○	○	○
ED-M15 - Recover - Awareness: Extent to which the ED sector is building confidence through communication regarding barriers posed by COVID-19 to support access to and uptake of ED services and programs.	○	○	○	○	○	○	○	○	○	○	○
ED-M16 - Adapt - Extent to which the ED sector is designing new system configurations, objectives and decision criteria.	○	○	○	○	○	○	○	○	○	○	○
ED-M17 - Adapt - Adaptive: Level of adaptation to respond to the COVID-19 threats to ED.	○	○	○	○	○	○	○	○	○	○	○

* Social: interaction, collaboration, and self-synchronization between individuals, entities and institutions.

	1	2	3	4	5	6	7	8	9	10	Don't know
ED-M18 - Plan/Prepare - Extent to which training on outbreak/crisis management was conducted through, and which leveraged, social networks, social capital and institutional and cultural norms prior to the crisis event.	○	○	○	○	○	○	○	○	○	○	○

	1	2	3	4	5	6	7	8	9	10	Don't know
ED-M19 - Absorb - Extent to which personnel and social institutions have been resourceful and accessible for the outbreak/crisis response.	◯	◯	◯	◯	◯	◯	◯	◯	◯	◯	◯
ED-M20 - Absorb - Self-Regulating: Extent to which the national leadership had the authority to make sectoral changes in a timely manner through flexible infrastructure.	◯	◯	◯	◯	◯	◯	◯	◯	◯	◯	◯
ED-M21 - Recover - Extent to which the ED system is engaging with team and knowledge sharing to enhance systematic recovery.	◯	◯	◯	◯	◯	◯	◯	◯	◯	◯	◯
ED-M22 - Recover - Diversity: The level of engagement of a multidisciplinary team to mitigate the effect of COVID-19 on ED.	◯	◯	◯	◯	◯	◯	◯	◯	◯	◯	◯
ED-M23 - Adapt - Extent to which there are additions or changes being made to ED institutions, policies, trainings, programs, and culture.	◯	◯	◯	◯	◯	◯	◯	◯	◯	◯	◯
ED-M24 - Adapt - Leadership and Management: Level of leadership demonstrated by the ED leaders to mitigate the effect of COVID-19 on ED.	◯	◯	◯	◯	◯	◯	◯	◯	◯	◯	◯

* ED M-25 Timeline: Extent of timeliness of the Government reaction - communication and implementation of measures - to mitigate the immediate and longterm impact of COVID-19.
Note: This refers to the entirety of the national reaction - not the reaction that is specific to your sector.

	1	2	3	4	5	6	7	8	9	10	Don't know
1-Lowest -> Highest-10	◯	◯	◯	◯	◯	◯	◯	◯	◯	◯	◯

ED M-26 Comments: Please use this section to provide any additional comments about the resilience and sustainability of the national education systems based on your experience during the COVID-19 pandemics. If you marked “I don’t know” for any of the questions, also use this section to explain why you gave that rating.

JOHNS HOPKINS
BLOOMBERG SCHOOL
of PUBLIC HEALTH

Bill & Melinda Gates Institute for Population and Reproductive Health

Demographic Dividend Effort Index Survey

Women's Empowerment (WE) Questionnaire

Please respond to the following questions based on your experience/expertise within this specific sector.

To give a summary picture of the national demographic dividend effort, please rate the following items. Score each item from 1 to 10, where 1 represents the lowest score (very weak or no effort) and 10 represents the highest score (strong effort). Where applicable, if a policy or activity is non-existent then mark your response as 0.

Answer each item. All responses will be recorded in the format exemplified below:

Lowest Effort	1	2	3	4	5	6	7	8	9	10	Highest Effort	I do not know

JOHNS HOPKINS
BLOOMBERG SCHOOL
of PUBLIC HEALTH

Bill & Melinda Gates Institute for Population and Reproductive Health

Demographic Dividend Effort Index Survey

Dimension 1. Policy/Policy making

* WE 1.1 Gender Equality Policy: Extent to which gender equality is considered/integrated in country development plans (or within plans in your sector).

	1	2	3	4	5	6	7	8	9	10	Don't know
1-Lowest -> Highest-10	○	○	○	○	○	○	○	○	○	○	○

* WE 1.2 Level of commitment to the Convention on the Elimination of all Forms of Discrimination Against Women (CEDAW).

	1	2	3	4	5	6	7	8	9	10	Don't know
1-Lowest -> Highest-10	○	○	○	○	○	○	○	○	○	○	○

* WE 1.3 Equity in Marriage and Family Policies: Extent to which the principle of equality between men and women is incorporated in national constitution and other legislation relating to marriage and family relations.

	1	2	3	4	5	6	7	8	9	10	Don't know
1-Lowest -> Highest-10	○	○	○	○	○	○	○	○	○	○	○

* WE 1.4 Policy on Age at Marriage: Extent to which legal age at marriage for females is set at 18 years or higher and is enforced.

	1	2	3	4	5	6	7	8	9	10	Don't know
1-Lowest -> Highest-10	○	○	○	○	○	○	○	○	○	○	○

* WE 1.5 Violence Against Women Measures: Level of regulations and measures on violence against women, including domestic violence, rape, sexual harassment and trafficking of girls and women.

	1	2	3	4	5	6	7	8	9	10	Don't know
1-Lowest -> Highest-10	○	○	○	○	○	○	○	○	○	○	○

* WE 1.6 Female Genital Cutting/Mutilation: Level of regulation and measures on female genital cutting/mutilation.

	1	2	3	4	5	6	7	8	9	10	Don't know
1-Lowest -> Highest-10	○	○	○	○	○	○	○	○	○	○	○

* WE 1.7 Women's Health Measures: Level of protocols for the protection of women's health, including sexually transmitted infections including HIV/AIDS, maternal mortality and contraception.

	1	2	3	4	5	6	7	8	9	10	Don't know
1-Lowest -> Highest-10	○	○	○	○	○	○	○	○	○	○	○

* WE 1.8 Education Measures: Extent to which there are mechanisms to promote girls' education.

	1	2	3	4	5	6	7	8	9	10	Don't know
WE 1.8.1 Level of enforcement of policies on girl's education.	○	○	○	○	○	○	○	○	○	○	○
WE 1.8.2 Extent to which there are mechanisms in place to prevent girls' school dropouts.	○	○	○	○	○	○	○	○	○	○	○

* WE 1.9 Rights Measures: Level of enforcement of policies on women's rights.

	1	2	3	4	5	6	7	8	9	10	Don't know
1-Lowest -> Highest-10	○	○	○	○	○	○	○	○	○	○	○

* WE 1.10 Equitable Work: Extent to which there are policies on equitable working conditions and remunerations between men and women.

	1	2	3	4	5	6	7	8	9	10	Don't know
1-Lowest -> Highest-10	○	○	○	○	○	○	○	○	○	○	○

* WE 1.11 Equitable Land/Asset Ownership: Extent to which there are policies to affirm equitable access to and ownership of land.

	1	2	3	4	5	6	7	8	9	10	Don't know
1-Lowest -> Highest-10	○	○	○	○	○	○	○	○	○	○	○

* WE 1.12 Conflict, Women & Peacebuilding: Level of implementation of Security UN Resolution 1325 on the impacts of conflict on women and their role in peacebuilding.

	1	2	3	4	5	6	7	8	9	10	Don't know
1-Lowest -> Highest-10	○	○	○	○	○	○	○	○	○	○	○

* WE 1.13 Gender Quotas in Politics: Existence, and level of implementation, of quotas for gender-balanced political systems.

	1	2	3	4	5	6	7	8	9	10	Don't know
WE 1.13.1 Extent to which there is gender equitable decision-making, including support for electoral quotas and gender mainstreaming in all government ministries and departments.	○	○	○	○	○	○	○	○	○	○	○
WE 1.13.2 Extent to which women participate and/or have a significant role in traditional governance systems (e.g. chiefs and village head).	○	○	○	○	○	○	○	○	○	○	○

* WE 1.14 Sustainable Development Goal 5: Level of commitment to fulfilling Sustainable Development Goal 5, to achieve gender equality and empower all women and girls.

	1	2	3	4	5	6	7	8	9	10	Don't know
1-Lowest -> Highest-10	○	○	○	○	○	○	○	○	○	○	○

* WE 1.15 Customary Law Revision: Extent to which customary laws have been reviewed and modified to align with national policies/frameworks. Or to which existing customary laws reflect efforts towards women's empowerment.

	1	2	3	4	5	6	7	8	9	10	Don't know
1-Lowest -> Highest-10	○	○	○	○	○	○	○	○	○	○	○

* WE 1.16 Costing for WE: Extent to which there is sufficient planning and allocating to cover the costs needed to meet targets set in a national gender plan or equivalent national plan.

	1	2	3	4	5	6	7	8	9	10	Don't know
1-Lowest -> Highest-10	○	○	○	○	○	○	○	○	○	○	○

* WE 1.17 Staffing for WE: Extent to which:

	1	2	3	4	5	6	7	8	9	10	Don't know
WE 1.17.1 Sufficient and qualified staff are employed to implement gender-related activities, including efforts towards training said staff	○	○	○	○	○	○	○	○	○	○	○
WE 1.17.2 Efforts are made to appropriately and comprehensively train staff	○	○	○	○	○	○	○	○	○	○	○
WE 1.17.3 Staff have sufficient support to carry out their tasks	○	○	○	○	○	○	○	○	○	○	○
WE 1.17.4 Assigned staff are located at a sufficiently high level in the administration to work effectively	○	○	○	○	○	○	○	○	○	○	○

* WE 1.18 Transparency and Accountability: Extent to which the government provides stakeholders with full information on the intentions, plans, programs and budgets available to implement programs in order to ensure accountability and transparency regarding women's empowerment programs. (*Consider measure such as the passage and implementation of a right to information law, regular information sessions for instance in town halls or the provision of such information in leaflets or other material in the local language*)

	1	2	3	4	5	6	7	8	9	10	Don't know
1-Lowest -> Highest-10	○	○	○	○	○	○	○	○	○	○	○

WE 1.19 Comments: Please use this section to provide any additional comments about the level of effort, challenges, or successes in the country around policymaking for WE. If you marked “I don’t know” for any of the questions, also use this section to explain why you gave that rating.

JOHNS HOPKINS
BLOOMBERG SCHOOL
of PUBLIC HEALTH

Bill & Melinda Gates Institute for Population and Reproductive Health

Demographic Dividend Effort Index Survey

Dimension 2. Services or Programs

* WE 2.1 Gender Mainstreaming: Extent to which services or programs within and across sectors consider implications for women and men of any planned action.

	1	2	3	4	5	6	7	8	9	10	Don't know
1-Lowest -> Highest-10	○	○	○	○	○	○	○	○	○	○	○

* WE 2.2 Gender-Transformative Programming: Extent to which services or programs within and across sectors integrate gender-transformative principles/approaches.

	1	2	3	4	5	6	7	8	9	10	Don't know
1-Lowest -> Highest-10	○	○	○	○	○	○	○	○	○	○	○

* WE 2.3 Education: Extent to which education services or programs focus on girl's and women's empowerment within the classroom.

	1	2	3	4	5	6	7	8	9	10	Don't know
1-Lowest -> Highest-10	○	○	○	○	○	○	○	○	○	○	○

* WE 2.4. Dropouts: Extent to which there are programs to prevent female dropouts.

	1	2	3	4	5	6	7	8	9	10	Don't know
1-Lowest -> Highest-10	○	○	○	○	○	○	○	○	○	○	○

* WE 2.5 Technology Literacy: Extent to which technology literacy is promoted amongst girls in particular.

	1	2	3	4	5	6	7	8	9	10	Don't know
1-Lowest -> Highest-10	○	○	○	○	○	○	○	○	○	○	○

* WE 2.6 Employment: Extent to which job-creation programs and labor market polices aim to empower women economically and is designed/implemented accordingly.

	1	2	3	4	5	6	7	8	9	10	Don't know
1-Lowest -> Highest-10	○	○	○	○	○	○	○	○	○	○	○

* WE 2.7 Women as Mothers and Workers: Extent to which the government makes possible, through laws, regulations and programs, for women to combine the roles of childbearing, breast-feeding and child-rearing with participation in the workforce.

	1	2	3	4	5	6	7	8	9	10	Don't know
1-Lowest -> Highest-10	○	○	○	○	○	○	○	○	○	○	○

* WE 2.8 Family Planning for Empowerment: Extent to which FP services or programs are implemented with the fundamental approach towards empowering women.

	1	2	3	4	5	6	7	8	9	10	Don't know
1-Lowest -> Highest-10	○	○	○	○	○	○	○	○	○	○	○

* WE 2.9 Political and Public Representation: Extent of implementation of programs aiming at improving women's equal participation and equitable representation at all levels of the political process and public life.

	1	2	3	4	5	6	7	8	9	10	Don't know
1-Lowest -> Highest-10	○	○	○	○	○	○	○	○	○	○	○

* WE 2.10 Violence: Extent of fight against violence against women and children and its elimination nationwide (domestic violence, rape, sexual harassment, trafficking women).

	1	2	3	4	5	6	7	8	9	10	Don't know
1-Lowest -> Highest-10	○	○	○	○	○	○	○	○	○	○	○

* WE 2.11 Child Marriage: Extent of law enforcement to eliminate child marriage.

	1	2	3	4	5	6	7	8	9	10	Don't know
1-Lowest -> Highest-10	○	○	○	○	○	○	○	○	○	○	○

* WE 2.12 Female Genital Mutilation/Cutting: Extent of law enforcement to eliminate female genital mutilation/cutting.

	1	2	3	4	5	6	7	8	9	10	Don't know
1-Lowest -> Highest-10	○	○	○	○	○	○	○	○	○	○	○

WE 2.13 Comments: Please use this section to provide any additional comments about the level of effort, challenges, or successes in the country around services or programs for WE. If you marked “I don’t know” for any of the questions, also use this section to explain why you gave that rating

JOHNS HOPKINS
BLOOMBERG SCHOOL
of PUBLIC HEALTH

Bill & Melinda Gates Institute for Population and Reproductive Health

Demographic Dividend Effort Index Survey

Dimension 3. Advocacy

* WE 3.1 Advocates: Extent to which advocates for girls' and women's empowerment exists at the national and sub-national levels.

	1	2	3	4	5	6	7	8	9	10	Don't know
1-Lowest -> Highest-10	◯	◯	◯	◯	◯	◯	◯	◯	◯	◯	◯

* WE 3.2. Girls and Women: Extent to which the advocacy efforts in this area encompass the breadth of concerns in girls' and women's empowerment.

	1	2	3	4	5	6	7	8	9	10	Don't know
1-Lowest -> Highest-10	◯	◯	◯	◯	◯	◯	◯	◯	◯	◯	◯

* WE 3.3 Information Dissemination: Extent to which the government undertakes sufficient efforts to ensure that the population is aware of issues related to WE, and whether the information that is spread is correct and will reach people in a language they understand.

	1	2	3	4	5	6	7	8	9	10	Don't know
1-Lowest -> Highest-10	◯	◯	◯	◯	◯	◯	◯	◯	◯	◯	◯

* WE 3.4 Leveraging Partnerships: Extent to which the government leverages partnerships with CSOs to disseminate WE information through campaigns.

	1	2	3	4	5	6	7	8	9	10	Don't know
1-Lowest -> Highest-10	◯	◯	◯	◯	◯	◯	◯	◯	◯	◯	◯

* WE 3.5 Employment: Extent to which stakeholders call for laws, policies and programs for women to combine the roles of childbearing, breast-feeding and child-rearing with participation in the workforce.

	1	2	3	4	5	6	7	8	9	10	Don't know
1-Lowest -> Highest-10	○	○	○	○	○	○	○	○	○	○	○

* WE 3.6 Education: Extent to which a priority of advocates is to promote the fulfillment of girls' and women's potential through education and skills development.

	1	2	3	4	5	6	7	8	9	10	Don't know
1-Lowest -> Highest-10	○	○	○	○	○	○	○	○	○	○	○

* WE 3.7 Violence: Extent to which advocates successfully engage with the government to react to and address cases of violence or discrimination against women.

	1	2	3	4	5	6	7	8	9	10	Don't know
1-Lowest -> Highest-10	○	○	○	○	○	○	○	○	○	○	○

WE 3.8 Comments: Please use this section to provide any additional comments about the level of effort, challenges, or successes in the country around advocacy for WE. If you marked "I don't know" for any of the questions, also use this section to explain why you gave that rating

JOHNS HOPKINS
BLOOMBERG SCHOOL of PUBLIC HEALTH

Bill & Melinda Gates Institute for Population and Reproductive Health

Demographic Dividend Effort Index Survey

Dimension 4. Research

* WE 4.1 Strength of Observatory/Stakeholder/Technical Working Group for WE: Extent to which country-level WE stakeholders (e.g., stakeholder leadership group, technical working group, WE observatory, country coordination and facilitation group):

	1	2	3	4	5	6	7	8	9	10	Don't know
WE 4.1.1 Have representation in government, training institutions, civil society, nongovernmental and faith-based organizations, professional associations, the private sector, etc.	○	○	○	○	○	○	○	○	○	○	○
WE 4.1.2 Meet regularly, report and recommend policy for senior management within the appropriate ministries	○	○	○	○	○	○	○	○	○	○	○
WE 4.1.3 Make an impact on WE within the country	○	○	○	○	○	○	○	○	○	○	○

* WE 4.2 Women's Rights Research Strategy: Extent to which the national WE plan and/or other national documents include a comprehensive strategy/approach on women's empowerment research, shared among partners.

	1	2	3	4	5	6	7	8	9	10	Don't know
1-Lowest -> Highest-10	○	○	○	○	○	○	○	○	○	○	○

* WE 4.3 Data Gathering Partners:

	1	2	3	4	5	6	7	8	9	10	Don't know
WE 4.3.1 Extent to which research is undertaken by government agencies. (This may include statistical agencies/bureaus and/or ministries of economics/finances, education, health, gender, etc.)	○	○	○	○	○	○	○	○	○	○	○
WE 4.3.2 Extent to which women empowerment research is undertaken by research institutions.	○	○	○	○	○	○	○	○	○	○	○
WE 4.3.3 Extent to which women empowerment research is undertaken by independent researchers.	○	○	○	○	○	○	○	○	○	○	○

* WE 4.4 Topical Data Gathering:

	1	2	3	4	5	6	7	8	9	10	Don't know
WE 4.4.1 Extent to which social and cultural norms affecting women empowerment and gender equality are researched.	○	○	○	○	○	○	○	○	○	○	○
WE 4.4.2 Extent to which health and economic effects of women empowerment are researched.	○	○	○	○	○	○	○	○	○	○	○
WE 4.4.3 Extent to which child marriage is researched to inform policy.	○	○	○	○	○	○	○	○	○	○	○
WE 4.4.4 Extent to which girls' education and skills acquisition outcomes is researched.	○	○	○	○	○	○	○	○	○	○	○

* WE 4.5 Quality/Coverage of Data:

	1	2	3	4	5	6	7	8	9	10	Don't know
WE 4.5.1 Extent to which a routine statistical system provides reliable periodic age-disaggregated data.	○	○	○	○	○	○	○	○	○	○	○
WE 4.5.2 Extent to which the routine statistical system provides good reliable periodic age-disaggregated data on women's empowerment needs among populations in different communities.	○	○	○	○	○	○	○	○	○	○	○
WE 4.5.3 Extent to which evaluations of the monitoring and surveillance systems are conducted and applied to ensure the reliability of age-disaggregated data.	○	○	○	○	○	○	○	○	○	○	○

* WE 4.6 Quality Research Institutions: Extent to which the country supports and sustains research institutions that develop research/collect data that support the breadth and quality of information-gathering to inform national WE efforts.

	1	2	3	4	5	6	7	8	9	10	Don't know
1-Lowest -> Highest-10	○	○	○	○	○	○	○	○	○	○	○

* WE 4.7 Evaluation: Extent to which program statistics, surveys, and small studies are used by specialized staff to report on program operations and measure progress on WE and gender equality.

	1	2	3	4	5	6	7	8	9	10	Don't know
1-Lowest -> Highest-10	○	○	○	○	○	○	○	○	○	○	○

* WE 4.8 Management's Use of Evaluation Findings: Extent to which local-level program managers use research and evaluation findings to improve WE programs in ways suggested by findings.

	1	2	3	4	5	6	7	8	9	10	Don't know
1-Lowest -> Highest-10	○	○	○	○	○	○	○	○	○	○	○

* WE 4.9 Ministerial Use of Evaluation Findings: Extent to which the Ministry administrators systematically utilize data to inform policies and interventions to address women's empowerment-related issues.

	1	2	3	4	5	6	7	8	9	10	Don't know
1-Lowest -> Highest-10	◯	◯	◯	◯	◯	◯	◯	◯	◯	◯	◯

* WE 4.10 Dissemination of Information to other Implementing Bodies: Extent to which information is shared or spread across geographical areas and throughout various levels (national, state/province, localities).

	1	2	3	4	5	6	7	8	9	10	Don't know
1-Lowest -> Highest-10	◯	◯	◯	◯	◯	◯	◯	◯	◯	◯	◯

WE 4.11 Comments: Please use this section to provide any additional comments about the level of effort, challenges, or successes in the country around WE research. If you marked "I don't know" for any of the questions, also use this section to explain why you gave that rating

JOHNS HOPKINS
BLOOMBERG SCHOOL
of PUBLIC HEALTH

Bill & Melinda Gates Institute for Population and Reproductive Health

Demographic Dividend Effort Index Survey

Dimension 5. Civil Society Organizations (CSOs)

* WE 5.1 CSO Actor Power: Extent to which WE civil society actors are in strong position to influence national policy.

	1	2	3	4	5	6	7	8	9	10	Don't know
1-Lowest -> Highest-10	◯	◯	◯	◯	◯	◯	◯	◯	◯	◯	◯

* WE 5.2 Budget Analysis as a CSO Tool: Extent to which CSOs utilize budget analysis as a tool to build WE advocacy cases.

	1	2	3	4	5	6	7	8	9	10	Don't know
1-Lowest -> Highest-10	◯	◯	◯	◯	◯	◯	◯	◯	◯	◯	◯

* WE 5.3 Community-Based Service Delivery Support: Extent to which CSOs participate in bringing women's empowerment information and services to the communities where people live through activities such as supporting the capacity-building of girls, women and women's empowerment champions and/or undertaking information and education campaigns to increase the commitment to women's empowerment in families and communities.

	1	2	3	4	5	6	7	8	9	10	Don't know
1-Lowest -> Highest-10	◯	◯	◯	◯	◯	◯	◯	◯	◯	◯	◯

* WE 5.4 Human Rights and Quality of Implementation: Extent to which CSOs advocate for and support rights-based approaches to women's empowerment to ensure policy and program beneficiaries experience progress in women's empowerment and autonomy on their terms.

	1	2	3	4	5	6	7	8	9	10	Don't know
1-Lowest -> Highest-10	◯	◯	◯	◯	◯	◯	◯	◯	◯	◯	◯

* WE 5.5 Social and Behavior Change (SBC): Extent to which CSOs support SBC interventions relating, but not limited, to gender-transformative programming, life skills and peer education, adult health education, school-based education, couple's communication, community mobilization, working through gatekeepers, and/or mass media interventions for a general public audience. These may be supported through implementation at the community or individual levels.

	1	2	3	4	5	6	7	8	9	10	Don't know
1-Lowest -> Highest-10	◯	◯	◯	◯	◯	◯	◯	◯	◯	◯	◯

* WE 5.6 Youth: Extent to which CSOs promote gender empowerment through youth programs.

	1	2	3	4	5	6	7	8	9	10	Don't know
1-Lowest -> Highest-10	◯	◯	◯	◯	◯	◯	◯	◯	◯	◯	◯

* WE 5.7 Programming for Men: Extent to which CSOs support WE policies, interventions and programs targeting men.

	1	2	3	4	5	6	7	8	9	10	Don't know
1-Lowest -> Highest-10	◯	◯	◯	◯	◯	◯	◯	◯	◯	◯	◯

* WE 5.8 Advocacy/Accountability: Extent to which CSOs support the training and capacity building of advocates and community members to understand policies, processes, and activities to increase community-led WE issues identification, collaborative problem solving and more targeted advocacy actions.

	1	2	3	4	5	6	7	8	9	10	Don't know
1-Lowest -> Highest-10	◯	◯	◯	◯	◯	◯	◯	◯	◯	◯	◯

* WE 5.9 CSO-Led Assessment and Monitoring: Extent to which CSOs assess, monitor and report on the effectiveness of policies and programming to improve accountability at the policy and provider levels.

	1	2	3	4	5	6	7	8	9	10	Don't know
1-Lowest -> Highest-10	◯	◯	◯	◯	◯	◯	◯	◯	◯	◯	◯

* WE 5.10 Forming Partnerships between CSOs: Extent to which CSOs have formed national alliances and regional partnerships to strengthen their positioning and potentially improve leadership strength and funding mechanisms to advance the women empowerment agenda.

	1	2	3	4	5	6	7	8	9	10	Don't know
1-Lowest -> Highest-10	◯	◯	◯	◯	◯	◯	◯	◯	◯	◯	◯

WE 5.11 Comments: Please use this section to provide any additional comments about the level of effort, challenges, or successes around the role of CSOs in WE. If you marked “I don’t know” for any of the questions, also use this section to explain why you gave that rating

JOHNS HOPKINS
BLOOMBERG SCHOOL
of PUBLIC HEALTH

Bill & Melinda Gates Institute for Population and Reproductive Health

Demographic Dividend Effort Index Survey

Module: Sectoral Resilience and Sustainability

Considering the health and socio-economic impact of the COVID-19 and the likely impact on the DD progress, this DD effort index has integrated questions to assess the resilience and sustainability of systems in key sectors of the DD framework. The items included in the COVID-19 related questionnaire below follow the key dimensions of resilient systems and provide critical information on the potential of an effective response to emerging infectious disease threats or other public health emergencies.

Please score each item from 1 to 10, where 1 represents the lowest score (poor state/capability) and 10 represents the highest score (great state/capability).

* Physical: facilities, equipment, system states, and capabilities.

	1	2	3	4	5	6	7	8	9	10	Don't know
WE-M1 - Plan/Prepare - State and capability of equipment, personnel and WE sector structure prior to the crisis event. (1 = poor state/capability; 10 = great state/capability)	◯	◯	◯	◯	◯	◯	◯	◯	◯	◯	◯
WE-M2 - Absorb - Extent to which COVID-19 was recognized within the WE sector and the performance of the system was able to maintain functionality.	◯	◯	◯	◯	◯	◯	◯	◯	◯	◯	◯
WE-M3 - Absorb - Integration: The level of integration of WE services and programs to mitigate the effect of COVID-19 on WE.	◯	◯	◯	◯	◯	◯	◯	◯	◯	◯	◯
WE-M4 - Recover - Extent to which the WE programs have been able to induce change to recover the pre-COVID-19 level of functionality.	◯	◯	◯	◯	◯	◯	◯	◯	◯	◯	◯
WE-M5 - Recover - Financing and Donor Management: Level of resources mobilization and allocation to mitigate the effect of COVID-19 on WE	◯	◯	◯	◯	◯	◯	◯	◯	◯	◯	◯
WE-M6 - Adapt - Extent to which changes have been made to improve resilience in the WE sector.	◯	◯	◯	◯	◯	◯	◯	◯	◯	◯	◯

* Information: creation, manipulation, storage, and utilization of data.

	1	2	3	4	5	6	7	8	9	10	Don't know
WE-M7 - Plan/Prepare - Extent to which WE-related data was sufficiently prepared, presented, analyzed and stored prior to the crisis event.	○	○	○	○	○	○	○	○	○	○	○
WE-M8 - Absorb - Extent to which the WE sector has been able to conduct a real-time assessment of functionality, in anticipation of cascading losses in WE programs and services.	○	○	○	○	○	○	○	○	○	○	○
WE-M9 - Absorb - Awareness: Extent of the surveillance capacity to detect the WE limitations/threats posed by COVID-19.	○	○	○	○	○	○	○	○	○	○	○
WE-M10 - Recover - Extent to which WE data have been used to track the progress of recovery and to anticipate recovery scenarios.	○	○	○	○	○	○	○	○	○	○	○
WE-M11 - Adapt - Extent to which the WE sector is creating and improving WE data storage and real-time use protocols.	○	○	○	○	○	○	○	○	○	○	○

* Cognitive: understanding, mental models, preconceptions, biases, and values.

	1	2	3	4	5	6	7	8	9	10	Don't know
WE-M12 - Plan/Prepare - Extent to which the WE programs design and operation decisions were prepared in anticipation of crisis events.	○	○	○	○	○	○	○	○	○	○	○
WE-M13 - Absorb - Extent to which the WE sector is responding with sufficient contingency protocols and proactive crisis management.	○	○	○	○	○	○	○	○	○	○	○

	1	2	3	4	5	6	7	8	9	10	Don't know
WE-M14 - Absorb - Accountability: Level of effort to ensure optimal accountability for the resources allocated to support WE as part of the response to the COVID-19. (These may take the form of support for services attenuated to the needs of women and girls for protection).	◯	◯	◯	◯	◯	◯	◯	◯	◯	◯	◯
WE-M15 - Recover - Extent to which decisions are oriented towards recovery, which employ evidence-based communication to community members to promote safe behavior.	◯	◯	◯	◯	◯	◯	◯	◯	◯	◯	◯
WE-M16 - Recover - Awareness: Extent to which the WE sector is building confidence through communication regarding barriers posed by COVID-19 to support access to and uptake of WE services and programs. (This includes surveillance that is attenuated to the gendered disparities in COVID-19 responses and/or cases as well as increased surveillance of WE-related issues such as gender-based violence)	◯	◯	◯	◯	◯	◯	◯	◯	◯	◯	◯
WE-M17 - Adapt - Extent to which the WE sector is designing new system configurations, objectives and decision criteria.	◯	◯	◯	◯	◯	◯	◯	◯	◯	◯	◯
WE-M18 - Adapt - Adaptive: Level of adaptation to respond to the COVID-19 threats to WE.	◯	◯	◯	◯	◯	◯	◯	◯	◯	◯	◯

* Social: interaction, collaboration, and self-synchronization between individuals, entities and institutions.

	1	2	3	4	5	6	7	8	9	10	Don't know
WE-M19 - Plan/Prepare - Extent to which training on outbreak/crisis management was conducted through, and which leveraged, social networks, social capital and institutional and cultural norms prior to the crisis.											
WE-M20 - Absorb - Extent to which personnel and social institutions have been resourceful and accessible for the outbreak/crisis response.											
WE-M21 - Absorb - Self-Regulating: Extent to which the national leadership had the authority to make sectoral changes in a timely manner through flexible infrastructure.											
WE-M22 - Recover - Extent to which the WE sector is engaging with team and knowledge sharing to enhance systematic recovery.											
WE-M23 - Recover - Diversity: The level of engagement of a multidisciplinary team to mitigate the effect of COVID-19 on WE.											
WE-M24 - Adapt - Leadership and Management: Level of leadership demonstrated by the leaders in the WE sector and programs to mitigate the effect of COVID-19 on WE.											

* WE M-25 Timeline: Extent of timeliness of the Government reaction - communication and implementation of measures - to mitigate the immediate and longterm impact of COVID-19.

Note: This refers to the entirety of the national reaction - not the reaction that is specific to your sector.

	1	2	3	4	5	6	7	8	9	10	Don't know
1-Lowest -> Highest-10	◯	◯	◯	◯	◯	◯	◯	◯	◯	◯	◯

WE M-26 Comments: Please use this section to provide any additional comments about the resilience of the national women empowerment opportunities and systems based on your experience during the COVID-19 pandemics. If you marked “I don’t know” for any of the questions, also use this section to explain why you gave that rating

JOHNS HOPKINS
BLOOMBERG SCHOOL
of PUBLIC HEALTH

Bill & Melinda Gates Institute for Population and Reproductive Health

Demographic Dividend Effort Index Survey

Labor Market (LM) Questionnaire

Please respond to the following questions based on your experience/expertise within this specific sector.

To give a summary picture of the national demographic dividend effort, please rate the following items. Score each item from 1 to 10, where 1 represents the lowest score (very weak or no effort) and 10 represents the highest score (strong effort). Where applicable, if a policy or activity is non-existent then mark your response as 0.

Answer each item. All responses will be recorded in the format exemplified below:

Lowest effort	1	2	3	4	5	6	7	8	9	10	Highest effort	I do not know

JOHNS HOPKINS
BLOOMBERG SCHOOL of PUBLIC HEALTH

Bill & Melinda Gates Institute for Population and Reproductive Health

Demographic Dividend Effort Index Survey

Dimension 1. Policy/Policy making

* LM 1.1 Global Economic Integration: Extent to which the country aims to be integrated in the global economy.

	1	2	3	4	5	6	7	8	9	10	Don't know
LM 1.1.1 Extent to which the country legislates for trade openness.	○	○	○	○	○	○	○	○	○	○	○
LM 1.1.2 Level of effort towards growing national exports.	○	○	○	○	○	○	○	○	○	○	○

* LM 1.2 Regional Economic Integration: Extent to which the country aims to be integrated in the regional economy.

	1	2	3	4	5	6	7	8	9	10	Don't know
1-Lowest -> Highest-10	○	○	○	○	○	○	○	○	○	○	○

* LM 1.3 Technology: Extent to which the government has invested in infrastructure, both traditional and technological, to provide the basis for the country to leverage opportunities of the future and/or to integrate technology as a complement to labor.

	1	2	3	4	5	6	7	8	9	10	Don't know
1-Lowest -> Highest-10	○	○	○	○	○	○	○	○	○	○	○

* LM 1.4 Government Support: Extent to which policies and plans are in place that provide for social safety nets to smooth transitions between jobs or 'gigs' for individuals and buffer income volatility.

	1	2	3	4	5	6	7	8	9	10	Don't know
LM 1.4.1 Extent to which policies are in place that set a national minimum wage.											
LM 1.4.2 Extent to which policies exist that outline an unemployment insurance system, and the level to which they are effectively implemented.											

* LM 1.5 Unemployment: Extent to which policies account for and address unemployment in various population groups and geographic areas.

	1	2	3	4	5	6	7	8	9	10	Don't know
1-Lowest -> Highest-10											

* LM 1.6 Disadvantaged/Vulnerable Groups: Extent to which policies exist, and are effectively implemented, to support the employment opportunities of disadvantage/vulnerable groups (i.e. girls and women, youth, persons with disabilities, etc.).

	1	2	3	4	5	6	7	8	9	10	Don't know
1-Lowest -> Highest-10											

* LM 1.7 Climate Change and Environmental Sustainability: Extent to which the agricultural and manufacturing sectors are prepared to labor market impacts from climate change effects through planning and strategizing for the future labor market needs.

	1	2	3	4	5	6	7	8	9	10	Don't know
1-Lowest -> Highest-10											

* LM 1.8 Urbanization: Extent to which policies and planning anticipate and are prepared for increasing urbanization as a result of, and contributing to, climate change, and the particular impacts on labor markets.

	1	2	3	4	5	6	7	8	9	10	Don't know
1-Lowest -> Highest-10											

* LM 1.9 Service-Led Economy: Extent to which policies and framework are in place to move economies from informal labor to formalized labor responding to local, regional and global demands for goods and services.

	1	2	3	4	5	6	7	8	9	10	Don't know
1-Lowest -> Highest-10	◯	◯	◯	◯	◯	◯	◯	◯	◯	◯	◯

* LM 1.10 Corporate Social Responsibility: Extent to which national legislation in the labor market address corporate social responsibilities with the aim of supporting youth entrepreneurship.

	1	2	3	4	5	6	7	8	9	10	Don't know
1-Lowest -> Highest-10	◯	◯	◯	◯	◯	◯	◯	◯	◯	◯	◯

* LM 1.11 Targeted Labor Market Investments: Extent to which the government has targeted investments towards sectors with high job-multiplier effects, including Information and Communications Technology (ICT), manufacturing, agriculture and agro-industries in order to generate employment and spur inclusive growth.

	1	2	3	4	5	6	7	8	9	10	Don't know
1-Lowest -> Highest-10	◯	◯	◯	◯	◯	◯	◯	◯	◯	◯	◯

* LM 1.12 Self-Employment: Extent to which policies support raising the returns to self-employment and creating more opportunities to move from self-employment into higher paying wage employment. (This includes rural work).

	1	2	3	4	5	6	7	8	9	10	Don't know
1-Lowest -> Highest-10	◯	◯	◯	◯	◯	◯	◯	◯	◯	◯	◯

* LM 1.13 Microcredit: Extent to which the government enables access to microcredits for profitable activities, securing affordable credit for small and medium-sized enterprises and self-workers.

	1	2	3	4	5	6	7	8	9	10	Don't know
1-Lowest -> Highest-10	◯	◯	◯	◯	◯	◯	◯	◯	◯	◯	◯

* LM 1.14 Savings: Extent to which the government supports the creation of accessible financial products and policies to promote the culture of saving.

	1	2	3	4	5	6	7	8	9	10	Don't know
1-Lowest -> Highest-10	◯	◯	◯	◯	◯	◯	◯	◯	◯	◯	◯

* LM 1.15 Skill Acquisition: Extent to which policies exist, and are effectively implemented, to support the acquisition of currently and future-valued skills for the labor market.

	1	2	3	4	5	6	7	8	9	10	Don't know
1-Lowest -> Highest-10	◯	◯	◯	◯	◯	◯	◯	◯	◯	◯	◯

LM 1.16 Comments: Please use this section to provide any additional comments about the level of effort, challenges, or successes in the country around policies/policymaking for LM. If you marked “I don’t know” for any of the questions, also use this section to explain why you gave that rating

JOHNS HOPKINS
BLOOMBERG SCHOOL
of PUBLIC HEALTH

Bill & Melinda Gates Institute for Population and Reproductive Health

Demographic Dividend Effort Index Survey

Dimension 2. Services or Programs

* LM 2.1 Unemployment Reduction: Extent to which there are efforts toward developing and implementing strategies aimed at the reduction of the proportion of unemployed by target years (in accordance with Agenda 2063 First Ten Year Implementation Plan).

	1	2	3	4	5	6	7	8	9	10	Don't know
1-Lowest -> Highest-10	○	○	○	○	○	○	○	○	○	○	○

* LM 2.2. Youth Unemployment: Extent to which labor market programs have effectively lowered the rate of unemployment, especially among youth.

	1	2	3	4	5	6	7	8	9	10	Don't know
1-Lowest -> Highest-10	○	○	○	○	○	○	○	○	○	○	○

* LM 2.3 Access to Microcredit: Extent to which national and regional funds have been established and operationalized in order to increase people’s access to affordable business capital.

	1	2	3	4	5	6	7	8	9	10	Don't know
1-Lowest -> Highest-10	○	○	○	○	○	○	○	○	○	○	○

* LM 2.4 Harnessing Youth Special Capacities: Extent to which programs and services support the specific talents of youth to aid them in flourishing and achieving their maximum potential. (This includes specific programmatic aims around talent acquisition).

	1	2	3	4	5	6	7	8	9	10	Don't know
1-Lowest -> Highest-10	○	○	○	○	○	○	○	○	○	○	○

* LM 2.5 Early Labor Opportunities: Extent to which programs and government legislation engages with private sector partners to expand internships, apprenticeships and on-the-job training opportunities for women and youth.

	1	2	3	4	5	6	7	8	9	10	Don't know
1-Lowest -> Highest-10	○	○	○	○	○	○	○	○	○	○	○

* LM 2.6 Youth Entrance into Labor: Extent of the promotion of youth volunteer and junior professional programs and other internship opportunities to enhance capacity and grant exposure to youth in regional and international organizations.

	1	2	3	4	5	6	7	8	9	10	Don't know
1-Lowest -> Highest-10	○	○	○	○	○	○	○	○	○	○	○

* LM 2.7 Entrepreneurial Ventures: Extent to which people have enhanced access to government procurement and financial services to reduce the challenge of starting and/or doing business within and across countries.

	1	2	3	4	5	6	7	8	9	10	Don't know
1-Lowest -> Highest-10	○	○	○	○	○	○	○	○	○	○	○

* LM 2.8 Resources for Youth Entrepreneurship: Extent to which entrepreneurial ventures of youth receive direct and targeted resource mobilization, supported by a regulatory framework, to reduce the challenge of starting and/or doing business within and across countries.

	1	2	3	4	5	6	7	8	9	10	Don't know
1-Lowest -> Highest-10	○	○	○	○	○	○	○	○	○	○	○

* LM 2.9 Vocational Opportunities: Extent to which vocational training opportunities are expanded for skills acquisition for people to enhance their employability, productivity and competitiveness.

	1	2	3	4	5	6	7	8	9	10	Don't know
1-Lowest -> Highest-10	○	○	○	○	○	○	○	○	○	○	○

* LM 2.10 Education and Labor: Extent to which improvements have been made to improve inclusive access to education at all levels and to provide viable alternatives for the many young people, particularly adolescent girls, who drop out of the formal education system, by facilitating re-entry, revamping informal education and training through standardized certification within and between countries.

	1	2	3	4	5	6	7	8	9	10	Don't know
1-Lowest -> Highest-10	○	○	○	○	○	○	○	○	○	○	○

* LM 2.11 Women-Friendly Labor: Extent to which workplaces in the private and public sectors have adopted women-friendly policies and practices to eliminate any form of gender-based discrimination while allowing women to comfortably be part of the labor force.

	1	2	3	4	5	6	7	8	9	10	Don't know
1-Lowest -> Highest-10	◯	◯	◯	◯	◯	◯	◯	◯	◯	◯	◯

* LM 2.12 Job Security:

	1	2	3	4	5	6	7	8	9	10	Don't know
LM 2.12.1 Extent to which notice periods for redundancy or dismissal are implemented.	◯	◯	◯	◯	◯	◯	◯	◯	◯	◯	◯
LM 2.12.2 Extent to which severance pay for redundancy or dismissal is implemented.	◯	◯	◯	◯	◯	◯	◯	◯	◯	◯	◯

* LM 2.13 Minimum Wage: Extent to which a minimum wage is applied to the ‘typical’ worker (defined as an average wage-earner, in majority race and religious groups, in largest city, and non-union).

	1	2	3	4	5	6	7	8	9	10	Don't know
1-Lowest -> Highest-10	◯	◯	◯	◯	◯	◯	◯	◯	◯	◯	◯

* LM 2.14 Labor Market Programs: Extent to which labor market programs have effectively diversified the labor force and the needs of the labor market while simultaneously diversifying the skills of the labor force.

	1	2	3	4	5	6	7	8	9	10	Don't know
1-Lowest -> Highest-10	◯	◯	◯	◯	◯	◯	◯	◯	◯	◯	◯

* LM 2.15 Informality in the Labor Market: Extent to which the programs are implemented to transform informality in the labor market into formal labor.

	1	2	3	4	5	6	7	8	9	10	Don't know
1-Lowest -> Highest-10	◯	◯	◯	◯	◯	◯	◯	◯	◯	◯	◯

LM 2.16 Comments: Please use this section to provide any additional comments about the level of effort, challenges, or successes in the country around services or programs for LM. If you marked “I don’t know” for any of the questions, also use this section to explain why you gave that rating.

JOHNS HOPKINS BLOOMBERG SCHOOL of PUBLIC HEALTH

Bill & Melinda Gates Institute for Population and Reproductive Health

Demographic Dividend Effort Index Survey

Dimension 3. Advocacy

* LM 3.1 Gender Equality in the Labor Market: Extent to which groups and individuals call for, and see improvements in, gender equality in the workforce.

	1	2	3	4	5	6	7	8	9	10	Don't know
1-Lowest -> Highest-10	○	○	○	○	○	○	○	○	○	○	○

* LM 3.2 Youth Equality in the Labor Market: Extent to which individuals and groups call for shifts in the labor market and labor market regulations to benefit all rural and urban youth populations.

	1	2	3	4	5	6	7	8	9	10	Don't know
1-Lowest -> Highest-10	○	○	○	○	○	○	○	○	○	○	○

* LM 3.3 Minimum Wage Advocacy: Extent to which advocacy groups are successful in inciting legislation for a minimum wage, or a rise in the minimum wage if one already exists.

	1	2	3	4	5	6	7	8	9	10	Don't know
1-Lowest -> Highest-10	○	○	○	○	○	○	○	○	○	○	○

* LM 3.4 Government Advocacy: Extent to which members of the government advocates for improvements within its own structures and priorities. (These improvements may include labor market-focused progress for youth inclusion, opportunities, programming and rights among similar concepts across the population).

	1	2	3	4	5	6	7	8	9	10	Don't know
1-Lowest -> Highest-10	○	○	○	○	○	○	○	○	○	○	○

* LM 3.5 Protections: Extent to which advocacy exists for job protection, and social protection in the absence of jobs.

	1	2	3	4	5	6	7	8	9	10	Don't know
1-Lowest -> Highest-10	○	○	○	○	○	○	○	○	○	○	○

LM 3.6 Comments: Please use this section to provide any additional comments about the level of effort, challenges, or successes in advocacy work related to LM. If you marked “I don’t know” for any of the questions, also use this section to explain why you gave that rating.

JOHNS HOPKINS
BLOOMBERG SCHOOL
of PUBLIC HEALTH

Bill & Melinda Gates Institute for Population and Reproductive Health

Demographic Dividend Effort Index Survey

Dimension 4. Research

* LM 4.1 Strength of Observatory/Stakeholder/Technical Working Group for the LM: Extent to which country-level LM stakeholders (e.g., stakeholder leadership group, technical working group, LM observatory, country coordination and facilitation group):

	1	2	3	4	5	6	7	8	9	10	Don't know
LM 4.1.1 Have representation in government, training institutions, civil society, nongovernmental and faith-based organizations, professional associations, the private sector, etc.	○	○	○	○	○	○	○	○	○	○	○
LM 4.1.2 Meet regularly, report and recommend policy for senior management within the appropriate ministries	○	○	○	○	○	○	○	○	○	○	○
LM 4.1.3 Make an impact on LM within the country	○	○	○	○	○	○	○	○	○	○	○

* LM 4.2 Labor Market Research Strategy: Extent to which the national documents include a comprehensive strategy/approach on labor market-related research and is shared among partners.

	1	2	3	4	5	6	7	8	9	10	Don't know
1-Lowest -> Highest-10	○	○	○	○	○	○	○	○	○	○	○

* LM 4.3 Labor Market Analysis Partners:

	1	2	3	4	5	6	7	8	9	10	Don't know
LM 4.3.1 Extent to which labor market research is undertaken by government agencies.	◯	◯	◯	◯	◯	◯	◯	◯	◯	◯	◯
LM 4.3.2 Extent to which labor market research is undertaken by research institutions.	◯	◯	◯	◯	◯	◯	◯	◯	◯	◯	◯
LM 4.3.3 Extent to which labor market research is undertaken by independent researchers.	◯	◯	◯	◯	◯	◯	◯	◯	◯	◯	◯

* LM 4.4 Areas of Research in the Labor Market:

	1	2	3	4	5	6	7	8	9	10	Don't know
LM 4.4.1 Extent to which unemployment trends and patterns are researched.	◯	◯	◯	◯	◯	◯	◯	◯	◯	◯	◯
LM 4.4.2 Extent of research on youth in the labor market.	◯	◯	◯	◯	◯	◯	◯	◯	◯	◯	◯
LM 4.4.3 Extent of research on women in the labor market.	◯	◯	◯	◯	◯	◯	◯	◯	◯	◯	◯
LM 4.4.4 Extent of research on the trends and trajectories of the labor market needs.	◯	◯	◯	◯	◯	◯	◯	◯	◯	◯	◯

* LM 4.5 Quality/Coverage of Data:

	1	2	3	4	5	6	7	8	9	10	Don't know
LM 4.5.1 Extent to which a routine statistical system provides reliable periodic labor market data.	○	○	○	○	○	○	○	○	○	○	○
LM 4.5.2 Extent to which the routine statistical system provides reliable periodic information on LM needs among populations in different communities.	○	○	○	○	○	○	○	○	○	○	○
LM 4.5.3 Extent to which evaluations of the monitoring and surveillance systems are conducted and applied to ensure the reliability of data.	○	○	○	○	○	○	○	○	○	○	○

* LM 4.6 Record-Keeping: Extent to which systems for labor market recordkeeping, reporting and feedback of results are adequate.

	1	2	3	4	5	6	7	8	9	10	Don't know
1-Lowest -> Highest-10	○	○	○	○	○	○	○	○	○	○	○

* LM 4.7 Quality Research Institutions: Extent to which the country has the capacity to sustain and maintain research institutions that develop research/collect labor market-related data.

	1	2	3	4	5	6	7	8	9	10	Don't know
1-Lowest -> Highest-10	○	○	○	○	○	○	○	○	○	○	○

* LM 4.8 Evaluation: Extent to which program statistics, surveys, and small studies are used by specialized staff to report on program operations and measure progress.

	1	2	3	4	5	6	7	8	9	10	Don't know
1-Lowest -> Highest-10	○	○	○	○	○	○	○	○	○	○	○

* LM 4.9 Management's Use of Evaluation Findings: Extent to which local-level program managers use research and evaluation findings to improve LM related services and programs in ways suggested by findings.

	1	2	3	4	5	6	7	8	9	10	Don't know
1-Lowest -> Highest-10	○	○	○	○	○	○	○	○	○	○	○

* LM 4.10 Ministerial Use of Evaluation Findings: Extent to which the Ministry administrators systematically utilize data to inform policies and interventions to address LM-related issues.

	1	2	3	4	5	6	7	8	9	10	Don't know
1-Lowest -> Highest-10	◯	◯	◯	◯	◯	◯	◯	◯	◯	◯	◯

* LM 4.11 Dissemination of Information to Other Implementing Bodies: Extent to which information is shared or spread across geographical areas and throughout various levels (national, state/province, localities).

	1	2	3	4	5	6	7	8	9	10	Don't know
1-Lowest -> Highest-10	◯	◯	◯	◯	◯	◯	◯	◯	◯	◯	◯

LM 4.12 Comments: Please use this section to provide any additional comments about the level of effort, challenges, or successes in the country around LM research. If you marked “I don’t know” for any of the questions, also use this section to explain why you gave that rating.

Bill & Melinda Gates Institute for Population and Reproductive Health

Demographic Dividend Effort Index Survey

Dimension 5. Civil Society Organizations (CSOs)

* LM 5.1 CSO Actor Power: Extent to which CSO actors are in strong positions to influence policies and action on the LM.

	1	2	3	4	5	6	7	8	9	10	Don't know
1-Lowest -> Highest-10	◯	◯	◯	◯	◯	◯	◯	◯	◯	◯	◯

* LM 5.2 Budget Analysis as a CSO Tool: Extent to which CSOs utilize budget analysis as a tool to build an advocacy case for labor market.

	1	2	3	4	5	6	7	8	9	10	Don't know
1-Lowest -> Highest-10	◯	◯	◯	◯	◯	◯	◯	◯	◯	◯	◯

* LM 5.3 Community-Based Service Delivery Support: Extent to which CSOs participate in bringing LM needs, information and services to the policy makers and vice-versa.

	1	2	3	4	5	6	7	8	9	10	Don't know
1-Lowest -> Highest-10	◯	◯	◯	◯	◯	◯	◯	◯	◯	◯	◯

* LM 5.4 Diversification of Job Opportunities: Extent to which CSOs and the private sector coordinate with the government to ensure optimal job creation and equal participation of women and men.

	1	2	3	4	5	6	7	8	9	10	Don't know
1-Lowest -> Highest-10	◯	◯	◯	◯	◯	◯	◯	◯	◯	◯	◯

* LM 5.5 Human Rights and Quality of Implementation: Extent to which CSOs support rights-based approaches at workplaces.

	1	2	3	4	5	6	7	8	9	10	Don't know
1-Lowest -> Highest-10	◯	◯	◯	◯	◯	◯	◯	◯	◯	◯	◯

* LM 5.6 Youth: Extent to which CSOs support labor market policies, interventions and programming specifically targeting youth.

	1	2	3	4	5	6	7	8	9	10	Don't know
1-Lowest -> Highest-10											

* LM 5.7 Programming for Women: Extent to which CSOs support labor market policies, services and programs for women.

	1	2	3	4	5	6	7	8	9	10	Don't know
1-Lowest -> Highest-10											

* LM 5.8 Advocacy/Accountability: Extent to which CSOs support the training and capacity building of advocates and community members to understand policies, processes, and activities to increase community-led issue identification, collaborative problem solving and more targeted advocacy actions to address unemployment.

	1	2	3	4	5	6	7	8	9	10	Don't know
1-Lowest -> Highest-10											

* LM 5.9 CSO-Led Assessment and Monitoring: Extent to which CSOs assess, monitor and report on the effectiveness of policies and programming to improve accountability at the policy levels.

	1	2	3	4	5	6	7	8	9	10	Don't know
1-Lowest -> Highest-10											

* LM 5.10 Forming Partnerships between CSOs: Extent to which the private sector has formed national alliances and regional partnerships to strengthen the labor market.

	1	2	3	4	5	6	7	8	9	10	Don't know
1-Lowest -> Highest-10											

LM 5.11 Comments: Please use this section to provide any additional comments about the level of effort, challenges, or successes on the role of the CSO in the LM. If you marked “I don’t know” for any of the questions, also use this section to explain why you gave that rating

JOHNS HOPKINS
BLOOMBERG SCHOOL
of PUBLIC HEALTH

Bill & Melinda Gates Institute for Population and Reproductive Health

Demographic Dividend Effort Index Survey

Module: Sectoral Resilience and Sustainability

Considering the health and socio-economic impact of the COVID-19 and the likely impact on the DD progress, this DD effort index has integrated questions to assess the resilience and sustainability of systems in key sectors of the DD framework. The items included in the COVID-19 related questionnaire below follow the key dimensions of resilient systems and provide critical information on the potential of an effective response to emerging infectious disease threats or other public health emergencies.

Please score each item from 1 to 10, where 1 represents the lowest score (poor state/capability) and 10 represents the highest score (great state/capability).

* Physical: facilities, equipment, system states, and capabilities.

	1	2	3	4	5	6	7	8	9	10	Don't know
LM-M1 - Plan/Prepare - State and capability of equipment, personnel and LM sector structure prior to the crisis event. (1 = poor state/capability; 10 = great state/capability)	○	○	○	○	○	○	○	○	○	○	○
LM-M2 - Absorb - Extent to which COVID-19 was recognized within the LM sector and the performance of the system was able to maintain optimal employment and economic productivity.	○	○	○	○	○	○	○	○	○	○	○
LM-M3 - Recover - Extent to which the LM sector has been able to induce change to recover the pre-COVID-19 level of functionality.	○	○	○	○	○	○	○	○	○	○	○

	1	2	3	4	5	6	7	8	9	10	Don't know
LM-M4 - Recover - Financing and Donor Management: Extent to which there are established social protection mechanisms (and other forms of income smoothing) for unprotected workers (e.g. self-employed workers, that have no access to paid or sick leave).	○	○	○	○	○	○	○	○	○	○	○
LM-M5 - Adapt - Financing and Donor Management: Extent to which there has been institutional and policy reforms to build resilience by a demand-led approach through large-scale social protection systems that can act as economic stabilizers	○	○	○	○	○	○	○	○	○	○	○

* Information: creation, manipulation, storage, and utilization of data.

	1	2	3	4	5	6	7	8	9	10	Don't know
LM-M6 - Plan/Prepare - Extent to which LM-related data was sufficiently prepared, presented, analyzed and stored prior to the crisis event.	○	○	○	○	○	○	○	○	○	○	○
LM-M7 - Absorb - Extent to which the LM sector has been able to conduct a real-time assessment of functionality, in anticipation of cascading losses in LM and productivity.	○	○	○	○	○	○	○	○	○	○	○
LM-M8 - Recover - Extent to which LM data has been used to track the progress of recovery and to anticipate recovery scenarios.	○	○	○	○	○	○	○	○	○	○	○
LM-M9 - Adapt - Extent to which the LM sector is creating and improving LM data storage and real-time use protocols.	○	○	○	○	○	○	○	○	○	○	○

* Cognitive: understanding, mental models, preconceptions, biases, and values.

	1	2	3	4	5	6	7	8	9	10	Don't know
LM-M10 - Plan/Prepare - Extent to which the LM sector design and operation decisions were prepared in anticipation of crisis events.	○	○	○	○	○	○	○	○	○	○	○
LM-M11 - Absorb - Extent to which the LM sector is responding with sufficient contingency protocols and proactive crisis management.	○	○	○	○	○	○	○	○	○	○	○

	1	2	3	4	5	6	7	8	9	10	Don't know
LM-M12 - Absorb - Leadership and Management: Extent to which the government is ensuring workers and employers and their families are protected from the health risks of COVID-19.	○	○	○	○	○	○	○	○	○	○	○
LM-M13 - Absorb - Accountability: Level of effort to ensure optimal accountability for the resources allocated to ensure protection from the health risks of COVID-19 and the recovery of the labor market as part of the response to mitigate COVID-19 impacts.	○	○	○	○	○	○	○	○	○	○	○
LM-M14 - Recover - Extent to which decisions are oriented towards recovery, which employ evidence-based communication to community members to promote safe behavior.	○	○	○	○	○	○	○	○	○	○	○
LM-M15 - Recover - Awareness: Extent to which the government is building confidence through trust and dialogue for building the commitment of employers and workers to the joint action of the government and promoting enterprise-level social dialogue.	○	○	○	○	○	○	○	○	○	○	○
LM-M16 - Adapt - Extent to which the LM sector is designing new system configurations, objectives and decision criteria.	○	○	○	○	○	○	○	○	○	○	○

	1	2	3	4	5	6	7	8	9	10	Don't know
LM-M17 - Adapt - Adaptive: Extent to which the government is promoting change in the workspace to protect workers (e.g. occupational and safety health measures, adapted work arrangements, prevention of discrimination and exclusion, expanded access to paid leave, etc.).	○	○	○	○	○	○	○	○	○	○	○

* Social: interaction, collaboration, and self-synchronization between individuals, entities and institutions.

	1	2	3	4	5	6	7	8	9	10	Don't know
LM-M18 - Plan/Prepare - Extent to which training on outbreak/crisis management was conducted through, and which leveraged, social networks, social capital and institutional and cultural norms prior to the crisis event.	○	○	○	○	○	○	○	○	○	○	○
LM-M19 - Absorb - Extent to which personnel and social institutions have been resourceful and accessible for the outbreak/crisis response.	○	○	○	○	○	○	○	○	○	○	○
LM-M20 - Absorb - Self-Regulating: Extent to which the national leadership had the authority to make sectoral changes in a timely manner through flexible labor market infrastructure.	○	○	○	○	○	○	○	○	○	○	○
LM-M21 - Recover - Extent to which the LM sector is engaging with team and knowledge sharing to enhance systematic recovery.	○	○	○	○	○	○	○	○	○	○	○

	1	2	3	4	5	6	7	8	9	10	Don't know
LM-M22 - Recover - Diversity: Extent to which the government is facilitating a strong and fast recovery of the labor market by diversifying policies that favor both the supply (production of good and services) and demand (consumption and investment).	○	○	○	○	○	○	○	○	○	○	○
LM-M23 - Adapt - Extent to which there are additions or changes being made to LM institutions, policies, trainings, programs and culture.	○	○	○	○	○	○	○	○	○	○	○
LM-M24 - Adapt - Leadership and Management: Extent to which there has been established a tripartite fluent dialogue between government, workers, and employers' organizations.	○	○	○	○	○	○	○	○	○	○	○

* LM M-25 Timeline: Extent of timeliness of the Government reaction - communication and implementation of measures - to mitigate the immediate and longterm impact of COVID-19.
Note: This refers to the entirety of the national reaction - not the reaction that is specific to your sector.

	1	2	3	4	5	6	7	8	9	10	Don't know
1-Lowest -> Highest-10	○	○	○	○	○	○	○	○	○	○	○

LM M-26 Comments: Please use this section to provide any additional comments about the resilience and sustainability of the LM systems based on your experience during the COVID-19 pandemics. If you marked "I don't know" for any of the questions, also use this section to explain why you gave that rating.

JOHNS HOPKINS
BLOOMBERG SCHOOL
of PUBLIC HEALTH

Bill & Melinda Gates Institute for Population and Reproductive Health

Demographic Dividend Effort Index Survey

Governance and Economic Institutions (GEI) Questionnaire

Please respond to the following questions based on your experience/expertise within this specific sector.

To give a summary picture of the national demographic dividend effort, please rate the following items. Score each item from 1 to 10, where 1 represents the lowest score (very weak or no effort) and 10 represents the highest score (strong effort). Where applicable, if a policy or activity is non-existent then mark your response as 0.

Answer each item. All responses will be recorded in the format exemplified below:

Lowest effort	1	2	3	4	5	6	7	8	9	10	Highest effort	I do not know

JOHNS HOPKINS
BLOOMBERG SCHOOL
of PUBLIC HEALTH

Bill & Melinda Gates Institute for Population and Reproductive Health

Demographic Dividend Effort Index Survey

Dimension 1. Policies

* GEI 1.1 Democratic Values: Extent to which the government has ratified, domesticated and implemented regional instruments for shared values, including those relating to democracy, elections and governance and the right to health.

	1	2	3	4	5	6	7	8	9	10	Don't know
1-Lowest -> Highest-10	○	○	○	○	○	○	○	○	○	○	○

* GEI 1.2 Monitoring Democratic Values: Extent to which the government has created mechanisms for regular and periodic reporting on the implementation of the regional instruments on shared values, through existing mechanisms such as universal periodic review on the status of international or regional instruments or using independent bodies. (This may take the form of regular reports on the status of implementation).

	1	2	3	4	5	6	7	8	9	10	Don't know
1-Lowest -> Highest-10	○	○	○	○	○	○	○	○	○	○	○

* GEI 1.3 Affirmation of Youth Democratic Participation: Extent to which the government has explicitly removed all discriminatory laws and limitations to full participation of young people in electoral processes through inclusive electoral laws and constitutions. (This may include developing and/or strengthening youth leadership programs and/or providing capacity building on management and leadership skills).

	1	2	3	4	5	6	7	8	9	10	Don't know
1-Lowest -> Highest-10	○	○	○	○	○	○	○	○	○	○	○

* GEI 1.4. Removing Legal Youth Barriers: Extent to which the government has proscribed all laws posing barriers to the full exercise and enjoyment of the fundamental rights of young people to fully participate in the democratic governance processes at the continental, regional, national and grassroots levels. (This may also include sustaining the mainstreaming of adolescents and youth health and rights issues into the country's growth and transformation agenda).

	1	2	3	4	5	6	7	8	9	10	Don't know
1-Lowest -> Highest-10	○	○	○	○	○	○	○	○	○	○	○

* GEI 1.5 Youth Policy: Extent to which the youth policy takes into account the latest thinking in youth education, training and employment.

	1	2	3	4	5	6	7	8	9	10	Don't know
1-Lowest -> Highest-10	○	○	○	○	○	○	○	○	○	○	○

* GEI 1.6 Affirmation of Democratic Participation/Revision of Customary Law: Extent to which the government has reviewed, revised, amended or abolished all laws, regulations, policies, practices and customs that have a discriminatory impact on youth, especially girls and young women, without distinction of any kind, and ensure that the provisions of multiple legal systems comply with international human rights regulations and laws. (These must include protection from harmful practices like early, forced or child marriages, sexual and gender-based violence, female genital mutilation (FGM).

	1	2	3	4	5	6	7	8	9	10	Don't know
1-Lowest -> Highest-10	○	○	○	○	○	○	○	○	○	○	○

* GEI 1.7 Youth Policymaking Participation: Extent to which the government ensures the involvement of youth leadership and participation in decision-making processes in efforts relating to policymaking including youth and education policy making. Potentially includes efforts to establish a national youth parliament and assembly. (This may include supporting adolescent and youth participation and leadership in health planning and programming at all levels).

	1	2	3	4	5	6	7	8	9	10	Don't know
1-Lowest -> Highest-10	○	○	○	○	○	○	○	○	○	○	○

* GEI 1.8 Rule of Law: Extent to which the rule of law is formally, legally established to be equitable and implemented independently by the judiciary.

	1	2	3	4	5	6	7	8	9	10	Don't know
1-Lowest -> Highest-10	○	○	○	○	○	○	○	○	○	○	○

* GEI 1.9 Transparency and Accountability: Extent to which national legislation mandates transparency and accountability across all branches and throughout all sectors.

	1	2	3	4	5	6	7	8	9	10	Don't know
GEI 1.9.1 Extent to which the public can access public and legislative information as well as the records of state-owned organizations.	◯	◯	◯	◯	◯	◯	◯	◯	◯	◯	◯
GEI 1.9.2 Extent to which there are mandated actions and mechanisms for accountability and sanctioning.	◯	◯	◯	◯	◯	◯	◯	◯	◯	◯	◯

* GEI 1.10 National Security: Extent to which the country/government is involved in armed conflict, either internationally or domestically.

	1	2	3	4	5	6	7	8	9	10	Don't know
GEI 1.10.1 Extent to which the country has cross-border tensions.	◯	◯	◯	◯	◯	◯	◯	◯	◯	◯	◯
GEI 1.10.2 Extent to which the country contains internally displaced persons and/or refugees.	◯	◯	◯	◯	◯	◯	◯	◯	◯	◯	◯
GEI 1.10.3 Extent to which education facilities are protected and alternative learning opportunities are created for children and youth in time of conflict or displacement.	◯	◯	◯	◯	◯	◯	◯	◯	◯	◯	◯

* GEI 1.11 Rights: Extent to which the constitution affirms certain rights: freedom of expression, freedom of association and assembly, civil rights and liberties, and protection against ethnic and religious discrimination, health as well as an un-likelihood of human rights abuses by the government.

	1	2	3	4	5	6	7	8	9	10	Don't know
1-Lowest -> Highest-10	◯	◯	◯	◯	◯	◯	◯	◯	◯	◯	◯

* GEI 1.12 Education and Democratic Values: Extent to which education policies, strategies and programs promote academic freedom, democratic values and education as human right.

	1	2	3	4	5	6	7	8	9	10	Don't know
1-Lowest -> Highest-10	○	○	○	○	○	○	○	○	○	○	○

* GEI 1.13 Affirmation of Safe Learning Environments: Extent to which education policies provide for safe learning environments through protections for children. (This may come in the form of ensuring the safety of girls, banning corporal punishment, etc.).

	1	2	3	4	5	6	7	8	9	10	Don't know
1-Lowest -> Highest-10	○	○	○	○	○	○	○	○	○	○	○

GEI 1.14 Comments: Please use this section to provide any additional comments about the level of effort, challenges, or successes in the country around policies/policymaking for GEI. If you marked “I don’t know” for any of the questions, also use this section to explain why you gave that rating

JOHNS HOPKINS
BLOOMBERG SCHOOL
of PUBLIC HEALTH

Bill & Melinda Gates Institute for Population and Reproductive Health

Demographic Dividend Effort Index Survey

Dimension 2. Services or Programs

* GEI 2.1 Strengthening Youth Activities/Institutions: Extent to which efforts are being made to strengthen independent youth formations, networks and organizations including the establishment of independent youth commissions, student unions at the national and sub-regional levels to champion youth rights.

	1	2	3	4	5	6	7	8	9	10	Don't know
1-Lowest -> Highest-10	○	○	○	○	○	○	○	○	○	○	○

* GEI 2.2 Appointment of Youth Leader: Whether and to what extent there has been a youth envoy appointed to lead advocacy and champion the prioritization of youth issues (e.g. education, training and employment) within decision-making spaces at a variety of levels including the parliament.

	1	2	3	4	5	6	7	8	9	10	Don't know
1-Lowest -> Highest-10	○	○	○	○	○	○	○	○	○	○	○

* GEI 2.3 Youth Ideals and Aspirations: Extent to which programming is designed to institute youth leadership and empowerment trainings aimed at deepening cultural ideals and aspirations in the youth.

	1	2	3	4	5	6	7	8	9	10	Don't know
1-Lowest -> Highest-10	○	○	○	○	○	○	○	○	○	○	○

* GEI 2.4 Youth Services: Extent to which programming is designed to address youth-specific needs. (This should consider the development and implementation of adolescent and youth-friendly health services, among other similar programmatic endeavors).

	1	2	3	4	5	6	7	8	9	10	Don't know
1-Lowest -> Highest-10	○	○	○	○	○	○	○	○	○	○	○

* GEI 2.5 Youth Involvement in National Initiatives: Extent to which there are efforts to strengthen existing youth engagement and participation initiatives of the country *(e.g. at the level of the African Union these may include the African Youth Volunteers Corps, African Union Youth Clubs, AGA-YES activities including the Youth Advisory Panel, the Moot Court and Annual Youth Dialogue on Democracy, Human Rights and Governance*). (This may include engaging adolescents and youth in the provision of technical support to sector ministries and relevant federal and regional government bodies for developing policies, plans, and programs that integrate responses to priority health issues affecting this group).

	1	2	3	4	5	6	7	8	9	10	Don't know
1-Lowest -> Highest-10	◯	◯	◯	◯	◯	◯	◯	◯	◯	◯	◯

* GEI 2.6 Personal Safety: Extent to which individual safety is provided for through equitable, reliable police services/activities, contributing to an overall positive perception of personal safety at the household and community levels as well as the school environment. (This may include protecting youth form any form of abuse, neglect or disrespect in the use of health services).

	1	2	3	4	5	6	7	8	9	10	Don't know
1-Lowest -> Highest-10	◯	◯	◯	◯	◯	◯	◯	◯	◯	◯	◯

* GEI 2.7 Participation: Extent to which government functions involve the participation of citizens, civil society organizations, and other intersectoral actors to promote democratic elections and the effective power to govern.

	1	2	3	4	5	6	7	8	9	10	Don't know
1-Lowest -> Highest-10	◯	◯	◯	◯	◯	◯	◯	◯	◯	◯	◯

* GEI 2.8 Gender: Extent to which women participate in:

	1	2	3	4	5	6	7	8	9	10	Don't know
GEI 2.8.1 Governance at the national level	◯	◯	◯	◯	◯	◯	◯	◯	◯	◯	◯
GEI 2.8.2 Governance at the sub-national level	◯	◯	◯	◯	◯	◯	◯	◯	◯	◯	◯
GEI 2.8.3 Governance in rural areas	◯	◯	◯	◯	◯	◯	◯	◯	◯	◯	◯
GEI 2.8.4 Governance in urban areas	◯	◯	◯	◯	◯	◯	◯	◯	◯	◯	◯
GEI 2.8.5 Judiciary	◯	◯	◯	◯	◯	◯	◯	◯	◯	◯	◯

* GEI 2.9 Promotion of Equality: Extent to which gender equality in all aspects of society is promoted by the government. (This may include equality in education, equality before law, equality in healthcare, equality in employment and renumeration, among many other facets of society).

	1	2	3	4	5	6	7	8	9	10	Don't know
1-Lowest -> Highest-10	○	○	○	○	○	○	○	○	○	○	○

* GEI 2.10 Sustainable Economic Opportunity:

	1	2	3	4	5	6	7	8	9	10	Don't know
GEI 2.10.1 Extent to which the government provides for and ensures that a strong public management system is realized.	○	○	○	○	○	○	○	○	○	○	○
GEI 2.10.2 Extent to which the government provides for and ensures that a strong business environment is realized.	○	○	○	○	○	○	○	○	○	○	○
GEI 2.10.3 Extent to which the government provides for and ensures that a strong public development infrastructure is realized.	○	○	○	○	○	○	○	○	○	○	○
GEI 2.10.4 Extent to which the government provides for and ensures that a strong rural sector for development is realized.	○	○	○	○	○	○	○	○	○	○	○

* GEI 2.11 Human Development:

	1	2	3	4	5	6	7	8	9	10	Don't know
GEI 2.11.1 Extent to which the welfare of the general public is well-handled by the government (consider aspects related, but not limited, to welfare policies and services, socioeconomic integration of youth and social protections and safety nets, among many other areas). This may also that the government, with the obligations to protect, fulfil and respect basic health rights, has given the responsibility of its implementation to its ministerial cabinet.	◯	◯	◯	◯	◯	◯	◯	◯	◯	◯	◯
GEI 2.11.2 Extent to which the government is promoting the skills development of the population at workplaces.	◯	◯	◯	◯	◯	◯	◯	◯	◯	◯	◯
GEI 2.11.3 Extent to which the government is promoting the education, knowledge and capacities of the population through schools and continuing education opportunities.	◯	◯	◯	◯	◯	◯	◯	◯	◯	◯	◯
GEI 2.11.4 Extent to which the government is promoting the health and wellness of the population.	◯	◯	◯	◯	◯	◯	◯	◯	◯	◯	◯

GEI 2.12 Comments: Please use this section to provide any additional comments about the level of effort, challenges, or successes in the country around services or programs for GEI. If you marked “I don’t know” for any of the questions, also use this section to explain why you gave that rating.

JOHNS HOPKINS
BLOOMBERG SCHOOL
of PUBLIC HEALTH

Bill & Melinda Gates Institute for Population and Reproductive Health

Demographic Dividend Effort Index Survey

Dimension 3. Advocacy

* GEI 3.1 Youth Empowerment: Extent to which youth empowerment has been enhanced, potentially through the integration of civic education in national educational curricula, media platforms and other channels to instill principles of the rule of law, human rights and individual duties and responsibilities. (This may include the mainstreaming of adolescents and youth health and rights issues into the country’s growth and transformation agenda and educational curricula).

	1	2	3	4	5	6	7	8	9	10	Don't know
1-Lowest -> Highest-10	○	○	○	○	○	○	○	○	○	○	○

* GEI 3.2 Government Advocacy: Extent to which members of the government advocates for improvements within its own structures and priorities. (These improvements may include progress for youth inclusion, opportunities, programming and rights among similar concepts across the population).

	1	2	3	4	5	6	7	8	9	10	Don't know
1-Lowest -> Highest-10	○	○	○	○	○	○	○	○	○	○	○

* GEI 3.3 Non-governmental Advocacy: Extent to which non-governmental actors advocates for improvements in government structures and priorities. (These improvements may include progress for youth inclusion, opportunities, programming and rights among similar concepts across the population).

	1	2	3	4	5	6	7	8	9	10	Don't know
1-Lowest -> Highest-10	○	○	○	○	○	○	○	○	○	○	○

* GEI 3.4 Civil Society Organizations (CSOs): Extent to which CSOs specifically operate as instruments for advocacy in the GEI sector.

	1	2	3	4	5	6	7	8	9	10	Don't know
1-Lowest -> Highest-10	○	○	○	○	○	○	○	○	○	○	○

* GEI 3.5 Advocacy Voice: Extent to which advocates have a voice in government discussions.

	1	2	3	4	5	6	7	8	9	10	Don't know
1-Lowest -> Highest-10	◯	◯	◯	◯	◯	◯	◯	◯	◯	◯	◯

* GEI 3.6 Voice of Instrumental Sectors of the DD: Extent to which the government and stakeholders are committed to establish sustainable institutions to ensure:

	1	2	3	4	5	6	7	8	9	10	Don't know
GEI 3.6.1 Good governance	◯	◯	◯	◯	◯	◯	◯	◯	◯	◯	◯
GEI 3.6.2 Financial stability	◯	◯	◯	◯	◯	◯	◯	◯	◯	◯	◯
GEI 3.6.3 Optimal family planning programs	◯	◯	◯	◯	◯	◯	◯	◯	◯	◯	◯
GEI 3.6.4 Optimal maternal and child health	◯	◯	◯	◯	◯	◯	◯	◯	◯	◯	◯
GEI 3.6.5 Good quality education	◯	◯	◯	◯	◯	◯	◯	◯	◯	◯	◯
GEI 3.6.6 Diversified labor market.	◯	◯	◯	◯	◯	◯	◯	◯	◯	◯	◯

GEI 3.7 Comments: Please use this section to provide any additional comments about the level of effort, challenges, or successes in the country around advocacy for GEI. If you marked “I don’t know” for any of the questions, also use this section to explain why you gave that rating

JOHNS HOPKINS
BLOOMBERG SCHOOL
of PUBLIC HEALTH

Bill & Melinda Gates Institute for Population and Reproductive Health

Demographic Dividend Effort Index Survey

Dimension 4. Research

* GEI 4.1 Strength of Observatory/Stakeholder/Technical Working Group for GEI: Extent to which country-level GEI stakeholders (e.g., stakeholder leadership group, technical working group, GEI observatory, country coordination and facilitation group):

	1	2	3	4	5	6	7	8	9	10	Don't know
GEI 4.1.1 Have representation in government, training institutions, civil society, nongovernmental and faith-based organizations, professional associations, the private sector, etc.	○	○	○	○	○	○	○	○	○	○	○
GEI 4.1.2 Meet regularly, report and recommend policy for senior management within the appropriate ministries	○	○	○	○	○	○	○	○	○	○	○
GEI 4.1.3 Make an impact on GEI within the country	○	○	○	○	○	○	○	○	○	○	○

* GEI 4.2 Education/Research Strategy: Extent to which national planning documents include a comprehensive strategy/approach on improving GEI, shared among partners.

	1	2	3	4	5	6	7	8	9	10	Don't know
1-Lowest -> Highest-10	○	○	○	○	○	○	○	○	○	○	○

* GEI 4.3 Governance, Financial and Development Data Gathering Partners:

	1	2	3	4	5	6	7	8	9	10	Don't know
GEI 4.3.1 Extent to which research is undertaken by government agencies. (This may include statistical agencies/bureaus and/or ministries of economics/finances, education, health, gender, etc.)											
GEI 4.3.2 Extent to which research is undertaken by public research institutions.											
GEI 4.3.3 Extent to which research is undertaken by independent researchers.											

* GEI 4.4 Governance, Financial and Development Topical Data Gathering:

	1	2	3	4	5	6	7	8	9	10	Don't know
GEI 4.4.1 Extent to which safety and the rule of law is researched.											
GEI 4.4.2 Extent to which political participation and human rights is measured.											
GEI 4.4.3 Extent to which indicators of a sustainable economy are researched and disseminated to the community.											
GEI 4.4.4 Extent to which human capital development (education, training, and employment, etc.) is researched.											

* GEI 4.5 Quality/Coverage of Data:

	1	2	3	4	5	6	7	8	9	10	Don't know
GEI 4.5.1 Extent to which a routine statistical system provides reliable periodic information on governance indicators.	○	○	○	○	○	○	○	○	○	○	○
GEI 4.5.2 Extent to which the routine statistical system provides reliable periodic information on governance-related needs among populations in different communities.	○	○	○	○	○	○	○	○	○	○	○
GEI 4.5.3 Extent to which a routine statistical system provides reliable periodic information on economic indicators.	○	○	○	○	○	○	○	○	○	○	○
GEI 4.5.4 Extent to which evaluations of the monitoring and surveillance systems are conducted and applied to ensure the reliability of data.	○	○	○	○	○	○	○	○	○	○	○

* GEI 4.6 Quality Research Institutions: Extent to which the country has the capacity to support and sustain research institutions that develop research/collect data to inform efforts to ensure good governance, stable economy and strong public institutions.

	1	2	3	4	5	6	7	8	9	10	Don't know
1-Lowest -> Highest-10	○	○	○	○	○	○	○	○	○	○	○

* GEI 4.7 Evaluation: Extent to which program statistics, surveys, and small studies are used by specialized staff to report on good governance and financial institutions.

	1	2	3	4	5	6	7	8	9	10	Don't know
1-Lowest -> Highest-10	○	○	○	○	○	○	○	○	○	○	○

* GEI 4.8 Management's Use of Evaluation Findings: Extent to which local-level program managers use research and evaluation findings to improve programming around the rule of law, stabilizing the economy and strengthening institutions in ways suggested by findings.

	1	2	3	4	5	6	7	8	9	10	Don't know
1-Lowest -> Highest-10	○	○	○	○	○	○	○	○	○	○	○

* GEI 4.10 Dissemination of Information to Other Implementing Bodies: Extent to which information is shared or spread across geographical areas and throughout various levels (national, regional, localities).

	1	2	3	4	5	6	7	8	9	10	Don't know
1-Lowest -> Highest-10	○	○	○	○	○	○	○	○	○	○	○

GEI 4.11 Comments: Please use this section to provide any additional comments about the level of effort, challenges, or successes in research around GEI. If you marked "I don't know" for any of the questions, also use this section to explain why you gave that rating.

JOHNS HOPKINS
BLOOMBERG SCHOOL
of PUBLIC HEALTH

Bill & Melinda Gates Institute for Population and Reproductive Health

Demographic Dividend Effort Index Survey

Dimension 5. Civil Society Organizations (CSOs)

* GEI 5.1 CSO Actor Power: Extent to which CSO actors are in strong positions to positively contribute to the rule of law.

	1	2	3	4	5	6	7	8	9	10	Don't know
1-Lowest -> Highest-10	○	○	○	○	○	○	○	○	○	○	○

* GEI 5.2 Budget Analysis as a CSO Tool: Extent to which CSOs utilize budget analysis as a tool to build an advocacy case for good governance, economy stability and stronger public institutions

	1	2	3	4	5	6	7	8	9	10	Don't know
1-Lowest -> Highest-10	○	○	○	○	○	○	○	○	○	○	○

* GEI 5.3 Community-Based Service Delivery Support: Extent to which CSOs participate in bringing governance-related information and services to the communities where people live.

	1	2	3	4	5	6	7	8	9	10	Don't know
1-Lowest -> Highest-10	○	○	○	○	○	○	○	○	○	○	○

* GEI 5.4 Human Rights and Quality of Implementation: Extent to which CSOs advocate for and support rights-based approaches to governance to ensure policy and program beneficiaries experience progress on their terms.

	1	2	3	4	5	6	7	8	9	10	Don't know
1-Lowest -> Highest-10	○	○	○	○	○	○	○	○	○	○	○

* GEI 5.5 Youth: Extent to which CSOs support youth to participate in education, training, employment and leadership.

	1	2	3	4	5	6	7	8	9	10	Don't know
1-Lowest -> Highest-10	○	○	○	○	○	○	○	○	○	○	○

* GEI 5.6 Programs for Women: Extent to which CSOs ensure GEI policies support women and girls to participate in leadership.

	1	2	3	4	5	6	7	8	9	10	Don't know
1-Lowest -> Highest-10	○	○	○	○	○	○	○	○	○	○	○

* GEI 5.7 Advocacy/Accountability

	1	2	3	4	5	6	7	8	9	10	Don't know
GEI 5.7.1 Extent to which CSOs support the participation of the community in governance or enforcement of the rule of law.	○	○	○	○	○	○	○	○	○	○	○
GEI 5.7.2 Extent to which the media support the participation of the community in governance or enforcement of the rule of law.	○	○	○	○	○	○	○	○	○	○	○

* GEI 5.8 CSO-Led Assessment and Monitoring: Extent to which CSOs assess, monitor and report on the effectiveness of policies and government accountability mechanisms and structures.

	1	2	3	4	5	6	7	8	9	10	Don't know
1-Lowest -> Highest-10	○	○	○	○	○	○	○	○	○	○	○

* GEI 5.9 Forming Partnerships between CSOs: Extent to which CSOs have formed national alliances and regional partnerships to strengthen their positioning and potentially improve leadership strength and funding mechanisms for good governance and institutional strengthening related work.

	1	2	3	4	5	6	7	8	9	10	Don't know
1-Lowest -> Highest-10	○	○	○	○	○	○	○	○	○	○	○

GEI 5.10 Comments: Please use this section to provide any additional comments about the level of effort, challenges, or successes around the role of the CSOs and media in GEI. If you marked “I don’t know” for any of the questions, also use this section to explain why you gave that rating

JOHNS HOPKINS
BLOOMBERG SCHOOL
of PUBLIC HEALTH

Bill & Melinda Gates Institute for Population and Reproductive Health

Demographic Dividend Effort Index Survey

Module: Sectoral Resilience and Sustainability

Considering the health and socio-economic impact of the COVID-19 and the likely impact on the DD progress, this DD effort index has integrated questions to assess the resilience and sustainability of systems in key sectors of the DD framework. The items included in the COVID-19 related questionnaire below follow the key dimensions of resilient systems and provide critical information on the potential of an effective response to emerging infectious disease threats or other public health emergencies.

Please score each item from 1 to 10, where 1 represents the lowest score (poor state/capability) and 10 represents the highest score (great state/capability).

* Physical: facilities, equipment, system states, and capabilities.

	1	2	3	4	5	6	7	8	9	10	Don't know
GEI-M1 - Plan/Prepare - State and capability of equipment, personnel and GEI sector system structure prior to the crisis event. (1 = poor state/capability; 10 = great state/capability)	○	○	○	○	○	○	○	○	○	○	○
GEI-M2 - Absorb - Extent to which COVID-19 was recognized within the GEI sector system and the performance of the system was able to maintain functionality.	○	○	○	○	○	○	○	○	○	○	○
GEI-M3 - Absorb - Integration: The level of integration of services and institutional collaborations to ensure continuity of access to essential services during the COVID-19 crisis.	○	○	○	○	○	○	○	○	○	○	○
GEI-M4 - Recover - Extent to which the Government and Economic Institutions have been able to induce change to recover the pre-COVID-19 level of functionality.	○	○	○	○	○	○	○	○	○	○	○
GEI-M5 - Recover - Financing and Donor Management: National bank and donor support to ensure the availability of cash during the COVID-19 crisis	○	○	○	○	○	○	○	○	○	○	○
GEI-M6 - Adapt - Extent to which changes have been made to improve the resilience of the GEI sector system and infrastructure.	○	○	○	○	○	○	○	○	○	○	○

* Information: creation, manipulation, storage, and utilization of data.

	1	2	3	4	5	6	7	8	9	10	Don't know
GEI-M7 - Plan/Prepare - Extent to which GEI-related data was sufficiently prepared, presented, analyzed and stored prior to the crisis event.	○	○	○	○	○	○	○	○	○	○	○
GEI-M8 - Absorb - Awareness: Level of awareness about the threats caused by COVID-19 in the central government.	○	○	○	○	○	○	○	○	○	○	○
GEI-M9 - Recover - Extent to which GEI data has been used to track the progress of recovery and to anticipate recovery scenarios.	○	○	○	○	○	○	○	○	○	○	○
GEI-M10 - Adapt - Extent to which the GEI sector system is creating and improving GEI data storage and real-time use protocols.	○	○	○	○	○	○	○	○	○	○	○

* Cognitive: understanding, mental models, preconceptions, biases, and values.

	1	2	3	4	5	6	7	8	9	10	Don't know
GEI-M11 - Plan/Prepare - Extent to which the GEI sector system design and operation decisions were prepared in anticipation of crisis events.	○	○	○	○	○	○	○	○	○	○	○
GEI-M12 - Absorb - Extent to which the Government and Economic Institutions are responding with sufficient contingency protocols and proactive crisis management.	○	○	○	○	○	○	○	○	○	○	○

	1	2	3	4	5	6	7	8	9	10	Don't know
GEI-M13 - Absorb - Accountability: Level of effort to ensure optimal accountability for the resources allocated to support governance as part of the response to the COVID-19.	○	○	○	○	○	○	○	○	○	○	○
GEI-M14 - Recover - Extent to which decisions are oriented towards recovery, which employ evidence-based communication to community members to promote safe behavior.	○	○	○	○	○	○	○	○	○	○	○
GEI-M15 - Recover - Awareness: Extent to which the government is building confidence among the public though communication and dialogue for evidence-based policymaking and actions.	○	○	○	○	○	○	○	○	○	○	○
GEI-M16 - Adapt - Extent to which the GEI sector system is designing new system configurations, objectives and decision criteria.	○	○	○	○	○	○	○	○	○	○	○
GEI-M17 - Adapt - Adaptive: Level of adaptation of financial and social service systems to ensure easy access via technology-based platforms during the COVID-19.	○	○	○	○	○	○	○	○	○	○	○

* Social: interaction, collaboration, and self-synchronization between individuals, entities and institutions.

	1	2	3	4	5	6	7	8	9	10	Don't know
GEI-M18 - Plan/Prepare - Extent to which training on outbreak/crisis management was conducted through, and which leveraged, social networks, social capital and institutional and cultural norms prior to the crisis event.	○	○	○	○	○	○	○	○	○	○	○
GEI-M19 - Absorb - Extent to which personnel and social institutions have been resourceful and accessible for the outbreak/crisis response.	○	○	○	○	○	○	○	○	○	○	○
GEI-M20 - Absorb - Self-Regulating: Extent to which the national leadership had the authority to make sectoral changes in a timely manner through flexible infrastructure.	○	○	○	○	○	○	○	○	○	○	○
GEI-M21 - Recover - Extent to which the GEI sector system is engaging with team and knowledge sharing to enhance systematic recovery.	○	○	○	○	○	○	○	○	○	○	○
GEI-M22 - Recover - Diversity: The level of engagement of multidisciplinary taskforces to mitigate the effect of COVID-19 at the national and subnational levels.	○	○	○	○	○	○	○	○	○	○	○
GEI-M23 - Adapt - Leadership and Management: Stewardship and management demonstrated by Government in response to the COVID-19 at the national level.	○	○	○	○	○	○	○	○	○	○	○

* GEI M-24 Timeline: Extent of timeliness of the Government reaction - communication and implementation of measures - to mitigate the immediate and longterm impact of COVID-19.
Note: This refers to the entirety of the national reaction - not the reaction that is specific to your sector.

	1	2	3	4	5	6	7	8	9	10	Don't know
1-Lowest -> Highest-10	◯	◯	◯	◯	◯	◯	◯	◯	◯	◯	◯

GEI M-25 Comments: Please use this section to provide any additional comments about the resilience of the national GEI systems based on your experience during the COVID-19 pandemics.If you marked “I don’t know” for any of the questions, also use this section to explain why you gave that rating.

JOHNS HOPKINS
BLOOMBERG SCHOOL
of PUBLIC HEALTH

Bill & Melinda Gates Institute for Population and Reproductive Health

Enquête de l'Indice des efforts pour le dividende démographique

Introduction

Les programmes de développement ont besoin d'une mesure standard qui quantifie la portée des efforts nationaux en termes de politiques et programmes mis en œuvre pour cultiver, réaliser et exploiter le dividende démographique (DD). En dépit du progrès remarquable accompli par plusieurs pays à travers l'intégration des principes du DD dans leurs politiques et plans de développement, des outils d'évaluation de la performance et des plateformes documentant les leçons et meilleures pratiques pour bénéficier du DD font toujours défaut.

Ainsi, l'objectif de l'Indice des efforts pour le dividende démographique (IEDD) est de fournir une mesure standard qui permettra aux experts et membres de la société civile de quantifier la nature et la puissance des efforts pour le DD dans les pays, autant dans les sphères publiques que privées, à travers différentes dimensions de la société. En mesurant ces efforts, nous documenterons les intrants indépendamment des extrants, dont les revenus par habitant. Cela nous permettra d'apprécier la relation entre les intrants pris en compte dans l'élaboration des politiques de DD et les extrants du DD.

Cet IEDD est guidé par une question centrale : Dans quelle mesure les efforts politiques et programmatiques existants ont-ils permis un environnement favorable à la culture, la réalisation et l'exploitation des bénéfices d'un dividende démographique ? Pour répondre à cette question, l'IEDD résumera les perceptions d'informateurs clés dans six secteurs identifiés dans la littérature scientifique comme produisant un environnement favorable à l'exploitation du DD. Ces secteurs cadres du DD sont : 1) Planification familiale, 2) Santé maternelle et infantile, 3) Education, 4) Autonomisation des femmes, 5) Marché du travail, et 6) Bonne gouvernance, solidité institutionnelle et stabilité économique. Chaque secteur est mesuré via un questionnaire qui lui est propre et est évalué à travers cinq domaines identifiés dans la littérature scientifique, à savoir : la politique/ prise de décision, les services ou programmes, le plaidoyer, la recherche, et la société civile. Chacun des 6 questionnaires, remplis par 10 observateurs experts par pays, notera chaque élément sur une échelle de 1 à 10, sauf indication contraire.

Étant donné l'impact sanitaire et socioéconomique de la pandémie de COVID-19, et sa relation étroite avec les politiques qui permettent un environnement politique favorable au DD, cet Indice des efforts de DD intègre un module pour évaluer la résilience et la durabilité des systèmes dans chaque secteur. La notation suit les principes clés des systèmes de santé résilients et durables pouvant répondre efficacement aux menaces de maladies infectieuses émergentes et autres crises de santé publiques.

L'IEDD permet de visualiser les efforts nationaux, tout en évaluant la mesure dans laquelle ces efforts soutiennent les objectifs du pays pour cultiver, réaliser et exploiter le dividende démographique. L'IEDD servira d'outil pour mesurer la performance et suivre le progrès, ce qui offrira une évaluation opportune des succès et des marges de progression, des meilleures pratiques et des améliorations possibles.

JOHNS HOPKINS
BLOOMBERG SCHOOL of PUBLIC HEALTH

Bill & Melinda Gates Institute for Population and Reproductive Health

Enquête de l’Indice des efforts pour le dividende démographique

Formulaire de consentement

Merci d’avoir pris le temps de répondre à ce questionnaire, particulièrement en cette période sans précédent.

L’Institut Gates développe actuellement un Indice des efforts pour le dividende démographique (IEDD) afin d’établir une mesure standard qui permettra d’évaluer le statut existant des politiques, services et programmes nationaux de planification familiale, santé maternelle et infantile, et dans d’autres secteurs sociaux (ex : emploi et éducation), ainsi qu’en matière de gouvernance pour permettre un environnement politique favorable au dividende démographique (DD). En mesurant ces efforts, cet outil documentera les intrants indépendamment des extrants, dont les revenus par habitant. Ceci permettra d’évaluer la relation entre les intrants pris en compte dans l’élaboration des politiques de DD et les extrants du DD observés et communément utilisés pour évaluer le progrès.

Nous aimerions vous poser quelques questions en raison de votre expertise dans ce domaine. Les informations collectées seront confidentielles, et nous ne collecterons ni votre nom, ni aucun autre détail permettant de vous identifier. Ce questionnaire devrait vous prendre environ 45-60 min.

Nous vous serions très reconnaissants si vous pouviez participer à cette enquête. Les informations générées par cette interview seront utilisées par l’Institut Gates et la communauté de pratique sur le DD dans votre pays pour évaluer les efforts nationaux, identifier les domaines de réussites qui doivent continuer d’être soutenus et les domaines nécessitant d’être renforcés. Les chercheurs pourront aussi utiliser les données collectées à des fins d’analyse comparative entre les pays.

Vous êtes invité(e) à participer à cette enquête. Sachez que la participation à cette enquête est entièrement volontaire. En participant, vous ne percevrez aucun bénéfice direct, mais il y a des bénéfices sociétaux à permettre aux gouvernements et aux partenaires du développement de renforcer leurs efforts pour cultiver un environnement favorable au DD.

Vous pouvez refuser de répondre à toute question. Si vous avez des inquiétudes ou problèmes concernant cette étude, vous pouvez contacter son coordinateur à **jcrusatira@jhu.edu** ou au **+14436510824** par téléphone ou WhatsApp.

* Veuillez cocher “Oui” si vous souhaitez procéder et répondre à l’enquête.

- ◯ Oui
- ◯ Non

JOHNS HOPKINS
BLOOMBERG SCHOOL of PUBLIC HEALTH

Bill & Melinda Gates Institute for Population and Reproductive Health

Enquête de l’Indice des efforts pour le dividende démographique

Avis important

Veuillez ne pas sélectionner "Page précédente" au milieu d'une page de questions car la page sur laquelle vous travaillez ne sera pas sauvegardée tant que vous n'aurez pas finalisé ladite page et sélectionner "Page suivante". Si vous sélectionnez "Page précédente" sans avoir terminé, vos réponses sur la page inachevée seront perdus.

Sachez que votre enquête est terminée lorsque vous lisez "Merci d'avoir rempli l'indice d'effort du dividende démographique". Merci !

JOHNS HOPKINS
BLOOMBERG SCHOOL
of PUBLIC HEALTH

Bill & Melinda Gates Institute for Population and Reproductive Health

Enquête de l’Indice des efforts pour le dividende démographique

Pays et expertise de l’enquêté(e)

* Veuillez sélectionner le pays duquel vous répondez.

- ○ Kenya
- ○ Senegal
- ○ Nigeria
- ○ Rwanda
- ○ Tanzania
- ○ Ethiopia

JOHNS HOPKINS
BLOOMBERG SCHOOL
of PUBLIC HEALTH

Bill & Melinda Gates Institute for Population and Reproductive Health

Enquête de l’Indice des efforts pour le dividende démographique

Level of expertise

* At which level of Government is your expertise (If your expertise is at the national level please select "National" otherwise select a county?

JOHNS HOPKINS
BLOOMBERG SCHOOL of PUBLIC HEALTH

Bill & Melinda Gates Institute for Population and Reproductive Health

Enquête de l'Indice des efforts pour le dividende démographique

Niveau d'expertise

* A quel niveau de gouvernement votre expertise se situe-t-elle (Si votre expertise se situe au niveau national, veuillez sélectionner "National", sinon sélectionnez une région ?

JOHNS HOPKINS
BLOOMBERG SCHOOL
of PUBLIC HEALTH

Bill & Melinda Gates Institute for Population and Reproductive Health

Enquête de l'Indice des efforts pour le dividende démographique

Expertise

* Veuillez sélectionner le type d'organisation dans laquelle vous avez le plus travaillé au cours des cinq dernières années. Si votre type d'organisation n'est pas référencé, veuillez préciser de quel type d'organisation il s'agit.

- Secteur public
- Secteur privé
- Organisation non gouvernementale
- Université/ Institut de recherche
- Autre (veuillez préciser)
- Other (please specify)

* Dans quel domaine avez-vous le plus travaillé au cours des cinq dernières années ?

- Politique/ Prise de décision
- Services ou programmes
- Plaidoyer
- Recherche
- Société Civile

* Dans quel secteur avez-vous le plus travaillé dans les cinq dernières années ?

- Planification familiale
- Santé maternelle et infantile
- Education
- Autonomisation des femmes
- Marché du travail
- Gouvernance et institutions économiques

JOHNS HOPKINS
BLOOMBERG SCHOOL
of PUBLIC HEALTH

Bill & Melinda Gates Institute for Population and Reproductive Health

Enquête de l'Indice des efforts pour le dividende démographique

Questionnaire sur la planification familiale (PF)

Répondez aux questions suivantes en fonction de votre expérience/ expertise dans ce secteur spécifique.

Afin d'obtenir une représentation synthétique des efforts nationaux pour le dividende démographique, notez les éléments suivants sur une échelle de 1 à 10, 1 étant le score le plus faible (efforts très faibles ou quasi-inexistants) et 10 le plus élevé (efforts robustes). Le cas échéant, si une politique ou activité n'existe pas, répondez 0.

Donnez un score à chaque élément. Toutes les réponses seront enregistrées au format illustré ci-dessous :

Effort le plus faible	1	2	3	4	5	6	7	8	9	10	Effort le plus élevé	Je ne sais pas

JOHNS HOPKINS
BLOOMBERG SCHOOL
of PUBLIC HEALTH

Bill & Melinda Gates Institute for Population and Reproductive Health

Enquête de l'Indice des efforts pour le dividende démographique

Domaine 1. Politique/ Prise de décision

* FP 1.1 La proéminence de la planification familiale (PF) : Mesure dans laquelle il existe un bureau ou poste permanent et bien placé au ministère compétent qui développe et surveille les politiques et stratégies du secteur, et dispose d'effectifs adéquats et de personnel qualifié.

	1	2	3	4	5	6	7	8	9	10	Je ne sais pas
1-Plus faible -> Plus élevé-10	○	○	○	○	○	○	○	○	○	○	○

* FP 1.2 La politique de PF et réduction de la fécondité : Mesure dans laquelle la politique gouvernementale met l'accent sur la PF pour promouvoir la taille des familles en lien avec les ressources disponibles ou des raisons démographiques plutôt que des raisons sanitaires.

	1	2	3	4	5	6	7	8	9	10	Je ne sais pas
1-Plus faible -> Plus élevé-10	○	○	○	○	○	○	○	○	○	○	○

* FP 1.3 Le plan national stratégique de PF : Mesure dans laquelle il existe un plan national actualisé, exhaustif et stratégique pour la PF, définissant les politiques, lois et régulations pour l'offre de PF. (Évaluez la mesure dans laquelle le plan a une vision, comprend des stratégies sur plusieurs années, des calendriers annuels, et des plans de mise en œuvre, tout en étant fondés sur des données probantes, avec des indicateurs de progrès, et tenant compte du marché de la PF).

	1	2	3	4	5	6	7	8	9	10	Je ne sais pas
1-Plus faible -> Plus élevé-10	○	○	○	○	○	○	○	○	○	○	○

* FP 1.4 La politique sur l'âge légal du mariage : Mesure dans laquelle l'âge légal du mariage pour les filles, établi à 18 ans ou plus, est appliqué.

	1	2	3	4	5	6	7	8	9	10	Je ne sais pas
1-Plus faible -> Plus élevé-10	○	○	○	○	○	○	○	○	○	○	○

* FP 1.5 La prise de décision fondée sur des données probantes : Mesure dans laquelle le ministère compétent soutient et utilise activement et régulièrement les données clés et rapports de PF pour élaborer ses politiques et ses plans.

	1	2	3	4	5	6	7	8	9	10	Je ne sais pas
1-Plus faible -> Plus élevé-10	○	○	○	○	○	○	○	○	○	○	○

* FP 1.6 Le suivi et évaluation des politiques/plans/stratégies nationaux : Mesure dans laquelle le plan national de PF établit une stratégie de suivi et évaluation complète pour suivre et mesurer les accomplissements prévus dans le plan. (Évaluez si la stratégie de suivi et évaluation inclut des buts et objectifs, un cadre de résultats, des indicateurs mesurables [ex : "SMART": spécifiques, mesurables, atteignables, réalistes et temporellement définis], et un plan/calendrier exploitable de mise en œuvre.)

	1	2	3	4	5	6	7	8	9	10	Je ne sais pas
1-Plus faible -> Plus élevé-10	○	○	○	○	○	○	○	○	○	○	○

* FP 1.7 L'inclusion des acteurs non gouvernementaux et intersectoriels au développement de politique/stratégie : Mesure dans laquelle les agents non-gouvernementaux, les associations (ex : de la société civile), les travailleurs de la santé, les parties prenantes (ex : organisations confessionnelles, à but non lucratif et à but lucratif) et les acteurs d'autres secteurs que celui de la PF sont inclus dans le plan national de PF et/ou autres documents nationaux établissant les stratégies et systèmes du pays pour planifier, gérer et développer la PF.

	1	2	3	4	5	6	7	8	9	10	Je ne sais pas
1-Plus faible -> Plus élevé-10	○	○	○	○	○	○	○	○	○	○	○

* FP 1.8 Le financement de la PF : Mesure dans laquelle le gouvernement a établi et appliqué des directives pour la budgétisation et le paiement de la PF en termes d'offre et de demande (soit les financements intérieurs vs. internationaux, et leur continuité/durabilité, coût des produits contraceptifs, coût de la prestation de services et du transport, etc.).

(Évaluez si les directives et le budget tiennent compte de la durée de vie des coûts des soins et services de PF ; des tarifications variables par méthode de PF ; des coûts de livraison et prestation de services par spécialité, lieu et risque ; des coûts et profits des plans/politiques d'assurance ; du coût financier de fournir de la PF en comparaison au coût financier de ne pas le faire, etc.).

	1	2	3	4	5	6	7	8	9	10	Je ne sais pas
1-Plus faible -> Plus élevé-10	○	○	○	○	○	○	○	○	○	○	○

* FP 1.9 Le pourcentage du financement intérieur (domestique) pour le budget de la PF : Mesure dans laquelle le budget total de la planification familiale/population est dérivé de sources domestiques (ex : 1 pour 10%, 5 pour 50%, 10 pour 100%).

	1	2	3	4	5	6	7	8	9	10	Je ne sais pas
1-Plus faible -> Plus élevé-10	○	○	○	○	○	○	○	○	○	○	○

* FP 1.10 L'infrastructure/ capacité des Technologies de l'Information et la Communication (TIC) : Mesure dans laquelle il existe des éléments d'infrastructure (ex : ordinateurs, serveurs, imprimantes, réseau local, connexion internet, alimentation électrique fiable) ainsi que des ressources humaines (ex : pour la maintenance et le soutien) au niveau national et infranational permettant de soutenir des systèmes d'informations fonctionnels sur la PF.

(Pour cet élément, évaluez si le pays conduit au moins des évaluations annuelles de ses systèmes informatiques ; a des programmes de formation couvrant la saisie et l'utilisation des données, l'administration et le développement de logiciels ; et forme continuellement le personnel des institutions clés [ex : Ministère de la Santé, Ministère de l'Éducation, les agences régulatrices et les institutions de formation]).

	1	2	3	4	5	6	7	8	9	10	Je ne sais pas
1-Plus faible -> Plus élevé-10	○	○	○	○	○	○	○	○	○	○	○

* FP 1.11 Les lois et régulations sur l'importation :

	1	2	3	4	5	6	7	8	9	10	Je ne sais pas
FP 1.11.1 Mesure dans laquelle les lois et régulations sur l'importation facilitent l'importation des commodités et produits de PF.	◯	◯	◯	◯	◯	◯	◯	◯	◯	◯	◯
FP 1.11.2 Mesure dans laquelle les contraceptifs sont fabriqués localement.	◯	◯	◯	◯	◯	◯	◯	◯	◯	◯	◯

* FP 1.12 La publicité des contraceptifs autorisés : Mesure dans laquelle la publicité des contraceptifs dans les médias de masse n'est soumise à aucune restriction.

	1	2	3	4	5	6	7	8	9	10	Je ne sais pas
1-Plus faible -> Plus élevé-10	◯	◯	◯	◯	◯	◯	◯	◯	◯	◯	◯

* FP 1.13 La présence de politiques de PF favorisant les populations pauvres : Mesure dans laquelle les politiques ciblent spécifiquement les populations pauvres en milieu rural et urbain.

	1	2	3	4	5	6	7	8	9	10	Je ne sais pas
1-Plus faible -> Plus élevé-10	◯	◯	◯	◯	◯	◯	◯	◯	◯	◯	◯

* FP 1.14 Le soutien politique à la PF : Mesure dans laquelle les élus dans le pays priorisent les besoins de PF pour renforcer les contributions de la PF au DD en adoptant des lois et régulations, et en appuyant des actions et politiques visant à améliorer l'utilisation et l'adhérence à la PF.

	1	2	3	4	5	6	7	8	9	10	Je ne sais pas
1-Plus faible -> Plus élevé-10	◯	◯	◯	◯	◯	◯	◯	◯	◯	◯	◯

FP 1.15 Commentaires : Veuillez utiliser cette section pour tout commentaire additionnel sur le niveau d'effort, les difficultés et les succès rencontrés dans le pays autour des politiques/ prise de décision pour la PF. Si vous avez répondu « Je ne sais pas » à l'une des questions, utilisez aussi cette section pour expliquer pourquoi.

Johns Hopkins Bloomberg School of Public Health

Bill & Melinda Gates Institute for Population and Reproductive Health

Enquête de l'Indice des efforts pour le dividende démographique

Domaine 2. Services ou programmes

* FP 2.1 La structure administrative : Mesure dans laquelle la structure administrative et le personnel au niveau national, provincial et des comtés est suffisant pour mettre en œuvre le programme de planification familiale (PF).

	1	2	3	4	5	6	7	8	9	10	Je ne sais pas
1-Plus faible -> Plus élevé-10	○	○	○	○	○	○	○	○	○	○	○

* FP 2.2 Le niveau des responsables du programme : Niveau d'ancienneté relatif du directeur du programme national de planification familiale et mesure dans laquelle le supérieur hiérarchique direct de ce directeur est haut placé au gouvernement.

	1	2	3	4	5	6	7	8	9	10	Je ne sais pas
1-Plus faible -> Plus élevé-10	○	○	○	○	○	○	○	○	○	○	○

* FP. 2.3 La distribution à base communautaire : Mesure dans laquelle les zones du pays non desservies par les cliniques et autres sites de prestation de santé sont couvertes par des programmes de distribution à base communautaire de produits contraceptifs (particulièrement les zones rurales).

	1	2	3	4	5	6	7	8	9	10	Je ne sais pas
1-Plus faible -> Plus élevé-10	○	○	○	○	○	○	○	○	○	○	○

* FP 2.4 Le programme de PF postpartum (PFPP) : Mesure dans laquelle toutes les femmes en postpartum reçoivent des services PFPP.

	1	2	3	4	5	6	7	8	9	10	Je ne sais pas
1-Plus faible -> Plus élevé-10	○	○	○	○	○	○	○	○	○	○	○

* FP 2.5 Les agents travaillant à domicile : Mesure de la couverture de la population par les agents dont la tâche principale est de rendre visite aux femmes (en milieu rural) chez elles pour discuter de planification familiale et de santé maternelle et infantile.

	1	2	3	4	5	6	7	8	9	10	Je ne sais pas
1-Plus faible -> Plus élevé-10	○	○	○	○	○	○	○	○	○	○	○

* FP 2.6 La disponibilité et l'accessibilité des commodités/produits et services de PF (première partie) : Mesure dans laquelle l'ensemble de la population a facilement et directement accès à (1-le plus faible -> le plus élevé-10):

	1	2	3	4	5	6	7	8	9	10	Je ne sais pas
FP 2.6.1 DIU	○	○	○	○	○	○	○	○	○	○	○
FP 2.6.2 Pilule	○	○	○	○	○	○	○	○	○	○	○
FP 2.6.3 Injectable	○	○	○	○	○	○	○	○	○	○	○
FP 2.6.4 Implant	○	○	○	○	○	○	○	○	○	○	○
FP 2.6.5 Stérilisation féminine	○	○	○	○	○	○	○	○	○	○	○
FP 2.6.6 Stérilisation masculine	○	○	○	○	○	○	○	○	○	○	○
FP 2.6.7 Préservatif masculin	○	○	○	○	○	○	○	○	○	○	○
FP 2.6.8 Contraception d'urgence	○	○	○	○	○	○	○	○	○	○	○
FP 2.6.9 Avortement/ régulation de la menstruation (légal ou non)	○	○	○	○	○	○	○	○	○	○	○
FP 2.6.10 Autres (ex : préservatif féminin, mousse, collier du cycle, etc.)	○	○	○	○	○	○	○	○	○	○	○

* FP 2.7 La disponibilité et l'accessibilité des commodités/produits et services de PF, (deuxième partie) : Quel est le niveau de fonctionnalité du système d'approvisionnement (évite t-il les ruptures de stock ou l'interruption de l'approvisionnement, ou permet-il un flux fiable à tous les niveaux ?) pour les méthodes suivantes (1-le plus faible -> le plus élevé-10) :

	1	2	3	4	5	6	7	8	9	10	Don't know
FP 2.7.1 DIU	○	○	○	○	○	○	○	○	○	○	○
FP 2.7.2 Pilule	○	○	○	○	○	○	○	○	○	○	○
FP 2.7.3 Injectable	○	○	○	○	○	○	○	○	○	○	○
FP 2.7.4 Implant	○	○	○	○	○	○	○	○	○	○	○
FP 2.7.5 Préservatif masculin	○	○	○	○	○	○	○	○	○	○	○
FP 2.7.6 Contraception d'urgence	○	○	○	○	○	○	○	○	○	○	○
FP 2.7.7 Autres (ex : préservatif féminin, mousse, collier du cycle, etc.)	○	○	○	○	○	○	○	○	○	○	○

* FP 2.8 La disponibilité et l'accessibilité des commodités/produits et services de PF (troisième partie) : Dans quelle mesure le système d'approvisionnement fournit-il l'équipement et les produits médicaux nécessaires aux structures cliniques pour les méthodes suivantes (1-le plus faible -> le plus élevé-10) :

	1	2	3	4	5	6	7	8	9	10	Je ne sais pas
FP 2.8.1 Stérilisation féminine	○	○	○	○	○	○	○	○	○	○	○
FP 2.8.2 Stérilisation masculine	○	○	○	○	○	○	○	○	○	○	○
FP 2.8.3. DIU	○	○	○	○	○	○	○	○	○	○	○
FP 2.8.4 Implant	○	○	○	○	○	○	○	○	○	○	○

Other (please specify)

[ ]

* FP 2.9 La qualité des services de PF : Veuillez noter la qualité générale des services de planification familiale. (Des services de haute qualité impliquent d'être orientés sur les besoins des clients, et de fournir du conseil, des informations complètes, un large choix de méthodes, et des procédures cliniques sûres).

	1	2	3	4	5	6	7	8	9	10	Je ne sais pas
1-Plus faible -> Plus élevé-10	○	○	○	○	○	○	○	○	○	○	○

* FP 2.10 Les avantages et les effets dissuasifs : Mesure dans laquelle des avantages monétaires ou d'autre type sont utilisés pour encourager l'adoption de la planification familiale.

	1	2	3	4	5	6	7	8	9	10	Je ne sais pas
1-Plus faible -> Plus élevé-10	◯	◯	◯	◯	◯	◯	◯	◯	◯	◯	◯

* FP 2.11 Les programmes de PF pour les adolescents : Mesure dans laquelle les informations, services et activités sont focalisés sur les adolescents et encouragent ces derniers à accéder aux services contraceptifs.

	1	2	3	4	5	6	7	8	9	10	Je ne sais pas
1-Plus faible -> Plus élevé-10	◯	◯	◯	◯	◯	◯	◯	◯	◯	◯	◯

* FP 2.12 Les programmes de formation : Mesure dans laquelle les programmes de formation, pour chaque catégorie de personnel dans le programme de planification familiale, sont adéquats pour transmettre au personnel les informations et compétences nécessaires à la conduite efficace de leur travail.

	1	2	3	4	5	6	7	8	9	10	Je ne sais pas
1-Plus faible -> Plus élevé-10	◯	◯	◯	◯	◯	◯	◯	◯	◯	◯	◯

* FP 2.13 Les tâches assignées remplies par le personnel : Mesure dans laquelle toutes les catégories du personnel du programme de planification familiale (administratif, médical, paramédical, de terrain) remplissent leurs tâches assignées efficacement.

	1	2	3	4	5	6	7	8	9	10	Je ne sais pas
1-Plus faible -> Plus élevé-10	◯	◯	◯	◯	◯	◯	◯	◯	◯	◯	◯

* FP 2.14 La logistique et le transport : Mesure dans laquelle les systèmes logistiques et de transport sont suffisants pour maintenir les stocks de produits contraceptifs et l'équipement disponible sur tous les sites de prestation de santé, à tout moment et à tous les niveaux.

	1	2	3	4	5	6	7	8	9	10	Je ne sais pas
1-Plus faible -> Plus élevé-10	◯	◯	◯	◯	◯	◯	◯	◯	◯	◯	◯

* FP 2.15 Le système de supervision : Mesure dans laquelle le système de supervision à tous les niveaux est adéquat (visite de supervision régulières, suivies par des actions correctives ou d'appui/accompagnantes).

	1	2	3	4	5	6	7	8	9	10	Je ne sais pas
1-Plus faible -> Plus élevé-10	◯	◯	◯	◯	◯	◯	◯	◯	◯	◯	◯

* FP 2.16 L'implication d'autres ministères et agences publiques : Mesure dans laquelle d'autres ministères, agences gouvernementales et parties prenantes non gouvernementales s'impliquent dans les activités de PF (ex : livraison de produits, prestation de services, information, éducation) ou autres activités pour la population.

	1	2	3	4	5	6	7	8	9	10	Je ne sais pas
1-Plus faible -> Plus élevé-10	◯	◯	◯	◯	◯	◯	◯	◯	◯	◯	◯

* FP 2.17 L'implication d'agences et groupes du secteur privé : Mesure dans laquelle des agences ou groupes du secteur privé contribuent aux activités de PF ou autres activités pour la population.

	1	2	3	4	5	6	7	8	9	10	Je ne sais pas
1-Plus faible -> Plus élevé-10	◯	◯	◯	◯	◯	◯	◯	◯	◯	◯	◯

* FP 2.18 Les médias de masse pour la communication, l'éducation et l'information : Fréquence et couverture des messages des médias de masse fournissant à la population des informations sur a planification familiale et les sites de prestation de services.

	1	2	3	4	5	6	7	8	9	10	Je ne sais pas
1-Plus faible -> Plus élevé-10	◯	◯	◯	◯	◯	◯	◯	◯	◯	◯	◯

* FP 2.19 Les barrières normatives, culturelles et sociales : Mesure dans laquelle les programmes de planification familiale répondent aux barrières culturelles et sociales.

	1	2	3	4	5	6	7	8	9	10	Je ne sais pas
1-Plus faible -> Plus élevé-10	◯	◯	◯	◯	◯	◯	◯	◯	◯	◯	◯

* FP 2.20 Les jeunes : Mesure dans laquelle le gouvernement soutient des initiatives de PF dirigées par les jeunes en matière de planification, mise en œuvre et évaluation des programmes de PF.

	1	2	3	4	5	6	7	8	9	10	Je ne sais pas
1-Plus faible -> Plus élevé-10	◯	◯	◯	◯	◯	◯	◯	◯	◯	◯	◯

* FP 2.21 Les influences des efforts de PF (les forces influençant le programme de PF) : Les forces influençant le programme de PF peuvent soit renforcer, soit diminuer son efficacité, Cochez zéro (0) s'il n'y a aucune différence ; cochez un nombre négatif de -1 à -5 si l'influence est négative ; ou cochez un nombre positif de 1 à 5 si l'influence est positive. (1 dans chaque direction signifie peu d'influence et 5 dans chaque direction signifie beaucoup d'influence).

	-5	-4	-3	-2	-1	0	1	2	3	4	5	Je ne sais pas
FP 2.21.1 Décentralisation	○	○	○	○	○	○	○	○	○	○	○	○
FP 2.21.2 Programmes VIH/SIDA	○	○	○	○	○	○	○	○	○	○	○	○
FP 2.21.3 Incorporation de la PF au contexte plus large de la santé reproductive (SR)	○	○	○	○	○	○	○	○	○	○	○	○
FP 2.21.4 Intégration de la PF à d'autres services de santé	○	○	○	○	○	○	○	○	○	○	○	○
FP 2.21.5 Changements dans les financements des bailleurs de fonds	○	○	○	○	○	○	○	○	○	○	○	○
FP 2.21.6 Changements dans les financements du gouvernement	○	○	○	○	○	○	○	○	○	○	○	○

FP 2.22 Commentaires : Veuillez utiliser cette section pour tout commentaire additionnel sur le niveau d'effort, les difficultés et les succès rencontrés dans le pays autour des services ou programmes de PF. Si vous avez répondu « Je ne sais pas » à l'une des questions, utilisez aussi cette section pour expliquer pourquoi.

JOHNS HOPKINS
BLOOMBERG SCHOOL of PUBLIC HEALTH

Bill & Melinda Gates Institute for Population and Reproductive Health

Enquête de l'Indice des efforts pour le dividende démographique

Domaine 3. Plaidoyer

* FP 3.1 L'influence des dirigeants/ champions de PF : Mesure dans laquelle le pays compte sur un ou plusieurs dirigeants ou champions clairement influents plaidant avec succès au profit des besoins (sectoriels) de la PF auprès des décideurs, promouvant la PF dans le pays à travers des déclarations publiques positives, et/ou soutenant des actions et politiques visant à améliorer l'environnement de la PF et ses contributions au DD.

	1	2	3	4	5	6	7	8	9	10	Je ne sais pas
1-Plus faible -> Plus élevé-10	○	○	○	○	○	○	○	○	○	○	○

* FP 3.2 Les déclarations des dirigeants : Mesure dans laquelle le chef du gouvernement, ainsi que d'autres hauts fonctionnaires, parlent publiquement et favorablement de la planification familiale au moins une fois par an.

	1	2	3	4	5	6	7	8	9	10	Je ne sais pas
1-Plus faible -> Plus élevé-10	○	○	○	○	○	○	○	○	○	○	○

* FP 3.3 La couverture médiatique de la PF : Mesure dans laquelle et fréquence à laquelle la couverture médiatique nationale des sujets de PF apporte à la population des informations sur la planification familiale et les sites de prestation de services.

	1	2	3	4	5	6	7	8	9	10	Je ne sais pas
1-Plus faible -> Plus élevé-10	○	○	○	○	○	○	○	○	○	○	○

* FP 3.4 Les influences des efforts de PF (justification) : Quelle est l'importance de chacun des éléments suivants en tant que justification actuelle par le gouvernement de son programme national de PF ? (1 signifie une importance négligeable ; 10 signifie une grande importance.)

	1	2	3	4	5	6	7	8	9	10	Je ne sais pas
3.4.1 Réduire le taux de croissance démographique	○	○	○	○	○	○	○	○	○	○	○
3.4.2 Stimuler le développement économique	○	○	○	○	○	○	○	○	○	○	○
3.4.3 Aider les hommes et les femmes à éviter des naissances non planifiées et non désirées	○	○	○	○	○	○	○	○	○	○	○
3.4.4 Améliorer la santé de la femme	○	○	○	○	○	○	○	○	○	○	○
3.4.5 Améliorer la santé de l'enfant	○	○	○	○	○	○	○	○	○	○	○
3.4.6 Réduire les grossesses chez les adolescentes non mariées	○	○	○	○	○	○	○	○	○	○	○
3.4.7 Réduire les besoins contraceptifs non satisfaits	○	○	○	○	○	○	○	○	○	○	○

* FP 3.5 Les influences des efforts de PF (les populations vulnérables) : Dans quelle mesure le programme de planification familiale met-il l’accent sur les populations vulnérables ou spécifiques ? (1 signifie un accent négligeable ; 10 signifie un accent important).

	1	2	3	4	5	6	7	8	9	10	Je ne sais pas
FP 3.5.1 Les jeunes non mariés	○	○	○	○	○	○	○	○	○	○	○
FP 3.5.2 Les populations vivant dans la pauvreté	○	○	○	○	○	○	○	○	○	○	○
FP 3.5.3 Les personnes en situation de handicap	○	○	○	○	○	○	○	○	○	○	○
FP 3.5.4 Les populations rurales	○	○	○	○	○	○	○	○	○	○	○
FP 3.5.5 Autres groupes vulnérables (ex : les minorités et/ou les groupes indigènes)	○	○	○	○	○	○	○	○	○	○	○
FP 3.5.6 Les femmes en postpartum pour le conseil et les services de contraception	○	○	○	○	○	○	○	○	○	○	○
FP 3.5.7 Les femmes en post-avortement pour le conseil et les services de contraception	○	○	○	○	○	○	○	○	○	○	○

* FP 3.6 Le marketing social : Mesure de la couverture du programme de marketing social du pays (ventes des contraceptifs subventionnés à faible coût dans le secteur commercial, particulièrement en milieu urbain).

	1	2	3	4	5	6	7	8	9	10	Je ne sais pas
1-Plus faible -> Plus élevé-10	○	○	○	○	○	○	○	○	○	○	○

FP 3.7 Commentaires : Veuillez utiliser cette section pour tout commentaire additionnel sur le niveau d’effort, les difficultés et les succès rencontrés dans le pays autour du plaidoyer pour la PF. Si vous avez répondu « Je ne sais pas » à l’une des questions, utilisez aussi cette section pour expliquer pourquoi.

JOHNS HOPKINS
BLOOMBERG SCHOOL
of PUBLIC HEALTH

Bill & Melinda Gates Institute for Population and Reproductive Health

Enquête de l'Indice des efforts pour le dividende démographique

Domaine 4. Recherche

* FP 4.1 La force des observatoires/parties prenantes/groupes de travail pour la PF : Mesure dans laquelle les parties prenantes du secteur de la planification familiale du pays (ex : groupe directif de parties prenantes, groupes de travail techniques, observatoire de la PF, groupe de coordination ou facilitation) :

	1	2	3	4	5	6	7	8	9	10	Je ne sais pas
FP 4.1.1 Ont un représentant du gouvernement, des institutions de formation, de la société civile, des organisations non gouvernementales et confessionnelles, des associations professionnelles, du secteur privé, etc.	○	○	○	○	○	○	○	○	○	○	○
FP 4.1.2 Se réunissent régulièrement, émettent des rapports et recommandent des politiques aux directions des ministères compétents.	○	○	○	○	○	○	○	○	○	○	○
FP 4.1.3 Ont un impact sur la PF dans le pays.	○	○	○	○	○	○	○	○	○	○	○

* FP 4.2 La stratégie de recherche en PF : Mesure dans laquelle le plan national de PF et/ou d'autres documents nationaux comprennent une stratégie/approche complète de recherche sur la planification familiale.

	1	2	3	4	5	6	7	8	9	10	Je ne sais pas
1-Plus faible -> Plus élevé-10	○	○	○	○	○	○	○	○	○	○	○

* FP 4.3 Les partenaires collectant les données :

	1	2	3	4	5	6	7	8	9	10	Je ne sais pas
FP 4.3.1 Mesure dans laquelle la collecte des données est entreprise par des agences gouvernementales. (Cela peut comprendre les agences/bureaux de la statistique et/ou les ministères de l'économie/finances, éducation, santé, genre, etc.)	◯	◯	◯	◯	◯	◯	◯	◯	◯	◯	◯
FP 4.3.2 Mesure dans laquelle la collecte de données est entreprise par des institutions de recherche.	◯	◯	◯	◯	◯	◯	◯	◯	◯	◯	◯
FP 4.3.3 Mesure dans laquelle la collecte des données est entreprise par des chercheurs indépendants.	◯	◯	◯	◯	◯	◯	◯	◯	◯	◯	◯

* FP 4.4 La recherche thématique :

	1	2	3	4	5	6	7	8	9	10	Je ne sais pas
FP 4.4.1 Mesure dans laquelle la recherche est menée sur l'adoption de la planification familiale (dont la distribution des méthodes), la demande et les besoins non satisfaits.	◯	◯	◯	◯	◯	◯	◯	◯	◯	◯	◯
FP 4.4.2 Mesure dans laquelle de la recherche est menée sur les barrières à l'adoption de la PF	◯	◯	◯	◯	◯	◯	◯	◯	◯	◯	◯
FP 4.4.3 Mesure dans laquelle de la recherche est menée sur les programmes de PF	◯	◯	◯	◯	◯	◯	◯	◯	◯	◯	◯
FP 4.4.4 Mesure dans laquelle de la recherche est menée sur la qualité de la prestation des services de PF	◯	◯	◯	◯	◯	◯	◯	◯	◯	◯	◯

* FP 4.5 La qualité/couverture des données : Mesure dans laquelle la recherche/les données actuelles sont désagrégées par sexe et par âge. Mesure dans laquelle le pays participe aux tests standardisés et la place qu'il occupe au classement.

	1	2	3	4	5	6	7	8	9	10	Je ne sais pas
FP 4.5.1 Mesure dans laquelle un système statistique de routine offre de bonnes informations périodiques sur les produits et les établissements de PF	○	○	○	○	○	○	○	○	○	○	○
FP 4.5.2 Mesure dans laquelle un système statistique de routine offre de bonnes informations régulières sur les services et le personnel du secteur de la PF	○	○	○	○	○	○	○	○	○	○	○
FP 4.5.3 Mesure dans laquelle un système statistique de routine offre de bonnes informations périodiques sur les besoins de PF des populations de différentes communautés	○	○	○	○	○	○	○	○	○	○	○
FP 4.5.4 Mesure dans laquelle des évaluations du système de suivi des informations sanitaires sont menées et appliquées pour assurer la fiabilité des données	○	○	○	○	○	○	○	○	○	○	○

* FP 4.6 Les registres : Mesure dans laquelle les systèmes de registres des clients, rapports des cliniques et retours/commentaires sur les résultats sont adéquats et connectés à tous les niveaux (fédéral, état/province, comté, etc.).

	1	2	3	4	5	6	7	8	9	10	Je ne sais pas
1-Plus faible -> Plus élevé-10	○	○	○	○	○	○	○	○	○	○	○

* FP 4.7 La qualité des institutions de recherche : Mesure dans laquelle le pays a la capacité de soutenir et maintenir des institutions de recherche qui développent des études permettant de soutenir la portée et la qualité de la collecte de données pour informer les efforts nationaux de PF.

	1	2	3	4	5	6	7	8	9	10	Je ne sais pas
1-Plus faible -> Plus élevé-10	○	○	○	○	○	○	○	○	○	○	○

* FP 4.8 L'évaluation : Mesure dans laquelle les statistiques, enquêtes et études sur le programme de PF sont utilisées par du personnel spécialisé pour rapporter les opérations du programme et mesurer le progrès.

	1	2	3	4	5	6	7	8	9	10	Je ne sais pas
1-Plus faible -> Plus élevé-10	○	○	○	○	○	○	○	○	○	○	○

* FP 4.9 L'utilisation des résultats d'évaluation par les gestionnaires de programmes : Mesure dans laquelle les gestionnaires locaux du programme de PF utilisent les conclusions de recherche et d'évaluations pour améliorer ces programmes en suivant les recommandations émises.

	1	2	3	4	5	6	7	8	9	10	Je ne sais pas
1-Plus faible -> Plus élevé-10	○	○	○	○	○	○	○	○	○	○	○

* FP 4.10 L'utilisation des résultats d'évaluation par les ministères : Mesure dans laquelle les administrateurs ministériels compétents utilisent systématiquement les données pour informer les politiques et interventions visant à résoudre les problèmes liés à la PF.

	1	2	3	4	5	6	7	8	9	10	Je ne sais pas
1-Plus faible -> Plus élevé-10	○	○	○	○	○	○	○	○	○	○	○

* FP 4.11 La dissémination d'informations à d'autres entités chargées de la mise en œuvre des programmes : Mesure dans laquelle des informations sont partagées ou disséminées entre géographies et à différents niveaux (national, état/province/comté, localités/sous-comtés).

	1	2	3	4	5	6	7	8	9	10	Je ne sais pas
1-Plus faible -> Plus élevé-10	○	○	○	○	○	○	○	○	○	○	○

FP 4.12 Commentaires : Veuillez utiliser cette section pour tout commentaire additionnel sur le niveau d'effort, les difficultés et les succès rencontrés dans le pays autour de la recherche sur la PF. Si vous avez répondu « Je ne sais pas » à l'une des questions, utilisez aussi cette section pour expliquer pourquoi.

JOHNS HOPKINS
BLOOMBERG SCHOOL
of PUBLIC HEALTH

Bill & Melinda Gates Institute for Population and Reproductive Health

Enquête de l'Indice des efforts pour le dividende démographique

Domaine 5. Organisations de la société civile (OSC)

* FP 5.1 Le pouvoir des acteurs des OSC : Mesure dans laquelle les acteurs des OSC occupent des postes d'influence dans les institutions, les groupes de travail techniques ou les réseaux concernés par la PF.

	1	2	3	4	5	6	7	8	9	10	Je ne sais pas
1-Plus faible -> Plus élevé-10	◯	◯	◯	◯	◯	◯	◯	◯	◯	◯	◯

* FP 5.2 L'analyse budgétaire comme outil des OSC : Mesure dans laquelle les OSC utilisent l'analyse budgétaire comme un outil pour développer le plaidoyer en matière de PF.

	1	2	3	4	5	6	7	8	9	10	Je ne sais pas
1-Plus faible -> Plus élevé-10	◯	◯	◯	◯	◯	◯	◯	◯	◯	◯	◯

* FP 5.3 Le soutien aux services offerts en structure sanitaire : Mesure dans laquelle les OSC sont impliquées dans les efforts visant à augmenter l'utilisation et/ou la qualité des services, particulièrement à travers une plus grande capacité technique et/ou opérationnelle des cliniques travaillant en partenariat avec des CSO pour fournir des services de PF.

	1	2	3	4	5	6	7	8	9	10	Je ne sais pas
1-Plus faible -> Plus élevé-10	◯	◯	◯	◯	◯	◯	◯	◯	◯	◯	◯

* FP 5.4 Le soutien aux services à base communautaire : Mesure dans laquelle les OSC participent au rapprochement des informations et services de PF avec les communautés à travers des activités telles que le renforcement de compétences des agents de santé communautaire (ASC), l'expansion de la gamme de méthodes contraceptives fournies par les ASC, la formation des cadres inférieurs de professionnels de la santé et/ou les campagnes d'information et d'éducation pour augmenter l'acceptabilité de la contraception.

	1	2	3	4	5	6	7	8	9	10	Je ne sais pas
1-Plus faible -> Plus élevé-10	○	○	○	○	○	○	○	○	○	○	○

* FP 5.5 Le soutien aux services à base communautaire à travers des campagnes mobiles de sensibilisation : Mesure dans laquelle les OSC soutiennent les modèles de services en dehors de cliniques pour que les prestataires de santé aient les commodités et produits de PF, l'équipement et les véhicules nécessaires pour être envoyés ensuite comme équipe spécialiste et fournir des informations et méthodes de PF directement aux communautés.

	1	2	3	4	5	6	7	8	9	10	Je ne sais pas
1-Plus faible -> Plus élevé-10	○	○	○	○	○	○	○	○	○	○	○

* FP 5.6 Mesure dans laquelle les OSC sont impliquées dans la franchise sociale des services de PF de qualité aux client(e)s à moindre coût.

	1	2	3	4	5	6	7	8	9	10	Je ne sais pas
1-Plus faible -> Plus élevé-10	○	○	○	○	○	○	○	○	○	○	○

* FP 5.7 La santé mobile : Mesure dans laquelle les OSC mettent à profit la technologie mobile, comme l'utilisation de la messagerie téléphonique (SMS), pour améliorer l'accès aux informations et services de PF.

	1	2	3	4	5	6	7	8	9	10	Je ne sais pas
1-Plus faible -> Plus élevé-10	○	○	○	○	○	○	○	○	○	○	○

* FP 5.8 Les droits humains, l'accès et la qualité de l'éducation : Mesure dans laquelle les OSC plaident pour des approches basées sur les droits et veillent à ce que les bénéficiaires des services de PF soit traités correctement, n'aient pas peur de poser des questions, et reçoivent des conseils de qualité, entre autres sujets d'importance.

	1	2	3	4	5	6	7	8	9	10	Je ne sais pas
1-Plus faible -> Plus élevé-10	○	○	○	○	○	○	○	○	○	○	○

* FP 5.9 Le changement social et des comportements (CSC) : Mesure dans laquelle les OSC soutiennent les interventions pour le CSC comprenant, mais sans s'y limiter, la promotion des compétences de vie et de l'éducation entre pairs, l'éducation sur la santé de l'adulte, l'éducation en milieu scolaire, la communication au sein du couple, la mobilisation communautaire, les interventions à travers les gardiens de la communauté et/ou les interventions médiatiques destinées au grand public. Ces interventions peuvent être mises en œuvre au niveau communautaire ou individuel, et avoir pour résultat une plus grande utilisation de la PF et des changements dans les connaissances et attitudes liées à la PF.

	1	2	3	4	5	6	7	8	9	10	Je ne sais pas
1-Plus faible -> Plus élevé-10	○	○	○	○	○	○	○	○	○	○	○

* FP 5.10 Les jeunes : Mesure dans laquelle les OSC soutiennent les politiques, interventions et programmes de PF ciblant les jeunes spécifiquement.

	1	2	3	4	5	6	7	8	9	10	Je ne sais pas
1-Plus faible -> Plus élevé-10	○	○	○	○	○	○	○	○	○	○	○

* FP 5.11 Les programmes pour les hommes : Mesure dans laquelle les OSC soutiennent des politiques, interventions et programmes de PF pour les hommes.

	1	2	3	4	5	6	7	8	9	10	Je ne sais pas
1-Plus faible -> Plus élevé-10	○	○	○	○	○	○	○	○	○	○	○

* FP 5.12 Le plaidoyer et la redevabilité : Mesure dans laquelle les OSC soutiennent la formation et le renforcement de compétences des plaideurs et des membres de la communauté pour améliorer leur compréhension des politiques et des processus d'identification de problèmes, de résolution collaborative de ces derniers, et des actions de plaidoyer plus ciblées pour améliorer les programmes de planification familiale.

	1	2	3	4	5	6	7	8	9	10	Je ne sais pas
1-Plus faible -> Plus élevé-10	○	○	○	○	○	○	○	○	○	○	○

* FP 5.13 L'évaluation et le suivi dirigés par les OSC : Mesure dans laquelle les OSC évaluent, suivent et émettent des rapports sur l'efficacité des politiques et programmes pour améliorer la redevabilité des prestataires de santé et des politiques de PF.

	1	2	3	4	5	6	7	8	9	10	Je ne sais pas
1-Plus faible -> Plus élevé-10	○	○	○	○	○	○	○	○	○	○	○

* FP 5.14 Les partenariats entre OSC : Mesure dans laquelle les OSC ont formé des alliances nationales et des partenariats régionaux pour renforcer leur position et potentiellement améliorer le leadership et les mécanismes de financement.

	1	2	3	4	5	6	7	8	9	10	Je ne sais pas
1-Plus faible -> Plus élevé-10	○	○	○	○	○	○	○	○	○	○	○

FP 5.15 Commentaires : Veuillez utiliser cette section pour tout commentaire additionnel sur le niveau d'effort, les difficultés et les succès rencontrés dans le pays autour des organisations de la société civile (OSC) pour la PF. Si vous avez répondu « Je ne sais pas » à l'une des questions, utilisez aussi cette section pour expliquer pourquoi.

JOHNS HOPKINS
BLOOMBERG SCHOOL
of PUBLIC HEALTH

Bill & Melinda Gates Institute for Population and Reproductive Health

Enquête de l'Indice des efforts pour le dividende démographique

Module : Résilience et durabilité du secteur

Étant donné l'impact sanitaire et socioéconomique de la pandémie de COVID-19, et son impact probable sur la progression vers le DD, cet indice des efforts pour le DD intègre des questions pour évaluer la résilience et la durabilité des systèmes dans les secteurs clés du DD. Les éléments couverts par les questions liées à la pandémie de COVID-19 ci-dessous relèvent des dimensions clés des systèmes résilients et visent à apporter des informations déterminantes sur le potentiel d'une réponse efficace aux menaces de maladies infectieuses émergentes et autres crises de santé publique.

Veuillez noter chaque élément sur une échelle de 1 à 10, 1 étant le score le plus faible (faible état/capacité) et 10 le plus élevé (très bon état/capacité).

* Dimension physique : structures, équipements, états du système et capacités

	1	2	3	4	5	6	7	8	9	10	Je ne sais pas
FP M1 - Plan/Préparation - État et capacité de l'équipement, du personnel et de la structure du programme de PF avant la crise. (1 = faible état/capacité ; 10 = très bon état/capacité)	◯	◯	◯	◯	◯	◯	◯	◯	◯	◯	◯
FP M2 - Absorption – Intégration : Niveau d'intégration des services et programmes de PF pour mitiger les effets de la pandémie de COVID-19 sur la PF.	◯	◯	◯	◯	◯	◯	◯	◯	◯	◯	◯
FP M3 - Absorption – Intégration : Mesure dans laquelle la gestion efficace de la chaîne d'approvisionnement a pu mitiger les effets de la pandémie de COVID-19 sur l'approvisionnement/distribution des commodités de PF.	◯	◯	◯	◯	◯	◯	◯	◯	◯	◯	◯
FP M4 - Récupération - Mesure dans laquelle le secteur de la PF a été capable d'induire des changements pour récupérer un niveau de fonctionnalité pré-COVID-19.	◯	◯	◯	◯	◯	◯	◯	◯	◯	◯	◯
FP M5 - Récupération – Gestion du financement et des bailleurs de fonds : Niveau de mobilisation des ressources et allocations pour mitiger les effets de la pandémie de COVID-19 sur la PF.	◯	◯	◯	◯	◯	◯	◯	◯	◯	◯	◯
FP M6 - Adaptation – Mesure dans laquelle des changements ont été effectués pour améliorer la résilience du secteur PF.	◯	◯	◯	◯	◯	◯	◯	◯	◯	◯	◯

* Dimension informatique : création, gestion, stockage et utilisation des données.

	1	2	3	4	5	6	7	8	9	10	Je ne sais pas
FP M7 - Plan/Préparation – Mesure dans laquelle les données liées à la PF étaient suffisamment préparées, présentées, analysées et stockées avant la crise.	○	○	○	○	○	○	○	○	○	○	○
FP M8 - Absorption – Connaissance : Mesure dans laquelle le programme de PF a pu conduire des évaluations en temps réel de son état de fonctionnement, en anticipation des pertes en cascade des services de PF.	○	○	○	○	○	○	○	○	○	○	○
FP M9 - Récupération – Mesure dans laquelle les données de la PF ont été utilisées pour suivre le progrès vers la récupération et anticiper des scenari de récupération.	○	○	○	○	○	○	○	○	○	○	○
FP M10 - Adaptation – Mesure dans laquelle le système de la PF crée et améliore actuellement ses protocoles de stockage et d'utilisation des données de PF en temps réel.	○	○	○	○	○	○	○	○	○	○	○

* Dimension cognitive : compréhension, modèles cognitifs, idées reçues, préjugés et valeurs.

	1	2	3	4	5	6	7	8	9	10	Je ne sais pas
FP M11 - Planification/Préparation – Mesure dans laquelle le système de la PF et les décisions opérationnelles étaient préparés à anticiper la crise.	○	○	○	○	○	○	○	○	○	○	○

	1	2	3	4	5	6	7	8	9	10	Je ne sais pas
FP M12 - Absorption – Mesure dans laquelle la réponse du secteur de la PF a des protocoles de contingence suffisants et une gestion de crise proactive.	○	○	○	○	○	○	○	○	○	○	○
FP M13 - Absorption – Redevabilité : Niveau d'effort pour assurer une redevabilité optimale vis-à-vis des ressources allouées à la PF dans le cadre de la réponse à la pandémie de COVID-19.	○	○	○	○	○	○	○	○	○	○	○
FP M14 - Récupération – Mesure dans laquelle les décisions sont orientées vers la récupération et communiquées aux communautés sur la base de données probantes pour promouvoir des comportements sûrs.	○	○	○	○	○	○	○	○	○	○	○
FP M15 - Récupération – Connaissance/Conscience : Mesure dans laquelle le secteur de la PF établit une certaine confiance à travers sa communication sur les barrières posées par la pandémie de COVID-19 pour soutenir l'accès et l'utilisation des services et programmes de PF.	○	○	○	○	○	○	○	○	○	○	○
FP M16 - Adaptation : Niveau d'adaptation pour répondre aux menaces posées par la pandémie de COVID-19 à la PF.	○	○	○	○	○	○	○	○	○	○	○

* Social dimension: interaction, collaboration and self-synchronization between people, entities and institutions.

	1	2	3	4	5	6	7	8	9	10	Je ne sais pas
FP M17 - Planification /Préparation – Mesure dans laquelle une formation sur la gestion d'épidémies/crises a été menée et a permis de mettre à profit les réseaux sociaux, le capital social et les normes institutionnelles et culturelles avant la crise.	○	○	○	○	○	○	○	○	○	○	○
FP M18 - Absorption – Mesure dans laquelle le personnel et les institutions sociales ont été accessibles et ont fait preuve d'ingéniosité dans leur réponse à l'épidémie/crise.	○	○	○	○	○	○	○	○	○	○	○
PF M19 - Absorption - Self-regulation: Extent to which national leaders had the authority to effect timely sectoral changes through an infrastructureflexible.	○	○	○	○	○	○	○	○	○	○	○
FP M20 - Récupération – Mesure dans laquelle le secteur de la PF est impliqué dans le partage de connaissances et d'équipes pour améliorer la récupération du système.	○	○	○	○	○	○	○	○	○	○	○
FP M21 - Récupération – Diversité : Niveau d'engagement d'une équipe multidisciplinaire pour mitiger les effets de la pandémie de COVID-19 sur la PF.	○	○	○	○	○	○	○	○	○	○	○
FP M22 - Adaptation – Mesure dans laquelle des ajouts ou changements sont apportés aux institutions, politiques, formations, programmes et à la culture de la PF.	○	○	○	○	○	○	○	○	○	○	○

	1	2	3	4	5	6	7	8	9	10	Je ne sais pas
FP M23 - Adaptation - Leadership et management : Niveau de leadership démontré par les dirigeants du secteur de la PF pour mitiger les effets de la pandémie de COVID-19 sur la PF.	◯	◯	◯	◯	◯	◯	◯	◯	◯	◯	◯

* FP M-24 Temporalité : Mesure de la rapidité de la réaction du gouvernement –sa communication et mise en œuvre des mesures- pour mitiger l'impact immédiat et sur le long terme de la pandémie de COVID-19. Cette question porte sur la riposte nationale dans son ensemble, et non la réponse spécifique de votre secteur.

	1	2	3	4	5	6	7	8	9	10	Je ne sais pas
1-Plus faible -> Plus élevé-10	◯	◯	◯	◯	◯	◯	◯	◯	◯	◯	◯

FP M-25 Commentaires : Veuillez utiliser cette section pour tout commentaire additionnel sur la résilience et la durabilité des systèmes de PF nationaux en fonction de votre expérience de la pandémie de COVID-19. Si vous avez répondu « Je ne sais pas » à l'une des questions, utilisez aussi cette section pour expliquer pourquoi.

JOHNS HOPKINS
BLOOMBERG SCHOOL
of PUBLIC HEALTH

Bill & Melinda Gates Institute for Population and Reproductive Health

Enquête de l'Indice des efforts pour le dividende démographique

Questionnaire sur la santé maternelle et infantile (SMI)

Répondez aux questions suivantes en fonction de votre expérience/ expertise dans ce secteur spécifique.

Afin d'obtenir une représentation synthétique des efforts nationaux pour le dividende démographique, notez les éléments suivants sur une échelle de 1 à 10, 1 étant le score le plus faible (efforts très faibles ou quasi-inexistants) et 10 le plus élevé (efforts robustes). Le cas échéant, si une politique ou activité n'existe pas, répondez 0.

Donnez un score à chaque élément. Toutes les réponses seront enregistrées au format illustré ci-dessous :

Effort le plus faible	1	2	3	4	5	6	7	8	9	10	Effort le plus élevé	Je ne sais pas

JOHNS HOPKINS
BLOOMBERG SCHOOL
of PUBLIC HEALTH

Bill & Melinda Gates Institute for Population and Reproductive Health

Enquête de l'Indice des efforts pour le dividende démographique

Domaine 1. Politique/ Prise de décision

* MCH 1.1 L'adéquation de la politique : Politiques du Ministère de la Santé en faveur des services pendant la grossesse et l'accouchement qui tiennent compte de toutes les conditions requises pour s'assurer que la mère et son nouveau-né survivent.

	1	2	3	4	5	6	7	8	9	10	Je ne sais pas
MCH 1.1.1 Mesure dans laquelle la santé reproductive, maternelle et infantile a été intégrée à la politique/plan/stratégie de santé nationale.	○	○	○	○	○	○	○	○	○	○	○
MCH 1.1.2 Mesure dans laquelle le/les plan(s) de mise en œuvre de la/les stratégie(s) nationale(s) de santé reproductive, maternelle et infantile sont disponibles.	○	○	○	○	○	○	○	○	○	○	○

* MCH 1.2 L'accès universel : Mesure dans laquelle des droits légaux et constitutionnels existent pour faciliter l'accès universel aux services de santé reproductive, maternelle et infantile.

	1	2	3	4	5	6	7	8	9	10	Je ne sais pas
1-Plus faible -> Plus élevé-10	○	○	○	○	○	○	○	○	○	○	○

* MCH 1.3 L'implication de divers secteurs : Mesure dans laquelle des politiques sont élaborées à travers une consultation adéquate des parties intéressées, dont les ministères, les ONG, les praticiens privés et les associations de femmes.

	1	2	3	4	5	6	7	8	9	10	Je ne sais pas
1-Plus faible -> Plus élevé-10	○	○	○	○	○	○	○	○	○	○	○

* MCH 1.4 La compétence des prestataires de santé : Mesure dans laquelle les politiques sont rationnelles et fondées sur des données probantes établissant quel personnel peut fournir des services de santé maternelle (ex : les sages-femmes formées peuvent effectuer une vaste gamme de procédures).

	1	2	3	4	5	6	7	8	9	10	Je ne sais pas
1-Plus faible -> Plus élevé-10	○	○	○	○	○	○	○	○	○	○	○

* MCH 1.5 La légalisation de l'avortement : Mesure dans laquelle une politique favorable existe pour assurer l'offre légale de services d'avortement.

	1	2	3	4	5	6	7	8	9	10	Je ne sais pas
1-Plus faible -> Plus élevé-10	○	○	○	○	○	○	○	○	○	○	○

* MCH 1.6. La légalisation des soins post-avortement : Mesure dans laquelle une politique favorable existe pour le traitement des complications dues aux avortements, y compris les complications résultant d'avortements clandestins.

	1	2	3	4	5	6	7	8	9	10	Je ne sais pas
1-Plus faible -> Plus élevé-10	○	○	○	○	○	○	○	○	○	○	○

* MCH 1.7 La révision et mise à jour des politiques : Mesure dans laquelle les politiques sont régulièrement revues par des législateurs de haut niveau et utilisées dans les plans d'action.

	1	2	3	4	5	6	7	8	9	10	Je ne sais pas
1-Plus faible -> Plus élevé-10	○	○	○	○	○	○	○	○	○	○	○

* MCH 1.8 Le haut placement des acteurs : Mesure dans laquelle le/la directeur/trice des services de santé maternelle et infantile est haut placé(e) au gouvernement et impliqué(e) dans la prise de décision.

	1	2	3	4	5	6	7	8	9	10	Je ne sais pas
1-Plus faible -> Plus élevé-10	○	○	○	○	○	○	○	○	○	○	○

* MCH 1.9 Le financement de la SMI : Mesure dans laquelle les budgets du gouvernement pour la grossesse et l'accouchement sans risque, ainsi que pour les soins périnataux (pour les structures de santé, le personnel, les produits, etc.) et la santé infantile sont adaptés aux besoins, et si ces financements proviennent du Ministère de la Santé, gouvernement provincial/ des comtés ou des bailleurs de fonds.

	1	2	3	4	5	6	7	8	9	10	Je ne sais pas
1-Plus faible -> Plus élevé-10	◯	◯	◯	◯	◯	◯	◯	◯	◯	◯	◯

* MCH 1.10 L'accessibilité financière : Mesure dans laquelle les services et médicaments liés à la SMI sont à un prix abordable pour toutes les clientes.

	1	2	3	4	5	6	7	8	9	10	Je ne sais pas
1-Plus faible -> Plus élevé-10	◯	◯	◯	◯	◯	◯	◯	◯	◯	◯	◯

* MCH 1.11 Le secteur privé : Mesure dans laquelle le secteur privé (médecins, sages-femmes, cliniques) est actif et couvre une part importance des soins de grossesse et d'accouchement.

	1	2	3	4	5	6	7	8	9	10	Je ne sais pas
1-Plus faible -> Plus élevé-10	◯	◯	◯	◯	◯	◯	◯	◯	◯	◯	◯

* MCH 1.12 Le directorat de SMI : Mesure dans laquelle le Ministère de la Santé a un directorat spécifiquement dédié à la SMI.

	1	2	3	4	5	6	7	8	9	10	Je ne sais pas
1-Plus faible -> Plus élevé-10	◯	◯	◯	◯	◯	◯	◯	◯	◯	◯	◯

* MCH 1.13 Les solutions potentielles : Mesure dans laquelle le gouvernement reconnait des solutions potentielles aux problèmes de SMI par les moyens suivants :

	1	2	3	4	5	6	7	8	9	10	Je ne sais pas
MCH 1.13.1 Efficience régulatrice	◯	◯	◯	◯	◯	◯	◯	◯	◯	◯	◯
MCH 1.13.2 Performance et redevabilité	◯	◯	◯	◯	◯	◯	◯	◯	◯	◯	◯
MCH 1.13.3 Approche fondée sur le droit de la SMNI	◯	◯	◯	◯	◯	◯	◯	◯	◯	◯	◯
MCH 1.13.4 Infrastructure de santé essentielle	◯	◯	◯	◯	◯	◯	◯	◯	◯	◯	◯
MCH 1.13.5 Engagement de la communauté	◯	◯	◯	◯	◯	◯	◯	◯	◯	◯	◯

MCH 1.14 Commentaires : Utilisez cette section pour apporter des commentaires additionnels sur le niveau d’effort, les défis et les succès du pays autour de la politique/ prise de décision pour la SMI. Si vous avez répondu « Je ne sais pas » à l’une des questions précédentes, utilisez aussi cette section pour expliquer pourquoi.

JOHNS HOPKINS BLOOMBERG SCHOOL of PUBLIC HEALTH

Bill & Melinda Gates Institute for Population and Reproductive Health

Enquête de l’Indice des efforts pour le dividende démographique

Dimension 2. Services or Programs

* MCH 2.1 La disponibilité des soins obstétriques – Dans tous les centres : Mesure dans laquelle les structures de santé primaire ont du personnel qualifié, en place, et peuvent fournir des soins obstétriques :

	1	2	3	4	5	6	7	8	9	10	Je ne sais pas
MCH 2.1.1 Cas d’hémorragie postpartum adéquatement gérés	○	○	○	○	○	○	○	○	○	○	○
MCH 2.1.2 Administration adéquate et opportune d’antibiotiques en intraveineuse lorsque nécessaire.	○	○	○	○	○	○	○	○	○	○	○
MCH 2.1.3 Expulsion manuelle dans les cas de rétention placentaire.	○	○	○	○	○	○	○	○	○	○	○
MCH 2.1.4 Aspiration de l’utérus manuelle (Méthode de Karman) ou à l’aide d’un appareil à succion électrique.	○	○	○	○	○	○	○	○	○	○	○
MCH 2.1.5 Utilisation d’un partogramme pour savoir quand référer.	○	○	○	○	○	○	○	○	○	○	○
MCH 2.1.6 Transport organized to quickly transfer a woman with obstructed labor to the district / sub-county hospital.	○	○	○	○	○	○	○	○	○	○	○
MCH 2.1.7 Antibiotiques adéquats disponibles (suffisamment de produits du bon type).	○	○	○	○	○	○	○	○	○	○	○

* MCH 2.2 La disponibilité des soins obstétriques – Les structures de santé secondaires : Mesure dans laquelle tous les hôpitaux de districts/sous-comtés ont du personnel formé, en place, capable de :

	1	2	3	4	5	6	7	8	9	10	Je ne sais pas
MCH 2.2.1 Remplir toutes les fonctions citées ci-dessus pour les structures de santé primaires	◯	◯	◯	◯	◯	◯	◯	◯	◯	◯	◯
MCH 2.2.2 Effectuer des transfusions sanguines (et avoir un approvisionnement en sang sûr, adéquat et disponible).	◯	◯	◯	◯	◯	◯	◯	◯	◯	◯	◯
MCH 2.2.3 Effectuer des césariennes.	◯	◯	◯	◯	◯	◯	◯	◯	◯	◯	◯

* MCH 2.3 Les services liés à la grossesse : Mesure dans laquelle toutes les femmes enceintes ont accès à :

	1	2	3	4	5	6	7	8	9	10	Je ne sais pas
MCH 2.3.1 Traitement des hémorragies postpartum pendant ou peu après l'accouchement.	○	○	○	○	○	○	○	○	○	○	○
MCH 2.3.2 Gestion de la dystocie.	○	○	○	○	○	○	○	○	○	○	○
MCH 2.3.3 Management de la pré-éclampsie, éclampsie et ses complications.	○	○	○	○	○	○	○	○	○	○	○
MCH 2.3.4 Soins post-avortement ou pour les complications liées aux avortements.	○	○	○	○	○	○	○	○	○	○	○
MCH 2.3.5 Offre de services d'avortement sans risque.	○	○	○	○	○	○	○	○	○	○	○
MCH 2.3.6 Soins prénatals pendant la grossesse.	○	○	○	○	○	○	○	○	○	○	○
MCH 2.3.7 Soins d'accouchement par un professionnel formé.	○	○	○	○	○	○	○	○	○	○	○
MCH 2.3.8 Services de planification familiale postpartum.	○	○	○	○	○	○	○	○	○	○	○
MCH 2.3.9 Hôpitaux des districts/ sous-comtés ouverts 24h/24 et 7j/7.	○	○	○	○	○	○	○	○	○	○	○

* MCH 2.4 Les soins prénatals pour les femmes non mariées et séropositives : Mesure dans laquelle, à toutes les consultations prénatales, toutes les femmes enceintes :

	1	2	3	4	5	6	7	8	9	10	Je ne sais pas
MCH 2.4.1 Reçoivent des soins prénatals complets conformément aux protocoles recommandés par l'OMS pour les femmes séronégatives.	◯	◯	◯	◯	◯	◯	◯	◯	◯	◯	◯
MCH 2.4.2 Reçoivent des soins prénatals complets conformément aux protocoles recommandés par l'OMS pour les femmes séropositives.	◯	◯	◯	◯	◯	◯	◯	◯	◯	◯	◯
MCH 2.4.3 Reçoivent des soins prénatals complets conformément aux protocoles recommandés par l'OMS pour les jeunes femmes non mariées.	◯	◯	◯	◯	◯	◯	◯	◯	◯	◯	◯
MCH 2.4.4 Sont informées des signes de danger de complications obstétriques et néonatales, et sont assistées pour planifier toute urgence éventuelle.	◯	◯	◯	◯	◯	◯	◯	◯	◯	◯	◯

* MCH 2.5 Les soins néonatals : Mesure dans laquelle, pour les soins néonatals, tous les nouveau-nés, qu'ils soient nés à la maison ou en structure médicale :

	1	2	3	4	5	6	7	8	9	10	Je ne sais pas
MCH 2.5.1 Ont leur bouche et voie nasale dégagées.	○	○	○	○	○	○	○	○	○	○	○
MCH 2.5.2 Sont séchés et gardés au chaud immédiatement après la naissance.	○	○	○	○	○	○	○	○	○	○	○
MCH 2.5.3 Reçoivent de la vitamine A.	○	○	○	○	○	○	○	○	○	○	○
MCH 2.5.4 Ont leur cordon ombilical coupé avec une lame propre.	○	○	○	○	○	○	○	○	○	○	○
MCH 2.5.5 Suivent le calendrier de vaccination recommandé par l'OMS.	○	○	○	○	○	○	○	○	○	○	○
MCH 2.5.6 Bénéficient des activités de promotion de l'allaitement exclusif.	○	○	○	○	○	○	○	○	○	○	○
MCH 2.5.7 Bénéficient des programmes de soutien alimentaire lorsqu'ils en ont besoin.	○	○	○	○	○	○	○	○	○	○	○

* MCH 2.6 La planification familiale – Dans tous les centres : Mesure dans laquelle la planification familiale est dûment fournie dans tous les centres de santé en :

	1	2	3	4	5	6	7	8	9	10	Je ne sais pas
MCH 2.6.1 Offrant régulièrement des services de planification familiale aux cas post-avortement.	○	○	○	○	○	○	○	○	○	○	○
MCH 2.6.2 Offrant régulièrement des services de planification familiale lors des consultations postpartum.	○	○	○	○	○	○	○	○	○	○	○
MCH 2.6.3 Ayant des méthodes contraceptives réversibles de courte et longue durée régulièrement en stock.	○	○	○	○	○	○	○	○	○	○	○
MCH 2.6.4 Ayant du personnel formé, sur place, pouvant fournir les méthodes LARC.	○	○	○	○	○	○	○	○	○	○	○
MCH 2.6.5 Suivant les directives et protocoles nécessaires à la prestation de services de planification familiale.	○	○	○	○	○	○	○	○	○	○	○

* MCH 2.7 La planification familiale – Mesure dans laquelle la planification familiale est dûment fournie dans tous hôpitaux de districts/sous-comtés en :

	1	2	3	4	5	6	7	8	9	10	Je ne sais pas
MCH 2.7.1 Offrant régulièrement des services de planification familiale aux cas post-avortement.	○	○	○	○	○	○	○	○	○	○	○
MCH 2.7.2 Offrant régulièrement des services de planification familiale lors des consultations postpartum.	○	○	○	○	○	○	○	○	○	○	○
MCH 2.7.3 Ayant des méthodes contraceptives réversibles de courte et longue durée régulièrement en stock.	○	○	○	○	○	○	○	○	○	○	○
MCH 2.7.4 Ayant du personnel formé, sur place, pouvant fournir les méthodes LARC.	○	○	○	○	○	○	○	○	○	○	○
MCH 2.7.5 Pouvant offrir la stérilisation féminine.	○	○	○	○	○	○	○	○	○	○	○
MCH 2.7.6 Pouvant offrir la stérilisation masculine.	○	○	○	○	○	○	○	○	○	○	○
MCH 2.7.7 Suivant les directives et protocoles nécessaires à la prestation de services de planification familiale.	○	○	○	○	○	○	○	○	○	○	○

* MCH 2.8 Les maladies infantiles : Mesure dans laquelle la gestion de maladies infantiles recommandée par l'OMS est mise en œuvre en termes de :

	1	2	3	4	5	6	7	8	9	10	Je ne sais pas
MCH 2.8.1 Gestion à base communautaire des maladies infantiles.	○	○	○	○	○	○	○	○	○	○	○
MCH 2.8.2 Efficacité des services de référence du niveau communautaire vers les structures de santé.	○	○	○	○	○	○	○	○	○	○	○

MCH 2.9 Commentaires : Utilisez cette section pour apporter des commentaires additionnels sur le niveau d'effort, les défis et les succès du pays autour des programmes ou services de SMI. Si vous avez répondu « Je ne sais pas » à l'une des questions précédentes, utilisez aussi cette section pour expliquer pourquoi.

JOHNS HOPKINS BLOOMBERG SCHOOL of PUBLIC HEALTH

Bill & Melinda Gates Institute for Population and Reproductive Health

Enquête de l'Indice des efforts pour le dividende démographique

Domaine 3. Le plaidoyer

* MCH 3.1 Les messages du gouvernement : Mesure dans laquelle les hauts fonctionnaires au gouvernement, dont au Ministère de la Santé, s'adressent fréquemment à toutes les parties prenantes, y compris la presse, pour soutenir des améliorations et promouvoir les grossesses et les accouchements sans risque, et les enfants en bonne santé.

	1	2	3	4	5	6	7	8	9	10	Je ne sais pas
1-Plus faible -> Plus élevé-10	○	○	○	○	○	○	○	○	○	○	○

* MCH 3.2 Les médias de masse : Mesure dans laquelle le programme national utilise les médias de masse pour éduquer le public sur les symptômes des complications liées aux grossesses, sur les accouchements sans risque et les enfants en bonne santé.

	1	2	3	4	5	6	7	8	9	10	Je ne sais pas
1-Plus faible -> Plus élevé-10	○	○	○	○	○	○	○	○	○	○	○

* MCH 3.3 Les médias au niveau communautaire : Mesure dans laquelle les groupes communautaires prennent part aux programmes systématiques pour éduquer le public sur les grossesses et accouchements sans risque et les enfants en bonne santé.

	1	2	3	4	5	6	7	8	9	10	Je ne sais pas
1-Plus faible -> Plus élevé-10	○	○	○	○	○	○	○	○	○	○	○

* MCH 3.4 Les supports éducatifs : Mesure dans laquelle le ministère compétent (Ministère de la Santé) fournit des supports éducatifs adéquats (posters, brochures, etc.) aux structures de santé pour qu'elles instruisent les client(e)s sur les pratiques sûres/sans risque.

	1	2	3	4	5	6	7	8	9	10	Je ne sais pas
1-Plus faible -> Plus élevé-10	○	○	○	○	○	○	○	○	○	○	○

* MCH 3.5 Auto-révision des cas de mortalité maternelle : Mesure dans laquelle chaque structure de santé suit une procédure régulière de révision de tous ses cas de décès maternels ayant eu lieu dans la structure pour en tirer des leçons.

	1	2	3	4	5	6	7	8	9	10	Je ne sais pas
1-Plus faible -> Plus élevé-10	○	○	○	○	○	○	○	○	○	○	○

* MCH 3.6 Informer les politiques avec de nouvelles données probantes : Mesure dans laquelle chaque structure de santé transmet les leçons tirées des cas de décès/ mortalité maternelle aux ministères pour informer les nouvelles politiques et interventions.

	1	2	3	4	5	6	7	8	9	10	Je ne sais pas
1-Plus faible -> Plus élevé-10	○	○	○	○	○	○	○	○	○	○	○

MCH 3.7 Commentaires : Utilisez cette section pour apporter des commentaires additionnels sur le niveau d'effort, les défis et les succès du pays autour du plaidoyer pour la SMI. Si vous avez répondu « Je ne sais pas » à l'une des questions précédentes, utilisez aussi cette section pour expliquer pourquoi.

JOHNS HOPKINS
BLOOMBERG SCHOOL of PUBLIC HEALTH

Bill & Melinda Gates Institute for Population and Reproductive Health

Enquête de l'Indice des efforts pour le dividende démographique

Domaine 4. Recherche

* MCH 4.1 La force des observatoires/parties prenantes/groupes de travail pour la SMI : Mesure dans laquelle les parties prenantes de la SMI du pays (ex : groupe directif de parties prenantes, groupes de travail techniques, observatoire de SMI, groupe de coordination ou facilitation) :

	1	2	3	4	5	6	7	8	9	10	Je ne sais pas
MCH 4.1.1 Ont un représentant du gouvernement, des institutions de formation, de la société civile, des organisations non gouvernementales et confessionnelles, des associations professionnelles, du secteur privé, etc.	◯	◯	◯	◯	◯	◯	◯	◯	◯	◯	◯
MCH 4.1.2 Se réunissent régulièrement, émettent des rapports et recommandent des politiques aux directions des ministères compétents.	◯	◯	◯	◯	◯	◯	◯	◯	◯	◯	◯
MCH 4.1.3 Ont un impact sur l'éducation dans le pays.	◯	◯	◯	◯	◯	◯	◯	◯	◯	◯	◯

* MCH 4.2 MCH La stratégie d'éducation/recherche : Mesure dans laquelle le plan national de SMI et/ou d'autres documents nationaux comprennent une stratégie/approche complète de recherche liée sur la SMI et est partagée entre partenaires.

	1	2	3	4	5	6	7	8	9	10	Don't know
1-Plus faible -> Plus élevé-10	◯	◯	◯	◯	◯	◯	◯	◯	◯	◯	◯

* MCH 4.3 Les partenaires collectant les données:

	1	2	3	4	5	6	7	8	9	10	Je ne sais pas
MCH 4.3.1 Mesure dans laquelle la collecte des données est entreprise par des agences gouvernementales. Cela peut comprendre les agences/bureaux de la statistique et/ou les ministères de l'économie/finances, éducation, santé, genre, etc.).	◯	◯	◯	◯	◯	◯	◯	◯	◯	◯	◯
MCH 4.3.2 Mesure dans laquelle la collecte de données est entreprise par des institutions de recherche.	◯	◯	◯	◯	◯	◯	◯	◯	◯	◯	◯
MCH 4.3.3 Mesure dans laquelle la collecte des données est entreprise par des chercheurs indépendants.	◯	◯	◯	◯	◯	◯	◯	◯	◯	◯	◯

* MCH 4.4 La recherche thématique :

	1	2	3	4	5	6	7	8	9	10	Je ne sais pas
MCH 4.4.1 Mesure dans laquelle de la recherche est menée sur la mortalité maternelle.	◯	◯	◯	◯	◯	◯	◯	◯	◯	◯	◯
MCH 4.4.2 Mesure dans laquelle de la recherche est menée sur la mortalité néonatale et infantile.	◯	◯	◯	◯	◯	◯	◯	◯	◯	◯	◯
MCH 4.4.3 Mesure dans laquelle de la recherche est menée sur la planification familiale et la planification familiale postpartum.	◯	◯	◯	◯	◯	◯	◯	◯	◯	◯	◯
MCH 4.4.4 Mesure dans laquelle de la recherche est menée sur la prestation/accès aux services et la qualité des prestataires de SMI.	◯	◯	◯	◯	◯	◯	◯	◯	◯	◯	◯

* MCH 4.5 La qualité/couverture des données : Mesure dans laquelle la recherche/les données actuelles sont désagrégées par sexe, âge, lieu, ethnicité, niveau d'études, quintile de revenus et statut d'handicap. Mesure dans laquelle le pays participe à des tests standardisés et son classement.

	1	2	3	4	5	6	7	8	9	10	Je ne sais pas
MCH 4.5.1 Mesure dans laquelle un système statistique de routine offre de bonnes données périodiques sur les produits/l'approvisionnement et les structures de santé.	○	○	○	○	○	○	○	○	○	○	○
MCH 4.5.2 Mesure dans laquelle un système statistique de routine offre de bonnes données périodiques sur les services et le personnel de SMI.	○	○	○	○	○	○	○	○	○	○	○
MCH 4.5.3 Mesure dans laquelle un système statistique de routine offre de bonnes données périodiques sur les besoins en SMI de différentes populations dans les communautés.	○	○	○	○	○	○	○	○	○	○	○
MCH 4.5.4 4 Mesure dans laquelle des évaluations du système de suivi de l'éducation sont menées et appliquées pour assurer la fiabilité des données.	○	○	○	○	○	○	○	○	○	○	○
MCH 4.5.5 Mesure dans laquelle des évaluations du système de suivi de l'éducation sont menées et appliquées pour explorer les inégalités dans les services et fardeaux de la SMNI.	○	○	○	○	○	○	○	○	○	○	○

* MCH 4.6 Les registres : Mesure dans laquelle les systèmes de registres, rapports et feedback sur les résultats de SMI sont adéquats, y compris en étant connectés à tous les niveaux (fédéral, état/province, comté, etc.).

	1	2	3	4	5	6	7	8	9	10	Je ne sais pas
1-Plus faible -> Plus élevé-10	○	○	○	○	○	○	○	○	○	○	○

* MCH 4.7 La qualité des institutions de recherche : Mesure dans laquelle le pays a la capacité de soutenir et maintenir des institutions de recherche qui développent des études et/ou collectent des données permettant des services SMI de haute qualité.

	1	2	3	4	5	6	7	8	9	10	Je ne sais pas
1-Plus faible -> Plus élevé-10	◯	◯	◯	◯	◯	◯	◯	◯	◯	◯	◯

* MCH 4.8 L'évaluation : Mesure dans laquelle les statistiques, enquêtes et études sur le programme de SMI sont utilisées par du personnel spécialisé pour rapporter les opérations du programme et mesurer le progrès.

	1	2	3	4	5	6	7	8	9	10	Je ne sais pas
1-Plus faible -> Plus élevé-10	◯	◯	◯	◯	◯	◯	◯	◯	◯	◯	◯

* MCH 4.9 L'utilisation des résultats d'évaluations par les gestionnaires de programmes : Mesure dans laquelle les gestionnaires des programme locaux utilisent les conclusions de recherche et d'évaluations pour améliorer ces programmes en suivant les recommandations émises.

	1	2	3	4	5	6	7	8	9	10	Je ne sais pas
1-Plus faible -> Plus élevé-10	◯	◯	◯	◯	◯	◯	◯	◯	◯	◯	◯

* MCH 4.10 L'utilisation des résultats d'évaluation par les ministères : Mesure dans laquelle les administrateurs ministériels compétents et responsables d'élaborer les politiques utilisent systématiquement les données pour informer les politiques et interventions visant à résoudre les problèmes liés à la SMI.

	1	2	3	4	5	6	7	8	9	10	Je ne sais pas
1-Plus faible -> Plus élevé-10	◯	◯	◯	◯	◯	◯	◯	◯	◯	◯	◯

* MCH 4.11 La dissémination d'informations à d'autres entités chargées de la mise en œuvre des programmes : Mesure dans laquelle les informations sont communiquées ou disséminées entre différentes aires géographiques et à différents niveaux (national, état/province, collectivités territoriales).

	1	2	3	4	5	6	7	8	9	10	Je ne sais pas
1-Plus faible -> Plus élevé-10	◯	◯	◯	◯	◯	◯	◯	◯	◯	◯	◯

MCH 4.12 Commentaires : Utilisez cette section pour apporter des commentaires additionnels sur le niveau d'effort, les défis et les succès du pays autour de la recherche sur la SMI. Si vous avez répondu « Je ne sais pas » à l'une des questions précédentes, utilisez aussi cette section pour expliquer pourquoi.

JOHNS HOPKINS
BLOOMBERG SCHOOL
of PUBLIC HEALTH

Bill & Melinda Gates Institute for Population and Reproductive Health

Enquête de l'Indice des efforts pour le dividende démographique

Domaine 5. Organisations de la société civile (OSC)

* MCH 5.1 Le pouvoir des acteurs des OSC : Mesure dans laquelle les acteurs des OSC occupent des postes d'influence dans les institutions, les groupes de travail techniques ou les réseaux concernés par la SMI.

	1	2	3	4	5	6	7	8	9	10	Je ne sais pas
1-Plus faible -> Plus élevé-10	○	○	○	○	○	○	○	○	○	○	○

* MCH 5.2 L'analyse budgétaire comme outil des OSC : Mesure dans laquelle les OSC utilisent l'analyse budgétaire comme un outil pour développer le plaidoyer en matière de SMI.

	1	2	3	4	5	6	7	8	9	10	Je ne sais pas
1-Plus faible -> Plus élevé-10	○	○	○	○	○	○	○	○	○	○	○

* MCH 5.3 Le soutien à la prestation de services en structure sanitaire : Mesure dans laquelle les OSC sont impliquées dans des efforts visant à augmenter l'utilisation et/ou la qualité des services, particulièrement à travers l'augmentation des capacités techniques et/ou opérationnelles des cliniques partenaires des OSC afin de fournir des services de SMI.

	1	2	3	4	5	6	7	8	9	10	Je ne sais pas
1-Plus faible -> Plus élevé-10	○	○	○	○	○	○	○	○	○	○	○

* MCH 5.4 Le soutien à la prestation des services à base communautaire : Mesure dans laquelle les OSC participent au rapprochement des informations et services de SMI avec les communautés à travers des activités comprenant le renforcement de compétences des agents de santé communautaire (ASC), l’expansion de la gamme de services SMI offerts par les ASC, l’orientation/éducation des travailleurs de la santé de cadres inférieurs et/ou des campagnes d’information ou d’éducation.

	1	2	3	4	5	6	7	8	9	10	Je ne sais pas
1-Plus faible -> Plus élevé-10											

* MCH 5.5 Le soutien à la sensibilisation mobile pour l’offre de services à base communautaire : Mesure dans laquelle les OSC soutiennent les modèles de sensibilisation mobile pour équiper les prestataires de santé en leur fournissant des commodités et produits liés à la SMI, de l’équipement et des véhicules afin de fournir en tant qu’équipe spécialiste des services SMI directement aux communautés.

	1	2	3	4	5	6	7	8	9	10	Je ne sais pas
1-Plus faible -> Plus élevé-10											

* MCH 5.6 La franchise sociale : Mesure dans laquelle les OSC sont impliquées dans des opportunités/ services de franchise sociale pour offrir des services de SMI dans le secteur privé à un prix réduit.

	1	2	3	4	5	6	7	8	9	10	Je ne sais pas
1-Plus faible -> Plus élevé-10											

* MCH 5.7 La santé mobile : Mesure dans laquelle les OSC mettent à profit la technologie mobile en utilisant la messagerie (textos) et autres technologies mobiles pour améliorer l’accès aux informations et services de SMI.

	1	2	3	4	5	6	7	8	9	10	Je ne sais pas
1-Plus faible -> Plus élevé-10											

* MCH 5.8 Les droits humains et la qualité des soins : Mesure dans laquelle les OSC soutiennent et plaident pour des approches fondées sur le droit pour s’assurer que les clientes de la SMI soient bien traitées, n’aient pas peur de poser des questions et reçoivent du conseil de qualité, entre autres sujets.

	1	2	3	4	5	6	7	8	9	10	Je ne sais pas
1-Plus faible -> Plus élevé-10											

* MCH 5.9 Le changement social et des comportements (CSC) : Mesure dans laquelle les OSC soutiennent les interventions pour le CSC comprenant, mais sans s'y limiter, les compétences de vie et l'éducation entre pairs, l'éducation sur la santé de l'adulte, l'éducation en milieu scolaire, la communication au sein du couple, la mobilisation communautaire, les interventions à travers les gardiens de la communauté et/ou dans les médias de masse pour le grand public.

	1	2	3	4	5	6	7	8	9	10	Je ne sais pas
1-Plus faible -> Plus élevé-10	○	○	○	○	○	○	○	○	○	○	○

* MCH 5.10 Le plaidoyer/ la redevabilité : Mesure dans laquelle les OSC soutiennent la formation et le renforcement de compétences des plaideurs et des membres de la communauté pour améliorer leur compréhension des politiques et processus, leurs activités d'identification de problèmes, la résolution collaborative de ces problèmes, et des actions de plaidoyer plus ciblées pour améliorer la SMI.

	1	2	3	4	5	6	7	8	9	10	Je ne sais pas
1-Plus faible -> Plus élevé-10	○	○	○	○	○	○	○	○	○	○	○

* MCH 5.11 L'évaluation et le suivi dirigés par les OSC : Mesure dans laquelle les OSC évaluent, suivent et émettent des rapports sur l'efficacité des politiques et programmes pour améliorer la redevabilité des prestataires et des politiques de SMI.

	1	2	3	4	5	6	7	8	9	10	Je ne sais pas
1-Plus faible -> Plus élevé-10	○	○	○	○	○	○	○	○	○	○	○

* MCH 5.12 Les partenariats entre OSC : Mesure dans laquelle les OSC ont formé des alliances nationales et des partenariats régionaux pour renforcer leur position et potentiellement améliorer la force du leadership et les mécanismes de financement pour la SMI.

	1	2	3	4	5	6	7	8	9	10	Je ne sais pas
1-Plus faible -> Plus élevé-10	○	○	○	○	○	○	○	○	○	○	○

MCH 5.13 Commentaires : Utilisez cette section pour apporter des commentaires additionnels sur le niveau d'effort, les défis et les succès des OSC travaillant pour la SMI. Si vous avez répondu « Je ne sais pas » à l'une des questions précédentes, utilisez aussi cette section pour expliquer pourquoi.

JOHNS HOPKINS
BLOOMBERG SCHOOL
of PUBLIC HEALTH

Bill & Melinda Gates Institute for Population and Reproductive Health

Enquête de l'Indice des efforts pour le dividende démographique

Module : Résilience et durabilité du secteur

Étant donné l'impact sanitaire et socioéconomique de la pandémie de COVID-19, et son impact probable sur la progression vers le DD, cet indice des efforts pour le DD intègre des questions pour évaluer la résilience et la durabilité des systèmes dans les secteurs clés du DD. Les éléments couverts par les questions liées à la pandémie de COVID-19 ci-dessous relèvent des dimensions clés des systèmes résilients et visent à apporter des informations déterminantes sur le potentiel d'une réponse efficace aux menaces de maladies infectieuses émergeantes et autres crises de santé publique.

Veuillez noter chaque élément sur une échelle de 1 à 10, 1 étant le score le plus faible (faible état/capacité) et 10 le plus élevé (très bon état/capacité).

* Dimension physique : structures, équipements, états du système et capacités.

	1	2	3	4	5	6	7	8	9	10	Je ne sais pas
MCH-M1 - Planification/Préparation – État et capacité de l'équipement, du personnel et de la structure du programme de SMI avant la crise.	○	○	○	○	○	○	○	○	○	○	○
MCH-M2 – Absorption – Intégration : Niveau d'intégration des services et programmes de SMI pour mitiger les effets de la pandémie de COVID-19 sur la SMI.	○	○	○	○	○	○	○	○	○	○	○

	1	2	3	4	5	6	7	8	9	10	Je ne sais pas
MCH-M3 - Absorption – Intégration : Mesure dans laquelle la gestion efficace de la chaîne d'approvisionnement a permis de mitiger les effets de la pandémie de COVID-19 sur l'approvisionnement/livraison des commodités liées à la SMI.	○	○	○	○	○	○	○	○	○	○	○
MCH-M4 - Récupération – Mesure dans laquelle le programme de SMI a été capable d'induire des changements pour récupérer un niveau de fonctionnalité pre-COVID-19.	○	○	○	○	○	○	○	○	○	○	○
MCH-M5 - Récupération – Gestion du financement et des bailleurs de fonds : Niveau de mobilisation de ressources et d'allocation à la mitigation des effets de la pandémie de COVID-19 sur la SMI.	○	○	○	○	○	○	○	○	○	○	○
MCH-M6 - Adaptation – Mesure dans laquelle des changements ont été effectués pour améliorer la résilience du secteur SMI.	○	○	○	○	○	○	○	○	○	○	○

* Dimension informatique : création, gestion, stockage et utilisation des données.

	1	2	3	4	5	6	7	8	9	10	Don't know
MCH-M7 - Planification/Préparation - Mesure dans laquelle les données liées à la SMI étaient suffisamment préparées, présentées, analysées et stockées avant la crise.	○	○	○	○	○	○	○	○	○	○	○
MCH-M8 - Absorption – Connaissance/Conscience : Mesure dans laquelle la capacité de surveillance a pu détecter les limites/menaces posées par la pandémie de COVID-19 à la SMI.	○	○	○	○	○	○	○	○	○	○	○
MCH-M9 - Récupération – Mesure dans laquelle les données de SMI ont été utilisées pour suivre le progrès vers la récupération et anticiper des scenarii de récupération.	○	○	○	○	○	○	○	○	○	○	○
MCH-M10 - Adaptation – Mesure dans laquelle le système de SMI crée et améliore actuellement ses protocoles de stockage et d’utilisation de ses données en temps réel.	○	○	○	○	○	○	○	○	○	○	○

* Dimension cognitive: compréhension, modèles cognitifs, préconception, biais et valeurs.

	1	2	3	4	5	6	7	8	9	10	Je ne sais pas
MCH-M11 - Planification/Préparation - Mesure dans laquelle les décisions de conceptualisation et opérationnelle du secteur SMI étaient préparées à anticiper la crise.	○	○	○	○	○	○	○	○	○	○	○
MCH-M12 - Absorption - Mesure dans laquelle le secteur de la SMI a des protocoles de contingence suffisants et une gestion de crise proactive.	○	○	○	○	○	○	○	○	○	○	○

	1	2	3	4	5	6	7	8	9	10	Je ne sais pas
MCH-M13 - Absorption - Redevabilité: Niveau d'effort pour assurer une redevabiilté optimale vis-à-vis des ressources allouées en soutien à la SMI dans le cadre de la réponse à la pandémie de COVID-19.	○	○	○	○	○	○	○	○	○	○	○
MCH-M14 - Récupération - Mesure dans laquelle les décisions sont orientées vers la récupération, et communiquées sur la base de données probantes pour promouvoir des comportements sûrs auprès des membres de la communauté.	○	○	○	○	○	○	○	○	○	○	○
MCH-M15 - Récupération - Connaissance/Conscience: Mesure dans laquelle le secteur SMI établit une certaine confiance à travers ses communications concernant les barrières posées par la pandémie de COVID-19 pour soutenir l'accès et l'utilisation des services et programmes de SMI.	○	○	○	○	○	○	○	○	○	○	○
MCH-M16 - Adaptation – Adaptif : Mesure dans laquelle les dirigeants nationaux avaient l'autorité d'effectuer des changements pour s'adapter et répondre aux menaces posées par la pandémie de COVID-19 aux services et programmes de SMI.	○	○	○	○	○	○	○	○	○	○	○

* Dimension sociale : interaction, collaboration et auto-synchronisation entre les personnes, les entités et les institutions.

	1	2	3	4	5	6	7	8	9	10	Je ne sais pas
MCH-M17 - Planification/Préparation – Mesure dans laquelle une formation sur la gestion de crise/épidémie a été menée et a permis de mettre à profit les réseaux sociaux, le capital social et institutionnel, et les normes culturelles avant la crise.	◯	◯	◯	◯	◯	◯	◯	◯	◯	◯	◯
MCH- M18 - Absorption – Mesure dans laquelle le personnel et les institutions sociales ont fait preuve d'ingéniosité et ont été accessibles pendant la réponse à l'épidémie/crise.	◯	◯	◯	◯	◯	◯	◯	◯	◯	◯	◯
MCH-M19 - Absorption – Autorégulation : Mesure dans laquelle les dirigeants nationaux avaient l'autorité d'effectuer des changements sectoriels en temps opportun à travers une infrastructure flexible.	◯	◯	◯	◯	◯	◯	◯	◯	◯	◯	◯
MCH-M20 - Récupération – Mesure dans laquelle le secteur SMI est engagé dans le partage de connaissances et d'équipes pour améliorer la récupération systémique.	◯	◯	◯	◯	◯	◯	◯	◯	◯	◯	◯
MCH-M21 - Récupération – Diversité : Niveau d'engagement d'une équipe multidisciplinaire pour mitiger les effets de la pandémie de COVID-19 sur la SMI.	◯	◯	◯	◯	◯	◯	◯	◯	◯	◯	◯

	1	2	3	4	5	6	7	8	9	10	Je ne sais pas
MCH-M22 - Adaptation – Mesure dans laquelle des ajouts ou modifications ont été apportés aux institutions, politiques, programmes, formations et cultures de SMI.	◯	◯	◯	◯	◯	◯	◯	◯	◯	◯	◯
MCH-M23 - Adaptation - Leadership et management : Niveau de leadership démontré par les dirigeants de la SMI pour mitiger les effets de la pandémie de COVID-19 sur la SMI.	◯	◯	◯	◯	◯	◯	◯	◯	◯	◯	◯

* MCH M-24 Temporalité : Mesure de la rapidité de la réaction du gouvernement –sa communication et mise en œuvre des mesures- pour mitiger l'impact immédiat et sur le long terme de la pandémie de COVID-19. Cette question porte sur la riposte nationale dans son ensemble, et non la réponse spécifique de votre secteur.

	1	2	3	4	5	6	7	8	9	10	Je ne sais pas
1-Plus faible -> Plus élevé-10	◯	◯	◯	◯	◯	◯	◯	◯	◯	◯	◯

MCH M-25 Commentaires : Utilisez cette section pour apporter tout commentaire additionnel sur la résilience et la durabilité du système nationaux de SMI en vous référant à votre expérience de la pandémie de COVID-19. Si vous avez répondu « Je ne sais pas » à l'une des questions, utilisez cette section pour expliquer pourquoi

JOHNS HOPKINS
BLOOMBERG SCHOOL
of PUBLIC HEALTH

Bill & Melinda Gates Institute for Population and Reproductive Health

Enquête de l'Indice des efforts pour le dividende démographique

Questionnaire sur l'éducation (ED)

Répondez aux questions suivantes en fonction de votre expérience/ expertise dans ce secteur spécifique.

Afin d'obtenir une représentation synthétique des efforts nationaux pour le dividende démographique, notez les éléments suivants sur une échelle de 1 à 10, 1 étant le score le plus faible (efforts très faibles ou quasi-inexistants) et 10 le plus élevé (efforts robustes). Le cas échéant, si une politique ou activité n'existe pas, répondez 0.

Donnez un score à chaque élément. Toutes les réponses seront enregistrées au format illustré ci-dessous :

Effort le plus faible	1	2	3	4	5	6	7	8	9	10	Effort le plus élevé	Je ne sais pas

JOHNS HOPKINS
BLOOMBERG SCHOOL
of PUBLIC HEALTH

Bill & Melinda Gates Institute for Population and Reproductive Health

Enquête de l’Indice des efforts pour le dividende démographique

Domaine 1. Politique/ Prise de décision

* ED 1.1 La qualité : Mesure dans laquelle il existe un plan/stratégie/programme pour améliorer la qualité de l’enseignement en milieu scolaire.

	1	2	3	4	5	6	7	8	9	10	Je ne sais pas
1-Plus faible -> Plus élevé - 10	○	○	○	○	○	○	○	○	○	○	○

* ED 1.2 Les groupes défavorisés :

	1	2	3	4	5	6	7	8	9	10	Je ne sais pas
ED 1.2.1. Mesure dans laquelle les stratégies ont été développées et mises en œuvre/déployées pour surmonter les obstacles d’apprentissage chez les filles enceintes et les mères adolescentes	○	○	○	○	○	○	○	○	○	○	○
ED 1.2.2 Mesure dans laquelle des stratégies ont été développées et mises en œuvre/déployées pour surmonter les obstacles d’apprentissage chez les personnes souffrant d’un handicap	○	○	○	○	○	○	○	○	○	○	○

* ED 1.3 Les enseignants : Mesure dans laquelle les problèmes liés au recrutement, développement et management du personnel enseignant sont inclus à la politique visant à améliorer les résultats d'apprentissage.

	1	2	3	4	5	6	7	8	9	10	Je ne sais pas
1-Plus faible->Plus élevé-10	○	○	○	○	○	○	○	○	○	○	○

* ED 1.4 La redevabilité des enseignants : Mesure dans laquelle les plans nationaux incluent des mesures spécifiques, dont le suivi et évaluation, visant à rendre les enseignants redevables de l'amélioration de l'apprentissage.

	1	2	3	4	5	6	7	8	9	10	Je ne sais pas
1-Plus faible -> Plus élevé - 10	○	○	○	○	○	○	○	○	○	○	○

* ED 1.5 La qualité des enseignants : Mesure dans laquelle les politiques/stratégies/programmes d'éducation incluent des indicateurs vérifiables de la qualité des enseignants.

	1	2	3	4	5	6	7	8	9	10	Je ne sais pas
1-Plus faible -> Plus élevé - 10	○	○	○	○	○	○	○	○	○	○	○

* ED 1.6 La collaboration dans l'éducation : Mesure dans laquelle la politique prévoit des dispositions pour engager plusieurs parties prenantes dans l'éducation (ex : écoles, communautés du milieu scolaire, parents, enseignants, gouvernement local, associations de parents d'élèves-élèves-enseignants, etc.).

	1	2	3	4	5	6	7	8	9	10	Je ne sais pas
1-Plus faible -> Plus élevé - 10	○	○	○	○	○	○	○	○	○	○	○

* ED 1.7 L'ODD sur la parité : Mesure dans laquelle les politiques/plans/stratégies nationales tiennent compte de l'Objectif de Développement Durable (ODD) sur la parité totale à tous les niveaux d'enseignement.

	1	2	3	4	5	6	7	8	9	10	Je ne sais pas
1-Plus faible -> Plus élevé - 10	○	○	○	○	○	○	○	○	○	○	○

* ED 1.8 Le financement de l'éducation : Mesure dans laquelle les nouveaux plans d'éducation incluent des propositions financières avec des budgets alloués aux réformes de l'enseignement et de l'apprentissage.

	1	2	3	4	5	6	7	8	9	10	Je ne sais pas
1-Plus faible -> Plus élevé - 10	○	○	○	○	○	○	○	○	○	○	○

* ED 1.9 La planification de la parité : Mesure dans laquelle il existe des plans/stratégies nationales pour atteindre 100% d'inscriptions avec parité en :

	1	2	3	4	5	6	7	8	9	10	Je ne sais pas
ED 1.9.1 Ecole primaire	○	○	○	○	○	○	○	○	○	○	○
ED 1.9.2 Ecole secondaire	○	○	○	○	○	○	○	○	○	○	○
ED 1.9.3 Ecole tertiaire/supérieure	○	○	○	○	○	○	○	○	○	○	○

* ED 1.10 L'analphabétisme chez l'adulte : Mesure dans laquelle des politiques/plans/stratégies nationales visent à répondre à l'analphabétisme.

	1	2	3	4	5	6	7	8	9	10	Je ne sais pas
ED 1.10.1 Mesure dans laquelle les politiques/plans/stratégies nationales visent à répondre à l'analphabétisme chez l'adulte.	○	○	○	○	○	○	○	○	○	○	○
ED 1.10.2 Mesure dans laquelle les politiques/plans/stratégies nationales visant à répondre à l'analphabétisme chez l'adulte visent aussi à résorber les inégalités entre les sexes.	○	○	○	○	○	○	○	○	○	○	○

* ED 1.11 Les enfants déscolarisés : Mesure dans laquelle des politiques répondent à la détresse des enfants déscolarisés pour les réintégrer au système scolaire ou leur offrir des opportunités alternatives de développement éducatif.

	1	2	3	4	5	6	7	8	9	10	Je ne sais pas
1-Plus faible -> Plus élevé - 10	○	○	○	○	○	○	○	○	○	○	○

* ED 1.12 L'intégration de la technologie : Mesure dans laquelle des stratégies ont été développées et mises en œuvre/déployées pour intégrer les technologies de l'information et de la communication (TIC) à l'éducation.

	1	2	3	4	5	6	7	8	9	10	Je ne sais pas
1-Plus faible -> Plus élevé - 10	○	○	○	○	○	○	○	○	○	○	○

* ED 1.13. La maîtrise des technologies : Mesure dans laquelle des ressources ont été allouées à l’amélioration de la maîtrise de l’informatique à tous les niveaux d’enseignement.

	1	2	3	4	5	6	7	8	9	10	Je ne sais pas
1-Plus faible -> Plus élevé - 10	◯	◯	◯	◯	◯	◯	◯	◯	◯	◯	◯

ED 1.14. Commentaires : Utilisez cette section pour nous faire part de tout commentaire additionnel sur le niveau d’effort, les défis ou les succès du pays autour des politiques/ de la prise de décision dans le secteur de l’éducation. Si vous avez répondu « Je ne sais pas » à l’une des questions précédentes, veuillez utiliser cette section pour expliquer pourquoi.

JOHNS HOPKINS
BLOOMBERG SCHOOL of PUBLIC HEALTH

Bill & Melinda Gates Institute for Population and Reproductive Health

Enquête de l'Indice des efforts pour le dividende démographique

Domaine 2. Services ou programmes

* ED 2.1 Le déploiement des enseignants : Mesure dans laquelle des stratégies/plans sont utilisés pour veiller à ce que les enseignants soient déployés dans les endroits du pays qui en ont le plus besoin.

	1	2	3	4	5	6	7	8	9	10	Je ne sais pas
1-Plus faible -> Plus élevé-10	○	○	○	○	○	○	○	○	○	○	○

* ED 2.2 La qualité des enseignants : Mesure dans laquelle la qualité des enseignants est reconnue comme un facteur clé pour améliorer les résultats d'apprentissage.

	1	2	3	4	5	6	7	8	9	10	Je ne sais pas
ED 2.2.1 Mesure dans laquelle la qualité de l'enseignement est soulignée dans les politiques/ plans/ stratégies éducatives plus générales.	○	○	○	○	○	○	○	○	○	○	○
ED 2.2.2 Mesure dans laquelle la qualité de la formation et du développement professionnel continu des enseignants est soulignée dans les politiques/ plans/ stratégies éducatives plus générales.	○	○	○	○	○	○	○	○	○	○	○

* ED 2.3 La parité : Mesure dans laquelle les ressources ont été utilisées pour améliorer la parité dans l'éducation entre les garçons et les filles.

	1	2	3	4	5	6	7	8	9	10	Je ne sais pas
1-Plus faible -> Plus élevé-10	○	○	○	○	○	○	○	○	○	○	○

* ED 2.4 L'implication des parents dans l'éducation : Mesure dans laquelle les parents sont impliqués dans le développement et l'exécution des stratégies éducatives (c'est-à-dire à travers l'implication des comités de parents d'élèves et d'enseignants).

	1	2	3	4	5	6	7	8	9	10	Je ne sais pas
1-Plus faible -> Plus élevé-10	○	○	○	○	○	○	○	○	○	○	○

* ED 2.5 Les évaluations standardisées : Mesure dans laquelle toutes les écoles utilisent des évaluations standardisées des écoles et des élèves/étudiants en accord avec les instructions émises par le Ministère de l'Éducation.

	1	2	3	4	5	6	7	8	9	10	Je ne sais pas
1-Plus faible -> Plus élevé-10	○	○	○	○	○	○	○	○	○	○	○

* ED 2.6. L'utilisation des résultats d'évaluation pour informer le progrès : Mesure dans laquelle des ajustements sont apportés aux écoles en fonction des résultats des évaluations scolaires nationales standardisées.

	1	2	3	4	5	6	7	8	9	10	Je ne sais pas
1-Plus faible -> Plus élevé-10	○	○	○	○	○	○	○	○	○	○	○

ED 2.7 Commentaires : Veuillez utiliser cette section pour nous faire part de tout commentaire additionnel sur le niveau d'effort, les défis ou les succès du pays autour des services/ programmes dans le secteur de l'éducation. Si vous avez répondu « Je ne sais pas » à l'une des questions précédentes, veuillez utiliser cette section pour expliquer pourquoi.

JOHNS HOPKINS
BLOOMBERG SCHOOL
of PUBLIC HEALTH

Bill & Melinda Gates Institute for Population and Reproductive Health

Enquête de l'Indice des efforts pour le dividende démographique

Domaine 3. Plaidoyer

* ED 3.1 L'égalité : Mesure dans laquelle l'égalité et l'éducation équitable ont été soulignés par les plaideurs.

	1	2	3	4	5	6	7	8	9	10	Je ne sais pas
1-Plus faible -> Plus élevé-10	○	○	○	○	○	○	○	○	○	○	○

* ED 3.2 La qualité : Mesure dans laquelle la qualité de l'éducation est intégrée aux évaluations liées au plaidoyer pour le système éducatif.

	1	2	3	4	5	6	7	8	9	10	Je ne sais pas
1-Plus faible -> Plus élevé-10	○	○	○	○	○	○	○	○	○	○	○

* ED 3.3 Les obstacles des enseignants : Mesure dans laquelle les besoins des enseignants ont été mis en évidence par les plaideurs.

	1	2	3	4	5	6	7	8	9	10	Je ne sais pas
1-Plus faible -> Plus élevé-10	○	○	○	○	○	○	○	○	○	○	○

* ED 3.4 Les obstacles des institutions : Mesure dans laquelle les difficultés des institutions éducatives ont été mises en évidence dans les différents efforts de plaidoyer.

	1	2	3	4	5	6	7	8	9	10	Je ne sais pas
1-Plus faible -> Plus élevé-10	○	○	○	○	○	○	○	○	○	○	○

ED 3.5 Commentaires : Veuillez utiliser cette section pour nous faire part de tout commentaire additionnel sur le niveau d'effort, les défis ou les succès du pays autour du plaidoyer dans le secteur de l'éducation. Si vous avez répondu « Je ne sais pas » à l'une des questions précédentes, veuillez utiliser cette section pour expliquer pourquoi.

JOHNS HOPKINS
BLOOMBERG SCHOOL
of PUBLIC HEALTH

Bill & Melinda Gates Institute for Population and Reproductive Health

Enquête de l'Indice des efforts pour le dividende démographique

Domaine 4. Recherche

* ED 4.1 La force des observatoires/parties prenantes/groupes de travail pour l'ED : Mesure dans laquelle les parties prenantes du secteur de l'éducation du pays (ex : groupe directif de parties prenantes, groupes de travail techniques, observatoire de l'ED, groupe de coordination ou facilitation) :

	1	2	3	4	5	6	7	8	9	10	Je ne sais pas
ED 4.1.1 Ont un représentant du gouvernement, des institutions de formation, de la société civile, des organisations non gouvernementales et confessionnelles, des associations professionnelles, du secteur privé, etc.	◯	◯	◯	◯	◯	◯	◯	◯	◯	◯	◯
ED 4.1.2 Se réunissent régulièrement, émettent des rapports et recommandent des politiques aux directions des ministères compétents.	◯	◯	◯	◯	◯	◯	◯	◯	◯	◯	◯
ED 4.1.3 Ont un impact sur l'éducation dans le pays.	◯	◯	◯	◯	◯	◯	◯	◯	◯	◯	◯

* ED 4.2 La stratégie d'éducation/recherche : Mesure dans laquelle le plan d'éducation nationale et/ou d'autres documents nationaux comprennent une stratégie/approche complète de recherche liée à l'éducation, partagée entre partenaires.

	1	2	3	4	5	6	7	8	9	10	Je ne sais pas
1-Plus faible -> Plus élevé-10	◯	◯	◯	◯	◯	◯	◯	◯	◯	◯	◯

* ED 4.3 Les partenaires collectant les données :

	1	2	3	4	5	6	7	8	9	10	Je ne sais pas
ED 4.3.1 Mesure dans laquelle la collecte des données est entreprise par des agences gouvernementales. (Cela peut comprendre les agences/bureaux de la statistique et/ou les ministères de l'économie/finances, éducation, santé, genre, etc.)	○	○	○	○	○	○	○	○	○	○	○
ED 4.3.2 Mesure dans laquelle la collecte de données est entreprise par des institutions de recherche.	○	○	○	○	○	○	○	○	○	○	○
ED 4.3.3 Mesure dans laquelle la collecte des données est entreprise par des chercheurs indépendants.	○	○	○	○	○	○	○	○	○	○	○

* ED 4.4 La recherche thématique :

	1	2	3	4	5	6	7	8	9	10	Je ne sais pas
ED 4.4.1 Mesure dans laquelle de la recherche est menée sur les inégalités de genre dans l'éducation.	○	○	○	○	○	○	○	○	○	○	○
ED 4.4.2 Mesure dans laquelle de la recherche est menée sur la qualité de l'enseignement.	○	○	○	○	○	○	○	○	○	○	○
ED 4.4.3 Mesure dans laquelle de la recherche est menée sur les obstacles auxquels font face les enseignants.	○	○	○	○	○	○	○	○	○	○	○
ED 4.4.4 Mesure dans laquelle de la recherche est menée sur les obstacles auxquels font face les institutions.	○	○	○	○	○	○	○	○	○	○	○

* ED 4.5 La qualité/couverture des données : Mesure dans laquelle la recherche/les données actuelles sont désagrégées par sexe et par âge. Mesure dans laquelle le pays participe aux tests standardisés et son classement.

	1	2	3	4	5	6	7	8	9	10	Je ne sais pas
ED 4.5.1 Mesure dans laquelle un système statistique de routine (utilisant les informations des écoles/institutions) offre de bonnes informations régulières sur les produits et les établissements	◯	◯	◯	◯	◯	◯	◯	◯	◯	◯	◯
ED 4.5.2 Mesure dans laquelle un système statistique de routine (utilisant les informations des écoles/institutions) offre de bonnes informations régulières sur les services et le personnel du secteur éducatif	◯	◯	◯	◯	◯	◯	◯	◯	◯	◯	◯
ED 4.5.3 Mesure dans laquelle un système statistique de routine offre de bonnes informations régulières sur les besoins éducatifs des populations de différentes communautés	◯	◯	◯	◯	◯	◯	◯	◯	◯	◯	◯
ED 4.5.4 Mesure dans laquelle des évaluations du système de suivi de l'éducation sont menées et appliquées pour assurer la fiabilité des données	◯	◯	◯	◯	◯	◯	◯	◯	◯	◯	◯

* ED 4.6 Les registres : Mesure dans laquelle les systèmes de registres, rapports et feedback sur les résultats de l'éducation sont adéquats.

	1	2	3	4	5	6	7	8	9	10	Je ne sais pas
1-Plus faible -> Plus élevé-10	◯	◯	◯	◯	◯	◯	◯	◯	◯	◯	◯

* ED 4.7 La qualité des institutions de recherche : Mesure dans laquelle le pays a la capacité de soutenir et maintenir des institutions de recherche qui développent des études et/ou collectent des données permettant d'informer les efforts pour l'amélioration de l'éducation/ l'enseignement.

	1	2	3	4	5	6	7	8	9	10	Je ne sais pas
1-Plus faible -> Plus élevé-10	◯	◯	◯	◯	◯	◯	◯	◯	◯	◯	◯

* ED 4.8 L'évaluation : Mesure dans laquelle les statistiques, enquêtes et études sur le programme d'éducation sont utilisées par du personnel spécialisé pour rapporter les opérations du programme et mesurer le progrès.

	1	2	3	4	5	6	7	8	9	10	Je ne sais pas
1-Plus faible -> Plus élevé-10	◯	◯	◯	◯	◯	◯	◯	◯	◯	◯	◯

* ED 4.9 L'utilisation des résultats d'évaluation par les gestionnaires de programmes : Mesure dans laquelle les gestionnaires locaux du programme d'éducation utilisent les conclusions de recherche et d'évaluations pour améliorer ces programmes en suivant les recommandations émises.

	1	2	3	4	5	6	7	8	9	10	Je ne sais pas
1-Plus faible -> Plus élevé - 10	◯	◯	◯	◯	◯	◯	◯	◯	◯	◯	◯

* ED 4.10 L'utilisation des résultats d'évaluation par les ministères : Mesure dans laquelle les administrateurs ministériels compétents (soit le Ministère de l'Éducation) utilisent systématiquement les données pour informer les politiques et interventions visant à résoudre les problèmes éducatifs.

	1	2	3	4	5	6	7	8	9	10	Je ne sais pas
1-Plus faible -> Plus élevé-10	◯	◯	◯	◯	◯	◯	◯	◯	◯	◯	◯

* ED 4.11 La dissémination d'informations à d'autres entités chargées de la mise en œuvre des programmes :

	1	2	3	4	5	6	7	8	9	10	Je ne sais pas
ED 4.11.1 Mesure dans laquelle des informations sont partagées ou disséminées entres géographies/ régions.	◯	◯	◯	◯	◯	◯	◯	◯	◯	◯	◯
ED. 4.11.2 Mesure dans laquelle des informations sont partagées ou disséminées à différents niveaux (national, états/provinces, localités).	◯	◯	◯	◯	◯	◯	◯	◯	◯	◯	◯

ED 4.12 Commentaires : Veuillez utiliser cette section pour nous faire part de tout commentaire additionnel sur le niveau d'effort, les défis ou les succès du pays autour de la recherche dans le secteur de l'éducation. Si vous avez répondu « Je ne sais pas » à l'une des questions précédentes, veuillez utiliser cette section pour expliquer pourquoi.

JOHNS HOPKINS
BLOOMBERG SCHOOL
of PUBLIC HEALTH

Bill & Melinda Gates Institute for Population and Reproductive Health

Enquête de l’Indice des efforts pour le dividende démographique

Domaine 5. Organisations de la société civile (OSC)

* ED 5.1 Le pouvoir des acteurs des OSC : Mesure dans laquelle les acteurs des OSC occupent des postes d’influence dans les institutions, les groupes de travail techniques ou les réseaux concernés par l’éducation.

	1	2	3	4	5	6	7	8	9	10	Je ne sais pas
1-Plus faible -> Plus élevé-10	○	○	○	○	○	○	○	○	○	○	○

* ED 5.2 L’analyse budgétaire comme outil des OSC : Mesure dans laquelle les OSC utilisent l’analyse budgétaire comme un outil pour développer le plaidoyer en matière d’éducation.

	1	2	3	4	5	6	7	8	9	10	Je ne sais pas
1-Plus faible -> Plus élevé-10	○	○	○	○	○	○	○	○	○	○	○

* ED 5.3 L’inclusion communautaire : Mesure dans laquelle les OSC sont impliquées dans les efforts d’exploration d’opportunités pour l’amélioration de la qualité de l’éducation.

	1	2	3	4	5	6	7	8	9	10	Je ne sais pas
1-Plus faible -> Plus élevé-10	○	○	○	○	○	○	○	○	○	○	○

* ED 5.4 L’implication de la communauté dans l’éducation : Mesure dans laquelle les OSC participent au rapprochement des informations et services éducatifs avec les communautés à travers des activités telles que le renforcement des compétences des enseignants et des parents et/ou les campagnes d’information ou d’éducation pour augmenter l’engagement des familles et des communautés en faveur de l’éducation.

	1	2	3	4	5	6	7	8	9	10	Je ne sais pas
1-Plus faible -> Plus élevé-10	○	○	○	○	○	○	○	○	○	○	○

* ED 5.5 La franchise sociale : Mesure dans laquelle les OSC sont impliquées dans la franchise sociale des services/opportunités éducatives de qualité aux familles et enfants à moindre coût.

	1	2	3	4	5	6	7	8	9	10	Je ne sais pas
1-Plus faible -> Plus élevé-10	○	○	○	○	○	○	○	○	○	○	○

* ED 5.6 La technologie : Mesure dans laquelle les OSC mettent à profit les technologies de l'information et de la communication (TIC) pour améliorer l'accès à l'éducation de qualité de diverses communautés.

	1	2	3	4	5	6	7	8	9	10	Je ne sais pas
1-Plus faible -> Plus élevé-10	○	○	○	○	○	○	○	○	○	○	○

* ED 5.7 Les droits humains, l'accès et la qualité de l'éducation : Mesure dans laquelle les OSC plaident pour le soutien d'approches éducatives basées sur les droits et veillent à ce que les élèves/étudiants soit traités correctement, n'aient pas peur de poser des questions, et reçoivent un enseignement de qualité, entre autres préoccupations.

	1	2	3	4	5	6	7	8	9	10	Je ne sais pas
1-Plus faible -> Plus élevé-10	○	○	○	○	○	○	○	○	○	○	○

* ED 5.8 Le changement social et des comportements (CSC) : Mesure dans laquelle les OSC soutiennent les interventions pour le CSC comprenant, mais sans s'y limiter, aux compétences de vie et à l'éducation entre pairs, l'éducation sur le santé de l'adulte, l'éducation en milieu scolaire, la mobilisation communautaire et/ou les interventions médiatiques destinées au grand public.

	1	2	3	4	5	6	7	8	9	10	Je ne sais pas
1-Plus faible -> Plus élevé-10	○	○	○	○	○	○	○	○	○	○	○

* ED 5.9 Le plaidoyer/ la redevabilité : Mesure dans laquelle les OSC soutiennent la formation et le renforcement de compétences des plaideurs et des membres de la communauté pour améliorer leur compréhension des politiques et processus, leurs activités d'identification de problèmes, la résolution collaborative de ces problèmes, et des actions de plaidoyer plus ciblées pour améliorer l'éducation.

	1	2	3	4	5	6	7	8	9	10	Je ne sais pas
1-Plus faible -> Plus élevé-10	○	○	○	○	○	○	○	○	○	○	○

* ED 5.10 L'évaluation et le suivi dirigés par les OSC : Mesure dans laquelle les OSC évaluent, suivent et émettent des rapports sur l'efficacité des politiques et programmes pour améliorer la redevabilité des enseignants et des politiques d'éducation.

	1	2	3	4	5	6	7	8	9	10	Don't know
1-Plus faible -> Plus élevé-10	○	○	○	○	○	○	○	○	○	○	○

* ED 5.11 Les partenariats entre OSC : Mesure dans laquelle les OSC ont formé des alliances nationales et des partenariats régionaux pour renforcer leur position et potentiellement améliorer la force du leadership et les mécanismes de financement en vue d'améliorer la qualité de l'éducation.

	1	2	3	4	5	6	7	8	9	10	Je ne sais pas
1-Plus faible -> Plus élevé-10	○	○	○	○	○	○	○	○	○	○	○

ED 5.12 Commentaires : Veuillez utiliser cette section pour nous faire part de tout commentaire additionnel sur le niveau d'effort, les défis ou les succès du pays autour des organisations de la société civile impliquées dans le secteur de l'éducation. Si vous avez répondu « Je ne sais pas » à l'une des questions précédentes, veuillez utiliser cette section pour expliquer pourquoi.

JOHNS HOPKINS
BLOOMBERG SCHOOL
of PUBLIC HEALTH

Bill & Melinda Gates Institute for Population and Reproductive Health

Enquête de l'Indice des efforts pour le dividende démographique

Module : Résilience et durabilité du secteur

Étant donné l'impact sanitaire et socioéconomique de la pandémie de COVID-19, et son impact probable sur la progression vers le DD, cet indice des efforts pour le DD intègre des questions pour évaluer la résilience et la durabilité des systèmes dans les secteurs clés du DD. Les éléments couverts par les questions liées à la pandémie de COVID-19 ci-dessous relèvent des dimensions clés des systèmes résilients et visent à apporter des informations déterminantes sur le potentiel d'une réponse efficace aux menaces de maladies infectieuses émergeantes et autres crises de santé publique.

Veuillez noter chaque élément sur une échelle de 1 à 10, 1 étant le score le plus faible (faible état/capacité) et 10 le plus élevé (très bon état/capacité).

* Dimension physique : structures, équipements, états du système et capacités

	1	2	3	4	5	6	7	8	9	10	Je ne sais pas
ED-M1 - Planification/Préparation – État et capacité de l'équipement, du personnel et de la structure du secteur de l'ED avant la crise. (1 = faible état/capacité; 10 = très bon état/capacité)	○	○	○	○	○	○	○	○	○	○	○
ED-M2 - Absorption – Mesure dans laquelle la pandémie de COVID-19 a été reconnue par le secteur de l'ED, et mesure dans laquelle le système a pu continuer d'être fonctionnel	○	○	○	○	○	○	○	○	○	○	○

	1	2	3	4	5	6	7	8	9	10	Je ne sais pas
ED-M3 - Absorption – Intégration : Niveau d'intégration des services et programmes d'ED pour mitiger les effets de la pandémie de COVID-19 sur l'ED.	○	○	○	○	○	○	○	○	○	○	○
ED-M4 - Récupération – Mesure dans laquelle le secteur de l'ED a été capable d'induire des changements pour récupérer un niveau de fonctionnalité pré-COVID-19.	○	○	○	○	○	○	○	○	○	○	○
ED-M5 - Récupération – Gestion du financement et des bailleurs de fonds : Niveau de mobilisation des ressources et allocations pour mitiger les effets de la pandémie de COVID-19 sur l'ED.	○	○	○	○	○	○	○	○	○	○	○
ED-M6 - Adaptation – Mesure dans laquelle des changements ont été effectués pour améliorer la résilience du secteur de l'ED.	○	○	○	○	○	○	○	○	○	○	○

* Information : création, gestion, stockage et utilisation des données.

	1	2	3	4	5	6	7	8	9	10	Je ne sais pas
ED-M7 - Planification/ Préparation – Mesure dans laquelle les données liées à l'ED étaient suffisamment préparées, présentées, analysées et stockées avant la crise.	○	○	○	○	○	○	○	○	○	○	○
ED-M8 - Absorption - Knowledge: Extent to which the monitoring capacity was able to detect the limits / threats posed to the ED sector by the COVID-19 pandemic. (This includes data on the various key subjects of the education sector at each level of education [primary, secondary, tertiary / higher]).	○	○	○	○	○	○	○	○	○	○	○
ED-M9 - Récupération – Mesure dans laquelle les données de l'ED ont été utilisées pour suivre le progrès vers la récupération et anticiper des scenarii de récupération.	○	○	○	○	○	○	○	○	○	○	○
ED-M10 - Adaptation – Mesure dans laquelle le système d'ED crée et améliore actuellement ses protocoles de stockage et d'utilisation de ses données en temps réel.	○	○	○	○	○	○	○	○	○	○	○

* Dimension cognitive : compréhension, modèles cognitifs, idées reçues, préjugés et valeurs.

	1	2	3	4	5	6	7	8	9	10	Je ne sais pas
ED-M11 – Planification/ Préparation – Mesure dans laquelle le système d'ED et les décisions opérationnelles étaient préparés pour anticiper la crise.	○	○	○	○	○	○	○	○	○	○	○

	1	2	3	4	5	6	7	8	9	10	Je ne sais pas
ED-M12 - Absorption – Mesure dans laquelle la réponse du système d'ED a des protocoles de contingence suffisants et une gestion de crise proactive.											
ED-M13 - Absorption – Redevabilité : Niveau d'effort pour assurer une redevabilité optimale vis-à-vis des ressources allouées à l'ED dans le cadre de la réponse à la pandémie de COVID-19.											
ED-M14 - Récupération – Mesure dans laquelle les décisions sont orientées sur la récupération et communiquées aux communautés sur la base de données probantes pour promouvoir des comportements sûrs.											
ED-M15 - Récupération – Connaissance : Mesure dans laquelle le secteur de l'ED établit une certaine confiance à travers sa communication concernant les barrières posées par la pandémie de COVID-19 pour soutenir l'accès et l'utilisation des services et programmes d'ED.											
ED-M16 - Adaptation – Mesure dans laquelle le secteur de l'ED conçoit de nouvelles configurations systémiques, de nouveaux objectifs et de nouveaux critères de décision.											

	1	2	3	4	5	6	7	8	9	10	Je ne sais pas
ED-M17 – Adaptation : Niveau d'adaptation pour répondre aux menaces posées par la pandémie de COVID-19 à l'ED.	○	○	○	○	○	○	○	○	○	○	○

* Social dimension: interaction, collaboration and self-synchronization between people, entities and institutions.

	1	2	3	4	5	6	7	8	9	10	Je ne sais pas
ED-M18 – Planification /Préparation – Mesure dans laquelle une formation sur la gestion d'épidémies/crises a été menée et a permis de mettre à profit les réseaux sociaux, le capital social et les normes institutionnelles et culturelles avant la crise.	○	○	○	○	○	○	○	○	○	○	○
ED-M19 - Absorption – Mesure dans laquelle le personnel et les institutions sociales ont été accessibles et ont fait preuve d'ingéniosité dans leur réponse à l'épidémie/crise.	○	○	○	○	○	○	○	○	○	○	○
ED-M20 - Absorption - Self-regulation: Extent to which national leaders had the authority to effect timely sectoral changes through an infrastructure flexible.	○	○	○	○	○	○	○	○	○	○	○
ED-M21 – Récupération – Mesure dans laquelle le système d'ED est impliqué dans le partage de connaissances et d'équipes pour améliorer la récupération du système.	○	○	○	○	○	○	○	○	○	○	○

	1	2	3	4	5	6	7	8	9	10	Je ne sais pas
ED-M22 - Récupération – Diversité : Niveau d'engagement d'une équipe multidisciplinaire pour mitiger les effets de la pandémie de COVID-19 sur l'ED.	◯	◯	◯	◯	◯	◯	◯	◯	◯	◯	◯
ED-M23 - Adaptation – Mesure dans laquelle des ajouts ou changements sont apportés aux institutions, politiques, formations, programmes et à la culture de l'ED.	◯	◯	◯	◯	◯	◯	◯	◯	◯	◯	◯
ED-M24 - Adaptation - Leadership et management : Niveau de leadership démontré par les dirigeants du secteur de l'ED pour mitiger les effets de la pandémie de COVID-19 sur l'ED.	◯	◯	◯	◯	◯	◯	◯	◯	◯	◯	◯

* ED M-25 Temporalité : Mesure de la rapidité de la réaction du gouvernement –sa communication et mise en œuvre des mesures- pour mitiger l'impact immédiat et sur le long terme de la pandémie de COVID-19. Cette question porte sur la riposte nationale dans son ensemble, et non la réponse spécifique de votre secteur.

	1	2	3	4	5	6	7	8	9	10	Je ne sais pas
1-Plus faible -> Plus élevé-10	◯	◯	◯	◯	◯	◯	◯	◯	◯	◯	◯

ED M-26 Commentaires : Utilisez cette section pour apporter tout commentaire additionnel sur la résilience et la durabilité du système d'éducation nationale en vous référant à votre expérience de la pandémie de COVID-19. Si vous avez répondu « Je ne sais pas » à l'une des questions, utilisez cette section pour expliquer pourquoi.

JOHNS HOPKINS
BLOOMBERG SCHOOL
of PUBLIC HEALTH

Bill & Melinda Gates Institute for Population and Reproductive Health

Enquête de l'Indice des efforts pour le dividende démographique

Questionnaire sur l'autonomisation des femmes (AF)

Répondez aux questions suivantes en fonction de votre expérience/ expertise dans ce secteur spécifique.

Afin d'obtenir une représentation synthétique des efforts nationaux pour le dividende démographique, notez les éléments suivants sur une échelle de 1 à 10, 1 étant le score le plus faible (efforts très faibles ou quasi-inexistants) et 10 le plus élevé (efforts robustes). Le cas échéant, si une politique ou activité n'existe pas, répondez 0.

Donnez un score à chaque élément. Toutes les réponses seront enregistrées au format illustré ci-dessous :

Effort le plus faible	1	2	3	4	5	6	7	8	9	10	Effort le plus élevé	Je ne sais pas

JOHNS HOPKINS
BLOOMBERG SCHOOL
of PUBLIC HEALTH

Bill & Melinda Gates Institute for Population and Reproductive Health

Enquête de l'Indice des efforts pour le dividende démographique

Domaine 1. Politique/ Prise de décision

* WE 1.1 La politique de parité : Mesure dans laquelle la parité est prise en compte/intégrée aux plans de développement du pays (ou aux plans de votre secteur).

	1	2	3	4	5	6	7	8	9	10	Je ne sais pas
1-Plus faible -> Plus élevé-10	◯	◯	◯	◯	◯	◯	◯	◯	◯	◯	◯

* WE 1.2 Niveau d'engagement envers la Convention sur l'élimination de toutes les formes de discrimination à l'égard des femmes (CEDAW) :

	1	2	3	4	5	6	7	8	9	10	Je ne sais pas
1-Plus faible -> Plus élevé-10	◯	◯	◯	◯	◯	◯	◯	◯	◯	◯	◯

* WE 1.3 La parité dans les politiques familiales et de mariage : Mesure dans laquelle le principe d'égalité hommes-femmes est incorporé à la constitution nationale et autres législations concernant le mariage et les relations familiales.

	1	2	3	4	5	6	7	8	9	10	Je ne sais pas
1-Plus faible -> Plus élevé-10	◯	◯	◯	◯	◯	◯	◯	◯	◯	◯	◯

* WE 1.4 L'âge légal du mariage : Mesure dans laquelle l'âge légal du mariage pour les filles est établi à 18 ans ou plus et est appliqué.

	1	2	3	4	5	6	7	8	9	10	Je ne sais pas
1-Plus faible -> Plus élevé-10	◯	◯	◯	◯	◯	◯	◯	◯	◯	◯	◯

* WE 1.5 Les mesures contre les violences à l’égard des femmes : Niveau des régulations et mesures contre les violences faites aux femmes, dont la violence intraconjugale, le viol, le harcèlement sexuel et la traite des filles et des femmes.

	1	2	3	4	5	6	7	8	9	10	Je ne sais pas
1-Plus faible -> Plus élevé-10	○	○	○	○	○	○	○	○	○	○	○

* WE 1.6 La mutilation génitale féminine : Niveau de régulation et mesures contre la mutilation génitale féminine.

	1	2	3	4	5	6	7	8	9	10	Je ne sais pas
1-Plus faible -> Plus élevé-10	○	○	○	○	○	○	○	○	○	○	○

* WE 1.7 Les mesures de santé des femmes : Niveau des protocoles de protection de la santé des femmes, dont les infections sexuellement transmissibles, y compris le VIH/SIDA, la mortalité maternelle et la contraception.

	1	2	3	4	5	6	7	8	9	10	Je ne sais pas
1-Plus faible -> Plus élevé-10	○	○	○	○	○	○	○	○	○	○	○

* WE 1.8 Les mesures éducatives : Mesure dans laquelle il existe des mécanismes pour promouvoir l’éducation des filles.

	1	2	3	4	5	6	7	8	9	10	Je ne sais pas
WE 1.8.1 Niveau d’application des politiques sur l’éducation des filles.	○	○	○	○	○	○	○	○	○	○	○
WE 1.8.2 Mesure dans laquelle des mécanismes sont en place pour prévenir l’échec/l’abandon scolaire des filles.	○	○	○	○	○	○	○	○	○	○	○

* WE 1.9 Les mesures relatives à l’exercice des droits : Niveau d’application des politiques sur les droits des femmes.

	1	2	3	4	5	6	7	8	9	10	Je ne sais pas
1-Plus faible -> Plus élevé-10	○	○	○	○	○	○	○	○	○	○	○

* WE 1.10 Le travail équitable : Mesure dans laquelle il existe des politiques sur les conditions de travail équitables et l'égalité des salaires entre hommes et femmes.

	1	2	3	4	5	6	7	8	9	10	Je ne sais pas
1-Plus faible -> Plus élevé-10	○	○	○	○	○	○	○	○	○	○	○

* WE 1.11 L'égalité de la propriété de biens/biens fonciers : Mesure dans laquelle il existe des politiques pour affirmer l'accès équitable à la propriété de biens fonciers.

	1	2	3	4	5	6	7	8	9	10	Je ne sais pas
1-Plus faible -> Plus élevé-10	○	○	○	○	○	○	○	○	○	○	○

* WE 1.12 Le conflit, les femmes et le rétablissement de la paix : Niveau de mise en œuvre de la Résolution 1325 du Conseil de sécurité des Nations Unies sur les impacts des conflits sur les femmes et leur rôle dans le rétablissement de la paix.

	1	2	3	4	5	6	7	8	9	10	Je ne sais pas
1-Plus faible -> Plus élevé-10	○	○	○	○	○	○	○	○	○	○	○

* WE 1.13 Les quotas de femmes dans les politiques : Existence et niveau de mise en œuvre des quotas dans les systèmes politiques observant la parité.

	1	2	3	4	5	6	7	8	9	10	Je ne sais pas
WE 1.13.1 Mesure dans laquelle il existe une prise de décision équitable du point de vue du genre, y compris en soutien au quotas électoraux et à l'intégration des femmes dans tous les ministères et départements du gouvernement.	○	○	○	○	○	○	○	○	○	○	○
WE 1.13.2 Mesure dans laquelle les femmes participant et/ou jouent un rôle significatif dans les systèmes traditionnels de gouvernance (ex : chefs de village, etc.).	○	○	○	○	○	○	○	○	○	○	○

* WE 1.14 L'Objectif de Développement Durable (ODD) 5 : Niveau d'engagement pour remplir l'Objectif 5 des ODD, pour réaliser la parité et assurer l'autonomisation de toutes les filles et femmes et les filles.

	1	2	3	4	5	6	7	8	9	10	Je ne sais pas
1-Plus faible -> Plus élevé-10	○	○	○	○	○	○	○	○	○	○	○

* WE 1.15 La révision de la loi coutumière : Mesure dans laquelle les lois coutumières ont été revues et modifiées par des politiques/cadres nationaux ; ou mesure dans laquelle les lois coutumières existantes reflètent les efforts pour l'autonomisation des femmes.

	1	2	3	4	5	6	7	8	9	10	Je ne sais pas
1-Plus faible -> Plus élevé-10	○	○	○	○	○	○	○	○	○	○	○

* WE 1.16 La budgétisation pour l'autonomisation des femmes : Mesure dans laquelle la budgétisation et l'allocation budgétaire sont suffisantes pour couvrir les coûts nécessaires à l'atteinte des cibles établies dans le plan national de la femme ou son équivalent.

	1	2	3	4	5	6	7	8	9	10	Je ne sais pas
1-Plus faible -> Plus élevé-10	○	○	○	○	○	○	○	○	○	○	○

* WE 1.17 Les ressources humaines pour l'autonomisation des femmes : Mesure dans laquelle :

	1	2	3	4	5	6	7	8	9	10	Je ne sais pas
WE 1.17.1 Du personnel en quantité et qualité suffisante est employé pour mettre en œuvre les activités liées au genre, y compris les efforts pour former ce personnel	○	○	○	○	○	○	○	○	○	○	○
WE 1.17.2 Des efforts sont menés pour former le personnel de manière appropriée et intégrale.	○	○	○	○	○	○	○	○	○	○	○
WE 1.17.3 Le personnel est suffisamment soutenu pour mener à bien ses tâches.	○	○	○	○	○	○	○	○	○	○	○
WE 1.17.4 Le personnel assigné est situé à un niveau administratif suffisamment élevé pour travailler efficacement.	○	○	○	○	○	○	○	○	○	○	○

* WE 1.18 La transparence et la redevabilité : Mesure dans laquelle le gouvernement fournit aux parties prenantes des informations complètes sur les intentions, plans, programmes et budgets disponibles pour mettre en œuvre les programmes d’autonomisation des femmes et en assurer la redevabilité et la transparence. (*Peuvent être incluses l’adoption et l’application d’une loi sur le droit à l’information, des sessions informatives régulières, par exemple dans les mairies, ou la transmission de ces informations via des brochures ou autres supports de communication en langue locale*).

	1	2	3	4	5	6	7	8	9	10	Je ne sais pas
1-Plus faible -> Plus élevé-10	○	○	○	○	○	○	○	○	○	○	○

WE 1.19 Commentaire : Utilisez cette section pour apporter des commentaires additionnels sur le niveau d’efforts, les défis et les succès du pays autour de la politique/ prise de décision pour l’autonomisation des femmes. Si vous avez répondu « Je ne sais pas » à l’une des questions ci-dessus, utilisez aussi cette section pour expliquer pourquoi.

JOHNS HOPKINS
BLOOMBERG SCHOOL
of PUBLIC HEALTH

Bill & Melinda Gates Institute for Population and Reproductive Health

Enquête de l'Indice des efforts pour le dividende démographique

Domaine 2. Services ou programmes

* WE 2.1 L'intégration des femmes : Mesure dans laquelle les services ou programmes sectoriels et intersectoriels tiennent compte des implications des actions planifiées pour les hommes et pour les femmes.

	1	2	3	4	5	6	7	8	9	10	Je ne sais pas
1-Plus faible -> Plus élevé-10	○	○	○	○	○	○	○	○	○	○	○

* WE 2.2 Les programmes transformateurs de genre : Mesure dans laquelle les services ou programmes sectoriels ou intersectoriels intègrent des principes/ approches transformateurs de genre.

	1	2	3	4	5	6	7	8	9	10	Je ne sais pas
1-Plus faible -> Plus élevé-10	○	○	○	○	○	○	○	○	○	○	○

* WE 2.3 L'éducation : Mesure dans laquelle les services ou programmes éducatifs se focalisent sur l'autonomisation des filles et des femmes dans les salles de classe.

	1	2	3	4	5	6	7	8	9	10	Je ne sais pas
1-Plus faible -> Plus élevé-10	○	○	○	○	○	○	○	○	○	○	○

* WE 2.4. L'abandon scolaire : Mesure dans laquelle il existe des programmes pour prévenir l'abandon scolaire des filles.

	1	2	3	4	5	6	7	8	9	10	Je ne sais pas
1-Plus faible -> Plus élevé-10	○	○	○	○	○	○	○	○	○	○	○

* WE 2.5 La maîtrise des technologies : Mesure dans laquelle la maîtrise des technologies est promue auprès des filles en particulier.

	1	2	3	4	5	6	7	8	9	10	Je ne sais pas
1-Plus faible -> Plus élevé-10	◯	◯	◯	◯	◯	◯	◯	◯	◯	◯	◯

* WE 2.6 L'emploi : Mesure dans laquelle les programmes de création d'emploi et les politiques du marché du travail visent à garantir l'indépendance économique des femmes et sont conçus/mis en œuvre en conséquence.

	1	2	3	4	5	6	7	8	9	10	Je ne sais pas
1-Plus faible -> Plus élevé-10	◯	◯	◯	◯	◯	◯	◯	◯	◯	◯	◯

* WE 2.7 Les femmes-mères-travailleuses : Mesure dans laquelle le gouvernement permet aux femmes, à travers la loi, les régulations et les programmes, de combiner leur rôle de mère pendant la grossesse, l'allaitement et l'éducation de leurs enfants à leur participation à la vie active.

	1	2	3	4	5	6	7	8	9	10	Je ne sais pas
1-Plus faible -> Plus élevé-10	◯	◯	◯	◯	◯	◯	◯	◯	◯	◯	◯

* WE 2.8 La planification familiale pour l'autonomisation des femmes : Mesure dans laquelle les services ou programmes de PF sont mis en œuvre selon une approche fondamentale d'autonomisation des femmes.

	1	2	3	4	5	6	7	8	9	10	Je ne sais pas
1-Plus faible -> Plus élevé-10	◯	◯	◯	◯	◯	◯	◯	◯	◯	◯	◯

* WE 2.9 La représentation politique et publique : Mesure de la mise en œuvre des programmes visant à améliorer la participation et la représentation équitables des femmes à tous les niveaux du processus politique et de la vie publique.

	1	2	3	4	5	6	7	8	9	10	Je ne sais pas
1-Plus faible -> Plus élevé-10	◯	◯	◯	◯	◯	◯	◯	◯	◯	◯	◯

* WE 2.10 La violence : Portée de la lutte contre les violences faites à l'égard des femmes et des enfants, et leur élimination à l'échelle du pays (violences intraconjugales, viol, harcèlement sexuel, traite des femmes).

	1	2	3	4	5	6	7	8	9	10	Je ne sais pas
1-Plus faible -> Plus élevé-10	◯	◯	◯	◯	◯	◯	◯	◯	◯	◯	◯

* WE 2.11 Le mariage d'enfants : Mesure de l'application des lois pour éliminer le mariage d'enfants.

	1	2	3	4	5	6	7	8	9	10	Je ne sais pas
1-Plus faible -> Plus élevé-10	○	○	○	○	○	○	○	○	○	○	○

* WE 2.12 La mutilation génitale féminine : Mesure de l'application des lois pour éliminer la mutilation génitale féminine.

	1	2	3	4	5	6	7	8	9	10	Je ne sais pas
1-Plus faible -> Plus élevé-10	○	○	○	○	○	○	○	○	○	○	○

WE 2.13 Commentaire : Utilisez cette section pour apporter des commentaires additionnels sur le niveau d'efforts, les défis et les succès du pays autour des services et programmes pour l'autonomisation des femmes. Si vous avez répondu « Je ne sais pas » à l'une des questions ci-dessus, utilisez aussi cette section pour expliquer pourquoi.

JOHNS HOPKINS
BLOOMBERG SCHOOL
of PUBLIC HEALTH

Bill & Melinda Gates Institute for Population and Reproductive Health

Enquête de l'Indice des efforts pour le dividende démographique

Domaine 3. Plaidoyer

* WE 3.1 Les plaideurs : Mesure dans laquelle des plaideurs pour l'autonomisation des filles et des femmes existent au niveau national et infranational.

	1	2	3	4	5	6	7	8	9	10	Je ne sais pas
1-Plus faible -> Plus élevé-10	○	○	○	○	○	○	○	○	○	○	○

* WE 3.2. Les filles et les femmes : Mesure dans laquelle les efforts de plaidoyer dans ce domaine recouvrent l'ensemble des aspects de l'autonomisation des filles et des femmes.

	1	2	3	4	5	6	7	8	9	10	Je ne sais pas
1-Plus faible -> Plus élevé-10	○	○	○	○	○	○	○	○	○	○	○

* WE 3.3 La dissémination de l'information : Mesure dans laquelle le gouvernement entreprend suffisamment d'efforts pour veiller à ce que la population soit consciente des problèmes liés à l'autonomisation des femmes, et si les informations disséminées sont exactes et communiquées dans la langue parlée par les destinataires/publics cibles.

	1	2	3	4	5	6	7	8	9	10	Je ne sais pas
1-Plus faible -> Plus élevé-10	○	○	○	○	○	○	○	○	○	○	○

* WE 3.4 La mise à profit des partenariats : Mesure dans laquelle le gouvernement met à profit ses partenariats avec des OSC pour disséminer des informations sur l'autonomisation des femmes à travers des campagnes de sensibilisation/information.

	1	2	3	4	5	6	7	8	9	10	Je ne sais pas
1-Plus faible -> Plus élevé-10	○	○	○	○	○	○	○	○	○	○	○

* WE 3.5 L'emploi : Mesure dans laquelle les parties prenantes appellent à l'élaboration de lois, politiques et programmes pour que les femmes puissent combiner leur rôle de mère pendant la grossesse, l'allaitement et l'éducation de leurs enfants avec leur participation à la vie active.

	1	2	3	4	5	6	7	8	9	10	Je ne sais pas
1-Plus faible -> Plus élevé-10	◯	◯	◯	◯	◯	◯	◯	◯	◯	◯	◯

* WE 3.6 L'éducation : Mesure dans laquelle l'une des priorités des plaideurs est de promouvoir la réalisation du plein potentiel des filles et des femmes à travers l'école et le développement de compétences.

	1	2	3	4	5	6	7	8	9	10	Je ne sais pas
1-Plus faible -> Plus élevé-10	◯	◯	◯	◯	◯	◯	◯	◯	◯	◯	◯

* WE 3.7 La violence : Mesure dans laquelle les plaideurs réussissent à interpeller le gouvernement pour le faire réagir et répondre aux cas de violence et de discrimination contre les femmes.

	1	2	3	4	5	6	7	8	9	10	Je ne sais pas
1-Plus faible -> Plus élevé-10	◯	◯	◯	◯	◯	◯	◯	◯	◯	◯	◯

WE 3.8 Commentaire : Utilisez cette section pour apporter des commentaires additionnels sur le niveau d'efforts, les défis et les succès du pays autour du plaidoyer pour l'autonomisation des femmes. Si vous avez répondu « Je ne sais pas » à l'une des questions ci-dessus, utilisez aussi cette section pour expliquer pourquoi.

JOHNS HOPKINS
BLOOMBERG SCHOOL
of PUBLIC HEALTH

Bill & Melinda Gates Institute for Population and Reproductive Health

Enquête de l'Indice des efforts pour le dividende démographique

Domaine 4. Recherche

* WE 4.1 La force des observatoires/parties prenantes/groupes de travail pour l'autonomisation des femmes : Mesure dans laquelle les parties prenantes du secteur (ex : groupe directif de parties prenantes, groupes de travail techniques, observatoire de l'autonomisation des femmes, groupe de coordination ou facilitation) :

	1	2	3	4	5	6	7	8	9	10	Je ne sais pas
WE 4.1.1 Ont un représentant du gouvernement, des institutions de formation, de la société civile, des organisations non gouvernementales et confessionnelles, des associations professionnelles, du secteur privé, etc.	○	○	○	○	○	○	○	○	○	○	○
WE 4.1.2 Se réunissent régulièrement, émettent des rapports et recommandent des politiques aux directions des ministères compétents.	○	○	○	○	○	○	○	○	○	○	○
WE 4.1.3 Ont un impact sur l'autonomisation des femmes dans le pays.	○	○	○	○	○	○	○	○	○	○	○

* WE 4.2 La stratégie de recherche sur les droits des femmes : Mesure dans laquelle le plan national pour l'autonomisation des femmes et/ou autres documents nationaux comprennent une approche/stratégie de recherche sur l'autonomisation des femmes, partagée entre les partenaires.

	1	2	3	4	5	6	7	8	9	10	Je ne sais pas
1-Plus faible -> Plus élevé-10	○	○	○	○	○	○	○	○	○	○	○

* WE 4.3 Partenaires collectant des données :

	1	2	3	4	5	6	7	8	9	10	Je ne sais pas
WE 4.3.1 Mesure dans laquelle la collecte des données est entreprise par des agences gouvernementales. (Cela peut comprendre les agences/bureaux de la statistique et/ou les ministères de l'économie/finances, éducation, santé, genre, etc.)	◯	◯	◯	◯	◯	◯	◯	◯	◯	◯	◯
WE 4.3.2 Mesure dans laquelle la collecte de données est entreprise par des institutions de recherche.	◯	◯	◯	◯	◯	◯	◯	◯	◯	◯	◯
WE 4.3.3 Mesure dans laquelle la collecte des données est entreprise par des chercheurs indépendants.	◯	◯	◯	◯	◯	◯	◯	◯	◯	◯	◯

* WE 4.4 La recherche thématique :

	1	2	3	4	5	6	7	8	9	10	Je ne sais pas
WE 4.4.1 Mesure dans laquelle de la recherche est menée sur les normes sociales et culturelles affectant l'autonomisation des femmes et la parité.	◯	◯	◯	◯	◯	◯	◯	◯	◯	◯	◯
WE 4.4.2 Mesure dans laquelle de la recherche est menée sur les effets économiques et sanitaires de l'autonomisation des femmes.	◯	◯	◯	◯	◯	◯	◯	◯	◯	◯	◯
WE 4.4.3 Mesure dans laquelle de la recherche est menée sur le mariage d'enfants pour informer la politique.	◯	◯	◯	◯	◯	◯	◯	◯	◯	◯	◯
WE 4.4.4 Mesure dans laquelle de la recherche est menée sur les résultats de l'éducation et de l'acquisition de compétences chez les filles.	◯	◯	◯	◯	◯	◯	◯	◯	◯	◯	◯

* WE 4.5 La qualité/ couverture des données :

	1	2	3	4	5	6	7	8	9	10	Je ne sais pas
WE 4.5.1 Mesure dans laquelle un système statistique de routine fiable fournit des données périodiques désagrégées par âge.	○	○	○	○	○	○	○	○	○	○	○
WE 4.5.2 Mesure dans laquelle un système statistique de routine fiable fournit de bonnes données périodiques désagrégées par âge sur les besoins relatifs à l'autonomie des femmes parmi les populations de différentes communautés.	○	○	○	○	○	○	○	○	○	○	○
WE 4.5.3 Mesure dans laquelle les systèmes de suivi et de surveillance sont évalués, et ces évaluations appliquées pour assurer la fiabilité des données désagrégées par âge.	○	○	○	○	○	○	○	○	○	○	○

* WE 4.6 La qualité des institutions de recherche : Mesure dans laquelle le pays soutient et maintient des institutions de recherche qui développent des études permettant de soutenir la portée et la qualité de la collecte de données pour informer les efforts nationaux d'autonomisation des femmes.

	1	2	3	4	5	6	7	8	9	10	Je ne sais pas
1-Plus faible -> Plus élevé-10	○	○	○	○	○	○	○	○	○	○	○

* WE 4.7 L'évaluation : Mesure dans laquelle les statistiques, enquêtes et études sur les programmes sont utilisées par du personnel spécialisé pour rapporter les opérations du programme et mesurer les progrès en matière d'autonomisation des femmes et de parité.

	1	2	3	4	5	6	7	8	9	10	Je ne sais pas
1-Plus faible -> Plus élevé-10	○	○	○	○	○	○	○	○	○	○	○

* WE 4.8 L’utilisation des résultats d’évaluation par les gestionnaires de programme : Mesure dans laquelle les gestionnaires locaux des programmes d’autonomisation des femmes utilisent les conclusions de recherche et d’évaluations pour améliorer ces programmes en suivant les recommandations émises.

	1	2	3	4	5	6	7	8	9	10	Je ne sais pas
1-Plus faible -> Plus élevé-10	◯	◯	◯	◯	◯	◯	◯	◯	◯	◯	◯

* WE 4.9 L’utilisation des résultats d’évaluation par les ministères : Mesure dans laquelle les administrateurs ministériels compétents utilisent systématiquement les données pour informer les politiques et interventions visant à résoudre les problèmes liés à l’autonomisation des femmes.

	1	2	3	4	5	6	7	8	9	10	Je ne sais pas
1-Plus faible -> Plus élevé-10	◯	◯	◯	◯	◯	◯	◯	◯	◯	◯	◯

* WE 4.10 La dissémination d’informations à d’autres entités chargées de la mise en œuvre des programmes : Mesure dans laquelle des informations sont partagées ou disséminées entre géographies et à différents niveaux (national, état/province/comté, localités/sous-comtés).

	1	2	3	4	5	6	7	8	9	10	Je ne sais pas
1-Plus faible -> Plus élevé-10	◯	◯	◯	◯	◯	◯	◯	◯	◯	◯	◯

WE 4.11 Commentaire : Utilisez cette section pour apporter des commentaires additionnels sur le niveau d’efforts, les défis et les succès du pays autour de la recherche sur l’autonomisation des femmes. Si vous avez répondu « Je ne sais pas » à l’une des questions ci-dessus, utilisez aussi cette section pour expliquer pourquoi.

JOHNS HOPKINS
BLOOMBERG SCHOOL of PUBLIC HEALTH

Bill & Melinda Gates Institute for Population and Reproductive Health

Enquête de l'Indice des efforts pour le dividende démographique

Domaine 5. Organisations de la Société Civile (OSC)

* WE 5.1 Le pouvoir des acteurs des OSC : Mesure dans laquelle les acteurs des OSC sont bien placés pour influencer la politique nationale concernant l'autonomisation des femmes.

	1	2	3	4	5	6	7	8	9	10	Je ne sais pas
1-Plus faible -> Plus élevé-10	◯	◯	◯	◯	◯	◯	◯	◯	◯	◯	◯

* WE 5.2 L'analyse budgétaire comme outil des OSC : Mesure dans laquelle les OSC utilisent l'analyse budgétaire comme un outil pour développer le plaidoyer en matière d'autonomisation des femmes

	1	2	3	4	5	6	7	8	9	10	Je ne sais pas
1-Plus faible -> Plus élevé-10	◯	◯	◯	◯	◯	◯	◯	◯	◯	◯	◯

* WE 5.3 Le soutien des services à base communautaire : Mesure dans laquelle les OSC participent au rapprochement des informations et services en matière d'autonomisation des femmes avec les communautés à travers des activités comprenant, mais sans s'y limiter, le renforcement de compétences des filles, les défenseurs/champions de l'autonomisation des femmes, et des campagnes d'éducation ou d'information pour augmenter l'engagement des familles et des communautés pour l'autonomie des femmes.

	1	2	3	4	5	6	7	8	9	10	Je ne sais pas
1-Plus faible -> Plus élevé-10	◯	◯	◯	◯	◯	◯	◯	◯	◯	◯	◯

* WE 5.4 Les droits humains et la qualité de la mise en œuvre : Mesure dans laquelle les OSC plaident et soutiennent des approches de l'autonomisation des femmes fondées sur le droit pour veiller à ce que les bénéficiaires des programmes et politiques d'autonomisation des femmes puissent voir des avancées selon leurs propres termes.

	1	2	3	4	5	6	7	8	9	10	Je ne sais pas
1-Plus faible -> Plus élevé-10	○	○	○	○	○	○	○	○	○	○	○

* WE 5.5 Le changement social et des comportements (CSC) : Mesure dans laquelle les OSC soutiennent des interventions pour le CSC comprenant, mais sans s'y limiter, les programmes transformateurs de genre, les compétences de vie et l'éducation entre pairs, l'éducation à la santé de l'adulte, l'éducation en milieu scolaire, la communication au sein du couple, la mobilisation communautaire, les interventions des gardiens de la communauté et/ou des interventions dans les médias de masse pour le grand public. Ces interventions peuvent être mises en œuvre au niveau communautaire ou individuel.

	1	2	3	4	5	6	7	8	9	10	Je ne sais pas
1-Plus faible -> Plus élevé-10	○	○	○	○	○	○	○	○	○	○	○

* WE 5.6 Les jeunes : Mesure dans laquelle les OSC promeuvent l'autonomisation des femmes à travers les programmes pour jeunes.

	1	2	3	4	5	6	7	8	9	10	Je ne sais pas
1-Plus faible -> Plus élevé-10	○	○	○	○	○	○	○	○	○	○	○

* WE 5.7 Les programmes pour les hommes : Mesure dans laquelle les OSC soutiennent les politiques, interventions et programmes d'autonomisation des femmes ciblant les hommes.

	1	2	3	4	5	6	7	8	9	10	Je ne sais pas
1-Plus faible -> Plus élevé-10	○	○	○	○	○	○	○	○	○	○	○

* WE 5.8 Le plaidoyer/ la redevabilité : Mesure dans laquelle les OSC soutiennent la formation et le renforcement de compétences des membres de la communauté pour augmenter leur compréhension des politiques, processus et activités, et pour renforcer leurs capacités à identifier les problèmes liés à l'autonomisation des femmes, résoudre collectivement ces problèmes et mener des actions de plaidoyer plus ciblées.

	1	2	3	4	5	6	7	8	9	10	Je ne sais pas
1-Plus faible -> Plus élevé-10	○	○	○	○	○	○	○	○	○	○	○

* WE 5.9 L'évaluation et le suivi dirigés par les OSC : Mesure dans laquelle les OSC évaluent, suivent et émettent des rapports sur l'efficacité des politiques et programmes pour améliorer la redevabilité des prestataires et des politiques d'autonomisation des femmes.

	1	2	3	4	5	6	7	8	9	10	Je ne sais pas
1-Plus faible -> Plus élevé-10	◯	◯	◯	◯	◯	◯	◯	◯	◯	◯	◯

* WE 5.10 Les partenariats entre OSC : Mesure dans laquelle les OSC ont formé des alliances nationales et des partenariats régionaux pour renforcer leur position et potentiellement améliorer le leadership et les mécanismes de financement afin de faire progresser l'agenda de l'autonomisation des femmes.

	1	2	3	4	5	6	7	8	9	10	Je ne sais pas
1-Plus faible -> Plus élevé-10	◯	◯	◯	◯	◯	◯	◯	◯	◯	◯	◯

WE 5.11 Commentaire : Utilisez cette section pour apporter des commentaires additionnels sur le niveau d'efforts, les défis et les succès du pays concernant le rôle des OSC pour l'autonomisation des femmes. Si vous avez répondu « Je ne sais pas » à l'une des questions ci-dessus, utilisez aussi cette section pour expliquer pourquoi.

Bill & Melinda Gates Institute for Population and Reproductive Health

Enquête de l’Indice des efforts pour le dividende démographique

Module : Résilience et durabilité du secteur

Étant donné l’impact sanitaire et socioéconomique de la pandémie de COVID-19, et son impact probable sur la progression vers le DD, cet indice des efforts pour le DD intègre des questions pour évaluer la résilience et la durabilité des systèmes dans les secteurs clés du DD. Les éléments couverts par les questions liées à la pandémie de COVID-19 ci-dessous relèvent des dimensions clés des systèmes résilients et visent à apporter des informations déterminantes sur le potentiel d’une réponse efficace aux menaces de maladies infectieuses émergentes et autres crises de santé publique.

Veuillez noter chaque élément sur une échelle de 1 à 10, 1 étant le score le plus faible (faible état/capacité) et 10 le plus élevé (très bon état/capacité).

* Dimension physique : structures, équipements, états du système et capacités.

	1	2	3	4	5	6	7	8	9	10	Je ne sais pas
WE-M1 - Plan/Préparation - État et capacité de l’équipement, du personnel et de la structure du secteur de l’autonomisation des femmes avant la crise. (1 = faible état/capacité ; 10 = très bon état/capacité)	○	○	○	○	○	○	○	○	○	○	○
WE-M2 - Absorption – Mesure dans laquelle le secteur de l’autonomisation des femmes a reconnu la pandémie de COVID-19 et pu continuer à être fonctionnel.	○	○	○	○	○	○	○	○	○	○	○

	1	2	3	4	5	6	7	8	9	10	Je ne sais pas
WE-M3 - Absorption – Intégration : Niveau d'intégration des services et programmes d'autonomisation des femmes pour mitiger les effets de la pandémie de COVID-19 sur l'autonomie des femmes.	○	○	○	○	○	○	○	○	○	○	○
WE-M4 - Récupération – Mesure dans laquelle les programmes d'autonomisation des femmes ont été capables d'induire des changements pour récupérer un niveau de fonctionnalité pré-COVID-19.	○	○	○	○	○	○	○	○	○	○	○
WE-M5 - Récupération – Gestion du financement et des bailleurs de fonds : Niveau de mobilisation des ressources et de l'allocation budgétaire pour mitiger les effets de la pandémie de COVID-19 sur l'autonomie des femmes.	○	○	○	○	○	○	○	○	○	○	○
WE-M6 - Adaptation – Mesure dans laquelle des changements ont été effectués pour améliorer la résilience du secteur de l'autonomisation des femmes.	○	○	○	○	○	○	○	○	○	○	○

* Dimension informatique : création, gestion, stockage et utilisation des données.

	1	2	3	4	5	6	7	8	9	10	Je ne sais pas
WE-M7 - Plan/Préparation – Mesure dans laquelle les données liées à l'autonomisation des femmes étaient suffisamment préparées, présentées, analysées et stockées avant la crise.	○	○	○	○	○	○	○	○	○	○	○

	1	2	3	4	5	6	7	8	9	10	Je ne sais pas
WE-M8 - Absorption – Connaissance : Mesure dans laquelle le secteur de l'autonomisation des femmes a pu conduire des évaluations en temps réel de son état de fonctionnement, en anticipation des pertes en cascade des services liés à l'autonomie des femmes.	○	○	○	○	○	○	○	○	○	○	○
WE-M9 - Absorption – Connaissance/ Conscience : Capacité du système de surveillance à détecter les limites/ menaces posées à l'autonomie des femmes par la pandémie de COVID-19.	○	○	○	○	○	○	○	○	○	○	○
WE-M10 - Mesure dans laquelle les données sur l'autonomisation des femmes ont été utilisées pour suivre les progrès vers la récupération et anticiper des scenarii de récupération.	○	○	○	○	○	○	○	○	○	○	○
WE-M11 - Adaptation – Mesure dans laquelle le secteur de l'autonomisation des femmes crée et améliore ses protocoles de stockage et utilisation en temps réel de ses données sur l'autonomisation des femmes.	○	○	○	○	○	○	○	○	○	○	○

* Dimension cognitive : compréhension, modèles cognitifs, idées reçues, préjugés et valeurs.

	1	2	3	4	5	6	7	8	9	10	Je ne sais pas
WE-M12 - Planification/Préparation – Mesure dans laquelle le système de conception et les décisions opérationnelles étaient préparés à anticiper la crise.	○	○	○	○	○	○	○	○	○	○	○

	1	2	3	4	5	6	7	8	9	10	Je ne sais pas
WE-M13 - Absorption – Mesure dans laquelle le secteur de l'autonomisation des femmes a des protocoles de contingence suffisants et une gestion de crise proactive.	○	○	○	○	○	○	○	○	○	○	○
WE-M14 - Absorption – Redevabilité : Niveau d'effort pour assurer une redevabilité optimale vis-à-vis des ressources allouées à l'autonomisation des femmes dans le cadre de la réponse à la pandémie de COVID-19. (Cela peut prendre la forme de soutien aux services atténués pour répondre aux besoins de protection des femmes et des filles).	○	○	○	○	○	○	○	○	○	○	○
WE-M15 - Récupération – Mesure dans laquelle les décisions sont orientées sur la récupération et communiquées aux communautés sur la base de données probantes pour promouvoir des comportements sûrs	○	○	○	○	○	○	○	○	○	○	○

	1	2	3	4	5	6	7	8	9	10	Je ne sais pas
WE-M16 - Récupération – Connaissance/Conscience : Mesure dans laquelle le secteur établit une certaine confiance à travers sa communication sur les barrières posées par la pandémie de COVID-19 pour soutenir l’accès et l’utilisation des services et programmes pour l’autonomisation des femmes. (Cela comprend la surveillance des disparités de genre dans les réponses à la pandémie et/ou la surveillance augmentée des questions liées à l’autonomie des femmes, comme la violence basée sur le genre).	○	○	○	○	○	○	○	○	○	○	○
WE-M17 - Adaptation – Mesure dans laquelle le secteur de l’autonomisation des femmes conçoit de nouvelles configurations systémiques, objectifs et critères de décision.	○	○	○	○	○	○	○	○	○	○	○
WE-M18 Adaptation - Niveau d'adaptation pour répondre aux menaces COVID-19 qui pèsent sur l’autonomisation des femmes	○	○	○	○	○	○	○	○	○	○	○

* Social dimension: interaction, collaboration and self-synchronization between people, entities and institutions.

	1	2	3	4	5	6	7	8	9	10	Je ne sais pas
WE-M19 - Planification /Préparation – Mesure dans laquelle une formation sur la gestion d'épidémies/crises a été menée et a permis de mettre à profit les réseaux sociaux, le capital social et les normes institutionnelles et culturelles avant la crise.	○	○	○	○	○	○	○	○	○	○	○
WE-M20 - Absorption – Mesure dans laquelle le personnel et les institutions sociales ont été accessibles et ont fait preuve d'ingéniosité dans leur réponse à l'épidémie/crise.	○	○	○	○	○	○	○	○	○	○	○
WE-M21 - Absorption - Self-regulation: Extent to which national leaders had the authority to effect timely sectoral changes through an infrastructure flexible.	○	○	○	○	○	○	○	○	○	○	○
WE-M22 - Récupération – Mesure dans laquelle le secteur de l'autonomisation des femmes est impliqué dans le partage de connaissances et d'équipes pour améliorer la récupération du système.	○	○	○	○	○	○	○	○	○	○	○
WE-M23 – Niveau d'engagement d'une équipe multidisciplinaire pour mitiger les effets de la pandémie de COVID-19 sur l'autonomisation des femmes.	○	○	○	○	○	○	○	○	○	○	○

	1	2	3	4	5	6	7	8	9	10	Je ne sais pas
WE-M24 - Adaptation - Leadership et management : Niveau de leadership démontré par les dirigeants du secteur de l'autonomisation des femmes pour mitiger les effets de la pandémie de COVID-19 sur l'autonomie des femmes.	◯	◯	◯	◯	◯	◯	◯	◯	◯	◯	◯

* WE M-25 Temporalité : Mesure de la rapidité de la réaction du gouvernement –sa communication et mise en œuvre des mesures- pour mitiger l'impact immédiat et sur le long terme de la pandémie de COVID-19. Cette question porte sur la riposte nationale dans son ensemble, et non la réponse spécifique de votre secteur.

	1	2	3	4	5	6	7	8	9	10	Je ne sais pas
1-Plus faible -> Plus élevé-10	◯	◯	◯	◯	◯	◯	◯	◯	◯	◯	◯

WE M-26 Commentaires : Veuillez utiliser cette section pour tout commentaire additionnel sur la résilience et la durabilité des opportunités et systèmes nationaux d'autonomisation des femmes et des filles en fonction de votre expérience de la pandémie de COVID-19. Si vous avez répondu « Je ne sais pas » à l'une des questions, utilisez aussi cette section pour expliquer pourquoi.

JOHNS HOPKINS
BLOOMBERG SCHOOL
of PUBLIC HEALTH

Bill & Melinda Gates Institute for Population and Reproductive Health

Enquête de l'Indice des efforts pour le dividende démographique

Questionnaire sur le marché du travail (MT)

Répondez aux questions suivantes en fonction de votre expérience/ expertise dans ce secteur spécifique.

Afin d'obtenir une représentation synthétique des efforts nationaux pour le dividende démographique, notez les éléments suivants sur une échelle de 1 à 10, 1 étant le score le plus faible (efforts très faibles ou quasi-inexistants) et 10 le plus élevé (efforts robustes). Le cas échéant, si une politique ou activité n'existe pas, répondez 0.

Donnez un score à chaque élément. Toutes les réponses seront enregistrées au format illustré ci-dessous :

Effort le plus faible	1	2	3	4	5	6	7	8	9	10	Effort le plus élevé	Je ne sais pas

JOHNS HOPKINS
BLOOMBERG SCHOOL
of PUBLIC HEALTH

Bill & Melinda Gates Institute for Population and Reproductive Health

Enquête de l’Indice des efforts pour le dividende démographique

Domaine 1. Politique/ Prise de décision

* LM 1.1 L’intégration de l’économie mondiale : Mesure dans laquelle le pays vise à s’intégrer à l’économie mondiale.

	1	2	3	4	5	6	7	8	9	10	Je ne sais pas
LM 1.1.1 Mesure dans laquelle le pays légifère pour l’ouverture des échanges commerciaux.	◯	◯	◯	◯	◯	◯	◯	◯	◯	◯	◯
LM 1.1.2 Niveau d’effort en faveur de la croissance des exportations nationales.	◯	◯	◯	◯	◯	◯	◯	◯	◯	◯	◯

* LM 1.2 L’intégration économique régionale : Mesure dans laquelle le pays vise à s’intégrer à l’économie régionale.

	1	2	3	4	5	6	7	8	9	10	Je ne sais pas
1-Plus faible -> Plus élevé-10	◯	◯	◯	◯	◯	◯	◯	◯	◯	◯	◯

* LM 1.3 La technologie : Mesure dans laquelle le gouvernement a investi dans l’infrastructure, à la fois traditionnelle et technologique, pour permettre au pays de saisir les opportunités de demain/ d’intégration technologique en complément de la main d’œuvre.

	1	2	3	4	5	6	7	8	9	10	Je ne sais pas
1-Plus faible -> Plus élevé-10	◯	◯	◯	◯	◯	◯	◯	◯	◯	◯	◯

* LM 1.4 Le soutien du gouvernement : Mesure dans laquelle des politiques et plans sont en place et prévoient des dispositifs de protection sociale pour une transition harmonieuse entre les différents emplois, ou « petits boulots », des personnes, et pour les protéger de la précarité/ volatilité des revenus.

	1	2	3	4	5	6	7	8	9	10	Je ne sais pas
LM 1.4.1 Mesure dans laquelle une politique nationale établissant un salaire minimum est en place.	◯	◯	◯	◯	◯	◯	◯	◯	◯	◯	◯
LM 1.4.2 Mesure dans laquelle une politique nationale définissant un système d'assurance chômage est en place, et niveau d'efficacité de sa mise en œuvre.	◯	◯	◯	◯	◯	◯	◯	◯	◯	◯	◯

* LM 1.5 Le chômage : Mesure dans laquelle les politiques rendent compte et répondent au chômage chez différents groupes de la population et dans différentes aires géographiques.

	1	2	3	4	5	6	7	8	9	10	Je ne sais pas
1-Plus faible -> Plus élevé-10	◯	◯	◯	◯	◯	◯	◯	◯	◯	◯	◯

* LM 1.6 Les groupes défavorisés : Mesure dans laquelle des politiques existent, et sont effectivement mises en œuvre, pour soutenir les opportunités d'embauche des groupes défavorisés (c'est-à-dire les filles et les femmes, les jeunes, les personnes en situation de mobilité réduite ou de handicap, etc.).

	1	2	3	4	5	6	7	8	9	10	Je ne sais pas
1-Plus faible -> Plus élevé-10	◯	◯	◯	◯	◯	◯	◯	◯	◯	◯	◯

* LM 1.7 Le changement climatique et le développement durable : Mesure dans laquelle les secteurs de l'agriculture et de la production industrielle sont préparés aux impacts des effets du changement climatique sur le marché du travail en élaborant des stratégies de planification familiale en vue des besoins du marché de travail de demain.

	1	2	3	4	5	6	7	8	9	10	Je ne sais pas
1-Plus faible -> Plus élevé-10	◯	◯	◯	◯	◯	◯	◯	◯	◯	◯	◯

* LM 1.8 L'urbanisation : Mesure dans laquelle les politiques et la planification anticipent et sont préparées à la croissance urbaine résultant et contribuant au changement climatique, et ses impacts particuliers sur les marchés du travail.

	1	2	3	4	5	6	7	8	9	10	Je ne sais pas
1-Plus faible -> Plus élevé-10	○	○	○	○	○	○	○	○	○	○	○

* LM 1.9 L'économie de services : Mesure dans laquelle des politiques et des cadres de travail sont en place pour faire passer les économies basées sur du travail informel au travail formalisé répondant aux demandes locales, régionales et mondiales de biens et de services.

	1	2	3	4	5	6	7	8	9	10	Je ne sais pas
1-Plus faible -> Plus élevé-10	○	○	○	○	○	○	○	○	○	○	○

* LM 1.10 La responsabilité sociale d'entreprise : Mesure dans laquelle la législation nationale du marché du travail utilise la responsabilité sociale des entreprises (RSE) pour soutenir l'entreprenariat chez les jeunes.

	1	2	3	4	5	6	7	8	9	10	Je ne sais pas
1-Plus faible -> Plus élevé-10	○	○	○	○	○	○	○	○	○	○	○

* LM 1.11 Les investissements ciblés dans le marché du travail : Mesure dans laquelle le gouvernement a ciblé ses investissements sur les secteurs générateurs d'emplois, y compris les Technologies de l'Information et la Communication (TIC), le secteur de production industrielle, l'agriculture et les entreprises industrielles agricoles, pour générer de l'emploi et encourager la croissance inclusive.

	1	2	3	4	5	6	7	8	9	10	Je ne sais pas
1-Plus faible -> Plus élevé-10	○	○	○	○	○	○	○	○	○	○	○

* LM 1.12 Les travailleurs indépendants : Mesure dans laquelle les politiques soutiennent l'élévation des retours sur investissement des travailleurs indépendants et la création de plus d'opportunités pour passer du travail indépendant à des emplois mieux rémunérés. (Cela comprend le travail en milieu rural).

	1	2	3	4	5	6	7	8	9	10	Je ne sais pas
1-Plus faible -> Plus élevé-10	○	○	○	○	○	○	○	○	○	○	○

* LM 1.13 Le microcrédit : Mesure dans laquelle le gouvernement permet l'accès à des microcrédits pour exercer des activités à but lucratif, sécurisant des crédits abordables pour les petites et moyennes entreprises et les travailleurs indépendants.

	1	2	3	4	5	6	7	8	9	10	Je ne sais pas
1-Plus faible -> Plus élevé-10											

* LM 1.14 L'épargne : Mesure dans laquelle le gouvernement soutient la création de produits financiers accessibles et des politiques promouvant la culture de l'épargne.

	1	2	3	4	5	6	7	8	9	10	Je ne sais pas
1-Plus faible -> Plus élevé-10											

* LM 1.15 L'acquisition de compétences : Mesure dans laquelle des politiques existent, et sont mises en œuvre efficacement, pour soutenir l'acquisition de compétences valorisées sur le marché du travail d'aujourd'hui et de demain.

	1	2	3	4	5	6	7	8	9	10	Je ne sais pas
1-Plus faible -> Plus élevé-10											

LM 1.16 Commentaires : Veuillez utiliser cette section pour nous faire part de tout commentaire additionnel sur le niveau d'effort, les défis ou les succès du pays autour de la politique/prise de décision dans le secteur du marché du travail. Si vous avez répondu « Je ne sais pas » à l'une des questions précédentes, veuillez utiliser cette section pour expliquer pourquoi.

JOHNS HOPKINS
BLOOMBERG SCHOOL of PUBLIC HEALTH

Bill & Melinda Gates Institute for Population and Reproductive Health

Enquête de l'Indice des efforts pour le dividende démographique

Domaine 2. Services ou programmes

* LM 2.1 La réduction du chômage : Mesure dans laquelle des efforts sont menés pour développer et mettre en œuvre des stratégies visant à réduire la proportion des personnes au chômage par année cible (en accord avec le Plan de mise en œuvre des dix premières années de l'Agenda 2063).

	1	2	3	4	5	6	7	8	9	10	Je ne sais pas
1-Plus faible -> Plus élevé-10											

* LM 2.2. Le chômage chez les jeunes : Mesure dans laquelle des programmes liés au marché du travail ont effectivement réduit le taux de chômage chez les jeunes en particulier.

	1	2	3	4	5	6	7	8	9	10	Je ne sais pas
1-Plus faible -> Plus élevé-10											

* LM 2.3 L'accès au microcrédit : Mesure dans laquelle les fonds nationaux et régionaux ont été établis et opérationnalisés pour augmenter l'accès des personnes au capital commercial abordable.

	1	2	3	4	5	6	7	8	9	10	Je ne sais pas
1-Plus faible -> Plus élevé-10											

* LM 2.4 L'exploitation des capacités uniques des jeunes : Mesure dans laquelle des programmes et services soutiennent les talents spécifiques des jeunes pour les aider à s'épanouir et réaliser leur plein potentiel. (Cela couvre les objectifs programmatiques spécifiques liés à l'acquisition de talents).

	1	2	3	4	5	6	7	8	9	10	Je ne sais pas
1-Plus faible -> Plus élevé-10											

* LM 2.5 Les opportunités de première embauche : Mesure dans laquelle les programmes et la législation du gouvernement engagent les partenaires du secteur privé à élargir les opportunités de stages, d'apprentissage professionnel et de formation continue au travail pour les femmes et les jeunes.

	1	2	3	4	5	6	7	8	9	10	Je ne sais pas
1-Plus faible -> Plus élevé-10	○	○	○	○	○	○	○	○	○	○	○

* LM 2.6 L'entrée des jeunes dans la population active : Mesure de la promotion du volontariat des jeunes, des programmes pour jeunes professionnelles et autres opportunités similaires pour améliorer leurs capacités et les exposer aux organisations régionales et internationales.

	1	2	3	4	5	6	7	8	9	10	Je ne sais pas
1-Plus faible -> Plus élevé-10	○	○	○	○	○	○	○	○	○	○	○

* LM 2.7 Les risques de l'entreprenariat : Mesure dans laquelle les personnes ont un meilleur accès aux services d'achat et de finance du gouvernement pour réduire les difficultés liées au lancement d'un projet professionnel et/ou à la conduite d'activités commerciales dans et entre les pays.

	1	2	3	4	5	6	7	8	9	10	Je ne sais pas
1-Plus faible -> Plus élevé-10	○	○	○	○	○	○	○	○	○	○	○

* LM 2.8 Les ressources pour l'entreprenariat des jeunes : Mesure dans laquelle les projets d'entreprenariat des jeunes bénéficient d'une mobilisation de ressources directe et ciblée, soutenue par un cadre régulateur, pour réduire les difficultés liées au lancement d'un projet professionnel et/ou à la conduite d'activités commerciales dans et entre les pays.

	1	2	3	4	5	6	7	8	9	10	Je ne sais pas
1-Plus faible -> Plus élevé-10	○	○	○	○	○	○	○	○	○	○	○

* LM 2.9 Les opportunités professionnelles : Mesure dans laquelle les opportunités de formation professionnelles sont amplifiées pour permettre l'acquisition de compétences et améliorer les capacités d'insertion professionnelle, la productivité et la compétitivité.

	1	2	3	4	5	6	7	8	9	10	Je ne sais pas
1-Plus faible -> Plus élevé-10	○	○	○	○	○	○	○	○	○	○	○

* LM 2.10 L’éducation et l’insertion professionnelle : Mesure dans laquelle des améliorations ont été apportées pour favoriser l’accès inclusif à l’éducation à tous les niveaux et offrir des alternatives viables à de nombreux jeunes, particulièrement les filles adolescentes, qui abandonnent le système d’éducation scolaire formel, en facilitant leur réinsertion, en restructurant l’éducation informelle et la formation via une certification standardisée dans et entre les pays.

	1	2	3	4	5	6	7	8	9	10	Je ne sais pas
1-Plus faible -> Plus élevé-10	○	○	○	○	○	○	○	○	○	○	○

* LM 2.11 Le travail « ami des femmes » : Mesure dans laquelle les lieux de travail dans les secteurs privé et public ont adopté des politiques et pratiques « amies des femmes » pour éliminer toute forme de discrimination basée sur le genre, tout en leur permettant de prendre pleinement part à la population active.

	1	2	3	4	5	6	7	8	9	10	Je ne sais pas
1-Plus faible -> Plus élevé-10	○	○	○	○	○	○	○	○	○	○	○

* LM 2.12 La sécurité de l’emploi :

	1	2	3	4	5	6	7	8	9	10	Je ne sais pas
LM 2.12.1 Mesure dans laquelle les périodes de préavis relatifs aux plans sociaux et au licenciement sont respectées.	○	○	○	○	○	○	○	○	○	○	○
LM 2.12.2 Mesure dans laquelle les indemnités de départ dans le cadre de plans sociaux ou de licenciements sont appliquées.	○	○	○	○	○	○	○	○	○	○	○

* LM 2.13 Le salaire minimum : Mesure dans laquelle un salaire minimum est appliqué au travailleur « type » (défini comme un salarié moyen, issu de groupes ethniques et religieux majoritaires, vivant dans les grandes villes, et non syndicalisé).

	1	2	3	4	5	6	7	8	9	10	Je ne sais pas
1-Plus faible -> Plus élevé-10	○	○	○	○	○	○	○	○	○	○	○

* LM 2.14 Les programmes du marché du travail : Mesure dans laquelle les programmes du marché du travail ont effectivement diversifié la main d'œuvre et les besoins du marché du travail, tout en diversifiant simultanément les compétences de cette main d'œuvre.

	1	2	3	4	5	6	7	8	9	10	Je ne sais pas
1-Plus faible -> Plus élevé-10	○	○	○	○	○	○	○	○	○	○	○

* LM 2.15 L'informalité sur le marché du travail : Mesure dans laquelle des programmes sont mis en œuvre pour transformer le travail informel en travail légal.

	1	2	3	4	5	6	7	8	9	10	Je ne sais pas
1-Plus faible -> Plus élevé-10	○	○	○	○	○	○	○	○	○	○	○

LM 2.16 Commentaires : Veuillez utiliser cette section pour nous faire part de tout commentaire additionnel sur le niveau d'effort, les défis ou les succès du pays autour des services et programmes dans le secteur du marché du travail. Si vous avez répondu « Je ne sais pas » à l'une des questions précédentes, veuillez utiliser cette section pour expliquer pourquoi.

Johns Hopkins Bloomberg School of Public Health

Bill & Melinda Gates Institute for Population and Reproductive Health

Enquête de l’Indice des efforts pour le dividende démographique

Domaine 3. Plaidoyer

* LM 3.1 La parité sur le marché du travail : Mesure dans laquelle les groupes ou individus appellent, et voient des améliorations, en matière de parité et d’égalité hommes-femmes sur les lieux de travail.

	1	2	3	4	5	6	7	8	9	10	Je ne sais pas
1-Plus faible -> Plus élevé-10	○	○	○	○	○	○	○	○	○	○	○

* LM 3.2 L’égalité des jeunes sur le marché du travail : Mesure dans laquelle les groupes et les individus appellent à des changements du marché du travail et des régulations au bénéfice des populations jeunes rurales et urbaines.

	1	2	3	4	5	6	7	8	9	10	Je ne sais pas
1-Plus faible -> Plus élevé-10	○	○	○	○	○	○	○	○	○	○	○

* LM 3.3 Le plaidoyer pour le salaire minimum : Mesure dans laquelle les groupes de plaidoyer réussissent à inciter des lois pour le salaire minimum, ou une augmentation du salaire minimum si une telle loi existe déjà.

	1	2	3	4	5	6	7	8	9	10	Je ne sais pas
1-Plus faible -> Plus élevé-10	○	○	○	○	○	○	○	○	○	○	○

* LM 3.4 Le plaidoyer du gouvernement : Mesure dans laquelle les membres du gouvernement plaident pour des améliorations de ses propres structures et priorités. (Cela peut comprendre des progrès sur le marché du travail en matière d’inclusion, opportunités, programmes et droits des jeunes, et autres concepts similaires dans différentes populations).

	1	2	3	4	5	6	7	8	9	10	Je ne sais pas
1-Plus faible -> Plus élevé-10	○	○	○	○	○	○	○	○	○	○	○

* LM 3.5 Les protections sociales : Mesure dans laquelle il existe un plaidoyer pour la protection de l’emploi et l’assurance chômage.

	1	2	3	4	5	6	7	8	9	10	Je ne sais pas
1-Plus faible -> Plus élevé-10	○	○	○	○	○	○	○	○	○	○	○

LM 3.6 Commentaires : Veuillez utiliser cette section pour nous faire part de tout commentaire additionnel sur le niveau d’effort, les défis ou les succès du pays autour du plaidoyer dans le secteur du marché du travail. Si vous avez répondu « Je ne sais pas » à l’une des questions précédentes, veuillez utiliser cette section pour expliquer pourquoi.

JOHNS HOPKINS
BLOOMBERG SCHOOL of PUBLIC HEALTH

Bill & Melinda Gates Institute for Population and Reproductive Health

Enquête de l'Indice des efforts pour le dividende démographique

Domaine 4. Recherche

* LM 4.1 La force des observatoires/parties prenantes/groupes de travail dans le secteur du marché du travail : Mesure dans laquelle les parties prenantes du secteur du marché du travail (ex : groupe directif de parties prenantes, groupes de travail techniques, observatoire du marché du travail, groupe de coordination ou facilitation) :

	1	2	3	4	5	6	7	8	9	10	Je ne sais pas
LM 4.1.1 Ont un représentant du gouvernement, des institutions de formation, de la société civile, des organisations non gouvernementales et confessionnelles, des associations professionnelles, du secteur privé, etc.	◯	◯	◯	◯	◯	◯	◯	◯	◯	◯	◯
LM 4.1.2 Se réunissent régulièrement, émettent des rapports et recommandent des politiques aux directions des ministères compétents.	◯	◯	◯	◯	◯	◯	◯	◯	◯	◯	◯
LM 4.1.3 Ont un impact sur le marché du travail dans le pays.	◯	◯	◯	◯	◯	◯	◯	◯	◯	◯	◯

* LM 4.2 La stratégie de recherche sur le marché du travail : Mesure dans laquelle les documents nationaux prévoient une stratégie/approche complète pour la recherche sur le marché du travail qui soit partagée entre partenaires.

	1	2	3	4	5	6	7	8	9	10	Je ne sais pas
1-Plus faible -> Plus élevé-10	◯	◯	◯	◯	◯	◯	◯	◯	◯	◯	◯

* LM 4.3 Les partenaires de l'analyse du marché du travail :

	1	2	3	4	5	6	7	8	9	10	Je ne sais pas
LM 4.3.1 Mesure dans laquelle la collecte des données est entreprise par des agences gouvernementales.	○	○	○	○	○	○	○	○	○	○	○
LM 4.3.2 Mesure dans laquelle la collecte de données est entreprise par des institutions de recherche.	○	○	○	○	○	○	○	○	○	○	○
LM 4.3.3 Mesure dans laquelle la collecte des données est entreprise par des chercheurs indépendants.	○	○	○	○	○	○	○	○	○	○	○

* LM 4.4 Les domaines de la recherche sur le marché du travail :

	1	2	3	4	5	6	7	8	9	10	Je ne sais pas
LM 4.4.1 Mesure dans laquelle de la recherche est menée sur les tendances du chômage.	○	○	○	○	○	○	○	○	○	○	○
LM 4.4.2 Mesure dans laquelle de la recherche est menée sur les jeunes et le marché du travail.	○	○	○	○	○	○	○	○	○	○	○
LM 4.4.3 Mesure dans laquelle de la recherche est menée sur les femmes et le marché du travail.	○	○	○	○	○	○	○	○	○	○	○
LM 4.4.4 Mesure dans laquelle de la recherche est menée sur les tendances et les trajectoires des besoins du marché du travail.	○	○	○	○	○	○	○	○	○	○	○

* LM 4.5 La qualité/ couverture des données :

	1	2	3	4	5	6	7	8	9	10	Je ne sais pas
LM 4.5.1 Mesure dans laquelle un système statistique de routine fiable fournit des données périodiques sur le marché du travail.	○	○	○	○	○	○	○	○	○	○	○
LM 4.5.2 Mesure dans laquelle un système statistique de routine fiable fournit de bonnes données périodiques sur les besoins du marché du travail parmi les populations de différentes communautés.	○	○	○	○	○	○	○	○	○	○	○
LM 4.5.3 Mesure dans laquelle les systèmes de suivi et de surveillance sont évalués, et ces évaluations appliquées pour assurer la fiabilité des données.	○	○	○	○	○	○	○	○	○	○	○

* LM 4.6 Les registres : Mesure dans laquelle les systèmes de registre, rapports et retours/commentaires sur les résultats du marché du travail sont adéquats.

	1	2	3	4	5	6	7	8	9	10	Je ne sais pas
1-Plus faible -> Plus élevé-10	○	○	○	○	○	○	○	○	○	○	○

* LM 4.7 La qualité des institutions de recherche : Mesure dans laquelle le pays a la capacité de soutenir et maintenir les institutions de recherche qui développent des études/ collectent des données liées au marché du travail.

	1	2	3	4	5	6	7	8	9	10	Je ne sais pas
1-Plus faible -> Plus élevé-10	○	○	○	○	○	○	○	○	○	○	○

* LM 4.8 L'évaluation : Mesure dans laquelle les statistiques de programmes, enquêtes et études sont utilisées par du personnel spécialisé pour rapporter sur les opérations des programmes et mesurer le progrès.

	1	2	3	4	5	6	7	8	9	10	Je ne sais pas
1-Plus faible -> Plus élevé-10	○	○	○	○	○	○	○	○	○	○	○

* LM 4.9 L'utilisation des résultats d'évaluation par les décideurs : Mesure dans laquelle les gestionnaires locaux des programmes du marché du travail utilisent les conclusions de recherche et d'évaluations pour améliorer ces programmes en suivant les recommandations émises.

	1	2	3	4	5	6	7	8	9	10	Je ne sais pas
1-Plus faible -> Plus élevé-10	◯	◯	◯	◯	◯	◯	◯	◯	◯	◯	◯

* LM 4.10 L'utilisation des résultats d'évaluation par les ministères : Mesure dans laquelle les administrateurs ministériels compétents utilisent systématiquement les données pour informer les politiques et interventions visant à résoudre les problèmes liés au marché du travail.

	1	2	3	4	5	6	7	8	9	10	Je ne sais pas
1-Plus faible -> Plus élevé-10	◯	◯	◯	◯	◯	◯	◯	◯	◯	◯	◯

* LM 4.11 La dissémination d'informations à d'autres entités chargées de la mise en œuvre des programmes : Mesure dans laquelle des informations sont partagées ou disséminées entre géographies et à différents niveaux (national, état/province/comté, localités/sous-comtés).

	1	2	3	4	5	6	7	8	9	10	Je ne sais pas
1-Plus faible -> Plus élevé-10	◯	◯	◯	◯	◯	◯	◯	◯	◯	◯	◯

LM 4.12 Commentaires : Veuillez utiliser cette section pour nous faire part de tout commentaire additionnel sur le niveau d'effort, les défis ou les succès du pays autour de la recherche dans le secteur du marché du travail. Si vous avez répondu « Je ne sais pas » à l'une des questions précédentes, veuillez utiliser cette section pour expliquer pourquoi.

JOHNS HOPKINS
BLOOMBERG SCHOOL
of PUBLIC HEALTH

Bill & Melinda Gates Institute for Population and Reproductive Health

Enquête de l’Indice des efforts pour le dividende démographique

Dimension 5. Organisations de la société civile (OSC)

* LM 5.1 Le pouvoir des acteurs des OSC : Mesure dans laquelle les acteurs des OSC sont bien placés pour influencer la politique et l’action nationale concernant le marché du travail.

	1	2	3	4	5	6	7	8	9	10	Je ne sais pas
1-Plus faible -> Plus élevé-10	○	○	○	○	○	○	○	○	○	○	○

* LM 5.2 L’analyse budgétaire comme outil des OSC : Mesure dans laquelle les OSC utilisent l’analyse budgétaire comme un outil pour développer le plaidoyer en matière de marché du travail.

	1	2	3	4	5	6	7	8	9	10	Je ne sais pas
1-Plus faible -> Plus élevé-10	○	○	○	○	○	○	○	○	○	○	○

* LM 5.3 Le soutien des services à base communautaire : Mesure dans laquelle les OSC participent à présenter les besoins, informations et services du marché du travail aux décideurs, et vice-versa.

	1	2	3	4	5	6	7	8	9	10	Je ne sais pas
1-Plus faible -> Plus élevé-10	○	○	○	○	○	○	○	○	○	○	○

* LM 5.4 La diversification des opportunités d’embauche : Mesure dans laquelle les OSC et le secteur privé travaillent en coordination avec le gouvernement pour assurer une création d’emplois optimale et une participation hommes-femmes équitable sur le marché du travail.

	1	2	3	4	5	6	7	8	9	10	Je ne sais pas
1-Plus faible -> Plus élevé-10	○	○	○	○	○	○	○	○	○	○	○

* LM 5.5 Les droits humains et la qualité de la mise en œuvre : Mesure dans laquelle les OSC soutiennent des approches fondées sur le droit sur les lieux de travail.

	1	2	3	4	5	6	7	8	9	10	Je ne sais pas
1-Plus faible -> Plus élevé-10	○	○	○	○	○	○	○	○	○	○	○

* LM 5.6 Les jeunes : Mesure dans laquelle les OSC soutiennent des politiques, interventions et programmes pour l'emploi des jeunes spécifiquement.

	1	2	3	4	5	6	7	8	9	10	Je ne sais pas
1-Plus faible -> Plus élevé-10	○	○	○	○	○	○	○	○	○	○	○

* LM 5.7 Les programmes pour les femmes : Mesure dans laquelle les OSC soutiennent des politiques, services et programmes pour l'emploi des femmes spécifiquement.

	1	2	3	4	5	6	7	8	9	10	Je ne sais pas
1-Plus faible -> Plus élevé-10	○	○	○	○	○	○	○	○	○	○	○

* LM 5.8 Le plaidoyer/ la redevabilité : Mesure dans laquelle les OSC soutiennent la formation et le renforcement de compétences des membres de la communauté pour augmenter leur compréhension des politiques, processus et activités, et pour renforcer leurs capacités à identifier les problèmes liés au marché du travail, résoudre collectivement ces problèmes et mener des actions de plaidoyer plus ciblées pour combattre le chômage.

	1	2	3	4	5	6	7	8	9	10	Je ne sais pas
1-Plus faible -> Plus élevé-10	○	○	○	○	○	○	○	○	○	○	○

* LM 5.9 L'évaluation et le suivi dirigés par les OSC : Mesure dans laquelle les OSC évaluent, suivent et émettent des rapports sur l'efficacité des politiques et programmes pour améliorer la redevabilité au niveau des politiques.

	1	2	3	4	5	6	7	8	9	10	Je ne sais pas
1-Plus faible -> Plus élevé-10	○	○	○	○	○	○	○	○	○	○	○

* LM 5.10 Les partenariats entre OSC : Mesure dans laquelle les OSC ont formé des alliances nationales et des partenariats régionaux pour renforcer le marché du travail.

	1	2	3	4	5	6	7	8	9	10	Je ne sais pas
1-Plus faible -> Plus élevé-10	◯	◯	◯	◯	◯	◯	◯	◯	◯	◯	◯

LM 5.11. Commentaires : Veuillez utiliser cette section pour nous faire part de tout commentaire additionnel sur le niveau d’effort, les défis ou les succès du pays autour des organisations de la société civile dans le secteur du marché du travail. Si vous avez répondu « Je ne sais pas » à l’une des questions précédentes, veuillez utiliser cette section pour expliquer pourquoi.

JOHNS HOPKINS BLOOMBERG SCHOOL of PUBLIC HEALTH

Bill & Melinda Gates Institute for Population and Reproductive Health

Enquête de l'Indice des efforts pour le dividende démographique

Module : Résilience et durabilité du secteur

Étant donné l'impact sanitaire et socioéconomique de la pandémie de COVID-19, et son impact probable sur la progression vers le DD, cet indice des efforts pour le DD intègre des questions pour évaluer la résilience et la durabilité des systèmes dans les secteurs clés du DD. Les éléments couverts par les questions liées à la pandémie de COVID-19 ci-dessous relèvent des dimensions clés des systèmes résilients et visent à apporter des informations déterminantes sur le potentiel d'une réponse efficace aux menaces de maladies infectieuses émergentes et autres crises de santé publique.

Veuillez noter chaque élément sur une échelle de 1 à 10, 1 étant le score le plus faible (faible état/capacité) et 10 le plus élevé (très bon état/capacité).

* Dimension physique : structures, équipements, états du système et capacités.

	1	2	3	4	5	6	7	8	9	10	Je ne sais pas
LM-M1 - Plan/Préparation - État et capacité de l'équipement, du personnel et de la structure du secteur du marché du travail avant la crise. (1 = faible état/capacité ; 10 = très bon état/capacité)	○	○	○	○	○	○	○	○	○	○	○
LM-M2 - Absorption – Mesure dans laquelle le secteur du marché du travail a reconnu la pandémie de COVID-19 et pu continuer à maintenir un niveau d'emploi et une productivité économique optimale.	○	○	○	○	○	○	○	○	○	○	○

	1	2	3	4	5	6	7	8	9	10	Je ne sais pas
LM-M3 - Récupération – Mesure dans laquelle le secteur du marché du travail été capable d'induire des changements pour récupérer un niveau de fonctionnalité pré-COVID-19.	○	○	○	○	○	○	○	○	○	○	○
LM-M4 - Récupération – Gestion du financement et des bailleurs de fonds : Mesure dans laquelle il existe des mécanismes établis de protection sociale (et autres formes de stabilisation des revenus) pour les travailleurs sans protection (ex : les travailleurs indépendants, qui non pas accès au congé payé.)	○	○	○	○	○	○	○	○	○	○	○
LM-M5 - Adaptation – Gestion du financement et des bailleurs de fonds : Mesure dans laquelle des réformes institutionnelles et politiques ont été initiées pour renforcer la résilience par une approche déterminée par la demande à travers des systèmes de protection sociale à grande échelle pouvant agir comme stabilisateurs économiques.	○	○	○	○	○	○	○	○	○	○	○

* Dimension informatique : création, gestion, stockage et utilisation des données.

	1	2	3	4	5	6	7	8	9	10	Je ne sais pas
LM-M6 - Plan/Préparation – Mesure dans laquelle les données liées au marché du travail étaient suffisamment préparées, présentées, analysées et stockées avant la crise.	○	○	○	○	○	○	○	○	○	○	○
LM-M7 - Absorption – Connaissance : Mesure dans laquelle le secteur du marché du travail a pu conduire des évaluations en temps réel de son état de fonctionnement, en anticipation des pertes en cascade de la productivité du marché du travail.	○	○	○	○	○	○	○	○	○	○	○
LM-M8 - Mesure dans laquelle les données du marché du travail ont été utilisées pour suivre les progrès vers la récupération et anticiper des scenarii de récupération.	○	○	○	○	○	○	○	○	○	○	○
LM-M9 - Adaptation – Mesure dans laquelle le secteur du marché du travail crée et améliore ses protocoles de stockage et utilisation des données en temps réel.	○	○	○	○	○	○	○	○	○	○	○

* Dimension cognitive : compréhension, modèles cognitifs, idées reçues, préjugés et valeurs.

	1	2	3	4	5	6	7	8	9	10	Je ne sais pas
LM-M10 - Planification/Préparation – Mesure dans laquelle le système de conception et les décisions opérationnelles du secteur du marché du travail étaient préparés à anticiper la crise.	○	○	○	○	○	○	○	○	○	○	○

	1	2	3	4	5	6	7	8	9	10	Je ne sais pas
LM-M11 - Absorption – Mesure dans laquelle le secteur du marché du travail a des protocoles de contingence suffisants et une gestion de crise proactive.	○	○	○	○	○	○	○	○	○	○	○
LM-M12 - Absorption - Leadership et management : Mesure dans laquelle le gouvernement garantit aux salariés/ travailleurs et leurs famille une protection contre les risques sanitaires de la pandémie de COVID-19.	○	○	○	○	○	○	○	○	○	○	○
LM-M13 - Absorption – Redevabilité : Niveau d'effort pour assurer une redevabilité optimale vis-à-vis des ressources allouées à la protection contre les risques sanitaires de la pandémie de COVID-19 et la récupération du marché du travail dans le cadre de la réponse pour mitiger les impacts du COVID-19.	○	○	○	○	○	○	○	○	○	○	○
LM-M14 - Récupération – Mesure dans laquelle les décisions sont orientées vers la récupération et communiquées aux membres de la communauté sur la base de données probantes pour promouvoir des comportements sûrs.	○	○	○	○	○	○	○	○	○	○	○

	1	2	3	4	5	6	7	8	9	10	Je ne sais pas
LM-M15 - Récupération – Connaissance/Conscience : Mesure dans laquelle le secteur établit une certaine confiance et un dialogue entre les employeurs et les salariés pour renforcer l'engagement d'action commune du gouvernement et promouvoir le dialogue social au niveau des entreprises.	◯	◯	◯	◯	◯	◯	◯	◯	◯	◯	◯
LM-M16 - Adaptation – Mesure dans laquelle le secteur du marché du travail conçoit de nouvelles configurations systémiques, objectifs et critères de décision.	◯	◯	◯	◯	◯	◯	◯	◯	◯	◯	◯
LM-M17 - Adaptation – Adaptatif : Mesure dans laquelle le gouvernement promeut des changements sur les lieux de travail pour protéger les travailleurs (ex : mesures de sécurité et gestes barrières, prévention de la discrimination et de l'exclusion, accès élargi au congé payé, etc.).	◯	◯	◯	◯	◯	◯	◯	◯	◯	◯	◯

* Dimension sociale : interaction, collaboration et auto-synchronisation entre les personnes, les entités et les institutions.

	1	2	3	4	5	6	7	8	9	10	Je ne sais pas
LM-M18 - Planification /Préparation – Mesure dans laquelle une formation sur la gestion d'épidémies/crises a été menée et a permis de mettre à profit les réseaux sociaux, le capital social et les normes institutionnelles et culturelles avant la crise.	◯	◯	◯	◯	◯	◯	◯	◯	◯	◯	◯

	1	2	3	4	5	6	7	8	9	10	Je ne sais pas
LM-M19 - Absorption – Mesure dans laquelle le personnel et les institutions sociales ont été accessibles et ont fait preuve d'ingéniosité dans leur réponse à l'épidémie/crise.	○	○	○	○	○	○	○	○	○	○	○
LM-M20 - Absorption – Autorégulation : Mesure dans laquelle les dirigeants nationaux avaient l'autorité d'effectuer des changements sectoriels en temps opportun à travers une infrastructure du marché du travail flexible.	○	○	○	○	○	○	○	○	○	○	○
LM-M21 - Récupération – Mesure dans laquelle le secteur du marché du travail est impliqué dans le partage de connaissances et d'équipes pour améliorer la récupération du système.	○	○	○	○	○	○	○	○	○	○	○
LM-M22 - Récupération – Diversité : Mesure dans laquelle le gouvernement facilite une récupération solide et rapide du marché du travail en diversifiant les politiques favorisant à la fois l'offre (production de biens et services) et la demande (consommation et investissement).	○	○	○	○	○	○	○	○	○	○	○
LM-M23 - Adaptation – Mesure dans laquelle des ajouts ou modifications sont apportés aux institutions, politiques, formations, programmes et à la culture du secteur du marché du travail.	○	○	○	○	○	○	○	○	○	○	○

	1	2	3	4	5	6	7	8	9	10	Je ne sais pas
LM-M24 - Adaptation - Leadership et management : Mesure dans laquelle il y a eu un dialogue tripartite fluide entre le gouvernement, les organisations des travailleurs/salariés et celles des employeurs.	○	○	○	○	○	○	○	○	○	○	○

* LM M-25 Temporalité : Mesure de la rapidité de la réaction du gouvernement –sa communication et mise en œuvre des mesures- pour mitiger l’impact immédiat et sur le long terme de la pandémie de COVID-19. Cette question porte sur la riposte nationale dans son ensemble, et non la réponse spécifique de votre secteur.

	1	2	3	4	5	6	7	8	9	10	Je ne sais pas
1-Plus faible -> Plus élevé-10	○	○	○	○	○	○	○	○	○	○	○

LM M-26 Commentaires : Utilisez cette section pour apporter tout commentaire additionnel sur la résilience et la durabilité des systèmes du marché du travail en vous référant à votre expérience de la pandémie de COVID-19. Si vous avez répondu « Je ne sais pas » à l’une des questions, utilisez cette section pour expliquer pourquoi.

JOHNS HOPKINS
BLOOMBERG SCHOOL of PUBLIC HEALTH

Bill & Melinda Gates Institute for Population and Reproductive Health

Enquête de l'Indice des efforts pour le dividende démographique

Questionnaire sur la gouvernance et les institutions économiques (GIE)

Répondez aux questions suivantes en fonction de votre expérience/ expertise dans ce secteur spécifique.

Afin d'obtenir une représentation synthétique des efforts nationaux pour le dividende démographique, notez les éléments suivants sur une échelle de 1 à 10, 1 étant le score le plus faible (efforts très faibles ou quasi-inexistants) et 10 le plus élevé (efforts robustes). Le cas échéant, si une politique ou activité n'existe pas, répondez 0.

Donnez un score à chaque élément. Toutes les réponses seront enregistrées au format illustré ci-dessous :

Effort le plus faible	1	2	3	4	5	6	7	8	9	10	Effort le plus élevé	Je ne sais pas

JOHNS HOPKINS
BLOOMBERG SCHOOL
of PUBLIC HEALTH

Bill & Melinda Gates Institute for Population and Reproductive Health

Enquête de l'Indice des efforts pour le dividende démographique

Domaine 1. Politique/ Prise de décision

* GEI 1.1 Les valeurs démocratiques : Mesure dans laquelle le gouvernement a ratifié, appliqué à son contexte national et mis en œuvre les instruments régionaux sur les valeurs communes, y compris ceux liés à la démocratie, les élections, la gouvernance et le droit à la santé.

	1	2	3	4	5	6	7	8	9	10	Je ne sais pas
1-Plus faible -> Plus élevé-10	○	○	○	○	○	○	○	○	○	○	○

* GEI 1.2 Le suivi des valeurs démocratiques : Mesure dans laquelle le gouvernement a créé des mécanismes de rapport régulier et périodique sur la mise en œuvre des instruments régionaux de valeurs communes à travers les mécanismes existants tels que les revues périodiques universelles du statut des instruments internationaux ou régionaux, ou à travers des entités indépendantes. (Cela peut prendre la forme de rapports réguliers sur le statut de la mise en œuvre).

	1	2	3	4	5	6	7	8	9	10	Je ne sais pas
1-Plus faible -> Plus élevé-10	○	○	○	○	○	○	○	○	○	○	○

* GEI 1.3 L'affirmation de la participation démocratique des jeunes : Mesure dans laquelle le gouvernement a explicitement abrogé toutes les lois et contraintes discriminatoires empêchant la pleine participation des jeunes au processus électoral à travers des lois électorales et constitutions inclusives. (Cela peut inclure le développement et/ou renforcement des programmes de leadership des jeunes et/ou le renforcement de compétences en management et leadership).

	1	2	3	4	5	6	7	8	9	10	Je ne sais pas
1-Plus faible -> Plus élevé-10	○	○	○	○	○	○	○	○	○	○	○

* GEI 1.4. La suppression des barrières légales auxquelles se heurtent les jeunes : Mesure dans laquelle le gouvernement a proscrit toutes les lois posant des barrières au plein exercice et à la pleine jouissance des droits fondamentaux des jeunes à participer pleinement aux processus de gouvernance démocratique au niveau continental, régional, national et communautaire/à la base. (Cela pourrait aussi inclure le soutien de l'intégration des questions de droits et de santé des jeunes et adolescents à l'agenda du gouvernement sur la croissance et la transformation du pays).

	1	2	3	4	5	6	7	8	9	10	Je ne sais pas
1-Plus faible -> Plus élevé-10	○	○	○	○	○	○	○	○	○	○	○

* GEI 1.5 La politique de la jeunesse : Mesure dans laquelle la politique de la jeunesse du pays reflète les paradigmes éducatifs, de formation et d'emploi les plus récents.

	1	2	3	4	5	6	7	8	9	10	Je ne sais pas
1-Plus faible -> Plus élevé-10	○	○	○	○	○	○	○	○	○	○	○

* GEI 1.6 L'affirmation de la participation à la vie démocratique/ révision de la loi coutumière : Mesure dans laquelle le gouvernement a examiné, révisé, amendé ou aboli toutes les lois, régulations, politiques, pratiques et coutumes ayant un effet discriminatoire sur les jeunes, particulièrement les filles et les jeunes filles, sans distinction aucune, s'est assuré que les dispositions des différents systèmes légaux soient conformes aux droits humains internationaux. (Cela doit impérativement inclure la protection contre les pratiques malfaisantes, telles que le mariage précoce ou forcé, la violence sexuelle et basée sur le genre [VBG], et la mutilation génitale féminine [MGF]).

	1	2	3	4	5	6	7	8	9	10	Je ne sais pas
1-Plus faible -> Plus élevé-10	○	○	○	○	○	○	○	○	○	○	○

* GEI 1.7 La participation des jeunes dans la prise de décision politique : Mesure dans laquelle le gouvernement veille à l'implication des jeunes leaders et à leur participation dans les processus de prise de décision politique, y compris la politique sur la jeunesse et l'éducation. (Cela peut inclure les efforts visant à établir un parlement ou une assemblée nationale de jeunes. Cela peut aussi inclure le soutien à la participation et au leadership des jeunes et adolescents dans la planification et les programmes de santé à tous les niveaux de gouvernance).

	1	2	3	4	5	6	7	8	9	10	Je ne sais pas
1-Plus faible -> Plus élevé-10	○	○	○	○	○	○	○	○	○	○	○

* GEI 1.8 L'État de droit : Mesure dans laquelle l'État de droit est formellement et légalement établi de manière équitable et mis en œuvre indépendamment par le pouvoir judiciaire.

	1	2	3	4	5	6	7	8	9	10	Je ne sais pas
1-Plus faible -> Plus élevé-10											

* GEI 1.9 La transparence et la redevabilité : Mesure dans laquelle la législation nationale promeut la transparence et la redevabilité de toutes les branches et secteurs.

	1	2	3	4	5	6	7	8	9	10	Je ne sais pas
GEI 1.9.1 Mesure dans laquelle le public peut accéder aux informations législatives et aux registres sur les organisations publiques.											
GEI 1.9.2 Mesure dans laquelle il existe des mécanismes de redevabilité rendant cette dernière obligatoire et des sanctions associées.											

* GEI 1.10 La sécurité nationale : Mesure dans laquelle le pays/gouvernement est impliqué dans un conflit armé, qu'il soit international ou intérieur.

	1	2	3	4	5	6	7	8	9	10	Je ne sais pas
GEI 1.10.1 Mesure dans laquelle le pays souffre de tensions transfrontalières.											
GEI 1.10.2 Mesure dans laquelle le pays contient des personnes internement déplacées et/ou des réfugiés.											
GEI 1.10.3 Mesure dans laquelle les établissements éducatifs sont protégés et des opportunités d'apprentissage alternatives créées pour les enfants et les jeunes déplacées ou vivant en situation de conflit.											

* GEI 1.11 Les droits : Mesure dans laquelle la constitution affirme certains droits : liberté d'expression, liberté d'association et d'assemblée, droits et libertés civiques, et protection contre la discrimination ethnique et religieuse ; et mesure dans laquelle la violation des droits humains par le gouvernement est improbable.

	1	2	3	4	5	6	7	8	9	10	Je ne sais pas
1-Plus faible -> Plus élevé-10	◯	◯	◯	◯	◯	◯	◯	◯	◯	◯	◯

* GEI 1.12 L'éducation et les valeurs démocratiques : Mesure dans laquelle les politiques, stratégies et programmes d'éducation promeuvent la liberté de penser, les valeurs démocratiques et l'éducation comme un droit fondamental/humain.

	1	2	3	4	5	6	7	8	9	10	Je ne sais pas
1-Plus faible -> Plus élevé-10	◯	◯	◯	◯	◯	◯	◯	◯	◯	◯	◯

* GEI 1.13 L'affirmation des environnements d'apprentissage sûrs : Mesure dans laquelle les politiques d'éducation garantissent des environnements d'apprentissage sûrs à travers la protection de l'enfance. (Cela peut se manifester par la protection de la sécurité des filles, l'interdiction des punitions corporelles, etc.).

	1	2	3	4	5	6	7	8	9	10	Je ne sais pas
1-Plus faible -> Plus élevé-10	◯	◯	◯	◯	◯	◯	◯	◯	◯	◯	◯

GEI 1.14 Commentaires : Utilisez cette section pour nous faire part de tout commentaire additionnel sur le niveau d'effort, les défis ou les succès du pays autour des politiques/ de la prise de décision dans le secteur de la gouvernance et des institutions économiques. Si vous avez répondu « Je ne sais pas » à l'une des questions précédentes, veuillez utiliser cette section pour expliquer pourquoi.

Bill & Melinda Gates Institute for Population and Reproductive Health

Enquête de l’Indice des efforts pour le dividende démographique

Domaine 2. Services ou programmes

* GEI 2.1 Renforcer les activités/ institutions pour la jeunesse : Mesure dans laquelle des efforts sont menés pour renforcer les formations, réseaux et organisations indépendantes de jeunes, y compris l’établissement de commissions indépendantes de jeunes et de syndicats d’étudiants au niveau national et infranational pour promouvoir les droits des jeunes.

	1	2	3	4	5	6	7	8	9	10	Je ne sais pas
1-Plus faible -> Plus élevé-10	○	○	○	○	○	○	○	○	○	○	○

* GEI 2.2 La nomination de jeunes leaders : Si et mesure dans laquelle un jeune représentant a été nommé pour diriger le plaidoyer et promouvoir la priorisation des questions concernant les jeunes (ex : éducation, formation et emploi) dans les espaces de prise de décision à différents niveaux, y compris au parlement.

	1	2	3	4	5	6	7	8	9	10	Je ne sais pas
1-Plus faible -> Plus élevé-10	○	○	○	○	○	○	○	○	○	○	○

* GEI 2.3 Les idéaux et aspirations des jeunes : Mesure dans laquelle les programmes sont conçus pour instituer le leadership des jeunes, et mesure dans laquelle les formations pour l’autonomisation des jeunes visent à approfondir les idéaux et aspirations culturelles des jeunes.

	1	2	3	4	5	6	7	8	9	10	Je ne sais pas
1-Plus faible -> Plus élevé-10	○	○	○	○	○	○	○	○	○	○	○

* GEI 2.4 Les services des jeunes : Mesure dans laquelle les programmes sont conçus pour répondre aux besoins spécifiques des jeunes. (Cela devrait prendre en compte le développement et la mise en œuvre de services de santé amis des jeunes et adolescents, entre autres programmes similaires).

	1	2	3	4	5	6	7	8	9	10	Je ne sais pas
1-Plus faible -> Plus élevé-10	○	○	○	○	○	○	○	○	○	○	○

* GEI 2.5 L'implication des jeunes dans les initiatives nationales : Mesure dans laquelle des efforts sont menés pour renforcer les initiatives existantes du pays en matière d'engagement et de participation des jeunes (e*x : au niveau de l'Union africaine, cela peut inclure le Corps des Jeunes Volontaires de l'Union Africaine, les Clubs de Jeunes de l'Union Africaine, et les activités de AGA-YES, dont le comité consultatif de jeunes, les procès fictifs et le dialogue annuel des jeunes sur la démocratie, les droits de l'Homme et la gouvernance).* (Cela peut inclure l'implication des jeunes et des adolescents dans l'assistance technique apportée aux ministères et entités gouvernementales fédérales ou régionales compétentes pour l'élaboration de politiques, plans et programmes qui intègrent les questions de santé prioritaires affectant ce groupe).

	1	2	3	4	5	6	7	8	9	10	Je ne sais pas
1-Plus faible -> Plus élevé-10	○	○	○	○	○	○	○	○	○	○	○

* GEI 2.6 La sécurité : Mesure dans laquelle la sécurité personnelle est garantie à travers des services/ activités de police fiables, contribuant à une perception générale positive de la sécurité des personnes dans les ménages, les communautés et en milieu scolaire. (Cela pourrait inclure la protection des jeunes contre tout type d'abus, négligence ou manque de respect dans l'utilisation des services de santé).

	1	2	3	4	5	6	7	8	9	10	Je ne sais pas
1-Plus faible -> Plus élevé-10	○	○	○	○	○	○	○	○	○	○	○

* GEI 2.7 La participation : Mesure dans laquelle les fonctions du gouvernement impliquent la participation des citoyens, des organisations de la société civile et autres acteurs intersectoriels pour promouvoir des élections démocratiques et le pouvoir effectif de gouverner.

	1	2	3	4	5	6	7	8	9	10	Je ne sais pas
1-Plus faible -> Plus élevé-10	○	○	○	○	○	○	○	○	○	○	○

* GEI 2.8 Le genre : Mesure dans laquelle les femmes participent à :

	1	2	3	4	5	6	7	8	9	10	Je ne sais pas
GEI 2.8.1 La gouvernance au niveau national	◯	◯	◯	◯	◯	◯	◯	◯	◯	◯	◯
GEI 2.8.2 La gouvernance au niveau infranational	◯	◯	◯	◯	◯	◯	◯	◯	◯	◯	◯
GEI 2.8.3 La gouvernance en milieu rural	◯	◯	◯	◯	◯	◯	◯	◯	◯	◯	◯
GEI 2.8.4 La gouvernance en milieu urbain	◯	◯	◯	◯	◯	◯	◯	◯	◯	◯	◯
GEI 2.8.5 Judiciaire	◯	◯	◯	◯	◯	◯	◯	◯	◯	◯	◯

* GEI 2.9 La promotion de l’égalité : Mesure dans laquelle la parité est promue par le gouvernement dans tous les aspects de la société. (Ceci peut inclure la parité et l’égalité dans l’éducation, devant la loi, dans le système de santé, à l’embauche et en termes de rémunération, entre autres facettes de la société).

	1	2	3	4	5	6	7	8	9	10	Je ne sais pas
1-Plus faible -> Plus élevé-10	◯	◯	◯	◯	◯	◯	◯	◯	◯	◯	◯

* GEI 2.10 La durabilité des opportunités économiques :

	1	2	3	4	5	6	7	8	9	10	Je ne sais pas
GEI 2.10.1 Mesure dans laquelle le gouvernement pourvoit et s’assure qu’un système de gestion publique solide soit réalisé.	○	○	○	○	○	○	○	○	○	○	○
GEI 2.10.2 Mesure dans laquelle le gouvernement pourvoit et s’assure qu’un environnement commercial solide soit réalisé.	○	○	○	○	○	○	○	○	○	○	○
GEI 2.10.3 Mesure dans laquelle le gouvernement pourvoit et s’assure qu’une infrastructure publique solide soit réalisée.	○	○	○	○	○	○	○	○	○	○	○
GEI 2.10.4 Mesure dans laquelle le gouvernement pourvoit et s’assure qu’un secteur rural fort pour le développement soit réalisé.	○	○	○	○	○	○	○	○	○	○	○

* GEI 2.11 Le développement humain :

	1	2	3	4	5	6	7	8	9	10	Je ne sais pas
GEI 2.11.1 Mesure dans laquelle le bien-être du public en général est adéquatement géré par le gouvernement (y compris, mais sans s'y limiter, les politiques et services de sécurité sociale, l'intégration socioéconomique et la protection sociale des jeunes, entre autres sujets). Cela peut aussi impliquer la mesure dans laquelle le gouvernement, ayant l'obligation de protéger, remplir et faire respecter les droits fondamentaux de santé, a donné la responsabilité de leur mise en œuvre à son cabinet ministériel.	○	○	○	○	○	○	○	○	○	○	○
GEI 2.11.2 Mesure dans laquelle le gouvernement promeut le développement de compétences de la population sur les lieux de travail.	○	○	○	○	○	○	○	○	○	○	○
GEI 2.11.3 Mesure dans laquelle le gouvernement promeut l'éducation, le développement de connaissances et des capacités de la population à travers l'école et des opportunités de formation professionnelle continue.	○	○	○	○	○	○	○	○	○	○	○
GEI 2.11.4 Mesure dans laquelle le gouvernement promeut la santé et le bien-être de la population.	○	○	○	○	○	○	○	○	○	○	○

GEI 2.12 Commentaires : Utilisez cette section pour nous faire part de tout commentaire additionnel sur le niveau d’effort, les défis ou les succès du pays autour des services ou programmes dans le secteur de la gouvernance et des institutions économiques. Si vous avez répondu « Je ne sais pas » à l’une des questions précédentes, veuillez utiliser cette section pour expliquer pourquoi.

JOHNS HOPKINS
BLOOMBERG SCHOOL of PUBLIC HEALTH

Bill & Melinda Gates Institute for Population and Reproductive Health

Enquête de l'Indice des efforts pour le dividende démographique

Domaine 3. Plaidoyer

* GEI 3.1 L'autonomisation des jeunes : Mesure dans laquelle l'autonomisation des jeunes a été améliorée, potentiellement à travers l'intégration de l'éducation civique au programme d'éducation scolaire nationale, aux plateformes médiatiques et autres canaux pour inculquer les principes de l'État de droit, les droits humains, et les devoirs et responsabilités individuelles. (Cela peut comprendre l'inclusion de la santé des jeunes et des adolescents et des questions relatives au droit dans l'agenda de croissance et transformation du pays et dans les programmes scolaires).

	1	2	3	4	5	6	7	8	9	10	Je ne sais pas
1-Plus faible -> Plus élevé-10	○	○	○	○	○	○	○	○	○	○	○

* GEI 3.2 Le plaidoyer du gouvernement : Mesure dans laque le gouvernement plaide pour des améliorations au sein de ses propres structures et priorités. (Ces améliorations peuvent comprendre des avancées vers l'inclusion, les opportunités, les programmes et les droits des jeunes, ou autres concepts similaires dans différentes populations.).

	1	2	3	4	5	6	7	8	9	10	Je ne sais pas
1-Plus faible -> Plus élevé-10	○	○	○	○	○	○	○	○	○	○	○

* GEI 3.3 Le plaidoyer non-gouvernemental : Mesure dans laquelle les acteurs du gouvernement plaident pour des améliorations des structures et priorités du gouvernement. (Ces améliorations peuvent comprendre des avancées vers l'inclusion les opportunités, les programmes et les droits des jeunes, ou autres concepts similaires dans différentes populations.).

	1	2	3	4	5	6	7	8	9	10	Je ne sais pas
1-Plus faible -> Plus élevé-10	○	○	○	○	○	○	○	○	○	○	○

* GEI 3.4 La voix du plaidoyer : Mesure dans laquelle les plaideurs se font entendre dans les discussions gouvernementales.

	1	2	3	4	5	6	7	8	9	10	Je ne sais pas
1-Plus faible -> Plus élevé-10	○	○	○	○	○	○	○	○	○	○	○

* GEI 3.5 La voix des secteurs clés du DD : Mesure dans laquelle le gouvernement et les parties prenantes sont engagés à établir des institutions durables pour assurer :

	1	2	3	4	5	6	7	8	9	10	Je ne sais pas
GEI 3.5.1 Bonne gouvernance	○	○	○	○	○	○	○	○	○	○	○
GEI 3.5.2 Stabilité financière	○	○	○	○	○	○	○	○	○	○	○
GEI 3.5.3 Programmes optimaux de planification familiale	○	○	○	○	○	○	○	○	○	○	○
GEI 3.5.4 Programmes optimaux de santé maternelle et infantile	○	○	○	○	○	○	○	○	○	○	○
GEI 3.5.5 Éducation de bonne qualité	○	○	○	○	○	○	○	○	○	○	○
GEI 3.5.6 Marché du travail diversifié	○	○	○	○	○	○	○	○	○	○	○

GEI 3.6 Commentaires : Utilisez cette section pour nous faire part de tout commentaire additionnel sur le niveau d’effort, les défis ou les succès du pays autour du plaidoyer dans le secteur de la gouvernance et des institutions économiques. Si vous avez répondu « Je ne sais pas » à l’une des questions précédentes, veuillez utiliser cette section pour expliquer pourquoi.

JOHNS HOPKINS
BLOOMBERG SCHOOL
of PUBLIC HEALTH

Bill & Melinda Gates Institute for Population and Reproductive Health

Enquête de l'Indice des efforts pour le dividende démographique

Domaine 4. Recherche

* GEI 4.1 La force des observatoires/parties prenantes/groupes de travail sur la gouvernance et les institutions économiques (GIE) : Mesure dans laquelle les parties prenantes de la gouvernance et des institutions économiques du pays (ex : groupe directif de parties prenantes, groupes de travail techniques, observatoire de la gouvernance, groupe de coordination ou facilitation) :

	1	2	3	4	5	6	7	8	9	10	Je ne sais pas
GEI 4.1.1 Ont un représentant du gouvernement, des institutions de formation, de la société civile, des organisations non gouvernementales et confessionnelles, des associations professionnelles, du secteur privé, etc.	◯	◯	◯	◯	◯	◯	◯	◯	◯	◯	◯
GEI 4.1.2 Se réunissent régulièrement, émettent des rapports et recommandent des politiques aux directions des ministères compétents.	◯	◯	◯	◯	◯	◯	◯	◯	◯	◯	◯
GEI 4.1.3 Ont un impact sur la PF dans le pays.	◯	◯	◯	◯	◯	◯	◯	◯	◯	◯	◯

* GEI 4.2 La stratégie de recherche sur la gouvernance : Mesure dans laquelle les documents de planification nationale comprennent une stratégie/approche complète de recherche liée à la GIE.

	1	2	3	4	5	6	7	8	9	10	Je ne sais pas
1-Plus faible -> Plus élevé-10	◯	◯	◯	◯	◯	◯	◯	◯	◯	◯	◯

* GEI 4.3 Les partenaires collectant des données sur la gouvernance, la finance et le développement :

	1	2	3	4	5	6	7	8	9	10	Je ne sais pas
GEI 4.3.1 Mesure dans laquelle la collecte des données est entreprise par des agences gouvernementales. (Cela peut comprendre les agences/bureaux de la statistique et/ou les ministères de l'économie/finances, éducation, santé, genre, etc.).	○	○	○	○	○	○	○	○	○	○	○
GEI 4.3.2 Mesure dans laquelle la collecte de données est entreprise par des institutions de recherche.	○	○	○	○	○	○	○	○	○	○	○
GEI 4.3.3 Mesure dans laquelle la collecte des données est entreprise par des chercheurs indépendants.	○	○	○	○	○	○	○	○	○	○	○

* GEI 4.4 La recherche thématique :

	1	2	3	4	5	6	7	8	9	10	Je ne sais pas
GEI 4.4.1 Mesure dans laquelle de la recherche est menée sur la sécurité et l'État de droit.	○	○	○	○	○	○	○	○	○	○	○
GEI 4.4.2 Mesure dans laquelle la participation politique et les droits humains sont mesurés.	○	○	○	○	○	○	○	○	○	○	○
GEI 4.4.3 Mesure dans laquelle de la recherche est menée sur les indicateurs de l'économie durable et est disséminée à la communauté.	○	○	○	○	○	○	○	○	○	○	○
GEI 4.4.4 Mesure dans laquelle de la recherche est menée sur le développement du capital humain (éducation, formation et emploi, etc.).	○	○	○	○	○	○	○	○	○	○	○

* GEI 4.5 La qualité/couverture des données :

	1	2	3	4	5	6	7	8	9	10	Je ne sais pas
GEI 4.5.1 Mesure dans laquelle le système statistique de routine fournit des informations périodiques sur la GIE.	○	○	○	○	○	○	○	○	○	○	○
GEI 4.5.2 Mesure dans laquelle le système statistique de routine fournit des informations périodiques fiables sur les besoins des populations de différentes communautés en matière de gouvernance.	○	○	○	○	○	○	○	○	○	○	○
GEI 4.5.3 Mesure dans laquelle des évaluations des systèmes de suivi et surveillance sont conduites et appliquées pour assurer la fiabilité des données.	○	○	○	○	○	○	○	○	○	○	○

* GEI 4.6 La qualité des institutions de recherche : Mesure dans laquelle le pays a la capacité de soutenir et maintenir des institutions de recherche qui développent des études et/ou collectent des données permettant de soutenir la bonne gouvernance, la stabilité de l'économie et la solidité des institutions publiques.

	1	2	3	4	5	6	7	8	9	10	Je ne sais pas
1-Plus faible -> Plus élevé-10	○	○	○	○	○	○	○	○	○	○	○

* GEI 4.7 L'évaluation : Mesure dans laquelle les statistiques, enquêtes et études sont utilisées par du personnel spécialisé pour générer des rapports sur la bonne gouvernance et les institutions financières

	1	2	3	4	5	6	7	8	9	10	Je ne sais pas
1-Plus faible -> Plus élevé-10	○	○	○	○	○	○	○	○	○	○	○

* GEI 4.8 L'utilisation des résultats d'évaluation par les gestionnaires de programmes : Mesure dans laquelle les gestionnaires locaux de programmes utilisent les conclusions de recherche et d'évaluations pour améliorer l'État de droit, stabiliser l'économie et renforcer les institutions en suivant les recommandations émises.

	1	2	3	4	5	6	7	8	9	10	Je ne sais pas
1-Plus faible -> Plus élevé-10	○	○	○	○	○	○	○	○	○	○	○

* GEI 4.10 La dissémination d'informations à d'autres entités chargées de la mise en œuvre des programmes : Mesure dans laquelle des informations sont partagées ou disséminées entre géographies et à différents niveaux (national, état/province/comté, localités/sous-comtés).

	1	2	3	4	5	6	7	8	9	10	Je ne sais pas
1-Plus faible -> Plus élevé-10	◯	◯	◯	◯	◯	◯	◯	◯	◯	◯	◯

GEI 4.11 Commentaires : Utilisez cette section pour nous faire part de tout commentaire additionnel sur le niveau d'effort, les défis ou les succès du pays autour de la recherche sur la gouvernance et les institutions économiques. Si vous avez répondu « Je ne sais pas » à l'une des questions précédentes, veuillez utiliser cette section pour expliquer pourquoi.

JOHNS HOPKINS
BLOOMBERG SCHOOL of PUBLIC HEALTH

Bill & Melinda Gates Institute for Population and Reproductive Health

Enquête de l'Indice des efforts pour le dividende démographique

Domaine 5. Organisations de la société civile (OSC)

* GEI 5.1 Le pouvoir des acteurs des OSC : Mesure dans laquelle les acteurs des OSC occupent des postes d'influence pour contribuer positivement à l'État de droit.

	1	2	3	4	5	6	7	8	9	10	Je ne sais pas
1-Plus faible -> Plus élevé-10	○	○	○	○	○	○	○	○	○	○	○

* GEI 5.2 L'analyse budgétaire comme outil des OSC : Mesure dans laquelle les OSC utilisent l'analyse budgétaire comme un outil pour développer le plaidoyer pour la bonne gouvernance, la stabilité de l'économie et la consolidation des institutions publiques.

	1	2	3	4	5	6	7	8	9	10	Je ne sais pas
1-Plus faible -> Plus élevé-10	○	○	○	○	○	○	○	○	○	○	○

* GEI 5.3 Le soutien aux services à base communautaire : Mesure dans laquelle les OSC participent au rapprochement des informations et services liés à la gouvernance avec les communautés.

	1	2	3	4	5	6	7	8	9	10	Je ne sais pas
1-Plus faible -> Plus élevé-10	○	○	○	○	○	○	○	○	○	○	○

* GEI 5.4 Les droits humains et la qualité de la mise en œuvre : Mesure dans laquelle les OSC plaident et soutiennent des approches de gouvernance fondées sur le droit pour veiller à ce que les bénéficiaires de projets et programmes puissent voir des avancées selon leurs propres termes.

	1	2	3	4	5	6	7	8	9	10	Je ne sais pas
1-Plus faible -> Plus élevé-10	○	○	○	○	○	○	○	○	○	○	○

* GEI 5.5 Les jeunes : Mesure dans laquelle les OSC aident les jeunes à participer à l’éducation, la formation, l’emploi et le leadership.

	1	2	3	4	5	6	7	8	9	10	Je ne sais pas
1-Plus faible -> Plus élevé-10	○	○	○	○	○	○	○	○	○	○	○

* GEI 5.6 Les programmes pour les femmes : Mesure dans laquelle les OSC veillent à ce que les politiques de GIE soutiennent la participation des femmes et des filles au leadership.

	1	2	3	4	5	6	7	8	9	10	Je ne sais pas
1-Plus faible -> Plus élevé-10	○	○	○	○	○	○	○	○	○	○	○

* GEI 5.7 Le plaidoyer/ redevabilité :

	1	2	3	4	5	6	7	8	9	10	Je ne sais pas
GEI 5.7.1 Mesure dans laquelle les OSC et les médias soutiennent la participation de la communauté dans la gouvernance et l’application de l’État de droit.	○	○	○	○	○	○	○	○	○	○	○
GEI 5.7.2 Mesure dans laquelle les médias soutiennent la participation de la communauté à la gouvernance ou au respect de l'État de droit.	○	○	○	○	○	○	○	○	○	○	○

* GEI 5.8 Les évaluations et le suivi menés par les OSC : Mesure dans laquelle les OSC évaluent, suivent et émettent des rapports sur l’efficacité des politiques et des mécanismes et structures de redevabilité du gouvernement.

	1	2	3	4	5	6	7	8	9	10	Je ne sais pas
1-Plus faible -> Plus élevé-10	○	○	○	○	○	○	○	○	○	○	○

* GEI 5.9 Les partenariats entre OSC : Mesure dans laquelle les OSC ont formé des alliances nationales et des partenariats régionaux pour renforcer leur position et potentiellement améliorer le leadership et les mécanismes de financement pour une bonne gouvernance et le renforcement institutionnel.

	1	2	3	4	5	6	7	8	9	10	Je ne sais pas
1-Plus faible -> Plus élevé-10	◯	◯	◯	◯	◯	◯	◯	◯	◯	◯	◯

GEI 5.10 Commentaires : Utilisez cette section pour nous faire part de tout commentaire additionnel sur le niveau d'effort, les défis ou les succès du pays autour des OSC dans le secteur de la gouvernance et des institutions économiques. Si vous avez répondu « Je ne sais pas » à l'une des questions précédentes, veuillez utiliser cette section pour expliquer pourquoi.

Bill & Melinda Gates Institute for Population and Reproductive Health

Enquête de l'Indice des efforts pour le dividende démographique

Module : Résilience et durabilité du secteur

Étant donné l'impact sanitaire et socioéconomique de la pandémie de COVID-19, et son impact probable sur la progression vers le DD, cet indice des efforts pour le DD intègre des questions pour évaluer la résilience et la durabilité des systèmes dans les secteurs clés du DD. Les éléments couverts par les questions liées à la pandémie de COVID-19 ci-dessous relèvent des dimensions clés des systèmes résilients et visent à apporter des informations déterminantes sur le potentiel d'une réponse efficace aux menaces de maladies infectieuses émergeantes et autres crises de santé publique.

Veuillez noter chaque élément sur une échelle de 1 à 10, 1 étant le score le plus faible (faible état/capacité) et 10 le plus élevé (très bon état/capacité).

* Dimension physique : structures, équipements, états du système et capacités.

	1	2	3	4	5	6	7	8	9	10	Je ne sais pas
GEI-M1 – Planification/Préparation : État et capacité de l'équipement, du personnel et de la structure du secteur GIE avant la crise. (1 = faible état/capacité ; 10 = très bon état/capacité)	○	○	○	○	○	○	○	○	○	○	○
GEI-M2 - Absorption - Mesure dans laquelle le système du secteur GIE a reconnu la pandémie de COVID-19 et pu maintenir sa fonctionnalité.	○	○	○	○	○	○	○	○	○	○	○

	1	2	3	4	5	6	7	8	9	10	Je ne sais pas
GEI-M3 - Absorption – Intégration : Niveau d’intégration des services et collaborations institutionnelles pour assurer la continuité de l’accès aux services essentiels pendant la crise COVID-19.	○	○	○	○	○	○	○	○	○	○	○
GEI-M4 - Récupération – Mesure dans laquelle les institutions économiques et gouvernementales ont été capables de générer des changements pour récupérer un niveau de fonctionnalité pré-COVID-19.	○	○	○	○	○	○	○	○	○	○	○
GEI-M5 - Récupération – Gestion du financement et des bailleurs de fonds : Banque nationale et soutien des bailleurs de fonds pour assurer la disponibilité de liquidités pendant la crise COVID-19.	○	○	○	○	○	○	○	○	○	○	○
GEI-M6 - Adaptation – Mesure dans laquelle des changements ont été apportés pour améliorer la résilience du système et de l’infrastructure du secteur GIE.	○	○	○	○	○	○	○	○	○	○	○

* Dimension informatique : création, gestion, stockage et utilisation des données.

	1	2	3	4	5	6	7	8	9	10	Je ne sais pas
GEI-M7 - Plan/Préparation – Mesure dans laquelle les données du secteur GIE étaient suffisamment préparées, présentées, analysées et stockées avant la crise.	○	○	○	○	○	○	○	○	○	○	○
GEI-M8 - Absorption – Connaissance : Niveau de connaissance/conscience des menaces causées par la pandémie de COVID-19 dans le gouvernement central.	○	○	○	○	○	○	○	○	○	○	○
GEI-M9 - Récupération – Mesure dans laquelle les données de GIE ont été utilisées pour suivre le progrès vers la récupération et anticiper des scenarii de récupération.	○	○	○	○	○	○	○	○	○	○	○
GEI-M10 - Adaptation – Mesure dans laquelle le système du secteur GIE crée et améliore actuellement ses protocoles de stockage et d'utilisation des données en temps réel.	○	○	○	○	○	○	○	○	○	○	○

* Dimension cognitive : compréhension, modèles cognitifs, idées reçues, préjugés et valeurs.

	1	2	3	4	5	6	7	8	9	10	Je ne sais pas
GEI-M11 - Planification/Préparation – Mesure dans laquelle le système du secteur GIE et les décisions opérationnelles étaient préparés à anticiper la crise	○	○	○	○	○	○	○	○	○	○	○

	1	2	3	4	5	6	7	8	9	10	Je ne sais pas
GEI-M12 - Absorption – Mesure dans laquelle la réponse du secteur GIE a des protocoles de contingence suffisants et une gestion de crise proactive.											
GEI-M13 - Absorption – Redevabilité : Niveau d’effort pour assurer une redevabilité optimale vis-à-vis des ressources allouées pour soutenir la gouvernance dans le cadre de la réponse à la pandémie de COVID-19.											
GEI-M14 - Récupération – Mesure dans laquelle les décisions sont orientées vers la récupération et communiquées aux communautés sur la base de données probantes pour promouvoir des comportements sûrs.											
GEI-M15 - Récupération – Connaissance/Conscience : Mesure dans laquelle le secteur de la PF établit une certaine confiance avec le public à travers sa communication et le dialogue pour la prise de décision et l’action politique fondées sur des données probantes.											
GEI-M16 - Adaptation – Mesure dans laquelle le système du secteur GIE conçoit de nouvelles configurations systémiques, objectifs et critères de décision.											
GEI-M17 - Adaptation – Adaptatif : Niveau d’adaptation des systèmes financiers et des services sociaux pour assurer et faciliter l’accès aux plateformes technologiques pendant la pandémie de COVID-19.											

* Social dimension: interaction, collaboration and self-synchronization between people, entities and institutions.

	1	2	3	4	5	6	7	8	9	10	Je ne sais pas
GEI-M18 - Planification /Préparation – Mesure dans laquelle une formation sur la gestion d'épidémies/crises a été menée et a permis de mettre à profit les réseaux sociaux, le capital social et les normes institutionnelles et culturelles avant la crise.	○	○	○	○	○	○	○	○	○	○	○
GEI-M19 - Absorption – Mesure dans laquelle le personnel et les institutions sociales ont été accessibles et ont fait preuve d'ingéniosité dans leur réponse à l'épidémie/crise.	○	○	○	○	○	○	○	○	○	○	○
GEI-M20 - Absorption - Self-regulation: Extent to which national leaders had the authority to effect timely sectoral changes through an infrastructure flexible.	○	○	○	○	○	○	○	○	○	○	○
GEI-M21 - Récupération – Mesure dans laquelle le secteur GIE est impliqué dans le partage de connaissances et d'équipes pour améliorer la récupération du système.	○	○	○	○	○	○	○	○	○	○	○
GEI-M22 - Récupération – Diversité : Niveau d'engagement d'une équipe multidisciplinaire pour mitiger les effets de la pandémie de COVID-19 au niveau national et infranational.	○	○	○	○	○	○	○	○	○	○	○
GEI-M23 - Adaptation - Leadership et management : Gérance et gestion démontrées par le gouvernement en réponse à la pandémie de COVID-19 au niveau national.	○	○	○	○	○	○	○	○	○	○	○

* GEI M-24 Temporalité : Mesure de la rapidité de la réaction du gouvernement –sa communication et mise en œuvre des mesures- pour mitiger l'impact immédiat et sur le long terme de la pandémie de COVID-19. *Cette question porte sur la riposte nationale dans son ensemble, et non la réponse spécifique de votre secteur.*

	1	2	3	4	5	6	7	8	9	10	Je ne sais pas
1-Plus faible -> Plus élevé-10	○	○	○	○	○	○	○	○	○	○	○

GEI M-25 Commentaires : Utilisez cette section pour apporter tout commentaire additionnel sur la résilience et la durabilité des systèmes nationaux de gouvernance et des institutions économiques en vous référant à votre expérience de la pandémie de COVID-19. Si vous avez répondu « Je ne sais pas » à l'une des questions, utilisez cette section pour expliquer pourquoi.

Appendix 6.

Demographic Dividend Effort Index (DDEI) References to Source Indices

Family Planning (FP) DDEI Questionnaire

DDEI Questionnaire Item	Reference/ Source Index	Reference/ Source Index Item
Dimension 1. Policy/Policymaking		
*FP 1.2 **Policy on Fertility Reduction and FP**: Extent to which government policy emphasizes FP for family size as it relates to available resources or demographic reasons over health reasons or is simply neutral or opposed.	Family Planning Effort Index (FPEI)	Extent to which government policy stresses family planning for demographic reasons over health reasons or is simply neutral or opposed
*FP 1.4 **Policy on Age at Marriage**: Extent to which legal age at marriage for females is set at 18 years or higher and is enforced.	FPEI	Extent to which legal age at marriage for females is set at18 years or higher and is enforced
*FP 1.9 **Percent of in-country funding of FP budget**: Extent to which total family planning/population budget is derived from in-country sources (e.g., 1 for 10 percent, 5 for 50 percent, 10 for 100 percent).	FPEI	Extent to which total familyplanning/population budget isderived from in-countrysources (e.g., 1 for 10percent, 5 for 50 percent, 10for 100 percent)
FP 1.11.1 **Import Laws and Legal Regulations**: Extent to which import laws and legal regulations facilitate the importation of FP commodities and supplies	FPEI	Extent to which import laws and legal regulations facilitatethe importation ofcontraceptive supplies orextent to whichcontraceptives aremanufactured locally
*FP 1.11.2 **Import Laws and Legal Regulations**: Extent to which contraceptives are manufactured locally.	FPEI	Extent to which import laws and legal regulations facilitatethe importation ofcontraceptive supplies

Note

- *DDEI items are not always sourced from the reference index verbatim.*
- **Items that were dropped because they loaded poorly on the domain factors.*

For peer review only - http://bmjopen.bmj.com/site/about/guidelines.xhtml

DDEI Questionnaire Item	Reference/ Source Index	Reference/ Source Index Item
		orextent to whichcontraceptives aremanufactured locally
*FP 1.12 **Advertising of Contraceptives Allowed**: Extent of freedom from restrictions on advertising of contraceptives in the mass media.	FPEI	Extent of freedom from restrictions on advertising of contraceptives in the mass media
FP 1.14 **Political support for FP**: Extent to which elected officials in the country prioritize meeting FP needs to strengthen the FP contributions to the DD by passing laws and regulations and sponsoring actions and policies aimed at improving FP uptake and adherence.	FPEI	Extent to which the head of government, as well as other officials, speak publicly and favorably about family planning at least once or twice a year
Dimension 2. Services or Programs		
*FP 2.1 **Administrative Structure**: Extent to which administrative structure and staff at national, provincial and county levels are sufficient to implement the family planning program.	FPEI	Extent to which administrative structure and staff at national, provincial and county levels are adequate to implement the family planning program
*FP 2.2 **Level of Program Leaders**: High level of seniority of the director of the national family planning program and whether director reports to a high level of government.	FPEI	High level of seniority of the director of the national family planning program and whether director reports to ahigh level of government
*FP. 2.3 **Community-Based Distribution**: Extent to which areas of country not easily serviced by clinics or other service points are covered by community-based distribution programs for distribution of contraceptive supplies (especially rural areas)	FPEI	Extent to which areas of country not easily serviced by clinics or other service points are covered by community-based distribution programs for distribution of contraceptives (especially rural areas)

Note

- *DDEI items are not always sourced from the reference index verbatim.*
- **Items that were dropped because they loaded poorly on the domain factors.*

For peer review only - http://bmjopen.bmj.com/site/about/guidelines.xhtml

**DDEI Questionnaire Item**	**Reference/ Source Index**	**Reference/ Source Index Item**
*FP 2.4 **Postpartum Program**: Extent to which all postpartum women receive postpartum family planning services.	FPEI	Extent to which all new mothers in the country receive postpartum family planning assistance.
*FP 2.5 **Home-Visiting Workers**: Extent of coverage of population by workers whose primary task is to visit (rural) women in their homes to talk about family planning and maternal and child health.	FPEI	Extent of coverage of population by workers whose primary task is to visit (rural)women in their homes to talk about family planning and maternal and child health
FP 2.6.1 **Availability and Accessibility of FP Commodities/Supplies and Services Part 1**: Extent to which entire population has ready and easy access to: *IUD*	FPEI	Extent to which entire population has ready and easy access to IUDs *(Or: Pills, Injectables, voluntary Female Sterilization, voluntary male Sterilization, Condoms, safe Abortion or menstrual regulation (whether legal or not)*
FP 2.6.2 **Availability and Accessibility of FP Commodities/Supplies and Services Part 1**: Extent to which entire population has ready and easy access to: *Pills*	FPEI	Extent to which entire population has ready and easy access to IUDs *(Or: Pills, Injectables, voluntary Female Sterilization, voluntary male Sterilization, Condoms, safe Abortion or menstrual regulation (whether legal or not)*
FP 2.6.3 **Availability and Accessibility of FP Commodities/Supplies and Services Part 1**: Extent to which entire population has ready and easy access to: *Pills*	FPEI	Extent to which entire population has ready and easy access to IUDs *(Or: Pills, Injectables, voluntary Female Sterilization, voluntary male Sterilization, Condoms, safe Abortion or menstrual regulation (whether legal or not)*
FP 2.6.4 **Availability and Accessibility of FP Commodities/Supplies and Services Part 1**: Extent to which entire population has ready and easy access to: *Injectables*	FPEI	Extent to which entire population has ready and easy access to IUDs *(Or: Pills, Injectables, voluntary Female Sterilization, voluntary male Sterilization, Condoms,*

Note

- *DDEI items are not always sourced from the reference index verbatim.*
- **Items that were dropped because they loaded poorly on the domain factors.*

For peer review only - http://bmjopen.bmj.com/site/about/guidelines.xhtml

**DDEI Questionnaire Item**	**Reference/ Source Index**	**Reference/ Source Index Item**
		safe Abortion or menstrual regulation (whether legal or not)
*FP 2.6.5 **Availability and Accessibility of FP Commodities/Supplies and Services Part 1**: Extent to which entire population has ready and easy access to: *Female Sterilization*	FPEI	Extent to which entire population has ready and easy access to IUDs *(Or: Pills, Injectables, voluntary Female Sterilization, voluntary male Sterilization, Condoms, safe Abortion or menstrual regulation (whether legal or not)*
*FP 2.6.6 **Availability and Accessibility of FP Commodities/Supplies and Services Part 1**: Extent to which entire population has ready and easy access to: *Male Sterilization*	FPEI	Extent to which entire population has ready and easy access to IUDs *(Or: Pills, Injectables, voluntary Female Sterilization, voluntary male Sterilization, Condoms, safe Abortion or menstrual regulation (whether legal or not)*
*FP 2.6.7 **Availability and Accessibility of FP Commodities/Supplies and Services Part 1**: Extent to which entire population has ready and easy access to: Male *Condoms*	FPEI	Extent to which entire population has ready and easy access to IUDs *(Or: Pills, Injectables, voluntary Female Sterilization, voluntary male Sterilization, Condoms, safe Abortion or menstrual regulation (whether legal or not)*
FP 2.6.8 **Availability and Accessibility of FP Commodities/Supplies and Services Part 1**: Extent to which entire population has ready and easy access to: *Emergency Contraception*	FPEI	Extent to which entire population has ready and easy access to IUDs *(Or: Pills, Injectables, voluntary Female Sterilization, voluntary male Sterilization, Condoms, safe Abortion or menstrual regulation (whether legal or not)*
FP 2.6.9 **Availability and Accessibility of FP Commodities/Supplies and Services Part 1**: Extent to which entire population has ready and easy access to *Abortion/menstrual regulation (whether legal or not)*	FPEI	Extent to which entire population has ready and easy access to IUDs *(Or: Pills, Injectables, voluntary Female Sterilization, voluntary male Sterilization, Condoms, safe Abortion or menstrual regulation (whether legal or not)*

Note

- *DDEI items are not always sourced from the reference index verbatim.*
- **Items that were dropped because they loaded poorly on the domain factors.*

For peer review only - http://bmjopen.bmj.com/site/about/guidelines.xhtml

DDEI Questionnaire Item	Reference/ Source Index	Reference/ Source Index Item
FP 2.6.10 **Availability and Accessibility of FP Commodities/Supplies and Services Part 1**: Extent to which entire population has ready and easy access to: *Others*	FPEI	Extent to which entire population has ready and easy access to IUDs *(Or: Pills, Injectables, voluntary Female Sterilization, voluntary male Sterilization, Condoms, safe Abortion or menstrual regulation (whether legal or not)*
FP 2.7 **Availability and Accessibility of FP Commodities/Supplies and Services Part 2:** How well does the supply system operate (it avoids stockouts or interrupted supplies and guarantees a reliable flow at local levels) for the following: *IUD, Pills, Injectables, Condoms, Emergency Contraception*	FPEI	How well does the IUD *(Or: Pill, Injectable, Condom)* supply system operate (it avoids stockouts orinterrupted supplies and guarantees a reliable flow at local levels)
FP 2.8.1 **Availability and Accessibility of FP Commodities/Supplies and Services Part 3:** How well does the supply system provide necessary equipment and medical supplies at clinical facilities for the following: *Female Sterilization*	FPEI	How well does the supply system provide necessary equipment and medical supplies for female *(Or male)* sterilization services at clinical facilities?
FP 2.8.2 **Availability and Accessibility of FP Commodities/Supplies and Services Part 3:** How well does the supply system provide necessary equipment and medical supplies at clinical facilities for the following: *Male Sterilization*	FPEI	How well does the supply system provide necessary equipment and medical supplies for female *(Or male)* sterilization services at clinical facilities?
FP 2.8.3 **Availability and Accessibility of FP Commodities/Supplies and Services Part 3:** How well does the supply system provide necessary equipment and medical supplies at clinical facilities for the following: *IUDs*	FPEI	How well does the supply system provide necessary equipment and medical supplies for female *(Or male)* sterilization services at clinical facilities?
FP 2.8.4 **Availability and Accessibility of FP Commodities/Supplies and Services Part 3:** How well does the supply system provide necessary equipment and	FPEI	How well does the supply system provide necessary equipment and medical supplies for female *(Or male)* sterilization services at clinical facilities?

Note

- *DDEI items are not always sourced from the reference index verbatim.*
- **Items that were dropped because they loaded poorly on the domain factors.*

DDEI Questionnaire Item	Reference/ Source Index	Reference/ Source Index Item
medical supplies at clinical facilities for the following: *Implants*		
*FP 2.10 **Incentives and Disincentives**: Extent to which monetary or other incentives are used to encourage the adoption of family planning.	FPEI	Extent to which monetary or other incentives are used to encourage the adoption of family planning
FP 2.12 **Training Program**: Extent to which training programs, for each category of staff in the family planning program, are adequate to provide personnel with information and skills necessary to carry out their jobs effectively.	FPEI	Extent to which training programs, for each category of staff in the family planning program, are adequate to provide personnel with information and skills necessary to carry out their jobs effectively
FP 2.13 **Personnel Carry Out Assigned Tasks**: Extent to which all categories of family planning program staff (administrative, medical, paramedical, field) carry out assigned tasks effectively	FPEI	Extent to which all categories of family planning program staff (administrative, medical, paramedical, field) carry out assigned tasks effectively
FP 2.14 **Logistics and Transport**: Extent to which the logistics and transport systems are sufficient to keep stocks of contraceptive supplies and related equipment available at all service points, at all times and at all levels.	FPEI	Extent to which the logistics and transport systems are sufficient to keep stocks of contraceptive supplies and related equipment available at all service points, at all times and at all levels (central, provincial, local)
FP 2.15 **Supervision System**: Extent to which the system of supervision at all levels is adequate (regular monitoring visits with corrective or supportive action).	FPEI	Extent to which the system of supervision at all levels is adequate (regular monitoring visits with corrective or supportive action)
*FP 2.16 **Involvement of Other Ministries and Public Agencies**: Extent to which other ministries, government	FPEI	Extent to which other ministries and government agencies assist with family planning activities (e.g.,

Note

- *DDEI items are not always sourced from the reference index verbatim.*
- **Items that were dropped because they loaded poorly on the domain factors.*

For peer review only - http://bmjopen.bmj.com/site/about/guidelines.xhtml

DDEI Questionnaire Item	Reference/ Source Index	Reference/ Source Index Item
agencies and non-governmental stakeholders engage with FP activities (e.g., delivery of supplies, services, information, education) or other population activities.		delivery of supplies, services, information, education) or other population activities
FP 2.17 **Involvement of private-sector agencies and groups**: Extent to which private-sector agencies and groups assist with FP or other population activities.	FPEI	Extent to which private-sector agencies and groups assist with family planning or other population activities
*FP 2.18 **Mass Media for Information Education Communication**: Frequency and extent of coverage of mass media messages that provide population with information on family planning and service sites.	FPEI	Frequency and extent of coverage of mass media messages that provide population with information on family planning and service sites
*FP 2.21 **FP Effort Influences – Forces Affecting the FP Program**: Forces affecting the FP program can either strengthen or detract from its effectiveness. Check zero (0) if there is no difference either way; check a negative number from -1 to -5 if the influence is negative; or check a positive number from 1 to 5 if the influence is positive. (1 in either direction means little influence and 5 in either direction means strong influence.): a)*Decentralization, b)HIV/AIDS Programs, c)Incorporation of FP into a broader context of reproductive health,d) Integration of FP with other health services, e)Changes in donor funding, f)Changes in domestic government funding*	FPEI	Forces affecting the family planning program can either strengthen or detract from its effectiveness. Check zero (0) if there is no difference either way; check a negative number from-1 to -5 if the influence is negative; or check a positive number from 1 to 5 is the influence is positive. (1 in either direction means little influence and 5 in either direction means strong influence.): *Decentralization (the shift of decision making and resources from the central government to lower administrative levels)), HIV/AIDS Programs, Incorporation of FP into a broader context of reproductive health, Integration of FP with other health services, Changes in donor funding, Changes in domestic government funding*
Dimension 3. Advocacy		

Note

- *DDEI items are not always sourced from the reference index verbatim.*
- **Items that were dropped because they loaded poorly on the domain factors.*

For peer review only - http://bmjopen.bmj.com/site/about/guidelines.xhtml

DDEI Questionnaire Item	Reference/ Source Index	Reference/ Source Index Item
FP 3.4 **FP Effort Influences - Justification**: How important is each of the following as a current government justification for the national FP program? (1 means negligible importance; 5 means great importance.): *Reduce rate of population growth, Enhance economic development, Help women and men avoid unplanned and unwanted births, Improve women's health, Improve child health, Reduce unmarried adolescent childbearing, Reduce unmet need for contraceptive services*	FPEI	How important is each of the following as a current justification for the national family planning program? (1 means negligible importance; 10 means great importance.): *Reduce rate of population growth, Enhance economic development, Help women and men avoid unplanned and unwanted births, Improve women's health, Improve child health, Reduce unmarried adolescent childbearing, Reduce unmet need for contraceptive services*
FP 3.5 **FP Effort Influences – Special Populations**: To what extent does the family planning program give particular emphasis to special populations? (1 means negligible emphasis; 5 means great emphasis): *Unmarried youth, The poor, The disabled, Rural population, Other vulnerable groups (e.g. minotirty and/or indigenous groups), Postpartum women for counseling and contraceptive services, Post-abortion women for counseling and contraceptive services*	FPEI	To what extent does the family planning program give particular emphasis to special populations? (1means negligible emphasis; 10 means great emphasis: *Unmarried youth, The poor, Rural population, Postpartum women for counseling and contraceptive services, Post-abortion women for counseling and contraceptive services*
FP 3.6 **Social Marketing:** Extent of coverage of the country by a social marketing program (subsidized contraceptive sales at low cost in commercial sector, especially in urban areas).	FPEI	Extent of coverage of the country by a social marketing program (subsidized contraceptive sales at low cost in commercial sector, especially in urban areas)
Dimension 4. Research		
FP 4.6 **Record-Keeping**: Extent to which systems for client recordkeeping, clinic reporting and feedback of results are adequate, including being connected at all levels (federal, state/province, county, etc.).	FPEI	Extent to which systems forclient recordkeeping, clinicreporting and feedback ofresults are adequate

Note

- *DDEI items are not always sourced from the reference index verbatim.*
- **Items that were dropped because they loaded poorly on the domain factors.*

For peer review only - http://bmjopen.bmj.com/site/about/guidelines.xhtml

DDEI Questionnaire Item	Reference/ Source Index	Reference/ Source Index Item
FP 4.8 **Evaluation**: Extent to which program statistics, surveys, and small studies are used by specialized staff to report on program operations and measure progress.	FPEI	Extent to which programstatistics, national surveys, andsmall studies are used byspecialized staff to report onprogram operations andmeasure progress
FP 4.9 **Management's Use of Evaluation Findings**: Extent to which local-level program managers use research and evaluation findings to improve programming in ways suggested by findings.	FPEI	Extent to which program managers use research and evaluation findings to improve the program in ways suggested by findings

Source:
The Family Planning Effort Index: 1999, 2004, and 2009. (USAID 2010).
http://www.healthpolicyplus.com/archive/ns/pubs/hpi/Documents/1110_1_FP_Effort_Index_1999_2004_2009__FINAL_05_08_10_acc.pdf

Note

- *DDEI items are not always sourced from the reference index verbatim.*
- **Items that were dropped because they loaded poorly on the domain factors.*

For peer review only - http://bmjopen.bmj.com/site/about/guidelines.xhtml

Maternal and Child Health (MCH) DDEI Questionnaire

DDEI Questionnaire Item	Reference/ Source Index	Reference/ Source Index Item
Dimension 1. Policy/Policymaking		
MCH 1.1 **Adequate Policy**: Extent to which the Ministry of Health's policies toward pregnancy and delivery services account for the breadth of requirements to ensure that every mother and newborn can survive.	Maternal neonatal program index (MNPI)	Ministry of Health policies toward pregnancy and delivery services are adequate. *(Present effort and Effort 3 years ago)*
MCH 1.3 **Multisector Involvement**: Extent to which policies are developed through adequate consultation with interested parties such as other ministries, NGOs, private practitioners, and women's groups.	MNPI	Policies are developed through adequate consultation with interested parties such as otherministries, NGOs, private practitioners, women's groups. *(Present effort and Effort 3 years ago)*
*MCH 1.4 **Competent Service Providers**: Extent to which policies are reasonable and evidence-based concerning which personnel can provide maternal health services (e.g. trained midwives can perform a wide range of medical procedures).	MNPI	Policies are reasonable and fair concerning which personnel can provide maternal health services (e.g. trained midwives can perform a wide range of medical procedures)
MCH 1.6. **Post-Abortion Care Legislation**: Extent to which a favorable policy exists toward the treatment of complications of abortions, including complications seen from illegal abortions.	MNPI	A favorable policy exists toward the treatment of complications of abortions, including complications seen from illegal abortions
MCH 1.7 **Policy Reviews and Updates**: Extent to which policies are regularly reviewed by high-level policymakers and reviews are used to update action plans.	MNPI	Policies are vigorously implemented through regular high-level reviews and updated action plans

Note

- *DDEI items are not always sourced from the reference index verbatim.*
- **Items that were dropped because they loaded poorly on the domain factors.*

DDEI Questionnaire Item	Reference/ Source Index	Reference/ Source Index Item
MCH 1.8 **High-Level Actor Placement**: Extent and level to which the director of maternal and child health services is involved in high-level decision making.	MNPI	The director of services for maternal health is placed at a high administrative level
MCH 1.9 **Financing for MCH**: Extent to which the government budget for safe pregnancy, delivery, perinatal care (for facilities, personnel, supplies, etc.) and children's health is adequate for the needs, whether from the Ministry of Health, provincial/ county government or donor support.	MNPI	The government budget for safe pregnancy, delivery, and postpartum care (for facilities, personnel, supplies, etc.) is adequate for the needs, whether from the Ministry of Health, provincial government or donor support
MCH 1.10 **Affordability**: Extent to which all MCH-related services and drugs are affordable to all clients.	MNPI	All services and drugs are provided free to all clients
*MCH 1.11 **Private Sector**: Extent to which the private sector (doctors, midwives, clinics) is active and covers a substantial share of pregnancy and delivery cases.	MNPI	he private sector (doctors, midwives, clinics, maternity homes) is active and covers a substantial share of pregnancy and delivery cases.
Dimension 2. Services or Programs		
MCH 2.1 **Obstetric Care Availability – All Centers**: Extent to which all primary health facilities have trained staff, in place, who can provide obstetric care to:<br>MCH 2.1.1 Properly manage postpartum hemorrhage cases.<br>MCH 2.1.2 Timely and properly administer antibiotics intravenously when needed.<br>MCH 2.1.3 Perform manual removal of retained placenta.	MNPI	All health centers have trained staff, in place, who can provide obstetric care:<br>Properly manage postpartum hemorrhage cases.<br>Timely and properly administer antibiotics intravenously when needed.<br>Perform manual removal of retained placenta.<br>Perform vacuum aspiration of the uterus, using MVA (manual vacuum aspiration) or an electric suction device.

Note

- *DDEI items are not always sourced from the reference index verbatim.*
- **Items that were dropped because they loaded poorly on the domain factors.*

For peer review only - http://bmjopen.bmj.com/site/about/guidelines.xhtml

DDEI Questionnaire Item	Reference/ Source Index	Reference/ Source Index Item
MCH 2.1.4 Perform vacuum aspiration of the uterus, using MVA (manual vacuum aspiration) or an electric suction device.<br>MCH 2.1.5 Use a partograph to determine when to refer.<br>MCH 2.1.6 Have transportation arrangements to quickly move a woman with obstructed labor to a district/sub-county hospital.<br>MCH 2.1.7 Have adequate antibiotic supplies on hand (sufficient supplies of the correct types).		Use a partograph to determine when to refer.<br>Have transportation arrangements to quickly move a woman with obstructed labor to a district/sub-county hospital.<br>Have adequate antibiotic supplies on hand (sufficient supplies of the correct types).
MCH 2.2 **Obstetric Care Availability – Secondary healthcare facilities:** Extent to which all district/sub-county hospitals have trained staff, in place, who can:<br>MCH 2.2.1 Provide all functions listed above for primary health facilities.<br>MCH 2.2.2 Perform blood transfusions (and have adequate supplies of safe blood on hand).<br>MCH 2.2.3 Perform Cesarean section.	MNPI	All First Referral Facilities-District Hospitals-have trained staff, in place, who can:<br>Provide all functions listed above for primary health facilities.<br>Perform blood transfusions (and have adequate supplies of safe blood on hand).<br>Perform Cesarean section.
MCH 2.3 **Pregnancy-Related Services**: Extent to which all pregnant women have adequate access to:<br>MCH 2.3.1 Treatment for postpartum hemorrhage during or soon after delivery.<br>MCH 2.3.2 Management of obstructed labor.<br>MCH 2.3.3 Management of pre-eclampsia, eclampsia and its complications.<br>MCH 2.3.4 Treatment of post abortion care or for abortion complications.<br>MCH 2.3.5 Provision of safe abortion services.<br>MCH 2.3.6 Antenatal care during pregnancy.	MNPI	Access to Services by Pregnant Women – Many women do not have access to a trained professional attendant, a health center with beds, or a district health center—and even if they do, the nearest attendant or facility may not have staff or equipment, or the service may be too expensive. So this section pertains to the percentage of pregnant women with adequate access to each service. (Enter percentage in each cell.)<br>What percentage of pregnant women have adequate access to: *(Separated by Rural and Urban women):*

Note

- *DDEI items are not always sourced from the reference index verbatim.*
- **Items that were dropped because they loaded poorly on the domain factors.*

For peer review only - http://bmjopen.bmj.com/site/about/guidelines.xhtml

DDEI Questionnaire Item	Reference/ Source Index	Reference/ Source Index Item
MCH 2.3.7 Delivery care by a trained professional attendant.<br>MCH 2.3.8 Postpartum family planning services.<br>MCH 2.3.9 District/sub-county hospitals that are open 24 hours/day.		Treatment for postpartum hemorrhage during or soon after delivery.<br>Management of obstructed labor.<br>Management of pre-eclampsia, eclampsia and its complications.<br>Treatment of post abortion care or for abortion complications.<br>Provision of safe abortion services.<br>Antenatal care during pregnancy.<br>Delivery care by a trained professional attendant.<br>Postpartum family planning services.<br>District/sub-county hospitals that are open 24 hours/day.
MCH 2.5 **Newborn Care**: Extent to which, for newborn care, all infants whether delivered at home or in a facility:<br>MCH 2.5.1 Have their mouth and nasal passageways cleared<br>MCH 2.5.2 Are dried and kept warm immediately after birth<br>*MCH 2.5.3 Receive vitamin A<br>MCH 2.5.4 Have their umbilical cord cut with a clean blade<br>MCH 2.5.5 Complete WHO recommended vaccination calendar<br>MCH 2.5.6 Benefit from exclusive breastfeeding promotion and education activities<br>MCH 2.5.7 Benefit from nutritional support programs when in need.	MNPI	Delivery and antenatal care – For newborn care, all infants whether delivered at home or in a facility:<br>Have their mouth and nasal passageways cleared:<br>Have their mouth and nasal passageways cleared<br>Are dried and kept warm immediately after birth<br>Receive prophylactic treatment for their eyes<br>Have their umbilical cord cut with a clean blade<br>Receive a DPT injection at 3 months<br>Are scheduled for subsequent immunizations

Note

- *DDEI items are not always sourced from the reference index verbatim.*
- **Items that were dropped because they loaded poorly on the domain factors.*

For peer review only - http://bmjopen.bmj.com/site/about/guidelines.xhtml

DDEI Questionnaire Item	Reference/ Source Index	Reference/ Source Index Item
MCH 2.6 **Family Planning – All Centers**: Extent to which there exists proper provision of family planning at all health centers that:<br>MCH 2.6.1 Routinely offer family planning services for post-abortion cases<br>MCH 2.6.2 Routinely offer family planning at postpartum visits<br>MCH 2.6.3 Have short-term and LARC contraceptive supplies regularly in stock<br>MCH 2.6.4 Have trained staff, in place, who can provide services for LARCs.<br>MCH 2.6.5 Have guidelines and protocols necessary for the provision of family planning services.	MNPI	Provision of Family Planning – All health centers:<br>Routinely offer family planning services for post-abortion cases<br>Routinely offer family planning at postpartum visits<br>Have short-term and LARC contraceptive supplies regularly in stock<br>Have trained staff, in place, who can provide services for LARCs.<br>Have guidelines and protocols necessary for the provision of family planning services.
MCH 2.7 **Family Planning – District/sub-county Hospitals:** Extent to which there exists proper provision of family planning at district/sub-county hospitals that:<br>MCH 2.7.1 Routinely offer family planning services for post-abortion cases<br>MCH 2.7.2 Routinely offer family planning at postpartum visits<br>MCH 2.7.3 Have short term and LARC contraceptive supplies regularly in stock<br>MCH 2.7.4 Have trained staff, in place, who can provide services for LARCs<br>*MCH 2.7.5 Can offer sterilization to female clients<br>*MCH 2.7.6 Can offer sterilization to male clients<br>MCH 2.7.7 Have guidelines and protocols necessary for the provision of family planning services.	MNPI	Provision of Family Planning – All first referral facilities-district hospitals:<br>Routinely offer family planning services for post-abortion cases<br>Routinely offer family planning at postpartum visits<br>Have short term and LARC contraceptive supplies regularly in stock<br>Have trained staff, in place, who can provide services for LARCs<br>Can offer sterilization to female clients<br>Can offer sterilization to male clients

Note

- *DDEI items are not always sourced from the reference index verbatim.*
- **Items that were dropped because they loaded poorly on the domain factors.*

For peer review only - http://bmjopen.bmj.com/site/about/guidelines.xhtml

DDEI Questionnaire Item	Reference/ Source Index	Reference/ Source Index Item
Dimension 3. Advocacy		
MCH 3.1 **Government Messages**: Extent to which high officials in the government, including the Ministry of Health, issue frequent statements to all stakeholders including the press to support improvements for safe pregnancy, safe delivery and healthy childhood.	MNPI	High officials in the government, including the Ministry of Health, issue frequent statements to the press and public to support improvements for safe pregnancy and delivery
MCH 3.2 **Mass Media**: Extent to which the national program uses the mass media to educate the public about symptoms of pregnancy complications, safe childbirth and healthy childhood.	MNPI	The national program uses the mass media to educate the public about symptoms of pregnancy complications and safe places for childbirth
MCH 3.3.1 **Community-Level Media**: Extent to which community groups take part in systematic programs to educate the public about safe pregnancy	MNPI	Community-level organizations take part in systematic programs to educate the public about safe pregnancy and delivery
MCH 3.3.2 **Community-Level Media**: Extent to which community groups take part in systematic programs to educate the public about safe delivery	MNPI	Community-level organizations take part in systematic programs to educate the public about safe pregnancy and delivery
MCH 3.3.3 **Community-Level Media**: Extent to which community groups take part in systematic programs to educate the public about healthy childhood.	MNPI	Community-level organizations take part in systematic programs to educate the public about safe pregnancy and delivery
MCH 3.4 **Educational Materials**: Extent to which the appropriate ministry (MOH) supplies adequate educational materials (posters, pamphlets, etc.) to delivery facilities to instruct clients about safe practices.	MNPI	he Ministry of Health supplies adequate educational materials (posters, pamphlets, etc.) to delivery facilities to instruct clients about safe practices

Source
Rating Maternal and Neonatal Health Programs in Developing Countries. (Bulatao & Ross). (Measure Evaluation – USAID). 2000. Appendix A Questionnaire Items and Variable Codes for the Maternal and Neonatal Program Effort Index (MNPI)
https://pdf.usaid.gov/pdf_docs/pnacl165.pdf

Note
- *DDEI items are not always sourced from the reference index verbatim.*
- **Items that were dropped because they loaded poorly on the domain factors.*

For peer review only - http://bmjopen.bmj.com/site/about/guidelines.xhtml

Education (ED) DEI Questionnaire

DDEI Questionnaire Item	Reference/ Source Index	Reference/ Source Index Item
Dimension 1. Policy/Policymaking		
ED 1.1 **Quality**: Extent to which there are national plans/strategies/programs to improve the quality of learning in schools.	UNESCO. EFA Global Monitoring Report. 2013-14. *The Education for All Development Index.*	Extent of existing strategies to improve quality learning in schools?
ED 1.2 **Disadvantaged Groups**:<br>ED 1.2.1 Extent to which strategies have been developed and implemented/deployed to overcome learning obstacles for pregnant girls and adolescent mothers.<br>ED 1.2.2 Extent to which strategies have been developed and implemented/deployed to overcome learning obstacles for disabled populations.	UNESCO. EFA Global Monitoring Report. 2013-14.	Strategies to overcome learning obstacles for disadvantaged groups.
ED 1.3 **Teachers**: Extent to which teacher recruitment, development and management issues are included in policy aimed at improving learning outcomes.	UNESCO. EFA Global Monitoring Report. 2013-14.	Inclusion of teacher recruitment, development and management issues in policy aimed at improving learning outcomes? If so, what linkages are identified?
ED 1.4 **Teachers' Accountability**: Extent to which national plans include specific measures, including monitoring and evaluation, that aim to hold teachers' accountable for improving learning.	UNESCO. EFA Global Monitoring Report. 2013-14.	Inclusion of specific measures that aim to hold teachers' accountable for improving learning.
ED 1.5 **Teacher Quality:** Extent to which education policies/strategies/programs include verifiable indicators for teacher quality.	UNESCO. EFA Global Monitoring Report. 2013-14.	Inclusion of verifiable indicators for improving learning and for teacher qualityin education policies?

Note

- *DDEI items are not always sourced from the reference index verbatim.*
- **Items that were dropped because they loaded poorly on the domain factors.*

For peer review only - http://bmjopen.bmj.com/site/about/guidelines.xhtml

DDEI Questionnaire Item	Reference/ Source Index	Reference/ Source Index Item
ED 1.7 **Gender Parity SDG**: Extent to which national policies/plans/strategies consider the (SDG) goal for complete gender parity at all levels of education.	UNESCO. EFA Global Monitoring Report. 2005. *The Education for All Development Index.*	The EDI constituents and related indicators includes:<br>a. gender: gender-specific EFA index (GEI, thearithmetical mean of the GPIs for the primaryand secondary gross enrolment ratios and theadult literacy rate)
ED 1.8 **Education Financing**: Extent to which new education plans include financing proposals with budgets allocated for learning and teaching reforms.	UNESCO. EFA Global Monitoring Report. 2013-14.	Extent to which new education plans include financing proposals with budgetsallocated for learning and teaching reforms
ED 1.10 **Adult Illiteracy**: Extent to which national policies/plans/strategies aim to address illiteracy.	UNESCO. EFA Global Monitoring Report. 2005. *The Education for All Development Index.*	The EDI constituents and related indicators includes:<br>b. adult literacy: literacy rate of the group aged 15and over;
Dimension 2. Services or Programs		
ED 2.1 **Teacher Deployment**: Extent to which strategies/plans are used to ensure teachers are deployed to areas of the country where they are most needed.	UNESCO. EFA Global Monitoring Report. 2013-14.	Strategies to ensure teachers are deployed to areas of countries where they aremost needed?
ED 2.2 **Teacher Quality**: Extent to which teacher quality is recognized as a key factor in improving learning outcomes.<br>ED 2.2.1 Extent to which the quality of teacher education is emphasized in wider education policies/plans/strategies.<br>ED 2.2.2 Extent to which the quality of training and continuous professional development is emphasized in wider education policies/plans/strategies.	UNESCO. EFA Global Monitoring Report. 2013-14.	How is teacher quality recognized as a key factor in improving learning outcomes?<br><br>Extent of specific strategies to recognize teacher quality

Note

- *DDEI items are not always sourced from the reference index verbatim.*
- **Items that were dropped because they loaded poorly on the domain factors.*

For peer review only - http://bmjopen.bmj.com/site/about/guidelines.xhtml

DDEI Questionnaire Item	Reference/ Source Index	Reference/ Source Index Item
ED 2.3 **Gender Parity**: Extent to which resources have been utilized to improve equality in education between boys and girls.	UNESCO. EFA Global Monitoring Report. 2005. *The Education for All Development Index.*	The EDI constituents and related indicators includes: gender: gender-specific EFA index (GEI, thearithmetical mean of the GPIs for the primaryand secondary gross enrolment ratios and theadult literacy rate)

Sources

1. UNESCO. Teaching and learning: achieving quality for all; EFA Global Monitoring Report. 2013-2014. *Part 1: Monitoring Progress Toward the EFA Goals.* https://unesdoc.unesco.org/ark:/48223/pf0000225660

2. UNESCO. Education for all: the quality imperative; EFA Global Monitoring Report. *Chapter 3: Assessing Progress Toward the EFA Goals – The Education for All Development Index.* 2005. https://unesdoc.unesco.org/ark:/48223/pf0000137333

3. SYSTEM APPROACH FOR BETTER EDUCATION RESULTS (SABER) Data Collection Instrument for SABER - School Autonomy and Accountability 2.0: http://wbgfiles.worldbank.org/documents/hdn/ed/saber/supporting_doc/Background/SAA/SABER_SAA_%20Data_Collection_Instrument.pdf

4. What Matters Most for School Autonomy and Accountability: A Framework Paper18: https://documents1.worldbank.org/curated/en/385451468172788612/pdf/What-matters-most-for-school-autonomy-and-accountability-a-framework-paper.pdf

Note

- *DDEI items are not always sourced from the reference index verbatim.*
- **Items that were dropped because they loaded poorly on the domain factors.*

For peer review only - http://bmjopen.bmj.com/site/about/guidelines.xhtml

Women Empowerement (WE) DDEI Questionnaire

There was not a specific effort index that was integrated with the Women's Empowerment DDEI Questionnaire. Rather, an existing index was relied on heavily to help the authors draft the DDEI items specifically in reference to levels of effort. This was the African Gender Development Index (AGDI) of 2011: https://archive.uneca.org/sites/default/files/PublicationFiles/agdi_2011_eng_fin.pdf

Labor Market (LM) DDEI Questionnaire

There was not a specific effort index that was integrated with the Labor Market DDEI Questionnaire. Rather, a handful of existing frameworks were relied on heavily to help the authors draft the DDEI items specifically in reference to levels of effort. These were:

1. IMF. The Future of Work in Sub-Saharan Africa. 2018. https://www.imf.org/en/Publications/Departmental-Papers-Policy-Papers/Issues/2018/12/14/The-Future-of-Work-in-Sub-Saharan-Africa-46333
2. IMF. Designing Labor Market Institutions in Emerging Market and Developing Economies: Evidence and Policy Options. 2019. https://www.imf.org/en/Publications/Staff-Discussion-Notes/Issues/2019/05/15/Designing-Labor-Market-Institutions-in-Emerging-and-Developing-Economies-Evidence-and-Policy-46855
3. The African Union Commission & UNFPA. AU Roadmap on Harnessing the Demographic Dividend Through Investments In Youth. 2017. https://wcaro.unfpa.org/sites/default/files/pub-pdf/AU 2017 DD ROADMAP Final - EN.pdf

Good governance and economic institutions (GGEI) DDEI Questionnaire

There was not a specific effort index that was integrated with the Women's Empowerment DDEI Questionnaire. Rather, an existing index was relied on heavily to help the authors draft the DDEI items specifically in reference to levels of effort. This was the AU Roadmap on Harnessing the Demographic Dividend Through Investments In Youth (2017): https://wcaro.unfpa.org/sites/default/files/pub-pdf/AU%202017%20DD%20ROADMAP%20Final%20-%20EN.pdf. We also examined the World Bank Worldwide Governance Indicators: https://info.worldbank.org/governance/wgi/Home/Documents.

General note:

Across all DDEI questionnaires the domain on Civil Society pulls largely from:

1. USAID. Civil Society Involvement in Family Planning A Review of Global Programming and Evidence. 2017. https://evidenceproject.popcouncil.org/wp-content/uploads/2017/12/India-CSO_Research-Report.pdf; and
2. WHO. Strategies, tactics and approaches: Conducting and evaluating national civil society advocacy for reproductive, maternal and child health. 2014. https://apps.who.int/iris/bitstream/handle/10665/100626/9789241506687_eng.pdf;jsessionid=514C2178A6164B5E84306E330B300E64?sequence=1

Note

- *DDEI items are not always sourced from the reference index verbatim.*
- **Items that were dropped because they loaded poorly on the domain factors.*

For peer review only - http://bmjopen.bmj.com/site/about/guidelines.xhtml

Appendix 3. Results from factor analysis

Sector - Family planning

**Policy**		**Service and Programs**		**Advocacy**		**Research**		**Civil society**	
**Obs.**	**75**	**Obs.**	**53**	**Obs.**	**73**	**Obs.**	**75**	**Obs.**	**80**
FP1_1	0.54	FP2_1	**0.47**	FP3_1	**0.45**	FP4_1a	0.66	FP5_1	0.73
FP1_2	**0.41**	FP2_2	**0.39**	FP3_2	**0.31**	FP4_1b	0.70	FP5_2	0.70
FP1_3	0.62	FP2_3	**0.46**	FP3_3	**0.38**	FP4_1c	0.70	FP5_3	0.88
FP1_4	**0.49**	FP2_4	**0.50**	FP3_4a	0.50	FP4_2	0.71	FP5_4	0.88
FP1_5	0.74	FP2_5	**0.25**	FP3_4b	0.59	FP4_3a	0.64	FP5_5	0.87
FP1_6	0.72	FP2_6a	0.74	FP3_4c	0.78	FP4_3b	0.73	FP5_6	0.75
FP1_7	0.61	FP2_6b	0.65	FP3_4d	0.78	FP4_3c	0.60	FP5_7	0.80
FP1_8	0.54	FP2_6c	0.62	FP3_4e	0.81	FP4_4a	0.77	FP5_8	0.88
FP1_9	**0.45**	FP2_6d	0.75	FP3_4f	0.75	FP4_4b	0.74	FP5_9	0.88
FP1_10	0.67	FP2_6e	**0.44**	FP3_4g	0.81	FP4_4c	0.77	FP5_10	0.84
FP1_11a	0.59	FP2_6f	**0.45**	FP3_5a	0.73	FP4_4d	0.67	FP5_11	0.78
FP1_11b	**0.48**	FP2_6g	0.50	FP3_5b	0.77	FP4_5a	0.69	FP5_12	0.87
FP1_12	**0.39**	FP2_6h	0.57	FP3_5c	0.79	FP4_5b	0.78	FP5_13	0.89
FP1_13	0.52	FP2_6i	**0.38**	FP3_5d	0.72	FP4_5c	0.76	FP5_14	0.87
FP1_14	0.66	FP2_6j	0.63	FP3_5e	0.77	FP4_5d	0.79		
		FP2_7a	0.75	FP3_5f	0.70	FP4_6	0.66		
		FP2_7b	0.61	FP3_5g	0.71	FP4_7	0.69		
		FP2_7c	0.66	FP3_6	0.54	FP4_8	0.74		
		FP2_7d	0.75			FP4_9	0.68		
		FP2_7e	0.58			FP4_10	0.72		
		FP2_7f	0.53			FP4_11	0.68		
		FP2_7g	0.57						
		FP2_8a	0.52						
		FP2_8b	**0.38**						
		FP2_8c	0.67						
		FP2_8d	0.68						
		FP2_9	0.62						
		FP2_10	**0.16**						
		FP2_11	0.51						
		FP2_12	0.63						
		FP2_13	0.78						
		FP2_14	0.72						
		FP2_15	0.57						
		FP2_16	**0.43**						
		FP2_17	0.54						
		FP2_18	**0.36**						
		FP2_19	**0.45**						
		FP2_20	0.54						
		FP2_21a	**0.20**						
		FP2_21b	**0.25**						
		FP2_21c	**0.11**						
		FP2_21d	**0.06**						
		FP2_21e	**0.29**						
		FP2_21f	**0.09**						

Note

Bolded and shaded are the loadings of items that were dropped because they loaded poorly (<0.5) on domain factors.

For peer review only

Sector - Maternal and Child Health

Policy		Service and Programs		Advocacy		Research		Civil society	
**Obs.**	**60**	**Obs.**	**40**	**Obs.**	**67**	**Obs.**	**49**	**Obs.**	**52**
MCH1_1a	0.58	MCH2_1a	0.80	MCH3_1	0.78	MCH4_1a	0.80	MCH5_1	0.87
MCH1_1b	0.69	MCH2_1b	0.80	MCH3_2	0.89	MCH4_1b	0.84	MCH5_2	0.87
MCH1_2	0.67	MCH2_1c	0.73	MCH3_3	0.86	MCH4_1c	0.82	MCH5_3	0.91
MCH1_3	0.65	MCH2_1d	0.67	MCH3_4	0.85	MCH4_2	0.87	MCH5_4	0.91
MCH1_4	**0.40**	MCH2_1e	0.83	MCH3_5	0.75	MCH4_3a	0.85	MCH5_5	0.86
MCH1_5	**0.49**	MCH2_1f	0.78	MCH3_6	0.80	MCH4_3b	0.89	MCH5_6	0.88
MCH1_6	0.66	MCH2_1g	0.71			MCH4_3c	0.74	MCH5_7	0.84
MCH1_7	0.81	MCH2_2a	0.86			MCH4_4a	0.88	MCH5_8	0.91
MCH1_8	0.65	MCH2_2b	0.74			MCH4_4b	0.74	MCH5_9	0.88
MCH1_9	0.82	MCH2_2c	0.73			MCH4_4c	0.84	MCH5_10	0.91
MCH1_10	0.68	MCH2_3a	0.87			MCH4_4d	0.86	MCH5_11	0.91
MCH1_11	**0.24**	MCH2_3b	0.82			MCH4_5a	0.88	MCH5_12	0.90
MCH1_12	0.58	MCH2_3c	0.84			MCH4_5b	0.91		
MCH1_13a	0.79	MCH2_3d	0.91			MCH4_5c	0.94		
MCH1_13b	0.81	MCH2_3e	**0.44**			MCH4_5d	0.91		
MCH1_13c	0.82	MCH2_3f	0.62			MCH4_5e	0.92		
MCH1_13d	0.82	MCH2_3g	0.84			MCH4_6	0.77		
MCH1_13e	0.73	MCH2_3h	0.78			MCH4_7	0.74		
		MCH2_3i	0.79			MCH4_8	0.93		
		MCH2_4a	0.84			MCH4_9	0.87		
		MCH2_4b	0.85			MCH4_10	0.89		
		MCH2_4c	0.88			MCH4_11	0.87		
		MCH2_4d	0.87						
		MCH2_5a	0.84						
		MCH2_5b	0.80						
		MCH2_5c	**0.22**						
		MCH2_5d	0.81						
		MCH2_5e	0.79						
		MCH2_5f	0.87						
		MCH2_5g	0.72						
		MCH2_6a	0.81						
		MCH2_6b	0.87						
		MCH2_6c	0.80						
		MCH2_6d	0.77						
		MCH2_6e	0.84						
		MCH2_7a	0.80						
		MCH2_7b	0.87						
		MCH2_7c	0.67						
		MCH2_7d	0.65						
		MCH2_7e	**0.35**						
		MCH2_7f	**0.26**						
		MCH2_7g	0.66						
		MCH2_8a	0.78						
		MCH2_8b	0.86						

Note

Bolded and shaded are the loadings of items that were dropped because they loaded poorly (<0.5) on domain factors.

For peer review only

Sector – Education

Policy		Service and Programs		Advocacy		Research		Civil society	
**Obs.**	**41**	**Obs.**	**52**	**Obs.**	**53**	**Obs.**	**35**	**Obs.**	**44**
ED1_1	0.74	ED2_1	0.78	ED3_1	0.76	ED4_1a	0.77	ED5_1	0.92
ED1_2a	0.79	ED2_2a	0.79	ED3_2	0.80	ED4_1b	0.88	ED5_2	0.92
ED1_2b	0.76	ED2_2b	0.85	ED3_3	0.87	ED4_1c	0.85	ED5_3	0.94
ED1_3	0.77	ED2_3	0.84	ED3_4	0.88	ED4_2	0.86	ED5_4	0.93
ED1_4	0.77	ED2_4	0.88			ED4_3a	0.76	ED5_5	0.94
ED1_5	0.87	ED2_5	0.70			ED4_3b	0.74	ED5_6	0.85
ED1_6	0.77	ED2_6	0.74			ED4_3c	0.79	ED5_7	0.91
ED1_7	0.78					ED4_4a	0.82	ED5_8	0.92
ED1_8	0.73					ED4_4b	0.80	ED5_9	0.93
ED1_9a	0.72					ED4_4c	0.77	ED5_10	0.94
ED1_9b	0.82					ED4_4d	0.74	ED5_11	0.94
ED1_9c	0.85					ED4_5a	0.84		
ED1_10a	0.78					ED4_5b	0.88		
ED1_10b	0.76					ED4_5c	0.92		
ED1_11	0.81					ED4_5d	0.92		
ED1_12	0.71					ED4_6	0.87		
ED1_13	0.74					ED4_7	0.85		
						ED4_8	0.89		
						ED4_9	0.87		
						ED4_10	0.90		
						ED4_11a	0.93		
						ED4_11b	0.91		

For peer review only

Note
Bolded and shaded are the loadings of items that were dropped because they loaded poorly (<0.5) on domain factors.

Sector - Women Empowerment

Policy		Service and Programs		Advocacy		Research		Civil society	
Obs.	**55**	Obs.	**62**	Obs.	**66**	Obs.	**42**	Obs.	**60**
WE1_1	0.69	WE2_1	0.90	WE3_1	0.88	WE4_1a	0.81	WE5_1	0.84
WE1_2	0.79	WE2_2	0.90	WE3_2	0.87	WE4_1b	0.86	WE5_2	0.90
WE1_3	0.77	WE2_3	0.73	WE3_3	0.85	WE4_1c	0.84	WE5_3	0.89
WE1_4	0.84	WE2_4	0.90	WE3_4	0.86	WE4_2	0.91	WE5_4	0.85
WE1_5	0.88	WE2_5	0.86	WE3_5	0.76	WE4_3a	0.85	WE5_5	0.91
WE1_6	0.75	WE2_6	0.80	WE3_6	0.86	WE4_3b	0.82	WE5_6	0.89
WE1_7	0.74	WE2_7	0.76	WE3_7	0.85	WE4_3c	0.73	WE5_7	0.83
WE1_8a	0.72	WE2_8	0.77			WE4_4a	0.86	WE5_8	0.92
WE1_8b	0.87	WE2_9	0.89			WE4_4b	0.95	WE5_9	0.87
WE1_9	0.91	WE2_10	0.74			WE4_4c	0.95	WE5_10	0.90
WE1_10	0.81	WE2_11	0.87			WE4_4d	0.93		
WE1_11	0.76	WE2_12	0.68			WE4_5a	0.88		
WE1_12	0.83					WE4_5b	0.93		
WE1_13a	0.75					WE4_5c	0.92		
WE1_13b	0.91					WE4_6	0.95		
WE1_14	0.88					WE4_7	0.94		
WE1_15	0.92					WE4_8	0.96		
WE1_16	0.84					WE4_9	0.90		
WE1_17a	0.87					WE4_10	0.83		
WE1_17b	0.91								
WE1_17c	0.86								
WE1_17d	0.93								
WE1_18	0.89								

For peer review only

Note

Bolded and shaded are the loadings of items that were dropped because they loaded poorly (<0.5) on domain factors.

Sector - Labor market

**Policy**		**Service and Programs**		**Advocacy**		**Research**		**Civil society**	
**Obs.**	**51**	**Obs.**	**51**	**Obs.**	**54**	**Obs.**	**46**	**Obs.**	**47**
LM1_1a	**0.47**	LM2_1	0.75	LM3_1	0.56	LM4_1a	0.79	LM5_1	0.84
LM1_1b	**0.36**	LM2_2	0.87	LM3_2	0.81	LM4_1b	0.78	LM5_2	0.92
LM1_2	**0.33**	LM2_3	0.73	LM3_3	0.61	LM4_1c	0.88	LM5_3	0.91
LM1_3	0.71	LM2_4	0.88	LM3_4	0.84	LM4_2	0.80	LM5_4	0.92
LM1_4a	0.58	LM2_5	0.88	LM3_5	0.88	LM4_3a	0.75	LM5_5	0.87
LM1_4b	0.62	LM2_6	0.88			LM4_3b	0.76	LM5_6	0.92
LM1_5	0.82	LM2_7	0.86			LM4_3c	0.79	LM5_7	0.87
LM1_6	0.82	LM2_8	0.78			LM4_4a	0.74	LM5_8	0.89
LM1_7	0.88	LM2_9	0.64			LM4_4b	0.79	LM5_9	0.87
LM1_8	0.86	LM2_10	0.73			LM4_4c	0.71	LM5_10	0.84
LM1_9	0.84	LM2_11	0.63			LM4_4d	0.77		
LM1_10	0.81	LM2_12a	0.58			LM4_5a	0.84		
LM1_11	0.82	LM2_12b	0.67			LM4_5b	0.84		
LM1_12	0.87	LM2_13	0.66			LM4_5c	0.78		
LM1_13	0.70	LM2_14	0.80			LM4_6	0.88		
LM1_14	0.76	LM2_15	0.82			LM4_7	0.78		
LM1_15	0.76					LM4_8	0.79		
						LM4_9	0.73		
						LM4_10	0.77		
						LM4_11	0.82		

Governance and Economic Institution

**Policy**		**Service and Programs**		**Advocacy**		**Research**		**Civil society**	
**Obs.**	**37**	**Obs.**	**40**	**Obs.**	**30**	**Obs.**	**26**	**Obs.**	**43**
GEI1_1	0.85	GEI2_1	0.91	GEI3_1	0.65	GEI4_1a	0.85	GEI5_1	0.72
GEI1_2	0.78	GEI2_2	0.91	GEI3_2	0.81	GEI4_1b	0.89	GEI5_2	0.89
GEI1_3	0.84	GEI2_3	0.88	GEI3_3	0.62	GEI4_1c	0.70	GEI5_3	0.84
GEI1_4	0.86	GEI2_4	0.91	GEI3_4	0.64	GEI4_2	0.72	GEI5_4	0.83
GEI1_5	0.89	GEI2_5	0.82	GEI3_5	0.81	GEI4_3a	0.87	GEI5_5	0.78
GEI1_6	0.82	GEI2_6	0.91	GEI3_6a	0.90	GEI4_3b	0.88	GEI5_6	0.73
GEI1_7	0.90	GEI2_7	0.79	GEI3_6b	0.80	GEI4_3c	0.84	GEI5_7a	0.77
GEI1_8	0.89	GEI2_8a	0.88	GEI3_6c	0.67	GEI4_4a	0.85	GEI5_7b	0.71
GEI1_9a	0.90	GEI2_8b	0.88	GEI3_6d	0.59	GEI4_4b	0.92	GEI5_8	0.84
GEI1_9b	0.91	GEI2_8c	0.80	GEI3_6e	0.87	GEI4_4c	0.87	GEI5_9	0.72
GEI1_10a	0.57	GEI2_8d	0.80	GEI3_6f	0.80	GEI4_4d	0.91		
GEI1_10b	**0.42**	GEI2_8e	0.79			GEI4_5a	0.92		
GEI1_10c	0.76	GEI2_9	0.77			GEI4_5b	0.77		
GEI1_11	0.73	GEI2_10a	0.89			GEI4_5c	0.89		
GEI1_12	0.70	GEI2_10b	0.87			GEI4_5d	0.93		
GEI1_13	0.79	GEI2_10c	0.83			GEI4_6	0.88		
		GEI2_10d	0.79			GEI4_7	0.84		
		GEI2_11a	0.85			GEI4_8	0.94		
		GEI2_11c	0.82			GEI4_10	0.81		
		GEI2_11d	0.82						

Note

Bolded and shaded are the loadings of items that were dropped because they loaded poorly (<0.5) on domain factors.

For peer review only

Appendix 5. Heterogeneity testing

1. Heterogeneity testing by domains and countries (Fixed effect)

	Policy	Programs	Advocacy	Research	Civil society	Overall	Between sub-groups test
**Ethiopia**							
Statistic	0.37	0.58	1.44	0.96	0.46	5.74	1.94
P-Value	1.00	0.99	0.92	0.97	0.99	1.00	0.75
I-squared	0.00%	0.00%	0.00%	0.00%	0.00%	0.00%	
**Kenya**							
Statistic	0.52	1.47	0.95	0.80	0.20	4.53	0.59
P-Value	0.99	0.92	0.97	0.98	1.00	1.00	0.96
I-squared	0.00%	0.00%	0.00%	0.00%	0.00%	0.00%	
**Nigeria**							
Statistic	1.54	1.34	1.20	0.91	0.99	6.58	0.60
P-Value	0.91	0.93	0.95	0.97	0.96	1.00	0.96
I-squared	0.00%	0.00%	0.00%	0.00%	0.00%	0.00%	
**Rwanda**							
Statistic	3.33	11.24	3.78	4.68	3.24	29.93	3.66
P-Value	0.65	0.05	0.58	0.46	0.66	0.42	0.45
I-squared	0.00%	**55.50%**	0.00%	0.00%	0.00%	3.10%	
**Senegal**							
Statistic	0.33	2.00	1.29	0.92	1.57	8.23	2.12
P-Value	1.00	0.85	0.94	0.97	0.90	1.00	0.71
I-squared	0.00%	0.00%	0.00%	0.00%	0.00%	0.00%	
**Tanzania**							
Statistic	1.17	1.16	1.22	1.28	3.15	10.13	2.14
P-Value	0.95	0.95	0.94	0.94	0.68	1.00	0.71
I-squared	0.00%	0.00%	0.00%	0.00%	0.00%	0.00%	

2. Heterogeneity testing by domains and countries (Random effect)

		Policy	Programs	Advocacy	Research	CSO	Overall
**Ethiopia**							
	Statistic	0.37	0.58	1.44	0.96	0.46	5.74
	P	1.00	0.99	0.92	0.97	0.99	1.00
	I-squared	0.00%	0.00%	0.00%	0.00%	0.00%	0.00%
	Tau-squared	0.00	0.00	0.00	0.00	0.00	0.00
**Kenya**							
	Statistic	0.52	1.47	0.95	0.80	0.20	4.53
	P	0.99	0.92	0.97	0.98	1.00	1.00
	I-squared	0.00%	0.00%	0.00%	0.00%	0.00%	0.00%
	Tau-squared	0.00	0.00	0.00	0.00	0.00	0.00
**Nigeria**							
	Statistic	1.54	1.34	1.20	0.91	0.99	6.58
	P	0.91	0.93	0.95	0.97	0.96	1.00
	I-squared	0.00%	0.00%	0.00%	0.00%	0.00%	0.00%
	Tau-squared	0.00	0.00	0.00	0.00	0.00	0.00
**Rwanda**							
	Statistic	3.33	11.24	3.78	4.68	3.24	29.93
	P	0.65	0.05	0.58	0.46	0.66	0.42
	I-squared	0.00%	**55.50%**	0.00%	0.00%	0.00%	3.10%
	Tau-squared	0.00	**0.46**	0.00	0.00	0.00	0.01
**Senegal**							
	Statistic	0.33	2.00	1.29	0.92	1.57	8.23
	P	1.00	0.85	0.94	0.97	0.90	1.00
	I-squared	0.00%	0.00%	0.00%	0.00%	0.00%	0.00%
	Tau-squared	0.00	0.00	0.00	0.00	0.00	0.00
**Tanzania**							
	Statistic	1.17	1.16	1.22	1.28	3.15	10.13
	P	0.95	0.95	0.94	0.94	0.68	1.00
	I-squared	0.00%	0.00%	0.00%	0.00%	0.00%	0.00%
	Tau-squared	0.00	0.00	0.00	0.00	0.00	0.00

Appendix 6. Percentages of “don’t know” by question/item

Variable	Ethiopia		Kenya		Nigeria		Rwanda		Senegal		Tanzania	
	n	%	n	%	n	%	n	%	n	%	n	%
FP 1.1 Prominence of Family Planning (FP): Extent to which there is a permanent												
Don't know	0	0	0	0	0	0	0	0	0	0	1	2.6
FP 1.1 Prominence of Family Planning (FP): Extent to which there is a permanent												
Don't know	0	0	0	0	0	0	0	0	0	0	1	2.6
FP 1.2 Policy on Fertility Reduction and FP: Extent to which government policy e												
FP 1.3 National FP Strategic Plan: Extent to which there is a current, comprehen												
FP 1.4 Policy on Age at Marriage: Extent to which legal age at marriage for fema												
Don't know	0	0	0	0	0	0	0	0	0	0	1	2.6
FP 1.5 Evidence-Based Policymaking: Extent to which the appropriate ministry act												
FP 1.6 Monitoring and Evaluation of National Policies/Plans/Strategies: Extent t												
Don't know	0	0	0	0	0	0	0	0	0	0	3	7.9
FP 1.7 Inclusion of Nongovernmental and Cross-sectoral Actors in Policy/Strategy												
Don't know	0	0	0	0	0	0	0	0	0	0	1	2.6

For peer review only

Note:

- For readability purpose, complete wording for questions/variables has been provided as Appendix 1.
- Only the percentages of respondents who responded “don’t know” to the respective question or sub-question was included in this table.
- Highlighted are values > 10%

FP 1.8 Financing for FP: Extent to which the government has established and appl												
Don't know	1	5.9	1	5.9	0	0	0	0	0	0	2	5.3
FP 1.9 Percent of In-country Funding of FP Budget: Extent to which the total fam												
Don't know	4	**23.5**	1	5.9	0	0	0	0	0	0	3	7.9
FP 1.10 Information/Communications Technology Infrastructure/Capacity:¬† Extent t												
Don't know	0	0	1	5.9	1	5.6	0	0	0	0	0	0
FP 1.11 Import Laws and Legal Regulations:¬†												
Don't know	0	0	5	**29.4**	1	5.6	0	0	0	0	2	5.3
Don't know	1	5.9	3	**17.6**	1	5.6	0	0	0	0	4	**12.1**
FP 1.12 Advertising of Contraceptives Allowed: Extent of freedom from restrictio												
Don't know	0	0	1	5.9	0	0	0	0	0	0	0	0
FP 1.13 Presence of Pro-Poor FP Policies: Extent to which policies specifically												
Don't know	0	0	0	0	0	0	0	0	0	0	1	2.6
FP 1.14 Political Support for FP: Extent to which elected officials in the count												
FP 2.1 Administrative Structure: Extent to which the administrative structure an												
Don't know	0	0	0	0	0	0	0	0	0	0	1	2.9
FP 2.2 Level of Program Leaders: Relative level of seniority of the director of												
Don't know	2	**13.3**	0	0	4	**25**	0	0	0	0	1	2.9

Note:

- For readability purpose, complete wording for questions/variables has been provided as Appendix 1.
- Only the percentages of respondents who responded “don’t know” to the respective question or sub-question was included in this table.
- Highlighted are values > 10%

For peer review only

FP. 2.3 Community-Based Distribution: Extent to which areas of the country not e												
Don't know	0	0	1	6.2	0	0	0	0	0	0	1	2.9
FP 2.4 Postpartum Program: Extent to which all postpartum women receive postpart												
Don't know	0	0	0	0	1	6.2	0	0	0	0	0	0
FP 2.5 Home-Visiting Workers: Extent of coverage of population by workers whose												
Don't know	0	0	1	6.2	1	6.2	0	0	0	0	1	2.9
FP 2.6 Availability and Accessibility of FP Commodities/Supplies and Services Pa												
FP 2.6.1	1	6.7	2	**12.5**	0	0	0	0	0	0	3	8.6
FP 2.6.2	0	0	1	6.2	0	0	0	0	0	0	1	3
FP 2.6.3	0	0	1	6.2	2	**13.3**	0	0	0	0	2	5.7
FP 2.6.4	0	0	1	6.2	0	0	0	0	0	0	1	2.9
FP 2.7 Availability and Accessibility of FP Commodities/Supplies and Services Pa												
FP 2.7.1	0	0	0	0	0	0	0	0	0	0	3	8.6
FP 2.7.2	0	0	0	0	0	0	0	0	0	0	3	8.6
FP 2.7.3	0	0	0	0	0	0	0	0	1	**14.3**	3	8.6
FP 2.7.4	0	0	0	0	1	6.2	0	0	0	0	3	8.6
FP 2.7.5	0	0	0	0	0	0	0	0	1	**14.3**	3	8.8
FP 2.7.6	0	0	0	0	3	**18.8**	0	0	0	0	4	**11.4**
FP 2.7.7	0	0	1	6.2	1	6.2	0	0	0	0	4	**11.4**

Note:

- For readability purpose, complete wording for questions/variables has been provided as Appendix 1.
- Only the percentages of respondents who responded “don’t know” to the respective question or sub-question was included in this table.
- Highlighted are values > 10%

For peer review only

FP 2.8 Availability and Accessibility of FP Commodities/Supplies and Services Pa												
FP 2.8.1	0	0	2	**12.5**	1	6.2	0	0	0	0	4	**11.4**
FP 2.8.2	0	0	2	**12.5**	1	6.2	0	0	0	0	4	**11.4**
FP 2.8.3	0	0	0	0	0	0	0	0	0	0	3	8.6
FP 2.8.4	0	0	0	0	0	0	0	0	0	0	3	8.6
FP 2.9 FP Service Quality: Please rate the general quality of family planning se												
FP 2.10 Incentives and Disincentives: Extent to which monetary or other incentiv												
Don't know	0	0	1	6.2	1	6.2	0	0	0	0	3	8.6
FP 2.11 Adolescent Programs: Extent to which information, services and activitie												
Don't know	0	0	1	6.2	0	0	0	0	0	0	0	0
FP 2.12 Training Program: Extent to which training programs, for each category o												
Don't know	0	0	1	6.2	0	0	0	0	0	0	1	2.9
FP 2.13 Personnel Carry Out Assigned Tasks: Extent to which all categories of fa												
Don't know	0	0	1	6.2	0	0	0	0	0	0	2	5.7
FP 2.14 Logistics and Transport: Extent to which the logistics and transport sys												
Don't know	0	0	0	0	0	0	0	0	0	0	3	8.6
FP 2.15 Supervision System: Extent to which the system of supervision at all lev												
Don't know	0	0	0	0	0	0	0	0	0	0	1	2.9

Note:

- For readability purpose, complete wording for questions/variables has been provided as Appendix 1.
- Only the percentages of respondents who responded “don’t know” to the respective question or sub-question was included in this table.
- Highlighted are values > 10%

For peer review only

For peer review only - http://bmjopen.bmj.com/site/about/guidelines.xhtml

FP 2.16 Involvement of Other Ministries and Public Agencies: Extent to which oth												
Don't know	0	0	0	0	0	0	0	0	0	0	1	2.9
FP 2.17 Involvement of Private-Sector Agencies and Groups: Extent to which priva												
FP 2.18 Mass Media for Information Education Communication: Frequency and extent												
FP 2.19 Cultural and Social Norms Barriers: Extent to which cultural and social												
FP 2.20 Youth: Extent to which the government supports youth-led FP initiatives												
FP 2.21 FP Effort Influences ‚Äì Forces Affecting the FP Program: Forces affecting												
FP 2.21.1	0	0	0	0	0	0	0	0	0	0	2	5.9
FP 2.21.2	0	0	0	0	0	0	0	0	0	0	1	3
FP 2.21.3	0	0	0	0	0	0	0	0	0	0	2	5.9
FP 2.21.4	0	0	1	6.2	0	0	0	0	0	0	1	2.9
FP 2.21.5	0	0	0	0	0	0	0	0	0	0	2	5.9
FP 3.1 Influence of FP Leaders/Champions: Extent to which the country has one or												
Don't know	0	0	0	0	0	0	0	0	0	0	1	2.9
FP 3.2 Statement by Leaders: Extent to which the head of government, as well as												
FP 3.3 Media Coverage for FP: Extent to and frequency with which national media												

Note:

- For readability purpose, complete wording for questions/variables has been provided as Appendix 1.
- Only the percentages of respondents who responded "don't know" to the respective question or sub-question was included in this table.
- Highlighted are values > 10%

For peer review only

FP 3.4 FP Effort Influences - Justification: How important is each of the follow												
FP 3.4.1	0	0	0	0	0	0	0	0	0	0	1	2.9
FP 3.4.2	0	0	1	6.2	0	0	0	0	0	0	0	0
FP 3.4.3	0	0	0	0	0	0	0	0	0	0	1	2.9
FP 3.4.4	0	0	1	6.2	0	0	0	0	0	0	3	8.8
FP 3.4.5	0	0	1	6.7	0	0	0	0	0	0	3	9.4
FP 3.4.6	0	0	1	6.2	0	0	0	0	0	0	1	2.9
FP 3.4.7	0	0	0	0	0	0	0	0	0	0	1	2.9
FP 3.5 FP Effort Influences ‚Äì Special Populations: To what extent does the famil												
Don't know	0	0	2	**12.5**	0	0	0	0	0	0	1	2.9
FP 3.6 Social Marketing: Extent of coverage of the country by a social marketing												
Don't know	0	0	2	**12.5**	2	**13.3**	0	0	0	0	0	0
FP 4.1 Strength of Observatory/Stakeholder/Technical Working Group for FP: Exten												
Don't know	0	0	1	6.2	0	0	0	0	0	0	1	3.1
FP 4.2 Education/Research Strategy: Extent to which the national FP plan and/or												
Don't know	0	0	0	0	1	6.7	0	0	0	0	1	3.1
FP 4.3 Data Gathering Partners:												
FP 4.3.1	0	0	0	0	1	6.7	0	0	0	0	0	0
FP 4.3.2	0	0	0	0	3	**20**	0	0	0	0	0	0

Note:

- For readability purpose, complete wording for questions/variables has been provided as Appendix 1.
- Only the percentages of respondents who responded “don’t know” to the respective question or sub-question was included in this table.
- Highlighted are values > 10%

For peer review only

FP 4.3.3	0	0	0	0	3	**20**	0	0	0	0	1	3.1
FP 4.4 Topical Data Gathering:												
FP 4.4.1	0	0	1	6.2	0	0	0	0	0	0	0	0
FP 4.4.2	0	0	0	0	1	6.7	0	0	0	0	0	0
FP 4.4.3	0	0	0	0	1	6.7	0	0	0	0	0	0
FP 4.4.4	0	0	0	0	2	**13.3**	0	0	0	0	2	6.2
FP 4.5 Quality/Coverage of Data: Extent to which current research/data is sex an												
FP 4.5.1	0	0	0	0	1	6.7	0	0	0	0	1	3.1
FP 4.5.2	0	0	0	0	0	0	0	0	0	0	2	6.2
FP 4.5.3	0	0	0	0	1	6.7	0	0	0	0	3	9.4
FP 4.5.4	0	0	0	0	1	6.7	0	0	0	0	2	6.2
FP 4.6 Record-Keeping: Extent to which systems for client recordkeeping, clinic												
Don't know	0	0	0	0	1	6.7	0	0	0	0	1	3.1
FP 4.7 Quality Research Institutions: Extent to which the country supports and s												
Don't know	0	0	1	6.2	1	6.7	0	0	0	0	1	3.1
FP 4.8 Evaluation: Extent to which program statistics, surveys, and small studie												
Don't know	0	0	1	6.2	2	**13.3**	0	0	0	0	4	**12.5**
FP 4.9 Management‚Äôs Use of Evaluation Findings: Extent to which local-level prog												
Don't know	0	0	1	6.2	1	6.7	0	0	0	0	1	3.1

Note:

- For readability purpose, complete wording for questions/variables has been provided as Appendix 1.
- Only the percentages of respondents who responded “don’t know” to the respective question or sub-question was included in this table.
- Highlighted are values > 10%

For peer review only

FP 4.10 Ministerial Use of Evaluation Findings: Extent to which the Ministry adm												
Don't know	0	0	1	6.2	1	6.7	0	0	0	0	1	3.1
FP 4.11 Dissemination of Information to Other Implementing Bodies: Extent to whi												
Don't know	0	0	1	6.2	0	0	0	0	0	0	1	3.1
FP 5.1 CSO Actor Power: Extent to which CSO actors are in strong placements with												
Don't know	0	0	0	0	0	0	0	0	0	0	1	3.2
FP 5.2 Budget Analysis as a CSO Tool: Extent to which CSOs utilize budget analys												
Don't know	1	6.7	1	6.2	0	0	0	0	0	0	1	3.2
FP 5.3 Facility-Based Service Delivery Support: Extent to which CSOs are involve												
FP 5.4 Community-Based Service Delivery Support: Extent to which CSOs participat												
FP 5.5 Mobile Outreach for Community-Based Service Delivery Support: Extent to w												
Don't know	0	0	0	0	0	0	0	0	0	0	1	3.2
FP 5.6 Social Franchising: Extent to which CSOs are involved in social franchisi												
Don't know	0	0	1	6.2	0	0	0	0	0	0	3	9.7
FP 5.7 mHealth: Extent to which CSOs are leveraging mHealth to utilize text mess												
Don't know	0	0	0	0	2	**13.3**	0	0	0	0	2	6.5

Note:

- For readability purpose, complete wording for questions/variables has been provided as Appendix 1.
- Only the percentages of respondents who responded “don’t know” to the respective question or sub-question was included in this table.
- Highlighted are values > 10%

For peer review only

FP 5.8 Human Rights and Quality of Care: Extent to which CSOs advocate for and s												
FP 5.9 Social and Behavior Change (SBC): Extent to which CSOs support SBC interv												
Don't know	0	0	0	0	0	0	0	0	0	0	2	6.5
FP 5.10 Youth: Extent to which CSOs support FP policies, interventions and progr												
Don't know	0	0	0	0	0	0	0	0	0	0	1	3.2
FP 5.11 Programming for Men: Extent to which CSOs support FP policies, intervent												
Don't know	0	0	0	0	0	0	0	0	0	0	2	6.5
FP 5.12 Advocacy/Accountability: Extent to which CSOs support the training and c												
Don't know	0	0	0	0	0	0	0	0	0	0	1	3.2
FP 5.13 CSO-Led Assessment and Monitoring: Extent to which CSOs assess, monitor												
Don't know	0	0	0	0	0	0	0	0	0	0	3	9.7
FP 5.14 Forming Partnerships between CSOs: Extent to which CSOs have formed nati												
Don't know	0	0	0	0	0	0	0	0	0	0	2	6.5
MCH 1.1 Adequate Policy: Ministry of Health policies toward pregnancy and delive												
Don't know	0	0	0	0	0	0	0	0	1	5.9	0	0
Don't know	0	0	0	0	0	0	0	0	1	5.9	0	0
MCH 1.2 Universal Access: Extent to which there are constitutional and legal ent												

Note:

- For readability purpose, complete wording for questions/variables has been provided as Appendix 1.
- Only the percentages of respondents who responded “don’t know” to the respective question or sub-question was included in this table.
- Highlighted are values > 10%

For peer review only

For peer review only - http://bmjopen.bmj.com/site/about/guidelines.xhtml

Don't know	0	0	0	0	0	0	0	0	1	5.9	0	0
MCH 1.3 Multisector Involvement: Extent to which policies are developed through												
Don't know	0	0	0	0	0	0	0	0	1	5.9	0	0
MCH 1.4 Competent Service Providers: Extent to which policies are reasonable and												
Don't know	0	0	0	0	0	0	0	0	1	5.9	0	0
MCH 1.5 Abortion Legislation: Extent to which a favorable policy exists towards												
Don't know	0	0	1	5.6	0	0	0	0	1	5.9	2	**10.5**
MCH 1.6. Post-Abortion Care Legislation: Extent to which a favorable policy exis												
Don't know	0	0	4	**22.2**	0	0	0	0	1	5.9	2	**10.5**
MCH 1.7 Policy Reviews and Updates: Extent to which policies are regularly revie												
Don't know	0	0	2	**11.1**	0	0	0	0	1	5.9	0	0
MCH 1.8 High-Level Actor Placement: Extent and level to which the director of ma												
Don't know	0	0	4	**22.2**	0	0	0	0	1	5.9	1	5.3
MCH 1.9 Financing for MCH: Extent to which the government budgets for safe pregn												
Don't know	1	6.7	0	0	0	0	0	0	1	5.9	2	**10.5**
MCH 1.10 Affordability: Extent to which all MCH-related services and drugs are a												
Don't know	0	0	0	0	0	0	0	0	1	5.9	0	0

Note:

- For readability purpose, complete wording for questions/variables has been provided as Appendix 1.
- Only the percentages of respondents who responded "don't know" to the respective question or sub-question was included in this table.
- Highlighted are values > 10%

For peer review only

MCH 1.11 Private Sector: Extent to which the private sector (doctors, midwives,												
Don't know	0	0	0	0	0	0	0	0	1	5.9	1	5.3
MCH 1.12 MCH Department: Extent to which the Ministry of Health has a department												
Don't know	0	0	1	5.6	0	0	0	0	1	5.9	0	0
MCH 1.13 Potential Solutions: Extent to which the government recognizes potentia												
MCH 1.13.1	1	6.7	1	5.6	0	0	0	0	1	5.9	0	0
MCH 1.13.2	0	0	1	5.9	0	0	0	0	1	5.9	0	0
MCH 1.13.3	0	0	2	**11.1**	0	0	0	0	1	5.9	0	0
MCH 1.13.4	0	0	1	5.6	0	0	0	0	1	5.9	0	0
MCH 1.13.5	0	0	1	5.6	0	0	0	0	1	5.9	0	0
MCH 2.1 Obstetric Care Availability ‚Äì All Centers: Extent to which all primary h												
MCH 2.1.2	0	0	1	6.2	0	0	0	0	1	6.2	1	5.9
MCH 2.1.3	1	7.7	2	**12.5**	0	0	0	0	1	6.2	1	5.9
MCH 2.1.4	1	7.7	2	**12.5**	0	0	0	0	1	6.2	1	5.9
MCH 2.1.5	1	8.3	2	**12.5**	0	0	0	0	1	6.2	2	**11.8**
MCH 2.1.6	1	7.7	0	0	0	0	0	0	1	6.2	1	5.9
MCH 2.1.7	0	0	1	6.2	0	0	0	0	1	6.2	0	0
MCH 2.1.8	0	0	0	0	0	0	0	0	1	6.2	0	0
MCH 2.2 Obstetric Care Availability ‚Äì Secondary Healthcare Facilities: Extent to												
MCH 2.2.1	0	0	0	0	0	0	0	0	1	6.2	1	5.9

Note:

- For readability purpose, complete wording for questions/variables has been provided as Appendix 1.
- Only the percentages of respondents who responded “don’t know” to the respective question or sub-question was included in this table.
- Highlighted are values > 10%

For peer review only

MCH 2.2.2	1	8.3	0	0	0	0	0	0	1	6.2	3	**17.6**
MCH 2.2.3	0	0	1	6.2	0	0	0	0	1	6.2	1	6.7
MCH 2.3 Pregnancy-Related Services: Extent to which all pregnant women have adeq												
MCH 2.3.1	1	7.7	1	6.2	0	0	0	0	1	6.2	2	**11.8**
MCH 2.3.2	1	7.7	0	0	0	0	0	0	1	6.2	2	**11.8**
MCH 2.3.3	1	7.7	1	6.2	0	0	0	0	1	6.2	2	**11.8**
MCH 2.3.4	1	7.7	1	6.2	0	0	0	0	1	6.2	1	6.2
MCH 2.3.5	1	7.7	1	6.2	0	0	0	0	1	6.2	1	5.9
MCH 2.3.6	0	0	1	6.2	0	0	0	0	1	6.2	1	6.7
MCH 2.3.7	0	0	1	6.2	0	0	0	0	1	6.2	0	0
MCH 2.3.8	0	0	0	0	0	0	0	0	1	6.2	0	0
MCH 2.3.9	1	7.7	1	6.7	0	0	0	0	1	6.2	2	**12.5**
MCH 2.4 Ante-natal Care for Unmarried and HIV Positive Women: Extent to which, a												
MCH 2.4.1	0	0	1	6.2	1	**12.5**	0	0	1	6.2	3	**17.6**
MCH 2.4.2	1	7.7	1	6.2	1	**12.5**	0	0	1	6.2	3	**17.6**
MCH 2.4.3	0	0	2	**12.5**	0	0	0	0	1	6.2	3	**17.6**
MCH 2.4.4	0	0	1	6.7	0	0	0	0	1	6.2	1	5.9
MCH 2.5 Newborn Care: Extent to which, for newborn care, all infants whether del												
MCH 2.5.1	1	7.7	2	**12.5**	0	0	0	0	1	6.2	3	**17.6**
MCH 2.5.2	1	7.7	2	**12.5**	0	0	0	0	1	6.2	3	**17.6**
MCH 2.5.3	0	0	1	6.2	0	0	0	0	1	6.2	0	0
MCH 2.5.4	0	0	2	**12.5**	0	0	0	0	1	6.2	1	5.9

Note:

- For readability purpose, complete wording for questions/variables has been provided as Appendix 1.
- Only the percentages of respondents who responded "don't know" to the respective question or sub-question was included in this table.
- Highlighted are values > 10%

For peer review only

MCH 2.5.5	1	7.7	3	**18.8**	0	0	0	0	1	6.2	3	**17.6**
MCH 2.5.6	0	0	1	6.2	0	0	0	0	1	6.2	0	0
MCH 2.5.7	0	0	1	6.2	0	0	0	0	1	6.2	0	0
MCH 2.6 Family Planning ‚Äì All Centers: Extent to which there exists proper provi												
MCH 2.6.1	0	0	0	0	1	**12.5**	0	0	1	6.2	1	5.9
MCH 2.6.2	0	0	1	6.2	0	0	0	0	1	6.2	0	0
MCH 2.6.3	0	0	2	**12.5**	0	0	0	0	1	6.2	0	0
MCH 2.6.4	0	0	1	6.2	0	0	0	0	1	6.2	0	0
MCH 2.6.5	0	0	1	6.7	0	0	0	0	1	6.2	0	0
MCH 2.7 Family Planning ‚Äì District/Sub-county Hospitals: Extent to which there e												
MCH 2.7.1	1	7.7	1	6.2	1	**12.5**	0	0	1	6.2	1	5.9
MCH 2.7.2	1	7.7	1	6.7	0	0	0	0	1	6.2	0	0
MCH 2.7.3	0	0	1	6.2	0	0	0	0	1	6.2	0	0
MCH 2.7.4	0	0	2	**12.5**	0	0	0	0	1	6.2	0	0
MCH 2.7.5	0	0	1	6.2	1	**12.5**	0	0	1	6.2	2	**12.5**
MCH 2.7.6	0	0	2	**12.5**	1	**12.5**	0	0	1	6.2	2	**11.8**
MCH 2.7.7	0	0	1	6.2	0	0	0	0	1	6.2	0	0
MCH 2.8 Childhood Illness: Extent to which the WHO-recommended case management o												
MCH 2.8.1	0	0	1	6.7	1	**12.5**	0	0	1	6.2	1	5.9
MCH 2.8.2	0	0	1	6.2	0	0	0	0	1	6.7	1	5.9
MCH 3.1 Government Messages: Extent to which high officials in the government, i												

Note:

- For readability purpose, complete wording for questions/variables has been provided as Appendix 1.
- Only the percentages of respondents who responded “don’t know” to the respective question or sub-question was included in this table.
- Highlighted are values > 10%

For peer review only

Don't know	0	0	0	0	0	0	0	0	1	6.2	0	0
MCH 3.2 Mass Media: Extent to which the national program uses the mass media to												
Don't know	0	0	0	0	0	0	0	0	1	6.2	0	0
MCH 3.3 Community-Level Media: Extent to which community groups take part in sys												
Don't know	0	0	0	0	0	0	0	0	1	6.2	0	0
MCH 3.4 Educational Materials: Extent to which the appropriate ministry (MOH) su												
Don't know	1	7.7	0	0	0	0	0	0	1	6.2	0	0
MCH 3.5 Self-Review of Maternal Mortality Cases: Extent to which each health fac												
Don't know	0	0	1	8.3	0	0	0	0	1	6.2	1	6.7
MCH 3.6 Informing Policies with New Evidence: Extent to which each health facili												
Don't know	0	0	1	8.3	0	0	0	0	1	6.2	0	0
MCH 4.1 Strength of Observatory/Stakeholder/Technical Working Group for MCH: Ext												
MCH 4.1.1	0	0	0	0	1	**12.5**	0	0	1	6.2	0	0
MCH 4.1.2	0	0	1	8.3	0	0	0	0	1	6.2	1	7.7
MCH 4.1.3	0	0	0	0	0	0	0	0	1	6.2	1	7.1
MCH 4.2 MCH Research Strategy: Extent to which national documents include a comp												
Don't know	0	0	0	0	0	0	0	0	3	**18.8**	1	7.1
MCH 4.3 Data-Gathering Partners:												

Note:

- For readability purpose, complete wording for questions/variables has been provided as Appendix 1.
- Only the percentages of respondents who responded "don't know" to the respective question or sub-question was included in this table.
- Highlighted are values > 10%

For peer review only

MCH 4.3.1	0	0	1	8.3	0	0	0	0	1	6.2	1	7.1
MCH 4.3.2	0	0	2	**16.7**	0	0	0	0	1	6.2	1	7.1
MCH 4.3.3	0	0	1	9.1	1	**12.5**	0	0	1	6.2	1	7.7
MCH 4.4 Topical Research:												
MCH 4.4.1	0	0	2	**16.7**	1	**12.5**	0	0	1	6.2	1	7.1
MCH 4.4.2	0	0	1	9.1	1	**12.5**	0	0	1	6.2	1	7.1
MCH 4.4.3	0	0	1	8.3	1	**12.5**	0	0	1	6.2	1	7.1
MCH 4.4.4	0	0	4	**33.3**	1	**12.5**	0	0	1	6.2	1	7.1
MCH 4.5 Quality/Coverage of Data: Extent to which current research/data is disag												
MCH 4.5.1	0	0	2	**16.7**	0	0	0	0	1	6.2	1	7.1
MCH 4.5.2	0	0	1	8.3	0	0	0	0	1	6.2	1	7.1
MCH 4.5.3	0	0	2	**16.7**	0	0	0	0	1	6.2	1	7.1
MCH 4.5.4	0	0	2	**16.7**	0	0	0	0	1	6.2	2	**14.3**
MCH 4.5.5	0	0	3	**25**	0	0	0	0	1	6.2	2	**14.3**
MCH 4.6 Record-Keeping: Extent to which systems for client recordkeeping, clinic												
Don't know	0	0	1	8.3	0	0	0	0	1	6.2	1	7.1
MCH 4.7 Quality Research Institutions: Extent to which the country has the capac												
Don't know	0	0	1	8.3	0	0	0	0	1	6.2	1	7.1
MCH 4.8 Evaluation: Extent to which program statistics, surveys, and small studi												
Don't know	0	0	1	8.3	0	0	0	0	1	6.2	1	7.1

Note:

- For readability purpose, complete wording for questions/variables has been provided as Appendix 1.
- Only the percentages of respondents who responded "don't know" to the respective question or sub-question was included in this table.
- Highlighted are values > 10%

For peer review only

MCH 4.9 Management‚Äôs Use of Evaluation Findings: Extent to which local-level pro												
Don't know	0	0	1	8.3	0	0	0	0	1	6.2	2	**14.3**
MCH 4.10 Ministerial Use of Evaluation Findings: Extent to which the Ministry ad												
Don't know	0	0	1	8.3	0	0	0	0	1	6.2	1	7.1
MCH 4.11 Dissemination of Information to Other Implementing Bodies: Extent to wh												
Don't know	0	0	2	**16.7**	0	0	0	0	1	6.2	1	7.1
MCH 5.1 CSO Actor Power: Extent to which CSO actors are in strong placements wit												
Don't know	0	0	1	10	0	0	0	0	1	6.7	2	**14.3**
MCH 5.2 Budget Analysis as a CSO Tool: Extent to which CSOs utilize budget analy												
Don't know	2	**20**	1	10	0	0	0	0	1	6.7	3	**21.4**
MCH 5.3 Facility-Based Service Delivery Support: Extent to which CSOs are involv												
Don't know	0	0	0	0	0	0	0	0	1	6.7	1	7.1
MCH 5.4 Community-Based Service Delivery Support: Extent to which CSOs participa												
Don't know	0	0	0	0	0	0	0	0	1	6.7	1	7.1
MCH 5.5 Mobile Outreach for Community-Based Service Delivery Support: Extent to												
Don't know	0	0	0	0	0	0	0	0	1	6.7	1	7.1
MCH 5.6 Social Franchising: Extent to which CSOs are involved with social franch												

Note:

- For readability purpose, complete wording for questions/variables has been provided as Appendix 1.
- Only the percentages of respondents who responded “don’t know” to the respective question or sub-question was included in this table.
- Highlighted are values > 10%

For peer review only

Don't know	0	0	0	0	0	0	0	0	1	6.7	2	**14.3**
MCH 5.7 mHealth: Extent to which CSOs are leveraging mHealth to utilize text mes												
Don't know	0	0	1	10	0	0	0	0	1	6.7	2	**14.3**
MCH 5.8 Human Rights and Quality of Care: Extent to which CSOs advocate for and												
Don't know	0	0	1	10	0	0	0	0	1	6.7	2	**14.3**
MCH 5.9 Social and Behavior Change (SBC): Extent to which CSOs support SBC inter												
Don't know	0	0	1	10	0	0	0	0	1	6.7	1	7.1
MCH 5.10 Advocacy/Accountability: Extent to which CSOs support the training and												
Don't know	0	0	0	0	0	0	0	0	1	6.7	1	7.1
MCH 5.11 CSO-Led Assessment and Monitoring: Extent to which CSOs assess, monitor												
Don't know	0	0	0	0	0	0	0	0	1	6.7	1	7.1
MCH 5.12 Forming Partnerships between CSOs: Extent to which CSOs have formed nat												
Don't know	1	10	1	10	0	0	0	0	1	6.7	2	**14.3**
ED 1.1 Quality: Extent to which there are national plans/strategies/programs to												
ED 1.2 Disadvantaged Groups:												
Don't know	3	**20**	0	0	2	**18.2**	0	0	0	0	0	0
Don't know	1	6.7	1	**20**	0	0	0	0	0	0	0	0
ED 1.3 Teachers: Extent to which teacher recruitment, development and management												

Note:

- For readability purpose, complete wording for questions/variables has been provided as Appendix 1.
- Only the percentages of respondents who responded “don’t know” to the respective question or sub-question was included in this table.
- Highlighted are values > 10%

For peer review only

ED 1.4 Teachers,Äô Accountability: Extent to which national plans include specific												
Don't know	1	6.7	0	0	0	0	0	0	0	0	0	0
ED 1.5 Teacher Quality: Extent to which education policies/strategies/programs i												
Don't know	1	6.7	0	0	0	0	0	0	0	0	0	0
ED 1.6 Collaboration in Education: Extent to which the policy makes provisions t												
ED 1.7 Gender Parity SDG: Extent to which national policies/plans/strategies con												
Don't know	1	6.7	0	0	0	0	0	0	0	0	0	0
ED 1.8 Education Financing: Extent to which new education plans include financin												
Don't know	2	**13.3**	0	0	0	0	0	0	0	0	0	0
ED 1.9 Gender Parity Planning: Extent to which there are national plans/strategi												
ED 1.9.1	0	0	0	0	0	0	1	**14.3**	0	0	0	0
ED 1.9.2	0	0	0	0	0	0	1	**14.3**	0	0	0	0
ED 1.9.3	0	0	0	0	3	**27.3**	0	0	0	0	0	0
ED 1.10 Adult Illiteracy: Extent to which national policies/plans/strategies aim												
ED 1.10.1	2	**13.3**	0	0	1	8.3	0	0	0	0	0	0
ED 1.10.2	2	**13.3**	0	0	1	8.3	0	0	0	0	0	0
ED 1.11 Out-of-School Children: Extent to which policies address the plight of o												
Don't know	1	6.7	0	0	0	0	0	0	0	0	0	0

Note:

- For readability purpose, complete wording for questions/variables has been provided as Appendix 1.
- Only the percentages of respondents who responded "don't know" to the respective question or sub-question was included in this table.
- Highlighted are values > 10%

For peer review only

ED 1.12 Technology Integration: Extent to which strategies have been developed a												
ED 1.13. Technology Literacy: Extent to which resources have been allocated to i												
ED 2.1 Teacher Deployment: Extent to which strategies/plans are used to ensure t												
Don't know	2	**13.3**	0	0	0	0	0	0	0	0	0	0
ED 2.2 Teacher Quality: Extent to which teacher quality is recognized as a key f												
Don't know	0	0	1	**20**	0	0	0	0	0	0	0	0
ED 2.3 Gender Parity: ¬†Extent to which resources have been utilized to improve e												
Don't know	1	6.7	0	0	0	0	0	0	0	0	0	0
ED 2.4 Parental Involvement in Education: Extent to which parents are involved i												
ED 2.5 Standardized Assessments: Extent to which all schools utilize standardize												
Don't know	2	**13.3**	0	0	0	0	0	0	0	0	0	0
ED 2.6. Use of Assessment to Inform Progress: Extent to which adjustments to sch												
Don't know	2	**13.3**	0	0	1	8.3	0	0	0	0	0	0
ED 3.1 Equality: Extent to which equity and equality in education have been high												
ED 3.2 Quality: Extent to which quality of education takes part in advocacy-rela												
Don't know	1	7.1	0	0	0	0	0	0	0	0	0	0

Note:

- For readability purpose, complete wording for questions/variables has been provided as Appendix 1.
- Only the percentages of respondents who responded "don't know" to the respective question or sub-question was included in this table.
- Highlighted are values > 10%

For peer review only

ED 3.3 Teachers‚Äô Obstacles: Extent to which the needs of teachers have been high												
ED 3.4 Institutions‚Äô Obstacles: Extent to which the challenges of educational in												
ED 4.1 Strength of Observatory/Stakeholder/Technical Working Group for ED: Exten												
ED 4.1.1	2	**14.3**	1	**20**	0	0	0	0	0	0	0	0
ED 4.1.2	2	**16.7**	0	0	1	8.3	0	0	0	0	0	0
ED 4.1.3	2	**15.4**	0	0	0	0	0	0	0	0	0	0
ED 4.2 Education/Research Strategy: Extent to which the national education plan												
Don't know	1	7.1	0	0	1	8.3	0	0	0	0	0	0
ED 4.3 Data Gathering Partners:												
ED 4.3.1	1	7.1	0	0	0	0	0	0	0	0	0	0
ED 4.3.2	1	7.1	0	0	1	9.1	0	0	0	0	0	0
ED 4.3.3	0	0	0	0	1	8.3	0	0	0	0	0	0
ED 4.4 Topical Research:												
ED 4.4.1	0	0	0	0	1	8.3	0	0	0	0	0	0
ED 4.4.2	0	0	0	0	1	8.3	0	0	0	0	0	0
ED 4.4.3	0	0	0	0	1	9.1	0	0	0	0	0	0
ED 4.4.4	0	0	1	**20**	1	8.3	0	0	0	0	0	0
ED 4.5 Quality/Coverage of Data: Extent to which current research/data is sex an												
ED 4.5.1	2	**14.3**	0	0	0	0	0	0	0	0	0	0

Note:

- For readability purpose, complete wording for questions/variables has been provided as Appendix 1.
- Only the percentages of respondents who responded “don’t know” to the respective question or sub-question was included in this table.
- Highlighted are values > 10%

For peer review only

ED 4.5.2	1	7.1	0	0	0	0	0	0	0	0	0	0
ED 4.5.3	1	7.1	0	0	0	0	0	0	0	0	0	0
ED 4.5.4	1	7.1	0	0	0	0	0	0	0	0	0	0
ED 4.6 Record-Keeping: Extent to which systems for education-related recordkeepi												
Don't know	1	7.1	0	0	1	8.3	0	0	0	0	0	0
ED 4.7 Quality Research Institutions: Extent to which the country has the capaci												
Don't know	1	7.1	1	**20**	1	8.3	0	0	0	0	0	0
ED 4.8 Evaluation: Extent to which education program statistics, surveys, and sm												
Don't know	0	0	0	0	1	8.3	0	0	0	0	0	0
ED 4.9 Management‚Äôs Use of Evaluation Findings: Extent to which local-level educ												
Don't know	1	7.1	0	0	1	8.3	0	0	0	0	0	0
ED 4.10 Ministerial Use of Evaluation Findings: Extent to which the relevant min												
Don't know	4	**28.6**	0	0	1	8.3	0	0	0	0	0	0
ED 4.11 Dissemination of Information to Other Implementing Bodies:												
ED 5.1 CSO Actor Power: Extent to which CSO actors are in strong placements with												
Don't know	1	7.1	1	**20**	1	8.3	0	0	0	0	0	0
ED 5.2 Budget Analysis as a CSO Tool: Extent to which CSOs utilize budget analys												
Don't know	5	**35.7**	2	**40**	2	**16.7**	0	0	0	0	0	0

Note:

- For readability purpose, complete wording for questions/variables has been provided as Appendix 1.
- Only the percentages of respondents who responded “don’t know” to the respective question or sub-question was included in this table.
- Highlighted are values > 10%

For peer review only

ED 5.3 Community inclusion: Extent to which CSOs are involved in efforts to expl												
Don't know	0	0	0	0	2	**16.7**	0	0	0	0	0	0
ED 5.4 Community Involvement in Education: Extent to which CSOs participate in b												
Don't know	0	0	0	0	1	8.3	0	0	0	0	0	0
ED 5.5 Social Franchising: Extent to which CSOs are involved with social franchi												
Don't know	0	0	1	**20**	1	8.3	0	0	0	0	0	0
ED 5.6 Technology: Extent to which CSOs are leveraging information communication												
Don't know	0	0	1	**20**	1	8.3	0	0	0	0	0	0
ED 5.7 Human Rights and Access to/Quality of Education: Extent to which CSOs adv												
Don't know	0	0	0	0	2	**16.7**	0	0	0	0	0	0
ED 5.8 Social and Behavior Change (SBC): Extent to which CSOs support SBC interv												
Don't know	0	0	0	0	2	**16.7**	0	0	0	0	0	0
ED 5.9 Advocacy/Accountability: Extent to which CSOs support the training and ca												
Don't know	0	0	1	**20**	2	**16.7**	0	0	0	0	0	0
ED 5.10 CSO-Led Assessment and Monitoring: Extent to which CSOs assess, monitor												
Don't know	1	7.1	1	**20**	2	**16.7**	0	0	1	**11.1**	0	0
ED 5.11 Forming Partnerships between CSOs: Extent to which CSOs have formed nati												

Note:

- For readability purpose, complete wording for questions/variables has been provided as Appendix 1.
- Only the percentages of respondents who responded “don’t know” to the respective question or sub-question was included in this table.
- Highlighted are values > 10%

For peer review only

Don't know	1	7.1	1	**20**	2	**16.7**	0	0	0	0	0	0
WE 1.1 Gender Equality Policy: Extent to which gender equality is considered/int												
WE 1.2 Level of commitment to the Convention on the Elimination of all Forms of												
WE 1.3 Equity in Marriage and Family Policies: Extent to which the principle of												
WE 1.4 Policy on Age at Marriage: Extent to which legal age at marriage for fema												
WE 1.5 Violence Against Women Measures: Level of regulations and measures on vio												
Don't know	0	0	0	0	0	0	1	6.2	0	0	0	0
WE 1.6 Female Genital Cutting/Mutilation: Level of regulation and measures on fe												
Don't know	0	0	0	0	0	0	1	6.2	0	0	0	0
WE 1.7 Women,Äôs Health Measures: Level of protocols for the protection of women,Äôs												
WE 1.8 Education Measures: Extent to which there are mechanisms to promote girls												
WE 1.9 Rights Measures: Level of enforcement of policies on women,Äôs rights.												
WE 1.10 Equitable Work: Extent to which there are policies on equitable working												
WE 1.11 Equitable Land/Asset Ownership: Extent to which there are policies to af												

Note:

- For readability purpose, complete wording for questions/variables has been provided as Appendix 1.
- Only the percentages of respondents who responded “don’t know” to the respective question or sub-question was included in this table.
- Highlighted are values > 10%

For peer review only

WE 1.12 Conflict, Women & Peacebuilding: Level of implementation of Security UN												
Don't know	3	**37.5**	1	10	0	0	0	0	0	0	1	7.7
WE 1.13 Gender Quotas in Politics: Existence, and level of implementation, of qu												
WE 1.14 Sustainable Development Goal 5: Level of commitment to fulfilling Sustai												
Don't know	1	**12.5**	0	0	0	0	0	0	0	0	0	0
WE 1.15 Customary Law Revision: Extent to which customary laws have been reviewe												
Don't know	0	0	0	0	0	0	4	**25**	0	0	0	0
WE 1.16 Costing for WE: Extent to which there is sufficient planning and allocat												
Don't know	2	**25**	2	**20**	0	0	0	0	0	0	0	0
WE 1.17 Staffing for WE: Extent to which:												
WE 1.18 Transparency and Accountability: Extent to which the government provides												
Don't know	1	**12.5**	0	0	1	**11.1**	0	0	0	0	0	0
WE 2.1 Gender Mainstreaming: Extent to which services or programs within and acr												
WE 2.2 Gender-Transformative Programming: Extent to which services or programs w												
Don't know	0	0	1	10	0	0	0	0	0	0	0	0
WE 2.3 Education: Extent to which education services or programs focus on girl‚Äôs												

Note:

- For readability purpose, complete wording for questions/variables has been provided as Appendix 1.
- Only the percentages of respondents who responded "don't know" to the respective question or sub-question was included in this table.
- Highlighted are values > 10%

For peer review only

WE 2.4. Dropouts: Extent to which there are programs to prevent female dropouts.												
WE 2.5 Technology Literacy: Extent to which technology literacy is promoted amon												
WE 2.6 Employment: Extent to which job-creation programs and labor market police												
WE 2.7 Women as Mothers and Workers: Extent to which the government makes possib												
WE 2.8 Family Planning for Empowerment: Extent to which FP services or programs												
Don't know	0	0	0	0	0	0	0	0	0	0	1	8.3
WE 2.9 Political and Public Representation: Extent of implementation of programs												
WE 2.10 Violence: Extent of fight against violence against women and children an												
WE 2.11 Child Marriage: Extent of law enforcement to eliminate child marriage.												
WE 2.12 Female Genital Mutilation/Cutting: Extent of law enforcement to eliminat												
Don't know	1	**12.5**	0	0	0	0	3	**18.8**	0	0	0	0
WE 3.1 Advocates: Extent to which advocates for girls,Äô and women,Äôs empowerment e												
Don't know	1	**12.5**	0	0	0	0	0	0	0	0	0	0
WE 3.2. Girls and Women: Extent to which the advocacy efforts in this area encom												
Don't know	1	**12.5**	0	0	0	0	0	0	0	0	0	0

Note:

- For readability purpose, complete wording for questions/variables has been provided as Appendix 1.
- Only the percentages of respondents who responded “don’t know” to the respective question or sub-question was included in this table.
- Highlighted are values > 10%

For peer review only

WE 3.3 Information Dissemination: Extent to which the government undertakes suff												
Don't know	1	**12.5**	0	0	0	0	0	0	0	0	0	0
WE 3.4 Leveraging Partnerships: Extent to which the government leverages partner												
Don't know	1	**12.5**	0	0	0	0	0	0	0	0	0	0
WE 3.5 Employment: Extent to which stakeholders call for laws, policies and prog												
Don't know	1	**12.5**	0	0	0	0	0	0	0	0	0	0
WE 3.6 Education: Extent to which a priority of advocates is to promote the fulf												
Don't know	1	**12.5**	0	0	0	0	0	0	0	0	0	0
WE 3.7 Violence: Extent to which advocates successfully engage with the governme												
Don't know	1	**12.5**	0	0	0	0	1	6.2	0	0	0	0
WE 4.1 Strength of Observatory/Stakeholder/Technical Working Group for WE: Exten												
WE 4.1.1	1	**12.5**	0	0	0	0	0	0	0	0	0	0
WE 4.1.2	2	**28.6**	0	0	0	0	0	0	0	0	0	0
WE 4.1.3	1	**14.3**	0	0	0	0	0	0	0	0	0	0
WE 4.2 Women,Äôs Rights Research Strategy: Extent to which the national¬†WE plan an												
Don't know	1	**12.5**	0	0	1	**11.1**	0	0	0	0	1	8.3
WE 4.3 Data Gathering Partners:												
WE 4.3.1	1	**12.5**	0	0	0	0	0	0	0	0	1	8.3

Note:

- For readability purpose, complete wording for questions/variables has been provided as Appendix 1.
- Only the percentages of respondents who responded “don’t know” to the respective question or sub-question was included in this table.
- Highlighted are values > 10%

For peer review only

WE 4.3.2	1	**14.3**	0	0	0	0	0	0	0	0	1	8.3
WE 4.3.3	2	**25**	0	0	0	0	0	0	0	0	1	8.3
WE 4.4 Topical Data Gathering:												
WE 4.4.1	1	**12.5**	0	0	0	0	0	0	0	0	1	8.3
WE 4.4.2	1	**12.5**	0	0	0	0	0	0	0	0	1	8.3
WE 4.4.3	1	**12.5**	0	0	1	**11.1**	0	0	0	0	1	9.1
WE 4.4.4	1	**12.5**	0	0	0	0	0	0	0	0	1	10
WE 4.5 Quality/Coverage of Data:												
WE 4.5.1	1	**12.5**	1	10	0	0	0	0	0	0	1	9.1
WE 4.5.2	1	**12.5**	1	10	0	0	0	0	0	0	1	8.3
WE 4.5.3	2	**25**	1	10	0	0	0	0	0	0	1	8.3
WE 4.6 Quality Research Institutions: Extent to which the country supports and s												
Don't know	2	**25**	1	10	0	0	0	0	0	0	2	**16.7**
WE 4.7 Evaluation: Extent to which program statistics, surveys, and small studie												
Don't know	3	**37.5**	1	10	0	0	0	0	0	0	1	8.3
WE 4.8 Management‚Äôs Use of Evaluation Findings: Extent to which local-level prog												
Don't know	3	**37.5**	0	0	0	0	0	0	0	0	1	8.3
WE 4.9 Ministerial Use of Evaluation Findings: Extent to which the Ministry admi												
Don't know	3	**37.5**	1	10	1	**11.1**	0	0	0	0	1	8.3
WE 4.10 Dissemination of Information to other Implementing Bodies: Extent to whi												

Note:

- For readability purpose, complete wording for questions/variables has been provided as Appendix 1.
- Only the percentages of respondents who responded “don’t know” to the respective question or sub-question was included in this table.
- Highlighted are values > 10%

For peer review only

Don't know	2	25	0	0	0	0	0	0	0	0	1	8.3
WE 5.1 CSO Actor Power: Extent to which WE civil society actors are in strong po												
Don't know	1	14.3	0	0	0	0	0	0	0	0	1	8.3
WE 5.2 Budget Analysis as a CSO Tool: Extent to which CSOs utilize budget analys												
Don't know	1	14.3	1	11.1	0	0	0	0	0	0	1	8.3
WE 5.3 Community-Based Service Delivery Support: Extent to which CSOs participat												
Don't know	1	14.3	0	0	0	0	0	0	0	0	1	8.3
WE 5.4 Human Rights and Quality of Implementation: Extent to which CSOs advocate												
Don't know	1	14.3	0	0	0	0	0	0	0	0	1	8.3
WE 5.5 Social and Behavior Change (SBC): Extent to which CSOs support SBC interv												
Don't know	1	14.3	0	0	0	0	0	0	0	0	1	8.3
WE 5.6 Youth: Extent to which CSOs promote gender empowerment through youth prog												
Don't know	1	14.3	0	0	1	11.1	0	0	0	0	1	8.3
WE 5.7 Programming for Men: Extent to which CSOs support WE policies, interventi												
Don't know	1	14.3	0	0	0	0	0	0	0	0	1	8.3
WE 5.8 Advocacy/Accountability: Extent to which CSOs support the training and ca												
Don't know	1	14.3	0	0	0	0	0	0	0	0	1	8.3

Note:

- For readability purpose, complete wording for questions/variables has been provided as Appendix 1.
- Only the percentages of respondents who responded “don’t know” to the respective question or sub-question was included in this table.
- Highlighted are values > 10%

For peer review only

WE 5.9 CSO-Led Assessment and Monitoring: Extent to which CSOs assess, monitor a												
Don't know	1	**14.3**	0	0	0	0	0	0	0	0	1	8.3
WE 5.10 Forming Partnerships between CSOs: Extent to which CSOs have formed nati												
Don't know	1	**14.3**	0	0	0	0	0	0	0	0	1	8.3
LM 1.1 Global Economic Integration: Extent to which the country aims to be integ												
LM 1.2 Regional Economic Integration: Extent to which the country aims to be int												
LM 1.3 Technology: Extent to which the government has invested in infrastructure												
LM 1.4 Government Support: Extent to which policies and plans are in place that												
Don't know	0	0	1	7.7	0	0	0	0	0	0	0	0
LM 1.5 Unemployment: Extent to which policies account for and address unemployme												
LM 1.6 Disadvantaged/Vulnerable Groups: Extent to which policies exist, and are												
LM 1.7 Climate Change and Environmental Sustainability: Extent to which the agri												
LM 1.8 Urbanization: ¬†Extent to which policies and planning anticipate and are p												
Don't know	1	**11.1**	0	0	0	0	0	0	0	0	0	0
LM 1.9 Service-Led Economy: Extent to which policies and framework are in place												

Note:

- For readability purpose, complete wording for questions/variables has been provided as Appendix 1.
- Only the percentages of respondents who responded “don’t know” to the respective question or sub-question was included in this table.
- Highlighted are values > 10%

For peer review only

LM 1.10 Corporate Social Responsibility: Extent to which national legislation in												
Don't know	2	**22.2**	0	0	0	0	1	**11.1**	0	0	0	0
LM 1.11 Targeted Labor Market Investments: Extent to which the government has ta												
LM 1.12 Self-Employment: Extent to which policies support raising the returns to												
LM 1.13 Microcredit: Extent to which the government enables access to microcredi												
Don't know	0	0	0	0	0	0	1	**11.1**	0	0	0	0
LM 1.14 Savings: Extent to which the government supports the creation of accessi												
LM 1.15 Skill Acquisition: Extent to which policies exist, and are effectively i												
LM 2.1 Unemployment Reduction: Extent to which there are efforts toward developi												
Don't know	0	0	1	7.7	0	0	0	0	0	0	0	0
LM 2.2. Youth Unemployment: Extent to which labor market programs have effective												
Don't know	0	0	1	7.7	0	0	0	0	0	0	0	0
LM 2.3 Access to Microcredit: Extent to which national and regional funds have b												
Don't know	0	0	1	7.7	0	0	0	0	0	0	0	0
LM 2.4 Harnessing Youth Special Capacities: Extent to which programs and service												
Don't know	0	0	0	0	0	0	1	**11.1**	0	0	0	0

Note:

- For readability purpose, complete wording for questions/variables has been provided as Appendix 1.
- Only the percentages of respondents who responded “don’t know” to the respective question or sub-question was included in this table.
- Highlighted are values > 10%

For peer review only

LM 2.5 Early Labor Opportunities: Extent to which programs and government legisl												
LM 2.6 Youth Entrance into Labor: Extent of the promotion of youth volunteer and												
LM 2.7 Entrepreneurial Ventures: Extent to which people have enhanced access to												
LM 2.8 Resources for Youth Entrepreneurship: Extent to which entrepreneurial ven												
Don't know	0	0	0	0	0	0	1	**11.1**	0	0	0	0
LM 2.9 Vocational Opportunities: Extent to which vocational training opportuniti												
LM 2.10 Education and Labor: Extent to which improvements have been made to impr												
LM 2.11 Women-Friendly Labor: Extent to which workplaces in the private and publ												
LM 2.12 Job Security:												
Don't know	0	0	0	0	1	**12.5**	0	0	0	0	0	0
LM 2.13 Minimum Wage: Extent to which a minimum wage is applied to the ‚Äòtypical‚Äô												
LM 2.14 Labor Market Programs: Extent to which labor market programs have effect												
Don't know	1	**11.1**	0	0	0	0	0	0	0	0	0	0
LM 2.15 Informality in the Labor Market: Extent to which the programs are implem												
LM 3.1 Gender Equality in the Labor Market: Extent to which groups and individua												

Note:

- For readability purpose, complete wording for questions/variables has been provided as Appendix 1.
- Only the percentages of respondents who responded “don’t know” to the respective question or sub-question was included in this table.
- Highlighted are values > 10%

For peer review only

LM 3.2 Youth Equality in the Labor Market: Extent to which individuals and group												
Don't know	1	**11.1**	0	0	0	0	0	0	0	0	0	0
LM 3.3 Minimum Wage Advocacy: Extent to which advocacy groups are successful in												
Don't know	0	0	1	7.7	0	0	0	0	0	0	0	0
LM 3.4 Government Advocacy: Extent to which members of the government advocates												
Don't know	0	0	1	7.7	0	0	0	0	0	0	0	0
LM 3.5 Protections: Extent to which advocacy exists for job protection, and soci												
Don't know	0	0	1	7.7	0	0	0	0	0	0	0	0
LM 4.1 Strength of Observatory/Stakeholder/Technical Working Group for the LM: E												
LM 4.1.1	1	**11.1**	2	**15.4**	0	0	0	0	0	0	0	0
LM 4.1.2	1	**11.1**	2	**15.4**	0	0	0	0	0	0	0	0
LM 4.1.3	1	**11.1**	3	**23.1**	0	0	0	0	0	0	0	0
LM 4.2 Labor Market Research Strategy: Extent to which the national documents in												
Don't know	0	0	3	**23.1**	0	0	0	0	0	0	0	0
LM 4.3 Labor Market Analysis Partners:												
LM 4.3.1	0	0	2	**15.4**	0	0	0	0	0	0	0	0
LM 4.3.2	0	0	2	**15.4**	0	0	0	0	0	0	0	0
LM 4.3.3	0	0	2	**15.4**	1	**12.5**	0	0	0	0	0	0
LM 4.4 Areas of Research in the Labor Market:												

Note:

- For readability purpose, complete wording for questions/variables has been provided as Appendix 1.
- Only the percentages of respondents who responded “don’t know” to the respective question or sub-question was included in this table.
- Highlighted are values > 10%

For peer review only

LM 4.4.1	0	0	1	7.7	1	**12.5**	0	0	0	0	0	0
LM 4.4.2	0	0	1	7.7	1	**12.5**	0	0	0	0	0	0
LM 4.4.3	0	0	1	7.7	1	**12.5**	0	0	0	0	0	0
LM 4.4.4	0	0	2	**15.4**	1	**12.5**	0	0	0	0	0	0
LM 4.5 Quality/Coverage of Data:												
LM 4.5.1	0	0	2	**15.4**	1	**12.5**	0	0	0	0	0	0
LM 4.5.2	0	0	2	**15.4**	1	**12.5**	0	0	0	0	0	0
LM 4.5.3	0	0	2	**15.4**	1	**12.5**	0	0	0	0	0	0
LM 4.6 Record-Keeping: Extent to which systems for labor market recordkeeping, r												
Don't know	0	0	2	**15.4**	1	**12.5**	0	0	0	0	0	0
LM 4.7 Quality Research Institutions: Extent to which the country has the capaci												
Don't know	0	0	2	**15.4**	1	**12.5**	0	0	0	0	0	0
LM 4.8 Evaluation: Extent to which program statistics, surveys, and small studie												
Don't know	0	0	1	7.7	1	**12.5**	0	0	0	0	0	0
LM 4.9 Management‚Äôs Use of Evaluation Findings: Extent to which local-level prog												
Don't know	0	0	1	7.7	1	**12.5**	0	0	0	0	0	0
LM 4.10 Ministerial Use of Evaluation Findings: Extent to which the Ministry adm												
Don't know	0	0	2	**15.4**	1	**12.5**	0	0	0	0	0	0
LM 4.11 Dissemination of Information to Other Implementing Bodies: Extent to whi												

Note:

- For readability purpose, complete wording for questions/variables has been provided as Appendix 1.
- Only the percentages of respondents who responded “don’t know” to the respective question or sub-question was included in this table.
- Highlighted are values > 10%

For peer review only

Don't know	0	0	1	7.7	1	**12.5**	0	0	0	0	0	0
LM 5.1 CSO Actor Power: Extent to which CSO actors are in strong positions to in												
Don't know	1	**11.1**	1	7.7	0	0	0	0	0	0	0	0
LM 5.2 Budget Analysis as a CSO Tool: Extent to which CSOs utilize budget analys												
Don't know	2	**22.2**	2	**15.4**	0	0	1	**11.1**	0	0	0	0
LM 5.3 Community-Based Service Delivery Support: Extent to which CSOs participat												
Don't know	2	**22.2**	1	7.7	0	0	1	**11.1**	0	0	0	0
LM 5.4 Diversification of Job Opportunities: Extent to which CSOs and the privat												
Don't know	2	**22.2**	1	7.7	0	0	1	**11.1**	0	0	0	0
LM 5.5 Human Rights and Quality of Implementation: Extent to which CSOs support												
Don't know	3	**33.3**	1	7.7	0	0	1	**11.1**	0	0	0	0
LM 5.6 Youth: Extent to which CSOs support labor market policies, interventions												
Don't know	2	**22.2**	1	7.7	0	0	0	0	0	0	0	0
LM 5.7 Programming for Women: Extent to which CSOs support labor market policies												
Don't know	2	**22.2**	1	7.7	0	0	0	0	0	0	0	0
LM 5.8 Advocacy/Accountability: Extent to which CSOs support the training and ca												
Don't know	2	**22.2**	1	7.7	0	0	1	**11.1**	0	0	0	0

Note:

- For readability purpose, complete wording for questions/variables has been provided as Appendix 1.
- Only the percentages of respondents who responded “don’t know” to the respective question or sub-question was included in this table.
- Highlighted are values > 10%

For peer review only

LM 5.9 CSO-Led Assessment and Monitoring: Extent to which CSOs assess, monitor a												
Don't know	2	**22.2**	1	7.7	0	0	1	**11.1**	0	0	0	0
LM 5.10 Forming Partnerships between CSOs: Extent to which the private sector ha												
Don't know	2	**22.2**	1	7.7	0	0	1	**11.1**	0	0	0	0
GEI 1.1 Democratic Values: Extent to which the government has ratified, domestic												
Don't know	1	**11.1**	0	0	0	0	1	**16.7**	0	0	0	0
GEI 1.2 Monitoring Democratic Values: Extent to which the government has created												
Don't know	1	**11.1**	0	0	1	8.3	1	**16.7**	0	0	1	**12.5**
GEI 1.3 Affirmation of Youth Democratic Participation: Extent to which the gover												
Don't know	0	0	0	0	0	0	1	**16.7**	0	0	0	0
GEI 1.4. Removing Legal Youth Barriers: Extent to which the government has prosc												
Don't know	0	0	0	0	0	0	1	**16.7**	0	0	0	0
GEI 1.5 Youth Policy: Extent to which the youth policy takes into account the la												
Don't know	0	0	0	0	0	0	1	**16.7**	0	0	0	0
GEI 1.6 Affirmation of Democratic Participation/Revision of Customary Law: Exten												
Don't know	0	0	0	0	1	8.3	1	**16.7**	0	0	0	0
GEI 1.7 Youth Policymaking Participation: Extent to which the government ensures												

Note:

- For readability purpose, complete wording for questions/variables has been provided as Appendix 1.
- Only the percentages of respondents who responded "don't know" to the respective question or sub-question was included in this table.
- Highlighted are values > 10%

For peer review only

Don't know	0	0	1	**16.7**	1	8.3	1	**16.7**	0	0	0	0
GEI 1.8 Rule of Law: Extent to which the rule of law is formally, legally establ												
Don't know	1	**11.1**	0	0	1	8.3	1	**16.7**	0	0	0	0
GEI 1.9 Transparency and Accountability: Extent to which national legislation ma												
Don't know	0	0	0	0	0	0	1	**16.7**	0	0	0	0
Don't know	0	0	0	0	1	8.3	1	**16.7**	0	0	0	0
GEI 1.10 National Security: Extent to which the country/government is involved i												
Don't know	1	**11.1**	1	**16.7**	0	0	1	**16.7**	0	0	2	**25**
Don't know	0	0	0	0	0	0	1	**16.7**	0	0	2	**25**
Don't know	1	**11.1**	0	0	0	0	1	**16.7**	0	0	3	**37.5**
GEI 1.11 Rights: Extent to which the constitution affirms certain rights: freedo												
Don't know	0	0	0	0	0	0	1	**16.7**	0	0	0	0
GEI 1.12 Education and Democratic Values: Extent to which education policies, st												
Don't know	0	0	0	0	0	0	1	**16.7**	0	0	0	0
GEI 1.13 Affirmation of Safe Learning Environments: Extent to which education po												
Don't know	0	0	0	0	0	0	1	**16.7**	0	0	0	0
GEI 2.1 Strengthening Youth Activities/Institutions: Extent to which efforts are												
Don't know	1	**12.5**	0	0	0	0	0	0	0	0	0	0

Note:

- For readability purpose, complete wording for questions/variables has been provided as Appendix 1.
- Only the percentages of respondents who responded “don’t know” to the respective question or sub-question was included in this table.
- Highlighted are values > 10%

For peer review only

GEI 2.2 Appointment of Youth Leader: Whether and to what extent there has been a												
Don't know	1	**12.5**	0	0	0	0	0	0	0	0	1	**12.5**
GEI 2.3 Youth Ideals and Aspirations: Extent to which programming is designed to												
Don't know	1	**12.5**	0	0	0	0	0	0	0	0	0	0
GEI 2.4 Youth Services: Extent to which programming is designed to address youth												
Don't know	1	**12.5**	0	0	1	8.3	0	0	0	0	0	0
GEI 2.5 Youth Involvement in National Initiatives: Extent to which there are eff												
Don't know	1	**12.5**	0	0	1	8.3	0	0	0	0	0	0
GEI 2.6 Personal Safety: Extent to which individual safety is provided for throu												
GEI 2.7 Participation: Extent to which government functions involve the particip												
GEI 2.8 Gender: Extent to which women participate in:												
GEI 2.8.1	2	**25**	0	0	0	0	0	0	0	0	0	0
GEI 2.8.2	0	0	1	**20**	0	0	0	0	0	0	0	0
GEI 2.8.3	0	0	0	0	0	0	0	0	0	0	2	**25**
GEI 2.9 Promotion of Equality: Extent to which gender equality in all aspects of												
Don't know	0	0	0	0	1	8.3	0	0	0	0	0	0
GEI 2.10 Sustainable Economic Opportunity:												
GEI 2.11 Human Development:												

Note:

- For readability purpose, complete wording for questions/variables has been provided as Appendix 1.
- Only the percentages of respondents who responded “don’t know” to the respective question or sub-question was included in this table.
- Highlighted are values > 10%

For peer review only

GEI 2.11.1	0	0	0	0	1	8.3	0	0	0	0	0	0
GEI 2.11.2	0	0	0	0	1	8.3	0	0	0	0	0	0
GEI 2.11.3	0	0	0	0	1	8.3	0	0	0	0	0	0
GEI 2.11.4	0	0	0	0	1	8.3	0	0	0	0	0	0
GEI 3.1 Youth Empowerment: Extent to which youth empowerment has been enhanced,												
Don't know	0	0	0	0	1	9.1	0	0	0	0	0	0
GEI 3.2 Government Advocacy: Extent to which members of the government advocates												
Don't know	0	0	1	**20**	1	9.1	0	0	0	0	0	0
GEI 3.3 Non-governmental Advocacy: Extent to which non-governmental actors advoc												
Don't know	0	0	1	**20**	2	**18.2**	0	0	0	0	0	0
GEI 3.4 Civil Society Organizations (CSOs): Extent to which CSOs specifically op												
Don't know	0	0	0	0	1	9.1	0	0	0	0	0	0
GEI 3.5 Advocacy Voice: Extent to which advocates have a voice in government dis								**Tanzania**	**Tanzania**	**Total**	**Total**	
Don't know	0	0	0	0	1	9.1	0	0	0	0	1	2.7
GEI 3.6 Voice of Instrumental Sectors of the DD: Extent to which the government												
GEI 3.6.1	0	0	0	0	1	9.1	0	0	0	0	0	0
GEI 3.6.2	0	0	0	0	1	9.1	0	0	0	0	0	0
GEI 3.6.3	0	0	0	0	1	9.1	0	0	0	0	0	0
GEI 3.6.4	0	0	0	0	1	9.1	0	0	0	0	0	0

Note:

- For readability purpose, complete wording for questions/variables has been provided as Appendix 1.
- Only the percentages of respondents who responded "don't know" to the respective question or sub-question was included in this table.
- Highlighted are values > 10%

For peer review only

GEI 3.6.5	0	0	0	0	2	**18.2**	0	0	0	0	0	0
GEI 3.6.6	0	0	0	0	1	9.1	0	0	0	0	0	0
GEI 4.1 Strength of Observatory/Stakeholder/Technical Working Group for GEI: Ext												
GEI 4.1.1	1	**12.5**	0	0	1	9.1	0	0	0	0	0	0
GEI 4.1.2	2	**25**	0	0	1	9.1	0	0	0	0	0	0
GEI 4.1.3	2	**25**	0	0	1	9.1	0	0	0	0	0	0
GEI 4.2¬†Education/Research Strategy: Extent to which national planning documents												
Don't know	0	0	0	0	2	**18.2**	0	0	0	0	1	**14.3**
GEI 4.3 Governance, Financial and Development Data Gathering Partners:												
GEI 4.3.1	0	0	0	0	1	9.1	0	0	0	0	0	0
GEI 4.3.2	0	0	0	0	2	**18.2**	0	0	0	0	0	0
GEI 4.3.3	0	0	1	**20**	1	9.1	0	0	0	0	0	0
GEI 4.4 Governance, Financial and Development Topical Data Gathering:												
GEI 4.4.1	1	**12.5**	0	0	1	9.1	0	0	0	0	0	0
GEI 4.4.2	0	0	0	0	1	9.1	0	0	0	0	2	**28.6**
GEI 4.4.3	0	0	0	0	2	**18.2**	0	0	0	0	0	0
GEI 4.4.4	0	0	0	0	1	9.1	0	0	0	0	0	0
GEI 4.5 Quality/Coverage of Data:												
GEI 4.5.1	0	0	0	0	2	**18.2**	0	0	0	0	1	**14.3**
GEI 4.5.2	0	0	0	0	2	**18.2**	0	0	0	0	1	**14.3**

Note:

- For readability purpose, complete wording for questions/variables has been provided as Appendix 1.
- Only the percentages of respondents who responded “don’t know” to the respective question or sub-question was included in this table.
- Highlighted are values > 10%

For peer review only

GEI 4.5.3	0	0	0	0	2	18.2	0	0	0	0	0	0
GEI 4.5.4	0	0	0	0	2	18.2	0	0	2	28.6	4	10.8
GEI 4.6 Quality Research Institutions: Extent to which the country has the capac												
Don't know	0	0	0	0	1	9.1	0	0	0	0	0	0
GEI 4.7 Evaluation: Extent to which program statistics, surveys, and small studi												
Don't know	0	0	0	0	1	9.1	0	0	0	0	0	0
GEI 4.8 Management‚Äôs Use of Evaluation Findings: Extent to which local-level pro												
Don't know	0	0	0	0	1	9.1	0	0	0	0	0	0
GEI 4.10 Dissemination of Information to Other Implementing Bodies: Extent to wh												
Don't know	0	0	0	0	1	9.1	0	0	0	0	0	0
GEI 5.1 CSO Actor Power: Extent to which CSO actors are in strong positions to p												
Don't know	0	0	0	0	1	9.1	1	16.7	0	0	0	0
GEI 5.2 Budget Analysis as a CSO Tool: Extent to which CSOs utilize budget analy												
Don't know	3	42.9	0	0	1	9.1	1	16.7	0	0	0	0
GEI 5.3 Community-Based Service Delivery Support: Extent to which CSOs participa												
Don't know	0	0	0	0	1	9.1	1	16.7	0	0	0	0
GEI 5.4 Human Rights and Quality of Implementation: Extent to which CSOs advocat												
Don't know	1	14.3	0	0	1	9.1	1	16.7	0	0	0	0

Note:

- For readability purpose, complete wording for questions/variables has been provided as Appendix 1.
- Only the percentages of respondents who responded “don’t know” to the respective question or sub-question was included in this table.
- Highlighted are values > 10%

For peer review only

GEI 5.5 Youth: Extent to which CSOs support youth to participate in education, t												
Don't know	1	**14.3**	0	0	1	9.1	1	**16.7**	0	0	0	0
GEI 5.6 Programs for Women: Extent to which CSOs ensure GEI policies support wom												
Don't know	0	0	0	0	1	9.1	1	**16.7**	0	0	0	0
GEI 5.7 Advocacy/Accountability												
Don't know	0	0	0	0	2	**18.2**	1	**16.7**	0	0	0	0
Don't know	0	0	0	0	2	**18.2**	1	**16.7**	0	0	0	0
GEI 5.8 CSO-Led Assessment and Monitoring: Extent to which CSOs assess, monitor												
Don't know	0	0	0	0	1	9.1	1	**16.7**	0	0	0	0
GEI 5.9 Forming Partnerships between CSOs: Extent to which CSOs have formed nati												
Don't know	0	0	0	0	1	9.1	1	**16.7**	0	0	0	0

For peer review only

Note:

- For readability purpose, complete wording for questions/variables has been provided as Appendix 1.
- Only the percentages of respondents who responded "don't know" to the respective question or sub-question was included in this table.
- Highlighted are values > 10%

STROBE checklist — A new demographic dividend effort index (DDEI): results from an experts' survey from six sub-Saharan African countries.

	Page	Recommendation (Strobe)
**Title and abstract**	1	(*a*) Indicate the study's design with a commonly used term in the title or the abstract
		(*b*) Provide in the abstract an informative and balanced summary of what was done and what was found
**Introduction**		
Background/rationale	3-4	Explain the scientific background and rationale for the investigation being reported
**Methods**		
Study design	5	Present key elements of study design early in the paper
Setting	5-6	Describe the setting, locations, and relevant dates, including periods of recruitment, exposure, follow-up, and data collection
Participants	6	*Cross-sectional study*—Give the eligibility criteria, and the sources and methods of selection of participants
Variables	6	Clearly define all outcomes, exposures, predictors, potential confounders, and effect modifiers. Give diagnostic criteria, if applicable
Data sources/ measurement	5-7	For each variable of interest, give sources of data and details of methods of assessment (measurement). Describe comparability of assessment methods if there is more than one group
Bias	6	Describe any efforts to address potential sources of bias
Study size	6	Explain how the study size was arrived at
Quantitative variables	6-7	Explain how quantitative variables were handled in the analyses. If applicable, describe which groupings were chosen and why
Statistical methods	6-7	(*a*) Describe all statistical methods, including those used to control for confounding
		(*b*) Describe any methods used to examine subgroups and interactions
		(*c*) Explain how missing data were addressed
		(*d*) *Cross-sectional study*—If applicable, describe analytical methods taking account of sampling strategy
		(*e*) Describe any sensitivity analyses

Continued on next page

For peer review only

**Results**		
Participants	6	(a) Report numbers of individuals at each stage of study—eg numbers potentially eligible, examined for eligibility, confirmed eligible, included in the study, completing follow-up, and analysed
Descriptive data	8	(a) Give characteristics of study participants (eg demographic, clinical, social) and information on exposures and potential confounders
Outcome data	8-9	*Cross-sectional study*—Report numbers of outcome events or summary measures
Main results	7-10	(*a*) Give unadjusted estimates and, if applicable, confounder-adjusted estimates and their precision (eg, 95% confidence interval). Make clear which confounders were adjusted for and why they were included
		(*b*) Report category boundaries when continuous variables were categorized
		(*c*) If relevant, consider translating estimates of relative risk into absolute risk for a meaningful time period
Other analyses	10	Report other analyses done—eg analyses of subgroups and interactions, and sensitivity analyses
**Discussion**		
Key results	9 -10	Summarise key results with reference to study objectives
Limitations	12	Discuss limitations of the study, taking into account sources of potential bias or imprecision. Discuss both direction and magnitude of any potential bias
Interpretation	10 -11	Give a cautious overall interpretation of results considering objectives, limitations, multiplicity of analyses, results from similar studies, and other relevant evidence
Generalisability	11-12	Discuss the generalisability (external validity) of the study results
**Other information**		
Funding	14	Give the source of funding and the role of the funders for the present study and, if applicable, for the original study on which the present article is based

For peer review only

BMJ Open

A new demographic dividend effort index (DDEI): results from an experts' survey from six sub-Saharan African countries.

Journal:	*BMJ Open*
Manuscript ID	bmjopen-2021-059937.R3
Article Type:	Original research
Date Submitted by the Author:	06-Mar-2023
Complete List of Authors:	Rusatira, Jean Christophe; Johns Hopkins University Bloomberg School of Public Health, PHFR<br>Cardona, Carolina; Johns Hopkins University Bloomberg School of Public Health, Population Family and Reproductive Health<br>Martinez-Baack, Michelle ; Johns Hopkins University Bloomberg School of Public Health, PHFR<br>Rimon, Jose; Johns Hopkins University Bloomberg School of Public Health, Population, Family and Reproductive Health<br>Ahmed, Saifuddin; Johns Hopkins University Bloomberg School of Public Health, Population, Family And Reproductive Health
**Primary Subject Heading**:	Global health
Secondary Subject Heading:	Global health, Health policy
Keywords:	PUBLIC HEALTH, HEALTH ECONOMICS, Demography < TROPICAL MEDICINE, STATISTICS & RESEARCH METHODS, International health services < HEALTH SERVICES ADMINISTRATION & MANAGEMENT

SCHOLARONE™
Manuscripts

I, the Submitting Author has the right to grant and does grant on behalf of all authors of the Work (as defined in the below author licence), an exclusive licence and/or a non-exclusive licence for contributions from authors who are: i) UK Crown employees; ii) where BMJ has agreed a CC-BY licence shall apply, and/or iii) in accordance with the terms applicable for US Federal Government officers or employees acting as part of their official duties; on a worldwide, perpetual, irrevocable, royalty-free basis to BMJ Publishing Group Ltd ("BMJ") its licensees and where the relevant Journal is co-owned by BMJ to the co-owners of the Journal, to publish the Work in this journal and any other BMJ products and to exploit all rights, as set out in our licence.

The Submitting Author accepts and understands that any supply made under these terms is made by BMJ to the Submitting Author unless you are acting as an employee on behalf of your employer or a postgraduate student of an affiliated institution which is paying any applicable article publishing charge ("APC") for Open Access articles. Where the Submitting Author wishes to make the Work available on an Open Access basis (and intends to pay the relevant APC), the terms of reuse of such Open Access shall be governed by a Creative Commons licence – details of these licences and which Creative Commons licence will apply to this Work are set out in our licence referred to above.

Other than as permitted in any relevant BMJ Author's Self Archiving Policies, I confirm this Work has not been accepted for publication elsewhere, is not being considered for publication elsewhere and does not duplicate material already published. I confirm all authors consent to publication of this Work and authorise the granting of this licence.

For peer review only

A new demographic dividend effort index (DDEI): results from an experts' survey from six sub-Saharan African countries.

Authors
Jean Christophe Rusatira*, Carolina Cardona*, Michelle Martinez-Baack*, Jose G. Rimon*, Saifuddin Ahmed*

* Department of Population, Family and Reproductive Health, Johns Hopkins Bloomberg School of Public Health

Corresponding Author
E-mail address: jcrusatira@jhu.edu
Department of Population, Family and Reproductive Health
Johns Hopkins Bloomberg School of Public Health
615 N. Wolfe St., Baltimore, MD 21030, USA

Objective: This study aimed at developing a tool to measure the extent of national efforts in policies, services, research, and programs implemented to cultivate and harness the benefits of a potential demographic dividend in six Sub-Saharan African countries.

Design: The survey was self-administered online using the SurveyMonkey platform. The survey questionnaire covered six key sectors: governance and economic institutions, family planning, maternal and child health, education, women's empowerment, and the labor market. Each sector-specific questionnaire was structured around five practice domains: policymaking, services and programs, advocacy, research, and civil society. Each item was scored from 1 to 10. Factor analysis was used to select the items to be retained for final score estimation. Simple averages were computed to estimate sectoral and domain scores and overall country scores were estimated using weighted country mean scores. Internal consistency, construct validity and reliability were examined using factor analysis and Cronbach's alpha.

Setting: Sub-Saharan Africa

Participants: A total of 440 knowledgeable informants from six countries; namely, Ethiopia (73), Kenya (69), Nigeria (67), Rwanda (54), Senegal (81), and Tanzania (96).

Results: Based on the results from factor analysis, 38 items were dropped from the analysis and Cronbach's alpha results ranged from 0.84-0.98 across domains. The overall Demographic Dividend Effort Index (DDEI) scores ranged between 5.4 (95% CI:5.1-5.8) in Ethiopia to 7.7 (95% CI:7.5-8.0) in Rwanda. In most countries, the disaggregated scores by sector revealed low scores in the labor market and women's empowerment.

Conclusion: The DDEI scores highlight important gaps in key health and development sectors. The DDEI proved to be a reliable and internally consistent tool for effort measurement in key demographic dividend sectors. The DDEI can serve as a self-evaluation tool for local actors and may complement existing quantitative tools such as the Global Gender Gap and the Human Capital Index.

Strengths and limitations of the study

- The study questionnaire was developed using existing effort indices and integrated inputs from local partners working in different health and development sectors.
- The study collected data from knowledgeable informants from six countries that are in the early stages of their demographic transition to explore the applicability and utility of a demographic dividend effort index.
- The Demographic Dividend Effort Index revealed overall high reliability and internal consistency for measuring the efforts in six key demographic dividend sectors across the six countries.
- The study relied on expert opinions working in different sectors in respective countries, which may be prone to bias and may not be comparable across countries.
- A structural equation model could not be implemented for validation because of sample size limitations.

peer review only

For peer review only - http://bmjopen.bmj.com/site/about/guidelines.xhtml

Background

The reduction of births and infant deaths during the third stage of the demographic transition produces changes in the age structure of a population.[1] These changes present countries with an opportunity to experience a demographic dividend (DD) characterized by economic growth, sustainable development, and social change.[2-4] These demographic changes can spur economic growth by increasing the number of working-age individuals compared to the number of young dependents. However, favorable policies and investments are required for countries to harness the DD opportunity presented by such population age structure changes.

Across the Sub-Saharan African (SSA) region, more than three in four countries are still in the early stages of their demographic transition. They are projected not to reach the peak of the transition, where the DD occurs, until 2050.[5] Although this transition started more than 50 years ago, with declines in fertility levels from an average of 6.8 children per woman to an average of 4.8 children by the mid-2010s, it has been slower compared to other continents.[2] Currently, working-age individuals represent about 60% of the population, and this proportion is expected to keep increasing in the coming few decades.[6] These shifting population dynamics present a unique opportunity for SSA countries to grow their economies and break the cycle of poverty and poor health that has become chronic in the majority of these countries.

Previous literature has identified that, in Asian countries, the DD was responsible for around one-third of their economic growth between the 1960s and 1990s.[7, 8] These experiences have sparked an interest among African countries. Over the last decade, countries in the region have embarked on the DD development agenda. The majority of the African Union (AU) member states have developed their DD roadmaps and strategic plans.[9] However, there is no standard tool to measure the extent of national efforts in policies, services, research, advocacy, and programs implemented to cultivate, realize, and harness the benefits of a potential DD in different SSA countries.

The AU and member states recognize that the DD is a key component to achieving sustainable socio-economic growth. In 2017, the AU established the theme of the year as "Harnessing the Demographic Dividend Through Investments in Youth". This theme generated momentum for the DD across the continent, particularly after the development of the AU DD roadmap. The roadmap identifies labor, education, health, governance, and youth empowerment as five fundamental pillars of the DD.[9] Cardona and colleagues further expanded these pillars based on their findings from a systematic literature review that highlighted six key sectors for cultivating and capitalizing on the DD potential in SSA.[10] These sectors are governance and economic institutions (GEI), family planning (FP), maternal and child health (MCH), education (ED), women's empowerment (WE), and the labor market (LM).

A central feature to enable a favorable policy environment, and attain a DD, is good governance and strong economic institutions. Governments have the power to attract foreign direct investments to create jobs and contribute to the economy. Strong financial institutions can promote a culture of saving, supported by favorable interest rates to fuel development. Bloom and colleagues have previously documented the correlation between economic stability, institutional quality and economic growth and have evidenced that unstable countries miss out on their DD opportunities.[2, 11, 12]

Political and economic stability creates an opportunity for a more prosperous community through successful health and development programs. Successful FP programs have been proven to improve

women's participation in the workforce and have been identified as key interventions to reduce maternal and child mortality due to their impact on birth spacing and fertility levels.[13-16] The reduction of infant mortality is also intertwined with the reduction in fertility and is directly associated with the reduction in maternal mortality. Conley and colleagues evidenced that a reduction of 0.5-0.9 points in the total fertility rate (TFR) was associated with a fall of 50 deaths per 1,000 live births in the child mortality rate.[17] This association is concordant with Becker's theory about the quantity-quality trade-off of children, which predicts that parental investments per child increase as fertility levels decline.[18] As families become smaller, more resources are freed to support children's development and the household as a whole.[15, 19]

Related to the quantity-quality trade-off, investments in human capital per child increase as fertility lowers, which can accelerate the economy. Ahmed and colleagues found a potential additional 22.4% increase in GDP per capita in SSA from an increase of just 13% in education attainment between 2011-2030, which eventually increases the availability of skilled workers.[20] Equitable access to quality education for boys and girls is also necessary for countries to generate skilled workers that can later support their economies. Moreover, the education of girls plays an important role in empowering women, who can later enter the labor force and foster socio-economic growth in SSA countries.[21]

Africa's young population presents an opportunity for SSA countries to transform their economies. However, to seize this opportunity, countries may require different strategies depending on their demographic transition stage. On the one hand, countries that are still in the initial stage of their demographic transition, also referred to as "pre-dividend", still need to accelerate their demographic transition and develop the human capital of the growing youth population.[19, 22] These countries are characterized by a total fertility rate (TFR) of 4 or higher children per woman and a projected growing working-age population by 2030.[19, 22] On the other hand, countries that are in the middle of their demographic transition, also referred to as "early-dividend", require smart investments in human capital development so that they can fill various job opportunities in the labor market. These countries are characterized by having a TFR of fewer than 4 children per woman and a projected growing working-age population by 2030.[19, 22] For the socio-economic growth to happen, multisectoral efforts are required and countries need to generate jobs faster than their population growth, explore innovative strategies to transition young graduates to formal jobs, and facilitate job creation in the private sector.[23]

Over the last four decades, effort measurements have been proven to be reliable approaches to informing international health and development programs. Since the 1970s, the Family Planning Effort Index (FPEI) has provided reliable estimates to measure the strength of family planning programs in more than 90 countries and has been proven to be valid relative to family planning outcomes.[24-26] Building on the success of the FPEI, the same approach has been applied in HIV/AIDS programs and maternal and child health which led to the development of the AIDS Program Effort Index (API) in more than 35 countries[27] and the Maternal and Neonatal Program Index (MNPI) in more than 46 countries.[28] However, because health and development systems do not operate in silos, the successful implementation of FPEI, API and MNPI presents an opportunity to develop a multidimensional index to measure DD progress in SSA.

This study aimed at developing an effort assessment index as a standard measure to quantify the nature and strength of in-country DD efforts in the key sectors identified as necessary to harness a DD. This

For peer review only - http://bmjopen.bmj.com/site/about/guidelines.xhtml

index is meant to be used by national and global actors in the development sector working in five development domains: policy, services and programs, advocacy, research, and civil society.

Methodology

Study design

Data were collected through an online survey that measured the efforts that countries are devoting to maximize their potential to reap the benefits of a DD. The survey was administered through an internet-based modality using the SurveyMonkey platform in six countries: Kenya, Senegal, Nigeria, Rwanda, Tanzania, and Ethiopia. These countries were selected because of where they stand in the demographic transition and the convenience to successfully implement the study. The selection of countries sought to include both pre-dividend and early-dividend countries[29] in which the study team had ongoing existing partners with the ability to engage a diverse pool of respondents. The survey(s) was administered in two waves to accommodate Coronavirus Disease 2019 (COVID-19) lockdowns in different countries. The first wave occurred from August to November 2020, and the second wave that covered Senegal alone took place from March to May 2021. The main language of the questionnaire was English (Appendix 1), and a French version (Appendix 2) was created for Senegal by a certified translator.

The questionnaires were developed for each of the six sectors recommended by Cardona and colleagues: FP, MCH, ED, WE, LM, and GEI.[10] All questionnaires were structured around five domains: 1) policy; 2) services and programs; 3) advocacy; 4) research; and 5) civil society. These domains were selected based on prior similar effort indices implemented in various health and development areas.[27, 28, 30, 31] These domains also corresponded to the areas of practice in the field and were confirmed by the local partners during the study design process. The Demographic Dividend Effort Index (DDEI) questionnaire was composed of 490 items divided across the six sectors (112 items for FP, 101 items for MCH, 61 items for ED, 71 items for WE, 68 items for LM, and 77 items for GEI). Where available, the development of each sector-specific questionnaire integrated validated questions from existing effort indices. For instance, the Family Planning Effort Index (FPEI) questionnaire[30] was integrated into the FP questionnaire. Similarly, specific questions from the Maternal and Neonatal Program Index (MNPI),[28] the Rwanda Governance Scorecard,[32] and the AIDS Program Effort Index (API)[27] were integrated into different sector-specific questionnaires. Additional questions were drafted based on DD-favorable interventions for respective sectors as summarized in the wheel of prosperity by Cardona et al.[10] More details on the questions drawn from existing indices have been provided as supplemental material (Appendix 3). During data collection, participants only ever received single surveys that pertained to their sector of expertise and never the entirety of the aggregate survey. Each item was scored on a scale of 1 to 10, from weaker (1) to stronger (10) perceived level of effort or strength. The scoring range of 1-10 has been used in the FPEI and API[27, 30] and allowed a wide variability needed to detect differences between domains and sectors.

The DDEI questionnaire was pilot-tested among 27 experts from Nigeria, Ethiopia, Tanzania, Senegal, Kenya and Rwanda from June to August 2020. This pilot study allowed local experts to provide their feedback and inputs to the questionnaire, which were integrated into the final revision. The local experts were requested to provide qualitative feedback on content, conciseness, and completeness by sector, and were requested to provide recommendations on changes to be made. Following the feedback from local experts, the language and content of the questionnaire were amended to improve clarity and conciseness. The final questionnaire was shared with local project partners for final review and approval before

administering at the national level across all six countries. Each sector-specific questionnaire included items that assessed the presence and strength of policies, services and programs, advocacy efforts, research, and the involvement of civil society organizations in that sector. No personal identification or socio-demographic characteristics information was collected to limit the concern of being identifiable for the respondents and minimize information bias.

Study participants

The in-country partners identified the potential participants who were knowledgeable in respective domains and sectors in each country. In-country partners included the Centre d'Excellence Régional en Economie Générationnelle in Senegal, the Center for Communication Programs in Nigeria, the National Council for Population and Development in Kenya, the Tanzania Communication and Development Center, the Ethiopian Academy of Sciences, and the Centre for Integrated Development Research and Action in Rwanda. Participants were considered for the survey if they were regarded to have demonstrated expertise in at least one of the six sectors by the in-country partner. Expertise was defined as having worked in one of the health or development sectors at the national level as a policymaker, advocate, service provider, researcher, or member of a civil society organization. The local study partners had the best knowledge of potential respondents and led the data collection process locally.

As with other similar indices, sampling was necessarily purposive given the need to identify knowledgeable respondents. The design entailed the selection of 10 respondents for each sector. Whenever possible, diversity across the five domains of expertise was prioritized. As such, to reach 10 respondents per sector, each of the six sectors was designed to have two respondents with sector expertise in one of the five domains. These were exclusive classifications such that respondents could only be counted toward the completion of one sector-specific questionnaire. The survey was administered through an online modality, "SurveyMonkey", in each country. Local partners in respective countries appointed one contact person to respond to any queries regarding the study. Requests to complete the surveys were sent through existing mailing lists of national technical working groups and up to three follow-up calls were made to all individuals for improving survey response rates. The sample size goal was 60 respondents per country.

The expected sample size was on the higher end of sample sizes for similar indices such as the Human Resources Effort Index (N = 16-28 per country), the FPEI (N = 1-12 per country), the API (N = 20-25 per country), and the MNPI (N=10-25).[27, 28, 30, 31] These indices similarly surveyed across various sectors and, sometimes, domains of expertise. However, each of the existing indices was focused on one specific topic, while the DD required a multisectoral sample frame. As this study aimed for 60 respondents per country, considering an expected response rate similar to that of the FPEI of 49%, which was the highest among the other comparative indices, this study ultimately aimed for at least 30 respondents per country.

Patient and Public involvement

No patients or members of the public were involved in the research design, data analysis, or dissemination of the findings. Local researchers and policymakers contributed to the design of the questionnaire, the implementation of the survey, and were involved in the interpretation of the results.

Analysis

Exploratory data analysis

Exploratory data analysis was done to summarize sample characteristics and distribution of respondents across sectors and domains of expertise at the country level. The proportions of respondents by sector, area of expertise and country were presented in a table to facilitate an easier visualization.

Factor and Cronbach's alpha analysis

Factor analysis was implemented to assess construct internal validity for each domain in each sector. This process allowed us to identify the latent structure revealed by the data. The analysis was conducted by respective sectors retaining one factor for each domain to identify which items loaded on that domain. This process allowed us to assess how each of the items included in the questionnaire loaded on the domain under which the item was administered. Items were retained if their factor loadings were 0.5 or higher. This threshold has been recommended by Costello and Osborne as a more reliable threshold that allows for the selection of items that strongly influence the domain, which improves the internal validity of the measurement.[33] Cronbach's alpha estimates were generated to assess the reliability of the retained items for each domain. A coefficient above 0.60 signals an adequate reliability coefficient.[34]

Index scores

The items retained from factor analysis were used to compute mean scores for countries, sectors, and domains. Sectoral and domain scores were computed as the simple average across the items retained. The total score for each country was computed as a weighted average that accounts for differences in the number of participants across sectors. Hence, sectoral weights for the total score were constructed as the ratio between the number of participants within a sector and the total number of participants for each country. For all scores, corresponding 95% confidence intervals (CI) were calculated with a *t*-distribution because the sample size was relatively small. These estimates were presented graphically for ease of interpretation and comparison across sectors per country.

Data translation and availability

To establish policy relevance, the results from this analysis were shared with in-country partners for review and policy translation. The partners from respective countries conducted a series of multisectoral meetings and workshops, in-person and virtual, to generate policy recommendations based on the results from the analysis. The data have been made available through the Harvard Dataverse open-access repository.[35]

Heterogeneity testing

Because respondents with different levels of expertise may have systematically assigned different ratings to the same items and the domains of expertise of respondents were differently distributed across countries, we checked for heterogeneity across domains using meta-analytical techniques. This analysis used pooled data by country to allow for sufficient sample size. To assess the overall extent of heterogeneity and whether it was adequate to compute summary estimates, the fixed-effects were compared to random-effect estimates. By using this method, substantial discrepancies between the estimates from the two models indicate considerable heterogeneity that can make summary estimates misleading.[36] To further assess heterogeneity across domains in respective countries I-square (I^2) and Tau-squared were used. The I2 values of 25%, 50%, and 75% have been interpreted as representing small, moderate, and high levels of

For peer review only - http://bmjopen.bmj.com/site/about/guidelines.xhtml

heterogeneity.[37] Tau-squared captures the variance between measurements of efforts by domains which can be captured by random-effect meta-analytic models.

Results

Respondents

The sample characteristics of the participants are presented by sector and domain of expertise at the country level (Table 1). The total sample consisted of 440 respondents distributed as follows: Ethiopia (73), Kenya (69), Nigeria (67), Rwanda (54), Senegal (81), and Tanzania (96). The number of respondents, domain of expertise, and sectors varied across countries. In all countries, except Ethiopia, the majority of respondents were from the public sector and the services and programs domains. The average completion rate was 82% and ranged from 78% in Tanzania to 92% in Rwanda. The intended number of participants per sector was achieved in all six countries.

Table 1: Sample Characteristics

	**Ethiopia**	**Kenya**	**Nigeria**	**Rwanda**	**Senegal**	**Tanzania**
	**% (Obs.)**	**% (Obs.)**	**% (Obs.)**	**% (Obs.)**	**% (Obs.)**	**% (Obs.)**
**Sector**						
Family Planning	23.3 (17)	24.6 (17)	26.9 (18)	13.0 (7)	8.6 (7)	39.6 (38)
Maternal and Child Health	20.5 (15)	24.6 (17)	11.9 (8)	16.7 (9)	19.8 (16)	19.8 (19)
Education	20.5 (15)	7.2 (5)	17.9 (12)	13.0 (7)	13.6 (11)	10.4 (10)
Women's Empowerment	11.0 (8)	14.5 (10)	13.4 (9)	29.6 (16)	21.0 (17)	13.5 (13)
Labor Market	12.3 (9)	18.8 (13)	11.9 (8)	16.7 (9)	14.8 (12)	8.3 (8)
Governance and Economic Institutions	12.3 (9)	10.1 (7)	17.9 (12)	11.1 (6)	22.2 (18)	8.3 (8)
**Organization**						
Public Sector	21.9 (16)	68.1 (47)	56.7 (38)	83.3 (45)	63.6 (49)	13.5 (13)
Private Sector	5.5 (4)	0.0 (0)	3.0 (2)	13.0 (7)	10.4 (8)	9.4 (9)
Non-governmental organization	27.4 (20)	27.5 (19)	28.4 (19)	1.9 (1)	20.8 (16)	66.7 (64)
University/research institute	41.1 (30)	1.4 (1)	3.0 (2)	0.0 (0)	3.9 (3)	2.1 (2)
Other (please specify)	4.1 (3)	2.9 (2)	9.0 (6)	1.9 (1)	1.3 (1)	8.3 (8)
**Domain**						
Policymaking	13.7 (10)	20.3 (14)	26.9 (18)	14.8 (8)	17.3 (14)	7.3 (7)
Services or programs	21.9 (16)	58.0 (40)	37.3 (25)	18.5 (10)	66.7 (54)	52.1 (50)
Advocacy	8.2 (6)	11.6 (8)	13.4 (9)	14.8 (8)	8.6 (7)	25.0 (24)
Research	53.4 (39)	5.8 (4)	17.9 (12)	46.3 (25)	2.5 (2)	10.4 (10)
Civil Society	2.7 (2)	4.3 (3)	4.5 (3)	5.6 (3)	4.9 (4)	5.2 (5)
**Obs.**	**73**	**69**	**67**	**54**	**81**	**96**

Factor and Cronbach's alpha analysis

The items that loaded poorly (<0.5) were dropped from further analysis, which reduced the number of items from 112 to 85 items for FP, 101 to 94 items for MCH, 68 to 65 items for LM and 77 to 76 items for GEI. For detailed factor analysis results by domain and sector, see Appendix 4. Cronbach's alpha analysis results revealed excellent reliability with alpha estimates ranging from 0.84 in LM to 0.98 in ED (Table 2).

Index scores

The weighted overall country scores were computed to account for differences in the number of participants per sector. For example, in Ethiopia, there were 17 participants from the family planning sector, while there were only 8 participants in the women's empowerment sector.

Table 2. Results from Cronbach's alpha analysis

Sector	Domain				
	Policy	Services and Programs	Advocacy	Research	Civil Society
Family Planning	0.85	0.93	0.93	0.95	0.97
Maternal and Child Health	0.94	0.99	0.92	0.98	0.97
Education	0.95	0.92	0.89	0.97	0.98
Women's Empowerment	0.98	0.96	0.94	0.98	0.97
Labor Market	0.95	0.95	0.84	0.96	0.97
Governance and Economic Institutions	0.95	0.97	0.94	0.97	0.94

It would not be a balanced score if it assigned the same weight across sectors. Overall, the weighted average scores ranged from 5.4 (95% CI:5.1-5.8) in Ethiopia to 7.7 (95% CI:7.5-8.0) in Rwanda (Table 3). The second highest scores were for Tanzania (6.3, 95% CI:6.0-6.6) and Senegal (6.3, 95% CI:5.9-6.7). The second and third lowest scores were for Nigeria (5.5, 95% CI: 5.1-5.9) and Kenya (5.9, 95% CI: 5.5-6.2), respectively.

Table 3. National overall and sector scores

	Ethiopia	Kenya	Nigeria	Rwanda	Senegal	Tanzania
Sector	Mean (95% CIs) (Obs.)					
Family Planning	5.8 (5.3-6.3) (17)	6.2 (5.6-6.8) (17)	6.1 (5.2-7.0) (18)	6.5 (6.3-6.7) (7)	6.5 (5.1-7.8) (7)	6.3 (5.9-6.7) (38)
Maternal and Child Health	6.0 (4.7-7.2) (15)	6.2 (5.5-7.0) (18)	5.1 (3.8-6.4) (8)	7.4 (7.1-7.6) (9)	7.2 (6.6-7.8) (16)	6.9 (6.3-7.6) (19)
Education	5.3 (4.6-6.1) (15)	5.6 (3.8-7.4) (5)	5.9 (5.1-6.8) (12)	8.2 (7.3-9.1) (7)	5.9 (4.5-7.4) (11)	5.2 (4.1-6.4) (10)
Women's Empowerment	4.8 (3.4-6.1) (8)	5.1 (4.2-5.9) (10)	5.1 (4.1-6.1) (9)	8.3 (8.1-8.6) (16)	6.4 (5.6-7.3) (17)	6.9 (6.1-7.8) (13)
Labor Market	5.1 (4.3-5.9) (9)	5.3 (4.7-5.9) (13)	4.2 (2.9-5.5) (8)	7.6 (7.3-7.8) (9)	5.2 (3.7-6.7) (12)	5.7 (4.3-7.2) (8)
Governance and Economic Institutions	4.9 (4.1-5.7) (9)	6.5 (4.6-8.5) (6)	5.7 (4.7-6.7) (12)	7.8 (7.2-8.4) (6)	6.3 (5.7-6.9) (18)	5.4 (4.3-6.6) (8)
**Weighted Overall Score**	**5.4 (5.1-5.8) (73)**	**5.9 (5.5-6.2) (69)**	**5.5 (5.1-5.9) (67)**	**7.7 (7.5-8.0) (54)**	**6.3 (5.9-6.7) (81)**	**6.3 (6.0-6.6) (96)**

The mean scores per sector were disaggregated by domain of expertise for ease of interpretability and policy relevance. The total scores disaggregated by sector are presented in Figure 1. The radar plots indicate that the sectors with most needs across countries were LM and WE. Both in Nigeria and Ethiopia, the average score for LM and WE struggled to reach the mid-point. However, this was not the case for Rwanda and Tanzania which scored highest in WE, 8.3 (95% CI:8.1-8.6) and 6.9 (95% CI:6.1-7.8), respectively. Ethiopia was the only country that struggled to reach the mid-point in the GEI sector, 4.9 (95% CI:4.1-5.7). By contrast, the MCH, ED, and FP sectors received scores above the mid-point across all countries. For example, MCH ranged from a low of 5.1 (95% CI:3.8-6.4) in Nigeria to a high of 7.4 (95% CI:7.1-7.6) in Rwanda. Furthermore, sectoral scores revealed several interesting variations across domains and countries.

For peer review only - http://bmjopen.bmj.com/site/about/guidelines.xhtml

In Ethiopia, across the six sectors, none scored 5 or higher across all five domains. The scores were lowest for civil society engagement relative to other domains in FP, MCH, LM, and GEI. In most cases, policy or policymaking scored highest across sectors. Of all the scores, the highest average score was recorded in FP advocacy (6.9, 95% CI:6.0-7.9), and the lowest in research for WE (3.8, 95% CI:2.0-5.6) as well as civil society engagement in LM (3.8, 95% CI:2.3-5.4). The other scores that were below the mid-point were for services and programs in WE (4.7, 95% CI:3.3-6.1) and LM (4.8, 95% CI:4.1-5.4), advocacy in LM (4.7, 95% CI:4.1-5.4), research in WE (3.8, 95% CI:2.0-5.6) and GEI (4.6, 95% CI:3.6-5.6), and civil society engagement in MCH (4.9, 95% CI:3.4-6.4), ED (4.0, 95% CI:3.1-4.9) and GEI 4.7 (3.5-5.8). However, in most instances, the differences in scores between domains were not statistically significant due to overlapping confidence intervals.

In Kenya, the average scores were higher than the mid-point across sectors and domains except for research in LM (4.6, 95% CI:3.7-5.4) and civil society in MCH (4.9, 95% CI:3.2-6.7). Scores were around 6 for FP in all the domains. Similarly, except for civil society, the scores were around 6 for MCH. The scores for ED, WE and LM were mostly around 5 and hardly reached 6. GEI scored consistently highest compared to the other sectors across the five domains with the highest score in advocacy (7.0, 95% CI:4.5-9.5).

In Nigeria, most scores reached the mid-point or higher except for LM which hardly reached 5 in any of the five domains. LM scored lowest in services and programs (3.7, 95% CI:2.4-5.1), followed by policymaking (3.9, 95% CI:2.6-5.3), advocacy (4.3, 95% CI:3.0-5.7) and research (4.4, 95% CI:3.0-5.8). The FP scores were highest in nearly all domains compared to the other sectors followed by ED. MCH scores consistently scored around the mid-point or lower in research (4.8, 95% CI:3.2-6.5) and civil society (4.6, 95% CI:2.8-6.5). The scores for WE were consistently second or third lowest across the domains.

In Rwanda, scores were mostly around 7 and none of the domains were scored below the mid-point. The scores were consistently higher for WE and ED across domains and the highest was in policymaking for WE (8.7, 95% CI:8.4-9.0) and research for ED (8.4, 95% CI:7.5-9.3). The lower scores were recorded in FP in nearly all the domains and the lowest score was in services and programs (5.7, 95% CI:5.2-6.2). MCH and LM recorded second or third-lowest scores across the five domains. GEI scored the third highest scores across all five domains.

In Senegal, most scores were above the mid-point across all sectors and domains except in LM. The scores were higher in MCH with the highest in services and programs (8.0, 95% CI:7.3-8.7). The lowest scores were recorded across domains in LM, civil society (4.3, 95% CI:2.7-5.8), advocacy (4.5, 95% CI:2.8-6.2), research (4.7, 95% CI:3.2-6.3) and in services and programs (4.8, 95% CI:3.4-6.3). The scores for WE were consistently around 6 or higher except in research (5.7, 95% CI:4.5-6.9). The scores for WE were consistently around 6 or higher, except in research (5.7, 95% CI:4.5-6.9).

In Tanzania, all the scores across sectors and domains were around 5 or higher. In nearly all the domains, WE scored higher with the highest score in civil society (8.1, 95% CI:7.5-8.6). The lowest scores were recorded mostly in ED and GEI with the lowest score in civil society participation in ED (5.0, 95% CI:3.3-6.6). MCH and FP scored second and third highest across domains, respectively, with the scores ranging from 6.2 (95% CI:5.5-6.9) to 6.9 (95% CI:6.1-7.6).

For peer review only - http://bmjopen.bmj.com/site/about/guidelines.xhtml

Heterogeneity testing

The heterogeneity tests by domains across countries yielded similar results for fixed and random effect models. The I^2 estimates ranged from 0.0% to 55.5% in services and programs for Rwanda. In all the other countries I^2 was 0.0% across all the domains and sectors, suggesting no heterogeneity. Tau-squared was 0.0 across all the domains, sectors, and countries, except in Rwanda where it was 0.46. For detailed heterogeneity testing results, see Appendix 5.

Discussion

This study aimed to develop a tool to assess national efforts in establishing a favorable policy environment to cultivate a demographic dividend. The study collected and analyzed data across six key sectors in Nigeria, Rwanda, Ethiopia, Senegal, Tanzania, and Kenya from key informants identified by the in-country study collaborators.

The DDEI scores revealed patterns that were helpful to guide discussions regarding needed improvements in respective countries across five domains: namely, policymaking, services and programs, advocacy, research, and civil society. The patterns of efforts scores for the LM, WE, FP, GEI and ED sectors suggested great challenges for women's self-determination and empowerment, employment, and leadership. These findings agreed with previous predictions by the World Economic Forum that women may have to wait for 100 years to pair males in leadership roles in bigger institutions and take up ministerial roles. However, in some countries remarkable progress has been recorded indicating potential opportunity to attain gender parity much sooner. This is currently seen in the Global Gender Gap (GGG) Report, which ranks Rwanda in the top ten countries with the smallest gender gap among countries worldwide.[38, 39]

Relatedly, Rwanda scored remarkably high on WE, although the scores were also moderately high for Tanzania and Senegal. These findings aligned with how these countries performed on the 2022 GGG report, except for Kenya. Of the 146 countries ranked globally and on a maximum score possible of 1, Rwanda was ranked 6th with a score of 0.811 and Tanzania 64th with a score of 0.719. In contrast, Ethiopia ranked 74nd (0.710), Senegal 112th (0.668), and Nigeria 123th (0.639).[38] Kenya's rank has remarkably improved from the 2020 GGG report, having moved from 109th with a score of 0.671 to 57th with a score of 0.729. It is possible that the transformations that occurred in the last two years were not captured in the effort measurements, especially because the experts were asked about their experiences over the last 3-5 years prior to the DDEI survey. However, such rapid change in GGG rank indicates the importance of repeating the effort measurements on an annual basis. Further, although the intent of the DDEI was not to rank countries, the study findings underscored the potential for mutual learning from countries and the utility of the DDEI to dive deeper into specific items that can reveal special lessons or areas that require improvements. This is especially crucial for countries that had a much larger change in ranking during the two years that preceded the report, such as Ethiopia which experienced an improvement of more than 40 ranks, and Kenya with a rise of 80 ranks in the last few years.[38, 40]

Furthermore, human capital development is essential for a country to cultivate and harness its DD potential. The 2020 Human Capital Index report revealed that the scores for all six countries of this study ranged from 0.36 in Nigeria to 0.55 in Kenya. These findings indicate that children born today in the six countries could miss out on 45% to 64% of their future earnings potential because of incomplete education and health that is not full.[41] The DDEI provided an opportunity to qualitatively compare different

ongoing efforts initiated to develop human capital and the findings in MCH, ED, and WE were in agreement with the 2020 HCI findings. For instance, the lowest score for MCH was recorded in Nigeria (5.1, 95% CI:3.8-6.4), and domain-specific scores revealed larger gaps in research and civil engagement although the scores in the other domains were also low.

This study offers several strengths. First, the questionnaires and the implementation of the DDEI were conducted in collaboration with in-country partners. This collaboration fosters local ownership of the effort measurement tool. Second, high factor loadings and Cronbach's alpha suggested high internal consistency and reliability of the tool across countries. Our analysis also highlighted 38 items that did not seem to measure the same construct and were dropped from the analysis. Dropping these items improved the quality and length of the questionnaire of the DDEI. Third, the heterogeneity tests across domains revealed negligible heterogeneity in all countries except in the domain of services for Rwanda, which indicates score consistency across domains in these countries and the validity of the summary scores generated.[42] In the future, local institutions can regularly replicate these measurements at national and sub-national levels. Fourth, unlike prior effort indices, such as the FPEI, (created in 1972), the API (created in 1999), and MNPI (created in 2000),[27, 43, 44] the DDEI integrated critical sectors into one tool, and sectoral effort measures were not done in silos due to the interconnectedness and potential synergy between sectors (e.g. family planning, education, maternal and child health, and women's empowerment). Finally, this tool provides information to constituents that could be used to hold their governments accountable and provide unconventional evidence for planning and evaluation purposes.

The DDEI was tested at the national level which opened the opportunity to be applied at the sub-national level. In countries where health and development programs are decentralized, sub-national DDEI can be transformational in guiding local budget allocation and self-evaluation. Given the paucity of quantitative data and poor utilization of the existing information systems, the DDEI has the potential to become a unique data source that informs local discussions and conversations at all administrative and planning levels. However, special emphasis should always be made that beyond strategic allocation of resources, favorable changes in age structure accompanied by a mix of jobs that allows for optimal productivity for all workers are important preconditions to fully realize the country's DD potential.[45, 46]

This study also presented a few limitations. The DDEI relied on experts' opinions, which may be affected by different factors. The respondent's judgment may be heavily influenced by their recent experience, which may not reflect the efforts placed over the years. Their judgment may be influenced by the level of their expectations, which may also be correlated with their level of satisfaction. For instance, where people have low expectations, the scores may be high despite the weak levels of effort, and vice-versa. The challenges related to the reliability of observers' judgments have been documented in other effort measurements such as the FPEI, API and MNPI.[28] However, despite this limitation, effort measures capture unique data that can guide planning and action, especially in countries where the availability of program data is limited. Although the DDEI could be used as a monitoring tool to assess the progress in the indicators that have shown relevance or relationship to DD achievements in the literature, due to the duration that may be required for a DD to happen, the DDEI estimates should not be interpreted as a prediction of achieving a DD. In addition, in some of the countries, the sample size was small for some of the domains which may have affected the reliability and validity of tests. While the sample size could be limited by available knowledgeable respondents or experts in respective sectors for specific countries, the small sample size made it impossible to conduct reliable confirmatory factor analysis.

For peer review only - http://bmjopen.bmj.com/site/about/guidelines.xhtml

Furthermore, other limitations worth mentioning are related to the nature of the responses and scores obtained from the respondents. The proportions of respondents who did not know the answer to the questions and consequently could not provide a score varied across sectors and countries, although no questions had a high (>10%) proportion of "don't know" responses across all six countries. The proportions were notably high in MCH research in Kenya, Nigeria and Tanzania, in ED civil society in Kenya and Nigeria, in WE advocacy in Ethiopia, in LM research and civil society in Ethiopia, Kenya and Nigeria and GEI advocacy and civil society in Nigeria and Rwanda. This finding indicates that for future surveys, some of the questions could be amended to make "don't know" items easier to interpret or removed where found ambiguous. For detailed percentages of "don't know" responses received by item across countries, see Appendix 6. Another limitation is that the aggregate average scores for the sectors modulated centering to the mid-point of 5 over the maximum possible 10. This tendency may indicate that respondents may have compensated for less generous scores that they gave to some questions with more generous scoring for others. However, this is less plausible because the item-specific scores within domains revealed wide variations across scores.

Lastly, the DDEI questionnaire was administered in the midst of the COVID-19 pandemic, which led to abrupt changes in health and development systems in all countries. Although respondents provided great insights about their respective sectors, it is not unrealistic to assume the response rates and responses received may have been affected by the status of pandemics in their countries. Additional research will be needed to nurture the index, include questions that may be used for validation and assess the need for weighing items and domains based on the stage of demographic transition, while improving the usability of the DDEI data by local and global health and development stakeholders.

Conclusion

This study developed the DDEI for Nigeria, Ethiopia, Kenya, Tanzania, Rwanda, and Senegal and demonstrated the utility of this index. The DDEI yielded several recommendations on opportunities to accelerate socio-economic development and take advantage of the DD potential in these countries. The scores for each sector and domain provided variations that allowed for comparison within a sector in each country. By engaging with local experts and partnering with local institutions in a participatory process, the data from the DDEI were used by local policymakers, service providers, program implementers, advocates, and researchers for self-evaluation and to develop recommendations to strengthen ongoing efforts and programs in respective sectors.

The DDEI allowed for multisectoral analysis and provided an opportunity to identify sectors in most need and those that can serve as models in each country. For instance, based on sector-specific scores, LM and WE stood out as the sectors that required particular attention. However, despite higher sector-specific scores, a closer look at item-specific scores across domains revealed wide variations that were the basis of generating policy recommendations. The DDEI can be applied to all SSA countries regularly to monitor progress and foster local conversation around cultivating sustainable socio-economic development, which ultimately may contribute to realizing the benefits of a DD.

The DDEI can serve as a complementary tool to existing global development monitoring tools. The findings from the DDEI aligned with other existing measures such as the GGG and HCI in various countries. Given the quantitative nature of these tools, the DDEI provides an opportunity to assess the inputs and guide policies, investments and programs in specific sectors and items that can positively affect

For peer review only - http://bmjopen.bmj.com/site/about/guidelines.xhtml

the gender gap and human capital. In addition to driving conversation amongst stakeholders in FP, MCH, ED, WE, GEI and LM, the DDEI focuses on assessing the inputs that are critical for the outcomes measured by GGG and HCI. The current DDEI has been translated into English and French. In the future, it should be translated into other languages such as Portuguese and Spanish to make it applicable to other countries in the early stages of their demographic transition.

Ethical considerations

The study was conducted in compliance with the Helsinki Declaration and was approved by the Johns Hopkins Bloomberg School of Public Health ethical review board (IRB Number: IRB00011522). All participants provided informed consent before taking part in this research.

Contributorship statement

Jean Christophe Rusatira, Carolina Cardona, Michelle Martinez-Baack, and Jose G. Rimon conceived the study. Jean Christophe Rusatira, Carolina Cardona, Michelle Martinez-Baack, Saifuddin Ahmed, and Jose G. Rimon developed the questionnaire, led data collection, data analysis, and interpretation. Jean Christophe prepared the first draft of the manuscript. All authors revised and approved the final and revised versions of the paper and agree to be accountable for all aspects of the work.

Acknowledgments

We express our deepest thanks to all participants for their contributions to this study. We would also like to thank our local partners and staff who facilitated this work at the Centre d'Excellence Régional en Economie Générationnelle in Senegal, the Center for Communication Programs in Nigeria, the National Council for Population and Development in Kenya, the Tanzania Communication and Development Center, the Ethiopian Academy of Sciences, and the Centre for Integrated Development Research and Action in Rwanda.

Competing interests

The are no competing interests for any author.

Funding

This work was supported by the Bill and Melinda Gates Foundation under the grant OPP1181398.

Data sharing statement

The data and questionnaires used to generate this article are available on Harvard Dataverse open-access repository accessible at https://doi.org/10.7910/DVN/ZSNPMV.

References

[1] A. L. González and M. J. González-González, "Third demographic transition and demographic dividend: An application based on panel data analysis," *Bulletin of Geography. Socio-economic Series,* no. 42, pp. 59-82, 2018.

[2] D. Bloom, D. Canning, and J. Sevilla, *The demographic dividend: A new perspective on the economic consequences of population change*. Rand Corporation, 2003.

[3] D. S. Reher, "Economic and Social Implications of the Demographic Transition," *Population and Development Review,* vol. 37, pp. 11-33, 2011. [Online]. Available: http://www.jstor.org/stable/41762397.

[4] D. Herzer, H. Strulik, and S. Vollmer, "The long-run determinants of fertility: one century of demographic change 1900–1999," *Journal of Economic Growth,* vol. 17, no. 4, pp. 357-385, 2012/12/01 2012, doi: 10.1007/s10887-012-9085-6.

[5] D. Bloom, S. Humair, L. Rosenberg, J. P. Sevilla, and T. J. Trussell, "A Demographic Dividend for Sub-Saharan Africa: Source, Magnitude, and Realization," *IZA Discussion Paper,* no. 7855, p. 26, 2014. [Online]. Available: SSRN: https://ssrn.com/abstract=2374636.

[6] UNFPA, "Demographic Dividend Atlas for Africa: Tracking the Potential for a Demographic Dividend," United Nations Population Fund, 2017.

[7] D. E. Bloom and J. G. Williamson, "Demographic Transitions and Economic Miracles in Emerging Asia," *The World Bank Economic Review,* vol. 12, no. 3, pp. 419-455, 1998, doi: 10.1093/wber/12.3.419.

[8] D. Bloom, D. Canning, and P. N. Malaney, "Demographic Change and Economic Growth in Asia," Center for International Development at Harvard University, 1999. [Online]. Available: https://EconPapers.repec.org/RePEc:wop:cidhav:15

[9] African Union Commission, "AU Roadmap on Harnessing the Demographic Dividend through Investments in Youth," in "Assembly of the Union Twenty- Sixth Ordinary Session 30-31," African Union Commission, 23 February 2017 2017.

[10] C. Cardona *et al.*, "Generating and capitalizing on the demographic dividend potential in sub-Saharan Africa: a conceptual framework from a systematic literature review," *Gates Open Research,* vol. 4, no. 145, p. 145, 2020.

[11] D. Bloom, D. Canning, and P. N. Malaney, "Demographic change and economic growth in Asia," *CID Working Paper Series,* 1999.

[12] D. E. Bloom and D. Canning, "Global demographic change: Dimensions and economic significance," National Bureau of Economic Research, 2004.

[13] M. S. Fabic *et al.*, "Meeting demand for family planning within a generation: the post-2015 agenda," *The Lancet,* vol. 385, no. 9981, pp. 1928-1931, 2015, doi: 10.1016/S0140-6736(14)61055-2.

[14] D. E. Bloom, D. Canning, L. Hu, Y. Liu, A. Mahal, and W. Yip, "The Contribution of Population Health and Demographic Change to Economic Growth in China and India," *J Comp Econ,* vol. 38, no. 1, pp. 17-33, Mar 1 2010, doi: 10.1016/j.jce.2009.11.002.

[15] Q. Li, A. O. Tsui, L. Liu, and S. Ahmed, "Mortality, fertility, and economic development: An analysis of 201 countries from 1960 to 2015," (in eng), *Gates Open Res,* vol. 2, p. 14, Mar 1 2018, doi: 10.12688/gatesopenres.12804.1.

[16] S. Ahmed, Q. Li, L. Liu, and A. O. Tsui, "Maternal deaths averted by contraceptive use: an analysis of 172 countries," (in eng), *Lancet,* vol. 380, no. 9837, pp. 111-25, Jul 14 2012, doi: 10.1016/s0140-6736(12)60478-4.

For peer review only

[17] D. Conley, G. C. McCord, and J. D. Sachs, "Africa's lagging demographic transition: Evidence from exogenous impacts of malaria ecology and agricultural technology," ed: National Bureau of Economic Research Cambridge, Mass., USA, 2007.
[18] G. S. Becker and G. S. Becker, *A Treatise on the Family*. Harvard university press, 2009.
[19] S. A. Ahmed, M. Cruz, B. Quillin, and P. Schellekens, *Demographic Change and Development: Looking at Challenges and Opportunities through a New Typology* (Policy Research Working Papers). The World Bank, 2016, p. 42.
[20] S. A. Ahmed, M. Cruz, D. S. Go, M. Maliszewska, and I. Osorio-Rodarte, *How Significant is Africa's Demographic Dividend for its Future Growth and Poverty Reduction?* (Policy Research Working Papers). The World Bank, 2014, p. 39.
[21] M. Shekar, A. Yazbeck, R. Hasan, and A. Bakilana, "Population and Development in the Sahel: Policy Choices to Catalyze a Demographic Dividend," *Health, Nutrition and Population (HNP) Discussion Paper, World Bank,* 2016, doi: 10.1596/25293.
[22] WBG, *Global Monitoring Report 2015/2016: Development Goals in an Era of Demographic Change*. Washington, DC: The World Bank, 2016.
[23] S. A. Ahmed and M. Cruz, *Making the Most of Demographic Change in Southern Africa* (Policy Research Working Papers). The World Bank, 2016, p. 30.
[24] J. A. Ross and J. Stover, *Effort Indices for national family planning programs, 1999 cycle*. MEASURE Evaluation, Carolina Population Center, University of North Carolina, 2000.
[25] J. A. Ross and W. P. Mauldin, "Family planning programs: efforts and results, 1972-94," *Studies in family planning,* pp. 137-147, 1996.
[26] W. P. Mauldin, B. Berelson, and Z. Sykes, "Conditions of fertility decline in developing countries, 1965-75," *Studies in Family Planning,* vol. 9, no. 5, pp. 89-147, 1978.
[27] J. Stover, "The AIDS Program Effort Index (API): Results from the Field Test," *Washington, DC: Futures Group,* 1999.
[28] J. A. Ross, O. Campbell, and R. Bulatao, "The maternal and neonatal programme effort index (MNPI)," *Tropical medicine & international health,* vol. 6, no. 10, pp. 787-798, 2001.
[29] W. Bank, "Global monitoring report 2015/2016: Development goals in an era of demographic change," ed: The World Bank, 2015.
[30] J. Ross and J. Stover, "The family planning program effort index: 1999 cycle," *International Family Planning Perspectives,* pp. 119-129, 2001.
[31] A. L. Fort, R. Deussom, R. Burlew, K. Gilroy, and D. Nelson, "The Human Resources for Health Effort Index: A tool to assess and inform strategic health workforce investments," *Human resources for health,* vol. 15, no. 1, pp. 1-11, 2017.
[32] F. Usengumukiza, J. P. Munyandamutsa, and E. Nshutiraguma, "Strengthening democratic governance and accountability in Rwanda: The role of the Rwanda Governance Board (RGB)," in *Democratizing Public Governance in Developing Nations*: Routledge, 2017, pp. 109-133.
[33] A. B. Costello and J. Osborne, "Best practices in exploratory factor analysis: Four recommendations for getting the most from your analysis," *Practical assessment, research, and evaluation,* vol. 10, no. 1, p. 7, 2005.
[34] R. DeVellis, "Chapter 6: Factor Analysis," *Scale development: Theory and applications,* vol. 26, p. 95, 2012.
[Dataset] [35] J. C. Rusatira, C. Cardona, M. Martinez-Baack, J. G. Rimon, and S. Ahmed. *Demographic Dividend Effort Index (DDEI)*, Harvard Dataverse, doi: doi:10.7910/DVN/ZSNPMV.

For peer review only

[36] C. Poole and S. Greenland, "Random-effects meta-analyses are not always conservative," *American Journal of Epidemiology,* vol. 150, no. 5, pp. 469-475, 1999.
[37] J. P. Higgins, S. G. Thompson, J. J. Deeks, and D. G. Altman, "Measuring inconsistency in meta-analyses," *Bmj,* vol. 327, no. 7414, pp. 557-560, 2003.
[38] W. E. Forum, "Global Gender Gap Report 2020: Insight Report," pp. 1-371, 2019.
[39] K. Schwab, "The Global Competitiveness Report 2019. Geneva. World Economic Forum," ed, 2019.
[40] W. E. Forum, "Global Gender Gap Report 2022," 2022. [Online]. Available: https://www.weforum.org/reports/global-gender-gap-report-2022/
[41] W. B. Group, "The Human Capital Index 2020 Update," in *The Human Capital Index 2020 Update*, 2020, doi: 10.1596/978-1-4648-1552-2.
[42] J. Fletcher, "What is heterogeneity and is it important?," *Bmj,* vol. 334, no. 7584, pp. 94-96, 2007.
[43] R. J. Lapham and W. P. Mauldin, "Contraceptive prevalence: the influence of organized family planning programs," *Studies in Family Planning,* vol. 16, no. 3, pp. 117-137, 1985.
[44] J. A. Ross, O. M. Campbell, and R. Bulatao, "The Maternal and Neonatal Programme Effort Index (MNPI)," (in eng), *Trop Med Int Health,* vol. 6, no. 10, pp. 787-98, Oct 2001, doi: 10.1046/j.1365-3156.2001.00787.x.
[45] D. E. Bloom, D. Canning, G. Fink, and J. Finlay, *Realizing the demographic dividend: Is Africa any different?* 2007.
[46] D. E. Bloom, "7 billion and counting," *Science,* vol. 333, no. 6042, pp. 562-569, 2011.

For peer review only

Figure 1: Average scores per country and sector

For peer review only

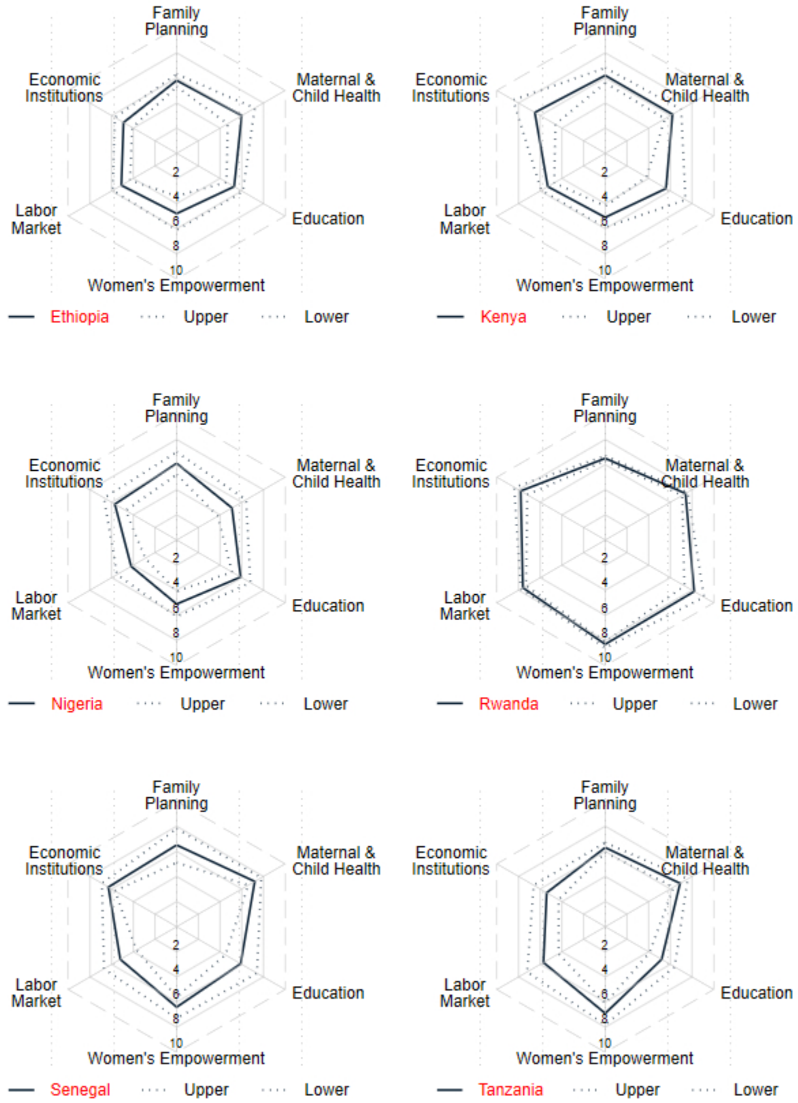

Average scores per country and sector

232x310mm (72 x 72 DPI)

JOHNS HOPKINS
BLOOMBERG SCHOOL
of PUBLIC HEALTH

Bill & Melinda Gates Institute for Population and Reproductive Health

Demographic Dividend Effort Index Survey

Introduction

There is a programmatic need for a standard measure that quantifies the extent of national efforts in policies and programs implemented to cultivate, realize and harness the benefits of a demographic dividend (DD). Despite the remarkable progress achieved by various countries through the integration of demographic dividend principles into their policies and development plans, performance assessment tools and platforms to document best practices and lessons learned to harness the DD are still missing.

Thus, the purpose of the DD Effort Index (DDEI) is to provide a standard measure that will quantify the nature and strength of in-country DD efforts by experts and civil society members across the public and private spheres that represent different dimensions of the society. By measuring efforts, we are capturing inputs independent of outputs, such as income per capita. This will allow us to assess the relationship between inputs that go into the development of DD policies and DD outputs.

This DDEI is guided by one central question: To what extent have the existing national policy and program efforts enabled a favorable policy environment to cultivate, realize and harness the benefits of a demographic dividend? To answer this question, the DDEI will summarize key informant perceptions across six sectors identified in the literature to produce a favorable environment to harness a DD. These DD framework sectors are: 1) Family Planning, 2) Maternal and Child Health, 3) Education, 4) Women's Empowerment, 5) Labor Market, and 6) Good Governance, Strong Institutions and Stable Economy. Each sector is measured through its own questionnaire and will be assessed in five dimensions identified in the literature; namely, policy, services or programs, advocacy, research, and civil society. Each of the 6 questionnaires, completed by 10 expert observers per country, score items on a scale from 1 to 10, unless otherwise noted.

Considering the health and socio-economic impact of COVID-19, and its close relationship with policies that enable a DD favorable policy environment, this DD Effort Index is integrating a module to assess the resilience and sustainability of systems in each sector. The scoring follows the key principles of sustainable and resilient health systems that can effectively respond to emerging infectious disease threats and other public health emergencies.

The DDEI provides an opportunity to visualize the national efforts while assessing the extent to which these efforts support a country's goals to cultivate, realize and harness the demographic dividend. The DDEI will be a tool for performance measurement and progress monitoring, which will provide a timely assessment of successes and gaps and reveal best practices and areas of improvement.

JOHNS HOPKINS
BLOOMBERG SCHOOL
of PUBLIC HEALTH

Bill & Melinda Gates Institute for Population and Reproductive Health

Demographic Dividend Effort Index Survey

Consent form

Thank you for taking the time to complete this questionnaire, especially in this unprecedented time.

The Gates Institute is developing a Demographic Dividend Effort Index (DDEI) to establish a standard measure that will allow the assessment of the existing status of national policies, services, and programs in family planning, maternal and child health, other social sectors (e.g., employment and education), and governance to enable a favorable policy environment to cultivate a demographic dividend (DD). By measuring efforts, this tool will capture inputs independent of outputs, such as income per capita. This will allow the assessment of the relationship between inputs that go into the development of DD policies and DD outputs that are observed and commonly used to assess progress.

We would like to ask you some questions because of your expertise in the area. The information collected will be kept confidential, and we will not be collecting your names or any other identification details. Completing this questionnaire will take around 45-60 min of your time.

We will appreciate your participation in this survey. Information from this interview will be used by the Gates Institute and the community of DD practice in your country to assess the national efforts and identify areas to continue supporting the successes and those that need to be strengthened. Researchers may also use the data collected for cross-country analyses.

You are invited to participate in this survey. Please know that this survey is completely voluntary. By participating, there is no direct benefit to you but there is a societal benefit in enabling the government and development partners to strengthen their efforts to cultivate a DD-favorable environment.

You may refuse to answer any question. Should you have any concerns or issues about the study, you may contact the study coordinator at **jcrusatira@jhu.edu** or **+14436510824** by a phone call or WhatsApp.

* Please check “Yes” if you would like to proceed with the survey.

- ○ Yes
- ○ No

Bill & Melinda Gates Institute for Population and Reproductive Health

Demographic Dividend Effort Index Survey

Important notice

Please do not select 'Back' while in the middle of answering a page of questions as the page you are working on will not be saved until you finalize the said page and select 'Next'. If you select 'Back' without finalizing, the progress you have made on the unfinished page will be lost.

Please be aware that your survey is completed when you read "Thank you for completing the demographic dividend effort index. Stay well and safe!"

JOHNS HOPKINS
BLOOMBERG SCHOOL
of PUBLIC HEALTH

Bill & Melinda Gates Institute for Population and Reproductive Health

Demographic Dividend Effort Index Survey

Respondent country and expertise

* Please select the country where you are responding from.

- ○ Kenya
- ○ Senegal
- ○ Nigeria
- ○ Rwanda
- ○ Tanzania
- ○ Ethiopia

JOHNS HOPKINS
BLOOMBERG SCHOOL
of PUBLIC HEALTH

Bill & Melinda Gates Institute for Population and Reproductive Health

Demographic Dividend Effort Index Survey

Level of expertise

* At which level of Government is your expertise (If your expertise is at the national level please select "National" otherwise select a county?

JOHNS HOPKINS
BLOOMBERG SCHOOL
of PUBLIC HEALTH

Bill & Melinda Gates Institute for Population and Reproductive Health

Demographic Dividend Effort Index Survey

Expertise

* Please select the organization you have worked in the most during the last five years. If your organization is not listed, please write it down.

- Public sector
- Private sector
- Non-governmental organization
- University/research institute
- Other (please specify)

* Which dimension have you worked in the most during the last five years?

- Policy/policymaking
- Services or programs
- Advocacy
- Research
- Civil Society

* In which sector have you worked the most during the last five years?

- Family Planning
- Maternal and Child Health
- Education
- Women's Empowerment
- Labor Market
- Governance and Economic Institutions

JOHNS HOPKINS
BLOOMBERG SCHOOL
of PUBLIC HEALTH

Bill & Melinda Gates Institute for Population and Reproductive Health

Demographic Dividend Effort Index Survey

Family Planning Questionnaire

Please respond to the following questions based on your experience/expertise within this specific sector.

To give a summary picture of the national demographic dividend effort, please rate the following items. Score each item from 1 to 10, where 1 represents the lowest score (very weak or no effort) and 10 represents the highest score (strong effort). Where applicable, if a policy or activity is non-existent then mark your response as 0. Please note that a few questions have a different scoring scale.

Please answer each item. All responses will be recorded in the format exemplified below:

Lowest effort	1	2	3	4	5	6	7	8	9	10	Highest effort	I do not know

JOHNS HOPKINS
BLOOMBERG SCHOOL
of PUBLIC HEALTH

Bill & Melinda Gates Institute for Population and Reproductive Health

Demographic Dividend Effort Index Survey

Dimension 1. Policy/Policy making

* FP 1.1 Prominence of Family Planning (FP): Extent to which there is a permanent office or post within the appropriate ministry that develops and monitors sectoral policies and strategies, that is well placed within the government, and staffed by adequately skilled personnel.

	1	2	3	4	5	6	7	8	9	10	Don't know
1-Lowest -> Highest-10	○	○	○	○	○	○	○	○	○	○	○

* FP 1.2 Policy on Fertility Reduction and FP: Extent to which government policy emphasizes FP for family size as it relates to available resources or demographic reasons over health reasons.

	1	2	3	4	5	6	7	8	9	10	Don't know
1-Lowest -> Highest-10	○	○	○	○	○	○	○	○	○	○	○

* FP 1.3 National FP Strategic Plan: Extent to which there is a current, comprehensive, strategic national plan for FP that outlines policies, laws, and regulations for the provisioning of FP. (In making this assessment, consider whether the plan defines a vision; includes multiyear strategies, annual timelines, and implementation plans; is evidence-based; has indicators to measure progress; and considers the FP market.)

	1	2	3	4	5	6	7	8	9	10	Don't know
1-Lowest -> Highest-10	○	○	○	○	○	○	○	○	○	○	○

* FP 1.4 Policy on Age at Marriage: Extent to which legal age at marriage for females is set at 18 years or higher and is enforced.

	1	2	3	4	5	6	7	8	9	10	Don't know
1-Lowest -> Highest-10	○	○	○	○	○	○	○	○	○	○	○

* FP 1.5 Evidence-Based Policymaking: Extent to which the appropriate ministry actively and regularly supports and uses key FP data and reports to engage in policy development and planning.

	1	2	3	4	5	6	7	8	9	10	Don't know
1-Lowest -> Highest-10	◯	◯	◯	◯	◯	◯	◯	◯	◯	◯	◯

* FP 1.6 Monitoring and Evaluation of National Policies/Plans/Strategies: Extent to which the national FP plan outlines a comprehensive monitoring and evaluation strategy to track and measure accomplishments in the plan.

(In making this assessment, consider whether the monitoring and evaluation strategy includes goals and objectives, a results framework, measurable indicators [e.g., “SMART”: specific, measurable, attainable, relevant and time-bound]; and an actionable monitoring and evaluation implementation plan/timeline.)

	1	2	3	4	5	6	7	8	9	10	Don't know
1-Lowest -> Highest-10	◯	◯	◯	◯	◯	◯	◯	◯	◯	◯	◯

* FP 1.7 Inclusion of Nongovernmental and Cross-sectoral Actors in Policy/Strategy Development: Extent to which nongovernmental agencies, groups (e.g., from civil society), health workers, stakeholders (e.g., from faith-based, nonprofit, and private-for-profit organizations) and actors across non-FP-specific sectors are included in the national FP plan and/or other national documents outlining the country’s strategies and systems related to planning, managing, and developing FP.

	1	2	3	4	5	6	7	8	9	10	Don't know
1-Lowest -> Highest-10	◯	◯	◯	◯	◯	◯	◯	◯	◯	◯	◯

* FP 1.8 Financing for FP: Extent to which the government has established and applied guidelines for the costing and payments for FP across supply and demand considerations (i.e. domestic versus international funding – and its expectations for continuity/sustainability, cost of contraceptive supplies, cost of service delivery and transportation, etc.).

(In making this assessment, consider whether the guidelines and budget address the following: the lifespan of costs for FP care and services; variable structures of costs per FP method; costs of delivery and service provision by specialization, location and risk; costs and profits of healthcare/insurance schemes, the financial costs of providing FP weighed against the financing costs of not doing so.)

	1	2	3	4	5	6	7	8	9	10	Don't know
1-Lowest -> Highest-10	◯	◯	◯	◯	◯	◯	◯	◯	◯	◯	◯

* FP 1.9 Percent of In-country Funding of FP Budget: Extent to which the total family planning/population budget is derived from in-country sources (e.g., 1 for 10 percent, 5 for 50 percent, 10 for 100 percent).

	1	2	3	4	5	6	7	8	9	10	Don't know
1-Lowest -> Highest-10	◯	◯	◯	◯	◯	◯	◯	◯	◯	◯	◯

* FP 1.10 Information/Communications Technology Infrastructure/Capacity: Extent to which there are adequate infrastructure components (e.g., computers, servers, printers, a local area network, Internet connectivity, and reliable electric power) as well as human resources (e.g., for maintenance and support) at the national and subnational levels to support the functioning of FP-related information systems.
(In making this assessment, consider whether the country conducts at least annual assessments of its information systems; has training curricula that cover data entry, use, software administration, and development; and continuously trains staff from key stakeholder institutions [e.g., Ministry of Health, Ministry of Education, regulatory agencies, training institutions].)

	1	2	3	4	5	6	7	8	9	10	Don't know
1-Lowest -> Highest-10	◯	◯	◯	◯	◯	◯	◯	◯	◯	◯	◯

* FP 1.11 Import Laws and Legal Regulations:

	1	2	3	4	5	6	7	8	9	10	Don't know
FP 1.11.1 Extent to which import laws and legal regulations facilitate the importation of FP commodities and supplies	◯	◯	◯	◯	◯	◯	◯	◯	◯	◯	◯
FP 1.11.2 Extent to which contraceptives are manufactured locally.	◯	◯	◯	◯	◯	◯	◯	◯	◯	◯	◯

* FP 1.12 Advertising of Contraceptives Allowed: Extent of freedom from restrictions on advertising of contraceptives in the mass media.

	1	2	3	4	5	6	7	8	9	10	Don't know
1-Lowest -> Highest-10	◯	◯	◯	◯	◯	◯	◯	◯	◯	◯	◯

* FP 1.13 Presence of Pro-Poor FP Policies: Extent to which policies specifically target the poor in rural or urban places.

	1	2	3	4	5	6	7	8	9	10	Don't know
1-Lowest -> Highest-10	◯	◯	◯	◯	◯	◯	◯	◯	◯	◯	◯

* FP 1.14 Political Support for FP: Extent to which elected officials in the country prioritize meeting FP needs to strengthen the FP contributions to the DD by passing laws and regulations and sponsoring actions and policies aimed at improving FP uptake and adherence.

	1	2	3	4	5	6	7	8	9	10	Don't know
1-Lowest -> Highest-10	○	○	○	○	○	○	○	○	○	○	○

FP 1.15 Comments: Please use this section to provide any additional comments about the level of effort, challenges, or successes in the country around policies/policymaking for FP. If you marked “I don’t know” for any of the questions, also use this section to explain why you gave that rating

JOHNS HOPKINS
BLOOMBERG SCHOOL
of PUBLIC HEALTH

Bill & Melinda Gates Institute for Population and Reproductive Health

Demographic Dividend Effort Index Survey

Dimension 2. Services or Programs

* FP 2.1 Administrative Structure: Extent to which the administrative structure and staff at national, provincial and county levels are sufficient to implement the family planning program.

	1	2	3	4	5	6	7	8	9	10	Don't know
1-Lowest -> Highest-10	◯	◯	◯	◯	◯	◯	◯	◯	◯	◯	◯

* FP 2.2 Level of Program Leaders: Relative level of seniority of the director of the national family planning program and whether said director reports to a high level of government.

	1	2	3	4	5	6	7	8	9	10	Don't know
1-Lowest -> Highest-10	◯	◯	◯	◯	◯	◯	◯	◯	◯	◯	◯

* FP. 2.3 Community-Based Distribution: Extent to which areas of the country not easily serviced by clinics or other service points are covered by community-based distribution programs for distribution of contraceptive supplies (especially rural areas).

	1	2	3	4	5	6	7	8	9	10	Don't know
1-Lowest -> Highest-10	◯	◯	◯	◯	◯	◯	◯	◯	◯	◯	◯

* FP 2.4 Postpartum Program: Extent to which all postpartum women receive postpartum family planning services.

	1	2	3	4	5	6	7	8	9	10	Don't know
1-Lowest -> Highest-10	◯	◯	◯	◯	◯	◯	◯	◯	◯	◯	◯

* FP 2.5 Home-Visiting Workers: Extent of coverage of population by workers whose primary task is to visit (rural) women in their homes to talk about family planning and maternal and child health.

	1	2	3	4	5	6	7	8	9	10	Don't know
1-Lowest -> Highest-10	○	○	○	○	○	○	○	○	○	○	○

* FP 2.6 Availability and Accessibility of FP Commodities/Supplies and Services Part 1: Extent to which entire population has ready and easy access to (1-Lowest -> Highest-10):

	1	2	3	4	5	6	7	8	9	10	Don't know
FP 2.6.1 IUD	○	○	○	○	○	○	○	○	○	○	○
FP 2.6.2 Pills	○	○	○	○	○	○	○	○	○	○	○
FP 2.6.3 Injectables	○	○	○	○	○	○	○	○	○	○	○
FP 2.6.4 Implants	○	○	○	○	○	○	○	○	○	○	○
FP 2.6.5 Female Sterilization	○	○	○	○	○	○	○	○	○	○	○
FP 2.6.6 Male Sterilization	○	○	○	○	○	○	○	○	○	○	○
FP 2.6.7 Male Condoms	○	○	○	○	○	○	○	○	○	○	○
FP 2.6.8 Emergency Contraception	○	○	○	○	○	○	○	○	○	○	○
FP 2.6.9 Abortion/menstrual regulation (whether legal or not)	○	○	○	○	○	○	○	○	○	○	○
FP 2.6.10 Others (e.g. female condoms, foam, beads, etc.)	○	○	○	○	○	○	○	○	○	○	○

* FP 2.7 Availability and Accessibility of FP Commodities/Supplies and Services Part 2: How well does the supply system operate (it avoids stockouts or interrupted supplies and guarantees a reliable flow at local levels) for the following (1-Lowest -> Highest-10):

	1	2	3	4	5	6	7	8	9	10	Don't know
FP 2.7.1 IUD	○	○	○	○	○	○	○	○	○	○	○
FP 2.7.2 Pills	○	○	○	○	○	○	○	○	○	○	○
FP 2.7.3 Injectables	○	○	○	○	○	○	○	○	○	○	○
FP 2.7.4 Implants	○	○	○	○	○	○	○	○	○	○	○
FP 2.7.5 Male Condoms	○	○	○	○	○	○	○	○	○	○	○
FP 2.7.6 Emergency Contraception	○	○	○	○	○	○	○	○	○	○	○
FP 2.7.7 Others (e.g. female condoms, foam, beads, etc.)	○	○	○	○	○	○	○	○	○	○	○

* FP 2.8 Availability and Accessibility of FP Commodities/Supplies and Services Part 3: How well does the supply system provide necessary equipment and medical supplies at clinical facilities for the following (1-Lowest -> Highest-10):

	1	2	3	4	5	6	7	8	9	10	Don't know
FP 2.8.1 Female Sterilization	○	○	○	○	○	○	○	○	○	○	○
FP 2.8.2 Male Sterilization	○	○	○	○	○	○	○	○	○	○	○
FP 2.8.3. IUDs	○	○	○	○	○	○	○	○	○	○	○
FP 2.8.4 Implants	○	○	○	○	○	○	○	○	○	○	○

* FP 2.9 FP Service Quality: Please rate the general quality of family planning services. (Good quality includes a focus on client needs, with counseling, full information, wide method choice, and safe clinical procedures.)

	1	2	3	4	5	6	7	8	9	10	Don't know
1-Lowest -> Highest-10	○	○	○	○	○	○	○	○	○	○	○

* FP 2.10 Incentives and Disincentives: Extent to which monetary or other incentives are used to encourage the adoption of family planning.

	1	2	3	4	5	6	7	8	9	10	Don't know
1-Lowest -> Highest-10	○	○	○	○	○	○	○	○	○	○	○

* FP 2.11 Adolescent Programs: Extent to which information, services and activities are focused towards adolescents and encourage adolescents to access contraceptive services.

	1	2	3	4	5	6	7	8	9	10	Don't know
1-Lowest -> Highest-10	○	○	○	○	○	○	○	○	○	○	○

* FP 2.12 Training Program: Extent to which training programs, for each category of staff in the family planning program, are adequate to provide personnel with information and skills necessary to carry out their jobs effectively.

	1	2	3	4	5	6	7	8	9	10	Don't know
1-Lowest -> Highest-10	○	○	○	○	○	○	○	○	○	○	○

* FP 2.13 Personnel Carry Out Assigned Tasks: Extent to which all categories of family planning program staff (administrative, medical, paramedical, field) carry out assigned tasks effectively

	1	2	3	4	5	6	7	8	9	10	Don't know
1-Lowest -> Highest-10	○	○	○	○	○	○	○	○	○	○	○

* FP 2.14 Logistics and Transport: Extent to which the logistics and transport systems are sufficient to keep stocks of contraceptive supplies and related equipment available at all service points, at all times and at all levels.

	1	2	3	4	5	6	7	8	9	10	Don't know
1-Lowest -> Highest-10	○	○	○	○	○	○	○	○	○	○	○

* FP 2.15 Supervision System: Extent to which the system of supervision at all levels is adequate (regular monitoring visits with corrective or supportive action).

	1	2	3	4	5	6	7	8	9	10	Don't know
1-Lowest -> Highest-10	○	○	○	○	○	○	○	○	○	○	○

* FP 2.16 Involvement of Other Ministries and Public Agencies: Extent to which other ministries, government agencies and non-governmental stakeholders engage with FP activities (e.g., delivery of supplies, services, information, education) or other population activities.

	1	2	3	4	5	6	7	8	9	10	Don't know
1-Lowest -> Highest-10	○	○	○	○	○	○	○	○	○	○	○

* FP 2.17 Involvement of Private-Sector Agencies and Groups: Extent to which private-sector agencies and groups assist with FP or other population activities.

	1	2	3	4	5	6	7	8	9	10	Don't know
1-Lowest -> Highest-10	◯	◯	◯	◯	◯	◯	◯	◯	◯	◯	◯

* FP 2.18 Mass Media for Information Education Communication: Frequency and extent of coverage of mass media messages that provide the population with information on family planning and service sites.

	1	2	3	4	5	6	7	8	9	10	Don't know
1-Lowest -> Highest-10	◯	◯	◯	◯	◯	◯	◯	◯	◯	◯	◯

* FP 2.19 Cultural and Social Norms Barriers: Extent to which cultural and social barriers are addressed in family planning programs.

	1	2	3	4	5	6	7	8	9	10	Don't know
1-Lowest -> Highest-10	◯	◯	◯	◯	◯	◯	◯	◯	◯	◯	◯

* FP 2.20 Youth: Extent to which the government supports youth-led FP initiatives or involve youth in planning, implementation and evaluation of FP programs.

	1	2	3	4	5	6	7	8	9	10	Don't know
1-Lowest -> Highest-10	◯	◯	◯	◯	◯	◯	◯	◯	◯	◯	◯

* FP 2.21 FP Effort Influences – Forces Affecting the FP Program: Forces affecting the FP program can either strengthen or detract from its effectiveness. Check zero (0) if there is no difference either way; check a negative number from -1 to -5 if the influence is negative; or check a positive number from 1 to 5 if the influence is positive. (1 in either direction means little influence and 5 in either direction means strong influence.)

	-5	-4	-3	-2	-1	0	1	2	3	4	5	Don't know
FP 2.21.1 Decentralization	○	○	○	○	○	○	○	○	○	○	○	○
FP 2.21.2 HIV/AIDS Programs	○	○	○	○	○	○	○	○	○	○	○	○
FP 2.21.3 Incorporation of FP into a broader context of reproductive health	○	○	○	○	○	○	○	○	○	○	○	○
FP 2.21.4 Integration of FP with other health services	○	○	○	○	○	○	○	○	○	○	○	○
FP 2.21.5 Changes in donor funding	○	○	○	○	○	○	○	○	○	○	○	○
FP 2.21.6 Changes in domestic government funding	○	○	○	○	○	○	○	○	○	○	○	○

FP 2.22 Comments: Please use this section to provide any additional comments about the level of effort, challenges, or successes in the country around services or programs for FP. If you marked “I don’t know” for any of the questions, also use this section to explain why you gave that rating

JOHNS HOPKINS
BLOOMBERG SCHOOL
of PUBLIC HEALTH

Bill & Melinda Gates Institute for Population and Reproductive Health

Demographic Dividend Effort Index Survey

Dimension 3. Advocacy

* FP 3.1 Influence of FP Leaders/Champions: Extent to which the country has one or more clearly influential leaders or champions who successfully advocate for FP (sectoral) needs at high levels, promote FP in the country by making positive public statements about it, and/or support actions and policies aimed at improving the FP environment and its contributions towards the DD.

	1	2	3	4	5	6	7	8	9	10	Don't know
1-Lowest -> Highest-10	◯	◯	◯	◯	◯	◯	◯	◯	◯	◯	◯

* FP 3.2 Statement by Leaders: Extent to which the head of government, as well as other officials, speak publicly and favorably about family planning at least once or twice a year.

	1	2	3	4	5	6	7	8	9	10	Don't know
1-Lowest -> Highest-10	◯	◯	◯	◯	◯	◯	◯	◯	◯	◯	◯

* FP 3.3 Media Coverage for FP: Extent to and frequency with which national media cover/broadcast FP topics that provides the population with information on family planning and service sites.

	1	2	3	4	5	6	7	8	9	10	Don't know
1-Lowest -> Highest-10	◯	◯	◯	◯	◯	◯	◯	◯	◯	◯	◯

* FP 3.4 FP Effort Influences - Justification: How important is each of the following as a current government justification for the national FP program? (1 means negligible importance; 10 means great importance.)

	1	2	3	4	5	6	7	8	9	10	Don't know
FP 3.4.1 Reduce the rate of population growth											
FP 3.4.2 Enhance economic development											
FP 3.4.3 Help women and men avoid unplanned and unwanted births											
FP 3.4.4 Improve women's health											
FP 3.4.5 Improve child health											
FP 3.4.6 Reduce unmarried adolescent childbearing											
FP 3.4.7 Reduce unmet need for contraceptive services											

* FP 3.5 FP Effort Influences – Special Populations: To what extent does the family planning program give particular emphasis to special populations? (1 means negligible emphasis; 10 means great emphasis)

	1	2	3	4	5	6	7	8	9	10	Don't know
FP 3.5.1 Unmarried youth											
FP 3.5.2 The poor											
FP 3.5.3 The disabled											
FP 3.5.4 Rural population											
FP 3.5.5 Other vulnerable groups (e.g. minority and/or indigenous groups)											
FP 3.5.6 Postpartum women for counseling and contraceptive services											
FP 3.5.7 Post-abortion women for counseling and contraceptive services											

* FP 3.6 Social Marketing: Extent of coverage of the country by a social marketing program (subsidized contraceptive sales at low cost in commercial sector, especially in urban areas).

	1	2	3	4	5	6	7	8	9	10	Don't know
1-Lowest -> Highest-10	○	○	○	○	○	○	○	○	○	○	○

FP 3.7 Comments: Please use this section to provide any additional comments about the level of effort, challenges, or successes in the country around advocacy for FP. If you marked “I don’t know” for any of the questions, also use this section to explain why you gave that rating.

JOHNS HOPKINS
BLOOMBERG SCHOOL
of PUBLIC HEALTH

Bill & Melinda Gates Institute for Population and Reproductive Health

Demographic Dividend Effort Index Survey

Dimension 4. Research

* FP 4.1 Strength of Observatory/Stakeholder/Technical Working Group for FP: Extent to which country-level FP stakeholders (e.g., stakeholder leadership group, technical working group, FP observatory, country coordination and facilitation group):

	1	2	3	4	5	6	7	8	9	10	Don't know
FP 4.1.1 Have representation in government, training institutions, civil society, nongovernmental and faith-based organizations, professional associations, the private sector, etc.	○	○	○	○	○	○	○	○	○	○	○
FP 4.1.2 Meet regularly, report and recommend policy for senior management within the appropriate ministries	○	○	○	○	○	○	○	○	○	○	○
FP 4.1.3 Make an impact on FP within the country	○	○	○	○	○	○	○	○	○	○	○

* FP 4.2 Education/Research Strategy: Extent to which the national FP plan and/or other national documents include a comprehensive strategy/approach on FP-related research.

	1	2	3	4	5	6	7	8	9	10	Don't know
1-Lowest -> Highest-10	○	○	○	○	○	○	○	○	○	○	○

* FP 4.3 Data Gathering Partners:

	1	2	3	4	5	6	7	8	9	10	Don't know
FP 4.3.1 Extent to which research is undertaken by government agencies. (This may include statistical agencies/bureaus and/or ministries of economics/finances, education, health, gender, etc.)											
FP 4.3.2 Extent to which research is undertaken by research institutions.											
FP 4.3.3 Extent to which research is undertaken by independent researchers.											

* FP 4.4 Topical Data Gathering:

	1	2	3	4	5	6	7	8	9	10	Don't know
FP 4.4.1 Extent to which family planning uptake (including method mix), demand, and unmet need is researched.											
FP 4.4.2 Extent to which barriers to FP uptake is researched.											
FP 4.4.3 Extent to which the impacts of FP programs are researched.											
FP 4.4.4 Extent to which the quality of service provision for FP is researched.											

* FP 4.5 Quality/Coverage of Data: Extent to which current research/data is sex and age disaggregated. Extent to which the country participates in standardized tests and its ranking.

	1	2	3	4	5	6	7	8	9	10	Don't know
FP 4.5.1 Extent to which a routine statistical system provides reliable periodic information on FP supplies and facilities.	○	○	○	○	○	○	○	○	○	○	○
FP 4.5.2 Extent to which a routine statistical system provides good periodic information on FP services and personnel.	○	○	○	○	○	○	○	○	○	○	○
FP 4.5.3 Extent to which the routine statistical system provides good periodic information on FP needs among populations in different communities.	○	○	○	○	○	○	○	○	○	○	○
FP 4.5.4 Extent to which evaluations of the health monitoring and information system are conducted and applied to ensure the reliability of data.	○	○	○	○	○	○	○	○	○	○	○

* FP 4.6 Record-Keeping: Extent to which systems for client recordkeeping, clinic reporting and feedback of results are adequate, including being connected at all levels (federal, state/province, county, etc.).

	1	2	3	4	5	6	7	8	9	10	Don't know
1-Lowest -> Highest-10	○	○	○	○	○	○	○	○	○	○	○

* FP 4.7 Quality Research Institutions: Extent to which the country supports and sustains research institutions that develop research/collect data that support the breadth and quality of information-gathering to inform national FP efforts.

	1	2	3	4	5	6	7	8	9	10	Don't know
1-Lowest -> Highest-10	○	○	○	○	○	○	○	○	○	○	○

* FP 4.8 Evaluation: Extent to which program statistics, surveys, and small studies are used by specialized staff to report on program operations and measure progress.

	1	2	3	4	5	6	7	8	9	10	Don't know
1-Lowest -> Highest-10	◯	◯	◯	◯	◯	◯	◯	◯	◯	◯	◯

* FP 4.9 Management's Use of Evaluation Findings: Extent to which local-level program managers use research and evaluation findings to improve programming in ways suggested by findings.

	1	2	3	4	5	6	7	8	9	10	Don't know
1-Lowest -> Highest-10	◯	◯	◯	◯	◯	◯	◯	◯	◯	◯	◯

* FP 4.10 Ministerial Use of Evaluation Findings: Extent to which the Ministry administrators/policymakers systematically utilize data to inform policies and interventions to address FP-related issues.

	1	2	3	4	5	6	7	8	9	10	Don't know
1-Lowest -> Highest-10	◯	◯	◯	◯	◯	◯	◯	◯	◯	◯	◯

* FP 4.11 Dissemination of Information to Other Implementing Bodies: Extent to which information is shared or spread across geographical areas and throughout various levels (national, state/province/county, localities/sub-counties).

	1	2	3	4	5	6	7	8	9	10	Don't know
1-Lowest -> Highest-10	◯	◯	◯	◯	◯	◯	◯	◯	◯	◯	◯

FP 4.12 Comments: Please use this section to provide any additional comments about the level of effort, challenges, or successes in the country around FP research. If you marked "I don't know" for any of the questions, also use this section to explain why you gave that rating.

JOHNS HOPKINS
BLOOMBERG SCHOOL
of PUBLIC HEALTH

Bill & Melinda Gates Institute for Population and Reproductive Health

Demographic Dividend Effort Index Survey

Dimension 5. Civil Society Organizations (CSOs)

* FP 5.1 CSO Actor Power: Extent to which CSO actors are in strong placements within institutions or technical working groups or networks concerned with FP.

	1	2	3	4	5	6	7	8	9	10	Don't know
1-Lowest -> Highest-10	○	○	○	○	○	○	○	○	○	○	○

* FP 5.2 Budget Analysis as a CSO Tool: Extent to which CSOs utilize budget analysis as a tool to build FP advocacy cases.

	1	2	3	4	5	6	7	8	9	10	Don't know
1-Lowest -> Highest-10	○	○	○	○	○	○	○	○	○	○	○

* FP 5.3 Facility-Based Service Delivery Support: Extent to which CSOs are involved in efforts to increase the use and/or quality of services, particularly through increasing the technical and/or operational capacity of CSO-partnered clinics to provide FP services.

	1	2	3	4	5	6	7	8	9	10	Don't know
1-Lowest -> Highest-10	○	○	○	○	○	○	○	○	○	○	○

* FP 5.4 Community-Based Service Delivery Support: Extent to which CSOs participate in bringing FP information and services to the communities where people live through activities such as supporting the capacity-building of community-level health workers, expanding the range of contraceptive methods provided by community health workers, educating lower cadre health workers, and/or undertaking information and education campaigns to increase the acceptability of contraception.

	1	2	3	4	5	6	7	8	9	10	Don't know
1-Lowest -> Highest-10	○	○	○	○	○	○	○	○	○	○	○

* FP 5.5 Mobile Outreach for Community-Based Service Delivery Support: Extent to which CSOs support outreach models to equip health care providers with FP commodities, supplies, equipment and vehicles, which are then sent as specialist team to provide FP information and methods directly to communities.

	1	2	3	4	5	6	7	8	9	10	Don't know
1-Lowest -> Highest-10	○	○	○	○	○	○	○	○	○	○	○

* FP 5.6 Social Franchising: Extent to which CSOs are involved in social franchising opportunities/services to bring private sector services to clients at reduced prices.

	1	2	3	4	5	6	7	8	9	10	Don't know
1-Lowest -> Highest-10	○	○	○	○	○	○	○	○	○	○	○

* FP 5.7 mHealth: Extent to which CSOs are leveraging mHealth to utilize text messages and other mobile technologies to improve access to FP information and services.

	1	2	3	4	5	6	7	8	9	10	Don't know
1-Lowest -> Highest-10	○	○	○	○	○	○	○	○	○	○	○

* FP 5.8 Human Rights and Quality of Care: Extent to which CSOs advocate for and support rights-based approaches to FP to ensure FP service recipients experience good treatment, confidence in asking questions, and quality counselling, amongst other concerns.

	1	2	3	4	5	6	7	8	9	10	Don't know
1-Lowest -> Highest-10	○	○	○	○	○	○	○	○	○	○	○

* FP 5.9 Social and Behavior Change (SBC): Extent to which CSOs support SBC interventions relating, but not limited, to life skills and peer education, adult health education, school-based education, couple’s communication, community mobilization, working through gatekeepers, and/or mass media interventions for a general public audience. These may be supported through implementation at the community or individual levels and may relate to outcomes such as increase in FP uptake and changes in FP knowledge and attitudes.

	1	2	3	4	5	6	7	8	9	10	Don't know
1-Lowest -> Highest-10	○	○	○	○	○	○	○	○	○	○	○

* FP 5.10 Youth: Extent to which CSOs support FP policies, interventions and programming targeting youth specifically.

	1	2	3	4	5	6	7	8	9	10	Don't know
1-Lowest -> Highest-10	○	○	○	○	○	○	○	○	○	○	○

* FP 5.11 Programming for Men: Extent to which CSOs support FP policies, interventions and programming for men.

	1	2	3	4	5	6	7	8	9	10	Don't know
1-Lowest -> Highest-10	◯	◯	◯	◯	◯	◯	◯	◯	◯	◯	◯

* FP 5.12 Advocacy/Accountability: Extent to which CSOs support the training and capacity building of advocates and community members to understand policies, processes, and activities to increase community-led issue identification, collaborative problem solving and more targeted advocacy actions.

	1	2	3	4	5	6	7	8	9	10	Don't know
1-Lowest -> Highest-10	◯	◯	◯	◯	◯	◯	◯	◯	◯	◯	◯

* FP 5.13 CSO-Led Assessment and Monitoring: Extent to which CSOs assess, monitor and report on the effectiveness of policies and programming to improve accountability at the policy and provider levels.

	1	2	3	4	5	6	7	8	9	10	Don't know
1-Lowest -> Highest-10	◯	◯	◯	◯	◯	◯	◯	◯	◯	◯	◯

* FP 5.14 Forming Partnerships between CSOs: Extent to which CSOs have formed national alliances and regional partnerships to strengthen their positioning and potentially improve leadership strength and funding mechanisms.

	1	2	3	4	5	6	7	8	9	10	Don't know
1-Lowest -> Highest-10	◯	◯	◯	◯	◯	◯	◯	◯	◯	◯	◯
[truncated: 573,293 more chars]
